# Genome-wide DNA methylation and gene expression patterns provide insight into polycystic ovary syndrome development

**DOI:** 10.18632/oncotarget.2224

**Published:** 2014-07-16

**Authors:** Xiu-Xia Wang, Jing-Zan Wei, Jiao Jiao, Shu-Yi Jiang, Da-Hai Yu, Da Li

**Affiliations:** ^1^ Department of Obstetrics and Gynecology, Shengjing Hospital of China Medical University, Shenyang, China

**Keywords:** DNA methylation profiling, expression profiling, epigenetic, PCOS

## Abstract

Polycystic ovary syndrome (PCOS) is one of the most common endocrine disorders in women. However, the epigenetic mechanism involved in PCOS progression remains largely unknown. Here, combining the DNA methylation profiling together with transcriptome analysis, we showed that (i) there were 7929 differentially methylated CpG sites (β > 0.1, *P* < 0.05) and 650 differential transcripts (fold change > 1.5, *P* < 0.005) in PCOS compared to normal ovaries; (ii) 54 genes were identified with methylated levels that were correlated with gene transcription in PCOS; and (iii) there were less hypermethylated sites, but many more hypomethylated sites residing in CpG islands and N_Shore in PCOS. Among these genes, we identified that several significant pathways, including the type I diabetes mellitus pathway, p53 signaling pathway and NOD-like receptor signaling pathway, and some immune and inflammatory diseases may be highly involved in PCOS development. These results suggested that differences in genome-wide DNA methylation and expression patterns exist between PCOS ovaries and normal ovaries; epigenetic mechanisms may in part be responsible for the different gene expression and PCOS phenotype. All of this may improve our understanding of the basic molecular mechanism underlying the development of PCOS.

## INTRODUCTION

Polycystic ovary syndrome (PCOS) is the most common endocrinopathy, affecting about 10% of the reproductive-age female population [1], which has significant and diverse clinical symptoms including reproductive dysfunction (chronic anovulation, polycystic ovary, hyperandrogenism and hirsutism), metabolic disorders (insulin resistance, abdominal obesity, hypertension and dyslipidemia), and psychological distress (anxiety, depression, and sensitivity to stress) [1-4]. In addition to genetic predisposition, environmental and lifestyle factors contribute to the pathogenesis of PCOS [5]; an emerging body of evidence suggests that epigenetic events may in part be responsible for the development of PCOS [6-7]. In the past several years, genomics, proteomics and metabolomics studies have been performed with the goal of identifying factors linked to these disorders [5,8], and have provided reliable information about the molecular pathways involved in PCOS. However, to date, little is known about epigenomics, especially the DNA methylation profiles in the pathophysiology of PCOS. DNA methylation is an important epigenetic phenomenon that modulates gene expression without affecting the DNA sequence, to adapt to the environmental and lifestyle changes [9]. For this reason, the present study was undertaken to investigate comprehensive DNA methylation profiling, together with transcriptome analysis in ovaries from PCOS patients and normal ovaries, and to provide novel insight into the epigenetic-mediated dysfunction in the pathogenesis of PCOS.

## RESULTS

### Differentially methylated and expressed profiling in PCOS

**Fig 1:**
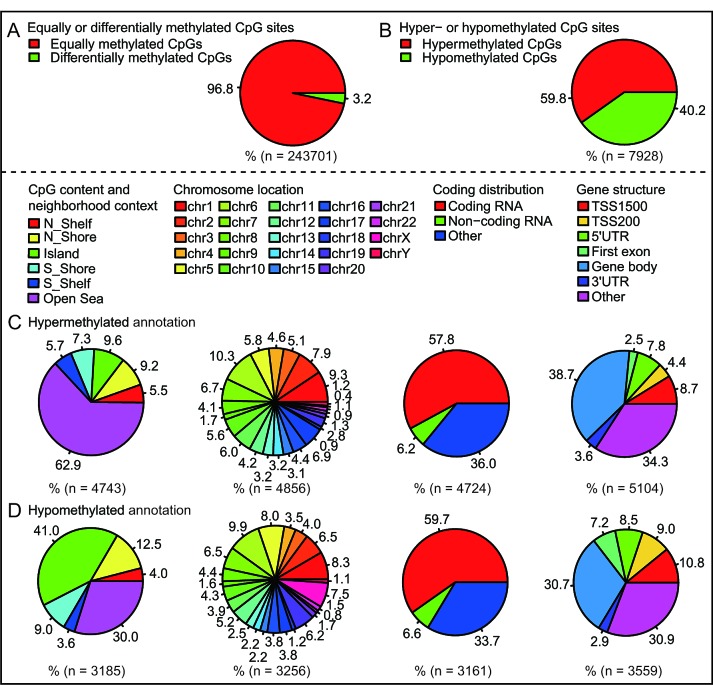
Characteristic methylation patterns between PCOS ovaries and normal ovaries A, Percentage of equally or differentially methylated CpG sites. B, Percentage of hypermethylated or hypomethylated CpG sites. C, Hypermethylated annotation of differentially methylated CpG sites. D, Hypomethylated annotation of differentially methylated CpG sites.

DNA methylation analysis showed 7929 differentially methylated CpG sites, about 3.2% of total sites in PCOS ovaries compared to normal ovaries (Fig. 1A and Supplementary Table 1); the hypermethylated and hypomethylated CpG sites were about 59.8% or 40.2%, respectively (Fig. 1B). Interestingly, there was a significant difference in methylated CpG content and neighborhood context (N_Shelf, N_Shore, Island, S_Shore, S_Shelf, Open Sea) between hypermethylated or hypomethylated CpG sites; in particular, less hypermethylated sites, but many more hypomethylated sites were residing in CpG islands and N_Shore (Fig. 1C and D). However, there was no significant difference in methylated CpG sites residing in chromosomal locations (Chr1-22, and XY), coding distribution (Coding RNA, Non-coding RNA, Other) and gene structure (TSS1500, TSS200, 5′UTR, First exon, Gene body, 3′UTR, Other) (Fig. 1C and D). As shown in Supplementary Table 2, expression profiling analysis showed 650 differential transcripts in PCOS ovaries compared to normal ovaries. Hierarchical clustering of the differentially methylated and expressed probes identified PCOS-specific gene clusters and similarities (Supplementary Fig. 1). Overall, these data suggest the existence of a marked difference in methylation and expression patterns, both of which are implicated in the development of PCOS.

### GO analysis of the differentially methylated and expressed genes associated with PCOS

**Fig 2:**
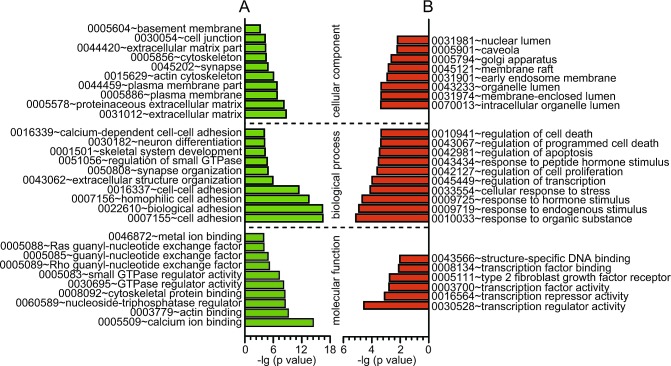
GO analysis of the differentially methylated and expressed genes A, GO analysis of differential methylated genes (β > 0.1, *P* < 0.05). B, GO analysis of differential expressed genes (fold change > 1.5, *P* < 0.005). X axis, negative logarithm (-lg) of the p value; Y axis, GO category. The top 10 GO terms were shown if there were more than 10 terms.

To begin defining the functional significance of the extensive changes in DNA methylation and gene expression profiling of PCOS, GO analysis was performed, which revealed distinct functional categories for the PCOS-associated gene lists (Supplementary Table 3). As shown in Fig. 2A, these differentially methylated genes were widely associated with cell adhesion, the regulation of GTPase activity, neuron differentiation, synapse organization, skeletal system development and extracellular structure organization. In addition, differentially expressed genes were found to be highly involved in basal cellular processes like the regulation of transcription, cell death, apoptosis, cell proliferation, and response to stress, endogenous stimuli, hormone stimuli and organic substances (Fig. 2B).

### KEGG analysis of the differentially methylated and expressed genes associated with PCOS

**Fig 3:**
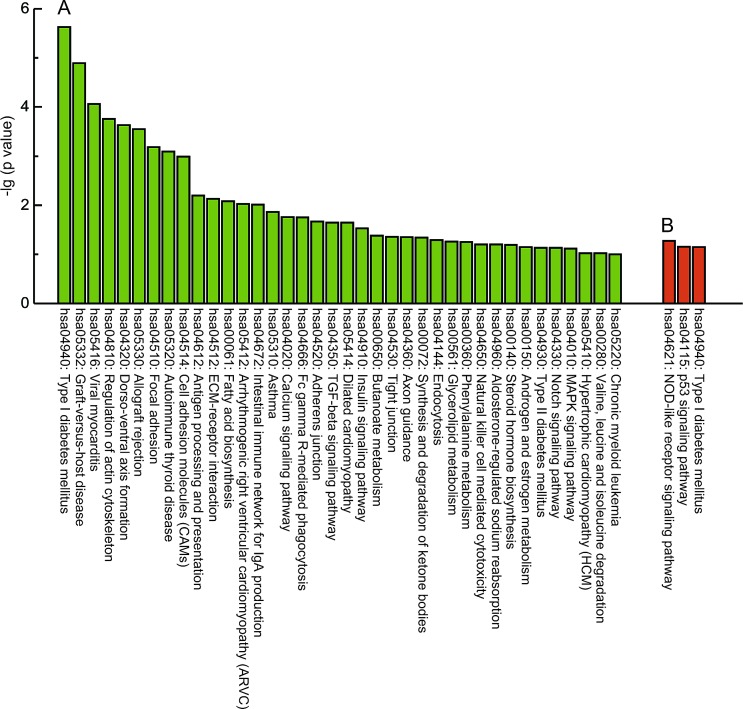
KEGG analysis of the differentially methylated and expressed genes A, KEGG analysis of differential methylated genes (β > 0.1, *P* < 0.05). B, KEGG analysis of differential expressed genes (fold change > 1.5, *P* < 0.005). Y axis, negative logarithm (-lg) of the p value; X axis, KEGG category. The top 10 KEGG terms were shown if there were more than 10 terms.

To further investigate key pathways linked to these distinct genes, the interaction of the significant pathways associated with PCOS was built according to the KEGG database; the distinct functional categories for the PCOS-associated gene lists are shown in Supplementary Table 4. Our analysis showed that these differentially methylated genes are widely involved in various cellular pathways; the top 10 are linked to type I diabetes mellitus, graft-versus-host disease, viral myocarditis, regulation of actin cytoskeleton, dorso-ventral axis formation, allograft rejection, focal adhesion, autoimmune thyroid disease, cell adhesion molecules and antigen processing and presentation (Fig. 3A), while these differentially expressed genes were only distributed in three significant pathways, including type I diabetes mellitus, the p53 signaling pathway and the NOD-like receptor signaling pathway (Fig. 3B). Notably, the co-existence of the type I diabetes mellitus pathway was observed in differentially expressed and methylated profiles.

### Integrative analysis of differential DNA methylation and gene expression associated with PCOS

**Fig 4:**
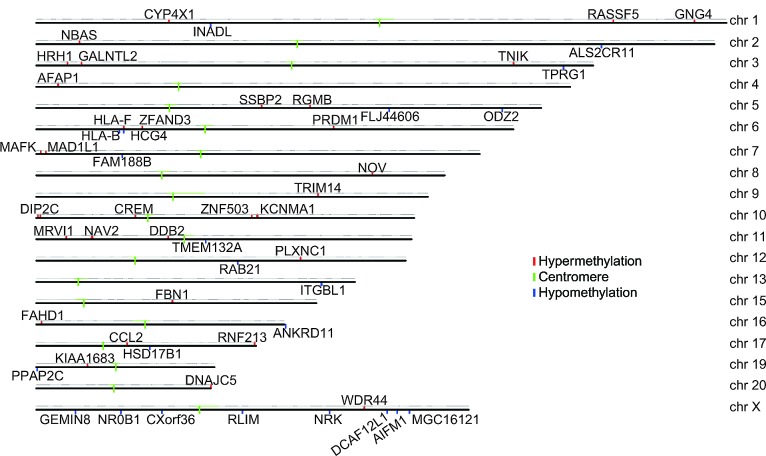
Chromosome graph of differentially methylated genes correlated with gene transcription 54 genes exhibited a significant correlation between DNA methylation and gene expression levels, which were located at different chromosome (chr) positions.

Accumulating evidence demonstrates that DNA methylation is an important epigenetic modification involved in regulating gene expression [10,11]. To date, mounting evidence indicates that DNA hypomethylation or hypermethylation is an alternative mechanism for gene activation or silencing, respectively. However, an emerging body of evidence suggests that DNA methylation is sometimes positively correlated with gene transcription [12,13]. As shown in Fig. 4 and Table 1, we identified 16 hypomethylated and up-regulated genes, 17 hypermethylated and down-regulated genes, 15 hypermethylated and up-regulated genes and 6 hypomethylated and down-regulated genes by integrating DNA methylation and gene expression data; the correlation coefficient data are shown in Supplementary Table 5. In addition, some of the differences were located in methylation patterns in promoter regions, which can modulate gene expression by affecting the binding of transcription factors. Therefore, as shown in Supplementary Table 6, all transcription factors which may bind the differentially methylated sites were predicted.

**Table 1:** Integrated analysis of DNA methylation and mRNA expression associated with PCOS

	Gene_Symbol
Hypomethylated and upregulated genes	AIFM1, ALS2CR11, ANKRD11, CXorf36, DCAF12L1, FAM188B, FLJ44606, GEMIN8, HCG4, HLA-B, HSD17B1, ITGBL1, MGC16121, NR0B1, TMEM132A, TPRG1
Hypermethylated and downregulated genes	AFAP1, CCL2, CREM, CYP4X1, DDB2, DIP2C, GALNTL2, GNG4, KIAA1683, MAFK, NOV, PRDM1, RASSF5, RGMB, SSBP2, WDR44, ZNF503
Hypermethylated and upregulated genes	DNAJC5, FAHD1, FBN1, HLA-F, HRH1, KCNMA1, MAD1L1, MRVI1, NAV2, NBAS, PLXNC1, RNF213, TNIK, TRIM14, ZFAND3
Hypomethylated and downregulated genes	INADL, NRK, ODZ2, PPAP2C, RAB21, RLIM

## DISCUSSION

Emerging evidence indicates that epigenetic mechanisms may in part be responsible for the PCOS phenotype [6,7,14]. However, to date, few studies have examined genome-wide DNA methylation profiling, especially together with gene expression profiling for PCOS ovaries. In this study, we reported for the first time that there were at least 3% differentially methylated sites and 650 differential transcripts between PCOS ovaries and normal ovaries.

Functional analysis of these genes associated with differentially methylated sites and expressed transcripts revealed strong enrichment in the biological process and molecular function. For gene expression profiling, these differentially expressed genes were found to be highly involved in basal cellular processes, such as transcriptional regulation, cell proliferation, apoptosis and death, and response to stress and stimulus. For DNA methylation profiling, these differentially methylated genes are especially associated with the regulation of cell adhesion and small GTPase activity. Similarly, several lines of evidence have demonstrated that intercellular and intracellular adhesion molecule-1, vascular cell adhesion molecule-1, and platelet endothelial cell adhesion molecule-1 all have an important role in PCOS [15-17]. Likewise, Small GTPases are a family of hydrolase enzymes that can bind and hydrolyze guanosine triphosphate. The Ras superfamily is the most common of small GTPases, which is further divided into subfamilies: RAS, RHO, RAB, RAP, ARF, RAN, RHEB, RAD and RIT. To date, RAS [18,19], RAC1 [20], RAB5B [21] and RAB8A [22] have been shown to be involved in the PCOS pathophysiology, but other GTPase-related members were not reported.

Epidemiological studies have reported that individuals with type I diabetes have a higher prevalence of PCOS than the general population [23]. Consistent with this, KEGG analysis showed that the type I diabetes mellitus pathway was enriched in differentially expressed profiling. Intriguingly, the type I diabetes mellitus pathway was also significantly enriched in differentially methylated profiling. It seems that abnormal epigenetic and transcriptional regulation may be jointly involved in the progression of type I diabetes-related PCOS. In addition, the p53 signaling pathway is crucial for regulating proliferation, apoptosis, genomic stability and the inhibition of angiogenesis [24]. The NOD-like receptor signaling pathway is a critical regulator of innate and adaptive immune responses [25]. KEGG analysis of expression profiling suggested that the two pathways may potentially play a direct or indirect role in PCOS, although few reports have linked them to PCOS. Immune diseases (such as graft-versus-host disease, allograft rejection, autoimmune thyroid disease and intestinal immune network for IgA production) and inflammatory diseases (such as viral myocarditis and asthma) were significantly enriched in differentially methylated profiling. According to these results, it can be speculated that immune and inflammatory processes are a key regulator of PCOS. It should be noted that chronic inflammation and immune responses have been increasingly recognized as an important cause of endocrine, metabolic and reproductive disorders that characterize PCOS [26].

As shown in Table 1, 54 genes were identified in which the methylated levels were positively correlated with gene transcription. These data may help to identify several novel epigenetically regulated genes that are possibly involved in PCOS, and these aberrantly methylated and expressed genes may also be potential biomarkers of PCOS. Notably, there were several changes in DNA methylation which were located in gene coding regions, therefore not directly regulating gene transcription, but still having the capacity to influence gene expression via alternative splicing programs [27]. Until recent years, CCL2 [28,29] and FBN1 [30] have been suggested to contribute to PCOS, but other genes are still worthy of further investigation.

Taken together, our study demonstrated that differences in genome-wide DNA methylation and gene expression patterns exist between PCOS ovaries and normal ovaries, and understanding the epigenetic mechanisms involved in PCOS may yield new insight into the pathophysiology of the disorder.

## METHODS

### Ethical Statements

The investigation was conducted in accordance with the ethical standards and according to the Helsinki Declaration of 1975.

### Patients and tissue collection

This study was approved by the Institutional Review Board at China Medical University. Cervical cancer patients with normal ovaries and PCOS patients were enrolled between April 2012 and July 2012, and all patients gave informed consent. Fresh ovarian tissue was obtained from three cervical cancer patients (Control 1-3) at the follicular phase, with regular menstrual cycles (25–35 days) and younger than 40 years, who underwent radical hysterectomy and pelvic lymphadenectomy for cervical cancer. Fresh ovarian tissue was obtained from three PCOS patients (PCOS 1-3) at the follicular phase, with irregular menstrual cycle and younger than 40 years, who underwent laparoscopic wedge resection. PCOS was defined according to the 2003 Rotterdam criteria [4]. The subject's characteristics are given in Supplementary Table 7.

### DNA methylation profiling analysis

Genomic DNA was extracted from ovarian tissue with a DNeasy Blood and Tissue Kit (Qiagen, Valencia, CA). A total of 1.25 μg of genomic DNA was subjected to bisulfite conversion using the EZ DNA Methylation Gold Kit (Zymo Research, Irvine, CA). About 600 ng of the bisulfite-converted DNA was analyzed on an Infinium HumanMethylation450 BeadChip (Illumina, San Diego, CA) following the manufacturer's guidelines. These Chips feature more than 450,000 methylation sites within and outside CpG islands. Methylation values for individual CpG sites were obtained as β-values, calculated as the ratio of the methylated signal intensity to the sum of both methylated and unmethylated signals after background subtraction. The β-values were reported as a DNA methylation score ranging from 0 (non-methylated) to 1 (completely methylated).

### Gene expression profiling analysis

Total RNA was extracted from ovarian tissue with Trizol reagents (Invitrogen, Carlsbad, CA) and purified using an RNeasy minikit (Qiagen). RNA purity was assessed by measuring the A260/A280 ratio using a Nanodrop spectrophotometer (NanoDrop, Wilmington, DE), and RNA quality was assessed using an Agilent 2100 Bioanalyzer (Agilent, Palo Alto, CA). Then, 1 μg of total RNA was used for amplification with the MessageAMP amplified RNA kit (Ambion, Foster City, CA). Fragmented and labeled RNA was hybridized to the Affymetrix Human Genome U133 Plus 2.0 Array (Affymetrix, Santa Clara, CA) according to the manufacturer's protocol. Arrays were analyzed on a Hewlett-Packard Genearray scanner (Hewlett Packard, Palo Alto, CA) using the GeneChip software (Affymetrix).

### Data analysis

DNA methylation profiling data were processed by the Methylation Module of GenomeStudio v1.9 software using default parameters (Illumina). In each individual, probes with a detection *P* > 0.05 were removed, and β-values in PCOS cases were compared against controls using two-sided Student's *t*-tests. Statistical differences were considered significant at β > 0.1. Expression profiling data were assessed using the Affymetrix GeneChip Operating Software (Affymetrix). Statistical differences were considered significant at fold change > 1.5, *P* < 0.005. DAVID (Database for Annotation, Visualization, and Integrated Discovery) was used to identify functional groups of differential genes and biological processes which are associated with PCOS; Gene Ontology (GO) and Kyoto Encyclopedia of Genes and Genomes (KEGG) annotations were also made.

### SUPPLEMENTAL MATERIAL FIGURE AND TABLES


