# Supplementary material for: Genome-wide DNA methylation and gene expression patterns provide insight into polycystic ovary syndrome development
Source: Oncotarget. 2014 Jul 16;5(16):6603–10. doi: 10.18632/oncotarget.2224 (PMC4196149; doi:10.18632/oncotarget.2224)
Supplement: Supplementary file 1 [file oncotarget-05-6603-s001.pdf]

Genome-wide DNA methylation and gene expression patterns provide insight into polycystic ovary syndrome development

Supplementary Material

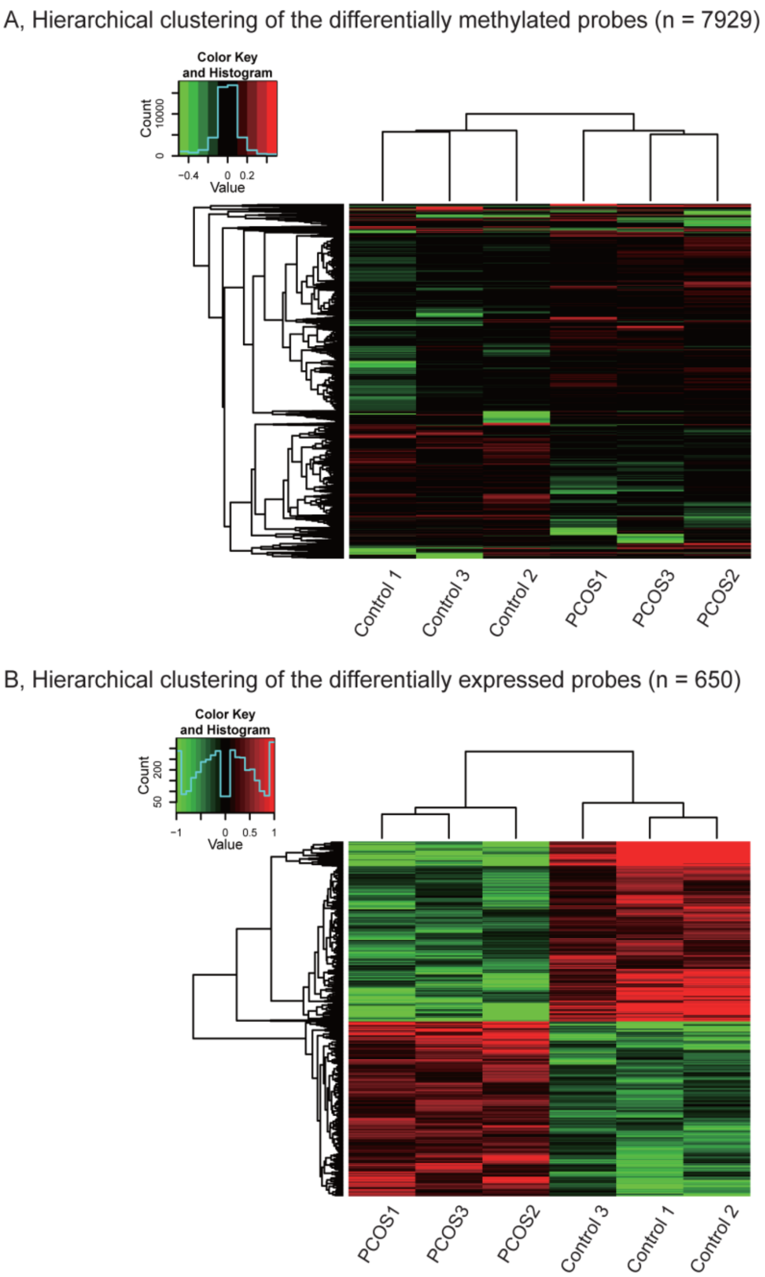

Supplemental Figure 1: Hierarchical clustering of the differentially methylated probes and expressed probes
