# Supplementary material for: Genome-wide DNA methylation and gene expression patterns provide insight into polycystic ovary syndrome development
Source: Oncotarget. 2014 Jul 16;5(16):6603–10. doi: 10.18632/oncotarget.2224 (PMC4196149; doi:10.18632/oncotarget.2224)
Supplement: Supplementary file 2 [file oncotarget-05-6603-s002.pdf]

Supplementary Table 1. Differentially methylated CpG sites between normal ovaries and PCOS ovaries

Index	TargetID	ProbeID_A	ProbeID_B	Control.AV	Control.Dif	Control.Del	PCOS.AV	PCOS.Diff
98	cg0000407	38766357	38766357	0.4877385	0	1	0.6701809	54.39702
159	cg0000681	18804378	18804378	0.3903461	0	1	0.2755172	-25.25451
224	cg0000930	74728390	74728390	0.3015871	0	1	0.4091758	21.95797
231	cg0000952	42761437	42761437	0.5701457	0	1	0.0714224	-371.3336
329	cg0001365	44675355	31736405	0.2812709	0	1	0.4013246	26.90836
415	cg0001715	53626407	53626407	0.6147654	0	1	0.469742	-34.69786
560	cg0002299	43668467	31767442	0.3937696	0	1	0.2653006	-30.7724
562	cg0002302	37787505	37787505	0.232677	0	1	0.4068173	54.01976
599	cg0002451	15677432	15677432	0.2896788	0	1	0.4033222	24.34162
668	cg0002715	36681337	36681337	0.8852143	0	1	0.7519315	-59.68505
695	cg0002829	38749489	38749489	0.3398032	0	1	0.4454381	20.50223
696	cg0002830	58620505	58620505	0.2323112	0	1	0.1259464	-33.1063
824	cg0003320	35667425	35667425	0.654247	0	1	0.4958171	-41.77448
825	cg0003321	73611364	73611364	0.0963077	0	1	0.2558973	69.0565
831	cg0003349	67770302	67770302	0.5435941	0	1	0.7134237	50.91593
882	cg0003544	51625403	51625403	0.6984555	0	1	0.5373095	-45.47
903	cg0003613	62807489	62807489	0.6000488	0	1	0.700279	21.05247
946	cg0003802	71732358	71732358	0.5287921	0	1	0.6662758	33.27204
976	cg0003948	26722425	26722425	0.1949387	0	1	0.3052893	27.94724
993	cg0004010	63772389	63772389	0.8424819	0	1	0.7041904	-51.85731
1008	cg0004053	34668426	34668426	0.6787458	0	1	0.9038784	138.4738
1113	cg0004507	61760432	61760432	0.6724313	0	1	0.7748435	25.88484
1166	cg0004769	54689478	54689478	0.2974049	0	1	0.4016206	20.98347
1235	cg0005115	70761365	70761365	0.7306336	0	1	0.3669483	-220.0996
1344	cg0005643	13810448	13810448	0.5771261	0	1	0.4552421	-25.3231
1380	cg0005800	47670413	34681339	0.6615468	0	1	0.5506545	-23.26248
1460	cg00061551	72748427	72748427	0.6708563	0	1	0.8283972	60.10462
1517	cg0006398	53716300	53716300	0.6853769	0	1	0.8041213	35.63484
1556	cg0006623	46751326	46751326	0.2044397	0	1	0.3116123	26.06163
1642	cg00069771	30764342	30764342	0.9147561	0	1	0.6208712	-213.0645
1933	cg0008379	46801467	13659322	0.2334792	0	1	0.3459002	26.30115
1944	cg00084271	36640339	36640339	0.6616695	0	1	0.7781184	31.87796
2173	cg0009347	16757450	14611473	0.5856227	0	1	0.466532	-24.44925
2228	cg0009525	60656333	60656333	0.3817442	0	1	0.517468	30.37065
2312	cg0009818	47651469	50685321	0.4850096	0	1	0.8497164	374.3439
2371	cg0010115	30746351	30746351	0.2640281	0	1	0.3892573	29.57645
2372	cg0010122	59747444	59747444	0.5819107	0	1	0.6917845	23.97051
2380	cg0010160	33735361	33735361	0.2292879	0	1	0.3313853	22.81434
2470	cg0010656	74721382	74721382	0.6489338	0	1	0.7792793	38.17567
2530	cg0010894	24657395	24657395	0.2200168	0	1	0.1174222	-32.48067
2741	cg0011878	71730440	30680362	0.1737434	0	1	0.2995465	36.38452

2767	cg00119778	65649460	65649460	0.1914038	0	1	0.3250918	38.1762
2807	cg00121626	19779393	19779393	0.0492732	0	1	0.1891522	71.8237
2811	cg00121876	11641490	11641490	0.5379918	0	1	0.6982139	45.01088
2825	cg00122779	50730351	50730351	0.6064615	0	1	0.8073435	82.9363
2842	cg00123214	16683390	16683390	0.6684531	0	1	0.7865573	33.41087
2870	cg00124902	66794348	66794348	0.5088089	0	1	0.3622006	-34.95581
2873	cg00124993	26637438	24755330	0.5281388	0	1	0.4094916	-24.03202
2889	cg00125893	31787444	31787444	0.7427309	0	1	0.6356573	-25.48688
2948	cg00128718	64701377	64701377	0.8697295	0	1	0.6599032	-108.8322
3090	cg00136547	12733385	12733385	0.1711139	0	1	0.3477179	63.16172
3277	cg00146240	44697466	44697466	0.7899763	0	1	0.4230724	-237.9115
3348	cg00149537	28618375	52810459	0.6130087	0	1	0.7180613	23.43864
3466	cg00154902	59771461	71747396	0.6442125	0	1	0.7989946	53.121
3510	cg00156497	74780459	74780459	0.9142308	0	1	0.7955391	-59.92753
3525	cg00157016	54695379	54695379	0.3825853	0	1	0.5304716	35.2604
3530	cg00157199	67794389	67704375	0.4445814	0	1	0.6982733	102.7366
3682	cg00163702	59703408	59703408	0.2837618	0	1	0.4388668	41.08584
3739	cg00166722	55760432	14676335	0.767555	0	1	0.6264921	-41.94953
3748	cg00167248	69734324	69734324	0.7318605	0	1	0.5775554	-44.79804
3756	cg00167670	47678333	47678333	0.5687125	0	1	0.6990626	31.95439
3764	cg00167913	34621465	34621465	0.6590453	0	1	0.800259	46.11084
3835	cg00171092	47771303	47771303	0.2128353	0	1	0.3149059	23.67109
3871	cg00172603	50632452	73639386	0.3547425	0	1	0.2439154	-25.1876
4081	cg00182893	64744407	64744407	0.7273014	0	1	0.8796801	70.548
4160	cg00187244	54743510	54743510	0.3251532	0	1	0.0850805	-134.3289
4251	cg00192882	58670408	58670408	0.1446309	0	1	0.2477272	29.41486
4434	cg00204802	64623441	47756405	0.4341714	0	1	0.6211633	54.7084
4467	cg00206507	70732476	70732476	0.5442189	0	1	0.6689163	28.40732
4516	cg00209612	27678431	27678431	0.6774021	0	1	0.8240893	52.9756
4543	cg00210856	12772328	12772328	0.5153254	0	1	0.6204551	20.42368
4550	cg00211215	12790319	39622342	0.6676571	0	1	0.3398855	-169.9291
4566	cg00212031	29674443	38703326	0.1133291	0	1	0.3524014	121.9356
4601	cg00213805	71737411	71737411	0.4638245	0	1	0.3373573	-27.57928
4693	cg00218628	60625336	60625336	0.4282582	0	1	0.6003063	46.57256
4753	cg00221185	54678344	24788422	0.2144859	0	1	0.337492	31.61109
4776	cg00222015	17719383	17719383	0.6054494	0	1	0.4968444	-21.26645
4797	cg00223245	50693357	50693357	0.4734471	0	1	0.6036795	28.67913
4919	cg00230120	14662484	19720437	0.7819089	0	1	0.8837823	40.13985
4957	cg00231519	19627374	19627374	0.4116288	0	1	0.7533038	374.3439
4980	cg00232388	35709315	35709315	0.6401244	0	1	0.9173192	374.3439
5022	cg00234370	48684425	48684425	0.1582235	0	1	0.2792385	35.90932
5050	cg00235754	28719502	28719502	0.724811	0	1	0.8266135	30.59868
5141	cg00239742	51742392	51742392	0.6707383	0	1	0.7900235	34.29369

5168	cg00241002	54771452	57737407	0.2905654	0	1	0.4230091	31.22339
5186	cg00241907	43619318	10732399	0.4018812	0	1	0.2754538	-29.51978
5252	cg00244920	47710335	47710335	0.6551744	0	1	0.4654171	-57.52374
5296	cg00247094	22631327	22631327	0.4299978	0	1	0.6470946	73.29041
5332	cg00248861	64710346	64710346	0.357409	0	1	0.6233817	108.2252
5435	cg00253735	60729344	60729344	0.4596887	0	1	0.2940491	-45.48247
5443	cg00254095	69780487	69780487	0.5708256	0	1	0.7835477	85.92299
5446	cg00254306	54748461	17645336	0.4332845	0	1	0.3230464	-22.29561
5482	cg00255882	63789443	63789443	0.3060787	0	1	0.1953582	-28.06241
5486	cg00256046	73700305	73700305	0.6378322	0	1	0.7757366	41.34131
5497	cg00256329	34663423	34663423	0.6128048	0	1	0.7193904	24.04136
5572	cg00259849	15806472	15806472	0.6960363	0	1	0.9377137	374.3439
5770	cg00269725	67766493	67766493	0.6012082	0	1	0.840215	121.0875
5771	cg00269800	61752345	61752345	0.3269524	0	1	0.2234852	-23.58945
5802	cg00271873	11808416	11808416	0.0068376	0	1	0.2126269	153.5253
5810	cg00272795	74632377	74632377	0.5579413	0	1	0.6697971	23.85066
5811	cg00272903	41724510	41724510	0.5025862	0	1	0.6065403	19.83427
5840	cg00274640	47758438	47758438	0.6215541	0	1	0.4705952	-37.36186
5858	cg00275828	19804392	19804392	0.7183216	0	1	0.8323202	37.13152
5914	cg00279755	13644355	13644355		0	1		-4.34E-09
5952	cg00281837	56772418	56772418	0.6166537	0	1	0.4934157	-26.38166
6017	cg00286102	10622467	10622467	0.7655643	0	1	0.8761911	43.42421
6059	cg00287841	34718404	34718404	0.7733057	0	1	0.5908943	-64.77754
6070	cg00288598	34722501	44649372	0.3508348	0	1	0.5102373	40.70222
6110	cg00290607	64718396	62628499	0.5104077	0	1	0.245746	-112.7841
6157	cg00292662	13644450	13644450		0	1	0.1353777	4.34E-09
6201	cg00294550	52807349	52807349	0.3588103	0	1	0.4835544	26.57167
6225	cg00295418	41681387	41681387	0.5740335	0	1	0.7436702	53.73215
6260	cg00296903	27749328	27749328	0.6293271	0	1	0.7333634	23.84962
6278	cg00297876	24757500	24757500	0.4720151	0	1	0.2497136	-80.97279
6381	cg00302763	60641446	60641446	0.4486659	0	1	0.5543896	19.87812
6418	cg00304520	27747375	37645353	0.0170795	0	1	0.1268006	64.10091
6572	cg00311883	43616362	62666317	0.1853134	0	1	0.3118581	35.55143
6605	cg00314000	54769436	54769436	0.6604575	0	1	0.7701944	28.41892
6684	cg00318166	30729329	30729329	0.8876944	0	1	0.775729	-47.02844
6699	cg00318631	64702450	64702450	0.6962287	0	1	0.5672875	-31.24535
6757	cg00321007	34743356	34743356	0.2244426	0	1	0.3250996	22.55418
6777	cg00321709	29758495	29758495	0.3482544	0	1	0.5029081	38.6384
6844	cg00324979	11750373	59799481	0.7907788	0	1	0.6015454	-71.90366
6846	cg00325125	73798400	39785417	0.3920734	0	1	0.2261386	-50.36866
6847	cg00325139	31648385	31648385	0.6550539	0	1	0.7630957	27.20718
6860	cg00325917	14694367	14694367	0.6974657	0	1	0.8516454	63.48054
6911	cg00328219	72703382	72703382	0.4842268	0	1	0.6437661	41.86397

6919	cg0032859[illegible]	38675330	38675330	0.8108135	0	1	0.9215826	57.23984
6931	cg0032891[illegible]	72644443	72644443	0.931043	0	1	0.7796789	-92.78088
7171	cg0033948[illegible]	50602324	21801407	0.3874254	0	1	0.2324567	-44.38596
7207	cg0034106[illegible]	18773370	41686495	0.3376615	0	1	0.2062459	-35.81312
7317	cg0034662[illegible]	47697349	29663318	0.6527706	0	1	0.5289307	-27.53068
7340	cg0034762[illegible]	51782356	65801309	0.6607138	0	1	0.7848387	35.92228
7359	cg0034803[illegible]	20645464	20645464	0.8101259	0	1	0.6225847	-74.73534
7468	cg0035257[illegible]	46794399	46794399	0.517943	0	1	0.640379	26.65797
7481	cg0035340[illegible]	29767306	29767306	0.9285453	0	1	0.8022724	-71.74111
7497	cg0035438[illegible]	53661303	53661303	0.2916796	0	1	0.4594197	46.61121
7685	cg0036375[illegible]	73802349	73802349		0	1	0.3235186	4.34E-09
7745	cg0036660[illegible]	71632347	71632347	0.3948264	0	1	0.5678179	46.72945
7795	cg0036905[illegible]	22723346	22723346	0.4061009	0	1	0.5156639	21.05602
7837	cg0037041[illegible]	68769470	68769470	0.2537548	0	1	0.3615871	23.68404
7845	cg0037106[illegible]	70750478	70750478	0.3083043	0	1	0.4087651	19.53635
7897	cg0037343[illegible]	35689309	35689309	0.5450431	0	1	0.7143257	50.71206
7911	cg0037401[illegible]	34614467	34614467	0.4690894	0	1	0.3394035	-28.71997
8081	cg0038175[illegible]	35726399	51656485	0.2326673	0	1	0.1054444	-47.22473
8126	cg0038470[illegible]	45767476	45767476	0.497659	0	1	0.3572219	-32.52185
8235	cg0039019[illegible]	12611477	12611477	0.8008755	0	1	0.6807278	-35.89886
8332	cg0039471[illegible]	24737432	24737432		0	1		-4.34E-09
8413	cg0039804[illegible]	15783450	15783450	0.2432983	0	1	0.3720044	32.04579
8449	cg0039995[illegible]	24614302	24614302	0.3313107	0	1	0.4366068	20.54902
8486	cg0040110[illegible]	36612418	59687450	0.5505452	0	1	0.4457809	-19.56911
8535	cg0040347[illegible]	62610321	62610321	0.4293275	0	1	0.3234897	-20.87441
8548	cg0040418[illegible]	59806369	59806369	0.5678366	0	1	0.4353766	-29.0793
8588	cg0040601[illegible]	60735327	60735327	0.5489819	0	1	0.6625613	24.20676
8633	cg0040823[illegible]	57607304	33655303	0.6827136	0	1	0.5681886	-25.24189
8692	cg0041089[illegible]	41650376	41650376	0.2531839	0	1	0.1177812	-49.32533
8738	cg0041335[illegible]	40637333	40637333	0.5117602	0	1	0.6875343	52.00707
8774	cg0041538[illegible]	36702431	36702431	0.7964512	0	1	0.456613	-205.6306
9085	cg0042845[illegible]	36769351	65619420	0.6925805	0	1	0.5923864	-20.75856
9233	cg0043696[illegible]	51693491	51693491	0.6664475	0	1	0.50984	-41.49477
9293	cg0044079[illegible]	46608309	46608309	0.1441054	0	1	0.2595815	35.0344
9483	cg0045002[illegible]	70611456	70611456	0.5669092	0	1	0.7607941	69.78075
9619	cg0045634[illegible]	16712478	16712478	0.8580703	0	1	0.541483	-203.6598
9682	cg0045893[illegible]	69765306	69765306	0.1242167	0	1	0.244417	40.11396
9703	cg0045962[illegible]	19772395	27705498	0.1675778	0	1	0.0292722	-79.92907
9817	cg0046374[illegible]	55797393	55797393	0.3337444	0	1	0.4375008	20.02645
9866	cg0046611[illegible]	36716493	36716493	0.1775915	0	1	0.4147727	102.4503
9894	cg0046727[illegible]	57640486	71694449	0.7709463	0	1	0.9259002	92.6925
10020	cg0047284[illegible]	20810380	20810380	0.4264116	0	1	0.5842133	39.66524
10059	cg0047409[illegible]	13684467	13684467	0.6616473	0	1	0.4905437	-48.13664

10065	cg00474373	72662453	72662453	0.381675	0	1	0.685994	146.1217
10159	cg00478479	38625510	38625510	0.5106996	0	1	0.4068238	-19.2938
10177	cg00479827	45616421	45616421	0	0	1	0.1308355	91.91531
10295	cg00486259	67693416	67693416	0.444092	0	1	0.5632187	24.26474
10344	cg00488829	41699391	28727411	0.1401444	0	1	0.0328822	-55.27039
10448	cg00493304	45680342	44609483	0.9227313	0	1	0.766117	-92.24722
10554	cg00497909	62600457	62600457	0.1753982	0	1	0.2959976	33.88949
10583	cg00499139	38801390	38801390	0.565307	0	1	0.6920523	30.17727
10613	cg00500027	48607448	48607448	0.4419087	0	1	0.5569382	22.85472
10753	cg00505001	47660336	47660336	0.4624488	0	1	0.5719085	21.1501
10796	cg00507055	33615313	33615313	0.8909215	0	1	0.5396249	-264.5838
10872	cg00510320	60780470	60780470	0.2356981	0	1	0.3564608	29.3422
10887	cg00510718	38763374	38763374	0.4743826	0	1	0.5809178	20.31512
10898	cg00511334	20665459	20665459	0.3095471	0	1	0.427974	25.3995
11134	cg00523604	70747313	70747313	0.1748342	0	1	0.2767201	26.14756
11135	cg00523628	39768400	39768400	0.5779462	0	1	0.7304254	43.79782
11150	cg00524374	56689304	56689304	0.2831903	0	1	0.401425	26.18002
11185	cg00525828	73696304	22732478	0.9839413	0	1	0.714254	-253.0507
11252	cg00529742	39696318	39696318	0.2902426	0	1	0.4325881	35.23641
11303	cg00532122	61800426	61800426	0.0524256	0	1	0.503257	374.3439
11354	cg00534215	61781303	50644369	0.9852603	0	1	0.5683572	-371.3336
11453	cg00539360	59744470	59744470	0.3836901	0	1	0.4886221	19.74508
11470	cg00540295	37809366	37809366	0.6904176	0	1	0.4131027	-121.3784
11552	cg00544032	23632499	23632499	0.3385054	0	1	0.4891903	37.13638
11597	cg00546757	74625369	74625369	0.4546407	0	1	0.8299717	374.3439
11795	cg00555085	42616447	42616447	0.2194534	0	1	0.3229758	23.82449
11803	cg00555438	12606324	12606324	0.0938143	0	1	0.2525676	69.23317
11940	cg00562262	13644472	13644472		0	1	0.7723164	4.34E-09
11941	cg00562265	74692473	62764304	0.1954326	0	1	0.0912162	-37.49272
11970	cg00563352	54631509	54631509	0.5267427	0	1	0.6363078	22.15691
11979	cg00563824	42786475	62697453	0.1597987	0	1	0.0488238	-52.07261
12030	cg00566187	64810375	64810375	0.6199332	0	1	0.9046483	374.3439
12050	cg00566982	54768470	54768470	0.4335096	0	1	0.5363402	18.94921
12089	cg00569101	11653362	11653362	0.7706046	0	1	0.9408507	114.9219
12123	cg00570635	17775315	17775315	0.2787165	0	1	0.3878819	23.18136
12145	cg00571519	45708350	45708350	0.5151454	0	1	0.413296	-18.66733
12165	cg00572586	55740317	55740317	0.2644066	0	1	0.0938616	-77.10144
12185	cg00573410	69654380	69654380	0.5319522	0	1	0.6499016	25.33753
12240	cg00575645	16668481	16668481	0.5604975	0	1	0.7242595	48.77661
12257	cg00576139	22668323	22668323	0.5659805	0	1	0.6673153	20.31834
12519	cg00586527	54681481	17654386	0.6761359	0	1	0.5498232	-29.30053
12535	cg00587301	23781313	23781313	0.6486195	0	1	0.7500512	23.85412
12537	cg00587342	39716491	39716491	0.4286765	0	1	0.5290638	18.21761

12550	cg0058794[illegible]	55710466	28715476	0.6664951	0	1	0.9265002	374.3439
12577	cg0058892[illegible]	70627410	70627410	0.1391872	0	1	0.2802477	48.55938
12732	cg0059505[illegible]	15623412	15623412	0.4701355	0	1	0.3527191	-24.11751
12788	cg0059820[illegible]	37644350	37644350	0.3284011	0	1	0.4475105	25.16149
12858	cg0060145[illegible]	48675301	10730370	0.1295909	0	1	0.2765968	53.54831
13060	cg0061275[illegible]	14741434	14741434	0.6286693	0	1	0.7398571	26.85531
13137	cg0061657[illegible]	49789428	49789428	0.7180879	0	1	0.5802696	-36.20538
13229	cg0062155[illegible]	41739321	41739321	0.3986572	0	1	0.504959	20.06866
13233	cg0062166[illegible]	67737500	67737500	0.2662944	0	1	0.1503767	-34.4824
13356	cg0062821[illegible]	55631375	55631375	0.7837675	0	1	0.8869645	41.6381
13423	cg0063132[illegible]	61711456	61711456	0.2644017	0	1	0.0953791	-75.59556
13428	cg0063175[illegible]	42626386	42626386	0.6123421	0	1	0.8420122	114.1186
13430	cg0063187[illegible]	61665304	61665304	0.9072573	0	1	0.637906	-182.2036
13476	cg0063465[illegible]	52795423	41801408	0.1474447	0	1	0.2733383	39.56255
13485	cg0063534[illegible]	45612424	45612424	0.5873916	0	1	0.7038772	26.86475
13504	cg0063619[illegible]	62744391	62744391	0.3024641	0	1	0.4207427	25.55326
13547	cg0063802[illegible]	71783431	71783431	0.2866273	0	1	0.4059949	26.46232
13578	cg0063961[illegible]	64697321	64697321	0.5713869	0	1	0.4034761	-44.2562
13874	cg0065555[illegible]	27604337	27604337	0.3895652	0	1	0.4944894	19.69985
14054	cg0066445[illegible]	16703404	16703404	0.1884971	0	1	0.3289084	41.60725
14127	cg0066778[illegible]	36621334	42661429	0.2047575	0	1	0.086431	-46.28261
14134	cg0066794[illegible]	51734472	51734472	0.8212805	0	1	0.9345529	64.79012
14141	cg0066821[illegible]	47690491	47690491	0.5186922	0	1	0.6734148	40.98476
14276	cg0067533[illegible]	68697397	68697397	0.2694949	0	1	0.3834738	25.18804
14355	cg0067962[illegible]	47712346	47712346	0.3294179	0	1	0.1914336	-40.15641
14606	cg0069194[illegible]	53710382	53710382	0.4040763	0	1	0.5112553	20.31585
14655	cg0069404[illegible]	56600501	56600501	0.7070803	0	1	0.8492287	55.57246
14662	cg0069435[illegible]	61688483	61688483	0.7468158	0	1	0.932932	123.0061
14701	cg0069604[illegible]	51669430	51669430	0.574074	0	1	0.7415552	52.36635
14767	cg0069877[illegible]	31681444	63692413	0.4114547	0	1	0.2395079	-52.24946
14819	cg0070096[illegible]	15679389	15679389	0.3057416	0	1	0.1840003	-33.56726
14859	cg0070256[illegible]	19740489	19740489	0.6008085	0	1	0.2466059	-204.8976
15202	cg0071826[illegible]	53605491	53605491	0.4853301	0	1	0.6194556	30.46602
15263	cg0072058[illegible]	60679475	73642307	0.5321772	0	1	0.4103576	-25.12515
15336	cg0072431[illegible]	60657423	60657423	0.5194259	0	1	0.6650076	36.57686
15344	cg0072514[illegible]	49647457	13676315	0.6071774	0	1	0.7252793	28.61739
15384	cg0072777[illegible]	72788303	72788303	0.7628074	0	1	0.9101859	78.89736
15423	cg0072970[illegible]	15681478	15681478	0.3841349	0	1	0.5006087	23.42867
15522	cg0073397[illegible]	58790502	58790502	0.6241071	0	1	0.778683	50.05071
15567	cg0073588[illegible]	19649370	23759389	0.4380135	0	1	0.3367122	-19.22618
15620	cg0073884[illegible]	73775371	38801335	0.638712	0	1	0.5344118	-20.52921
15654	cg0074051[illegible]	31607333	31607333	0.6839408	0	1	0.4819172	-66.28573
15772	cg0074585[illegible]	28753437	28753437	0.8517084	0	1	0.9545701	67.32502

15803	cg0074747	73744465	73744465	0.5113566	0	1	0.6145996	19.74185
15807	cg0074765	11786475	19759505	0.9510524	0	1	0.7137111	-184.815
15862	cg00750481	35758454	35758454	0.2242119	0	1	0.3601344	36.34761
15921	cg0075354	23677356	23677356	0.1365604	0	1	0.2630899	41.41857
15933	cg0075392	13628431	13628431	0.3443798	0	1	0.4725431	28.05628
15955	cg0075498	16601458	16601458	0.6120485	0	1	0.7285987	28.21721
16036	cg0075905	52808361	52808361	0.65514	0	1	0.805392	51.67983
16121	cg0076283	66714317	68648306	0.6449261	0	1	0.5125805	-30.47187
16162	cg00764981	12689492	12689492	0.73082	0	1	0.8457513	39.74984
16230	cg0076817	17658469	17658469	0.6121849	0	1	0.7156454	22.7749
16431	cg0077756	58611311	58611311	0.6606444	0	1	0.8075203	50.19828
16459	cg0077905	11738337	36742405	0.6415886	0	1	0.7601179	31.20117
16550	cg0078313	19738414	19738414	0.4193072	0	1	0.316181	-20.18185
16577	cg00784581	47661355	23706371	0.5830381	0	1	0.4750648	-20.78384
16602	cg00785941	52804397	52804397	0.1426994	0	1	0.2737515	42.7915
16641	cg0078799	72773359	72773359	0.3611874	0	1	0.4618725	18.69161
16649	cg0078817	10758443	27641454	0.3711234	0	1	0.2314366	-37.45719
16776	cg0079393	36619332	72628509	0.1528303	0	1	0.30523	52.45032
16782	cg0079399	29629398	29629398	0.8292232	0	1	0.6447983	-77.18924
16803	cg00794911	25661471	25661471	0.3818859	0	1	0.5499886	44.36048
16809	cg0079520	56635320	56635320	0.146375	0	1	0.2582214	33.09596
16975	cg0080424	54773334	45725488	0.5166212	0	1	0.6194642	19.70126
17196	cg0081421	64602355	64602355	0.4157308	0	1	0.6547706	88.50002
17251	cg0081651	14806404	14806404	0.5409213	0	1	0.6637129	27.51303
17335	cg0082118	70683476	70683476	0.7822867	0	1	0.584215	-75.94869
17351	cg0082203	18798349	10688394	0.7912116	0	1	0.6584904	-40.6733
17487	cg0082964	61646498	38687433	0.6045564	0	1	0.7089638	22.80337
17547	cg0083213	62629352	62629352	0.2065738	0	1	0.3081315	23.84835
17630	cg00836101	33668367	33668367	0.3909716	0	1	0.5580688	43.8467
17664	cg0083789	55725467	55725467	0.5070915	0	1	0.6213215	23.37319
17674	cg0083842	16654482	16654482	0.1840394	0	1	0.2916504	27.68867
17763	cg0084363	68647386	68647386	0.7016295	0	1	0.5976094	-22.36484
17860	cg0084837	19743484	19743484	0.597561	0	1	0.699817	21.70268
17934	cg0085151	47624412	47624412	0.1765089	0	1	0.2927083	31.87593
17956	cg0085260	47669404	30621348	0.2964385	0	1	0.3992281	20.55665
17984	cg0085394	60716487	60716487	0.2127016	0	1	0.345275	35.78688
18101	cg0085987	32640506	32640506	0.6921458	0	1	0.9145197	142.5562
18125	cg0086121	49625401	49625401	0.3880026	0	1	0.5745876	53.79526
18193	cg00864171	52799325	52799325	0.4539747	0	1	0.3164589	-32.31712
18309	cg0086930	71621326	71621326	0.9080755	0	1	0.6845008	-139.5938
18401	cg00873601	18668468	60679306	0.4840441	0	1	0.3253206	-41.03856
18483	cg0087675	43778390	41784387	0.490046	0	1	0.3892749	-18.4576
18507	cg0087816	39764486	58726385	0.6287758	0	1	0.4911793	-32.04788

18523	cg0087921	19790496	19790496	0.7853533	0	1	0.6714219	-31.4803
18527	cg0087949	49788354	13644469		0	1	0.6680779	4.34E-09
18528	cg0087954	47671476	47671476	0.4011107	0	1	0.604714	63.69984
18574	cg0088189	68677465	68677465	0.4249568	0	1	0.9164275	374.3439
18646	cg0088657	15612500	15612500	0.3214796	0	1	0.4399989	25.1193
18688	cg0088875	24721332	24721332	0.6842133	0	1	0.7940284	30.55881
18712	cg0089007	63730395	63730395	0.3654357	0	1	0.4662722	18.68779
18756	cg0089236	48731416	51671432	0.507829	0	1	0.4020602	-19.88889
18894	cg0090073	29702359	14789326	0.8092887	0	1	0.9216885	58.44061
18998	cg0090531	64770334	64770334	0.2555588	0	1	0.3640394	23.8325
19003	cg0090552	33623413	24656365	0.4397592	0	1	0.3105673	-29.27958
19006	cg0090574	62698386	62698386	0.4424376	0	1	0.3359763	-20.82791
19087	cg0090860	70794326	70794326	0.5711282	0	1	0.4688715	-18.93096
19222	cg0091395	72679382	72679382	0.6058694	0	1	0.7599317	47.48814
19300	cg0091818	70622484	70622484	0.6018964	0	1	0.2456335	-207.6492
19315	cg0091912	14642320	14642320	0.4832474	0	1	0.5976112	23.01564
19353	cg0092066	36693338	23620361	0.5525063	0	1	0.6759126	28.19801
19447	cg0092508	70796393	70796393	0.1868355	0	1	0.3216949	39.17377
19474	cg0092642	61804339	73696387	0.2358557	0	1	0.3895555	43.50841
19757	cg0093968	23660333	23660333	0.3908856	0	1	0.5946859	63.68467
19852	cg0094463	33644501	33644501	0.5413433	0	1	0.8024355	127.6763
19916	cg0094778	22655509	11725434	0.0255214	0	1	0.1703407	87.0724
19963	cg0094998	23653380	50799380	0.4252528	0	1	0.312909	-23.23013
20014	cg0095278	20734465	20734465	0.3781765	0	1	0.4996309	25.17802
20082	cg0095578	48794300	48794300	0.6264518	0	1	0.4875776	-32.49273
20100	cg0095649	30773316	30773316	0.7737476	0	1	0.6698388	-26.38488
20123	cg0095769	27697394	27697394	0.3202077	0	1	0.4729107	38.5239
20347	cg0096816	41790387	41790387	0.262027	0	1	0.3717615	24.00037
20363	cg0096877	64794380	16624467	0.6011539	0	1	0.500222	-18.84724
20395	cg0097027	38750401	43783437	0.3350363	0	1	0.1992646	-38.37355
20478	cg0097452	12731470	12731470	0.4506799	0	1	0.5616955	21.57428
20480	cg0097457	37653347	37653347	0.6202055	0	1	0.5128465	-21.11871
20506	cg0097574	57691312	57691312	0.1209652	0	1	0.2862327	66.24072
20569	cg0097884	52680431	52680431	0.3957377	0	1	0.5690189	46.87591
20586	cg0097928	64652323	10616422	0.443745	0	1	0.3079751	-31.89791
20651	cg0098187	15731476	28704329	0.5849195	0	1	0.4045814	-50.54544
20680	cg0098298	48702333	48702333	0.61374	0	1	0.4788722	-30.62597
20689	cg0098369	51787353	51787353	0.6497052	0	1	0.7716147	33.62696
20690	cg0098370	22623502	22623502	0.8175781	0	1	0.9276887	59.29258
20721	cg0098538	36700401	36700401	0.1408757	0	1	0.2413798	28.69344
20774	cg0098805	37707449	31720502	0.7761006	0	1	0.573949	-77.47209
20836	cg0099076	68787438	68787438	0.3733887	0	1	0.4814401	20.80149
20907	cg0099383	67633457	67633457	0.6469377	0	1	0.7486776	23.88445

20909	cg00993903	26725474	26725474	0.7580072	0	1	0.6445808	-28.96066
20953	cg00995368	49810395	49810395	0.7723302	0	1	0.614825	-50.75345
20995	cg00997251	40617310	40617310	0.259348	0	1	0.3639905	22.36749
21042	cg00999163	34802473	57752377	0.2487538	0	1	0.4115882	46.90366
21049	cg00999638	58704438	58704438	0.7710326	0	1	0.6629233	-27.83346
21074	cg01000657	67673486	35768392	0.147542	0	1	0.0173908	-80.01437
21086	cg01001314	49793311	49793311	0.7203438	0	1	0.8417692	42.45524
21093	cg01001959	57762422	57762422	0.4722784	0	1	0.3706741	-18.86017
21158	cg01005180	72747446	72747446	0.4078679	0	1	0.5138558	19.93423
21166	cg01005425	13730504	13730504	0.639474	0	1	0.7681657	36.28926
21169	cg01005486	49765459	49765459	0.2629581	0	1	0.1562112	-29.79357
21191	cg01006802	39651497	64691402	0.3083982	0	1	0.2007137	-26.50354
21317	cg01013031	35631361	35690419	0.5807728	0	1	0.795859	90.15868
21376	cg01015662	21666465	26648376	0.6919252	0	1	0.5058638	-57.78933
21384	cg01016092	21694469	21694469	0.5326455	0	1	0.36054	-46.5186
21528	cg01022117	17782395	42638353	0.4536819	0	1	0.5545027	18.40499
21531	cg01022189	25786466	25786466	0.9164178	0	1	0.800821	-58.60995
21544	cg01022670	15660474	15660474	0.5954645	0	1	0.7630956	54.96465
21559	cg01023242	67743501	67743501	0.7373105	0	1	0.6250218	-27.11636
21569	cg01023703	33783394	33783394	0.2909643	0	1	0.3981129	22.11601
21604	cg01025095	68672467	64763345	0.5315912	0	1	0.3736983	-39.68333
21693	cg01029331	73787312	14657451	0.9667825	0	1	0.7639384	-166.9801
21707	cg01029685	63766436	63766436	0.3929411	0	1	0.2905731	-20.58465
21741	cg01031312	11690361	11690361	0.3223857	0	1	0.4388436	24.38768
21803	cg01034813	28693312	28693312	0.5059395	0	1	0.6539437	37.15122
21945	cg01042202	43649477	21654418	0.8294322	0	1	0.6535847	-71.64841
22088	cg01048272	66719465	66719465	0.6311114	0	1	0.5056903	-27.49313
22249	cg01055691	63796358	63796358	0.7108428	0	1	0.8203871	33.37178
22251	cg01055696	51629480	25614359	0.7707782	0	1	0.6627012	-27.80069
22272	cg01056467	10629329	16649394	0.3430042	0	1	0.2116207	-35.35745
22327	cg01059116	69731430	69731430	0.5559142	0	1	0.3966959	-40.15797
22332	cg01059385	61780487	61780487	0.3570359	0	1	0.2142153	-40.24322
22334	cg01059432	37645427	38781310	0.3565813	0	1	0.2547905	-21.59309
22368	cg01061164	34769367	25702481	0.7413358	0	1	0.8923135	74.08971
22373	cg01061520	61760464	61760464	0.3114851	0	1	0.1932178	-31.28918
22400	cg01062539	57764394	21718483	0.6619301	0	1	0.7643456	25.13253
22410	cg01062942	52752434	52752434	0.1040089	0	1	0.2456348	55.84582
22413	cg01063088	53684349	53684349	0.1687982	0	1	0.3097011	44.14992
22435	cg01064265	58621422	51611437	0.4557274	0	1	0.3073374	-37.10577
22616	cg01072388	62787373	62787373	0.4063252	0	1	0.5261592	24.44593
22650	cg01074083	17770500	17770500	0.655261	0	1	0.8867612	134.6604
22824	cg01081584	63773393	63773393	0.7438583	0	1	0.2739802	-371.3336
22920	cg01085399	34667319	34667319	0.5902252	0	1	0.7186599	32.31227

23150	cg0109744[illegible]	13617312	37610325		0	1	0.017107	4.34E-09
23253	cg0110166[illegible]	57773374	57773374	0.454101	0	1	0.3256346	-28.58717
23254	cg0110167[illegible]	35652338	35652338	0.5880892	0	1	0.7054773	27.27685
23339	cg0110540[illegible]	58625305	58625305	0.7139421	0	1	0.3134831	-271.9647
23403	cg0110863[illegible]	52758389	52758389	0.4574442	0	1	0.5730567	23.15359
23523	cg0111413[illegible]	71625399	71625399	0.5532788	0	1	0.702067	39.96653
23579	cg0111727[illegible]	24632368	24632368	0.7418416	0	1	0.5774972	-50.7658
23602	cg0111864[illegible]	72739319	72739319	0.5072579	0	1	0.2260535	-129.6227
23703	cg0112413[illegible]	53638343	53638343	0.7368794	0	1	0.6176736	-29.77578
23756	cg0112760[illegible]	24715331	24715331	0.233899	0	1	0.0879151	-62.68643
23762	cg0112804[illegible]	18724455	18724455	0.5443834	0	1	0.6651385	26.82159
23998	cg0113969[illegible]	45607388	45607388	0.6763784	0	1	0.916365	374.3439
23999	cg0113969[illegible]	16700378	16700378	0.2481685	0	1	0.3549436	23.55949
24270	cg0115262[illegible]	32644411	32644411	0.3067365	0	1	0.4399427	30.93636
24288	cg0115337[illegible]	45738320	50626330	0.3677785	0	1	0.584482	71.64358
24311	cg0115433[illegible]	44652369	44652369	0.6299962	0	1	0.7445893	28.47252
24448	cg0116105[illegible]	62747307	62747307	0.3750246	0	1	0.5085895	29.60917
24543	cg0116590[illegible]	35653429	35653429	0.6443579	0	1	0.7607034	30.34842
24734	cg0117514[illegible]	47769365	47769365	0.4946208	0	1	0.2574361	-90.45715
24872	cg0118207[illegible]	37774380	37774380	0.6685133	0	1	0.7855726	32.85421
24947	cg0118564[illegible]	22706469	22706469	0.3806311	0	1	0.4980236	23.76656
25051	cg0119091[illegible]	14707477	27750351	0.7839438	0	1	0.9178117	72.35423
25059	cg0119115[illegible]	50612384	50612384	0.2017615	0	1	0.317154	29.45092
25106	cg0119336[illegible]	21647360	26755371	0.6902781	0	1	0.5579758	-32.28323
25117	cg0119407[illegible]	72697401	32694336	0.2663782	0	1	0.3758532	23.7383
25129	cg0119476[illegible]	24770508	24770508	0.1637495	0	1	0.2820756	34.07571
25164	cg0119633[illegible]	64764433	64764433	0.2586228	0	1	0.3599216	21.28437
25275	cg0120251[illegible]	59676304	47600463	0.4357339	0	1	0.3027748	-30.97444
25303	cg0120376[illegible]	74635411	74635411	0.3315597	0	1	0.4493576	24.6396
25544	cg01215761	23623339	62672339	0.3909582	0	1	0.271363	-27.13545
25657	cg0122158[illegible]	24784320	24784320	0.8916348	0	1	0.686173	-115.1591
25792	cg01228941	55600356	35613357	0.7909966	0	1	0.4978701	-153.6902
25817	cg0123032[illegible]	18649346	18649346	0.6445942	0	1	0.9360674	374.3439
25824	cg0123063[illegible]	72809365	72809365	0.3362632	0	1	0.4726653	31.34789
25848	cg01231921	35765480	35765480	0.4886275	0	1	0.37521	-22.49986
25856	cg01232331	11624391	11624391	0.5923041	0	1	0.7602555	54.77787
25860	cg01232511	34701365	35621479	0.6021866	0	1	0.491347	-21.9318
25943	cg0123656[illegible]	38743488	38743488	0.5860829	0	1	0.7413703	46.1566
26102	cg0124522[illegible]	70663375	70663375	0.2962722	0	1	0.458753	43.88079
26170	cg0124874[illegible]	44690486	44690486	0.4397899	0	1	0.3009491	-33.37103
26272	cg0125430[illegible]	32766403	50639305	0.1246399	0	1	0.4224883	374.3439
26338	cg0125778[illegible]	25672372	25672372	0.3976262	0	1	0.5113661	22.43646
26468	cg0126491[illegible]	73763380	73763380	0.5032518	0	1	0.6944514	60.83808

26535	cg01269212	37698464	37698464	0.7409231	0	1	0.8651326	48.72203
26553	cg01270001	52631391	43650379	0.1506638	0	1	0.0342277	-61.2644
26642	cg01274907	52722315	52722315	0.6468804	0	1	0.4794667	-45.67482
26800	cg01283332	61774489	61774489	0.7005204	0	1	0.5785991	-28.73744
26819	cg01284289	64720495	64720495	0.4253918	0	1	0.6394984	71.07223
26841	cg01285501	50703500	50703500	0.446228	0	1	0.3398747	-20.72545
26849	cg01285926	28747346	62630489	0.3706437	0	1	0.2571912	-25.51675
26859	cg01286685	67740487	61726375	0.1059447	0	1	0.2243159	42.28858
26864	cg01287000	71733390	38620464	0.4503463	0	1	0.3500149	-18.73097
26927	cg01289544	36615507	36615507	0.3754013	0	1	0.2710961	-21.82288
26935	cg01289874	24806427	24806427	0.6559485	0	1	0.7761502	33.30973
27002	cg01292539	68748466	68748466	0.6726671	0	1	0.7840507	30.2772
27008	cg01292810	50683422	50683422	0.1971644	0	1	0.3188645	32.40123
27041	cg01294283	63706392	63706392	0.2101784	0	1	0.3241834	28.30911
27067	cg01295646	63606406	63606406	0.2833413	0	1	0.1649635	-33.97006
27129	cg01298102	35734489	35734489	0.2307952	0	1	0.3773761	40.5964
27136	cg01298678	12736491	12736491	0.4538442	0	1	0.5619927	20.66872
27143	cg01298912	35809352	35809352	0.9004936	0	1	0.636203	-172.8215
27192	cg01301282	10771424	10771424	0.4275876	0	1	0.3089637	-25.48488
27517	cg01316476	67705342	67705342	0.6811837	0	1	0.5685879	-24.50689
27539	cg01318188	35704452	62802401	0.5825996	0	1	0.467898	-22.94254
27570	cg01320579	46731406	41796418	0.6581322	0	1	0.5253892	-31.07323
27595	cg01321962	47774394	47774394	0.3568466	0	1	0.5055475	35.91703
27634	cg01324264	30671344	30671344	0.5188496	0	1	0.6570737	33.20673
27658	cg01325465	40647340	40647340	0.7453305	0	1	0.8589378	41.45735
27671	cg01326421	32630496	32630496	0.6288849	0	1	0.4421675	-54.79694
27723	cg01329690	30690303	30690303	0.3227202	0	1	0.5065502	53.40446
27948	cg01341801	23754361	23754361	0.0427401	0	1	0.2150719	99.45876
28061	cg01348584	55740334	17663399	0.2795927	0	1	0.407914	30.06989
28109	cg01350803	47628373	47628373	0.4235545	0	1	0.7001749	121.7572
28189	cg01354782	34742332	34742332	0.2821983	0	1	0.3963863	24.78479
28281	cg01359822	28651441	46697474	0.6968617	0	1	0.7982233	27.4092
28460	cg01369220	20742450	20742450	0.6364825	0	1	0.4778726	-41.18238
28478	cg01369895	55620332	55620332	0.2381542	0	1	0.3403322	22.42401
28509	cg01371420	44612418	44612418	0.2542953	0	1	0.3553513	21.3693
28617	cg01377268	55689445	71681318	0.4093705	0	1	0.5167109	20.34803
28712	cg01381829	15732482	15732482	0.1770395	0	1	0.310531	39.58197
28736	cg01382860	19729390	55803378	0.616247	0	1	0.4668537	-36.56134
28803	cg01385959	28748320	28748320	0.3093176	0	1	0.470599	42.76727
28837	cg01387905	36640437	36640437	0.7403839	0	1	0.8496817	37.51372
28854	cg01388693	49677502	49677502	0.3887718	0	1	0.2019348	-64.576
28957	cg01394167	12717498	12717498	0.3958937	0	1	0.6255424	80.75972
28992	cg01395541	62685325	62685325	0.6447748	0	1	0.8885707	146.5356

29041	cg01397495	73746324	73746324	0.4617675	0	1	0.8173266	374.3439
29076	cg01399317	13760327	70639478	0.7726565	0	1	0.5890194	-65.40347
29086	cg01400040	49616453	49616453	0.525993	0	1	0.6411438	24.13321
29249	cg01407447	43638467	43638467	0.6552965	0	1	0.7771925	34.14968
29286	cg01409343	55651304	55651304	0.1947286	0	1	0.3027769	27.06044
29299	cg01409709	70799373	70799373	0.1931905	0	1	0.3296989	39.29461
29334	cg01410923	56726407	56726407	0.4708053	0	1	0.6465733	49.77295
29362	cg01412404	49660476	49660476	0.6731756	0	1	0.775337	25.82468
29363	cg01412419	72610302	69694413	0.8008996	0	1	0.6783165	-37.03577
29379	cg01412970	57688456	12650324	0.3761131	0	1	0.2465737	-32.19078
29401	cg01413582	32689494	32689494	0.2904899	0	1	0.3970934	21.95148
29407	cg01413848	38718438	40801417	0.8961551	0	1	0.7858641	-48.32117
29411	cg01414185	57719415	39804430	0.9472281	0	1	0.8357391	-70.09364
29460	cg01416563	12766393	31702477	0.7356013	0	1	0.5811127	-45.2233
29707	cg01428378	39602445	61800414	0.2808608	0	1	0.1138398	-69.09982
29731	cg01429449	48610458	38713324	0.2427424	0	1	0.1288747	-36.28166
29960	cg01440489	67620425	67620425	0.4301635	0	1	0.5462449	23.16638
30020	cg01442843	62673347	62673347	0.168951	0	1	0.2704632	26.47497
30091	cg01445689	17793335	37710470	0.7977538	0	1	0.8994616	43.92517
30092	cg01445793	46745432	29695498	0.6976989	0	1	0.5223101	-52.53246
30171	cg01448562	10771502	27630358	0.9260696	0	1	0.7985137	-71.50535
30229	cg01451101	39754340	39754340	0.2109727	0	1	0.3116128	23.26398
30293	cg01454592	48725359	48725359	0.3372683	0	1	0.1617614	-64.105
30398	cg01460430	61777484	61777484	0.3223408	0	1	0.4362579	23.52855
30443	cg01462727	58768363	58768363	0.5330851	0	1	0.6502417	25.0708
30451	cg01463110	10715421	10715421	0.4047323	0	1	0.2829981	-27.45662
30463	cg01464067	49746427	49746427	0.5167318	0	1	0.6792452	44.9157
30575	cg01469864	10809390	10809390	0.3772071	0	1	0.2446948	-33.51675
30671	cg01474688	10701448	10701448	0.3222894	0	1	0.0294513	-220.7617
30685	cg01475387	20620412	53605402	0.4068924	0	1	0.2992853	-22.02687
30738	cg01478179	69693345	69693345	0.5144091	0	1	0.3686331	-34.5224
30808	cg01483656	70649496	70649496	0.6018847	0	1	0.4124464	-55.59628
30825	cg01484843	23790396	23790396	0.3134336	0	1	0.5064957	58.75988
30925	cg01490094	53695420	53695420	0.2047913	0	1	0.3967248	67.21056
30948	cg01491428	15709507	15709507	0.2500164	0	1	0.3984726	40.05838
30971	cg01493009	12753507	12753507	0.5676609	0	1	0.711545	38.40781
31096	cg01499815	57646393	43678317	0.2369099	0	1	0.0647248	-88.92109
31148	cg01502466	54808314	54808314	0.55853	0	1	0.6979724	35.63928
31448	cg01518225	50676477	19679386	0.8884813	0	1	0.768931	-51.91384
31476	cg01519225	64745443	64745443	0.3862339	0	1	0.1421627	-116.6658
31485	cg01519508	36802474	67694477	0.6294492	0	1	0.5235194	-20.84557
31566	cg01523029	18756452	18756452	0.3925437	0	1	0.5773168	52.82951
31756	cg01534253	27658321	27658321	0.6013397	0	1	0.7087295	23.82435

31925	cg0154358	33710356	33710356	0.6960163	0	1	0.4716074	-81.40386
31976	cg0154681	53787464	53787464	0.9259464	0	1	0.6532279	-199.1004
32005	cg0154846	66707495	66707495	0.2023638	0	1	0.3099241	26.34541
32015	cg0154922	16643509	16643509	0.5673799	0	1	0.6767282	23.23245
32037	cg0155034	57800438	57800438	0.2690748	0	1	0.3884188	27.13938
32061	cg0155138	59663305	59663305	0.3168015	0	1	0.7259785	374.3439
32139	cg0155477	29748349	29748349	0.3332668	0	1	0.5089561	48.88505
32251	cg0155930	21687389	21687389	0.1138182	0	1	0.2182894	33.89233
32291	cg0156087	16674376	16674376	0.5873391	0	1	0.4714103	-23.40243
32311	cg0156175	53619391	53619391	0.3964749	0	1	0.7989693	374.3439
32318	cg0156186	48704344	48704344	0.523517	0	1	0.4202498	-19.0795
32332	cg0156281	21632448	21632448	0.4540577	0	1	0.5580661	19.37351
32509	cg0156998	42778479	42778479	0.8464193	0	1	0.7091262	-52.11637
32722	cg0157960	67692433	67692433	0.171493	0	1	0.2721469	25.92834
32736	cg0158022	26693475	26693475	0.6258196	0	1	0.7502161	32.86076
32750	cg0158103	35778319	35778319	0.2386489	0	1	0.3459068	24.16847
32759	cg0158134	45618385	45618385	0.8144183	0	1	0.6728895	-48.45395
32770	cg0158206	61600338	61600338	0.4121338	0	1	0.5273644	22.87776
32834	cg0158572	34695470	34695470	0.5517092	0	1	0.3730979	-49.63688
32937	cg0159134	35649365	35649365	0.8515914	0	1	0.542827	-191.9235
32960	cg0159235	74631308	74631308	0.7318841	0	1	0.8375754	33.85196
33130	cg0160171	22645432	22645432	0.4790837	0	1	0.7011731	80.34711
33180	cg0160400	56664402	56664402	0.5230348	0	1	0.6547742	30.56437
33341	cg0161229	38678327	60736393	0.9101471	0	1	0.746197	-91.42204
33352	cg0161265	33661463	14650472	0.3493249	0	1	0.4532256	19.81639
33383	cg0161410	66709389	51669418	0.6232883	0	1	0.5091808	-23.37203
33529	cg01620701	45739403	45739403	0.4203559	0	1	0.5403606	24.48945
33548	cg0162139	23710488	23710488	0.3970261	0	1	0.533228	30.46095
33677	cg0162900	29673359	29673359	0.2772619	0	1	0.3821853	21.81691
33717	cg0163133	58620420	58620420	0.5066646	0	1	0.6109964	20.01785
33803	cg0163657	66756369	66756369	0.1752286	0	1	0.2830292	28.48321
33812	cg0163687	50613508	13770417	0.5485265	0	1	0.7561784	77.75213
33823	cg0163724	14719443	14719443	0.4210663	0	1	0.5347021	22.34325
33826	cg0163748	31720477	31720477	0.2364439	0	1	0.337005	21.95085
33833	cg0163778	63665456	63665456	0.325869	0	1	0.5071182	51.94813
33844	cg0163822	51719450	43603388	0.2493636	0	1	0.3613093	25.33134
34017	cg0164794	29767500	29767500	0.5367252	0	1	0.6916203	42.08566
34043	cg01649611	69642419	69642419	0.5658734	0	1	0.7725711	79.82115
34067	cg0165116	18772368	18772368	0.2762174	0	1	0.4394817	45.29072
34089	cg0165224	20802475	26638420	0.9778587	0	1	0.8739803	-85.46902
34143	cg0165565	14611329	14611329	0.4332351	0	1	0.060365	-296.9087
34146	cg01655721	42695500	61791376	0.7862659	0	1	0.8878734	40.9292
34369	cg0166865	34664417	57628330	0.862324	0	1	0.6873432	-80.28867

34511	cg0167870	12684333	14738326	0.5033584	0	1	0.3553087	-35.66949
34567	cg0168186	38675349	38675349	0.2029171	0	1	0.3117504	26.79529
34628	cg0168588	49689328	49689328	0.3970692	0	1	0.2583829	-35.28607
34763	cg0169332	52662346	52662346	0.638665	0	1	0.764219	34.53627
34803	cg0169599	26726427	26726427	0.2463662	0	1	0.376129	32.29324
34864	cg0169988	10749443	10749443	0.9119216	0	1	0.8099425	-47.78122
34908	cg0170250	66667497	66667497	0.6160984	0	1	0.9224252	374.3439
35024	cg0170837	50717377	50717377	0.3627818	0	1	0.4719499	21.2728
35083	cg0171067	14745317	42785427	0.6524809	0	1	0.4813695	-47.70937
35131	cg0171350	66622481	66622481	0.272829	0	1	0.0788423	-99.47445
35232	cg0171748	71740418	71740418	0.5164223	0	1	0.6351503	25.21323
35593	cg0173702	66789447	66618329	0.6184667	0	1	0.5158275	-19.62201
35645	cg0174013	69676427	69676427	0.4575159	0	1	0.2198209	-95.37771
35659	cg0174105	70642504	16632414	0.4499165	0	1	0.3113638	-32.8918
35661	cg0174137	49677332	49677332	0.3166417	0	1	0.4932537	49.90146
35708	cg0174439	37747425	37747425	0.1747992	0	1	0.0576023	-53.31369
35780	cg0174875	24802426	24802426	0.3459311	0	1	0.4500897	19.94644
35819	cg0175037	37724327	13644489	0.1569815	0	1		-4.34E-09
35898	cg0175442	68737322	68737322	0.4911289	0	1	0.6406024	37.29332
35899	cg0175452	18686381	21736365	0.5850595	0	1	0.3793183	-64.78815
36014	cg0175967	23650393	23650393	0.4733613	0	1	0.3498896	-26.2625
36024	cg0176009	33662376	33662376	0.5796831	0	1	0.4096609	-45.32622
36091	cg0176394	32688424	32688424	0.6103902	0	1	0.9243134	374.3439
36119	cg0176532	16704479	16704479	0.8682088	0	1	0.9692668	75.71433
36577	cg0178670	56664461	32808388	0.4102499	0	1	0.5947229	52.81959
36700	cg0179260	34798432	34798432	0.5211605	0	1	0.4195439	-18.58651
36775	cg0179622	65731500	65731500	0.6864066	0	1	0.8100846	38.72423
36801	cg0179737	52795419	10640304	0.0538221	0	1	0.2056299	78.54268
36810	cg0179765	23670458	23670458	0.8964718	0	1	0.7871281	-47.82
36887	cg0180109	59654474	59654474	0.6823622	0	1	0.8875042	112.9095
36992	cg0180493	30652499	30652499	0.7751557	0	1	0.6719716	-26.20895
37176	cg0181243	42648449	42648449	0.6373375	0	1	0.4878725	-37.18172
37207	cg0181373	70692408	70692408		0	1	0.9210706	4.34E-09
37249	cg0181556	38792328	71641451	0.7186694	0	1	0.5482057	-51.69393
37313	cg0181836	48799312	48799312	0.9381061	0	1	0.7434837	-134.1726
37356	cg0182027	37709389	66738353	0.5475357	0	1	0.3801396	-44.03192
37440	cg0182358	54804382	54804382	0.8096027	0	1	0.5270173	-149.1471
37449	cg0182406	43696400	43696400	0.2195804	0	1	0.327788	25.52014
37560	cg0182963	74607370	74607370	0.6504281	0	1	0.7630561	29.02316
37701	cg0183592	21638313	21638313	0.0930566	0	1	0.2074354	42.62806
37709	cg0183645	27725379	27725379	0.683126	0	1	0.804101	36.67192
37748	cg0183800	62755506	62755506	0.3849495	0	1	0.2380734	-40.18
37791	cg0184018	13742471	19667410	0.6269426	0	1	0.4610209	-44.26107

37828	cg01842774	44678464	17673382	0.6269081	0	1	0.9391788	374.3439
37841	cg01843264	27791413	27791413	0.2916803	0	1	0.4491116	41.75764
37893	cg01847062	74642468	74642468	0.6129752	0	1	0.8972105	374.3439
38025	cg01855010	51601347	51601347	0.2304163	0	1	0.3335453	23.11826
38067	cg01857151	24772402	24772402	0.6219208	0	1	0.7733738	47.75494
38082	cg01857838	36653390	36653390	0.217901	0	1	0.3324095	28.00059
38096	cg01858712	38792447	38792447	0.2269235	0	1	0.3498659	30.7396
38160	cg01861555	52778438	52778438	0.6604158	0	1	0.9507636	374.3439
38182	cg01862688	36734461	45740407	0.5691721	0	1	0.4661144	-19.15614
38210	cg01864404	28711435	49785475	0.6954322	0	1	0.5756943	-27.6723
38211	cg01864468	10669341	10669341	0.1525481	0	1	0.0069841	-99.49196
38348	cg01870916	16718504	16718504	0.1912917	0	1	0.3344822	42.63706
38352	cg01871025	49793376	49793376	0.7730291	0	1	0.6613412	-29.44091
38389	cg01872988	51730335	51730335	0.4207536	0	1	0.6732321	99.62267
38419	cg01874863	43663398	43663398	0.5696757	0	1	0.4545685	-22.95914
38420	cg01874867	31606304	31606304	0.5887598	0	1	0.4591583	-28.22039
38433	cg01875467	26673508	26673508	0.1878343	0	1	0.3310125	43.00665
38444	cg01876338	43725462	43725462	0.2286536	0	1	0.3451487	28.10733
38542	cg01880569	54806507	54806507	0.4783973	0	1	0.5801036	18.84234
38553	cg01881062	58767390	58767390	0.3446805	0	1	0.4657449	25.50859
38559	cg01881287	35697305	35697305	0.7434219	0	1	0.6203596	-31.79001
38587	cg01882058	57666334	44643344	0.6664376	0	1	0.8008917	42.77571
38588	cg01882084	44622465	44622465	0.4418976	0	1	0.5420421	18.16308
38604	cg01882774	21623380	21623380	0.6489518	0	1	0.5323298	-24.84839
38634	cg01883777	19693345	19693345	0.5887425	0	1	0.4688626	-24.75678
38741	cg01889893	13602332	13602332	0.2632144	0	1	0.3799356	26.43277
38744	cg01889956	41802369	41802369	0.3937032	0	1	0.502818	20.97256
38767	cg01891583	60666352	60666352	0.4757539	0	1	0.2033439	-125.7698
38769	cg01891736	43638342	59657395	0.3329616	0	1	0.4348417	19.46641
38821	cg01894875	64760482	64760482	0.4770377	0	1	0.227017	-103.5937
38950	cg01901708	48643410	48643410	0.4219288	0	1	0.528578	20.11219
39019	cg01905478	34688463	34688463	0.6403254	0	1	0.7455028	24.9523
39190	cg01913196	66697436	54617510	0.5183525	0	1	0.416204	-18.75014
39246	cg01916400	20640482	20640482	0.6273031	0	1	0.7773836	47.55752
39253	cg01916697	35690442	35690442	0.5760315	0	1	0.8777246	374.3439
39287	cg01918604	26687384	26687384	0.5279275	0	1	0.7364756	75.55006
39316	cg01919941	33759365	33759365	0.4437957	0	1	0.6027378	40.45235
39349	cg01921484	24711510	24711510	0.5489081	0	1	0.8239898	146.9932
39452	cg01925738	27652432	36748389	0.4854667	0	1	0.2885086	-62.21781
39472	cg01926813	48794458	48794458	0.8362092	0	1	0.7132779	-42.28316
39487	cg01927825	17753408	26749428	0.3719826	0	1	0.2302443	-38.45297
39545	cg01930745	24720508	24720508	0.5870671	0	1	0.8417969	135.0434
39553	cg01931222	23666344	23666344	0.4848226	0	1	0.588046	19.3765

39790	cg0194158	61651486	61651486	0.3159329	0	1	0.4262615	22.48854
39816	cg0194255	57796400	57796400	0.1948463	0	1	0.30069	26.20066
39895	cg0194609	31608438	31608438	0.571136	0	1	0.8819889	374.3439
39945	cg0194821	66810339	66810339	0.7198527	0	1	0.8335621	37.17409
39947	cg0194822	71746375	71746375	0.5248659	0	1	0.7070929	56.91673
39969	cg0194983	55734495	55734495	0.9139854	0	1	0.5000361	-371.3336
40076	cg0195458	21647351	21647351	0.5880929	0	1	0.7193787	33.52977
40133	cg0195818	26702322	26702322	0.4764547	0	1	0.6482149	47.84415
40193	cg0196074	32809432	32809432	0.8742878	0	1	0.7290584	-64.08896
40277	cg0196363	16698453	16698453	0.6820107	0	1	0.5319122	-39.36488
40338	cg0196627	46681479	46681479	0.5604058	0	1	0.7117552	41.79499
40362	cg0196692	27633458	27633458	0.4186853	0	1	0.5864527	44.30391
40424	cg0197033	73637467	73637467	0.2847533	0	1	0.4048403	26.79015
40541	cg0197454	33705326	33705326	0.4534852	0	1	0.642799	56.71609
40560	cg0197559	69648422	69648422	0.3960138	0	1	0.2820518	-24.71116
40566	cg0197585	59694310	59694310	0.6067417	0	1	0.7727221	55.34533
40605	cg0197823	29660412	29660412	0.1611298	0	1	0.266229	28.62439
40613	cg0197870	27711305	69625371	0.5235168	0	1	0.6255644	19.56752
40810	cg0198920	11611407	11611407	0.4157505	0	1	0.5202097	19.44686
40852	cg0199174	60789496	60789496	0.388667	0	1	0.6813217	134.4673
40884	cg0199355	15679463	15679463	0.2603859	0	1	0.4165175	42.89376
40962	cg0199741	48680440	22726367	0.2005657	0	1	0.0884544	-42.32327
40967	cg0199759	59640444	59640444	0.6871272	0	1	0.5737658	-25.00423
40974	cg0199788	22666461	22666461	0.554424	0	1	0.7027309	39.7934
41034	cg0200106	66717351	66717351	0.1852845	0	1	0.290633	26.70031
41095	cg0200440	14658436	25690377	0.3855273	0	1	0.2216446	-49.75232
41253	cg0201139	63759488	63759488	0.6265416	0	1	0.5098924	-24.29762
41266	cg0201172	73613315	54669365	0.1649072	0	1	0.0584884	-46.2268
41277	cg0201237	30653507	30653507	0.4111057	0	1	0.6300857	73.81918
41289	cg0201282	54691403	54691403	0.5114045	0	1	0.3633514	-35.54225
41320	cg0201439	72718380	72718380	0.5673051	0	1	0.7441345	57.6771
41401	cg0201829	31768427	31768427	0.2569881	0	1	0.378258	28.40474
41443	cg0202038	41641503	41641503	0.5226818	0	1	0.6424748	25.77604
41473	cg0202191	66773311	66773311	0.6239618	0	1	0.7591847	38.37726
41655	cg0203112	43772385	43772385	0.8539966	0	1	0.9641249	79.99045
41924	cg0204597	36753465	49639309	0.5639834	0	1	0.4005626	-42.1046
41949	cg0204705	69657408	69657408	0.3843833	0	1	0.5108482	26.8959
42086	cg0205313	40754486	40754486	0.5472087	0	1	0.7034014	43.4586
42101	cg0205389	62761334	62761334	0.3413335	0	1	0.2274373	-27.18982
42200	cg0205840	20630471	73725428	0.2960832	0	1	0.1243581	-69.49243
42475	cg0206959	11701350	11701350	0.5300692	0	1	0.6411781	22.7783
42576	cg0207346	18787317	18787317	0.5778268	0	1	0.7059793	31.45205
42624	cg0207579	35650440	35650440	0.3288946	0	1	0.4388082	22.06589

42893	cg02087985	44624356	44624356	0.4812312	0	1	0.581548	18.44476
42896	cg02088197	26700405	26700405	0.6848962	0	1	0.5804092	-21.89103
43007	cg02094283	73653485	73653485	0.2612579	0	1	0.4192665	43.71553
43028	cg02095601	71613376	71613376	0.5911394	0	1	0.7123171	29.06641
43042	cg02096220	38701337	38701337	0.7570575	0	1	0.8881804	59.20897
43169	cg02102075	42690304	42690304	0.2101439	0	1	0.3153349	24.96397
43251	cg02106072	67692407	67692407	0.6038589	0	1	0.8887458	374.3439
43378	cg02113055	22668459	22668459	0.6874413	0	1	0.408947	-122.1351
43486	cg02118671	58650332	35735454	0.742529	0	1	0.5306357	-78.51492
43502	cg02119693	12715504	12715504	0.1195057	0	1	0.2473439	44.88185
43505	cg02119792	41653398	69806312	0.159513	0	1	0.036569	-64.97071
43510	cg02119938	57761451	43808480	0.2875295	0	1	0.4275816	34.40289
43635	cg02126896	21603365	21603365	0.6415803	0	1	0.3576401	-124.2992
43648	cg02127418	19740340	19740340	0.9197813	0	1	0.8179865	-50.31499
43741	cg02132058	46729363	46729363	0.3469575	0	1	0.7727129	374.3439
43788	cg02134022	49760428	49760428	0.4915508	0	1	0.6163087	26.9177
43809	cg02135122	57807302	57807302	0.4456778	0	1	0.5479659	18.81386
43898	cg02140579	26657344	26657344	0.3547099	0	1	0.4700493	23.38919
43902	cg02141208	69664372	69664372	0.7346956	0	1	0.6116027	-31.18751
43934	cg02143936	34696308	34696308	0.623396	0	1	0.421169	-63.31657
44118	cg02152233	55703450	34610416	0.9339578	0	1	0.8064886	-75.44418
44212	cg02156723	14803389	14803389	0.1589014	0	1	0.2672166	30.19634
44235	cg02157626	53781451	53781451	0.7141777	0	1	0.8259446	35.14265
44265	cg02159489	29807491	20791359	0.3257598	0	1	0.0428483	-200.0124
44318	cg02161919	64650410	31701429	0.5345104	0	1	0.4319921	-18.85352
44322	cg02162286	12807354	56601450	0.3938442	0	1	0.2891739	-21.36191
44433	cg02168584	26797508	26797508	0.2238123	0	1	0.3270618	23.4952
44457	cg02169713	72641379	72641379	0.4453712	0	1	0.3146632	-29.72948
44466	cg02170103	39697472	39697472	0.2058756	0	1	0.3112944	25.31107
44517	cg02172885	49699431	49699431	0.8077406	0	1	0.9147736	52.1055
44523	cg02173067	36681393	26757342	0.4177277	0	1	0.2596273	-43.86496
44545	cg02174092	15664467	72608378	0.2312004	0	1	0.1164894	-38.56046
44709	cg02184226	37622508	37622508	0.6567134	0	1	0.7688935	29.30266
44716	cg02184540	42771474	42771474	0.9303995	0	1	0.63525	-226.6221
44724	cg02185007	72694378	72694378	0.2252795	0	1	0.3383596	27.00717
44774	cg02188142	44745437	44745437	0.778127	0	1	0.4451059	-192.3109
44784	cg02188665	10787450	10787450	0.5484048	0	1	0.7275042	57.11839
44853	cg02192520	64801367	64801367	0.3616371	0	1	0.470724	21.26149
44878	cg02193806	59631353	59631353	0.5323994	0	1	0.6696471	33.31846
44899	cg02194908	38687336	48773344	0.6049986	0	1	0.4328817	-46.69324
44914	cg02196256	37619333	37619333	0.3850197	0	1	0.5035523	24.1151
44948	cg02197629	19743370	41784469	0.5948592	0	1	0.479586	-23.27904
44999	cg02201753	46662451	46662451	0.5063073	0	1	0.894922	374.3439

45042	cg02203761	43722355	43722355	0.3999472	0	1	0.5070291	20.30355
45078	cg02205962	38789501	38789501	0.5978894	0	1	0.4889227	-21.27277
45217	cg02214597	46607504	63775303	0.3974456	0	1	0.2587899	-35.25015
45270	cg02217247	28616345	28616345	0.3870442	0	1	0.2795718	-22.58373
45296	cg02218932	13644467	13644467		0	1	0.799684	4.34E-09
45325	cg02220418	18675470	51631381	0.7385683	0	1	0.8389265	31.50704
45364	cg02222362	34644438	34644438	0.4830998	0	1	0.369072	-22.76566
45373	cg02222844	63665449	46801339	0.7396	0	1	0.5555776	-61.17403
45397	cg02224314	54760479	33722311	0.7577752	0	1	0.6308674	-34.55595
45470	cg02227879	58654437	58654437	0.5390796	0	1	0.6406272	19.717
45578	cg02233197	26765412	26765412	0.606338	0	1	0.7091116	22.25379
45626	cg02235843	11800397	11800397	0.618974	0	1	0.7315788	26.89237
45768	cg02243386	16646325	16646325	0.4047467	0	1	0.5430256	31.25382
45774	cg02243630	59795312	59795312	0.5066769	0	1	0.4018738	-19.59454
45896	cg02249577	19661302	16740379	0.7094428	0	1	0.5855684	-29.9549
45950	cg02251771	14751370	14751370	0.5811924	0	1	0.763914	63.56574
45995	cg02254774	32607413	54600312	0.7700156	0	1	0.6451574	-34.76543
46151	cg02262167	45666375	70666403	0.7420775	0	1	0.475075	-119.3185
46156	cg02262553	12658476	12658476	0.3772548	0	1	0.6019183	76.95147
46247	cg02267270	60657497	60657497	0.6218292	0	1	0.4912821	-29.1626
46272	cg02268561	13671358	13671358	0.6329834	0	1	0.5293907	-20.18693
46411	cg02275226	65730497	65730497	0.8380045	0	1	0.6439107	-86.15196
46658	cg02286623	72747447	27628432	0.3665011	0	1	0.2533374	-25.58605
46659	cg02286642	26615326	26615326	0.2436031	0	1	0.1283641	-37.02055
46683	cg02287371	10605360	10605360	0.4238224	0	1	0.2523476	-50.95076
46701	cg02288345	19809433	19809433	0.493922	0	1	0.642342	36.90208
46779	cg02291561	15691331	15691331	0.1275901	0	1	0.2375913	34.60401
46813	cg02293501	28714496	28714496	0.8566543	0	1	0.7480882	-38.3448
46857	cg02295509	45796496	45796496	0.5035459	0	1	0.6122734	21.41411
46860	cg02295574	26612374	26612374	0.7854671	0	1	0.9128643	65.52663
46881	cg02296931	39721443	24783471	0.1828304	0	1	0.3332416	47.17025
46920	cg02299007	57603479	57603479	0.3663283	0	1	0.7338303	374.3439
46954	cg02300652	30661432	30661432	0.4268643	0	1	0.5287384	18.65824
46988	cg02301920	24763311	24763311	0.5437921	0	1	0.7810419	103.116
47009	cg02303209	34697354	34697354	0.6082451	0	1	0.7219917	26.77797
47036	cg02304092	47773302	48721351	0.4661187	0	1	0.179545	-143.6083
47052	cg02304584	30626500	30626500	0.4711645	0	1	0.311944	-41.72014
47082	cg02305687	48699436	48699436	0.6230667	0	1	0.5219465	-19.24103
47196	cg02311337	70681353	70681353		0	1	0.9501108	4.34E-09
47227	cg02312593	64807420	64807420	0.5030409	0	1	0.3806887	-25.46743
47239	cg02313379	22653369	51809345	0.1797345	0	1	0.2922796	30.04821
47288	cg02315732	23732351	23732351	0.3489465	0	1	0.4620878	22.75173
47310	cg02317299	64623415	58695329	0.4776257	0	1	0.5860178	20.94673

47368	cg0231982[illegible]	46797387	46797387	0.8366599	0	1	0.6531951	-78.4893
47369	cg0231986[illegible]	63707303	63707303	0.5814595	0	1	0.6848262	21.52022
47488	cg0232656[illegible]	37675446	37675446	0.589416	0	1	0.4835986	-20.18297
47577	cg0233050[illegible]	53770462	53770462	0.1873493	0	1	0.297888	28.60008
47682	cg0233525[illegible]	67807477	67807477	0.4818901	0	1	0.6055936	26.33998
47762	cg0233884[illegible]	58657370	58657370		0	1		-4.34E-09
47797	cg0234009[illegible]	19620379	19620379	0.0766284	0	1	0.1977872	50.34867
47854	cg0234275[illegible]	41775434	41775434	0.8106781	0	1	0.635265	-67.21432
47930	cg0234575[illegible]	37710395	37710395	0.7449138	0	1	0.9520096	156.5356
47984	cg0234843[illegible]	47783343	47783343	0.1907469	0	1	0.3368587	44.11095
48035	cg0235127[illegible]	33786418	33786418	0.6326909	0	1	0.7494628	29.67407
48066	cg0235263[illegible]	32803433	32803433	0.3103139	0	1	0.4208224	22.68652
48120	cg0235580[illegible]	29764301	29764301	0.5323228	0	1	0.2934264	-88.82812
48169	cg0235838[illegible]	54608494	54608494	0.6636671	0	1	0.775162	29.54073
48274	cg0236359[illegible]	62732492	62732492	0.184226	0	1	0.3443543	52.03344
48486	cg0237240[illegible]	33619327	33619327	0.3807668	0	1	0.0779546	-198.9454
48522	cg0237498[illegible]	62778419	62778419	0.6188626	0	1	0.7585712	40.38428
48565	cg0237691[illegible]	22802390	22802390	0.8317934	0	1	0.6986246	-46.94765
48607	cg0237954[illegible]	52684309	52684309	0.4247615	0	1	0.1814153	-106.3663
48619	cg0238023[illegible]	18773369	59737510	0.5609909	0	1	0.7623963	74.71088
48654	cg0238185[illegible]	43676505	43676505	0.6086692	0	1	0.7329549	31.55353
48809	cg0238904[illegible]	74604407	74604407	0.601229	0	1	0.4085817	-57.35221
48815	cg0238918[illegible]	30634354	30634354	0.2719853	0	1	0.3984532	29.68691
48822	cg0238931[illegible]	61663314	61663314	0.3166648	0	1	0.5338346	72.98967
48829	cg0238963[illegible]	56808505	56808505	0.4901232	0	1	0.6094358	24.87179
48837	cg0238994[illegible]	74642445	74642445	0.2988162	0	1	0.1048185	-90.02982
48860	cg0239081[illegible]	72750329	66630365	0.9019092	0	1	0.7942221	-48.36368
48938	cg0239424[illegible]	19662495	19662495	0.4142188	0	1	0.5705864	38.91103
49040	cg0239848[illegible]	27722479	27722479	0.5237533	0	1	0.721069	66.82908
49101	cg0240077[illegible]	67767450	67767450	0.7177667	0	1	0.827729	34.52588
49110	cg0240135[illegible]	19620448	19620448	0.3288485	0	1	0.2003707	-35.03163
49113	cg0240152[illegible]	63767396	63767396	0.9280311	0	1	0.8135502	-62.6526
49129	cg0240220[illegible]	19740380	19740380	0.3153247	0	1	0.4243779	22.08628
49164	cg0240388[illegible]	70783472	70783472	0.6470628	0	1	0.5167499	-29.76199
49217	cg0240741[illegible]	66778398	61773490	0.2245016	0	1	0.0835365	-60.74213
49293	cg0241304[illegible]	29678340	29678340	0.1116266	0	1	0.2150054	33.68401
49331	cg0241505[illegible]	47715360	39807380	0.1716064	0	1	0.0644593	-45.06026
49497	cg0242617[illegible]	36803354	36803354	0.5265145	0	1	0.6341314	21.47952
49519	cg0242757[illegible]	56689398	56689398	0.4620129	0	1	0.5967543	30.26687
49635	cg0243455[illegible]	73652409	73652409	0.4086443	0	1	0.306122	-20.22578
49675	cg0243802[illegible]	65654422	65654422	0.3363521	0	1	0.4395677	19.81422
49848	cg0244829[illegible]	21728441	21728441	0.1633467	0	1	0.2946992	40.16185
49851	cg0244848[illegible]	36664410	10774420	0.9778093	0	1	0.7296582	-223.4693

49859	cg0244893	43770317	43770317	0.8241751	0	1	0.9462521	78.15036
49870	cg0244967(	21667300	21667300	0.2943068	0	1	0.5916757	137.7275
49917	cg0245241	22803434	22803434	0.4287428	0	1	0.1901069	-101.0385
49931	cg0245296	32611456	19780360	0.2854286	0	1	0.0559143	-140.0173
49978	cg0245534	49740456	49740456	0.5770062	0	1	0.6801599	21.29377
50033	cg0245803	70648417	18723357	0.0512264	0	1		-4.34E-09
50041	cg0245818	32715306	32715306	0.6936828	0	1	0.8146709	38.03944
50129	cg0246219	34754444	34754444	0.479681	0	1	0.1034197	-276.0428
50140	cg0246266	52800491	52800491	0.1574624	0	1	0.2650353	30.0219
50174	cg0246445	35647474	35647474	0.5769475	0	1	0.8014255	98.23613
50175	cg0246448	41709400	41709400	0.3790692	0	1	0.5176941	31.5244
50183	cg0246480	26621487	26621487	0.8983376	0	1	0.7059019	-107.9352
50301	cg0247069	29660440	63650414	0.9242489	0	1	0.7970902	-70.34007
50461	cg0247836	62778410	62778410	0.5781619	0	1	0.7682375	68.58669
50479	cg0247902	28787373	28787373	0.5628821	0	1	0.6980047	33.83672
50486	cg0247930	73614314	73614314	0.4139893	0	1	0.5180516	19.32992
50505	cg0248032(	55675493	55675493	0.5093203	0	1	0.615023	20.50763
50514	cg0248067	70810398	70810398	0.5421442	0	1	0.7142702	52.17098
50748	cg0249046(	11677337	11677337	0.6483889	0	1	0.5049977	-35
50785	cg0249194	21602370	21602370	0.1808959	0	1	0.3582393	62.0506
50870	cg0249576(	45745344	45745344	0.3075523	0	1	0.4544	36.44147
50932	cg0249930	62710352	62710352	0.7760295	0	1	0.8806436	40.99618
50940	cg0249964(	72752352	72752352	0.6683778	0	1	0.7708466	25.6114
50996	cg0250214	13619464	13619464	0.2523493	0	1	0.4467317	63.40131
51072	cg0250595	60701359	60701359	0.500505	0	1	0.6473742	36.42036
51090	cg0250672	65732360	65732360	0.0614257	0	1	0.1755187	50.17897
51094	cg0250690	30785352	30785352	0.4194273	0	1	0.5494572	28.09144
51101	cg0250718	29708358	29708358	0.3484003	0	1	0.4953985	35.35902
51106	cg0250757	55603348	55603348	0.8030216	0	1	0.3299237	-371.3336
51209	cg0251355	50623440	50623440	0.5066915	0	1	0.6369669	29.44685
51238	cg0251521	57774402	57774402	0.1182887	0	1	0.2235625	33.63655
51253	cg0251589	45775478	45775478	0.5199417	0	1	0.6218052	19.44383
51270	cg0251669	55685455	55685455	0.8243901	0	1	0.6283048	-83.8158
51299	cg0251813	12627503	12627503	0.4826888	0	1	0.2752464	-69.28884
51317	cg0251926	24663407	24663407	0.4345119	0	1	0.5418841	20.34695
51384	cg0252293	29680371	29680371	0.3552572	0	1	0.118342	-118.389
51411	cg0252423	33677426	33677426	0.5761748	0	1	0.6806192	21.73048
51487	cg0252781	41782488	41782488	0.7218978	0	1	0.8264074	31.80667
51583	cg0253264	46751358	31767336	0.021427	0	1	0.1231208	55.98919
51610	cg0253372	37758449	37758449	0.6511697	0	1	0.5065007	-35.63333
51769	cg0254144	55644471	55644471	0.4434434	0	1	0.3085763	-31.52319
51785	cg0254281	47704302	47704302	0.3929986	0	1	0.5053567	22.01267
51891	cg0254877	44637449	44637449	0.6484476	0	1	0.5400781	-22.06163

51923	cg02550308	18647437	18647437	0.1619093	0	1	0.2687373	29.27465
51934	cg02550738	31707440	31707440	0.7256363	0	1	0.8648773	57.65231
52178	cg02564175	13637463	48691441	0.1369203	0	1	0.3516967	95.57898
52179	cg02564259	66654397	66654397	0.2689828	0	1	0.3726953	21.70159
52215	cg02566259	72648474	72648474	0.4606391	0	1	0.356926	-19.65383
52242	cg02567750	59810364	59810364	0.7460223	0	1	0.6251642	-31.07703
52245	cg02567769	56600477	34766486	0.6399065	0	1	0.5309547	-22.04087
52248	cg02567879	44632442	44632442	0.4737625	0	1	0.598668	26.65534
52409	cg02574844	38756423	17704375	0.8559373	0	1	0.702683	-64.02131
52467	cg02577387	31618469	37668450	0.4725696	0	1	0.320291	-38.33617
52505	cg02578709	62603494	62603494	0.5493556	0	1	0.6628218	24.17611
52529	cg02579959	55778355	55778355	0.2499334	0	1	0.3617383	25.25437
52554	cg02580986	23730425	66742361	0.3804917	0	1	0.2789723	-20.65911
52644	cg02584498	29652303	29652303	0.3702662	0	1	0.5181566	35.38246
52664	cg02585440	56782510	56782510	0.5429543	0	1	0.6739902	31.03119
52756	cg02590088	44686465	44686465	0.2012578	0	1	0.305061	25.00144
52835	cg02594532	73663496	73663496	0.4606605	0	1	0.5918122	28.83652
52903	cg02598335	10689459	10689459	0.4264881	0	1	0.5309663	19.44368
52918	cg02598807	51673307	71733392	0.6014717	0	1	0.7023478	21.34434
52934	cg02599498	27809452	55635418	0.2950025	0	1	0.4297433	31.95529
53007	cg02603022	64671423	64671423	0.5746658	0	1	0.4694651	-19.84821
53040	cg02604730	51751449	51751449	0.5199868	0	1	0.6733103	40.34913
53064	cg02605760	24668498	31717474	0.5508568	0	1	0.4389275	-21.81676
53080	cg02606566	17781341	17781341	0.0944493	0	1	0.2476444	65.45028
53172	cg02611240	22734449	22734449	0.250102	0	1	0.0575675	-107.9412
53217	cg02613370	17799351	17634499	0.5362187	0	1	0.3912513	-33.99244
53244	cg02614549	32703318	32703318	0.7219862	0	1	0.8526608	49.66444
53364	cg02620335	31791443	31791443	0.5648934	0	1	0.6748021	23.35627
53407	cg02622251	38804348	38804348	0.5770063	0	1	0.6812023	21.66859
53433	cg02623604	61753476	17783396	0.2676648	0	1	0.1438546	-39.03301
53495	cg02626269	50637301	50637301	0.7385549	0	1	0.9320853	128.8492
53530	cg02628016	59615327	59615327	0.5589347	0	1	0.6695458	23.41905
53648	cg02632314	27615380	27615380	0.4673632	0	1	0.6357874	45.74184
53662	cg02633036	25763469	25763469	0.3221044	0	1	0.447286	27.47062
53851	cg02640147	44716428	44716428	0.3016914	0	1	0.470838	46.81897
53890	cg02642117	40687465	40687465	0.5915287	0	1	0.7172577	31.13506
53921	cg02643580	26774414	26774414	0.2934281	0	1	0.4678902	49.84671
54036	cg02649063	16701425	16701425	0.585595	0	1	0.6894001	21.82572
54102	cg02653117	71715378	60658342	0.304143	0	1	0.4224251	25.50432
54222	cg02659217	18801476	18801476	0.3201953	0	1	0.4238216	20.25095
54248	cg02660643	59607315	59607315	0.519718	0	1	0.6243448	20.33861
54368	cg02667103	35784414	35784414	0.5102207	0	1	0.6203215	21.99783
54586	cg02678768	14641394	39634389	0.4445547	0	1	0.3361188	-21.45051

54655	cg0268357	16704484	16704484	0.5849316	0	1	0.6909274	22.6129
54691	cg0268666	44685306	44685306	0.8650162	0	1	0.7592995	-38.38007
54785	cg0269211	29670508	29670508	0.664956	0	1	0.7707963	26.91746
54813	cg0269322	27785423	27785423	0.4505386	0	1	0.6626707	70.9939
54928	cg0269858	47740452	35810392	0.6554446	0	1	0.5453252	-22.82586
54968	cg0270010	66802462	66802462	0.4594787	0	1	0.5634323	19.38744
54997	cg0270102	56803304	56803304	0.672082	0	1	0.7782823	27.64913
55014	cg0270244	66611456	66611456	0.3185478	0	1	0.0404804	-196.6535
55030	cg0270324	47632385	47632385	0.8600272	0	1	0.6638431	-94.6985
55053	cg0270448	17772323	17772323	0.6691594	0	1	0.7897791	34.86643
55184	cg0271029	65777339	10733343	0.5494024	0	1	0.3525682	-59.74054
55222	cg0271166	19654361	19654361	0.8525824	0	1	0.7348357	-42.47304
55252	cg0271258	67736389	67736389	0.7030313	0	1	0.6000983	-22.05582
55330	cg0271630	64765382	64765382	0.6139092	0	1	0.4973323	-24.01914
55335	cg0271649	66661469	66661469	0.295307	0	1	0.4199241	28.07097
55387	cg0271922	61739485	61739485	0.7320845	0	1	0.8802692	67.92042
55393	cg0271963	57633449	57633449	0.8591261	0	1	0.746266	-41.08969
55444	cg0272259	57608460	57602461	0.3794911	0	1	0.2740677	-22.08952
55463	cg0272339	12798321	69766403		0	1	0.0267209	4.34E-09
55565	cg0272748	10611473	10611473	0.1959111	0	1	0.31571	31.70716
55615	cg0273030	18609364	18609364	0.2242887	0	1	0.0423244	-107.4237
55623	cg0273086	35730376	35730376	0.1709745	0	1	0.2760911	27.75393
55702	cg0273505	64732455	72650504	0.8350692	0	1	0.7015931	-47.71207
55708	cg0273542	24708417	24708417	0.8485552	0	1	0.5395985	-190.7297
55797	cg0273928	22683502	22683502	0.5479608	0	1	0.6706976	27.74738
55835	cg0274132	66636431	66636431	0.8404231	0	1	0.373017	-371.3336
55891	cg0274363	30668416	30668416	0.5050168	0	1	0.6461591	33.99766
55933	cg0274578	40672311	40672311	0.6324273	0	1	0.4253642	-66.48005
56067	cg0275293	28722350	28722350	0.1829682	0	1	0.4388489	115.9998
56102	cg0275494	28662413	28662413	0.512208	0	1	0.4062653	-19.92576
56140	cg0275668	49771487	49771487	0.655277	0	1	0.7950402	44.64226
56163	cg0275757	74665399	74665399	0.6577504	0	1	0.4992469	-41.95947
56371	cg0276763	35693455	11653451	0.2374446	0	1	0.364238	31.626
56394	cg0276867	37621403	37621403	0.5332478	0	1	0.6925833	44.21772
56527	cg0277358	48657414	17667350	0.7459394	0	1	0.64452	-23.60898
56608	cg0277665	25789310	25789310	0.1281171	0	1	0.2297321	30.63873
56748	cg0278607	25802510	25802510	0.2724483	0	1	0.373575	20.74245
56777	cg0278798	38673342	59767330	0.5514458	0	1	0.4349332	-23.32553
56828	cg0279069	44660376	44660376	0.2986192	0	1	0.3989334	19.72582
56874	cg0279332	74753409	74753409	0.1785468	0	1	0.3584966	63.93426
56900	cg0279492	41734374	41734374	0.8118175	0	1	0.6564318	-55.62379
56932	cg0279647	47728452	47728452	0.2428308	0	1	0.3680468	30.67732
57041	cg0280250	54621330	54621330	0.0787037	0	1	0.2875847	111.1756

57200	cg02811434	29749452	29749452	0.8954307	0	1	0.6356049	-165.4051
57224	cg02812891	35743415	15771381	0.13205	0	1	0.2960579	62.98347
57241	cg02814135	45768426	45768426	0.5493886	0	1	0.8223261	144.4944
57328	cg02819655	57804497	57804497	0.5564154	0	1	0.7799467	92.77396
57341	cg02820413	55667427	55667427	0.5092604	0	1	0.6399505	29.68904
57440	cg02826233	10684376	10684376	0.5339791	0	1	0.6372038	20.16105
57453	cg02827061	39741477	39741477	0.2928917	0	1	0.4087482	25.00449
57557	cg02832512	17780384	17780384	0.4905379	0	1	0.6895947	64.96909
57631	cg02836233	29694415	29632381	0.7359896	0	1	0.9148808	105.7346
57666	cg02837536	23802370	41627337	0.5851619	0	1	0.4675988	-23.92549
57715	cg02839824	54743405	54743405	0.459275	0	1	0.3512388	-21.07258
57781	cg02843349	16645310	16645310	0.2078092	0	1	0.3399639	36.00541
57838	cg02847220	32642474	32642474	0.1689623	0	1	0.3018381	40.21508
57873	cg02848835	71766444	71766444	0.8438092	0	1	0.9628161	86.86865
57881	cg02849507	51739320	51739320	0.2104454	0	1	0.3117939	23.54754
58022	cg02854902	33789400	33789400	0.4292993	0	1	0.5434766	22.52939
58130	cg02859554	61635426	65725476	0.4112353	0	1	0.2321517	-56.72171
58164	cg02861178	67749342	67749342	0.2561886	0	1	0.3590929	21.90876
58231	cg02864638	17648329	17648329	0.3073495	0	1	0.4756558	46.16729
58354	cg02871313	14607466	14607466	0.7854568	0	1	0.6845468	-26.12533
58379	cg02872767	38641425	28689384	0.8261415	0	1	0.6611586	-64.09856
58404	cg02873783	31665328	31665328	0.3718165	0	1	0.104539	-150.1653
58468	cg02877261	15650309	15650309	0.4886603	0	1	0.221096	-118.5856
58534	cg02880169	62794434	62794434	0.2818074	0	1	0.4175724	32.90649
58624	cg02884661	36660387	36660387	0.7295953	0	1	0.6135654	-28.07707
58746	cg02890235	64667483	64667483	0.76537	0	1	0.2863828	-371.3336
58749	cg02890259	67607477	67607477	0.0615517	0	1	0.2677569	117.0495
58752	cg02890368	70618332	23673423	0.3534715	0	1	0.2443443	-24.55974
58915	cg02897667	28808457	28808457	0.3885963	0	1	0.256864	-32.50154
58927	cg02898159	31806316	31806316	0.2357237	0	1	0.3490851	26.52897
58999	cg02901522	50743364	50743364	0.6539343	0	1	0.4862311	-46.11378
59175	cg02909570	57637340	57637340	0.6400204	0	1	0.8692443	124.4137
59200	cg02910494	48751465	48751465	0.8499777	0	1	0.665711	-82.84892
59271	cg02914097	19613479	19613479	0.5850543	0	1	0.7024533	27.13399
59368	cg02918253	74716395	71640376	0.2696206	0	1	0.1581115	-31.65674
59381	cg02919082	13612435	13612435	0.3791015	0	1	0.5076897	27.71315
59390	cg02919564	60730314	13641509	0.02886	0	1	0.1600752	74.68999
59410	cg02920196	33797315	33797315	0.5436841	0	1	0.7121131	50.09339
59468	cg02922734	50807418	50807418	0.6544135	0	1	0.7998989	48.27808
59620	cg02929371	27744394	65657383	0.3529751	0	1	0.1421148	-91.39391
59679	cg02931604	27668432	27668432	0.8049569	0	1	0.6565455	-50.61364
59743	cg02934719	64691421	64691421	0.3869179	0	1	0.4892555	18.93824
59782	cg02936593	33623322	33623322	0.63216	0	1	0.8296803	86.21307

59804	cg02937671	38742373	38742373	0.4712198	0	1	0.3618225	-21.35755
59815	cg0293806	50657379	15635405	0.760397	0	1	0.8990289	68.00166
59931	cg02944007	46759419	47701300	0.7068905	0	1	0.5869141	-28.31994
60072	cg02951719	61754442	61754442	0.838622	0	1	0.6513644	-81.59679
60156	cg02954704	44606340	44606340	0.5782227	0	1	0.6831238	21.9662
60322	cg02961798	42782402	42782402	0.2889422	0	1	0.4013418	23.93868
60334	cg02962597	17628444	17628444	0.4551424	0	1	0.5558054	18.36466
60384	cg02964385	23729497	23729497	0.7888586	0	1	0.9083361	58.26189
60405	cg02965295	40706498	40706498	0.1579178	0	1	0.2667973	30.53663
60426	cg02966475	62807390	62807390	0.4451267	0	1	0.6000808	38.62899
60450	cg02968094	57674469	57674469	0.3047724	0	1	0.4176747	23.63343
60462	cg02968537	10625360	10625360	0.7519359	0	1	0.8661215	43.23056
60677	cg02978201	39717400	39717400	0.5343648	0	1	0.7551358	86.34249
60706	cg02979711	62796426	62796426	0.2156268	0	1	0.3340989	29.70193
60717	cg02980047	65768429	65768429	0.3820184	0	1	0.5402244	39.75377
60777	cg0298309	36689346	36689346	0.2075979	0	1	0.3246459	29.69143
60788	cg02983911	73756507	73756507	0.6133736	0	1	0.7159575	22.48844
60952	cg02992067	64607371	64607371	0.6590289	0	1	0.7664607	27.21629
61052	cg02996355	19683374	42635509	0.4543187	0	1	0.3230792	-29.69384
61121	cg0299939	74772397	25804416	0.675084	0	1	0.5432359	-31.39637
61200	cg03003335	29612384	53756368	0.3400439	0	1	0.503265	42.68961
61281	cg03007623	25767405	62797494	0.1376031	0	1	0.283172	51.27799
61396	cg0301432	21794330	42801352	0.2380085	0	1	0.1180171	-41.01349
61406	cg03014829	51660482	51660482	0.647522	0	1	0.4515947	-60.59442
61475	cg03018931	19795429	62677307	0.3912751	0	1	0.5118592	24.77256
61486	cg03019812	47769345	47769345	0.3505855	0	1	0.6277224	117.8952
61491	cg0302000	44797510	16750437	0.8325795	0	1	0.7255087	-33.81332
61495	cg03020379	14783428	14783428	0.9143205	0	1	0.7325081	-107.7949
61504	cg03020684	71641342	71641342	0.5031481	0	1	0.7826048	138.6117
61544	cg03022891	74698328	19758354	0.7586831	0	1	0.58969	-55.14503
61571	cg03024489	39704422	39704422	0.7987553	0	1	0.6574794	-45.92762
61611	cg03025825	49712375	49712375	0.1771186	0	1	0.2774437	25.36258
61612	cg0302583	28705352	36682333	0.6333725	0	1	0.5288313	-20.49029
61618	cg03026119	66747309	66747309	0.4221438	0	1	0.5230502	18.37323
61663	cg03028786	16691439	16691439	0.373799	0	1	0.2363728	-36.12506
61684	cg03030267	43711485	43711485	0.3492257	0	1	0.5446337	58.99117
61788	cg03035213	57606356	46785348	0.6431295	0	1	0.3995139	-91.2673
61896	cg03040423	74732478	74732478	0.9552442	0	1	0.8361211	-81.489
61955	cg03043127	74784429	74784429	0.7870818	0	1	0.6402945	-47.13943
61989	cg03044281	45628426	45628426	0.3504729	0	1	0.4573827	20.73009
62089	cg03048372	36677492	47662333	0.7607927	0	1	0.6502753	-28.01112
62092	cg03048488	26676361	26676361	0.1964829	0	1	0.3590012	51.76283
62097	cg03048921	34718455	34718455	0.5938275	0	1	0.8496003	139.0537

62107	cg0304944!	69758346	69758346	0.6321641	0	1	0.7572389	33.72087
62156	cg0305200	67625419	67625419	0.7984551	0	1	0.9109881	54.49392
62171	cg0305250	31713501	40601382	0.4678152	0	1	0.351877	-23.63286
62188	cg0305335	71775473	31601472	0.4834427	0	1	0.7462232	116.5109
62207	cg0305398	64799492	64799492	0.3481477	0	1	0.4530445	20.13968
62303	cg0305841	44779447	44779447	0.4363786	0	1	0.5380269	18.5986
62331	cg0305987	19659320	64653445	0.2522212	0	1	0.3770965	30.02832
62388	cg0306294	33708469	57620507	0.4493307	0	1	0.6401196	57.41329
62433	cg0306509	31620397	31620397	0.2101744	0	1	0.3103386	23.13916
62532	cg0306973	58731498	58731498	0.2793725	0	1	0.4456541	46.56261
62575	cg0307208	22794385	22794385	0.0687551	0	1	0.2643136	105.0335
62651	cg0307588	20639350	20639350	0.502463	0	1	0.2472484	-105.0351
62686	cg0307800	29670350	51658304	0.5354564	0	1	0.6576177	27.07607
62694	cg0307836	37712341	24651301	0.793761	0	1	0.6781958	-33.03359
62734	cg0308033	16655374	16655374	0.3773166	0	1	0.4976463	24.79751
62793	cg0308418	52659317	67714456	0.1520565	0	1	0.3549166	83.45274
62837	cg0308670	23709502	23709502	0.2925703	0	1	0.3944398	20.3714
62872	cg0308895	65682469	65682469	0.1946908	0	1	0.3478983	47.18609
62879	cg0308971	54644378	54644378		0	1		-4.34E-09
63009	cg0309713	20764406	20764406	0.5412318	0	1	0.692463	40.46712
63092	cg0310249	54735359	29622494	0.6314608	0	1	0.5257484	-20.81814
63148	cg0310555	36725473	36725473	0.5478951	0	1	0.4374672	-21.32525
63188	cg0310807	69757501	42616429	0.5522762	0	1	0.4364722	-23.09198
63227	cg0310991	21688303	21688303	0.1526918	0	1	0.3278771	65.57548
63272	cg0311251	69725325	69725325	0.6874291	0	1	0.5836226	-21.75567
63328	cg0311553	54616458	54616458	0.3781948	0	1	0.515346	30.95581
63338	cg0311601	27631430	27631430	0.2937029	0	1	0.4047323	23.3241
63375	cg0311761	46765327	46765327	0.2800465	0	1	0.4092774	30.40187
63420	cg0312014	10786355	33658325	0.8938226	0	1	0.7751781	-52.91061
63425	cg0312046	43717452	72646479	0.5870118	0	1	0.440071	-35.00428
63463	cg0312292	52703317	52703317	0.536204	0	1	0.2982558	-87.86978
63478	cg0312370	27808379	27808379	0.0748012	0	1	0.266215	99.18104
63532	cg0312673	72746442	72746442	0.2671841	0	1	0.4702105	67.23669
63552	cg0312758	51677413	51677413	0.2437076	0	1	0.3936278	41.15914
63576	cg0312849	50803403	50803403	0.2806188	0	1	0.3922864	23.96792
63624	cg0313091	18723328	71768350	0.3438088	0	1	0.5464743	63.28363
63676	cg0313340	67624445	67624445	0.8930849	0	1	0.7894709	-43.39633
63717	cg0313594	16702378	16702378	0.8221748	0	1	0.9367501	66.87945
63721	cg0313615	72796315	72796315	0.7418388	0	1	0.9251688	115.5027
63724	cg0313649	25795438	25795438	0.5089836	0	1	0.6142426	20.35665
63840	cg0314274	53804372	53804372	0.3658462	0	1	0.4850114	24.52642
63861	cg0314359	72635510	72635510	0.7743165	0	1	0.9014348	60.98091
63965	cg0314785	61755437	61755437	0.6597325	0	1	0.7625607	25.16676

64063	cg0315250(	20603307	30748437	0.7410886	0	1	0.6278043	-27.73442
64125	cg0315654(	54620402	54620402	0.5448032	0	1	0.6809006	33.35898
64295	cg0316501⁄	37801383	26609473	0.2045365	0	1	0.0950136	-39.60526
64296	cg0316517(	25652440	25652440	0.5039909	0	1	0.3883477	-23.12007
64343	cg0316788:	40793322	40793322	0.2860181	0	1	0.4453466	42.90721
64649	cg0318370(	27689358	27689358	0.6664761	0	1	0.9088972	156.5356
64674	cg0318477(	64615357	64615357	0.4747957	0	1	0.5946598	24.82888
64686	cg0318570⁄	46675353	46675353	0.4930738	0	1	0.3865386	-20.21539
64726	cg0318766ξ	65795451	65795451	0.7075221	0	1	0.5468251	-45.90239
64742	cg0318839(	40618341	40618341	0.583124	0	1	0.4623162	-25.01106
64751	cg0318902:	58780320	40648391	0.4646834	0	1	0.3631201	-18.93001
64967	cg0320133:	64645445	64645445	0.184081	0	1	0.3137348	37.06762
64976	cg0320206(	22773327	65733324	0.3816589	0	1	0.211026	-54.38079
65185	cg0321320:	71778483	19757345	0.4109575	0	1	0.2441514	-49.23264
65371	cg0322139(	23660495	23660495	0.8114033	0	1	0.232072	-371.3336
65373	cg0322148:	32772437	32772437	0.5687267	0	1	0.6832715	25.27058
65442	cg0322462:	65721411	65721411	0.3710231	0	1	0.496402	26.63685
65456	cg0322544⁄	36697398	38670430	0.6120681	0	1	0.4830543	-28.38307
65540	cg0322903:	61601301	12768448	0.3547814	0	1		-4.34E-09
65740	cg03240981	23786387	23786387	0.6117014	0	1	0.738581	33.0176
65838	cg0324647ξ	74694436	74694436	0.3396165	0	1	0.4433999	19.93281
65969	cg0325433(	14684453	14684453	0.2377247	0	1	0.3420901	23.1979
66000	cg0325609(	35665324	46752368	0.2252183	0	1	0.1233847	-31.33154
66006	cg0325668⁄	48703475	55689305	0.4025598	0	1	0.2958796	-21.8199
66024	cg0325742:	11697450	11697450	0.2451067	0	1	0.3540328	24.45273
66038	cg0325813ς	25724501	25724501	0.3102023	0	1	0.4347669	27.57458
66083	cg03260621	32632363	32632363	0.3836093	0	1	0.5372689	37.72635
66126	cg0326269(	30772379	30772379	0.5910429	0	1	0.7170851	31.24999
66140	cg0326319:	36697485	36697485	0.1705744	0	1	0.2796466	29.4134
66159	cg0326413:	34617348	15745431	0.5621538	0	1	0.243724	-163.462
66166	cg0326455(	20767486	68790453	0.3244422	0	1	0.2017491	-32.44657
66180	cg0326526ξ	38785503	38785503	0.6515241	0	1	0.758857	26.63012
66252	cg0326936:	74623352	74623352		0	1	0.7857058	4.34E-09
66284	cg0327148:	29659310	29659310	0.4762709	0	1	0.6719469	61.77413
66294	cg0327190:	49612398	49612398	0.3479614	0	1	0.4535728	20.36345
66311	cg0327249ς	45687351	45687351	0.777857	0	1	0.892138	49.66611
66313	cg0327293:	26785499	26785499	0.7212726	0	1	0.8412647	41.61351
66380	cg0327688:	24762468	24762468	0.5490087	0	1	0.6506964	19.99161
66381	cg0327692(	38610449	38610449	0.6321263	0	1	0.8723428	135.2323
66393	cg0327755(	15760501	55775356	0.9302716	0	1	0.7876168	-85.37446
66558	cg0328704(	24810478	24810478	0.3038893	0	1	0.4117783	21.99423
66661	cg0329222:	48643368	40807439	0.9422039	0	1	0.7550021	-130.7037
66675	cg0329320(	71631447	10656419	0.8926784	0	1	0.7721364	-53.77606

66679	cg03293507	65794484	67747316	0.2711247	0	1	0.378318	22.78388
66733	cg03296304	43767507	53655437	0.2533529	0	1	0.5323856	124.1077
66855	cg03302281	17625478	17625478	0.5036703	0	1	0.6111256	20.9922
66940	cg03307177	36784509	36784509	0.8281302	0	1	0.7080747	-39.51239
66947	cg03307659	29779377	21661421	0.6392015	0	1	0.507629	-30.00267
67054	cg03312406	35720308	35720308	0.4400452	0	1	0.5966074	39.28075
67060	cg03312587	12722321	12722321	0.3727398	0	1	0.5413052	44.65098
67098	cg03314029	36773421	19738374	0.7263099	0	1	0.5288669	-67.47447
67131	cg03315469	32760433	15738422	0.5140034	0	1	0.8827224	374.3439
67371	cg03327325	56714412	56714412	0.3734586	0	1	0.2478622	-30.54495
67395	cg03328299	72790425	72790425	0.3284386	0	1	0.4980368	46.03003
67425	cg03329597	30766334	30766334	0.3039433	0	1	0.4860495	53.26237
67427	cg03329714	17746301	17746301	0.7474611	0	1	0.8790839	56.79912
67504	cg03333149	61768320	73756355		0	1		-4.34E-09
67545	cg03335262	61766455	67632339	0.462972	0	1	0.3063478	-40.75814
67571	cg03337057	69804327	64785367	0.5065246	0	1	0.3394868	-44.49525
67660	cg03340754	38784345	38784345	0.5279606	0	1	0.4034443	-26.09268
67675	cg03341641	41716440	41716440	0.5579544	0	1	0.7338946	56.061
67684	cg03342032	24770426	24770426	0.248874	0	1	0.3570396	24.01302
67713	cg03343571	20631385	65602498	0.0266368	0	1	0.253008	156.5356
67846	cg03349091	47708439	47708439	0.7369801	0	1	0.6224329	-27.96018
67869	cg03350138	23625367	23625367	0.6495028	0	1	0.5433753	-21.36354
67961	cg03354554	44712471	44712471	0.7122416	0	1	0.8171164	30.85732
67963	cg03354616	52704320	52704320	0.4880176	0	1	0.709479	80.77098
67999	cg03356172	47793323	47793323	0.5318978	0	1	0.6608149	29.69736
68033	cg03357999	64655397	63787408	0.5298198	0	1	0.3155503	-71.20322
68254	cg03370193	35655452	43662303	0.5967351	0	1	0.4931015	-19.6027
68330	cg03373960	61720304	61720304	0.6559454	0	1	0.7561769	23.78902
68331	cg03374148	66647422	66647422	0.6689925	0	1	0.7747427	27.18649
68387	cg03378197	63667510	24806498	0.4451108	0	1	0.3279701	-24.49286
68532	cg03386715	21618356	21618356	0.6223033	0	1	0.8494228	114.7229
68600	cg03389890	73783404	27792304	0.4379037	0	1	0.3176101	-25.83728
68641	cg03392100	62733384	62733384	0.9012131	0	1	0.7905684	-50.04179
68757	cg03398919	41748327	41748327	0.7091421	0	1	0.5648351	-38.43362
68759	cg03398989	26691355	26691355	0.8445033	0	1	0.412993	-361.1803
68834	cg03401875	47738484	47738484	0.66998	0	1	0.5564182	-24.44328
68966	cg03408135	10759351	10759351	0.3702856	0	1	0.1741872	-74.55656
68971	cg03408497	40659498	40659498	0.5095244	0	1	0.6368232	28.33098
69043	cg03412618	19668486	19668486	0.2725483	0	1	0.4319811	43.68996
69130	cg03418136	42620358	47803332	0.9728447	0	1	0.860968	-88.56892
69172	cg03420970	59754449	59754449	0.8613195	0	1	0.5683349	-179.9176
69206	cg03422552	17711503	17711503	0.5108354	0	1	0.6552935	35.70187
69207	cg03422583	33804439	33804439	0.5250508	0	1	0.825414	374.3439

69209	cg03422749	46741444	46741444	0.7036464	0	1	0.8177525	35.16476
69319	cg03428619	66699313	66699313	0.6646553	0	1	0.5327565	-30.98973
69356	cg03431028	17719341	17719341	0.7105713	0	1	0.8107383	28.1186
69380	cg03432172	40652429	40652429	0.4328155	0	1	0.5639415	28.55441
69383	cg03432241	60770382	60770382	0.1994723	0	1	0.3114417	28.25581
69387	cg03432589	39795458	39795458	0.4317319	0	1	0.210166	-85.40671
69405	cg03433313	52731440	52731440	0.5839351	0	1	0.6986109	25.95921
69443	cg03434907	68617390	68617390	0.4593382	0	1	0.587404	27.63964
69523	cg03438552	74622436	14758435	0.3399796	0	1	0.4456719	20.51713
69590	cg03441493	23789460	23789460	0.0794624	0	1	0.2238951	64.18126
69596	cg03441844	29779397	29779397	0.3580693	0	1	0.22555	-34.78594
69632	cg03443143	44675453	11721413	0.3008666	0	1	0.195337	-26.04642
69865	cg03455107	28621319	28621319	0.4518984	0	1	0.552866	18.44105
69870	cg03455316	49799466	27783425	0.826606	0	1	0.7200844	-32.76095
70053	cg03464416	62602353	62602353	0.5167838	0	1	0.4048104	-21.83135
70112	cg03466587	37758489	37758489	0.5727733	0	1	0.683242	23.84743
70118	cg03466780	29736451	29736451	0.6080379	0	1	0.253856	-204.123
70132	cg03467235	14657490	14657490	0.3749022	0	1	0.4823554	20.59957
70188	cg03469682	72709302	72709302	0.1586619	0	1	0.261	27.70803
70191	cg03469766	40613300	40613300	0.7481936	0	1	0.9313701	119.415
70472	cg03485667	52759305	52759305	0.3103098	0	1	0.4289208	25.44264
70488	cg03486379	65799476	65799476	0.1259738	0	1	0.2388229	36.19925
70502	cg03487027	61625442	24710412	0.5768982	0	1	0.719795	38.54693
70521	cg03487983	13617335	13617335	0.6540635	0	1	0.7565265	24.63263
70579	cg03491895	62794477	62794477	0.1074061	0	1	0.2107418	34.30007
70623	cg03494612	42732495	42732495	0.6382176	0	1	0.9209131	374.3439
70634	cg03495084	65757507	65757507	0.4808925	0	1	0.6713472	58.80442
70699	cg03498886	42799501	42799501	0.3675101	0	1	0.2658285	-21.14772
70853	cg03507593	51754433	13666340	0.229197	0	1	0.3562538	32.25643
70889	cg03509467	58778459	58778459	0.4559609	0	1	0.7072551	101.8517
70997	cg03514086	28743489	28743489	0.4113219	0	1	0.3101971	-19.69691
71065	cg03517284	20641477	53717306	0.5527264	0	1	0.3258087	-79.03867
71093	cg03518729	66687328	66687328	0.6125053	0	1	0.7946221	68.20657
71338	cg03529900	51795445	51795445	0.7352316	0	1	0.8429879	35.66042
71386	cg03532013	34623394	57743396	0.2875279	0	1	0.4387253	39.15532
71450	cg03534453	41700477	41700477	0.6786669	0	1	0.5062319	-49.73705
71510	cg03536885	51663335	51663335	0.2478392	0	1	0.3569957	24.40819
71660	cg03543861	52790378	64740380	0.5872692	0	1	0.4762505	-21.79502
71964	cg03557725	50699401	50699401	0.4776279	0	1	0.349286	-28.03008
72053	cg03562350	17636481	55688419	0.559204	0	1	0.377744	-51.0819
72088	cg03563842	54607508	20655340	0.7320652	0	1	0.8451912	38.72309
72213	cg03570263	38765376	38765376	0.7734368	0	1	0.6548809	-32.34997
72357	cg03577157	69605321	69605321	0.5705237	0	1	0.7021099	32.61829

72455	cg03582285	21687304	21687304	0.4389023	0	1	0.2735006	-46.47985
72533	cg03585888	60646402	60646402	0.6548398	0	1	0.3511242	-143.6871
72549	cg03586820	72795423	72795423	0.6454001	0	1	0.5121335	-30.84225
72655	cg03592824	40639392	40639392	0.2859149	0	1	0.388122	20.66794
72686	cg03594506	20602395	20602395	0.4608296	0	1	0.5679458	20.38603
72695	cg03594907	59742309	59742309	0.9275497	0	1	0.8187574	-58.27331
72708	cg03595348	65622382	65622382	0.6559044	0	1	0.4193611	-86.78516
72771	cg03598159	10604387	10604387	0.2803742	0	1	0.1443025	-45.11832
72795	cg03599197	19632507	19632507	0.3177539	0	1	0.4210176	20.19037
72830	cg03601053	38759429	38759429	0.3711364	0	1	0.5648552	57.7693
72936	cg03605116	69666366	69666366	0.6558692	0	1	0.5496073	-21.57333
73347	cg03626579	46612344	46612344	0.6852565	0	1	0.5738766	-24.23801
73447	cg03631455	14740468	68777388	0.4422379	0	1	0.2052878	-97.08677
73514	cg03635442	42783481	43778393	0.1225399	0	1	0.2271272	32.72548
73553	cg03637769	31719455	24706345	0.5486533	0	1	0.6715567	27.84144
73703	cg03645522	17765492	31614469	0.1288839	0	1	0.311371	75.32765
73764	cg03648679	13644314	13644314		0	1	0.0886288	4.34E-09
73784	cg03649293	24798428	24798428	0.670414	0	1	0.4679156	-65.63519
73867	cg03653573	42760314	42760314	0.6120999	0	1	0.7136736	22.04394
73870	cg03653726	31769394	27709421	0.8545868	0	1	0.705676	-60.95823
73887	cg03654299	14661378	68652322	0.343896	0	1	0.2274539	-28.15807
73979	cg03658557	30737368	30737368	0.5004591	0	1	0.6449187	35.32215
74023	cg03660901	38787488	38787488	0.4170183	0	1	0.5253922	20.65511
74034	cg03661387	20751449	20751449	0.7226513	0	1	0.8283706	32.61577
74089	cg03664443	24714510	24714510	0.3432446	0	1	0.4908634	35.72712
74178	cg03668763	17638311	17638311	0.1655544	0	1	0.2675365	26.9499
74269	cg03672997	21735405	21735405	0.2128672	0	1	0.3687781	46.61259
74364	cg03679269	10777355	18679416	0.4994668	0	1	0.3739174	-26.66544
74412	cg03682112	69640406	46602385	0.7642501	0	1	0.6628082	-24.75926
74420	cg03682581	10616328	10616328	0.1354536	0	1	0.2676596	44.49441
74424	cg03682719	49754473	58606305	0.515417	0	1	0.3560024	-40.61189
74441	cg03683899	30785408	48621402	0.1608287	0	1	0.0556844	-46.22451
74489	cg03686964	49768495	49768495	0.6101272	0	1	0.4901566	-25.11125
74711	cg03699074	23641470	23641470	0.7620561	0	1	0.6508655	-28.37199
74727	cg03699765	35785392	35785392	0.6372208	0	1	0.8403605	93.30875
74854	cg03705784	39696395	39696395	0.6709207	0	1	0.7973119	38.39444
74921	cg03709428	55726380	55726380	0.4914461	0	1	0.3751039	-23.46711
75068	cg03716984	47778337	37801394	0.5666075	0	1	0.4630089	-19.30089
75136	cg03719428	50610459	50610459	0.3404592	0	1	0.2387213	-22.24828
75193	cg03721887	65787500	65787500	0.9243839	0	1	0.6712942	-178.1602
75230	cg03723481	74699400	74699400	0.7497771	0	1	0.8768048	53.31104
75271	cg03725115	63676473	69618420	0.1213002	0	1	0.2593617	50.12405
75508	cg03736235	36778506	36778506	0.3034489	0	1	0.4102725	21.66004

75529	cg03737424	31720431	31720431	0.5371137	0	1	0.6469983	22.52061
75581	cg03739955	62631370	62631370	0.4542488	0	1	0.30343	-38.30273
75657	cg03743861	47696316	18739419	0.4253424	0	1	0.5884994	42.15722
75666	cg03744383	39625318	39625318	0.2910418	0	1	0.3944592	20.90527
75689	cg03745491	21769466	21769466	0.2181913	0	1	0.339134	30.51537
75739	cg03748376	23756382	36759456	0.0827994	0	1	0.2941691	111.4038
75753	cg03749207	32766445	20798314	0.2221185	0	1	0.0914314	-52.33752
75769	cg03750315	42668374	62616418	0.5028204	0	1	0.3870043	-23.18723
75798	cg03751967	21636307	26676348	0.5258483	0	1	0.4019244	-25.88726
75867	cg03754882	45731391	45731391	0.355326	0	1	0.5979172	89.60994
75985	cg03761216	26675439	26675439	0.3576562	0	1	0.4767795	24.6237
76113	cg03767353	65697342	43604403	0.1321144	0	1	0.4052435	145.0743
76250	cg03773862	16713429	16713429	0.4997854	0	1	0.6151202	23.62337
76269	cg03774803	58629324	58727362	0.3143368	0	1	0.2028637	-27.82084
76328	cg03777875	59709412	27678374	0.4240888	0	1	0.5435477	24.30483
76334	cg03778207	59698354	59698354	0.1496094	0	1	0.2738301	38.46903
76400	cg03781748	23616336	23616336	0.5237368	0	1	0.6853552	44.84992
76445	cg03784363	63810344	11686466	0.5317448	0	1	0.6826223	39.74437
76455	cg03784886	72630371	72630371	0.691379	0	1	0.7920093	26.56031
76555	cg03790740	38691422	38691422	0.7853925	0	1	0.6612133	-36.00604
76660	cg03796943	60733357	60733357	0.4973647	0	1	0.6277229	29.23317
76686	cg03798915	12611322	65706440	0.3947006	0	1	0.2942289	-19.88879
76816	cg03805357	48606424	48606424	0.8862692	0	1	0.7700229	-49.25449
76866	cg03808001	51627426	51627426	0.1751868	0	1	0.3344626	52.8019
76895	cg03810198	33712327	33712327	0.9243516	0	1	0.7548982	-103.4992
76896	cg03810282	69694309	69694309	0.6433545	0	1	0.8817958	138.2105
76941	cg03813151	38671404	63737358	0.689262	0	1	0.498844	-60.00003
76969	cg03814957	14746308	14746308	0.1792828	0	1	0.2826737	26.3911
76991	cg03816625	61605441	68702328	0.9135029	0	1	0.8132669	-47.14553
77108	cg03823901	18676319	18676319	0.4052261	0	1	0.5100851	19.59791
77163	cg03826976	60791386	60791386	0.321767	0	1	0.1978168	-33.2997
77164	cg03827118	71608382	71608382	0.8369331	0	1	0.7274372	-35.60378
77210	cg03830006	22735337	22735337	0.6199766	0	1	0.4275178	-57.65442
77297	cg03834116	73629419	73629419	0.5887989	0	1	0.6973295	23.73013
77341	cg03836586	25758330	25758330	0.4228269	0	1	0.5285418	19.82308
77351	cg03837313	29653407	29653407	0.2506523	0	1	0.4300662	55.23475
77361	cg03837909	52677414	52677414	0.3735937	0	1	0.2422116	-33.20248
77392	cg03839782	33725393	33725393	0.5738992	0	1	0.6840203	23.75688
77476	cg03844894	46743301	42629423	0.4190891	0	1	0.317561	-19.65882
77484	cg03845409	21660398	21660398	0.3945526	0	1	0.2790519	-25.35944
77535	cg03847896	47666370	47666370	0.2890778	0	1	0.436025	37.23429
77671	cg03854098	52617426	52617426	0.7697346	0	1	0.6596402	-28.52622
77749	cg03858673	15689482	27753327	0.4819301	0	1	0.3266482	-39.45785

77803	cg03861065	37747316	37747316	0.3245088	0	1	0.4834375	41.15671
77906	cg03867465	20600339	24667343	0.2422971	0	1	0.3851921	38.10147
77921	cg03868770	45755499	45755499	0.4309246	0	1	0.5403146	20.97798
77972	cg03871140	62706422	36772431	0.0792667	0	1	0.3767724	374.3439
78008	cg03873153	20701450	20701450	0.582825	0	1	0.403232	-50.14304
78054	cg03875819	24674469	24674469	0.7076665	0	1	0.8409057	48.6945
78067	cg03876548	54735462	54735462	0.3748675	0	1	0.5053021	28.43615
78136	cg03880506	37672364	17649329	0.2827784	0	1	0.4224078	34.43868
78230	cg03885343	20804380	20804380	0.3957615	0	1	0.5630865	43.9541
78249	cg03886436	44773505	44773505	0.6552508	0	1	0.7609237	26.13599
78265	cg03887438	32679333	32679333	0.4886374	0	1	0.5946276	20.29846
78275	cg03887721	38655438	38655438	0.549849	0	1	0.7231951	53.611
78278	cg03887906	46708323	46708323	0.6050515	0	1	0.7307452	31.97812
78289	cg03888553	17680413	17680413	0.4409047	0	1	0.5489768	20.58437
78456	cg03896685	28760490	28760490	0.388794	0	1	0.5710621	51.48518
78472	cg03897406	14727422	14727422	0.8512599	0	1	0.6212722	-117.7298
78509	cg03899215	45683355	45683355	0.3351305	0	1	0.4681907	30.0853
78532	cg03900028	73681507	73681507	0.3996596	0	1	0.7201023	374.3439
78647	cg03905819	35676433	35676433	0.4746813	0	1	0.248843	-83.43768
78733	cg03910557	41728473	41728473	0.2330527	0	1	0.3429125	25.38408
78762	cg03913278	55660410	55660410	0.4035897	0	1	0.6436369	88.69113
78805	cg03915932	55706303	55706303	0.6428354	0	1	0.50463	-32.70231
78821	cg03916630	13715369	13715369	0.3435929	0	1	0.5184953	48.22385
78920	cg03922251	50715360	50715360	0.6153749	0	1	0.5129048	-19.51903
78924	cg03922381	15647405	15647405	0.6964605	0	1	0.8048183	31.03384
78978	cg03925642	46631507	60719487	0.4652608	0	1	0.6342214	45.94609
79004	cg03927302	21607475	55799503	0.4700404	0	1	0.3362457	-30.34041
79055	cg03930097	64626443	64626443	0.6807285	0	1	0.8773652	101.6938
79180	cg03936031	19732471	19732471	0.8030005	0	1	0.6859914	-34.70386
79274	cg03940883	47771340	47771340	0.5142586	0	1	0.2531964	-109.0094
79330	cg03944375	55626364	55626364	0.4269406	0	1	0.5376198	21.38539
79339	cg03945122	34680310	34680310	0.9219007	0	1	0.8183455	-52.30064
79380	cg03947384	41640303	41640303	0.5754514	0	1	0.6943769	27.32248
79457	cg03951394	27605463	27605463	0.7994831	0	1	0.6889589	-31.41779
79463	cg03951833	60764350	55616472	0.5677499	0	1	0.4406013	-27.11578
79472	cg03952520	30712464	30712464	0.4342769	0	1	0.5768722	33.11036
79493	cg03953626	73703331	52738498	0.1599204	0	1	0.2640299	28.32517
79587	cg03957898	65681454	65681454	0.576456	0	1	0.7203988	39.05753
79599	cg03958308	52675474	52675474	0.3445699	0	1	0.46909	26.7319
79643	cg03960382	55717341	46725335	0.9770927	0	1	0.8632431	-94.24936
79663	cg03961283	35678323	54648301	0.4700567	0	1	0.336354	-30.30275
79665	cg03961401	34642452	34642452	0.7888157	0	1	0.6794767	-29.85758
79673	cg03961824	10714496	72799304	0.4085963	0	1	0.2970542	-23.39611

79732	cg03964373	12623394	12623394	0.867262	0	1	0.4919419	-282.762
79780	cg03966955	46709395	20713394	0.2114293	0	1	0.3325586	31.06457
79791	cg03967448	17763502	17763502	0.4904777	0	1	0.5952593	19.93436
79844	cg03970567	30702313	30702313	0.1564359	0	1	0.3564586	80.58689
79852	cg03970900	29755467	29755467	0.256814	0	1	0.3882062	32.36697
79967	cg03977174	60708333	60708333	0.4106514	0	1	0.5606018	36.06932
79982	cg03977831	65797345	71688451	0.2344157	0	1	0.4245715	62.67719
79985	cg03978067	49650305	49650305	0.9380382	0	1	0.8058623	-81.44724
80037	cg03980550	36689398	36689398	0.4760651	0	1	0.5779344	18.87129
80073	cg03982831	47650436	40804378	0.3760654	0	1	0.4815	19.96445
80186	cg03987818	51643436	51643436	0.7416827	0	1	0.8580146	42.75845
80193	cg03988092	13710427	13710427	0.1417867	0	1	0.3644946	100.1277
80226	cg03989520	49681350	49681350	0.816204	0	1	0.9172961	48.97188
80233	cg03989711	33807483	71810430	0.5481194	0	1	0.8139292	135.2002
80348	cg03995300	41704350	11604489	0.755233	0	1	0.5625631	-68.23992
80386	cg03997145	28610445	28610445	0.3728726	0	1	0.4892297	23.4954
80415	cg03998338	15708503	15708503	0.5767573	0	1	0.7375431	48.51908
80442	cg03999583	28696355	30670361	0.7772583	0	1	0.9754465	374.3439
80461	cg04001071	19737355	19737355	0.5606012	0	1	0.6809327	27.26326
80531	cg04004561	11720499	11720499	0.446358	0	1	0.5935723	35.20842
80643	cg04010296	12795452	12795452	0.3166391	0	1	0.42383	21.45437
80991	cg04026937	41732346	41732346	0.5296656	0	1	0.7607715	94.58603
81027	cg04028570	24670475	10773329	0.0652271	0	1	0.1925806	57.49886
81034	cg04028695	31763342	31763342	0.2790486	0	1	0.439036	43.58172
81051	cg04029664	66718398	31689385	0.534752	0	1	0.423287	-21.63642
81295	cg04045544	41600309	41600309	0.4742397	0	1	0.6711654	62.43921
81355	cg04049719	47707333	47707333	0.1681762	0	1	0.6758328	374.3439
81370	cg04050824	65726481	42808445	0.3862748	0	1	0.2442024	-37.61185
81395	cg04052466	15726311	30744315	0.1707228	0	1	0.0401221	-68.99545
81424	cg04054303	50614476	50614476	0.5398355	0	1	0.7105599	51.13646
81462	cg04056384	65659471	65659471	0.5238913	0	1	0.3953442	-27.5815
81484	cg04057378	61607418	61607418	0.4015113	0	1	0.6704062	112.5771
81516	cg04058752	74773314	53740335	0.5436546	0	1	0.3858602	-39.54559
81568	cg04061372	42790393	42790393	0.1461052	0	1	0.3671507	97.75995
81646	cg04064998	44739322	44739322	0.5576388	0	1	0.7011841	37.59137
81773	cg04071689	13644319	44696344	0.9829204	0	1		-4.34E-09
81788	cg04072301	34790439	17704395	0.6214567	0	1	0.3038459	-158.117
81831	cg04074536	22685497	26702345	0.8480115	0	1	0.6987988	-59.52073
81859	cg04076044	25793345	25793345	0.3834851	0	1	0.4856257	18.90513
81899	cg04077979	22760414	22760414	0.3320453	0	1	0.4448713	22.95504
81944	cg04080091	53682455	53682455	0.9349995	0	1	0.5992186	-278.2814
81948	cg04080282	47697447	16806436	0.3532918	0	1	0.2216025	-34.72027
82011	cg04084173	33785396	33785396	0.737121	0	1	0.6092664	-33.31854

82016	cg0408435[illegible]	19803510	19803510	0.3796192	0	1	0.5092841	28.10407
82087	cg0408847[illegible]	55790411	55790411	0.1178288	0	1	0.2183608	31.4546
82113	cg0408974[illegible]	63791383	63791383	0.8554007	0	1	0.7222791	-51.67941
82132	cg0409079[illegible]	14706411	14706411	0.4358236	0	1	0.5527944	23.48401
82188	cg0409363[illegible]	50690440	50690440	0.3411904	0	1	0.4597333	24.70109
82286	cg0409817[illegible]	40694344	40694344	0.9239377	0	1	0.8215073	-52.28312
82399	cg0410289[illegible]	65796411	51770392	0.2661571	0	1	0.3861063	27.4883
82408	cg0410325[illegible]	70694425	70694425	0.5586307	0	1	0.661999	20.81325
82454	cg0410541[illegible]	62734499	36735379	0.3138981	0	1	0.4328499	25.46405
82687	cg0411646[illegible]	16629405	16629405	0.6133292	0	1	0.7156096	22.36921
82745	cg0411861[illegible]	24633384	24633384	0.0690853	0	1	0.4741311	374.3439
82978	cg0413055[illegible]	60630321	60630321	0.1999053	0	1	0.331923	36.6286
83011	cg0413196[illegible]	65713421	65713421	0.934383	0	1	0.2236527	-371.3336
83026	cg0413241[illegible]	10725414	10725414	0.4229158	0	1	0.273153	-39.2482
83138	cg0413732[illegible]	38671432	38671432	0.2223368	0	1	0.3313859	25.67274
83149	cg0413804[illegible]	28701482	28701482	0.701883	0	1	0.8174673	35.82554
83220	cg0414108[illegible]	48617498	10741347	0.4186882	0	1	0.6191063	62.14251
83273	cg0414334[illegible]	20794319	20794319	0.1690652	0	1	0.2968345	37.79781
83277	cg0414359[illegible]	62748360	62748360	0.3272408	0	1	0.4336496	20.97789
83319	cg0414553[illegible]	29613448	29613448	0.563121	0	1	0.3266778	-85.59254
83325	cg0414568[illegible]	64786405	64786405	0.5232766	0	1	0.0513057	-371.3336
83367	cg0414816[illegible]	72691505	72691505	0.479932	0	1	0.5940154	22.87421
83456	cg0415279[illegible]	69793334	69793334	0.8131344	0	1	0.6765642	-45.63487
83498	cg0415465[illegible]	26748302	31658404	0.4680455	0	1	0.6946056	82.72103
83513	cg0415528[illegible]	66691464	66691464	0.8559113	0	1	0.7510618	-36.2879
83563	cg0415727[illegible]	37726509	40626453	0.4902683	0	1	0.6996026	72.0122
83657	cg0416339[illegible]	32771384	44700368	0.417028	0	1	0.548413	28.59596
83767	cg0416847[illegible]	67712315	67712315	0.1527279	0	1	0.404072	119.639
83790	cg0416974[illegible]	29742458	29742458	0.503036	0	1	0.6907691	58.62139
83843	cg0417234[illegible]	19607404	19607404	0.721167	0	1	0.910812	112.2411
83928	cg0417645[illegible]	28673491	69744457	0.590883	0	1	0.903857	374.3439
83959	cg0417801[illegible]	73754376	73754376	0.8743023	0	1	0.6366229	-133.9316
83992	cg0418004[illegible]	54600475	54600475	0.1647565	0	1	0.2659159	26.6868
84005	cg0418062[illegible]	55606429	55606429		0	1		-4.34E-09
84118	cg0418703[illegible]	20735354	20735354	0.3723914	0	1	0.4845239	22.11115
84144	cg0418892[illegible]	22781319	14717364	0.3292363	0	1	0.4505731	25.92136
84148	cg0418918[illegible]	55635408	34753355	0.5255608	0	1	0.739248	79.23567
84201	cg0419241[illegible]	49720372	49720372	0.2495103	0	1	0.3551477	23.10798
84227	cg0419377[illegible]	29756474	29756474	0.4582918	0	1	0.6089945	36.95764
84284	cg0419594[illegible]	33747415	33747415	0.6904966	0	1	0.7948305	28.32497
84359	cg0420001[illegible]	11606507	11606507	0.6667445	0	1	0.7758063	28.60055
84457	cg0420565[illegible]	23688303	23688303	0.7025515	0	1	0.824071	39.63373
84474	cg0420634[illegible]	10679419	52733447	0.3157426	0	1	0.2068781	-26.54517

84550	cg04209913	14635360	48705467	0.635618	0	1	0.4787018	-40.39012
84656	cg04214938	58754429	17690433	0.5833479	0	1	0.8209072	113.585
84666	cg04215287	29723399	29723399	0.4275618	0	1	0.5342715	20.13122
84709	cg04217177	48747345	48747345	0.589594	0	1	0.7066301	27.20291
84893	cg04225785	63641301	36627303	0.5074603	0	1	0.3766415	-28.56049
84901	cg04226110	21633351	21633351	0.5515469	0	1	0.669337	25.90502
85013	cg04231042	42740411	42740411	0.2365657	0	1	0.3403609	23.05436
85027	cg04231677	26740419	26740419	0.2006339	0	1	0.3334993	36.94058
85062	cg04233620	54649330	54649330	0.7065014	0	1	0.8091908	29.03661
85150	cg04237918	54624473	54624473	0.2108624	0	1	0.3290062	29.89914
85244	cg04242655	43759422	43759422	0.4289553	0	1	0.5297029	18.32401
85296	cg04245540	41682325	41682325	0.1953014	0	1	0.3019128	26.46383
85306	cg04245870	55739300	55739300	0.8598088	0	1	0.6673245	-91.90171
85325	cg04246708	15809316	15809316	0.6233433	0	1	0.7266642	23.25092
85410	cg04251208	12723403	12723403	0.371675	0	1	0.5361431	42.71761
85514	cg04256864	49672435	57665313	0.7328553	0	1	0.6038628	-33.48778
85550	cg04258138	29610361	29610361	0.2044167	0	1	0.3054936	23.80237
85561	cg04258676	64664362	64664362	0.3345172	0	1	0.439041	20.24968
85673	cg04264433	55714325	55714325	0.8257955	0	1	0.7040751	-39.99305
85745	cg04267772	19708438	19708438	0.6734753	0	1	0.5311313	-35.59092
85896	cg04275695	25630462	25630462	0.3052494	0	1	0.4393807	31.34645
85926	cg04276626	65695394	65695394	0.1885428	0	1	0.3217938	38.25949
85938	cg04277055	63702454	63702454	0.4166876	0	1	0.3117034	-20.86631
85961	cg04278168	65795333	65795333	0.6146146	0	1	0.5121018	-19.51968
85976	cg04279411	10740331	10740331	0.4854253	0	1	0.6096587	26.60093
86061	cg04284140	66658300	66658300	0.3845228	0	1	0.2806058	-21.37996
86097	cg04285477	63703394	54606510		0	1		-4.34E-09
86126	cg04286737	54677371	57724403	0.4903956	0	1	0.626947	31.58899
86215	cg04291404	54610397	54610397	0.3299553	0	1	0.4356389	20.69618
86239	cg04292976	57794473	57794473	0.5706849	0	1	0.6729354	20.77428
86331	cg04297406	22686369	22686369	0.6601667	0	1	0.7650251	26.11159
86337	cg04297660	53709380	53709380	0.749018	0	1	0.590394	-48.59758
86345	cg04297995	36722316	36722316	0.61777	0	1	0.7223579	23.4826
86391	cg04301343	31784487	18629332	0.4144304	0	1	0.2829354	-31.16166
86511	cg04306489	20604407	20604407	0.3954047	0	1	0.5204814	26.32563
86544	cg04308089	23717416	23717416	0.1426346	0	1	0.2448223	29.23385
86666	cg04314361	48680374	48680374	0.25214	0	1	0.3951428	37.50583
86701	cg04316537	27713329	27713329	0.9164054	0	1	0.7345114	-109.0422
86743	cg04318327	48628377	48628377	0.4212812	0	1	0.5367271	22.94229
86932	cg04329382	59712409	59712409	0.1068195	0	1	0.2314664	45.52414
86974	cg04331561	34804418	34804418	0.3650765	0	1	0.7273562	374.3439
87001	cg04332927	31677454	31677454	0.6356366	0	1	0.7653466	36.47517
87196	cg04342483	51793470	51793470	0.4276364	0	1	0.3083388	-25.73938

87237	cg0434419(	24707481	24707481	0.2854054	0	1	0.4129149	29.52238
87258	cg0434533:	62676500	62676500	0.6416653	0	1	0.7882924	47.22067
87275	cg0434628:	57650456	57650456	0.4458468	0	1	0.6216109	48.89748
87363	cg0434998:	14680348	14680348	0.2361821	0	1	0.3873725	42.30546
87369	cg0435021:	20649484	20649484	0.6081352	0	1	0.7181373	25.20078
87469	cg0435467	50762343	12708313	0.4925851	0	1	0.3376226	-39.01622
87661	cg0436607(	64714487	64714487	0.2613747	0	1	0.368571	23.15295
87960	cg0438264:	63775343	41620489	0.2937678	0	1	0.1790932	-30.99425
87997	cg0438468!	58699305	58699305	0.7091278	0	1	0.809664	28.16639
88033	cg0438675!	30780483	12632309	0.4880698	0	1	0.6080368	25.07308
88043	cg0438739(	74728348	74728348	0.1828835	0	1	0.2950455	29.6224
88146	cg0439229:	29727438	31669319	0.7320919	0	1	0.8781807	65.85463
88278	cg0439845	31692348	53722489	0.7833999	0	1	0.6737272	-29.50334
88436	cg0440678	23738383	23738383	0.6243641	0	1	0.8088194	72.4782
88649	cg0441611	47656320	47656320	0.2018476	0	1	0.3277087	33.77929
88718	cg0442086:	39624466	39624466	0.2734469	0	1	0.3877408	25.14479
89016	cg0443699	50606366	10676470	0.3386595	0	1	0.4604085	25.86429
89200	cg0444736:	37705438	37705438	0.6325727	0	1	0.756167	33.00124
89288	cg0445312	70784496	64778315	0.6088598	0	1	0.4890315	-25.03955
89356	cg0445649:	42668379	42668379	0.8928843	0	1	0.4037198	-371.3336
89403	cg0445892	46692322	31679409	0.8265943	0	1	0.9320531	57.77433
89452	cg0446114:	74779469	74779469	0.6207346	0	1	0.7268503	24.24968
89515	cg0446452:	17674305	17674305	0.5535296	0	1	0.8001494	115.0121
89565	cg0446763!	63703470	63703470	0.540747	0	1	0.4091279	-28.682
89586	cg0446874	24733338	24733338	0.3462595	0	1	0.4696049	26.28212
89608	cg0447005	46713489	71707481	0.7299043	0	1	0.8469911	41.09357
89664	cg0447340:	37756496	37756496	0.5121402	0	1	0.6411626	29.09546
89669	cg0447365	34713510	34713510	0.1140271	0	1	0.2391589	44.42192
89692	cg0447498!	21737403	67710393	0.5184531	0	1	0.41803	-18.23606
89755	cg0447818(	67706389	67706389	0.5697587	0	1	0.4512109	-24.11266
89805	cg0448107:	24728365	73620326	0.4286607	0	1	0.2560326	-51.26035
89823	cg0448192:	52700462	52700462	0.5679423	0	1	0.4071978	-40.87634
89870	cg0448398!	51717475	51717475	0.2125449	0	1	0.3146936	23.71558
89922	cg0448683!	61780346	61780346	0.5338998	0	1	0.8762054	374.3439
90135	cg0449761	64633508	64633508	0.6220059	0	1	0.8345391	98.43392
90140	cg0449782(	17807329	17807329	0.2809226	0	1	0.0578472	-133.2784
90187	cg0449970	50767325	50767325	0.6500983	0	1	0.7505444	23.52645
90205	cg0450090!	59763439	59763439	0.5584885	0	1	0.6600826	20.20086
90294	cg0450611	60742376	60742376	0.2334549	0	1	0.3364277	22.91662
90336	cg0450846:	12601343	12601343	0.4793909	0	1	0.3339598	-35.03519
90341	cg0450860(	60779380	60779380	0.8388102	0	1	0.5962427	-123.5478
90349	cg0450887(	13685312	13685312	0.4667192	0	1	0.6148336	35.98759
90363	cg0450954:	20802351	20802351	0.1857829	0	1	0.2911039	26.65218

90522	cg0451818(	24810402	24810402	0.8088288	0	1	0.7077608	-28.30766
90560	cg0452025	25723405	25723405	0.7378203	0	1	0.8468866	36.93364
90703	cg0452701{	59694500	61646402	0.5554521	0	1	0.4468546	-20.77489
90946	cg0454088:	55660423	55660423	0.2639273	0	1	0.3733285	23.80887
91043	cg0454641:	27692361	48797310	0.5710542	0	1	0.4360095	-30.08548
91070	cg0454767:	56639317	60731417	0.4390171	0	1	0.557623	24.05703
91146	cg0455150(	35639474	35639474	0.433055	0	1	0.5443085	21.58016
91256	cg0455655(	30700461	30700461	0.2022389	0	1	0.380586	59.66002
91316	cg0455950{	67748477	67748477	0.3409329	0	1	0.5417819	62.28558
91432	cg0456520	26796328	26796328	0.4570683	0	1	0.5737395	23.51233
91438	cg0456533'	16658336	16658336	0.5364695	0	1	0.3822196	-37.99432
91457	cg0456647:	27702400	27702400	0.5180882	0	1	0.7980468	143.3134
91539	cg0457020{	20621488	20621488	0.8944752	0	1	0.7818292	-49.31246
91581	cg0457184'	47714450	67730454	0.156132	0	1	0.0277391	-73.09524
91605	cg0457331(	43739323	43739323	0.2651044	0	1	0.3999968	33.3503
91686	cg0457660'	51641403	51641403	0.6475827	0	1	0.7837798	41.45161
91706	cg0457745	72658302	72658302	0.3980019	0	1	0.5303466	28.98168
91750	cg0457939{	69669504	19709324	0.2448193	0	1	0.1360604	-32.92365
91767	cg0458034	25651423	25651423	0.7747241	0	1	0.5982918	-61.5034
91776	cg0458101{	13632488	13632488	0.4084252	0	1	0.278865	-30.57981
91890	cg0458722(	28773426	49617473	0.2037384	0	1	0.3121404	26.57521
92139	cg0460079:	12768416	12768416		0	1	0.9071345	4.34E-09
92326	cg0460997	51746418	51746418	0.6252135	0	1	0.8340586	95.56445
92345	cg0461074:	73737474	73737474	0.4540644	0	1	0.6305962	49.5588
92346	cg0461089'	33741308	33741308	0.6524378	0	1	0.7645305	28.92187
92397	cg0461345:	46669321	46669321	0.6190464	0	1	0.8133456	79.89603
92399	cg0461373	53697417	53697417	0.3543251	0	1	0.8290947	374.3439
92412	cg0461433'	58717451	58717451	0.6335045	0	1	0.7487967	29.03694
92509	cg0461988:	26668420	26668420	0.1740536	0	1	0.2985101	35.74258
92645	cg0462874	36639383	36639383	0.848596	0	1	0.6403324	-99.75523
92646	cg0462874:	62642490	62642490	0.6731626	0	1	0.7990417	38.36358
92718	cg0463367(	32794308	32794308	0.3615389	0	1	0.5337963	46.57854
92787	cg0463772'	26709488	26709488	0.1640144	0	1	0.3252766	55.54493
92803	cg0463871(	51654333	51654333	0.3945563	0	1	0.512054	23.70065
92901	cg0464507(	31737440	31737440	0.3229088	0	1	0.5149415	57.82364
92903	cg0464515(	66707449	64668343	0.7070335	0	1	0.5002807	-71.11276
92919	cg0464591	26762316	26762316	0.3080914	0	1	0.2018455	-25.88061
92931	cg0464667	17674322	17674322	0.292868	0	1	0.4228835	30.19388
92938	cg0464698'	38752364	20800510	0.7331859	0	1	0.8927659	80.39609
92953	cg0464791{	34703440	34703440	0.67722	0	1	0.5599416	-26.0109
92978	cg0464958'	28701345	28701345	0.4253874	0	1	0.570974	34.28618
92995	cg0465067(	19801302	12656422	0.1992815	0	1	0.3145924	29.60254
93062	cg0465429'	26694475	26694475	0.4653251	0	1	0.638586	48.20424

93090	cg04656015	48755337	48755337	0.3484223	0	1	0.459751	22.16882
93122	cg04657470	40674393	71674460	0.0696852	0	1	0.2621754	102.2177
93433	cg04674421	29671443	29671443	0.5753838	0	1	0.6847888	23.54
93551	cg04680738	32758383	32758383	0.4343387	0	1	0.5651982	28.46251
93600	cg04683035	38634378	38634378	0.8793342	0	1	0.7661745	-45.63044
93611	cg04683516	51686418	51686418	0.4477043	0	1	0.635264	55.47098
93664	cg04686117	58738369	58738369	0.8022925	0	1	0.9189939	60.41079
93680	cg04687040	70733410	70733410	0.6461043	0	1	0.4627043	-53.68753
93722	cg04689178	65787344	65787344	0.7454386	0	1	0.8529456	37.14716
93729	cg04689606	53746496	74787350	0.4185177	0	1	0.5965209	49.48533
93731	cg04689676	52679475	38638386	0.1649455	0	1	0.0548964	-49.55379
93739	cg04690342	22674468	22674468	0.5552648	0	1	0.6722671	25.72938
93788	cg04693014	37737489	25700413	0.227966	0	1	0.0575231	-90.75703
93841	cg04696840	22623365	22623365	0.1740395	0	1	0.309071	40.65081
94018	cg04706655	49736325	23731389	0.4156328	0	1	0.3143144	-19.66442
94167	cg04716530	32649445	27671323	0.3973107	0	1	0.2959179	-20.13141
94188	cg04717802	70612470	43628375	0.2326547	0	1	0.0729862	-76.64477
94261	cg04721775	71733492	40773328	0.8424913	0	1	0.9523476	71.79822
94285	cg04722610	69631444	69631444	0.4922163	0	1	0.6045848	22.46358
94303	cg04723343	56759391	73789489	0.5048302	0	1	0.6188418	23.25188
94335	cg04724556	51798439	51798439	0.4446238	0	1	0.6607531	73.38917
94395	cg04727458	15647330	15647330	0.720044	0	1	0.5730056	-40.42879
94590	cg04738028	50687365	50687365	0.8588581	0	1	0.960593	70.16787
94659	cg04741094	34664418	34664418	0.5717647	0	1	0.719034	40.45842
94673	cg04741728	29673307	55646402	0.6775353	0	1	0.8159602	47.05571
94694	cg04742556	38787419	38787419	0.6320981	0	1	0.5198475	-22.94335
94806	cg04748098	67770356	59728417	0.9066746	0	1	0.6913075	-131.567
94851	cg04750517	12704357	12704357	0.8077068	0	1	0.9477659	94.10933
94933	cg04755386	67777468	67777468	0.9003145	0	1	0.7961362	-45.69021
95033	cg04762129	59633472	59633472	0.3854823	0	1	0.5119354	26.883
95038	cg04762351	33726399	33726399	0.6361988	0	1	0.7805021	45.03209
95067	cg04764476	23729413	48630353	0.4162549	0	1	0.2757723	-35.1219
95140	cg04768488	11726510	11726510	0.6951048	0	1	0.8104118	34.80332
95294	cg04775631	63713495	63713495	0.5146707	0	1	0.6246902	22.05631
95333	cg04777551	51795368	51795368	0.5281041	0	1	0.7544398	90.09959
95502	cg04785902	13637343	13637343	0.1369698	0	1	0.2434538	31.77579
95509	cg04786142	21802497	69752328	0.685837	0	1	0.7944445	30.0842
95542	cg04787784	17610381	17610381	0.3385318	0	1	0.7144521	374.3439
95580	cg04789475	59774428	65809342	0.5767478	0	1	0.4752295	-18.75664
95643	cg04791718	43680426	43680426	0.7869757	0	1	0.6370361	-48.75393
95768	cg04798314	22669441	22669441	0.8849808	0	1	0.7080871	-89.90721
95787	cg04799210	33704489	58709466	0.8347	0	1	0.7168572	-39.41828
95837	cg04801779	70767489	28790376	0.5058546	0	1	0.7215377	78.30984

95988	cg04810745	71757363	71757363	0.5775908	0	1	0.7212179	38.97638
96053	cg04814784	23715415	23715415	0.4383512	0	1	0.0785488	-267.1822
96076	cg04816171	52656395	52656395	0.4023894	0	1	0.2931713	-22.73388
96107	cg04818594	10772349	10772349	0.5747904	0	1	0.6790394	21.6139
96126	cg04819497	65742498	50659359	0.4027613	0	1	0.7205006	374.3439
96195	cg04823299	27605338	27605338	0.4611258	0	1	0.5923807	28.883
96214	cg04824469	60670408	60670408	0.6865287	0	1	0.8064528	36.46878
96218	cg04824555	35717372	35717372	0.327229	0	1	0.4356849	21.62998
96330	cg04830958	62753323	62753323	0.8403749	0	1	0.6235906	-103.4817
96358	cg04832245	74756468	74756468	0.5064057	0	1	0.7081137	68.14713
96433	cg04835652	11793330	11793330	0.3976585	0	1	0.5151639	23.68686
96460	cg04836851	42810329	42810329	0.6470757	0	1	0.4346657	-70.34494
96534	cg04840163	21717353	40702418	0.1753526	0	1	0.3135152	42.00229
96593	cg04843066	14661409	53617378	0.5043691	0	1	0.3815333	-25.6293
96604	cg04843555	11792390	11792390	0.2825597	0	1	0.4347444	39.83435
96605	cg04843568	20664453	20664453	0.3646265	0	1	0.5230826	40.04493
96614	cg04843821	65737502	65737502	0.9379416	0	1	0.7652332	-114.9746
96621	cg04844038	71684466	71684466	0.5399772	0	1	0.6449615	20.8878
96767	cg04850148	49780437	49780437	0.6041432	0	1	0.8571135	139.6336
96771	cg04850286	40677367	40677367	0.2525081	0	1	0.3815579	31.66004
96816	cg04852399	66716355	66716355	0.8355354	0	1	0.7149929	-40.93212
96972	cg04860432	47788359	47788359	0.429716	0	1	0.328675	-19.28987
97042	cg04864199	64737453	64737453	0.5454916	0	1	0.3360404	-67.53727
97278	cg04875706	64738345	64738345	0.0715336	0	1	0.4227591	374.3439
97326	cg04878159	11640487	11640487	0.4989346	0	1	0.6241145	27.23838
97370	cg04880091	72782399	30807398	0.6660654	0	1	0.4799034	-56.14834
97483	cg04885068	37672455	19804385	0.67229	0	1	0.5310305	-35.08301
97489	cg04885343	33675510	33675510	0.3053872	0	1	0.419879	24.15598
97606	cg04890607	31650394	33631300	0.4653584	0	1	0.6387139	48.25584
97621	cg04891577	71749414	71749414	0.2205915	0	1	0.4492476	88.80526
97650	cg04892643	44686303	44686303	0.4879772	0	1	0.6434843	40.033
97656	cg04892766	32609356	32609356	0.4339045	0	1	0.5928693	40.29867
97796	cg04900186	51623431	51623431	0.8322492	0	1	0.6210119	-96.78413
97893	cg04904561	46702384	28655481	0.4063672	0	1	0.2736108	-32.07234
97957	cg04907849	38628462	38628462	0.7271008	0	1	0.8474522	42.91127
97960	cg04908077	59694326	59694326	0.4723703	0	1	0.5862384	22.70812
97974	cg04908625	39653503	65636462	0.3693899	0	1	0.2628225	-22.88251
98086	cg04914221	39603336	39603336	0.6072674	0	1	0.71249	23.24003
98338	cg04926385	55636431	55636431	0.2859139	0	1	0.4363066	38.87749
98414	cg04930693	57798484	57798484	0.2847239	0	1	0.4145908	30.45413
98488	cg04935109	52753423	52753423	0.0930709	0	1	0.2219644	50.88702
98681	cg04944853	11722332	11722332	0.3309492	0	1	0.4531299	26.18073
98744	cg04947555	58797382	58797382	0.4225937	0	1	0.5483419	26.52357

98747	cg04947680	52674493	40797436	0.5825382	0	1	0.4345706	-35.38099
98869	cg04955116	55807479	32768451		0	1		-4.34E-09
98918	cg04957903	23701353	23701353	0.5212257	0	1	0.6325655	22.65326
98990	cg04961665	43701398	43701398	0.5345632	0	1	0.6691079	32.21039
99027	cg04963697	39699304	39699304	0.2143389	0	1	0.324733	26.65146
99123	cg04969808	31745472	31745472	0.0540754	0	1	0.2224621	90.77164
99133	cg04970287	42628435	42628435	0.7971191	0	1	0.9026398	47.20562
99446	cg04986324	10608343	10608343	0.3441105	0	1	0.4657015	25.7033
99536	cg04990378	57701483	57701483	0.678371	0	1	0.512132	-46.65612
99593	cg04993279	49795447	49795447	0.365009	0	1	0.4822766	23.89286
99601	cg04993605	40755441	40755441	0.0803998	0	1	0.2372933	72.16726
99627	cg04995311	61765307	60715439	0.8425586	0	1	0.7337939	-36.09475
99678	cg04998327	66624499	66624499	0.6603729	0	1	0.8098125	51.96743
99754	cg05001932	58718381	58718381	0.4277182	0	1	0.3043726	-27.30698
99816	cg05005073	68653306	68653306	0.5838602	0	1	0.7603753	59.51945
99828	cg05005382	43602318	35604442	0.569892	0	1	0.7044037	33.94742
99848	cg05006231	29722314	32626329	0.6730338	0	1	0.5204675	-39.99636
99900	cg05008825	29686412	29686412	0.5485117	0	1	0.6734822	28.68631
99937	cg05010648	62808422	62808422	0.465718	0	1	0.5896247	26.17262
100010	cg05014721	35803400	11650317	0.6523047	0	1	0.4930613	-42.06523
100161	cg05023192	73670346	73670346	0.4112937	0	1	0.694602	127.0809
100330	cg05032848	40772427	40772427	0.6947245	0	1	0.815995	38.35678
100339	cg05033239	56793502	56793502	0.365779	0	1	0.478935	22.51898
100346	cg05033369	13689323	13689323	0.550189	0	1	0.6507845	19.656
100380	cg05035061	31708457	31708457	0.5082215	0	1	0.6381546	29.35127
100497	cg05040348	17788344	17788344	0.2593885	0	1	0.4196127	44.88744
100503	cg05040501	56744475	56744475	0.3861738	0	1	0.2827186	-21.16441
100562	cg05043716	44750351	44750351	0.5057768	0	1	0.6094333	19.78666
100630	cg05046589	10713425	10713425	0.5780141	0	1	0.7077041	32.16635
100634	cg05046722	73735407	73735407	0.4162827	0	1	0.540558	25.98778
100709	cg05050053	57704510	69704315	0.3796997	0	1	0.5110887	28.74347
100730	cg05051262	27733467	27733467	0.3017143	0	1	0.1775656	-35.24593
100793	cg05054683	47799466	29804509	0.9498188	0	1	0.8389568	-71.09911
100800	cg05055001	53600355	53600355	0.6000075	0	1	0.7422553	40.04251
100841	cg05057352	48676426	48676426	0.2220129	0	1	0.3247259	23.3996
100955	cg05062489	55672506	55672506	0.3688583	0	1	0.5140426	34.27551
101041	cg05065846	36760325	36760325	0.4938982	0	1	0.5953907	18.9415
101115	cg05070273	74735361	74735361	0.3201436	0	1	0.4232598	20.09376
101196	cg05075308	53659444	53659444	0.5777795	0	1	0.6984356	28.15722
101243	cg05079049	37750481	20718489	0.6168587	0	1	0.4759458	-33.07233
101375	cg05086444	40631330	40631330	0.7189029	0	1	0.607112	-25.89069
101395	cg05087623	65796378	65796378	0.8263854	0	1	0.668494	-59.89026
101407	cg05088151	70699479	11603418	0.7153841	0	1	0.5894368	-31.11082

101481	cg05092371	44765425	44765425	0.7974231	0	1	0.6836408	-32.6322
101541	cg0509641[illegible]	16639308	16639308	0.9349862	0	1	0.7359739	-135.7564
101549	cg0509663[illegible]	23740403	23740403	0.1424627	0	1	0.2438296	28.89732
101623	cg0510006[illegible]	74770356	74770356	0.6280153	0	1	0.525404	-19.78588
101763	cg0510846[illegible]	49607442	49607442	0.1242305	0	1	0.2280646	32.14932
101769	cg0510956[illegible]	63659395	63659395	0.3570979	0	1	0.4842598	27.46822
101800	cg0511164[illegible]	35658390	35658390	0.4613468	0	1	0.6400992	51.02819
101804	cg0511189[illegible]	60744436	60744436	0.3427811	0	1	0.4856773	33.78705
102042	cg0512651[illegible]	31756325	31756325	0.6392564	0	1	0.8564253	109.404
102145	cg0513148[illegible]	22610307	22610307	0.2122725	0	1	0.3359213	32.0364
102152	cg0513169[illegible]	26788413	26788413	0.5864746	0	1	0.4794215	-20.53293
102209	cg0513473[illegible]	21667314	21667314	0.3690366	0	1	0.1684136	-78.5538
102301	cg0513889[illegible]	23627462	23627462	0.6175989	0	1	0.7404417	31.48499
102306	cg0513902[illegible]	44804476	44804476	0.7966906	0	1	0.6715418	-37.73316
102430	cg0514481[illegible]	43802320	43802320	0.1662558	0	1	0.26674	26.28941
102619	cg0515504[illegible]	74711312	74711312	0.2793415	0	1	0.4009028	27.5347
102680	cg0515762[illegible]	67795434	67795434	0.1798367	0	1	0.2816136	25.71954
102740	cg0516056[illegible]	37643374	37643374	0.3185539	0	1	0.4231217	20.5801
102758	cg0516177[illegible]	17630455	17630455	0.737543	0	1	0.4721424	-117.2348
102823	cg0516454[illegible]	27745334	27745334	0.5469084	0	1	0.6786997	31.53243
102832	cg0516493[illegible]	60782338	35771437	0.5457045	0	1	0.4388943	-20.1815
102840	cg0516525[illegible]	11635354	11635354	0.7130585	0	1	0.5972485	-27.07211
103011	cg0517388[illegible]	46787473	46787473	0.638967	0	1	0.9167242	374.3439
103041	cg0517580[illegible]	48646417	48646417	0.6517056	0	1	0.7920858	44.55603
103050	cg05176551	36699356	36699356	0.4477629	0	1	0.5995412	37.2386
103098	cg0517949[illegible]	30707435	30707435	0.7317395	0	1	0.8649902	54.03433
103155	cg05181941	23806365	74751351	0.7050812	0	1	0.5955749	-24.4035
103157	cg0518221[illegible]	61755450	61755450	0.3394938	0	1	0.4892493	36.71579
103239	cg0518715[illegible]	30652418	30652418	0.3233918	0	1	0.4551593	29.88584
103357	cg0519442[illegible]	29773460	29773460	0.3473811	0	1	0.5109004	42.65977
103365	cg0519463[illegible]	50704485	50704485	0.6921065	0	1	0.8309203	49.87082
103379	cg0519531[illegible]	64698331	64698331	0.4474664	0	1	0.5675395	24.61722
103410	cg0519706[illegible]	15746499	57747438	0.0348209	0	1	0.2016795	99.42105
103421	cg0519766[illegible]	30610409	30610409	0.3220524	0	1	0.4270897	20.65262
103615	cg0520860[illegible]	35706389	35706389	0.0596871	0	1	0.1968997	65.93365
103663	cg0521068[illegible]	50646447	50646447	0.2655058	0	1	0.3676265	21.30244
103708	cg0521289[illegible]	20718326	20718326	0.5288414	0	1	0.7460648	82.53926
103889	cg05223441	19777426	19777426	0.4694902	0	1	0.5736855	19.53399
103952	cg05226061	13644381	49734466		0	1		-4.34E-09
104113	cg0523294[illegible]	55732352	55732352	0.4398991	0	1	0.9194194	374.3439
104151	cg0523456[illegible]	22664389	63716334	0.8161591	0	1	0.6756575	-48.19193
104200	cg0523726[illegible]	38699421	38699421	0.3128603	0	1	0.462261	37.34072
104308	cg0524182[illegible]	69780466	22600367	0.1855408	0	1	0.3234845	40.75354

104350	cg05244013	60716387	60716387	0.1485159	0	1	0.2583863	31.96555
104354	cg05244234	63742314	55731365	0.0129557	0	1	0.1196668	63.85846
104358	cg05244682	61615347	61615347	0.3466013	0	1	0.4968332	36.76001
104376	cg05245515	46695455	46695455	0.2437699	0	1	0.3877216	38.46774
104421	cg05248234	35685425	33667479	0.214865	0	1	0.0292448	-118.295
104462	cg05250932	16606320	24728332	0.506601	0	1	0.607647	18.97099
104600	cg05258009	22771355	74616339	0.8053862	0	1	0.9483696	97.00725
104774	cg05265884	44763466	18750386	0.7296329	0	1	0.6217679	-25.02807
104798	cg05266786	17733366	17733366	0.2153139	0	1	0.3290685	27.8744
104806	cg05267394	24754470	74622419	0.3819155	0	1	0.0578278	-235.4707
104839	cg05269323	56681340	56681340	0.4289831	0	1	0.552107	25.59879
104876	cg05270750	60810486	60810486	0.2393531	0	1	0.1250832	-37.02207
104922	cg05274316	30668497	30668497	0.7696814	0	1	0.8813788	45.26786
104955	cg05276155	55614329	55614329	0.299427	0	1	0.4163534	25.17174
104965	cg05276469	56713435	56713435	0.451605	0	1	0.3138366	-32.499
104968	cg05276972	48800415	48800415	0.0993151	0	1	0.3808953	374.3439
104981	cg05277504	14769334	22620442	0.3028125	0	1	0.4883712	55.14283
105011	cg05279330	65802421	65802421	0.49545	0	1	0.6895855	62.12418
105020	cg05279738	10734455	10734455	0.5888029	0	1	0.7373613	42.54586
105034	cg05280336	46730385	46730385	0.5129238	0	1	0.6206507	21.24352
105071	cg05282260	54763316	74643422	0.681569	0	1	0.7829333	26.10168
105107	cg05284727	26726429	26726429	0.2407449	0	1	0.3755079	34.7016
105150	cg05287372	26636348	26636348	0.3771577	0	1	0.4958939	24.25367
105165	cg05288172	43740492	43740492	0.4227976	0	1	0.5622219	31.73971
105421	cg05301470	34644326	16671467	0.2497139	0	1	0.1457358	-29.67962
105438	cg05302100	63795474	63795474	0.2798277	0	1	0.398788	26.56691
105532	cg05306029	57645327	57645327	0.1541428	0	1	0.2574526	28.54026
105580	cg05308244	24788496	24788496	0.6226157	0	1	0.8230758	86.49803
105620	cg05309989	43799373	43799373	0.2310008	0	1	0.3730786	38.54743
105639	cg05310990	61756328	12600421	0.3294418	0	1	0.2154231	-27.96945
105686	cg05313910	66616450	66616450	0.4534197	0	1	0.5747131	25.09563
105749	cg05317207	55733446	55733446	0.5500979	0	1	0.42184	-27.43879
105768	cg05318275	29749314	29749314	0.3092286	0	1	0.4388572	29.48579
105805	cg05321225	18708411	26771434	0.4344945	0	1	0.31341	-26.22954
105920	cg05328057	12687466	40657444	0.8779778	0	1	0.6877397	-97.05561
105984	cg05331763	73723426	73723426	0.2281518	0	1	0.7962071	374.3439
106054	cg05336056	61607478	61607478	0.7761353	0	1	0.6680248	-28.24254
106096	cg05337761	29669478	52623471	0.5235026	0	1	0.1377316	-274.2978
106121	cg05338731	63790402	63790402	0.2548933	0	1	0.0844595	-80.12089
106205	cg05342469	33611312	33611312	0.5039571	0	1	0.6277536	26.82456
106238	cg05343811	37807421	22788356	0.160053	0	1	0.2792693	34.87949
106247	cg05344495	41766314	59774305	0.4873297	0	1	0.2773363	-70.70065
106257	cg05345154	71714446	74795345	0.236239	0	1	0.0254148	-142.9293

106348	cg0534885	47681322	47681322	0.4323739	0	1	0.5909655	40.1036
106357	cg0534901	48603467	48603467	0.1039658	0	1	0.2120162	37.23025
106462	cg0535419	43785387	43785387	0.9077501	0	1	0.802061	-48.87491
106515	cg0535703	73681375	73681375	0.3295087	0	1	0.4705399	33.3513
106584	cg0536017	13644444	57798408		0	1	0.0051239	4.34E-09
106632	cg0536231	51624441	51624441	0.6272603	0	1	0.5003451	-27.94407
106665	cg0536395	48629338	48629338	0.3973395	0	1	0.5201062	25.49823
106683	cg0536456	35650419	35650419	0.4223966	0	1	0.3155487	-21.3696
106697	cg0536503	50628338	50656399	0.8347162	0	1	0.6577964	-73.65076
106722	cg0536605	36747348	36747348	0.7376692	0	1	0.8421532	33.92982
106728	cg0536616	46809478	46809478	0.6934081	0	1	0.8608121	74.63701
106815	cg0537063	62802309	14641348	0.5313552	0	1	0.4137804	-23.66078
106860	cg0537244	68766487	12685351	0.1002916	0	1	0.2567034	65.89664
106919	cg0537480	18625342	18625342	0.2476643	0	1	0.3639035	26.97975
106983	cg0537712	43729306	43729306	0.5915694	0	1	0.6917572	20.72746
107014	cg0537869	58773343	58773343	0.2368732	0	1	0.0574713	-97.63934
107112	cg0538361	34752490	34752490	0.5457646	0	1	0.6900917	37.28179
107172	cg0538571	24681459	20709500	0.8345914	0	1	0.684692	-56.90888
107203	cg0538739	55706492	67765452		0	1	0.0671452	4.34E-09
107306	cg0539244	18694399	53806343	0.7982588	0	1	0.5781485	-94.29161
107320	cg0539298	48634406	48634406	0.4571436	0	1	0.3499008	-20.84296
107325	cg0539308	60695447	60695447	0.5405786	0	1	0.6554396	24.42895
107479	cg0540073	22781377	22781377	0.2039884	0	1	0.3044145	23.59196
107490	cg0540157	16750363	16750363	0.4683267	0	1	0.7465642	129.7962
107580	cg0540720	36709476	36709476	0.1984926	0	1	0.5726491	374.3439
107605	cg0540867	25687314	25687314	0.1127184	0	1	0.2748564	66.28784
107649	cg0541159	29600456	29600456	0.2300908	0	1	0.3376186	24.69485
107660	cg0541213	27777372	27777372	0.4157422	0	1	0.2676185	-38.82939
107680	cg0541295	70659300	70659300	0.584977	0	1	0.4276479	-39.46237
107683	cg0541306	47649504	47649504	0.3041865	0	1	0.4214372	25.14221
107744	cg0541633	23758313	23758313	0.6182364	0	1	0.4512805	-44.50652
107803	cg0541962	15679354	15679354	0.0262978	0	1	0.1381617	61.35777
107889	cg0542436	62680335	62680335	0.3266785	0	1	0.1747545	-48.97697
107937	cg0542845	45790381	45790381	0.7479971	0	1	0.8818128	58.99155
107984	cg0543203	65788499	65788499	0.7528928	0	1	0.8570474	36.12513
108033	cg0543539	54763400	54763400	0.7979914	0	1	0.9016854	45.77775
108088	cg0544082	31727406	31727406	0.391471	0	1	0.6518676	104.5473
108220	cg0545033	18797427	18797427	0.646566	0	1	0.7818211	40.76951
108267	cg0545300	28709352	48752321	0.5029329	0	1	0.7247487	82.74958
108274	cg0545335	26780499	39755368	0.7639693	0	1	0.6635687	-24.35099
108307	cg0545537	25618490	25618490	0.6829837	0	1	0.8232775	49.29497
108344	cg0545773	20682433	46804394	0.3725333	0	1	0.185414	-67.12616
108436	cg0546358	74757478	42741510	0.5362997	0	1	0.3064851	-81.77359

108535	cg05469819	27641340	27641340	0.4128228	0	1	0.5738739	41.04808
108563	cg05471169	66715450	58792400	0.3406461	0	1	0.487611	35.51186
108573	cg05471495	35724480	35724480	0.4849016	0	1	0.656144	47.94038
108593	cg05472468	38742324	51726304	0.1155135	0	1	0.2700105	60.89796
108647	cg05475524	11770420	54677365	0.2371243	0	1	0.1023666	-52.179
108689	cg05477582	72609395	29778323	0.5741422	0	1	0.7746835	76.18436
108727	cg05479627	41672359	41672359	0.3436664	0	1	0.4601519	23.9552
108759	cg05481961	20808492	20808492	0.2042373	0	1	0.3212866	29.9351
108789	cg05483388	64758315	64758315	0.6620594	0	1	0.5619633	-19.78614
108800	cg05483731	33804472	66666305	0.2176494	0	1	0.3201548	23.55951
108823	cg05484949	74646447	32655314	0.2473982	0	1	0.41054	47.15711
108849	cg05486191	13749432	13749432	0.1580932	0	1	0.2709526	32.25068
109165	cg05502360	29714300	29714300	0.682013	0	1	0.233032	-361.9467
109305	cg05509228	16658506	16658506	0.6629541	0	1	0.901006	148.0846
109384	cg05513157	59733359	59733359	0.8375681	0	1	0.957857	84.31824
109406	cg05514299	19661387	19661387	0.5952669	0	1	0.7192737	30.57014
109425	cg05515244	48649474	48649474	0.6117806	0	1	0.8121421	83.70258
109584	cg05524038	13743318	13743318	0.537143	0	1	0.6729763	32.89118
109616	cg05526364	47799399	47799399	0.5740361	0	1	0.6785442	21.6823
109625	cg05526809	44650361	44650361	0.6346212	0	1	0.7932999	54.36098
109667	cg05528899	54661458	54661458	0.7876841	0	1	0.9273376	81.42205
109728	cg05531409	13799421	13799421	0.4407108	0	1	0.8297278	374.3439
109982	cg05548393	70714466	70714466	0.7583455	0	1	0.6493049	-27.25546
109984	cg05548425	50692329	50692329	0.4133222	0	1	0.5542628	32.31908
110212	cg05563515	33659451	37700348	0.1196907	0	1	0.27063	57.83602
110423	cg05574272	67759423	31698311	0.4300393	0	1	0.2673617	-45.54379
110446	cg05575505	44635308	44635308	0.378571	0	1	0.6520595	115.4287
110448	cg05575614	64745329	21727313	0.4930258	0	1	0.385443	-20.54852
110518	cg05579598	25738399	25738399	0.4232076	0	1	0.6919174	114.1846
110540	cg05580809	64802438	64802438	0.7060372	0	1	0.5145601	-61.96921
110554	cg05581878	30628482	30628482	0.5865272	0	1	0.7555486	54.77495
110582	cg05583636	46659302	46659302	0.5396081	0	1	0.6600834	26.54872
110691	cg05589784	11736345	69604436	0.3565287	0	1	0.2513405	-22.87624
110701	cg05590257	30699439	69690416	0.1991113	0	1	0.0752229	-52.38939
110846	cg05597181	32754347	62789334	0.3236563	0	1	0.4240088	19.1771
110929	cg05601623	24741423	24741423	0.1730212	0	1	0.0617676	-48.30635
111009	cg05606115	19715310	19715310	0.8491317	0	1	0.6631688	-83.77103
111015	cg05606455	19706326	12671388	0.6222477	0	1	0.7661796	43.17974
111122	cg05612744	18642335	18642335	0.2240393	0	1	0.1095159	-39.62247
111293	cg05621349	31682310	67795408	0.0299439	0	1	0.3342476	374.3439
111313	cg05622577	27694449	27694449	0.5266725	0	1	0.6405618	23.69116
111431	cg05628506	41697390	41697390	0.552953	0	1	0.6718998	26.4113
111460	cg05630111	44650460	45650392	0.7058975	0	1	0.5722696	-33.66928

111464	cg05630272	63604429	63604429	0.5248813	0	1	0.4198509	-19.61521
111523	cg05634637	71771403	71771403	0.3858883	0	1	0.4937499	20.6321
111551	cg05636175	29763331	29763331	0.5205776	0	1	0.4108285	-21.1011
111601	cg05638648	29643404	46795366	0.711623	0	1	0.8979532	102.8937
111638	cg05640533	28683489	22722453	0.6182386	0	1	0.7199274	22.36289
111791	cg05649077	57772441	57772441	0.287241	0	1	0.5025141	73.32173
111875	cg05653481	59616435	59616435	0.7306267	0	1	0.8545583	46.25751
111945	cg05656210	17758490	64650421	0.5079496	0	1	0.1105668	-303.9002
111982	cg05657656	20687501	20687501	0.326219	0	1	0.480153	38.87645
112146	cg05666820	51636353	49620410	0.4421226	0	1	0.3026008	-33.58598
112413	cg05682128	54772377	54772377	0.1185407	0	1	0.2443547	43.97498
112434	cg05683630	11611477	63673338	0.4878224	0	1	0.3073408	-52.26245
112523	cg05689413	43736462	43736462	0.3576692	0	1	0.47909	25.41705
112536	cg05690644	61628317	41624466	0.1715239	0	1	0.2832747	30.44867
112619	cg05695699	55753504	70682427	0.5021583	0	1	0.6164778	23.30945
112621	cg05695876	51767359	51767359	0.4154906	0	1	0.5167193	18.47832
112718	cg05701478	15736509	15736509	0.6568927	0	1	0.7743914	31.98551
112767	cg05704942	54720467	54720467	0.185864	0	1	0.0465912	-72.51063
112779	cg05705335	57763359	24659427	0.2366095	0	1	0.3456203	24.89438
112866	cg05710060	58692328	58692328	0.8788147	0	1	0.7635155	-46.7636
112941	cg05713856	24654401	24654401	0.4663995	0	1	0.566416	18.23565
112944	cg05713954	15619486	15619486	0.3501269	0	1	0.2186673	-34.84143
113101	cg05722035	37645504	37645504	0.2198813	0	1	0.3394968	29.87207
113119	cg05723179	19643461	19643461	0.1873858	0	1	0.2971903	28.30267
113139	cg05724777	42650320	17720392	0.5101254	0	1	0.3816257	-27.65788
113200	cg05728166	38761400	38761400	0.413994	0	1	0.7161129	147.5047
113250	cg05730975	57661493	57661493	0.3761342	0	1	0.5984299	75.34387
113293	cg05733463	12626393	12626393	0.2699385	0	1	0.3723854	21.25401
113365	cg05738749	26798357	26798357	0.1012268	0	1	0.2061623	36.09843
113393	cg05740465	26607306	68601390	0.7750757	0	1	0.658468	-31.66861
113445	cg05743713	10754333	21656445	0.3890468	0	1	0.2722188	-26.09555
113549	cg05751055	71632416	71632416	0.3391707	0	1	0.8595735	374.3439
113554	cg05751189	62667342	62667342	0.0522794	0	1	0.1760638	59.44805
113685	cg05758978	15708458	48696497	0.6040829	0	1	0.4884092	-23.5475
113803	cg05766605	57620314	40810434	0.55098	0	1	0.4318137	-24.22164
113822	cg05767411	31782365	34773427	0.226885	0	1	0.3340213	24.72296
113912	cg05772155	60764301	51746329	0.3746723	0	1	0.2421841	-33.66081
113975	cg05775862	21695381	21695381	0.271219	0	1	0.4008043	30.93006
114053	cg05779595	65709488	65709488	0.2603545	0	1	0.3706553	24.26596
114083	cg05781417	29648301	64671432	0.4775724	0	1	0.3490518	-28.09994
114170	cg05785424	14634377	37653502	0.172236	0	1	0.2848649	30.75374
114194	cg05786140	34710350	34710350	0.1947445	0	1	0.3234012	35.60611
114286	cg05790828	57639389	57639389	0.7951227	0	1	0.5415973	-119.3387

114421	cg05797854	34754487	34754487	0.6701692	0	1	0.7781195	28.34156
114456	cg05799169	45648303	49757422	0.5499204	0	1	0.6576573	22.10228
114585	cg05806645	42653354	42653354	0.4551519	0	1	0.3500666	-20.16928
114625	cg05809586	47748455	47748455	0.8472602	0	1	0.9501417	64.76236
114783	cg05819221	19633331	19633331	0.4334054	0	1	0.5357398	18.79981
114810	cg05820861	61761478	42702498	0.9091506	0	1	0.804485	-48.65236
114940	cg05828605	17657409	17657409	0.6546339	0	1	0.7610032	26.40891
114977	cg05831083	60732331	60732331	0.8847328	0	1	0.7813577	-41.21788
115008	cg05833351	43687316	60660364	0.9182571	0	1	0.8153679	-50.52065
115089	cg05837990	67797503	63734392	0.8164073	0	1	0.6399422	-69.06747
115117	cg05839741	31774410	27712404	0.7779002	0	1	0.6355197	-43.76244
115119	cg05839762	60790473	42737448	0.002694	0	1	0.1565977	109.9111
115226	cg05845592	15645506	58733352	0.017141	0	1	0.184776	111.1189
115257	cg05847075	71762411	71762411	0.6589811	0	1	0.7792485	33.63522
115342	cg05852276	24780491	33620417	0.5760545	0	1	0.3090031	-109.7586
115440	cg05857999	35725469	53770372	0.4836798	0	1	0.2960043	-56.62321
115494	cg05860956	46756506	46756506	0.4186194	0	1	0.1258054	-166.1658
115566	cg05865243	60680491	45667398	0.2679316	0	1	0.4022016	32.95407
115607	cg05867245	72691431	72691431	0.8995842	0	1	0.7837218	-52.8988
115619	cg05867884	70766497	70766497	0.4280412	0	1	0.3081467	-25.95575
115639	cg05868531	23655430	16636475	0.6835118	0	1	0.5595915	-28.70655
115813	cg05876883	62609471	62609471	0.3043869	0	1	0.4868974	53.45394
115863	cg05879075	19630488	19630488	0.7896008	0	1	0.9159509	66.05614
116056	cg05888894	36646450	36646450	0.3219005	0	1	0.4264139	20.49172
116088	cg05890457	73789453	73789453	0.4677153	0	1	0.6503761	53.48736
116103	cg05890956	28736421	28736421	0.5037169	0	1	0.6080366	19.96859
116159	cg05893845	47708472	47708472	0.7131306	0	1	0.9195104	131.622
116213	cg05896682	43787408	43787408	0.2639716	0	1	0.1393367	-40.06682
116300	cg05900567	64696429	64696429	0.9414544	0	1	0.7301759	-151.813
116314	cg05901462	61780480	59786389	0.4647294	0	1	0.7522579	139.2928
116362	cg05903720	15673451	15673451	0.5583972	0	1	0.6619192	20.85998
116556	cg05914894	34617449	34617449	0.6734615	0	1	0.7897923	32.95995
116629	cg05918715	47716437	47716437	0.3022402	0	1	0.4667998	44.59313
116686	cg05921762	68642362	68642362	0.862406	0	1	0.6792791	-86.04171
116695	cg05922210	55756442	55756442	0.6976388	0	1	0.5800409	-26.98141
116696	cg05922253	29795415	18626373	0.453424	0	1	0.3276671	-27.54101
116782	cg05925927	19725472	19725472	0.91217	0	1	0.6906285	-140.3073
116861	cg05929272	68636326	68636326	0.1608778	0	1	0.2885714	38.71649
116874	cg05929864	57686485	57686485	0.3920987	0	1	0.5038061	21.80825
116903	cg05931366	61691305	61691305	0.8311029	0	1	0.5685768	-138.0171
116997	cg05935904	19706355	19706355	0.4490286	0	1	0.5492091	18.19528
117004	cg05936219	73743360	65633311	0.5474922	0	1	0.4408823	-20.12477
117046	cg05938207	13669487	13669487	0.4249734	0	1	0.2702187	-41.65883

117095	cg0594045‌2	42729309	54606483	0.3931223	0	1		-4.34E-09
117146	cg0594234‌9	66605322	66605322	0.7857263	0	1	0.9398773	100.6067
117148	cg0594245‌9	33666445	24706418	0.6407966	0	1	0.4336956	-66.82456
117219	cg0594578‌2	11806482	41603461	0.632854	0	1	0.497714	-31.19626
117271	cg0594895‌5	69613476	69613476	0.8054051	0	1	0.7045493	-27.87844
117272	cg0594896‌2	65710411	65710411	0.2952443	0	1	0.4087438	24.11551
117307	cg0595057‌2	57658365	57658365	0.5014794	0	1	0.6376676	31.74394
117372	cg0595324‌3	19760425	19760425	0.5216274	0	1	0.643625	26.59083
117435	cg0595651‌8	14636450	14636450	0.5319307	0	1	0.7870957	118.683
117456	cg0595754‌4	21789302	21635341	0.7442222	0	1	0.8576552	41.11595
117497	cg0595943‌0	73612424	73612424	0.3774399	0	1	0.5440338	43.66822
117639	cg0596643‌1	59724427	59724427	0.8072281	0	1	0.6923323	-34.23193
117729	cg0597110‌2	74626404	74626404	0.8016809	0	1	0.3146167	-371.3336
117747	cg0597191‌1	22669432	35631500	0.0149929	0	1	0.1379134	75.5998
117863	cg0597733‌3	32673456	32673456	0.9372555	0	1	0.8305113	-61.12087
117930	cg0598103‌8	66692405	66692405	0.2678284	0	1	0.3868437	27.07181
118211	cg0599481‌9	30625300	30682303	0.4865621	0	1	0.3506869	-30.84411
118213	cg0599497‌4	24671300	24671300	0.3042638	0	1	0.4320042	28.93303
118309	cg0599936‌4	65737401	65737401	0.1882883	0	1	0.3238976	39.3723
118313	cg0599962‌8	74634432	74634432	0.4736419	0	1	0.5755129	18.851
118335	cg0600087‌8	57671398	57671398	0.3473116	0	1	0.4667682	24.90405
118360	cg0600220‌3	56698398	56698398	0.8446839	0	1	0.6670154	-76.80507
118375	cg0600268‌7	24632477	52784353	0.4168306	0	1	0.2839114	-31.68063
118554	cg0601339‌5	47646471	47646471	0.2565102	0	1	0.3857324	31.5138
118559	cg0601376‌9	64700324	57768426	0.5406662	0	1	0.4124859	-27.40166
118612	cg0601668‌4	35736339	35736339	0.5211477	0	1	0.3416399	-50.59887
118695	cg0602199‌0	49639420	49639420	0.3411598	0	1	0.4593832	24.59217
118725	cg0602335‌1	36691480	36691480	0.8984178	0	1	0.6149057	-190.8725
118731	cg0602379‌1	53738457	53738457	0.2886775	0	1	0.398767	23.16377
118851	cg0603233‌7	48708300	48708300	0.3408251	0	1	0.7485536	374.3439
119076	cg0604494‌5	71687311	71687311	0.4199374	0	1	0.5224575	18.85547
119190	cg0605166‌2	33803347	33803347	0.4040831	0	1	0.2977246	-21.66379
119195	cg0605209‌0	21676383	21676383	0.4444728	0	1	0.5464591	18.71907
119199	cg0605237‌2	47624464	47624464	0.3324273	0	1	0.4337794	19.31586
119343	cg0606108‌1	71649437	71649437	0.720751	0	1	0.6126468	-24.6496
119424	cg0606549‌5	26611336	26611336	0.1056077	0	1	0.2328872	47.22313
119600	cg0607539‌3	49713492	49713492	0.3372018	0	1	0.0765563	-157.1448
119628	cg0607730‌5	66671508	25699377	0.8670314	0	1	0.7581241	-40.50022
119783	cg0608572‌6	47672424	47672424	0.1533693	0	1	0.2609603	30.44476
119880	cg0609081‌8	58734382	66670400	0.7586129	0	1	0.8944813	64.45393
119935	cg0609386‌1	67783467	67783467	0.4766338	0	1	0.344104	-29.6902
120193	cg0610676‌3	29736310	29736310	0.1436122	0	1	0.0365102	-53.77113
120225	cg0610866‌1	20803430	20803430	0.6754048	0	1	0.8211871	51.93651

120272	cg06112171	16684338	16684338	0.45849	0	1	0.6386257	51.68275
120273	cg0611220[illegible]	64656387	64656387	0.7911128	0	1	0.9126001	61.2501
120365	cg0611830[illegible]	40647492	40647492	0.4450401	0	1	0.5527503	20.48543
120406	cg0612031[illegible]	47767466	47767466	0.8246896	0	1	0.6208112	-89.32552
120563	cg0612945[illegible]	58661340	58661340	0.5678368	0	1	0.8211257	126.6145
120578	cg0613036[illegible]	19774377	53699496	0.5416677	0	1	0.3789999	-41.81366
120613	cg0613272[illegible]	67672504	63621429	0.4927728	0	1	0.3784019	-22.78144
120640	cg0613467[illegible]	43631399	43631399	0.5737156	0	1	0.7361615	49.22223
120657	cg0613552[illegible]	33738316	65637472	0.5041897	0	1	0.3912412	-22.21519
120677	cg0613670[illegible]	48627478	48627478	0.2024512	0	1	0.3154695	28.45639
120706	cg0613900[illegible]	24717442	41733314	0.4880476	0	1	0.6613349	49.20662
120772	cg0614331[illegible]	73685375	56706412	0.8626935	0	1	0.7139863	-62.97623
120841	cg0614719[illegible]	51660422	51660422	0.764342	0	1	0.8997394	66.0274
120866	cg0614873[illegible]	73691439	49675416	0.2781022	0	1	0.1456643	-43.05858
120886	cg0615014[illegible]	12697439	55755303	0.1283661	0	1	0.2558017	43.16036
120896	cg0615077[illegible]	24683399	24683399	0.3869818	0	1	0.4944783	20.51018
120932	cg0615285[illegible]	10766356	10766356	0.577328	0	1	0.4722094	-19.84612
121021	cg0615792[illegible]	56734322	28736442	0.8204116	0	1	0.947542	83.12888
121051	cg0615940[illegible]	30625391	28698390	0.3403523	0	1	0.0995841	-128.6469
121253	cg0617331[illegible]	35726343	60720359	0.2796359	0	1	0.4236035	36.40211
121317	cg0617665[illegible]	43701306	43701306	0.3009454	0	1	0.4352599	31.56797
121383	cg0617922[illegible]	58688505	58688505	0.1824941	0	1	0.2980912	31.08175
121588	cg0619073[illegible]	21644359	21644359	0.110196	0	1	0.2135248	33.87246
121647	cg0619359[illegible]	54605425	40778416	0.5994748	0	1	0.3904907	-66.86836
121655	cg0619395[illegible]	66632377	66632377	0.2278544	0	1	0.1260238	-30.99562
121797	cg0620247[illegible]	56672358	50807436	0.7982261	0	1	0.5884879	-86.94759
121805	cg0620280[illegible]	59743377	59743377	0.688089	0	1	0.8175909	42.682
121874	cg0620680[illegible]	20679486	20679486	0.3083452	0	1	0.4379857	29.51802
121963	cg0621155[illegible]	55734452	52660431	0.4164638	0	1	0.5215471	19.63589
122021	cg0621390[illegible]	18623376	18623376	0.3652681	0	1	0.5086122	33.57015
122248	cg0622563[illegible]	29665383	29665383	0.4517908	0	1	0.6579676	67.09174
122312	cg0623066[illegible]	34757465	34757465	0.3651556	0	1	0.2470765	-27.69577
122326	cg0623136[illegible]	54718487	54718487	0.4939315	0	1	0.3787579	-23.04298
122451	cg0623831[illegible]	64747498	64747498	0.5699689	0	1	0.4442704	-26.60947
122503	cg0624120[illegible]	32773363	32773363	0.282364	0	1	0.4426707	43.55225
122573	cg06245711	53646379	53646379	0.2406766	0	1	0.3458404	23.33801
122602	cg0624740[illegible]	48713397	48713397	0.6366484	0	1	0.4851473	-38.03648
122614	cg0624870[illegible]	39711332	39711332	0.3319832	0	1	0.4435087	22.52842
122623	cg0624960[illegible]	59671348	41668405	0.010442	0	1	0.1854611	122.3675
122639	cg0625075[illegible]	20661503	20661503	0.8036682	0	1	0.6947199	-31.16982
122666	cg0625227[illegible]	71777325	74715399	0.4584931	0	1	0.3261132	-30.04717
122709	cg0625500[illegible]	12730365	42684362	0.7658338	0	1	0.6551291	-28.46583
122862	cg0626488[illegible]	13672375	13672375	0.6893077	0	1	0.3600159	-173.0218

122879	cg0626622[illegible]	73642449	73642449	0.8594989	0	1	0.737659	-46.11888
122919	cg0626887[illegible]	60793361	25655400	0.5810696	0	1	0.4752084	-20.10865
122961	cg0627104[illegible]	52771317	52771317	0.0965081	0	1	0.2216742	47.95071
122969	cg0627138[illegible]	16652309	16652309	0.2064118	0	1	0.3499793	41.29748
122974	cg0627169[illegible]	55809408	32768487		0	1		-4.34E-09
123109	cg0628252[illegible]	26626360	44734401	0.3018198	0	1	0.1789693	-34.53072
123152	cg0628557[illegible]	52656335	52656335	0.3274021	0	1	0.4454299	24.81012
123188	cg0628754[illegible]	10792391	10792391	0.6186359	0	1	0.719256	21.96826
123199	cg0628808[illegible]	66766464	66766464	0.3877151	0	1	0.516829	27.83521
123302	cg0629378[illegible]	28697451	28697451	0.3770683	0	1	0.1223469	-131.9776
123389	cg0629795[illegible]	46733479	29713394	0.2159378	0	1	0.1086032	-36.10389
123398	cg0629848[illegible]	56632370	56632370	0.3329375	0	1	0.4378775	20.40766
123440	cg0630088[illegible]	17736396	17736396	0.6647685	0	1	0.868347	103.3985
123456	cg0630202[illegible]	32682378	56655426	0.4846727	0	1	0.3836638	-18.56799
123506	cg0630575[illegible]	33684488	33684488	0.781123	0	1	0.6045734	-62.56127
123514	cg0630656[illegible]	34681342	34681342	0.7520805	0	1	0.3156782	-340.2837
123598	cg0631246[illegible]	31807475	31807475	0.2857866	0	1	0.3989867	24.31805
123641	cg0631488[illegible]	31801434	31801434	0.7995651	0	1	0.9222823	66.1516
123648	cg0631539[illegible]	12656471	71690449	0.2836423	0	1	0.169473	-31.67188
123659	cg0631610[illegible]	32645422	59657486	0.3421159	0	1	0.5772165	84.29151
123749	cg0632227[illegible]	28786458	65607459	0.1836153	0	1	0.0748604	-43.43335
123887	cg0633059[illegible]	45767371	34810417	0.8082952	0	1	0.659722	-51.24242
123933	cg0633323[illegible]	68734430	68734430	0.3650567	0	1	0.5028927	31.37621
123944	cg0633423[illegible]	63754370	37773495	0.7410017	0	1	0.8587899	43.71332
124072	cg0634207[illegible]	39699315	39699315	0.3008035	0	1	0.4594947	41.924
124175	cg0634877[illegible]	21770427	34717427	0.3283757	0	1	0.4435366	23.81157
124190	cg0634976[illegible]	43635502	69640364	0.3255757	0	1	0.4452055	25.40755
124206	cg0635054[illegible]	36770426	46765377	0.9445748	0	1	0.7701086	-121.3202
124248	cg0635261[illegible]	58777301	47654342	0.1576189	0	1	0.0471314	-52.31247
124292	cg0635469[illegible]	25729301	25729301	0.2653281	0	1	0.42721	45.29131
124338	cg06358191	63797319	63797319	0.5448875	0	1	0.6507796	21.32047
124369	cg0635988[illegible]	38799348	38799348	0.3296655	0	1	0.4309811	19.35479
124384	cg0636082[illegible]	31759367	31759367	0.8162514	0	1	0.6338391	-72.7927
124427	cg0636303[illegible]	39683376	39683376	0.1885537	0	1	0.3251589	39.8082
124491	cg06366791	47605483	47605483	0.4081666	0	1	0.2736104	-32.79396
124661	cg0637443[illegible]	53655465	53655465	0.6549168	0	1	0.8059267	52.18045
124788	cg06380401	16791420	16791420	0.6828575	0	1	0.7952408	31.79003
124953	cg0639036[illegible]	12768371	71778401		0	1	0.9012603	4.34E-09
125033	cg0639482[illegible]	64600507	64600507	0.083242	0	1	0.2110712	52.67126
125080	cg0639752[illegible]	13644325	70688368		0	1		-4.34E-09
125089	cg0639824[illegible]	62623438	62629443	0.1426565	0	1	0.0236616	-67.94889
125234	cg0640704[illegible]	50810438	50810438	0.4265207	0	1	0.2445544	-57.13295
125378	cg0641668[illegible]	29808391	29808391	0.3462587	0	1	0.2429499	-22.59585

125484	cg0642203[illegible]	49645414	28720374	0.0881867	0	1	0.196751	40.42129
125504	cg0642275[illegible]	16691453	16691453	0.3472868	0	1	0.5273753	50.76881
125592	cg0642919[illegible]	10616505	10616505	0.5634673	0	1	0.6779373	25.04177
125613	cg0643068[illegible]	65688320	65688320	0.6151443	0	1	0.487167	-28.06318
125627	cg0643168[illegible]	14667388	14667388	0.3109756	0	1	0.4340647	27.01629
125658	cg0643380[illegible]	15792435	15792435	0.2544277	0	1	0.3796901	30.06413
125720	cg0643720[illegible]	60686369	60686369	0.9177117	0	1	0.800786	-60.05438
125736	cg0643793[illegible]	70650364	70650364	0.3798566	0	1	0.2308581	-41.70465
125752	cg0643855[illegible]	27606375	27606375	0.5648144	0	1	0.6789536	24.96334
125942	cg0645037[illegible]	51739444	51739444	0.7918526	0	1	0.9129556	61.11934
126068	cg0645773[illegible]	67621496	67621496	0.5318604	0	1	0.7332391	70.66274
126098	cg0645910[illegible]	35778480	35778480	0.5279873	0	1	0.4213854	-20.09781
126119	cg0646018[illegible]	51774338	51774338	0.4991718	0	1	0.6230404	26.74227
126123	cg0646047[illegible]	36624324	55735341	0.6827845	0	1	0.5585932	-28.77746
126141	cg0646158[illegible]	71687503	71687503	0.5223001	0	1	0.8940502	374.3439
126191	cg0646407[illegible]	49764417	49764417	0.3432816	0	1	0.7599837	374.3439
126263	cg0646838[illegible]	70632375	70632375	0.7172235	0	1	0.617089	-21.71362
126285	cg0646944[illegible]	73698361	73698361	0.4473203	0	1	0.5489936	18.63455
126307	cg0647085[illegible]	57681456	66764464	0.7618363	0	1	0.5746795	-65.87816
126334	cg0647242[illegible]	26623439	26623439	0.6958822	0	1	0.5747123	-28.22923
126406	cg0647669[illegible]	48787484	48787484	0.6985025	0	1	0.8185366	38.10655
126432	cg0647850[illegible]	67604416	42625372	0.6984588	0	1	0.5957973	-21.78481
126530	cg0648412[illegible]	69796300	69796300	0.3718366	0	1	0.5188625	35.0026
126533	cg0648416[illegible]	56663455	56663455	0.370115	0	1	0.6129801	89.9006
126673	cg0649159[illegible]	35796438	35796438	0.6149403	0	1	0.3999029	-70.89273
126687	cg0649237[illegible]	65606397	65606397	0.30199	0	1	0.4048279	20.42706
126714	cg0649380[illegible]	35687442	35687442	0.6722213	0	1	0.8022178	40.75277
126884	cg0650325[illegible]	39702400	23802427	0.8705717	0	1	0.6818839	-92.99949
126897	cg0650398[illegible]	38736460	38736460	0.6934296	0	1	0.9078639	131.457
126965	cg0650763[illegible]	70618405	70618405	0.2625931	0	1	0.1251798	-49.03654
127012	cg0651118[illegible]	53644305	31626381	0.2607636	0	1	0.1431367	-36.16415
127016	cg0651133[illegible]	14741422	14741422	0.2939254	0	1	0.4141659	26.5227
127055	cg0651386[illegible]	73790437	56733367		0	1	0.0289087	4.34E-09
127191	cg0652147[illegible]	29670416	38737311	0.5048316	0	1	0.394823	-21.2488
127233	cg0652315[illegible]	20745504	20745504	0.5990594	0	1	0.7967461	78.502
127258	cg0652426[illegible]	16628418	16628418	0.588086	0	1	0.4608111	-27.36042
127280	cg0652545[illegible]	49601316	49601316	0.3004597	0	1	0.4393887	33.42489
127427	cg0653157[illegible]	15760498	15760498	0.3286444	0	1	0.1779307	-47.93805
127532	cg0653615[illegible]	72678327	72678327	0.5075263	0	1	0.1565896	-220.0791
127712	cg0654520[illegible]	29750446	66809442	0.9046402	0	1	0.8003846	-46.99668
127790	cg0654923[illegible]	10808332	37751507	0.4495991	0	1	0.347598	-19.26519
127803	cg0654987[illegible]	32756434	32756434	0.2952527	0	1	0.4932916	62.45049
127806	cg0654992[illegible]	51784479	16726332	0.1710357	0	1	0.0710223	-39.29499

127818	cg06550274	44615360	44615360	0.9256324	0	1	0.6456885	-206.3931
127978	cg06559575	11755457	11755457	0.4371162	0	1	0.5641296	27.04319
128030	cg06562718	51790496	51790496	0.942102	0	1	0.7944105	-96.68732
128090	cg06566034	36644315	30733327	0.721475	0	1	0.6158665	-23.79836
128123	cg06568346	44629445	44629445	0.7460089	0	1	0.854023	37.59364
128168	cg06570432	32654390	32654390	0.6551806	0	1	0.5438523	-23.22192
128203	cg06573096	26705468	26705468	0.5213007	0	1	0.4091724	-21.86781
128303	cg06578276	16637439	16637439	0.2207256	0	1	0.3344437	27.5214
128394	cg06583549	34632461	67678312	0.797662	0	1	0.6348735	-57.35844
128405	cg06584218	10757344	10757344	0.2517639	0	1	0.3899198	35.4486
128442	cg06586265	45613487	45613487	0.2316863	0	1	0.3865294	44.38956
128459	cg06587249	21769327	21685348	0.5518469	0	1	0.7146884	47.50702
128466	cg06587955	36701322	36701322	0.0833657	0	1	0.2098002	51.79736
128479	cg06588556	53606434	63683508	0.6544884	0	1	0.5246623	-29.82086
128493	cg06589191	27753383	13726352	0.6581166	0	1	0.7587938	24.11684
128494	cg06589199	35615321	35615321	0.2554225	0	1	0.3555531	21.02202
128559	cg06593753	36733361	36733361	0.4049804	0	1	0.2418023	-47.61417
128577	cg06595154	23641489	23641489	0.2093621	0	1	0.3334266	32.42647
128608	cg06596943	71618389	71618389	0.3451556	0	1	0.4718676	27.51118
128994	cg06617490	32784332	32784332	0.915158	0	1	0.6760054	-158.2923
129009	cg06618134	49798311	49798311	0.6653062	0	1	0.5591956	-21.7916
129041	cg06619621	23669487	68801420	0.3867481	0	1	0.2660491	-27.77125
129046	cg06619938	66703337	55610394	0.2058238	0	1	0.101464	-35.76552
129051	cg06620117	10731450	10731450	0.4938672	0	1	0.5961281	19.18052
129076	cg06621027	43726361	43726361	0.1615877	0	1	0.278955	33.87855
129172	cg06624527	25798433	10630451	0.554207	0	1	0.4482887	-19.93701
129249	cg06628792	19665466	19665466	0.1748868	0	1	0.033033	-80.73279
129283	cg06630839	52715317	32741367	0.3299969	0	1	0.2251095	-24.00941
129299	cg06631775	24644310	56680385	0.3608665	0	1	0.5692111	66.42929
129353	cg06634367	16610505	11714504	0.4162266	0	1	0.9232849	374.3439
129361	cg06634576	33763414	35721391	0.8135511	0	1	0.4916213	-190.7719
129365	cg06634717	14804417	35630510	0.3209844	0	1	0.4459929	27.43675
129393	cg06635764	50764414	53675436	0.6963878	0	1	0.8253763	43.63864
129624	cg06645921	61626420	38633476	0.2366855	0	1	0.1262347	-35.00513
129675	cg06647930	43653440	43653440	0.3509032	0	1	0.5030199	37.48166
129694	cg06649034	54780312	22711458	0.2024755	0	1	0.099191	-35.59822
129723	cg06650246	51790402	33744484	0.4867047	0	1	0.662387	50.46664
129739	cg06650786	41798378	41798378	0.1303292	0	1	0.2949326	63.71275
129885	cg06657917	49756328	30766450	0.8804027	0	1	0.7687411	-45.01006
130008	cg06665333	11641398	43666501	0.6074587	0	1	0.498263	-21.48519
130013	cg06665622	27804429	27804429	0.2073497	0	1	0.3379641	35.3719
130146	cg06674117	25647452	25647452	0.6909047	0	1	0.8330295	52.12275
130167	cg06675417	36780493	36780493	0.4300424	0	1	0.5949184	43.04725

130376	cg0668642[illegible]	43760371	43760371	0.8664057	0	1	0.6384469	-122.1321
130416	cg0668880[illegible]	54745316	54745316	0.5453171	0	1	0.3675949	-49.2342
130431	cg0668961[illegible]	67729462	67729462	0.8189496	0	1	0.9210645	50.99629
130439	cg0669008[illegible]	36632421	36632421	0.3385201	0	1	0.4908964	37.86454
130465	cg0669161[illegible]	52766478	52766478	0.5474404	0	1	0.6881224	35.61942
130573	cg0669833[illegible]	67659489	67659489	0.3016113	0	1	0.4309106	29.60889
130601	cg0669948[illegible]	29618468	29618468	0.8517437	0	1	0.6522403	-94.29826
130608	cg0669966[illegible]	53673361	48603472	0.904757	0	1	0.7035603	-118.4479
130736	cg0670644[illegible]	74749383	74749383	0.9265606	0	1	0.6679045	-185.3064
130748	cg0670723[illegible]	59657317	55660419	0.7441181	0	1	0.6069927	-37.88247
130795	cg0670929[illegible]	23667375	23667375	0.4919438	0	1	0.6265461	30.82058
130846	cg0671141[illegible]	59719305	59719305	0.3758124	0	1	0.4965731	24.96113
130881	cg0671311[illegible]	15697445	15697445	0.0665271	0	1	0.2281911	80.49416
130977	cg0671815[illegible]	36768332	36768332	0.2187036	0	1	0.4458254	87.99928
130984	cg0671892[illegible]	19726359	19726359	0.3485556	0	1	0.4654372	24.00422
131042	cg0672154[illegible]	71751418	71751418	0.3380242	0	1	0.1609095	-65.21651
131127	cg0672623[illegible]	11759300	11759300	0.4313909	0	1	0.5420669	21.39039
131150	cg0672733[illegible]	52647447	52647447	0.1358528	0	1	0.2429689	32.1971
131167	cg0672825[illegible]	17687483	53744414	0.3267144	0	1	0.1964936	-36.13859
131312	cg0673622[illegible]	35758367	35758367	0.4060301	0	1	0.5936494	54.48923
131325	cg0673730[illegible]	27715457	27715457	0.8146468	0	1	0.6778713	-45.97857
131340	cg0673806[illegible]	27652434	27652434	0.3435914	0	1	0.4956057	37.5897
131412	cg0674133[illegible]	37611325	50676357	0.7008451	0	1	0.8015528	27.44827
131450	cg0674345[illegible]	20769409	20769409	0.6211321	0	1	0.8663349	136.8508
131530	cg0674733[illegible]	50797362	50797362	0.3777644	0	1	0.2281913	-42.18738
131695	cg0675525[illegible]	12697311	12697311	0.8066372	0	1	0.9172908	55.49945
131770	cg0675819[illegible]	30630472	30630472	0.1615884	0	1	0.3270701	58.30573
131850	cg0676090[illegible]	36602415	16749429	0.6528393	0	1	0.7705718	31.74533
131869	cg0676191[illegible]	63615329	63615329	0.2530061	0	1	0.5318499	123.9805
131900	cg0676377[illegible]	49682499	49682499	0.2918882	0	1	0.4328868	34.60833
131903	cg0676382[illegible]	31694485	27758434	0.1239852	0	1	0.0201577	-58.14479
132028	cg0676982[illegible]	11806416	11806416	0.2950535	0	1	0.4528845	41.78418
132201	cg0677866[illegible]	26715408	26609404	0.5532014	0	1	0.6702121	25.65905
132265	cg0678194[illegible]	26739477	26739477	0.6155054	0	1	0.7960716	67.52711
132424	cg0678982[illegible]	73669314	73669314	0.8387184	0	1	0.6886593	-57.89428
132481	cg0679226[illegible]	54757347	18617354	0.494887	0	1	0.3510306	-34.01086
132488	cg0679242[illegible]	17787407	17787407	0.6392149	0	1	0.2384541	-272.7782
132525	cg0679414[illegible]	30800394	30800394	0.114762	0	1	0.2363345	42.39663
132572	cg0679643[illegible]	60704500	63750357	0.2803451	0	1	0.1293125	-55.89855
132579	cg0679671[illegible]	55650417	55650417	0.1537052	0	1	0.2553406	27.88104
132617	cg0679811[illegible]	30656349	30656349	0.357432	0	1	0.5039128	34.97811
132630	cg0679888[illegible]	43788459	43788459	0.6283844	0	1	0.7717711	43.53958
132648	cg0679974[illegible]	61602392	61602392	0.5042233	0	1	0.6514327	36.71506

132653	cg0680022	69728463	69728463	0.3527836	0	1	0.4824601	28.46075
132733	cg0680441	43684436	43684436	0.219976	0	1	0.3462436	32.56204
132778	cg0680686	35635464	35635464	0.1865344	0	1	0.2971103	28.67954
132838	cg0680928	44783365	44783365	0.8520042	0	1	0.739407	-39.62689
132884	cg0681206	47611437	50686435	0.764048	0	1	0.6591793	-26.04634
132902	cg0681279	49703384	49703384	0.4517816	0	1	0.6570248	66.48178
132904	cg0681283	70635328	70635328	0.3045815	0	1	0.4180554	23.8322
132991	cg0681655	29782437	29782437	0.8036452	0	1	0.9171333	57.41179
133024	cg0681815	51757488	51757488	0.4160916	0	1	0.537834	25.0929
133046	cg0681886	24810505	24810505	0.5125247	0	1	0.6321293	25.44501
133104	cg0682107	72731423	72731423	0.6669881	0	1	0.775818	28.50751
133118	cg0682182	17685494	17685494	0.6946895	0	1	0.5673252	-30.54743
133130	cg0682219	59677439	59677439	0.3734156	0	1	0.49317	24.63983
133146	cg0682303	74683424	66622482	0.1935842	0	1	0.0666021	-56.83191
133210	cg0682612	41603422	42687466	0.9199639	0	1	0.778935	-78.72994
133254	cg0682814	52714396	52714396	0.5484653	0	1	0.6543366	21.40639
133324	cg0683106	47715326	23803388	0.7074202	0	1	0.603037	-22.72867
133389	cg0683398	30630339	30630339	0.7577312	0	1	0.4877199	-124.7242
133437	cg0683581	54768320	54768320	0.6534176	0	1		-4.34E-09
133495	cg0683821	18744339	18744339	0.6513389	0	1	0.8338683	77.12499
133512	cg0683911	24758505	24758505	0.2709469	0	1	0.3722482	20.84856
133623	cg0684471	72651489	72651489	0.8080668	0	1	0.698742	-31.81477
133625	cg0684484	19796492	19796492	0.8792211	0	1	0.610693	-164.6727
133696	cg0684848	21716407	21716407	0.2853105	0	1	0.3998363	24.79395
133902	cg0685808	53796358	53796358	0.5734274	0	1	0.6852916	24.40771
134034	cg0686564	73713481	73713481	0.6684377	0	1	0.4951816	-49.5619
134080	cg0686748	27650399	24604317	0.7028953	0	1	0.8052731	28.50645
134146	cg0687011	38774451	38774451	0.8210083	0	1	0.7034237	-37.26043
134171	cg0687134	41643338	41643338	0.1935619	0	1	0.3938987	74.00336
134297	cg0687607	28674327	28674327	0.3715269	0	1	0.2626098	-23.69421
134461	cg0688449	46682499	46682499	0.6436164	0	1	0.539021	-20.7332
134495	cg0688673	61790357	42731362	0.3367413	0	1	0.4593748	26.21573
134532	cg0688846	43681321	32771453	0.7356281	0	1	0.6009943	-36.09797
134709	cg0689576	37722354	37722354	0.6809694	0	1	0.5297065	-39.82669
134737	cg0689744	31666360	31666360	0.4789838	0	1	0.3616538	-23.95775
134809	cg0690057	11749329	11749329	0.5668501	0	1	0.6769024	23.47729
134905	cg0690576	30674312	29675455		0	1		-4.34E-09
134982	cg0690925	19690510	19690510	0.3922799	0	1	0.5007782	20.78735
135075	cg0691351	62658445	69802376	0.8512978	0	1	0.7235714	-47.70833
135269	cg0692490	13759383	13759383	0.8817637	0	1	0.5708129	-210.1768
135276	cg0692530	19801472	19801472	0.4646529	0	1	0.5744858	21.28961
135403	cg0693253	31652477	31652477	0.327464	0	1	0.4361291	21.69233
135468	cg0693543	28700404	28700404	0.4878466	0	1	0.6396315	38.25864

135485	cg0693629(	23804420	23804420	0.4264482	0	1	0.6044539	49.62234
135531	cg0693792'	36675346	36675346	0.3186301	0	1	0.4551023	31.8396
135541	cg0693826‹	35664356	40687337	0.3179055	0	1	0.1683432	-48.61809
135594	cg0694115(	46659489	46659489	0.6185852	0	1	0.7408825	31.29373
135614	cg0694211l	15602396	15602396	0.578531	0	1	0.7519274	56.71382
135757	cg0694898‹	20774355	58714333	0.4988409	0	1	0.3865838	-22.02349
135793	cg0695112‹	14612478	44784383	0.7610736	0	1	0.6362478	-33.94233
135893	cg0695595‹	16625306	16625306	0.8969814	0	1	0.6048881	-198.9609
135948	cg0695876(	24600374	60790506	0.4480273	0	1	0.611859	42.88183
135964	cg0695944{	23708426	23708426	0.7731875	0	1	0.8951507	55.45601
135988	cg0696071'	65763472	65763472	0.7549906	0	1	0.5652317	-66.46093
136015	cg0696142‹	69617424	69617424	0.5661094	0	1	0.6838949	26.45539
136156	cg0696871‹	14737440	14737440	0.7003436	0	1	0.5880243	-25.19012
136215	cg0697122‹	39602432	39602432	0.4141939	0	1	0.6116691	60.26929
136307	cg0697597‹	22680370	58647446	0.4715982	0	1	0.3713763	-18.44416
136314	cg0697622‹	32734337	32734337	0.84817	0	1	0.7459216	-33.73677
136362	cg0697902(	11702434	11702434	0.5212708	0	1	0.6378168	24.52594
136370	cg0697938(	27693451	27693451	0.3080958	0	1	0.1685015	-43.53809
136417	cg0698216‹	50801467	50801467	0.7218206	0	1	0.6074159	-27.01455
136429	cg0698282'	50657301	50657301	0.8054988	0	1	0.9137506	52.57099
136451	cg0698420l	15792440	15792440	0.657383	0	1	0.5551925	-20.31675
136613	cg0699550‹	72669423	72669423	0.3631596	0	1	0.581502	72.71685
136856	cg0700712‹	48715323	48715323	0.6249961	0	1	0.7258225	22.34372
136959	cg0701153{	24806349	24806349	0.4523519	0	1	0.5998566	35.43084
137048	cg0701574‹	14780443	28697376	0.7940425	0	1	0.618704	-64.01305
137124	cg0701843‹	69725465	69725465	0.7009133	0	1	0.5962396	-22.55669
137191	cg0702153‹	23767478	23767478	0.0850509	0	1	0.2563781	80.54282
137227	cg0702349‹	16735430	16735430	0.2723078	0	1	0.4233221	39.8508
137273	cg0702598‹	61761330	60706463	0.1918252	0	1	0.2962985	25.88662
137328	cg0702895(	16757359	16757359	0.2902935	0	1	0.4132683	27.64246
137343	cg0702977.	36775473	36775473	0.2374855	0	1	0.3400856	22.59919
137370	cg0703140{	19606305	30614363	0.7151587	0	1	0.5885044	-31.38016
137404	cg0703329‹	55606483	55606483	0.2383559	0	1	0.3420588	22.93891
137466	cg0703596l	32670378	50677405	0.2187151	0	1	0.1139958	-33.98669
137505	cg0703796‹	34643500	34643500	0.9134346	0	1	0.8121894	-47.78734
137555	cg0704148{	57693376	57693376	0.8802577	0	1	0.7788242	-39.11638
137674	cg0704821(	11754467	11754467	0.3219377	0	1	0.4279205	20.95289
137732	cg0705116‹	37752468	24601384	0.2367418	0	1	0.0978054	-55.71799
137773	cg0705311‹	69632377	18728350	0.6780066	0	1	0.7817257	26.93745
137811	cg0705468'	27678345	12778447	0.2752807	0	1	0.425844	39.48902
137951	cg0706178‹	17644464	65610316	0.5887991	0	1	0.4223737	-43.69286
137991	cg0706355‹	13760348	30774479	0.6358883	0	1	0.7397204	24.12474
138013	cg0706449‹	58683483	13674339	0.2015432	0	1	0.0537086	-75.71036

138084	cg07068181	28608460	28608460	0.9316788	0	1	0.8179788	-63.7425
138233	cg07075347	59760319	26780402	0.359107	0	1	0.2499979	-24.29396
138234	cg07075387	49604440	42704427	0.7488379	0	1	0.6266093	-31.85004
138296	cg07078958	64671370	64671370	0.6148985	0	1	0.8017944	72.5717
138366	cg07082452	47687428	35795412	0.7467541	0	1	0.6361019	-27.08945
138384	cg07083818	16708468	16708468	0.2826065	0	1	0.4042865	27.45168
138562	cg07092880	17741334	17741334	0.7211084	0	1	0.8532647	50.645
138567	cg07093060	24677315	24677315	0.4900171	0	1	0.3718168	-24.12735
138738	cg07101208	16671320	16671320	0.2685241	0	1	0.3741143	22.34052
138746	cg07101841	23606375	67630445	0.1434442	0	1	0.2471044	29.78679
138786	cg07103846	31649399	60701397	0.2849885	0	1	0.4654329	53.37933
138830	cg07105947	28654322	29639424	0.1692813	0	1	0.0570069	-50.33597
138850	cg07106927	30800449	30800449	0.8911998	0	1	0.7859344	-43.90322
138964	cg07113199	56696398	18634480	0.2401372	0	1	0.1344815	-31.69878
138979	cg07114310	16646394	16646394	0.388598	0	1	0.2461652	-37.6397
138990	cg07114810	63676506	63676506	0.279444	0	1	0.3915718	24.16762
139031	cg07116631	69808469	36794428	0.5429079	0	1	0.4402729	-18.90276
139033	cg07116712	20799343	72652348	0.148087	0	1	0.2702562	37.68419
139086	cg07119157	37708397	37708397	0.416292	0	1	0.5593679	33.19637
139188	cg07124183	18742431	18742431	0.6300343	0	1	0.8739329	139.2117
139289	cg07128503	47802391	47802391	0.7753536	0	1	0.9057165	64.78946
139412	cg07134368	31604480	31604480	0.4202435	0	1	0.5454443	26.32223
139456	cg07136254	18664440	18664440	0.4060949	0	1	0.5112257	19.67759
139471	cg07136920	11658305	10709499	0.457676	0	1	0.6292428	47.04786
139479	cg07137336	25632370	25632370	0.4763469	0	1	0.340482	-31.0442
139542	cg07139527	17733357	17733357	0.7070895	0	1	0.822872	36.64743
139620	cg07143158	32685420	35752322	0.280761	0	1	0.4185829	33.788
140064	cg07161993	15623461	15623461	0.4218968	0	1	0.3133266	-21.97714
140187	cg07167933	69741325	69741325	0.4997515	0	1	0.6100785	21.88975
140294	cg07173547	13618386	13618386	0.1964482	0	1	0.0852096	-42.52386
140419	cg07179033	38633370	38633370	0.1891595	0	1	0.3257124	39.72149
140489	cg07181702	61745374	61745374	0.1845014	0	1	0.3042365	32.6574
140500	cg07182616	60691506	60691506	0.4773466	0	1	0.2501387	-84.23383
140591	cg07187202	60725377	60725377	0.4787579	0	1	0.6473965	46.28606
140616	cg07188591	73779374	32785438	0.1516852	0	1	0.0444619	-51.03911
140842	cg07200874	62797430	40779370	0.7989091	0	1	0.6938999	-28.9935
140845	cg07200939	66618309	66618309	0.3414045	0	1	0.5186955	49.47559
140855	cg07201475	51673397	51673397	0.501365	0	1	0.6527978	38.58912
140917	cg07204662	35802327	63636368	0.2452544	0	1	0.3665987	29.0322
140943	cg07205796	40763390	29665340	0.2668081	0	1	0.3672222	20.70293
141121	cg07213181	31748390	31748390	0.2399205	0	1	0.3668068	31.51364
141137	cg07213780	13608319	13608319	0.3197476	0	1	0.4420325	26.48801
141160	cg07215504	63601400	69745364	0.5731859	0	1	0.3688527	-63.94715

141165	cg0721569…	25803509	69798377	0.7194481	0	1	0.6001083	-28.75616
141185	cg0721652…	44631403	44631403	0.2645345	0	1	0.3748462	24.10006
141187	cg0721661…	16609425	16609425	0.7221271	0	1	0.269128	-370.3835
141262	cg0722065…	24778345	24778345	0.2595974	0	1	0.3610176	21.2879
141275	cg0722125…	20797361	20797361	0.1955063	0	1	0.3049413	27.54582
141319	cg0722320…	65740494	49653476	0.8266762	0	1	0.6557544	-67.88306
141398	cg0722718…	68679473	13794443	0.6455215	0	1	0.7763352	38.07747
141409	cg0722824…	56749353	60751355	0.5021158	0	1	0.604713	19.39539
141427	cg0722976…	26611405	26611405	0.585779	0	1	0.695962	24.248
141434	cg0723035…	72645482	46695468	0.4300697	0	1	0.541086	21.4978
141465	cg0723200…	46717453	46717453	0.3386892	0	1	0.5169298	50.02559
141632	cg0724084…	35619467	35619467	0.2216574	0	1	0.3675859	41.03727
141636	cg0724108…	48806509	37709383	0.3174666	0	1	0.421354	20.39084
141784	cg0724948…	24611488	24611488	0.6580282	0	1	0.9006954	151.7644
141865	cg0725442…	19650479	19650479	0.6246968	0	1	0.8131824	76.02532
141910	cg0725782…	28751331	39657373	0.2644255	0	1	0.3835478	27.25898
141915	cg0725840…	41739423	37800497	0.8692248	0	1	0.3585295	-371.3336
141934	cg0725898…	61749335	61749335	0.830179	0	1	0.5365865	-167.0316
141964	cg0726059…	60648498	60648498	0.4523048	0	1	0.3417867	-22.01738
142067	cg0726580…	13716447	13716447	0.6858023	0	1	0.7885029	27.09414
142094	cg0726760…	14746373	14746373	0.120221	0	1	0.2405058	40.79978
142107	cg0726824…	30758428	30758428	0.4695117	0	1	0.6046403	30.54819
142116	cg0726872…	12744392	12744392	0.8593485	0	1	0.6499932	-104.4061
142181	cg0727322…	35708382	35708382	0.5394451	0	1	0.6811516	35.6927
142384	cg0728426…	13671359	13671359	0.3720583	0	1	0.5455776	47.0773
142406	cg0728523…	64612381	64612381	0.4613942	0	1	0.6523701	58.056
142413	cg0728567…	27652378	27652378	0.6598333	0	1	0.7836698	35.6682
142538	cg0729411…	58629344	58629344	0.7118466	0	1	0.6066574	-23.19952
142551	cg0729478…	68716445	27622334	0.5279478	0	1	0.6564484	29.38494
142558	cg0729524…	57635507	57635507	0.5044901	0	1	0.3931727	-21.67634
142677	cg0730150…	71714478	67810399		0	1		-4.34E-09
142826	cg0731050…	58778310	58778310	0.2371814	0	1	0.3375164	21.84122
142840	cg0731102…	59645339	59645339	0.2548713	0	1	0.1366871	-37.28358
142973	cg0731673…	53683400	53683400	0.5727301	0	1	0.7013881	31.4031
143081	cg0732214…	48755323	48755323	0.3532428	0	1	0.2449856	-24.22197
143320	cg0733712…	41648304	41648304	0.7777936	0	1	0.6701486	-28.19092
143585	cg0735116…	15625403	13644383		0	1	0.0279873	4.34E-09
143673	cg0735568…	39768474	39768474	0.2848807	0	1	0.38616	20.40382
143681	cg0735592…	25621344	61624473	0.4016612	0	1	0.2796725	-27.67919
143725	cg0735771…	33649493	33649493	0.2839445	0	1	0.4378465	40.53376
143842	cg0736512…	45618394	45618394	0.2970636	0	1	0.1569792	-45.32166
143850	cg0736574…	61780399	61780399	0.63161	0	1	0.4603078	-46.99538
143950	cg0737141…	51725339	51725339	0.4850506	0	1	0.6209527	31.18734

143960	cg0737174[illegible]	40673411	40673411	0.3927568	0	1	0.4992716	20.16716
144031	cg0737638[illegible]	10710408	19680443	0.3173959	0	1	0.2136259	-24.23935
144116	cg0738020[illegible]	11717415	11717415	0.4266227	0	1	0.5819161	38.52582
144143	cg0738150[illegible]	33659300	30603504	0.4040647	0	1	0.2616905	-36.66499
144166	cg0738234[illegible]	18809480	52801344	0.0349598	0	1	0.3161171	374.3439
144203	cg0738452[illegible]	35693419	35693419	0.7101743	0	1	0.8666489	69.13546
144525	cg0740293[illegible]	29764440	29764440	0.5186281	0	1	0.6444268	27.99355
144550	cg0740448[illegible]	14653354	14653354	0.6194373	0	1	0.514022	-20.49441
144593	cg0740773[illegible]	64794458	64794458	0.1128337	0	1	0.2586352	56.30824
144599	cg0740823[illegible]	10764503	10764503	0.5970359	0	1	0.7279721	33.95928
144669	cg0741395[illegible]	24651408	24651408	0.4806562	0	1	0.6459058	44.60377
144677	cg0741442[illegible]	17763405	17763405	0.5807054	0	1	0.7617205	62.26743
144763	cg0741970[illegible]	13644507	12806455		0	1	0.1759494	4.34E-09
144765	cg0741979[illegible]	53683423	53683423	0.2056965	0	1	0.3123724	25.79322
144802	cg0742132[illegible]	14643465	14643465	0.440189	0	1	0.5574115	23.58827
145093	cg0743792[illegible]	41664365	41664365	0.4376923	0	1	0.6144984	49.23421
145099	cg0743824[illegible]	58738403	58738403	0.473044	0	1	0.5846203	21.945
145153	cg0744127[illegible]	32783348	32783348	0.1882135	0	1	0.2971657	27.89864
145321	cg0745180[illegible]	29693347	29693347	0.3032554	0	1	0.428712	28.11593
145352	cg0745344[illegible]	22631335	22631335	0.709561	0	1	0.3696769	-187.3133
145360	cg0745385[illegible]	63781398	63781398	0.3688247	0	1	0.480698	22.06648
145391	cg0745579[illegible]	47702338	44613324	0.6457832	0	1	0.4058378	-88.71102
145411	cg0745658[illegible]	68672490	68672490	0.2191868	0	1	0.4594216	97.33704
145487	cg0746084[illegible]	25602402	25602402	0.4155318	0	1	0.2455744	-50.71325
145544	cg0746344[illegible]	18688340	18688340	0.1661307	0	1	0.2715715	28.30924
145598	cg0746616[illegible]	66752401	36759444	0.5186785	0	1	0.3638076	-38.4631
145713	cg0747096[illegible]	52763427	52763427	0.6918033	0	1	0.7944984	27.6183
145798	cg0747467[illegible]	68614381	68614381	0.4909409	0	1	0.3646953	-27.02688
145816	cg0747539[illegible]	18794413	28623314	0.6510935	0	1	0.5505644	-19.64292
145833	cg0747623[illegible]	16794477	16794477	0.580516	0	1	0.7490373	53.74023
145910	cg0747961[illegible]	71612425	25622453	0.8906528	0	1	0.7823595	-45.58389
146073	cg0748814[illegible]	69607345	69607345	0.2500978	0	1	0.3555929	23.03399
146108	cg0749007[illegible]	35716390	47692406	0.3122988	0	1	0.1506862	-57.96284
146142	cg0749204[illegible]	65743337	57795458	0.5559128	0	1	0.4016248	-37.94548
146161	cg0749304[illegible]	39621398	32729495	0.4017543	0	1	0.5093382	20.45139
146163	cg0749319[illegible]	52717438	52717438	0.4282987	0	1	0.6029607	47.91171
146206	cg0749540[illegible]	53603450	53603450	0.3935118	0	1	0.5989568	64.71526
146247	cg0749808[illegible]	32632486	32632486	0.5018086	0	1	0.6635982	43.75057
146270	cg0749918[illegible]	19642415	19642415	0.1802037	0	1	0.3129531	38.89052
146302	cg0750102[illegible]	48646448	48646448	0.3986031	0	1	0.2365616	-47.48291
146404	cg0750615[illegible]	30804471	30804471	0.9517365	0	1	0.8371479	-75.35973
146476	cg0750993[illegible]	35669497	40808423	0.2396571	0	1	0.0477566	-112.5421
146619	cg0751773[illegible]	41655378	41655378	0.5170674	0	1	0.6215166	20.23154

146644	cg0751942(	40786373	54737359	0.7256919	0	1	0.8402264	38.62522
146655	cg0751990{	68617489	40707471	0.3804106	0	1	0.2571374	-29.15942
146704	cg0752297(	19647471	19647471	0.2631283	0	1	0.3930335	31.44499
146718	cg0752371:	15798465	15798465	0.8391458	0	1	0.5343207	-182.1912
146750	cg0752547]	59768415	45696375	0.5292978	0	1	0.6363153	21.3358
146812	cg0752912:	51782346	55734325	0.2499731	0	1	0.3611517	25.02852
146875	cg0753208!	66676374	52766421	0.8445504	0	1	0.7172697	-46.11628
146926	cg0753484:	40738358	28717345	0.2234419	0	1	0.0873077	-56.68053
146983	cg0753756:	34743307	34743307	0.4432833	0	1	0.2754633	-47.54159
146993	cg0753782]	60767362	60767362	0.3091408	0	1	0.4274648	25.37487
147474	cg0756361]	57705400	69681379	0.1420827	0	1	0.2749263	43.79524
147508	cg0756497!	69750510	69750510	0.7914305	0	1	0.6383789	-51.0286
147711	cg0757468:	65680381	65680381	0.3028323	0	1	0.4132535	22.85593
147899	cg0758409:	20662320	20662320	0.2750582	0	1	0.3779875	21.23864
147908	cg0758462(	33708372	33708372	0.6273305	0	1	0.9041181	374.3439
147918	cg0758485!	18629366	26751504	0.4411911	0	1	0.5871307	34.59011
147927	cg0758542'	50730344	50730344	0.4875546	0	1	0.6035434	23.64451
147960	cg0758698	38602306	38602306	0.5568754	0	1	0.6630372	21.74172
148094	cg0759272:	36761355	36761355	0.4377516	0	1	0.3241956	-23.36541
148096	cg0759277!	12673368	12673368	0.8074283	0	1	0.5829436	-99.73914
148200	cg0759797(	32649400	32649400	0.5968012	0	1	0.7169671	28.93409
148226	cg0759895:	44802330	54623508	0.4295614	0	1	0.3179693	-22.85641
148228	cg0759913(	30633361	30633361	0.5643181	0	1	0.8069795	113.6625
148253	cg0760062:	71672475	12755445	0.4158979	0	1	0.6529431	86.98335
148378	cg0760752!	39677319	39677319	0.441299	0	1	0.3175653	-27.06555
148435	cg0761112]	29737489	29737489	0.8381348	0	1	0.7117807	-44.42548
148529	cg0761580:	60627488	60627488	0.772718	0	1	0.6276082	-44.4942
148596	cg0761968:	27762364	39611482	0.49187	0	1	0.6025833	21.897
148615	cg0762085:	70743481	70743481	0.6211501	0	1	0.8547722	122.0733
148635	cg0762169'	53769468	53769468	0.2294347	0	1	0.3933275	49.02733
148711	cg0762577	59768425	70628390	0.3056701	0	1	0.4249829	25.82224
148780	cg0762962!	50723326	15760401	0.4922109	0	1	0.8226824	374.3439
148844	cg0763366	37800461	37800461	0.7096494	0	1	0.6026205	-23.73973
148946	cg0763919{	19621471	19621471	0.8902836	0	1	0.5484398	-252.0738
148993	cg0764157{	58611359	58611359	0.9465476	0	1	0.832108	-72.09411
149013	cg0764276'	12687381	12687381	0.7341797	0	1	0.9146224	106.8496
149119	cg0764961!	45763323	73790462		0	1	0.0453841	4.34E-09
149152	cg0765218!	49739311	49739311	0.5897704	0	1	0.7174112	31.91293
149204	cg0765526]	68715408	68715408	0.9113884	0	1	0.7174591	-116.1058
149292	cg0766083{	70620322	70620322	0.3736822	0	1	0.2640119	-23.89854
149320	cg0766212]	33664390	12712374	0.0481372	0	1	0.1653274	56.45424
149363	cg0766457!	35709459	35709459	0.7326398	0	1	0.5905578	-39.16093
149379	cg0766522:	40601507	40601507	0.2889297	0	1	0.4005153	23.66162

149464	cg0767051	47794342	47794342	0.6589616	0	1	0.5447557	-24.31004
149504	cg0767281	11618327	11618327	0.8333495	0	1	0.727267	-33.4495
149507	cg0767302	10645390	10645390	0.3947096	0	1	0.2811269	-24.61185
149532	cg0767413	66667457	22611489	0.3722043	0	1	0.2593864	-25.19431
149576	cg0767636	48778458	46705324	0.8349367	0	1	0.6906264	-53.74508
149585	cg0767715	18621333	18621333	0.7424531	0	1	0.8619332	45.30906
149737	cg0768694	62687300	70644435	0.4856949	0	1	0.3392237	-35.33887
149789	cg0768973	47623328	47623328	0.1398371	0	1	0.2435396	30.20187
149901	cg0769627	11720369	11720369	0.8021473	0	1	0.9160036	57.23637
149925	cg0769722	26785447	13644463		0	1		-4.34E-09
149950	cg0769819	22657484	22657484	0.3776085	0	1	0.4908533	22.42012
149997	cg0770039	59645308	59645308	0.7015389	0	1	0.8247829	40.60561
150021	cg0770130	54789502	54789502	0.2416711	0	1	0.3459703	22.99311
150031	cg0770175	57744315	13608390	0.7448199	0	1	0.6360977	-26.23208
150037	cg0770223	20646417	20646417	0.3579215	0	1	0.5012036	33.65566
150229	cg0771108	22672493	66748471	0.5496975	0	1	0.6669875	25.64569
150241	cg0771216	36762442	63613473	0.4591823	0	1	0.6672152	68.75102
150474	cg0772462	62627470	62627470	0.6344336	0	1	0.5210387	-23.37727
150759	cg0774030	60667476	32658314	0.3985036	0	1	0.5263575	27.31366
150857	cg0774570	28677340	68655410	0.9461694	0	1	0.8062468	-92.90601
150888	cg0774725	64713395	39679370	0.7444395	0	1	0.6055681	-38.69505
150907	cg0774796	30725447	24621454	0.1438762	0	1	0.3257674	71.50509
150939	cg0774959	52632437	52632437	0.5591731	0	1	0.6956364	34.26067
151000	cg0775240	42685376	42685376	0.7418952	0	1	0.6199451	-31.22429
151012	cg0775336	65787348	65787348	0.4507769	0	1	0.5899691	31.89937
151179	cg0776405	27785330	27785330	0.7084142	0	1	0.8094648	28.36471
151210	cg0776598	58716480	58716480	0.0827275	0	1	0.2199086	58.58063
151325	cg0777442	26672398	26672398	0.3930528	0	1	0.2617883	-32.05433
151332	cg0777488	43685418	43685418	0.281127	0	1	0.1055695	-76.95684
151367	cg0777704	71750361	71750361	0.4093586	0	1	0.6279089	73.47298
151422	cg0778034	20804304	20804304	0.627684	0	1	0.5148189	-23.05055
151462	cg0778170	42706506	66726492	0.52256	0	1	0.6630753	34.3764
151554	cg0778754	21627306	21627306	0.4630288	0	1	0.5795686	23.52227
151657	cg0779287	37774469	37774469	0.6909856	0	1	0.2737306	-299.3461
151697	cg0779516	41604509	41702418	0.3263245	0	1	0.5603312	83.8867
151872	cg0780447	29663367	29663367	0.2061276	0	1	0.3172422	27.45816
151890	cg0780506	48751392	48751392	0.7659854	0	1	0.8782819	44.87391
151901	cg0780554	11719474	33743421	0.2626745	0	1	0.4374904	51.89871
151936	cg0780711	28755369	28755369	0.3964482	0	1	0.5787122	51.50676
151942	cg0780737	56788301	56788301	0.347375	0	1	0.6320238	124.743
152217	cg0782078	37641313	69718340	0.5160674	0	1	0.4094844	-20.11283
152228	cg0782137	36780352	36780352	0.7291528	0	1	0.6006014	-33.04596
152412	cg0783131	61647402	61647402	0.3919386	0	1	0.5858384	57.87875

152422	cg07832006	45781488	70708348	0.7717862	0	1	0.6137207	-50.97744
152575	cg07839926	62620439	52777432	0.5478258	0	1	0.4249084	-25.51474
152666	cg07845136	13644465	13644465		0	1	0.9444641	4.34E-09
152689	cg07846874	12763311	12763311	0.7222976	0	1	0.8652934	60.09278
152711	cg07848485	28767464	28767464	0.7276893	0	1	0.4796936	-101.8943
152740	cg07850323	53702395	53702395	0.4613978	0	1	0.6532553	58.58609
152766	cg07851262	56711481	56711481	0.8012025	0	1	0.6267867	-64.76083
152803	cg07854113	10734336	10734336	0.503373	0	1	0.6219854	24.87281
152833	cg07855575	14780479	14780479	0.4912502	0	1	0.6744604	55.06049
152927	cg07862461	34704447	34704447	0.2897675	0	1	0.4369828	37.3185
152940	cg07863022	57771331	68707371	0.3701217	0	1	0.4705316	18.51207
152992	cg07866725	28615375	28615375	0.4330168	0	1	0.1401722	-161.0091
152995	cg07866909	41604333	41604333	0.3898901	0	1	0.5391595	35.79926
153057	cg07870541	55751327	55751327	0.6947929	0	1	0.594503	-20.86874
153065	cg07870920	17666406	17666406	0.8899102	0	1	0.6736146	-123.2732
153123	cg07874011	16673473	16673473	0.816714	0	1	0.7121019	-30.69435
153142	cg07875068	46632507	46632507	0.3679916	0	1	0.4867688	24.36756
153170	cg07876320	17621427	17621427	0.8266126	0	1	0.9452972	74.80877
153179	cg07876831	50661376	50661376	0.874776	0	1	0.7612961	-44.74007
153205	cg07878625	18757475	18757475	0.6351039	0	1	0.3375252	-137.0987
153282	cg07882838	55609344	55609344	0.5246048	0	1	0.3561826	-44.80286
153315	cg07884867	20720372	20720372	0.6120472	0	1	0.7654341	47.78
153373	cg07889003	49677481	55756510	0.6296168	0	1	0.5278493	-19.55855
153506	cg07896009	35729356	35729356	0.1889738	0	1	0.2941399	26.35678
153617	cg07901514	28689449	28689449	0.2454481	0	1	0.3988475	42.64138
153657	cg07904290	58651475	39795354	0.7295662	0	1	0.8382076	35.37425
153698	cg07906262	13766454	53717395	0.5712174	0	1	0.440017	-28.63169
153763	cg07909178	30800340	30800340	0.2971048	0	1	0.3983184	20.04422
153900	cg07915976	42781406	42781406	0.6297705	0	1	0.747811	30.06479
153933	cg07917609	69634403	69634403	0.4968379	0	1	0.3670061	-28.30536
154087	cg07925064	41794502	24744335	0.2754197	0	1	0.3905331	25.35882
154195	cg07929768	27741510	27741510	0.4752218	0	1	0.3443685	-29.05379
154210	cg07930587	63750350	63750350	0.7974007	0	1	0.6167723	-67.8131
154241	cg07932899	25694489	25694489	0.8068022	0	1	0.6989552	-31.02296
154245	cg07933078	54680309	28641391	0.7289073	0	1	0.6061733	-30.66089
154323	cg07936950	73760356	74623336	0.3401639	0	1	0.2395954	-21.81328
154422	cg07943832	28624394	28624394	0.5279411	0	1	0.6705053	35.5472
154542	cg07950979	33721323	33721323	0.2440025	0	1	0.1295917	-36.44369
154571	cg07952421	11676351	11676351	0.4276774	0	1	0.3140261	-23.64042
154620	cg07955105	72695488	72695488	0.4488847	0	1	0.901095	374.3439
154718	cg07960624	62713349	62713349	0.4471646	0	1	0.5929185	34.59227
154768	cg07962934	60709444	30743500	0.8963992	0	1	0.7175492	-96.33426
154803	cg07964219	48640371	48640371	0.2468919	0	1	0.36418	27.40888

154809	cg0796475[illegible]	67661509	23759375	0.7169023	0	1	0.8681728	66.18538
154918	cg0797079[illegible]	46613418	31718497	0.1577416	0	1	0.284904	38.84739
154931	cg0797159[illegible]	30638432	30638432	0.6104464	0	1	0.7116907	21.84765
154972	cg0797316[illegible]	72765306	72765306	0.3208818	0	1	0.1227038	-87.60863
154975	cg0797330[illegible]	71741400	71741400	0.7934957	0	1	0.9205216	68.53181
155030	cg0797678[illegible]	44746439	44746439	0.5229662	0	1	0.6387951	24.305
155066	cg0797934[illegible]	53691440	53691440	0.4801704	0	1	0.5959164	23.45093
155108	cg0798126[illegible]	67766331	67766331	0.1002127	0	1	0.2325623	51.26328
155162	cg0798438[illegible]	69795483	69795483	0.0752829	0	1	0.2139658	61.72425
155361	cg0799683[illegible]	28746438	28746438	0.2410423	0	1	0.3412217	21.61955
155436	cg0800073[illegible]	52628380	52628380	0.3274027	0	1	0.4411957	23.37373
155473	cg0800340[illegible]	55749387	55749387	0.7048661	0	1	0.8276444	40.81111
155555	cg0800823[illegible]	50707487	50707487	0.9014547	0	1	0.6812903	-132.7291
155587	cg0801033[illegible]	64762362	64762362	0.6856555	0	1	0.8670691	86.42607
155703	cg0801763[illegible]	39801427	39801427	0.530213	0	1	0.6502585	26.08677
155717	cg0801814[illegible]	71767433	71767433	0.455568	0	1	0.5583754	19.01374
155814	cg0802426[illegible]	66713451	66713451	0.1974193	0	1	0.5259973	374.3439
155890	cg0802929[illegible]	48617371	48617371	0.3394081	0	1	0.4712277	29.5193
155897	cg0802996[illegible]	61672364	73645392	0.3790468	0	1	0.490403	21.79552
155911	cg0803092[illegible]	36730347	36730347	0.3049318	0	1	0.4492903	35.49082
155996	cg0803535[illegible]	43606408	43606408	0.3495753	0	1	0.4599204	21.83322
156056	cg0803803[illegible]	10739488	10739488	0.2135275	0	1	0.3314539	29.62771
156095	cg0803958[illegible]	10696356	10696356	0.6972103	0	1	0.8372053	51.74782
156131	cg0804144[illegible]	36801357	14739409	0.4733297	0	1	0.5986517	26.80418
156247	cg0804826[illegible]	49647508	41755442	0.6195458	0	1	0.2848347	-178.0858
156318	cg0805313[illegible]	35684432	35684432	0.7361644	0	1	0.4817337	-108.1265
156468	cg0806220[illegible]	39733387	35620477	0.4161235	0	1	0.5999547	52.55811
156507	cg0806429[illegible]	58725450	58725450	0.0961571	0	1	0.2202621	47.42279
156522	cg0806522[illegible]	56800391	56800391	0.4436899	0	1	0.5581894	22.68607
156743	cg0807643[illegible]	38678341	17774500	0.5436103	0	1	0.3693095	-47.49291
156844	cg0808178[illegible]	53621420	53621420	0.6680812	0	1	0.7692778	25.01332
156889	cg0808415[illegible]	46694365	21755423	0.8735717	0	1	0.761645	-43.57604
157032	cg0809166[illegible]	65671470	65671470	0.2623669	0	1	0.4244458	45.57776
157137	cg0809781[illegible]	37758335	37758335	0.4709447	0	1	0.6047426	30.04036
157208	cg0810130[illegible]	20654307	28717434	0.253865	0	1	0.1380037	-35.9946
157230	cg0810256[illegible]	29676374	29676374	0.5832511	0	1	0.3507328	-82.36449
157244	cg0810347[illegible]	27698360	27698360	0.1667331	0	1	0.2829968	32.85236
157308	cg0810637[illegible]	57799424	57799424	0.6337157	0	1	0.7547454	31.81048
157375	cg0810980[illegible]	54645374	54645374	0.5033714	0	1	0.4018361	-18.61339
157539	cg0811828[illegible]	33687506	63705377	0.8360828	0	1	0.6873968	-56.51527
157569	cg0811952[illegible]	29757498	29757498	0.6662713	0	1	0.8876209	126.3862
157613	cg0812140[illegible]	50683352	50683352	0.5769901	0	1	0.8242858	122.5841
157614	cg0812145[illegible]	29648478	69782310	0.7171095	0	1	0.5906408	-31.42239

157620 cg0812168(	67779397	67779397	0.454441	0	1	0.5579813	19.23178
157668 cg0812341:	47778399	47778399	0.1259625	0	1	0.3111877	77.86628
157723 cg0812582	74679390	74679390	0.1864904	0	1	0.3009533	30.26879
157764 cg0812826	12676359	12676359	0.6652054	0	1	0.8869808	126.4284
157885 cg0813366	65716403	65716403	0.3750424	0	1	0.5036352	27.75538
157886 cg0813369	66746321	66746321	0.4513209	0	1	0.6415713	57.17692
158020 cg0814011	17754498	17754498	0.3166531	0	1	0.4288426	23.08608
158088 cg0814304	39734330	39734330	0.7583328	0	1	0.9124593	85.02718
158152 cg0814670	24809380	58767470	0.9241979	0	1	0.718407	-134.1786
158156 cg0814686	26716431	26716431	0.9021776	0	1	0.5705329	-247.4586
158231 cg0815162:	67731429	47650332	0.3359508	0	1	0.224	-26.65697
158334 cg0815731(	34648300	34648300	0.1161208	0	1	0.242952	44.95237
158350 cg0815810	26747343	26747343	0.4805285	0	1	0.7655529	139.7232
158417 cg0816094	31700426	31700426	0.0182292	0	1	0.1566081	86.1452
158539 cg0816663	15660481	15660481	0.5829183	0	1	0.4063224	-48.61238
158606 cg0817029:	38600397	32703303	0.3642831	0	1	0.2614951	-21.66774
158646 cg0817210	29651467	29651467	0.2093445	0	1	0.3373784	34.10011
158651 cg0817247	43682494	43682494	0.5675597	0	1	0.3872365	-50.46397
158694 cg0817478	29778377	66723452	0.648998	0	1	0.5201566	-29.26649
158721 cg0817641(	41780301	41780301	0.6786843	0	1	0.4763577	-66.08575
158727 cg0817701	10682320	10682320	0.2697694	0	1	0.3765563	22.69628
158981 cg0819178	53774317	53774317	0.6812408	0	1	0.7856931	27.56233
159021 cg0819432:	26713357	26713357	0.4867583	0	1	0.374073	-22.2729
159060 cg0819598:	24722422	33772334	0.5868458	0	1	0.4751966	-21.99311
159156 cg0820054:	49726317	49726317	0.4670585	0	1	0.1758283	-148.9279
159160 cg0820062	13714477	49744305	0.3154812	0	1	0.4304278	24.04178
159214 cg0820346(	15742320	15742320	0.3843769	0	1	0.6329061	94.564
159265 cg0820609	64636505	64636505	0.4730451	0	1	0.6039075	28.91784
159389 cg0821286	13607487	13607487	0.8903419	0	1	0.7756007	-49.44648
159479 cg0821735	21657325	21657325	0.6552961	0	1	0.9512551	374.3439
159511 cg0821917(	23631406	23631406	0.0401664	0	1	0.1729856	70.59248
159514 cg0821921	10722412	37623347	0.8468269	0	1	0.951526	67.04122
159575 cg0822251:	65721447	29784479	0.4412928	0	1	0.2688529	-50.26077
159580 cg0822261	38686484	44694343	0.406462	0	1	0.252176	-42.58023
159747 cg0823169	38645482	71666438	0.7846342	0	1	0.5011969	-142.7912
159765 cg0823314	35780324	35780324	0.7590563	0	1	0.593694	-53.24065
159856 cg0823756	55635419	55635419	0.4804826	0	1	0.6158429	30.86109
159867 cg0823831	35661439	35661439	0.824931	0	1	0.6315804	-82.08709
159868 cg0823837	47615483	47615483	0.5420833	0	1	0.0520686	-371.3336
159886 cg0823969	56785368	50644420	0.5768607	0	1	0.4634635	-22.45782
159947 cg0824223	61655384	16797406	0.5860413	0	1	0.7531648	53.4739
159957 cg0824285	16673423	16673423	0.5289068	0	1	0.6344367	20.82306
159967 cg0824372	43786494	33657422	0.4485365	0	1	0.3227541	-27.67276

160103	cg08250118	44675342	44675342	0.6271629	0	1	0.214448	-295.6992
160175	cg08253809	62661502	62661502	0.216493	0	1	0.6379082	374.3439
160244	cg08258654	11782430	11782430	0.0506667	0	1	0.2055061	82.14428
160336	cg08263140	54700302	54700302	0.2986088	0	1	0.413599	24.52474
160385	cg08265392	51662354	51662354	0.2872509	0	1	0.4215587	32.09138
160464	cg08268692	68794349	68794349	0.6697163	0	1	0.7699409	24.69174
160489	cg08269974	27605303	16682363	0.4110267	0	1	0.1879704	-90.10209
160546	cg08272429	24608456	24608456	0.6190628	0	1	0.8863562	374.3439
160550	cg08272591	70605446	70605446	0.8438547	0	1	0.9573036	78.03999
160591	cg08274589	28666467	37803421	0.3764062	0	1	0.2345916	-38.16601
160756	cg08282819	42753354	42753354	0.2027964	0	1	0.3186589	29.56342
160821	cg08285768	34793433	34793433	0.3212919	0	1	0.4872897	44.53801
160891	cg08289272	23652432	23652432	0.3721999	0	1	0.7491265	374.3439
160970	cg08292959	70754447	36781418	0.3511442	0	1	0.5264274	48.25304
160972	cg08293002	20705346	20705346	0.2747902	0	1	0.4124361	33.99496
160978	cg08293242	47695457	43706315	0.8831775	0	1	0.7344519	-69.25495
160982	cg08293392	58736354	58736354	0.5661416	0	1	0.8093105	114.7149
161041	cg08296868	37627429	27764490	0.8515797	0	1	0.9558761	69.32859
161089	cg08299859	52694424	52694424	0.7268049	0	1	0.9682131	374.3439
161100	cg08300475	10660302	10660302	0.9510549	0	1	0.7223728	-176.4181
161196	cg08305942	20600451	20600451	0.5736362	0	1	0.7525421	59.851
161231	cg08308214	44803398	44803398	0.9392874	0	1	0.8361272	-59.35726
161254	cg08309687	50726380	50726380	0.3375191	0	1	0.4636754	27.46282
161311	cg08312765	63701438	63701438	0.4332794	0	1	0.568503	30.13568
161383	cg08316699	12625334	12625334	0.448076	0	1	0.5483056	18.20646
161404	cg08317694	40661481	58673376	0.3010083	0	1	0.2000512	-24.0254
161435	cg08319103	33739454	52676471	0.3676842	0	1	0.4763572	21.05796
161691	cg08333400	22642441	22642441	0.5209359	0	1	0.3895032	-28.68314
161720	cg08335008	54613424	69684370	0.8934143	0	1	0.7831979	-47.5032
161917	cg08348121	70650432	41667361	0.5761405	0	1	0.475657	-18.44573
162040	cg08355045	52778306	52778306	0.6957594	0	1	0.8401001	54.83845
162043	cg08355157	52777483	52777483	0.367596	0	1	0.2106378	-47.35567
162054	cg08355456	49704335	42675320	0.3305451	0	1	0.1792151	-48.0901
162090	cg08357436	14790391	62719414	0.678686	0	1	0.5460451	-31.86807
162141	cg08360511	36755457	30758505	0.1468022	0	1	0.2593333	33.35671
162257	cg08367801	35601365	35601365	0.574309	0	1	0.7494455	57.37041
162275	cg08368875	46750400	46750400	0.2040234	0	1	0.3056329	24.02006
162362	cg08373528	57676503	38676483	0.1674856	0	1	0.2683154	26.32463
162366	cg08373622	71729412	71729412	0.3250417	0	1	0.4982589	47.90746
162370	cg08373989	65751422	65751422	0.8173308	0	1	0.9448684	81.7797
162455	cg08378932	65704464	65704464	0.5507699	0	1	0.7055823	42.96406
162495	cg08381046	64661310	64661310	0.2662478	0	1	0.3720835	22.50609
162501	cg08381537	45777500	32712359	0.8349286	0	1	0.6668861	-68.01244

162636	cg08390209	16629508	16629508	0.6111504	0	1	0.4623588	-36.19107
162677	cg08392746	41786390	41786390	0.5076207	0	1	0.6702995	44.50493
162721	cg08395748	63799460	63799460	0.3388141	0	1	0.45129	22.70853
162722	cg08395784	42682355	42682355	0.6936617	0	1	0.8356495	52.56507
162723	cg08395793	70795332	34745451	0.3878398	0	1	0.2561163	-32.53844
162755	cg08397993	71613387	71613387	0.1529159	0	1	0.4060536	121.0726
162809	cg08401725	14735409	14735409	0.3958159	0	1	0.5273632	28.69123
162815	cg08401938	24802413	60775315	0.6869977	0	1	0.8147354	41.36852
162847	cg08404009	43785333	43785333	0.217813	0	1	0.1101681	-36.01071
162858	cg08404513	10750439	10750439	0.1679989	0	1	0.2957181	37.89486
162970	cg08412215	41660507	41660507	0.2352186	0	1	0.3373999	22.56009
163093	cg08419450	65690351	65690351	0.6555871	0	1	0.7618137	26.41089
163120	cg08421351	30674480	30674480	0.4819859	0	1	0.641538	41.78784
163134	cg08422420	47792331	14673478	0.3617313	0	1	0.1367715	-103.2045
163206	cg08426157	32668320	55698420	0.1326006	0	1	0.2391228	32.32499
163255	cg08429705	51699499	51699499	0.5505616	0	1	0.6591185	22.41317
163263	cg08430277	70781380	70781380	0.683938	0	1	0.8928223	118.5629
163297	cg08431931	62738320	38635408	0.3097551	0	1	0.1848079	-34.89292
163316	cg08433110	15624481	15624481	0.8470139	0	1	0.7088457	-52.74929
163413	cg08439880	23712406	17785323	0.7993295	0	1	0.6060874	-76.134
163460	cg08442052	52701303	52701303	0.4193655	0	1	0.5266932	20.32502
163481	cg08443563	54652399	54652399	0.6395811	0	1	0.7561741	30.09832
163533	cg08447044	55731332	55731332	0.5192577	0	1	0.6229188	20.01352
163586	cg08449138	33641400	33641400	0.9242579	0	1	0.818987	-54.4053
163593	cg08449558	39708435	39708435	0.8735501	0	1	0.7362522	-58.80029
163685	cg08454015	10677480	10677480	0.6826198	0	1	0.825673	51.24271
163805	cg08460078	54647413	54647413	0.3259273	0	1	0.5457079	74.32734
163850	cg08461772	65755313	65755313	0.5397853	0	1	0.3617912	-49.44389
163936	cg08465120	54709363	54709363	0.2111066	0	1	0.1109957	-32.19878
164036	cg08470157	41736488	59769377	0.3314852	0	1	0.1328017	-85.23451
164158	cg08476485	66810458	66810458	0.576548	0	1	0.8139461	111.4181
164168	cg08476984	12689487	12689487	0.4892699	0	1	0.5932684	19.67095
164258	cg08481002	13800314	13800314	0.0933234	0	1	0.2853926	92.51911
164326	cg08484992	56696315	56696315	0.5209221	0	1	0.6995172	54.35192
164431	cg08491487	35789426	50652323	0.0198527	0	1	0.1241001	58.59933
164498	cg08494843	49659388	49659388	0.6753677	0	1	0.8247396	54.61556
164509	cg08495239	12625354	24649304	0.7048984	0	1	0.5740604	-32.47111
164579	cg08498719	12646452	12646452	0.163922	0	1	0.2885159	36.90763
164597	cg08499861	71658448	71658448	0.0739824	0	1	0.1820693	43.25249
164603	cg08500138	22635426	22635426	0.3478611	0	1	0.4565468	21.3298
164665	cg08504662	47748432	47748432	0.52685	0	1	0.3126611	-71.2748
164700	cg08506606	25649385	25649385	0.796208	0	1	0.6531757	-46.46873
164702	cg08506672	22731330	22731330	0.2277635	0	1	0.336197	25.14624

164765	cg08510234	26625348	26625348	0.1882924	0	1	0.3055301	31.27019
164817	cg08514194	72705460	72705460	0.9338599	0	1	0.6652828	-201.1628
164827	cg08514594	26704441	26704441	0.6226327	0	1	0.724568	22.6659
164861	cg08516507	74769420	31620311	0.8391353	0	1	0.7273204	-37.08321
164895	cg08519385	40635303	40635303	0.4908264	0	1	0.6386597	36.52955
164958	cg08522473	53649408	53649408	0.5105103	0	1	0.6960643	57.82872
165066	cg08528516	50744305	50744305	0.3362347	0	1	0.0057803	-285.3784
165184	cg08534016	31770413	31770413	0.4213873	0	1	0.5302418	20.80279
165198	cg08534830	72715472	72715472	0.4423249	0	1	0.548735	20.06709
165372	cg08544331	36602436	36602436	0.2790386	0	1	0.3934163	24.96462
165382	cg08545129	49601481	35603438	0.4182189	0	1	0.2975518	-26.55601
165438	cg08548044	70708459	57646326	0.6202254	0	1	0.7392963	29.88383
165470	cg08549335	25671469	25671469	0.3035304	0	1	0.4856754	53.30183
165584	cg08554554	30790360	30790360	0.2919155	0	1	0.4245808	31.25752
165605	cg08555866	41732344	67769494	0.4589783	0	1	0.327523	-29.65977
165611	cg08556511	73691397	73691397	0.2230005	0	1	0.3344684	26.53417
165764	cg08568145	46628382	46628382	0.9020637	0	1	0.7792975	-58.2679
165798	cg08570077	41775416	41775416	0.8861473	0	1	0.766003	-51.62368
165801	cg08570243	44686396	44686396	0.3194541	0	1	0.4261431	21.23076
165867	cg08573687	50731446	73728429	0.3224545	0	1	0.4409716	25.09486
165896	cg08575621	17797489	42789326	0.1426659	0	1	0.0275519	-63.19968
165991	cg08579577	36793409	64755341	0.6328108	0	1	0.4994132	-30.52113
166008	cg08580224	21671300	73718316	0.6017116	0	1	0.7717276	57.43339
166023	cg08580942	46631351	46631351	0.3712543	0	1	0.259092	-24.97407
166164	cg08590939	22698447	22698447	0.2706348	0	1	0.1085425	-67.32371
166204	cg08593141	34654499	34654499	0.1388917	0	1	0.0260417	-62.2038
166220	cg08594246	23637373	23637373	0.65649	0	1	0.7891831	40.41285
166272	cg08597142	12766306	12766306	0.8779361	0	1	0.7181674	-75.00363
166322	cg08599792	56784382	10679329	0.345558	0	1	0.2451803	-21.51066
166330	cg08600378	27601382	27601382	0.7131137	0	1	0.8255851	35.43004
166342	cg08601628	40606444	64681421	0.6098142	0	1	0.4771032	-29.72241
166359	cg08602346	59802479	59802479	0.643197	0	1	0.5278468	-24.25435
166378	cg08603678	74712324	74712324	0.6481928	0	1	0.2764684	-226.2648
166414	cg08605899	73641389	73641389	0.5014809	0	1	0.0887933	-340.2166
166487	cg08610426	44703481	34722474	0.4788939	0	1	0.2846424	-60.91383
166521	cg08611810	14733362	65701446	0.2625514	0	1	0.3639502	21.17253
166553	cg08613577	38600454	38600454	0.3708372	0	1	0.2510585	-28.13394
166596	cg08616111	28786359	28786359	0.357944	0	1	0.4827718	26.61458
166615	cg08617160	16688382	27638409	0.5983524	0	1	0.4077921	-56.15481
166645	cg08619295	58688430	58688430	0.4822907	0	1	0.6049896	25.97444
166707	cg08621926	17740470	17740470	0.2049059	0	1	0.0918702	-42.12975
166708	cg08621957	31758403	31758403	0.4867662	0	1	0.5977464	21.914
166712	cg08622198	36738381	36738381	0.4954726	0	1	0.6182631	26.25579

166769	cg08624915	55739509	55739509	0.6061144	0	1	0.713865	24.18776
166808	cg08626646	26754487	20803365	0.5520323	0	1	0.4091966	-33.08308
166866	cg08629394	27702369	27702369	0.4187686	0	1	0.2366138	-57.96794
166905	cg08632164	30708378	47671432	0.2605713	0	1	0.1054625	-63.71534
166926	cg08633479	67714503	57641490	0.7343004	0	1	0.5563427	-57.20208
166942	cg08634229	56607509	74768387	0.6162201	0	1	0.423697	-57.59128
166968	cg08635406	52656319	52656319	0.6810945	0	1	0.7821164	25.90233
167021	cg08638632	56799350	56799350	0.453342	0	1	0.5812154	27.49803
167062	cg08640901	44605456	44605456	0.7115362	0	1	0.6048208	-23.71591
167133	cg08643994	15644504	38770333	0.1981669	0	1	0.0907821	-39.26753
167345	cg08655953	34747398	53704486	0.6621757	0	1	0.7736731	29.41656
167356	cg08656770	52684461	49781466	0.5964491	0	1	0.4911463	-20.10942
167363	cg08657228	67723380	67723380	0.579806	0	1	0.4788806	-18.60809
167433	cg08660971	45613376	45613376	0.4142652	0	1	0.5510772	30.67802
167449	cg08661678	73644489	73644489	0.7758573	0	1	0.9170396	77.44259
167564	cg08669168	33740361	33740361	0.9421169	0	1	0.7444332	-139.9831
167618	cg08673225	10730369	10730369	0.2977825	0	1	0.1111111	-82.92038
167640	cg08675117	65772402	65772402	0.5603033	0	1	0.4235799	-30.66994
167720	cg08679971	33602320	31609499	0.5919006	0	1	0.4789327	-22.48129
167753	cg08681473	36789373	36789373	0.2966128	0	1	0.4088916	23.65816
167782	cg08682981	35702373	35702373	0.3817978	0	1	0.2756213	-22.28601
167959	cg08692676	73808406	73808406	0.1964184	0	1	0.347211	45.78951
167967	cg08693140	63669365	63669365	0.5346779	0	1	0.6640779	30.00229
167968	cg08693172	40800304	40800304	0.4009558	0	1	0.2425653	-45.22002
168049	cg08697163	11621437	11621437	0.5541505	0	1	0.6860381	31.90986
168059	cg08697732	33712307	33712307	0.2627137	0	1	0.3761776	25.28263
168063	cg08698010	58624364	58624364		0	1	0.1316801	4.34E-09
168087	cg08698943	23600491	23600491	0.3541684	0	1	0.589639	84.42652
168088	cg08698997	28655508	44682395	0.6107435	0	1	0.7642152	47.68039
168178	cg08703522	69614440	66759488	0.2088311	0	1	0.3549026	42.24659
168192	cg08704154	41716415	41716415	0.6585505	0	1	0.8232947	63.34044
168202	cg08704562	26630392	14673368	0.3260767	0	1	0.219552	-24.90395
168264	cg08708231	26660462	26660462	0.7472957	0	1	0.8843607	61.88085
168370	cg08714433	62790489	69754401	0.7562539	0	1	0.5132127	-102.6377
168409	cg08716255	20732476	20732476	0.7091486	0	1	0.818189	32.86744
168478	cg08720028	69803482	44618480	0.1014576	0	1	0.3046428	97.89915
168529	cg08723131	54643455	54643455		0	1	0.0674847	4.34E-09
168544	cg08723977	72721356	72721356	0.7033581	0	1	0.8091391	30.37
168567	cg08725366	64717485	64717485	0.116929	0	1	0.2254507	35.40794
168590	cg08726801	26735476	62677324	0.3261904	0	1	0.2072065	-30.4996
168895	cg08743751	19709509	19709509	0.4588658	0	1	0.3552237	-19.65475
168925	cg08745965	36726498	36726498	0.5198793	0	1	0.3813346	-31.47268
169000	cg08750439	31749440	31749440	0.6403495	0	1	0.5184133	-26.47841

169024	cg0875184	37652351	37652351	0.6126353	0	1	0.853043	127.0809
169026	cg0875187	66702311	66702311	0.3615352	0	1	0.5697774	66.36089
169071	cg0875472	53774390	57711316	0.7052496	0	1	0.9035379	115.3788
169098	cg0875659	11755499	11755499	0.2634603	0	1	0.3675413	22.02339
169207	cg0876242	72782327	44675452	0.2661629	0	1	0.1136323	-60.14341
169212	cg0876260	64718325	57715309	0.6672922	0	1	0.5575141	-23.06589
169276	cg0876676	41769319	41769319	0.5774102	0	1	0.6794798	20.921
169290	cg0876768	26708436	54725351	0.9166197	0	1	0.7711518	-80.46941
169295	cg0876782	64622436	17761482	0.351326	0	1	0.1961667	-48.00384
169400	cg0877513	13644397	10761385		0	1		-4.34E-09
169447	cg0877859	20629338	18760384	0.4691324	0	1	0.1960893	-127.7329
169591	cg0878762	68644470	20784419	0.1151444	0	1	0.0072844	-67.86231
169600	cg0878798	69718393	69718393	0.1875841	0	1	0.2886009	24.86798
169616	cg0878910	60601499	47658449	0.5411084	0	1	0.6498514	22.21997
169794	cg0880003	55608402	55608402	0.6199999	0	1	0.4966289	-26.49846
169879	cg0880524	36619474	36619474	0.2516243	0	1	0.3580723	23.29644
169908	cg0880668	72794300	58644482	0.5033822	0	1	0.2899139	-72.01818
169921	cg0880804	57720364	19705326	0.5273578	0	1	0.3205138	-66.45077
169991	cg0881210	49736318	70704466	0.5585554	0	1	0.6808471	27.98397
170025	cg0881402	10723353	10723353	0.4898458	0	1	0.6334146	34.58541
170092	cg0881748	32807411	31629403	0.4268393	0	1	0.2824118	-36.46492
170123	cg0881882	12738459	62639458	0.3880298	0	1	0.5169967	27.77899
170128	cg0881902	64789365	34786465	0.8851324	0	1	0.7450277	-64.13069
170152	cg0882078	37658307	37658307	0.2750175	0	1	0.1185957	-61.35696
170215	cg0882335	50670510	63789405	0.3067807	0	1	0.0425617	-181.1113
170363	cg0883107	43701438	50623425	0.5166517	0	1	0.6762649	43.38257
170393	cg0883260	22730507	24800396	0.2940853	0	1	0.1780523	-31.6629
170490	cg0883748	46643471	46643471	0.5651588	0	1	0.6790565	24.88245
170533	cg0883980	50807329	42772412	0.6001059	0	1	0.8352727	116.2377
170542	cg0884044	53646483	46678500	0.6674201	0	1	0.5188331	-37.97564
170575	cg0884244	73622480	73622480	0.3330327	0	1	0.469516	31.45643
170726	cg0884962	21611369	21611369	0.7931623	0	1	0.6708576	-36.00357
170817	cg0885483	32621455	32621455	0.5130242	0	1	0.6288685	24.07535
170850	cg0885631	28731361	28731361	0.0903643	0	1	0.2770661	89.65047
170887	cg0885771	66670482	66670482	0.3695879	0	1	0.492997	25.9541
170907	cg0885829	54657471	54657471	0.4468091	0	1	0.5591272	21.97906
170916	cg0885854	11782329	40764344	0.6082141	0	1	0.5030541	-20.2301
170933	cg0885921	24711352	24711352	0.5328096	0	1	0.6336004	19.3394
170975	cg0886143	43725335	30676486	0.3036144	0	1	0.5191234	72.52983
171035	cg0886434	47644483	47644483	0.6505757	0	1	0.5079613	-34.75739
171073	cg0886666	29655384	52639411	0.4935813	0	1	0.6054059	22.29928
171074	cg0886669	30707379	30707379	0.2029244	0	1	0.3370951	37.31868
171081	cg0886699	25793447	61788475	0.317312	0	1	0.4437099	28.04331

171141	cg0887003(	13778435	13778435	0.2113656	0	1	0.3836516	55.23022
171143	cg0887009:	44761460	44761460	0.2556939	0	1	0.3852595	31.69403
171150	cg0887064(	66725454	51730363	0.3422841	0	1	0.2368865	-23.59789
171165	cg0887123(	15681417	15681417	0.4486316	0	1	0.5804207	28.93624
171233	cg0887394(	33689432	70689423	0.743366	0	1	0.6378047	-24.96587
171361	cg0887984(	19624427	19624427	0.877722	0	1	0.6380454	-137.3138
171534	cg0888820:	16725414	55726318	0.7433766	0	1	0.6367199	-25.37114
171550	cg0888891{	24794482	24794482	0.4604765	0	1	0.583588	25.81949
171556	cg0888911<	12723366	12723366	0.6708527	0	1	0.7838081	30.91859
171632	cg0889289(	44664353	44664353	0.3673443	0	1	0.2603493	-23.12484
171634	cg0889303(	34672391	34672391	0.4618217	0	1	0.583865	25.44839
171648	cg0889381'	32715460	32715460	0.4023581	0	1	0.5371838	29.91279
171744	cg0889886(	31694463	31694463	0.5160125	0	1	0.6344743	25.10412
171899	cg0890630(	45651328	45651328	0.4492163	0	1	0.5607098	21.72187
171972	cg0891185:	23691310	23691310	0.2135559	0	1	0.3549752	39.65863
171993	cg0891352:	23809314	23809314	0.2222248	0	1	0.3336511	26.56397
172038	cg0891683(	34624375	13683355	0.441122	0	1	0.6951228	102.6607
172101	cg0892045{	34635319	43616387	0.9522975	0	1	0.6424561	-262.9484
172149	cg0892359<	37730375	37730375	0.5188257	0	1	0.674565	41.50566
172153	cg0892366(	53698352	16617450	0.0948589	0	1	0.2206318	48.66361
172156	cg0892402:	58652340	58652340	0.5850517	0	1	0.7996452	90.66657
172266	cg0893090<	32738498	66800498	0.1891645	0	1	0.3621121	58.35657
172314	cg0893328<	74759488	74759488	0.6223224	0	1	0.7311918	25.48088
172412	cg0893879:	40803324	40803324	0.5977814	0	1	0.3419102	-99.88429
172484	cg0894252'	72750374	72750374	0.6292511	0	1	0.7437918	28.39865
172518	cg0894417(	61667441	61667441	0.2069995	0	1	0.3727133	52.22459
172551	cg0894580:	30799426	30799426	0.7758877	0	1	0.8925386	51.26611
172761	cg0895918:	49644505	49644505	0.4661785	0	1	0.5837877	23.92573
172778	cg0896054(	30662428	30662428	0.6567084	0	1	0.7643871	27.15966
172826	cg0896301:	43682431	43682431	0.7266797	0	1	0.3391154	-253.3111
172845	cg0896473(	40751468	40751468	0.0482564	0	1	0.3008485	374.3439
172895	cg0896758<	38708324	38708324	0.7187826	0	1	0.3899455	-175.7379
173001	cg0897321'	53746316	53746316	0.7089574	0	1	0.8549773	59.27909
173042	cg0897583<	58620476	58620476	0.9346009	0	1	0.6831127	-184.5269
173074	cg0897731	31717420	31717420	0.2496905	0	1	0.8059332	374.3439
173086	cg0897793	50694496	50694496	0.288382	0	1	0.4313738	35.5872
173205	cg0898557(	29724312	29724312	0.6056124	0	1	0.4421931	-42.55631
173213	cg0898597(	11618323	11618323	0.3147985	0	1	0.4244324	22.28819
173301	cg0899222(	64715381	64715381	0.259542	0	1	0.3844528	29.67017
173338	cg0899408:	62731396	62731396	0.5367939	0	1	0.6468371	22.56867
173347	cg0899489	60713466	19662452	0.3357445	0	1	0.2241249	-26.5256
173373	cg0899659'	14613307	14613307	0.6233762	0	1	0.8583409	124.6239
173381	cg0899680:	29671410	29671410	0.1756385	0	1	0.3150711	42.58471

173526	cg0900681(	68781379	68781379	0.4453638	0	1	0.3435344	-19.2749
173636	cg0901241l	47722454	47722454	0.2950757	0	1	0.1924082	-25.1597
173678	cg0901480l	46749397	46749397	0.7411058	0	1	0.5524853	-63.99528
173682	cg0901523	65740305	65740305	0.7249683	0	1	0.6221981	-22.97489
173723	cg0901792	40738312	63803425	0.1977784	0	1	0.3252214	34.80586
173844	cg0902517	52707318	52707318	0.8633522	0	1	0.7240856	-57.27165
173919	cg0902990	43605483	43605483	0.4874037	0	1	0.5930554	20.17467
174081	cg0903956l	31788448	31788448	0.462234	0	1	0.5628427	18.3867
174104	cg0904099	16652505	16652505	0.5867714	0	1	0.7722996	66.47557
174222	cg0904773	18789360	20681432	0.7239047	0	1	0.6209677	-22.9812
174270	cg0905083	39669307	39669307	0.6749867	0	1	0.5133877	-44.24471
174277	cg0905134	70723444	70723444	0.4285501	0	1	0.6349089	66.14703
174289	cg0905218	30675395	30675395	0.4473975	0	1	0.8181534	374.3439
174302	cg0905324	48664509	48664509	0.6949756	0	1	0.5925711	-21.56765
174305	cg0905353(	13687487	13687487	0.8016937	0	1	0.9060659	47.50165
174497	cg0906562	56721316	48793480	0.2549883	0	1	0.3616419	23.22562
174564	cg0906944(	23607486	61723462	0.4569018	0	1	0.6011899	34.12292
174576	cg0907019l	69715359	69715359	0.3522551	0	1	0.4600466	20.98049
174631	cg0907334	62617399	62617399	0.4722117	0	1	0.2409397	-88.04536
174670	cg0907555	44691391	55726507	0.4426804	0	1	0.3258347	-24.44012
174675	cg0907584	15775384	15775384	0.607035	0	1	0.4895266	-24.21369
174735	cg0907988	29693349	29693349	0.3660031	0	1	0.4811855	23.18322
174750	cg0908078	25733472	25733472	0.6242965	0	1	0.9390052	374.3439
174775	cg0908251	65781502	65781502	0.7101709	0	1	0.9331223	156.5356
174800	cg0908424	53720344	53720344	0.1998204	0	1	0.3712314	56.11899
174805	cg0908439l	26694311	30630454	0.6229621	0	1	0.5117054	-22.4244
174869	cg0908815	18651434	18651434	0.1087831	0	1	0.2373659	47.30885
174917	cg0909118	45778436	58766345	0.6825773	0	1	0.5301105	-40.45372
175061	cg0910027l	65753302	65753302	0.6453886	0	1	0.5214995	-27.32369
175062	cg0910033	34641304	16627472	0.5310171	0	1	0.3525566	-49.83025
175071	cg0910106	50699371	50699371	0.7420939	0	1	0.9299841	122.5216
175085	cg0910203(	50621483	50621483	0.7261704	0	1	0.8819749	73.67636
175119	cg0910402	44752306	44752306	0.6353878	0	1	0.5097847	-27.66943
175169	cg0910743	71729464	43641419	0.7061759	0	1	0.8131029	31.31369
175272	cg0911500	43694400	43694400	0.471864	0	1	0.6241601	38.00843
175305	cg0911744	10664468	25779417	0.3632743	0	1	0.2207401	-39.55891
175341	cg0911968	14679432	14679432	0.5545091	0	1	0.6562318	20.1405
175484	cg0912627	46810317	46810317	0.3417407	0	1	0.5332644	56.98519
175532	cg0912906	63697332	24746334	0.2794336	0	1	0.4096579	30.8138
175661	cg0913565(	31614324	10734365	0.638662	0	1	0.3999766	-87.51292
175698	cg0913820	34645343	34645343	0.4114006	0	1	0.5832729	46.26031
175734	cg0914027	72623414	72623414	0.5999129	0	1	0.7056094	23.10089
175874	cg0914706	14689356	14689356	0.148135	0	1	0.267674	36.43332

175908	cg0914885[illegible]	49790464	49790464	0.6922365	0	1	0.8819519	98.3005
176077	cg0915801[illegible]	33693506	33693506	0.4978822	0	1	0.2064249	-142.3941
176164	cg0916300[illegible]	61695508	61695508	0.3620392	0	1	0.2592994	-21.7343
176222	cg0916641[illegible]	46645488	46645488	0.5843428	0	1	0.7268875	38.91755
176225	cg0916653[illegible]	23692396	23692396	0.5822021	0	1	0.4758216	-20.2799
176259	cg0916872[illegible]	39751484	39751484	0.4406474	0	1	0.6748803	86.36989
176384	cg0917481[illegible]	34733453	34733453	0.5119133	0	1	0.612632	18.94766
176526	cg0918208[illegible]	37645409	37645409	0.3602442	0	1	0.5514979	56.49776
176623	cg0918750[illegible]	18689460	24679402	0.7604192	0	1	0.6464732	-29.35141
176911	cg0920874[illegible]	38683434	42741493	0.1784315	0	1	0.3234154	44.98112
176974	cg0921417[illegible]	21745343	21745343	0.6101031	0	1	0.3828999	-78.76447
177116	cg0922236[illegible]	29764455	29764455	0.4727685	0	1	0.5810839	20.87144
177127	cg0922289[illegible]	35723454	35723454	0.4863149	0	1	0.7231948	93.04712
177161	cg0922537[illegible]	29799387	29799387	0.266534	0	1	0.4434576	52.71833
177214	cg0922766[illegible]	14777384	14777384	0.8267264	0	1	0.678144	-54.55079
177276	cg0923117[illegible]	55751311	55751311	0.2143734	0	1	0.1116858	-33.33986
177350	cg0923445[illegible]	34761355	47705509	0.7376238	0	1	0.8417609	33.70439
177382	cg0923556[illegible]	57682351	15675506	0.7877218	0	1	0.6625323	-36.72071
177458	cg0923970[illegible]	50684442	50684442	0.5510408	0	1	0.6539586	20.45703
177551	cg0924500[illegible]	53729338	53729338	0.9009416	0	1	0.7859304	-52.77589
177616	cg0924797[illegible]	28668413	28668413	0.6338316	0	1	0.7756608	43.22834
177845	cg0925887[illegible]	14635367	14635367	0.2165145	0	1	0.3278112	26.85868
177903	cg0926179[illegible]	39749313	39749313	0.305135	0	1	0.5200583	72.08475
177987	cg0926614[illegible]	54763332	54763332	0.5690172	0	1	0.7112554	37.66446
178033	cg0926871[illegible]	66806458	13759348	0.2516864	0	1	0.3840139	33.03014
178077	cg0927086[illegible]	67639301	67639301	0.067822	0	1		-4.34E-09
178219	cg0927869[illegible]	52623414	52623414	0.3188208	0	1	0.4595697	33.54047
178242	cg0927973[illegible]	20706454	15790347	0.0679777	0	1	0.3604961	374.3439
178302	cg0928420[illegible]	49729377	49729377	0.8066376	0	1	0.6375997	-62.57341
178316	cg0928494[illegible]	32618445	50615413	0.4797885	0	1	0.3380983	-33.40384
178347	cg0928688[illegible]	73669337	73669337	0.512529	0	1	0.6139001	19.16317
178441	cg0929266[illegible]	47722311	47722311	0.3098127	0	1	0.4133719	20.46196
178476	cg0929415[illegible]	14668374	35739312	0.2686144	0	1	0.1634989	-28.41269
178477	cg0929424[illegible]	21626310	21626310	0.4632969	0	1	0.5663173	19.12501
178507	cg0929645[illegible]	49654360	35808326	0.0568209	0	1	0.20803	76.86203
178593	cg0930149[illegible]	49688431	49688431	0.4554175	0	1	0.555796	18.28106
178693	cg0930719[illegible]	26614375	26614375	0.5006491	0	1	0.3732935	-27.32334
178739	cg0930926[illegible]	14719371	14719371	0.4380485	0	1	0.3355562	-19.60705
178789	cg0931289[illegible]	52654394	52654394	0.534934	0	1	0.7813733	110.3068
178890	cg0931767[illegible]	46637510	46637510	0.5071033	0	1	0.6625476	40.78531
178993	cg0932225[illegible]	56681355	27673401	0.5242091	0	1	0.4177822	-20.04711
179016	cg0932340[illegible]	54606301	54606301		0	1		-4.34E-09
179021	cg0932372[illegible]	42679433	10738438	0.2653106	0	1	0.0913646	-80.24617

179035	cg0932411	55750413	55750413	0.1960625	0	1	0.0847399	-42.67231
179448	cg0934961	47637471	47637471	0.591204	0	1	0.693631	21.52525
179465	cg0935089	67715351	54602303	0.2932147	0	1	0.1685856	-36.40353
179494	cg0935233	36766335	36766335	0.3683144	0	1	0.5116568	33.52905
179496	cg0935251	49644447	49644447	0.7126532	0	1	0.512118	-67.88604
179539	cg0935455	24636339	62621337	0.7789991	0	1	0.9558817	132.6439
179596	cg0935776	61605474	61605474	0.6207642	0	1	0.7425843	31.22059
179623	cg0935911	46760494	24619494	0.4472842	0	1	0.3218369	-27.57427
179641	cg0936004	50648414	50648414	0.6656801	0	1	0.8018059	43.73949
179668	cg0936149	64613328	49665304	0.4486147	0	1	0.2560323	-62.05223
179678	cg0936196	16705402	23736493	0.8996255	0	1	0.7553259	-72.20609
179761	cg0936651	38653371	38653371	0.4100685	0	1	0.5832218	46.89156
179912	cg0937351	45691334	45691334	0.2548008	0	1	0.3595076	22.5697
179941	cg0937506	51652388	51652388	0.9331796	0	1	0.8195656	-64.38776
179985	cg0937828	47684491	47684491	0.5157493	0	1	0.7076378	62.34765
180016	cg0938006	55661463	55661463	0.7042555	0	1	0.8093061	30.06936
180127	cg0938748	42803315	72613508	0.4090157	0	1	0.2524303	-43.63806
180206	cg0939291	27725506	27725506	0.926664	0	1	0.8177193	-58.01007
180238	cg0939519	68777485	68777485	0.3519962	0	1	0.4778285	27.0701
180320	cg0939943	37793317	37793317	0.1677982	0	1	0.2791767	30.63932
180414	cg0940621	73790463	73790463		0	1		-4.34E-09
180457	cg0940819	20795464	20795464	0.093373	0	1	0.2055179	41.33955
180508	cg0941051	48782467	48782467	0.2945387	0	1	0.479352	55.15993
180538	cg0941191	53774381	53774381	0.6343389	0	1	0.8467322	102.2494
180565	cg0941325	20802377	20802377	0.6821866	0	1	0.8222267	48.97311
180576	cg0941394	35711450	35711450	0.9003445	0	1	0.7878276	-50.9768
180601	cg0941494	63742393	46678324	0.2220872	0	1	0.3357355	27.41197
180625	cg0941618	13620337	13620337	0.3172721	0	1	0.5528352	85.28867
180645	cg0941703	69626417	69626417	0.5152205	0	1	0.3341304	-51.6118
180693	cg0941925	61797307	61797307	0.4702044	0	1	0.575819	19.98478
180699	cg0941967	15624446	15624446	0.2089775	0	1	0.0861501	-48.96566
180782	cg0942516	70661382	70661382	0.6269778	0	1	0.5145121	-22.90345
180807	cg0942701	14785328	14785328	0.5812104	0	1	0.7301581	42.1095
180872	cg0943064	69676349	69676349	0.8371281	0	1	0.7059276	-46.85369
180942	cg0943460	38809442	38809442	0.3932112	0	1	0.5190409	26.60713
180953	cg0943517	59669343	59669343	0.478694	0	1	0.5892348	21.66461
181001	cg0943747	22650352	45648320	0.1402884	0	1	0.2482293	32.04155
181010	cg0943806	12618375	12618375	0.8205082	0	1	0.6535293	-64.10413
181095	cg0944245	27751347	69745434	0.1840146	0	1	0.5477698	374.3439
181104	cg0944279	22708472	22708472	0.6013082	0	1	0.7123479	25.2886
181265	cg0945121	17618421	17618421	0.3296444	0	1	0.4574502	28.24396
181343	cg0945588	50789347	50789347	0.573104	0	1	0.71881	39.73333
181461	cg0946228	35725439	46723333	0.6414244	0	1	0.9152712	374.3439

181553	cg09467607	22690379	22690379	0.157091	0	1	0.3252039	60.58217
181751	cg09478002	17627420	17627420	0.3268988	0	1	0.452129	27.36827
181891	cg09487575	43673449	43673449	0.4783346	0	1	0.6062667	27.87619
181954	cg09491897	52670370	62692490	0.8568454	0	1	0.7509024	-37.01231
182033	cg09496634	23706474	23706474	0.2126886	0	1	0.3182282	24.94025
182121	cg09501687	31735424	31735424	0.5093102	0	1	0.6948875	57.75003
182257	cg09508496	10692335	13645385	0.2728151	0	1	0.1295712	-51.44361
182308	cg09510698	21692351	21692351	0.7076231	0	1	0.8126894	30.44362
182403	cg09516200	19782308	19782308	0.6860366	0	1	0.792096	28.78951
182549	cg09524613	48745352	48745352	0.3180727	0	1	0.4355897	24.8568
182683	cg09532840	50750421	50750421	0.4571919	0	1	0.5924933	30.41825
182703	cg09533869	19733415	19733415	0.1642961	0	1	0.0318124	-74.19953
182706	cg09534232	66770392	66770392	0.672105	0	1	0.9091468	151.7644
182714	cg09535226	32765492	32765492	0.609586	0	1	0.7363603	32.81319
183178	cg09560078	22606468	13648395	0.2299967	0	1	0.0415629	-113.1558
183188	cg09560533	57647300	57647300	0.2790385	0	1	0.4334581	41.01914
183454	cg09576209	74797377	74797377	0.6755686	0	1	0.5684205	-22.45518
183581	cg09581911	74718307	74718307	0.508298	0	1	0.3877958	-24.76791
183601	cg09583302	46605357	46605357	0.6306406	0	1	0.7430556	27.52629
183627	cg09584711	41796336	41796336	0.4779373	0	1	0.5832113	19.94949
183724	cg09592244	53629317	53629317	0.0921259	0	1	0.2191183	49.99501
183801	cg09597022	10787338	42678415	0.5778665	0	1	0.7017497	29.55114
183808	cg09597400	23622386	31801457	0.5355945	0	1	0.4286543	-20.20368
183901	cg09603707	68689333	68689333	0.2211661	0	1	0.4553385	92.63416
183908	cg09603919	51701470	51701470	0.2225964	0	1	0.3910253	51.99865
184043	cg09611620	29737300	29737300	0.6208648	0	1	0.7711366	46.88228
184138	cg09616536	50800424	50800424	0.8921114	0	1	0.5918748	-204.6371
184191	cg09619488	13684495	48642443	0.7056608	0	1	0.9695261	374.3439
184193	cg09619624	68746346	37790478	0.4580032	0	1	0.2870935	-48.4037
184246	cg09623286	56608399	65619429	0.2078104	0	1	0.0653593	-67.48984
184347	cg09630417	40758440	40758440	0.3789285	0	1	0.5062954	27.2697
184351	cg09630706	20687478	18721434	0.3210278	0	1	0.4307062	22.15811
184371	cg09632136	35642329	35642329	0.2783172	0	1	0.4244795	37.40643
184452	cg09636756	53767385	53767385	0.6907277	0	1	0.3994482	-134.0059
184506	cg09639931	31687307	58640502	0.3289524	0	1	0.228089	-22.44559
184513	cg09640425	21705366	40779491	0.6808748	0	1	0.5687845	-24.31976
184598	cg09644806	51682447	51682447	0.5913343	0	1	0.9008769	374.3439
184662	cg09648454	12751316	12751316	0.621088	0	1	0.7691625	45.54799
184693	cg09650180	74722344	74722344	0.6234786	0	1	0.7616761	39.98866
184703	cg09650895	54737362	54737362	0.8505308	0	1	0.665442	-83.59003
184735	cg09653371	18653342	18653342	0.669421	0	1	0.7911394	35.51104
184943	cg09664314	31701446	31701446	0.2870664	0	1	0.1359193	-54.69399
184969	cg09665351	48780377	48780377	0.232896	0	1	0.3417048	25.01105

184978	cg09666011	60688471	74774454	0.437109	0	1	0.3289942	-21.48488
185098	cg0967225	62646475	62646475	0.4333909	0	1	0.8145311	374.3439
185218	cg0968000	59639463	59639463	0.3621626	0	1	0.5090215	35.06061
185762	cg0971485	52742438	52742438	0.7837039	0	1	0.8912041	45.24896
185764	cg0971500	49694411	49694411	0.2465337	0	1	0.3602335	26.10139
185770	cg0971554	66620425	66620425	0.1547001	0	1	0.2547874	27.14597
185797	cg0971792	48701360	48701360	0.2042592	0	1	0.3089979	25.15934
185820	cg0971934	56680359	56680359	0.7793909	0	1	0.6013561	-63.1543
185889	cg0972363	18749365	18749365	0.6810573	0	1	0.7858271	27.70242
185943	cg0972720	37740333	52756471	0.7242907	0	1	0.5251445	-68.27187
185944	cg0972721	65647467	35657353	0.7278513	0	1	0.5884029	-37.6139
185978	cg0972927	57748486	57748486	0.2046039	0	1	0.3048751	23.49935
185986	cg0972962	12741436	12741436	0.631385	0	1	0.7360851	24.23537
186031	cg0973194	64723470	64723470	0.677051	0	1	0.5738681	-21.20246
186173	cg0974045	64711383	64711383	0.1696904	0	1	0.293934	36.10781
186392	cg0975578	69641461	69641461	0.2715471	0	1	0.3758114	21.79374
186484	cg0976072	66609391	66609391	0.6606979	0	1	0.5360966	-28.07372
186548	cg0976343	64613357	64613357	0.5092474	0	1	0.6270097	24.6887
186636	cg0976898	34706422	34706422	0.4895225	0	1	0.7415864	107.1162
186691	cg0977345	29730503	29730503	0.2813194	0	1	0.383816	20.89918
186730	cg0977553	33713314	33713314	0.1693602	0	1	0.0641436	-43.98021
186763	cg0977777	26638501	52687504	0.70637	0	1	0.5761286	-32.31305
186764	cg0977777	22604405	22604405	0.2319665	0	1	0.1031309	-48.6116
186788	cg0977957	40607380	40607380	0.6864695	0	1	0.796056	30.66172
186829	cg0978256	32622429	32622429	0.6293653	0	1	0.7356244	24.77444
186879	cg0978537	41742374	41742374	0.7239154	0	1	0.9174019	119.2594
186893	cg0978609	67720469	32798444	0.8424972	0	1	0.7222394	-41.96803
186970	cg0979022	62741461	62741461	0.4380192	0	1	0.6237568	54.1135
187076	cg0979580	70611337	46645328	0.5095512	0	1	0.6395296	29.40769
187107	cg0979720	70668390	70668390	0.6136455	0	1	0.4960202	-24.37008
187197	cg0980206	33648424	33648424	0.2689523	0	1	0.3916993	28.40867
187246	cg0980464	21670387	21670387	0.1380257	0	1	0.2428499	30.90571
187306	cg0980781	21771379	21771379	0.5933853	0	1	0.738461	41.01535
187349	cg0981008	39664424	26752459	0.7315532	0	1	0.5763027	-45.22768
187367	cg0981151	18684453	18684453	0.6317528	0	1	0.7493091	29.97796
187404	cg0981402	45712411	45712411	0.5080515	0	1	0.623686	23.89269
187711	cg0982964	34701417	34701417	0.6022221	0	1	0.4646255	-31.46272
187719	cg0982990	70657476	70657476	0.1844671	0	1	0.3048092	32.92077
187724	cg0983008	20631334	20631334	0.4489259	0	1	0.5553185	20.08808
187748	cg0983187	38750445	57622344	0.1731637	0	1	0.2901517	32.53178
187758	cg0983227	21697348	21697348	0.2492222	0	1	0.3658915	27.06207
187789	cg0983417	61602344	61602344	0.1705607	0	1	0.2930169	35.20677
187795	cg0983446	31765305	31765305	0.3226633	0	1	0.1965032	-34.3604

187841	cg09837648	20708437	20708437	0.3117304	0	1	0.4229502	22.8859
187886	cg09840472	58662317	58662317	0.4152603	0	1	0.5188969	19.19853
188007	cg09847368	14607396	14607396	0.4806013	0	1	0.7504684	123.2213
188115	cg09852918	44761470	44761470	0.5490596	0	1	0.66919	26.73968
188145	cg09854620	39768378	39768378	0.8806001	0	1	0.6928937	-96.21102
188171	cg09856092	69773355	50633420	0.1560156	0	1	0.2830738	39.01263
188344	cg09863413	70614430	70614430	0.2174482	0	1	0.4615201	100.4617
188393	cg09866143	29794391	29794391	0.7115539	0	1	0.9539192	374.3439
188424	cg09867322	73678369	73678369	0.3090388	0	1	0.4158393	21.51006
188427	cg09867452	59609402	59609402	0.910131	0	1	0.7929403	-57.35209
188560	cg09874333	20609458	20609458	0.632669	0	1	0.7695681	40.1925
188682	cg09883286	41728417	13794301	0.6081927	0	1	0.5076206	-18.83745
188712	cg09884419	61781459	61781459	0.4899685	0	1	0.6219921	29.72293
188714	cg09884451	51639506	51639506	0.4115501	0	1	0.2979454	-24.06925
188727	cg09885502	37733313	33735343	0.3494864	0	1	0.5340031	53.03399
188769	cg09887906	57657397	57657397	0.5794308	0	1	0.6823773	21.29996
188913	cg09895325	38715470	39628431	0.1964277	0	1	0.3985101	74.66545
188952	cg09897374	56749413	70790434	0.359843	0	1	0.2380523	-29.60633
188975	cg09898988	20754477	20754477	0.8499075	0	1	0.9504977	63.0996
189050	cg09903452	12791479	12791479	0.3816852	0	1	0.5097098	27.48386
189108	cg09906928	40799410	40799410	0.7152289	0	1	0.8586565	58.61893
189134	cg09908042	51631444	51631444	0.542432	0	1	0.3465576	-59.30728
189283	cg09916651	63625400	63625400	0.3427527	0	1	0.4479896	20.32945
189289	cg09916840	25639320	25639320	0.8046491	0	1	0.3716293	-347.4175
189400	cg09923954	14773345	14773345	0.2943814	0	1	0.4964115	64.81797
189453	cg09927251	36675362	65676467	0.734139	0	1	0.8455633	37.91451
189456	cg09927311	58759457	58759457	0.6473473	0	1	0.815248	63.57607
189547	cg09933458	50635407	30770374	0.3272159	0	1	0.1969574	-36.11335
189919	cg09957864	64723399	46800423	0.2360386	0	1	0.3439856	24.54164
189988	cg09963080	64809446	64809446	0.8598362	0	1	0.6306602	-120.414
190140	cg09972607	36629427	36629427	0.7691446	0	1	0.8817136	45.86422
190297	cg09981432	25773436	25773436	0.3437622	0	1	0.4600218	23.87731
190306	cg09981929	29649484	74797315	0.7718962	0	1	0.6034445	-56.5895
190323	cg09982942	49748412	18605351	0.6893285	0	1	0.5887882	-20.75669
190363	cg09985260	32797451	32797451	0.5509197	0	1	0.4170865	-29.52718
190430	cg09989037	24657327	24657327	0.427	0	1	0.5350013	20.53456
190448	cg09990194	65605381	65605381	0.2246959	0	1	0.3430478	29.06562
190504	cg09993319	14642304	14642304	0.6540813	0	1	0.2595722	-261.1608
190575	cg09997546	45748362	57671390	0.500368	0	1	0.6049589	20.00641
190588	cg09998151	66795482	66795482	0.2414367	0	1	0.3828064	37.49044
190594	cg09998474	39694500	12708434	0.3817688	0	1	0.2567984	-29.82977
190934	cg10017626	46734487	46734487	0.1050228	0	1	0.2322955	47.33858
191017	cg10021878	53774376	63605419	0.2342991	0	1	0.0679807	-83.27925

191138	cg1002882[illegible]	67770391	12704501	0.2522267	0	1	0.3627138	24.68074
191394	cg1004417[illegible]	20628397	39648458	0.5833757	0	1	0.4365759	-34.90226
191486	cg1005149[illegible]	50641429	50641429	0.7916456	0	1	0.6901632	-26.87244
191487	cg1005158[illegible]	36644327	36644327	0.6120553	0	1	0.7451665	36.19341
191574	cg1005648[illegible]	42642363	44755425	0.8489318	0	1	0.7447746	-34.79751
191608	cg1005820[illegible]	46658359	46658359	0.4394643	0	1	0.2386764	-68.48821
191705	cg1006357[illegible]	36606308	36606308	0.5360671	0	1	0.6593155	27.52862
191735	cg1006492[illegible]	53768307	53768307	0.4813076	0	1	0.6974249	76.09773
191774	cg1006751[illegible]	64604404	21764398	0.8786032	0	1	0.734111	-64.93639
191775	cg1006752[illegible]	74698434	74698434	0.3821718	0	1	0.4848748	19.08353
191795	cg1006846[illegible]	41718349	41718349	0.7076383	0	1	0.8240324	37.10529
191836	cg1007032[illegible]	25698303	25698303	0.920518	0	1	0.7073883	-138.205
191875	cg1007231[illegible]	74723489	74723489	0.1882872	0	1	0.2917003	25.73081
191937	cg1007606[illegible]	10770329	10770329	0.0961358	0	1	0.2255839	50.50133
191943	cg1007656[illegible]	19623402	67797490	0.1035021	0	1	0.2760525	75.53871
192027	cg1008199[illegible]	39717404	39717404	0.5088095	0	1	0.6210374	22.70477
192041	cg1008264[illegible]	42713510	12690452	0.5410049	0	1	0.4052341	-30.27216
192081	cg1008513[illegible]	51671322	51671322	0.3509364	0	1	0.4968503	34.85882
192116	cg1008778[illegible]	18679499	18679499	0.6311012	0	1	0.509632	-26.07555
192135	cg1008889[illegible]	60701395	60701395	0.2098172	0	1	0.1046189	-35.67121
192182	cg1009059[illegible]	26699355	26699355	0.7044186	0	1	0.8081965	29.40134
192204	cg1009168[illegible]	65664331	65664331	0.1162655	0	1	0.2204243	33.38848
192210	cg1009179[illegible]	46644403	46644403	0.3497359	0	1	0.5054184	39.06125
192350	cg1009731[illegible]	35801339	35801339	0.4031441	0	1	0.5062025	19.06178
192367	cg1009837[illegible]	26730410	26730410	0.6101993	0	1	0.7421328	35.43556
192383	cg1009927[illegible]	36626469	36626469	0.9011351	0	1	0.6343961	-175.633
192415	cg1010058[illegible]	10701414	10701414	0.7806164	0	1	0.3974653	-257.9031
192483	cg1010425[illegible]	49750464	62603505	0.3441199	0	1	0.5939384	95.13174
192485	cg1010433[illegible]	48667441	48667441	0.2126734	0	1	0.3152021	23.84504
192486	cg1010442[illegible]	65770434	65770434	0.2901523	0	1	0.3980844	22.39861
192492	cg1010467[illegible]	63686350	63686350	0.3629066	0	1	0.4658832	19.35643
192589	cg1010963[illegible]	66763415	66763415	0.4060071	0	1	0.5379331	28.80147
192590	cg1010973[illegible]	26610493	57771424	0.2875279	0	1	0.4160501	29.82548
192599	cg1011033[illegible]	56642304	56642304	0.6318156	0	1	0.7685103	39.98561
192631	cg1011162[illegible]	17632460	17632460	0.2096096	0	1	0.3236614	28.3658
192700	cg1011532[illegible]	59657306	59657306	0.8512689	0	1	0.6925194	-66.26144
192734	cg1011707[illegible]	38767335	38767335	0.6287603	0	1	0.4638844	-43.82382
192743	cg1011759[illegible]	58659388	58659388	0.2393609	0	1	0.3595036	28.89316
192921	cg1012737[illegible]	33693488	33693488	0.49366	0	1	0.59539	19.01244
192974	cg1013037[illegible]	62620348	35738443	0.6686992	0	1	0.5238076	-36.44757
193163	cg1014053[illegible]	63698418	26738315	0.9421256	0	1	0.8010952	-91.16594
193186	cg1014171[illegible]	68626466	68626466	0.2718245	0	1	0.1368362	-45.72508
193242	cg1014388[illegible]	20708490	20708490	0.7934275	0	1	0.9230481	71.617

193251	cg10144473	65705415	65705415	0.5950872	0	1	0.8296739	113.932
193262	cg10144863	23692465	23692465	0.3428894	0	1	0.4937552	37.1115
193299	cg10146330	74684498	33624416	0.3115919	0	1	0.6753516	374.3439
193386	cg10150902	71758432	71758432	0.5160663	0	1	0.3926893	-25.73768
193420	cg10152997	46736327	46736327	0.3377355	0	1	0.4830004	34.87336
193677	cg10166986	67703338	67703338	0.2089177	0	1	0.6021848	374.3439
193679	cg10167233	62695354	62695354	0.5884505	0	1	0.7111549	29.59332
193684	cg10167378	13650306	66699494	0.5178072	0	1	0.7072139	60.90091
193696	cg10167978	65742433	65742433	0.9356256	0	1	0.6982182	-171.6428
193895	cg10177987	71804313	71804313	0.6666328	0	1	0.8702114	104.1397
193937	cg10180092	18728379	18728379	0.5005173	0	1	0.6102572	21.70411
194006	cg10184328	37717325	37717325	0.6948512	0	1	0.5772682	-26.84933
194178	cg10193721	28779428	15772359	0.1756654	0	1	0.0649176	-47.12617
194261	cg10197336	24672333	24672333	0.2552858	0	1	0.1080415	-58.26193
194305	cg10200291	43600311	32739338	0.2273326	0	1	0.0885824	-57.95707
194308	cg10200373	33809357	33809357	0.5756686	0	1	0.4460659	-28.07678
194351	cg10202544	64646353	64646353	0.7167193	0	1	0.5717798	-39.23738
194363	cg10202835	41624389	21791419	0.0738164	0	1	0.2905851	119.7999
194418	cg10205431	14745467	57800340	0.8270274	0	1	0.6403946	-78.10133
194503	cg10208777	40616479	40616479	0.6083827	0	1	0.7087994	21.45019
194584	cg10213302	58784432	72762391	0.5275703	0	1	0.4143333	-22.21757
194593	cg10213658	68644458	68644458	0.2201541	0	1	0.3585359	37.74213
194599	cg10213875	36762446	71698444	0.8471951	0	1	0.709359	-52.59661
194652	cg10217052	62734347	62734347	0.1436247	0	1	0.5432426	374.3439
194715	cg10220544	68744315	68744315	0.7233846	0	1	0.8264785	31.16823
194724	cg10221014	48768314	48768314	0.2611159	0	1	0.3712521	24.17717
194779	cg10224537	48788306	48788306	0.6257455	0	1	0.8371242	98.37041
194802	cg10225951	63694309	63694309	0.5502698	0	1	0.7320655	59.08015
194940	cg10234477	34641380	34641380	0.3824147	0	1	0.4975428	22.9955
194959	cg10235453	72747457	72747457	0.565957	0	1	0.4339885	-28.88202
195048	cg10240356	61792426	61792426	0.4285659	0	1	0.5669634	31.35979
195063	cg10241183	58737492	58737492	0.5867655	0	1	0.6981156	24.74886
195118	cg10243348	44789485	58730396	0.773494	0	1	0.662371	-29.2484
195126	cg10243855	18793443	18793443	0.4121045	0	1	0.5361413	25.90452
195217	cg10249074	54753406	54753406	0.7426081	0	1	0.9263428	116.5898
195276	cg10252249	51767492	47795418	0.5358065	0	1	0.6924985	42.97214
195433	cg10262052	73673357	73673357	0.2865395	0	1	0.4026526	25.3073
195449	cg10263003	16672373	16672373	0.9502533	0	1	0.3737271	-371.3336
195473	cg10265387	20622426	14759462	0.3488677	0	1	0.2105158	-38.5299
195499	cg10266681	48690416	48690416	0.234715	0	1	0.3843098	41.67818
195845	cg10288525	36721481	36721481	0.4557852	0	1	0.5566803	18.43722
195862	cg10289744	39617333	39617333	0.3757955	0	1	0.5196625	33.65699
195894	cg10291990	69803510	47734337	0.4490813	0	1	0.3054608	-35.16396

195904	cg10292584	16687334	39646344	0.5834739	0	1	0.3561824	-78.70958
195919	cg10293824	58769488	58769488	0.7023255	0	1	0.8218706	38.33691
195948	cg10295955	55774489	55774489	0.7095852	0	1	0.5620083	-39.92223
195974	cg10297211	31682503	57660444	0.4108887	0	1	0.2969954	-24.19672
196106	cg10306192	19754448	19754448	0.1924088	0	1	0.4026679	80.59982
196148	cg10308629	37725360	37725360	0.470383	0	1	0.5877722	23.8972
196199	cg10310917	32759433	32759433	0.3690113	0	1	0.4924473	25.97047
196202	cg10311048	12710436	12710436	0.3841892	0	1	0.5495459	43.04004
196231	cg10312513	48627497	62620306	0.4461756	0	1	0.579567	29.53123
196334	cg10317314	11754397	46702490	0.2917202	0	1	0.0985281	-91.53165
196361	cg10318368	54669481	54669481	0.1383874	0	1	0.2424935	30.54426
196395	cg10319942	49746468	49746468	0.6193115	0	1	0.51891	-18.96021
196538	cg10328089	74799317	74799317	0.333906	0	1	0.4581059	26.83479
196551	cg10328548	42739345	42793430	0.9076254	0	1	0.6289781	-191.9018
196567	cg10329418	17781495	17781495	0.4231446	0	1	0.2601616	-46.1609
196642	cg10333144	72648369	72648369	0.8003443	0	1	0.9093319	51.55349
196675	cg10334741	62616428	41794445	0.5044827	0	1	0.397832	-20.18052
196702	cg10336025	59765370	59765370	0.8287678	0	1	0.9324754	56.66745
196707	cg10336193	66689473	66689473	0.3150435	0	1	0.4286817	23.61042
196745	cg10338539	32639331	32639331	0.1604938	0	1	0.2997841	44.43414
196779	cg10340904	67667448	68789383	0.6005599	0	1	0.3917626	-66.76717
196792	cg10342016	73764451	73764451	0.6021795	0	1	0.7082717	23.35287
196867	cg10347293	31743462	36775445	0.4947979	0	1	0.3861383	-20.88016
196923	cg10350492	28641376	28641376	0.4288897	0	1	0.3108577	-25.22528
196934	cg10351284	59704379	59704379	0.2989727	0	1	0.3993524	19.73711
196953	cg10352237	31715407	31715407	0.3216773	0	1	0.4526606	29.63467
197255	cg10370305	23744363	23744363	0.097594	0	1	0.2132536	42.42099
197299	cg10372770	17666483	17666483	0.736187	0	1	0.5773075	-47.4348
197354	cg10375597	16737369	16737369	0.7355627	0	1	0.5722157	-49.62096
197507	cg10384133	38628314	38628314	0.4891091	0	1	0.6374159	36.69022
197532	cg10385522	25755461	25755461	0.0310391	0	1	0.3656456	374.3439
197630	cg10392572	12774369	12774369	0.2809121	0	1	0.3943084	24.55468
197852	cg10407894	65651440	65651440	0.4506465	0	1	0.5640883	22.37311
197877	cg10409680	57807397	57807397	0.2482482	0	1	0.3939453	38.94124
197914	cg10413089	26784326	26784326	0.5706108	0	1	0.7135342	38.11898
197929	cg10413861	18762412	18762412	0.5218606	0	1	0.6264007	20.35061
197950	cg10415021	27769303	27769303	0.223686	0	1	0.4000007	56.07333
198088	cg10423607	11618340	11618340	0.2261839	0	1	0.3772828	43.07069
198093	cg10423842	61754407	61754407	0.5733827	0	1	0.7041187	32.38601
198106	cg10424681	43636484	56690452	0.4706398	0	1	0.3415228	-28.46481
198320	cg10436792	59662421	59662421	0.6268254	0	1	0.7395742	27.4325
198389	cg10440578	70725460	29631307	0.2378397	0	1	0.1062571	-49.5091
198546	cg10449329	28685469	28685469	0.8517932	0	1	0.7431114	-37.55486

198823	cg1046642	37620417	37620417	0.4036501	0	1	0.5124132	20.8126
198970	cg1047569	69766330	69766330	0.6993225	0	1	0.8059638	30.40638
199058	cg1048107	27771430	27771430	0.6930797	0	1	0.5742033	-27.24592
199084	cg1048251	72767486	72767486	0.3984154	0	1	0.6204134	75.5007
199102	cg1048366	52770325	52770325	0.4644748	0	1	0.6840159	77.16663
199118	cg1048467	70756443	70756443	0.678826	0	1	0.5744061	-21.66272
199143	cg1048606	26671487	74620448	0.764287	0	1	0.5797523	-64.68176
199177	cg1048805	73621324	73621324	0.4871798	0	1	0.680346	60.90435
199268	cg1049343	16743503	16743503	0.2858937	0	1	0.4100147	28.2285
199348	cg1049789	27600436	27600436	0.5744884	0	1	0.6947286	27.82707
199393	cg1050002	42755347	56698327	0.3886822	0	1	0.2157543	-55.08994
199416	cg1050070	52679452	52679452	0.7192851	0	1	0.8540182	52.30589
199429	cg1050109	58622410	29711507	0.2562732	0	1	0.3628876	23.15919
199432	cg1050121	43774458	66775341	0.1911157	0	1	0.321625	36.76616
199600	cg1050775	43648365	43648365	0.8576421	0	1	0.6936008	-71.4525
199603	cg1050796	33752434	33752434	0.2323495	0	1	0.1091288	-44.30248
199673	cg1051142	51782318	51782318	0.2923642	0	1	0.1893016	-25.52485
199716	cg1051332	69603337	69603337	0.535141	0	1	0.6582032	27.42328
199732	cg1051399	44786356	44786356	0.7243018	0	1	0.8742558	67.17198
199816	cg1051815	24682375	24682375	0.7390864	0	1	0.8392172	31.43956
200018	cg1052842	27765350	41611371	0.7818816	0	1	0.6005414	-65.51028
200255	cg1053989	35729325	38628452	0.6458896	0	1	0.5145916	-30.10046
200285	cg1054184	13613324	13613324	0.257839	0	1	0.3606395	21.8102
200329	cg1054401	16767305	42611322	0.9610099	0	1	0.8022776	-120.79
200360	cg1054621	74613345	74613345	0.5187827	0	1	0.6589482	34.06738
200416	cg1054907	59810379	59810379	0.7151939	0	1	0.8240839	33.53853
200489	cg1055310	33767380	33767380	0.4738955	0	1	0.3622203	-22.08229
200490	cg1055320	61671309	19659376	0.4352826	0	1	0.5357017	18.23189
200491	cg1055321	15624313	15624313	0.9605852	0	1	0.6355776	-289.2523
200558	cg1055768	47625505	47625505	0.7624828	0	1	0.6093349	-47.31697
200564	cg1055790	15629450	15629450	0.2271293	0	1	0.3317635	23.8139
200577	cg1055915	48602325	48602325	0.2615158	0	1	0.3920603	31.78065
200601	cg1056056	65732339	65732339	0.3428967	0	1	0.4851842	33.53755
200633	cg1056400	41624467	41624467	0.2090445	0	1	0.3113703	23.98133
200687	cg1056806	39730413	41722336	0.0403308	0	1	0.4199293	374.3439
200780	cg1057449	39690462	39690462	0.2261563	0	1	0.3268936	22.49675
200816	cg1057624	37667504	37667504	0.1093096	0	1	0.2677152	64.80154
200860	cg1057877	72761345	11607503	0.8885319	0	1	0.7759256	-47.64005
201035	cg1058667	73667307	73667307	0.2182739	0	1	0.3273773	25.92854
201045	cg1058718	16804357	16804357	0.7131625	0	1	0.8273014	36.47205
201181	cg1059341	19741306	19741306	0.4707285	0	1	0.3573278	-22.71008
201269	cg1059938	17680427	17680427	0.2261501	0	1	0.1018973	-46.29323
201316	cg1060117	16611329	16611329	0.644876	0	1	0.7474643	24.1209

201402	cg1060612[illegible]	54774472	52617432	0.5934281	0	1	0.4139176	-50.21238
201467	cg1060986[illegible]	20806344	20806344	0.4233478	0	1	0.5544385	28.49908
201540	cg1061480[illegible]	44746361	45807398	0.4845547	0	1	0.2847721	-64.07866
201559	cg1061621[illegible]	28810412	28810412	0.5772939	0	1	0.4635919	-22.56145
201629	cg1062088[illegible]	53719509	39727434	0.472358	0	1	0.5810629	20.99366
201659	cg1062255[illegible]	28629497	70809301	0.2987296	0	1	0.1980028	-24.06924
201733	cg1062616[illegible]	26755468	26755468	0.5138428	0	1	0.723377	74.57735
201826	cg1063128[illegible]	49739470	12756462	0.3781751	0	1	0.4923107	22.70624
201849	cg1063214[illegible]	73771431	16606388	0.839309	0	1	0.9426655	61.16409
201863	cg1063265[illegible]	45693300	45693300	0.2672202	0	1	0.1429529	-39.37914
201867	cg1063277[illegible]	49764464	49764464	0.7117471	0	1	0.2673025	-352.0149
201871	cg1063289[illegible]	49752455	45646503	0.4429239	0	1	0.3303583	-22.90353
201947	cg1063589[illegible]	47783382	19651386	0.7556244	0	1	0.5695279	-64.38499
202116	cg1064696[illegible]	21727497	21727497	0.4620561	0	1	0.5651382	19.13577
202190	cg1065165[illegible]	67683365	67683365	0.7154658	0	1	0.8196237	30.82731
202251	cg1065555[illegible]	74623332	35659384	0.507873	0	1	0.3895772	-24.00131
202280	cg1065820[illegible]	19749453	19749453	0.3955228	0	1	0.2907744	-21.34026
202348	cg1066231[illegible]	27686326	47643473	0.3134922	0	1	0.1228278	-82.36134
202383	cg1066430[illegible]	60733432	60733432	0.6241343	0	1	0.7546375	35.85216
202422	cg1066634[illegible]	71792334	71792334	0.4685312	0	1	0.6186957	36.94909
202492	cg1067039[illegible]	42685325	42685325	0.4731885	0	1	0.3672798	-20.20056
202524	cg1067256[illegible]	28763342	42751473	0.8611569	0	1	0.6611953	-97.92007
202565	cg1067480[illegible]	20619509	60731404	0.6728159	0	1	0.7986975	38.32244
202638	cg1068022[illegible]	18770414	41604395	0.6654974	0	1	0.5510105	-24.61408
202657	cg1068139[illegible]	40676346	40676346	0.5699594	0	1	0.7548859	63.60258
202732	cg1068708[illegible]	68731469	64679387	0.7982158	0	1	0.6366978	-56.73355
202734	cg1068713[illegible]	55799382	55799382	0.2602974	0	1	0.3734396	25.27067
202760	cg1068940[illegible]	62688381	20720486	0.1939053	0	1	0.3049618	28.30341
202772	cg1069015[illegible]	37773346	49711327	0.0574568	0	1	0.2139518	80.42786
202803	cg1069214[illegible]	24663433	24663433	0.2439508	0	1	0.3836262	36.58721
202839	cg1069472[illegible]	10773446	10773446	0.3171908	0	1	0.4613881	35.00292
202853	cg1069554[illegible]	33611395	33611395	0.8132875	0	1	0.7127901	-28.52244
202863	cg1069635[illegible]	56781474	56781474	0.2721733	0	1	0.3728996	20.623
202904	cg1069917[illegible]	26650456	26650456	0.6426752	0	1	0.7499738	26.02229
202940	cg1070103[illegible]	65615318	65615318	0.5172709	0	1	0.6837956	47.1201
203131	cg1071247[illegible]	43792404	43792404	0.5438232	0	1	0.6837046	35.05529
203155	cg1071358[illegible]	33739367	33739367	0.5278577	0	1	0.6358107	21.62428
203180	cg1071527[illegible]	51719344	51719344	0.5035151	0	1	0.3862242	-23.68622
203267	cg1071979[illegible]	70612465	70612465	0.7515518	0	1	0.9322144	117.8374
203315	cg1072244[illegible]	53683501	53683501	0.1782638	0	1	0.3043761	36.05555
203346	cg1072463[illegible]	51626331	51626331	0.344129	0	1	0.220982	-31.21485
203361	cg1072534[illegible]	26603501	26603501	0.2086065	0	1	0.3328277	32.54938
203369	cg1072584[illegible]	54602367	54602367	0.8153774	0	1	0.9419471	79.22176

203396	cg1072766(	21678464	21678464	0.795144	0	1	0.676253	-34.66993
203430	cg1072949(	56800480	63799480	0.7864443	0	1	0.6563604	-38.8513
203508	cg1073547:	54683477	54683477	0.3416869	0	1	0.2229668	-29.31868
203511	cg1073560'	16760467	16760467	0.3560254	0	1	0.4896533	29.88937
203518	cg1073630:	64642498	64642498	0.7839056	0	1	0.944113	108.6565
203552	cg1073864{	44601351	44601351	0.7356861	0	1	0.6105205	-32.10106
203586	cg1074141{	63646479	63646479	0.523804	0	1	0.7160012	63.31508
203615	cg1074407(	37605414	37605414	0.2528036	0	1	0.3963363	37.69538
203651	cg1074748:	53606504	65610464	0.7267292	0	1	0.9623065	374.3439
203681	cg1075026	57779501	57779501	0.7549734	0	1	0.5109869	-103.1303
203682	cg1075030(	47659388	47659388	0.1500118	0	1	0.430079	145.7438
203724	cg1075361(	19627345	19627345	0.3739349	0	1	0.5012141	27.28862
203736	cg1075439:	26699375	46663315	0.6100686	0	1	0.7764744	56.10493
203825	cg1075981'	14739477	14739477	0.5728632	0	1	0.4665663	-20.16994
203853	cg1076163(	48664460	48664460	0.7517073	0	1	0.5726035	-59.85733
203882	cg1076323	64765384	33699316	0.2892794	0	1	0.1814269	-27.9923
204049	cg1077253:	66671425	66671425	0.3987997	0	1	0.6883728	132.2059
204144	cg1077734'	74689428	74689428	0.3787054	0	1	0.5132801	29.95687
204230	cg1078077{	24632369	24632369	0.5064656	0	1	0.6273555	25.78312
204254	cg1078220(	18809487	18809487	0.6197484	0	1	0.7542864	37.61365
204318	cg1078515:	28625444	28625444	0.1680725	0	1	0.3606892	73.46073
204443	cg1079230:	51612373	31716425	0.5345616	0	1	0.3145645	-74.88975
204453	cg1079291:	14625395	14625395	0.307455	0	1	0.4730254	44.85247
204486	cg1079511(	22779306	39773476	0.6104859	0	1	0.7121995	22.02747
204575	cg1080034(	60620379	60620379	0.7173258	0	1	0.820271	30.36083
204636	cg1080357'	57726430	57726430	0.8414994	0	1	0.7381079	-33.30662
204659	cg1080497	54606328	54606328		0	1		-4.34E-09
204737	cg1080919:	30762401	38713319	0.8658586	0	1	0.716709	-64.14434
204738	cg1080928:	17670482	17670482	0.2667298	0	1	0.3854108	26.99623
204774	cg1081142(	47696321	47696321	0.1815158	0	1	0.2891982	27.91696
204796	cg1081263	67759420	70703360	0.3842651	0	1	0.1684404	-88.83799
204802	cg1081288(	45734474	45734474	0.6824914	0	1	0.7890756	28.73178
204846	cg1081565'	53795414	51795457	0.47833	0	1	0.1873725	-145.8998
204847	cg10815671	17802397	17802397	0.6156853	0	1	0.7404875	32.29366
204936	cg1082004:	59617490	60758463	0.3113853	0	1	0.197335	-29.24178
204950	cg1082073(	54723450	54723450	0.6204485	0	1	0.8333055	98.30006
204972	cg1082196	48742463	48742463	0.7807511	0	1	0.3807185	-283.5222
204999	cg10823511	39601303	39601303	0.8199483	0	1	0.9282041	58.16123
205079	cg1082765(	41715408	41715408	0.6266115	0	1	0.733838	25.0271
205136	cg1083124(	19744411	48735439	0.4236372	0	1	0.3005267	-27.34332
205151	cg1083209:	50693368	52631312	0.6452745	0	1	0.4668614	-51.06237
205266	cg1083778:	26715316	36715368	0.5779297	0	1	0.4238193	-37.96814
205305	cg1083972:	28804403	28804403	0.2365492	0	1	0.1237875	-36.47261

205377	cg1084427:	53653457	53653457	0.5560196	0	1	0.7267843	52.59577
205379	cg1084438:	29765465	29765465	0.940088	0	1	0.8171902	-75.19838
205399	cg1084661:	67669371	10643503		0	1	0.2168128	4.34E-09
205527	cg1085542:	37707303	37707303	0.6412026	0	1	0.8141305	66.39854
205586	cg1085864(	61677417	61677417	0.6208779	0	1	0.9139124	374.3439
205654	cg1086246:	52672494	52672494	0.3045819	0	1	0.4222862	25.28893
205656	cg1086253:	23757363	23757363	0.5242746	0	1	0.3966323	-27.24479
205667	cg1086296(	59766311	59766311	0.6649225	0	1	0.862384	96.4939
205676	cg1086373:	74759421	74759421	0.6147417	0	1	0.7950428	67.17581
205812	cg1087409:	47607347	47607347	0.5161307	0	1	0.6351062	25.29829
205871	cg1087823(	55679444	55679444	0.6579117	0	1	0.7811295	35.1274
205969	cg1088362l	13644317	13644317		0	1	0.230087	4.34E-09
206050	cg1088881l	56793416	56793416	0.2300164	0	1	0.3651753	35.59412
206070	cg1089064:	66636357	66636357	0.3019593	0	1	0.1170732	-79.99968
206084	cg1089157(	20679318	20679318	0.8547381	0	1	0.7192701	-52.89954
206112	cg1089327:	41806353	41806353	0.4471269	0	1	0.3054949	-34.34173
206153	cg1089587:	18781332	18781332	0.5977092	0	1	0.4640116	-29.88329
206226	cg1090031:	20670427	20670427	0.2368239	0	1	0.3570893	29.08419
206280	cg1090285(	42604354	42604354	0.1413821	0	1	0.3259104	73.73145
206376	cg1090918:	69618411	69736444	0.5077274	0	1	0.6354414	28.44716
206413	cg1091128:	63683505	63683505	0.641649	0	1	0.5290422	-23.28782
206442	cg1091297:	12672439	53693413	0.5717084	0	1	0.4471695	-26.20936
206540	cg1092075:	66645436	66645436	0.4632343	0	1	0.3420536	-25.59842
206628	cg1092685l	59711416	59711416	0.7123902	0	1	0.8217647	33.46705
206687	cg1093030:	73636451	49750505	0.0417148	0	1	0.2531583	132.5911
206779	cg1093740:	40607452	37633345	0.3355566	0	1	0.4586455	26.40232
206830	cg1094020:	51614481	58624406	0.4973477	0	1	0.6452363	36.77664
206934	cg1094626:	21687351	21687351	0.8159381	0	1	0.9330034	66.92367
207073	cg1095315:	22773335	22773335	0.4387712	0	1	0.5466077	20.50333
207092	cg1095433(	25645386	25645386	0.7416039	0	1	0.8697947	52.19648
207137	cg1095700l	62702473	62702473	0.1979232	0	1	0.5817538	374.3439
207310	cg1096702:	13720352	28601475	0.3311312	0	1	0.1967048	-38.00304
207353	cg1096946:	74632373	25717329	0.7696246	0	1	0.8873321	50.44429
207365	cg1097034:	48671357	48671357	0.8324281	0	1	0.7261056	-33.43758
207413	cg1097362:	17606321	17606321	0.6628187	0	1	0.5033469	-42.63545
207426	cg1097441:	44729430	44729430	0.9378366	0	1	0.7316631	-144.3126
207438	cg1097535:	39806406	39806406	0.3100429	0	1	0.1806353	-37.30603
207494	cg1097858:	71789303	59608377	0.5895876	0	1	0.4853458	-19.70225
207518	cg1097984(	62606363	62606363	0.5924067	0	1	0.7103962	27.75023
207568	cg1098204:	10766315	10766315	0.6534234	0	1	0.5528562	-19.7112
207605	cg1098348:	56735443	56735443	0.0929003	0	1	0.2224925	51.33793
207692	cg1098784(	27796427	27796427	0.0925731	0	1	0.1995458	38.70404
207720	cg1098921:	70728324	53740485	0.2113909	0	1	0.3298191	29.97598

207724	cg1098931	24719408	24719408	0.9805223	0	1	0.705961	-254.0816
207740	cg1098998	66708439	66708439	0.6650235	0	1	0.5456252	-26.32907
207744	cg1099018	18656427	18656427	0.9392588	0	1	0.8335811	-61.27249
207750	cg1099073	42615324	42615324	0.5388529	0	1	0.3127538	-78.97857
207770	cg1099185	69672418	69672418	0.8676916	0	1	0.9678956	73.91393
207830	cg1099494	64607349	66644423	0.4310613	0	1	0.3137291	-24.90491
207831	cg1099508	25735316	13767374	0.335272	0	1	0.1919212	-42.64685
207838	cg1099542	23622307	23622307	0.2163901	0	1	0.3930907	57.06613
207846	cg1099587	36602457	36602457	0.3070396	0	1	0.4202823	23.68605
207875	cg1099719	20780397	20780397	0.4712307	0	1	0.5774867	20.19679
207959	cg1100268	70796444	70796444	0.720292	0	1	0.6101689	-25.35443
207966	cg1100292	28620325	28620325	0.6738339	0	1	0.5640972	-23.26531
208000	cg1100562	70706315	47670353	0.4418814	0	1	0.300606	-34.37594
208005	cg1100584	30791422	39669490	0.431447	0	1	0.327748	-20.12075
208046	cg1100812	46645482	54708500	0.4166279	0	1	0.5370355	24.62822
208086	cg1101074	21757438	21757438	0.6297506	0	1	0.809072	69.30208
208090	cg1101151	10722313	43809366	0.525847	0	1	0.3645859	-41.31843
208103	cg1101215	72808343	33661308	0.3889416	0	1	0.6068175	72.55235
208164	cg1101576	73789466	73789466	0.7656347	0	1	0.8846037	50.47218
208168	cg1101615	72708381	72708381	0.3980041	0	1	0.4989367	18.45365
208228	cg1101979	68715414	68715414	0.5997161	0	1	0.4755575	-26.40534
208302	cg1102444	51660345	58651460	0.4999854	0	1	0.3433639	-39.61333
208339	cg1102660	17765356	17765356	0.3394212	0	1	0.4571091	24.44222
208424	cg1103156	21750367	21750367	0.3414563	0	1	0.4566872	23.57345
208492	cg1103530	11654432	11654432	0.1936242	0	1	0.0383847	-88.32603
208507	cg1103635	58702337	58702337	0.7560548	0	1	0.5053615	-108.5207
208539	cg1103823	68803380	68803380	0.470262	0	1	0.58449	22.80687
208616	cg1104399	40798374	40798374	0.6462311	0	1	0.7817932	40.91311
208620	cg1104416	68801378	68801378	0.866635	0	1	0.6581475	-106.5339
208655	cg1104677	29796509	29796509	0.4585247	0	1	0.60374	34.54988
208664	cg1104744	36635354	44677485	0.4905699	0	1	0.6272872	31.66284
208672	cg1104879	33699510	33699510	0.5951623	0	1	0.7556099	50.25614
208738	cg1105306	11802432	11802432		0	1		-4.34E-09
208749	cg1105363	38637330	38637330	0.5598042	0	1	0.3990816	-40.84737
208817	cg1105915	12606302	12606302	0.6824833	0	1	0.5491797	-32.30656
208844	cg1106053	27709499	27709499	0.3564525	0	1	0.2459902	-24.95792
208886	cg1106308	17792508	17792508	0.7278963	0	1	0.8471847	42.2944
208948	cg1106624	42649325	42649325	0.5200703	0	1	0.6602395	34.11985
208953	cg1106660	57787312	34636361	0.2128265	0	1	0.3569528	40.96913
208991	cg1106888	34643333	34643333	0.4642768	0	1	0.5713174	20.38772
209083	cg1107357	60741458	60741458	0.5157655	0	1	0.6410564	27.71017
209101	cg1107435	10705406	10705406	0.6356436	0	1	0.5218451	-23.54081
209107	cg1107478	49661459	42679464	0.343799	0	1	0.2191169	-31.9695

209237	cg1108127[illegible]	66631343	66631343	0.1907746	0	1	0.3121785	32.81573
209265	cg1108263[illegible]	27700403	27700403	0.9410316	0	1	0.8043659	-86.88321
209421	cg1109283[illegible]	13748326	13748326	0.5451713	0	1	0.6479825	20.27625
209570	cg1110408[illegible]	31681490	62791345	0.1078669	0	1	0.207959	32.64331
209634	cg1110847[illegible]	10605372	42766351	0.3860861	0	1	0.2357326	-41.95108
209675	cg1111146[illegible]	25733438	25733438	0.2709657	0	1	0.4046874	32.58414
209754	cg1111627[illegible]	71647392	71647392	0.195675	0	1	0.3006259	25.80253
209830	cg1111976[illegible]	11633490	11633490	0.2862088	0	1	0.4546289	47.22604
209881	cg1112361[illegible]	44666354	44666354	0.6779099	0	1	0.9030443	137.9623
209893	cg1112413[illegible]	70793333	67682493	0.720764	0	1	0.4688723	-103.9554
209948	cg1112802[illegible]	56652322	56652322	0.6554573	0	1	0.7611081	26.14035
209950	cg1112821[illegible]	30791331	30791331	0.4221212	0	1	0.5399126	23.73216
209960	cg1112895[illegible]	52764301	52764301	0.2760793	0	1	0.378131	20.92164
210003	cg1113253[illegible]	34739436	34739436	0.4604956	0	1	0.5618817	18.61023
210020	cg1113365[illegible]	35656319	35656319	0.9061135	0	1	0.772486	-67.23
210110	cg1113924[illegible]	65602468	65602468	0.4028031	0	1	0.5269845	25.97564
210200	cg1114410[illegible]	33619359	33619359	0.1730007	0	1	0.4634277	148.7541
210266	cg1114786[illegible]	21731367	21731367	0.1911095	0	1	0.2963606	26.23596
210412	cg1115543[illegible]	23722457	23722457	0.1687002	0	1	0.2866773	33.4056
210438	cg1115687[illegible]	24770432	34671340	0.4987013	0	1	0.6071665	21.24845
210600	cg1116645[illegible]	71682363	20729361	0.6056774	0	1	0.4417006	-42.81678
210619	cg1116810[illegible]	66770303	39654448	0.7473283	0	1	0.6275854	-30.71834
210635	cg1116984[illegible]	48640443	48640443	0.5895336	0	1	0.7362809	41.60292
210694	cg1117360[illegible]	30675437	30675437	0.1457856	0	1	0.2581055	33.37979
210697	cg1117382[illegible]	12663331	12663331	0.3434559	0	1	0.4892893	34.97807
210819	cg1118169[illegible]	17786435	54779449	0.7710768	0	1	0.6407216	-37.32569
210937	cg1118866[illegible]	68683459	68683459	0.518903	0	1	0.6470894	28.96039
211112	cg1119885[illegible]	26635475	26635475	0.3219083	0	1	0.4251541	20.09718
211130	cg1119963[illegible]	35723384	44733367	0.3774671	0	1	0.2667739	-24.14397
211153	cg1120074[illegible]	15605331	15605331	0.6974393	0	1	0.8011534	28.66555
211179	cg1120165[illegible]	31712468	31712468	0.2430516	0	1	0.3495678	23.69996
211250	cg1120497[illegible]	74804386	74804386	0.4602429	0	1	0.270528	-59.31372
211316	cg1120832[illegible]	35677461	35677461	0.933093	0	1	0.8235528	-61.27816
211429	cg1121450[illegible]	61632480	61632480	0.3885368	0	1	0.5215592	29.28752
211440	cg1121496[illegible]	17728452	10767414	0.1827169	0	1	0.3018158	32.54634
211453	cg1121617[illegible]	50717467	50717467	0.3022987	0	1	0.4259999	27.50185
211504	cg1121941[illegible]	51645476	51645476	0.1869043	0	1	0.4474087	118.9654
211593	cg1122393[illegible]	45608323	45608323	0.191155	0	1	0.30846	31.05895
211618	cg1122509[illegible]	21723504	21723504	0.3193361	0	1	0.1357149	-74.43496
211702	cg1122927[illegible]	37646416	37646416	0.5270033	0	1	0.6710918	36.21606
211766	cg1123173[illegible]	29646482	29646482	0.5218753	0	1	0.665724	35.87502
211835	cg1123559[illegible]	33659459	33659459	0.3715601	0	1	0.4954851	26.11337
211852	cg1123645[illegible]	23667335	23667335	0.6533093	0	1	0.5356111	-25.35006

211897	cg11238952	10745507	55675345	0.4897759	0	1	0.3675637	-25.5584
211989	cg11244648	35662458	53636494	0.4082751	0	1	0.5132422	19.62024
212007	cg11245569	73703494	63631386	0.6325395	0	1	0.5282506	-20.3943
212200	cg11256764	74600393	27676391	0.6979563	0	1	0.8634738	74.18694
212208	cg11257300	34664436	34664436	0.4809797	0	1	0.6125954	29.36408
212262	cg11261412	56712505	56712505	0.3387745	0	1	0.4698528	29.25383
212462	cg11273655	61728324	61728324	0.5664344	0	1	0.4515389	-22.86572
212500	cg11276189	13703378	13703378	0.7823308	0	1	0.9083332	62.75547
212606	cg11282828	71675337	46766498	0.4160318	0	1	0.5817083	43.26226
212723	cg11290168	19810503	19810503	0.5614215	0	1	0.4567191	-19.60237
212791	cg11294620	15746406	27760466	0.9217269	0	1	0.7969202	-67.43902
212794	cg11294750	45729316	45729316	0.2972092	0	1	0.5123948	72.66722
212824	cg11296826	43725393	43725393	0.4874635	0	1	0.3838736	-19.33661
212974	cg11305940	47709386	47709386	0.6536415	0	1	0.7665765	29.42354
212998	cg11307715	46738432	46738432	0.4019943	0	1	0.650975	95.6173
213101	cg11313468	45809489	45809489	0.5337873	0	1	0.6467831	23.55077
213123	cg11314617	20789330	20789330	0.7256096	0	1	0.604864	-29.66638
213146	cg11315989	67658342	67658342	0.3666238	0	1	0.2442085	-29.5028
213250	cg11322406	43709503	64619450	0.3665135	0	1	0.2457971	-28.76226
213348	cg11328149	20625410	20625410	0.6863796	0	1	0.8164043	42.76876
213382	cg11330362	67757423	67757423	0.2388374	0	1	0.1072955	-49.26899
213402	cg11331837	11745317	11745317	0.6998564	0	1	0.8185186	37.43895
213425	cg11333230	43751304	43751304	0.2859545	0	1	0.153246	-42.12817
213438	cg11334097	66706319	66706319	0.8210284	0	1	0.9213623	49.88009
213584	cg11342198	69764364	65674451	0.3197677	0	1	0.1926309	-35.12944
213610	cg11343211	47637401	23764398	0.2337108	0	1	0.1051849	-48.00745
213641	cg11344614	14770336	14770336	0.56354	0	1	0.6976743	33.41254
213693	cg11347582	66606414	66679395	0.214705	0	1	0.051269	-88.31603
213716	cg11348583	70762458	29807455	0.8345629	0	1	0.9521316	78.10484
213841	cg11356767	10795372	11779447	0.3109052	0	1	0.1965046	-29.44564
213909	cg11361344	23608346	46604473	0.4057396	0	1	0.2631176	-36.6906
214001	cg11367354	65625460	38750478	0.4485747	0	1	0.3412755	-20.99878
214136	cg11376198	36695359	39793413	0.4037376	0	1	0.2920181	-23.60924
214223	cg11381655	40714400	40714400	0.6839551	0	1	0.8118886	41.05838
214229	cg11382082	33670461	33670461	0.5971221	0	1	0.4650625	-29.25456
214241	cg11382666	54618301	61739303	0.7073964	0	1	0.5425337	-47.93173
214252	cg11383134	12727427	48709346	0.7537018	0	1	0.875077	49.44318
214263	cg11383474	46689417	46689417	0.6230831	0	1	0.5018517	-25.80768
214338	cg11387985	10603474	10603474	0.6942661	0	1	0.8051134	32.16422
214398	cg11391732	49616463	49616463	0.3523542	0	1	0.5066684	38.40882
214447	cg11395062	59607487	59607487	0.8278616	0	1	0.6676263	-61.59982
214535	cg11399582	35765460	69623425	0.6993468	0	1	0.202855	-371.3336
214548	cg11400162	65791488	65791488	0.5720056	0	1	0.3551852	-71.79395

214569	cg1140129	74707387	18753308	0.5665837	0	1	0.6696383	20.93255
214579	cg1140182	57610366	57610366	0.7641246	0	1	0.8655544	36.20969
214607	cg1140373	71720352	71720352	0.1423688	0	1	0.2488738	31.15832
214658	cg1140697	23807351	23807351	0.2142932	0	1	0.3324831	29.68023
214728	cg1141064	41707436	41707436	0.3024811	0	1	0.4080416	21.2787
214900	cg1141860	28663477	28663477	0.5315717	0	1	0.842285	374.3439
214927	cg1142014	40618306	40618306	0.540506	0	1	0.4170044	-25.71263
214959	cg1142231	15807379	15807379	0.671384	0	1	0.7850139	31.31901
214999	cg1142437	15684506	15684506	0.4703139	0	1	0.5970374	27.28437
215008	cg1142477	12730499	12730499	0.3731512	0	1	0.5462905	46.8792
215145	cg1143470	48736392	60678355	0.5202268	0	1	0.3824514	-31.16157
215159	cg1143550	47788310	12673349	0.2322841	0	1	0.0994558	-51.71298
215190	cg1143746	63779342	40684324	0.3029003	0	1	0.8135076	374.3439
215200	cg1143828	53729382	53729382	0.6394736	0	1	0.8180851	70.78357
215293	cg1144510	30735412	38697402	0.2354865	0	1	0.3383436	22.78041
215347	cg1144907	31795338	10773428	0.3144431	0	1	0.1566519	-54.79686
215407	cg1145371	39675490	39675490	0.1164709	0	1	0.2290418	37.48499
215439	cg1145689	12760376	12760376	0.4565664	0	1	0.566057	21.11667
215467	cg1145964	61650494	61741509	0.5254964	0	1	0.3684612	-39.3587
215525	cg1146342	54630353	54630353	0.6402375	0	1	0.8407581	91.53431
215598	cg1146763	49685357	49685357	0.2402808	0	1	0.0678964	-87.74922
215606	cg1146814	52715444	31789306	0.5230007	0	1	0.6413345	25.23429
215655	cg1147140	36806374	36806374	0.410064	0	1	0.5459256	30.30399
215966	cg1149560	45775369	34713473	0.5165632	0	1	0.7187785	69.5304
216175	cg1151058	29790445	29707302	0.4148569	0	1	0.2580924	-43.33859
216261	cg1151746	17608352	57733485	0.3860582	0	1	0.23376	-43.0141
216299	cg1151970	66764502	66764502	0.6843808	0	1	0.880725	102.8845
216460	cg1152832	41789306	59802488	0.5213579	0	1	0.4062291	-22.8585
216477	cg1152923	58607316	58607316	0.5099645	0	1	0.6527044	34.88763
216492	cg1153040	29795504	29795504	0.4369635	0	1	0.6105291	47.52275
216541	cg1153371	60750330	60750330	0.2999468	0	1	0.4020431	20.24658
216548	cg1153429	70761405	70761405	0.0632959	0	1	0.1754161	48.41527
216625	cg1153967	24742430	24742430	0.5260989	0	1	0.4024965	-25.7715
216648	cg1154070	71744395	71744395	0.8369718	0	1	0.693469	-53.69868
216745	cg1154720	64696506	64696506	0.7392564	0	1	0.8518692	39.611
216822	cg1155207	42614480	65626337	0.2244246	0	1	0.3318569	24.9635
216884	cg1155506	18610442	18610442	0.8982424	0	1	0.7684466	-61.66685
216940	cg1155865	62620354	62620354	0.430835	0	1	0.3002748	-30.13559
216995	cg1156231	42736499	42736499	0.6870574	0	1	0.8025177	33.94339
217018	cg1156423	46721362	46721362	0.19426	0	1	0.2977983	25.36299
217047	cg1156591	24744444	24744444	0.385607	0	1	0.2830465	-20.86672
217088	cg1156831	11631401	11631401	0.8745821	0	1	0.7721705	-38.47167
217127	cg1157112	50647374	50647374	0.4834522	0	1	0.6172727	30.30003

217190	cg1157474	66802480	66802480	0.824422	0	1	0.7238856	-29.7697
217281	cg1158079	25751411	25751411	0.4870382	0	1	0.5980086	21.91442
217324	cg1158335	19794335	19794335	0.4414143	0	1	0.558842	23.66438
217329	cg1158376	34739476	34739476	0.409167	0	1	0.3064761	-20.26987
217374	cg1158618	55749302	55749302	0.3744833	0	1	0.2322563	-38.52031
217394	cg1158706	36628408	11720427	0.1038771	0	1	0.290589	85.1457
217642	cg1160073	47754482	47754482	0.566654	0	1	0.6670715	20.0232
217688	cg1160418	55609355	55609355	0.1581142	0	1	0.2697572	31.71498
217739	cg1160815	10777317	61659390	0.5148925	0	1	0.3889622	-26.66973
217800	cg1161158	64602334	64602334	0.0595495	0	1	0.1933854	63.68414
217841	cg1161374	59635467	59635467	0.3031674	0	1	0.5103564	67.41902
217860	cg1161502	59612379	60706338	0.2673221	0	1	0.4214762	41.55375
217912	cg1161809	39746495	39746495	0.2905219	0	1	0.3920862	20.33204
217935	cg1162013	63721382	63721382	0.6299401	0	1	0.8387764	96.99706
218016	cg1162649	39632421	39632421	0.2103747	0	1	0.3158791	25.06576
218028	cg1162796	65699398	65699398	0.824706	0	1	0.9416815	71.3711
218078	cg1163093	34709464	15666478	0.3809974	0	1	0.511948	28.56818
218157	cg1163519	58689347	58611358	0.8020265	0	1	0.6635191	-44.97785
218197	cg1163796	65748382	65748382	0.4161679	0	1	0.582495	43.57742
218225	cg1163961	56766398	56766398	0.4278066	0	1	0.5285497	18.32251
218349	cg1164545	70678460	70678460	0.4358856	0	1	0.5399305	19.32096
218352	cg1164553	36627324	36627324	0.4419008	0	1	0.563723	25.19695
218469	cg1165193	11709328	11709328	0.9093309	0	1	0.796193	-54.32259
218542	cg1165549	72635435	72635435	0.312673	0	1	0.4193631	21.38647
218571	cg1165732	45643453	45643453	0.5829905	0	1	0.6855543	21.28299
218630	cg1166081	22797392	22797392	0.2373671	0	1	0.0903477	-62.6596
218658	cg1166206	39723430	39723430	0.2125247	0	1	0.3138781	23.43159
218682	cg1166339	67761493	15688477	0.5470316	0	1	0.4415186	-19.78572
218688	cg1166369	17615336	17615336	0.8144469	0	1	0.9299193	64.25076
218734	cg1166653	18631405	18631405	0.8557835	0	1	0.6737002	-83.13294
218751	cg1166738	56612305	14770408	0.9655964	0	1	0.7557905	-172.3707
218768	cg1166841	74728327	74728327	0.3628491	0	1	0.4795811	23.74049
219053	cg1168584	13703448	22787371	0.8126988	0	1	0.5727512	-113.0893
219113	cg1168975	29610312	29610312	0.4466894	0	1	0.5728208	26.78771
219168	cg1169240	58733431	58733431	0.5887049	0	1	0.7091013	28.59458
219208	cg1169504	74647411	43604473	0.162902	0	1	0.264737	27.12078
219517	cg1171250	54694405	54694405	0.415379	0	1	0.5256584	21.26088
219645	cg1171950	52666370	10666383	0.5529512	0	1	0.6724634	26.63599
219669	cg1172110	36771440	15759409	0.4348116	0	1	0.3291032	-20.72048
219723	cg1172392	34635449	34635449	0.8606858	0	1	0.6591631	-98.92348
219728	cg1172414	50745468	50745468	0.7190585	0	1	0.6058688	-26.41352
219760	cg1172541	13727428	13727428	0.3726257	0	1	0.4763756	19.48453
219812	cg1172874	26802426	26802426	0.3818157	0	1	0.4952874	22.45625

219887	cg1173313:	52698501	52698501	0.5252626	0	1	0.7438297	83.11549
219971	cg1173775:	73704494	73704494	0.6038076	0	1	0.4487953	-38.72269
220030	cg1174034:	44639397	37643418	0.056576	0	1	0.160147	44.99642
220205	cg1175073:	59636326	59636326	0.3341493	0	1	0.4378639	20.00629
220238	cg1175269:	65627450	65627450	0.2459998	0	1	0.4231149	54.40114
220356	cg1175828:	23682302	23682302	0.3927826	0	1	0.5031104	21.36239
220444	cg1176474:	39628357	39628357	0.6289418	0	1	0.4960905	-30.20888
220452	cg1176531:	68621315	68621315	0.1659584	0	1	0.2784274	31.28239
220492	cg1176934:	36795500	38669411	0.5337167	0	1	0.6932505	44.35443
220636	cg1177871:	50746371	50746371	0.4175494	0	1	0.5859791	44.61594
220669	cg1178130:	26694315	41715429	0.2800793	0	1	0.1776298	-26.15014
220711	cg1178352:	71794496	71794496	0.8121118	0	1	0.7111664	-28.59194
220723	cg1178429:	38799338	38799338	0.3288919	0	1	0.5686469	87.9016
220773	cg1178658:	20739373	20739373	0.8006346	0	1	0.6851696	-33.73335
220867	cg1179107:	19636496	19636496	0.4424365	0	1	0.834098	374.3439
220871	cg1179128:	10745380	10745380	0.5543872	0	1	0.4335872	-24.79547
221018	cg1179840:	64656334	64656334	0.4730869	0	1	0.603614	28.78766
221089	cg1180172:	13714508	58789321	0.7021484	0	1	0.5965115	-22.93473
221098	cg1180229:	35756476	35756476	0.7990912	0	1	0.6966066	-27.95412
221215	cg1180715:	64719320	64719320	0.4425535	0	1	0.5706379	27.47736
221232	cg1180810:	30622386	30622386	0.1024422	0	1	0.2030343	33.71416
221438	cg1181858:	62638468	62638468	0.6125335	0	1	0.4746457	-31.77544
221472	cg1182065:	25615372	25615372	0.792249	0	1	0.9222964	71.58326
221556	cg1182456:	27806415	27806415	0.7127539	0	1	0.8652808	66.15262
221586	cg11825731	18661402	62632378	0.4406294	0	1	0.3220358	-25.13156
221609	cg1182672:	52788439	16650359	0.5631977	0	1	0.3581339	-64.47083
221753	cg1183463:	39775431	39775431	0.4102972	0	1	0.5171019	20.17915
221779	cg1183554:	52804376	52804376	0.8373618	0	1	0.6186863	-103.9512
221861	cg1183956:	17742472	17742472	0.7519878	0	1	0.8941473	68.63291
221864	cg11839681	69605379	59741403	0.8071133	0	1	0.7050806	-28.54428
221911	cg11841771	72702348	19639374	0.3655064	0	1	0.2549031	-24.60373
221912	cg1184186:	29652450	29652450	0.8145001	0	1	0.5988466	-95.0665
222065	cg1184957:	48660405	27635396	0.8840119	0	1	0.7680746	-48.46543
222248	cg1185780:	30613358	30613358	0.7297926	0	1	0.4207316	-156.2247
222288	cg1185992:	34662471	34662471	0.2735391	0	1	0.4006304	29.85748
222311	cg1186138:	19803414	63613441	0.4194958	0	1	0.2325082	-61.06801
222328	cg1186190:	67809443	30721352	0.2940986	0	1	0.1852809	-28.07273
222499	cg1187106:	30688439	30688439	0.5853923	0	1	0.6903075	22.22757
222506	cg1187154:	13644500	13644500		0	1	0.3703268	4.34E-09
222562	cg1187497:	48786440	45700445	0.3138621	0	1	0.470516	40.4886
222612	cg1187781:	26647449	26647449	0.6896395	0	1	0.8465458	63.89029
222633	cg1187909:	30655510	30655510	0.3410534	0	1	0.4449941	19.95727
222658	cg1188103:	24635354	24635354	0.8858743	0	1	0.7713125	-48.12226

222681	cg1188235{	66678315	66678315	0.5495096	0	1	0.8864846	374.3439
222691	cg1188314l	22757330	22757330		0	1		-4.34E-09
222721	cg1188483‹	35693488	35693488	0.211885	0	1	0.3821143	54.06985
222729	cg1188535‹	13736432	71638330	0.3797682	0	1	0.515689	30.4641
222826	cg1189139‹	51682484	51682484	0.4679013	0	1	0.3277285	-33.05333
222912	cg1189762‹	51606301	51606301	0.1639939	0	1	0.2903845	37.73452
222916	cg1189788‹	41757509	41757509	0.3296389	0	1	0.2132173	-29.05287
222921	cg1189834‹	24804430	24804430	0.0211864	0	1	0.1758714	97.62872
222943	cg1190032‹	63649415	63649415	0.3272738	0	1	0.1715188	-51.43425
222945	cg1190044‹	23645312	30728493	0.3245839	0	1	0.1044071	-109.8989
222998	cg1190320‹	37679389	37679389	0.2178999	0	1	0.4146295	68.40018
223060	cg1190732‹	56729493	56729493	0.4663833	0	1	0.6076525	33.01941
223098	cg1190957‹	70734487	69691433	0.6960154	0	1	0.5334089	-46.00715
223213	cg1191728‹	28715494	28715494	0.820695	0	1	0.7107793	-33.58269
223238	cg1191882‹	44775332	44775332	0.3877787	0	1	0.4929764	19.79562
223292	cg1192392‹	16652322	16652322	0.0711275	0	1	0.3051988	135.8168
223459	cg1193417‹	51707449	21754348	0.2222522	0	1	0.0895898	-53.97507
223584	cg1194106‹	59797491	41669341	0.8531722	0	1	0.7334856	-43.63805
223600	cg1194192‹	45725411	73626375	0.4013075	0	1	0.228428	-53.75978
223607	cg1194220‹	70765391	70765391	0.8871918	0	1	0.5994335	-188.3987
223618	cg1194259‹	32810500	32810500	0.2922952	0	1	0.3963276	21.06639
223703	cg1194671‹	14668488	73718429	0.2687017	0	1	0.0662853	-111.6415
223742	cg1194890‹	14775413	14775413	0.2777216	0	1	0.4305133	40.35461
223747	cg1194917‹	55763401	55763401	0.2852994	0	1	0.3991513	24.56009
223820	cg1195316‹	26752472	26752472	0.3147408	0	1	0.4644758	37.4196
223881	cg1195644‹	51600385	51600385	0.4637512	0	1	0.0772999	-305.6266
223902	cg1195740‹	13796331	13796331	0.4789735	0	1	0.3144516	-44.08382
224115	cg1196952‹	45736441	45736441	0.3930406	0	1	0.5922818	60.98225
224201	cg1197398l	49644409	49644409	0.255354	0	1	0.4384666	56.84241
224222	cg1197539‹	61717500	61717500	0.3682247	0	1	0.2658222	-21.38336
224244	cg1197673‹	56765422	56765422	0.171478	0	1	0.2796977	28.97971
224291	cg1197987‹	28791379	28791379	0.7571641	0	1	0.6511134	-26.02304
224336	cg1198319‹	11806442	11806442	0.6049253	0	1	0.7097884	22.99569
224361	cg1198497l	37634448	37634448	0.3044581	0	1	0.4442253	33.61829
224387	cg1198664‹	25709418	25709418	0.5839757	0	1	0.3883522	-58.84389
224388	cg1198674‹	44644324	44644324	0.4896352	0	1	0.6091478	24.93563
224407	cg1198775l	11733380	65689343	0.7548022	0	1	0.9080826	82.3342
224508	cg1199335‹	62621397	50713487	0.1585534	0	1	0.5745974	374.3439
224634	cg1200245‹	55794335	55794335	0.2382256	0	1	0.3392455	22.02648
224670	cg1200541‹	47709311	47709311	0.5836288	0	1	0.6888992	22.29435
224728	cg1201047‹	35705472	35705472	0.1911765	0	1	0.2978867	26.79608
224750	cg1201242‹	66781467	66781467	0.3873818	0	1	0.7557994	374.3439
224777	cg1201418l	17657505	15640378	0.7096951	0	1	0.8675346	70.30808

224931	cg12024822	38736307	72718469	0.4791617	0	1	0.3243047	-39.34095
224941	cg12025137	23680449	23680449	0.1955618	0	1	0.3084413	28.90596
225158	cg12036633	34610328	34610328	0.941532	0	1	0.6962793	-184.1435
225293	cg12044531	73684393	73684393	0.7877888	0	1	0.8986788	49.43438
225429	cg12051819	59730440	59730440	0.267755	0	1	0.3678576	20.57042
225542	cg12058262	30635302	56774418	0.5834765	0	1	0.433304	-36.32347
225774	cg12070022	48721390	27615377	0.6813709	0	1	0.5549706	-29.55545
225806	cg12072024	71732385	71732385	0.2946308	0	1	0.4327879	33.33619
225877	cg12074485	39644373	39644373	0.876909	0	1	0.6664634	-112.3659
225923	cg12076350	71604490	71604490	0.6519912	0	1	0.5149825	-32.53362
225979	cg12078775	51701343	39654350	0.6011941	0	1	0.3789909	-75.30598
226051	cg12082015	69749311	69749311	0.245325	0	1	0.3675958	29.38582
226166	cg12089079	39701347	39701347	0.170755	0	1	0.2710976	25.86488
226265	cg12094863	13752450	13752450		0	1	0.7826383	4.34E-09
226293	cg12096487	69672368	69672368	0.2532988	0	1	0.37734	29.64859
226312	cg12097550	74670410	74670410	0.1559067	0	1	0.2783825	36.85933
226313	cg12097671	42757396	42757396	0.5819258	0	1	0.7002902	27.38888
226407	cg12102973	59743375	59743375	0.7416903	0	1	0.6261526	-28.64882
226461	cg12105899	13790458	24602442	0.4284386	0	1	0.29476	-31.54582
226517	cg12109260	74638358	51763428	0.9371647	0	1	0.8266972	-63.92368
226553	cg12111149	36794431	36794431	0.7770219	0	1	0.9027598	60.51626
226701	cg12119988	38752400	38752400	0.1402686	0	1	0.245641	30.89285
226731	cg12121446	66659507	20782356	0.5406867	0	1	0.4046572	-30.37337
226736	cg12121660	45689375	52608417	0.4414684	0	1	0.3352932	-20.75045
226773	cg12123879	64640419	64640419	0.3698711	0	1	0.2130735	-47.03159
226840	cg12127811	51740387	51740387	0.5752826	0	1	0.7228913	40.90622
226852	cg12128445	15615502	15615502	0.7799673	0	1	0.9541526	128.7396
226880	cg12130015	11796352	72630412	0.3557671	0	1	0.2305993	-31.39827
227134	cg12146763	20676448	20676448	0.4711462	0	1	0.5978984	27.30783
227342	cg12159023	19779377	57795385	0.7516051	0	1	0.6042306	-43.31899
227374	cg12160424	74764369	61763322	0.6494986	0	1	0.5410362	-22.11905
227403	cg12161971	14739431	73762406	0.7807986	0	1	0.6599059	-34.06201
227500	cg12167002	63634497	57679423	0.6179948	0	1	0.9687865	374.3439
227527	cg12168509	25749369	25749369	0.7873477	0	1	0.9156411	67.32613
227591	cg12173150	14667323	12605439	0.3969585	0	1	0.2390581	-45.25869
227653	cg12177182	32796360	28663432	0.8181521	0	1	0.6902618	-41.94897
227708	cg12179658	10600406	10600406	0.5864068	0	1	0.7526056	52.91804
228006	cg12198140	17740335	17740335	0.2668806	0	1	0.3863184	27.26792
228168	cg12208638	27767440	27767440	0.7255276	0	1	0.475769	-102.9465
228188	cg12209881	44636452	44636452	0.9290205	0	1	0.7115759	-147.9599
228246	cg12213037	23751502	23751502	0.4657535	0	1	0.6909885	81.50523
228320	cg12217954	29749437	29749437	0.6091592	0	1	0.7856293	63.2453
228353	cg12219469	24798379	24798379	0.5553163	0	1	0.6834716	30.32408

228358	cg12219707	51713307	51713307	0.584762	0	1	0.472795	-22.07262
228466	cg12226028	21727322	44763391	0.4643249	0	1	0.3330836	-29.44329
228484	cg12226948	15672418	15672418	0.2051638	0	1	0.3549879	44.39921
228568	cg12232463	48788346	48788346	0.1942945	0	1	0.337907	42.52177
228570	cg12232731	71697384	42681335	0.2495721	0	1	0.3781721	31.64234
228685	cg12241333	32689477	32689477	0.5067064	0	1	0.6824954	51.68195
228713	cg12243267	65639315	48785495	0.8131636	0	1	0.6641158	-52.32315
228827	cg12250645	40669385	40669385	0.5870113	0	1	0.7027532	26.53782
228873	cg12253071	71659403	71659403	0.41829	0	1	0.6022834	52.68311
228928	cg12255897	74711503	74711503	0.2768776	0	1	0.1605894	-33.50395
228969	cg12258179	15655389	20620474	0.5928235	0	1	0.4618432	-28.78673
228992	cg12259892	53781503	53781503	0.518872	0	1	0.6353485	24.44138
229112	cg12269972	43644410	43644410	0.3815855	0	1	0.2302317	-42.85474
229134	cg12271317	10739502	10739502	0.5300159	0	1	0.6900672	44.39683
229254	cg12279734	32767338	32767338	0.3943494	0	1	0.7424559	374.3439
229492	cg12296550	28660318	28660318	0.5874946	0	1	0.6982366	24.53968
229515	cg12297590	50692476	66741323	0.6067707	0	1	0.4938032	-22.69405
229631	cg12303318	36649384	36649384	0.1319913	0	1	0.2882963	58.42113
229642	cg12303813	51607504	51607504	0.3352495	0	1	0.4517303	24.11384
229687	cg12307373	49606365	49606365	0.5745502	0	1	0.6944543	27.68917
229736	cg12310025	72708489	39781349	0.6523412	0	1	0.1856884	-371.3336
229777	cg12312457	30631400	39730423	0.3335862	0	1	0.2012448	-36.65305
229880	cg12321669	29698322	29698322	0.8634787	0	1	0.6432385	-114.5984
230113	cg12341429	16642311	16642311	0.6385858	0	1	0.8608939	115.2316
230121	cg12342501	18752444	18752444	0.6483566	0	1	0.8546035	100.3088
230361	cg12360046	70788465	42634485	0.2757527	0	1	0.3880798	24.36982
230389	cg12361987	14683391	14683391	0.4895566	0	1	0.6005669	21.96326
230493	cg12370174	58620371	58620371	0.4554617	0	1	0.6356357	51.59139
230601	cg12377816	55643407	55643407	0.1596576	0	1	0.3013085	45.74591
230680	cg12383699	64618426	60658391	0.3554719	0	1	0.243134	-25.77213
230727	cg12386614	42678409	42678409	0.2089041	0	1	0.6983482	374.3439
230753	cg12388523	15780459	15780459	0.4250042	0	1	0.2337547	-63.37432
230803	cg12391372	17745430	17745430	0.0444328	0	1	0.4683039	374.3439
230805	cg12391576	47783490	47783490	0.8604262	0	1	0.9612546	69.82124
230820	cg12392475	67717362	45613312	0.6544989	0	1	0.5539746	-19.72447
230876	cg12395823	42695344	11610410	0.679946	0	1	0.7893981	29.95971
230961	cg12400864	67609391	67609391	0.765239	0	1	0.6404853	-34.27985
230984	cg12401953	23721353	23721353	0.5322771	0	1	0.6386269	21.17458
231001	cg12403162	37764433	37764433	0.0980902	0	1	0.2093853	39.9729
231072	cg12406683	39642444	39642444	0.4649148	0	1	0.6212908	39.72307
231137	cg12410980	63628360	74804390	0.8181513	0	1	0.9327269	64.89903
231169	cg12412775	64764403	64764403	0.2373906	0	1	0.4105788	53.08447
231244	cg12417704	50725348	50627440	0.8173519	0	1	0.6658628	-54.42774

231254	cg1241804[illegible]	15670433	15670433	0.2431093	0	1	0.3437491	21.68545
231277	cg1241938[illegible]	38641411	13767478	0.4978532	0	1	0.3972379	-18.36427
231291	cg1242010[illegible]	64652348	45732372	0.4109531	0	1	0.2727539	-34.32194
231303	cg1242108[illegible]	74634484	74634484	0.7604606	0	1	0.9074767	77.42461
231326	cg1242253[illegible]	12612468	29704333	0.5179629	0	1	0.6632922	36.39559
231390	cg1242586[illegible]	52664318	52664318	0.0629725	0	1	0.1797269	51.40348
231416	cg1242716[illegible]	57684372	27774501	0.3194048	0	1	0.4341889	23.88989
231465	cg1243077[illegible]	11781506	11781506	0.2257702	0	1	0.0836729	-61.42344
231503	cg1243280[illegible]	10741417	10741417	0.6342942	0	1	0.8339296	88.88631
231545	cg1243488[illegible]	20673311	20673311	0.1861326	0	1	0.2979376	29.20927
231546	cg1243489[illegible]	20616326	20616326	0.871666	0	1	0.6288278	-137.207
231681	cg1244224[illegible]	17757417	65807459	0.3508424	0	1	0.095214	-143.543
231728	cg1244484[illegible]	39719478	59656491		0	1		-4.34E-09
231825	cg1245001[illegible]	26697303	26697303	0.5021087	0	1	0.6642054	43.92389
231887	cg1245362[illegible]	64643493	64643493	0.4141798	0	1	0.5524747	31.2623
231896	cg1245416[illegible]	55636429	55636429	0.8174929	0	1	0.497235	-190.3397
231995	cg1246013[illegible]	47668470	56640492	0.5295671	0	1	0.4190632	-21.32851
232135	cg1246877[illegible]	65744355	33762399	0.182422	0	1	0.0311707	-89.02635
232142	cg1246947[illegible]	44602352	24782322	0.3438418	0	1	0.149825	-78.03783
232282	cg1247818[illegible]	24720423	24720423	0.2547732	0	1	0.374206	27.82075
232290	cg1247844[illegible]	51764498	72769417	0.8481046	0	1	0.6114407	-122.032
232362	cg1248300[illegible]	53609398	53609398	0.4711101	0	1	0.5909411	24.76773
232413	cg1248518[illegible]	37798323	33742393	0.5410249	0	1	0.4145313	-26.78573
232562	cg1249376[illegible]	15634478	15634478	0.3206894	0	1	0.4260614	20.78679
232569	cg1249420[illegible]	61705307	61705307	0.5691471	0	1	0.7225373	43.57268
232677	cg1250030[illegible]	19792426	19792426	0.3302423	0	1	0.5285039	61.0834
232697	cg1250128[illegible]	16765481	16765481	0.6525944	0	1	0.4227051	-81.99045
232701	cg1250176[illegible]	15663356	10749417	0.7574485	0	1	0.6562065	-24.23962
232769	cg1250508[illegible]	33715503	33715503	0.8734401	0	1	0.7489312	-50.9059
232868	cg1251099[illegible]	50637410	50637410	0.5605531	0	1	0.6981315	34.86389
232939	cg1251565[illegible]	21610491	21610491	0.458475	0	1	0.8401865	374.3439
233052	cg1252397[illegible]	10612336	69641414	0.231195	0	1	0.1102482	-42.87266
233093	cg1252629[illegible]	62624444	62624444		0	1		-4.34E-09
233192	cg1253209[illegible]	51755472	51755472	0.377305	0	1	0.4950498	23.91628
233267	cg1253700[illegible]	60713304	60713304	0.2052492	0	1	0.3113556	25.60782
233309	cg1253941[illegible]	58609470	58609470	0.9002162	0	1	0.7997731	-43.35353
233388	cg1254635[illegible]	61744370	67790412	0.5637949	0	1	0.4237195	-32.00613
233420	cg1254817[illegible]	37622434	37622434	0.3010996	0	1	0.4311589	29.91558
233559	cg1255656[illegible]	63705396	63705396	0.2354085	0	1	0.0740663	-77.42553
233645	cg1256282[illegible]	25616402	66711311	0.6710239	0	1	0.4630792	-68.93953
233655	cg1256352[illegible]	59700446	59700446	0.6180523	0	1	0.7206977	22.72734
233702	cg1256578[illegible]	36716357	36716357	0.3064352	0	1	0.4096504	20.43506
233758	cg1256988[illegible]	11794464	11794464	0.8452786	0	1	0.577348	-148.3084

233796	cg1257266…	21613381	21613381	0.1554521	0	1	0.2671196	32.00587
233921	cg1258089…	61808327	61808327	0.331181	0	1	0.1955642	-38.65339
233929	cg1258129…	29693368	29693368	0.2487326	0	1	0.3505445	21.84284
234009	cg1258496…	55799317	55799317	0.701965	0	1	0.5771188	-29.93514
234032	cg1258638…	21646472	10807359	0.608285	0	1	0.5066509	-19.15657
234136	cg1259166…	67703325	67703325	0.6352931	0	1	0.7802215	45.3097
234147	cg1259236…	57675375	57675375	0.0795845	0	1	0.1933029	45.20319
234163	cg1259322…	33788324	33788324	0.3788313	0	1	0.4916551	22.27168
234254	cg1259761…	30728473	30728473	0.7808426	0	1	0.6633936	-32.56305
234321	cg1260053…	13627485	13627485	0.3240187	0	1	0.4368273	23.12035
234419	cg1260669…	62677356	62677356	0.1670757	0	1	0.3070486	43.90762
234531	cg1261240…	42723447	42723447	0.6132676	0	1	0.7173205	23.05624
234644	cg1261827…	44631474	44631474	0.1946709	0	1	0.298601	25.48338
234647	cg1261858…	12792490	12792490	0.6524761	0	1	0.7715274	32.38957
234669	cg1261974…	24654347	24654347	0.4721056	0	1	0.8392389	374.3439
234715	cg1262265…	12675381	12675381	0.9437426	0	1	0.6319389	-257.1492
234729	cg1262314…	27656419	73632474	0.4473141	0	1	0.3422014	-20.29995
234902	cg1263315…	50649400	56766334	0.0738524	0	1	0.3824774	374.3439
234933	cg1263512…	15798509	15798509	0.3843247	0	1	0.5250856	32.32751
234959	cg1263649…	66659489	66659489	0.3902459	0	1	0.4915746	18.61574
234975	cg1263744…	60623484	60623484	0.1565736	0	1	0.2615962	29.02588
235061	cg1264271…	36748372	17689406	0.8883985	0	1	0.7500029	-64.07191
235295	cg1265576…	18755483	18755483	0.6627605	0	1	0.7978751	42.69756
235305	cg12656291	44615392	44615392	0.3949992	0	1	0.5099523	22.84931
235333	cg1265741…	36650391	36650391	0.2484756	0	1	0.4137904	48.14706
235352	cg1265892…	68601326	68601326	0.6225815	0	1	0.7304665	25.08078
235519	cg1266852…	10768401	37791311	0.175063	0	1	0.2804157	27.50661
235561	cg1267163…	66621406	66621406	0.3890637	0	1	0.6626118	116.0225
235600	cg1267558…	45738312	45738312	0.4670207	0	1	0.336539	-29.07903
235672	cg1268140…	16744343	16744343	0.1858859	0	1	0.2892313	25.87789
235719	cg1268405…	63666330	69645424	0.6252205	0	1	0.504581	-25.64703
235728	cg1268495…	25719432	25719432	0.2632972	0	1	0.3928087	31.28075
235859	cg1269206…	19807430	19807430	0.2921481	0	1	0.4181414	28.69495
235921	cg1269733…	32605374	18611433	0.6735914	0	1	0.5009858	-49.52338
235934	cg1269862…	45734417	45734417	0.4273204	0	1	0.543625	23.23575
236166	cg1271528…	56624398	17604473	0.5683655	0	1	0.4555622	-22.19448
236206	cg1271858…	59674465	59674465	0.1356094	0	1	0.2415288	31.68273
236220	cg1271984…	69677404	69677404	0.1919239	0	1	0.3152707	33.5469
236221	cg1271990…	20778318	20778318	0.6147985	0	1	0.7347751	29.94304
236321	cg1272736…	60634435	60634435	0.9010549	0	1	0.5246919	-305.8616
236333	cg1272794…	67795441	67795441	0.1491573	0	1	0.2598146	32.24471
236469	cg1273687…	10770349	50650477	0.204291	0	1	0.3450457	40.18801
236587	cg1274341…	66736391	66736391	0.6779101	0	1	0.4572176	-77.44901

236679	cg12748890	17670473	47654449	0.0883348	0	1	0.192641	38.08446
236988	cg12770425	38801326	38801326	0.7651343	0	1	0.5603232	-77.33035
237042	cg12775223	27621489	27621489	0.8378477	0	1	0.6912586	-55.66658
237371	cg12798157	24768440	62638473	0.3104047	0	1	0.1926187	-31.13832
237569	cg12807764	26801402	26801402	0.3740569	0	1	0.4878676	22.64019
237682	cg12813768	16676310	16676310	0.4448159	0	1	0.3321611	-22.89616
237692	cg12814117	53619503	69695446	0.3899815	0	1	0.6073291	72.22063
237764	cg12819537	19709454	19709454	0.2027446	0	1	0.5539941	374.3439
237877	cg12829045	29706324	29706324	0.1842989	0	1	0.3769988	70.70338
237950	cg12833683	69732368	69732368	0.8507268	0	1	0.5553244	-177.432
238100	cg12844324	17775456	17775456	0.4500162	0	1	0.5551679	19.70662
238137	cg12846656	31662374	31662374	0.3302405	0	1	0.4362056	20.77906
238198	cg12850379	41671327	30766302	0.4789055	0	1	0.3266447	-38.13328
238199	cg12850396	62624375	62624375	0.822189	0	1	0.7003076	-39.53596
238254	cg12854428	17691497	17691497	0.5011985	0	1	0.6288797	28.26541
238257	cg12854611	51755382	51755382	0.408858	0	1	0.5240834	22.88312
238286	cg12856521	38625322	38625322	0.2615845	0	1	0.4380583	52.83631
238317	cg12858166	24803314	24803314	0.2578575	0	1	0.67059	374.3439
238327	cg12858577	48618427	35705303	0.5790612	0	1	0.4334181	-34.37719
238412	cg12865025	19674471	19674471	0.424895	0	1	0.6315171	66.18296
238456	cg12867728	65606362	65606362	0.2740432	0	1	0.4263207	40.32127
238464	cg12868067	39648318	39735482	0.1729416	0	1	0.0458944	-64.07825
238465	cg12868173	42635367	30607507	0.2266878	0	1	0.0911066	-55.35735
238482	cg12869097	41744326	41744326	0.9328297	0	1	0.8327449	-54.16114
238502	cg12870217	72752369	72752369	0.9160991	0	1	0.6953551	-142.0941
238648	cg12880095	59650300	64739460	0.8581001	0	1	0.7286758	-50.14249
238800	cg12890750	49745331	28793481	0.4633369	0	1	0.3191287	-34.94659
238848	cg12894371	26658403	26658403	0.4291601	0	1	0.5354218	19.99329
239038	cg12906381	62636340	14765369	0.6773036	0	1	0.5474749	-30.70189
239202	cg12916580	58790404	62624315	0.1312605	0	1	0.2896021	59.76602
239354	cg12927641	68672507	24708479	0.9037697	0	1	0.7642641	-70.45378
239477	cg12934884	11642389	68758402	0.194939	0	1	0.0906306	-37.65064
239508	cg12936992	52695442	52695442	0.6925316	0	1	0.8353965	53.01299
239518	cg12937413	65750346	65750346	0.1827579	0	1	0.2892069	27.32714
239552	cg12939425	61804397	16640327	0.8230531	0	1	0.7099686	-35.37945
239677	cg12947138	68707481	68707481	0.2015841	0	1	0.3289842	34.46176
239799	cg12955305	69802385	69802385	0.8857693	0	1	0.7829531	-41.13615
239807	cg12955789	26792344	56703446	0.6833653	0	1	0.5333623	-39.39597
239910	cg12964420	62633339	62633339	0.3884577	0	1	0.5181441	28.04102
240032	cg12972705	51653422	51653422	0.3839129	0	1	0.5149442	28.57364
240161	cg12980549	66767338	66767338	0.6198227	0	1	0.7344789	27.84657
240178	cg12981577	28711442	28711442	0.3982684	0	1	0.5218071	25.76571
240379	cg12995004	21612374	21612374	0.3028207	0	1	0.4154733	23.60378

240425	cg1299908	57650306	57650306	0.3265461	0	1	0.4346171	21.52066
240430	cg1299926	39645503	39645503	0.4426917	0	1	0.5430561	18.23011
240485	cg1300250	34656427	34656427	0.6148694	0	1	0.7243883	25.34867
240510	cg1300450	43734355	12744437	0.077142	0	1	0.177571	38.21029
240562	cg1300833	67763423	67763423	0.1175546	0	1	0.298102	76.95275
240584	cg1300981	15796314	15796314	0.4901381	0	1	0.6058751	23.5974
240602	cg1301079	65732385	65732385	0.2023934	0	1	0.0673648	-61.83634
240605	cg1301100	65677367	65677367	0.7319189	0	1	0.841349	36.23831
240700	cg1301702	52689403	52689403	0.6346708	0	1	0.4963047	-32.51705
240710	cg1301792	74607403	74607403	0.5610073	0	1	0.7873926	96.40231
240733	cg1301955	20649506	58748301	0.0878536	0	1	0.2293449	59.863
240773	cg1302253	29702360	29702360	0.3207839	0	1	0.424296	20.2031
240779	cg1302290	74729492	42717468	0.8000829	0	1	0.692127	-30.36595
240807	cg1302451	48753301	14609420	0.9305454	0	1	0.7328062	-131.4557
240902	cg1303102	61688421	61688421	0.5402336	0	1	0.6475101	21.68321
240943	cg1303385	18605367	18605367	0.600883	0	1	0.7300901	33.38311
240994	cg1303724	43731416	43731416	0.1780646	0	1	0.3150239	41.11062
241101	cg1304535	23605470	23605470	0.5015422	0	1	0.381022	-24.82855
241118	cg1304660	18809380	18809380	0.2076392	0	1	0.4078858	71.88676
241126	cg1304715	42694384	42694384	0.3725342	0	1	0.2117337	-49.17806
241136	cg1304786	40640376	68808498	0.8200174	0	1	0.6077039	-94.10555
241224	cg1305263	49751453	49751453	0.6476868	0	1	0.761356	29.31162
241260	cg1305435	18653470	18653470	0.2913803	0	1	0.4068471	24.91786
241311	cg1305845	46747447	46747447	0.3834266	0	1	0.5101224	26.98696
241318	cg1305881	20716341	15736324	0.6132072	0	1	0.4779482	-30.76564
241357	cg1306146	31625467	31625467	0.3500343	0	1	0.4642403	23.08529
241440	cg1306646	74730398	74730398	0.5886862	0	1	0.7970328	85.72559
241466	cg1306763	73615343	73615343	0.4289751	0	1	0.5303132	18.49901
241472	cg1306797	72755340	72755340	0.6177325	0	1	0.9060673	374.3439
241520	cg1307150	74728372	74728372	0.7485004	0	1	0.953283	156.5356
241606	cg1307678	63802308	63802308	0.8379873	0	1	0.7371871	-31.58792
241639	cg1307838	67681305	10652505	0.4138924	0	1	0.2926397	-26.93168
241645	cg1307879	67702356	67702356	0.448294	0	1	0.2861601	-44.2113
241680	cg1308056	34620490	68688337	0.5383705	0	1	0.3717897	-43.71228
241697	cg1308149	62689375	62689375	0.4816394	0	1	0.3655651	-23.48548
241974	cg1309942	10639300	10639300	0.5025279	0	1	0.37742	-26.47146
241989	cg1310019	73796403	73796403	0.7368536	0	1	0.6069959	-34.1421
242154	cg1311240	69686420	49608353	0.824861	0	1	0.6909717	-46.12651
242348	cg1312792	14629366	14629366	0.820268	0	1	0.6605777	-59.70412
242437	cg1313444	35631470	35631470	0.0841207	0	1	0.3074593	120.5619
242497	cg1313780	60688366	70753505	0.5851917	0	1	0.3149155	-112.2526
242501	cg1313808	10675383	71751402	0.3046832	0	1	0.4264278	26.71846
242553	cg1314213	40624323	40624323	0.6875776	0	1	0.4936472	-61.85572

242742	cg1315370{	41729481	41729481	0.1620166	0	1	0.2637346	27.15123
242844	cg1316085[illegible]	56732304	56732304	0.1869043	0	1	0.5741636	374.3439
242947	cg1316804[illegible]	44665366	48645411	0.3692113	0	1	0.2675754	-21.07175
242963	cg1316869{	54790415	54790415	0.676715	0	1	0.7877169	30.45659
242973	cg1316913[illegible]	28732384	17730383	0.6769601	0	1	0.5101209	-46.86648
242974	cg1316922[illegible]	50629351	50629351	0.4567163	0	1	0.5801847	25.90859
243016	cg1317167[illegible]	34693303	34693303	0.5349425	0	1	0.663837	29.79998
243045	cg1317339[illegible]	72711319	72711319	0.6115093	0	1	0.712408	21.76206
243135	cg1317859[illegible]	38711495	38711495	0.4566972	0	1	0.5637735	20.34567
243217	cg1318365[illegible]	24757360	24757360	0.6595852	0	1	0.8720126	111.849
243229	cg1318444{	30610477	30610477	0.6373649	0	1	0.771579	39.15033
243250	cg1318541[illegible]	68665427	33656307	0.0418245	0	1	0.2591177	137.6707
243256	cg1318598[illegible]	11653303	11653303	0.7248683	0	1	0.8369176	36.85909
243552	cg1320552{	18746348	18746348	0.3223837	0	1	0.1977947	-33.57412
243586	cg1320732[illegible]	65656400	58709472	0.4376158	0	1	0.3182116	-25.50939
243666	cg1321100{	13615312	13615312	0.6626372	0	1	0.7652709	25.27894
243700	cg1321353[illegible]	20774335	20774335	0.6601167	0	1	0.8046049	48.46562
243802	cg1321940[illegible]	51703337	51703337	0.8864864	0	1	0.7298713	-75.86552
243805	cg1321955[illegible]	30733342	30733342	0.4512776	0	1	0.5818401	28.49384
243848	cg1322192[illegible]	60630370	60630370	0.5352118	0	1	0.659956	28.10087
243913	cg1322556[illegible]	53699406	53630336	0.500696	0	1	0.6032282	19.35503
244002	cg1323020{	39736499	39736499	0.5028976	0	1	0.3912541	-21.79306
244024	cg1323207[illegible]	48807345	48807345	0.1999595	0	1	0.3888256	66.04156
244067	cg1323509{	55804302	55804302	0.5837803	0	1	0.7163266	33.85787
244114	cg1323847[illegible]	24692327	24692327	0.5156854	0	1	0.6503743	31.56018
244414	cg1325749{	39804309	39804309	0.1733861	0	1	0.2748803	26.10817
244419	cg1325769(	17725308	48652391	0.2158021	0	1	0.321913	24.96747
244500	cg1326332[illegible]	60798335	60798335	0.7537975	0	1	0.8834415	56.81323
244533	cg1326481[illegible]	66687414	66687414	0.5061541	0	1	0.7496372	101.8276
244597	cg1327005[illegible]	61675434	61675434	0.4827353	0	1	0.3316756	-37.47223
244677	cg1327512[illegible]	67743403	67743403	0.6405745	0	1	0.4957344	-35.33458
244777	cg1328052[illegible]	13644367	13644367		0	1	0.6855243	4.34E-09
244827	cg1328395[illegible]	28728410	21693303	0.2244088	0	1	0.1173206	-34.65089
244977	cg1329348{	10679379	45674507	0.3751965	0	1	0.4837176	20.93094
245005	cg1329508[illegible]	59763340	59763340	0.6275535	0	1	0.7278925	22.2757
245191	cg1330544[illegible]	22750457	22750457	0.2002576	0	1	0.4274304	90.86579
245202	cg1330608[illegible]	31664352	31664352	0.7794716	0	1	0.6725878	-28.02226
245209	cg1330681[illegible]	19670468	19670468	0.692622	0	1	0.8432845	59.28519
245282	cg1331238[illegible]	71734364	71734364	0.1451826	0	1	0.2808894	44.79698
245333	cg1331514[illegible]	44686337	44686337	0.3344813	0	1	0.4546334	25.39142
245347	cg1331605[illegible]	34636377	34636377	0.2041236	0	1	0.318793	28.99088
245373	cg1331681[illegible]	32773489	32773489	0.2058591	0	1	0.0530169	-79.64192
245424	cg1331976[illegible]	44669307	44669307	0.8777326	0	1	0.6065354	-166.4706

245459	cg1332110!	71768423	71768423	0.3539111	0	1	0.4588709	20.07572
245507	cg1332387!	43608313	10720370	0.8962931	0	1	0.7939833	-43.44407
245582	cg1332791!	12684348	12684348	0.5919544	0	1	0.7789523	68.4354
245588	cg1332820!	57655330	42684337	0.4706749	0	1	0.5897218	24.48362
245615	cg1333086!	17795352	20737304		0	1	0.2473243	4.34E-09
245644	cg1333214!	37674436	37674436	0.6508198	0	1	0.7590081	26.97124
245766	cg1333894!	36617380	36617380	0.4346221	0	1	0.602437	44.56366
245791	cg1334089!	15707495	15707495	0.8783576	0	1	0.7709628	-42.06691
245900	cg1334739!	46672501	46672501	0.8306066	0	1	0.9389238	63.13958
245962	cg1335186!	43675447	43675447	0.5000463	0	1	0.6067606	20.69108
245999	cg1335371!	46781383	46781383	0.5884622	0	1	0.3811899	-65.731
246041	cg1335625!	42751506	57649361	0.2877034	0	1	0.4071747	26.46122
246055	cg1335689!	63763425	15707487	0.3943298	0	1	0.2667271	-30.36616
246100	cg1335999!	29763477	29763477	0.4625971	0	1	0.7149005	103.5306
246101	cg1336000!	73724421	73724421	0.4714031	0	1	0.5770193	19.99574
246206	cg1336540!	61745505	61745505	0.528227	0	1	0.6413038	23.43654
246233	cg1336677!	45710478	45710478	0.3664874	0	1	0.4858359	24.58109
246236	cg1336686!	62607382	62607382	0.4858223	0	1	0.5976356	22.1815
246251	cg1336788!	35644510	35644510	0.2835798	0	1	0.1620688	-35.69932
246398	cg1337546!	62719409	62719409	0.2582768	0	1	0.3641994	22.8422
246434	cg1337696!	31640450	30715301	0.7026455	0	1	0.3700519	-178.0362
246441	cg1337726!	17690478	17690478	0.2983812	0	1	0.1882675	-28.35768
246505	cg1338089!	56660355	56660355	0.7716054	0	1	0.5497401	-89.96902
246509	cg1338111!	20648364	20648364	0.3611884	0	1	0.4857099	26.45777
246689	cg1339107!	54702388	63745430	0.6398894	0	1	0.5395279	-19.33848
246896	cg1340024!	41720443	41720443	0.4257692	0	1	0.5460528	24.59284
246935	cg1340189!	47792379	39659478	0.0424393	0	1	0.3821086	374.3439
247049	cg1340802!	50619367	50619367	0.4263614	0	1	0.539596	22.21444
247110	cg1341128!	25692487	25692487	0.4181468	0	1	0.5414911	25.65726
247156	cg1341371!	32644368	32644368	0.6982208	0	1	0.8153481	36.28014
247180	cg1341507!	12740426	12740426	0.6437503	0	1	0.7600432	30.27494
247391	cg1342596!	30702347	30702347	0.3943378	0	1	0.4980367	19.29848
247496	cg1343067!	64741438	64741438	0.7082282	0	1	0.8224811	35.8595
247522	cg1343168!	48649402	48649402	0.7551338	0	1	0.2579772	-371.3336
247610	cg1343513!	39755502	39755502	0.9036121	0	1	0.6975042	-121.9278
247711	cg1343994!	16700406	15807510	0.6716674	0	1	0.7728276	25.25323
247733	cg1344110!	25778496	25778496	0.8066393	0	1	0.9144312	52.49877
247897	cg1344729!	62628313	49742397	0.7935605	0	1	0.6898898	-27.93454
247915	cg1344819!	43789481	12730440	0.1864866	0	1	0.0693413	-49.84581
248015	cg1345281!	44787453	44787453	0.3520457	0	1	0.6197797	109.6641
248049	cg1345422!	19696331	19696331	0.425963	0	1	0.5381626	21.87603
248112	cg1345752!	24638403	24638403	0.584585	0	1	0.7059832	28.81442
248122	cg1345800!	27735482	36709474	0.7316374	0	1	0.6297396	-23.00321

248228	cg1346255[illegible]	37731366	37731366	0.8094186	0	1	0.5189734	-156.6134
248335	cg1346691[illegible]	38778399	38778399	0.5680251	0	1	0.6785354	23.69089
248369	cg1346821[illegible]	41673352	41673352	0.5238411	0	1	0.6451255	26.37841
248443	cg1347118[illegible]	18756308	18756308	0.3466837	0	1	0.5041823	39.93119
248445	cg1347125[illegible]	29732479	29732479	0.6509331	0	1	0.7594452	27.12782
248454	cg1347173[illegible]	39803450	39803450	0.6435589	0	1	0.7467491	24.29784
248508	cg1347426[illegible]	52638466	52638466	0.715217	0	1	0.9308317	147.5047
248718	cg1348460[illegible]	20602304	57658429	0.3057357	0	1	0.4166789	22.95046
248853	cg1349023[illegible]	64762462	64762462	0.3391007	0	1	0.4951569	39.46295
249012	cg1349675[illegible]	61637457	61637457	0.928196	0	1	0.8202918	-57.90193
249048	cg1349875[illegible]	39792356	49742459	0.4447905	0	1	0.2041763	-99.99402
249113	cg1350190[illegible]	35694441	35694441	0.4199907	0	1	0.316563	-20.26728
249199	cg1350628[illegible]	66615479	66615479	0.9358892	0	1	0.8158821	-70.64651
249230	cg1350789[illegible]	13644391	13644391		0	1		-4.34E-09
249411	cg1351836[illegible]	12695312	12695312	0.6191549	0	1	0.4642755	-39.00537
249462	cg1352053[illegible]	38730337	33620350	0.8663632	0	1	0.7172652	-64.25539
249466	cg1352077[illegible]	57808455	57808455	0.3721836	0	1	0.5444163	46.44032
249500	cg1352194[illegible]	14787487	14787487	0.1161373	0	1	0.2356167	41.06459
249547	cg1352371[illegible]	57794434	57794434	0.2185081	0	1	0.042248	-102.859
249548	cg1352373[illegible]	66664363	66664363	0.473844	0	1	0.6172462	34.0895
249680	cg1353032[illegible]	66646497	66646497	0.7936878	0	1	0.9116921	58.48505
249713	cg1353166[illegible]	29803329	29803329	0.2482448	0	1	0.3873014	36.04713
249847	cg1353857[illegible]	57745326	57745326	0.3767518	0	1	0.6253238	94.39517
249901	cg1354135[illegible]	27690439	21763337	0.2660766	0	1	0.3975604	31.92614
249916	cg1354197[illegible]	25616349	25616349	0.5995367	0	1	0.4978302	-19.05742
250042	cg1354855[illegible]	46607455	46607455	0.4777049	0	1	0.5987106	25.28197
250092	cg1355107[illegible]	73747308	73747308	0.5623506	0	1	0.7087646	39.34459
250409	cg1356445[illegible]	21762471	21762471	0.4761512	0	1	0.5768102	18.50309
250412	cg1356452[illegible]	11616419	11616419	0.6064156	0	1	0.3686834	-86.08218
250459	cg1356646[illegible]	63681301	63681301	0.8767838	0	1	0.700201	-86.4728
250549	cg1357089[illegible]	72672431	72672431	0.4269825	0	1	0.5724057	34.22899
250615	cg1357337[illegible]	46721383	46721383	0.671468	0	1	0.4502768	-77.31814
250720	cg1357816[illegible]	67712382	70778385	0.3905179	0	1	0.27957	-23.75786
250772	cg1358028[illegible]	34644314	34644314	0.2583469	0	1	0.3586069	20.95476
250786	cg1358075[illegible]	74636371	74636371	0.2432755	0	1	0.3920906	40.68531
250799	cg1358115[illegible]	64768399	64768399	0.4070688	0	1	0.2912292	-25.05288
250832	cg1358245[illegible]	41775406	41775406	0.2813849	0	1	0.60901	374.3439
250865	cg1358366[illegible]	73671306	66714354	0.1112595	0	1	0.2146284	33.73349
250896	cg1358574[illegible]	49600485	60740487	0.6913523	0	1	0.3598379	-175.723
250908	cg1358645[illegible]	41752475	41752475	0.3624995	0	1	0.4647694	19.14859
251036	cg1359339[illegible]	64652509	60798489	0.618108	0	1	0.5167363	-19.23168
251095	cg1359637[illegible]	59628448	62637351	0.6758426	0	1	0.5693559	-22.24474
251222	cg1360199[illegible]	39620393	36651379	0.4650169	0	1	0.5857798	25.02557

251249	cg1360331{	73652478	73652478	0.2887954	0	1	0.08326	-106.5949
251260	cg1360387{	13715484	18624335	0.2717901	0	1	0.1432734	-41.43358
251303	cg1360602{	73649470	73649470	0.8551086	0	1	0.7423993	-40.25003
251387	cg1361013{	21652347	21652347	0.2715658	0	1	0.3792762	22.94228
251394	cg1361045{	51627488	51627488	0.2681685	0	1	0.4120885	36.9691
251515	cg1361599{	23611369	70657374	0.5492647	0	1	0.3589099	-56.02069
251549	cg1361760{	28731396	28731396	0.5009352	0	1	0.6141092	22.88663
251618	cg1362003{	64629354	64629354	0.5550408	0	1	0.6651064	23.09389
251676	cg1362289{	18704435	18704435	0.4055986	0	1	0.3001199	-21.3148
251831	cg1363131{	47616465	47616465	0.420254	0	1	0.5717996	36.80696
252016	cg1364164{	49614330	49614330	0.2836403	0	1	0.4585276	50.59103
252191	cg1364986{	13798483	13798483	0.5426722	0	1	0.6427079	19.30005
252217	cg1365120{	20695422	20695422	0.7046109	0	1	0.5271402	-54.16187
252233	cg1365200{	73691307	73691307	0.2014134	0	1	0.312569	27.79814
252325	cg1365598{	25619417	25619417	0.8312378	0	1	0.9450247	70.69141
252438	cg1366164{	70649379	70649379	0.2743409	0	1	0.6024499	374.3439
252601	cg1367028{	63774398	63803416	0.2389484	0	1	0.3459263	24.05504
252623	cg1367141{	15647332	15647332	0.9114727	0	1	0.7889261	-61.55375
252643	cg1367220{	45645324	45645324	0.750173	0	1	0.5334108	-82.84312
252657	cg1367273{	20690433	10609506	0.9300014	0	1	0.6876105	-172.0215
252921	cg1368393{	11708424	11708424	0.6996031	0	1	0.8100316	32.53079
252930	cg1368437{	46808320	46808320	0.8080493	0	1	0.934418	74.82985
252950	cg1368534{	31701488	31701488	0.9122123	0	1	0.696952	-134.7909
252952	cg1368550{	45677454	45677454	0.595244	0	1	0.7241929	32.8773
252982	cg1368673{	15614366	15614366	0.2548662	0	1	0.3616341	23.26995
253016	cg1368818{	47755386	47755386	0.7375128	0	1	0.6157336	-30.84851
253077	cg1369054{	20743482	20743482	0.1031361	0	1	0.2143222	38.99758
253166	cg1369468{	73687362	73687362	0.3374357	0	1	0.4375544	18.85917
253239	cg1369816{	64769494	64769494	0.1501924	0	1	0.2538731	29.08948
253264	cg1369915{	55619346	65658365	0.2108116	0	1	0.1096026	-32.92911
253290	cg1370045{	27626458	27626458	0.7954698	0	1	0.9084336	53.89686
253404	cg1370605{	10640380	67719307	0.377728	0	1	0.2462045	-33.01966
253433	cg1370756{	33610388	33610388	0.2540613	0	1	0.4117617	44.05107
253522	cg1371131{	52794450	19687301	0.5782614	0	1	0.7286152	42.63945
253550	cg1371321{	57666459	24714498	0.6558367	0	1	0.4809974	-49.71798
253627	cg1371676{	12705336	65645460	0.239952	0	1	0.3579205	28.02932
253778	cg1372411{	64661376	64661376	0.6528854	0	1	0.8216063	65.39885
253793	cg1372455{	73734496	73734496	0.4570354	0	1	0.5777108	24.90958
253804	cg1372508{	46769405	46769405	0.7650678	0	1	0.5717542	-70.07537
253867	cg1372794{	62699392	62699392	0.2160749	0	1	0.3170114	23.08776
253869	cg1372800{	70610397	70610397	0.4544662	0	1	0.5708027	23.37627
253908	cg1372981{	60602308	17792313	0.4921111	0	1	0.3362585	-39.44399
254012	cg1373546{	40754500	46743332	0.741776	0	1	0.5479232	-67.16181

254066	cg1373872	20662462	20662462	0.5639425	0	1	0.6822518	26.57882
254095	cg1374063	37775382	37775382	0.615212	0	1	0.1968072	-308.6905
254234	cg1374835	14625475	14625475	0.3334559	0	1	0.041722	-210.2102
254252	cg1374911	27651417	15717451	0.3757418	0	1	0.2345371	-37.90046
254265	cg1374954	70725351	59636392	0.2975473	0	1	0.7469212	374.3439
254326	cg1375272	14643338	14643338	0.7078744	0	1	0.4356256	-118.8686
254346	cg1375361	41669312	41669312	0.7122397	0	1	0.8177181	31.19681
254356	cg1375435	66771492	66771492	0.464051	0	1	0.5721828	20.73452
254382	cg1375551	13644358	50618434		0	1		-4.34E-09
254629	cg1376991	50803368	50803368	0.9405962	0	1		-4.34E-09
254666	cg1377179	67666468	63745415	0.0829029	0	1	0.1926989	42.21125
254673	cg1377221	48807457	25694326	0.255552	0	1	0.3693948	25.73024
254690	cg1377330	32769322	32769322	0.7446499	0	1	0.9284402	117.9938
254779	cg1377771	57708408	30628336	0.1977439	0	1	0.0961209	-35.2688
254799	cg1377856	69709303	69709303	0.7887281	0	1	0.6844592	-27.74966
254899	cg1378319	21682334	21682334	0.2519428	0	1	0.3855734	33.54962
255143	cg1379440	41639341	41639341	0.5105354	0	1	0.6444196	31.05069
255213	cg1379746	73751365	73751365	0.5331711	0	1	0.6660002	31.40625
255240	cg1379874	18768427	18768427	0.2027032	0	1	0.3236037	31.62445
255420	cg1380648	44707417	44707417	0.7964861	0	1	0.9436297	97.25165
255450	cg1380803	47649309	47649309	0.2798931	0	1	0.056	-135.0197
255625	cg1381703	67727454	67727454	0.5838038	0	1	0.6863701	21.31187
255699	cg1382105	40694387	40694387	0.6056516	0	1	0.3547856	-95.90824
255789	cg1382528	31738487	31738487	0.7945987	0	1	0.9024069	48.59037
255798	cg1382593	15798480	33600463	0.4250093	0	1	0.3113038	-23.72869
255874	cg1382910	60688420	60688420	0.5662876	0	1	0.7318295	50.37844
255880	cg1382962	59605469	59605469	0.7830966	0	1	0.5782682	-80.51099
255885	cg1382984	55793300	55793300	0.7555031	0	1	0.8736761	47.20859
255896	cg1383061	56803382	37715327	0.746716	0	1	0.9285896	116.4658
256092	cg1384263	66679384	27632433	0.8297709	0	1	0.7077386	-40.76558
256185	cg1384744	17788362	17788362	0.868524	0	1	0.7385201	-52.96652
256382	cg1385721	19772354	19772354	0.5923454	0	1	0.7396476	42.15827
256388	cg1385764	47803479	47803479	0.7780548	0	1	0.5640386	-85.74109
256437	cg1385977	57630509	57630509	0.6940321	0	1	0.8017692	30.44405
256447	cg1386028	15706467	15706467	0.1861625	0	1	0.2956474	28.27049
256562	cg1386626	69686405	30663316	0.5080699	0	1	0.6761523	47.42726
256635	cg1387053	25770477	25770477	0.3084787	0	1	0.4086667	19.44877
256704	cg1387442	52603344	64705354	0.3124393	0	1	0.4500385	32.47624
256712	cg1387475	73714364	10734352	0.9749987	0	1	0.6318512	-327.2581
256778	cg1387791	73751347	73751347	0.1486742	0	1	0.312943	59.85676
256794	cg1387864	29736459	29736459	0.4716889	0	1	0.3698715	-18.93187
256945	cg1388578	52680473	52680473	0.7845245	0	1	0.6159126	-58.53608
257098	cg1389485	52642474	62668499	0.4607156	0	1	0.5773474	23.53156

257112	cg1389565(	22740391	22740391	0.2388765	0	1	0.3491512	25.23613
257149	cg13897348	53632377	53632377	0.0729315	0	1	0.2874463	118.3547
257151	cg13897388	15654377	15654377	0.3531185	0	1	0.5479693	58.60798
257223	cg13900773	15714346	48699432	0.2925563	0	1	0.1611993	-40.42974
257257	cg13902645	65734370	23712404	0.8316638	0	1	0.6592568	-69.98885
257311	cg13905298	20689494	20689494	0.6475716	0	1	0.5369359	-22.78345
257437	cg13910785	34779364	34779364	0.0591716	0	1	0.1993037	68.12987
257531	cg1391399(	33706467	33706467	0.8667772	0	1	0.6385564	-122.5085
257565	cg1391572(	52735355	52735355	0.6344593	0	1	0.4832704	-37.8314
257593	cg13916633	18655427	18655427	0.9367281	0	1	0.8304715	-60.49958
257658	cg13918754	21719430	20681309	0.0304538	0	1	0.1599074	72.56838
257761	cg13923371	59739471	61650427	0.3326574	0	1	0.1701902	-55.26378
257774	cg13924287	58759478	58759478	0.2156843	0	1	0.0822089	-56.40917
257782	cg13924635	48740453	71705427	0.9580324	0	1	0.8197492	-100.1331
257783	cg13924715	44757399	34704415	0.6397325	0	1	0.5160538	-27.08743
257810	cg13925809	54709451	54709451	0.2214627	0	1	0.3351819	27.47685
257843	cg13927504	24795399	24795399	0.1914593	0	1	0.2931058	24.83939
257866	cg13928473	73643338	73643338	0.4266686	0	1	0.3234923	-20.04338
257930	cg1393125(	43640424	43640424	0.6613383	0	1	0.8122061	53.16344
258019	cg1393499(	53634405	53634405	0.7301214	0	1	0.9317006	135.672
258082	cg13937627	67746374	52673303	0.4360551	0	1	0.3177271	-25.14648
258169	cg13941978	27797357	68657390	0.098831	0	1	0.2237847	47.32819
258195	cg13943333	68725365	68725365	0.347643	0	1	0.2292329	-28.81076
258219	cg13944507	44749313	44749313	0.5585244	0	1	0.6913856	32.56396
258239	cg1394557(	18780428	18780428	0.2094133	0	1	0.321803	27.73194
258415	cg13954292	14722349	14722349	0.2860695	0	1	0.391879	21.82577
258610	cg13965612	73643485	73643485	0.3378117	0	1	0.4413115	19.87624
258672	cg13969327	70696502	70696502	0.4818303	0	1	0.5998353	24.26797
258677	cg13969662	59724471	67729467	0.7928274	0	1	0.6837459	-30.12912
258706	cg13971504	63642379	63642379	0.1975126	0	1	0.311347	29.14178
258724	cg13972423	15728324	15728324	0.4083484	0	1	0.5246246	23.23433
258728	cg13972661	29688442	29688442	0.5929356	0	1	0.470963	-25.53613
258774	cg13974562	71763399	56810505	0.4346081	0	1	0.2798745	-41.11744
258907	cg13979581	62695310	62695310	0.3189812	0	1	0.4606567	33.91163
258968	cg13982037	67788333	67788333	0.5042235	0	1	0.7285435	84.92231
259022	cg13984289	74759420	74759420	0.4263038	0	1	0.538715	21.9452
259137	cg13989295	58727359	58727359	0.4013708	0	1	0.2356953	-49.37134
259204	cg13993183	44692337	66773386	0.6638252	0	1	0.5230765	-34.50343
259237	cg1399473(	32778500	32778500	0.1224696	0	1	0.4207934	374.3439
259252	cg13995427	31735405	17760352	0.3522932	0	1	0.4786488	27.25398
259289	cg13996925	58622429	58622429	0.2738946	0	1	0.1522579	-36.90876
259368	cg14001023	47714395	23603428	0.2911385	0	1	0.140661	-53.45439
259568	cg1400990(	60778356	60778356	0.6359326	0	1	0.7395734	24.04751

259663	cg14013597	15685493	14745503	0.9839626	0	1	0.849817	-120.7767
259709	cg14015502	17614455	17614455	0.1220765	0	1	0.2368886	37.73879
259777	cg14018363	55785372	55785372	0.629591	0	1	0.7437955	28.26766
259845	cg14021170	63800497	63800497	0.106532	0	1	0.2110997	35.04827
259944	cg14024893	14639392	39722331	0.6093548	0	1	0.4829298	-27.38358
259968	cg14026106	56747341	56747341	0.2736718	0	1	0.4056893	31.7756
260111	cg14032261	16779407	16779407	0.3557106	0	1	0.5043125	35.89477
260165	cg14035247	61732499	61732499	0.9164067	0	1	0.8011369	-58.37681
260318	cg14041812	66763350	66763350	0.9510764	0	1	0.841617	-70.69642
260373	cg14044167	13767316	13767316	0.8155226	0	1	0.6688274	-51.43736
260387	cg14044669	49635493	49635493	0.9176259	0	1	0.3760553	-371.3336
260445	cg14047561	13705510	13705510	0.6630851	0	1	0.7931126	39.61281
260524	cg14052235	27659421	50627420	0.1521574	0	1	0.0276802	-70.14994
260558	cg14054279	48704449	48704449	0.6608447	0	1	0.7672736	26.88387
260574	cg14055004	23702406	23702406	0.5236322	0	1	0.6332124	22.09252
260600	cg14056849	70781354	70781354	0.6467785	0	1	0.8870775	142.5562
260670	cg14059835	34704426	34704426	0.280655	0	1	0.3874784	22.33198
260678	cg14060113	10718499	11806350	0.4312769	0	1	0.1768906	-116.1201
260779	cg14065526	69708390	69708390	0.3259925	0	1	0.2011556	-33.39984
260780	cg14065590	62675467	62675467	0.8109962	0	1	0.7104761	-28.29507
260813	cg14067524	50729438	50729438	0.3099527	0	1	0.4224599	23.36083
260826	cg14068184	49793437	49793437	0.6106209	0	1	0.2672452	-189.499
260834	cg14068788	35601496	35601496	0.1513634	0	1	0.2646067	33.15538
260991	cg14075454	74698433	10612313	0.1729188	0	1	0.0578438	-51.87217
261071	cg14079359	32652312	66804337	0.5306846	0	1	0.4244938	-19.96951
261073	cg14079463	47692328	47692328	0.810434	0	1	0.6040344	-87.45534
261121	cg14081667	28661448	28661448	0.1500588	0	1	0.266929	34.95728
261209	cg14085606	28693342	52792431	0.8027155	0	1	0.6699349	-42.19168
261249	cg14087413	64747435	64747435	0.3658754	0	1	0.4663019	18.56172
261281	cg14089045	16772482	16772482	0.7406406	0	1	0.8601296	44.93333
261283	cg14089103	36734471	36734471	0.355047	0	1	0.5326576	49.35351
261285	cg14089267	67756328	26777382	0.5688228	0	1	0.4470324	-25.22038
261368	cg14092997	42766372	42766372	0.5474284	0	1	0.4339606	-22.30572
261454	cg14096180	47710489	47710489	0.2614264	0	1	0.3913613	31.54252
261539	cg14100550	42714442	42714442	0.9048905	0	1	0.7214196	-104.0802
261621	cg14104700	24740407	24740407	0.8024318	0	1	0.4435572	-231.3076
261631	cg14105536	23766507	23766507	0.713701	0	1	0.8221337	33.07725
261636	cg14105781	54737451	54737451	0.2314495	0	1	0.0798923	-68.74492
261656	cg14106680	47642418	64766423	0.8558267	0	1	0.7541685	-34.6504
261688	cg14108567	29795429	13648308	0.5194591	0	1	0.3121332	-67.08037
261715	cg14110168	56687339	56687339	0.5612291	0	1	0.7936675	102.3236
261827	cg14116332	41652368	54719324	0.4961128	0	1	0.3688246	-27.35093
261840	cg14116764	30664401	39692389	0.1133416	0	1	0.0046312	-70.10843

261943	cg14121740	40715491	40715491	0.1777438	0	1	0.2796037	25.91095
261956	cg14122403	36711497	36711497	0.2251973	0	1	0.3701291	40.2873
261966	cg14123034	72770339	72770339	0.7866347	0	1	0.915963	68.21288
262147	cg14132620	19770390	19770390	0.5292098	0	1	0.6369699	21.58824
262159	cg14133106	27738492	27738492	0.4992857	0	1	0.612669	22.93008
262443	cg14148981	52708325	52708325	0.1435245	0	1	0.2579757	34.63021
262487	cg14151065	69684374	69684374	0.3345845	0	1	0.1006271	-122.4931
262515	cg14152465	26803312	26803312	0.568252	0	1	0.7019495	33.47089
262527	cg14152974	61620504	61620504	0.2806014	0	1	0.414321	32.14023
262631	cg14157527	32645447	32645447	0.543829	0	1	0.4120967	-28.72342
262647	cg14158558	49786440	49786440	0.588774	0	1	0.6966582	23.47967
262691	cg14160433	22798367	22798367	0.3733926	0	1	0.4927304	24.49743
262833	cg14166701	59676319	27655364	0.4337101	0	1	0.5640446	28.26036
262843	cg14167033	72735486	72735486	0.7820945	0	1	0.9346978	95.99923
262904	cg14170201	66762308	66762308	0.7185978	0	1	0.8804621	77.34793
262991	cg14174221	12618450	12618450	0.1636856	0	1	0.2663349	27.38311
263044	cg14177084	13644487	13644487		0	1		-4.34E-09
263104	cg14179288	57618362	57618362	0.3343967	0	1	0.1843696	-46.78915
263125	cg14180415	54715503	54715503	0.767092	0	1	0.8763618	42.68726
263233	cg14184954	18710350	18710350	0.6973331	0	1	0.8214221	40.52407
263298	cg14187813	38654363	38654363	0.5584601	0	1	0.4496821	-20.84642
263322	cg14188774	21618351	21618351	0.4084084	0	1	0.2995826	-22.41836
263330	cg14189116	57754348	57754348	0.5151849	0	1	0.6753632	43.59772
263341	cg14189534	16783506	16783506	0.6723546	0	1	0.7743348	25.67949
263353	cg14189782	48730327	48730327	0.3306698	0	1	0.4573836	27.8187
263416	cg14192299	66716342	54642479	0.8873494	0	1	0.7848228	-41.33937
263473	cg14195178	26730510	26730510	0.5760067	0	1	0.6912544	25.84501
263515	cg14197113	18656448	18656448	0.3245684	0	1	0.438543	23.49708
263529	cg14198221	55768438	55768438	0.3897829	0	1	0.5337019	33.56934
263594	cg14201424	33795429	24648384	0.5306402	0	1	0.6984327	48.72123
263660	cg14204081	63639347	63639347	0.2712959	0	1	0.145723	-39.63856
263786	cg14209518	40741482	40741482	0.3863198	0	1	0.533374	34.88596
263865	cg14212038	33638311	33638311	0.3616368	0	1	0.4999461	31.61075
263902	cg14213394	67685329	33758460	0.3490418	0	1	0.2373627	-25.82347
263937	cg14214739	54746417	31784456	0.1514847	0	1	0.0471719	-48.24405
263976	cg14216068	38746430	28745335	0.285497	0	1	0.4133002	29.63058
264165	cg14223671	38743305	38743305	0.3131503	0	1	0.2096144	-24.38812
264229	cg14227325	42669375	42669375	0.4631503	0	1	0.6032661	32.47551
264243	cg14228103	31747469	31747469	0.9070572	0	1	0.5302983	-310.7961
264381	cg14234912	14768483	14768483	0.3825736	0	1	0.4891114	20.24695
264446	cg14238120	69799509	69799509	0.2390862	0	1	0.5567071	374.3439
264480	cg14239655	15745447	15745447	0.3776328	0	1	0.2671524	-24.05542
264504	cg14240790	66778462	66778462	0.1694831	0	1	0.2829944	31.38856

264524	cg1424183	55738497	55738497	0.3668687	0	1	0.4951278	27.72904
264602	cg1424589	56606470	56606470	0.4976738	0	1	0.6039938	20.52704
264731	cg1425223	20612393	20612393	0.7968827	0	1	0.9191704	64.48829
264754	cg1425359	36807492	36807492	0.743337	0	1	0.5565782	-63.18744
264922	cg1426077	44645465	44645465	0.609821	0	1	0.5049901	-20.15322
265033	cg1426623	69689428	36775365	0.6926097	0	1	0.5075383	-57.30016
265105	cg1427000	69655308	36605363	0.8881696	0	1	0.7550253	-60.52542
265130	cg1427102	64732448	64732448	0.5776953	0	1	0.7489324	55.1841
265221	cg1427608	12607466	35645443	0.8452189	0	1	0.6983074	-57.48339
265248	cg1427729	28755433	28755433	0.5799425	0	1	0.7112358	33.02574
265319	cg1428103	65637437	65637437	0.3487586	0	1	0.4591554	21.8626
265384	cg1428364	50728485	50728485	0.3559449	0	1	0.4655093	21.48919
265387	cg1428375	10749446	42607466	0.238556	0	1	0.1326528	-32.03485
265493	cg1428804	27600478	27600478	0.6730114	0	1	0.7806712	28.43786
265544	cg1429007	13604340	13604340	0.4363575	0	1	0.5865431	36.36507
265609	cg1429367	29710310	29710310	0.4512209	0	1	0.8301659	374.3439
265634	cg1429495	42717353	42717353	0.4702838	0	1	0.6706018	64.36662
265635	cg14294971	27708424	27708424	0.7490953	0	1	0.5351524	-80.8025
265768	cg1430213	11677360	11677360	0.2539387	0	1	0.7158458	374.3439
265871	cg1430747	14724497	57715430	0.8528931	0	1	0.7217177	-50.00092
265887	cg1430831	58623424	58623424	0.2662575	0	1	0.3982834	32.13377
265899	cg1430864	38611396	38611396	0.6971207	0	1	0.8769147	88.86023
265938	cg1431087	55658463	55658463	0.4013088	0	1	0.2561323	-38.20879
266063	cg1431662	66809447	66809447	0.2612819	0	1	0.4285937	48.20387
266064	cg1431666	45791404	71766344	0.2851007	0	1	0.4453334	43.37456
266079	cg1431738	74681441	19676415	0.5954162	0	1	0.4903379	-20.02731
266194	cg1432320	16761452	16761452	0.5659432	0	1	0.4502995	-23.11004
266205	cg1432391	22757305	22757305	0.1948483	0	1	0.4408661	105.772
266224	cg1432481	42657509	42657509	0.4435144	0	1	0.3131405	-29.64815
266225	cg1432483	24619461	24619461	0.8175089	0	1	0.9388477	73.27152
266232	cg1432515	62672390	63721444	0.6780618	0	1	0.5421557	-33.14874
266235	cg1432518	27635449	25748318	0.7929899	0	1	0.6823524	-30.80896
266242	cg1432577	50772323	50772323	0.2481602	0	1	0.3512368	22.29229
266324	cg1432978	52733448	52733448	0.7419873	0	1	0.5385836	-73.02491
266327	cg1432988	38785507	32800433	0.6982418	0	1	0.8277644	44.31025
266351	cg1433086	63688403	63688403	0.4330111	0	1	0.5406998	20.44346
266359	cg1433120	35601349	35601349	0.8465849	0	1	0.6915289	-62.7523
266360	cg1433122	36734408	36734408	0.4483927	0	1	0.3032037	-35.90339
266526	cg1433946	37733330	37733330	0.4733411	0	1	0.6213549	36.10343
266549	cg1434046	66713386	66713386	0.3289846	0	1	0.4747267	35.29547
266559	cg1434092	43655507	43655507	0.3803439	0	1	0.2319891	-41.3177
266767	cg1435250	70794396	70794396	0.7082798	0	1	0.5889124	-28.16082
266854	cg1435691	39662411	39662411	0.2757204	0	1	0.3824396	22.46318

266921	cg14361252	20779300	43644329	0.6061346	0	1	0.4458355	-41.13301
267111	cg14373189	54713385	54713385	0.0449579	0	1		-4.34E-09
267246	cg14378564	49632458	49632458	0.3830594	0	1	0.2507172	-33.09151
267367	cg14384416	36707502	36707502	0.7392151	0	1	0.8418161	32.972
267438	cg14388237	21625490	21625490	0.2064538	0	1	0.0946117	-40.93117
267563	cg14395298	46769505	22755310	0.5467375	0	1	0.4197417	-26.97203
267708	cg14402224	60720388	60720388	0.2909254	0	1	0.3958434	21.39062
267822	cg14408831	71685333	71685333	0.693909	0	1	0.9464952	374.3439
267955	cg14414911	28722498	28722498	0.6358953	0	1	0.7518438	29.5163
268024	cg14417784	18609379	17793438	0.6379536	0	1	0.4905938	-36.30626
268096	cg14420715	40655403	40655403	0.8449991	0	1	0.9557542	74.71031
268125	cg14422240	28641377	28641377	0.3304976	0	1	0.4880102	40.33964
268136	cg14422500	64736341	64736341	0.473855	0	1	0.7031047	85.37443
268194	cg14425564	65709411	11727418	0.1914921	0	1	0.3200137	35.84457
268286	cg14429748	38615468	38615468	0.667946	0	1	0.9009311	143.7481
268337	cg14431577	17720381	17720381	0.4349861	0	1	0.5353531	18.21614
268349	cg14432251	73751357	73751357	0.3761774	0	1	0.4962313	24.71392
268389	cg14434755	67710342	67710342	0.6864648	0	1	0.794689	29.945
268402	cg14435550	10673464	18684454	0.6269901	0	1	0.5261468	-19.22838
268436	cg14437446	27759462	27759462	0.2546418	0	1	0.3608009	23.07051
268479	cg14440272	14740455	14740455	0.5337917	0	1	0.6751411	35.25216
268731	cg14454094	25606396	25606396	0.473458	0	1	0.6033274	28.5383
268787	cg14457452	28653324	28653324	0.8591209	0	1	0.9617472	71.71175
268891	cg14462402	22679490	22679490	0.1647421	0	1	0.280854	32.98922
268928	cg14463736	31733355	31733355	0.2282683	0	1	0.0705467	-76.17141
268991	cg14467015	53763328	53763328	0.118191	0	1	0.2240766	33.94419
268998	cg14467415	17628308	17628308	0.4789638	0	1	0.5915698	22.35926
269207	cg14476852	36706322	36706322	0.1048666	0	1	0.3274563	111.6404
269383	cg14485643	74770465	74770465	0.1790819	0	1	0.2809207	25.80062
269523	cg14493094	40603491	40603491	0.8639356	0	1	0.6869424	-82.22099
269603	cg14497649	38770382	38770382	0.6205413	0	1	0.8143981	79.84689
269656	cg14500486	70643436	26786310	0.6805371	0	1	0.5422706	-34.22951
269770	cg14506194	53678387	53678387	0.9054555	0	1	0.7396255	-90.59117
269976	cg14515364	57740498	21692339	0.8579797	0	1	0.7275575	-50.69371
270076	cg14519356	71655353	71655353	0.7077175	0	1	0.8078294	27.8039
270144	cg14521995	11608485	70606468		0	1		-4.34E-09
270156	cg14522441	38809464	64726319	0.6866211	0	1	0.583764	-21.41557
270232	cg14525390	54717343	54717343	0.1845204	0	1	0.3346226	46.80887
270387	cg14531093	48658334	23714502	0.912662	0	1	0.7650406	-80.21509
270462	cg14534987	67687408	15645431	0.2866144	0	1	0.1428361	-49.41331
270473	cg14535332	26651379	56756319	0.2783394	0	1	0.1723521	-27.98993
270735	cg14548901	45722301	45722301	0.3716836	0	1	0.5099393	31.45653
270764	cg14550066	67643448	67643448	0.3907734	0	1	0.2798792	-23.72873

270836	cg14553444	68711481	68711481	0.4240926	0	1	0.5387981	22.69755
270974	cg14559139	59614346	59614346	0.5723448	0	1	0.6943988	28.49276
271065	cg14562786	49608347	49608347	0.8109947	0	1	0.7083575	-29.20248
271201	cg14569423	25733453	25733453	0.4774488	0	1	0.6331053	39.74677
271284	cg14573817	56676465	56676465	0.6541107	0	1	0.5468007	-21.8656
271371	cg14577706	65723423	65723423	0.6727042	0	1	0.7794287	27.95433
271523	cg14583999	21607472	21607472	0.394039	0	1	0.649137	100.2271
271686	cg14592638	33702337	33702337	0.3333814	0	1	0.5600187	78.63268
271789	cg14597862	40691394	40691394	0.4094799	0	1	0.6022761	57.4521
271802	cg14598338	31791482	59746308	0.1382622	0	1	0.2845853	51.57705
271807	cg14598478	54608430	54608430	0.1217972	0	1	0.2846262	64.53538
271933	cg14604474	29608370	29608370	0.3689715	0	1	0.2577577	-24.69578
271936	cg14604522	48802381	62611436	0.6696108	0	1	0.8583446	88.68325
271952	cg14605357	29713486	29713486	0.2104319	0	1	0.3221306	27.39811
272022	cg14608770	49753305	39714411	0.7647989	0	1	0.6523743	-29.07649
272025	cg14609104	52683440	52683440	0.7111526	0	1	0.4837367	-84.96564
272069	cg14611455	52757336	52757336	0.5493169	0	1	0.6822329	32.13997
272155	cg14615266	69601336	40624490	0.3180754	0	1	0.1333303	-75.72861
272275	cg14621900	48627421	48627421	0.7078931	0	1	0.5555239	-41.99105
272339	cg14625636	10802457	10802457	0.7132637	0	1	0.5965301	-27.42793
272392	cg14629010	15711375	15711375	0.6539914	0	1	0.8442137	85.26231
272415	cg14630630	21656316	21656316	0.5739272	0	1	0.6745954	20.32117
272536	cg14636261	25713488	25713488	0.3710389	0	1	0.4955398	26.32363
272655	cg14642338	21795454	21795454	0.345681	0	1	0.4472888	19.17533
272675	cg14643264	24747360	24747360	0.3857371	0	1	0.487446	18.75965
272717	cg14645244	64690436	36600482	0.4349092	0	1	0.2802211	-41.07868
272748	cg14646641	67606429	67606429		0	1		-4.34E-09
272836	cg14651435	32620478	32620478	0.5473084	0	1	0.6630592	24.97389
272887	cg14653593	60626498	60626498	0.3450853	0	1	0.4674776	25.96679
272897	cg14654171	51679367	51679367	0.4736582	0	1	0.6003775	27.33481
273008	cg14659129	74709337	74709337	0.1474755	0	1	0.2540554	30.62715
273011	cg14659346	38621481	24713432	0.2848175	0	1	0.1799087	-26.94019
273017	cg14659662	52679362	47783399	0.3444168	0	1	0.4604881	23.80226
273019	cg14659698	13644387	66685405		0	1		-4.34E-09
273054	cg14661431	51625352	51625352	0.406243	0	1	0.2404309	-49.03829
273155	cg14666908	61604365	61604365	0.3933466	0	1	0.5480171	38.12434
273204	cg14669842	55635406	55635406	0.7717183	0	1	0.6134934	-51.05244
273206	cg14669863	52722450	17619465	0.3018773	0	1	0.4962214	60.00425
273211	cg14670092	72621416	72621416	0.2662963	0	1	0.420764	41.75839
273343	cg14679255	28650444	28650444	0.8410414	0	1	0.6094815	-115.2996
273439	cg14685946	17609450	17609450	0.4925787	0	1	0.6023154	21.57994
273444	cg14686042	23646341	23646341	0.2633644	0	1	0.7407238	374.3439
273463	cg14687298	15679356	67606421	0.3146757	0	1	0.1068815	-99.49695

273487	cg14688662	27700493	27700493	0.1564962	0	1	0.2760212	35.42366
273536	cg14691596	70786483	70786483	0.51829	0	1	0.6278555	21.97361
273625	cg14696601	62721321	62721321	0.5621378	0	1	0.6711928	22.94829
273648	cg14698238	33789329	33789329	0.7754866	0	1	0.8756339	37.44656
273669	cg14699257	45651331	28619402	0.2698505	0	1	0.1675121	-26.92634
273720	cg14701867	39801313	39801313	0.1941851	0	1	0.3395154	43.3592
273761	cg14704124	11634302	11634302	0.5863835	0	1	0.7198893	34.49076
273774	cg14705010	51719320	39673433	0.337536	0	1	0.4919545	38.77923
273832	cg14708411	48720320	40616345	0.4584895	0	1	0.3376932	-25.54982
273874	cg14710850	27638448	27638448	0.8266957	0	1	0.6847706	-50.80645
273883	cg14711243	18781419	18781419	0.231952	0	1	0.0730167	-76.12132
273954	cg14715697	46756442	66644400	0.6013563	0	1	0.8710731	374.3439
273980	cg14717122	16806408	16806408	0.3786618	0	1	0.7879277	374.3439
274187	cg14731462	20687329	20687329	0.6883	0	1	0.847442	65.51565
274249	cg14736087	48721339	48721339	0.8311306	0	1	0.7231991	-34.02399
274293	cg14738806	29643446	29643446	0.7083349	0	1	0.5605668	-39.92296
274317	cg14740976	28727498	28727498	0.5035692	0	1	0.6384467	31.25425
274351	cg14742445	39780473	39780473	0.5183096	0	1	0.6662767	37.65529
274353	cg14742615	14627388	14627388	0.3246969	0	1	0.2131141	-27.18065
274514	cg14753094	44624466	44624466	0.3751697	0	1	0.6023035	78.62325
274602	cg14758525	73712349	73712349	0.4744564	0	1	0.6419047	45.49614
274618	cg14759366	41780443	41780443	0.408395	0	1	0.2866919	-27.30709
274621	cg14759494	14686387	14686387	0.3962163	0	1	0.5225854	26.78535
274971	cg14780132	15659311	15659311	0.4517468	0	1	0.3509602	-18.85319
274979	cg14780466	61629303	19625435	0.2315567	0	1	0.3462225	27.24502
275030	cg14782559	50621445	50621445	0.3441009	0	1	0.1993556	-42.54236
275242	cg14793137	46638458	74760501	0.7636892	0	1	0.6204308	-42.58686
275292	cg14795253	25701421	58790505	0.1527366	0	1	0.0490328	-47.27384
275297	cg14795409	13644342	13644342		0	1	0.9281332	4.34E-09
275332	cg14797147	24788474	24788474	0.5231422	0	1	0.8227758	374.3439
275368	cg14799277	74607488	74607488	0.2125143	0	1	0.4892837	127.9083
275376	cg14799809	62630444	62630444	0.4690977	0	1	0.7479784	130.625
275438	cg14804903	47779475	47779475	0.4515153	0	1	0.6161584	43.36322
275508	cg14811011	62676339	62676339	0.2159809	0	1	0.3240038	25.66261
275509	cg14811014	69627345	69627345	0.2210358	0	1	0.3287652	25.26088
275567	cg14815891	59655461	24684340	0.4217027	0	1	0.5579849	30.48927
275599	cg14818277	46681424	46681424	0.6541591	0	1	0.7761948	34.11446
275602	cg14818413	70770367	70770367	0.5897468	0	1	0.4749664	-23.04998
275604	cg14818621	49708459	49708459	0.2815626	0	1	0.3917637	23.43485
275615	cg14819368	13644372	13644372		0	1		-4.34E-09
275727	cg14825858	17683414	17683414	0.7611327	0	1	0.6251812	-38.8975
275788	cg14829303	40753304	40753304	0.6010907	0	1	0.9093254	374.3439
275831	cg14831390	28714477	25660312	0.3929458	0	1	0.2554406	-34.95807

275847	cg14832352	73681385	73681385	0.1950233	0	1	0.3441515	45.12037
275855	cg14832904	22792504	22792504	0.2442318	0	1	0.3498992	23.35071
275886	cg14834300	36615392	36615392	0.8408129	0	1	0.7068557	-49.07571
275998	cg14839919	46686446	46686446	0.6294982	0	1	0.5291092	-19.13808
276017	cg14840848	42796501	17759429	0.5728108	0	1	0.6751133	20.85779
276233	cg14851460	45778357	45778357	0.4397212	0	1	0.5518877	21.89718
276239	cg14851544	53669497	53669497	0.3103178	0	1	0.4176516	21.65028
276315	cg14855378	58649403	58649403	0.579708	0	1	0.6933854	25.37308
276335	cg14856563	65740428	65740428	0.5975759	0	1	0.7278944	33.69515
276369	cg14858786	33709449	35701323	0.5214626	0	1	0.6234248	19.50259
276388	cg14859874	14694304	14694304	0.502335	0	1	0.3940264	-20.71729
276612	cg14873022	39715368	39715368	0.6401664	0	1	0.7960715	53.2735
276672	cg14877637	70646322	70646322	0.6412477	0	1	0.5384416	-20.11934
276678	cg14877834	28804443	28804443	0.2527637	0	1	0.4534916	67.08424
276757	cg14882966	42626317	31600403	0.1701885	0	1	0.0545772	-53.24293
276767	cg14883605	57691462	57691462	0.2292332	0	1	0.3474859	28.74979
276871	cg14891195	74756405	18734405	0.6263829	0	1	0.5187993	-21.30784
276900	cg14893857	25788482	25788482	0.3533182	0	1	0.2529225	-21.20502
276906	cg14894216	47779456	47779456	0.5179728	0	1	0.6387309	26.01656
276919	cg14895374	64717338	64717338	0.5532839	0	1	0.6617917	22.47474
277013	cg14900909	47622405	73707479	0.7180169	0	1	0.8932858	92.20353
277026	cg14902204	74772470	74772470	0.8792735	0	1	0.7780471	-38.79079
277154	cg14909614	36667507	36667507	0.2387392	0	1	0.3557332	27.72619
277267	cg14915878	47698493	71778502	0.3575494	0	1	0.2241495	-35.26356
277430	cg14926196	35619359	74680402	0.5695794	0	1	0.468958	-18.43505
277438	cg14926485	28731492	28731492	0.8547553	0	1	0.5259745	-215.9837
277455	cg14927745	56710417	64784332	0.7465494	0	1	0.6049225	-40.13873
277538	cg14931776	40692459	40692459	0.4795575	0	1	0.6357683	40.07955
277541	cg14931884	57642487	63670403	0.1957441	0	1	0.2994155	25.31319
277603	cg14936938	67655453	67655453	0.5120274	0	1	0.618138	20.68859
277625	cg14938419	51773359	40665371	0.8585226	0	1	0.7269951	-51.46165
277648	cg14939821	36751420	36751420	0.5560834	0	1	0.7076939	41.62743
277695	cg14943002	49717489	49717489	0.4253058	0	1	0.5307325	19.73394
277717	cg14944696	34806455	34806455	0.6361952	0	1	0.7440438	25.85574
277786	cg14949065	38663360	11652426	0.75594	0	1	0.6168943	-39.82117
277823	cg14951292	68654498	68654498	0.7713868	0	1	0.6702578	-25.13737
277850	cg14953379	73726329	73726329	0.730256	0	1	0.8523908	44.82435
277965	cg14960373	62676503	62676503	0.417033	0	1	0.5222617	19.67949
278121	cg14970273	18770301	18770301	0.636618	0	1	0.5359661	-19.35833
278221	cg14975347	11777315	11777315	0.746446	0	1	0.8485084	33.69691
278251	cg14977018	62660356	62660356	0.5863732	0	1	0.720856	34.9679
278315	cg14981468	30768379	30768379		0	1	0.8627156	4.34E-09
278340	cg14983135	52602503	12660507	0.8425899	0	1	0.7307357	-37.64853

278359	cg14983838	60699371	60699371	0.569271	0	1	0.451207	-23.94499
278371	cg14984341	43800433	55752431	0.5438069	0	1	0.4289097	-22.76761
278416	cg14986890	48640421	48640421	0.5473917	0	1	0.7398703	66.10843
278434	cg14987787	16646398	66768392	0.7820057	0	1	0.6793602	-26.53351
278474	cg14989977	17783337	17783337	0.2168143	0	1	0.3173974	22.92317
278535	cg14992972	21762414	21762414	0.6598659	0	1	0.7725611	29.81541
278624	cg14997132	63806388	63806388	0.4295923	0	1	0.5384129	20.79537
278633	cg14997592	25771491	25771491	0.5796251	0	1	0.4749421	-19.73411
278659	cg14999036	12701332	12701332	0.1852933	0	1	0.2936267	27.87835
278680	cg15000813	67606447	67606447	0.18625	0	1	0.2915319	26.60207
278752	cg15004787	28786454	28786454	0.8119246	0	1	0.925899	61.34002
278806	cg15007959	49757401	55726326	0.3008598	0	1	0.130415	-67.28383
278821	cg15009232	64739341	64739341	0.5148922	0	1	0.6192147	20.15145
278912	cg15014361	14649308	14649308	0.6041504	0	1	0.7647503	51.42089
279008	cg15020568	70810338	64604420	0.3101277	0	1	0.1813745	-36.93263
279047	cg15022609	55620414	19793382	0.5400599	0	1	0.3240196	-71.99827
279085	cg15025200	48763405	22691478	0.2923617	0	1	0.1473418	-49.34667
279140	cg15027721	22689478	22689478	0.314063	0	1	0.4238452	22.35445
279237	cg15033013	39655305	39655305	0.7940681	0	1	0.6172248	-64.91439
279251	cg15033552	74678456	74678456	0.4320931	0	1	0.5686673	30.65847
279423	cg15044146	38798388	38798388	0.9475304	0	1	0.8376011	-69.00249
279432	cg15044573	72727466	72727466	0.3499393	0	1	0.4583301	21.20418
279436	cg15044767	64648420	64648420	0.432609	0	1	0.3195578	-23.30379
279437	cg15044932	49637505	49637505	0.867865	0	1	0.7658162	-36.96303
279568	cg15054873	33754404	33754404	0.261564	0	1	0.3740487	24.98346
279586	cg15055782	61647408	61647408	0.4929564	0	1	0.61119	24.53286
279672	cg15059553	12650335	12650335	0.5477436	0	1	0.4420253	-19.85092
279682	cg15060081	66673325	30638301	0.3174776	0	1	0.4793259	42.7269
279690	cg15060599	73696342	72780487	0.1987519	0	1	0.0955848	-36.15641
279836	cg15068552	56712415	46733418	0.8065151	0	1	0.7054895	-28.05867
279932	cg15074709	39607394	23616423	0.3227798	0	1	0.0530335	-179.6317
280024	cg15079934	32752434	14738451	0.4082618	0	1	0.5240945	23.0865
280034	cg15080433	40634450	40634450	0.7131956	0	1	0.8885154	90.19691
280115	cg15084803	42709321	42709321	0.5044903	0	1	0.7387561	93.30235
280177	cg15087907	41711429	19799359	0.7181204	0	1	0.6129323	-23.48877
280257	cg15092219	41636348	41636348	0.2986402	0	1	0.1969954	-24.4727
280260	cg15092343	24699391	17737346	0.2370926	0	1	0.1219235	-37.93986
280352	cg15098643	37710373	37710373	0.5762125	0	1	0.6775874	20.638
280445	cg15104622	12797491	31705328	0.6809847	0	1	0.7948654	32.40652
280452	cg15105011	18792450	18792450	0.5597843	0	1	0.3881671	-46.04846
280654	cg15116095	69657441	69657441	0.7148057	0	1	0.5998309	-26.85208
280660	cg15116481	63633329	19609360	0.8195281	0	1	0.7096616	-33.41318
280671	cg15117754	27693398	27693398	0.6553584	0	1	0.4872814	-46.35828

280754	cg1512299:	56723420	53652348	0.6586772	0	1	0.497493	-43.23799
280946	cg1513229:	40628329	40628329	0.743957	0	1	0.8739604	54.37629
281002	cg1513516(	56688395	56688395	0.6907663	0	1	0.8131198	38.49052
281007	cg1513528(	26780484	26780484	0.1816819	0	1	0.2843361	25.92117
281014	cg1513576:	14697311	44624470	0.3313319	0	1	0.4585824	27.99985
281050	cg1513828(	48800482	48800482	0.2546221	0	1	0.5066262	101.7301
281129	cg1514269⁄	16736447	23617466	0.8228424	0	1	0.6117935	-93.95747
281165	cg1514517⁄	30645385	73619499	0.1179614	0	1	0.2238124	33.9603
281233	cg1514964:	68604460	68604460	0.1448447	0	1	0.2789127	44.00752
281489	cg1516603(	14662427	18800399	0.7012739	0	1	0.501335	-66.46135
281583	cg1517179]	54705472	54705472	0.6390637	0	1	0.7746951	40.14685
281622	cg1517429⁄	65800308	65800308	0.5614785	0	1	0.4369834	-26.12438
281685	cg1517761:	37632356	48713472	0.3964663	0	1	0.2391833	-44.95192
281797	cg1518766:	36727374	25731376	0.5057255	0	1	0.6403576	31.21594
281835	cg1519038:	54644436	54644436	0.7044578	0	1	0.5778816	-30.74373
281871	cg1519313:	25755329	31659427	0.6545675	0	1	0.5307515	-27.57944
281875	cg1519321:	24717412	24717412	0.486775	0	1	0.590556	19.57318
281947	cg1519814(	12726408	12726408	0.8095459	0	1	0.5737763	-108.9121
281973	cg1519998'	18726423	18726423	0.4066938	0	1	0.5188763	21.89257
281974	cg1520000(	30605453	30605453	0.1513813	0	1	0.2778662	39.32922
282058	cg1520542(	67685347	41726414	0.7156742	0	1	0.6006703	-26.90821
282080	cg1520705:	73778471	73778471	0.41982	0	1	0.5342778	22.61425
282225	cg1521534(	72600300	65779478	0.2718959	0	1	0.1437625	-41.17289
282261	cg1521922(	11753320	16794305		0	1		-4.34E-09
282359	cg1522634(	58751381	58751381	0.8864602	0	1	0.6047232	-181.7855
282528	cg1523654]	56680387	69765318	0.4647488	0	1	0.3127482	-38.46948
282642	cg1524385(	56716505	30775414	0.2118992	0	1	0.099969	-39.96179
282675	cg1524613]	37628397	37628397	0.8916965	0	1	0.7859694	-44.30798
282706	cg1524748:	68781455	68781455	0.6622935	0	1	0.1068407	-371.3336
282740	cg1524934]	35748431	35748431	0.5059884	0	1	0.6184062	22.71676
282756	cg1525063:	26733393	16683386	0.1373376	0	1	0.274254	46.65878
282760	cg1525114(	39710464	50745432	0.3412436	0	1	0.1582214	-69.31096
282767	cg1525138:	43642427	65714457	0.2371556	0	1	0.1077025	-48.01125
282802	cg1525464(	56711445	70679503	0.0405843	0	1	0.1596565	60.56231
282828	cg1525638'	65765430	65765430	0.3996904	0	1	0.5351008	30.14538
283130	cg1527810(	50758462	48714335	0.7365966	0	1	0.6194949	-28.928
283151	cg1527954]	46670416	46670416	0.4312818	0	1	0.2621572	-49.04557
283193	cg1528297:	10728416	10728416	0.1563554	0	1	0.328844	63.28057
283235	cg1528603:	48760415	48760415	0.6565873	0	1	0.7849373	37.87111
283257	cg1528780(	47793319	47793319	0.4537552	0	1	0.5703053	23.44373
283289	cg1529031:	34647475	24790449	0.9021891	0	1	0.6305247	-181.2075
283310	cg1529209'	57607317	57607317	0.4953502	0	1	0.6074794	22.4313
283314	cg1529235(	21607304	21607304	0.8493967	0	1	0.6681601	-80.5832

283323	cg15293181	46700503	46700503	0.795612	0	1	0.6955823	-26.63084
283348	cg15295441	37744473	37744473	0.5072793	0	1	0.7067806	66.7029
283381	cg1529832[illegible]	27651418	27651418	0.333419	0	1	0.2056415	-34.28796
283498	cg1530900[illegible]	44725368	44725368	0.7400873	0	1	0.8909221	73.46546
283670	cg1532090[illegible]	38807401	45758409	0.6096193	0	1	0.7548363	42.60964
283770	cg1532653[illegible]	39665482	39665482	0.8883465	0	1	0.7881083	-40.24679
283788	cg15328131	12768428	12768428		0	1	0.385128	4.34E-09
283810	cg1532955[illegible]	20721361	20721361	0.6546869	0	1	0.8613686	103.1033
283876	cg1533409[illegible]	60662308	60662308	0.881725	0	1	0.7678078	-46.66662
283970	cg1534052[illegible]	13611435	13611435	0.2531501	0	1	0.3584446	22.83698
284028	cg1534507[illegible]	48773449	29760424	0.1909337	0	1	0.3635179	57.91338
284063	cg1534715[illegible]	27772368	48705311	0.7841962	0	1	0.6716931	-30.76301
284224	cg15359321	23642399	23642399	0.4177842	0	1	0.5515418	29.49948
284232	cg15360181	48806320	48806320	0.0796393	0	1	0.1840415	39.89093
284234	cg15360451	33713458	69692479	0.6627392	0	1	0.7811483	33.00874
284253	cg1536169[illegible]	68641388	54794394	0.7182375	0	1	0.5518184	-49.58957
284297	cg1536461[illegible]	65807359	65807359	0.3776088	0	1	0.21049	-52.57658
284311	cg1536550[illegible]	35642301	35642301	0.6788164	0	1	0.9350349	374.3439
284543	cg1538132[illegible]	44630311	44630311	0.3359811	0	1	0.4414815	20.527
284584	cg1538363[illegible]	21613480	21613480	0.6363997	0	1	0.5356039	-19.39762
284670	cg1538906[illegible]	30747383	74746378	0.7770789	0	1	0.6680869	-28.67794
284673	cg15389191	17749315	17749315	0.6319572	0	1	0.7494153	29.94621
284731	cg1539214[illegible]	28645389	28645389	0.3455479	0	1	0.540241	58.66245
284769	cg1539349[illegible]	20622482	20622482	0.4377989	0	1	0.5414894	19.21683
284786	cg1539425[illegible]	58728307	58728307	0.2606134	0	1	0.363442	21.71413
284814	cg15395971	51694313	51694313	0.4470171	0	1	0.5936782	34.9796
284901	cg1540147[illegible]	10713410	10713410	0.2118614	0	1	0.3160815	24.5055
284932	cg1540351[illegible]	31703427	31703427	0.30291	0	1	0.4612995	41.6974
284948	cg1540466[illegible]	41703431	29770308	0.2440423	0	1	0.0561099	-104.9913
284960	cg1540543[illegible]	18616329	18616329	0.4904562	0	1	0.3254337	-43.94165
285011	cg1540851[illegible]	34757439	54742333	0.8930662	0	1	0.5538918	-250.5538
285038	cg15410411	39631417	32801472	0.5082831	0	1	0.3035472	-65.93059
285098	cg1541474[illegible]	72610507	14768412	0.6402913	0	1	0.4866215	-39.10587
285162	cg1541849[illegible]	31742425	31742425	0.4873055	0	1	0.6643266	51.25002
285261	cg1542527[illegible]	35773437	35773437	0.4247049	0	1	0.6022211	49.33233
285297	cg1542779[illegible]	60680371	60680371	0.7700835	0	1	0.9058024	68.58278
285341	cg1543165[illegible]	48761366	24686486	0.8373002	0	1	0.7351916	-32.10971
285394	cg1543576[illegible]	20696420	31691373	0.3983824	0	1	0.518989	24.74136
285413	cg1543695[illegible]	56792351	56792351	0.4937544	0	1	0.0331296	-371.3336
285528	cg1544316[illegible]	31604507	31604507	0.4733497	0	1	0.5874863	22.81069
285547	cg1544414[illegible]	10800311	46618328	0.5561246	0	1	0.7282121	53.42755
285666	cg1545009[illegible]	23785345	23785345	0.3784948	0	1	0.4905687	22.03179
285953	cg1546564[illegible]	26715498	51783462	0.3997017	0	1	0.2939995	-21.55578

285980	cg1546695	21606362	21606362	0.5003697	0	1	0.6512119	38.26929
285992	cg1546783	24807351	55780486	0.896091	0	1	0.7915183	-44.76789
286163	cg1547704	44725479	44725479	0.2047078	0	1	0.3607564	47.51559
286390	cg1549143	57685373	57685373	0.2058202	0	1	0.3086824	24.36924
286478	cg1549840	15694411	15694411	0.3810802	0	1	0.5207084	31.90269
286511	cg1550152	58784502	16797443	0.5185298	0	1	0.3059831	-70.558
286566	cg1550561	35654344	42775501	0.7123709	0	1	0.6095983	-22.3938
286622	cg1550917	29713300	29713300	0.6672997	0	1	0.5484667	-26.20562
286662	cg1551271	67790443	67790443	0.635158	0	1	0.7552092	31.44119
286725	cg1551826	73628441	73628441	0.8949994	0	1	0.384464	-371.3336
286894	cg1553151	15600489	15600489	0.5152028	0	1	0.625874	22.29186
286906	cg1553264	57808427	30685341	0.5128649	0	1	0.6261412	23.15435
286972	cg1553785	38629356	38629356	0.5352216	0	1	0.6421579	21.4434
287031	cg1554117	26800467	26800467	0.1729881	0	1	0.283622	29.84712
287065	cg1554302	33733322	33733322	0.5389736	0	1	0.6392699	19.30552
287093	cg1554463	21638446	11751488	0.0162287	0	1	0.1612749	92.82227
287149	cg1554819	68666500	68666500	0.6070249	0	1	0.4914535	-23.56079
287285	cg1555496	12639475	12639475	0.7744004	0	1	0.8962258	55.7078
287402	cg1556149	45804321	45804321	0.7075087	0	1	0.8345642	44.16853
287495	cg1556736	45616309	45616309	0.3203527	0	1	0.6038721	123.7664
287504	cg1556807	21673461	21673461	0.6843171	0	1	0.4413901	-93.33034
287512	cg1556861	50717475	50717475	0.2661532	0	1	0.3707948	22.1106
287529	cg1557014	51775306	51775306	0.7407752	0	1	0.6232798	-29.35808
287602	cg1557443	46744474	42759335	0.4574795	0	1	0.3513491	-20.47412
287633	cg1557588	53732368	52664438	0.6845766	0	1	0.5743033	-23.82996
287741	cg1558194	56612410	56612410	0.5193249	0	1	0.7109004	62.46905
287799	cg1558479	15687443	40654329	0.7679191	0	1	0.6124419	-49.14602
287906	cg1559180	14631428	14631428	0.5576638	0	1	0.7351441	57.03144
287967	cg1559575	16787451	67647405	0.4055644	0	1	0.2632014	-36.57495
288025	cg1559966	26719502	26719502	0.2066782	0	1	0.3164281	26.89626
288040	cg1560043	36605384	36605384	0.4717503	0	1	0.6113617	32.43219
288094	cg1560267	47615484	47615484	0.3326941	0	1	0.490962	40.61762
288230	cg1560937	18690463	18690463	0.2927623	0	1	0.4011092	22.45722
288270	cg1561160	23664305	23664305	0.3455444	0	1	0.1958846	-45.28833
288416	cg1561937	46684424	46684424	0.2569408	0	1	0.3698053	25.3173
288496	cg1562348	13644384	35762463		0	1		-4.34E-09
288514	cg1562433	44808445	44808445	0.6039252	0	1	0.7451347	39.82019
288544	cg1562611	42735374	42735374	0.5241941	0	1	0.4229557	-18.47078
288681	cg1563391	46646406	46646406	0.3989604	0	1	0.5426677	33.44701
288684	cg1563408	38800332	62793370	0.2735491	0	1	0.4794303	68.46638
288829	cg1564285	39634377	61726325	0.6330839	0	1	0.3727773	-103.7732
288939	cg1564838	31797330	31797330	0.3708084	0	1	0.2660149	-22.16624
289017	cg1565253	15619492	15619492	0.7789949	0	1	0.458664	-178.3552

289063	cg1565509?	24757507	24757507	0.7854072	0	1	0.8874848	41.11051
289206	cg1566394?	21660393	21660393	0.5446236	0	1	0.3776046	-43.86933
289296	cg1567145?	35775311	35775311	0.4335025	0	1	0.3173006	-24.42402
289409	cg1567768?	50760476	50760476	0.9209419	0	1	0.5869254	-264.3651
289463	cg1568060?	51706372	48692417	0.5279209	0	1	0.9219344	374.3439
289507	cg1568280?	60729467	58645414	0.2308179	0	1	0.0937619	-55.58208
289508	cg1568280?	29656395	71664408	0.3386456	0	1	0.2222958	-28.4366
289515	cg1568349?	66639442	56773445	0.3656646	0	1	0.4804579	23.05847
289520	cg1568395?	36756465	36756465	0.3988631	0	1	0.519715	24.82394
289534	cg1568466?	61802507	61802507	0.313911	0	1	0.4189591	20.83512
289634	cg1569034?	26736445	73634351	0.3847276	0	1	0.230592	-44.14248
289679	cg1569259?	11651429	11651429	0.3591137	0	1	0.5016841	33.34788
289701	cg1569393?	46625496	46625496	0.2390964	0	1	0.5342223	140.625
289800	cg1569908?	35717465	35717465	0.4633707	0	1	0.3552838	-21.03025
289809	cg1569955?	21738485	70790419	0.4202602	0	1	0.29074	-30.084
289909	cg1570428?	65743393	65743393	0.4439898	0	1	0.3284844	-23.92549
289921	cg1570487?	66726310	66726310	0.2194223	0	1	0.3821743	49.39448
289950	cg1570615?	23704322	37737444	0.3191231	0	1	0.2083462	-27.18039
289957	cg1570656?	47785393	47785393	0.6216803	0	1	0.3864862	-84.5145
290091	cg1571468?	57675362	57675362	0.2857705	0	1	0.4077911	27.45741
290120	cg1571662?	39768442	39768442	0.8639882	0	1	0.7491679	-43.13729
290138	cg1571761?	60736362	60736362	0.5141554	0	1	0.7141826	67.73473
290271	cg1572487?	62645487	62645487	0.5470525	0	1	0.6538692	21.69909
290293	cg1572612?	31721364	31721364	0.4948838	0	1	0.5972829	19.23647
290347	cg1572869?	12782313	12782313	0.3144147	0	1	0.4321859	25.03895
290352	cg1572890?	34605452	34605452	0.1586832	0	1	0.2641679	29.01709
290391	cg1573093?	17635330	17635330	0.4282073	0	1	0.302671	-28.1642
290415	cg1573210?	60715449	60715449	0.8033203	0	1	0.6805111	-37.4389
290511	cg1573717?	31610426	20809435	0.644744	0	1	0.488094	-40.59095
290559	cg1574050?	60703439	60703439	0.6519967	0	1	0.814938	60.56478
290731	cg1575275?	74780342	74780342	0.3042893	0	1	0.1663535	-42.99081
290805	cg1575800?	42601307	42601307	0.7563968	0	1	0.8847801	56.40752
290884	cg1576563?	43723466	43723466	0.9216133	0	1	0.7840503	-76.90823
290929	cg1576861?	16680480	16680480	0.8726946	0	1	0.6982785	-83.42019
291036	cg1577397?	44645334	16602440	0.4060423	0	1	0.6005511	58.38233
291129	cg1577989?	50778398	50778398	0.0772562	0	1	0.2202654	63.93254
291139	cg1578115?	66623406	66623406	0.3000196	0	1	0.164365	-42.12162
291177	cg1578384?	30621385	30621385	0.2053292	0	1	0.4678118	117.0072
291210	cg1578682?	26714352	50668485	0.4910823	0	1	0.3756546	-23.15709
291240	cg1578836?	40613314	25712368	0.4105665	0	1	0.3038189	-21.6258
291259	cg1579018?	32663490	32663490	0.3869713	0	1	0.5346084	35.12603
291295	cg1579325?	63613330	63613330	0.6148902	0	1	0.8512756	123.008
291368	cg1579886?	42764356	42764356	0.4842721	0	1	0.3686231	-23.30686

291401	cg1580134(	14789485	14789485	0.3185924	0	1	0.2002862	-30.74318
291546	cg1581143:	10741416	10741416	0.721324	0	1	0.5992697	-29.92173
291551	cg1581166{	34771443	39796501	0.5819454	0	1	0.4531812	-27.8314
291557	cg1581202(	19700315	69704377	0.3664055	0	1	0.2590937	-23.28341
291688	cg1581933:	15621481	15621481	0.5375273	0	1	0.4169301	-24.69383
291795	cg1582443:	59628421	59628421	0.5449327	0	1	0.6529014	22.04769
291800	cg1582470'	19774392	25755347	0.258644	0	1	0.1398395	-37.1557
291851	cg1582681(	22705486	22705486	0.3674149	0	1	0.5259826	40.05815
291899	cg1582908{	60661444	58706346	0.3333934	0	1	0.0945684	-129.0717
291916	cg1582996!	24632422	24632422	0.7869363	0	1	0.6837532	-27.15541
292089	cg1583794:	38745406	38745406	0.4334455	0	1	0.3178242	-24.2127
292134	cg1584055	17633478	17633478	0.6542606	0	1	0.5366246	-25.3569
292203	cg1584441!	24610482	24610482	0.2984421	0	1	0.4071066	22.39725
292227	cg1584536:	68737498	68737498	0.3902594	0	1	0.4915276	18.59779
292254	cg1584727:	43689412	43689412	0.3068841	0	1	0.4411722	31.35274
292318	cg1585073!	33748413	33748413	0.4322317	0	1	0.5345231	18.78564
292343	cg1585244(	33661343	33661343	0.2230374	0	1	0.326529	23.62165
292370	cg1585377l	13754467	13754467	0.5221677	0	1	0.8267791	374.3439
292426	cg1585747(	56623333	56623333	0.2959582	0	1	0.3993595	20.76337
292555	cg1586608!	51799376	51799376	0.3527398	0	1	0.5224009	45.46056
292672	cg1587462!	58630411	27706371	0.4265604	0	1	0.2542599	-51.22659
292677	cg1587470	60799356	60799356	0.2702819	0	1	0.3738493	21.60851
292718	cg1587752(	37656439	23680318	0.0196109	0	1	0.2571143	374.3439
292779	cg1588179:	17795419	17795419	0.419498	0	1	0.5259721	20.05973
292798	cg1588309	27730328	22789353	0.7549925	0	1	0.8878342	60.22037
292934	cg1589164:	12768347	12768347		0	1	0.8536991	4.34E-09
292999	cg1589512l	26673478	26673478	0.2039301	0	1	0.3122772	26.54153
293005	cg1589533!	39716426	39716426	0.2067117	0	1	0.3952885	64.96644
293013	cg1589578:	25801508	25801508	0.3740968	0	1	0.5319797	39.67484
293045	cg1589743:	60706393	60706393	0.6563886	0	1	0.0305888	-371.3336
293082	cg1589980(	37754400	37754400	0.2039615	0	1	0.4269547	87.34054
293242	cg1590897:	22676328	22676328	0.6607521	0	1	0.5558169	-21.27987
293252	cg1590944:	64786316	64786316	0.504548	0	1	0.7298574	85.76961
293287	cg1591116:	66707467	66707467	0.3505954	0	1	0.4519971	19.04202
293289	cg1591132{	50775463	50775463	0.5212218	0	1	0.408108	-22.19075
293392	cg1591981(	57785429	57785429	0.553853	0	1	0.6805859	29.64871
293440	cg1592188'	48629388	48629388	0.369014	0	1	0.4694181	18.52171
293489	cg1592456{	34681444	34681444	0.6161091	0	1	0.768154	47.42164
293719	cg1593805(	54774355	54774355	0.2697149	0	1	0.3976899	30.37136
293773	cg1594333:	64668323	64668323	0.1599416	0	1	0.2600695	26.68869
293825	cg1594659(	11734316	11734316	0.5885413	0	1	0.4273521	-41.25087
293857	cg1594841(	13644396	13644396		0	1	0.8234274	4.34E-09
293861	cg1594856'	31711352	26660377	0.3239217	0	1	0.217796	-24.85804

293871	cg1594955	61651509	61651509	0.2849879	0	1	0.4103867	28.74076
293895	cg1595146	62699310	62699310	0.4925581	0	1	0.3758554	-23.58013
293988	cg1595780	26701411	26701411	0.6662806	0	1	0.7886481	35.53431
293993	cg1595842	66711467	66711467	0.5585309	0	1	0.3466837	-68.77795
294010	cg1595946	59777444	59777444	0.3233137	0	1	0.6102071	126.8275
294080	cg1596398	15749386	15749386	0.639066	0	1	0.7692351	37.05794
294082	cg1596408	66682491	66682491	0.5572676	0	1	0.7726239	85.6431
294092	cg1596459	14786324	14786324	0.5823003	0	1	0.824546	118.4976
294109	cg1596538	64680473	64680473	0.5152832	0	1	0.6257485	22.22233
294331	cg1597849	23618398	23618398	0.832001	0	1	0.9382123	61.17387
294348	cg1597921	53601493	53601493	0.8891808	0	1	0.5955695	-195.7253
294420	cg1598398	33741381	33741381	0.7431206	0	1	0.846382	33.9628
294464	cg1598646	10602301	10602301	0.5310578	0	1	0.647926	24.90479
294491	cg1598828	22760510	22760510	0.1877936	0	1	0.2957126	27.52131
294498	cg1598856	68758493	68758493	0.0654411	0	1	0.2173734	74.0075
294557	cg1599147	59631436	14688462	0.8087332	0	1	0.5736487	-108.1572
294688	cg1599812	55707472	36666312	0.4533195	0	1	0.6213974	45.12617
294716	cg1599960	19649402	19649402	0.3155867	0	1	0.4252364	22.27452
294794	cg1600279	33698330	33698330	0.7177676	0	1	0.8592756	57.60218
294877	cg1600696	32687493	32687493	0.5198901	0	1	0.634483	23.78078
294927	cg1600955	74615493	74615493	0.519361	0	1	0.4172172	-18.74693
295035	cg1601603	34673336	34673336	0.6765033	0	1	0.776667	25.15268
295317	cg1603197	63740383	63740383	0.2272378	0	1	0.331606	23.71395
295363	cg1603499	67689422	67689422	0.3590652	0	1	0.6618293	143.1114
295384	cg1603604	20755439	20755439	0.3115329	0	1	0.4154709	20.54051
295420	cg1603914	51755452	51755452	0.5743988	0	1	0.6890345	25.53341
295460	cg1604179	54771465	54771465	0.2919096	0	1	0.4153656	27.76176
295464	cg1604209	73674391	73674391	0.3126736	0	1	0.4162007	20.38541
295590	cg1604891	74727377	74727377	0.4100622	0	1	0.5258941	23.0819
295614	cg1605032	73642398	73642398	0.800872	0	1	0.9091603	51.03382
295672	cg1605300	10756465	53707335	0.9604284	0	1	0.8430364	-83.66013
295751	cg1605827	73783407	73783407	0.4229881	0	1	0.571316	35.43279
295764	cg1605916	53712386	53712386	0.6506754	0	1	0.8240236	68.79951
295779	cg1606018	15760496	15760496	0.8178083	0	1	0.6886759	-42.527
295791	cg1606093	38683455	38683455	0.4404846	0	1	0.8351066	374.3439
295978	cg1607427	45695325	45695325	0.7848669	0	1	0.6098012	-62.2982
295986	cg1607499	40742491	40742491	0.6037436	0	1	0.8779263	374.3439
296021	cg1607749	13809348	13809348	0.538367	0	1	0.6416232	20.26772
296025	cg1607792	39679313	39679313	0.300071	0	1	0.429366	29.65985
296044	cg1607941	65692337	65692337	0.6691603	0	1	0.7802979	29.83696
296079	cg1608185	14675462	14675462	0.4923023	0	1	0.3289247	-43.06474
296093	cg1608269	35618508	61792419	0.5282061	0	1	0.6289619	19.23876
296103	cg1608355	58644371	58644371	0.6779037	0	1	0.9147054	156.5356

296112	cg1608419(	44650375	44650375	0.2165583	0	1	0.3827069	51.42514
296180	cg1608794(	45777327	18702508	0.7360705	0	1	0.602642	-35.60983
296229	cg1609195:	65657362	49721438	0.8345875	0	1	0.7122411	-41.70685
296416	cg1610273(	43808361	43808361	0.5259605	0	1	0.6435567	25.03713
296461	cg1610556(	38611458	38611458	0.6105332	0	1	0.7709132	52.08637
296526	cg1610938	32601352	32601352	0.341498	0	1	0.4868291	34.8135
296571	cg1611222(	27622453	32647322	0.460725	0	1	0.566128	19.84559
296578	cg1611288(	26722406	26722406	0.1070186	0	1	0.6581023	374.3439
296677	cg1611837(	21779474	21779474	0.3704955	0	1	0.5002744	28.2426
296837	cg1612768:	10784337	70648368	0.68599	0	1	0.3533891	-176.5426
296937	cg1613606(	69748499	69748499	0.4636084	0	1	0.5882282	26.40809
297053	cg1614290(	26759327	40629405	0.9596592	0	1	0.5679796	-371.3336
297070	cg1614365	55661306	47767338	0.8162634	0	1	0.6817343	-45.06232
297104	cg1614518	55618305	55618305	0.1897126	0	1	0.4727503	138.023
297187	cg1614992	41734416	29799492	0.6931872	0	1	0.580834	-24.89487
297282	cg1615611:	70607365	73797430	0.8677649	0	1	0.4794197	-302.0879
297352	cg1616293(	33786330	68681333	0.1176563	0	1	0.5612452	374.3439
297428	cg1616756:	23663418	23663418	0.895403	0	1	0.7009342	-108.1169
297512	cg1617209(	42695463	57651379	0.3057898	0	1	0.442541	32.36049
297604	cg1617660(	15652470	15652470	0.3825884	0	1	0.4861941	19.35153
297629	cg1617773:	18663429	18663429	0.4307377	0	1	0.3204401	-22.37334
297634	cg1617783(	26746466	26746466	0.3759551	0	1	0.5044276	27.70184
297684	cg1618079(	30757342	47612460	0.3018042	0	1	0.1758615	-36.22601
297784	cg1618752	34809454	34809454	0.8267871	0	1	0.9380893	65.02676
297813	cg1618964:	63781485	63781485	0.1666109	0	1	0.2711007	27.87654
297820	cg1618995:	45679329	45683363	0.7493176	0	1	0.6130059	-37.96711
297852	cg1619129	59785357	59785357	0.804257	0	1	0.6788486	-38.7953
297864	cg1619257:	56802419	56802419	0.4782598	0	1	0.3554277	-25.94555
297979	cg1619974	27783418	27783418	0.3008367	0	1	0.4369922	32.29897
298025	cg1620225(	61760497	15748418	0.8013321	0	1	0.9374048	84.20977
298086	cg1620481	48789442	68603443	0.2700917	0	1	0.1682116	-26.68349
298146	cg1620805:	34695480	32786455	0.7619013	0	1	0.5333392	-92.95425
298187	cg1621035:	44803341	44803341	0.6491815	0	1	0.7598598	28.00613
298201	cg1621097(	27661497	27661497	0.4768126	0	1	0.6433909	45.13708
298240	cg1621415:	52613413	17673389	0.7426543	0	1	0.8447846	33.17427
298530	cg1623379	19723383	19723383	0.3168474	0	1	0.5361595	74.35435
298537	cg1623433:	36711428	36711428	0.8675169	0	1	0.7627874	-38.32973
298556	cg1623570	60718451	60718451	0.2543268	0	1	0.0757533	-89.16023
298936	cg1625566:	63743407	63636440	0.6610634	0	1	0.8870625	130.0716
298977	cg1625753:	12611483	12611483	0.543346	0	1	0.6921868	39.38009
299061	cg1626185	71706473	71706473	0.6178725	0	1	0.7186968	22.01233
299084	cg1626318(	62685459	62685459	0.5023757	0	1	0.3496596	-37.76519
299280	cg1627305	23623343	23623343	0.2626429	0	1	0.3763061	25.35659

299466	cg1628443	53698374	51644413	0.8543755	0	1	0.742242	-39.81041
299747	cg1630055	27665417	27665417	0.5107633	0	1	0.6154389	20.19589
299819	cg1630607	24761450	24761450	0.7118324	0	1	0.813684	29.14816
299897	cg1631000	57621323	57621323	0.1819182	0	1	0.295949	30.47712
299912	cg1631095	59700357	46809418	0.4202176	0	1	0.1944596	-90.85233
300028	cg1631647	64721346	10688333	0.2860854	0	1	0.1261556	-61.7923
300032	cg1631662	44670340	45663379	0.3008173	0	1	0.1814234	-32.79126
300122	cg1632088	26742496	49705394	0.6988859	0	1	0.516909	-56.10768
300305	cg1633014	30726435	17606402	0.3146737	0	1	0.2015186	-28.57712
300415	cg1633655	52782469	52782469	0.6450634	0	1	0.7686647	34.09024
300432	cg1633756	11661353	11661353	0.2116314	0	1	0.3173281	25.06113
300515	cg1634262	46759427	46759427	0.5894082	0	1	0.7625558	57.92387
300562	cg1634556	52757468	52757468	0.8610928	0	1	0.622734	-128.6332
300707	cg1635434	37758333	37758333	0.8550025	0	1	0.9588401	70.827
300726	cg1635559	27706301	27706301	0.4721243	0	1	0.6287096	40.02522
300807	cg1635979	63684417	63684417	0.612131	0	1	0.5057077	-20.6846
301019	cg1637122	49810458	49810458	0.1575115	0	1	0.2642069	29.64285
301045	cg1637263	15747371	25733311	0.3430685	0	1	0.4457642	19.54447
301105	cg1637582	56793477	56793477	0.3700885	0	1	0.5091652	31.79854
301296	cg1638704	49634405	49634405	0.4401257	0	1	0.6162294	48.9238
301409	cg1639401	35675418	38755351	0.3915149	0	1	0.2194849	-54.21147
301462	cg1639703	31606396	68796418	0.877056	0	1	0.7579713	-48.57305
301465	cg1639717	25706345	25706345	0.2848352	0	1	0.3934794	22.80201
301481	cg1639796	15795447	15795447	0.9461744	0	1	0.7680312	-125.7757
301482	cg1639805	67633365	34624302	0.2832056	0	1	0.4802187	62.57718
301493	cg1639858	54711381	54711381	0.193644	0	1	0.2945955	24.43346
301542	cg1640063	15771335	39755401	0.5615179	0	1	0.3870193	-47.48415
301647	cg1640543	66643403	66643403	0.3008986	0	1	0.4091407	22.19017
301676	cg1640689	43608374	43608374	0.4580687	0	1	0.6559061	62.08949
301798	cg1641274	68659506	68659506	0.7028409	0	1	0.8960948	107.4835
301819	cg1641403	58663492	58663492	0.6993729	0	1	0.5956667	-22.16922
301832	cg1641469	39688492	39688492	0.4985638	0	1	0.6126729	23.17031
301845	cg1641564	18638488	18638488	0.4440588	0	1	0.5630692	24.22421
301858	cg1641604	43706325	55629312		0	1		-4.34E-09
301899	cg1641818	63772319	63772319	0.3213491	0	1	0.4459097	27.26475
301936	cg1641958	46647457	74795414	0.4279406	0	1	0.2672607	-44.59674
301972	cg1642141	54701456	61679335	0.4070428	0	1	0.5421903	30.0289
302000	cg1642309	42782411	42782411	0.4565434	0	1	0.2738515	-55.25969
302055	cg1642629	63806360	18607354	0.5576801	0	1	0.378323	-49.98666
302095	cg1642835	12763437	12702443	0.7734976	0	1	0.5507129	-90.97511
302096	cg1642851	19643510	19643510	0.5066665	0	1	0.6443894	32.56012
302179	cg1643290	56670338	56670338	0.6633084	0	1	0.769434	26.92648
302231	cg1643568	63603337	63603337	0.7213338	0	1	0.5164245	-71.45725

302256	cg1643676[illegible]	27747312	27747312	0.1962378	0	1	0.3071615	28.07812
302309	cg1644005[illegible]	61623436	42729406	0.3308452	0	1	0.0536269	-186.7895
302465	cg1644963[illegible]	11731360	11731360	0.2176924	0	1	0.329705	27.05786
302478	cg1645030[illegible]	36715420	71656455	0.5377487	0	1	0.3972445	-32.15514
302492	cg1645130[illegible]	74623409	74623409	0.1987959	0	1	0.3318265	37.18051
302533	cg1645305[illegible]	59689453	59689453	0.5804921	0	1	0.3858415	-58.27675
302565	cg1645533[illegible]	57651414	57651414	0.4614637	0	1	0.5655559	19.44292
302615	cg1645849[illegible]	66632451	10603352	0.3382264	0	1	0.2259826	-26.65221
302646	cg1646038[illegible]	61602507	61602507	0.7098797	0	1	0.6077645	-22.06393
302752	cg1646731[illegible]	13644470	13644470		0	1		-4.34E-09
302901	cg1647643[illegible]	53721323	53721323	0.8507974	0	1	0.7275891	-45.09935
302919	cg1647761[illegible]	39660439	39660439	0.2845065	0	1	0.4756728	59.19325
302953	cg1647963[illegible]	44641326	44641326	0.2809988	0	1	0.4079246	29.47717
302978	cg1648128[illegible]	54714317	54714317	0.0884219	0	1	0.1898353	36.52767
303022	cg1648449[illegible]	32709458	32709458	0.8626612	0	1	0.7244192	-56.4691
303112	cg1649012[illegible]	62797474	62797474	0.2005358	0	1	0.4911712	142.7335
303174	cg1649323[illegible]	66732473	66732473	0.4227222	0	1	0.5772014	38.12344
303193	cg1649462[illegible]	73728435	73728435	0.6162831	0	1	0.9405329	374.3439
303298	cg1650156[illegible]	73623317	73623317	0.3249343	0	1	0.4308199	20.85961
303394	cg1650756[illegible]	31743395	31743395	0.7073681	0	1	0.5677001	-36.29979
303531	cg1651408[illegible]	28678378	28678378	0.475455	0	1	0.2979321	-51.17556
303538	cg1651451[illegible]	17773335	58738411	0.3385929	0	1	0.4418839	19.79904
303547	cg1651538[illegible]	35604436	35604436	0.6460012	0	1	0.9054773	374.3439
303563	cg1651629[illegible]	64797394	64797394	0.3171358	0	1	0.4210356	20.40195
303802	cg1652709[illegible]	61749339	61749339	0.2368721	0	1	0.0880785	-64.43883
303821	cg1652762[illegible]	21787337	21787337	0.4645038	0	1	0.610768	35.12384
303906	cg1653162[illegible]	10689391	10689391	0.4064905	0	1	0.5249382	23.97155
303938	cg1653314[illegible]	14607344	14607344	0.3827396	0	1	0.5137035	28.55811
303985	cg1653565[illegible]	58647457	58647457	0.8529467	0	1	0.5995213	-138.3229
304078	cg1654059[illegible]	61661377	61661377	0.2752401	0	1	0.4584503	55.44495
304228	cg1654964[illegible]	69706449	55803491	0.5502781	0	1	0.6652904	24.78964
304238	cg1655026[illegible]	70779475	43672350	0.2475024	0	1	0.3586972	25.14898
304413	cg1655899[illegible]	54799486	54799486	0.4595723	0	1	0.231154	-87.23939
304527	cg1656482[illegible]	11720494	11720494	0.8149909	0	1	0.929245	63.03281
304556	cg1656619[illegible]	67803420	67803420	0.3910403	0	1	0.5354303	33.75585
304589	cg1656813[illegible]	38617479	16743341	0.4062205	0	1	0.2888528	-25.67035
304636	cg1657088[illegible]	40644306	40644306	0.9421397	0	1	0.8358577	-63.18084
304730	cg1657554[illegible]	38718324	38718324	0.2267781	0	1	0.3739387	41.18276
304747	cg1657654[illegible]	30685373	30685373	0.8808911	0	1	0.7794911	-39.23412
304757	cg1657693[illegible]	15746497	15746497	0.7899035	0	1	0.9267425	78.92889
304892	cg1658241[illegible]	31791419	56778505	0.1912797	0	1	0.3279531	39.56192
304916	cg1658355[illegible]	40659477	53641413	0.6600785	0	1	0.3371151	-164.2329
305190	cg1659631[illegible]	10704506	10704506	0.7506962	0	1	0.8574037	37.50733

305273	cg16600991	19676476	72679384	0.2252383	0	1	0.0710652	-73.51198
305298	cg1660231(	11806443	46648400	0.4157865	0	1	0.2781084	-33.8511
305316	cg1660357{	44617361	22607427	0.6547235	0	1	0.7649465	28.20552
305361	cg1660632⁄	10633509	48719469	0.7597994	0	1	0.6188178	-41.10265
305434	cg1661207:	21619432	21619432	0.0443862	0	1	0.1828281	72.85333
305483	cg1661535'	26703479	26703479	0.5768176	0	1	0.4494686	-27.26311
305524	cg1661725'	41734407	41734407	0.782349	0	1	0.9090788	63.54969
305631	cg1662289!	22712323	39604492	0.2607599	0	1	0.3792334	27.18361
305632	cg1662290(	32638312	13689444	0.294816	0	1	0.190916	-25.73115
305645	cg16623941	43748403	43748403	0.5894347	0	1	0.7088113	28.19176
305723	cg1662723(	15764432	15764432	0.1191729	0	1	0.2219666	32.33538
305742	cg16627911	55773343	51629453	0.736227	0	1	0.8440018	35.82656
305902	cg1663745{	33670508	33670508	0.2075808	0	1	0.3339121	33.51619
305979	cg1664163{	42786453	54806449	0.4604594	0	1	0.3476726	-22.65611
306114	cg1664972{	36757390	51722444	0.3434061	0	1	0.563465	74.05846
306145	cg1665131⁄	37671494	37671494	0.2960402	0	1	0.4615102	45.30652
306189	cg1665292(	40788502	40788502	0.2378091	0	1	0.0464104	-112.8698
306232	cg16655291	74683493	74683493	0.8658202	0	1	0.3885923	-371.3336
306236	cg1665534:	66739312	66739312	0.6995358	0	1	0.5978683	-21.49688
306270	cg1665687:	10784438	10784438	0.7918776	0	1	0.892424	41.40363
306304	cg1665846(	20777316	20777316	0.3123513	0	1	0.4344998	26.63876
306337	cg1665977:	41677386	41677386	0.2543658	0	1	0.4163984	46.10269
306352	cg1666049⁄	57758504	57758504	0.5304791	0	1	0.656203	28.33627
306406	cg1666293{	40787327	40787327	0.6420974	0	1	0.4811496	-42.44855
306456	cg1666531(	57698390	25800460	0.844126	0	1	0.7240322	-42.16996
306590	cg1667352:	49780451	49780451	0.5190889	0	1	0.6816107	45.05682
306675	cg1667815!	42723496	42723496	0.3297348	0	1	0.433183	20.00537
306748	cg1668164!	54754423	54754423	0.297895	0	1	0.4171609	26.04423
306779	cg1668306(	42782316	42782316	0.3550943	0	1	0.5127164	39.82182
306819	cg1668613:	12638432	12638432	0.4085988	0	1	0.5289063	24.6034
306935	cg1669273:	16718443	33758489	0.4884665	0	1	0.3656879	-25.78051
307086	cg1670002:	61810327	61810327		0	1		-4.34E-09
307151	cg1670266(	16631322	16631322	0.2021059	0	1	0.5348747	374.3439
307162	cg1670313:	46661465	72773385	0.5582091	0	1	0.3636516	-58.30186
307188	cg1670447(	12688449	12688449	0.0817437	0	1	0.2097961	53.19697
307193	cg1670470:	15797495	15797495	0.3770152	0	1	0.6409682	106.982
307233	cg1670582'	54802377	54802377	0.8086214	0	1	0.6263986	-71.02461
307341	cg16711011	48686329	61723395	0.2168174	0	1	0.3428429	32.68825
307393	cg16713321	50792364	50792364	0.2860757	0	1	0.3891236	20.93171
307398	cg1671362:	63809322	61685313	0.8455455	0	1	0.9582438	78.03788
307443	cg1671631(	61673380	13651442	0.1714536	0	1	0.0689733	-41.17925
307514	cg1671951'	46679337	32778503	0.0378919	0	1	0.1600098	63.81693
307580	cg1672288⁄	32800508	32800508	0.3342546	0	1	0.4412891	21.04006

307582	cg16722947	30626503	30626503	0.6148582	0	1	0.5125078	-19.4743
307595	cg16723716	34633487	57732437	0.5850449	0	1	0.7472765	50.25274
307608	cg16724332	72634429	72634429	0.2595156	0	1	0.4067252	38.89855
307616	cg16724720	14750373	14750373	0.7990934	0	1	0.9046678	47.8469
307666	cg16727923	26754342	26754342	0.1344609	0	1	0.2439864	33.47785
307680	cg16728846	66656509	66656509	0.2045223	0	1	0.3068113	24.23813
307792	cg16735072	38734362	38734362	0.4532207	0	1	0.6243594	46.68914
307917	cg16742075	65711321	65711321	0.4979563	0	1	0.7718205	131.0456
307951	cg16744531	57607381	73703501	0.257453	0	1	0.1163693	-52.81129
307960	cg16744961	60757411	60757411	0.7245603	0	1	0.9075423	105.0645
308011	cg16747885	39769375	52600460	0.443316	0	1	0.5572358	22.49093
308012	cg16747928	74666366	74666366	0.4367957	0	1	0.5421858	19.73533
308016	cg16748088	67644496	67644496	0.4314348	0	1	0.3243737	-21.2435
308020	cg16748433	62647492	62647492	0.5719034	0	1	0.6731833	20.47256
308056	cg16751493	61726311	61726311	0.2452411	0	1	0.3712898	30.86982
308107	cg16754788	43663360	43663360	0.3248268	0	1	0.4470391	26.33488
308125	cg16755833	46701389	11653323	0.1524975	0	1	0.2664401	33.34221
308287	cg16765068	22662319	22662319	0.3019964	0	1	0.436637	31.65876
308330	cg16767880	49709439	30660451	0.9162823	0	1	0.7223555	-118.9578
308345	cg16768697	63725310	11665446	0.5231948	0	1	0.6249443	19.46543
308446	cg16775598	26788369	26788369	0.7435698	0	1	0.6005	-40.53691
308491	cg16778382	29686413	57612478	0.3670968	0	1	0.5224723	38.64037
308520	cg16780454	17718342	17718342	0.0984927	0	1	0.2153134	42.87732
308546	cg16781907	15780398	15780398	0.3298245	0	1	0.4573131	28.12286
308599	cg16784985	65766375	63699429	0.819681	0	1	0.6825874	-46.96021
308604	cg16785291	11645344	11645344	0.2530811	0	1	0.363526	24.62783
308652	cg16787352	19624446	19624446	0.3068649	0	1	0.4165805	22.51416
308661	cg16788099	10720331	70768333	0.166477	0	1	0.2864305	34.51417
308674	cg16788857	53651382	53651382	0.6149881	0	1	0.3540419	-103.9828
308681	cg16789104	39704468	45642388	0.3500941	0	1	0.2359237	-26.81894
308734	cg16791832	37644405	37644405	0.3776696	0	1	0.6674838	130.8536
308928	cg16802801	11725370	11725370	0.2910279	0	1	0.1611296	-39.73665
308956	cg16803846	29616304	61796314	0.4682773	0	1	0.3575729	-21.8313
309056	cg16810310	57768347	57768347	0.4410996	0	1	0.3279156	-23.15978
309120	cg16814680	11738460	11738460	0.6324946	0	1	0.286193	-192.0642
309232	cg16822572	73715405	21760444	0.4654938	0	1	0.3476109	-24.35985
309325	cg16829924	48698301	64800418	0.5834819	0	1	0.729309	40.59598
309398	cg16835531	10670397	10670397	0.7866992	0	1	0.915653	67.80871
309410	cg16836675	50727449	50727449	0.717431	0	1	0.8971791	97.38839
309451	cg16841014	22679452	22679452	0.3930252	0	1	0.5743163	50.97884
309550	cg16848221	61804390	61804390	0.3043615	0	1	0.6119846	147.5047
309614	cg16852369	37748353	37748353	0.7060506	0	1	0.831935	43.10379
309652	cg16854917	10729435	51693408	0.4490778	0	1	0.3345491	-23.47068

309654	cg16855352	43625305	72735337	0.9553673	0	1	0.8485118	-71.16553
309724	cg16860392	17760330	17760330	0.2437899	0	1	0.3507501	23.8213
309774	cg16863522	37648364	37648364	0.6484804	0	1	0.7648216	30.67758
309792	cg16864658	54668354	54668354	0.2375043	0	1	0.1175235	-41.09077
309834	cg16867584	44708468	23715328	0.2868857	0	1	0.1708175	-32.36101
309880	cg16871435	21642333	21642333	0.2895208	0	1	0.5709014	123.0877
309926	cg16874185	35752411	35752411	0.4697155	0	1	0.5721605	18.99507
309977	cg16877087	61683422	10674439	0.8488013	0	1	0.7204029	-47.57346
309982	cg16877681	46660353	46660353	0.5774596	0	1	0.7062239	31.71028
310172	cg16890563	27803308	27803308	0.3809054	0	1	0.5396811	40.01845
310289	cg16898193	33771462	33771462	0.3488662	0	1	0.1806671	-56.89983
310397	cg16904261	15693510	15693510	0.4986694	0	1	0.6484993	37.72736
310503	cg16908938	70749340	70749340	0.6024926	0	1	0.7669861	53.77276
310594	cg16914370	20636316	44607314	0.5624067	0	1	0.4115275	-36.47301
310633	cg16917903	44682343	44682343	0.5917243	0	1	0.9149014	374.3439
310692	cg16921727	35751384	35751384	0.4049443	0	1	0.2390969	-49.16751
310842	cg16929496	60667430	60667430	0.3992323	0	1	0.2893681	-23.06636
310845	cg16929739	15669433	15669433	0.6979161	0	1	0.8317567	47.30434
310871	cg16931770	70737340	51735392	0.2838404	0	1	0.4363847	39.93094
310873	cg16931822	45695381	45695381	0.6065266	0	1	0.7144932	24.29393
310892	cg16932827	42802482	42802482	0.1766343	0	1	0.2826393	27.63946
310942	cg16936421	71731399	46620479	0.7787382	0	1	0.8790859	38.26331
311321	cg16967578	35635502	35635502	0.7280852	0	1	0.6147336	-26.971
311460	cg16977510	33808376	33808376	0.2090309	0	1	0.0997078	-38.63337
311609	cg16988212	48614330	48614330	0.6057853	0	1	0.7603608	47.7957
311629	cg16989201	37653313	37653313	0.8342689	0	1	0.6999672	-48.01468
311801	cg16999994	30712357	30712357	0.8195337	0	1	0.9437702	78.41379
311926	cg17007628	30775418	30775418	0.7206043	0	1	0.5803663	-37.4313
311963	cg17009813	36691300	36691300	0.2850574	0	1	0.3863674	20.40832
312019	cg17013691	14613376	11784376	0.4846376	0	1	0.6648617	52.93319
312059	cg17015844	73809492	73809492	0.1552042	0	1		-4.34E-09
312108	cg17017591	25761437	55660322	0.5362527	0	1	0.3201582	-72.17139
312252	cg17025908	31682354	31682354	0.4540306	0	1	0.9278698	374.3439
312357	cg17032848	47601482	47601482	0.6734195	0	1	0.7902061	33.20308
312379	cg17033622	20651472	20651472	0.7138301	0	1	0.5950636	-28.22553
312384	cg17033891	55690443	55690443	0.6958938	0	1	0.7965619	26.97454
312553	cg17044529	12616427	12616427	0.637351	0	1	0.8567682	111.3117
312593	cg17047033	32799388	32799388	0.6155395	0	1	0.7214644	23.90956
312617	cg17048610	29783469	46791320	0.8098223	0	1	0.6869699	-38.2911
312644	cg17050806	11688438	11688438	0.2633587	0	1	0.1281915	-47.27519
312650	cg17051124	15719372	15719372	0.7618254	0	1	0.8727172	42.79929
312733	cg17055585	19643331	56656336	0.9522556	0	1	0.840888	-72.99932
312751	cg17056132	14802443	14802443	0.2745326	0	1	0.4125088	34.14316

312780	cg17058383	70617305	70617305	0.2349265	0	1	0.3622519	31.99718
312789	cg17059388	18662368	18662368	0.6210375	0	1	0.4222621	-61.2467
312868	cg17066275	12657472	12657472	0.2339485	0	1	0.3453608	25.9043
312941	cg17070988	27606354	71630392	0.6812385	0	1	0.5726216	-23.14277
312991	cg17074339	11762494	11762494	0.1817244	0	1	0.3711454	69.10555
313095	cg17080138	25603418	25603418	0.5520417	0	1	0.6911724	35.13004
313142	cg17082366	43632386	55654463	0.6797357	0	1	0.5235894	-41.96865
313187	cg17084358	58779333	58779333	0.7881279	0	1	0.8913883	42.75457
313324	cg17093485	22676510	32753378	0.1850691	0	1	0.0827493	-38.10448
313380	cg17096289	33628390	46699443	0.6084637	0	1	0.7585656	45.36456
313390	cg17097293	30690393	30690393	0.5719228	0	1	0.6901979	26.89802
313411	cg17098979	46633412	46633412	0.6726079	0	1	0.5181701	-40.81506
313430	cg17100047	14740471	14740471	0.2737275	0	1	0.3839539	23.7196
313650	cg17112812	23659497	64687370	0.1542993	0	1	0.2853813	41.18405
313828	cg17125742	58737438	50649421	0.4678189	0	1	0.2911248	-51.09901
313845	cg17126924	44698367	44698367	0.3454106	0	1	0.5655248	74.05824
313972	cg17133045	54744446	54744446	0.3173677	0	1	0.4232549	21.02234
314032	cg17137077	52762384	70634493	0.7828429	0	1	0.6597117	-35.26468
314133	cg17143179	61729372	61729372	0.5869079	0	1	0.6881887	20.95678
314253	cg17149911	20707449	20707449	0.3502455	0	1	0.2475593	-22.18562
314274	cg17151123	21770331	21770331	0.7294517	0	1	0.8515061	44.60593
314288	cg17152375	49763499	74665311	0.5732834	0	1	0.450019	-25.77177
314352	cg17155524	21615390	21615390	0.4247264	0	1	0.2205589	-72.628
314476	cg17162031	50706343	50706343	0.6594748	0	1	0.7654548	26.57577
314490	cg17162911	45807323	45807323	0.4370746	0	1	0.7390651	150.515
314512	cg17164093	55707310	55707310	0.4608911	0	1	0.5682883	20.47575
314516	cg17164345	60691426	60691426	0.4814672	0	1	0.5945619	22.557
314532	cg17165241	69692400	23810364	0.4058194	0	1	0.6309155	77.85081
314556	cg17166262	67633425	67633425	0.2363582	0	1	0.3891475	43.03986
314578	cg17167832	40617361	40617361	0.526028	0	1	0.6635563	33.17897
314605	cg17168875	13743444	13743444	0.9080226	0	1	0.7999794	-50.49981
314782	cg17178175	69748353	69748353	0.4883425	0	1	0.5944995	20.34852
314798	cg17178900	24757439	18682492	0.498817	0	1	0.37844	-24.80291
314832	cg17180633	60646378	60646378	0.2305414	0	1	0.3977483	50.60918
314854	cg17181941	40626314	40626314	0.8354923	0	1	0.9446734	66.80719
314939	cg17186103	43674488	43674488	0.3848926	0	1	0.5046564	24.53608
314977	cg17187785	26692361	26692361	0.9392979	0	1	0.7456157	-134.2347
315053	cg17193417	51661351	51661351	0.6275207	0	1	0.5107348	-24.36572
315078	cg17196051	58774337	58774337	0.3673225	0	1	0.5408107	47.11081
315079	cg17196155	24754456	24754456	0.4553932	0	1	0.2634436	-61.09358
315104	cg17198051	69694432	69694432	0.5403956	0	1	0.6477695	21.72118
315224	cg17205386	53759508	53759508	0.9279098	0	1	0.8139494	-62.21404
315252	cg17207064	36718408	36718408	0.5989796	0	1	0.6996297	21.16547

315265	cg1720772	38676425	64765509	0.5587221	0	1	0.4354554	-25.67554
315300	cg1720976	57677340	57677340	0.6388379	0	1	0.8686441	124.6407
315386	cg1721440	74666430	66699412	0.7335418	0	1	0.6210537	-26.96424
315421	cg1721609	34794502	34794502	0.2623729	0	1	0.3698858	23.22175
315499	cg1722005	70646350	70646350	0.5675711	0	1	0.6704938	20.91582
315502	cg1722023	73774471	73774471	0.7286312	0	1	0.5014601	-86.89155
315518	cg1722131	42636396	42636396	0.2417163	0	1	0.1352751	-31.95987
315595	cg1722560	66787301	66787301	0.547062	0	1	0.4312138	-23.09057
315786	cg1724019	28741440	28741440	0.5183623	0	1	0.6210603	19.68481
315818	cg1724235	34734432	65731463	0.309887	0	1	0.1822532	-36.33825
315885	cg1724692	28738380	28738380	0.7823387	0	1	0.9101256	64.70179
315897	cg1724826	55762375	55762375	0.3507597	0	1	0.5487017	60.4046
315915	cg1725008	47751472	47751472	0.3445971	0	1	0.2265216	-28.84521
315940	cg1725142	57779473	33693464	0.3362934	0	1	0.4737391	31.75559
315955	cg1725340	26789304	26789304	0.4790182	0	1	0.5863785	20.62759
315965	cg1725398	39748337	39748337	0.2525374	0	1	0.4157294	46.79807
316021	cg1725751	45621509	45621509	0.3996858	0	1	0.5209815	24.97415
316237	cg1727283	49706393	49706393	0.5756191	0	1	0.7173336	37.84914
316241	cg1727309	39645372	39645372	0.7340154	0	1	0.8704136	57.37006
316249	cg1727368	24688344	24688344	0.6154201	0	1	0.7484029	36.40722
316302	cg1727685	64668397	18768374	0.5530733	0	1	0.4367585	-23.26532
316305	cg1727719	44648340	74611303	0.5190808	0	1	0.3547378	-42.89906
316606	cg1729233	34808478	34808478	0.361363	0	1	0.1709611	-71.54261
316639	cg1729486	18639475	18639475	0.3305275	0	1	0.5502935	74.17619
316656	cg1729583	62803373	62803373	0.1444652	0	1	0.2635265	36.67221
316795	cg1730258	33720308	33720308	0.8750167	0	1	0.6803946	-99.18848
316949	cg1731254	69604352	69604352	0.4647867	0	1	0.579852	23.03453
317007	cg1731607	55632466	55632466	0.3441748	0	1	0.4555658	22.25906
317265	cg1733004	48702307	48702307	0.195129	0	1	0.328206	37.54128
317272	cg1733025	61784461	61784461	0.6078717	0	1	0.4697842	-31.75963
317305	cg1733175	28761395	28761395	0.8776218	0	1	0.694259	-91.76206
317345	cg1733376	61796381	61796381	0.1821711	0	1	0.2956882	30.24191
317351	cg1733397	60796449	60796449	0.1468582	0	1	0.2538436	30.87206
317357	cg1733426	64752491	65758332	0.7023993	0	1	0.8264081	41.24341
317374	cg1733500	70662462	70662462	0.498963	0	1	0.621905	26.3867
317491	cg1734134	52784434	52784434	0.4334098	0	1	0.5404131	20.22948
317533	cg1734318	62626346	62626346	0.4291308	0	1	0.6313007	63.53567
317564	cg1734493	37756330	38659340	0.4032894	0	1	0.5311553	27.3013
317565	cg1734503	50705397	55735450	0.5477233	0	1	0.427638	-24.52802
317575	cg1734545	35667417	35667417	0.2815877	0	1	0.4360527	40.90693
317606	cg1734717	65677341	65677341	0.5462165	0	1	0.4381846	-20.56333
317623	cg1734824	34619474	34619474	0.70469	0	1	0.4488943	-105.0591
317672	cg1735192	69628380	69628380	0.9051263	0	1	0.6971627	-124.3365

317737	cg1735587	32770399	32770399	0.8216988	0	1	0.6383107	-74.67133
317791	cg1736055	40713497	40713497	0.8668122	0	1	0.7439568	-48.32395
317893	cg1736781	44703447	44703447	0.2549354	0	1	0.4191778	47.13845
317923	cg1736969	59740473	59740473	0.0477143	0	1	0.2647149	133.794
317937	cg1737061	38638395	38638395	0.5760504	0	1	0.8337633	134.7172
318127	cg1738230	34648306	34648306	0.514142	0	1	0.6435462	29.30962
318169	cg1738412	14738462	14738462	0.6040882	0	1	0.7217646	28.24976
318181	cg1738476	12738359	12738359	0.6942544	0	1	0.8072348	33.36071
318205	cg1738570	72697330	72697330	0.9005679	0	1	0.7976526	-44.97609
318220	cg1738624	56635354	56635354	0.4652892	0	1	0.7341294	119.5755
318225	cg1738647	58756404	58756404	0.3918203	0	1	0.529277	30.97341
318261	cg1738877	42751448	42751448	0.8002326	0	1	0.3385616	-371.3336
318275	cg1738981	70801395	70801395	0.5726092	0	1	0.7516804	59.83557
318328	cg1739314	44749493	44749493	0.4870976	0	1	0.771948	140.9726
318364	cg1739555	13802506	13802506	0.1594226	0	1	0.2676848	30.12221
318426	cg1739928	60611360	63726326	0.5185749	0	1	0.4006864	-23.80233
318499	cg1740444	64711377	64711377	0.1780982	0	1	0.8940328	374.3439
318633	cg1741308	33798377	33798377	0.9032179	0	1	0.8017747	-44.80687
318663	cg1741532	57742313	57742313	0.5272639	0	1	0.6464673	25.67917
318666	cg1741538	38694432	38694432	0.7363901	0	1	0.6347489	-23.16039
318773	cg1742104	62610408	62610408	0.3787687	0	1	0.8002156	374.3439
318798	cg1742254	50708387	40618459	0.2792918	0	1	0.1688351	-30.15199
318804	cg1742291	54728465	54728465	0.4811867	0	1	0.6637718	54.11304
318834	cg1742506	40649492	40649492	0.6930914	0	1	0.819237	41.23119
318859	cg1742692	46713318	46713318	0.6871563	0	1	0.7988936	31.887
319092	cg1744219	63790401	63790401	0.5639232	0	1	0.691759	30.58777
319110	cg1744335	60715486	13669344	0.6261959	0	1	0.4969318	-28.78642
319156	cg1744576	43701337	61732401	0.3850749	0	1	0.272982	-24.39159
319220	cg1744995	31684386	22617405	0.5215914	0	1	0.4196442	-18.68473
319369	cg1745902	60658314	48644424	0.9362338	0	1	0.8108831	-75.00918
319397	cg1746036	56761388	56761388	0.911088	0	1	0.7285244	-106.6212
319557	cg1746845	66656324	66656324	0.1356811	0	1	0.2432187	32.41045
319660	cg1747371	49701335	49701335	0.2717582	0	1	0.4408355	48.38907
319684	cg1747478	19620473	19620473	0.6350321	0	1	0.5320301	-20.04622
319685	cg1747479	58636369	58636369	0.6840568	0	1	0.5197532	-46.05761
319733	cg1747757	42763346	42763346	0.5768563	0	1	0.7769432	76.2869
319817	cg1748211	46717450	40620377	0.2008136	0	1	0.3650017	52.12914
319826	cg1748242	16662422	16662422	0.7428482	0	1	0.8490774	35.85689
319938	cg1749019	35727500	35727500	0.2073877	0	1	0.3136049	25.51427
319957	cg1749130	54757498	54757498	0.2806529	0	1	0.4147887	32.30394
320193	cg1750479	21760487	21760487	0.354075	0	1	0.4714751	24.09091
320397	cg1751548	22677494	22677494	0.3620661	0	1	0.2371982	-30.86957
320419	cg1751624	55616388	55616388	0.1018966	0	1	0.2069097	36.02585

320438	cg17516997	20670464	20670464	0.5747991	0	1	0.6847063	23.70901
320483	cg17519156	13806418	13806418	0.3845546	0	1	0.4894503	19.72729
320667	cg17529386	53793338	59806471	0.8536293	0	1	0.6790617	-77.30141
320770	cg17536720	30749407	30749407	0.7884089	0	1	0.5484067	-106.8782
320779	cg17537073	60713348	12623339	0.025485	0	1	0.2123049	121.7978
320857	cg17543884	29697321	47758315	0.4321603	0	1	0.291725	-34.38303
320861	cg17544364	58678487	58678487	0.4909344	0	1	0.3664036	-26.39063
320862	cg17544584	61713469	61713469	0.7208605	0	1	0.8281993	33.34999
320901	cg17546649	56642318	56642318	0.4057481	0	1	0.270155	-33.38128
320988	cg17553149	50636422	40688384	0.4166142	0	1	0.3122639	-20.65311
321140	cg17564498	73792452	73792452	0.7568338	0	1	0.8598211	36.00212
321169	cg17566592	13668422	13668422	0.3731334	0	1	0.2482409	-30.24781
321214	cg17569842	47650435	47650435	0.2415787	0	1	0.3566008	26.8371
321365	cg17581485	31794477	31794477	0.2939709	0	1	0.4307061	32.78956
321402	cg17583957	61737317	61737317	0.1877052	0	1	0.3593203	57.81299
321450	cg17586885	24687467	20660370	0.2192692	0	1	0.3204122	22.99001
321484	cg17588704	11801433	11801433	0.6683885	0	1	0.9175507	374.3439
321488	cg17588812	33720486	49762315	0.8055172	0	1	0.9221388	61.65353
321513	cg17590135	46682420	64784322	0.7989658	0	1	0.9452695	97.66172
321566	cg17593342	63762391	63762391	0.5644003	0	1	0.6934122	31.13088
321620	cg17597639	32787408	32787408	0.2671093	0	1	0.1512168	-34.37271
321679	cg17601785	24640334	24640334	0.4396382	0	1	0.5668515	27.13306
321806	cg17611045	51783342	12662447	0.5262827	0	1	0.4256533	-18.28923
321822	cg17611936	70725376	53803433	0.2984665	0	1	0.1012902	-93.50549
321845	cg17613924	44667300	21715415	0.6294596	0	1	0.7621455	37.50827
321906	cg17619311	62762491	33638303	0.166312	0	1	0.0357217	-70.94254
321991	cg17626671	19642505	19642505	0.897045	0	1	0.6271537	-176.2052
322045	cg17631184	49774438	49774438	0.3615149	0	1	0.4919427	28.60144
322080	cg17633431	60767302	60767302	0.3928355	0	1	0.5109309	23.91256
322141	cg17639056	60754322	27808404	0.5927973	0	1	0.4899378	-19.32051
322202	cg17643624	10698454	10698454	0.4193567	0	1	0.5291328	21.09607
322240	cg17646499	21603415	21603415	0.9040995	0	1	0.7855889	-56.12054
322387	cg17655970	38694502	26658331	0.3223683	0	1	0.1735205	-47.55289
322440	cg17658874	38622405	38622405	0.8886123	0	1	0.7086577	-93.71603
322493	cg17662493	73732393	73732393	0.7414938	0	1	0.5539048	-63.4436
322808	cg17687367	46774480	46774480	0.5553201	0	1	0.676038	27.21032
322883	cg17694130	11600347	35689408	0.2398912	0	1	0.3603394	28.98125
322902	cg17695537	62691303	62691303	0.165698	0	1	0.2828199	33.33599
323032	cg17706972	40715497	40715497	0.2344802	0	1	0.3579162	30.46444
323037	cg17707274	29773376	29773376	0.2653793	0	1	0.4034644	34.65122
323047	cg17707870	43670432	43670432	0.2016619	0	1	0.5491044	374.3439
323093	cg17711541	57621337	57621337	0.5150888	0	1	0.3231969	-57.73568
323116	cg17713488	29754432	57727428	0.7593654	0	1	0.6153446	-42.49911

323143	cg17714861	39643303	48785319	0.9602498	0	1	0.7545544	-163.1745
323161	cg1771610(	15612475	40706329	0.3110152	0	1	0.1865028	-34.54255
323281	cg1772320(	20761434	20761434	0.0307993	0	1	0.2023662	105.5932
323401	cg1773197:	50780340	11685308	0.6783835	0	1	0.9718837	374.3439
323458	cg1773559:	61740315	61740315	0.5844696	0	1	0.4331921	-36.80703
323495	cg1773777(	29669336	50711333	0.4376936	0	1	0.651701	71.56374
323507	cg17738521	64607510	64607510	0.6255415	0	1	0.7284933	23.21321
323508	cg1773861:	33678338	33678338	0.288736	0	1	0.1093102	-78.48743
323561	cg1774144(	15746504	15746504	0.4965869	0	1	0.3914568	-19.75024
323621	cg1774487(	51762459	51762459	0.6005998	0	1	0.7913635	72.97719
323701	cg17749961	47763379	47763379	0.5499074	0	1	0.2503045	-143.4812
323776	cg1775447:	12657385	72717470	0.9028391	0	1	0.7894499	-52.32525
323797	cg1775548:	73631423	73631423	0.4016082	0	1	0.5381541	30.58023
323865	cg1775925:	33732332	49729500	0.6568346	0	1	0.3430696	-154.1397
323875	cg1775959:	69703437	69703437	0.8257265	0	1	0.9414295	70.22217
323896	cg1776126:	70764415	70764415	0.7970147	0	1	0.9074135	51.85064
323926	cg1776356(	44796313	44796313	0.7114335	0	1	0.8280375	37.78325
323936	cg1776483(	29623345	29623345	0.6360726	0	1	0.7413979	24.76346
323939	cg1776499:	38698498	38698498	0.1417339	0	1	0.2513158	32.61374
324096	cg1777657(	30768498	42792354	0.5355801	0	1	0.4085654	-26.97957
324153	cg1778044:	41752359	41752359	0.7228076	0	1	0.6220676	-22.17176
324206	cg1778369:	24653317	24653317	0.5680506	0	1	0.676379	22.87674
324228	cg1778525(	31652419	60723394	0.6837915	0	1	0.5587871	-29.12904
324363	cg1779481:	58733440	39617452	0.4388123	0	1	0.332496	-20.84645
324413	cg1779794(	54673319	54673319	0.2829642	0	1	0.4066657	28.18496
324492	cg1780326(	12663410	12663410	0.915674	0	1	0.5688277	-276.4792
324505	cg1780411:	71690317	21626456	0.4317781	0	1	0.2525479	-54.98187
324586	cg1780818:	22641331	22641331	0.792326	0	1	0.8931251	41.72671
324677	cg17812431	20662375	20662375	0.9022205	0	1	0.6973413	-120.1381
324679	cg1781251:	38646365	38646365	0.1052588	0	1	0.2347045	48.5097
324741	cg1781617:	21802414	21802414	0.313087	0	1	0.4894513	49.91438
324758	cg17817521	74747366	74747366	0.7854325	0	1	0.6276235	-52.70511
324804	cg1782006(	39603403	39603403	0.9172739	0	1	0.7221239	-120.586
324851	cg1782294:	64808470	10634487	0.1896414	0	1	0.0542881	-66.64465
324931	cg1782689:	42747303	42747303	0.3401022	0	1	0.4526045	22.69356
325005	cg1783117(	69692402	69692402	0.3131219	0	1	0.187826	-34.76292
325040	cg1783303:	49649482	49649482	0.4000603	0	1	0.2541733	-38.64313
325125	cg1783716:	69810325	71724461	0.033033	0	1	0.3170331	374.3439
325144	cg1783816:	10653390	10653390	0.5073164	0	1	0.3349075	-47.21096
325147	cg1783836(	66669365	66669365	0.5236962	0	1	0.6976428	51.7824
325184	cg1783975(	70682329	70682329	0.649311	0	1	0.4981505	-38.33473
325187	cg1784004:	58761423	58761423	0.1363783	0	1	0.2572238	38.62074
325195	cg1784025(	18672453	18672453	0.1448488	0	1	0.3784071	107.5617

325214	cg1784099	52603312	52603312	0.303921	0	1	0.4077063	20.67675
325261	cg1784341	42727506	33790411	0.9228884	0	1	0.7294632	-122.611
325291	cg1784568	70783337	70783337	0.3989882	0	1	0.2979477	-19.9684
325324	cg1784840	51725388	51725388	0.3740588	0	1	0.2667431	-22.9867
325377	cg1785127	36699438	36699438	0.6836915	0	1	0.4268135	-103.8609
325398	cg1785232	64767390	64767390	0.2582933	0	1	0.3610885	21.79103
325459	cg1785522	58603355	58603355	0.6387314	0	1	0.7923458	51.46498
325577	cg1786255	38697493	38697493	0.4930505	0	1	0.6110404	24.44695
325582	cg1786290	23774440	23774440	0.7566592	0	1	0.8600459	36.24775
325665	cg1786777	40605438	40605438	0.1656484	0	1	0.296393	39.59282
325734	cg1787162	35794392	35794392	0.3272966	0	1	0.135052	-80.28081
325777	cg1787410	52697443	52697443	0.2698337	0	1	0.3971476	30.10976
325812	cg1787629	40769337	40769337	0.2572168	0	1	0.459458	67.59248
325857	cg1787850	26600357	26600357	0.4745415	0	1	0.6128138	31.9327
325895	cg1788070	48690350	48690350	0.1781317	0	1	0.0682336	-45.73792
325935	cg1788306	67673459	67673459	0.6438505	0	1	0.7494093	25.3336
325955	cg1788476	31729411	31729411	0.6351568	0	1	0.7413357	25.07311
325975	cg1788579	61754331	61754331	0.5995224	0	1	0.7027656	22.14926
326112	cg1789436	65804408	65804408	0.5357352	0	1	0.7126201	54.52546
326114	cg1789457	64615429	64615429	0.3258317	0	1	0.5005	48.60221
326212	cg1790146	13609457	13609457		0	1		-4.34E-09
326237	cg1790350	46677482	46677482	0.4297263	0	1	0.573643	33.61986
326284	cg1790685	16669375	68732485	0.6368647	0	1	0.4785149	-41.07749
326332	cg1791102	73700473	73700473	0.5104893	0	1	0.3782211	-29.08702
326356	cg1791340	68658442	51785358	0.7570223	0	1	0.5597861	-71.28596
326377	cg1791567	23747310	16603441	0.6054266	0	1	0.4902424	-23.40548
326498	cg1792299	31729313	31729313	0.5947859	0	1	0.7419345	42.29226
326584	cg1792904	61732494	61785438	0.7620562	0	1	0.6482746	-29.41111
326599	cg1792963	42778478	24628430	0.8531438	0	1	0.4687246	-288.265
326641	cg1793209	54680411	71634319	0.6626226	0	1	0.5552987	-22.10926
326682	cg1793427	43775397	43775397	0.5506068	0	1	0.6932991	36.75787
326845	cg1794332	10725345	74710311	0.1885139	0	1	0.4167035	93.6544
326978	cg1795197	54721318	54721318	0.8117399	0	1	0.6897764	-38.11708
327066	cg1795851	26612425	26612425	0.4112897	0	1	0.5169094	19.81117
327134	cg1796263	57691346	48753432	0.5051364	0	1	0.3861735	-24.25251
327205	cg1796767	42652479	47683506	0.8227691	0	1	0.6176594	-89.68224
327290	cg1797201	55801377	55801377	0.2689267	0	1	0.374784	22.41515
327368	cg1797647	18628445	18628445	0.36827	0	1	0.5133007	34.21976
327603	cg1799191	48806380	48806380	0.745115	0	1	0.8558784	39.36042
327732	cg1800172	11803395	11803395	0.9324747	0	1	0.7035893	-161.0954
327959	cg1801385	14741457	14741457	0.6558623	0	1	0.8882308	136.2014
327982	cg1801503	55684336	53622345	0.5220813	0	1	0.3878506	-29.7513
327993	cg1801562	51612387	51612387	0.6552783	0	1	0.9136589	374.3439

328102	cg18021992	22634366	22634366	0.6073286	0	1	0.919933	374.3439
328180	cg18025438	39724429	53600384	0.6715711	0	1	0.7841678	30.8
328224	cg18027903	64659303	64659303	0.3681504	0	1	0.4956544	27.43761
328239	cg18029321	14773369	15627427	0.7255613	0	1	0.4581514	-117.0947
328579	cg18049365	20749317	20749317	0.5424312	0	1	0.1729926	-239.2113
328720	cg18058072	19698435	19698435	0.3559769	0	1	0.1258258	-110.4913
328852	cg18065195	56632432	56632432	0.7385838	0	1	0.8406996	32.58183
328853	cg18065318	57722402	28716370	0.3870311	0	1	0.2284116	-46.49788
328870	cg18066181	15764473	15764473	0.1366897	0	1	0.3119733	68.90778
328873	cg18066206	40766387	40766387	0.3223476	0	1	0.4537924	29.79138
328989	cg18073702	10662424	10662424	0.669277	0	1	0.7927245	36.47935
329023	cg18075691	44667386	40687509	0.2918113	0	1	0.4021236	23.14087
329052	cg18077580	65638428	55721395	0.4539678	0	1	0.3368681	-24.29167
329183	cg18085660	18797349	18797349	0.2738461	0	1	0.3758015	20.96334
329217	cg18087477	46682331	46682331	0.6687909	0	1	0.5329335	-32.72168
329223	cg18087694	61657497	61657497	0.4470513	0	1	0.5529236	19.91737
329248	cg18088906	67808397	67808397	0.6181261	0	1	0.7506824	36.41365
329262	cg18089380	35673462	35673462	0.771614	0	1	0.8862407	48.25889
329264	cg18089426	46749487	21664493	0.4136949	0	1	0.5797859	43.44143
329333	cg18093448	50789402	50789402	0.7058408	0	1	0.9039107	115.3988
329376	cg18096251	15772317	31766326	0.7620376	0	1	0.921666	94.05123
329414	cg18098433	46705300	46705300	0.6725715	0	1	0.773006	24.98884
329417	cg18098534	45759375	45759375	0.9307752	0	1	0.7033051	-158.4831
329470	cg18101138	56696302	33732501	0.5228491	0	1	0.3377454	-53.61557
329471	cg18101140	73696454	73696454	0.4866157	0	1	0.6423947	40.11497
329496	cg18102738	16785412	16785412	0.5329032	0	1	0.6338857	19.4034
329516	cg18104070	74644413	74644413	0.4603672	0	1	0.6187979	40.57762
329641	cg18109485	23629378	23629378	0.5455559	0	1	0.6547687	22.50678
329676	cg18111114	51629438	51629438	0.0620669	0	1	0.2592066	109.4242
329719	cg18113731	67743477	67743477	0.6131727	0	1	0.7134579	21.60113
329795	cg18116815	20656325	20656325	0.5948337	0	1	0.7261586	33.99726
329863	cg18120259	60660316	60660316	0.3415511	0	1	0.4837571	33.534
330015	cg18127410	18628506	30793406	0.2536401	0	1	0.1130748	-53.20357
330044	cg18128914	41774408	31634314	0.3630823	0	1	0.2143863	-42.97245
330054	cg18129296	52643353	52643353	0.5763842	0	1	0.744308	52.89544
330060	cg18129748	64665323	27793432	0.2068249	0	1	0.0968053	-39.53337
330068	cg18129996	27733307	27733307	0.5704463	0	1	0.6807162	23.68672
330108	cg18132363	59669394	59669394	0.1819256	0	1	0.2879196	27.21183
330192	cg18137779	16712471	16712471	0.5146382	0	1	0.814813	374.3439
330208	cg18138788	67667410	67667410	0.5968889	0	1	0.4934854	-19.53482
330271	cg18142730	17650424	17650424	0.794555	0	1	0.8955789	42.48196
330349	cg18146506	31784507	35749469	0.2029474	0	1	0.0680825	-61.49603
330734	cg18167946	49786505	49786505	0.2532676	0	1	0.3599224	23.29796

330748	cg18168924	65652445	65652445	0.4153726	0	1	0.2456307	-50.59873
330777	cg18170545	51703355	51703355	0.6886752	0	1	0.8262272	48.32701
330805	cg18172516	47666425	47666425	0.5411718	0	1	0.7493226	77.05352
330919	cg18178836	62753314	62753314	0.4095902	0	1	0.6350469	78.23401
330991	cg18182148	65806464	14759469	0.2349426	0	1	0.129494	-32.22064
331035	cg18184218	39675302	60713458	0.3297843	0	1	0.4691085	32.65948
331119	cg18189112	24641487	24641487	0.7844589	0	1	0.6536827	-38.94998
331169	cg18191743	39654422	39654422	0.1228601	0	1	0.299924	73.2854
331280	cg18197594	30777480	22606501	0.2519412	0	1	0.1337343	-37.70445
331424	cg18206858	71800437	71800437	0.9270024	0	1	0.8238518	-53.98529
331463	cg18209359	39698449	30614324	0.5474907	0	1	0.6502117	20.3019
331554	cg18217622	63639332	29753412	0.3872938	0	1	0.5075049	24.67137
331622	cg18221341	44712443	44712443	0.3612622	0	1	0.4615443	18.5715
331765	cg18229196	32720509	32720509	0.7262168	0	1	0.8535526	47.95888
331816	cg18232235	37622316	19617432	0.6227914	0	1	0.2371148	-249.4768
331893	cg18235746	74796359	74796359	0.3090369	0	1	0.4416434	30.62933
331908	cg18236584	56748399	56748399	0.8705037	0	1	0.7427638	-52.10413
331928	cg18237398	54606493	54606493		0	1		-4.34E-09
331940	cg18237739	59617323	59617323	0.2576567	0	1	0.3584348	21.14977
331970	cg18239511	31669324	31669324	0.3367223	0	1	0.0750041	-158.9736
331985	cg18240390	34758345	20710362	0.3867281	0	1	0.2778055	-23.13077
332008	cg18241555	36766333	36766333	0.2939816	0	1	0.4014543	22.13339
332046	cg18243846	59632301	59632301	0.1823622	0	1	0.2973554	30.84021
332249	cg18256949	70761332	18774336	0.2446699	0	1	0.3478301	22.4698
332349	cg18263166	32613407	32613407	0.4462654	0	1	0.5551614	20.86741
332368	cg18264092	51614455	51614455	0.3781452	0	1	0.4914354	22.42989
332724	cg18285337	62704331	62704331	0.4971197	0	1	0.674755	52.15304
332730	cg18285636	42663455	42663455	0.762631	0	1	0.8721916	41.93575
332773	cg18288532	12763351	24637492	0.4644081	0	1	0.31727	-36.22987
332937	cg18302460	63637364	63637364	0.4616836	0	1	0.5823407	24.95046
333075	cg18310639	22750426	22750426	0.4664827	0	1	0.5717211	19.83591
333109	cg18313094	20750478	20750478	0.4768206	0	1	0.5978334	25.27134
333121	cg18313416	56641438	56641438	0.3485189	0	1	0.4659309	24.18442
333227	cg18319366	59609370	59609370	0.2720201	0	1	0.3904886	26.69684
333275	cg18321929	38679509	38679509	0.4978177	0	1	0.6277736	29.08359
333278	cg18322025	73790449	73790449	0.5840138	0	1	0.4736582	-21.54704
333285	cg18322589	38604422	32672417	0.6616206	0	1	0.521342	-34.21996
333361	cg18327128	43622441	72679507	0.617547	0	1	0.4597647	-40.25459
333462	cg18332814	24660360	24660360	0.2006587	0	1	0.3648207	52.13344
333463	cg18332838	15750345	15750345	0.5299505	0	1	0.6790578	38.77109
333466	cg18333092	60758320	60758320	0.5469537	0	1	0.3751456	-46.20603
333678	cg18346634	25688486	25688486	0.2409003	0	1	0.4640827	82.50576
333872	cg18359218	63713492	63713492	0.6273652	0	1	0.9204257	374.3439

333900	cg18361425	38652398	38652398	0.5802251	0	1	0.7376639	46.86113
333934	cg18363918	44720423	72657405	0.5809868	0	1	0.4605991	-24.84319
334002	cg18369034	41755416	41755416	0.5188235	0	1	0.6829551	45.90808
334070	cg18373318	57639333	70647447	0.1586511	0	1	0.0559425	-44.63885
334213	cg18383984	59712332	62658412	0.5297996	0	1	0.4217538	-20.54801
334216	cg18384097	54661343	54661343		0	1	0.9023973	4.34E-09
334338	cg18391209	15607400	15607400	0.6794859	0	1	0.4953755	-55.85996
334419	cg18395354	50618432	50618432	0.349294	0	1	0.5077437	40.30328
334465	cg18396987	42719428	42719428	0.4713216	0	1	0.2630824	-70.73015
334477	cg18397450	71715420	39692388	0.1085773	0	1	0.2436864	51.0266
334522	cg18400281	44685449	44685449	0.5803683	0	1	0.7069605	30.88643
334575	cg18403673	35631307	35631307	0.6914436	0	1	0.3046201	-248.979
334655	cg18408273	67686379	67686379	0.2523736	0	1	0.3693584	27.02542
334732	cg18413218	62675325	62675325	0.5748795	0	1	0.4116848	-42.04519
334790	cg18417245	30753390	30753390	0.5773631	0	1	0.3736985	-63.53197
334797	cg18417622	22740372	22740372	0.2211267	0	1	0.1055472	-40.87598
334809	cg18418242	24632416	24632416	0.6130697	0	1	0.7323276	29.50067
334913	cg18424635	21718331	21718331	0.2222003	0	1	0.4209923	69.12391
335041	cg18431856	55604411	55604411	0.4874539	0	1	0.3493559	-31.73549
335090	cg18434356	72748422	72748422	0.311249	0	1	0.4275726	24.62288
335125	cg18436272	17740354	17740354	0.6840287	0	1	0.7866417	26.90134
335149	cg18437193	12788411	58659490	0.9166568	0	1	0.7912771	-65.6549
335154	cg18437551	10800325	10800325	0.7763751	0	1	0.9112698	70.23811
335259	cg18443741	22728380	22728380	0.4879939	0	1	0.2567246	-86.36403
335278	cg18444757	20699419	20699419	0.6357454	0	1	0.4463561	-56.4793
335300	cg18446441	42799423	42799423	0.2508439	0	1	0.3739151	29.40124
335513	cg18456803	47788300	47788300	0.3641312	0	1	0.5019262	31.37304
335545	cg18458353	22756322	22756322	0.3288077	0	1	0.4294206	19.15808
335851	cg18476530	74600356	44758432	0.1177651	0	1	0.2664365	56.91661
335853	cg18476566	30646465	30646465	0.2150702	0	1	0.3322968	29.24598
335933	cg18480654	70607379	70607379	0.6090241	0	1	0.7203102	25.77916
336036	cg18486231	52645457	52645457	0.6257808	0	1	0.7406336	28.31223
336122	cg18492943	17605353	17605353	0.322009	0	1	0.4451023	26.71923
336208	cg18497238	43603334	43603334	0.5575003	0	1	0.7156238	45.28109
336225	cg18498115	74803328	74803328	0.2931491	0	1	0.4099472	25.32565
336272	cg18500448	18746310	21703329	0.4411721	0	1	0.3325738	-21.56915
336305	cg18502238	14718442	14718442	0.3043486	0	1	0.4365979	30.64579
336340	cg18503829	35600395	35600395	0.7284465	0	1	0.8675171	58.24728
336353	cg18504989	35754305	35754305	0.2273823	0	1	0.3550819	32.64116
336411	cg18509524	36778350	36778350	0.5779159	0	1	0.4652705	-22.22292
336454	cg18512930	43642477	43642477	0.1674744	0	1	0.2716536	27.67437
336476	cg18514705	18775345	18775345	0.7691008	0	1	0.6474537	-33.28407
336663	cg18526142	31777387	31777387	0.2186918	0	1	0.3282776	26.08366

336740	cg1853074	27674480	27674480	0.1793423	0	1	0.3032573	34.96523
336877	cg1853866	57613469	57613469	0.2507402	0	1	0.4501088	66.46104
336910	cg1854067	47703489	34638494	0.4297531	0	1	0.5408131	21.51144
336977	cg1854488	56619423	56619423	0.5226663	0	1	0.6744429	39.71522
337048	cg1855021	47738353	47738353	0.3815666	0	1	0.5182855	30.75719
337182	cg1855896	24730370	62631352		0	1	0.9666369	4.34E-09
337183	cg1855896	32634441	32634441	0.825627	0	1	0.6433914	-74.85944
337202	cg1855990	33808339	33808339	0.6760721	0	1	0.3609682	-156.516
337230	cg1856167	23642315	25610431	0.4309056	0	1	0.25462	-53.26261
337322	cg1856655	69619467	69619467	0.149552	0	1	0.2674836	35.50874
337439	cg1857414	41738380	41738380	0.7078447	0	1	0.8261194	38.32595
337474	cg1857604	68603321	68603321	0.4571579	0	1	0.0373717	-371.3336
337519	cg1857897	44742476	44742476	0.5926932	0	1	0.7132407	28.8744
337651	cg1858688	59686310	25634375	0.2080168	0	1	0.3292081	31.3401
337664	cg1858747	66713393	16746438	0.5544955	0	1	0.4517375	-18.97999
337781	cg1859405	42643354	42643354	0.5350921	0	1	0.6470637	23.21458
337792	cg1859470	16771328	16771328	0.6014525	0	1	0.7027463	21.49806
337802	cg1859513	50743404	50743404	0.4119592	0	1	0.5141801	18.78131
337811	cg1859534	49600459	49600459	0.6660563	0	1	0.7780934	30.01722
337832	cg1859662	67608471	56619403	0.8078573	0	1	0.9683897	129.2439
337838	cg1859693	45791316	45791316	0.7087877	0	1	0.8272844	38.60769
337879	cg1859920	22725391	22725391	0.1128467	0	1	0.2500431	51.32253
338013	cg1860912	20639370	20639370	0.3443438	0	1	0.4562727	22.43129
338058	cg1861216	19683443	19683443	0.3164183	0	1	0.2072769	-26.62469
338088	cg1861473	72666418	72666418	0.5300609	0	1	0.4051636	-26.22366
338139	cg1861881	72789442	72789442	0.4979449	0	1	0.641177	34.68813
338172	cg1862167	54794373	54794373	0.1111756	0	1	0.3984346	374.3439
338175	cg1862185	49649455	49649455	0.6228728	0	1	0.521647	-19.26952
338219	cg1862530	38786353	38786353	0.5792021	0	1	0.4559498	-25.82249
338267	cg1862885	15752508	15752508	0.703895	0	1	0.8459558	54.78287
338290	cg1863064	30663428	71604489	0.7161348	0	1	0.6159772	-21.67374
338352	cg1863421	65675489	65675489	0.3896903	0	1	0.2860489	-21.12308
338535	cg1864470	24744359	42742445	0.5031127	0	1	0.3566819	-34.96992
338634	cg1865021	26727330	26727330	0.5911745	0	1	0.6923388	21.0648
338638	cg1865036	44770510	44770510	0.4788339	0	1	0.6502652	47.76966
338860	cg1866765	18600413	47657432	0.666698	0	1	0.5275189	-33.9793
338868	cg1866851	73803381	73803381	0.839918	0	1	0.7178983	-42.44349
338925	cg1867203	61724489	61724489	0.4350933	0	1	0.5587056	25.79738
338999	cg1867696	62800469	62800469	0.5141556	0	1	0.4140259	-18.15799
339023	cg1867864	30651474	30651474	0.6107293	0	1	0.4609037	-36.62037
339037	cg1867950	24634453	45682460	0.5097145	0	1	0.317655	-58.03375
339050	cg1868018	55777484	55777484	0.5718491	0	1	0.6848956	24.80572
339109	cg1868475	48604510	48604510	0.8967968	0	1		-4.34E-09

339311	cg1869879(	71781488	71781488	0.6924856	0	1	0.9506763	374.3439
339327	cg1869974(	17610311	50803478	0.4560803	0	1	0.1325455	-196.1432
339458	cg1870719(	56623390	56623390	0.0690154	0	1	0.1694832	40.00816
339504	cg1870988(	13716416	35614448	0.7732904	0	1	0.6627821	-28.98092
339506	cg1870990(	36760466	36760466	0.8160102	0	1	0.9170898	48.89306
339507	cg1871005(	57759456	30709321	0.4906814	0	1	0.3254094	-44.05994
339508	cg1871016(	52787416	52787416	0.5618393	0	1	0.6757138	24.75216
339527	cg1871153(	17630435	17630435	0.312102	0	1	0.4880378	49.7375
339592	cg1871519(	55607332	55607332	0.7130747	0	1	0.8998646	104.1913
339664	cg1871956(	62610332	42774428	0.8310128	0	1	0.716185	-37.34642
339690	cg1872080(	36780455	36780455	0.675342	0	1	0.7821392	28.21136
339726	cg1872327(	24728328	69768312	0.8015083	0	1	0.7006012	-27.52666
339836	cg1873086(	44794322	44794322	0.3297567	0	1	0.4452925	23.90827
339929	cg1873683(	62763467	62763467	0.7520989	0	1	0.859219	38.04821
340103	cg1874852(	64734310	64734310	0.3576521	0	1	0.5143179	39.35141
340218	cg1875553(	65673494	26705407	0.5984052	0	1	0.4351116	-42.36542
340249	cg1875677(	58784415	58784415	0.5039467	0	1	0.604934	18.91495
340251	cg1875693(	18673412	18673412	0.5907264	0	1	0.7360843	40.94159
340258	cg1875717(	21604380	50633484	0.4023394	0	1	0.2966222	-21.48689
340266	cg1875781(	46676349	46676349	0.7775643	0	1	0.5578411	-89.60294
340357	cg1876195(	59759492	59759492	0.4988141	0	1	0.6791228	53.81607
340358	cg1876197(	13705477	13705477	0.2161784	0	1	0.3663139	43.49307
340383	cg1876280(	18783464	61769414	0.6268336	0	1	0.5148385	-22.74522
340406	cg1876416(	54684365	54684365	0.5476578	0	1	0.6722691	28.50394
340418	cg1876477(	23773404	23773404	0.1876915	0	1	0.2931187	26.5519
340431	cg1876556(	30701412	69737303	0.2498166	0	1	0.1088734	-54.3348
340535	cg1877094(	34736429	34736429	0.5636076	0	1	0.3805468	-51.91233
340549	cg1877155(	30745384	30745384	0.6813283	0	1	0.5427824	-34.38305
340550	cg1877157(	16795398	16795398	0.6085104	0	1	0.5018555	-20.69939
340560	cg1877193(	45603442	45603442	0.8158631	0	1	0.619577	-81.82607
340593	cg1877393(	66607479	66607479	0.5077007	0	1	0.6294277	26.12668
340597	cg1877417(	15750484	15750484	0.6022393	0	1	0.7328125	34.14894
340633	cg1877646(	59716352	59716352	0.3114254	0	1	0.5380567	79.39282
340722	cg1878260(	33640410	69771399	0.1056854	0	1	0.2371849	49.58233
340778	cg1878622(	15670369	15670369	0.2945916	0	1	0.4066034	23.62888
340856	cg1879034(	21802416	21802416	0.4242682	0	1	0.5426084	23.92127
340897	cg1879253(	59607453	37656490	0.6082008	0	1	0.0799996	-371.3336
340987	cg1879765(	65706357	65706357	0.5074733	0	1	0.3948926	-22.07363
341051	cg1880150(	29715318	29715318	0.9450908	0	1	0.7543037	-136.1507
341126	cg1880516(	51795502	49795427	0.3752558	0	1	0.6670289	132.6617
341153	cg1880671(	34733372	34733372	0.2818784	0	1	0.4097423	29.79938
341174	cg1880826(	31775510	31775510	0.59395	0	1	0.4788505	-23.20922
341341	cg1881612(	40800307	60804314	0.9207602	0	1	0.5098551	-371.3336

341344	cg1881639[illegible]	63781395	63781395	0.1629016	0	1	0.2791756	33.25225
341438	cg1882295[illegible]	39802455	39802455	0.9368634	0	1	0.8349522	-57.28105
341517	cg1882826[illegible]	45742508	45742508	0.2439752	0	1	0.7971303	374.3439
341521	cg1882836[illegible]	68786375	68786375	0.3416718	0	1	0.2171994	-32.01891
341585	cg1883224[illegible]	32721320	32721320	0.3904626	0	1	0.5576096	43.87134
341589	cg1883261[illegible]	64706315	64706315	0.6388124	0	1	0.459949	-51.03569
341734	cg1884235[illegible]	62643339	62643339	0.1644395	0	1	0.2823857	33.83308
341746	cg1884325[illegible]	23606377	23606377	0.3378499	0	1	0.1635622	-63.06949
341785	cg1884559[illegible]	64729449	64729449	0.3377612	0	1	0.6101079	113.6044
341792	cg1884595[illegible]	23655331	39745490	0.3955512	0	1	0.1239511	-146.4959
341814	cg1884759[illegible]	37742467	37742467	0.3055763	0	1	0.698347	374.3439
342047	cg1886176[illegible]	66694457	66694457	0.0460866	0	1	0.24395	118.4078
342058	cg1886221[illegible]	30730442	30730442	0.2286949	0	1	0.3354132	24.47672
342092	cg1886412[illegible]	54703465	54703465	0.6109429	0	1	0.7324404	30.39582
342141	cg1886621[illegible]	71749347	71749347	0.2739121	0	1	0.404691	31.27517
342175	cg1886791[illegible]	68708477	65731382	0.8815993	0	1	0.7386436	-64.87472
342223	cg1887201[illegible]	14605399	14605399	0.1324742	0	1	0.2365406	31.22047
342243	cg1887350[illegible]	40700434	40700434	0.7948557	0	1	0.5462527	-115.2495
342255	cg1887396[illegible]	54692329	10612501	0.5603995	0	1	0.6972625	34.51647
342370	cg1887982[illegible]	18622333	18622333	0.2638268	0	1	0.3738898	24.04198
342489	cg1888634[illegible]	15615488	15615488	0.4835311	0	1	0.6294149	35.42814
342545	cg1888978[illegible]	41772482	41772482	0.1315394	0	1	0.2345558	30.85814
342675	cg1889863[illegible]	73652446	73652446	0.7906515	0	1	0.6760207	-32.30295
342751	cg1890455[illegible]	57804445	57804445	0.5743998	0	1	0.6791028	21.76467
342859	cg1891220[illegible]	12717429	33798456	0.2608026	0	1	0.1113885	-58.78545
342872	cg1891285[illegible]	73627398	52719449	0.002572	0	1	0.1361168	92.37933
342886	cg1891384[illegible]	27768316	27768316	0.8744255	0	1	0.7223496	-68.631
342899	cg1891475[illegible]	22696464	22696464	0.5547554	0	1	0.6843486	30.92433
343015	cg1892198[illegible]	26643444	18749330	0.1646645	0	1	0.0569132	-47.50388
343128	cg1893091[illegible]	67639417	67639417	0.0848752	0	1	0.2309135	63.59873
343149	cg1893200[illegible]	13771476	13771476	0.6524233	0	1	0.7766727	35.13924
343223	cg1893739[illegible]	36698324	36698324	0.149874	0	1	0.2535601	29.12412
343239	cg1893890[illegible]	28607449	28607449	0.2283611	0	1	0.0689299	-77.97769
343252	cg1894004[illegible]	29738408	18643318	0.4791998	0	1	0.155589	-188.1278
343326	cg1894464[illegible]	17610306	24766383	0.8880503	0	1	0.760206	-57.04758
343411	cg1894972[illegible]	10737407	10737407	0.6438253	0	1	0.30303	-184.8848
343620	cg1896408[illegible]	60705361	60705361	0.530683	0	1	0.3632746	-44.20829
343653	cg1896615[illegible]	60689504	60689504	0.4500732	0	1	0.5670211	23.55267
343753	cg1897386[illegible]	53621510	53621510	0.3522439	0	1	0.4550041	19.42788
343762	cg1897492[illegible]	25750367	25750367	0.370111	0	1	0.5354228	43.1303
343861	cg1898292[illegible]	73759472	73759472	0.2711289	0	1	0.3815999	23.9
343891	cg1898498[illegible]	49726348	49726348	0.1999834	0	1	0.3118227	28.16782
343950	cg1898905[illegible]	47798444	58669418	0.5833601	0	1	0.4568913	-27.01257

344075	cg18997918	73799498	73799498	0.7231702	0	1	0.6019103	-29.72041
344126	cg19001290	33738369	33738369	0.4687113	0	1	0.5779831	21.14305
344211	cg19005485	19753347	19753347	0.8921852	0	1	0.7586502	-62.09918
344414	cg19017553	12791403	12791403	0.3547769	0	1	0.4794676	26.61493
344513	cg19024575	66683313	66683313	0.6054035	0	1	0.716043	25.32605
344555	cg19026811	72688464	72688464	0.6165887	0	1	0.7851219	58.58451
344583	cg19028462	23709435	23709435	0.557274	0	1	0.6615548	21.09225
344766	cg19039925	18772410	18772410	0.9461599	0	1	0.8459435	-60.47986
344820	cg19043679	28634462	28634462	0.7961045	0	1	0.8999589	45.38864
344823	cg19043800	37690425	37690425	0.3567912	0	1	0.4817185	26.66774
344862	cg19045970	38642499	38642499	0.0471248	0	1	0.2019099	83.71507
344878	cg19046933	62747452	56669392	0.6215106	0	1	0.7252344	23.32111
344891	cg19047660	39632473	19788466	0.3610322	0	1	0.2594075	-21.36209
344941	cg19050555	53626330	53626330	0.5259252	0	1	0.6401757	23.80341
344996	cg19054158	50692499	38620328	0.542834	0	1	0.2977385	-93.08405
345088	cg19061696	16654445	16654445	0.4103339	0	1	0.5281713	23.75519
345120	cg19064302	16806431	16806431	0.5307276	0	1	0.6402273	22.22686
345165	cg19070088	51641443	51641443	0.1790844	0	1	0.3001517	33.73648
345253	cg19077494	59686444	59686444	0.4310313	0	1	0.5350038	19.29219
345301	cg19081571	39616332	64729355	0.4978321	0	1	0.6836219	57.04412
345343	cg19084031	22697405	22697405	0.3564303	0	1	0.4815475	26.74129
345358	cg19084628	15678303	21616417	0.8513207	0	1	0.7506846	-33.4257
345428	cg19089141	13650302	13650302	0.7258458	0	1	0.3828045	-193.2507
345636	cg19101566	41708499	41708499	0.7781229	0	1	0.8880597	45.89928
345662	cg19103175	69780387	55632318	0.6867411	0	1	0.8087891	37.77049
345719	cg19106932	70727507	13769303	0.2547971	0	1	0.0886437	-75.76921
345796	cg19111378	14791415	14791415	0.6177515	0	1	0.7265921	25.21908
345811	cg19112359	43715460	43715460	0.5891195	0	1	0.7551997	53.15914
345819	cg19112977	39655422	58802319	0.2873517	0	1	0.1720052	-31.93472
345830	cg19113686	62787444	52670483	0.7983441	0	1	0.6846814	-32.6776
345870	cg19116197	58774450	58774450	0.4579478	0	1	0.3509105	-20.76348
345948	cg19121987	17612323	29675408		0	1	0.001653	4.34E-09
346140	cg19137348	53769423	68711421	0.1099217	0	1	0.2368978	46.19777
346183	cg19140335	53809370	22730398	0.9241293	0	1	0.7467934	-109.8516
346186	cg19140429	20787496	63779377	0.5351521	0	1	0.4296679	-19.75355
346243	cg19145082	18765434	18765434	0.4379143	0	1	0.5380897	18.16394
346249	cg19145320	42631416	42631416	0.5327259	0	1	0.6978726	47.37252
346351	cg19152818	72749376	72749376	0.4879148	0	1	0.6024632	23.14774
346356	cg19153228	19720454	19720454	0.334826	0	1	0.4440714	21.73371
346387	cg19155932	43749462	39638415	0.7636027	0	1	0.6427932	-32.44465
346388	cg19156046	24621302	24621302	0.9019821	0	1	0.694625	-122.0847
346472	cg19162300	19617336	51760401	0.9306303	0	1	0.8027493	-74.02177
346490	cg19163396	14795356	14795356	0.7415662	0	1	0.9053119	88.9351

346495	cg1916393	32709467	37750441	0.5244855	0	1	0.6421119	25.00901
346664	cg1917850	70712376	70712376	0.6852968	0	1	0.7939534	30.05739
346685	cg1917991	74661377	74661377	0.8170305	0	1	0.9429965	79.35651
346710	cg1918152	25651436	10665457	0.7199677	0	1	0.5815146	-36.60868
346728	cg1918291	41702481	41702481	0.8010167	0	1	0.9091657	50.94361
346731	cg1918316	34778379	27645367	0.4098645	0	1	0.2864651	-27.93933
346737	cg1918339	69684305	69684305	0.5027539	0	1	0.283162	-76.35562
346808	cg1918820	64642350	64642350	0.7141111	0	1	0.829941	37.68201
346849	cg1919127	48655375	24616329	0.8679047	0	1	0.7385652	-52.41254
346874	cg1919262	32620442	32620442	0.3871397	0	1	0.5463077	40.15981
346906	cg1919464	63739487	63739487	0.4729598	0	1	0.5819008	21.0764
346925	cg1919640	28656499	64758359	0.5278646	0	1	0.3880638	-31.91908
346943	cg1919774	60653364	12766507	0.3287248	0	1	0.4346958	20.8104
346947	cg1919788	49737495	42794429	0.7288728	0	1	0.5496053	-57.3499
346985	cg1920058	18783416	21641317	0.5199971	0	1	0.3943134	-26.54863
346989	cg1920095	57715418	57715418	0.3219753	0	1	0.4376214	24.12082
346993	cg1920114	12668447	12668447	0.2430307	0	1	0.3481534	23.2162
347022	cg1920357	41729330	73749471	0.2963631	0	1	0.4026336	21.67315
347035	cg1920492	52618403	52618403	0.675272	0	1	0.8233931	53.65117
347212	cg1921808	46650473	58732404	0.6849413	0	1	0.7946587	30.57907
347269	cg1922239	53804325	53804325	0.8176125	0	1	0.9435779	79.70516
347327	cg1922595	62655484	62655484	0.7362891	0	1	0.8473971	38.05762
347370	cg1922921	41615359	41615359	0.2539107	0	1	0.3621078	23.80403
347408	cg1923238	57630337	48795476	0.4668184	0	1	0.3647776	-19.05366
347467	cg1923632	16618399	16618399	0.7605293	0	1	0.6529618	-26.83817
347474	cg1923667	39622372	39622372	0.8953814	0	1	0.6918275	-115.4642
347518	cg1923904	36808446	36808446	0.6975978	0	1	0.5938287	-22.12082
347587	cg1924261	72628304	72628304	0.6036165	0	1	0.9323199	374.3439
347613	cg1924403	27615359	27615359	0.1875402	0	1	0.3938206	78.79754
347653	cg1924594	59606503	59606503		0	1	0.091861	4.34E-09
347723	cg1924981	17795473	24793332	0.6501189	0	1	0.902716	374.3439
347839	cg1925582	69638391	46603391	0.1936795	0	1	0.0387996	-87.83546
347914	cg1925980	70606369	70606369	0.2379941	0	1	0.1298825	-33.40734
347974	cg1926360	43620445	43620445	0.4919423	0	1	0.3770297	-22.97308
348216	cg1927634	52623476	66609339	0.383164	0	1	0.264066	-27.26591
348220	cg1927673	74733353	20648379	0.3888207	0	1	0.2678044	-27.81206
348332	cg1928552	71768474	71768474	0.5275542	0	1	0.7486898	85.57989
348415	cg1929169	13748308	13748308	0.4340755	0	1	0.6840219	98.53712
348501	cg1930019	10720447	10720447	0.5302716	0	1	0.3668022	-42.30801
348504	cg1930040	38724489	38724489	0.5240074	0	1	0.9543688	374.3439
348509	cg1930054	18720498	18720498	0.5073093	0	1	0.6528649	36.06946
348535	cg1930189	29810355	29810355	0.4966694	0	1		-4.34E-09
348565	cg1930352	69647415	69647415	0.6976451	0	1	0.8268489	43.99157

348620	cg19306496	16807337	43714304	0.0138607	0	1	0.1269365	68.39566
348659	cg19308989	11636377	11636377	0.5244894	0	1	0.6603292	32.37516
348697	cg19311375	60640379	60640379	0.4570967	0	1	0.6190054	42.1665
348871	cg19323289	63752427	63752427	0.2672704	0	1	0.3994749	32.15527
348911	cg19325791	16661434	16661434	0.2305625	0	1	0.3476009	28.20349
348983	cg19331988	72621403	72621403	0.5616847	0	1	0.6957268	33.26957
349075	cg19340296	51696419	51696419	0.2988004	0	1	0.5532883	100.1433
349141	cg19344878	68703407	68703407	0.8292461	0	1	0.934038	58.16003
349254	cg19353052	23654413	23654413	0.8005702	0	1	0.9187729	61.39021
349382	cg19360212	48673335	48673335	0.838863	0	1	0.9555675	79.64574
349417	cg19362478	31614361	31614361	0.4262342	0	1	0.5361714	21.14724
349422	cg19362774	36649386	13798457	0.9296191	0	1	0.6476705	-211.6877
349495	cg19367176	35634328	28751497	0.9162455	0	1	0.7549093	-92.49089
349507	cg19367859	54795417	54795417	0.6909698	0	1	0.8257307	46.73871
349585	cg19372507	60705397	60705397	0.5682935	0	1	0.7477569	59.57161
349598	cg19373347	37624483	37624483	0.3148361	0	1	0.1878864	-35.486
349636	cg19375418	29773383	29773383	0.577149	0	1	0.7508991	56.78347
349678	cg19377607	74654306	74654306	0.4386761	0	1	0.5393689	18.31838
349715	cg19380156	62608399	62608399	0.611867	0	1	0.7247692	26.61591
349763	cg19383360	62758370	61672505	0.5624186	0	1	0.4360801	-26.78939
349845	cg19389372	60619466	59765345	0.3104758	0	1	0.201242	-27.06333
349899	cg19392551	35758373	35758373	0.4984325	0	1	0.3876206	-21.5513
349971	cg19398192	65712353	42639385	0.4426404	0	1	0.3380793	-20.19851
349976	cg19398457	71803437	71803437	0.4518004	0	1	0.5556688	19.31925
350062	cg19403312	38619467	38619467	0.3246903	0	1	0.4690594	34.84681
350117	cg19405883	28672500	28672500	0.3798882	0	1	0.4817419	18.84884
350175	cg19408572	22647322	26687438	0.1546063	0	1	0.0331254	-65.40038
350232	cg19411734	60751362	60751362	0.4250448	0	1	0.5450627	24.49942
350310	cg19415746	68625351	68625351	0.8363782	0	1	0.3269764	-371.3336
350345	cg19418318	60709383	60709383	0.584891	0	1	0.7527375	53.80909
350347	cg19418525	40631412	40631412	0.3058752	0	1	0.4106711	20.94796
350361	cg19419291	39699342	71736313	0.2410572	0	1	0.3581411	27.63547
350461	cg19426572	74655465	14802305	0.6304041	0	1	0.5127789	-24.71841
350495	cg19428602	46662383	46662383	0.7387386	0	1	0.8812075	64.42834
350726	cg19442647	52704394	74665486	0.3012405	0	1	0.1674446	-40.83788
350813	cg19447962	46763425	59680396	0.5186131	0	1	0.3660188	-37.44006
350814	cg19447966	24796508	24796508	0.3852843	0	1	0.5554172	45.32836
350848	cg19449948	66788487	66788487	0.1159313	0	1	0.2220608	34.38812
350863	cg19450816	30628447	30628447	0.5868224	0	1	0.7148337	31.90656
351047	cg19463193	69779335	69779335	0.4182004	0	1	0.5672503	35.71313
351138	cg19469447	19685405	19685405	0.2332562	0	1	0.3670471	34.78641
351154	cg19470812	50687414	50687414	0.4862108	0	1	0.5902572	19.65036
351168	cg19471466	44792377	44792377	0.7041777	0	1	0.5783191	-30.44658

351236	cg1947590[illegible]	27728477	32691488	0.8813422	0	1	0.7612312	-50.29457
351258	cg1947678[illegible]	35650493	46680357	0.6957105	0	1	0.5873402	-23.60865
351349	cg1948168[illegible]	58680416	58680416	0.6507202	0	1	0.5426886	-22.01047
351379	cg1948409[illegible]	25692469	25692469	0.7595271	0	1	0.4485192	-163.7903
351385	cg1948430[illegible]	26682315	58741495	0.6224006	0	1	0.5188164	-19.98046
351433	cg1948786[illegible]	61791398	21674385	0.3607469	0	1	0.234549	-31.56462
351483	cg1949122[illegible]	43711300	43711300	0.9269265	0	1	0.7025824	-152.7557
351540	cg1949561[illegible]	31707422	31707422	0.3589788	0	1	0.4853075	27.13781
351682	cg1950324[illegible]	24659420	66736311	0.7938188	0	1	0.6661014	-38.6001
351732	cg1950625[illegible]	15645445	15645445	0.3738861	0	1	0.4944787	24.92317
351798	cg1950939[illegible]	37622407	20617345	0.7884414	0	1	0.6449326	-45.65047
351903	cg1951472[illegible]	72789432	72789432	0.4238575	0	1	0.3081917	-24.47997
351943	cg1951692[illegible]	32758486	32758486	0.4133815	0	1	0.5500488	30.62017
352008	cg1952023[illegible]	65796424	65796424	0.2917739	0	1	0.5121564	76.30926
352028	cg1952161[illegible]	37671427	54784313	0.445106	0	1	0.5587714	22.41541
352092	cg1952549[illegible]	22736306	55730490	0.4451723	0	1	0.3302982	-23.6738
352159	cg1952970[illegible]	53619307	36739481	0.9289843	0	1	0.8280843	-53.16954
352271	cg1953678[illegible]	37640352	37640352	0.8003348	0	1	0.9007878	43.51513
352285	cg1953771[illegible]	52694336	43710358	0.4123607	0	1	0.1913087	-88.10419
352323	cg1953998[illegible]	12758378	12758378	0.7688085	0	1	0.8817844	46.12268
352418	cg1954593[illegible]	59809335	59809335	0.4899275	0	1	0.3633187	-27.17657
352539	cg1955396[illegible]	32663387	32663387	0.610834	0	1	0.7144191	22.76233
352582	cg1955677[illegible]	10671472	27751496	0.1837201	0	1	0.0521546	-64.8221
352628	cg1956021[illegible]	53731484	53731484	0.8176084	0	1	0.9335281	66.30752
352798	cg1957077[illegible]	32771462	15679450	0.4752236	0	1	0.3656166	-21.37758
352939	cg1957921[illegible]	66676426	62762448	0.6621752	0	1	0.4832683	-52.10698
353006	cg1958423[illegible]	19601371	19601371	0.2578639	0	1	0.3655231	23.45268
353041	cg1958567[illegible]	44725418	44725418	0.7684495	0	1	0.5950246	-58.88111
353278	cg1959687[illegible]	71733384	71733384	0.5073517	0	1	0.7004678	62.41965
353427	cg1960541[illegible]	42717455	42717455	0.7300584	0	1	0.8488812	42.35754
353479	cg1960943[illegible]	37692420	37692420	0.2600009	0	1	0.40363	37.30289
353494	cg1961075[illegible]	47697491	27663422	0.3465779	0	1	0.2464237	-21.38491
353719	cg1962362[illegible]	13677339	54604468	0.4556975	0	1	0.3496732	-20.46584
353819	cg1962898[illegible]	70743474	70743474	0.5100589	0	1	0.7046419	63.62652
353880	cg1963260[illegible]	59792327	59792327	0.9010718	0	1	0.7874141	-51.93892
353921	cg1963540[illegible]	68605351	68605351	0.470171	0	1	0.6071023	31.29222
353946	cg1963662[illegible]	43728500	43728500	0.7061037	0	1	0.8485944	55.62983
354033	cg1964082[illegible]	63719500	63719500	0.5716633	0	1	0.6903795	27.0677
354037	cg1964132[illegible]	28633339	34644367	0.6260062	0	1	0.7345058	25.53182
354182	cg1965070[illegible]	51600390	51600390	0.4947255	0	1	0.627492	30.13937
354187	cg1965111[illegible]	65652432	65652432	0.4457499	0	1	0.2265889	-81.52558
354205	cg1965175[illegible]	50649322	50649322	0.5569314	0	1	0.6825032	29.2886
354220	cg1965324[illegible]	43737438	43737438	0.8258075	0	1	0.6170046	-93.15233

354269	cg19656425	41794410	41794410	0.8844773	0	1	0.5411072	-250.2995
354583	cg19678392	36727471	70710392	0.5349433	0	1	0.3471589	-54.82618
354664	cg19683417	53760422	53760422	0.4338461	0	1	0.5617643	27.35854
354666	cg19683494	50780489	50780489	0.3500599	0	1	0.5223153	46.775
354749	cg19689330	46677368	46677368	0.2902855	0	1	0.436756	36.97624
354758	cg19689876	31612432	31612432	0.6601417	0	1	0.7926125	40.72538
354781	cg19691260	36743375	36743375	0.2052592	0	1	0.3068822	23.95045
354882	cg19696794	34711400	34711400	0.2547052	0	1	0.3599685	22.76237
355034	cg19705210	32679304	32679304	0.428839	0	1	0.53843	21.03917
355076	cg19707653	57743487	57743487	0.5941113	0	1	0.728286	35.3541
355373	cg19722082	15638325	15638325	0.3529601	0	1	0.5285912	48.39274
355423	cg19724567	47774317	47774317	0.2833653	0	1	0.4295315	37.16263
355462	cg19726630	38736359	38736359	0.0405069	0	1	0.4224482	374.3439
355463	cg19726711	53789502	53789502	0.5217457	0	1	0.677107	41.46738
355491	cg19728223	63645353	63645353	0.4586281	0	1	0.357765	-18.78617
355517	cg19729930	20728450	20728450	0.4062992	0	1	0.2820609	-28.42054
355522	cg19730268	72622502	72622502	0.4244679	0	1	0.3226969	-19.62737
355581	cg19733736	63663437	63663437	0.3059203	0	1	0.4639537	41.41252
355627	cg19735421	44720402	44720402	0.8027945	0	1	0.6559876	-49.41541
355834	cg19745930	60730367	60730367	0.1459812	0	1	0.2613267	34.74383
355906	cg19749898	24694465	44652349	0.3271914	0	1	0.4369715	22.05781
355949	cg19752602	35665438	35665438	0.3419406	0	1	0.4499104	21.19945
355997	cg19754833	17683336	43763498	0.2840961	0	1	0.1776169	-27.7496
356017	cg19755813	10773369	26610429	0.9806752	0	1	0.7558216	-203.6608
356080	cg19758837	64717398	64717398	0.5498812	0	1	0.6631907	24.13405
356103	cg19759671	15716438	65697462	0.3092166	0	1	0.5101352	63.41466
356106	cg19759847	43696369	43696369	0.7844629	0	1	0.9039492	56.72085
356246	cg19766988	20616320	20616320	0.4341936	0	1	0.60885	48.02707
356312	cg19770671	19680502	19680502	0.4787757	0	1	0.6173392	32.14706
356385	cg19774683	50611302	50611302	0.0769861	0	1	0.3800698	374.3439
356404	cg19775763	22670481	73781407	0.7463527	0	1	0.6193709	-33.64829
356456	cg19778375	53777447	20734362	0.9415892	0	1	0.6747794	-205.905
356461	cg19778647	50607419	50607419	0.6174515	0	1	0.7208365	22.9897
356601	cg19787013	50641413	40741305	0.808175	0	1	0.5735565	-107.6446
356684	cg19791271	59625487	59625487	0.4609875	0	1	0.7243634	113.589
356799	cg19798881	10622317	42682438	0.1780426	0	1	0.3054575	36.66714
357092	cg19816075	31784354	31784354	0.7759207	0	1	0.8927818	51.46972
357319	cg19827875	67742337	31648360	0.2844851	0	1	0.3986211	24.68671
357361	cg19830372	21709404	60749337	0.9054318	0	1	0.8054129	-44.54776
357396	cg19832565	13755419	13755419	0.3325621	0	1	0.4667169	30.56514
357428	cg19836199	26695382	26695382	0.6577009	0	1	0.7586636	24.21389
357537	cg19843891	29795448	62706410	0.339476	0	1	0.1721078	-57.70022
357545	cg19844283	49775320	49775320	0.5495304	0	1	0.6550522	21.31301

357611	cg1984757	70786455	70786455	0.7311008	0	1	0.4375958	-141.1502
357634	cg1984873	22745427	22745427	0.695383	0	1	0.8381191	53.4955
357637	cg1984892	18686334	18686334	0.7609555	0	1	0.8824639	51.45022
357815	cg1985821	59655427	59655427	0.1437434	0	1	0.2566571	33.89427
357945	cg1986406	61679361	61679361	0.3893387	0	1	0.491991	19.01543
358118	cg1987392	17781439	17781439	0.7525593	0	1	0.6006301	-45.62813
358147	cg1987609	71761486	71761486	0.1732001	0	1	0.3180436	45.56104
358297	cg1988503	35712386	35712386	0.4663548	0	1	0.5875548	25.19805
358304	cg1988562	28772411	28772411	0.0763525	0	1	0.2351765	74.86142
358368	cg1988985	59725332	59725332	0.638928	0	1	0.757374	30.94861
358438	cg1989472	74609367	74609367	0.6650074	0	1	0.7891834	36.41729
358483	cg1989727	41718424	42744398	0.3122595	0	1	0.1580894	-52.57774
358491	cg1989810	11674459	11674459	0.624995	0	1	0.4696105	-39.38428
358511	cg1989953	42755505	42755505	0.4067885	0	1	0.2715148	-33.18473
358576	cg1990392	44719347	29742380	0.7733368	0	1	0.9570833	139.908
358589	cg1990435	31611431	31611431	0.4759423	0	1	0.3516021	-26.54203
358639	cg1990725	21714440	45748377	0.4632116	0	1	0.5723517	21.05267
358760	cg1991273	17667498	17667498	0.4637988	0	1	0.3568621	-20.6479
358915	cg1992165	42773329	42773329	0.339349	0	1	0.5022959	42.57971
359035	cg1992750	15756427	15756427	0.3133818	0	1	0.1619523	-50.5145
359088	cg1993000	17623415	17623415	0.3408652	0	1	0.443391	19.52811
359213	cg1993703	15742318	15742318	0.358866	0	1	0.4675586	21.17262
359215	cg1993706	31777487	56706476	0.1130607	0	1	0.2440193	47.78091
359381	cg1994746	53645471	53645471	0.3499853	0	1	0.4842319	30.23047
359494	cg1995423	44758434	44758434	0.5377216	0	1	0.6532055	24.57753
359566	cg1995859	45670346	45670346	0.350957	0	1	0.5246028	47.4423
359593	cg1996061	50669464	50669464	0.1966103	0	1	0.3375498	41.01741
359604	cg1996115	72760479	52661376	0.6799673	0	1	0.4400559	-90.786
359705	cg1996766	56696355	29681401	0.514884	0	1	0.3108659	-65.15995
359770	cg1997264	48651395	48651395	0.4463785	0	1	0.6431683	60.89179
359789	cg1997412	57652366	57652366	0.2775048	0	1	0.3780539	20.39569
359808	cg1997530	39678468	29753506	0.9147258	0	1	0.7943484	-61.30412
359822	cg1997603	23677489	23677489	0.5621706	0	1	0.7586873	71.08631
359826	cg1997644	44698394	44698394	0.8106945	0	1	0.6735854	-45.539
359874	cg1997910	45742455	45742455	0.1686136	0	1	0.3515968	67.39162
359926	cg1998203	70707453	70707453	0.4050255	0	1	0.5070317	18.73989
359954	cg1998394	63794307	63794307	0.1394307	0	1	0.2577879	36.99593
360008	cg1998790	72739359	72739359	0.2221246	0	1	0.3370514	27.8985
360152	cg1999706	59762379	22730465	0.3906364	0	1	0.2273391	-48.9185
360244	cg2000217	25675354	25675354	0.7767217	0	1	0.6694438	-27.95485
360255	cg2000290	18674314	73703502	0.3088954	0	1	0.4349969	28.17797
360310	cg2000665	60601461	55626498	0.8233851	0	1	0.6889071	-46.19022
360316	cg2000702	61733478	63671402	0.2331061	0	1	0.0567886	-95.57756

360532	cg20020143	32673453	68605367	0.3555678	0	1	0.0949914	-148.1468
360536	cg20020723	51764502	51764502	0.2856249	0	1	0.1796811	-27.36892
360550	cg20022036	44656440	44656440	0.8076671	0	1	0.559095	-118.6437
360616	cg20026367	55661387	55661387	0.7251983	0	1	0.6219346	-23.15829
360640	cg20027946	63760366	63760366	0.7971505	0	1	0.9245919	70.71347
360756	cg20035679	44729502	44729502	0.5144816	0	1	0.6920604	53.26024
360902	cg20045696	42794428	42794428	0.7612543	0	1	0.8869568	55.34857
360935	cg20048045	35687429	35687429	0.2714407	0	1	0.3792042	22.96475
360966	cg20050484	63714318	63714318	0.2239572	0	1	0.3295382	24.31927
361003	cg20053110	45641463	31810473	0.893038	0	1	0.7878139	-44.35354
361122	cg20060632	39792380	39792380	0.5603133	0	1	0.672946	24.2239
361182	cg20064153	49624409	49624409	0.4759908	0	1	0.3112196	-44.32398
361235	cg20066782	67725489	67725489	0.2229804	0	1	0.3260302	23.46799
361337	cg20073313	47630313	17625416	0.9606937	0	1	0.8160616	-107.9027
361384	cg20076126	49774361	49774361	0.3347906	0	1	0.4736753	32.35933
361391	cg20076516	41782446	41782446	0.3768478	0	1	0.4902939	22.49309
361421	cg20078646	66678384	66678384	0.1403305	0	1	0.2551729	35.21243
361423	cg20078807	66714495	66714495	0.321212	0	1	0.4444402	26.78789
361531	cg20087457	53631452	53631452	0.718343	0	1	0.8400479	42.30061
361532	cg20087519	10682449	49799315	0.5596287	0	1	0.3667906	-57.30722
361537	cg20088245	69751485	69751485	0.7719299	0	1	0.6534286	-32.19125
361555	cg20089799	37764504	37764504	0.4832535	0	1	0.6153803	29.61462
361558	cg20090066	41707350	41707350	0.7756066	0	1	0.6059618	-57.77951
361564	cg20090283	58795481	67686425	0.5000304	0	1	0.3894716	-21.45747
361624	cg20094343	27791426	27791426	0.383296	0	1	0.2356396	-40.71626
361816	cg20106077	49631492	49631492	0.4109924	0	1	0.9050019	374.3439
361931	cg20114524	72763473	72763473	0.7331464	0	1	0.8459559	38.68888
361950	cg20116159	33765311	59601326	0.5263703	0	1	0.4245509	-18.64247
362025	cg20119798	55721375	55721375	0.798093	0	1	0.6958864	-27.74577
362027	cg20119891	50617369	50617369	0.4276723	0	1	0.5676865	32.0039
362225	cg20132862	38701360	22791459	0.9859946	0	1	0.8650196	-110.28
362293	cg20137746	63661307	62647384	0.7394376	0	1	0.6302985	-26.05827
362348	cg20141398	63670429	25809507	0.2489433	0	1	0.3633007	26.22479
362373	cg20142762	73630387	73630387	0.4388228	0	1	0.2831319	-41.38373
362397	cg20144261	63723458	63723458	0.633904	0	1	0.7836271	48.18716
362735	cg20164601	24804471	68657454	0.4998106	0	1	0.3631192	-30.95735
362987	cg20184056	55689349	55689349	0.4925139	0	1	0.5962892	19.64013
363037	cg20188017	10758349	10758349	0.726352	0	1	0.8402475	38.30464
363038	cg20188212	12636503	12636503	0.3700344	0	1	0.1059147	-146.6745
363041	cg20188490	45773423	45773423	0.8491433	0	1	0.6738271	-76.49274
363050	cg20189244	41702417	58683406	0.227501	0	1	0.10935	-41.55076
363070	cg20190922	57708313	57708313	0.6630932	0	1	0.5629594	-19.82549
363251	cg20205188	29676313	29676313	0.4897632	0	1	0.7437349	108.9684

363276	cg20207327	15787404	15787404	0.6845664	0	1	0.7946008	30.70933
363300	cg20208600	74785399	74785399	0.5051143	0	1	0.405026	-18.17405
363364	cg20212912	45745470	45745470	0.437149	0	1	0.5601006	25.57155
363391	cg20214871	40789333	40789333	0.3446158	0	1	0.4847718	32.64444
363443	cg20218571	73790507	73790507		0	1	0.8059062	4.34E-09
363500	cg20223206	28672338	28672338	0.5201313	0	1	0.4110652	-20.88434
363507	cg20223687	42650326	16610447	0.7268397	0	1	0.8338968	33.98899
363614	cg20231299	36657311	36657311	0.7415528	0	1	0.8702478	52.61416
363769	cg20242889	15736503	71710493	0.4795277	0	1	0.2011996	-131.3751
363808	cg20246113	54789496	71748377	0.2933763	0	1	0.1383825	-56.42096
363867	cg20249327	11758417	11758417	0.0868917	0	1	0.3471197	151.7644
363887	cg20250341	67750401	67750401	0.213073	0	1	0.332092	30.09529
363989	cg20255370	26731480	30771301	0.6280044	0	1	0.1903462	-345.25
364092	cg20262330	47808302	47808302	0.4979733	0	1	0.3898217	-20.69468
364122	cg20264068	48676422	70637481	0.1693266	0	1	0.037984	-70.47643
364132	cg20264966	49718390	49718390	0.7517604	0	1	0.6499553	-24.09307
364163	cg20267322	41696395	41696395	0.6245784	0	1	0.8069725	70.7903
364197	cg20269046	33757366	20798360	0.151907	0	1	0.0310895	-65.82059
364258	cg20272648	41747302	41747302	0.2779222	0	1	0.4046048	29.5122
364267	cg20273260	67803443	67803443	0.3676986	0	1	0.4714323	19.53097
364331	cg20276780	49660309	49660309	0.8163517	0	1	0.6772324	-47.48735
364394	cg20281601	56683470	74788335	0.3917203	0	1	0.2503729	-36.89841
364405	cg20282550	43628367	30753492	0.3578856	0	1	0.2000047	-48.95467
364427	cg20284019	44796422	44796422	0.2865712	0	1	0.4074481	27.01026
364432	cg20284239	49746434	49746434	0.2414718	0	1	0.3653991	30.2468
364453	cg20285546	31603433	31603433	0.6583543	0	1	0.5556029	-20.51884
364486	cg20287936	60624477	60624477	0.7028851	0	1	0.8058474	28.81187
364499	cg20288617	50689426	50689426	0.4241486	0	1	0.5527808	27.5829
364520	cg20289854	48606406	48606406	0.3160733	0	1	0.441256	27.63232
364660	cg20299670	72697402	72697402	0.7407592	0	1	0.5411031	-70.54438
364680	cg20300514	53768450	53768450	0.3471187	0	1	0.0905595	-146.4888
364688	cg20300784	58697445	58697445	0.6126409	0	1	0.7347347	30.78685
364834	cg20310608	53748416	22764447	0.6748214	0	1	0.5450006	-30.59294
364842	cg20311002	67768324	67768324	0.5323716	0	1	0.7280303	66.60417
364856	cg20311673	48606478	48723444	0.7378013	0	1	0.6161256	-30.82652
364865	cg20312012	17683345	17683345	0.2382688	0	1	0.3827603	39.09198
364877	cg20312418	60802385	60802385	0.8112525	0	1	0.9211671	56.4715
365021	cg20321086	48745481	53754400	0.3280856	0	1	0.1948823	-37.62157
365091	cg20325573	39771403	39771403	0.7331306	0	1	0.8536687	44.20089
365125	cg20327845	24655434	41653386	0.2126275	0	1	0.3441429	35.33397
365168	cg20331155	58644321	58644321	0.2331734	0	1	0.3373554	23.35146
365190	cg20332503	45620310	45620310	0.5387569	0	1	0.6466264	21.85291
365215	cg20334115	33801340	33801340	0.5243319	0	1	0.6408646	24.59878

365246	cg20336016	58689457	58689457	0.6350965	0	1	0.9003653	374.3439
365282	cg20337996	28626323	28626323	0.3874956	0	1	0.2772626	-23.5947
365326	cg20340720	16728426	16728426	0.6305959	0	1	0.7435449	27.76396
365328	cg20340866	11678484	65669459	0.763635	0	1	0.6628905	-24.45696
365370	cg20344388	44636396	44636396	0.456428	0	1	0.5784769	25.39362
365414	cg20347665	68779491	14756391	0.2103894	0	1	0.0895374	-47.03036
365626	cg20364183	49676321	49676321	0.8583019	0	1	0.7317067	-48.55954
365714	cg20370184	70714459	70714459	0.3225039	0	1	0.196924	-34.07357
365742	cg20372591	61778393	61778393	0.4081773	0	1	0.5325401	26.02614
365747	cg20372745	67706308	12660310	0.536678	0	1	0.4117144	-26.23565
365806	cg20377305	39711501	16773420	0.2924596	0	1	0.1716644	-34.35033
365865	cg20381372	15777504	15777504	0.900367	0	1	0.608107	-201.3308
365929	cg20384620	19794463	64625488	0.6968254	0	1	0.8687511	80.29361
365993	cg20387775	13726478	13726478	0.2458639	0	1	0.3600912	26.32724
366013	cg20388732	67755386	67755386	0.1867404	0	1	0.2944283	27.51018
366047	cg20390711	56715415	53601482	0.9283862	0	1	0.7524211	-111.3614
366157	cg20399616	63653479	28680319	0.5416447	0	1	0.3931045	-35.47651
366185	cg20401549	66684405	66684405	0.0305556	0	1	0.1433082	60.15155
366206	cg20402783	66662387	33633366	0.8000922	0	1	0.3996026	-290.1862
366224	cg20403938	34752304	28762345	0.3533753	0	1	0.2092682	-41.28346
366296	cg20409752	48623369	48623369	0.5479506	0	1	0.7539557	76.35461
366320	cg20411555	25618398	25618398	0.6280798	0	1	0.7314377	23.50709
366427	cg20418529	64762372	39730349	0.1615125	0	1	0.2696716	29.87448
366531	cg20426275	36706349	36706349	0.7083478	0	1	0.9122332	124.9762
366664	cg20433858	17795371	17795371	0.3645118	0	1	0.482213	24.04554
366673	cg20434422	71609502	71609502	0.6005713	0	1	0.7203256	28.96648
366790	cg20441912	47727341	67792481	0.8645209	0	1	0.7579281	-38.75465
366916	cg20450471	12656497	38620431	0.531304	0	1	0.4252338	-19.93223
366964	cg20453861	63637430	63637430	0.4771613	0	1	0.6909537	74.04926
367013	cg20457051	74687328	74687328	0.2750336	0	1	0.4734294	63.92445
367053	cg20459037	27804461	27804461	0.0736094	0	1	0.2545153	91.73874
367150	cg20464155	27634393	27634393	0.242788	0	1	0.3428594	21.50889
367164	cg20464884	62676315	62676315	0.6096253	0	1	0.7191172	25.06603
367227	cg20469003	59735428	59735428	0.7180355	0	1	0.8215776	30.78043
367234	cg20469507	27692475	27692475	0.207376	0	1	0.3875124	60.02635
367352	cg20477705	37713507	37713507	0.5835086	0	1	0.4426894	-32.46484
367376	cg20479209	17633366	17633366	0.8864133	0	1	0.5910826	-195.8946
367514	cg20488673	16785488	16785488	0.5050418	0	1	0.6936952	59.35394
367614	cg20494779	42605490	42605490	0.403342	0	1	0.5081363	19.58563
367751	cg20503907	59775378	59775378	0.5820355	0	1	0.6856055	21.61382
367752	cg20503915	41745467	39679510	0.5518234	0	1	0.3953173	-38.93527
367790	cg20506807	21800380	46796390	0.8121165	0	1	0.9310523	67.39506
367794	cg20507276	25806371	25806371	0.1371507	0	1	0.3116933	68.34488

367816	cg2051003[illegible]	11709493	11709493	0.3086593	0	1	0.4633425	39.80043
367875	cg2051341[illegible]	68734497	68734497	0.6022296	0	1	0.7256293	30.71854
368004	cg2052239[illegible]	20643447	20643447	0.3226169	0	1	0.4499399	28.24166
368080	cg2052878[illegible]	28708301	28708301	0.4682872	0	1	0.6038607	30.7056
368106	cg2053084[illegible]	47798492	47798492	0.6215444	0	1	0.3955756	-78.16257
368143	cg2053398[illegible]	57741364	57741364	0.4454837	0	1	0.6138181	45.05351
368191	cg2053697[illegible]	48795471	48795471	0.823164	0	1	0.6324422	-79.86642
368238	cg2054023[illegible]	13606314	13606314	0.3864236	0	1	0.5776436	56.34305
368387	cg2054929[illegible]	73796474	73796474	0.86693	0	1	0.4972611	-274.6334
368394	cg2055001[illegible]	20688329	74727490	0.3226596	0	1	0.5984679	116.8173
368430	cg2055300[illegible]	16702322	16702322	0.3078088	0	1	0.4239518	24.65475
368450	cg2055437[illegible]	70636447	70636447	0.8012964	0	1	0.6956648	-29.4922
368537	cg2055921[illegible]	33655451	33655451	0.8933825	0	1	0.7213682	-89.79781
368573	cg2056126[illegible]	38732415	38732415	0.6388772	0	1	0.7757786	40.86765
368585	cg2056193[illegible]	32730395	32730395	0.6492131	0	1	0.4922702	-40.894
368604	cg2056327[illegible]	49686368	33708300	0.51098	0	1	0.3443833	-44.16901
368686	cg2056810[illegible]	36666492	36666492	0.7237514	0	1	0.8372064	37.60322
368697	cg2056842[illegible]	12740464	12740464	0.3875725	0	1	0.6624028	117.113
368722	cg2056980[illegible]	59676454	59676454	0.2536941	0	1	0.3677706	25.8992
368817	cg2057659[illegible]	58762444	58762444	0.3139131	0	1	0.4646403	37.87564
368865	cg2058085[illegible]	13714328	13714328	0.7174707	0	1	0.8206776	30.52481
368875	cg2058187[illegible]	71619415	65808494	0.5227512	0	1	0.4024395	-24.62281
368916	cg2058484[illegible]	54797458	54797458	0.1910119	0	1	0.2979496	26.89661
368920	cg2058499[illegible]	74778381	48629465	0.2646338	0	1	0.1315738	-45.57401
369018	cg2059201[illegible]	11637351	15737394	0.7951246	0	1	0.6638162	-40.47712
369020	cg2059212[illegible]	39750490	39750490	0.4480757	0	1	0.5680966	24.60332
369028	cg2059239[illegible]	69708495	69708495	0.4732343	0	1	0.1725345	-159.0313
369036	cg2059283[illegible]	43730356	43730356	0.2275112	0	1	0.3863622	46.69095
369111	cg2059677[illegible]	36751506	36751506	0.7832219	0	1	0.8901384	44.62083
369302	cg2061045[illegible]	62753398	39645307	0.4605663	0	1	0.3501312	-21.85352
369385	cg2061732[illegible]	18710494	18710494	0.6883821	0	1	0.5605771	-30.41426
369625	cg2063381[illegible]	73667326	73667326	0.2844774	0	1	0.1399638	-50.29267
369725	cg2063980[illegible]	35692332	35692332	0.7453901	0	1	0.6106843	-36.91371
369740	cg2064037[illegible]	18642456	31658443	0.5909355	0	1	0.8234947	110.3991
369764	cg2064167[illegible]	10738373	10738373	0.5908806	0	1	0.7278492	36.5277
369791	cg2064344[illegible]	42606486	42606486	0.4032811	0	1	0.5445696	32.45623
369915	cg2065164[illegible]	47692461	47692461	0.4100075	0	1	0.5981528	54.83483
369941	cg2065312[illegible]	32640330	32640330	0.721725	0	1	0.6017755	-29.12333
370012	cg2065770[illegible]	12673404	12673404	0.3605098	0	1	0.483838	26.0444
370091	cg2066291[illegible]	39793457	27705428	0.5238103	0	1	0.3881393	-30.30196
370096	cg2066304[illegible]	73745465	73745465	0.2051051	0	1	0.3541026	44.00518
370125	cg2066444[illegible]	44618449	49704310	0.7441545	0	1	0.8469439	33.81643
370345	cg2067625[illegible]	24744337	24744337	0.4193999	0	1	0.5355781	23.18692

370395	cg2067883[illegible]	50695330	50695330	0.3178956	0	1	0.4316968	23.59493
370396	cg2067898[illegible]	63806509	63806509	0.8022	0	1	0.6224673	-68.18082
370404	cg2067940[illegible]	43772339	43772339	0.7530649	0	1	0.8587273	37.19736
370475	cg2068344[illegible]	62708473	54684413	0.3770518	0	1	0.494663	23.87366
370530	cg2068620[illegible]	59637311	59637311	0.4110646	0	1	0.6007021	55.68552
370532	cg2068627[illegible]	71638430	63712320	0.5740101	0	1	0.6759968	20.78404
370704	cg2069561[illegible]	70661373	70661373	0.632953	0	1	0.7717505	41.32478
370787	cg2069927[illegible]	71654483	71654483	0.2178302	0	1	0.3191064	23.11343
370847	cg2070220[illegible]	28808505	28808505	0.23298	0	1	0.3350323	22.62085
370918	cg2070496[illegible]	21621390	21621390	0.8898481	0	1	0.767545	-54.04811
370939	cg2070589[illegible]	15653321	15653321	0.3942009	0	1	0.549243	38.28632
371113	cg2071381[illegible]	69709355	13644459		0	1		-4.34E-09
371221	cg2072059[illegible]	12740331	28690392	0.1480304	0	1	0.0341859	-59.42366
371272	cg2072403[illegible]	59755484	59755484	0.6308751	0	1	0.760881	36.19055
371406	cg2073193[illegible]	49698416	10682396	0.3242209	0	1	0.0659776	-160.6201
371447	cg2073506[illegible]	28732348	28732348	0.6237399	0	1	0.5130704	-22.2484
371537	cg2074104[illegible]	40687318	40687318	0.5077037	0	1	0.703177	64.01184
371550	cg2074152[illegible]	34749417	34749417	0.2698487	0	1	0.1283455	-50.7054
371633	cg2074648[illegible]	73669372	73669372	0.3653825	0	1	0.4730276	20.76175
371714	cg2075139[illegible]	11652370	11652370	0.6424641	0	1	0.7551792	28.47068
371768	cg2075602[illegible]	53647446	53647446	0.0693419	0	1	0.3836257	374.3439
371875	cg2076478[illegible]	54722475	54722475	0.5702368	0	1	0.6794658	23.28741
371895	cg2076734[illegible]	44648351	44648351	0.7314565	0	1	0.8434979	37.89013
371934	cg2076985[illegible]	44637311	44637311	0.5748351	0	1	0.7436078	53.26729
371979	cg2077314[illegible]	52728317	13657389	0.2683141	0	1	0.166472	-26.81504
372070	cg2077860[illegible]	25686351	25686351	0.2213906	0	1	0.3339642	27.04593
372076	cg2077891[illegible]	60629456	60629456	0.3230942	0	1	0.5327408	68.05485
372156	cg2078397[illegible]	43801338	43801338	0.4333084	0	1	0.5613723	27.40962
372253	cg2078948[illegible]	74659383	74659383	0.3413631	0	1	0.4492288	21.17628
372270	cg2079079[illegible]	26770430	26770430	0.7772505	0	1	0.6539559	-34.77253
372275	cg2079129[illegible]	71714440	71714440	0.6807304	0	1	0.3989983	-124.4246
372331	cg2079537[illegible]	57723448	63695473	0.5361319	0	1	0.4190379	-23.49496
372333	cg2079541[illegible]	63659363	63659363	0.8510549	0	1	0.5486093	-184.9206
372374	cg2079806[illegible]	69666477	69666477	0.745583	0	1	0.9561924	374.3439
372583	cg2081175[illegible]	53690493	53690493	0.7485319	0	1	0.6400557	-26.37507
372589	cg2081198[illegible]	31628448	12625453	0.3505895	0	1	0.5084917	40.03162
372625	cg2081417[illegible]	25742488	51751377	0.4847904	0	1	0.2574222	-83.61794
372718	cg2082104[illegible]	48808387	48808387	0.4606748	0	1	0.5784018	23.90978
372731	cg2082171[illegible]	57656364	57656364	0.6325151	0	1	0.747944	29.03136
372766	cg2082352[illegible]	35605502	35605502	0.7449214	0	1	0.899561	79.60409
372827	cg2082674[illegible]	47760413	47760413	0.0973814	0	1	0.2459202	61.70811
372843	cg2082828[illegible]	31612369	74658363	0.9464626	0	1	0.7125244	-177.385
372907	cg2083570[illegible]	39713425	39713425	0.4461663	0	1	0.547605	18.56023

372943	cg2083920(	72782462	14649377	0.7056608	0	1	0.5669013	-35.80577
373011	cg2084486	31733489	31733489	0.5104797	0	1	0.3756302	-30.09262
373107	cg2085098	46766420	46766420	0.6188899	0	1	0.7514718	36.49307
373217	cg2085984	69633331	69633331	0.6460682	0	1	0.4987642	-36.5578
373269	cg2086421	37637493	29685483	0.2094389	0	1	0.3167073	25.77943
373416	cg2087676(	39770411	39770411	0.2694902	0	1	0.1482706	-37.20943
373494	cg2088488	37803322	60728315	0.3482098	0	1	0.2461805	-22.02223
373525	cg2088736(	25669327	47748497	0.5010105	0	1	0.3604377	-32.52478
373616	cg2089393(	30740505	30740505	0.5520222	0	1	0.6663517	24.58766
373708	cg2089978	48636352	48636352	0.1554015	0	1	0.3018803	48.86944
373765	cg2090433(	62603309	62603309	0.3503083	0	1	0.5946319	90.91823
373798	cg2090713(	13706331	19607309	0.5039214	0	1	0.3531911	-36.8431
373800	cg2090740	33773300	33773300	0.597222	0	1	0.7049915	23.79107
373845	cg2091036	23728464	23728464	0.1766513	0	1	0.2916139	31.33624
373929	cg2091642	53682313	53682313	0.3926713	0	1	0.539799	34.88378
373937	cg2091707	17716311	17716311	0.3711587	0	1	0.4747123	19.43991
373944	cg2091749	70618476	70618476	0.0957924	0	1	0.3509225	142.9183
374027	cg2092442	30640339	30640339	0.2270145	0	1	0.3328826	24.2599
374067	cg2092739	13804374	13804374	0.1536907	0	1	0.2610944	30.33069
374075	cg2092765(	13616379	13616379	0.4644907	0	1	0.5817881	23.79914
374290	cg2094118	58669414	58669414	0.5062119	0	1	0.6739523	47.13514
374359	cg2094508	40674373	29626407	0.3096278	0	1	0.1796362	-37.67699
374416	cg2094827	36623459	36623459	0.2721245	0	1	0.3752112	21.38795
374417	cg2094836	49686431	19704373	0.4046733	0	1	0.2839544	-27.04932
374473	cg2095164	51768470	51768470	0.9110554	0	1	0.798351	-54.63363
374618	cg2096032	58758453	58758453	0.5052844	0	1	0.7812403	135.1055
374766	cg2096915	30775370	30775370	0.1882567	0	1	0.3131646	34.55321
374933	cg2097819	49784457	49784457	0.2912995	0	1	0.4031204	23.66616
374944	cg2097892	68705431	13754460	0.5856816	0	1	0.7056855	28.26889
374952	cg2097915	38808325	71720494	0.3367828	0	1	0.4534932	24.16111
374956	cg2097938	40604470	40604470	0.1497533	0	1	0.3017707	52.74504
374998	cg2098116	67601502	67601502	0.2297492	0	1	0.4198263	63.11954
375006	cg2098145	11725350	40708411	0.2534209	0	1	0.137138	-36.31013
375095	cg2098683	72743474	72743474	0.537437	0	1	0.4231513	-22.55793
375110	cg2098743	63774318	63774318	0.3933614	0	1	0.4996003	20.07841
375157	cg2099115	38770375	38770375	0.4467304	0	1	0.6354177	56.08587
375232	cg2099508	14801411	35782396	0.4672849	0	1	0.5748456	20.57819
375252	cg2099635	13809302	61637328	0.577804	0	1	0.4553035	-25.54482
375391	cg2100551(	35610381	44640449	0.8164231	0	1	0.6745073	-48.99407
375394	cg2100568	27653315	27653315	0.7954841	0	1	0.9283243	76.65974
375507	cg2101205	14677460	14677460	0.2304673	0	1	0.4113503	57.87186
375574	cg2101788	71619357	47602308	0.158208	0	1	0.2642565	29.30063
375699	cg2102831	71711491	71711491	0.4496761	0	1	0.7720986	374.3439

375778	cg21032567	19687445	50644406	0.4649571	0	1	0.0679255	-327.7817
375804	cg21034405	26684317	26684317	0.3573551	0	1	0.4630348	20.251
375908	cg21040096	70668361	70668361	0.2746461	0	1	0.380388	22.17517
375909	cg21040109	59656331	67614499	0.5618969	0	1	0.4602392	-18.69081
376001	cg21045575	10792451	10792451	0.1661726	0	1	0.2700827	27.67836
376051	cg21048700	59735421	59735421	0.0814998	0	1	0.2172677	58.03743
376064	cg21049397	71773502	71773502	0.4566918	0	1	0.6277366	46.74537
376116	cg21052766	51790432	70742444	0.2569605	0	1	0.3963489	35.66402
376153	cg21054919	63721486	63721486	0.5529037	0	1	0.4376485	-22.91208
376159	cg21055554	12765328	12765328	0.2246541	0	1	0.4536107	88.45956
376235	cg21062780	38635332	34689472	0.699432	0	1	0.5765367	-29.05499
376323	cg21070081	64787328	64787328	0.6777288	0	1	0.4227993	-101.854
376448	cg21078077	72651462	37638335	0.565383	0	1	0.2591555	-149.0719
376506	cg21082272	38628338	64679354	0.3044046	0	1	0.4064704	20.12523
376669	cg21093170	12741508	12741508	0.2572832	0	1	0.3816249	29.56263
376676	cg21093807	51790502	51609351	0.5027259	0	1	0.3905333	-21.97484
376726	cg21097788	58700335	58700335	0.1936137	0	1	0.3249343	36.89388
376842	cg21105875	34635360	37612334	0.5231825	0	1	0.4145033	-20.75504
376853	cg21106486	50801389	45649507	0.2378252	0	1	0.0772595	-75.7495
376981	cg21114725	10785309	39705421	0.6834013	0	1	0.92047	374.3439
377009	cg21116266	59722425	30706349	0.7542901	0	1	0.6491392	-25.49237
377100	cg21121609	23692348	23692348	0.3388166	0	1	0.4400185	19.16123
377177	cg21126626	56731381	56731381	0.4889053	0	1	0.3490507	-32.43571
377243	cg21130861	74646366	24683387	0.1479691	0	1	0.0315078	-62.43976
377373	cg21139795	27621380	27621380	0.8566965	0	1	0.4881204	-267.545
377454	cg21144063	26735430	26735430	0.6543867	0	1	0.5000661	-39.92746
377537	cg21148623	39620319	39620319	0.8426985	0	1	0.9428177	58.72391
377547	cg21149357	27800342	27800342	0.4641082	0	1	0.6278049	43.24257
377625	cg21153658	48737317	60749334	0.3227888	0	1	0.2166203	-24.9412
377675	cg21156383	62695318	38725337	0.4398873	0	1	0.3385229	-19.21503
377718	cg21158163	37710386	13616441	0.7674708	0	1	0.6088081	-50.7435
377724	cg21158501	51683349	24639308	0.092242	0	1	0.2148792	47.44757
377760	cg21160149	39644459	39644459	0.5118623	0	1	0.6270369	23.8087
377849	cg21164300	74616396	19793383	0.8873032	0	1	0.6991647	-99.39179
377920	cg21167402	36707329	28725425	0.3571028	0	1	0.1532683	-83.84389
377950	cg21169053	66643428	66643428	0.9185627	0	1	0.7980524	-62.95377
378064	cg21175685	33757409	33757409	0.7702675	0	1	0.893424	55.68649
378258	cg21186098	22682417	22682417	0.3134026	0	1	0.4308483	24.95283
378282	cg21187068	33657429	33657429	0.6744561	0	1	0.7765549	25.89251
378285	cg21187195	16608507	16608507	0.3741826	0	1	0.4786478	19.6863
378296	cg21187770	22600395	11635390	0.4634763	0	1	0.357877	-20.21932
378304	cg21188400	48623506	48623506	0.1674088	0	1	0.2749658	29.0695
378430	cg21195120	24682453	24682453	0.4021648	0	1	0.290966	-23.46583

378486	cg2119733(	14633388	50615402	0.5152339	0	1	0.6310411	24.11183
378538	cg2119992:	59607454	59607454	0.53951	0	1	0.6423208	20.14479
378593	cg2120227(	54709323	54709323	0.5721923	0	1	0.8056337	106.0611
378610	cg2120324(	16695486	16695486	0.862169	0	1	0.6002924	-149.9793
378711	cg2120795(	28669333	35692508	0.3743521	0	1	0.2498522	-30.00757
378718	cg2120827(	43681422	16804410	0.6676323	0	1	0.8214354	56.51179
378770	cg2121064:	15645328	15645328	0.2412238	0	1	0.3893266	40.50805
378909	cg2121742:	67633484	67633484	0.2783942	0	1	0.4678607	58.63872
378911	cg2121754(	29667425	31734421	0.5704355	0	1	0.4693281	-18.58458
379003	cg2122184(	11714457	17682427	0.8065147	0	1	0.6444941	-58.4539
379139	cg2122957(	72766402	28702423	0.7845829	0	1	0.6825995	-26.47966
379193	cg2123248(	13630343	40804496	0.2826405	0	1	0.1695181	-31.2188
379227	cg2123403:	44734473	44734473	0.333533	0	1	0.4661657	29.95969
379229	cg2123408:	14786388	14786388	0.8409468	0	1	0.43651	-313.0209
379406	cg2124244(	17615461	17615461	0.3701061	0	1	0.2357294	-34.8674
379416	cg2124306-	10697439	10697439	0.6933479	0	1	0.927962	374.3439
379444	cg2124413:	42731360	68807439	0.4243203	0	1	0.2322551	-64.00304
379507	cg2124772:	44771401	44771401	0.6571454	0	1	0.7724761	30.90167
379583	cg2125197(	28692399	22688415	0.7265748	0	1	0.6041094	-30.40567
379656	cg2125729:	11648326	11648326	0.4320303	0	1	0.5362517	19.36896
379730	cg2126554(	22798463	22798463	0.5409839	0	1	0.6778569	33.53206
379736	cg2126591'	54640409	37717456	0.8182366	0	1	0.9242337	54.90988
379811	cg2127227(	54669345	54669345	0.320075	0	1	0.1856769	-39.07959
379920	cg2128032(	38729303	38729303	0.4004499	0	1	0.2338137	-50.02277
380067	cg2129134:	39752480	39752480	0.4933335	0	1	0.5939655	18.66874
380079	cg2129185(	32715333	32715333	0.0726586	0	1	0.2543271	92.69468
380166	cg2129736(	43669411	43669411	0.636556	0	1	0.8577464	113.0997
380208	cg2130099:	10605381	14789475	0.4377831	0	1	0.325635	-22.86586
380214	cg2130134l	16715416	16715416	0.494581	0	1	0.7622218	123.5931
380258	cg2130428:	54764434	54764434	0.08041	0	1	0.1969225	46.63625
380457	cg2132310(	50718335	50718335	0.5269397	0	1	0.6494748	26.95473
380563	cg2133181:	39744307	14634387	0.5694107	0	1	0.463001	-20.17807
380568	cg2133230-	26794493	26794493	0.2472155	0	1	0.3588535	25.32161
380672	cg2134032(	63719354	44756317	0.7473341	0	1	0.8655586	45.38266
380738	cg2134594(	66710318	21782313	0.391282	0	1	0.2900524	-20.23979
381213	cg2138531-	72739466	72739466	0.7385551	0	1	0.5818486	-46.58456
381221	cg2138574(	67616437	74709458	0.3127013	0	1	0.4712366	41.38017
381317	cg2139270(	19729465	19729465	0.5061018	0	1	0.6454535	33.24783
381382	cg2139754(	31744475	31744475	0.6120285	0	1	0.5116508	-18.83657
381428	cg2140085l	43710318	43710318	0.5667462	0	1	0.7009485	33.62188
381486	cg2140493:	60704396	51742335	0.4356173	0	1	0.2517625	-57.54124
381528	cg2140881:	34738319	34738319	0.1793268	0	1	0.2906788	29.58987
381607	cg2141490:	15671471	15671471	0.5318605	0	1	0.7014604	49.8501

381612	cg2141522	65715345	65715345	0.3690517	0	1	0.4983342	28.07698
381872	cg2143568	35794359	57688310	0.3148757	0	1	0.5275582	70.24121
381882	cg2143657	37779393	35648401	0.8135568	0	1	0.7065507	-31.40064
381890	cg2143741	29648403	29648403	0.9532442	0	1	0.8252435	-87.67958
381939	cg2144077	25778431	25778431	0.6077376	0	1	0.7459221	38.51339
381959	cg2144227	17615445	17615445	0.2083612	0	1	0.3706429	50.29139
381997	cg2144555	33647476	14602378	0.8135054	0	1	0.6710781	-48.78455
382017	cg2144677	69795464	69795464	0.3297055	0	1	0.5488153	73.77641
382021	cg2144698	69682374	69682374	0.4984247	0	1	0.6847051	57.3862
382077	cg2145022	46640502	46640502	0.2646748	0	1	0.4431548	53.66253
382083	cg2145062	74746464	74746464	0.6842768	0	1	0.793528	30.26908
382091	cg2145078	55615448	70740454	0.8390936	0	1	0.6860288	-59.77098
382250	cg2146198	21782468	21782468	0.5045523	0	1	0.6219769	24.46504
382391	cg2147158	11682463	40651448	0.9517016	0	1	0.8452525	-68.58543
382431	cg2147410	18751494	18751494	0.5114477	0	1	0.6169875	20.49031
382433	cg2147424	27742412	27742412	0.3055505	0	1	0.444332	33.1811
382571	cg2148247	69797492	61690493	0.5551158	0	1	0.4485449	-20.14217
382598	cg2148388	37804335	36737432	0.2030119	0	1	0.092603	-40.54747
382622	cg2148494	20628314	57756386	0.8087281	0	1	0.6880582	-37.10659
382706	cg2149075	19684498	19684498	0.5434406	0	1	0.6675751	28.14767
382757	cg2149395	27713418	48651339	0.3163006	0	1	0.2089939	-25.8216
382846	cg2149854	45773411	45773411	0.4046936	0	1	0.2276191	-56.08248
383089	cg2151380	32806307	32806307	0.9139432	0	1	0.7920648	-62.05159
383263	cg2152406	26665478	26665478	0.62954	0	1	0.4890992	-33.20207
383307	cg2152677	28675375	28675375	0.245747	0	1	0.3870318	37.16672
383309	cg2152707	42730477	47738478	0.5180364	0	1	0.6271521	21.81397
383317	cg2152761	38647423	38647423	0.3233624	0	1	0.4387503	24.00077
383331	cg2152892	14672460	14672460	0.3177842	0	1	0.4459455	28.68269
383337	cg2152947	71704423	71704423	0.238061	0	1	0.3460626	24.46118
383352	cg2153026	59617486	59617486	0.7641841	0	1	0.6199353	-43.11637
383367	cg2153138	41625395	41625395	0.50995	0	1	0.6356765	27.72216
383459	cg2153594	39635363	52656337	0.3649332	0	1	0.2409752	-30.28738
383476	cg2153718	24764306	24764306	0.5230716	0	1	0.646555	27.21066
383587	cg2154343	25663371	25663371	0.7885462	0	1	0.9215078	73.39178
383718	cg2154895	60786424	38790471	0.7019211	0	1	0.5815402	-28.22297
383787	cg2155230	17661452	17661452	0.3295024	0	1	0.4326042	19.90311
383858	cg2155714	62615452	62615452	0.6616296	0	1	0.5547407	-21.93803
383926	cg2156170	32757473	44642452	0.4339457	0	1	0.3102345	-27.26304
384000	cg2156643	50669491	50669491	0.0818867	0	1	0.5758676	374.3439
384123	cg2157427	50724467	50724467	0.4306052	0	1	0.5419146	21.59336
384139	cg2157482	12645459	12645459	0.5924169	0	1	0.727861	35.86868
384208	cg2157845	62623454	62623454	0.3685702	0	1	0.5081815	32.02871
384213	cg2157864	11631406	11631406	0.6822707	0	1	0.9342765	374.3439

384277	cg21582112	52731499	52731499	0.7060221	0	1	0.8070317	28.10305
384360	cg21586152	42654505	16670326	0.5039039	0	1	0.3694088	-30.02771
384370	cg21587006	66612314	66612314	0.908246	0	1	0.7890509	-58.03677
384401	cg21589417	22623481	22623481	0.5462348	0	1	0.6908527	37.45011
384451	cg21593159	25634351	25634351	0.6945642	0	1	0.4776676	-76.34172
384455	cg21593409	12716411	12716411	0.5546481	0	1	0.4350744	-24.3743
384464	cg21593676	50643403	50643403	0.4832044	0	1	0.3804051	-19.12692
384487	cg21594702	46660491	46660491	0.2380165	0	1	0.4746043	92.15779
384538	cg21598489	17665351	17665351	0.9286903	0	1	0.8170953	-60.8172
384654	cg21605781	36656336	36656336	0.3959877	0	1	0.5096107	22.40682
384726	cg21609584	52647497	52647497	0.5920748	0	1	0.7136725	29.3032
384752	cg21610839	55730303	55730303	0.3893993	0	1	0.5096477	24.66927
384756	cg21610927	19687435	19687435	0.6110108	0	1	0.88429	374.3439
384791	cg21613620	10728413	10728413	0.2127492	0	1	0.3472142	36.61018
384798	cg21614201	42692387	42692387	0.4486778	0	1	0.5957739	35.1929
384851	cg21617357	49799346	49799346	0.6553689	0	1	0.7635642	27.3015
384963	cg21623445	31708474	31708474	0.5095599	0	1	0.6365659	28.21589
385069	cg21630843	13610445	13610445	0.8721364	0	1	0.5886596	-175.6861
385122	cg21634944	38617440	58629367		0	1	0.5200669	4.34E-09
385137	cg21635870	52726337	47718377	0.5398147	0	1	0.3779134	-41.46774
385234	cg21642050	46765335	46765335	0.6276009	0	1	0.8940746	374.3439
385259	cg21643262	28801369	28801369	0.4971504	0	1	0.5984914	18.93473
385308	cg21645268	58727386	58727386	0.6648075	0	1	0.5623981	-20.58713
385315	cg21645759	48612396	48612396	0.6836433	0	1	0.8379678	60.27367
385331	cg21646392	72635404	72635404	0.592128	0	1	0.6944864	21.53428
385340	cg21647182	72714356	72714356	0.2955058	0	1	0.4664361	47.98236
385368	cg21648376	28640480	14658488	0.5704911	0	1	0.4589818	-21.79244
385470	cg21655171	71731387	71731387	0.7956847	0	1	0.9160053	61.77373
385687	cg21670717	22640468	22640468	0.4124421	0	1	0.5254679	22.15185
385721	cg21672992	74799351	74799351	0.5522996	0	1	0.6583636	21.57758
385771	cg21679468	39808413	39808413	0.5360777	0	1	0.7012492	47.62571
385922	cg21690450	59707422	59707422	0.4381943	0	1	0.3307441	-21.23848
385936	cg21690921	14715322	14715322	0.4482821	0	1	0.5845949	30.70556
385959	cg21692620	65690437	49801463	0.446779	0	1	0.3160846	-29.68262
386003	cg21696374	46777321	46777321	0.2683593	0	1	0.4144535	37.89903
386021	cg21697769	72804500	72804500	0.9188037	0	1	0.7353595	-111.7129
386180	cg21712377	29754329	29754329	0.4539242	0	1	0.5768455	25.68315
386234	cg21717724	71663496	71663496	0.502512	0	1	0.3368187	-43.92754
386305	cg21722795	60720343	28699467	0.6377958	0	1	0.4752564	-43.03029
386431	cg21733531	33698309	73719447	0.3637859	0	1	0.2534342	-24.57933
386452	cg21735068	37686370	37686370	0.3669812	0	1	0.589389	75.38398
386531	cg21741284	42783350	70787499	0.7555836	0	1	0.643563	-28.2256
386532	cg21741515	32773475	32773475	0.3388011	0	1	0.5933025	98.83263

386539	cg21741998	38683447	38683447	0.5126107	0	1	0.6247501	22.74969
386663	cg21749951	72665489	72665489	0.3256211	0	1	0.4827499	40.31129
386671	cg21750589	62758508	62758508	0.6273454	0	1	0.7495726	31.90542
386874	cg21762589	63757329	63757329	0.6525785	0	1	0.534913	-25.31677
386943	cg21767657	60616459	60616459	0.5126514	0	1	0.6167845	20.05116
386988	cg21770393	66640321	66640321	0.5767751	0	1	0.7439848	52.4846
387103	cg21777188	40634335	40634335	0.3724647	0	1	0.2327768	-37.36037
387127	cg21778987	15666369	15666369	0.455241	0	1	0.5975516	33.25746
387142	cg21780600	15696410	15696410	0.7813752	0	1	0.9127911	68.37259
387192	cg21783442	12680303	12680303	0.6612715	0	1	0.7819543	34.0845
387195	cg21783672	60644498	60644498	0.6488736	0	1	0.5103934	-33.01344
387356	cg21792983	35623406	35623406	0.3364492	0	1	0.4539744	24.44505
387377	cg21794767	56615495	56615495	0.6119439	0	1	0.7210701	25.03346
387505	cg21805731	50674364	50674364	0.4780728	0	1	0.6181568	32.77024
387509	cg21805880	36696500	36696500	0.2679195	0	1	0.3837601	25.9154
387584	cg21809927	47711327	23679433	0.445282	0	1	0.2326858	-76.4931
387596	cg21810411	29679377	29679377	0.6695027	0	1	0.8130513	49.31119
387612	cg21811911	48689493	48689493	0.8624361	0	1	0.6365853	-118.7363
387620	cg21812850	39717419	39717419	0.8578901	0	1	0.7448976	-40.9238
387644	cg21814550	16735421	16735421	0.5561947	0	1	0.6968258	36.07794
387769	cg21823426	60751458	60751458	0.5939209	0	1	0.7035225	24.36785
387852	cg21829038	46608371	13645391	0.9619265	0	1	0.675687	-246.0255
387886	cg21830932	31754304	31754304	0.8978418	0	1	0.5960483	-209.9961
387891	cg21831183	65707402	65707402	0.5384276	0	1	0.6587961	26.46807
387938	cg21833776	73772414	73772414	0.4088507	0	1	0.5949272	53.67606
388026	cg21839504	44680459	47676368	0.5419165	0	1	0.6787891	33.57524
388055	cg21841632	55683413	55683413	0.4418806	0	1	0.5885237	34.8998
388076	cg21842920	66777402	66777402	0.1957162	0	1	0.3285809	37.39048
388153	cg21847118	37664311	37664311	0.4201423	0	1	0.5245898	19.43704
388271	cg21854332	11607314	11607314	0.2830375	0	1	0.4011841	26.15402
388290	cg21855211	12780345	12780345	0.2878896	0	1	0.1877429	-24.5291
388366	cg21859562	19654372	19654372	0.3305122	0	1	0.4407046	22.12351
388385	cg21860675	50647478	50647478	0.2873109	0	1	0.4621946	50.3835
388400	cg21862099	56698492	56698492	0.1692422	0	1	0.3224739	50.41677
388427	cg21864016	40755448	40755448	0.7998523	0	1	0.9089205	51.47
388511	cg21870038	37664451	37664451	0.6818969	0	1	0.8006335	35.22054
388742	cg21881798	61795328	61795328	0.2803326	0	1	0.3877998	22.55662
388784	cg21885112	73753465	56694324	0.3967316	0	1	0.6252814	80.00337
389026	cg21902325	60770320	60770320	0.545566	0	1	0.7223028	55.32927
389029	cg21902394	70630496	70630496	0.7401372	0	1	0.617349	-31.4411
389050	cg21903646	42616369	58643484	0.6006899	0	1	0.4913183	-21.44015
389182	cg21911276	15730437	15730437	0.4452923	0	1	0.3259387	-25.3028
389221	cg21913335	26716330	71672317	0.6522397	0	1	0.8731586	119.2334

389271	cg2191591(	53602438	24619471	0.7952899	0	1	0.6689677	-38.11767
389355	cg2192056(	47651462	47651462	0.8695798	0	1	0.7548887	-44.26131
389444	cg21926804	41692372	41692372	0.6695535	0	1	0.5633219	-21.95982
389458	cg21927991	25632330	25632330	0.7188519	0	1	0.4794983	-94.29424
389523	cg21931717	24684390	25616423	0.244835	0	1	0.0575984	-103.7091
389530	cg2193198(	73685456	73685456	0.3277007	0	1	0.4375556	22.07149
389538	cg21932368	26719328	25696394	0.4556391	0	1	0.276607	-53.14886
389618	cg21937395	69623491	69623491	0.5673686	0	1	0.4649624	-18.94836
389640	cg21938541	69691430	69691430	0.1673111	0	1	0.2770787	30.00361
389741	cg21945639	30642361	56781505	0.1318616	0	1	0.2784276	52.87147
389755	cg21946631	56710498	56710498	0.7161047	0	1	0.8546822	54.72126
389835	cg21951729	42767403	25663443	0.7858915	0	1	0.6531447	-40.04715
389876	cg2195465(	24766355	24766355	0.7266713	0	1	0.5708987	-45.05479
389909	cg21958394	46665387	46665387	0.9457412	0	1	0.8379295	-66.29672
390076	cg21969792	63779384	44754485	0.7223597	0	1	0.6019791	-29.32832
390160	cg21978135	11782505	11782505	0.868534	0	1	0.7171817	-66.34566
390317	cg21994818	16603301	16603301	0.4639165	0	1	0.6140305	36.81647
390329	cg21995652	63727351	63727351	0.3217861	0	1	0.4835386	42.53592
390338	cg21996068	29712302	29712302	0.5826002	0	1	0.4627176	-24.68662
390366	cg21997727	55681501	70735507	0.2737002	0	1	0.4685799	61.99565
390470	cg2200721(	13793461	13793461	0.606807	0	1	0.7990023	75.39091
390581	cg22014112	31741504	18749447	0.2595527	0	1	0.3877769	30.96108
390650	cg22018083	18651414	18651414	0.4416873	0	1	0.2700908	-49.75398
390778	cg22026089	60740480	60740480	0.5244514	0	1	0.6806412	42.03872
390829	cg22029015	40750308	10720446	0.6523764	0	1	0.8303252	73.22053
390845	cg22029879	29656450	29656450	0.6645311	0	1	0.8044388	46.04729
390883	cg2203202(	62667465	62667465	0.7347248	0	1	0.8477833	39.12802
390972	cg22037249	44755506	44755506	0.2072686	0	1	0.5461969	374.3439
391015	cg22040202	27756392	27756392	0.4925843	0	1	0.328835	-43.24052
391210	cg2205163(	32731457	32731457	0.7864084	0	1	0.9153653	67.68819
391264	cg22054912	18731335	18731335	0.3265512	0	1	0.5122464	54.26177
391390	cg22064048	72703306	72703306	0.3198975	0	1	0.4214014	19.60261
391447	cg2206840(	66663338	27656445	0.4065561	0	1	0.2292086	-56.0711
391472	cg22069441	74738411	27704357	0.3107005	0	1	0.1933163	-30.91493
391551	cg22076422	61764309	61764309	0.5305853	0	1	0.6905497	44.38602
391554	cg22076614	12701365	12701365	0.6117725	0	1	0.879005	374.3439
391569	cg22077313	39626373	39626373	0.3504719	0	1	0.4676178	24.06195
391585	cg22078459	53692421	53692421	0.4650132	0	1	0.5926638	27.55758
391663	cg2208280(	11799427	20794463	0.6037244	0	1	0.5027791	-18.88571
391700	cg22085055	24730483	57749407	0.0076213	0	1		-4.34E-09
391718	cg22087352	12768443	12768443		0	1		-4.34E-09
391813	cg22094923	37642502	37642502	0.8893602	0	1	0.5107641	-301.0478
391818	cg22095263	30706332	30706332	0.4571128	0	1	0.5675039	21.41249

391928	cg2210516	56718419	56718419	0.2230967	0	1	0.3414874	29.18129
391940	cg2210622	37606332	37606332	0.6611301	0	1	0.4385616	-77.5645
392000	cg2211015	56661428	56661428	0.2209299	0	1	0.34209	30.41842
392026	cg2211116	47652314	47652314	0.4971386	0	1	0.5992229	19.1666
392031	cg2211169	60693421	60693421	0.4264863	0	1	0.320739	-20.90411
392138	cg2211789	59706422	59706422	0.628781	0	1	0.4436828	-53.93056
392149	cg2211829	15809455	15809455	0.3258458	0	1	0.426147	19.11947
392184	cg2212155	41734419	40791476	0.6478111	0	1	0.4841326	-43.93885
392294	cg2212730	27724371	27724371	0.265956	0	1	0.4196291	41.41535
392499	cg2214086	53785452	53785452	0.5868223	0	1	0.7028897	26.66371
392516	cg2214214	37691483	37691483	0.5221379	0	1	0.6725561	39.01986
392530	cg2214328	74665360	74665360	0.4640823	0	1	0.3406687	-26.41272
392559	cg2214490	73794378	38745448	0.8431126	0	1	0.7407187	-33.05861
392584	cg2214716	47795484	47795484	0.8345392	0	1	0.7282333	-33.71973
392650	cg2215177	33752365	33752365	0.3934784	0	1	0.5128102	24.32918
392657	cg2215222	11755402	12615500	0.540364	0	1	0.3923529	-35.25611
392798	cg2215964	10709400	10709400	0.2923031	0	1	0.4343028	35.00548
392831	cg2216111	38611463	38611463	0.6686098	0	1	0.7772945	28.57095
392890	cg2216400	49680432	49680432	0.497942	0	1	0.6223635	26.9256
393018	cg2217205	43740344	43740344	0.6897797	0	1	0.5417866	-38.86484
393173	cg2218504	38609393	50609306	0.3150868	0	1	0.2106594	-24.65228
393238	cg2218978	50767389	72751456	0.529862	0	1	0.3268077	-63.97519
393240	cg2219002	37667321	37667321	0.5651157	0	1	0.3738992	-56.35646
393351	cg2219885	61682431	12810408	0.9576421	0	1	0.787721	-127.9035
393368	cg2219996	73764363	45746334	0.5801442	0	1	0.4527359	-27.31763
393403	cg2220238	73628497	73628497	0.4234029	0	1	0.5235379	18.145
393424	cg2220382	67639510	67639510		0	1		-4.34E-09
393493	cg2220991	30609421	30609421	0.5738113	0	1	0.6840573	23.80117
393503	cg2221049	24779331	24779331	0.7535822	0	1	0.608466	-42.43679
393563	cg2221480	62789388	62789388	0.4209014	0	1	0.5352613	22.58179
393589	cg2221619	69605422	58784418	0.2528209	0	1	0.3606713	23.73027
393682	cg2222279	47789410	47789410	0.7538859	0	1	0.8809336	54.45669
393686	cg2222311	39794491	39794491	0.6680666	0	1	0.7729544	26.70783
393829	cg2223175	13740491	69793339	0.5695213	0	1	0.6821657	24.56065
393887	cg2223493	32803418	32803418	0.4595907	0	1	0.6059874	35.08157
393908	cg2223606	25762347	25762347	0.0966936	0	1	0.2060309	39.19136
393923	cg2223701	27797434	27797434	0.8677745	0	1	0.6944856	-80.91566
393933	cg2223749	59714316	59714316	0.897593	0	1	0.7241391	-92.75117
393935	cg2223764	15786353	15786353	0.5486476	0	1	0.7086782	45.66673
394017	cg2224285	61748404	61748404	0.8463085	0	1	0.7315392	-39.76161
394022	cg2224310	39666377	19677413	0.7398638	0	1	0.6191919	-30.56381
394089	cg2224774	20605478	20605478	0.2915501	0	1	0.3928439	20.21837
394147	cg2225168	59672310	59672310	0.1642183	0	1	0.2883654	36.66763

394276	cg2226089[illegible]	17786473	17786473	0.5932368	0	1	0.7438729	44.13392
394340	cg2226498[illegible]	15656323	15656323	0.585569	0	1	0.7273147	38.59277
394449	cg2227411[illegible]	33784443	33784443	0.0866306	0	1	0.186678	36.13745
394516	cg2227808[illegible]	56639356	56639356	0.1430186	0	1	0.2461135	29.58649
394567	cg2228208[illegible]	48656396	48656396	0.4592757	0	1	0.5870127	27.515
394680	cg2229014[illegible]	28751506	70754366	0.5060711	0	1	0.6296974	26.80999
394755	cg2229658[illegible]	57770413	57770413	0.5842465	0	1	0.4800978	-19.61664
394844	cg2230451[illegible]	53635488	53635488	0.727563	0	1	0.4516998	-124.5926
394869	cg2230600[illegible]	71772431	71772431	0.107305	0	1	0.2528046	57.38768
394995	cg2231645[illegible]	36810353	36810353	0.6041884	0	1	0.7213249	28.01875
394997	cg2231661[illegible]	34764306	67739463	0.3303131	0	1	0.1289549	-88.11882
395053	cg2232132[illegible]	49710463	49710463	0.3448649	0	1	0.4474706	19.48914
395119	cg2232582[illegible]	43632388	43632388	0.5197949	0	1	0.4018997	-23.79969
395284	cg2233580[illegible]	39743399	34757343	0.294188	0	1	0.4407007	36.82543
395306	cg2233686[illegible]	55637506	55637506	0.417793	0	1	0.7252919	153.5253
395319	cg2233762[illegible]	36714448	36714448	0.4395871	0	1	0.6082395	45.07973
395354	cg2234050[illegible]	12808405	24758434	0.3189943	0	1	0.153841	-59.43993
395365	cg2234131[illegible]	70701466	70701466	0.4163271	0	1	0.1784419	-102.8048
395389	cg2234372[illegible]	26622337	59671385	0.688758	0	1	0.5729918	-25.92671
395519	cg2235277[illegible]	47718389	47718389	0.4095892	0	1	0.2822137	-29.59905
395636	cg2236110[illegible]	63806459	63806459	0.5290551	0	1	0.4176068	-21.63307
395685	cg2236489[illegible]	57682467	57682467	0.7673901	0	1	0.8891818	53.52679
395744	cg2236770[illegible]	65647306	59700369	0.2388867	0	1	0.0894386	-64.46755
395751	cg2236798[illegible]	25645372	25645372	0.2067226	0	1	0.3233875	29.60057
395835	cg2237309[illegible]	35685405	35685405	0.5118611	0	1	0.7477524	95.86665
396134	cg2239004[illegible]	59608335	59608335	0.1960288	0	1	0.3012995	25.90017
396288	cg2240212[illegible]	23779301	34774468	0.2331417	0	1	0.9273968	374.3439
396358	cg2240810[illegible]	60731414	60731414	0.7600778	0	1	0.874113	44.90738
396394	cg2241118[illegible]	47682498	47682498	0.8807091	0	1	0.768483	-45.4156
396488	cg2242051[illegible]	17693448	17693448	0.5207446	0	1	0.6432045	26.74476
396498	cg2242225[illegible]	73660487	73660487	0.5997406	0	1	0.7349595	36.30106
396565	cg2242869[illegible]	51644345	51644345	0.4560167	0	1	0.5787671	25.64179
396685	cg2243740[illegible]	36771482	36771482	0.2126433	0	1	0.330143	29.51863
396747	cg2244261[illegible]	62754307	62754307	0.2092905	0	1	0.355444	42.24211
396758	cg2244321[illegible]	65611468	65611468	0.107955	0	1	0.6172995	374.3439
396849	cg2245040[illegible]	27629332	27629332	0.3507572	0	1	0.5009546	36.66162
396881	cg2245223[illegible]	11725483	42645456	0.6526007	0	1	0.5283438	-27.6778
396936	cg2245566[illegible]	52783348	41720477	0.1430792	0	1	0.0309836	-59.49481
397167	cg2247333[illegible]	27676319	27676319	0.3208358	0	1	0.436088	24.01411
397272	cg2248116[illegible]	66810335	19775311	0.2412609	0	1	0.1343764	-32.2716
397633	cg2250230[illegible]	47810318	47810318	0.4530943	0	1	0.7278968	123.7207
397722	cg2250814[illegible]	59700445	59700445	0.6779606	0	1	0.932094	374.3439
397736	cg2250893[illegible]	73600419	73600419	0.5603319	0	1	0.6805413	27.20263

397746	cg2250911	58605415	63801383	0.5281082	0	1	0.4111552	-23.45507
398085	cg2253023	48703391	48703391	0.5252491	0	1	0.6356975	22.43133
398150	cg2253428	44735506	51738369	0.4006284	0	1	0.2899755	-23.31262
398271	cg2254125	14721442	14721442	0.5544266	0	1	0.4215063	-29.188
398298	cg2254245	17773359	17773359	0.7257101	0	1	0.8930556	86.18604
398315	cg2254337	59706414	59706414	0.3229509	0	1	0.4255982	19.89114
398320	cg2254392	63771360	63771360	0.8821498	0	1	0.6541543	-129.3412
398342	cg2254512	62657346	49710451	0.4686458	0	1	0.6420097	48.38554
398440	cg2255081	72640301	72640301	0.6362981	0	1	0.7520379	29.44688
398572	cg2256179	43675403	43675403	0.6537064	0	1	0.7738301	33.05916
398608	cg2256381	35731370	35731370	0.3734113	0	1	0.5192848	34.50652
398663	cg2256949	61810439	10741321	0.5460708	0	1	0.3929165	-37.46013
398754	cg2257626	46701454	46701454	0.432646	0	1	0.5530805	24.66429
398766	cg2257760	53709439	53709439	0.4109952	0	1	0.288844	-27.39261
398781	cg2257907	55763419	55763419	0.402379	0	1	0.5070975	19.56662
398871	cg2258595	29749339	29749339	0.3614897	0	1	0.2332549	-32.47409
398894	cg2258760	30659497	30659497	0.1385621	0	1	0.2535598	35.51274
398900	cg2258814	12768471	16693306		0	1		-4.34E-09
398920	cg2258972	73648503	73648503	0.5669671	0	1	0.4597178	-20.42045
398923	cg2258982	63618321	63618321	0.3860058	0	1	0.4963299	21.4076
399014	cg2259721	43696442	43696442	0.2803876	0	1	0.3820498	20.66087
399072	cg2260141	54726304	54726304	0.6965978	0	1	0.8031746	30.08775
399091	cg2260326	27673429	27673429	0.6202497	0	1	0.72391	23.23365
399231	cg2261112	23694300	23694300	0.652833	0	1	0.7734557	33.2401
399247	cg2261244	44674486	13747358	0.6181902	0	1	0.721445	22.97352
399369	cg2262068	48767360	48767360	0.8574259	0	1	0.7506029	-37.57136
399416	cg2262289	38644415	38644415	0.7889528	0	1	0.673421	-32.52495
399443	cg2262490	30641306	30641306	0.1148328	0	1	0.270674	61.87379
399481	cg2262702	35617474	36799401	0.1110105	0	1	0.2242578	38.71111
399557	cg2263161	49803433	49803433	0.3084199	0	1	0.4100789	19.90278
399566	cg2263252	35766468	37606499	0.6466745	0	1	0.4914203	-40.03856
399603	cg2263463	18779345	18779345	0.3934869	0	1	0.4972397	19.31973
399617	cg2263549	63726451	34610498	0.7026443	0	1	0.8197427	36.85211
399650	cg2263794	67726462	67726462	0.5657488	0	1	0.6947085	31.17534
399682	cg2264081	20771450	20771450	0.1485164	0	1	0.2487768	27.80837
399780	cg2264768	16783379	16783379	0.8225724	0	1	0.9338099	62.89035
399851	cg2265314	62627395	48804333	0.2485758	0	1	0.3742824	30.54918
399860	cg2265395	39672463	52765451	0.7602082	0	1	0.5820993	-60.39845
399912	cg2265705	31653389	31653389	0.5225477	0	1	0.3572705	-43.29248
399945	cg2265926	64795501	35739490	0.6143383	0	1	0.5000042	-23.27489
399979	cg2266093	47756478	13763395	0.5795959	0	1	0.4755512	-19.53982
400028	cg2266429	29680347	29680347	0.2046121	0	1	0.4982116	144.7747
400139	cg2267260	11633459	57720491	0.2263758	0	1	0.1176263	-35.4159

400154	cg2267354[illegible]	55723396	55723396	0.8467442	0	1	0.6182954	-114.8167
400208	cg2267607[illegible]	36774424	49760474	0.3224717	0	1	0.2145406	-25.71458
400254	cg2267867[illegible]	32674499	46613423	0.4188183	0	1	0.5307453	21.78675
400257	cg2267873[illegible]	10612410	10612410	0.3043341	0	1	0.4057745	19.93251
400264	cg2267901[illegible]	27807312	27807312	0.4432468	0	1	0.3352423	-21.32926
400310	cg2268170[illegible]	46668356	46668356	0.5580103	0	1	0.4579555	-18.19724
400317	cg2268194[illegible]	49730486	49730486	0.4705071	0	1	0.2528313	-77.62176
400324	cg2268220[illegible]	58691497	58691497	0.5287536	0	1	0.632605	20.2595
400344	cg2268301[illegible]	57666362	57666362	0.4279085	0	1	0.5334265	19.76275
400347	cg2268303[illegible]	19808382	19808382	0.5020616	0	1	0.3786345	-25.86367
400365	cg2268437[illegible]	13609479	44713442	0.365845	0	1	0.4762663	21.63443
400598	cg2269654[illegible]	17676435	17676435	0.7069091	0	1	0.5433004	-47.27618
400657	cg2269931[illegible]	66748409	66748409	0.2602775	0	1	0.4256188	47.30188
400664	cg2269962[illegible]	49665394	49665394	0.2546527	0	1	0.3817325	30.76368
400669	cg2270001[illegible]	22767393	52774327	0.6341645	0	1	0.4727607	-42.38141
400688	cg2270079[illegible]	67694374	53774307	0.3516768	0	1	0.1982048	-46.92708
400689	cg2270082[illegible]	22648488	22648488	0.2089493	0	1	0.3172039	26.18103
400769	cg2270595[illegible]	53786440	53786440	0.5595616	0	1	0.4451371	-22.66852
400830	cg2270896[illegible]	54703456	70629384	0.3889982	0	1	0.5033663	22.69427
400873	cg2271167[illegible]	55697508	55697508	0.2849548	0	1	0.3999608	24.97387
400946	cg2271626[illegible]	70793361	70793361	0.4887291	0	1	0.589566	18.67958
400967	cg2271723[illegible]	23800358	23800358	0.5190328	0	1	0.9133399	374.3439
400970	cg2271747[illegible]	45700416	37802319	0.9718113	0	1	0.7891722	-153.2203
401126	cg2272743[illegible]	16809362	16809362	0.8069081	0	1	0.9092528	47.17641
401167	cg2273002[illegible]	12650394	12650394	0.54175	0	1	0.6517867	22.69731
401169	cg2273004[illegible]	73676317	15721361	0.076341	0	1	0.2704524	100.6052
401214	cg2273212[illegible]	27620327	27620327	0.2127259	0	1	0.3204437	25.7449
401343	cg2274159[illegible]	26754377	26754377	0.088413	0	1	0.3251755	129.965
401376	cg2274510[illegible]	66648508	66648508	0.4698934	0	1	0.3510989	-24.61278
401387	cg2274562[illegible]	70606419	70606419	0.260825	0	1	0.3657812	22.41577
401405	cg2274686[illegible]	60650501	60650501	0.6269367	0	1	0.7442753	29.53448
401416	cg2274765[illegible]	63759321	63759321	0.2101037	0	1	0.091838	-45.05052
401578	cg2275926[illegible]	31682388	31682388	0.4565946	0	1	0.5610119	19.51341
401632	cg2276359[illegible]	41798424	41798424	0.3467341	0	1	0.4745324	27.87757
401652	cg2276459[illegible]	19801466	58622419	0.0170441	0	1	0.2887755	374.3439
401736	cg2277175[illegible]	63691435	63691435	0.1494712	0	1	0.4610172	374.3439
401786	cg2277560[illegible]	69745429	69745429	0.4759787	0	1	0.6214755	35.05423
401822	cg2277756[illegible]	14786382	14786382	0.5782979	0	1	0.8557957	374.3439
401879	cg2278176[illegible]	27713505	27713505	0.5736598	0	1	0.2815925	-133.3938
401951	cg2278647[illegible]	13717427	13717427	0.7077832	0	1	0.852942	58.25275
401958	cg2278674[illegible]	49745403	49745403	0.216578	0	1	0.3429044	32.83078
401996	cg2278931[illegible]	70685378	22676408	0.8090419	0	1	0.6603194	-51.44699
402005	cg2278998[illegible]	31797416	59652457	0.5732009	0	1	0.4693339	-19.43068

402016	cg2279087(	46786313	46786313	0.7351894	0	1	0.8467144	38.15707
402040	cg2279198?	67764353	57737497	0.611158	0	1	0.4906131	-25.3291
402068	cg2279306:	57707436	15678323	0.3210981	0	1	0.2187277	-23.453
402112	cg2279534:	41687317	15801383	0.9168879	0	1	0.7973307	-61.58786
402267	cg2280189(	31802445	31802445	0.6071253	0	1	0.4170686	-56.02983
402273	cg2280201‹	74740492	74740492	0.8197045	0	1	0.9310946	61.74411
402280	cg2280229(	17684406	17684406	0.6024109	0	1	0.4934837	-21.32358
402324	cg2280452{	39610321	39610321	0.3889289	0	1	0.5013361	22.05514
402330	cg2280480:	24717506	24717506	0.8104162	0	1	0.6323479	-68.80047
402470	cg2281261‹	12751429	12751429	0.4576235	0	1	0.5791595	25.22183
402498	cg2281402:	27604327	36741365	0.7947008	0	1	0.8997124	46.03905
402543	cg2281646:	30630456	13682306	0.9749551	0	1	0.8593658	-93.9101
402718	cg2282657?	48779380	19609302	0.2441453	0	1	0.0917659	-65.68613
402765	cg2282918:	30777395	30777395	0.5691448	0	1	0.4332213	-30.40987
402833	cg2283347{	69655472	69655472	0.5026202	0	1	0.630422	28.34856
402842	cg2283414?	39767488	39767488	0.2639292	0	1	0.4363217	50.56999
402850	cg2283454:	73755435	73755435	0.2710541	0	1	0.392123	27.69173
402860	cg2283563(	30777385	30777385	0.6449029	0	1	0.5208532	-27.36785
403112	cg2285194‹	27650394	27650394	0.7048311	0	1	0.5669025	-35.40279
403196	cg2285632‹	60663333	18745388	0.7359005	0	1	0.6352671	-22.7802
403304	cg2286235?	13670335	13670335	0.3340984	0	1	0.2268503	-24.76525
403331	cg2286370(	14810313	36777310	0.6281462	0	1	0.4360677	-57.68891
403839	cg2289106(	67760498	37743313	0.3429458	0	1	0.2295505	-26.88062
403973	cg2290047(	47648350	47648350	0.5262498	0	1	0.636961	22.54665
404050	cg2290440(	63689409	63689409	0.4986194	0	1	0.6411074	34.37992
404065	cg2290551]	10777300	62767353	0.4578744	0	1	0.1804948	-134.9608
404095	cg2290729:	25656423	25656423	0.339082	0	1	0.4773924	32.03207
404116	cg2290908:	31672308	31672308	0.6356965	0	1	0.51118	-27.28237
404227	cg2291734(	33807428	33807428	0.0678821	0	1	0.2285792	79.27639
404236	cg2291804:	56689381	56689381	0.0737593	0	1	0.1790572	41.69405
404358	cg2292686(	21730447	21730447	0.034012	0	1	0.2559091	146.9932
404425	cg2293115]	44794321	44794321	0.7672557	0	1	0.8925456	56.79327
404432	cg2293145:	33604408	33604408	0.3262543	0	1	0.4328881	21.069
404477	cg2293380(	53623508	53623508	0.3018807	0	1	0.586898	125.7221
404506	cg2293542:	58611458	58611458	0.6777628	0	1	0.7924351	32.50271
404521	cg2293625:	29744499	29744499	0.2011374	0	1	0.321553	31.54587
404529	cg2293735‹	10764462	10764462	0.4331809	0	1	0.5434399	21.25992
404567	cg2293970(	11632479	11632479	0.6508896	0	1	0.759137	27.00324
404656	cg2294480:	24604355	24604355	0.2227187	0	1	0.3498428	32.72346
404748	cg2294976?	42718400	42718400	0.5336661	0	1	0.4190356	-22.67155
404766	cg2295083]	38624309	38624309	0.7962929	0	1	0.91638	61.75909
404812	cg2295368:	59715476	59715476	0.4974471	0	1	0.6157031	24.62435
404865	cg2295663:	25629423	41654421	0.7643586	0	1	0.9308435	105.0237

405086	cg2296910{	51682366	51682366	0.5488732	0	1	0.6556907	21.74841
405099	cg2297000:	18714369	18714369	0.4216271	0	1	0.1991566	-87.75455
405112	cg2297121(	27643360	27643360	0.1552408	0	1	0.0475179	-50.36032
405136	cg2297280(	46726489	43680309	0.3594392	0	1	0.5914609	81.9668
405267	cg2297978:	67806326	24759318	0.6952532	0	1	0.5799178	-26.04873
405350	cg2298458'	37602338	29733506	0.7487687	0	1	0.9055986	83.85537
405383	cg2298666:	32810386	62806350	0.4182539	0	1	0.2389602	-56.18943
405460	cg2299132(	23775482	19733510	0.6932528	0	1	0.5928341	-20.85518
405483	cg2299249'	46762356	46762356	0.5851631	0	1	0.4726355	-22.25858
405489	cg2299273(	48680468	23639303	0.2679135	0	1	0.1638046	-27.96982
405494	cg2299319:	44704364	22743479	0.3569202	0	1	0.1993746	-48.85432
405561	cg2299676{	43608419	43608419	0.2216651	0	1	0.8172901	374.3439
405609	cg2299962(	31727365	47793449	0.6551377	0	1	0.7575078	24.66117
405692	cg2300510:	68655481	54730315	0.8904874	0	1	0.5312217	-274.8357
405737	cg2300856:	41675351	38711325	0.27444	0	1	0.1715488	-26.81053
405752	cg2300909‹	59611434	51731434	0.6217211	0	1	0.8494292	115.1996
405788	cg2301062‹	35619380	35619380	0.880191	0	1	0.7744509	-41.53605
405810	cg2301231(	50664382	30761362	0.1911133	0	1	0.3349106	42.9482
405818	cg2301265‹	12657463	12657463	0.6693857	0	1	0.8574325	87.88558
405934	cg2301958‹	31615444	31615444	0.5326928	0	1	0.2966114	-86.64081
405975	cg2302205:	67711384	67711384	0.6779407	0	1	0.8653693	90.17036
406084	cg2302874(	49756401	49756401	0.3752483	0	1	0.5624151	54.13054
406123	cg2303020(	37721486	37721486	0.2462409	0	1	0.3592704	25.8714
406203	cg2303492:	43682329	43682329	0.2753926	0	1	0.1595287	-33.42877
406253	cg2303726:	36602441	36602441	0.8864704	0	1	0.7753057	-46.22697
406392	cg2304527'	61622368	61622368	0.7498	0	1	0.8557931	36.85576
406526	cg2305192(	39676303	39676303	0.4899846	0	1	0.6955611	69.34681
406561	cg2305350(	68659434	44808484	0.3923484	0	1	0.6660577	116.3678
406676	cg2305831‹	36693466	67652467	0.4881247	0	1	0.7964069	374.3439
406732	cg2306111‹	46647510	28752344	0.5608889	0	1	0.4581231	-19.01504
406745	cg2306179:	73733463	73733463	0.4400916	0	1	0.6590264	75.08199
406816	cg2306628(	72707452	57736329	0.5146871	0	1	0.7234434	74.09391
406866	cg2306904(	53733425	53733425	0.5938032	0	1	0.7180648	30.59912
406949	cg2307493:	28749411	28749411	0.4435288	0	1	0.2792615	-45.59734
406972	cg2307561l	56645475	56645475	0.3138284	0	1	0.4198433	21.14365
407022	cg2307906:	39756485	39756485	0.4045087	0	1	0.553867	35.80553
407120	cg2308450(	33767397	33767397	0.3060832	0	1	0.4318568	28.14348
407129	cg2308514:	27684492	27684492	0.6816314	0	1	0.5505707	-31.37639
407220	cg2309020'	47796459	47796459	0.0375157	0	1	0.1466564	54.80779
407389	cg2309806{	12711505	12711505	0.4073636	0	1	0.5598912	37.1791
407401	cg2309878‹	23786356	23786356	0.918372	0	1	0.8015773	-60.22606
407514	cg2310604:	27662435	27662435	0.6790217	0	1	0.5595299	-26.87975
407570	cg2310986'	68739412	57770329	0.7792131	0	1	0.6738903	-27.37338

407638	cg2311461(	67614345	58772358	0.6297095	0	1	0.5170289	-23.03304
407672	cg2311738(	39656417	39656417	0.6692849	0	1	0.5430461	-28.99939
407766	cg2312325(	26689339	64768303	0.4219213	0	1	0.2797489	-35.638
407832	cg2312851(	34773406	34773406	0.7029406	0	1	0.4295192	-119.3035
407866	cg2313083:	41611411	41611411	0.6045898	0	1	0.7320585	32.79783
407916	cg2313674:	17660464	66795481	0.8493093	0	1	0.7236713	-46.14299
407977	cg23141851	72804303	72804303	0.7146705	0	1	0.6086539	-23.61623
408189	cg2315650(	69727490	69727490	0.2838677	0	1	0.3843736	20.18928
408213	cg2315818(	32629444	12713425	0.0764463	0	1	0.1889563	45.24425
408243	cg2315997(	72704311	72704311	0.0505989	0	1	0.6187553	374.3439
408284	cg2316259(	59687470	59687470	0.7915626	0	1	0.8968646	45.42547
408379	cg2316742:	59770497	59770497	0.7969657	0	1	0.6902326	-29.5336
408398	cg2316852(	67794317	67794317	0.9243932	0	1	0.7882752	-77.18129
408444	cg2317110:	19799376	19799376	0.3715729	0	1	0.4759429	19.6833
408477	cg2317340:	61624403	63721342	0.5890236	0	1	0.4857697	-19.3964
408484	cg2317368(	18794419	62765313	0.436703	0	1	0.3113373	-27.83432
408510	cg2317557:	51749314	51749314	0.4114062	0	1	0.5246253	22.21699
408530	cg2317661:	59602301	59602301	0.2649701	0	1	0.3748109	23.9196
408621	cg2318284(	34670367	65739445	0.5966171	0	1	0.4664963	-28.52278
408682	cg2318731(	59663416	59663416	0.5200337	0	1	0.3754832	-33.9397
408699	cg2318868‹	63604477	25783483	0.4166218	0	1	0.2532879	-46.81284
408819	cg2319576:	44748359	44748359	0.473251	0	1	0.7668971	148.0846
408955	cg2320272:	12694323	12694323	0.4498016	0	1	0.5570878	20.37318
409097	cg2320935:	59758416	59758416	0.8164232	0	1	0.936599	71.14987
409106	cg23209941	37721439	37721439	0.2608064	0	1	0.378354	26.84088
409126	cg23210521	49695350	49695350	0.3616306	0	1	0.4948979	29.66535
409156	cg2321244:	74799377	74799377	0.270762	0	1	0.3915617	27.60443
409197	cg2321443:	57770404	57770404	0.7806518	0	1	0.9075493	63.07859
409203	cg2321476(	62803315	22614428	0.4572536	0	1	0.349868	-20.88827
409265	cg2321835‹	60646428	18724379	0.3631931	0	1	0.219869	-39.99086
409266	cg2321836:	70727307	70727307	0.2907658	0	1	0.455511	45.21601
409272	cg2321855(	45740474	35708392	0.2045929	0	1	0.0318337	-105.9518
409280	cg2321895:	58717327	58717327	0.3110724	0	1	0.4602566	37.31065
409285	cg2321925:	26748333	26748333	0.470418	0	1	0.5896092	24.53152
409392	cg2322435(	55717393	30647326	0.3586785	0	1	0.2361708	-29.99686
409432	cg2322691‹	23658400	43769447	0.4413859	0	1	0.2995992	-34.62477
409465	cg2322852(	12778419	49678399	0.2993798	0	1	0.1529483	-49.21822
409502	cg2323083‹	54682481	54682481	0.0991077	0	1	0.2499453	62.69104
409516	cg23231631	39659445	39659445	0.8383591	0	1	0.6487145	-83.1618
409551	cg2323341(	15758300	15758300	0.2990346	0	1	0.0828666	-115.2362
409658	cg2323879:	13741416	64642364	0.423135	0	1	0.3226537	-19.23573
409730	cg2324214:	36799442	62803492	0.051834	0	1		-4.34E-09
409795	cg2324491(	70620478	16603329	0.4106607	0	1	0.2956852	-24.60962

409860	cg2324794[illegible]	46605301	54684461	0.6336008	0	1	0.495542	-32.36415
409871	cg2324842[illegible]	33740452	33740452	0.7350959	0	1	0.8473252	38.62151
409916	cg2325057[illegible]	40613384	62690339	0.3800264	0	1	0.2787434	-20.59078
410036	cg2325648[illegible]	71759373	63640366	0.6790089	0	1	0.7960313	33.92554
410094	cg2325954[illegible]	29766366	29766366	0.6705192	0	1	0.5430772	-29.50326
410134	cg2326117[illegible]	18708502	18708502	0.3641255	0	1	0.4759429	22.10423
410252	cg2326863[illegible]	63735467	63735467	0.147345	0	1	0.2605409	33.59543
410483	cg2328152[illegible]	59606390	43696426		0	1		-4.34E-09
410615	cg2328883[illegible]	68682381	68682381	0.5050247	0	1	0.6559646	38.50173
410619	cg2328902[illegible]	58802339	58802339	0.3187782	0	1	0.2019565	-30.01216
410630	cg2328985[illegible]	23752376	23752376	0.6625121	0	1	0.5537215	-22.58972
410639	cg2329031[illegible]	48724355	48724355	0.2716139	0	1	0.4337552	45.02586
410730	cg2329679[illegible]	72682370	72682370	0.7161036	0	1	0.9392219	374.3439
410750	cg2329886[illegible]	11700305	34632345	0.7162685	0	1	0.8465955	48.20394
410767	cg2329991[illegible]	19700344	20734385	0.5667751	0	1	0.2778776	-130.707
410817	cg2330274[illegible]	44725301	44725301	0.4619423	0	1	0.6046678	33.54959
410823	cg2330299[illegible]	10626312	10626312	0.4840671	0	1	0.5920718	20.89471
410853	cg2330512[illegible]	31657469	23720345	0.5801985	0	1	0.4418301	-31.4581
410863	cg2330589[illegible]	64730380	73795306	0.3881381	0	1	0.6078414	73.75003
411252	cg2333143[illegible]	24708349	24708349	0.2624297	0	1	0.6023744	374.3439
411288	cg2333349[illegible]	58633483	58633483	0.2113734	0	1	0.3999341	64.36359
411336	cg2333648[illegible]	21771471	21771471	0.5947858	0	1	0.48952	-20.07709
411362	cg2333765[illegible]	59667438	59667438	0.6248285	0	1	0.5221506	-19.7465
411371	cg2333838[illegible]	49774469	49774469	0.7878324	0	1	0.9441579	105.3765
411498	cg2334550[illegible]	42759326	42759326	0.3213141	0	1	0.4921148	46.84631
411570	cg2334897[illegible]	31695334	42783446	0.816954	0	1	0.6963251	-38.17688
411619	cg2335170[illegible]	21606469	21606469	0.5823336	0	1	0.6937031	24.5699
411632	cg2335224[illegible]	44763338	73707305	0.7036446	0	1	0.885105	93.36764
411818	cg2336404[illegible]	42654436	56704416	0.7802071	0	1	0.6439341	-41.06988
411937	cg2337147[illegible]	46780375	46780375	0.5006839	0	1	0.6572651	41.06365
411940	cg2337158[illegible]	61718377	61718377	0.339848	0	1	0.1392402	-84.93283
412033	cg2337655[illegible]	41759380	41759380	0.3261724	0	1	0.2012501	-33.42837
412200	cg2339011[illegible]	14778329	13618360	0.743937	0	1	0.6089664	-36.90956
412232	cg2339238[illegible]	29658437	29658437	0.042246	0	1	0.6463042	374.3439
412350	cg2340044[illegible]	53742492	53742492	0.2395868	0	1	0.3570669	27.86403
412376	cg2340179[illegible]	41668374	31702423	0.3483804	0	1	0.4504424	19.27205
412380	cg2340230[illegible]	44670458	44670458	0.6762795	0	1	0.8232073	52.94579
412426	cg2340473[illegible]	33614370	33614370	0.5486036	0	1	0.7836565	102.0218
412597	cg2341486[illegible]	60804361	49684479	0.7740956	0	1	0.6596086	-30.68946
412647	cg2341807[illegible]	63758467	63758467	0.1178929	0	1	0.2743414	61.53361
412667	cg2341896[illegible]	22654362	22654362	0.2532986	0	1	0.3812241	31.16965
412699	cg2342139[illegible]	14675470	14675470	0.5209352	0	1	0.7834492	123.9444
412704	cg2342189[illegible]	49705387	49705387	0.5168497	0	1	0.8257315	374.3439

412817	cg23429749	52632490	52632490	0.2909951	0	1	0.4290864	33.45576
412950	cg23438516	50695345	50695345	0.0628152	0	1	0.531554	374.3439
412974	cg23440389	18738325	18738325	0.8276032	0	1	0.9318193	56.75747
412987	cg23441248	59728457	59728457	0.2783223	0	1	0.4389344	43.9147
412999	cg23442198	23739407	62718433	0.7770117	0	1	0.6602638	-31.90382
413278	cg23462305	74612343	74612343	0.5395398	0	1	0.6469489	21.71198
413305	cg23463680	22625473	22625473	0.828022	0	1	0.6100127	-100.5683
413327	cg23464743	51804485	51804485	0.4751216	0	1	0.318157	-40.47815
413519	cg23476877	66602389	66725344	0.2070992	0	1	0.0527307	-80.88664
413583	cg23481024	16732386	16732386	0.5445687	0	1	0.6568854	23.60398
413597	cg23481862	74770344	74770344	0.3704439	0	1	0.4854752	23.08103
413737	cg23490161	59606495	34632365	0.3573621	0	1	0.173942	-66.74618
413834	cg23496178	69629438	69629438	0.841947	0	1	0.5855051	-136.6214
413853	cg23497306	38764414	53764352	0.5745441	0	1	0.4595755	-22.95312
413858	cg23497569	45679428	45679428	0.2303428	0	1	0.4089997	56.66803
413921	cg23501005	20681329	20681329	0.7005769	0	1	0.8073478	30.61017
413991	cg23505044	20795336	16688394	0.550159	0	1	0.6538691	20.70466
414035	cg23508201	50687494	50687494	0.3462586	0	1	0.466013	25.02528
414108	cg23513018	42775378	42775378	0.5132069	0	1	0.7345982	83.73095
414161	cg23515496	63698447	63698447	0.1252269	0	1	0.2870123	63.11938
414198	cg23517115	25611479	25611479	0.77143	0	1	0.5657279	-78.96844
414215	cg23517941	62643304	23765336	0.6057912	0	1	0.3989413	-65.66019
414264	cg23521140	52617473	47673385	0.2708308	0	1	0.3918105	27.66814
414308	cg23523359	40683383	40683383	0.960352	0	1	0.6430051	-279.8178
414652	cg23543481	13689325	13689325	0.1351722	0	1	0.2398814	31.18806
414762	cg23549902	36622495	65717441	0.4193697	0	1	0.2819422	-33.57387
414772	cg23550704	34740420	34740420	0.222442	0	1	0.3290791	24.78344
414784	cg23551722	73611464	73611464	0.6930503	0	1	0.5539377	-35.2243
414928	cg23564471	57798316	57798316	0.5426093	0	1	0.9224377	374.3439
414963	cg23567120	36634317	36634317	0.7653452	0	1	0.6467417	-31.66287
415112	cg23579537	26799415	26799415	0.4150767	0	1	0.6074977	57.33886
415145	cg23583501	31753327	31753327	0.7955022	0	1	0.9160056	61.90332
415181	cg23586272	54606442	54606442		0	1		-4.34E-09
415209	cg23588049	51692442	51692442	0.2394031	0	1	0.1272278	-35.69553
415251	cg23590389	65610329	52657479	0.3177719	0	1	0.6174315	139.2117
415267	cg23591463	18644450	18644450	0.3924322	0	1	0.2250394	-51.2225
415270	cg23591609	65720370	65720370	0.667514	0	1	0.7818643	31.33038
415317	cg23595304	11609369	11609369	0.9154767	0	1	0.7988142	-58.99099
415329	cg23596123	24660376	24660376	0.6632485	0	1	0.5058149	-41.71988
415479	cg23603995	14792375	14792375	0.604601	0	1	0.7064872	21.84562
415565	cg23610373	27760360	14661413	0.2687388	0	1	0.1108674	-64.07973
415583	cg23611697	50719487	50719487	0.448086	0	1	0.5692701	25.01318
415644	cg23616046	53728375	53728375	0.4272054	0	1	0.323627	-20.16509

415757	cg23622369	15693488	67650322	0.1325192	0	1	0.0260707	-57.47629
415908	cg23630179	14642480	14642480	0.7870033	0	1	0.9025965	53.6888
415931	cg23631321	50666408	50666408	0.6696314	0	1	0.5695119	-20.00238
415994	cg23634348	45738341	24724448	0.6453202	0	1	0.5269899	-25.34092
416038	cg23636571	41790318	41790318	0.4594063	0	1	0.6657149	67.61595
416064	cg23638042	54804489	54804489	0.7037724	0	1	0.5820832	-28.81219
416077	cg23638640	73647450	51656335	0.8973888	0	1	0.7908015	-46.36449
416155	cg23642270	48642510	48642510	0.6744662	0	1	0.7800616	27.55196
416272	cg23649088	27711488	29791450	0.34688	0	1	0.54238	59.08913
416394	cg23656083	49691371	66651389	0.8112246	0	1	0.7084604	-29.2816
416457	cg23659056	48605322	48605322	0.5029818	0	1	0.6192636	24.02159
416546	cg23664385	50730307	50730307	0.9573924	0	1		-4.34E-09
416553	cg23664775	29803410	29803410		0	1		-4.34E-09
416678	cg23670630	39727415	71635485	0.5609783	0	1	0.4444904	-23.3605
416725	cg23673397	56774429	56774429	0.8180048	0	1	0.9220538	52.70138
416842	cg23679344	31602493	31602493	0.7013369	0	1	0.8058393	29.46915
416843	cg23679376	57767364	57767364	0.625455	0	1	0.7321705	24.74896
416898	cg23681745	65620344	25792325	0.7253829	0	1	0.6066417	-28.87078
416899	cg23681866	25694317	25694317	0.4345615	0	1	0.3105775	-27.35207
416919	cg23683010	66739327	21636467	0.5742405	0	1	0.4595079	-22.87257
416934	cg23683800	72631320	72631320	0.4428604	0	1	0.6247308	52.10767
417014	cg23687434	65801402	65801402	0.352777	0	1	0.5978715	91.48782
417097	cg23691410	68601485	68601485	0.7826467	0	1	0.6633879	-33.52665
417151	cg23694490	15604455	15604455	0.285001	0	1	0.4764034	59.29432
417226	cg23698271	70810465	70810465	0.7830689	0	1	0.5135919	-129.8387
417341	cg23705155	51728396	51728396	0.1811913	0	1	0.2960157	30.87174
417439	cg23712018	41796445	17703398	0.0164757	0	1	0.1349236	71.22602
417479	cg23713909	53693410	53693410	0.7625609	0	1	0.6134407	-45.31707
417680	cg23727079	29693481	29693481	0.820823	0	1	0.716691	-30.95638
417737	cg23730606	11773436	10627324	0.2622694	0	1	0.3708334	23.58615
417784	cg23732629	27652365	27652365	0.5906639	0	1	0.4829431	-20.78882
417801	cg23733394	35610394	35610394	0.5356974	0	1	0.3912756	-33.76778
417856	cg23737190	63690442	30723353	0.6157725	0	1	0.5061908	-21.7474
417933	cg23742233	56640382	56640382	0.1372763	0	1	0.2752788	47.24438
418137	cg23758822	64672430	66743443	0.3354949	0	1	0.4733751	31.94526
418286	cg23772226	52666371	52666371	0.3395952	0	1	0.4469625	21.04916
418526	cg23793213	10789310	53791392	0.3416052	0	1	0.5092372	44.72596
418528	cg23793336	67739465	67739465	0.3071046	0	1	0.4494537	34.57897
418771	cg23812489	14714457	14714457	0.9111463	0	1	0.6210684	-206.3551
418789	cg23813394	59678432	59678432	0.3485412	0	1	0.1986297	-45.11705
418888	cg23819027	70699431	70699431	0.7260419	0	1	0.8538713	48.30541
418999	cg23827950	64759481	60656358	0.3887801	0	1	0.6008584	68.79803
419012	cg23828686	49800321	49800321	0.463702	0	1	0.3295974	-30.61887

419122	cg23835894	64778312	28624444		0	1		-4.34E-09
419123	cg23835923	36600420	36600420	0.6658716	0	1	0.9250589	374.3439
419167	cg23838005	29808339	29808339	0.4761305	0	1	0.5788486	19.13303
419412	cg23853861	46628393	19687406	0.5912781	0	1	0.3899554	-62.18856
419431	cg23855392	19709465	19709465	0.1799533	0	1	0.0629381	-51.57814
419453	cg23856611	37808387	37808387	0.4869511	0	1	0.6567374	47.25306
419555	cg23863670	43604369	43604369	0.3201162	0	1	0.4765087	40.1565
419564	cg23864202	57781427	57781427	0.4023894	0	1	0.5129126	21.37525
419622	cg23869232	28782332	28782332	0.7423873	0	1	0.8824259	63.21717
419684	cg23873996	17694510	17694510	0.5802777	0	1	0.6948656	25.76237
419716	cg23876149	63806393	64756379	0.8326911	0	1	0.6300744	-90.65371
419752	cg23878260	60638333	60638333	0.5145155	0	1	0.6377395	26.86978
419816	cg23881926	30680503	42733395	0.3013908	0	1	0.4022216	19.81602
419914	cg23887839	48673495	48673495	0.7270508	0	1	0.929018	134.1052
420007	cg23892766	48640474	48640474	0.53948	0	1	0.852127	374.3439
420064	cg23895439	67722339	67722339	0.5533514	0	1	0.6648383	23.5648
420133	cg23899527	21750485	21750485	0.8000174	0	1	0.9362794	83.70341
420232	cg23905789	48748494	48748494	0.2643935	0	1	0.6148587	374.3439
420234	cg23905908	35718450	59743342	0.3453174	0	1	0.2328684	-26.34448
420307	cg23910458	12614492	12614492	0.5049515	0	1	0.6154627	22.04139
420393	cg23915527	32806360	32806360	0.6090512	0	1	0.5030289	-20.51043
420438	cg23918315	36794403	36794403	0.4680055	0	1	0.3641157	-19.61871
420555	cg23924370	63705465	47704307	0.5104815	0	1	0.4088333	-18.62009
420593	cg23926439	69633370	54786464	0.798215	0	1	0.6537491	-47.49752
420626	cg23928165	56691490	34772365	0.156641	0	1	0.0561036	-43.25957
420639	cg23928763	30742476	40630333	0.9066724	0	1	0.7885545	-56.74693
420644	cg23929036	13644375	13644375		0	1	0.891229	4.34E-09
420664	cg23930313	52704340	52704340	0.4796336	0	1	0.3644286	-23.21029
420791	cg23937295	72720393	72720393	0.4497683	0	1	0.592779	33.46727
420820	cg23939001	38746305	51725359	0.5325723	0	1	0.3419122	-56.50654
420880	cg23942717	16778433	34674323	0.8781595	0	1	0.7632461	-46.37606
420956	cg23947654	71638364	71638364	0.4277216	0	1	0.7884097	374.3439
420968	cg23948391	44670393	44670393	0.5840508	0	1	0.448614	-30.36117
420974	cg23948811	37602467	74744491	0.4732455	0	1	0.5791942	20.11773
420979	cg23948993	39790306	12633348	0.3772372	0	1	0.2707287	-22.56819
421174	cg23958704	47736360	47736360	0.4527365	0	1	0.6405335	55.8012
421195	cg23959772	61661388	61661388	0.0822329	0	1	0.1997402	46.78356
421293	cg23966361	22690431	22690431	0.7501104	0	1	0.8800197	55.98884
421383	cg23971182	54750474	54750474	0.1739336	0	1	0.2763604	26.43238
421451	cg23974473	63659338	63659338	0.7170482	0	1	0.6019621	-27.01137
421493	cg23976981	29800510	29800510	0.3038432	0	1	0.4697619	45.16967
421572	cg23981702	22734320	22734320	0.5922344	0	1	0.6937067	21.21469
421645	cg23985370	59733300	33766476	0.3354442	0	1	0.2184089	-28.95065

421709	cg23988024	32618471	42732388	0.1469051	0	1	0.3535088	87.21752
421925	cg24000218	65621380	39740461	0.429673	0	1	0.2990466	-30.20393
422093	cg24007926	29756506	46755377	0.3906341	0	1	0.6475239	101.5603
422118	cg24009067	72650436	72650436	0.1005246	0	1	0.208475	37.77154
422191	cg24013589	26810484	26810484	0.3473829	0	1	0.2400745	-24.11786
422206	cg24014558	44688390	44688390	0.0694317	0	1	0.3102201	142.7335
422244	cg24016855	26718464	26718464	0.4052657	0	1	0.6293454	77.12507
422253	cg24018148	73806350	73806350	0.632293	0	1	0.7574753	33.78683
422502	cg24032445	17682431	17682431	0.5762355	0	1	0.7209205	39.42892
422514	cg24033103	32630505	22792481	0.9197221	0	1	0.8153231	-52.07969
422515	cg24033122	13790358	13790358	0.4583613	0	1	0.3366232	-25.90205
422521	cg24033503	12682383	13644361	0.9891426	0	1		-4.34E-09
422596	cg24036791	12614397	47768375	0.5467736	0	1	0.7810282	100.853
422761	cg24046087	53665466	59795326	0.3095572	0	1	0.2082422	-23.66252
422772	cg24046616	72694474	72694474	0.3015756	0	1	0.4262219	27.87151
422846	cg24051234	52757446	52757446	0.6916249	0	1	0.5386493	-41.20709
422854	cg24051749	70679390	70679390	0.6069399	0	1	0.4925652	-23.16082
422957	cg24057558	71674454	71674454	0.7460133	0	1	0.9531683	156.5356
423018	cg24060917	24667399	24667399	0.3963813	0	1	0.2752166	-27.55394
423033	cg24062389	70637368	70637368	0.2002918	0	1	0.5127901	374.3439
423080	cg24065641	25672361	25672361	0.7085683	0	1	0.5552901	-42.45999
423269	cg24076588	31766373	31766373	0.2865832	0	1	0.386935	20.06214
423272	cg24076747	22733497	22733497	0.3093601	0	1	0.4314503	26.70339
423289	cg24077401	42744339	42744339	0.5029694	0	1	0.1725564	-190.7101
423333	cg24080129	67640506	67640506	0.2214338	0	1	0.0598441	-83.1515
423369	cg24082680	51756305	51756305	0.5007183	0	1	0.3449548	-39.19734
423418	cg24084927	47735449	47735449	0.6192105	0	1	0.4643906	-38.98057
423621	cg24097094	63650427	63650427	0.6178524	0	1	0.4676195	-36.95755
423666	cg24100293	62648337	62648337	0.9358381	0	1	0.832675	-57.75484
423668	cg24100357	38660364	59766507	0.6941081	0	1	0.525584	-48.78091
423677	cg24100841	54650349	54650349	0.6373392	0	1	0.7520853	29.05366
423716	cg24103044	31723420	31723420	0.4918921	0	1	0.6332058	33.65831
423880	cg24114014	54677411	59640472	0.6970059	0	1	0.4545008	-94.1768
423901	cg24115019	64600307	64600307	0.3415529	0	1	0.4585597	24.17018
423925	cg24116380	18614375	18614375	0.5249069	0	1	0.6321126	21.30488
423947	cg24117504	56702336	56702336	0.9453554	0	1	0.8442572	-60.76154
424000	cg24120210	27697486	71668435	0.9893587	0	1	0.8749177	-107.6527
424121	cg24126849	70700395	37676493	0.8577062	0	1	0.7364435	-45.41896
424140	cg24127414	42665471	42665471	0.7804796	0	1	0.6352309	-45.50239
424198	cg24130284	41607326	41607326	0.6296816	0	1	0.5144298	-23.89297
424270	cg24134182	47714360	47714360	0.5111234	0	1	0.6307524	25.42071
424385	cg24139874	59650333	59650333	0.5873879	0	1	0.7755133	68.55569
424443	cg24142775	29730323	54661507	0.5343676	0	1	0.3695934	-42.88363

424667	cg24154853	54680357	72777479	0.5448244	0	1	0.7818443	103.1153
424691	cg24156181	35765340	35765340	0.4404821	0	1	0.5556592	22.89771
424752	cg24158878	73610478	73610478	0.5219744	0	1	0.663644	34.87644
424789	cg24160421	41739424	41739424	0.5958748	0	1	0.4645746	-28.95074
424891	cg24166916	13752399	13752399	0.4080535	0	1	0.2029158	-75.63499
424930	cg24169486	47650434	47650434	0.7432355	0	1	0.8713068	52.50742
424941	cg24170085	55767342	20663507	0.4962695	0	1	0.612398	23.84035
425097	cg24181174	60718359	60718359	0.319342	0	1	0.451883	30.29272
425107	cg24181662	72677445	72677445	0.5574719	0	1	0.6776661	27.08251
425142	cg24183504	47684365	47684365	0.1111033	0	1	0.2537913	54.86249
425150	cg24184022	22600456	71690340	0.8802173	0	1	0.7781592	-39.45778
425176	cg24185656	56687360	55750345	0.9004197	0	1	0.7719917	-61.50823
425261	cg24192559	54676328	72666434	0.81131	0	1	0.6804951	-42.41639
425264	cg24192660	40765501	62705329	0.2084554	0	1	0.092632	-43.51542
425375	cg24199384	29724343	29724343	0.5169359	0	1	0.6498379	30.84556
425460	cg24205048	22657328	22657328	0.3776284	0	1	0.4966642	24.35113
425505	cg24208588	52723466	52723466	0.5111631	0	1	0.6561134	35.94062
425528	cg24210810	37769364	37769364	0.2182821	0	1	0.3346363	28.69254
425837	cg24233679	60729308	60729308	0.2161503	0	1	0.3182211	23.48653
425895	cg24238409	13672463	13672463	0.6603431	0	1	0.7826724	34.8882
426221	cg24264239	51699370	51699370	0.9446412	0	1	0.8244122	-75.67595
426235	cg24265722	30724305	30724305	0.9315115	0	1	0.7619342	-108.0203
426415	cg24280832	57670475	16687490	0.7685197	0	1	0.6674572	-24.90934
426463	cg24284460	16800439	16800439	0.1311756	0	1	0.2383453	32.80274
426465	cg24284539	18760487	54751387	0.7874968	0	1	0.5395584	-112.9113
426500	cg24287218	17713396	17713396	0.6871635	0	1	0.574159	-24.87995
426515	cg24289452	30605485	63790501	0.8574162	0	1	0.7372429	-44.75303
426609	cg24296187	21741468	21741468	0.6550065	0	1	0.7622727	26.84556
426658	cg24299087	38674411	38674411	0.3046153	0	1	0.566089	105.3796
426662	cg24299306	44711379	44711379	0.5360461	0	1	0.7170058	57.11234
426745	cg24304617	73697392	73697392	0.3578403	0	1	0.4610003	19.47414
426830	cg24309769	31759311	31759311	0.3887808	0	1	0.9079667	374.3439
426870	cg24311643	56688344	56688344	0.4796391	0	1	0.6021166	25.84916
426889	cg24312520	27670303	27670303	0.5268342	0	1	0.626975	19.01636
427006	cg24320034	59641444	47739385	0.7805722	0	1	0.929999	90.61403
427033	cg24321971	62765508	62765508	0.3575049	0	1	0.5237774	43.75755
427074	cg24324837	15742383	13698310	0.8648215	0	1	0.7338532	-52.62548
427091	cg24325991	31734427	31734427	0.2929767	0	1	0.1793943	-30.50964
427162	cg24330456	40790492	71730422	0.2083076	0	1	0.405611	69.98405
427438	cg24343430	28716473	28716473	0.5702672	0	1	0.6909187	27.80739
427639	cg24355182	71711502	12653509	0.7254062	0	1	0.5582771	-50.58678
427694	cg24359926	20672497	20672497	0.5935822	0	1	0.7808861	68.94727
427710	cg24360871	71656418	71656418	0.351813	0	1	0.4702139	24.46663

427762	cg2436339	31666380	31666380	0.5309336	0	1	0.6692184	33.72146
427764	cg2436362	48790407	48790407	0.5658991	0	1	0.4574936	-20.77397
427839	cg2436784	48762444	37767480	0.7599493	0	1	0.6537801	-26.25868
427841	cg2436795	47744433	47744433	0.2136128	0	1	0.351129	37.89092
428092	cg2438424	27644355	36635317	0.1219311	0	1	0.011783	-67.19733
428209	cg2439293	57755443	57755443	0.765264	0	1	0.8722441	40.50816
428217	cg2439331	64658453	28662378	0.7167686	0	1	0.5729082	-38.75908
428326	cg2439934	68718419	55710367	0.4256148	0	1	0.30268	-27.21068
428452	cg2440624	67648488	67648488	0.667045	0	1	0.7766506	28.89218
428486	cg2440820	44687303	44687303	0.715891	0	1	0.8443062	46.69154
428624	cg2441715	72680508	72680508	0.8393643	0	1	0.4293944	-321.2908
428659	cg2441960	59679345	59679345	0.6717174	0	1	0.7778278	27.57614
428694	cg2442190	43631335	43631335	0.2426685	0	1	0.1134844	-46.72201
428750	cg2442597	73741498	73741498	0.6031618	0	1	0.7087306	23.19223
429001	cg2443968	53698457	53698457	0.5495297	0	1	0.6723212	27.82933
429013	cg2444065	20731321	52792477	0.7968475	0	1	0.6057312	-74.26487
429156	cg2445011	52684506	52684506	0.8696845	0	1	0.6777682	-95.06404
429184	cg2445187	39659345	39659345	0.3125602	0	1	0.6274981	156.5356
429199	cg2445245	14612502	58778375	0.6032935	0	1	0.3779329	-77.43323
429271	cg2445711	23806421	23806421	0.481265	0	1	0.6094553	28.02936
429325	cg2446012	22710467	12605372	0.696664	0	1	0.576318	-27.95672
429343	cg2446130	37723323	37723323	0.5627677	0	1	0.4577534	-19.70538
429398	cg2446450	48775334	48775334	0.1562209	0	1	0.271339	33.45571
429413	cg2446532	60633364	60633364	0.6026046	0	1	0.7723279	57.3549
429494	cg2447046	22782397	22782397	0.5529487	0	1	0.8338605	156.5356
429507	cg2447121	26675310	26675310	0.483148	0	1	0.3211208	-42.68491
429571	cg2447603	58697331	28755447	0.3422766	0	1	0.1681041	-62.23445
429602	cg2447812	64749312	64749312	0.7128827	0	1	0.8746982	75.30563
429626	cg2447938	54699405	54699405	0.6047888	0	1	0.7313121	32.35649
429736	cg2448592	50692366	50692366	0.5761124	0	1	0.1813818	-273.5412
429873	cg2449449	27809338	27809338	0.5116601	0	1	0.6138297	19.403
430089	cg2450622	61722487	69724377	0.2603967	0	1	0.0414393	-139.3636
430115	cg2450776	68607444	68607444	0.3668048	0	1	0.4688478	19.03176
430128	cg2450842	66660332	36656493	0.466139	0	1	0.1356327	-202.9142
430358	cg2452276	34629506	34629506	0.5623847	0	1	0.742954	59.61814
430448	cg2452709	19792331	19792331	0.2685748	0	1	0.1517991	-34.70868
430575	cg2453309	71673405	71673405	0.8008708	0	1	0.6868418	-33.11504
430645	cg2453634	21699463	21699463	0.4941041	0	1	0.6077223	22.92348
430654	cg2453678	11672327	35767456	0.6036297	0	1	0.4579052	-34.75807
430702	cg2453959	41691439	41691439	0.2846917	0	1	0.4050912	26.90575
430736	cg2454155	40673371	40673371	0.2229853	0	1	0.3321646	25.68388
430888	cg2455021	45679347	47717394	0.7230719	0	1	0.505564	-79.68359
430982	cg2455675	33642301	33642301	0.5883245	0	1	0.7263451	36.87562

431108	cg2456769	42736321	42736321	0.0683409	0	1	0.2146657	69.08398
431152	cg2457111	59631496	59631496	0.2487747	0	1	0.1268356	-40.5848
431246	cg2457759	35619362	35619362	0.2311009	0	1	0.4489712	80.11201
431374	cg2458541	12692438	59677393	0.7508775	0	1	0.6433305	-26.17474
431390	cg2458620	21656447	21656447	0.4294519	0	1	0.5528096	25.68353
431406	cg2458718	39810504	39810504	0.2871378	0	1	0.4315884	36.25702
431498	cg2459252	70651325	40737350	0.2118817	0	1	0.1055755	-36.07478
431500	cg2459257	21710401	21710401	0.160239	0	1	0.2603185	26.64286
431527	cg2459391	61624328	61624328	0.4482469	0	1	0.3189949	-29.05633
431540	cg2459463	52750506	52750506	0.7698184	0	1	0.871524	37.47903
431590	cg2459782	35631360	74648484	0.4715577	0	1	0.6364305	44.07395
431631	cg2459970	30746490	30746490	0.7911801	0	1	0.8912351	40.82504
431766	cg2460785	52763510	52626476	0.4355897	0	1	0.3110229	-27.55129
431784	cg2460910	50675301	50675301	0.4757461	0	1	0.605557	28.5557
431851	cg2461395	61737472	61737472	0.4426045	0	1	0.6112996	45.1676
432011	cg2462598	22730469	22730469	0.4839095	0	1	0.58792	19.61342
432057	cg2462923	16694492	16694492	0.6649213	0	1	0.7673293	25.33674
432192	cg2463633	22607313	74769447	0.3506465	0	1	0.6285958	118.6285
432205	cg2463696	10804440	12717471	0.0954677	0	1	0.2237142	49.95558
432209	cg2463704	28656357	39746472	0.1410471	0	1	0.3142425	66.68298
432397	cg2464838	31751309	31751309	0.1146638	0	1	0.2203247	34.34624
432400	cg2464871	71797347	71797347	0.3449329	0	1	0.2397102	-23.40335
432424	cg2465091	65768461	65768461	0.4367266	0	1	0.6131625	49.01543
432446	cg2465261	52635432	74687433	0.8562945	0	1	0.6429561	-106.3579
432495	cg2465570	20762315	20762315	0.2309112	0	1	0.3398248	25.15279
432573	cg2466116	59699382	59699382	0.6584959	0	1	0.7728188	30.51083
432576	cg2466123	30641453	35761407	0.6707745	0	1	0.5408635	-30.46068
432733	cg2467045	28689388	55775453	0.6342115	0	1	0.5329277	-19.50171
432771	cg2467277	55760423	48672464	0.6386265	0	1	0.7412767	23.78658
432801	cg2467450	11728364	29619326	0.6779678	0	1	0.5699834	-22.81538
432933	cg2468063	11688465	71701313	0.4197045	0	1	0.261173	-43.96722
432934	cg2468064	12746311	12746311	0.6632105	0	1	0.5277832	-32.32297
432936	cg2468074	27802424	27802424	0.7371216	0	1	0.9315445	129.2521
433055	cg2468655	68782416	68782416	0.582108	0	1	0.7160385	34.41767
433056	cg2468655	36667388	36667388	0.3039209	0	1	0.4084801	20.92198
433093	cg2468780	48707310	48707310	0.6809006	0	1	0.7919785	30.89849
433129	cg2468902	23719446	23719446	0.4780685	0	1	0.2209961	-110.0103
433150	cg2469009	30685388	12747325	0.2220275	0	1	0.0775884	-64.76555
433176	cg2469116	23766456	54758300	0.5399093	0	1	0.3278257	-69.39436
433178	cg2469133	45715405	45715405	0.6986918	0	1	0.5346751	-46.8768
433231	cg2469355	47721303	47721303	0.2265873	0	1	0.0980939	-49.50785
433260	cg2469483	20694418	20694418	0.2314072	0	1	0.7661498	374.3439
433297	cg2469743	54700308	54700308	0.3292851	0	1	0.1924592	-39.51419

433431	cg2470422(	54634418	54634418	0.7908504	0	1	0.9263266	77.66559
433494	cg2470748(	30779434	29621508	0.3746507	0	1	0.4773461	19.14715
433496	cg2470757:	37737351	22726360	0.6218021	0	1	0.4894086	-29.85883
433525	cg2470900l	60731329	16657340	0.917455	0	1	0.7818339	-73.4894
433752	cg2472105(	28716403	28716403	0.7165061	0	1	0.820792	31.02195
433779	cg2472257.	50656303	50656303	0.3523208	0	1	0.6301333	118.5305
433835	cg2472552:	32785398	32785398	0.2342462	0	1	0.3880309	43.67691
433917	cg2472983(	59719371	59719371	0.3077301	0	1	0.5020243	59.69727
434026	cg2473673(	60699433	60699433	0.3054082	0	1	0.4371571	30.41742
434099	cg2474051:	19624417	74651303	0.9788163	0	1	0.8585284	-102.033
434237	cg2474936:	39680485	18799420	0.7334564	0	1	0.560442	-54.4437
434297	cg2475309(	74793327	74793327	0.456947	0	1	0.5870433	28.38162
434303	cg2475376(	10639318	10639318	0.6748121	0	1	0.5357586	-34.28469
434325	cg2475513.	39620346	39620346	0.3691555	0	1	0.5329384	42.4253
434331	cg2475545(	24624480	55695461	0.4196704	0	1	0.5251786	19.76178
434412	cg2476058l	69733370	69733370	0.0164815	0	1	0.410164	374.3439
434490	cg2476578:	52708465	52708465	0.8270739	0	1	0.9419154	69.80421
434565	cg2476938l	24754359	24754359	0.542365	0	1	0.8297019	374.3439
434762	cg2478197:	32606446	32606446	0.2896307	0	1	0.4175237	29.50483
434809	cg2478617(	41677319	41677319	0.5141038	0	1	0.6577026	35.43709
434836	cg2478759:	42792334	42792334	0.6731968	0	1	0.9461449	374.3439
434899	cg2479121(	29765492	56755477	0.1622688	0	1	0.0242253	-82.42976
434985	cg2479664(	22726355	17683424	0.8846592	0	1	0.7498575	-60.49847
435052	cg2480022(	64678343	64678343	0.9081283	0	1	0.6901755	-134.6751
435209	cg2480926(	50603507	50603507	0.4568333	0	1	0.5902379	29.66272
435231	cg2481073:	41786312	59756367	0.1458601	0	1	0.2562411	32.49406
435339	cg2481926(	15679456	15679456	0.5800502	0	1	0.6881329	23.21397
435355	cg2482067:	50789471	18647489	0.5821205	0	1	0.4769471	-19.90781
435398	cg2482437l	12641307	12641307	0.47652	0	1	0.5889721	22.27764
435400	cg2482447:	46719372	46719372	0.3525546	0	1	0.4548506	19.28344
435418	cg2482533(	24658347	46787365	0.1736389	0	1	0.0199365	-97.60558
435574	cg2483439(	10618304	10618304	0.5193468	0	1	0.6522864	30.94287
435617	cg2483682(	22686440	17725391	0.8236086	0	1	0.4437848	-267.8958
435641	cg2483806:	38702310	31642452	0.8128767	0	1	0.6543378	-57.61354
435648	cg2483831(	27744377	27744377	0.5883106	0	1	0.4765627	-22.04182
435774	cg2484593:	71800302	71800302	0.1016439	0	1	0.2076396	36.5702
435776	cg2484600(	16757458	16757458	0.4368075	0	1	0.6464221	68.5943
435811	cg2484784(	57762353	62768401	0.2855425	0	1	0.0783531	-109.9785
435960	cg2485638:	28806483	28806483	0.3840616	0	1	0.2493312	-34.15719
435966	cg2485667:	25747460	25747460	0.4483499	0	1	0.2753453	-50.13119
436005	cg2485922(	69663383	69663383	0.1509795	0	1	0.2634327	32.84376
436020	cg2485964(	12639454	12639454	0.0526396	0	1	0.2055684	79.86792
436058	cg2486139(	16740353	16740353	0.0756329	0	1	0.2173088	63.56615

436066	cg24861747	28663414	28663414	0.1451371	0	1	0.2477863	29.16836
436139	cg24865482	48699411	48699411	0.798363	0	1	0.9230827	67.9864
436164	cg24866764	29671350	29671350	0.4736431	0	1	0.5880393	22.90262
436176	cg24867279	36727436	28609422	0.4662425	0	1	0.2614207	-68.78515
436237	cg24870391	44690397	44690397	0.374653	0	1	0.5013304	27.06316
436272	cg24872173	59689343	59689343	0.7188997	0	1	0.2796595	-341.8894
436311	cg24873924	29785495	29785495	0.8800977	0	1	0.6997616	-90.4962
436388	cg24877509	52639464	52639464	0.5835879	0	1	0.4653718	-24.12882
436399	cg24877964	66606448	66606448	0.4308363	0	1	0.5387061	20.49697
436498	cg24882709	60703325	60703325	0.603376	0	1	0.7281274	31.42158
436503	cg24883219	44800449	44800449	0.5149643	0	1	0.3464096	-45.05679
436588	cg24886788	14800312	14800312	0.3456224	0	1	0.4503436	20.12426
436604	cg24888257	43667408	38600394	0.7056611	0	1	0.5993813	-23.30392
436688	cg24893378	44810332	44810332	0.1929983	0	1	0.3819018	67.05425
436727	cg24896460	70722452	70722452	0.5736765	0	1	0.7255338	43.09238
436742	cg24897392	59659411	59659411	0.4991262	0	1	0.6322967	30.41993
436779	cg24900563	53796500	53796500	0.5823953	0	1	0.6830264	20.57421
436882	cg24906015	47620370	47620370	0.6206981	0	1	0.5004875	-25.39966
436907	cg24908198	58770325	17807473	0.9068198	0	1	0.7983664	-50.38023
436947	cg24911283	55669478	55669478	0.5995697	0	1	0.7036223	22.45707
436957	cg24911837	36781471	36781471	0.3828944	0	1	0.5257683	33.18923
436962	cg24912023	25801336	25801336	0.5061952	0	1	0.6094078	19.65086
436970	cg24912560	47689501	47689501	0.8588834	0	1	0.7331163	-48.21364
436978	cg24913168	64614493	59706366	0.4293315	0	1	0.5308102	18.54093
437033	cg24917131	33787301	33787301	0.583049	0	1	0.8577305	374.3439
437113	cg24923430	17678303	63754349	0.6499183	0	1	0.466263	-53.9906
437130	cg24923931	38724324	55774308	0.4985497	0	1	0.3510643	-35.51058
437159	cg24925741	20633404	20633404	0.3347109	0	1	0.4400429	20.49731
437170	cg24926185	62631457	62631457	0.4491606	0	1	0.5574181	20.67774
437197	cg24927769	57729426	57729426	0.783623	0	1	0.8967263	50.2573
437315	cg24933157	32625369	32625369	0.3559769	0	1	0.4640142	21.005
437456	cg24940951	38653505	38653505	0.3042475	0	1	0.4057006	19.93859
437468	cg24941469	15772351	15772351	0.1927107	0	1	0.6167701	374.3439
437470	cg24941603	69621483	10765423	0.6156996	0	1	0.7189992	22.87375
437522	cg24946117	46801340	46801340	0.6147243	0	1	0.8984246	374.3439
437554	cg24947637	72609377	19674510	0.5621516	0	1	0.7663849	77.16189
437569	cg24948743	22636366	15613336	0.6269843	0	1	0.5196031	-21.25541
437691	cg24955829	13663506	13663506	0.7568595	0	1	0.5395678	-84.24699
437694	cg24955955	20670468	20670468	0.5432607	0	1	0.7688479	92.08909
437709	cg24957518	67780307	67780307	0.6064682	0	1	0.7834708	63.20399
437723	cg24958687	10629423	10629423	0.5436311	0	1	0.3249099	-73.67802
437745	cg24960960	17612325	37779367	0.2692963	0	1	0.0904147	-84.02534
437748	cg24961286	72637374	72637374	0.6540124	0	1	0.7548794	23.9411

437814	cg2496636[illegible]	49617431	49617431	0.4213721	0	1	0.5745832	37.54813
437843	cg2496886[illegible]	19687446	19687446	0.792824	0	1	0.8957974	43.71581
437853	cg2496949[illegible]	15736439	15736439	0.415989	0	1	0.7593192	374.3439
437926	cg2497472[illegible]	28661329	20728454	0.7063808	0	1	0.5343077	-51.49058
437931	cg2497521[illegible]	18722400	18722400	0.3773249	0	1	0.4810376	19.4287
437983	cg2497916[illegible]	44756319	44756319	0.2549458	0	1	0.4136929	44.48614
438426	cg2500624[illegible]	42722446	55680418	0.5111055	0	1	0.3591882	-37.24884
438454	cg2500772[illegible]	69692414	68723455	0.5060306	0	1	0.3445486	-41.78151
438577	cg2501375[illegible]	25682361	25682361	0.3582745	0	1	0.5403031	51.57389
438587	cg2501422[illegible]	49645350	49645350	0.2501087	0	1	0.3637929	25.92409
438595	cg2501503[illegible]	67632390	67632390	0.5745226	0	1	0.6774984	21.14978
438632	cg2501722[illegible]	29608371	46697347	0.2658209	0	1	0.3953486	31.16314
438640	cg2501762[illegible]	27739443	28777354	0.267693	0	1	0.0373741	-152.283
438646	cg2501797[illegible]	47616304	59810484		0	1	0.0233081	4.34E-09
438849	cg2502699[illegible]	72730386	38693418	0.5268467	0	1	0.3373101	-56.00875
438895	cg2502944[illegible]	37766331	37766331	0.8214604	0	1	0.7206747	-29.53924
438992	cg2503494[illegible]	48777493	48777493	0.4957087	0	1	0.5998652	19.80258
439005	cg2503563[illegible]	61738421	30677325	0.3224285	0	1	0.1483453	-65.74887
439162	cg2504657[illegible]	23646384	23646384	0.2424391	0	1	0.4125378	51.0449
439332	cg2505940[illegible]	42669397	42669397	0.7258658	0	1	0.835012	35.15285
439340	cg2505969[illegible]	61613414	61613414	0.4564468	0	1	0.5829772	27.03048
439421	cg2506513[illegible]	12621490	12621490	0.4352489	0	1	0.5542101	24.16312
439439	cg2506666[illegible]	11755371	38749389	0.8693908	0	1	0.7020925	-77.29882
439701	cg2508536[illegible]	57758371	57758371	0.5348002	0	1	0.3827832	-37.01841
439894	cg2509909[illegible]	19746429	19746429	0.8096299	0	1	0.525535	-150.5824
439966	cg2510341[illegible]	40688342	40688342	0.2883688	0	1	0.4136531	28.56902
439982	cg2510423[illegible]	26625304	26625304	0.3851286	0	1	0.5923899	65.74813
440161	cg2511418[illegible]	24629421	24629421	0.2022981	0	1	0.3034057	23.94188
440308	cg2512356[illegible]	37785319	37785319	0.3688177	0	1	0.4856403	23.69677
440362	cg2512630[illegible]	24616484	24616484	0.5109201	0	1	0.9138074	374.3439
440393	cg2512912[illegible]	27730408	41638407	0.4255294	0	1	0.3248375	-19.25685
440489	cg2513464[illegible]	21646334	21646334	0.7026598	0	1	0.5384678	-47.24674
440574	cg2513875[illegible]	39780366	11757314	0.530273	0	1	0.6340232	20.25727
440600	cg2514021[illegible]	60681365	60681365	0.2968062	0	1	0.4877058	58.34538
440840	cg2515375[illegible]	57695325	57695325	0.36934	0	1	0.2520459	-27.15576
440976	cg2516307[illegible]	21787372	21787372	0.8152897	0	1	0.69151	-39.48505
441007	cg2516554[illegible]	18794378	49604510		0	1	0.7137342	4.34E-09
441062	cg2517111[illegible]	19601447	19601447	0.3572235	0	1	0.4707863	22.76479
441092	cg2517411[illegible]	42628414	42628414	0.654606	0	1	0.5171468	-32.80758
441096	cg2517433[illegible]	24627320	24627320	0.8053802	0	1	0.9101354	49.02666
441104	cg2517460[illegible]	67697330	21705410	0.3268157	0	1	0.4478187	25.85972
441174	cg2518117[illegible]	45797474	45797474	0.3434794	0	1	0.4514919	21.18716
441241	cg2518614[illegible]	71800390	71800390	0.7457542	0	1	0.8575065	40.1866

441381	cg25194822	29809481	29809481	0.3189672	0	1	0.4374059	25.15355
441418	cg25196716	64647415	64647415	0.49019	0	1	0.383335	-20.33892
441516	cg25202367	61615420	61615420	0.6467357	0	1	0.4726614	-48.92993
441536	cg25203245	67684364	67684364	0.8040926	0	1	0.701293	-28.56787
441578	cg25205438	18792373	18792373	0.8075005	0	1	0.6464034	-58.1007
441772	cg25217276	63639328	63639328	0.2173328	0	1	0.3837542	51.487
441807	cg25219495	71607444	71607444	0.4080805	0	1	0.5427735	29.85281
441813	cg25220063	30798484	26616353	0.2988595	0	1	0.0942669	-101.5495
441904	cg25225155	46784473	11652474	0.7991213	0	1	0.6357303	-57.94611
441934	cg25226891	74719354	74719354	0.4646975	0	1	0.3562166	-21.14157
441944	cg25227803	65744387	65744387	0.2556835	0	1	0.359805	22.3366
442118	cg25242423	57751408	57751408	0.3125382	0	1	0.4132217	19.50891
442130	cg25243082	55626484	43698461	0.6757144	0	1	0.5427124	-31.87951
442191	cg25246894	12721379	12721379	0.7382994	0	1	0.4957897	-99.287
442207	cg25247689	23714413	23714413	0.7710787	0	1	0.5831565	-67.72445
442232	cg25249286	36623314	71712407	0.3614733	0	1	0.4881682	27.23363
442235	cg25249362	17794346	17794346	0.6820153	0	1	0.8293214	54.33202
442273	cg25252017	36690375	36690375	0.6317003	0	1	0.7320586	22.48777
442274	cg25252197	42767506	42767506	0.1280498	0	1	0.2331236	32.23116
442339	cg25257671	57675383	36743314	0.7362666	0	1	0.590201	-41.28479
442513	cg25270899	54630401	54630401	0.9299902	0	1	0.8254924	-56.18255
442618	cg25277809	17760506	17760506	0.5489025	0	1	0.4387609	-21.23718
442675	cg25281849	53728444	53728444	0.2501398	0	1	0.3517545	21.71946
442701	cg25283336	20702363	20702363	0.2890834	0	1	0.7795317	374.3439
442803	cg25290227	65797502	65797502	0.5625722	0	1	0.4553191	-20.39312
442821	cg25291250	32617420	32617420	0.8354344	0	1	0.7047634	-46.25608
442891	cg25296938	39603442	39603442	0.3914981	0	1	0.5004312	20.92891
442908	cg25298319	46643482	51700386	0.3660332	0	1	0.0400202	-250.5329
443037	cg25307332	20611310	20611310	0.3846082	0	1	0.5188726	29.78759
443134	cg25312054	48710475	48710475	0.4436822	0	1	0.5524824	20.82575
443152	cg25312970	14611497	14611497	0.5476474	0	1	0.6546655	21.78573
443271	cg25319279	11772429	11772429	0.80401	0	1	0.6803386	-37.93377
443370	cg25325592	19657413	15603304	0.6379317	0	1	0.5282031	-22.24753
443384	cg25326289	69638390	20604404	0.2096539	0	1	0.3226596	27.95491
443396	cg25326998	52608434	46762308	0.1892742	0	1	0.0817915	-41.15245
443523	cg25336892	12802321	12802321	0.527218	0	1	0.719062	63.41651
443561	cg25339052	74719359	74719359	0.3779716	0	1	0.6271452	94.89543
443587	cg25340688	53782363	38662399	0.5679961	0	1	0.4494896	-24.08553
443689	cg25345738	41694346	41694346	0.123933	0	1	0.225194	30.9979
443889	cg25356468	40666356	40666356	0.5842804	0	1	0.7309638	41.12482
443909	cg25357666	67661507	67661507	0.7107604	0	1	0.8253083	36.38985
444047	cg25365934	33620336	59714368	0.4120016	0	1	0.2996636	-23.58803
444121	cg25369262	60723452	60723452	0.2218748	0	1	0.3262241	23.98937

444150	cg25371267	46633425	46633425	0.5636074	0	1	0.6740328	23.5055
444182	cg25372296	66787371	66810354	0.2719322	0	1	0.4419784	48.85971
444227	cg25375162	65732308	65732308	0.4181897	0	1	0.5757074	39.45872
444243	cg25376032	45623463	19763424	0.6777091	0	1	0.8005341	37.10494
444318	cg25381253	28714405	28714405	0.7257973	0	1	0.9222565	124.8037
444348	cg25382821	26632370	26632370	0.6074413	0	1	0.7125075	23.18671
444446	cg25388952	24657323	24657323	0.9474273	0	1	0.7030855	-188.4031
444552	cg25395546	21733385	21733385	0.510325	0	1	0.4100101	-18.22313
444608	cg25399309	59792335	59792335	0.5264575	0	1	0.4081033	-23.93549
444626	cg25400378	25778421	42739471	0.1296988	0	1	0.2378024	33.4275
444777	cg25408960	67762367	67762367	0.6933988	0	1	0.8785393	93.4474
444809	cg25410789	19742358	19742358	0.5206456	0	1	0.7136461	63.54901
444904	cg25417675	46678372	48657349	0.3828187	0	1	0.268193	-25.46983
444939	cg25420101	65616357	65616357	0.4184394	0	1	0.6430004	77.95456
445004	cg25423077	53686471	70602466	0.9058185	0	1	0.7748805	-65.22946
445030	cg25424279	44730478	44730478	0.2872234	0	1	0.4286197	34.97222
445055	cg25425994	55685434	55685434	0.4689274	0	1	0.3660949	-19.27648
445157	cg25432807	58726341	73736305	0.8814739	0	1	0.780819	-38.9419
445180	cg25434864	63772451	59708488	0.7542831	0	1	0.8922827	65.12864
445198	cg25436245	60784379	51657370	0.7368808	0	1		-4.34E-09
445223	cg25437886	14705510	14705510	0.6798612	0	1	0.7881452	29.35366
445237	cg25438876	46644374	46644374	0.376186	0	1	0.4787887	19.10506
445325	cg25445671	38619346	38619346	0.3295415	0	1	0.4888403	41.17265
445343	cg25446460	25720424	25720424	0.7079348	0	1	0.809203	28.43067
445411	cg25450121	23733451	23733451	0.1152491	0	1	0.2372686	42.54676
445447	cg25452165	58610460	10801428	0.8708485	0	1	0.7181571	-67.92208
445491	cg25455408	56705368	56705368	0.5632918	0	1	0.666102	20.75125
445616	cg25462858	19638351	19638351	0.1557525	0	1	0.2675084	32.01286
445684	cg25467833	56651484	56651484	0.5010929	0	1	0.6783146	52.15614
445703	cg25468618	69778302	23627486	0.7239861	0	1	0.5532198	-52.33312
445714	cg25468928	31686334	31686334	0.9349387	0	1	0.8341333	-55.59692
445726	cg25469418	47711344	68633335	0.1779492	0	1	0.2872099	28.84875
445747	cg25470758	73769489	72647366	0.5809531	0	1	0.4117401	-44.94452
445908	cg25481160	13663473	13663473	0.2669095	0	1	0.3748578	23.19532
445949	cg25483907	22723355	22723355	0.4808907	0	1	0.599512	24.47301
446017	cg25488284	57793476	57793476	0.811076	0	1	0.9121003	47.2133
446173	cg25498731	73789377	73789377	0.3123357	0	1	0.5473514	85.10367
446458	cg25516803	41793409	41793409	0.3785068	0	1	0.2314516	-40.76235
446640	cg25531220	19725311	19725311	0.718525	0	1	0.5516859	-49.82681
446653	cg25532201	26658455	52798333	0.5220832	0	1	0.393838	-27.47878
446703	cg25535982	14607324	14607324	0.3621943	0	1	0.5170528	38.48122
446712	cg25536467	34807466	34807466	0.4085344	0	1	0.6359653	79.59093
446740	cg25538571	64636442	64636442	0.3733552	0	1	0.5177575	33.90163

446800	cg2554231	49701389	49701389	0.4498976	0	1	0.5856267	30.49245
446807	cg2554287	56757475	22721337	0.3985451	0	1	0.5137158	22.90279
446827	cg2554438	16604387	16604387	0.2081208	0	1	0.321507	28.20652
446854	cg2554648	53758452	53758452	0.3787972	0	1	0.4826246	19.4503
446861	cg2554713	23714488	23714488	0.4790661	0	1	0.5908168	22.07263
446933	cg2555169	13644385	13644385		0	1		-4.34E-09
447007	cg2555600	48631359	48631359	0.5278742	0	1	0.6454979	25.09951
447051	cg2555840	49637374	11728357	0.3553143	0	1	0.2531143	-21.79519
447196	cg2556824	55683481	55683481	0.6874784	0	1	0.8070474	36.37588
447212	cg2556946	66638340	66638340	0.8206583	0	1	0.6552387	-63.19321
447213	cg2556948	54634366	66626421	0.7744395	0	1	0.6626862	-29.58595
447330	cg2557671	53674496	53674496	0.2602204	0	1	0.1416761	-36.78827
447334	cg2557696	67768472	67768472	0.7957621	0	1	0.4414288	-223.2663
447466	cg2558481	56767305	31807363	0.8505689	0	1	0.7218968	-48.0887
447468	cg2558493	19769332	19769332	0.4504659	0	1	0.1808995	-127.9737
447531	cg2558884	27759392	27759392	0.3317982	0	1	0.439577	21.32254
447605	cg2559319	33751503	33751503	0.5880287	0	1	0.4098297	-49.47576
447639	cg2559482	27779474	27779474	0.5261412	0	1	0.4061425	-24.50307
447665	cg2559628	35611367	35611367	0.39066	0	1	0.5454519	38.18669
447680	cg2559711	58743426	58743426	0.5344508	0	1	0.688991	41.76344
447693	cg2559762	32647412	32647412	0.2917382	0	1	0.3996586	22.34694
447696	cg2559774	43678384	43678384	0.5165083	0	1	0.3983612	-23.9003
447725	cg2559871	14714508	14714508	0.9066262	0	1	0.7022567	-122.1437
447791	cg2560170	25670452	25670452	0.8470733	0	1	0.64788	-92.54516
447860	cg2560531	43703500	43703500	0.7474386	0	1	0.6383031	-26.55494
447885	cg2560678	13640314	35797396	0.8940859	0	1	0.7721385	-55.11167
447896	cg2560721	47759413	47759413	0.3746516	0	1	0.4912356	23.55264
447950	cg2561051	14744466	13797329	0.3701088	0	1	0.2289015	-38.31791
447956	cg2561105	52649309	45790361	0.6149713	0	1	0.7630723	44.87158
447984	cg2561306	23731348	38682366	0.1967606	0	1	0.3043537	26.74025
448001	cg2561425	12786346	70791391	0.4617485	0	1	0.6508133	56.93473
448073	cg2561855	67639508	67639508	0.4535331	0	1	0.5914935	31.43595
448077	cg2561873	19602485	19602485	0.4494013	0	1	0.3131578	-31.91652
448211	cg2562647	56619478	56619478	0.3582218	0	1	0.5399784	51.43297
448229	cg2562767	13775313	13775313	0.837808	0	1	0.6705902	-68.19366
448328	cg2563306	72743407	72743407	0.8005056	0	1	0.9254932	69.30048
448351	cg2563500	13644333	13644333		0	1	0.8853487	4.34E-09
448373	cg2563646	54810503	54810503	0.2111749	0	1	0.3420571	35.17649
448449	cg2564151	10757490	10757490	0.2510192	0	1	0.3557293	22.72669
448471	cg2564295	33807370	33807370	0.6507074	0	1	0.7825602	39.24107
448486	cg2564409	61724465	61724465	0.6921556	0	1	0.5894439	-21.56493
448497	cg2564447	35724359	35724359	0.4578074	0	1	0.641721	53.77596
448561	cg2564951	25669495	25669495	0.2708427	0	1	0.6586671	374.3439

448609	cg2565275[illegible]	25660390	25660390	0.1595973	0	1	0.2696927	30.89137
448670	cg2565548[illegible]	41648465	41648465	0.9276263	0	1	0.7315214	-128.0261
448714	cg2565990[illegible]	32630357	32630357	0.5042471	0	1	0.6069688	19.46546
448778	cg2566405[illegible]	50613420	50613420	0.5746399	0	1	0.6844012	23.6476
448804	cg2566621[illegible]	13665497	13665497	0.4215521	0	1	0.6142181	57.6215
448873	cg2567124[illegible]	26733308	26733308	0.6542093	0	1	0.7593099	25.80691
448918	cg2567402[illegible]	35754413	35754413	0.8196559	0	1	0.6994819	-38.33115
448925	cg2567488[illegible]	24782425	24782425	0.3769865	0	1	0.5001368	25.78228
448952	cg2567739[illegible]	43683426	43683426	0.5572107	0	1	0.72374	50.12905
449099	cg2568787[illegible]	33636301	33636301	0.5085342	0	1	0.6165398	21.25783
449136	cg2569095[illegible]	18631435	50701307	0.410479	0	1	0.3016442	-22.36423
449160	cg2569213[illegible]	26791369	26791369	0.5789675	0	1	0.723433	39.51816
449177	cg2569292[illegible]	29610437	29610437	0.3169485	0	1	0.545288	80.31303
449197	cg2569479[illegible]	66718303	66718303	0.1857533	0	1	0.2887913	25.76914
449201	cg2569502[illegible]	20753405	34643314	0.2968803	0	1	0.1884046	-27.69505
449232	cg2569772[illegible]	66760371	35687481	0.6072488	0	1	0.3543063	-97.53401
449282	cg2570164[illegible]	20738426	20738426	0.3298644	0	1	0.4649318	30.984
449331	cg2570549[illegible]	42733480	11778350	0.0888154	0	1	0.2090859	46.85942
449371	cg2570774[illegible]	43624350	43624350	0.3980955	0	1	0.5063811	20.68777
449396	cg2570933[illegible]	29620365	29620365	0.677015	0	1	0.8011093	37.77287
449409	cg2571029[illegible]	31786453	31786453	0.4388989	0	1	0.5606223	25.14473
449534	cg2571874[illegible]	34656411	34656411	0.5304308	0	1	0.6414839	22.76735
449820	cg2573549[illegible]	58601483	28662440	0.1580191	0	1	0.3387406	67.93367
449832	cg2573625[illegible]	63709335	50624488	0.7713711	0	1	0.5634732	-80.40267
449843	cg2573698[illegible]	42624311	42624311	0.3207346	0	1	0.1885771	-37.75693
449867	cg2573817[illegible]	31778309	31778309	0.2437517	0	1	0.3572333	26.1588
450021	cg2574613[illegible]	45616428	71657317	0.735022	0	1	0.6295438	-24.45761
450045	cg2574767[illegible]	54620484	54620484	0.6124183	0	1	0.760487	44.5739
450070	cg2574925[illegible]	65713432	65713432	0.6246313	0	1	0.7357012	26.55838
450137	cg2575363[illegible]	22639435	43710382	0.6548347	0	1	0.5493541	-21.29402
450139	cg2575372[illegible]	32777378	32777378	0.1588749	0	1	0.2755531	33.86413
450178	cg2575585[illegible]	57716321	57716321	0.3463332	0	1	0.4603226	23.07449
450208	cg2575714[illegible]	55763352	55763352	0.7919567	0	1	0.6115489	-66.68143
450293	cg2576321[illegible]	40639344	40639344	0.7404023	0	1	0.8453119	34.61017
450333	cg2576519[illegible]	10671490	10671490	0.671647	0	1	0.796388	37.50579
450414	cg2577105[illegible]	13616491	13616491	0.6830693	0	1	0.8532966	74.28949
450416	cg2577111[illegible]	48691468	48691468	0.3754372	0	1	0.487609	22.09271
450430	cg2577185[illegible]	36780433	36780433	0.8609775	0	1	0.6443484	-110.7184
450530	cg2578002[illegible]	73614460	73614460	0.2012322	0	1	0.3103269	27.01031
450554	cg2578175[illegible]	67767325	67767325	0.1668594	0	1	0.3238076	52.74796
450598	cg2578398[illegible]	13644438	13644438		0	1	0.8719878	4.34E-09
450603	cg2578428[illegible]	20699410	20699410	0.3320479	0	1	0.439359	21.16918
450661	cg2578879[illegible]	68742426	68742426	0.3917991	0	1	0.5362904	33.79387

450677	cg25790133	24628433	24628433	0.2295408	0	1	0.3439857	27.27605
450888	cg25804357	56611493	56611493	0.7205114	0	1	0.5758775	-39.37574
451073	cg25817503	31787453	31787453	0.8078796	0	1	0.6822978	-39.35303
451207	cg25825627	69600381	69600381	0.7092423	0	1	0.8130499	29.93099
451243	cg25827670	53622440	53622440	0.2802672	0	1	0.4384772	42.68883
451265	cg25828963	72722475	72722475	0.1327973	0	1	0.2392302	32.25962
451312	cg25832410	54781444	42720419	0.6166686	0	1	0.4569475	-41.10725
451322	cg25832925	43714394	36726440	0.2799495	0	1	0.1594002	-35.5606
451548	cg25848037	69774465	29642457	0.4950904	0	1	0.3852165	-21.27057
451644	cg25856090	22744405	22744405	0.5814615	0	1	0.7850433	80.05802
451818	cg25873514	27674380	27674380	0.7657608	0	1	0.8915457	56.80021
451890	cg25879395	28614346	28614346	0.7434925	0	1	0.3158891	-322.374
451914	cg25880954	50802381	50802381	0.449187	0	1	0.689494	91.87236
452009	cg25886479	65601307	44698438	0.7500027	0	1	0.6054411	-41.82633
452031	cg25888867	16784456	16784456	0.5977738	0	1	0.4772047	-25.11667
452078	cg25892587	57739357	57739357	0.3186263	0	1	0.441425	26.70101
452119	cg25896603	41801497	48769415	0.628756	0	1	0.5183764	-22.25658
452128	cg25897043	11768491	11768491	0.7326587	0	1	0.5904273	-39.22984
452278	cg25910261	15796467	15796467	0.4373412	0	1	0.241752	-65.08949
452301	cg25912009	47703395	47703395	0.4898535	0	1	0.6124887	26.08645
452330	cg25914522	27699304	27699304	0.48274	0	1	0.6759387	60.62719
452401	cg25919922	37687374	18734419	0.1737643	0	1	0.0193367	-98.58508
452582	cg25929399	14621300	14621300	0.4191144	0	1	0.0276528	-349.2962
452640	cg25932869	72731368	14691388	0.3083745	0	1	0.4600335	38.47936
452712	cg25937216	64668503	31790314	0.9387382	0	1	0.7759559	-107.0919
452715	cg25937451	41673508	41673508	0.279543	0	1	0.4038485	28.54623
452980	cg25951210	33737452	37624370	0.7913222	0	1	0.6834554	-29.47314
452989	cg25951717	14715421	14715421	0.927412	0	1	0.7421162	-118.6018
453043	cg25954269	73614359	73614359	0.3322673	0	1	0.4383008	20.76192
453047	cg25954539	70697407	70697407	0.7812776	0	1	0.6238726	-51.89884
453070	cg25956089	32617505	32617505	0.3593568	0	1	0.4617875	19.23495
453072	cg25956106	33647352	33647352	0.3021441	0	1	0.4040059	20.11765
453110	cg25959131	39609353	39609353	0.4260786	0	1	0.581329	38.50097
453194	cg25964213	74775451	74775451	0.5734528	0	1	0.7305108	46.01398
453233	cg25966908	40606387	40606387	0.4932174	0	1	0.3516026	-33.09159
453426	cg25977769	66729380	66729380	0.1508845	0	1	0.299482	50.70055
453471	cg25980637	46736398	19772372	0.5302351	0	1	0.4124193	-23.74422
453503	cg25983380	67706311	71663305	0.3046974	0	1	0.1634418	-45.02779
453539	cg25985355	18673434	24707378	0.5126501	0	1	0.3879403	-26.24054
453541	cg25985455	57639456	57639456	0.4187259	0	1	0.6698399	98.33933
453578	cg25987452	27789451	10603416	0.543075	0	1	0.4014615	-32.59023
453689	cg25996061	26777302	26777302	0.803418	0	1	0.909972	50.15628
453690	cg25996077	14794347	14794347	0.0832583	0	1	0.187919	39.28132

453712	cg2599730(	67738491	67738491	0.2008994	0	1	0.3038986	24.7253
453754	cg2600054:	13707418	13707418	0.4215916	0	1	0.2464036	-53.35111
453756	cg2600061(	49723391	32777403	0.1978384	0	1	0.0591244	-67.1687
453830	cg2600477	43686443	49758509	0.4413912	0	1	0.3152336	-28.01296
453975	cg2601302	17626301	41699495	0.8866869	0	1	0.7625947	-54.24926
454097	cg2601968(	27660307	58793389	0.2691893	0	1	0.1673258	-26.75132
454202	cg2602705:	15694442	15694442	0.4776487	0	1	0.5782254	18.4911
454210	cg2602766(	67758452	36725493		0	1	0.0355917	4.34E-09
454322	cg2603431:	51738305	51738305	0.5324259	0	1	0.6720646	34.40134
454339	cg2603507	30783484	30783484	0.3344671	0	1	0.8626972	374.3439
454352	cg2603563:	61612472	61612472	0.4511722	0	1	0.3335597	-24.53387
454389	cg2603793(	22744448	22744448	0.3792316	0	1	0.5270568	35.26214
454399	cg2603851	11809492	11809492	0.4001659	0	1	0.6256903	77.97603
454585	cg2605329	18687306	60664323	0.394046	0	1	0.2242251	-52.56351
454801	cg2606709(	71710417	71710417	0.3124903	0	1	0.4606517	36.8238
454831	cg2606904	12603426	12603426	0.40095	0	1	0.5248661	25.88811
454842	cg2606953(	31662423	31662423	0.3301698	0	1	0.4746491	34.74027
454852	cg2606997:	46804344	46804344	0.6123749	0	1	0.7146379	22.3201
454971	cg2607623:	44657335	44657335	0.4961496	0	1	0.3208193	-49.10336
454992	cg2607713:	10724432	10724432	0.5187843	0	1	0.3995114	-24.27809
455130	cg2608690:	55766497	21660459	0.1986502	0	1	0.3419667	41.93422
455148	cg2608829	60663334	73801426	0.6425626	0	1	0.5060837	-32.00818
455169	cg2608970	62735412	62735412	0.583429	0	1	0.7281102	39.97725
455353	cg2610098(	30670307	30670307	0.6974872	0	1	0.5900663	-23.35417
455401	cg2610317(	47663399	47663399	0.30622	0	1	0.4232006	24.9894
455402	cg2610330(	59764449	59764449	0.5959872	0	1	0.7782497	65.46835
455630	cg2611437	31604358	39695312	0.3106791	0	1	0.1839608	-35.75967
455646	cg2611521(	38731321	55617402	0.858871	0	1	0.7063819	-64.32678
455663	cg2611632(	25741478	25741478	0.1389962	0	1	0.2576449	37.19402
455763	cg2612090:	64650457	64650457	0.4218938	0	1	0.5441847	25.2884
455822	cg2612459(	57687319	57687319	0.5117643	0	1	0.7000284	59.64408
455849	cg2612629	25720316	25720316	0.2610918	0	1	0.3687847	23.33392
455881	cg2612783(	32679334	32679334	0.2653538	0	1	0.161568	-28.03921
456030	cg2613557:	52734438	32772485	0.8041048	0	1	0.6577423	-49.38221
456059	cg2613741	49642328	49642328	0.7614554	0	1	0.5717822	-67.32548
456110	cg2614047	42804422	42804422	0.1034957	0	1	0.2447789	55.76197
456173	cg2614456	23648361	37618434	0.3387147	0	1	0.4406692	19.39011
456442	cg2615827(	45735423	45735423	0.1285971	0	1	0.2493193	39.69935
456506	cg2616105	16681300	16681300	0.2641151	0	1	0.3811444	26.50578
456621	cg2616685	22682412	22682412	0.2703612	0	1	0.4405727	49.04201
456669	cg2616978:	21710418	21710418	0.1915323	0	1	0.3154753	33.83815
456683	cg2617056(	68740498	52687301	0.2833878	0	1	0.1802527	-26.21647
456729	cg2617377:	55714323	64648378	0.3113224	0	1	0.564155	98.26444

456747	cg26174656	53648435	53648435	0.2811906	0	1	0.4320756	39.32589
456760	cg26175448	67607446	67607446	0.3870369	0	1	0.5335267	34.64615
456783	cg26177041	21601334	21601334	0.209334	0	1	0.3483855	38.94976
456784	cg26177096	34651357	34651357	0.4824789	0	1	0.6254478	34.11657
456813	cg26179400	24615388	24615388	0.9161152	0	1	0.7918727	-64.61189
456848	cg26181731	39672354	39672354	0.1267223	0	1	0.2796175	57.50088
456897	cg26184765	55672420	55672420	0.9580349	0	1	0.8540938	-70.39412
456978	cg26188621	61802303	61802303	0.4865208	0	1	0.6342611	36.35091
457121	cg26197930	23737425	23737425	0.8865655	0	1	0.7835912	-41.41427
457162	cg26201596	30699308	30699308	0.6450452	0	1	0.7469154	23.82813
457293	cg26209676	24801488	24801488	0.159748	0	1	0.2602482	26.85698
457324	cg26211724	16665412	16665412	0.5668288	0	1	0.3306991	-85.25916
457458	cg26220110	59617342	59617342	0.7503647	0	1	0.8542628	35.52598
457598	cg26227225	11801364	70688369	0.5212397	0	1	0.1090402	-328.5496
457615	cg26228123	38777402	13800396	0.235375	0	1	0.1129069	-43.20112
458016	cg26251585	33790464	33790464	0.5465709	0	1	0.7117989	48.45827
458241	cg26264769	12612401	12612401	0.2806867	0	1	0.3809875	20.2193
458469	cg26276301	58641425	58641425	0.4145022	0	1	0.5221066	20.41814
458470	cg26276327	69759301	69759301	0.3278179	0	1	0.2207669	-25.02765
458521	cg26278987	57773508	57773508	0.8727414	0	1	0.7647633	-41.16799
458599	cg26282792	50743508	35771465	0.19051	0	1	0.3115988	32.70448
458667	cg26286839	56743399	52663310	0.4695551	0	1	0.6084437	32.08654
458672	cg26287080	70697461	70697461	0.584962	0	1	0.8620189	374.3439
458674	cg26287152	28777449	28777449	0.8176677	0	1	0.9330938	65.7206
458678	cg26287521	48732370	48732370	0.9404292	0	1	0.7183041	-161.0438
458797	cg26294955	71770374	71770374	0.2324823	0	1	0.3425268	25.4814
458812	cg26295774	27637485	27637485	0.6523969	0	1	0.5136898	-33.22895
458823	cg26296371	32610338	32610338	0.4451529	0	1	0.7459268	150.515
459085	cg26310256	19693331	19693331	0.3923037	0	1	0.2625624	-31.40843
459089	cg26310429	66734380	50763482	0.8090089	0	1	0.9285548	66.66373
459092	cg26310551	26661506	36664364	0.2197306	0	1	0.0712646	-69.52829
459222	cg26316946	45600479	45600479	0.5506961	0	1	0.4486329	-18.7567
459310	cg26322739	60620301	60620301	0.2908681	0	1	0.4934297	65.33379
459326	cg26323797	57763307	58702312	0.5100664	0	1	0.3065289	-65.07499
459392	cg26328633	30791406	17743418	0.5956609	0	1	0.4606053	-30.3705
459437	cg26330809	63768469	63768469	0.6198224	0	1	0.7575938	39.39201
459521	cg26334518	40773309	40773309	0.2747751	0	1	0.4485495	50.55668
459639	cg26340003	23653500	13707336	0.3076309	0	1	0.1900161	-31.28259
459743	cg26345203	24719508	24719508	0.6653395	0	1	0.7797087	31.13984
459891	cg26352652	25639317	25639317	0.7502463	0	1	0.6495618	-23.59497
459910	cg26353598	56697335	56697335	0.4720878	0	1	0.6050292	29.71991
459955	cg26355755	26645385	26645385	0.2469071	0	1	0.1324594	-36.06394
460055	cg26361436	26635492	26635492	0.3397738	0	1	0.4747276	30.7072

460058	cg26361533	38608410	38608410	0.4262078	0	1	0.6073621	51.29596
460197	cg26368752	71640387	16671344	0.4024936	0	1	0.2769355	-29.12598
460286	cg26373733	30695325	45787391	0.3141225	0	1	0.212056	-23.71378
460358	cg26379258	38675371	61735303	0.699258	0	1	0.586956	-25.13607
460494	cg26387458	44610504	44610504	0.4575473	0	1	0.1159996	-224.1818
460510	cg26388509	37715492	37715492	0.5434363	0	1	0.6444257	19.62959
460567	cg26391794	61735467	61735467	0.3310822	0	1	0.5053777	48.25304
460606	cg26393629	63741303	63741303	0.5314956	0	1	0.4088782	-25.40734
460637	cg26394788	51750435	65769395	0.487191	0	1	0.669552	54.3175
460654	cg26395694	65681467	65681467	0.3206315	0	1	0.4420576	26.15995
460655	cg26395761	52649436	52649436	0.2806206	0	1	0.4012477	27.14226
460690	cg26398228	32682322	32682322	0.7947065	0	1	0.6216758	-62.76493
460818	cg26406176	59727345	59727345	0.4083213	0	1	0.3065502	-19.9817
460873	cg26409826	12685507	12685507	0.6866149	0	1	0.8620221	80.48644
460930	cg26413192	33663333	33663333	0.1995356	0	1	0.3112733	28.15995
461015	cg26419287	45633360	45633360	0.8826764	0	1	0.7763126	-42.46534
461076	cg26422465	50734334	50734334	0.2143522	0	1	0.4732714	112.5449
461084	cg26422762	39603443	39603443	0.2055696	0	1	0.3122146	25.78972
461199	cg26429017	38702376	38702376	0.7006841	0	1	0.8097247	31.87391
461200	cg26429022	34655409	34655409	0.4892434	0	1	0.6368251	36.36396
461384	cg26440059	59636307	59636307	0.3761652	0	1	0.5158364	31.96578
461385	cg26440142	57664313	57664313	0.4073588	0	1	0.5231922	23.08919
461407	cg26441877	71732460	71732460	0.4440356	0	1	0.589184	34.29033
461433	cg26444097	58806385	58806385	0.578476	0	1	0.6788645	20.36377
461459	cg26445985	44800343	44800343	0.168704	0	1	0.066091	-41.9464
461513	cg26450149	69747316	69747316	0.4865548	0	1	0.59159	19.96768
461519	cg26450259	23687451	51794485	0.727327	0	1	0.8980404	90.87402
461541	cg26451345	74750436	74750436	0.542206	0	1	0.7242417	58.40152
461548	cg26451735	47622446	16697437	0.5823957	0	1	0.4280691	-38.10598
461609	cg26456183	59694308	59694308	0.3847494	0	1	0.2350389	-41.70668
461630	cg26457160	43639448	43639448	0.5711594	0	1	0.4378035	-29.44211
461661	cg26458748	18645396	18645396	0.4919968	0	1	0.6291469	31.88185
461698	cg26461267	58808317	58808317	0.2714072	0	1	0.3771466	22.28713
461710	cg26461710	47652340	47652340	0.8286428	0	1	0.9352425	60.06337
461717	cg26462055	48657505	48657505	0.3257207	0	1	0.4981574	47.50028
461780	cg26465155	22688368	22688368	0.7682354	0	1	0.9269488	96.30986
461796	cg26465602	15791438	11686361	0.1541998	0	1	0.2832462	40.20321
461875	cg26469387	55800379	55800379	0.2200035	0	1	0.4149799	67.09354
461884	cg26469895	18752376	18752376	0.4728953	0	1	0.5915743	24.38171
461936	cg26472636	51751360	39664404	0.3691081	0	1	0.1763361	-72.07888
461998	cg26476502	48690435	48690435	0.5583146	0	1	0.4243476	-29.59939
462010	cg26477117	24774328	24774328	0.7895786	0	1	0.4725711	-177.6891
462275	cg26494916	43673381	43673381	0.3881784	0	1	0.4937826	19.91717

462322	cg2649763	49677468	49677468	0.1946703	0	1	0.3336752	40.30596
462335	cg2649896	34658301	55807442	0.1964179	0	1	0.2975804	24.33113
462369	cg2650081	69624364	69624364	0.057124	0	1		-4.34E-09
462436	cg2650426	69779320	69779320	0.2272524	0	1	0.3576086	33.75317
462444	cg2650461	54705308	54705308	0.3916058	0	1	0.5710781	50.02446
462487	cg2650742	19698378	19698378	0.5624205	0	1	0.441226	-24.97109
462491	cg2650763	53661308	62741421	0.8928398	0	1	0.7634498	-59.56969
462574	cg2651246	38741327	41740366	0.7502131	0	1	0.6035997	-42.81844
462596	cg2651374	30608337	30608337	0.9230144	0	1	0.7332752	-119.5555
462630	cg2651568	30779505	30779505	0.8817914	0	1	0.6573837	-126.1163
462709	cg2652001	21677360	23712417	0.4171472	0	1	0.3066634	-22.77318
462823	cg2652546	25746318	25746318	0.5800183	0	1	0.6848242	21.99392
462936	cg2653123	34794479	34794479	0.6991495	0	1	0.8323922	47.09525
462942	cg2653170	49604443	49604443	0.3280978	0	1	0.2129418	-28.57418
463079	cg2653932	64633410	44747338	0.8151851	0	1	0.6292304	-74.83304
463260	cg2654781	55630445	55630445	0.4083208	0	1	0.5376492	27.82823
463279	cg2654841	13797473	13797473	0.8615659	0	1	0.6940563	-74.93964
463325	cg2655086	65611427	60674478	0.7602031	0	1	0.6513026	-27.33329
463405	cg2655505	55781355	56735502	0.4211623	0	1	0.5658683	33.89164
463428	cg2655633	39802498	39802498	0.8685749	0	1	0.7655866	-37.59882
463446	cg2655727	57621386	57621386	0.2584664	0	1	0.3744254	26.36549
463455	cg2655775	55671425	55671425	0.2075353	0	1	0.3223357	28.80228
463610	cg2656618	51692407	51692407	0.4470692	0	1	0.0961874	-245.5379
463630	cg2656738	35810338	35810338	0.7865394	0	1	0.9138371	65.85504
463637	cg2656773	46682415	46682415	0.3375166	0	1	0.459772	26.06598
463685	cg2657071	31716491	31716491	0.7071573	0	1	0.5890477	-27.62988
463692	cg2657090	71802374	49785337	0.526945	0	1	0.3703399	-39.1448
463806	cg2657635	11695399	11695399	0.7508217	0	1	0.6400986	-27.38716
463923	cg2658334	33799355	36642399	0.4844858	0	1	0.3523984	-29.36766
464019	cg2659008	24751415	24751415	0.2847196	0	1	0.1145986	-70.89518
464020	cg2659010	61648332	61648332	0.2221794	0	1	0.4313095	75.55077
464132	cg2659987	46766455	46766455	0.269745	0	1	0.3963172	29.82766
464148	cg2660118	34774405	48758480	0.5347347	0	1	0.4235112	-21.55855
464175	cg2660327	56671362	56671362	0.5764431	0	1	0.6836433	22.75067
464233	cg2660658	29720320	58680349	0.510499	0	1	0.4100764	-18.25453
464241	cg2660703	53770489	53770489	0.2736636	0	1	0.3855493	24.2941
464250	cg2660762	65650307	74790425	0.6029274	0	1	0.4193684	-52.46578
464424	cg2661844	44629409	44629409	0.4193768	0	1	0.2920028	-29.21953
464468	cg2662053	72664469	62672337	0.5859732	0	1	0.3298015	-100.2993
464770	cg2664092	55773392	55773392	0.4791974	0	1	0.6279535	36.59499
464790	cg2664277	71617460	71617460	0.5996879	0	1	0.8048208	85.25845
464810	cg2664396	22660445	65702504	0.4811074	0	1	0.3629702	-24.21339
464812	cg2664404	50714320	56650334	0.2923621	0	1	0.1618079	-39.96765

464818	cg26644674	35767342	35767342	0.5126967	0	1	0.6283602	24.00278
464834	cg26645432	61731305	61731305	0.1365575	0	1	0.3254521	77.68653
464894	cg26648185	69716389	69716389	0.5026507	0	1	0.6124254	21.75145
464952	cg26651280	21716506	21716506	0.6428679	0	1	0.8766773	131.7069
464954	cg26651303	69748313	57691326	0.5355802	0	1	0.6866323	40.04563
465087	cg26658509	17793361	17793361	0.6717535	0	1	0.8160079	50.18817
465104	cg26659805	67781376	70743379	0.1330239	0	1	0.0300226	-53.3782
465197	cg26665035	32769455	73635397	0.6761742	0	1		-4.34E-09
465240	cg26667091	58684433	58684433	0.7938002	0	1	0.6816176	-31.55615
465382	cg26673648	20643483	20643483	0.5358475	0	1	0.7246571	62.2494
465423	cg26675336	25790323	25790323	0.7093742	0	1	0.8364338	44.49883
465449	cg26676440	29735503	29735503	0.6180683	0	1	0.7213076	22.96158
465502	cg26679004	24802369	37789498	0.3172181	0	1	0.1705321	-46.84028
465539	cg26680608	29604349	29604349	0.533429	0	1	0.6628928	29.98239
465634	cg26684363	50792331	19714383	0.4476502	0	1	0.2848537	-44.59229
465697	cg26687173	60616472	60616472	0.5096417	0	1	0.3893771	-24.67498
465769	cg26690407	41734414	41734414	0.088329	0	1	0.5863634	374.3439
465820	cg26693329	56719484	56719484	0.2102468	0	1	0.1041498	-36.20279
465929	cg26700919	54775355	54775355	0.6029847	0	1	0.7140504	25.38129
466003	cg26705599	44686317	44686317	0.0615172	0	1	0.2057635	70.11072
466007	cg26705764	56712347	55669349	0.5201651	0	1	0.6910145	49.73338
466169	cg26715559	19747404	16738355	0.6195966	0	1	0.4354482	-53.15301
466183	cg26716902	47627353	66605321	0.4667596	0	1	0.3031831	-44.08859
466193	cg26717554	42807306	54764402	0.1927466	0	1	0.0396371	-86.04832
466328	cg26725029	19733366	10692372	0.1992208	0	1	0.0663621	-60.73236
466410	cg26730543	60736495	60736495	0.4425139	0	1	0.5700766	27.28311
466461	cg26734249	51800501	51800501	0.4960846	0	1	0.3728518	-25.85464
466519	cg26738024	21643304	21643304	0.5748709	0	1	0.7087717	33.95289
466536	cg26739149	57760454	20729387	0.532464	0	1	0.4298697	-18.87473
466540	cg26739327	15674421	15674421	0.8188916	0	1	0.6008221	-98.0136
466621	cg26744682	25706443	32762320	0.4779202	0	1	0.2717919	-68.74731
466725	cg26751645	43756408	56742483	0.2294457	0	1	0.0996384	-49.92181
466756	cg26753908	45736477	19683378	0.8359046	0	1	0.7094229	-44.09849
466798	cg26757229	52677329	52677329	0.9072421	0	1	0.7370458	-94.8213
466871	cg26763945	21756503	21756503	0.5435055	0	1	0.6468631	20.42051
466937	cg26767974	69641403	69641403	0.1830597	0	1	0.2864293	26.09578
467004	cg26772386	47753436	47753436	0.6021035	0	1	0.7252483	30.59301
467036	cg26775123	44723436	44723436	0.6214463	0	1	0.4747091	-35.5699
467061	cg26776784	29600381	29600381	0.3962818	0	1	0.6344377	86.9821
467185	cg26784201	38639415	29731349	0.6121998	0	1	0.5099773	-19.39331
467239	cg26786615	41696393	41696393	0.4121014	0	1	0.6818519	114.1571
467258	cg26787381	18622475	38648505	0.4937791	0	1	0.3863368	-20.49808
467274	cg26788216	63738432	63738432	0.2526861	0	1	0.3574739	22.68373

467328	cg2679125	25630315	64734485	0.5189366	0	1	0.4093952	-21.03898
467393	cg2679470	25603505	25603505	0.6013666	0	1	0.7062845	22.8629
467426	cg2679604	51642431	66716324	0.8315139	0	1	0.9826413	135.4976
467429	cg2679608	70615357	70615357	0.4029786	0	1	0.5816132	49.6409
467526	cg2680088	38756410	38756410	0.1627276	0	1	0.2659621	27.70746
467536	cg2680124	35631475	35631475	0.4199775	0	1	0.560981	32.36438
467553	cg2680206	64604428	71645485	0.3038473	0	1	0.1632446	-44.72494
467574	cg2680326	21803377	47640414	0.3882493	0	1	0.2869738	-20.34166
467705	cg2680986	40618348	40618348	0.3422519	0	1	0.4624062	25.23708
467743	cg2681170	14659426	30701330	0.2742469	0	1	0.4270062	40.52684
467773	cg2681348	58803504	58803504	0.0555665	0	1	0.1981449	71.21186
467804	cg2681503	10657320	10657320	0.4970047	0	1	0.693324	63.67137
467857	cg2681775	38804485	38804485	0.5616929	0	1	0.6723583	23.53051
467878	cg2681878	13611506	13611506	0.2116449	0	1	0.3148978	24.16603
467915	cg2682025	40647335	40647335	0.7928856	0	1	0.6700979	-36.194
467956	cg2682217	43780356	43780356	0.7682899	0	1	0.6647125	-25.8526
468033	cg2682618	38808475	38808475	0.3599725	0	1	0.49838	31.67455
468080	cg2682859	55632485	55632485	0.2927101	0	1	0.5063658	71.96589
468323	cg2684104	10786344	10786344	0.4967498	0	1	0.3813044	-23.1101
468387	cg2684380	74679492	74679492	0.3654743	0	1	0.4934614	27.64792
468562	cg2685458	20690445	20690445	0.5683381	0	1	0.7136328	39.1782
468563	cg2685501	34739493	34739493	0.710413	0	1	0.8414311	47.56784
468599	cg2685663	31766367	31766367	0.3519212	0	1	0.2470596	-22.94751
468744	cg2686482	14772371	14772371	0.2210127	0	1	0.4129451	65.1735
468843	cg2687118	67801384	67801384	0.3942535	0	1	0.5175299	25.69332
468897	cg2687388	65743318	65743318	0.8473133	0	1	0.7104147	-52.08136
468902	cg2687422	66674405	42694358	0.74232	0	1	0.6152386	-33.38008
469019	cg2687964	72640510	72640510	0.5129722	0	1	0.6373462	27.27444
469058	cg2688126	42661402	42661402	0.4168494	0	1	0.2364482	-57.02462
469088	cg2688252	14744359	14744359	0.474404	0	1	0.2781773	-62.45633
469646	cg2691579	21626380	21626380	0.6014811	0	1	0.7208102	28.8301
469650	cg2691595	30713425	36747372	0.5628749	0	1	0.4596944	-19.1511
469860	cg2692598	23688339	23688339	0.4984899	0	1	0.6298445	29.66284
469890	cg2692754	17756352	60699427	0.890152	0	1	0.7719354	-51.55801
469908	cg2692878	60716382	60716382	0.0420629	0	1	0.1875691	79.11317
469935	cg2692992	48754322	25803318	0.5174887	0	1	0.3732188	-33.84893
469943	cg2693049	36600316	36600316	0.8601365	0	1	0.7288915	-51.67023
470059	cg2693698	69669477	69669477	0.8868213	0	1	0.7097054	-90.82958
470117	cg2693994	22713399	22713399	0.1800686	0	1	0.2845006	26.73948
470131	cg2694072	67799462	67799462		0	1	0.0760833	4.34E-09
470144	cg2694178	70637510	70637510	0.7212012	0	1	0.064938	-371.3336
470147	cg2694187	63650347	65750312	0.9371387	0	1	0.8282544	-62.698
470163	cg2694282	24618335	24618335	0.6151276	0	1	0.7424989	33.51572

470226	cg26945978	21758419	21758419	0.5991247	0	1	0.7126305	26.19884
470286	cg26949055	59707352	59707352	0.510498	0	1	0.6405457	29.46334
470328	cg26951705	42739502	49667370	0.1705284	0	1	0.883224	374.3439
470400	cg26955290	38625407	38625407	0.3918009	0	1	0.4987584	20.31002
470416	cg26955881	63760457	63760457	0.2840147	0	1	0.1473875	-44.94025
470632	cg26968378	17663376	17663376	0.7263461	0	1	0.613721	-26.60035
470660	cg26970042	43685411	43685411	0.294686	0	1	0.4208013	28.64967
470693	cg26972554	16777377	16777377	0.6155992	0	1	0.499868	-23.76535
470733	cg26975184	57631400	36712464	0.723347	0	1	0.8296869	33.0781
470792	cg26978379	22712463	22712463	0.6147664	0	1	0.8429322	113.2359
470827	cg26980152	59747386	59747386	0.5825824	0	1	0.7182892	35.31535
470836	cg26980937	24794368	24794368	0.4204075	0	1	0.5374599	23.48099
470873	cg26982601	50742459	23730422	0.7102292	0	1	0.601233	-24.45789
470990	cg26987928	16658418	16658418	0.4872152	0	1	0.6319625	35.02923
471047	cg26991571	42649388	18632350	0.6435825	0	1	0.5231168	-26.04406
471118	cg26995506	39753355	39753355	0.2142281	0	1	0.5506265	374.3439
471181	cg27000233	73632481	73632481	0.7148778	0	1	0.8478277	49.93367
471185	cg27000496	55698357	66762343	0.8027424	0	1	0.6698967	-42.2276
471186	cg27000503	74713304	74713304	0.6434106	0	1	0.889303	148.7541
471258	cg27005118	39757339	39757339	0.4808498	0	1	0.6330995	38.24883
471336	cg27010159	74726465	74726465	0.5639297	0	1	0.6897745	29.72116
471393	cg27014438	43626331	43626331	0.5291834	0	1	0.6383536	22.07552
471462	cg27018309	30775481	30775481	0.6608894	0	1	0.7688682	27.61395
471471	cg27018984	55730337	55730337	0.2492103	0	1	0.7153928	374.3439
471500	cg27020349	37797354	21763385	0.9093664	0	1	0.6751305	-149.9869
471551	cg27023360	29785438	37634479	0.7463422	0	1	0.6066685	-39.22553
471563	cg27023871	37744504	37744504	0.8420811	0	1	0.6692869	-72.87234
471578	cg27024408	40638350	40638350	0.368018	0	1	0.4837921	23.3556
471612	cg27026192	40729374	40729374	0.2487208	0	1	0.3604716	25.29137
471701	cg27032146	60613493	22725397	0.4482486	0	1	0.3193854	-28.90006
471755	cg27035997	58804463	58804463	0.8146815	0	1	0.9156724	48.35574
471990	cg27049594	56651508	56651508	0.3921471	0	1	0.5098249	23.77566
471991	cg27049643	65639453	36691449	0.3744866	0	1	0.0785343	-191.2851
472006	cg27050407	72697501	72697501	0.4621366	0	1	0.5822592	24.7654
472065	cg27053299	61762319	21655443	0.6330103	0	1	0.4782489	-39.3471
472165	cg27058480	71679446	71679446	0.656011	0	1	0.5495279	-21.64874
472225	cg27061791	49754427	49754427	0.5112742	0	1	0.6251848	23.3455
472363	cg27069422	52798451	14672356	0.5620713	0	1	0.4052102	-39.09737
472376	cg27070397	16613327	16613327	0.8145192	0	1	0.6778355	-45.91053
472386	cg27071152	49759419	74642481	0.2513356	0	1	0.147114	-29.63952
472430	cg27073349	28774445	28774445	0.5364553	0	1	0.6802009	36.50883
472438	cg27074174	42657472	53774367	0.5990794	0	1	0.4116417	-54.46663
472443	cg27074505	63669414	63669414	0.6452407	0	1	0.7484017	24.3859

472452	cg2707499	25671495	25671495	0.3665757	0	1	0.4770725	21.64992
472525	cg2707909	18698302	18698302	0.7155995	0	1	0.5234082	-63.27229
472528	cg2707919	22628361	22628361	0.4259663	0	1	0.6204415	58.79035
472546	cg2708034	23660470	67760433	0.8278413	0	1	0.6798989	-54.40378
472555	cg2708124	33704307	13644439		0	1		-4.34E-09
472576	cg2708308	26697460	69671508	0.5207891	0	1	0.6543442	31.25407
472597	cg2708402	33774509	33774509	0.3479825	0	1	0.4801289	29.4699
472600	cg2708423	55610395	55610395	0.7367988	0	1	0.8412686	33.79373
472626	cg2708615	40698319	40698319	0.4054468	0	1	0.5742245	44.68752
472627	cg2708630	55662450	55662450	0.0762081	0	1	0.1933797	48.05889
472740	cg2709224	58623436	58623436	0.3779662	0	1	0.5658135	54.48849
472827	cg2709743	26787346	56786397	0.3943252	0	1	0.1843031	-81.82818
472869	cg2710014	41639327	41639327	0.5384663	0	1	0.656572	25.59284
472987	cg2710729	33650305	33650305	0.653266	0	1	0.8833083	131.6642
472995	cg2710796	61804348	61804348	0.6137342	0	1	0.7441629	34.94736
473108	cg2711298	22625499	38637352	0.948352	0	1	0.4945295	-371.3336
473147	cg2711586	22626378	14698339	0.2744685	0	1	0.4829434	69.97648
473213	cg2711931	47786401	49631300	0.1556783	0	1	0.0455351	-52.6227
473235	cg2712054	62612508	62612508	0.170834	0	1	0.2812442	29.9484
473269	cg2712270	44638414	44638414	0.4372748	0	1	0.5580679	24.80904
473471	cg27134251	54780387	54780387	0.6259891	0	1	0.4804124	-35.20536
473498	cg2713579	38624406	38624406	0.6961677	0	1	0.9072369	128.1914
473522	cg2713725	40786356	16786492	0.442646	0	1	0.3088668	-31.08908
473538	cg2713808	25769382	23785427	0.3721734	0	1	0.1972291	-58.39659
473572	cg2714005	20615401	35637328	0.3360263	0	1	0.221503	-27.78181
473601	cg27141751	69713489	69713489	0.5569783	0	1	0.710283	42.59526
473603	cg2714180	55690505	55690505	0.8175783	0	1	0.331204	-371.3336
473679	cg2714534	33750455	33750455	0.5814308	0	1	0.7487887	53.09442
473682	cg2714545	56637356	56637356	0.376381	0	1	0.5215699	34.193
473709	cg2714755	67807427	29706375	0.2591509	0	1	0.3703953	24.64728
473728	cg2714907	11611421	11611421	0.4604256	0	1	0.2315561	-87.50206
473744	cg2714997	40726435	40726435	0.3641795	0	1	0.5097877	34.51276
473772	cg2715167	44778424	44778424	0.2500113	0	1	0.3573981	23.69097
473774	cg2715177	30703368	64807415	0.8991746	0	1	0.7783437	-56.01089
473783	cg2715221	48629373	11693393	0.4299943	0	1	0.3281509	-19.54337
473831	cg2715651	73639456	73639456	0.6134913	0	1	0.7650948	46.84022
473862	cg2715857	33730338	39658484	0.7896887	0	1	0.6829184	-28.86449
473885	cg2715998	14676344	14676344	0.424045	0	1	0.5420973	23.82277
473973	cg2716617	72774459	72774459	0.5809637	0	1	0.6866862	22.36488
473981	cg2716667	68632365	44726414	0.3580844	0	1	0.2290351	-33.08722
474104	cg2717314	18733325	35674409	0.3032802	0	1	0.4274666	27.64808
474141	cg2717511	18779347	18779347	0.3712738	0	1	0.4973964	26.90071
474183	cg2717715	20601316	20601316	0.336081	0	1	0.4468276	22.19484

474205	cg27178293	25683343	25683343	0.3038762	0	1	0.1878237	-30.81573
474225	cg27179375	42797416	64680471	0.806002	0	1	0.6619074	-48.47364
474235	cg27179810	53803352	53803352	0.2213047	0	1	0.3301382	25.65207
474274	cg27181375	69721302	55727389	0.0701657	0	1	0.1865908	49.18149
474312	cg27183036	71680393	71680393	0.6060561	0	1	0.7451934	38.88082
474366	cg27186519	58668500	58668500	0.3799189	0	1	0.5079635	27.50696
474404	cg27190024	53748501	53748501	0.2996215	0	1	0.419524	26.21602
474550	cg27199697	41754334	41754334	0.5512347	0	1	0.6936901	36.67837
474562	cg27200257	58746331	58746331	0.4958464	0	1	0.6079493	22.43035
474574	cg27200895	66639482	66639482	0.2175691	0	1	0.3298394	27.16343
474600	cg27202913	29707314	29707314	0.3893447	0	1	0.1540373	-106.4897
474781	cg27214960	27609506	15718384	0.2246604	0	1	0.3268245	23.069
474843	cg27219182	57769383	57769383	0.4864779	0	1	0.626011	32.74377
474965	cg27226147	16780412	38724384	0.135556	0	1	0.2756251	48.6395
474998	cg27227742	31712431	37619383	0.2449484	0	1	0.1252896	-39.67231
475009	cg27228272	25764475	25764475	0.3722155	0	1	0.4973722	26.54382
475055	cg27230769	49780490	49780490	0.3650911	0	1	0.4852826	24.88825
475084	cg27232360	27709322	27709322	0.2337672	0	1	0.3502915	27.82345
475093	cg27233151	13736483	13736483	0.1670715	0	1	0.3215551	51.3924
475251	cg27244972	74728376	33694484	0.6404576	0	1	0.4101748	-81.70122
475288	cg27247697	44759351	31768322	0.0904623	0	1	0.205772	43.68424
475308	cg27248583	16718309	16718309	0.4324445	0	1	0.3306483	-19.48265
475343	cg27251155	37707300	12721408	0.6473841	0	1	0.4106952	-86.45903
475352	cg27251926	43694332	52770307	0.4373225	0	1	0.3239358	-23.31472
475374	cg27253485	66610377	66610377	0.4729127	0	1	0.3454184	-27.79122
475442	cg27257987	20738498	20738498	0.4723905	0	1	0.7828887	374.3439
475454	cg27258561	65616364	65616364	0.5269969	0	1	0.4109381	-23.15577
475501	cg27261733	54743425	54743425	0.6382905	0	1	0.463028	-49.19096
475537	cg27263998	19633313	19633313	0.4635296	0	1	0.5757963	22.08084
475544	cg27264322	39635355	46659312	0.9652267	0	1	0.7944688	-135.5835
475615	cg27268835	74723367	74723367	0.2037286	0	1	0.3068616	24.5972
475675	cg27272246	35664454	35664454	0.8527514	0	1	0.6437397	-101.7537
475692	cg27273227	45740413	45740413	0.61983	0	1	0.8574861	126.5399
475845	cg27282397	14747339	14747339	0.3001115	0	1	0.4062	21.5113
475865	cg27284120	64691332	64691332	0.3998019	0	1	0.5061655	20.08214
475866	cg27284194	47622464	47622464	0.6865138	0	1	0.5316443	-41.76806
475966	cg27291304	33710361	33710361	0.2675749	0	1	0.1577269	-30.97403
475968	cg27291464	51768317	51768317	0.3228096	0	1	0.4306893	21.53812
475978	cg27292099	72810314	72810314	0.6053461	0	1	0.7301877	31.59373
475981	cg27292220	41607394	41607394	0.6200283	0	1	0.7538734	37.26595
476054	cg27295654	29685464	29685464	0.7776478	0	1	0.8780116	38.04894
476097	cg27297558	25602349	25602349	0.2584012	0	1	0.3829918	29.603
476119	cg27298349	26760337	39706440	0.5490946	0	1	0.3652275	-52.44751

476330	cg27307781	26809452	58752493	0.0027164	0	1	0.1191962	77.77986
476357	cg27308738	33793335	33793335	0.418588	0	1	0.7443705	374.3439
476739	cg27326620	66679486	63773438	0.4771303	0	1	0.346495	-28.93093
476776	cg27328673	11704424	11704424	0.739526	0	1	0.5801822	-47.98954
476794	cg27330006	14736422	14736422	0.9231387	0	1	0.8028324	-64.76322
476809	cg27331292	54688488	54688488	0.3382758	0	1	0.4446487	20.75927
476891	cg27336587	12720447	71636304	0.0760711	0	1	0.1818823	41.46307
476931	cg27338836	13644452	13644452		0	1		-4.34E-09
476991	cg27342919	33666436	15745448	0.4938667	0	1	0.2469567	-98.69467
477068	cg27347523	61768307	42802448	0.3149559	0	1	0.5412835	79.03873
477082	cg27348515	66743491	58797317	0.3476132	0	1	0.2148526	-35.69656
477112	cg27350579	16797429	16797429	0.4837574	0	1	0.589301	20.0974
477191	cg27355713	60743476	60743476	0.4860043	0	1	0.6025745	23.82461
477289	cg27362525	44659426	29653431	0.320819	0	1	0.1627293	-53.99532
477294	cg27362989	63766336	63766336	0.3147332	0	1	0.5123168	61.25398
477297	cg27363141	27793347	21680509	0.5078914	0	1	0.6171039	21.65271
477345	cg27365991	20770382	20770382	0.2444026	0	1	0.4209388	54.23112
477358	cg27366766	21651349	14699327	0.7098462	0	1	0.5187926	-62.07313
477412	cg27369096	46808467	44740455	0.455086	0	1	0.3359542	-25.00688
477450	cg27371456	11810404	11810404	0.6126637	0	1	0.7127938	21.52104
477463	cg27372452	18690453	19678397	0.67098	0	1	0.5620077	-22.9137
477557	cg27378537	31615459	31615459	0.3887984	0	1	0.1702539	-90.37083
477569	cg27379407	44699377	48766488	0.8468719	0	1	0.7334294	-39.17054
477651	cg27383418	29768342	29768342	0.3815296	0	1	0.2702782	-24.20201
477797	cg27391816	54750357	56763451	0.1462727	0	1	0.2560247	32.16477
477819	cg27392904	71676300	71676300	0.2796624	0	1	0.3871301	22.57915
478023	cg27405400	59651484	70740307	0.8838141	0	1	0.7814126	-40.44855
478037	cg27406070	59665410	59665410	0.7234855	0	1	0.8509437	47.48512
478063	cg27408171	39791422	12749411	0.4434051	0	1	0.6660975	77.94891
478132	cg27412857	28717352	28717352	0.4128891	0	1	0.5300817	23.53293
478167	cg27414868	44664477	21610445	0.4466959	0	1	0.8606907	374.3439
478186	cg27416337	71665371	71665371	0.3081909	0	1	0.4413012	30.85146
478279	cg27422345	53700466	53700466	0.4598596	0	1	0.5679919	20.70292
478394	cg27427743	16749426	30747495	0.8774992	0	1	0.7479622	-55.04924
478452	cg27431049	26641373	32768334		0	1		-4.34E-09
478490	cg27433479	42614354	42614354	0.6775389	0	1	0.9234161	374.3439
478504	cg27434368	42682384	42682384	0.7251097	0	1	0.6088726	-27.89226
478594	cg27440866	56672320	56672320	0.1053516	0	1	0.2099182	35.23546
478606	cg27441354	14756460	14756460	0.7072533	0	1	0.5944551	-25.67897
478635	cg27443332	28752456	28752456	0.1274147	0	1	0.2312843	31.75619
478767	cg27450744	50802392	50802392	0.2285548	0	1	0.6767671	374.3439
478775	cg27451362	24611496	24611496	0.6543968	0	1	0.513674	-34.12096
478796	cg27452255	58690410	58690410	0.8902772	0	1	0.7877361	-42.05327

478806	cg27452922	55621476	40702446	0.6632386	0	1	0.5566679	-21.88083
478822	cg27454102	73724445	73724445	0.3097949	0	1	0.4240102	23.94117
478920	cg27461254	62610464	62610464	0.5879353	0	1	0.726187	36.96496
479011	cg27467876	14632431	14632431	0.2009788	0	1	0.0431793	-87.8899
479026	cg27468880	29620403	12793431	0.6566043	0	1	0.9369063	374.3439
479041	cg27469738	38713346	31774471	0.8091847	0	1	0.6876946	-37.55527
479217	cg27481428	44744456	44744456	0.4895775	0	1	0.7638177	129.6336
479269	cg27484662	21666391	11780432	0.7278507	0	1	0.8388299	36.62023
479275	cg27485108	17605506	20768326	0.4244875	0	1	0.3036002	-26.44408
479345	cg27488741	48773302	48773302	0.731518	0	1	0.834564	32.18213
479433	cg27494164	20717367	20632490	0.3408422	0	1	0.45177	22.16658
479445	cg27494897	61748336	61748336	0.2771873	0	1	0.4254063	38.35656
479569	cg27502298	62806352	62687463	0.6436923	0	1	0.901582	374.3439
479589	cg27503950	45729369	45729369	0.7967566	0	1	0.696515	-26.81828
479675	cg27508180	42743387	42743387	0.6903453	0	1	0.8091993	36.30849
479693	cg27509125	34691381	34691381	0.5489126	0	1	0.6493522	19.57408
479707	cg27509967	30789337	30789337	0.1487208	0	1	0.3723611	99.01151
479793	cg27514038	50745409	46671445	0.9437393	0	1	0.7637284	-125.5406
479801	cg27514336	39653495	39653495	0.6316997	0	1	0.7502524	30.44968
479840	cg27516925	19682423	19682423	0.9341912	0	1	0.62705	-243.2921
479851	cg27517563	45782402	45698475	0.7236221	0	1	0.6119495	-26.09712
479911	cg27520549	67703307	67703307	0.4542818	0	1	0.5729957	24.19508
479920	cg27521160	34808321	50637434	0.6222686	0	1	0.9510186	374.3439
479994	cg27526429	10643372	64765396		0	1	0.6920689	4.34E-09
480000	cg27526774	30783419	30783419	0.303263	0	1	0.408354	21.10818
480133	cg27533859	34615334	34615334	0.7408047	0	1	0.8769957	59.08617
480195	cg27537199	17804389	17804389	0.512873	0	1	0.3920116	-24.86371
480275	cg27541892	73804305	73804305	0.7262226	0	1	0.5730417	-43.77585
480281	cg27542341	72691401	52700469	0.20769	0	1	0.3092656	23.78897
480293	cg27543040	11634337	11634337	0.5570147	0	1	0.3330365	-76.86561
480331	cg27544673	66683447	66683447	0.2178459	0	1	0.3309973	27.48253
480355	cg27546114	35768310	35768310	0.6210355	0	1	0.4496898	-46.69542
480415	cg27549186	60697329	60697329	0.3773793	0	1	0.6588255	122.7299
480440	cg27550663	32793334	54606331		0	1		-4.34E-09
480503	cg27553926	12790451	64639473	0.4206991	0	1	0.3168731	-20.38465
480563	cg27557782	19616307	19616307	0.8642442	0	1	0.6814982	-86.39791
480749	cg27567580	42654353	42654353	0.0586664	0	1	0.2058143	73.22786
480827	cg27572120	19647409	51674379	0.5820965	0	1	0.4205936	-41.32235
480892	cg27575622	15736467	15736467	0.7735316	0	1	0.9099112	70.73605
480920	cg27577149	16659309	44767473	0.6248013	0	1	0.5151716	-21.93246
481021	cg27582323	14704347	14704347	0.1393097	0	1	0.2410011	29.37763
481047	cg27583307	27634389	32665472	0.3459871	0	1	0.2050426	-40.20573
481082	cg27585147	24620463	24620463	0.4433997	0	1	0.5565931	22.25048

481137	cg2758835(	48702341	48702341	0.2576218	0	1	0.3732775	26.29324
481272	cg2759810'	72641436	72641436	0.3968972	0	1	0.5260394	27.79281
481324	cg2760157'	26690489	26690489	0.5875124	0	1	0.6999018	25.19558
481482	cg2761232'	51801389	51801389	0.3402431	0	1	0.1777217	-54.19957
481522	cg2761448:	62601359	47732508	0.5380088	0	1	0.4030845	-29.94905
481632	cg2762172l	62733489	62733489	0.3228862	0	1	0.4709342	36.43872
481639	cg2762199'	41605444	41605444	0.4156277	0	1	0.5252444	21.04958
481685	cg2762513l	15722311	15722311	0.767724	0	1	0.4598527	-162.4123
481704	cg2762618:	16807466	66634378	0.1143765	0	1	0.3425046	112.6678
481966	cg2763919(	49621366	49621366	0.7869173	0	1	0.686281	-26.1359
481989	cg2764025'	73669303	73669303	0.5763307	0	1	0.6821095	22.22181
482028	cg2764247(	58749395	48615364	0.6613992	0	1	0.4864583	-50.04103
482109	cg2764821(	25667382	25667382	0.3983145	0	1	0.5080811	21.15334
482112	cg2764827(	67670425	67670425	0.4815387	0	1	0.587833	20.3125
482208	cg2765521'	27784335	25657320	0.283587	0	1	0.1403717	-49.52032
482283	cg2765904(	50766426	51699366	0.7458662	0	1	0.6434876	-23.95097
482296	cg2765979(	58690368	58690368	0.225704	0	1	0.4109862	60.81833
484258	ch.22.4827'	62659432	62659432	0.1691802	0	1	0.045637	-61.72841
485513	rs10033147	53636471	37774439	0.6182185	0	1	0.4890457	-28.56932
485514	rs1019916	43769491	43769491	0.3735388	0	1	0.5216559	35.44028
485516	rs10457834	15748350	15748350	0.9695354	0	1	0.8480659	-94.54636
485517	rs10774834	22788456	30718329	0.6346762	0	1	0.3352349	-138.9381
485518	rs10796216	14622465	41635319	0.3364213	0	1	0.1695455	-57.81847
485520	rs10882854	47771383	39668349	0.4810969	0	1	0.7757474	151.7644
485521	rs10936224	34619331	30630453	0.76804	0	1	0.6245472	-43.17172
485522	rs11034952	58692423	44652497	0.1902064	0	1	0.0371009	-87.33179
485523	rs11249206	60753422	60753422	0.5155119	0	1	0.3411648	-48.00545
485524	rs13369115	44627385	74780462	0.5035905	0	1	0.345103	-40.40899
485525	rs133860	52724359	52724359	0.8465078	0	1	0.6601176	-83.27675
485526	rs1414097	23759362	39621311	0.7912084	0	1	0.9320854	84.98336
485527	rs1416770	28667385	28667385	0.8580264	0	1	0.6352852	-114.4526
485528	rs1467387	45739435	45739435	0.5543295	0	1	0.6637531	22.83754
485529	rs1484127	33780375	33780375	0.9717023	0	1	0.655333	-290.7561
485532	rs1510480	69633374	69633374	0.5190102	0	1	0.3614534	-39.68781
485534	rs1941955	33709340	33709340	0.500488	0	1	0.8212647	374.3439
485536	rs2032088	63663495	63663495	0.6841089	0	1	0.2068664	-371.3336
485537	rs2125573	25698376	25698376	0.7132156	0	1	0.3832534	-176.2696
485538	rs213028	10622451	24684377	0.639438	0	1	0.3323443	-146.7529
485539	rs2208123	12714403	19761497	0.1791884	0	1	0.4754308	153.5253
485540	rs2235751	17621345	17621345	0.8264972	0	1	0.5399352	-158.7121
485541	rs2385226	46691371	17623494	0.4607706	0	1	0.7682423	374.3439
485542	rs2468330	23632464	23632464	0.6958199	0	1	0.3528791	-189.5658
485543	rs2521373	12625304	12625304	0.5063571	0	1	0.6690394	44.44134

485544	rs264581	52695345	52695345	0.6711646	0	1	0.3841269	-128.5183
485545	rs2804694	13748334	13748334	0.6785095	0	1	0.9664133	374.3439
485546	rs2857639	23793421	23793421	0.5595321	0	1	0.4548768	-19.57744
485550	rs3936238	42802473	29627504	0.2996688	0	1	0.6114528	151.7644
485551	rs4331560	10654345	10654345	0.5533861	0	1	0.6969407	37.34011
485553	rs4742386	34623456	34623456	0.4267532	0	1	0.5944017	44.35028
485555	rs5931272	39615340	39615340	0.5126834	0	1	0.9819773	374.3439
485559	rs6471533	11697340	55602361	0.3647898	0	1	0.2173016	-42.13958
485560	rs654498	36707408	15665335	0.3428003	0	1	0.2399364	-22.57817
485562	rs6626309	15806372	69767339	0.6416932	0	1	0.7914119	49.26139
485569	rs798149	48789496	48789496	0.8004849	0	1	0.6402367	-56.39805
485570	rs845016	37661486	37661486	0.3433668	0	1	0.4997139	39.49137
485571	rs877309	54760445	54760445	0.393485	0	1	0.0252833	-317.2814
485572	rs9292570	48712372	14806497	0.1782431	0	1	0.3370402	52.12325
485573	rs9363764	36689314	36689314	0.8294031	0	1	0.9573595	90.60218
485574	rs939290	70759471	70759471	0.6131538	0	1	0.7430084	34.60989
485575	rs951295	11711409	39778441	0.8075045	0	1	0.3545702	-371.3336
485576	rs966367	16795360	16795360	0.0352012	0	1	0.2056667	102.114

PCOS.Delt	ILMNID	NAME	ADDRESS	ALLELEA_	ADDRESSB	ALLELEB_	INFINIUM	NEXT_BA
0.1824424	cg00004073	cg00004073	38766357	TTAAAAAACCAAAAACCATCA			II	
-0.114829	cg00006815	cg00006815	18804378	CACCTCCTCCCCRATACAATCA			II	
0.1075888	cg00009306	cg00009306	74728390	CACACTTACATAACCCATTTTA			II	
-0.498723	cg00009523	cg00009523	42761437	TACCRACAACAACRCTCATTAA			II	
0.1200536	cg00013655	cg00013655	44675355	CCCCCTA	31736405	CCCCCTA	I	A
-0.145023	cg00017157	cg00017157	53626407	AATACAATTTCTAAAACTATCC			II	
-0.128469	cg00022997	cg00022997	43668467	ATAATTC	31767442	ATAATTC	I	A
0.1741403	cg00023024	cg00023024	37787505	ACCTAAAAAAACCRCRAACTA			II	
0.1136433	cg00024516	cg00024516	15677432	TTTTAAAAAACCTAAACCAACT			II	
-0.133283	cg00027155	cg00027155	36681337	CTAACTAAATAACCACCTAAA			II	
0.1056348	cg00028299	cg00028299	38749489	ATTACCATAAATCCTCCCCTAT			II	
-0.106365	cg00028308	cg00028308	58620505	AAAAAAAATCCTCTCCCCCTCT			II	
-0.15843	cg00033209	cg00033209	35667425	AACCCCTATACCTAACCAAAA			II	
0.1595896	cg00033213	cg00033213	73611364	AAAAATACRATAAACRCCRAA			II	
0.1698296	cg00033490	cg00033490	67770302	ACTATATCATATCATAAAAAAC			II	
-0.161146	cg00035449	cg00035449	51625403	CTCCRATAAAAACCRTCAATAA			II	
0.1002302	cg00036137	cg00036137	62807489	AAAAAAAACCATCRAAAACAA			II	
0.1374837	cg00038020	cg00038020	71732358	CTCTTTCTCTATATAAATTACC			II	
0.1103506	cg00039489	cg00039489	26722425	CCTAAAACTCCCCCTACAATCC			II	
-0.138292	cg00040103	cg00040103	63772389	TCATACAAAATTCCCTACAATT			II	
0.2251326	cg00040530	cg00040530	34668426	ACCCATAACCTACAAAACTAA			II	
0.1024121	cg00045070	cg00045070	61760432	AAAAAACTAAACTTATACCTAC			II	
0.1042157	cg00047694	cg00047694	54689478	ATTACAAAACAAAAAACACAA			II	
-0.363685	cg00051154	cg00051154	70761365	TACCCTATTTTTTAAATACTAA			II	
-0.121884	cg00056433	cg00056433	13810448	ACTACAATAATTTCCACCAAAA			II	
-0.110892	cg00058009	cg00058009	47670413	ACCCTTC	34681339	ACCCTTC	I	C
0.1575409	cg00061551	cg00061551	72748427	AACCATAATAAAAATTTAATAT			II	
0.1187444	cg00063982	cg00063982	53716300	AAAAAATAAATCACTCTATCT			II	
0.1071726	cg00066239	cg00066239	46751326	ATAAAAATAAAATTATACAAA			II	
-0.293885	cg00069771	cg00069771	30764342	CACAACATACAATCCCCACAA			II	
0.112421	cg00083790	cg00083790	46801467	TACACAA	13659322	TACACAA	I	C
0.1164489	cg00084271	cg00084271	36640339	CACCTTCCTATAAACCTTACRA			II	
-0.119091	cg00093478	cg00093478	16757450	CTAAAAT	14611473	CTAAAAT	I	A
0.1357238	cg00095259	cg00095259	60656333	AAATCCAAAAACTTAAAAAAA			II	
0.3647068	cg00098182	cg00098182	47651469	TCCTCTTC	50685321	TCCTCTTC	I	C
0.1252292	cg00101154	cg00101154	30746351	AATTAATCTTAAACTTAACCCC			II	
0.1098738	cg00101227	cg00101227	59747444	ACATTACAAACTTCTTAAAATA			II	
0.1020973	cg00101602	cg00101602	33735361	ACAAATAATTACTTATCAAATA			II	
0.1303455	cg00106565	cg00106565	74721382	CATCCTCAATATCAAACTCRTA			II	
-0.102595	cg00108944	cg00108944	24657395	ACRAAACAAAACTTACTTACRT			II	
0.1258031	cg00118783	cg00118783	71730440	ATAACCA	30680362	ATAACCG	I	A

0.1336879	cg00119778	cg00119778	65649460	CTCTTTCTCTCTCTCACACTTT(			II	
0.139879	cg0012162(	cg0012162(	19779393	AAAACTAATAAAAATAACTTA			II	
0.1602222	cg0012187(	cg0012187(	11641490	AATAACATTATAAAACAATTC(			II	
0.200882	cg0012277!	cg0012277!	50730351	TCTTAAATTCAAATCAAAAAA'			II	
0.1181042	cg0012321<	cg0012321<	16683390	TATCACTCAATTTCCTCATCAA			II	
-0.146608	cg0012490!	cg0012490!	66794348	TAACCAAAAATACACCAATTC.			II	
-0.118647	cg0012499:	cg0012499:	26637438	AATACAA	24755330	AATACGA	I	C
-0.107074	cg0012589:	cg0012589:	31787444	CCAAATCAATATACAATCAAA.			II	
-0.209826	cg0012871{	cg0012871{	64701377	AAAAAATAATCCACACCTTAC.			II	
0.176604	cg0013654'	cg0013654'	12733385	ACAAATAATATACTAATAACT.			II	
-0.366904	cg0014624(	cg0014624(	44697466	AATACTCCACRATAAACCACT.			II	
0.1050526	cg0014953'	cg0014953'	28618375	CTTCCAT.	52810459	CTTCCGT.	I	A
0.1547821	cg0015490!	cg0015490!	59771461	TTTCATA'	71747396	TTTCATA'	I	A
-0.118692	cg0015649'	cg0015649'	74780459	ACTCAAAACTCACTATAAAAC.			II	
0.1478863	cg0015701(	cg0015701(	54695379	CAAATCTTAAACACTCACATT]			II	
0.2536919	cg0015719!	cg0015719!	67794389	AACTCTT'	67704375	AACTCTT'	I	C
0.155105	cg0016370!	cg0016370!	59703408	AATTCAAATTTCCTAACTTTAT			II	
-0.141063	cg0016672!	cg0016672!	55760432	ACCTACT	14676335	ACCTACT	I	T
-0.154305	cg0016724{	cg0016724{	69734324	AACAAAATTTTACATTCAAAA.			II	
0.1303501	cg0016767(	cg0016767(	47678333	ATCCTTATTAAATTCTTATCAA			II	
0.1412137	cg0016791:	cg0016791:	34621465	ACCATACCTATCCACCTACCT/			II	
0.1020706	cg0017109!	cg0017109!	47771303	AAAACTAACCTCATATTACAC(			II	
-0.110827	cg0017260:	cg0017260:	50632452	AAAAATT	73639386	AAAAATT	I	A
0.1523787	cg0018289:	cg0018289:	64744407	CTAAAAAAAACAACTACAATA			II	
-0.240073	cg0018724<	cg0018724<	54743510	CATAAAAATATAAAAACTACA			II	
0.1030963	cg0019288!	cg0019288!	58670408	TTCTTAACAAACCTAAAACAT]			II	
0.1869919	cg0020480!	cg0020480!	64623441	CCCCAAA	47756405	CCCCGAA	I	T
0.1246974	cg0020650'	cg0020650'	70732476	TTTCACATATTTACCCTAAACT			II	
0.1466873	cg0020961!	cg0020961!	27678431	CTTAAAACAAACATTTATTAC(			II	
0.1051298	cg0021085(	cg0021085(	12772328	CTCTTCCCTAAATCATTCTACA			II	
-0.327772	cg0021121:	cg0021121:	12790319	CTACAAA	39622342	CTACAAA	I	C
0.2390722	cg0021203	cg0021203	29674443	CCCAATT	38703326	CCCAATT	I	T
-0.126467	cg0021380!	cg0021380!	71737411	AACACAATATTAAACTAAACR			II	
0.1720481	cg0021862{	cg0021862{	60625336	TTCCAAATACATCCAAATACR/			II	
0.123006	cg0022118!	cg0022118!	54678344	AACAACT	24788422	AACAACT	I	A
-0.108605	cg0022201!	cg0022201!	17719383	CCCTCTCATAACCCAACCACC(			II	
0.1302324	cg0022324!	cg0022324!	50693357	ATCTAAAAAACTCCAAATACA'			II	
0.1018733	cg0023012(	cg0023012(	14662484	ACCCAAA	19720437	ACCCGAA	I	C
0.3416751	cg0023151!	cg0023151!	19627374	CCTACTCTAATAATAAATTTCT			II	
0.2771948	cg0023238{	cg0023238{	35709315	TCATTTAAATCTTCRTTCAAAA			II	
0.1210151	cg0023437(	cg0023437(	48684425	ATATATTCTCTCCCTAAATTAT			II	
0.1018026	cg0023575<	cg0023575<	28719502	AAAACTAACAAATCCAACRTT'			II	
0.1192852	cg0023974!	cg0023974!	51742392	AAAATAAACTCCTTAAATACT/			II	

0.1324437	cg00241002	cg00241002	54771452	ATAAAAC	57737407	ATAAAAC	I	T
-0.126427	cg00241907	cg00241907	43619318	AACTTTA	10732399	AACTTTA	I	C
-0.189757	cg00244920	cg00244920	47710335	TTAAACATTTACCCTATAACAA			II	
0.2170968	cg00247094	cg00247094	22631327	CRCTAAAAACTATAAAACTAA			II	
0.2659728	cg00248861	cg00248861	64710346	CAAACAACTTAACTAATTCCA			II	
-0.16564	cg00253735	cg00253735	60729344	AAAAAAAAAATTTCCRTCTAA			II	
0.2127221	cg00254095	cg00254095	69780487	TAACTCCAAACCTTCTAAACCA			II	
-0.110238	cg00254306	cg00254306	54748461	CCATTTCT	17645336	CCGTTTCT	I	A
-0.110721	cg00255882	cg00255882	63789443	TACTACRAATAAACRTACTATA			II	
0.1379045	cg00256046	cg00256046	73700305	AACCCAAAATTTATACTAAAAT			II	
0.1065856	cg00256329	cg00256329	34663423	ATTATTATATCTACRATATCCA			II	
0.2416773	cg00259849	cg00259849	15806472	TTTTATCTACRCTAAAAATCTA			II	
0.2390068	cg00269725	cg00269725	67766493	AAATTCCTAAACTTCAAATAAC			II	
-0.103467	cg00269800	cg00269800	61752345	ATAAAAAAAATCTTTCAAAAA			II	
0.2057893	cg00271873	cg00271873	11808416	CRCCAAATTTATTCAAACTTCT			II	
0.1118558	cg00272795	cg00272795	74632377	RCCACCTAATATCTACTAAAAC			II	
0.103954	cg00272903	cg00272903	41724510	ATTAATAAAAATTTACTAACAT			II	
-0.150959	cg00274640	cg00274640	47758438	AAACCCTTCRAAAAAACCRAA			II	
0.1139986	cg00275828	cg00275828	19804392	AACTCACTTAAAAATAAACTTA			II	
	cg00279755	cg00279755	13644355	AATACAAAACCCTTCCACAAA			II	
-0.123238	cg00281837	cg00281837	56772418	AAAATAAAAACRACCTAAACC			II	
0.1106268	cg00286102	cg00286102	10622467	ACTAAAAAAAAATCCTAAAAC			II	
-0.182411	cg00287841	cg00287841	34718404	CATTCATCTTTAAACCTACCAA			II	
0.1594026	cg00288598	cg00288598	34722501	TAAAAAA	44649372	TAAAAAA	I	A
-0.264662	cg00290607	cg00290607	64718396	CTCCTTCT	62628499	CTCCTTCT	I	A
	cg00292662	cg00292662	13644450	AACCCRAATAAAACCAAAACC			II	
0.1247441	cg00294550	cg00294550	52807349	AAAAAAAACTATAAATAACTT			II	
0.1696367	cg00295418	cg00295418	41681387	TACTAAAATAATTTTAAATACA			II	
0.1040363	cg00296903	cg00296903	27749328	ATTATAATTCTAACTCCTCCCC			II	
-0.222302	cg00297876	cg00297876	24757500	CAAATTCRCTCAATAAAAATA			II	
0.1057237	cg00302763	cg00302763	60641446	ACAATTAATTCTCTTAACTATC			II	
0.1097211	cg00304520	cg00304520	27747375	AACTACC	37645353	AACTACC	I	T
0.1265446	cg00311883	cg00311883	43616362	CTTATTA	62666317	CTTATTA	I	A
0.1097369	cg00314000	cg00314000	54769436	ATTCAATATACATTAAAATCTA			II	
-0.111965	cg00318166	cg00318166	30729329	TACTAAATCCTAAAAATAAAA			II	
-0.128941	cg00318631	cg00318631	64702450	ACTATAATACCCATAAAAAAT			II	
0.100657	cg00321007	cg00321007	34743356	ACCTCTCAATACCAAATCCAA			II	
0.1546537	cg00321709	cg00321709	29758495	CTCTAAAACAACAACAATACC			II	
-0.189233	cg00324979	cg00324979	11750373	ACATCAC	59799481	ACATCAC	I	A
-0.165935	cg00325125	cg00325125	73798400	TATTTAC	39785417	TATTTAC	I	C
0.1080418	cg00325139	cg00325139	31648385	TAATTACCATACTTAATAAATC			II	
0.1541798	cg00325917	cg00325917	14694367	TCATAAAATTCCTAAAAATACA			II	
0.1595393	cg00328219	cg00328219	72703382	TTTTATAATTATTTTAATTTATT			II	

0.1107691	cg00328597	cg00328597	38675330	AAAAAAAATCCTAATCATAAT			II	
-0.151364	cg00328916	cg00328916	72644443	TAATAAATATTAATTTTATACC			II	
-0.154969	cg00339488	cg00339488	50602324	CAACTAT	21801407	CGACTAT	I	A
-0.131416	cg00341060	cg00341060	18773370	AACCTAT	41686495	AACCTAT	I	C
-0.12384	cg00346623	cg00346623	47697349	TCTTAAC	29663318	TCTTAAC	I	T
0.1241249	cg00347620	cg00347620	51782356	TTCCAAA	65801309	TTCCAAA	I	C
-0.187541	cg00348031	cg00348031	20645464	AACAACTATAAAACCAAACTT			II	
0.122436	cg00352576	cg00352576	46794399	CTCTCACTACCATATTCCAATA			II	
-0.126273	cg00353407	cg00353407	29767306	CTCTCTAAACTTTTCAAAAATA			II	
0.1677401	cg00354381	cg00354381	53661303	TAATTAATCAATACAATCTAAC			II	
	cg00363750	cg00363750	73802349	CCCTATACTCTATCCACTTTCC			II	
0.1729915	cg00366603	cg00366603	71632347	TACTATCTACATAAAATTAAAT			II	
0.109563	cg00369056	cg00369056	22723346	CCTCATATTATAAATTTCCAAA			II	
0.1078323	cg00370414	cg00370414	68769470	TCRATAAAAAACCCTACAAAC			II	
0.1004608	cg00371061	cg00371061	70750478	CRTATCTAACCTCCCCAATACT			II	
0.1692826	cg00373436	cg00373436	35689309	TCTACCAACCCATTCTTAAACC			II	
-0.129686	cg00374016	cg00374016	34614467	AAAAACRATAAAATCTACAAA			II	
-0.127223	cg00381755	cg00381755	35726399	CTAAAAA	51656485	CTAAAAA	I	C
-0.140437	cg00384701	cg00384701	45767476	CRAAAAAAACRTCTTTCTCCR			II	
-0.120148	cg00390191	cg00390191	12611477	ACACTATCACTCATAAATTATA			II	
	cg00394712	cg00394712	24737432	TTAATATTATACCCATAAATCA			II	
0.1287061	cg00398048	cg00398048	15783450	CTAAACAAACATTCCAAACAC			II	
0.105296	cg00399951	cg00399951	24614302	TATACAAAATCCAACCCCCCR			II	
-0.104764	cg00401101	cg00401101	36612418	TTATTTA	59687450	TTATTTA	I	T
-0.105838	cg00403478	cg00403478	62610321	ATACAAAAACACTCTTTACAC			II	
-0.13246	cg00404187	cg00404187	59806369	CAAACTACATATTATAATATTA			II	
0.1135794	cg00406010	cg00406010	60735327	AATCTCRTATTAATAAAAATAT			II	
-0.114525	cg00408231	cg00408231	57607304	ATCTCTTA	33655303	ATCTCTTA	I	C
-0.135403	cg00410898	cg00410898	41650376	AACTTTTACAAACTAAAAACC			II	
0.175774	cg00413352	cg00413352	40637333	CRCAACAAAAAATAACCCCRA			II	
-0.339838	cg00415389	cg00415389	36702431	AATCTACTTAATTTTTAAATAT			II	
-0.100194	cg00428457	cg00428457	36769351	CCCAAAA	65619420	CCCAAAA	I	A
-0.156608	cg00436969	cg00436969	51693491	ATCAAAAATAATAAAATAAAC			II	
0.1154762	cg00440797	cg00440797	46608309	TTAATCCAACTAACTCCCTAAA			II	
0.1938849	cg00450029	cg00450029	70611456	AAATAAATATATCCTATACATT			II	
-0.316587	cg00456343	cg00456343	16712478	CTCAATACCTTAAAAAACCRA			II	
0.1202003	cg00458932	cg00458932	69765306	ATTCCTATTATAACTATCAAAA			II	
-0.138306	cg00459623	cg00459623	19772395	CAACAAA	27705498	CAACGAA	I	A
0.1037564	cg00463746	cg00463746	55797393	AAAAATACCTTAACAAAAAAC			II	
0.2371812	cg00466116	cg00466116	36716493	AAAAAACATTCAAATCTAAAT			II	
0.154954	cg00467278	cg00467278	57640486	TCCACAA	71694449	TCCACGA	I	A
0.1578017	cg00472840	cg00472840	20810380	AACTTTATATCCCCRACAATA			II	
-0.171104	cg00474091	cg00474091	13684467	AACCRAATTCTACAAAATCAA			II	

0.304319	cg0047437	cg0047437	72662453	TAATCAATAAAACTCCCTAAA			II	
-0.103876	cg0047847	cg0047847	38625510	RAAAATAAAATCATAAAACRA			II	
0.1308355	cg0047982	cg0047982	45616421	CTAAACTAACACATTCACRTT			II	
0.1191266	cg0048625	cg0048625	67693416	CTTCCAACAAATATAACACCT			II	
-0.107262	cg0048882	cg0048882	41699391	AATATCC	28727411	AATATCC	I	C
-0.156614	cg0049330	cg0049330	45680342	AAAAAA	44609483	AAAAAA	I	A
0.1205994	cg0049790	cg0049790	62600457	CTATACAACTCAATCAAAACT			II	
0.1267453	cg0049913	cg0049913	38801390	TCTATAACTTTCTCATCCCTAA			II	
0.1150294	cg0050002	cg0050002	48607448	CTAAACTTCAAAAAACTTCAA			II	
0.1094598	cg0050500	cg0050500	47660336	TAAACAAATATAACTACCRAA			II	
-0.351297	cg0050705	cg0050705	33615313	AAACATCTCAAACACTAATAA			II	
0.1207627	cg0051032	cg0051032	60780470	CRATACTCTTCTAATACTATCC			II	
0.1065352	cg0051071	cg0051071	38763374	CTCRAAATAATAACACTTCCT			II	
0.1184269	cg0051133	cg0051133	20665459	CTCACCRAAAAACAACAACAA			II	
0.1018859	cg0052360	cg0052360	70747313	CCAAAACAACAATTATAATAT			II	
0.1524791	cg0052362	cg0052362	39768400	AAAAATAAACACCATTAAAAT			II	
0.1182347	cg0052437	cg0052437	56689304	AATAACTAAAAACCTAATACT			II	
-0.269687	cg0052582	cg0052582	73696304	TCCTAAA	22732478	TCCTAAA	I	T
0.1423455	cg0052974	cg0052974	39696318	TTAACTAAAAATTTACTAAAC			II	
0.4508315	cg0053212	cg0053212	61800426	AAATAAACTTAAATCACAAAA			II	
-0.416903	cg0053421	cg0053421	61781303	ATCAAA	50644369	ATCAAA	I	C
0.104932	cg0053936	cg0053936	59744470	ACAATATACCATCTAAAACCC			II	
-0.277315	cg0054029	cg0054029	37809366	AAAAATAAAAAATCTACCCAA			II	
0.150685	cg0054403	cg0054403	23632499	AATAAAATTATACTAAATACT			II	
0.375331	cg0054675	cg0054675	74625369	AATTTATCAATCTTCCTATAAA			II	
0.1035224	cg0055508	cg0055508	42616447	AATACTAAAAACTTTACTCCA			II	
0.1587534	cg0055543	cg0055543	12606324	AAAATATTACAAAAATCAAAT			II	
	cg0056226	cg0056226	13644472	AAATCACAAAACTAATAAATA			II	
-0.104217	cg0056226	cg0056226	74692473	AAAACA	62764304	AAAACA	I	C
0.1095651	cg0056335	cg0056335	54631509	AAAAAAAATCCCAAACTACAC			II	
-0.110975	cg0056382	cg0056382	42786475	AAAAAA	62697453	GAAAAA	I	A
0.2847151	cg0056618	cg0056618	64810375	ACAAAAAAAACAACRTTAACR			II	
0.1028307	cg0056698	cg0056698	54768470	CCTACTACACCRACAACTCTA			II	
0.1702462	cg0056910	cg0056910	11653362	ACCTTCCTAACTACTTCCTATA			II	
0.1091654	cg0057063	cg0057063	17775315	TAAAATACCTTTCATAAAAT			II	
-0.101849	cg0057151	cg0057151	45708350	AAATATACTCAATTTATTATA			II	
-0.170545	cg0057258	cg0057258	55740317	CRTAACCTAATACRTAACAAT			II	
0.1179494	cg0057341	cg0057341	69654380	ATTACRAAAACCCATTCTCAA			II	
0.163762	cg0057564	cg0057564	16668481	CTAATACTTTATCCACRAATC			II	
0.1013348	cg0057613	cg0057613	22668323	TACTTCCTAAAATTTCCRACA			II	
-0.126313	cg0058652	cg0058652	54681481	TCCATCT	17654386	TCCATCT	I	A
0.1014317	cg0058730	cg0058730	23781313	AATATTCAACAAATCAATAAA			II	
0.1003872	cg0058734	cg0058734	39716491	AAAACCTAAAAAAAAACCAC			II	

0.2600051	cg0058794	cg0058794	55710466	CTACAAA	28715476	CTACAAA	I	A
0.1410605	cg0058892(	cg0058892(	70627410	TTAAATCCTCCAAACTTCTTAA			II	
-0.117416	cg0059505	cg0059505	15623412	TTCATTCTAAAAACCRTTCCAT			II	
0.1191094	cg0059820	cg0059820	37644350	CTAATACCTCTACCTAATACA			II	
0.147006	cg0060145(	cg0060145(	48675301	TCCAACC	10730370	TCCAACC	I	A
0.1111879	cg0061275	cg0061275	14741434	ACAAAACTCCTAAAATCTCAA			II	
-0.137818	cg0061657	cg0061657	49789428	TCTATACACCATACAACCTAA			II	
0.1063018	cg0062155	cg0062155	41739321	AAAAATCTCACRTCCTACTAT			II	
-0.115918	cg0062166	cg0062166	67737500	ATCCAATATTTCCATTAAACR			II	
0.103197	cg0062821	cg0062821	55631375	CTCAAAAAAACRACTAAACAC			II	
-0.169023	cg0063132	cg0063132	61711456	TAAAAACTAATTTCCRACCCT(			II	
0.2296702	cg0063175	cg0063175	42626386	CTCCAACTCTACCAATACRAA(			II	
-0.269351	cg0063187	cg0063187	61665304	CAATTACCTCTTCCTATTATCT			II	
0.1258936	cg0063465	cg0063465	52795423	AATAATC	41801408	AATAATC	I	C
0.1164856	cg0063534	cg0063534	45612424	CCTAATAATATACTTCTTAACT			II	
0.1182786	cg0063619	cg0063619	62744391	CCTCTCTCTTCTTTCRTATATA			II	
0.1193676	cg0063802	cg0063802	71783431	CTATATTCTCTCCTTCCAAAAT			II	
-0.167911	cg0063961	cg0063961	64697321	AAAAAACAAAAATTTCTAACT			II	
0.1049242	cg0065555	cg0065555	27604337	AAATCATATATATCCCTCCATC			II	
0.1404113	cg0066445	cg0066445	16703404	CAAAAATAACCCTAACTTCTA			II	
-0.118326	cg0066778	cg0066778	36621334	TTCATAA	42661429	TTCATAA	I	C
0.1132724	cg0066794	cg0066794	51734472	AAAAAAAAACATCCTCCTTCC			II	
0.1547226	cg0066821	cg0066821	47690491	CAAAACACATCTACCTAAAAA			II	
0.113979	cg0067533(	cg0067533(	68697397	TCTTCCTACTTTCCAATATCCC			II	
-0.137984	cg0067962	cg0067962	47712346	CACCCAAAAAATTCCRTAACR			II	
0.107179	cg0069194	cg0069194	53710382	AACCAATAAATTACATCTAAC			II	
0.1421484	cg0069404(	cg0069404(	56600501	CAAAATTAAATAATCRTCTAT(			II	
0.1861162	cg0069435	cg0069435	61688483	ACTCAATAATTAAATCRAAAA			II	
0.1674812	cg0069604	cg0069604	51669430	TACTATAAAAATCTAAAAACT			II	
-0.171947	cg0069877	cg0069877	31681444	AAAAATA	63692413	AAAAATA	I	T
-0.121741	cg0070096	cg0070096	15679389	ACACCAACAACCAATCAAAAA			II	
-0.354203	cg0070256	cg0070256	19740489	TTTTTCTAAACTACTTACATAA			II	
0.1341255	cg0071826	cg0071826	53605491	TATATATCTAACTATACRTACC			II	
-0.12182	cg0072058	cg0072058	60679475	TAAACAC	73642307	TAAACGC	I	A
0.1455817	cg0072431	cg0072431	60657423	TATTCTATATAACCCTCACACT			II	
0.1181018	cg0072514	cg0072514	49647457	AAAAAA	13676315	AAAAAA	I	C
0.1473785	cg0072777	cg0072777	72788303	TAACCATTCCAAATACTATCA			II	
0.1164738	cg0072970	cg0072970	15681478	RTAAAAACCCACRAAAATAAT			II	
0.1545759	cg0073397	cg0073397	58790502	AATAATTACTTAAAACTCAAC			II	
-0.101301	cg0073588	cg0073588	19649370	AAAATCC	23759389	AAAATCC	I	A
-0.1043	cg0073884	cg0073884	73775371	CCAATCT	38801335	CCGATCT	I	A
-0.202024	cg0074051(	cg0074051(	31607333	AACRAAAACTACAAAAAATAA			II	
0.1028617	cg0074585	cg0074585	28753437	ACAAATTTACAAAAAAATCCT			II	

0.1032431	cg00747477	cg00747477	73744465	TCAACCATTTCCACAAAACCA			II	
-0.237341	cg00747650	cg00747650	11786475	CTTAATT	19759505	CTTAATT	I	A
0.1359225	cg00750481	cg00750481	35758454	AAATATCATAAACACTATATA			II	
0.1265295	cg00753548	cg00753548	23677356	AAATAAATATTCCTCCATATTC			II	
0.1281634	cg00753924	cg00753924	13628431	ACCTATAAACATAAACACTAA			II	
0.1165502	cg00754989	cg00754989	16601458	TATCTAATTTTTAAATTAAAAA			II	
0.150252	cg00759058	cg00759058	52808361	AACTTATTCTTTCCTAAAACRC			II	
-0.132346	cg00762830	cg00762830	66714317	CTACTAT	68648306	CTACTAT	I	A
0.1149313	cg00764981	cg00764981	12689492	AACTCAAATATCTCTAACRTA			II	
0.1034604	cg00768179	cg00768179	17658469	AAACRCAATCCAATTATAATA			II	
0.1468759	cg00777560	cg00777560	58611311	ATAAACTTCTATACTAAAATC			II	
0.1185293	cg00779056	cg00779056	11738337	AACAACC	36742405	AACGACC	I	A
-0.103126	cg00783137	cg00783137	19738414	AATTTTAACAATTTACTCACCA			II	
-0.107973	cg00784581	cg00784581	47661355	CAAAACA	23706371	CAAAACC	I	A
0.1310521	cg00785941	cg00785941	52804397	AATATATAAAACAAAACCCTT			II	
0.1006851	cg00787996	cg00787996	72773359	AACAACTCCACCTCTATAATA			II	
-0.139687	cg00788177	cg00788177	10758443	ACAACAA	27641454	ACAACAA	I	C
0.1523997	cg00793935	cg00793935	36619332	TTTAAAC	72628509	TTTAAAC	I	A
-0.184425	cg00793992	cg00793992	29629398	AAAATAATAAAAATTTCAACC			II	
0.1681027	cg00794911	cg00794911	25661471	AAAAAATACCRTCAAAACAAA			II	
0.1118464	cg00795205	cg00795205	56635320	AACTTACTACTAAAATATAAA			II	
0.1028429	cg00804245	cg00804245	54773334	TCCTAAA	45725488	TCCTAAA	I	A
0.2390398	cg00814218	cg00814218	64602355	TAACRTATATACATAAAAAC			II	
0.1227916	cg00816515	cg00816515	14806404	TCAAATCCTCAATTTTAAAAA			II	
-0.198072	cg00821186	cg00821186	70683476	AAACAAAACTCCACTATACRA			II	
-0.132721	cg00822030	cg00822030	18798349	AAAACTC	10688394	AAAACTC	I	A
0.1044073	cg00829645	cg00829645	61646498	ATCCAAA	38687433	GTCCAAA	I	A
0.1015578	cg00832136	cg00832136	62629352	ATCACAAATATTACCAAAATA			II	
0.1670972	cg00836101	cg00836101	33668367	ACCRCAAATAACTTACAAATC			II	
0.11423	cg00837899	cg00837899	55725467	AAACTCCAAAACAACAAATAA			II	
0.107611	cg00838423	cg00838423	16654482	AAACRAAAAACATTATCCTAC			II	
-0.10402	cg00843634	cg00843634	68647386	AAACACAACACTAAACATAAC			II	
0.102256	cg00848374	cg00848374	19743484	ATTTAATAATATTTTCATCAAA			II	
0.1161994	cg00851518	cg00851518	47624412	AAAACRAAAAAACCRAAACCC			II	
0.1027896	cg00852603	cg00852603	47669404	AAACAAA	30621348	AAACGAA	I	A
0.1325734	cg00853940	cg00853940	60716487	AAAATAACCAAATTAAATATT			II	
0.2223739	cg00859877	cg00859877	32640506	TCAACTCCTAATAAAATAATA			II	
0.1865849	cg00861214	cg00861214	49625401	CCCCAATCTTTTTAACACCTAA			II	
-0.137516	cg00864171	cg00864171	52799325	CAACAAATAAACAACCTCCCC			II	
-0.223575	cg00869309	cg00869309	71621326	ATATAACRAAATACCATACTC			II	
-0.158724	cg00873601	cg00873601	18668468	ATACCTT	60679306	ATACCTT	I	A
-0.100771	cg00876757	cg00876757	43778390	AAAACAA	41784387	AAAACGA	I	A
-0.137596	cg00878163	cg00878163	39764486	ATCAAAA	58726385	ATCGAAA	I	A

-0.113931	cg00879218	cg00879218	19790496	RCATTATCAAAACTCTCAAACT			II	
	cg00879497	cg00879497	49788354	TATCCAC	13644469	TATCCGC	I	A
0.2036033	cg00879541	cg00879541	47671476	TAAAATTATATATTTTTAACCA			II	
0.4914707	cg00881894	cg00881894	68677465	ATTCCTTTTCCCCTCATCCTTAT			II	
0.1185193	cg00886571	cg00886571	15612500	CAAAAACTATACTAAAAACCC			II	
0.1098151	cg00888758	cg00888758	24721332	CCTAAAAAAAACCATTATTCC			II	
0.1008365	cg00890070	cg00890070	63730395	TAACATCATACCATCCATATTT			II	
-0.105769	cg00892368	cg00892368	48731416	AATCCAC	51671432	AATCCGC	I	A
0.1123998	cg00900735	cg00900735	29702359	TCCAATT	14789326	TCCGATT	I	T
0.1084806	cg00905312	cg00905312	64770334	TACCAAAAACTAAACACTTAC			II	
-0.129192	cg00905524	cg00905524	33623413	TAACACA	24656365	TAACACA	I	C
-0.106461	cg00905746	cg00905746	62698386	AAACCTAAAAATCCAAAAAAC			II	
-0.102257	cg00908601	cg00908601	70794326	ACACTTCCCCACTCCAATCTAA			II	
0.1540623	cg00913954	cg00913954	72679382	CCCCTTCTATCCAAACAAAAAT			II	
-0.356263	cg00918181	cg00918181	70622484	AAATACACAAACCCRAAAAAT			II	
0.1143639	cg00919126	cg00919126	14642320	AAAACACCCTTTACCTTAAAA			II	
0.1234062	cg00920668	cg00920668	36693338	CACCCAA	23620361	CACCCGA	I	T
0.1348595	cg00925087	cg00925087	70796393	CTTTACCACAAATATTAAAACA			II	
0.1536998	cg00926420	cg00926420	61804339	ATAATTA	73696387	ATAATTA	I	T
0.2038003	cg00939682	cg00939682	23660333	CAAAAACCAATCTTCTTAATAA			II	
0.2610922	cg00944631	cg00944631	33644501	ACTCATTCTCATATATCTTAAA			II	
0.1448193	cg00947782	cg00947782	22655509	ACAACAC	11725434	ACAACGC	I	T
-0.112344	cg00949980	cg00949980	23653380	ACACCAT	50799380	ACACCAT	I	A
0.1214544	cg00952789	cg00952789	20734465	CAAAAAAAATCCTACTCACAC			II	
-0.138874	cg00955780	cg00955780	48794300	TTAAAAAAATATAAACRTATCT			II	
-0.103909	cg00956490	cg00956490	30773316	RATACTACTAAAATATATTCTC			II	
0.152703	cg00957698	cg00957698	27697394	TAATAAAAAACTAACATAAAC			II	
0.1097345	cg00968167	cg00968167	41790387	AAACTCCTTATTTAAAAAAATA			II	
-0.100932	cg00968771	cg00968771	64794380	CTATACC	16624467	CTATACC	I	A
-0.135772	cg00970279	cg00970279	38750401	ACAAAAT	43783437	ACGAAAT	I	C
0.1110156	cg00974523	cg00974523	12731470	AAACTTCTCCCCTATATATATC			II	
-0.107359	cg00974579	cg00974579	37653347	CATAAAAATCRACTTTAAAACT			II	
0.1652674	cg00975746	cg00975746	57691312	TAATAATAAACATAATTACCTT			II	
0.1732812	cg00978846	cg00978846	52680431	ATTCCTCAAATCCACTCTAAAT			II	
-0.13577	cg00979288	cg00979288	64652323	CCAACAC	10616422	CCAACAC	I	A
-0.180338	cg00981879	cg00981879	15731476	AAATACC	28704329	AAATACC	I	C
-0.134868	cg00982984	cg00982984	48702333	AAAAAAACCCCAAAATATACA			II	
0.1219094	cg00983697	cg00983697	51787353	ACAAAAAAACAAACTAAACAC			II	
0.1101105	cg00983705	cg00983705	22623502	CRCTCCAAACAACACTAACTA			II	
0.1005041	cg00985388	cg00985388	36700401	CCTACACTTAAAAAATACTTCC			II	
-0.202152	cg00988056	cg00988056	37707449	AAAAAAA	31720502	AAAAAAA	I	A
0.1080514	cg00990769	cg00990769	68787438	AAACCCCTCACTACCTAAAAC			II	
0.1017399	cg00993830	cg00993830	67633457	TAATTTAAAATAATATTCCTTT			II	

-0.113426	cg00993903	cg00993903	26725474	CCTTTTTCCTCAAAATTTTATT			II	
-0.157505	cg00995368	cg00995368	49810395	ACCTATATCAACTTATCCCCCT			II	
0.1046425	cg00997251	cg00997251	40617310	TTACATTAACTAATTACTCACC			II	
0.1628344	cg00999163	cg00999163	34802473	ACCAACA	57752377	ACCGACC	I	C
-0.108109	cg00999638	cg00999638	58704438	TAAATATTACAACTCTTAAAA			II	
-0.130151	cg01000657	cg01000657	67673486	AAAAAA	35768392	AAAAAA	I	A
0.1214254	cg01001314	cg01001314	49793311	ACTTAAACCTAAAACCTTCTA			II	
-0.101604	cg01001959	cg01001959	57762422	CTTTACACTCTTCTACCTTCCC			II	
0.1059879	cg01005180	cg01005180	72747446	AACCTAAATCATAACTATAAA			II	
0.1286917	cg01005425	cg01005425	13730504	TTTTTCTTTTTAATTTCACAAA			II	
-0.106747	cg01005486	cg01005486	49765459	AAAACAAAAACTACTCTTAAC			II	
-0.107685	cg01006802	cg01006802	39651497	AAATTAA	64691402	AAATTAA	I	A
0.2150862	cg01013031	cg01013031	35631361	AAATTAC	35690419	AAATTAC	I	C
-0.186061	cg01015662	cg01015662	21666465	TTATAAA	26648376	TTATAAA	I	C
-0.172106	cg01016092	cg01016092	21694469	CCCACTCTAATCCTTAATTTTC			II	
0.1008208	cg01022117	cg01022117	17782395	AAATACC	42638353	AAATACC	I	A
-0.115597	cg01022189	cg01022189	25786466	AAATCTAAAAAAAATCCCTTA			II	
0.167631	cg01022670	cg01022670	15660474	CTAATAACTCAACCAATATAC			II	
-0.112289	cg01023242	cg01023242	67743501	TCCCTTTTAAAAATAACATCC			II	
0.1071486	cg01023703	cg01023703	33783394	AAACTTAAAACAAAACTAAAA			II	
-0.157893	cg01025095	cg01025095	68672467	CTATTAA	64763345	CTATTAA	I	C
-0.202844	cg01029331	cg01029331	73787312	CACATTA	14657451	CACATTA	I	C
-0.102368	cg01029685	cg01029685	63766436	AATATTTCTTCAAAAATTCAA			II	
0.1164579	cg01031312	cg01031312	11690361	TAAAAAAATTCCAATACTACT			II	
0.1480042	cg01034813	cg01034813	28693312	TACCTTTCCRTAAACAATAAA			II	
-0.175848	cg01042202	cg01042202	43649477	ACCATCA	21654418	ACCATCG	I	T
-0.125421	cg01048272	cg01048272	66719465	TAACCATTTATCTTTTAACTTT			II	
0.1095443	cg01055691	cg01055691	63796358	CAATAACCTTATCTAATTTATC			II	
-0.108077	cg01055696	cg01055696	51629480	AATAACC	25614359	AATAACC	I	C
-0.131384	cg01056467	cg01056467	10629329	CAAAACA	16649394	CAAAACC	I	C
-0.159218	cg01059116	cg01059116	69731430	AACTTTCATTTCCTATTCCAAT			II	
-0.142821	cg01059385	cg01059385	61780487	ACAAAAAACCCRAAACAAAA			II	
-0.101791	cg01059432	cg01059432	37645427	ATCAAAC	38781310	ATCGAAC	I	A
0.1509777	cg01061164	cg01061164	34769367	ATCTAAA	25702481	ATCTAAA	I	T
-0.118267	cg01061520	cg01061520	61760464	TAACCTATAAATAAACRAAAA			II	
0.1024155	cg01062539	cg01062539	57764394	TACATAA	21718483	TACGTAA	I	T
0.1416259	cg01062942	cg01062942	52752434	AAAAACTTCRCRATTACCTAA			II	
0.1409029	cg01063088	cg01063088	53684349	CTTCCRAAAACAACRACRATA			II	
-0.14839	cg01064265	cg01064265	58621422	ATACAAA	51611437	ATACAAA	I	A
0.119834	cg01072388	cg01072388	62787373	ATAATACTTTCCACATAAAAA			II	
0.2315002	cg01074083	cg01074083	17770500	ACTCTAATCTTCTACTAAAAA			II	
-0.469878	cg01081584	cg01081584	63773393	ATACCTATTTATACAAAACTC			II	
0.1284347	cg01085399	cg01085399	34667319	ATTCAAATAACTTACTAAAAT			II	

	cg01097444	cg01097444	13617312	AAAAAAC	37610325	AAAAAAC	I	A
-0.128466	cg01101663	cg01101663	57773374	CCACCACCAAATAAACAATAA			II	
0.1173881	cg01101677	cg01101677	35652338	CATTTCTAAAACATCTCCCAAC			II	
-0.400459	cg01105403	cg01105403	58625305	CRCTAAAACCATTTTCTATCCC			II	
0.1156125	cg01108633	cg01108633	52758389	TTAAAATCAAAAACTACTTTTA			II	
0.1487882	cg01114138	cg01114138	71625399	CTACTAAATACCRTAAAACAC			II	
-0.164344	cg01117274	cg01117274	24632368	AAATAAAAACTTTAAAAAACC			II	
-0.281204	cg01118640	cg01118640	72739319	TAAAAATCAAAAATCTAAAAT			II	
-0.119206	cg01124132	cg01124132	53638343	ATTAAATACTATATAAAATTAC			II	
-0.145984	cg01127608	cg01127608	24715331	TACTTCCRAAATCRAAAACTAT			II	
0.1207551	cg01128042	cg01128042	18724455	AAATCTATTCTCAACATTTACT			II	
0.2399866	cg01139696	cg01139696	45607388	TCCTACCTTAATCTCCCTCAAA			II	
0.1067752	cg01139699	cg01139699	16700378	ACAACTATTTATTAAATAACCA			II	
0.1332062	cg01152624	cg01152624	32644411	TTTCCATTTCACTAAATACTTC			II	
0.2167035	cg01153376	cg01153376	45738320	CAAAAAA	50626330	CAAAAAA	I	A
0.1145931	cg01154336	cg01154336	44652369	AAAATAACCATACTCTTCTCCC			II	
0.1335649	cg01161055	cg01161055	62747307	TTCCAAACACTAACTATATTTT			II	
0.1163456	cg01165909	cg01165909	35653429	ACATAAAATCCTAACTAACTA			II	
-0.237185	cg01175142	cg01175142	47769365	AACTAAAATACAACCTTAAAA			II	
0.1170594	cg01182076	cg01182076	37774380	TCRATAAAAAATATAACTATA			II	
0.1173925	cg01185640	cg01185640	22706469	TTCTATTTTCACRATACTAATA			II	
0.1338679	cg01190919	cg01190919	14707477	AAAACCA	27750351	AAAACCC	I	A
0.1153925	cg01191154	cg01191154	50612384	TAAAAAACCAAACAACTCACC			II	
-0.132302	cg01193368	cg01193368	21647360	AAAATAT	26755371	AAAATAT	I	A
0.109475	cg01194073	cg01194073	72697401	AAACCAA	32694336	AAACCGA	I	T
0.1183261	cg01194765	cg01194765	24770508	AAAAATCATAATTAATTTCTA			II	
0.1012988	cg01196336	cg01196336	64764433	ACAAAAAAAAACCTACCTCCT			II	
-0.132959	cg01202519	cg01202519	59676304	TAAAAAA	47600463	TAAAAAA	I	C
0.1177978	cg01203766	cg01203766	74635411	AACAAAACCCTAATCCCTTTA			II	
-0.119595	cg01215761	cg01215761	23623339	CAAATAA	62672339	CAAATAA	I	C
-0.205462	cg01221582	cg01221582	24784320	ACCTAACCTACCRAATAAAAT			II	
-0.293126	cg01228941	cg01228941	55600356	AATCAAA	35613357	AATCAAA	I	A
0.2914732	cg01230320	cg01230320	18649346	TAAAACCAATCAAAATTTTAA			II	
0.1364021	cg01230639	cg01230639	72809365	TTTTTTATTTTAAACATATCAA			II	
-0.113418	cg01231921	cg01231921	35765480	RACCCRACAAACACCATAACC			II	
0.1679514	cg01232331	cg01232331	11624391	AACCTACATAAACTAACTAAA			II	
-0.11084	cg01232511	cg01232511	34701365	AAACCAC	35621479	AAACCGC	I	C
0.1552874	cg01236565	cg01236565	38743488	TCAAAATTTCTTTCTACTTTAT			II	
0.1624808	cg01245224	cg01245224	70663375	AAAAATACCATAACAATATCC			II	
-0.138841	cg01248745	cg01248745	44690486	TTCCRAAATCAAAATCTAAAA			II	
0.2978484	cg01254303	cg01254303	32766403	CTACAAA	50639305	CTACGAA	I	A
0.1137398	cg01257780	cg01257780	25672372	CTACTTCCCACCATAAAAAAA			II	
0.1911996	cg01264919	cg01264919	73763380	ACTTCCCRACATACCRCTAAA			II	

0.1242095	cg0126921	cg0126921	37698464	RACCCTACAAAAATACATTAT			II	
-0.116436	cg0127000	cg0127000	52631391	TAAACTC	43650379	TAAACTC	I	C
-0.167414	cg0127490	cg0127490	52722315	TAAAAAAACATTTACTATATT			II	
-0.121921	cg0128333	cg0128333	61774489	ACAAACACTTTAATTTTCCTCC			II	
0.2141066	cg0128428	cg0128428	64720495	CAAACATAACTCATTATATAT			II	
-0.106353	cg0128550	cg0128550	50703500	AACCCTAACCTTAAACTATAA			II	
-0.113453	cg0128592	cg0128592	28747346	CAATCAA	62630489	CAATCAA	I	C
0.1183712	cg0128668	cg0128668	67740487	CCTAAAA	61726375	CCTAAAA	I	C
-0.100332	cg0128700	cg0128700	71733390	ACCATTA	38620464	ACCGTTA	I	A
-0.104305	cg0128954	cg0128954	36615507	AATTTTTCTACCTACCTACAAA			II	
0.1202017	cg0128987	cg0128987	24806427	TAAAAAAATACCTTTAATTAA			II	
0.1113836	cg0129253	cg0129253	68748466	CRAAACATTTTCTAAACAAAC			II	
0.1217001	cg0129281	cg0129281	50683422	TTAAAAACTAAAAATATTACT			II	
0.114005	cg0129428	cg0129428	63706392	AATAACRAAAAACRACTAATT			II	
-0.118378	cg0129564	cg0129564	63606406	CCAAAAATAAATAAACAAAA			II	
0.1465809	cg0129810	cg0129810	35734489	TTTCCTCCCCCTTCTACTACCT			II	
0.1081485	cg0129867	cg0129867	12736491	CRTAAAAAACATAATTTCATA			II	
-0.264291	cg0129891	cg0129891	35809352	ACRTACTAAATAAACRTACTA			II	
-0.118624	cg0130128	cg0130128	10771424	CRTAAATATTTATTTACTACCT			II	
-0.112596	cg0131647	cg0131647	67705342	CCAAATTCTTCCCAAACRAAC			II	
-0.114702	cg0131818	cg0131818	35704452	TAACTTA	62802401	TAACTTA	I	C
-0.132743	cg0132057	cg0132057	46731406	ACCTCCC	41796418	GCCTCCC	I	A
0.148701	cg0132196	cg0132196	47774394	TTAATCCACATATATCCCAAA			II	
0.1382241	cg0132426	cg0132426	30671344	CAAACRCRATAACTCACACCT			II	
0.1136073	cg0132546	cg0132546	40647340	CATCTAATACATTTAAACCAT			II	
-0.186718	cg0132642	cg0132642	32630496	TTAAATATTCTAATAATTAATA			II	
0.18383	cg0132969	cg0132969	30690303	ACCATACCAACTACTCRTTATA			II	
0.1723318	cg0134180	cg0134180	23754361	ATCTAATACTTTTACAAACAA			II	
0.1283213	cg0134858	cg0134858	55740334	CAACCAA	17663399	CAACCAA	I	C
0.2766204	cg0135080	cg0135080	47628373	TTAAAAACTTTAATTTTTACAT			II	
0.1141881	cg0135478	cg0135478	34742332	CTAATCACCAAAAAAACCAAC			II	
0.1013616	cg0135982	cg0135982	28651441	ACAATAA	46697474	ACGATAA	I	A
-0.15861	cg0136922	cg0136922	20742450	AATTAATCTAAACAATAAAAC			II	
0.1021779	cg0136989	cg0136989	55620332	AAATATCCTAAAAAAATTTTT			II	
0.101056	cg0137142	cg0137142	44612418	TAATAACTCCTACCTTCTCATA			II	
0.1073404	cg0137726	cg0137726	55689445	CCATCAA	71681318	CCGTCGA	I	A
0.1334915	cg0138182	cg0138182	15732482	AAAAACTTACTTACTTACTATT			II	
-0.149393	cg0138286	cg0138286	19729390	CAACAAA	55803378	CGACGAA	I	A
0.1612814	cg0138595	cg0138595	28748320	TCAAAATAAATATTATATACC			II	
0.1092978	cg0138790	cg0138790	36640437	AACACCTAAAACTTAAAAATC			II	
-0.186837	cg0138869	cg0138869	49677502	AAACCAATACCAAATAATATC			II	
0.2296487	cg0139416	cg0139416	12717498	TCTCAAACACCTAAAAACTTT			II	
0.2437959	cg0139554	cg0139554	62685325	ACAACAACTTACTCTCCCATA			II	

0.3555591	cg01397495	cg01397495	73746324	CCATCCTTCACTTCTTTCAATA			II	
-0.183637	cg01399317	cg01399317	13760327	TAACTAA	70639478	TAACTAA	I	C
0.1151507	cg01400040	cg01400040	49616453	ATAACRTAAACTAATTATTCAT			II	
0.121896	cg01407447	cg01407447	43638467	ACCCTAATACATTTTTTTAACT			II	
0.1080483	cg01409343	cg01409343	55651304	CATTTCAAAAATACCTAAAAA			II	
0.1365083	cg01409709	cg01409709	70799373	TTACCTAAAAATATAAACACT			II	
0.175768	cg01410923	cg01410923	56726407	TTTCATTCTTACATTCTTTAAC			II	
0.1021615	cg01412404	cg01412404	49660476	CRACAAAATCTCTTTCTTCACT			II	
-0.122583	cg01412419	cg01412419	72610302	TCACCCT	69694413	TCGCCCT	I	A
-0.129539	cg01412970	cg01412970	57688456	CCAATTT	12650324	CCGATTT	I	C
0.1066035	cg01413582	cg01413582	32689494	CTCAAAAACCAAAACCTCCAA			II	
-0.110291	cg01413848	cg01413848	38718438	ACCATAA	40801417	ACCATAA	I	A
-0.111489	cg01414185	cg01414185	57719415	TTAATCT	39804430	TTAATCT	I	A
-0.154489	cg01416563	cg01416563	12766393	CTACTCT	31702477	CTACTCT	I	T
-0.167021	cg01428378	cg01428378	39602445	CACAAAA	61800414	CACAAAA	I	T
-0.113868	cg01429449	cg01429449	48610458	TTATTCA	38713324	TTATTCG	I	C
0.1160814	cg01440489	cg01440489	67620425	ACAAACATACAAAATTATAAC			II	
0.1015121	cg01442843	cg01442843	62673347	CTACCAAAAAAAACAACTACT			II	
0.1017079	cg01445689	cg01445689	17793335	ACTAAAA	37710470	ACTAAAA	I	A
-0.175389	cg01445793	cg01445793	46745432	CAAATCC	29695498	CAAATCC	I	T
-0.127556	cg01448562	cg01448562	10771502	ATCCTCA	27630358	ATCCTCG	I	A
0.1006401	cg01451101	cg01451101	39754340	ATCTCCCCAAAACCAATAATA			II	
-0.175507	cg01454592	cg01454592	48725359	ACCCTAAAAACTAAACRAAAT			II	
0.1139171	cg01460430	cg01460430	61777484	AATCTACAACCTTTTCAAATTT			II	
0.1171566	cg01462727	cg01462727	58768363	AAAAACAAAACTTTAAAACTC			II	
-0.121734	cg01463110	cg01463110	10715421	AAAAATACATATAAATACCCC			II	
0.1625134	cg01464067	cg01464067	49746427	AAAACCTTTATAAACCCTAAA			II	
-0.132512	cg01469864	cg01469864	10809390	RAAAAAAAACAAATAATCCCC			II	
-0.292838	cg01474688	cg01474688	10701448	TACTTTTACCAACCAAATTCAA			II	
-0.107607	cg01475387	cg01475387	20620412	CTCCCCT	53605402	CTCCCCT	I	C
-0.145776	cg01478179	cg01478179	69693345	ACTTCTACTCCTTCTCTTCCAA			II	
-0.189438	cg01483656	cg01483656	70649496	TTTCTTCACTCAACCCAACAAC			II	
0.1930621	cg01484843	cg01484843	23790396	TACTTTCTCATCCTCAAACTAA			II	
0.1919336	cg01490094	cg01490094	53695420	AATCCRAACCTCTCTAACTCCT			II	
0.1484562	cg01491428	cg01491428	15709507	ACCTCCTAAAACAAAAACTCC			II	
0.1438841	cg01493009	cg01493009	12753507	AAAACTTATATATATATTACAC			II	
-0.172185	cg01499815	cg01499815	57646393	CAACACA	43678317	CAACACC	I	C
0.1394424	cg01502466	cg01502466	54808314	TAAAAATTTATCTCTTAATACC			II	
-0.11955	cg01518225	cg01518225	50676477	CTAAAAA	19679386	CTAAAAA	I	A
-0.244071	cg01519225	cg01519225	64745443	ATCTAACACCCTCCAAATAAT			II	
-0.10593	cg01519508	cg01519508	36802474	CAAACCT	67694477	CGAACCT	I	A
0.1847731	cg01523029	cg01523029	18756452	CRTCTTAAACACCTTACRTTTC			II	
0.1073898	cg01534253	cg01534253	27658321	AAACACATTAACTAAAAAATA			II	

-0.224409	cg01543583	cg01543583	33710356	AAAAATCAACACTTACCAAAA			II	
-0.272719	cg01546814	cg01546814	53787464	ATAACTATAATATTAACTTAC			II	
0.1075603	cg01548463	cg01548463	66707495	TTCCATATATTAAATACACCC			II	
0.1093484	cg01549226	cg01549226	16643509	CATAAACTCACAATCAAACTC			II	
0.119344	cg01550348	cg01550348	57800438	ATTATATTCACCAACTCCACA			II	
0.4091769	cg01551388	cg01551388	59663305	RTACTATTAATAAATTCCAAC			II	
0.1756893	cg01554777	cg01554777	29748349	AAATACTATTAAAACCAAAAT			II	
0.1044712	cg01559302	cg01559302	21687389	AAAACAATAATAAACRTAAAA			II	
-0.115929	cg01560873	cg01560873	16674376	AAATAAAACTAACTATAATAA			II	
0.4024945	cg01561758	cg01561758	53619391	AATCACCATAAAAATTAAAAC			II	
-0.103267	cg01561869	cg01561869	48704344	TCACCAATAACTTTAAATCTC			II	
0.1040084	cg01562813	cg01562813	21632448	AAAAACTCCCAACRATAAAAA			II	
-0.137293	cg01569984	cg01569984	42778479	ATCTCAAAACCCTTTACACTC			II	
0.100654	cg01579600	cg01579600	67692433	TCCTCTTTAAATAAATTTAACT			II	
0.1243966	cg01580228	cg01580228	26693475	AACTACAATATACTCTTACAA			II	
0.1072579	cg01581037	cg01581037	35778319	TAAAACTAAAAACCCTATTAA			II	
-0.141529	cg01581349	cg01581349	45618385	TAACATAATATTATCAAAAAT			II	
0.1152306	cg01582066	cg01582066	61600338	CAACCTATAACACACTTTAAA			II	
-0.178611	cg01585723	cg01585723	34695470	ATTCCAAATACACAAACTACC			II	
-0.308765	cg01591343	cg01591343	35649365	AAAAAATATTTATTTTAACTT			II	
0.1056913	cg01592350	cg01592350	74631308	AAACTAACACACCATATACAC			II	
0.2220894	cg01601712	cg01601712	22645432	AAAAAAACTCACTCTTTAACT			II	
0.1317395	cg01604009	cg01604009	56664402	TAAAAATTTACTTAAATCCCA			II	
-0.16395	cg01612292	cg01612292	38678327	TCAACCT	60736393	TCGACCT	I	C
0.1039007	cg01612656	cg01612656	33661463	ACTCCTC	14650472	ACTCCTC	I	C
-0.114108	cg01614102	cg01614102	66709389	CAAAAA	51669418	CAAAAA	I	A
0.1200047	cg01620701	cg01620701	45739403	ATAAATACTACCTACTAAAAC			II	
0.1362019	cg01621390	cg01621390	23710488	AAATCACTCATAATACAAACC			II	
0.1049234	cg01629007	cg01629007	29673359	ATCRACCATCAAACCATACTA			II	
0.1043318	cg01631333	cg01631333	58620420	AAACTACCAATCATTCTCATA			II	
0.1078006	cg01636575	cg01636575	66756369	ATTTTACTATTTCACATTTCTT			II	
0.2076519	cg01636873	cg01636873	50613508	TCATCAA	13770417	TCGTCAA	I	T
0.1136358	cg01637244	cg01637244	14719443	AACTACACCTTCTCCCATTAC			II	
0.1005611	cg01637482	cg01637482	31720477	ACTTTAACTTTAATAATCTATC			II	
0.1812492	cg01637789	cg01637789	63665456	ACAACACCCCCTATAAACAAA			II	
0.1119457	cg01638225	cg01638225	51719450	ACAAACT	43603388	ACGAACT	I	C
0.1548951	cg01647942	cg01647942	29767500	AATCCTAAACTTAAATTACCA			II	
0.2066977	cg01649611	cg01649611	69642419	TAAAAAATAACCAATAAACTA			II	
0.1632643	cg01651169	cg01651169	18772368	TCTAAAATAAATCATCATAAA			II	
-0.103878	cg01652244	cg01652244	20802475	AAACTTT	26638420	AAACTTT	I	C
-0.37287	cg01655658	cg01655658	14611329	ACTAAAACACTACRATACTAA			II	
0.1016076	cg01655721	cg01655721	42695500	CATCAAA	61791376	CATCAAA	I	C
-0.174981	cg01668653	cg01668653	34664417	AACCCTC	57628330	AACCCTC	I	C

-0.14805	cg0167870l	cg0167870l	12684333	ACTACCC	14738326	ACTACCC	I	C
0.1088333	cg0168186:	cg0168186:	38675349	CACAAATAAAAAAACCCCCTC			II	
-0.138686	cg0168588:	cg0168588:	49689328	TCCATAACTATTATATTAACCT			II	
0.125554	cg0169332{	cg0169332{	52662346	CAAACAAAAATACCTACATTT(			II	
0.1297629	cg0169599⁄	cg0169599⁄	26726427	AACCAACACCRTATTAACTCA'			II	
-0.101979	cg0169988(	cg0169988(	10749443	CTAAAACATAAAACTCCAATC'			II	
0.3063267	cg0170250l	cg0170250l	66667497	AAAATAATATCATTTCRTTTCA			II	
0.1091681	cg0170837.	cg0170837.	50717377	TTAACCATCATTCTCTACAAA⁄			II	
-0.171111	cg0171067(	cg0171067(	14745317	TCCCACA	42785427	TCCCGCA	I	C
-0.193987	cg0171350:	cg0171350:	66622481	CATACCRTTTCCTTAACACCTC			II	
0.118728	cg0171748:	cg0171748:	71740418	AAATCRACCRAAATAAAAAAC			II	
-0.102639	cg0173702(	cg0173702(	66789447	AACAAAC	66618329	AACAAAC	I	C
-0.237695	cg0174013:	cg0174013:	69676427	TCATTAACTTTCAAAAACCAT]			II	
-0.138553	cg0174105(	cg0174105(	70642504	CAATTCT.	16632414	CAATTCT.	I	A
0.176612	cg0174137:	cg0174137:	49677332	AAAAAAATATCCTACCACRTT(			II	
-0.117197	cg0174439(	cg0174439(	37747425	CRAATCCTAACACTAAAATTT⁄			II	
0.1041586	cg0174875.	cg0174875.	24802426	AAATAAAACCAACTTTCCCTC]			II	
	cg0175037:	cg0175037:	37724327	ACAACCT	13644489	ACGACCT	I	A
0.1494735	cg0175442:	cg0175442:	68737322	ATCRTATCTCTTAACCCACAA⁄			II	
-0.205741	cg0175452:	cg0175452:	18686381	AACAAAC	21736365	AACGAAC	I	A
-0.123472	cg0175967⁄	cg0175967⁄	23650393	ACAACAAAAACTAAATTCCRA			II	
-0.170022	cg0176009(	cg0176009(	33662376	CCATTACATTCACACCTATACT			II	
0.3139232	cg0176394.	cg0176394.	32688424	TTTTCCCACTAAAACRATATTA			II	
0.101058	cg0176532:	cg0176532:	16704479	AAAATATCACTACCTCCAACC(			II	
0.184473	cg0178670⁄	cg0178670⁄	56664461	TTATACA	32808388	TTATACG	I	C
-0.101617	cg0179260l	cg0179260l	34798432	ACACRAAAACTCTAAAAACTC'			II	
0.123678	cg0179622:	cg0179622:	65731500	AAACTCTTTATAAATACAACT]			II	
0.1518078	cg0179737l	cg0179737l	52795419	AACAATT	10640304	AACAATT	I	T
-0.109344	cg0179765:	cg0179765:	23670458	CACCAAAATTATACATTTATT⁄			II	
0.205142	cg0180109(	cg0180109(	59654474	TACTAATATAATATCTCTATTA			II	
-0.103184	cg0180493⁄	cg0180493⁄	30652499	ACTAAATCTCCRAAAAACTAA.			II	
-0.149465	cg0181243⁄	cg0181243⁄	42648449	AATTCAAACTTTTATACATAAT			II	
	cg0181373{	cg0181373{	70692408	TCRTTCCAACAAAACTATTAA(			II	
-0.170464	cg0181556.	cg0181556.	38792328	TCCACAA	71641451	TCCACAA	I	C
-0.194622	cg0181836{	cg0181836{	48799312	CAAATAAAATATCATCTTTAC]			II	
-0.167396	cg0182027:	cg0182027:	37709389	CACAAA⁄	66738353	CGCGAA⁄	I	C
-0.282585	cg0182358:	cg0182358:	54804382	RAATAACRAAAACTCTACACC'			II	
0.1082076	cg0182406:	cg0182406:	43696400	ACCTAATCCACCCTCAATCTA(			II	
0.112628	cg0182963:	cg0182963:	74607370	AAACTATAAAAATCAACTTAA.			II	
0.1143788	cg0183592:	cg0183592:	21638313	TTCTATACCAACRCTTCAACTA			II	
0.120975	cg0183645:	cg0183645:	27725379	TTAAATTTTTTATAAAAACAA⁄			II	
-0.146876	cg0183800⁄	cg0183800⁄	62755506	CCCCRAAAAACATAACAAAAT			II	
-0.165922	cg0184018:	cg0184018:	13742471	AAAAAA⁄	19667410	AAAAAA⁄	I	A

0.3122706	cg0184277	cg0184277	44678464	ACCAAAA	17673382	ACCAAAA	I	A
0.1574313	cg0184326	cg0184326	27791413	TAAAAACAAACCCTACRAAAA			II	
0.2842354	cg0184706	cg0184706	74642468	CCRTACAACTTTATATAACCCC			II	
0.103129	cg0185501	cg0185501	51601347	CATTCTCCTTAAACCACATATA			II	
0.151453	cg0185715	cg0185715	24772402	ACRTCAATACTTTATTACAACT			II	
0.1145085	cg0185783	cg0185783	36653390	AAAAAAACATAACATATAATA			II	
0.1229424	cg0185871	cg0185871	38792447	AATTTCCTAAATCTAAACCCA			II	
0.2903478	cg0186155	cg0186155	52778438	TCCCTAACCAATTACTTTTTAC			II	
-0.103058	cg0186268	cg0186268	36734461	TAAAACA	45740407	TAAAACC	I	C
-0.119738	cg0186440	cg0186440	28711435	TCAATAA	49785475	TCGATAA	I	C
-0.145564	cg0186446	cg0186446	10669341	AATTACTAACRATTAATCTCTA			II	
0.1431904	cg0187091	cg0187091	16718504	AACRAAAAACTTTCATTTACTC			II	
-0.111688	cg0187102	cg0187102	49793376	TATCACCATAAATTACAATACA			II	
0.2524785	cg0187298	cg0187298	51730335	ATCAAACCCTTACAAATTCACI			II	
-0.115107	cg0187486	cg0187486	43663398	TCAATCTTCRATAAAAATAATA			II	
-0.129602	cg0187486	cg0187486	31606304	CTATCCTTTCCCATAACAATTT			II	
0.1431783	cg0187546	cg0187546	26673508	CTCACCTAAACCTAAAATCTA			II	
0.1164951	cg0187633	cg0187633	43725462	TAATACCTCTAATCCTACATCA			II	
0.1017063	cg0188056	cg0188056	54806507	TAATAAAAAAAATCTTTTTACA			II	
0.1210644	cg0188106	cg0188106	58767390	ACAACCAATACCACTTTTTATT			II	
-0.123062	cg0188128	cg0188128	35697305	CAAACCRCTCACACCAAAAAT			II	
0.1344541	cg0188205	cg0188205	57666334	AAAACTA	44643344	AAAACTA	I	A
0.1001445	cg0188208	cg0188208	44622465	AATTTTATAAACTAACCTAAAT			II	
-0.116622	cg0188277	cg0188277	21623380	TTTTTACAATTATTTACACAAT			II	
-0.11988	cg0188377	cg0188377	19693345	TTCTTAATTTCCTTCTTAATCTT			II	
0.1167212	cg0188989	cg0188989	13602332	CTAAAAACCAATCTTACCCTAC			II	
0.1091149	cg0188995	cg0188995	41802369	AATAACAAATAAAAATCAAAA			II	
-0.27241	cg0189158	cg0189158	60666352	CAACAATCCAAAAATCCAACR			II	
0.1018801	cg0189173	cg0189173	43638342	AAAAAAC	59657395	AAAAAAC	I	A
-0.250021	cg0189487	cg0189487	64760482	ATCTCAACAAACCTCTCTATAA			II	
0.1066492	cg0190170	cg0190170	48643410	AATTATTAAAATAAAAAACAC			II	
0.1051775	cg0190547	cg0190547	34688463	CTAAACCTTAAATAAACCAAA			II	
-0.102149	cg0191319	cg0191319	66697436	ACAAAAT	54617510	ACAAAAT	I	A
0.1500805	cg0191640	cg0191640	20640482	ATTAATTATCTTAACCAAAATT			II	
0.3016931	cg0191669	cg0191669	35690442	AATTTACTCATCTATAAAATAA			II	
0.2085481	cg0191860	cg0191860	26687384	AATACCTCATAAACCCATAAA			II	
0.1589421	cg0191994	cg0191994	33759365	ATATCRCRTATAAATACRACA			II	
0.2750817	cg0192148	cg0192148	24711510	RCACTAAAATAACAACAAAAT			II	
-0.196958	cg0192573	cg0192573	27652432	ATACTAA	36748389	ATACTAA	I	T
-0.122931	cg0192681	cg0192681	48794458	CRAACAACRAAATCCTTTTTA			II	
-0.141738	cg0192782	cg0192782	17753408	TAATATT	26749428	TAATATT	I	T
0.2547298	cg0193074	cg0193074	24720508	CCTCCCCAACRAAATAACTAA			II	
0.1032234	cg0193122	cg0193122	23666344	TATTTATTTCCTACCATAAAAA			II	

0.1103286	cg0194158(	cg0194158(	61651486	CTTTCTTTAACCCTTACCTTCT]			II	
0.1058438	cg0194255{	cg0194255{	57796400	TAATTTTTTCAACTATAAAAT			II	
0.3108529	cg0194609;	cg0194609;	31608438	AACTAAAAAATATCATCATTT/			II	
0.1137094	cg0194821;	cg0194821;	66810339	ATACAACCTAAAAAACTCRCA'			II	
0.182227	cg0194822(	cg0194822(	71746375	TCTTCATTCAATTCTCAAAATC			II	
-0.413949	cg0194983:	cg0194983:	55734495	CACATAAATAAAAAACTAAAA			II	
0.1312857	cg0195458:	cg0195458:	21647351	AACTTACAAAATATTAATTAT/			II	
0.1717602	cg0195818(	cg0195818(	26702322	AACCATAAATATCCTAATACT/			II	
-0.145229	cg0196074{	cg0196074{	32809432	CRAACTTACAAAAACAAACAC			II	
-0.150099	cg0196363:	cg0196363:	16698453	ATTAAAAAATCCRTTAAAAAC			II	
0.1513494	cg0196627;	cg0196627;	46681479	AATAAAAAAAACAACAACAC]			II	
0.1677675	cg0196692:	cg0196692:	27633458	TTTAAATAAAACCCTACAATA/			II	
0.120087	cg0197033(	cg0197033(	73637467	CTCCCTCATAATTTAACCTACC			II	
0.1893138	cg0197454(	cg0197454(	33705326	TACTCTTAAAATAAACCTTTTC			II	
-0.113962	cg0197559]	cg0197559]	69648422	ACCCCCACTTTTTAAACTAAA/			II	
0.1659804	cg0197585{	cg0197585{	59694310	AATAACTATTCATCCTTAAACC			II	
0.1050991	cg0197823;	cg0197823;	29660412	AAATATCCTTAAACRTCATTA/			II	
0.1020476	cg0197870:	cg0197870:	27711305	TTACAAA	69625371	TTACAAA	I	A
0.1044592	cg0198920:	cg0198920:	11611407	CTATATATTCCCCATTACTCAT			II	
0.2926548	cg0199174:	cg0199174:	60789496	CACAATCTAATTCTACCCRAA/			II	
0.1561316	cg0199355:	cg0199355:	15679463	TCACTTCCTATCCAAATAAAC/			II	
-0.112111	cg0199741(	cg0199741(	48680440	AAAAAA(	22726367	AAAAAA(	I	C
-0.113361	cg0199759(	cg0199759(	59640444	TTCTCTAATATTTTCTAAACAC			II	
0.1483069	cg0199788·	cg0199788·	22666461	TCTATTACTCATTTAACTTTAT.			II	
0.1053485	cg0200106(	cg0200106(	66717351	TAACCTCCAACACCTTTTATTT			II	
-0.163883	cg0200440]	cg0200440]	14658436	AATACCT	25690377	AATACCT	I	C
-0.116649	cg0201139:	cg0201139:	63759488	AATCTCTACATTACCTCCCCAC			II	
-0.106419	cg0201172:	cg0201172:	73613315	CTAAAAC	54669365	CTAAAAC	I	C
0.21898	cg0201237(	cg0201237(	30653507	ACACCTATAAATCAATATCAA.			II	
-0.148053	cg0201282]	cg0201282]	54691403	TACTAAATAAAACTATTATTT/			II	
0.1768294	cg0201439(	cg0201439(	72718380	TTCTCCTTCACCCTTCACCTTA'			II	
0.1212699	cg0201829]	cg0201829]	31768427	AATATAACCCAAATCTACCAA.			II	
0.1197931	cg0202038·	cg0202038·	41641503	AAATATAAACTTTTAAACAAC'			II	
0.1352229	cg0202191(	cg0202191(	66773311	CACCACTATAACTTACCACAA(			II	
0.1101283	cg0203112]	cg0203112]	43772385	CAATAAATCTATAACCAAATT/			II	
-0.163421	cg0204597]	cg0204597]	36753465	CATATCC	49639309	CGTATCC	I	C
0.1264649	cg0204705]	cg0204705]	69657408	AAAAACTACTAAACATCTCAC.			II	
0.1561927	cg0205313:	cg0205313:	40754486	CACCCATAAAACAAAACCTCC			II	
-0.113896	cg0205389(	cg0205389(	62761334	CACACACACACACACAACACA			II	
-0.171725	cg0205840{	cg0205840{	20630471	CTTTTAT	73725428	CTTTTAT	I	A
0.111109	cg0206959:	cg0206959:	11701350	TCAATATAAAATACATAAAAA			II	
0.1281525	cg0207346:	cg0207346:	18787317	ACAAAAACTACTAAATAAAAA			II	
0.1099135	cg0207579]	cg0207579]	35650440	RCTTTATTACTCACTAATTTT(			II	

0.1003167	cg02087985	cg02087985	44624356	TTTTACTTTAAACCTAACTAAA			II	
-0.104487	cg02088197	cg02088197	26700405	CCTATATCCCRCACTAAAACAC			II	
0.1580086	cg02094283	cg02094283	73653485	AAAAAAATCCCTACCRATTTTT			II	
0.1211777	cg02095601	cg02095601	71613376	TACCAACATACTCCCAAACATA			II	
0.1311229	cg02096220	cg02096220	38701337	TCCTTTTCCTCTTACATCCCTAC			II	
0.105191	cg02102075	cg02102075	42690304	TAAATCATCTTCCRTAACAAAA			II	
0.2848869	cg02106072	cg02106072	67692407	TCTACAATCCTATCAAAAATTC			II	
-0.278494	cg02113055	cg02113055	22668459	TAAAACAATACTAAAAACCCC			II	
-0.211893	cg02118671	cg02118671	58650332	ACCAAAA	35735454	ACCAAAA	I	T
0.1278382	cg02119693	cg02119693	12715504	AACAAAAAATATTTTACATCCT			II	
-0.122944	cg02119792	cg02119792	41653398	CCCTAAC	69806312	CCCTAAC	I	A
0.1400521	cg02119938	cg02119938	57761451	CAACAAT	43808480	CAACGAT	I	A
-0.28394	cg02126896	cg02126896	21603365	CAAAAACAAAAAAACAAAAC			II	
-0.101795	cg02127418	cg02127418	19740340	AATCCAACATATAACACACAA			II	
0.4257554	cg02132058	cg02132058	46729363	TAAAACCCRCCAAAATCATTA			II	
0.124758	cg02134022	cg02134022	49760428	TCACTACCCAATATCCCATACC			II	
0.1022882	cg02135122	cg02135122	57807302	CATTATTTCAATCCCATAACCA			II	
0.1153394	cg02140579	cg02140579	26657344	CCTAACTAAACAAAAATAACA			II	
-0.123093	cg02141208	cg02141208	69664372	CAACCRAATTAATAAACACAT			II	
-0.202227	cg02143936	cg02143936	34696308	TAACRAAAAACAACACACCAA			II	
-0.127469	cg02152233	cg02152233	55703450	CCAAATC	34610416	CCGAATC	I	A
0.1083152	cg02156723	cg02156723	14803389	CAAATATACTTTAATCATCTAT			II	
0.1117669	cg02157626	cg02157626	53781451	CRTTTCCCCRAAAAACCCCAA			II	
-0.282912	cg02159489	cg02159489	29807491	AACTCAT	20791359	AACTCAT	I	T
-0.102518	cg02161919	cg02161919	64650410	TCAAACA	31701429	TCGAACG	I	A
-0.10467	cg02162286	cg02162286	12807354	TCAATAA	56601450	TCGATAA	I	A
0.1032495	cg02168584	cg02168584	26797508	TAAACCTTAATTTCCTCAATAT			II	
-0.130708	cg02169713	cg02169713	72641379	TACCCTCAAATATCCCTTAACT			II	
0.1054188	cg02170103	cg02170103	39697472	TATCCATATACTTAAATCAAAT			II	
0.1070331	cg02172885	cg02172885	49699431	AAAAACCCCTTTACAATACCA			II	
-0.1581	cg02173067	cg02173067	36681393	ACAACTA	26757342	ACGACTA	I	C
-0.114711	cg02174092	cg02174092	15664467	TAACAAA	72608378	TAACAAA	I	A
0.1121801	cg02184226	cg02184226	37622508	CATTACTCTTAATACTAACAAC			II	
-0.295149	cg02184540	cg02184540	42771474	ACAAATTTAAACAAACTAAAT			II	
0.1130801	cg02185007	cg02185007	72694378	TTTCTCTAAACATCTTCTCAAT			II	
-0.333021	cg02188142	cg02188142	44745437	AAAAAAAATTTAAATTTAAAC			II	
0.1790994	cg02188665	cg02188665	10787450	CCCATAAAACACAAAAAACRA			II	
0.109087	cg02192520	cg02192520	64801367	TAACTACTACTAAAACCAAAA			II	
0.1372477	cg02193806	cg02193806	59631353	CAATTTTACCCTAAACAAACCT			II	
-0.172117	cg02194908	cg02194908	38687336	AAAAAAC	48773344	AAAAAAC	I	A
0.1185326	cg02196256	cg02196256	37619333	ATAAAAAAAACTTACCCACTA			II	
-0.115273	cg02197629	cg02197629	19743370	AAAACAA	41784469	AAAACGA	I	A
0.3886147	cg02201753	cg02201753	46662451	ATTCTTACCTTACCRACCTAAA			II	

0.1070819	cg02203761	cg02203761	43722355	ACTAAATTTATACAAAAAAT			II	
-0.108967	cg02205962	cg02205962	38789501	RAAACCRAAACTACTATAAAA			II	
-0.138656	cg02214597	cg02214597	46607504	ACAAACA	63775303	ACAAACC	I	C
-0.107472	cg02217247	cg02217247	28616345	ACAAAACAAAATCACTCCCCA			II	
	cg02218932	cg02218932	13644467	AAAAATAAATAACCCCTAATT			II	
0.1003582	cg02220418	cg02220418	18675470	TAAAAAA	51631381	TAAAAAA	I	A
-0.114028	cg02222362	cg02222362	34644438	AAAAAAAACCCRCACAAAATA			II	
-0.184022	cg02222844	cg02222844	63665449	AAACCCT	46801339	AAACCCT	I	A
-0.126908	cg02224314	cg02224314	54760479	CACCTTA	33722311	CACCTTA	I	C
0.1015476	cg02227879	cg02227879	58654437	TATATACTAAAATCATCTCAAC			II	
0.1027737	cg02233197	cg02233197	26765412	CACACRCTAAAACTACTACTC			II	
0.1126048	cg02235843	cg02235843	11800397	AAAACATAAAACRACACTAAA			II	
0.138279	cg02243386	cg02243386	16646325	CAACAAAACTCTATAATTTTCA			II	
-0.104803	cg02243630	cg02243630	59795312	CAAATCCAAAATCTAACATCTC			II	
-0.123874	cg02249577	cg02249577	19661302	CTAAAAA	16740379	CTAAAAA	I	A
0.1827217	cg02251771	cg02251771	14751370	AAATCCTCCCCACTTTTAATAT			II	
-0.124858	cg02254774	cg02254774	32607413	TACCAAT	54600312	TACCGAT	I	C
-0.267003	cg02262167	cg02262167	45666375	TTTAAAC	70666403	TTTAAAC	I	A
0.2246636	cg02262553	cg02262553	12658476	ACAACATCCCAACTATATAAC			II	
-0.130547	cg02267270	cg02267270	60657497	AACCTAAACCTATAAAAAAAC			II	
-0.103593	cg02268561	cg02268561	13671358	CATTTACCCTTCTTACAAAATT			II	
-0.194094	cg02275226	cg02275226	65730497	CTCACAACRATAACRTATATA			II	
-0.113164	cg02286623	cg02286623	72747447	ATTATAC	27628432	ATTATAC	I	T
-0.115239	cg02286642	cg02286642	26615326	AAAAATCCTAACATCTTAACTA			II	
-0.171475	cg02287371	cg02287371	10605360	ACCACACRTACACAACRACATC			II	
0.14842	cg02288345	cg02288345	19809433	TCTAAAATCTATCCATCAAATA			II	
0.1100012	cg02291561	cg02291561	15691331	TAAAAATTTCAACTAAAATCRA			II	
-0.108566	cg02293501	cg02293501	28714496	AATCACTACRTATAACCACACA			II	
0.1087275	cg02295509	cg02295509	45796496	TAATTTTAACAATATCTTTTAT			II	
0.1273972	cg02295574	cg02295574	26612374	ATAAAAATACTTATCCTTTAAC			II	
0.1504112	cg02296931	cg02296931	39721443	ATACCTA	24783471	ATACCTA	I	A
0.367502	cg02299007	cg02299007	57603479	ATCACCAAACAACTATTTTAAC			II	
0.1018741	cg02300652	cg02300652	30661432	CAAAAACAAATCCCAAATAAC			II	
0.2372498	cg02301920	cg02301920	24763311	ACTCTCTCTACTTCCRCRACTT			II	
0.1137466	cg02303209	cg02303209	34697354	AATTCCATTTCTACAAAATAAA			II	
-0.286574	cg02304092	cg02304092	47773302	CTAAAAT	48721351	CTAAAAT	I	A
-0.15922	cg02304584	cg02304584	30626500	AAAATTACCCTAAAAAATCCR			II	
-0.10112	cg02305687	cg02305687	48699436	AACRAACAACCTACTAATTCTC			II	
	cg02311337	cg02311337	70681353	CAATATTAAATCRACCTTAAAC			II	
-0.122352	cg02312593	cg02312593	64807420	CAAAAACTAACAAATAACRAA			II	
0.1125451	cg02313379	cg02313379	22653369	ATTTATA	51809345	ATTTATA	I	T
0.1131413	cg02315732	cg02315732	23732351	TTACCATCTAAACTAAATTAA			II	
0.1083922	cg02317299	cg02317299	64623415	AAAAAAA	58695329	AAAAAAA	I	C

-0.183465	cg02319829	cg02319829	46797387	ATAATAACAATAACTAAAATA			II	
0.1033667	cg02319868	cg02319868	63707303	CACCATCCCATTCAATCCCACR			II	
-0.105817	cg02326566	cg02326566	37675446	ACAACRACATCATATAACTTCA			II	
0.1105387	cg02330500	cg02330500	53770462	ACTCACAAATAATCTAATTCAC			II	
0.1237034	cg02335251	cg02335251	67807477	CCTCTTAATACTATTTAACTT			II	
	cg02338840	cg02338840	58657370	AAAACATTTTTAATACCCTCTC			II	
0.1211588	cg02340092	cg02340092	19620379	ATCATAATAATAATCACCTCAT			II	
-0.175413	cg02342754	cg02342754	41775434	TTTTCCCTTCTTTAAAATTATCA			II	
0.2070957	cg02345758	cg02345758	37710395	ATAACCCCCCTAACTTAATAAC			II	
0.1461118	cg02348430	cg02348430	47783343	ACTCATATCTAAAAACCTATTT			II	
0.1167719	cg02351277	cg02351277	33786418	CTACCCTTTAAATCACACCAAA			II	
0.1105085	cg02352633	cg02352633	32803433	TCCACCRCRAATAATAATAAAT			II	
-0.238896	cg02355809	cg02355809	29764301	TTCCATTCATCATTACTAAAAC			II	
0.1114948	cg02358382	cg02358382	54608494	CAAACCTCCTCACCTTCTAATC			II	
0.1601282	cg02363593	cg02363593	62732492	TAAATCATAAACTCCTCTTATC			II	
-0.302812	cg02372404	cg02372404	33619327	ATAATTATACCAAAAACCAAC			II	
0.1397086	cg02374984	cg02374984	62778419	ACRAAACACTTTTTAAAATCCT			II	
-0.133169	cg02376916	cg02376916	22802390	ATTCCTTATTCCTTTAACRCAA			II	
-0.243346	cg02379549	cg02379549	52684309	AAACCATATATAAAAACATTT			II	
0.2014054	cg02380236	cg02380236	18773369	AATAACA	59737510	AATAACA	I	T
0.1242858	cg02381853	cg02381853	43676505	CAATAAATCCACACACAAATT			II	
-0.192647	cg02389040	cg02389040	74604407	AAAAAAAATTACCCTAAAAAA			II	
0.1264679	cg02389180	cg02389180	30634354	AATAAACTACAATTAACAATTA			II	
0.2171699	cg02389317	cg02389317	61663314	AAAACAACAAATTCATTTAAA			II	
0.1193126	cg02389634	cg02389634	56808505	AAAACTACTTAACATAAAAAA			II	
-0.193998	cg02389942	cg02389942	74642445	AAACACAAAAAAAATTTCAAA			II	
-0.107687	cg02390813	cg02390813	72750329	AACACCA	66630365	AACACCA	I	C
0.1563676	cg02394249	cg02394249	19662495	AAAACCTTTCCTCCCAAACCTC			II	
0.1973158	cg02398482	cg02398482	27722479	CCTTCCTCCTCTCTTACTAAAA			II	
0.1099623	cg02400773	cg02400773	67767450	ATAATTAAAAACCCCTAAAAC			II	
-0.128478	cg02401352	cg02401352	19620448	AACAAAAACCCTTCCTCCCTTA			II	
-0.114481	cg02401524	cg02401524	63767396	AACRAACATAAACRAACATAA			II	
0.1090533	cg02402208	cg02402208	19740380	CCCRAACCCCCAAAACCTAAA			II	
-0.130313	cg02403883	cg02403883	70783472	AATCTATAAATCAAACTACAA			II	
-0.140965	cg02407415	cg02407415	66778398	CAAAAAA	61773490	CGAAAAA	I	A
0.1033788	cg02413040	cg02413040	29678340	TTACAACTTTTCACCAATAACC			II	
-0.107147	cg02415057	cg02415057	47715360	AATTAAC	39807380	AATTAAC	I	A
0.1076169	cg02426178	cg02426178	36803354	TAAACCTACAAATCTTCAATAC			II	
0.1347415	cg02427576	cg02427576	56689398	AATAATAACATAACAATAACA			II	
-0.102522	cg02434554	cg02434554	73652409	AACCTATCRAAAAAAACTTAC			II	
0.1032155	cg02438026	cg02438026	65654422	ATTTCCCCTTCATCAAATAATT			II	
0.1313525	cg02448295	cg02448295	21728441	AACRCCCTATAAAATTACCATA			II	
-0.248151	cg02448481	cg02448481	36664410	TACCACC	10774420	TACCACC	I	C

0.122077	cg02448934	cg02448934	43770317	AATAAAATACACAAATATATA			II	
0.2973689	cg02449670	cg02449670	21667300	AAAAACTAACTTTACTCATCR			II	
-0.238636	cg02452418	cg02452418	22803434	TAAAAAACRCCAAAAAAATAA			II	
-0.229514	cg02452966	cg02452966	32611456	ACCAAAC	19780360	ACCGAAC	I	C
0.1031537	cg02455346	cg02455346	49740456	AATCCTTAACCTACCAACCTA			II	
	cg02458039	cg02458039	70648417	CCCACAA	18723357	CCCACGA	I	A
0.1209881	cg02458188	cg02458188	32715306	AACAAAAATTATAACTACCRT			II	
-0.376261	cg02462195	cg02462195	34754444	TCCCTTACTCCCAACCCACTA			II	
0.107573	cg02462661	cg02462661	52800491	CCACATTTATTTATCTAAAACT			II	
0.224478	cg02464458	cg02464458	35647474	ATAACTAAATTAAAACCAACC			II	
0.1386248	cg02464487	cg02464487	41709400	AAACTTTAATTCRATAACTCA			II	
-0.192436	cg02464808	cg02464808	26621487	AAATAATTCTCTAAATACATA			II	
-0.127159	cg02470697	cg02470697	29660440	CCCTCAA	63650414	CCCTCGA	I	C
0.1900756	cg02478369	cg02478369	62778410	AACAATCCAAAACTAATACAA			II	
0.1351225	cg02479022	cg02479022	28787373	TTATCTAATCCCRAATAACAA			II	
0.1040623	cg02479305	cg02479305	73614314	TACTCRAAAATCCTATAATTC			II	
0.1057028	cg02480320	cg02480320	55675493	CTCAAACCRAATAAAACTAAA			II	
0.172126	cg02480675	cg02480675	70810398	RCAAAACTACCCCCAACAATA			II	
-0.143391	cg02490460	cg02490460	11677337	AATAAAAACAATAACAATACT			II	
0.1773434	cg02491947	cg02491947	21602370	AATATTAAAATTACCTCTTTTT			II	
0.1468478	cg02495760	cg02495760	45745344	ACTTTACAAACCAATTAAATA			II	
0.1046141	cg02499308	cg02499308	62710352	TAAAACACTCCCAATAAACCA			II	
0.1024688	cg02499640	cg02499640	72752352	AACATATCTCACCRAAAATAA			II	
0.1943824	cg02502145	cg02502145	13619464	TAAAAAAATCATAACTCACTA			II	
0.1468692	cg02505956	cg02505956	60701359	TACAATCATTATCCATAAACA			II	
0.114093	cg02506728	cg02506728	65732360	TAAACRCRCAATCCAAACAAA			II	
0.1300299	cg02506908	cg02506908	30785352	TAAATCCTAAAATTAAAATAA			II	
0.1469981	cg02507181	cg02507181	29708358	AACATATCCCTCRTACTACTA			II	
-0.473098	cg02507579	cg02507579	55603348	TAATATCTCTCTCTAAATACA			II	
0.1302754	cg02513556	cg02513556	50623440	ATCCTACRAATAAATTACAAA			II	
0.1052738	cg02515217	cg02515217	57774402	TATTTTTTTATCAAATCCTACC			II	
0.1018634	cg02515899	cg02515899	45775478	CCAAAAATATATTTACCTCC			II	
-0.196085	cg02516696	cg02516696	55685455	CACCTAAAAATAACTAAAACA			II	
-0.207443	cg02518135	cg02518135	12627503	AAAAATTAAAACCCRAACCCT			II	
0.1073722	cg02519263	cg02519263	24663407	CCTACAAATAAAATCTCTATA			II	
-0.236915	cg02522936	cg02522936	29680371	CAATTTAATTCCTTACTACTCT			II	
0.1044444	cg02524236	cg02524236	33677426	AAACCATTCCTACAACCTAAA			II	
0.1045096	cg02527811	cg02527811	41782488	AAAACCTACTAATAACAACCA			II	
0.1016939	cg02532648	cg02532648	46751358	AACAAA	31767336	AACGAA	I	T
-0.144669	cg02533724	cg02533724	37758449	AACATTAAAATAAACAATTAA			II	
-0.134867	cg02541444	cg02541444	55644471	CTATATCTTTACCACAAAATTT			II	
0.1123581	cg02542817	cg02542817	47704302	TAATTAAATATACTTTCTACCA			II	
-0.10837	cg02548777	cg02548777	44637449	RCCTAAAAACCTAAACTTAAC			II	

0.106828	cg02550308	cg02550308	18647437	TAACAAAAAAAATCTAATTCA			II	
0.139241	cg02550738	cg02550738	31707440	TAACACTCTAATACTACTAACT			II	
0.2147764	cg02564175	cg02564175	13637463	TCCTAAA	48691441	TCCTAAA	I	C
0.1037125	cg02564259	cg02564259	66654397	CCCTTCRTCTCTAATAATAACA			II	
-0.103713	cg02566259	cg02566259	72648474	TAAAAATTAAAAACRCTAAAA			II	
-0.120858	cg02567750	cg02567750	59810364	AAAAACACTATATCACTTCATA			II	
-0.108952	cg02567769	cg02567769	56600477	CCAACAA	34766486	CCGACAA	I	C
0.1249054	cg02567879	cg02567879	44632442	TTACCACTTACCAATTATACCA			II	
-0.153254	cg02574844	cg02574844	38756423	CAACTAA	17704375	CAACTAA	I	A
-0.152279	cg02577387	cg02577387	31618469	ATTACAA	37668450	ATTACAA	I	C
0.1134661	cg02578709	cg02578709	62603494	RTAAAAAACCCTAATCCAACTA			II	
0.1118049	cg02579959	cg02579959	55778355	TAATACAATTACCTAACRATAC			II	
-0.101519	cg02580986	cg02580986	23730425	CAAAAAT	66742361	CAAAAAT	I	T
0.1478904	cg02584498	cg02584498	29652303	AAAATTTTTAAAAACTATTACC			II	
0.1310359	cg02585440	cg02585440	56782510	CTCCCTACATTCTTCTCTAAAA			II	
0.1038032	cg02590088	cg02590088	44686465	ATTTTCTCTCCTCACCAAAAAT			II	
0.1311516	cg02594532	cg02594532	73663496	CCRTAACTACRATATTCCTACT			II	
0.1044782	cg02598335	cg02598335	10689459	ACTTTACCAACTAAAAATAAA			II	
0.100876	cg02598807	cg02598807	51673307	CCCTTAA	71733392	CCCTTAA	I	T
0.1347407	cg02599498	cg02599498	27809452	ACAAAAT	55635418	ACAAAAT	I	C
-0.105201	cg02603022	cg02603022	64671423	AACCTAAAAATTTAACACAAA			II	
0.1533235	cg02604730	cg02604730	51751449	TATACTATTCTAAAAAAACAA			II	
-0.111929	cg02605760	cg02605760	24668498	ATTTTTT	31717474	ATTTTTT	I	A
0.1531951	cg02606566	cg02606566	17781341	CRCAATACATCCTAAAATCRA			II	
-0.192535	cg02611240	cg02611240	22734449	ACTTAATATTTCAACRTAAATC			II	
-0.144967	cg02613370	cg02613370	17799351	TCCTCAC	17634499	TCCTCGC	I	C
0.1306746	cg02614549	cg02614549	32703318	CAATTTAATATCTTTAATAACR			II	
0.1099087	cg02620335	cg02620335	31791443	CAAAAACRTAATATATCAACC			II	
0.104196	cg02622251	cg02622251	38804348	TAAAACTAAAATAAAAACCAA			II	
-0.12381	cg02623604	cg02623604	61753476	TAAAAAA	17783396	TAAAAAA	I	A
0.1935304	cg02626269	cg02626269	50637301	ACTCCAAAAATAAAAACTACC			II	
0.1106111	cg02628016	cg02628016	59615327	AACAACTCTAATCCAAATATTT			II	
0.1684242	cg02632314	cg02632314	27615380	AACACCACTTCCCCAATCTACA			II	
0.1251816	cg02633036	cg02633036	25763469	ACAACCAAAACCACCAACTCT			II	
0.1691466	cg02640147	cg02640147	44716428	CATAAATACATTCATCTCCCAA			II	
0.125729	cg02642117	cg02642117	40687465	TTCCAAAAACATAACAATATA			II	
0.1744621	cg02643580	cg02643580	26774414	TCATACCAAAACCACATAATA			II	
0.1038051	cg02649063	cg02649063	16701425	CRCRAACCAAACTAACTAAAC			II	
0.1182821	cg02653117	cg02653117	71715378	AAAAACA	60658342	AAAAACC	I	A
0.1036263	cg02659217	cg02659217	18801476	ATCTAAAAACACAACTTCRAA			II	
0.1046268	cg02660643	cg02660643	59607315	TAAACACTATAAAATTTAAAA			II	
0.1101008	cg02667103	cg02667103	35784414	AACAACATATTATTTCTACTTA			II	
-0.108436	cg02678768	cg02678768	14641394	CTCAAAT	39634389	CTCAAAT	I	T

0.1059958	cg02683577	cg02683577	16704484	TTTTCTTCCTCTTTACTATTCTA			II	
-0.105717	cg02686662	cg02686662	44685306	TTCACAAAAAAACACRAAAAC			II	
0.1058403	cg02692116	cg02692116	29670508	CCATTTATAAAACTATCTCCCT			II	
0.2121321	cg02693227	cg02693227	27785423	AATTCACCTACTTAATAATATT			II	
-0.110119	cg02698580	cg02698580	47740452	ACCCTAA	35810392	ACCCTAA	I	C
0.1039536	cg02700106	cg02700106	66802462	TAACACAACTCTAATAACAAA			II	
0.1062003	cg02701024	cg02701024	56803304	ACAAAAACTAACACACACAAA			II	
-0.278067	cg02702444	cg02702444	66611456	CTACATTCCCACCCCCATACCT			II	
-0.196184	cg02703248	cg02703248	47632385	ATACAAATACTAACRAAAAAC			II	
0.1206197	cg02704485	cg02704485	17772323	ATCTATAATTAAAACCTCTAA			II	
-0.196834	cg02710296	cg02710296	65777339	ATCACTA	10733343	ATCGCTA	I	A
-0.117747	cg02711665	cg02711665	19654361	TTAAAATAAAATCCCACTCTAC			II	
-0.102933	cg02712587	cg02712587	67736389	AAATTATATTTCCCCAAAATC			II	
-0.116577	cg02716302	cg02716302	64765382	AATTCAAATCTTCCCATTTAAA			II	
0.1246171	cg02716490	cg02716490	66661469	CTATTCTATAAAACTTTAAAAC			II	
0.1481848	cg02719221	cg02719221	61739485	RTAAAAAAACCCAACCTAAAC			II	
-0.11286	cg02719634	cg02719634	57633449	ATAACTTCTCCCRAAACTAACA			II	
-0.105423	cg02722596	cg02722596	57608460	CTAAAAT	57602461	CTAAAAT	I	A
	cg02723399	cg02723399	12798321	AACTAAC	69766403	GACTAAC	I	C
0.119799	cg02727484	cg02727484	10611473	CTTCRACATCTCACATACTCCT			II	
-0.181964	cg02730303	cg02730303	18609364	AAAAATAAACATATTCACATA			II	
0.1051166	cg02730865	cg02730865	35730376	AAATTTATCCTTAAATCCCATT			II	
-0.133476	cg02735058	cg02735058	64732455	TAAACCC	72650504	TAAACCC	I	A
-0.308957	cg02735423	cg02735423	24708417	AAAATAACACTCATACTTATCC			II	
0.1227368	cg02739280	cg02739280	22683502	CTAAAATAATCRTATTCTCTCC			II	
-0.467406	cg02741327	cg02741327	66636431	AAAATCTCATCCTACCCATAAA			II	
0.1411423	cg02743632	cg02743632	30668416	CAAAACTAAAATAACCTAAAT			II	
-0.207063	cg02745784	cg02745784	40672311	CCTACCCAACCAACTAAAACC			II	
0.2558808	cg02752932	cg02752932	28722350	TATTTAATTACAACACCTACTC			II	
-0.105943	cg02754945	cg02754945	28662413	CAAAATCCRCTCCRTTACATTC			II	
0.1397633	cg02756683	cg02756683	49771487	TACTAAATAAACCTAAAAACT			II	
-0.158504	cg02757577	cg02757577	74665399	TCTATCCCAATATCCAAAACAT			II	
0.1267934	cg02767633	cg02767633	35693455	ATATCAA	11653451	ATATCGA	I	A
0.1593354	cg02768671	cg02768671	37621403	ATCTCTAAACACCTACTATTAA			II	
-0.101419	cg02773588	cg02773588	48657414	AATAAAC	17667350	AATAAAC	I	A
0.101615	cg02776658	cg02776658	25789310	AAACCCCAAACRTAAATTAAT			II	
0.1011267	cg02786073	cg02786073	25802510	TTATTTCTCTTAAAATCTTCAC			II	
-0.116513	cg02787980	cg02787980	38673342	AATATAA	59767330	AATATAA	I	A
0.1003142	cg02790691	cg02790691	44660376	CTACATAACCATTAATTTAAAA			II	
0.1799498	cg02793323	cg02793323	74753409	TAATTTTATTCRACAATAAAAA			II	
-0.155386	cg02794920	cg02794920	41734374	AAACTTCCCTATATCTAAACCT			II	
0.1252159	cg02796478	cg02796478	47728452	CCAAACCTCTAACCTCCAAACA			II	
0.208881	cg02802508	cg02802508	54621330	CATTCACRAAACCCAACTCCAA			II	

-0.259826	cg0281143	cg0281143	29749452	ATTTTCCTTAAATTTCCTAATT			II	
0.1640079	cg0281289	cg0281289	35743415	CATTCTA	15771381	CGTTCTA	I	A
0.2729375	cg0281413	cg0281413	45768426	ACTTTAAAAAAAAACCTAAAT			II	
0.2235312	cg0281965	cg0281965	57804497	TTAAACAAAAATCCCAACCTT			II	
0.13069	cg0282041	cg0282041	55667427	CRAAAACTCTAAAACTAATAT			II	
0.1032247	cg0282623	cg0282623	10684376	AAACAAAACACTAACTAAAAT			II	
0.1158566	cg0282706	cg0282706	39741477	CTATACTCTCCTACAAAATAA			II	
0.1990569	cg0283251	cg0283251	17780384	AATAAAATCAACCTCATACAA			II	
0.1788912	cg0283623	cg0283623	29694415	CATAATA	29632381	CGTAATA	I	T
-0.117563	cg0283753	cg0283753	23802370	ACTAAAA	41627337	ACTAAAA	I	A
-0.108036	cg0283982	cg0283982	54743405	ATCCACAATAACCAAATTATT			II	
0.1321547	cg0284334	cg0284334	16645310	AAATCACCCCACCTAAAAATA			II	
0.1328758	cg0284722	cg0284722	32642474	TTATAACCATACTAAAACTAA			II	
0.1190069	cg0284883	cg0284883	71766444	AAAATAAACTCCACTCTACTA			II	
0.1013485	cg0284950	cg0284950	51739320	RTCCTAAACTACACTAAAACC			II	
0.1141773	cg0285490	cg0285490	33789400	CAATAACAAAAAATTCTTACC			II	
-0.179084	cg0285955	cg0285955	61635426	CAACTAC	65725476	CGACTAC	I	T
0.1029043	cg0286117	cg0286117	67749342	CAATCTAACCACCTAACTATA			II	
0.1683063	cg0286463	cg0286463	17648329	TAAACCACRACRCCCRACCAA			II	
-0.10091	cg0287131	cg0287131	14607466	RAAAATTCCCTACCCAAAACR			II	
-0.164983	cg0287276	cg0287276	38641425	AAACTAA	28689384	AAACTAA	I	A
-0.267278	cg0287378	cg0287378	31665328	TTCCTTCCTTTTATCCACATTT			II	
-0.267564	cg0287726	cg0287726	15650309	CAACTCATACTCCCRAAAACA			II	
0.135765	cg0288016	cg0288016	62794434	ATTAAAAATACTCTAAATTAC			II	
-0.11603	cg0288466	cg0288466	36660387	CTCTTAAATCCACTAACTCCAC			II	
-0.478987	cg0289023	cg0289023	64667483	AAATCTACTTTCTCATAATCTA			II	
0.2062052	cg0289025	cg0289025	67607477	AAAACAAAATTAACAACTTTC			II	
-0.109127	cg0289036	cg0289036	70618332	ATCCTAA	23673423	ATCCTAA	I	A
-0.131732	cg0289766	cg0289766	28808457	AACRAAAACTATACACATCTA			II	
0.1133614	cg0289815	cg0289815	31806316	TAATAAATTCTAAATCTCTTTC			II	
-0.167703	cg0290152	cg0290152	50743364	ATTCACRCCTAAATTCTCTAAC			II	
0.2292239	cg0290957	cg0290957	57637340	TCCTCACTTAAATCCTTATCCC			II	
-0.184267	cg0291049	cg0291049	48751465	AAAATTTTACAAAATATACTA			II	
0.117399	cg0291409	cg0291409	19613479	CTCATCCTATCRAACTACCCAC			II	
-0.111509	cg0291825	cg0291825	74716395	TATCCTT	71640376	TATCCTT	I	A
0.1285881	cg0291908	cg0291908	13612435	AAAATACATAATTAATAAAAT			II	
0.1312152	cg0291956	cg0291956	60730314	AATATCA	13641509	AATATCA	I	A
0.168429	cg0292019	cg0292019	33797315	AAATAATCCTAAACAAAACTC			II	
0.1454855	cg0292273	cg0292273	50807418	TATCATCCCCRAAAAAAAACT			II	
-0.21086	cg0292937	cg0292937	27744394	CAAACCA	65657383	CAAACCC	I	A
-0.148411	cg0293160	cg0293160	27668432	AAAAATAACTAAAAACAAAA			II	
0.1023377	cg0293471	cg0293471	64691421	ACTTAATTTTCCTAATAATCTC			II	
0.1975203	cg0293659	cg0293659	33623322	CAAACTAACAACTATTTAATA			II	

-0.109397	cg02937671	cg02937671	38742373	AAAAATAACAATAATAAACRC			II	
0.1386319	cg02938066	cg02938066	50657379	AACCCAA	15635405	AACCCAA	I	C
-0.119976	cg02944007	cg02944007	46759419	CCAAATA	47701300	CCAAATA	I	T
-0.187258	cg02951719	cg02951719	61754442	CACTAAAACCTAAAAAAACCC			II	
0.1049011	cg02954704	cg02954704	44606340	AAACAACTATTTAATTTTCRA			II	
0.1123996	cg02961798	cg02961798	42782402	AACCATAAATATACRAATTCCI			II	
0.100663	cg02962597	cg02962597	17628444	ACACAATAACAATTTCTAAAA			II	
0.1194775	cg02964385	cg02964385	23729497	TCATTACAAACCACACTCRAA			II	
0.1088794	cg02965295	cg02965295	40706498	ATCAATAATAAATCAAACTAT			II	
0.1549542	cg02966475	cg02966475	62807390	AAAATAACATAAACTAAATCC			II	
0.1129023	cg02968094	cg02968094	57674469	TATCTCCCTAAACAACCTATA			II	
0.1141856	cg02968537	cg02968537	10625360	ATAACTTAAAAAAAACTTAAC			II	
0.220771	cg02978201	cg02978201	39717400	ACAAAAATTTAATAAATATAC			II	
0.1184721	cg02979711	cg02979711	62796426	CAAACCCCRAAAACRATATAT			II	
0.158206	cg02980047	cg02980047	65768429	ATTAACCRTACATAAAAATTA			II	
0.1170481	cg02983090	cg02983090	36689346	AAAAATCTACTCCAACAAAAA			II	
0.1025839	cg02983911	cg02983911	73756507	AAACAAAAAAAAATACAACCT			II	
0.1074318	cg02992067	cg02992067	64607371	AAAATCAAAACATTCTCTAAC			II	
-0.13124	cg02996355	cg02996355	19683374	AAACCAT	42635509	AAACCGT	I	A
-0.131848	cg02999390	cg02999390	74772397	ACTTTCC	25804416	ACTTTCC	I	C
0.1632211	cg03003335	cg03003335	29612384	AACAAAC	53756368	AACAAAC	I	A
0.1455689	cg03007623	cg03007623	25767405	CACAATA	62797494	CGCGATA	I	T
-0.119991	cg03014326	cg03014326	21794330	TTCAAAA	42801352	TTCAAAA	I	A
-0.195927	cg03014829	cg03014829	51660482	AACATAAATTTAATTTCRAATA			II	
0.120584	cg03018931	cg03018931	19795429	AAAAACA	62677307	AAAAACC	I	A
0.277137	cg03019812	cg03019812	47769345	AAATTCCTACCCCRTAAATCTA			II	
-0.107071	cg03020006	cg03020006	44797510	AACATAA	16750437	AACGTAA	I	A
-0.181812	cg03020379	cg03020379	14783428	CTAAATACRTCCCRACRCTAA			II	
0.2794567	cg03020684	cg03020684	71641342	CCTACCACATAATTCTCCTAAA			II	
-0.168993	cg03022891	cg03022891	74698328	ACAAAAC	19758354	ACAAAAC	I	A
-0.141276	cg03024489	cg03024489	39704422	TTTATCTCTTCTTTCCCCTATCC			II	
0.1003251	cg03025825	cg03025825	49712375	CCCAACTTTTCCAAATTTTACC			II	
-0.104541	cg03025830	cg03025830	28705352	TAATAAA	36682333	TAATAAA	I	A
0.1009065	cg03026119	cg03026119	66747309	ACTTTTCCCTATAAACATATTA			II	
-0.137426	cg03028786	cg03028786	16691439	CCTAATTCAAAATTAAATCAA			II	
0.195408	cg03030267	cg03030267	43711485	AAAATCCACAATAAACTCTAT			II	
-0.243616	cg03035213	cg03035213	57606356	ATTCTAA	46785348	ATTCTAA	I	A
-0.119123	cg03040423	cg03040423	74732478	AATTATCTACCTAATTAATAAT			II	
-0.146787	cg03043127	cg03043127	74784429	TCCTAAAAACAACTATTTAAA			II	
0.1069099	cg03044281	cg03044281	45628426	ATTACCCTTTCCCRAATCTAAA			II	
-0.110517	cg03048372	cg03048372	36677492	AACAAAT	47662333	AACAAAT	I	C
0.1625182	cg03048488	cg03048488	26676361	AATCAACCTAACTCCCTTACCA			II	
0.2557728	cg03048921	cg03048921	34718455	TATAAAAACAAAACCAAAAAC			II	

0.1250749	cg03049445	cg03049445	69758346	AAATACCTCTCCCAAAAAACC			II	
0.112533	cg03052004	cg03052004	67625419	TATAACATTACTTAAAACAAA			II	
-0.115938	cg03052502	cg03052502	31713501	ATAAATT	40601382	ATAAATT	I	A
0.2627804	cg03053358	cg03053358	71775473	AAAAAAT	31601472	AAAAAAT	I	C
0.1048968	cg03053980	cg03053980	64799492	TAATACTAATCCCAAAAATAC			II	
0.1016483	cg03058412	cg03058412	44779447	CTAAAAAATCTCTTCAAATCA			II	
0.1248753	cg03059876	cg03059876	19659320	CTCATTC	64653445	CTCATTC	I	A
0.1907889	cg03062944	cg03062944	33708469	ATCCAAA	57620507	ATCCGAA	I	A
0.1001642	cg03065090	cg03065090	31620397	CCTAAAAACAAAATATACTTT			II	
0.1662816	cg03069731	cg03069731	58731498	AACTCAACCCAAAAAACAAAA			II	
0.1955584	cg03072082	cg03072082	22794385	ACAAACTACTAAATAAACAAA			II	
-0.255215	cg03075889	cg03075889	20639350	AATACTACACATAAACCTAAA			II	
0.1221613	cg03078006	cg03078006	29670350	AAACAAA	51658304	AAACAAA	I	T
-0.115565	cg03078363	cg03078363	37712341	CAAATAA	24651301	CGAATAA	I	C
0.1203298	cg03080336	cg03080336	16655374	TTCATTTTCCCCAATACCACAC			II	
0.2028601	cg03084184	cg03084184	52659317	ACCAAAA	67714456	ACCAAAA	I	A
0.1018695	cg03086707	cg03086707	23709502	TACTCAAAATATAACTACTTA			II	
0.1532076	cg03088958	cg03088958	65682469	CRACTACTCCAAACRTTTCTAT			II	
	cg03089717	cg03089717	54644378	CRATAAAAACAACATCTTTCT			II	
0.1512312	cg03097134	cg03097134	20764406	CAACCCATTATCCTAAACRAC			II	
-0.105712	cg03102494	cg03102494	54735359	CTAACTA	29622494	CTAACTA	I	A
-0.110428	cg03105556	cg03105556	36725473	AAAACAATCTAACCAATACTT			II	
-0.115804	cg03108070	cg03108070	69757501	AAACCTA	42616429	AAACCTA	I	A
0.1751854	cg03109913	cg03109913	21688303	AATACTTTCTCAACTACATCCT			II	
-0.103807	cg03112518	cg03112518	69725325	CTCTACCTAAAAAAACRACRA			II	
0.1371512	cg03115532	cg03115532	54616458	TTAAAACACAAAAATCTTTCT			II	
0.1110295	cg03116016	cg03116016	27631430	AAATTTTCCAAACTCRAAAAA			II	
0.129231	cg03117611	cg03117611	46765327	RAAAATTCCATTTAACRCCCC			II	
-0.118644	cg03120147	cg03120147	10786355	ATAAACA	33658325	ATAAACA	I	T
-0.146941	cg03120461	cg03120461	43717452	CAAAATC	72646479	CGAAATC	I	A
-0.237948	cg03122926	cg03122926	52703317	TTCCTACAAAACCTATCTCCCC			II	
0.1914138	cg03123709	cg03123709	27808379	CAAATCTTAAAAACATATTTT			II	
0.2030264	cg03126739	cg03126739	72746442	CCTAAACAAAACACTAACTCA			II	
0.1499202	cg03127584	cg03127584	51677413	TCAATACATAAAAACCRTATA			II	
0.1116676	cg03128499	cg03128499	50803403	TTTCTAAAAATATCTCCAAAA			II	
0.2026655	cg03130910	cg03130910	18723328	ATCAATA	71768350	GTCGATA	I	A
-0.103614	cg03133408	cg03133408	67624445	ACRACTTTCACTACTTACCTAA			II	
0.1145753	cg03135948	cg03135948	16702378	AATAACATTATTACCTTCCAA			II	
0.18333	cg03136151	cg03136151	72796315	CCCCCTATAAAAAATACATTA			II	
0.1052591	cg03136493	cg03136493	25795438	ATTTAATAACCAATTCAAACC			II	
0.1191652	cg03142741	cg03142741	53804372	AAACCAACAACTACTCTAAAT			II	
0.1271183	cg03143591	cg03143591	72635510	CAACTATACTATATATACTTCC			II	
0.1028282	cg03147852	cg03147852	61755437	AAAATAAAATAAAACATAAA			II	

-0.113284	cg0315250(	cg0315250(	20603307	ACACCAC	30748437	ACACCAC	I	C
0.1360974	cg0315654(	cg0315654(	54620402	TTCTACATATCTCAAAATAAAT			II	
-0.109523	cg0316501⁴	cg0316501⁴	37801383	AAAACCC	26609473	AAAACCC	I	C
-0.115643	cg0316517(	cg0316517(	25652440	CTACCCTCCCCCCAAATAAAA/			II	
0.1593285	cg0316788:	cg0316788:	40793322	CAAAACAAAAAACACAATAAT			II	
0.2424211	cg0318370(	cg0318370(	27689358	AACAATTATTAAACATTCAAA(			II	
0.1198641	cg0318477(	cg0318477(	64615357	AAATCAACAATTCTAAAACAA			II	
-0.106535	cg0318570⁴	cg0318570⁴	46675353	TCAATACATATTAAATAATAA			II	
-0.160697	cg0318766{	cg0318766{	65795451	CCCCAAAACTAAATACTTTCTT			II	
-0.120808	cg0318839(	cg0318839(	40618341	CRTAAAAAAAAATACAATACT			II	
-0.101563	cg0318902:	cg0318902:	58780320	AAAATAC	40648391	GAAATAC	I	C
0.1296538	cg0320133	cg0320133	64645445	AAAACRTTACTATTAAATCAA/			II	
-0.170633	cg0320206(	cg0320206(	22773327	CCCTTTC(	65733324	CCCTTTC(	I	A
-0.166806	cg0321320:	cg0321320:	71778483	TAAAATT	19757345	TAAAATT	I	C
-0.579331	cg0322139(	cg0322139(	23660495	TCTAATAAACATCTTCCCRAA/			II	
0.1145448	cg0322148:	cg0322148:	32772437	TAAAAAATATTTTATCTAAAA/			II	
0.1253789	cg0322462	cg0322462	65721411	ACTTTATTAAACATAACAATA/			II	
-0.129014	cg0322544⁴	cg0322544⁴	36697398	AAAAACA	38670430	AAAAACC	I	A
	cg0322903:	cg0322903:	61601301	ACTCCAC	12768448	ACTCCAC	I	C
0.1268796	cg0324098	cg0324098	23786387	TAAAAATTTTTCRTTAAATAA/			II	
0.1037835	cg0324647{	cg0324647{	74694436	AAAAAAATCTCTATCTCCTCR(			II	
0.1043654	cg0325433(	cg0325433(	14684453	AATATTACTAACTAAATCCTTC			II	
-0.101834	cg0325609(	cg0325609(	35665324	ACTCTCA	46752368	ACTCTCA	I	T
-0.10668	cg0325668⁴	cg0325668⁴	48703475	TTAATAT	55689305	TTAATAT	I	A
0.1089261	cg0325742:	cg0325742:	11697450	AAATTCTAAAAAACRTAATCC/			II	
0.1245646	cg0325813	cg0325813	25724501	CCAAATAATCACAATACACTT			II	
0.1536596	cg0326062	cg0326062	32632363	TTACCCRTTTTATTATACATTC(			II	
0.1260422	cg0326269(	cg0326269(	30772379	AAAACRATAAAAACCACTATA			II	
0.1090722	cg0326319	cg0326319	36697485	ACAAAATAACATACTAATTAC(			II	
-0.31843	cg0326413:	cg0326413:	34617348	CAAAACC	15745431	CGAAACC	I	C
-0.122693	cg0326455(	cg0326455(	20767486	AAATAAA	68790453	GAATAAA	I	C
0.1073329	cg0326526{	cg0326526{	38785503	AACAAACAAATATTCTTATTC(			II	
	cg0326936:	cg0326936:	74623352	TAAAATACCCCCAAAAACATA			II	
0.195676	cg0327148	cg0327148	29659310	AACTTAAACACAAACTTTCAT(			II	
0.1056114	cg0327190	cg0327190	49612398	TAATCRTCACAAACCRTCTAA/			II	
0.114281	cg0327249(	cg0327249(	45687351	CATATCCACCATTATAAATACT			II	
0.1199921	cg0327293:	cg0327293:	26785499	CTCTATCCCAATCTCCTATTAA			II	
0.1016877	cg0327688:	cg0327688:	24762468	TTCTTTTTAACCTTATATCTCT/			II	
0.2402165	cg0327692(	cg0327692(	38610449	CTCTTCTTTCCACAAATTCTCA			II	
-0.142655	cg0327755(	cg0327755(	15760501	AATTCAC	55775356	AATTCAC	I	A
0.107889	cg0328704(	cg0328704(	24810478	TAAATACAACCTCAAACTTTA/			II	
-0.187202	cg0329222:	cg0329222:	48643368	CACTTAC	40807439	CACTTAC	I	A
-0.120542	cg0329320(	cg0329320(	71631447	CTATCAA	10656419	CTATCGA	I	C

0.1071932	cg03293507	cg03293507	65794484	ATCACAA	67747316	ATCGCAA	I	C
0.2790326	cg03296304	cg03296304	43767507	CCAAAAA	53655437	CCGAAAA	I	A
0.1074553	cg03302281	cg03302281	17625478	ATAACCACTTCAAATACTACC			II	
-0.120055	cg03307177	cg03307177	36784509	AATCCCAAAACRAAACCCCCT			II	
-0.131572	cg03307659	cg03307659	29779377	CAAAACC	21661421	CAAAACC	I	T
0.1565622	cg03312406	cg03312406	35720308	TTCCAAACCTATCTATATTACT			II	
0.1685655	cg03312587	cg03312587	12722321	AAAAAAACAATTTCCTATAAT			II	
-0.197443	cg03314029	cg03314029	36773421	CCAAAAA	19738374	CCGAAAA	I	A
0.368719	cg03315469	cg03315469	32760433	AAAATAT	15738422	AAAATAT	I	T
-0.125596	cg03327325	cg03327325	56714412	RACATATATTTACACTAACACA			II	
0.1695981	cg03328299	cg03328299	72790425	CTACTTCTCTAAAAAATCCAA			II	
0.1821062	cg03329597	cg03329597	30766334	ACCACATCCATAAATACTTCAT			II	
0.1316228	cg03329714	cg03329714	17746301	RCRACCTTTTAAATCTAACTCC			II	
	cg03333149	cg03333149	61768320	CAACATA	73756355	CGACGTA	I	A
-0.156624	cg03335262	cg03335262	61766455	AAAAACC	67632339	AAAAACC	I	C
-0.167038	cg03337057	cg03337057	69804327	AATCCCC	64785367	AATCCCC	I	T
-0.124516	cg03340754	cg03340754	38784345	AACAAATATTAACCTTTATTTT			II	
0.1759402	cg03341641	cg03341641	41716440	AAAAACATTTACCTAAATAAC			II	
0.1081656	cg03342032	cg03342032	24770426	AATAATTCTAAAATAAAACAA			II	
0.2263712	cg03343571	cg03343571	20631385	CCACTAT	65602498	CCACTAT	I	T
-0.114547	cg03349091	cg03349091	47708439	AAAACCTATCCRAAAAATCTA			II	
-0.106128	cg03350138	cg03350138	23625367	AAACRCAACTAACAAACTAAA			II	
0.1048747	cg03355554	cg03355554	44712471	ATACATACAAACACRTACTCTC			II	
0.2214614	cg03354616	cg03354616	52704320	CCTACCAATTTCCACCTACACC			II	
0.1289171	cg03356172	cg03356172	47793323	TAAAAAACATCCCCRAAAATC			II	
-0.21427	cg03357999	cg03357999	64655397	AATACCA	63787408	AATACCA	I	A
-0.103634	cg03370193	cg03370193	35655452	AAAACAA	43662303	AAAACAA	I	A
0.1002315	cg03373960	cg03373960	61720304	CACTTAAAACCAAAAATATAA			II	
0.1057502	cg03374148	cg03374148	66647422	AAAAAAATAAAACAATCTATA			II	
-0.117141	cg03378197	cg03378197	63667510	CCTAAAT	24806498	CCTAAAT	I	A
0.2271194	cg03386715	cg03386715	21618356	ATCCACTCACCTTAATCTTAAC			II	
-0.120294	cg03389890	cg03389890	73783404	AAAAAAC	27792304	AAAAAAC	I	C
-0.110645	cg03392100	cg03392100	62733384	CATAAATCAAATTCTACAAACA			II	
-0.144307	cg03398919	cg03398919	41748327	TTTTAACRATATAAATAACRCT			II	
-0.43151	cg03398989	cg03398989	26691355	TTAATCTATAAACTCATATCCR			II	
-0.113562	cg03401875	cg03401875	47738484	AAAAAAATTTTATTTTCCTTT			II	
-0.196098	cg03408135	cg03408135	10759351	RAAAAAATAAAAATCTCTTCTC			II	
0.1272988	cg03408497	cg03408497	40659498	AAATTAATAAATAATACCTAC			II	
0.1594328	cg03412618	cg03412618	19668486	ACATATAAACAAACAAACAAA			II	
-0.111877	cg03418136	cg03418136	42620358	CCTCCTTC	47803332	CCTCCTTC	I	C
-0.292985	cg03420970	cg03420970	59754449	ACAACAATATTTTATACCTTAA			II	
0.1444581	cg03422552	cg03422552	17711503	TAAATATCCTCCCTACTCCTTA			II	
0.3003632	cg03422583	cg03422583	33804439	AAAAATCTTAAATCCAAACAC			II	

0.1141062	cg03422749	cg03422749	46741444	ACTCATCAAAACRCRTAAATTC			II	
-0.131899	cg03428619	cg03428619	66699313	CTTCCCAACACTCTTTAAACCA			II	
0.100167	cg03431028	cg03431028	17719341	TAAAATTTCTTCRTAAAAACTT			II	
0.131126	cg03432172	cg03432172	40652429	TTACTCTTTATAACTTTTTAATA			II	
0.1119694	cg03432241	cg03432241	60770382	TTCACTTAATACTATAACCTTA			II	
-0.221566	cg03432589	cg03432589	39795458	TCTTCATAAAAAACCAATATTT			II	
0.1146758	cg03433313	cg03433313	52731440	CTATATCTATACTCAAAACCTA			II	
0.1280657	cg03434907	cg03434907	68617390	AAATAACRTAAAATCCTAAAA			II	
0.1056923	cg03438552	cg03438552	74622436	ATAAAAA	14758435	ATAAAAA	I	C
0.1444326	cg03441493	cg03441493	23789460	AAACAACRTTTCCTCTAACAAC			II	
-0.132519	cg03441844	cg03441844	29779397	ATAATTTACAAACCTATATACA			II	
-0.10553	cg03443143	cg03443143	44675453	CAACACA	11721413	CAACGCA	I	A
0.1009677	cg03455107	cg03455107	28621319	AAAAAACACAAAATTAACTAT			II	
-0.106522	cg03455316	cg03455316	49799466	AACAAAA	27783425	AACGAAA	I	C
-0.111973	cg03464416	cg03464416	62602353	AAAAAAATACCTATAACCRCTA			II	
0.1104687	cg03466587	cg03466587	37758489	ATCCTTCATCAAATATAATATT			II	
-0.354182	cg03466780	cg03466780	29736451	TATAAAACCTTAACCTCAAACA			II	
0.1074532	cg03467235	cg03467235	14657490	TTTAACATTACCCTCTTACTAC			II	
0.1023381	cg03469682	cg03469682	72709302	ACATAATAACCCTATTTTTACC			II	
0.1831765	cg03469766	cg03469766	40613300	TAAAAATACAAATTCCATCATC			II	
0.1186109	cg03485667	cg03485667	52759305	TTTCCTTCRCTAACTCTACAAA			II	
0.1128491	cg03486379	cg03486379	65799476	ATCTACTAAATAACCAATCCRA			II	
0.1428968	cg03487027	cg03487027	61625442	AAAAATA	24710412	AAAAATA	I	A
0.1024629	cg03487983	cg03487983	13617335	ATTTAATATAACATTCAAAATC			II	
0.1033357	cg03491895	cg03491895	62794477	ACACCACCCACACATTCCTTTC			II	
0.2826955	cg03494612	cg03494612	42732495	ATATAAAAACTACATCTAATAA			II	
0.1904547	cg03495084	cg03495084	65757507	TTTCAAAATATTTTCAAACAAC			II	
-0.101682	cg03498886	cg03498886	42799501	AACCAACAACTAATACCCAAC			II	
0.1270568	cg03507593	cg03507593	51754433	ATATACA	13666340	ATATACA	I	T
0.2512942	cg03509467	cg03509467	58778459	TCTAAACCCATTCCACATATTC			II	
-0.101125	cg03514086	cg03514086	28743489	AATACATTTTTCTTAATCTTTCA			II	
-0.226918	cg03517284	cg03517284	20641477	CAAACAA	53717306	CAAACGA	I	C
0.1821169	cg03518729	cg03518729	66687328	CTTCACAAAAATACTATCAAAC			II	
0.1077563	cg03529900	cg03529900	51795445	TCCTTAAAAACCTAACAAAATA			II	
0.1511973	cg03532013	cg03532013	34623394	TTACATTC	57743396	TTACATTC	I	A
-0.172435	cg03534453	cg03534453	41700477	AAAATTTACCTAACAAAAAAC			II	
0.1091565	cg03536885	cg03536885	51663335	ATAAATCACAAAAAAATACTA			II	
-0.111019	cg03543861	cg03543861	52790378	CATAAAT	64740380	CATAAAT	I	A
-0.128342	cg03557725	cg03557725	50699401	TCTAAAATACTAAATACAATTC			II	
-0.18146	cg03562350	cg03562350	17636481	ATAAAAC	55688419	ATAAAAC	I	A
0.113126	cg03563842	cg03563842	54607508	TAAACAA	20655340	TAAACAA	I	C
-0.118556	cg03570263	cg03570263	38765376	ATCCCTAAAAATTAAATACCTC			II	
0.1315862	cg03577157	cg03577157	69605321	AAATCTCCTCATAAAAACCACA			II	

-0.165402	cg03582285	cg03582285	21687304	CTCCTCRCRATAAATCTCCTTA			II	
-0.303716	cg03585888	cg03585888	60646402	CTACRAATCCTAAAATTCAAC(			II	
-0.133267	cg03586820	cg03586820	72795423	ATCTCTCAAATTAATTACATTT			II	
0.1022071	cg03592824	cg03592824	40639392	ACATAATCCAAAAACTATTAA			II	
0.1071162	cg03594506	cg03594506	20602395	TCTTTCTAACCRATAAAAAATT			II	
-0.108792	cg03594907	cg03594907	59742309	ATCCAAACTCCTCCCCTTTTTC			II	
-0.236543	cg03595348	cg03595348	65622382	ACTTAAAAACACTTTACRTCA			II	
-0.136072	cg03598159	cg03598159	10604387	TAAAACTCTTCRTAAATTTCTA			II	
0.1032637	cg03599197	cg03599197	19632507	AAATACAAATATTTATTATAA(			II	
0.1937188	cg03601053	cg03601053	38759429	CCCTCTACAAAATAAACCTAA(			II	
-0.106262	cg03605116	cg03605116	69666366	TTCACCTAACCCTACCCAATA			II	
-0.11138	cg03626579	cg03626579	46612344	AAAACTATATCTAAAAACTAA			II	
-0.23695	cg03631455	cg03631455	14740468	TAACACT	68777388	TAACGCT	I	A
0.1045873	cg03635442	cg03635442	42783481	ACCACTA	43778393	ACCGCTA	I	C
0.1229034	cg03637769	cg03637769	31719455	TTCTTAA	24706345	TTCTTAA	I	T
0.1824871	cg03645522	cg03645522	17765492	TCAAAAA	31614469	TCAAAAA	I	C
	cg03648679	cg03648679	13644314	TACCTAAAATATTAATTTCCRT			II	
-0.202498	cg03649293	cg03649293	24798428	AAAAACAAAATAATCRAAAAA			II	
0.1015736	cg03653573	cg03653573	42760314	CRAATAAATACAAATCTATAA			II	
-0.148911	cg03653726	cg03653726	31769394	CTCTTCTA	27709421	CTCTTCTA	I	A
-0.116442	cg03654299	cg03654299	14661378	TATATCA	68652322	TATATCG	I	A
0.1444597	cg03658557	cg03658557	30737368	AACCACAATATATTATATAAC			II	
0.1083739	cg03660901	cg03660901	38787488	AAATAAACATTTATACATTAA			II	
0.1057193	cg03661387	cg03661387	20751449	TCTAAATTATAAAAATCTAAA(			II	
0.1476188	cg03664443	cg03664443	24714510	TTTTTAAAACTAAAACCAAAC(			II	
0.1019821	cg03668763	cg03668763	17638311	CCAAAAATTATATACACAAAA			II	
0.1559109	cg03672997	cg03672997	21735405	ATATATTCCCAATTCTATATTT			II	
-0.125549	cg03679269	cg03679269	10777355	AATCTAT	18679416	AATCTAT	I	C
-0.101442	cg03682112	cg03682112	69640406	AAAAAA(	46602385	GAAAAA(	I	C
0.132206	cg03682581	cg03682581	10616328	AATACACAAATTATTTCACAA(			II	
-0.159415	cg03682719	cg03682719	49754473	AAAACCA	58606305	AAAACCA	I	A
-0.105144	cg03683899	cg03683899	30785408	CCCAAAT	48621402	CCCAAAT	I	C
-0.119971	cg03686964	cg03686964	49768495	AAAAATCCCTAACAACTCCTC			II	
-0.111191	cg03699074	cg03699074	23641470	CRTCCCRTTCACACTAATCRTT			II	
0.2031397	cg03699765	cg03699765	35785392	ACCAAAAAAAAATTAAATCAT			II	
0.1263912	cg03705784	cg03705784	39696395	TATTTACCCAATCATAAACAT(			II	
-0.116342	cg03709428	cg03709428	55726380	RAAAAACACTTTAAATCCRCC			II	
-0.103599	cg03716984	cg03716984	47778337	ACTTCAT	37801394	ACTTCGT	I	A
-0.101738	cg03719428	cg03719428	50610459	RAAAAAAACATTTATACRAAC			II	
-0.25309	cg03721887	cg03721887	65787500	AAACAAAAATTTTATACCACC			II	
0.1270278	cg03723481	cg03723481	74699400	AAATAAAAAATCAAAAAACTA			II	
0.1380615	cg03725115	cg03725115	63676473	CCACAAT	69618420	CCGCGAT	I	T
0.1068236	cg03736235	cg03736235	36778506	RAAAATAAAATTAAAACTCCA			II	

0.1098846	cg03737424	cg03737424	31720431	ACTATTTCRCTAATCTTTCTCA			II	
-0.150819	cg03739955	cg03739955	62631370	AAACTAACCCAAATAAAAATA			II	
0.163157	cg03743861	cg03743861	47696316	TTCTAAA	18739419	TTCTAAA	I	A
0.1034174	cg03744383	cg03744383	39625318	AAAAAAACTTTTCTAATCTCT			II	
0.1209428	cg03745491	cg03745491	21769466	CAAAACRACTCTCTAATCCTC			II	
0.2113697	cg03748376	cg03748376	23756382	AAAACAA	36759456	AAAACAA	I	A
-0.130687	cg03749207	cg03749207	32766445	ACCTAAA	20798314	ACCTAAA	I	T
-0.115816	cg03750315	cg03750315	42668374	TAACTAT	62616418	TAACTAT	I	A
-0.123924	cg03751967	cg03751967	21636307	AACTACA	26676348	AACTACG	I	T
0.2425912	cg03754882	cg03754882	45731391	CTCCAATTTCTTTCTTTTATAA			II	
0.1191233	cg03761216	cg03761216	26675439	TCTAAATTATTCTAATATAACC			II	
0.273129	cg03767353	cg03767353	65697342	ACCAAAA	43604403	GCCAAAA	I	A
0.1153349	cg03773862	cg03773862	16713429	CAACTTACTACTTTTTTTCTAC			II	
-0.111473	cg03774803	cg03774803	58629324	CACCCCC	58727362	CGCCCCC	I	A
0.1194589	cg03777875	cg03777875	59709412	ACTTTCT	27678374	ACTTTCT	I	T
0.1242207	cg03778207	cg03778207	59698354	TTCCCTTCCTACCAAAAAATAT			II	
0.1616184	cg03781748	cg03781748	23616336	CAACCATAATTTAATCCTCCAT			II	
0.1508775	cg03784363	cg03784363	63810344	TAAAAAA	11686466	TAAAAAA	I	A
0.1006303	cg03784886	cg03784886	72630371	AACACCAAAATAAACCAATTT			II	
-0.124179	cg03790740	cg03790740	38691422	TACRAACACTTATACTACTAAT			II	
0.1303581	cg03796943	cg03796943	60733357	CAACATTTTACTACATTAACA			II	
-0.100472	cg03798915	cg03798915	12611322	AAAAATC	65706440	AAAAATC	I	C
-0.116246	cg03805357	cg03805357	48606424	AAAAACATATATATATTAACA			II	
0.1592759	cg03808001	cg03808001	51627426	AATAATAAATAATCRACATAC			II	
-0.169453	cg03810198	cg03810198	33712327	ACTAAAAAAACTATCACCCTA			II	
0.2384412	cg03810282	cg03810282	69694309	CAAAAACCACTAACCACAATT			II	
-0.190418	cg03813151	cg03813151	38671404	TCACATT	63737358	TCGCGTT	I	T
0.1033908	cg03814957	cg03814957	14746308	ATATCCCAACTAATAACCAAA			II	
-0.100236	cg03816625	cg03816625	61605441	ATCTATA	68702328	ATCTATA	I	C
0.104859	cg03823901	cg03823901	18676319	TACTTTTCATCCTCCTTACAAA			II	
-0.12395	cg03826976	cg03826976	60791386	ACCAAACCCAAAATAAACRCC			II	
-0.109496	cg03827118	cg03827118	71608382	AAAACTAAAAACTTAACTACC			II	
-0.192459	cg03830006	cg03830006	22735337	ATCATATCTAAAACAAATAAA			II	
0.1085305	cg03834116	cg03834116	73629419	TAAAAAATATATCRTAACATC			II	
0.1057149	cg03836586	cg03836586	25758330	ATCACAACCATCAACTATAAT			II	
0.179414	cg03837313	cg03837313	29653407	AACTTTTAACAAAAAAACRAC			II	
-0.131382	cg03837909	cg03837909	52677414	AAAATTCATCACAAATTAAAA			II	
0.1101211	cg03839782	cg03839782	33725393	AAAAATAACACAACACTATAA			II	
-0.101528	cg03844894	cg03844894	46743301	TACCAAA	42629423	TACCAAA	I	A
-0.115501	cg03845409	cg03845409	21660398	CAATACCTAAATATCCTAACT			II	
0.1469472	cg03847896	cg03847896	47666370	ATCCTACTTCTTACCAACTATA			II	
-0.110094	cg03854098	cg03854098	52617426	AACAATTCATTTCTAACAAAA			II	
-0.155282	cg03858673	cg03858673	15689482	AACACAA	27753327	AACACAA	I	C

0.1589287	cg03861065	cg03861065	37747316	TACTAATATAACAAACTTTTC			II	
0.1428951	cg03867465	cg03867465	20600339	ATAACTT	24667343	ATAACTT	I	A
0.10939	cg03868770	cg03868770	45755499	AAAAAAAATATAACTTAACCC			II	
0.2975057	cg03871140	cg03871140	62706422	ACTCCTC	36772431	ACTCCTC	I	A
-0.179593	cg03873153	cg03873153	20701450	TACCTAAAAAAACTACAACTC			II	
0.1332392	cg03875819	cg03875819	24674469	ACACAACCCCAAAATATAAAA			II	
0.1304347	cg03876548	cg03876548	54735462	AAAAAAAACTTATCTCTATAC			II	
0.1396295	cg03880506	cg03880506	37672364	TACCTTA	17649329	TACCTTA	I	A
0.167325	cg03885343	cg03885343	20804380	TTCCCCAAACAACACAAAATT			II	
0.1056728	cg03886436	cg03886436	44773505	AAACAACTTAACACCAAAAAA			II	
0.1059901	cg03887438	cg03887438	32679333	TTAACTTCCATATTAATACRAA			II	
0.1733462	cg03887721	cg03887721	38655438	TATTTCTATCCCAATAAAAAC			II	
0.1256937	cg03887906	cg03887906	46708323	ATTTACAAAAATTAAAAAACT			II	
0.108072	cg03888553	cg03888553	17680413	TTTCCAAACACCCCTAAAATT			II	
0.1822681	cg03896685	cg03896685	28760490	AAACTCCTCCTTATATTATTCA			II	
-0.229988	cg03897406	cg03897406	14727422	TCTCCTAAAATACAAACCCCTC			II	
0.1330602	cg03899215	cg03899215	45683355	ACCCATCCCTATACTTAATAA			II	
0.3204426	cg03900028	cg03900028	73681507	CCTCATTATCAAAAATATCCA			II	
-0.225838	cg03905819	cg03905819	35676433	TCCAACTTCCCCCAAAACCAA			II	
0.1098598	cg03910557	cg03910557	41728473	AATTAAAAAAATATTCTTTAA			II	
0.2400472	cg03913278	cg03913278	55660410	AAATTTCCAATAATCTAAAAT			II	
-0.138205	cg03915932	cg03915932	55706303	ACTATTCCCTCAAAACACTTA			II	
0.1749024	cg03916630	cg03916630	13715369	TTAACCCAACCTAAAAATAAA			II	
-0.10247	cg03922251	cg03922251	50715360	ACTCAAATATAAAACRATCCA			II	
0.1083578	cg03922381	cg03922381	15647405	TTACCTAATTAAAATTCCAAA			II	
0.1689606	cg03925642	cg03925642	46631507	TAAAAAC	60719487	TAAAAAC	I	A
-0.133795	cg03927302	cg03927302	21607475	CAAATAT	55799503	CAAATAT	I	A
0.1966367	cg03930097	cg03930097	64626443	TTATTACTTTACTTATTTCAAA			II	
-0.117009	cg03936031	cg03936031	19732471	ACATCTACTAAACTAATATAT			II	
-0.261062	cg03940883	cg03940883	47771340	AAAAACTATATCAATCTAAAA			II	
0.1106792	cg03944375	cg03944375	55626364	AAATAACTCAATAAAACCTAA			II	
-0.103555	cg03945122	cg03945122	34680310	ATTTAAAAAACAACAACAATA			II	
0.1189255	cg03947384	cg03947384	41640303	TTTCCAATAATCATCACAATA			II	
-0.110524	cg03951394	cg03951394	27605463	CCCTAAAATAAAACTACAAAA			II	
-0.127149	cg03951833	cg03951833	60764350	AATAAAA	55616472	AATAAAA	I	T
0.1425952	cg03952520	cg03952520	30712464	ATCTATCAACTCTTCAAAATT			II	
0.1041095	cg03953626	cg03953626	73703331	ACAAAAA	52738498	GCAAAAA	I	A
0.1439429	cg03957898	cg03957898	65681454	TTCCTCTCTTCCTAACAACACT			II	
0.1245202	cg03958308	cg03958308	52675474	ACRACRTATACRATATAACAA			II	
-0.11385	cg03960382	cg03960382	55717341	ATCATTA	46725335	ATCATTA	I	T
-0.133703	cg03961283	cg03961283	35678323	ACAAACA	54648301	ACAAACC	I	T
-0.109339	cg03961401	cg03961401	34642452	TAAAACCCTCRAAATAATCTC			II	
-0.111542	cg03961824	cg03961824	10714496	AAAAAAA	72799304	AAAAAAA	I	A

-0.37532	cg03964373	cg03964373	12623394	TAAAATTCTCCTATTAACTTTA			II	
0.1211293	cg03966955	cg03966955	46709395	AAACCCT	20713394	AAACCCT	I	A
0.1047816	cg03967448	cg03967448	17763502	CATACAACTCCTAATACAAAA			II	
0.2000227	cg03970567	cg03970567	30702313	CTTTCAAATAAAACTTCAATTA			II	
0.1313922	cg03970900	cg03970900	29755467	CAAACTAAAAAAATCCCTAAA			II	
0.1499503	cg03977174	cg03977174	60708333	TTAACTCATTTACATAATTCAT			II	
0.1901558	cg03977831	cg03977831	65797345	TCTAAAA	71688451	TCTAAAA	I	C
-0.132176	cg03978067	cg03978067	49650305	AAAACACCCTAACCCAATAAC			II	
0.1018693	cg03980550	cg03980550	36689398	CATAACATTCCRAAACCTAAA			II	
0.1054346	cg03982831	cg03982831	47650436	CTTTAAA	40804378	CTTTAAA	I	A
0.1163319	cg03987818	cg03987818	51643436	TCTTAAAATACATATCTAACCA			II	
0.2227079	cg03988092	cg03988092	13710427	ACACTTTACTACTTAAAAATTC			II	
0.101092	cg03989520	cg03989520	49681350	ATAAAAATTATATTTAACCTCT			II	
0.2658098	cg03989711	cg03989711	33807483	TAAACTA	71810430	TAAACTA	I	A
-0.19267	cg03995300	cg03995300	41704350	CACAACT	11604489	CACAACT	I	A
0.1163571	cg03997145	cg03997145	28610445	AACAAAAAAAACCCTAACTAA			II	
0.1607858	cg03998338	cg03998338	15708503	CTCCRCACACAAAAAAAATTT			II	
0.1981881	cg03999583	cg03999583	28696355	CTATACT	30670361	CTATACT	I	C
0.1203315	cg04001071	cg04001071	19737355	TCAAACTCCTATCATTACACAA			II	
0.1472143	cg04004561	cg04004561	11720499	ATTCTATAATATTACCTTCAAT			II	
0.1071909	cg04010296	cg04010296	12795452	CACAACAATAAACAAATATAA			II	
0.2311059	cg04026937	cg04026937	41732346	AAAAAATCAAAAAACTACTCA			II	
0.1273535	cg04028570	cg04028570	24670475	ACCCAAA	10773329	ACCCGAA	I	C
0.1599874	cg04028695	cg04028695	31763342	CTCCCTATTATCAAAAATTTA			II	
-0.111465	cg04029664	cg04029664	66718398	CAAAACT	31689385	CGAAACT	I	A
0.1969257	cg04045544	cg04045544	41600309	CTAACCAATAATACACAATAA			II	
0.5076566	cg04049719	cg04049719	47707333	TTTAAACCACCTCTTAACTATC			II	
-0.142072	cg04050824	cg04050824	65726481	ATAAACA	42808445	ATAAACA	I	C
-0.130601	cg04052466	cg04052466	15726311	TTCCACA	30744315	TTCCGCG	I	A
0.1707245	cg04054303	cg04054303	50614476	CCTTCCTTCCTTCCTTCCTCRCT			II	
-0.128547	cg04056384	cg04056384	65659471	CACTTTTTCACTACTAAAAATA			II	
0.2688949	cg04057378	cg04057378	61607418	AAAAAAAAACTCTAATCCCAA			II	
-0.157794	cg04058752	cg04058752	74773314	CAAACCC	53740335	CGAACCC	I	C
0.2210455	cg04061372	cg04061372	42790393	CRAACCAAAAACTTCTCAAAA			II	
0.1435453	cg04064998	cg04064998	44739322	ATTACAACAATAACTAAACTA			II	
	cg04071689	cg04071689	13644319	AACAACT	44696344	AACGACT	I	C
-0.317611	cg04072301	cg04072301	34790439	AAACACC	17704395	AAACGCC	I	C
-0.149213	cg04074536	cg04074536	22685497	ACAACCA	26702345	ACAACCA	I	C
0.1021407	cg04076044	cg04076044	25793345	TAAATATTTCRTTTAACATAAA			II	
0.112826	cg04077979	cg04077979	22760414	TTAAAATACCCAATTAATCTCT			II	
-0.335781	cg04080091	cg04080091	53682455	RCCATTTAAAAAAATTACAAA			II	
-0.131689	cg04080282	cg04080282	47697447	AAACCAA	16806436	AAACCAA	I	A
-0.127855	cg04084173	cg04084173	33785396	ATCTTTAATCCRTCTCTCTTAA			II	

0.129665	cg04084354	cg04084354	19803510	TCACTTCCCTCTAAATCTTACA			II	
0.100532	cg04088474	cg04088474	55790411	TATATAATAATACTTAACACAT			II	
-0.133122	cg04089743	cg04089743	63791383	CTACACAAAAAAACCATTATA			II	
0.1169708	cg04090794	cg04090794	14706411	ACTTAAACCCAAAAATACAAA			II	
0.1185429	cg04093633	cg04093633	50690440	TTCTCCTAACCAAATCTATCCT			II	
-0.102431	cg04098176	cg04098176	40694344	AACTAAATACAAATTAAAACC			II	
0.1199492	cg04102894	cg04102894	65796411	AAAAACT	51770392	AAAAACT	I	A
0.1033683	cg04103251	cg04103251	70694425	CCATTCCTAATTCTCTAAACCR			II	
0.1189518	cg04105416	cg04105416	62734499	CAAATAA	36735379	CAAATAA	I	C
0.1022803	cg04116469	cg04116469	16629405	TAACTTAATACAAAAACCCAA			II	
0.4050458	cg04118610	cg04118610	24633384	CTAAAAATTATTAATCRCTACC			II	
0.1320177	cg04130557	cg04130557	60630321	TCCAAACCAACRACTAAAATTA			II	
-0.71073	cg04131969	cg04131969	65713421	CACTCCCCCAAACACACACAA			II	
-0.149763	cg04132418	cg04132418	10725414	TCATAAAAAAACTCCCTAACTA			II	
0.1090492	cg04137323	cg04137323	38671432	ATAAAAAATCRTATATATATA			II	
0.1155843	cg04138046	cg04138046	28701482	CTATAAACCACAATTATTATCT			II	
0.2004181	cg04141080	cg04141080	48617498	AAAATAA	10741347	AAAATAA	I	A
0.1277693	cg04143348	cg04143348	20794319	TTATAATACTTCACTTAAAAAT			II	
0.1064088	cg04143592	cg04143592	62748360	CCCTCCAAACATTTAAAATCTA			II	
-0.236443	cg04145539	cg04145539	29613448	AAAACTAAAATAAATTCCCRTA			II	
-0.471971	cg04145681	cg04145681	64786405	CAAAATCAAACAAATTATTAA			II	
0.1140835	cg04148163	cg04148163	72691505	CATTTAATTTAAAACACTCACT			II	
-0.13657	cg04152793	cg04152793	69793334	AACTCAACTTCACTAACCCCAC			II	
0.2265601	cg04154653	cg04154653	26748302	TATAAAT	31658404	TATAAAT	I	C
-0.10485	cg04155289	cg04155289	66691464	AAAACTAATAATTCCTAAAAA			II	
0.2093343	cg04157272	cg04157272	37726509	CAAACTA	40626453	CAAACTA	I	A
0.1313851	cg04163391	cg04163391	32771384	AATATAA	44700368	AATATAA	I	A
0.2513441	cg04168479	cg04168479	67712315	TACCCACCCAATTAATCAATAT			II	
0.1877331	cg04169747	cg04169747	29742458	CRAAACTTATTTACTATCCAA			II	
0.1896449	cg04172345	cg04172345	19607404	ACCAACATAATAAAAACTCCA			II	
0.312974	cg04176452	cg04176452	28673491	CAAAAAA	69744457	CGAAAAA	I	T
-0.237679	cg04178011	cg04178011	73754376	ATCCTCCTCTATATACAATAA			II	
0.1011594	cg04180046	cg04180046	54600475	TAAATCCRACTAAACTAAAATA			II	
	cg04180623	cg04180623	55606429	CCATCAAACATAAAAAAACCA			II	
0.1121325	cg04187039	cg04187039	20735354	TAATAAAAATTTAATAAAACC			II	
0.1213368	cg04188920	cg04188920	22781319	CAACCTC	14717364	CGACCTC	I	A
0.2136872	cg04189187	cg04189187	55635408	CCAACAA	34753355	CCGACAA	I	A
0.1056374	cg04192412	cg04192412	49720372	CTCCAAAAACATATTACAAAA			II	
0.1507027	cg04193779	cg04193779	29756474	AACTACCCCATATATTTACTAA			II	
0.1043339	cg04195941	cg04195941	33747415	CTTCATATCCCTCCTCATCCCT			II	
0.1090618	cg04200010	cg04200010	11606507	ACTTTAATTTATAATCTACCAA			II	
0.1215195	cg04205653	cg04205653	23688303	TAATACATAAATTCAATACCTA			II	
-0.108865	cg04206342	cg04206342	10679419	AAACCTA	52733447	AAACCTA	I	A

-0.156916	cg04209913	cg04209913	14635360	TAACCTA	48705467	TAACCTA	I		A
0.2375593	cg04214938	cg04214938	58754429	ATAAATA	17690433	ATAAATA	I		C
0.1067098	cg04215287	cg04215287	29723399	CTAAAAAAACCTTCTTAAATCA			II		
0.1170361	cg04217177	cg04217177	48747345	ACCTCCRACAATTTACTTTAA			II		
-0.130819	cg04225785	cg04225785	63641301	CAAACTC	36627303	CGAACTC	I		C
0.1177901	cg04226110	cg04226110	21633351	TCCTTTAACCTCTTAACAAAAT			II		
0.1037952	cg04231042	cg04231042	42740411	CRAATTCAACTTAAAAACTACC			II		
0.1328654	cg04231677	cg04231677	26740419	TTATTTTATTTCATCAATAAAC			II		
0.1026894	cg04233620	cg04233620	54649330	TTCRTAATAAATCACATAACCT			II		
0.1181438	cg04237918	cg04237918	54624473	TCCTAACATTCCTATCTAAAAC			II		
0.1007476	cg04242655	cg04242655	43759422	TCTCCTACCACAAACTATAATA			II		
0.1066114	cg04245540	cg04245540	41682325	TCAAATTAAATTATTTCTAAAA			II		
-0.192484	cg04245870	cg04245870	55739300	CATATAAAAAACACRTTCCCRT			II		
0.1033209	cg04246708	cg04246708	15809316	TTTACTCTAACACTAAACACAA			II		
0.1644681	cg04251208	cg04251208	12723403	TAACCATAAAAAACCCAAATC			II		
-0.128993	cg04256864	cg04256864	49672435	AAACTCC	57665313	AAACTCC	I		C
0.1010769	cg04258138	cg04258138	29610361	AACTAACTCAAATAATACAAA			II		
0.1045238	cg04258676	cg04258676	64664362	ATAAAACTCTTACCATACRATC			II		
-0.12172	cg04264433	cg04264433	55714325	TTTATAATACTTCTTTACACCR			II		
-0.142344	cg04267772	cg04267772	19708438	CCCAATAACCCCAATAAAAAC			II		
0.1341313	cg04275695	cg04275695	25630462	ACTACCCTCACRAAAAATTTAT			II		
0.133251	cg04276626	cg04276626	65695394	AAAACAAAAACAAACCAAAAA			II		
-0.104984	cg04277055	cg04277055	63702454	AAATCACAAACTCCCAACTATA			II		
-0.102513	cg04278168	cg04278168	65795333	ACCACTAAAACATCAAAAACR			II		
0.1242334	cg04279411	cg04279411	10740331	TTTACTCAAAAAAAACCCCATA			II		
-0.103917	cg04284140	cg04284140	66658300	AACTTAAAAAACAAACCCAAA			II		
	cg04285477	cg04285477	63703394	TACAATT	54606510	TACGATT	I		A
0.1365514	cg04286737	cg04286737	54677371	ACAATCC	57724403	ACAATCC	I		C
0.1056836	cg04291404	cg04291404	54610397	TTTAAAATTTCTTTCCCAAAAC			II		
0.1022505	cg04292976	cg04292976	57794473	TAAAAAACAAAACTATATAAT			II		
0.1048584	cg04297406	cg04297406	22686369	TTAACACRCCTATTTAAACAAT			II		
-0.158624	cg04297660	cg04297660	53709380	CCAAAATATTCCTTCRCTAAAT			II		
0.1045879	cg04297995	cg04297995	36722316	CACAACAATATACCACAAACC			II		
-0.131495	cg04301343	cg04301343	31784487	AACAACT	18629332	AACAACT	I		C
0.1250767	cg04306489	cg04306489	20604407	RAATTAACTTAACCTTACCCAC			II		
0.1021877	cg04308089	cg04308089	23717416	CTTTACAATATCRCCTATAATT			II		
0.1430028	cg04314361	cg04314361	48680374	TCCACTTCATAACCTTCATCCA			II		
-0.181894	cg04316537	cg04316537	27713329	TTTAAACACCTATCAAATAACT			II		
0.1154459	cg04318327	cg04318327	48628377	TTCTTTTATTCAAATAACCCTA			II		
0.1246469	cg04329382	cg04329382	59712409	ACTAAAAAAAACTCTAACTAA			II		
0.3622797	cg04331561	cg04331561	34804418	ACCCAAAACTTAAAAAATATA			II		
0.1297101	cg04332927	cg04332927	31677454	AATTCTTATATCTACTTTTATA			II		
-0.119298	cg04342483	cg04342483	51793470	TACTTCCCACAATCAAACATAA			II		

0.1275096	cg0434419(	cg0434419(	24707481	AAATTAAAAATATTAAACAAC			II	
0.1466271	cg0434533	cg0434533	62676500	CAAATCAATTTCCCCTCCTTTC			II	
0.1757641	cg0434628	cg0434628	57650456	AATACCAACTTTAACAATTAC			II	
0.1511904	cg0434998	cg0434998	14680348	CAAAAAAACTAACTAACCAAC			II	
0.1100021	cg0435021	cg0435021	20649484	AACCTCACTATAAAATCCTAT			II	
-0.154963	cg0435467	cg0435467	50762343	CTTATAC	12708313	CTTATAC	I	A
0.1071963	cg0436607(	cg0436607(	64714487	CAAATTCAATCTACRACRCCC			II	
-0.114675	cg0438264	cg0438264	63775343	CTACTAA	41620489	CTACTAA	I	A
0.1005361	cg0438468	cg0438468	58699305	ACCCTAAATCATAAAAACTCA			II	
0.119967	cg0438675	cg0438675	30780483	ATCCCAA	12632309	ATCCCAA	I	A
0.112162	cg0438739(	cg0438739(	74728348	CAACAAAACCAAACAACCATT			II	
0.1460888	cg0439229	cg0439229	29727438	AAAAACA	31669319	AAAAACC	I	T
-0.109673	cg0439845	cg0439845	31692348	AAAAACA	53722489	AAAAACC	I	A
0.1844553	cg0440678	cg0440678	23738383	CACTATCAAAACCRTAACTTTC			II	
0.1258611	cg0441611	cg0441611	47656320	TCTACTCCCTAAAAACTAACTC			II	
0.114294	cg0442086	cg0442086	39624466	ATAAAACTTTCATACCCAATA			II	
0.121749	cg0443699	cg0443699	50606366	TTAAAAT	10676470	TTAAAAT	I	A
0.1235943	cg0444736	cg0444736	37705438	ATTTCAAAACTAAATAACAAA			II	
-0.119828	cg0445312	cg0445312	70784496	ATATATA	64778315	GTATATA	I	A
-0.489164	cg0445649	cg0445649	42668379	ATATACTCATTACTACTCCTAA			II	
0.1054588	cg0445892	cg0445892	46692322	ATATCAA	31679409	ATATCAA	I	T
0.1061156	cg0446114	cg0446114	74779469	TCCRAAATCACAACAAAAACT			II	
0.2466198	cg0446452	cg0446452	17674305	AAACCATTCTAACTTCAACAT			II	
-0.131619	cg0446763	cg0446763	63703470	TAAACCTCCCTATTCTATTCCC			II	
0.1233454	cg0446874	cg0446874	24733338	TAAACRCTCACAAAACCCAAA			II	
0.1170868	cg0447005	cg0447005	46713489	CCTAAAT	71707481	CCTAAAT	I	T
0.1290224	cg0447340	cg0447340	37756496	TTTATAACCCCCAAATTATCCC			II	
0.1251318	cg0447365	cg0447365	34713510	AACTATTATTACATATCCTATA			II	
-0.100423	cg0447498	cg0447498	21737403	AACATCA	67710393	AACGTCG	I	A
-0.118548	cg0447818(	cg0447818(	67706389	TCTAAAATAACAAACRAAATA			II	
-0.172628	cg0448107	cg0448107	24728365	ACAAAAT	73620326	ACAAAAT	I	A
-0.160744	cg0448192	cg0448192	52700462	AAATAACAAATTATACCRAAT			II	
0.1021487	cg0448398	cg0448398	51717475	TTCTCTAAACCTCTATAAAAA			II	
0.3423056	cg0448683	cg0448683	61780346	ATATTTCTAAACCTCAACTAA			II	
0.2125331	cg0449761	cg0449761	64633508	ATAACCCTTAAAAAATATAAA			II	
-0.223075	cg0449782(	cg0449782(	17807329	ATTCAATAAACAAAAATTTAA			II	
0.100446	cg0449970	cg0449970	50767325	TATTATTAACTTAAATATATAA			II	
0.1015942	cg0450090	cg0450090	59763439	TTCTACTAACTTCTAAATTAAC			II	
0.1029728	cg0450611	cg0450611	60742376	ACTACAACCCACATTTTTAAA			II	
-0.145431	cg0450846	cg0450846	12601343	CRCCCTTTTTCTTCCAAAAAAA			II	
-0.242568	cg0450860(	cg0450860(	60779380	TTTCCTATAAAAACAACAATC			II	
0.1481144	cg0450887(	cg0450887(	13685312	CAAAACCACATATCTACTCCA			II	
0.105321	cg0450954	cg0450954	20802351	TTCAATCTAATAACTCAACAC			II	

-0.101068	cg0451818(	cg0451818(	24810402	ACCTCTTCCATCTACTTTAAAA			II	
0.1090662	cg0452025l	cg0452025l	25723405	AACACAATCCACCTATAAACA.			II	
-0.108598	cg0452701{	cg0452701{	59694500	ACCACTA	61646402	GCCGCTA	I	C
0.1094011	cg0454088.	cg0454088.	55660423	AAACCACAACTAACTCTACCA.			II	
-0.135045	cg0454641.	cg0454641.	27692361	AAACAA/	48797310	AAACAA/	I	T
0.1186059	cg0454767.	cg0454767.	56639317	CCTTAAC	60731417	CCTTAAC	I	A
0.1112536	cg0455150(	cg0455150(	35639474	CTATATACTTATACRTAACTAA			II	
0.1783472	cg0455655(	cg0455655(	30700461	AACAACTTAAATAACTAAACA			II	
0.200849	cg0455950{	cg0455950{	67748477	TAAAAACTACACTACAAAACA			II	
0.1166713	cg0456520l	cg0456520l	26796328	CAATCCAACTTTATTTATTTTT			II	
-0.15425	cg0456533'	cg0456533'	16658336	CCTACCTCCTCTTCTAAACTAC			II	
0.2799587	cg0456647.	cg0456647.	27702400	AAAATCAAATCRTCCTATTACT			II	
-0.112646	cg0457020{	cg0457020{	20621488	CTCTATAAATTAACRCTACTAC			II	
-0.128393	cg0457184'	cg0457184'	47714450	TAAACAA	67730454	TAAACAA	I	C
0.1348925	cg0457331(	cg0457331(	43739323	CTTTCTATTTATTTTCTCTTAA/			II	
0.1361971	cg0457660'	cg0457660'	51641403	TAAAAATCACAATAACACAAA			II	
0.1323446	cg0457745l	cg0457745l	72658302	AATTTTAAAAAACAACTCAAC			II	
-0.108759	cg0457939{	cg0457939{	69669504	CCATCAA	19709324	CCGTCAA	I	T
-0.176432	cg0458034	cg0458034	25651423	ATATTAAACATAAACACRCRA'			II	
-0.12956	cg0458101{	cg0458101{	13632488	AACTAAACRAAATCCCAATTA.			II	
0.108402	cg0458722(	cg0458722(	28773426	ACACATA	49617473	ACGCGTA	I	A
	cg0460079.	cg0460079.	12768416	TTAATACRACCRAAAATAATA'			II	
0.2088451	cg0460997l	cg0460997l	51746418	AAATTTTTAATAAATTATAATC			II	
0.1765318	cg0461074.	cg0461074.	73737474	TACTCCTTTAAATCCAAATACA			II	
0.1120927	cg0461089'	cg0461089'	33741308	TATCCRAATCACTTAAAAAAC.			II	
0.1942991	cg0461345.	cg0461345.	46669321	CTCTAATATTCCCCATCTCACC			II	
0.4747696	cg0461373	cg0461373	53697417	ACCACACATAAACAAAAACAC			II	
0.1152922	cg0461433	cg0461433	58717451	ATTTTTAACCTTTATTCCCTCA.			II	
0.1244565	cg0461988.	cg0461988.	26668420	ACTTAAACTTTAACCTAACAC/			II	
-0.208264	cg0462874l	cg0462874l	36639383	CTTAATATCCCCAACACCACC(			II	
0.1258791	cg0462874.	cg0462874.	62642490	TAAACAAACCCACTTAAAATA'			II	
0.1722574	cg0463367(	cg0463367(	32794308	AAAAAAATCTAAAACACAACT			II	
0.1612622	cg0463772'	cg0463772'	26709488	CCAAACTATCCCTCTTAAATA/			II	
0.1174977	cg0463871(	cg0463871(	51654333	ATATAAATCATACCATATAAA'			II	
0.1920328	cg0464507(	cg0464507(	31737440	ATTTACTAAAACAACCATAAC			II	
-0.206753	cg0464515(	cg0464515(	66707449	AACCAAA	64668343	AACCGAA	I	A
-0.106246	cg0464591	cg0464591	26762316	AATTTACRCAATAACTAAAAT.			II	
0.1300156	cg0464667	cg0464667	17674322	AAACTAACAAACCTCTATAAA			II	
0.15958	cg0464698'	cg0464698'	38752364	CTTATCA	20800510	CTTATCG	I	T
-0.117278	cg0464791{	cg0464791{	34703440	ATAAAATAAAAAAACATCRCT			II	
0.1455866	cg0464958'	cg0464958'	28701345	AACAACTCTAATTTACTCTCA/			II	
0.1153109	cg0465067(	cg0465067(	19801302	CCACACA	12656422	CCACACA	I	A
0.173261	cg0465429	cg0465429	26694475	AAAACATCAAACTTAAAAAAT			II	

0.1113288	cg04656015	cg04656015	48755337	AAAACAAAAAATTAAAACCCA			II	
0.1924902	cg04654770	cg04654770	40674393	TACAAAT	71674460	TACGAAT	I	A
0.1094049	cg04674421	cg04674421	29671443	ATTCCAAAATCATAAAAATTCA			II	
0.1308595	cg04680738	cg04680738	32758383	TCCRCATACTAACATCTATAAA			II	
-0.11316	cg04683035	cg04683035	38634378	ACRAAAACCACATACTATAAT			II	
0.1875597	cg04683516	cg04683516	51686418	AAAAAAACTTAAATTTTACRTA			II	
0.1167014	cg04686117	cg04686117	58738369	TACCATACACCTTCATTCTAAA			II	
-0.1834	cg04687040	cg04687040	70733410	AAACTATAAACCCCAAAAATA			II	
0.107507	cg04689178	cg04689178	65787344	AAACATTTAAAAATAACCAAA			II	
0.1780032	cg04689606	cg04689606	53746496	AACAAAC	74787350	GACAAAC	I	A
-0.110049	cg04689676	cg04689676	52679475	ACTAAAT	38638386	ACTAAAT	I	C
0.1170023	cg04690342	cg04690342	22674468	TCTTTTAACAAAACTAAAAACT			II	
-0.170443	cg04693014	cg04693014	37737489	AAAAAAA	25700413	AAAAAAA	I	C
0.1350315	cg04696840	cg04696840	22623365	CTAAATAAAATACCTTCCTTCC			II	
-0.101318	cg04706655	cg04706655	49736325	CAATCCC	23731389	CGATCCC	I	A
-0.101393	cg04716530	cg04716530	32649445	AAAAAAC	27671323	AAAAAAC	I	A
-0.159669	cg04717802	cg04717802	70612470	TAATAAA	43628375	TAATAAA	I	C
0.1098564	cg04721775	cg04721775	71733492	TTTTAACT	40773328	TTTTAACT	I	A
0.1123685	cg04722610	cg04722610	69631444	CAAATATAATACTACAACACA			II	
0.1140116	cg04723343	cg04723343	56759391	ACCAACT	73789489	ACCGACT	I	C
0.2161292	cg04724556	cg04724556	51798439	CATACCAAATAACAAAAAACT			II	
-0.147038	cg04727458	cg04727458	15647330	ACTCTAAACCACTTCATTTAAT			II	
0.1017349	cg04738028	cg04738028	50687365	CCTATAATCTAACTCTCACTAT			II	
0.1472693	cg04741094	cg04741094	34664418	AAATTCCCTCTAACTATATAAT			II	
0.1384249	cg04741728	cg04741728	29673307	AAAAAAT	55646402	AAAAAAT	I	A
-0.112251	cg04742556	cg04742556	38787419	RACATAATAAACAAATCCCRC			II	
-0.215367	cg04748098	cg04748098	67770356	AACTCAT	59728417	AACTCAT	I	C
0.1400592	cg04750517	cg04750517	12704357	AATCCTATAAAAAACTACAAT			II	
-0.104178	cg04755386	cg04755386	67777468	AAAAATCACATAAACCAATAA			II	
0.126453	cg04762129	cg04762129	59633472	TACATCCAAAACCCAAACCAA			II	
0.1443034	cg04762351	cg04762351	33726399	AAATACAATTATTCCAAACCTT			II	
-0.140483	cg04764476	cg04764476	23729413	TATTTCA	48630353	TATTTCG	I	A
0.1153069	cg04768488	cg04768488	11726510	TAAAACRCTAATCACTCTACAA			II	
0.1100194	cg04775631	cg04775631	63713495	CATTTAATTATCTAATATAAAA			II	
0.2263358	cg04777551	cg04777551	51795368	AATAATAAAAATACCCTAAAA			II	
0.1064841	cg04785902	cg04785902	13637343	TCTTATCTTACTAATATTATCC			II	
0.1086075	cg04786142	cg04786142	21802497	ACTAAAA	69752328	ACTAAAA	I	A
0.3759203	cg04787784	cg04787784	17610381	CCACAAACRACCCCTCAAACT			II	
-0.101518	cg04789475	cg04789475	59774428	AATTAAA	65809342	AATTAAA	I	A
-0.14994	cg04791718	cg04791718	43680426	ATCTATTAAACRAAAATTCTA			II	
-0.176894	cg04798314	cg04798314	22669441	AATAAATATCTTAAATTAACTA			II	
-0.117843	cg04799210	cg04799210	33704489	CAAACTA	58709466	CGAACTA	I	C
0.2156831	cg04801779	cg04801779	70767489	AATAACT	28790376	AATAACT	I	T

0.1436272	cg0481074:	cg0481074:	71757363	TAAAAAAAATAAATTCCTAAT(		II	
-0.359802	cg0481478	cg0481478	23715415	AACCACTACACCCRACCATAA'		II	
-0.109218	cg0481617	cg0481617	52656395	CTTTACCTACAAACCATTATTA		II	
0.1042491	cg0481859	cg0481859	10772349	AACCAACTAACATTTAATACA(		II	
0.3177392	cg0481949	cg0481949	65742498	CAAAATA	50659359 CGAAATA	I	C
0.1312549	cg0482329	cg0482329	27605338	CAACAAATTAATAAAATACAC		II	
0.1199241	cg0482446	cg0482446	60670408	CCCCTCTACCTTATTTCATACT(		II	
0.1084559	cg0482455	cg0482455	35717372	ACCAACTATAACAACTTAACC'		II	
-0.216784	cg0483095	cg0483095	62753323	CACCCCCAACTCAATAAACCT		II	
0.201708	cg0483224	cg0483224	74756468	AAAATAACCTAATACTCCTAA		II	
0.1175054	cg0483565	cg0483565	11793330	AACACTCTAAAACTCCTTCCT		II	
-0.21241	cg0483685	cg0483685	42810329	AAACTACTCAAACTCAAAAAC		II	
0.1381626	cg0484016	cg0484016	21717353	TATACCC	40702418 TATACCC	I	A
-0.122836	cg0484306	cg0484306	14661409	ACTAAAA	53617378 GCTAAAA	I	A
0.1521847	cg0484355	cg0484355	11792390	TTTCTAAATAACTTACATCRAA		II	
0.1584561	cg0484356	cg0484356	20664453	TCRAATACACAAATCACTCCA(		II	
-0.172708	cg0484382	cg0484382	65737502	AACAATTACAATTTTTACCAT		II	
0.1049843	cg0484403	cg0484403	71684466	TACCCTCTTATATATAACCTAA		II	
0.2529703	cg0485014	cg0485014	49780437	TACCTAACCATAATCCATATA		II	
0.1290499	cg0485028	cg0485028	40677367	ACACCCTAAATAATAACCATA		II	
-0.120543	cg0485239	cg0485239	66716355	AATCTAAACTAAATTTCATAT		II	
-0.101041	cg0486043	cg0486043	47788359	AATCTAAATATAATTCTCTCCT		II	
-0.209451	cg0486419	cg0486419	64737453	CTAAAATAACCRCCATCTAAA(		II	
0.3512255	cg0487570	cg0487570	64738345	AAACCRTTTTTAAATCCCATCC		II	
0.1251799	cg0487815	cg0487815	11640487	CAATCTCAAAATAATTAAACC'		II	
-0.186162	cg0488009	cg0488009	72782399	AAATAAC	30807398 AAATAAC	I	A
-0.14126	cg0488506	cg0488506	37672455	CAAACTA	19804385 CGAACTA	I	T
0.1144918	cg0488534	cg0488534	33675510	CAATCTATAAATAATTAACAT		II	
0.1733555	cg0489060	cg0489060	31650394	AAACCCA	33631300 AAACCCC	I	A
0.2286561	cg0489157	cg0489157	71749414	ATCTAATATTTTTCCRTAAATT		II	
0.1555071	cg0489264	cg0489264	44686303	AACCACACTAATAATTTCCCA		II	
0.1589648	cg0489276	cg0489276	32609356	AAATAAACAAATTAAAATTAA		II	
-0.211237	cg0490018	cg0490018	51623431	CAACATAAATATTCAATCAAC		II	
-0.132756	cg0490456	cg0490456	46702384	CATTAAT	28655481 CGTTAAT	I	T
0.1203514	cg0490784	cg0490784	38628462	AAACCAAACCAACAATAATAC		II	
0.1138681	cg0490807	cg0490807	59694326	CTCCCTTTACAATTTTTCTCAA'		II	
-0.106567	cg0490862	cg0490862	39653503	CTCAATA	65636462 CTCGATA	I	A
0.1052226	cg0491422	cg0491422	39603336	AAAACTATAATCCTAACCTTT		II	
0.1503927	cg0492638	cg0492638	55636431	AAAATTAAATAATAATAATAT'		II	
0.1298669	cg0493069	cg0493069	57798484	CCCAAATATAAAACTAAACTA		II	
0.1288934	cg0493510	cg0493510	52753423	AATATCACCAAAACCTCCATC(		II	
0.1221807	cg0494485	cg0494485	11722332	CTCAACRCTACAACCCAAAAA		II	
0.1257482	cg0494755	cg0494755	58797382	AAAATACATAAAAAATACTTA		II	

-0.147968	cg0494768(	cg0494768(	52674493	TCAAAAT	40797436	TCGAAAT	I	T
	cg0495511(	cg0495511(	55807479	AAAAAA(	32768451	AAAAAA(	I	A
0.1113398	cg0495790:	cg0495790:	23701353	ACCAACAAAAAAACCTAACTA			II	
0.1345447	cg0496166:	cg0496166:	43701398	CRTAAAACAAATAATACCCCT.			II	
0.1103942	cg0496369:	cg0496369:	39699304	TTACCTATCATAACAAACAAA'			II	
0.1683867	cg0496980{	cg0496980{	31745472	AAATTAAACAACCTCCTCTAA/			II	
0.1055207	cg0497028:	cg0497028:	42628435	ACAACRAAAATATTTATAACC(			II	
0.1215911	cg0498632/	cg0498632/	10608343	ATATATCTATACRTATCCRTAT			II	
-0.166239	cg0499037{	cg0499037{	57701483	AAACAACCCCAAATATACAAC			II	
0.1172676	cg0499327{	cg0499327{	49795447	TAACCAAAACCAATAAATCTA			II	
0.1568935	cg0499360:	cg0499360:	40755441	ACTATAAAACTATTCTATATAT			II	
-0.108765	cg0499531l	cg0499531l	61765307	TACAATC	60715439	TACAATC	I	A
0.1494396	cg0499832:	cg0499832:	66624499	TATAAACCTCATAACTCCCTCT			II	
-0.123346	cg0500193:	cg0500193:	58718381	ACCTAAAACCTCTTATTAAAT/			II	
0.176515	cg0500507:	cg0500507:	68653306	TAATCCCACTAACTTACTTTAC			II	
0.1345117	cg0500538:	cg0500538:	43602318	TTAACAT	35604442	TTAACGT	I	T
-0.152566	cg0500623l	cg0500623l	29722314	AAAAACT	32626329	AAAAACT	I	A
0.1249706	cg0500882:	cg0500882:	29686412	CTTCCAATAAAACCAATAAAA'			II	
0.1239067	cg0501064{	cg0501064{	62808422	CTATCATTTTATTCTCACCTCT(			II	
-0.159243	cg0501472l	cg0501472l	35803400	AAATATA	11650317	AAATATA	I	A
0.2833083	cg0502319:	cg0502319:	73670346	TTTTAACAAAAAACAATTCTT(			II	
0.1212705	cg0503284{	cg0503284{	40772427	TTAAAACAATCAATAAACTAC(			II	
0.1131561	cg0503323{	cg0503323{	56793502	CTTTTTCTTTCATATTTCTAAA/			II	
0.1005955	cg0503336{	cg0503336{	13689323	TCTAACTATCCAAAACCCACA(			II	
0.1299331	cg0503506l	cg0503506l	31708457	TCACTCTTCTACCCTAATAACC			II	
0.1602242	cg0504034{	cg0504034{	17788344	AATCCTAATATTTAAAATTCA(			II	
-0.103455	cg0504050l	cg0504050l	56744475	ACTAAAAAATAATATAAACRT.			II	
0.1036565	cg0504371(	cg0504371(	44750351	CCAAAAATTACRATAAAAACC			II	
0.1296899	cg0504658{	cg0504658{	10713425	AATATCTCTAATTACATTAAC/			II	
0.1242753	cg0504672:	cg0504672:	73735407	AACCTAATCAAACRTAATATCI			II	
0.131389	cg0505005:	cg0505005:	57704510	AATACTT	69704315	AATACTT	I	T
-0.124149	cg0505126:	cg0505126:	27733467	AAAAACAAAAAATACACRTAC			II	
-0.110862	cg0505468:	cg0505468:	47799466	AAAACA/	29804509	AAAACA/	I	A
0.1422479	cg0505500l	cg0505500l	53600355	AAAATAAACCAAAACCACAA/			II	
0.1027131	cg0505735:	cg0505735:	48676426	CCCAAAACTCCCAATAAATAA			II	
0.1451842	cg0506248{	cg0506248{	55672506	ACTATAAATCCAACTCCTATA/			II	
0.1014925	cg0506584(	cg0506584(	36760325	ACTCRACTAAAATAATTCTAT/			II	
0.1031162	cg0507027:	cg0507027:	74735361	ATATATACAACCAATATAAAC			II	
0.1206561	cg0507530{	cg0507530{	53659444	TAATATTCACCRTACTATCCAC			II	
-0.140913	cg0507904{	cg0507904{	37750481	ACAAAAT	20718489	GCGAAAT	I	C
-0.111791	cg0508644/	cg0508644/	40631330	CTTACTCATTCAACAAATATTT			II	
-0.157891	cg0508762:	cg0508762:	65796378	TAAACTACAAATCTCTATATCT			II	
-0.125947	cg0508815l	cg0508815l	70699479	CCAAACC	11603418	CCGAACC	I	A

-0.113782	cg05092371	cg05092371	44765425	TACAAACAAACAATTATAACA			II	
-0.199012	cg05096415	cg05096415	16639308	ACTATATAACTTACATCATCAT			II	
0.1013669	cg05096637	cg05096637	23740403	ACAACCCCRAACCTACATCATA			II	
-0.102611	cg05100067	cg05100067	74770356	CTCTACTATCCTCAAATACAAA			II	
0.1038341	cg05108467	cg05108467	49607442	CRAATATTTTAAAATCTCRTC			II	
0.1271619	cg05109569	cg05109569	63659395	TTCTCAAAATAACRACTAAAAC			II	
0.1787525	cg05111645	cg05111645	35658390	TATACTCAAAAAACCCTCAATT			II	
0.1428963	cg05111898	cg05111898	60744436	AAATACCCTCTAAACCTCCAAA			II	
0.2171689	cg05126514	cg05126514	31756325	AACATACAATCTACATATATCC			II	
0.1236487	cg05131488	cg05131488	22610307	ATAAACTAACACTCTCAAACRC			II	
-0.107053	cg05131696	cg05131696	26788413	AATTAAAACACTTACTTCAATC			II	
-0.200623	cg05134736	cg05134736	21667314	CATTATCCACCTAACAAAACTC			II	
0.1228428	cg05138892	cg05138892	23627462	CATTATATATATCTATATACRT			II	
-0.125149	cg05139028	cg05139028	44804476	TAATCTAATCTCCACAAACTCA			II	
0.1004843	cg05144814	cg05144814	43802320	CTACCAACATATAAACCCTCAA			II	
0.1215612	cg05155047	cg05155047	74711312	CAAACAAATTCCCAAACTTCCT			II	
0.1017769	cg05157625	cg05157625	67795434	ACACCTACCRAACTTTACCTTT			II	
0.1045678	cg05160563	cg05160563	37643374	TACTCCTCTTACTCTAACATAA			II	
-0.265401	cg05161773	cg05161773	17630455	AATAACAAAAAAAACTAACCA			II	
0.1317914	cg05164540	cg05164540	27745334	TAAATCCAAAAAAATCACTAT			II	
-0.10681	cg05164937	cg05164937	60782338	AAAACAA	35771437	AAAACGA	I	T
-0.11581	cg05165250	cg05165250	11635354	AAAAATTAAACTACAATAATA			II	
0.2777572	cg05173889	cg05173889	46787473	CAATAACAAACTACCTAACCCT			II	
0.1403801	cg05175803	cg05175803	48646417	AATACCCAAAATATAATCCAA			II	
0.1517784	cg05176551	cg05176551	36699356	AACACCTACCTCTAAACTCCAA			II	
0.1332507	cg05179499	cg05179499	30707435	CCCTTTTACCTACCCTTAACAA			II	
-0.109506	cg05181941	cg05181941	23806365	TATCAAC	74751351	TATCAAC	I	A
0.1497556	cg05182217	cg05182217	61755450	ACTTACAATAATACAAATTCRA			II	
0.1317675	cg05187154	cg05187154	30652418	AATAAAAACCCCRAACATTTA			II	
0.1635193	cg05194426	cg05194426	29773460	AAATAAACTAATAAAAAACRC			II	
0.1388138	cg05194636	cg05194636	50704485	ACCCAAAAACRCAACCRAAAA			II	
0.1200731	cg05195318	cg05195318	64698331	AATTTCTATTTACACACCTATA			II	
0.1668587	cg05197062	cg05197062	15746499	AAATCTA	57747438	AAATCTA	I	A
0.1050373	cg05197667	cg05197667	30610409	AATTTCTTTTCCAACAACATAC			II	
0.1372126	cg05208607	cg05208607	35706389	ATCACATCAAAATTCTTCAAAA			II	
0.1021207	cg05210689	cg05210689	50646447	AAAATAAAAATTTATAACCCA			II	
0.2172234	cg05212892	cg05212892	20718326	RCTACTATATACCTAAACCTCT			II	
0.1041952	cg05223441	cg05223441	19777426	ATTTCCTCTATCCTAATTAACC			II	
	cg05226061	cg05226061	13644381	CTTCCCTC	49734466	CTTCCCTC	I	C
0.4795203	cg05232943	cg05232943	55732352	RCAAAATAATAACATAAACRA			II	
-0.140502	cg05234568	cg05234568	22664389	CAACTAC	63716334	CAACTAC	I	C
0.1494007	cg05237260	cg05237260	38699421	TTATCCCAACCRTACAAATAAA			II	
0.1379437	cg05241828	cg05241828	69780466	AAACCTA	22600367	AAACCTA	I	A

0.1098703	cg05244013	cg05244013	60716387	AATATACTCTTTTACCATATTA			II	
0.1067112	cg05244234	cg05244234	63742314	AAAAAAC	55731365	AAAAAAC	I	A
0.1502319	cg05244682	cg05244682	61615347	AACTTAATTTCATCCTATAACC			II	
0.1439517	cg05245515	cg05245515	46695455	TTTCCTATTTTAAATTAAACCA			II	
-0.18562	cg05248234	cg05248234	35685425	TTCATCA	33667479	TTCGTCG	I	C
0.101046	cg05250932	cg05250932	16606320	ATCAAAA	24728332	ATCAAAA	I	A
0.1429833	cg05258009	cg05258009	22771355	TTAAACA	74616339	TTAAACA	I	T
-0.107865	cg05265884	cg05265884	44763466	ACACAAC	18750386	ACGCGAC	I	A
0.1137545	cg05266786	cg05266786	17733366	CAAACCACATAAACAACAAAA			II	
-0.324088	cg05267394	cg05267394	24754470	AAAAACA	74622419	AAAAACA	I	C
0.1231239	cg05269323	cg05269323	56681340	CTAAAAAACAAATCTTTAAAC			II	
-0.11427	cg05270750	cg05270750	60810486	AAAAATAAAACRCRATAAATA			II	
0.1116974	cg05274316	cg05274316	30668497	CTCCCAAACAATACAAATATT			II	
0.1169264	cg05276155	cg05276155	55614329	TTACTAAAAAACRAAATCAAA			II	
-0.137768	cg05276469	cg05276469	56713435	CCAAAAACCAATAACRAAAAC			II	
0.2815802	cg05276972	cg05276972	48800415	RACCCTAAATACTCCTAACTTC			II	
0.1855587	cg05277504	cg05277504	14769334	AAAAAAC	22620442	AAAAAAC	I	A
0.1941355	cg05279330	cg05279330	65802421	ACTTAACAATCTAACCAAAAT			II	
0.1485584	cg05279738	cg05279738	10734455	CTATTCTTTATTTAACTTTTTC			II	
0.1077269	cg05280336	cg05280336	46730385	TAATAACCTAAAAAACCTACA			II	
0.1013643	cg05282260	cg05282260	54763316	AACCACC	74643422	AACCACC	I	C
0.1347631	cg05284727	cg05284727	26726429	TAAACCAAACCTAAACTTTAT			II	
0.1187361	cg05287372	cg05287372	26636348	TAATACTCACAACATTCCAAA			II	
0.1394243	cg05288172	cg05288172	43740492	AACRTACACCAAACTAAACAA			II	
-0.103978	cg05301470	cg05301470	34644326	TCCTTTC	16671467	TCCTTTC	I	A
0.1189604	cg05302100	cg05302100	63795474	TTTTTTTTTCTAAACTATTTAA			II	
0.1033098	cg05306029	cg05306029	57645327	TATAAATAACTAAACTTCCTA			II	
0.2004601	cg05308244	cg05308244	24788496	CCTAAATATATCCCTAAACCA			II	
0.1420778	cg05309989	cg05309989	43799373	AAATAACTAAAATTTCACTTA			II	
-0.114019	cg05310990	cg05310990	61756328	TAAAAAC	12600421	TAAAAAC	I	A
0.1212933	cg05313910	cg05313910	66616450	TAACTACAATTTCCCTCTCTTC			II	
-0.128258	cg05317207	cg05317207	55733446	CRATAACTTAAAAACTCCRAC			II	
0.1296286	cg05318275	cg05318275	29749314	CTTAATAACATACTAACCTCCC			II	
-0.121085	cg05321225	cg05321225	18708411	CCCACAC	26771434	CCCACGC	I	C
-0.190238	cg05328057	cg05328057	12687466	AAACATA	40657444	AAACGTA	I	A
0.5680553	cg05331763	cg05331763	73723426	CCAAAACTAAACCTAACACRC			II	
-0.108111	cg05336056	cg05336056	61607478	AACCTATACAACATTTATTATC			II	
-0.385771	cg05337761	cg05337761	29669478	TCTCTAC	52623471	TCTCTAC	I	A
-0.170434	cg05338731	cg05338731	63790402	TTTACCTTAAAAAACTAAAAA			II	
0.1237965	cg05342469	cg05342469	33611312	AAACTAACATATTACTTATTCC			II	
0.1192163	cg05343811	cg05343811	37807421	TCTCATT	22788356	TCTCATT	I	A
-0.209993	cg05344495	cg05344495	41766314	ACTAAAC	59774305	ACTAAAC	I	A
-0.210824	cg05345154	cg05345154	71714446	TCAAACC	74795345	TCGAACC	I	A

0.1585917	cg05348852	cg05348852	47681322	TTCCATACCCAAAACAAATTCC			II	
0.1080503	cg05349016	cg05349016	48603467	CATCCCAAAAACCAAAATTCA			II	
-0.105689	cg05354195	cg05354195	43785387	AAAAATCTCCCAAATTAATACA			II	
0.1410312	cg05357039	cg05357039	73681375	CTAAAAACAAATAATAATATTT			II	
	cg05360176	cg05360176	13644444	ATTCTCCT	57798408	GTTCTCCT	I	C
-0.126915	cg05362314	cg05362314	51624441	ACCCCCCAATCRCAAAAAATAT			II	
0.1227667	cg05363955	cg05363955	48629338	CAACCAAACTATCTTCCAAACC			II	
-0.106848	cg05364567	cg05364567	35650419	AAACACTTCTAACCCATATAAA			II	
-0.17692	cg05365033	cg05365033	50628338	AAAAAAT	50656399	AAAAAAT	I	T
0.104484	cg05366050	cg05366050	36747348	TATTTCAAAATATCCTAAATTT			II	
0.167404	cg05366160	cg05366160	46809478	CAAAAAACCTTCCCTAATATAC			II	
-0.117575	cg05370638	cg05370638	62802309	AAAAAAC	14641348	AAAAAAC	I	T
0.1564118	cg05372444	cg05372444	68766487	AAAACAC	12685351	AAAACGC	I	A
0.1162392	cg05374809	cg05374809	18625342	AAAAAAACAACCATCAATTCC			II	
0.1001878	cg05377120	cg05377120	43729306	AACAAACAAATAAACATATAA			II	
-0.179402	cg05378695	cg05378695	58773343	AAATTACCTACATAACCCTAAA			II	
0.144327	cg05383619	cg05383619	34752490	ATTTTACATAAATAATCACCAC			II	
-0.149899	cg05385718	cg05385718	24681459	CACAACA	20709500	CGCAACA	I	C
	cg05387399	cg05387399	55706492	AAACCCC	67765452	AAACCCC	I	A
-0.22011	cg05392448	cg05392448	18694399	CAAAAAC	53806343	CGAAAAC	I	C
-0.107243	cg05392989	cg05392989	48634406	AAACAACCAAAAATCTCACCC			II	
0.114861	cg05393086	cg05393086	60695447	AAAATACCATCAACTAAAAAA			II	
0.1004261	cg05400732	cg05400732	22781377	CAAAATTATTTATACAAACACT			II	
0.2782376	cg05401575	cg05401575	16750363	ATTTATTTAACAAACATAACAA			II	
0.3741565	cg05407200	cg05407200	36709476	AAACCATTCTAAACCATAAAA			II	
0.162138	cg05408674	cg05408674	25687314	ATAATTCAACTTTACTTCTCAA			II	
0.1075279	cg05411594	cg05411594	29600456	TCRTACAACTCTAAAACAAAAT			II	
-0.148124	cg05412137	cg05412137	27777372	AATATTTAAAACATCCRTACTT			II	
-0.157329	cg05412957	cg05412957	70659300	AATATACTATTCTTAAACAAAA			II	
0.1172507	cg05413061	cg05413061	47649504	AAAAAAATAAATCACAACATT			II	
-0.166956	cg05416337	cg05416337	23758313	AAATTTCCCTAATAATAATTTT			II	
0.1118639	cg05419622	cg05419622	15679354	AAAAAAACACTAACCATTTAA			II	
-0.151924	cg05424366	cg05424366	62680335	AACTCRAAATCACTAAAATACC			II	
0.1338157	cg05428452	cg05428452	45790381	TAAAACTCCTCRTCTCCATCAA			II	
0.1041546	cg05432034	cg05432034	65788499	CTAATACAACTTCTTACAAATA			II	
0.103694	cg05435390	cg05435390	54763400	ACTACCTCAACAAAATACAAA			II	
0.2603966	cg05440824	cg05440824	31727406	ATACTTAAAACATATTAATAAC			II	
0.1352552	cg05450336	cg05450336	18797427	AACAAAAATATAAAACTAACA			II	
0.2218158	cg05453001	cg05453001	28709352	AAAAACA	48752321	AAAAACA	I	A
-0.100401	cg05453358	cg05453358	26780499	ACAACAA	39755368	ACGACGA	I	A
0.1402938	cg05455372	cg05455372	25618490	ATCTCCAAAAATAACACTCCTT			II	
-0.187119	cg05457730	cg05457730	20682433	ATCAAAA	46804394	ATCAAAA	I	A
-0.229815	cg05463589	cg05463589	74757478	CTAAACA	42741510	CTAAACG	I	A

0.1610511	cg05469819	cg05469819	27641340	AATCACAAAAAAATTAATTAA			II	
0.1469649	cg05471169	cg05471169	66715450	AAAAAAA	58792400	AAAAAAA	I	A
0.1712424	cg05471495	cg05471495	35724480	TCCTATTCTCAAAAACTTATCA			II	
0.1544971	cg05472468	cg05472468	38742324	TAACTCC	51726304	TAACTCC	I	A
-0.134758	cg05475524	cg05475524	11770420	ACTACAA	54677365	ACTACAA	I	A
0.2005414	cg05477582	cg05477582	72609395	ACACCAA	29778323	ACGCCAA	I	A
0.1164855	cg05479627	cg05479627	41672359	TACTTATCTAACRTATATATAT			II	
0.1170493	cg05481961	cg05481961	20808492	TCCTTTAATCAAAAACATAAA			II	
-0.100096	cg05483388	cg05483388	64758315	TTAAAAACAATTACAAAAACT			II	
0.1025054	cg05483731	cg05483731	33804472	AAAAAAA	66666305	GAAAAAA	I	C
0.1631417	cg05484949	cg05484949	74646447	AAAACAA	32655314	AAAACAA	I	C
0.1128594	cg05486191	cg05486191	13749432	ATAAAAACCACRTATTCATTCC			II	
-0.448981	cg05502360	cg05502360	29714300	AAATAAAAAACCAACTCATTC			II	
0.2380519	cg05509228	cg05509228	16658506	CCRATTCAAAAAAACCCAAAA			II	
0.1202888	cg05513157	cg05513157	59733359	CCCTTAACTTCRTCAATAAACR			II	
0.1240068	cg05514299	cg05514299	19661387	TTCAAAACTACAACTATTAATA			II	
0.2003614	cg05515244	cg05515244	48649474	ACRAATAATTAACATTATTATT			II	
0.1358333	cg05524038	cg05524038	13743318	TCCCTTCAACTCTAAATCTCAT			II	
0.1045081	cg05526364	cg05526364	47799399	CATCATATACTCAACTATAATA			II	
0.1586787	cg05526809	cg05526809	44650361	RAAAAACATACCACAAAACRA			II	
0.1396535	cg05528899	cg05528899	54661458	ATAAAAATCCTCTAACCCRCTA			II	
0.389017	cg05531409	cg05531409	13799421	CAATTCAATTTAAATCCCTTAA			II	
-0.109041	cg05548393	cg05548393	70714466	AAACAACCAAAATTAAAACTA			II	
0.1409407	cg05548425	cg05548425	50692329	ACAAATAATCACTTAAATAAA			II	
0.1509393	cg05563515	cg05563515	33659451	TAAAAAA	37700348	TAAAAAA	I	A
-0.162678	cg05574272	cg05574272	67759423	ACCTACA	31698311	ACCTACG	I	C
0.2734885	cg05575505	cg05575505	44635308	AACTCTATCCACATAAAACTCA			II	
-0.107583	cg05575614	cg05575614	64745329	ACACAAC	21727313	ACACAAC	I	A
0.2687097	cg05579598	cg05579598	25738399	RACTACAAACTAAAACCCRAC			II	
-0.191477	cg05580809	cg05580809	64802438	CRATCCTTCRACTATAAACAAT			II	
0.1690214	cg05581878	cg05581878	30628482	AAATAACAATAATAAAACCCA			II	
0.1204754	cg05583636	cg05583636	46659302	AACAAAACAAAAACCCTATCT			II	
-0.105188	cg05589784	cg05589784	11736345	AAATCCA	69604436	AAATCCG	I	A
-0.123888	cg05590257	cg05590257	30699439	ATCCCAA	69690416	ATCCCGA	I	C
0.1003525	cg05597181	cg05597181	32754347	CCTACCA	62789334	CCTACCG	I	T
-0.111254	cg05601623	cg05601623	24741423	ATCTTACCTACTCCRAAAACCT			II	
-0.185963	cg05606115	cg05606115	19715310	ACTTTTCCTTACTAAATCAATT			II	
0.1439319	cg05606455	cg05606455	19706326	ATATTAA	12671388	ATATTAA	I	C
-0.114523	cg05612744	cg05612744	18642335	TAACRATAAAAACTATCACCA			II	
0.3043037	cg05621349	cg05621349	31682310	AACAAAC	67795408	AACAAAC	I	A
0.1138893	cg05622577	cg05622577	27694449	CATTCCCAACCAAAACTTCACA			II	
0.1189468	cg05628506	cg05628506	41697390	ATATTACAAAAATATTCCCTAA			II	
-0.133628	cg05630111	cg05630111	44650460	TTTTATAA	45650392	TTTTATAA	I	A

-0.10503	cg0563027	cg0563027	63604429	ACCTATAAATCTATTACACAA			II	
0.1078616	cg0563463	cg0563463	71771403	AATTACCTTATACTTCTTACTT			II	
-0.109749	cg0563617	cg0563617	29763331	ATACTTAAAAATAACTACTAC			II	
0.1863303	cg0563864	cg0563864	29643404	TCCTTCT	46795366	TCCTTCT	I	A
0.1016888	cg0564053	cg0564053	28683489	CAACTAC	22722453	CGACTAC	I	T
0.2152731	cg0564907	cg0564907	57772441	CTTACCAAAAATAACACATTA			II	
0.1239316	cg0565348	cg0565348	59616435	CTTAACTAATAACCRATACTT			II	
-0.397383	cg0565621	cg0565621	17758490	TCCACAA	64650421	TCCACAA	I	T
0.153934	cg0565765	cg0565765	20687501	ATAAATTACACAACAAAAATT			II	
-0.139522	cg0566682	cg0566682	51636353	AATCAAA	49620410	AATCGAA	I	C
0.125814	cg0568212	cg0568212	54772377	CAAATCAACTATATAAAACTA			II	
-0.180482	cg0568363	cg0568363	11611477	AAAAACA	63673338	AAAAACC	I	A
0.1214208	cg0568941	cg0568941	43736462	ATCACAAAACRTTCTCAAATA			II	
0.1117509	cg0569064	cg0569064	61628317	TCAACTC	41624466	TCAACTC	I	A
0.1143194	cg0569569	cg0569569	55753504	TCCACCT	70682427	TCCACCT	I	A
0.1012287	cg0569587	cg0569587	51767359	CTCTACTTTTAACTACAACTAA			II	
0.1174988	cg0570147	cg0570147	15736509	AACTAAAATCACAACACTAAA			II	
-0.139273	cg0570494	cg0570494	54720467	CAACACCAACAAAAACTTCCA			II	
0.1090108	cg0570533	cg0570533	57763359	AAATTCA	24659427	AAATTCA	I	T
-0.115299	cg0571006	cg0571006	58692328	TTACTTAATTATCAACCTCATC			II	
0.1000165	cg0571385	cg0571385	24654401	AAACAACACCAATATCAATAA			II	
-0.13146	cg0571395	cg0571395	15619486	TAAACTACTCCTAACTAAACR			II	
0.1196154	cg0572203	cg0572203	37645504	ACCCTATTAACCTTCAAACTA			II	
0.1098046	cg0572317	cg0572317	19643461	ACAAACTTTCTCTCCAAACTC			II	
-0.1285	cg0572477	cg0572477	42650320	ATCACCA	17720392	ATCGCCA	I	C
0.3021189	cg0572816	cg0572816	38761400	TACCTAAAATCAAAATAAAAA			II	
0.2222957	cg0573097	cg0573097	57661493	TCATTAAATATCACCCAAACA			II	
0.102447	cg0573346	cg0573346	12626393	AAAATATTAATCATTTCAAAA			II	
0.1049355	cg0573874	cg0573874	26798357	TTCATTTTCAATTTAAACCAAA			II	
-0.116608	cg0574046	cg0574046	26607306	ACAACAT	68601390	ACAACGT	I	T
-0.116828	cg0574371	cg0574371	10754333	AATATAT	21656445	GATATAT	I	A
0.5204028	cg0575105	cg0575105	71632416	AAACAACRAAAACTATACTCT			II	
0.1237844	cg0575118	cg0575118	62667342	AATCAAATCAAAACAAATAAT			II	
-0.115674	cg0575897	cg0575897	15708458	ACACACA	48696497	ACACACC	I	T
-0.119166	cg0576660	cg0576660	57620314	TTAAAAT	40810434	TTAAAAT	I	A
0.1071362	cg0576741	cg0576741	31782365	ACTACTT	34773427	ACTACTT	I	A
-0.132488	cg0577215	cg0577215	60764301	TACAAAC	51746329	TACGAAC	I	C
0.1295854	cg0577586	cg0577586	21695381	TCTCTAAAAAACTTTACATATA			II	
0.1103008	cg0577959	cg0577959	65709488	AACAAAACRAACTATACTTCT			II	
-0.128521	cg0578141	cg0578141	29648301	CAATCCC	64671432	CAATCCC	I	A
0.1126289	cg0578542	cg0578542	14634377	ACTACCA	37653502	ACTACCA	I	A
0.1286567	cg0578614	cg0578614	34710350	ACTTTATTAACATCTTAAAACA			II	
-0.253525	cg0579082	cg0579082	57639389	TCCAATAATCTAACCCAACTA			II	

0.1079503	cg05797854	cg05797854	34754487	TCAAATAAAAAATACTACTTA			II	
0.1077369	cg05799169	cg05799169	45648303	AACCTTC	49757422	AACCTTC	I	C
-0.105085	cg05806645	cg05806645	42653354	CRCTCTATACCACRAATATCA			II	
0.1028816	cg05809586	cg05809586	47748455	AAAACTAAAAAACACAAACCA			II	
0.1023344	cg05819221	cg05819221	19633331	AAAACCRTATCTATACRAAAA			II	
-0.104666	cg05820861	cg05820861	61761478	CAAAATA	42702498	CGAAATA	I	C
0.1063693	cg05828605	cg05828605	17657409	TATAACCAAAACTCAAAATTT			II	
-0.103375	cg05831083	cg05831083	60732331	ACATAACTTAATATAATAATT			II	
-0.102889	cg05833351	cg05833351	43687316	AACATCT	60660364	AACGTCT	I	T
-0.176465	cg05837990	cg05837990	67797503	TAATTCC	63734392	TAATTCC	I	C
-0.142381	cg05839741	cg05839741	31774410	AAAAACC	27712404	GAAAACC	I	A
0.1539037	cg05839762	cg05839762	60790473	TCTCACA	42737448	TCTCACA	I	C
0.167635	cg05845592	cg05845592	15645506	ATTACTC	58733352	GTTACTC	I	A
0.1202675	cg05847075	cg05847075	71762411	CTATTCCTCCCACTCCTCTTAA			II	
-0.267051	cg05852276	cg05852276	24780491	AACAAAC	33620417	AACGAAC	I	A
-0.187676	cg05857999	cg05857999	35725469	TTTACAA	53770372	TTTACGA	I	A
-0.292814	cg05860956	cg05860956	46756506	CTAAAAAATCAATACTAACTA			II	
0.13427	cg05865243	cg05865243	60680491	ACCACCC	45667398	ACCGCCC	I	A
-0.115862	cg05867245	cg05867245	72691431	AACCAACTCTAAAAAAAACCT			II	
-0.119895	cg05867884	cg05867884	70766497	ACRTCAAAAAAACAATAACCA			II	
-0.12392	cg05868531	cg05868531	23655430	AAAAAAC	16636475	AAAAAAC	I	C
0.1825105	cg05876883	cg05876883	62609471	AAAATAAAACTCATAACTAAA			II	
0.12635	cg05879075	cg05879075	19630488	ATAATATAAACAAAAAAACCA			II	
0.1045134	cg05888894	cg05888894	36646450	AAACCCTTATATATAACCTCTA			II	
0.1826608	cg05890457	cg05890457	73789453	TCATCTCTAAACCAAAATAAC			II	
0.1043198	cg05890956	cg05890956	28736421	AAACATTAAAACCCAAATATC			II	
0.2063798	cg05893845	cg05893845	47708472	TAAATCAAAAAATAAACTTTA			II	
-0.124635	cg05896682	cg05896682	43787408	ATAAAATAATTAAAACRACRA			II	
-0.211279	cg05900567	cg05900567	64696429	TAACCTCAATTTCTTATAACCT			II	
0.2875285	cg05901462	cg05901462	61780480	TATCTAA	59786389	TATCTAA	I	A
0.103522	cg05903720	cg05903720	15673451	TACACTTATAAATCTACRAAC			II	
0.1163308	cg05914894	cg05914894	34617449	TTATCTCTATATATCTATCAAC			II	
0.1645596	cg05918715	cg05918715	47716437	CCTTTAAAATCAACTCTATAA			II	
-0.183127	cg05921762	cg05921762	68642362	TTTTAATCTTATTCTTTCAAAA			II	
-0.117598	cg05922210	cg05922210	55756442	ACTATAAATCCACCCAAACAT			II	
-0.125757	cg05922253	cg05922253	29795415	AAAAAAC	18626373	AAAAAAC	I	A
-0.221542	cg05925927	cg05925927	19725472	CTAAATAACTATAATCCATCC			II	
0.1276935	cg05929272	cg05929272	68636326	TTCACCRTCCTAAAAAAACRA			II	
0.1117074	cg05929864	cg05929864	57686485	TTACTACTCTATTAATTTATTA			II	
-0.262526	cg05931360	cg05931360	61691305	AATATCTAAAATATTCTCTCCT			II	
0.1001805	cg05935904	cg05935904	19706355	TCTACRATCTAAACCTAAACT			II	
-0.10661	cg05936219	cg05936219	73743360	ACTAATT	65633311	ACTAATT	I	A
-0.154755	cg05938207	cg05938207	13669487	ACCTAACRCTAAATACTAAAA			II	

	cg0594045.	cg0594045.	42729309	TAAAAAC	54606483	TAAAAAC	I	C
0.154151	cg0594234	cg0594234	66605322	AAAAAATAAAATAAATAACCC			II	
-0.207101	cg0594245	cg0594245	33666445	ACTAACA	24706418	ACTAACA	I	T
-0.13514	cg0594578	cg0594578	11806482	AACTCCC	41603461	AACTCCC	I	A
-0.100856	cg0594895	cg0594895	69613476	TAACTCTACTCCRTTTTATATA			II	
0.1134995	cg0594896	cg0594896	65710411	ATTTATATTCACCATCTATAAT			II	
0.1361882	cg0595057	cg0595057	57658365	TTACCTACACAAACATTTCRTA			II	
0.1219976	cg0595324	cg0595324	19760425	CCACCAAATTCTTCTTTAAAAT			II	
0.255165	cg0595651	cg0595651	14636450	CACAATTTATTCACTAACCCCT			II	
0.113433	cg0595754	cg0595754	21789302	TATCACA	21635341	TATCACA	I	C
0.1665938	cg0595943	cg0595943	73612424	AATTATTACTACCTCRAAAACA			II	
-0.114896	cg0596643	cg0596643	59724427	TAACCCTTTCCCATTATTAAAA			II	
-0.487064	cg0597110	cg0597110	74626404	AATATTACTCTTTCCCCTTACT			II	
0.1229206	cg0597191	cg0597191	22669432	AACAACA	35631500	AACGACA	I	A
-0.106744	cg0597733	cg0597733	32673456	AACCAAACAATTAAAACCAAT			II	
0.1190154	cg0598103	cg0598103	66692405	TAAAATCCTTCAACATAATCCT			II	
-0.135875	cg0599481	cg0599481	30625300	CTAACAA	30682303	CTAACGA	I	C
0.1277404	cg0599497	cg0599497	24671300	CTATACCCCTCAACACATAACT			II	
0.1356093	cg0599936	cg0599936	65737401	AAATTTCTCCTCTTAAATACAA			II	
0.1018711	cg0599962	cg0599962	74634432	TAAAATCTAAAATCTAATCAA			II	
0.1194566	cg0600087	cg0600087	57671398	ACTACCCCTTAACTAATACTA			II	
-0.177669	cg0600220	cg0600220	56698398	TCRACCTACCTAAATTTCAACC			II	
-0.132919	cg0600268	cg0600268	24632477	AACTATT	52784353	GACTATT	I	A
0.1292222	cg0601339	cg0601339	47646471	ATTATTTATAATTTACCRAACR			II	
-0.12818	cg0601376	cg0601376	64700324	TACTAAA	57768426	TACTAAA	I	C
-0.179508	cg0601668	cg0601668	35736339	AATATATTTAAAATAAAAATA			II	
0.1182234	cg0602199	cg0602199	49639420	ACACAAATCCACAAATTACAT			II	
-0.283512	cg0602335	cg0602335	36691480	TTAACAACRCTAATTTACAAA			II	
0.1100895	cg0602379	cg0602379	53738457	AAAAAACRCRAAAATTAAAAA			II	
0.4077285	cg0603233	cg0603233	48708300	AACACATAAACRCATTCAATA			II	
0.1025201	cg0604494	cg0604494	71687311	ATAAAAAACATACAAACCTCT			II	
-0.106359	cg0605166	cg0605166	33803347	TTTCTTTCCTCCCTTTCTCTCTT			II	
0.1019863	cg0605209	cg0605209	21676383	TTCTAAAAACTTTAAACACTTA			II	
0.1013522	cg0605237	cg0605237	47624464	CAAAAACCACAATCTAAACTT			II	
-0.108104	cg0606108	cg0606108	71649437	CTCTTCCATCTACTTTAAAAAC			II	
0.1272795	cg0606549	cg0606549	26611336	TTTATCCACCTCAAAAACTAAC			II	
-0.260646	cg0607539	cg0607539	49713492	TCCTAACACTAAAAAAAATCT			II	
-0.108907	cg0607730	cg0607730	66671508	AAAAACA	25699377	AAAAACC	I	A
0.1075909	cg0608572	cg0608572	47672424	ATTTAAATTCTTCCRAAACCCT			II	
0.1358684	cg0609081	cg0609081	58734382	AAACAAA	66670400	AAACAAA	I	A
-0.13253	cg0609386	cg0609386	67783467	ATTAATCAATCTAATCTCCAAC			II	
-0.107102	cg0610676	cg0610676	29736310	RTAAATCCACTTTTATAAACCA			II	
0.1457823	cg0610866	cg0610866	20803430	TCTATCTAACTTATTAACACTC			II	

0.1801357	cg0611217	cg0611217	16684338	TTTTTCCTTTCAAATAATAACC			II	
0.1214873	cg0611220	cg0611220	64656387	CAACAATCRCATTCCTAAATA			II	
0.1077102	cg0611830	cg0611830	40647492	TCTAAAACCCCTAAACCAAAA			II	
-0.203878	cg0612031	cg0612031	47767466	AAAAATAAAAAACCCTCTAAA			II	
0.2532889	cg0612945	cg0612945	58661340	CCACCCCTACAAACTAAAATA			II	
-0.162668	cg0613036	cg0613036	19774377	ACAAAAT	53699496	ACGAAAT	I	T
-0.114371	cg0613272	cg0613272	67672504	AAAATA	63621429	AAAATA	I	A
0.162446	cg0613467	cg0613467	43631399	TAAAACTCTAATATATCATTTC			II	
-0.112949	cg0613552	cg0613552	33738316	AAAAAAC	65637472	AAAAAAC	I	A
0.1130183	cg0613670	cg0613670	48627478	TAACATTCCTCACCTTCTAAAT			II	
0.1732873	cg0613900	cg0613900	24717442	AACCAAC	41733314	AACCAAC	I	T
-0.148707	cg0614331	cg0614331	73685375	CCAACTC	56706412	CCAACTC	I	T
0.1353974	cg0614719	cg0614719	51660422	CTTAATCTTATCCTAAAAACC			II	
-0.132438	cg0614873	cg0614873	73691439	AACAAA	49675416	AACAAA	I	A
0.1274357	cg0615014	cg0615014	12697439	TCCTATC	55755303	TCCTATC	I	A
0.1074965	cg0615077	cg0615077	24683399	ACRCACTAAAATATAAACTAT			II	
-0.105119	cg0615285	cg0615285	10766356	AAAATAACTAACTTTAACCTA			II	
0.1271304	cg0615792	cg0615792	56734322	TATACTC	28736442	TATACTC	I	A
-0.240768	cg0615940	cg0615940	30625391	AAATAAA	28698390	AAATAA	I	A
0.1439676	cg0617331	cg0617331	35726343	AAAAAA	60720359	AAAAAA	I	T
0.1343144	cg0617665	cg0617665	43701306	AAAAAATACCAACAAACCTTA			II	
0.1155971	cg0617922	cg0617922	58688505	CCCAAAAAACCAAAAACCCTA			II	
0.1033288	cg0619073	cg0619073	21644359	ATCTAACAAACCCRTATTACC			II	
-0.208984	cg0619359	cg0619359	54605425	CTACCCA	40778416	CTACCCA	I	C
-0.101831	cg0619395	cg0619395	66632377	AAAAAATTAACCAAAAAAAA			II	
-0.209738	cg0620247	cg0620247	56672358	TAAAACT	50807436	TAAAACT	I	A
0.1295019	cg0620280	cg0620280	59743377	ATACAAATAAATAATAAATAC			II	
0.1296405	cg0620680	cg0620680	20679486	CAACAATTTTAAATACTACTT			II	
0.1050833	cg0621155	cg0621155	55734452	TAACAAA	52660431	TAACGAA	I	C
0.1433441	cg0621390	cg0621390	18623376	AACAACAACTAACTTTTAAAA			II	
0.2061768	cg0622563	cg0622563	29665383	AAAAATCCATAACRAACRCAA			II	
-0.118079	cg0623066	cg0623066	34757465	TAAAATACRATAAACACTATC			II	
-0.115174	cg0623136	cg0623136	54718487	CTATCCTTACACCAATACCAC			II	
-0.125699	cg0623831	cg0623831	64747498	AAAATCCAAAACTACCCTAAA			II	
0.1603068	cg0624120	cg0624120	32773363	TCTATTCTAAAAAAATTTACA			II	
0.1051638	cg0624571	cg0624571	53646379	AATCTAAATATTCACTTAATA			II	
-0.151501	cg0624740	cg0624740	48713397	CCTAACAAAACCTTTACTAAC			II	
0.1115254	cg0624870	cg0624870	39711332	CTTAATAACTAAAACCRCCCA			II	
0.175019	cg0624960	cg0624960	59671348	CTTCTAA	41668405	CTTCTAA	I	A
-0.108948	cg0625075	cg0625075	20661503	ATCCTCTAAAACCAATCACAA			II	
-0.13238	cg0625227	cg0625227	71777325	CATAACT	74715399	CATAACT	I	A
-0.110705	cg0625500	cg0625500	12730365	ATCAATA	42684362	ATCAATA	I	A
-0.329292	cg0626488	cg0626488	13672375	CTAATAAATCAAAACTTCTAT			II	

-0.12184	cg06266227	cg06266227	73642449	CAACATATCAAAAAAATACAA			II	
-0.105861	cg06268875	cg06268875	60793361	TTTTCCA	25655400	TTTTCCG	I	A
0.1251661	cg06271046	cg06271046	52771317	CAAACTACCTTAAATCCAAAA			II	
0.1435675	cg06271387	cg06271387	16652309	CAACCTCTATCTTTCATCAACA			II	
	cg06271696	cg06271696	55809408	ATACAAA	32768487	ATACAAA	I	T
-0.122851	cg06282524	cg06282524	26626360	CTCCATA	44734401	CTCCATA	I	T
0.1180278	cg06285575	cg06285575	52656335	AACCCCTCCTAAAAACAAACT			II	
0.1006202	cg06287548	cg06287548	10792391	CRTATAAACTCCCTTCACATTT			II	
0.1291139	cg06288089	cg06288089	66766464	CAAAATATAAACTTCTAAAAA			II	
-0.254721	cg06293782	cg06293782	28697451	TTAAAATTCATAATAAAACAA			II	
-0.107335	cg06297958	cg06297958	46733479	CACATAA	29713394	CGCGTAA	I	A
0.10494	cg06298480	cg06298480	56632370	AAAACAAATACAAAAACTACT			II	
0.2035785	cg06300880	cg06300880	17736396	TATTTACACAAATAAAACCTA			II	
-0.101009	cg06302025	cg06302025	32682378	ATAACAA	56655426	ATAACAA	I	A
-0.17655	cg06305758	cg06305758	33684488	AATTTCACCATATTAACCAAA			II	
-0.436402	cg06306564	cg06306564	34681342	TAATATCAAACTACTTTTAAA			II	
0.1132001	cg06312469	cg06312469	31807475	TCTAAAATCAAATCTTAAATA			II	
0.1227171	cg06314883	cg06314883	31801434	AAAACCCAAATCCAAAACACT			II	
-0.114169	cg06315390	cg06315390	12656471	CCTTATA	71690449	CCTTATA	I	A
0.2351006	cg06316104	cg06316104	32645422	CCTATTC	59657486	CCTATTC	I	A
-0.108755	cg06322277	cg06322277	28786458	AACAACA	65607459	AACAACC	I	A
-0.148573	cg06330593	cg06330593	45767371	ACATATA	34810417	ACGTATA	I	T
0.1378359	cg06333233	cg06333233	68734430	CTCACCCTAAAAACAATATTA			II	
0.1177881	cg06334238	cg06334238	63754370	AACCATA	37773495	AACCGTA	I	C
0.1586913	cg06342072	cg06342072	39699315	CCATTCTTTTATAAATATTAT			II	
0.1151609	cg06348773	cg06348773	21770427	AAACTAT	34717427	AAACTAT	I	A
0.1196298	cg06349762	cg06349762	43635502	AAAACCA	69640364	AAAACCA	I	A
-0.174466	cg06350542	cg06350542	36770426	CTAAACT	46765377	CTAAACT	I	A
-0.110488	cg06352616	cg06352616	58777301	ACTAAAA	47654342	ACTAAAA	I	T
0.1618819	cg06354695	cg06354695	25729301	CAACACACAAAACCTCTATTA			II	
0.1058921	cg06358191	cg06358191	63797319	RAACCTCCTTAAAACCCACCC			II	
0.1013157	cg06359884	cg06359884	38799348	ATATAATTATAATTAAACATT			II	
-0.182412	cg06360820	cg06360820	31759367	AAACAATAAACRCAAATCCCA			II	
0.1366052	cg06363034	cg06363034	39683376	TAAATATAAAAAAAACCAATA			II	
-0.134556	cg06366791	cg06366791	47605483	CTCATTTTCCATATTCAAATTA			II	
0.15101	cg06374430	cg06374430	53655465	AAAAAACRAAAACTATACACC			II	
0.1123834	cg06380401	cg06380401	16791420	AATAATAAACTCCTAATTCTA			II	
	cg06390368	cg06390368	12768371	TAACCAA	71778401	TAACCGA	I	A
0.1278291	cg06394820	cg06394820	64600507	CTAAACAAAAAAATATCCCRA			II	
	cg06397523	cg06397523	13644325	ACAACTC	70688368	ACAACTC	I	A
-0.118995	cg06398242	cg06398242	62623438	ACTAAAA	62629443	ACTAAAA	I	A
-0.181966	cg06407043	cg06407043	50810438	TATATATAAAATCATCTAATA			II	
-0.103309	cg06416685	cg06416685	29808391	TTACCCTCTACAAAATTATAC			II	

0.1085643	cg06422039	cg06422039	49645414	AAAACAC	28720374	AAAACGC	I	A
0.1800885	cg06422757	cg06422757	16691453	AATTTAATCTTAAAAACCTAA			II	
0.1144699	cg06429194	cg06429194	10616505	TAACATATACACTTTCTAAACA			II	
-0.127977	cg06430688	cg06430688	65688320	CTAATCATCTATCACCACAAAT			II	
0.1230891	cg06431681	cg06431681	14667388	AAAAAAACTCCAACCTAAAAA			II	
0.1252624	cg06433801	cg06433801	15792435	ATTCCTTTTCCTCAATAAAAAA			II	
-0.116926	cg06437209	cg06437209	60686369	CCAAACCTATAAAAAACCTCC			II	
-0.148999	cg06437931	cg06437931	70650364	CTCTATAAATCCAAATTTCCCC			II	
0.1141392	cg06438559	cg06438559	27606375	AAACAATAAAAAACTAACATA			II	
0.121103	cg06450373	cg06450373	51739444	TTCTTACTCTTCTCTCAATAAC			II	
0.2013787	cg06457736	cg06457736	67621496	RAATTAAACAATAACTTATCA			II	
-0.106602	cg06459104	cg06459104	35778480	CTTCTCACTAAACTAAACCCA			II	
0.1238686	cg06460189	cg06460189	51774338	CRACCAATTTAACTACAAATA			II	
-0.124191	cg06460475	cg06460475	36624324	CACACCC	55735341	CGCGCCC	I	C
0.3717501	cg06461588	cg06461588	71687503	TAAATAAAAATACAAAACCCA			II	
0.416702	cg06464078	cg06464078	49764417	CTAAAAACACACTCAAATTCC			II	
-0.100135	cg06468385	cg06468385	70632375	AAAAAAATCRCAAAACCTACA			II	
0.1016733	cg06469440	cg06469440	73698361	TCTTTATTTATTACAATCTCTT			II	
-0.187157	cg06470855	cg06470855	57681456	CCAAAAT	66764464	CCAAAAT	I	C
-0.12117	cg06472423	cg06472423	26623439	TTACCTTCTAATCACCCTTAAT			II	
0.1200341	cg06476693	cg06476693	48787484	AATTCACTTCTTTACCCTACTC			II	
-0.102662	cg06478504	cg06478504	67604416	AACAAAC	42625372	AACGAAC	I	T
0.1470259	cg06484123	cg06484123	69796300	CCATTTCCCTATTTACTACTCC			II	
0.2428651	cg06484169	cg06484169	56663455	AAAATTTCTCCATATTAATCAA			II	
-0.215037	cg06491590	cg06491590	35796438	CTACAAAACTCAAAAACACTA			II	
0.1028378	cg06492372	cg06492372	65606397	AAAATAACCCCATAACTTCTA			II	
0.1299965	cg06493806	cg06493806	35687442	AAAAAAACTATACCRAATACT			II	
-0.188688	cg06503255	cg06503255	39702400	AAATAAA	23802427	AAATAAA	I	A
0.2144342	cg06503981	cg06503981	38736460	CCTACTCCTCAATTCTTACATT			II	
-0.137413	cg06507634	cg06507634	70618405	ACTTCACACCTTTATTATATCC			II	
-0.117627	cg06511189	cg06511189	53644305	CCAAAAA	31626381	CCAAAAA	I	C
0.1202405	cg06511330	cg06511330	14741422	CACTCCCRACACCRCATTTCTA			II	
	cg06513864	cg06513864	73790437	CACCACA	56733367	CGCCGCA	I	A
-0.110009	cg06521472	cg06521472	29670416	ACACCAA	38737311	ACACCGA	I	A
0.1976867	cg06523151	cg06523151	20745504	AATCTTTAATTTTCCTACACAT			II	
-0.127275	cg06524264	cg06524264	16628418	CTAAATAATCTCRATCAACTCC			II	
0.138929	cg06525453	cg06525453	49601316	TCTACTTAACCAATTATAAAAC			II	
-0.150714	cg06531573	cg06531573	15760498	CRAATCCCTTTACCATCCCCCT			II	
-0.350937	cg06536159	cg06536159	72678327	ATAAACRCCACCTAATAACCA			II	
-0.104256	cg06545200	cg06545200	29750446	CTCAAAA	66809442	CTCAAAA	I	C
-0.102001	cg06549238	cg06549238	10808332	CCCTAAA	37751507	CCCTAAA	I	A
0.198039	cg06549874	cg06549874	32756434	ATTCACCCCTAAAAAATCCTAC			II	
-0.100013	cg06549928	cg06549928	51784479	CTCCTAT	16726332	CTCCTAT	I	A

-0.279944	cg06550274	cg06550274	44615360	AACRAAAACCCAACACTAACC			II	
0.1270134	cg06559575	cg06559575	11755457	TCCCTCTTCCTCTCTCTTATAA			II	
-0.147692	cg06562718	cg06562718	51790496	AAAAACACCCRATATAAATCC			II	
-0.105609	cg06566034	cg06566034	36644315	ACAACAA	30733327	ACGACAA	I	A
0.1080141	cg06568346	cg06568346	44629445	AAACTTAAAATAAAACRTAAA			II	
-0.111328	cg06570432	cg06570432	32654390	RATACTAAATTCCTTTCTATAT			II	
-0.112128	cg06573096	cg06573096	26705468	TTTAATCATATCTTATTCTCAA			II	
0.1137182	cg06578276	cg06578276	16637439	TTAAAACCCACAAAATACTCC			II	
-0.162789	cg06583549	cg06583549	34632461	ATTCCAA	67678312	ATTCCGA	I	A
0.1381559	cg06584218	cg06584218	10757344	ACRTTCCCAAAACAAATATAT			II	
0.1548431	cg06586265	cg06586265	45613487	AAATAACAATAAACACAAAAC			II	
0.1628414	cg06587249	cg06587249	21769327	TACTTCA	21685348	TACTTCG	I	A
0.1264344	cg06587955	cg06587955	36701322	RATCTATTTTTCCTTTAATTCC			II	
-0.129826	cg06588556	cg06588556	53606434	AAATAAA	63683508	GAATAAA	I	A
0.1006772	cg06589191	cg06589191	27753383	ACTATAA	13726352	ACTATAA	I	C
0.1001306	cg06589199	cg06589199	35615321	TCCAAACAAACCCRATAACTT			II	
-0.163178	cg06593753	cg06593753	36733361	CCCTAAACTAACAAACTTTAC			II	
0.1240645	cg06595154	cg06595154	23641489	CTACTAAATATTTTCTAAATAA			II	
0.126712	cg06596943	cg06596943	71618389	CAACRTATTAAAAACACRCAA			II	
-0.239153	cg06617490	cg06617490	32784332	CAAAAAACTAAATACCAACAA			II	
-0.106111	cg06618134	cg06618134	49798311	TAAATCACTTTCCTCAAATCAC			II	
-0.120699	cg06619621	cg06619621	23669487	ACTATAC	68801420	ACTATAC	I	T
-0.10436	cg06619938	cg06619938	66703337	AACCTCA	55610394	AACCTCG	I	C
0.1022609	cg06620117	cg06620117	10731450	CAAAACTAAACTATACCACAA			II	
0.1173673	cg06621027	cg06621027	43726361	CCACAAAAACTACCAATCCTC			II	
-0.105918	cg06624527	cg06624527	25798433	AAAACAA	10630451	AAAACAA	I	A
-0.141854	cg06628792	cg06628792	19665466	AAACTTCACCCAATACTTCTAT			II	
-0.104887	cg06630839	cg06630839	52715317	AACAAAC	32741367	AACGAAC	I	A
0.2083445	cg06631775	cg06631775	24644310	AATATTT	56680385	AATATTT	I	A
0.5070584	cg06634367	cg06634367	16610505	AATTCTA	11714504	AATTCTA	I	C
-0.32193	cg06634576	cg06634576	33763414	TCTTTTTA	35721391	TCTTTTTA	I	T
0.1250085	cg06634717	cg06634717	14804417	ACAAAAC	35630510	ACGAAAC	I	T
0.1289884	cg06635764	cg06635764	50764414	TATCCTT	53675436	TATCCTT	I	C
-0.110451	cg06645921	cg06645921	61626420	ACAAAAC	38633476	ACGAAAC	I	C
0.1521166	cg06647930	cg06647930	43653440	ATTTACTATTCTATCTTCCACA			II	
-0.103284	cg06649034	cg06649034	54780312	AAAATCA	22711458	AAAATCC	I	C
0.1756823	cg06650246	cg06650246	51790402	TTCTCTTA	33744484	TTCTCTTA	I	C
0.1646034	cg06650786	cg06650786	41798378	AAACTTACATTTATACRTCCRT			II	
-0.111662	cg06657917	cg06657917	49756328	AAAAACA	30766450	AAAAACA	I	A
-0.109196	cg06665333	cg06665333	11641398	ACAAATA	43666501	ACGAATA	I	A
0.1306144	cg06665622	cg06665622	27804429	AACATAAACACTCRACTACAA			II	
0.1421248	cg06674117	cg06674117	25647452	AATTCAAAACTTCCTACTACC			II	
0.164876	cg06675417	cg06675417	36780493	TATATAAAATAAAATAAACRC			II	

-0.227959	cg06686422	cg06686422	43760371	TTAAAAAACACTAATTCATTCA			II	
-0.177722	cg06688803	cg06688803	54745316	TCTATATTTAAACCTCAATTTC			II	
0.1021149	cg06689619	cg06689619	67729462	CTAAAACTCTTATTATACTTCT			II	
0.1523764	cg06690085	cg06690085	36632421	AAAACCCCTCCCTCTAAAATCA			II	
0.140682	cg06691616	cg06691616	52766478	TCCATAAAACTACATCTTTTTT			II	
0.1292993	cg06698332	cg06698332	67659489	AAATTTAAAAAAAACCTAAAT			II	
-0.199503	cg06699489	cg06699489	29618468	TAAAAACCACAAAAACAAAAC			II	
-0.201197	cg06699669	cg06699669	53673361	ATTCCTTA	48603472	GTTCCTTA	I	T
-0.258656	cg06706443	cg06706443	74749383	CTCTACTCCTAAAAACCATTAA			II	
-0.137125	cg06707236	cg06707236	59657317	ACCCAAA	55660419	ACCCAAA	I	C
0.1346024	cg06709297	cg06709297	23667375	ATCRATTCTCTCAATCAATCAA			II	
0.1207606	cg06711418	cg06711418	59719305	TAAAAAACRCRACTTCCACAA			II	
0.161664	cg06713116	cg06713116	15697445	CAAAACCCCAACTACTAATAA			II	
0.2271218	cg06718159	cg06718159	36768332	ACCAAACTATTAAAAAACCAA			II	
0.1168816	cg06718920	cg06718920	19726359	CTATAAAAAACCCCTAACTAA			II	
-0.177115	cg06721546	cg06721546	71751418	CAAAAAACCACCCACRAAACT			II	
0.110676	cg06726231	cg06726231	11759300	CTAAAAATCCCCACACRCAAA			II	
0.1071161	cg06727338	cg06727338	52647447	CRACTATATAATCTCCTAACTA			II	
-0.130221	cg06728252	cg06728252	17687483	TACATAA	53744414	TACATAA	I	T
0.1876193	cg06736229	cg06736229	35758367	CAAAAACTTCACTACAAACTC			II	
-0.136776	cg06737308	cg06737308	27715457	TTATCTTCTCAAAAACATACA			II	
0.1520143	cg06738063	cg06738063	27652434	CTTTATCCTATATATATAACCR			II	
0.1007078	cg06741336	cg06741336	37611325	AAAAAAC	50676357	AAAAAAC	I	A
0.2452028	cg06743454	cg06743454	20769409	CCCCACCAACAAAATACAAAA			II	
-0.149573	cg06747330	cg06747330	50797362	CAAAACCCTTCTAAATCCTCRA			II	
0.1106536	cg06755258	cg06755258	12697311	AACRACTACATCTTCCTATCAA			II	
0.1654817	cg06758191	cg06758191	30630472	TAAAAAAAAATCCTTAACTCTC			II	
0.1177325	cg06760904	cg06760904	36602415	ATATTAA	16749429	ATATTAA	I	C
0.2788438	cg06761912	cg06761912	63615329	TATCTATATTTCTTAACCTAAA			II	
0.1409986	cg06763776	cg06763776	49682499	TATTTCTATCTTATAAAAATAC			II	
-0.103828	cg06763829	cg06763829	31694485	AAAAACA	27758434	AAAAACC	I	T
0.157831	cg06769820	cg06769820	11806416	TTACAACCAAAAAAAAACTAC			II	
0.1170107	cg06778662	cg06778662	26715408	CCTACTC	26609404	CCTACTC	I	C
0.1805662	cg06781948	cg06781948	26739477	TATTTCTCATTTATAAACTCCA			II	
-0.150059	cg06789824	cg06789824	73669314	ACTTACAACCAAAAATATAAA			II	
-0.143856	cg06792262	cg06792262	54757347	AAAAAAA	18617354	AAAAAAA	I	A
-0.400761	cg06792428	cg06792428	17787407	CAAAAACTATAAATACCCRAT			II	
0.1215725	cg06794141	cg06794141	30800394	TATACATAAAAATCACTCTAAA			II	
-0.151033	cg06796435	cg06796435	60704500	AAACACC	63750357	AAACACC	I	C
0.1016354	cg06796713	cg06796713	55650417	RTAAAAAAAATACCTACTCCA			II	
0.1464808	cg06798115	cg06798115	30656349	CTCAAAAAAATACTTTCCAAAC			II	
0.1433868	cg06798887	cg06798887	43788459	CTATAAAAACCTTCCCACCAA			II	
0.1472094	cg06799747	cg06799747	61602392	CCTACTACTCTAAATATTATAA			II	

0.1296765	cg0680022	cg0680022	69728463	CCRAATCCCAACCATATCCAA			II	
0.1262676	cg0680441	cg0680441	43684436	CTAAAATCATTAAAACCAATC			II	
0.1105759	cg0680686	cg0680686	35635464	ACAAAAACTTACTAATTCTCT			II	
-0.112597	cg0680928	cg0680928	44783365	TCATATCCCAACTCAACTAAT			II	
-0.104869	cg0681206	cg0681206	47611437	CCCCATC	50686435	CCCCGTC	I	C
0.2052432	cg0681279	cg0681279	49703384	TAACCAAAAAACCRATATTTC			II	
0.113474	cg0681283	cg0681283	70635328	CCRATATTTACTAACCRTATA			II	
0.1134881	cg0681655	cg0681655	29782437	AAACACAATAAAACCAAATCC			II	
0.1217424	cg0681815	cg0681815	51757488	TCATAATATATATCTCACTACC			II	
0.1196045	cg0681886	cg0681886	24810505	CCTTTCAATTCCTTAAATATAT			II	
0.1088299	cg0682107	cg0682107	72731423	ACAACCTAAATAACTTTTAAC			II	
-0.127364	cg0682182	cg0682182	17685494	AAAATCTAAATTACRTACTCC			II	
0.1197544	cg0682219	cg0682219	59677439	TTTAAAATTCCTACTTTACAAA			II	
-0.126982	cg0682303	cg0682303	74683424	TAAAAA	66622482	TAAAAA	I	T
-0.141029	cg0682612	cg0682612	41603422	AACACAT	42687466	AACACGT	I	T
0.1058714	cg0682814	cg0682814	52714396	CTAATTCTTCTAATAAAAAAA			II	
-0.104383	cg0683106	cg0683106	47715326	AACAAAC	23803388	AACGAAC	I	A
-0.270011	cg0683398	cg0683398	30630339	AATACCAAACATATACCCTTC			II	
	cg0683581	cg0683581	54768320	AACRTATAAAAAATAAACCTA			II	
0.1825294	cg0683821	cg0683821	18744339	TAACCTCTACTTACACCTTAAC			II	
0.1013014	cg0683911	cg0683911	24758505	ATCCAAACAATAAATATACAA			II	
-0.109325	cg0684471	cg0684471	72651489	TCATTTATTCTCACAATAAAA			II	
-0.268528	cg0684484	cg0684484	19796492	CCATCCTAAAAATAACAAATA			II	
0.1145258	cg0684848	cg0684848	21716407	ATTCCCTAAATTCTCTCCTCCA			II	
0.1118641	cg0685808	cg0685808	53796358	TAATTAAAAACCTAAAAACTA			II	
-0.173256	cg0686564	cg0686564	73713481	TAAACATTCAATAAATACRCR			II	
0.1023777	cg0686748	cg0686748	27650399	CCAAACA	24604317	CCAAACA	I	T
-0.117585	cg0687011	cg0687011	38774451	TCCCAAAATCCAAATTCCCTA			II	
0.2003369	cg0687134	cg0687134	41643338	CAAAAAACTCTAAAAACACRA			II	
-0.108917	cg0687607	cg0687607	28674327	CTAATAACACCTATAACCTAA			II	
-0.104595	cg0688449	cg0688449	46682499	CAAACTTCCCCTAAAATCCTA			II	
0.1226335	cg0688673	cg0688673	61790357	ACACACA	42731362	ACGCACC	I	T
-0.134634	cg0688846	cg0688846	43681321	ATACAAA	32771453	GTACGAA	I	C
-0.151263	cg0689576	cg0689576	37722354	AACTTTAAAAAACCAACAAAC			II	
-0.11733	cg0689744	cg0689744	31666360	TACAAATATCAAAAAACAACC			II	
0.1100523	cg0690057	cg0690057	11749329	RTACCATCTCCCCRAAATCTA			II	
	cg0690576	cg0690576	30674312	AAATAAA	29675455	AAATAAA	I	C
0.1084983	cg0690925	cg0690925	19690510	TACTCTATTACTAAACCAAAC			II	
-0.127726	cg0691351	cg0691351	62658445	AAACATC	69802376	AAACGTC	I	A
-0.310951	cg0692490	cg0692490	13759383	TTTTTCATCATCCCATAAAAA			II	
0.1098329	cg0692530	cg0692530	19801472	AACCAAAAAACCTTTCCAAAA			II	
0.1086651	cg0693253	cg0693253	31652477	CATAATAAAAACTTCCAAACC			II	
0.1517849	cg0693543	cg0693543	28700404	CCTAAAACTCTCTCTATTTCTT			II	

0.1780057	cg0693629(	cg0693629(	23804420	TCTCTAAACTTAACAAAAAAC.			II	
0.1364722	cg0693792	cg0693792	36675346	AAAATAACTAAACAAATCTAT			II	
-0.149562	cg0693826	cg0693826	35664356	CCAAAAA	40687337	CCGAAAA	I	C
0.1222973	cg0694115	cg0694115	46659489	ACAAAATAAAACCAACRTAAA			II	
0.1733964	cg0694211	cg0694211	15602396	AAATCCAACCTTACTTATCTTA			II	
-0.112257	cg0694898	cg0694898	20774355	CACCACC	58714333	CACCACC	I	C
-0.124826	cg0695112	cg0695112	14612478	AAACCTC	44784383	AAACCTC	I	A
-0.292093	cg0695595	cg0695595	16625306	ACCTACCTTATATCRCAATATA			II	
0.1638317	cg0695876	cg0695876	24600374	AACTATA	60790506	AACTATA	I	A
0.1219632	cg0695944	cg0695944	23708426	AAATTCTACTCTCCAAATCTAA			II	
-0.189759	cg0696071	cg0696071	65763472	TTATCAAACCCATTCACACATA			II	
0.1177855	cg0696142	cg0696142	69617424	ATATTATTTTAACTAATATCAT			II	
-0.112319	cg0696871	cg0696871	14737440	ATCRCCTCTAAAAAACAAAAT			II	
0.1974752	cg0697122	cg0697122	39602432	ATATTAAAAAAACCCRAAACA			II	
-0.100222	cg0697597	cg0697597	22680370	AAAAACC	58647446	AAAAACC	I	A
-0.102248	cg0697622	cg0697622	32734337	ATACAAAACAAAAATATCCAT			II	
0.116546	cg0697902(	cg0697902(	11702434	AATTACCTCCATCTAAAAAAA			II	
-0.139594	cg0697938	cg0697938	27693451	ATCTTCTCAACCAAAAACTATA			II	
-0.114405	cg0698216	cg0698216	50801467	ACCACCCRAATAATACAAAAT			II	
0.1082518	cg0698282	cg0698282	50657301	TTTATTTATCTCTCCCCTAATC			II	
-0.10219	cg0698420	cg0698420	15792440	TAAACACACTATCATCTCRCA			II	
0.2183425	cg0699550	cg0699550	72669423	TCTACTCATATACACATCTAAA			II	
0.1008264	cg0700712	cg0700712	48715323	CTACATTTTCATTTCTAACCTA			II	
0.1475047	cg0701153	cg0701153	24806349	TACCTCTTAACAAAAACTAAC			II	
-0.175339	cg0701574	cg0701574	14780443	CAATATA	28697376	CAATATA	I	A
-0.104674	cg0701843	cg0701843	69725465	TAATTTCCAAAAAAAACAACA			II	
0.1713272	cg0702153	cg0702153	23767478	TAAAATTACAAACCTAACCAA			II	
0.1510144	cg0702349	cg0702349	16735430	ATCAAATATTTATACCTCAATC			II	
0.1044733	cg0702598	cg0702598	61761330	TCCTCAA	60706463	TCCTCGA	I	C
0.1229748	cg0702895(	cg0702895(	16757359	AAAAACAAAAAACCRAACRTA			II	
0.1026001	cg0702977	cg0702977	36775473	TAATTCCTAACRTCAATCRAAA			II	
-0.126654	cg0703140	cg0703140	19606305	AAAAACA	30614363	AAAAACC	I	A
0.1037028	cg0703329	cg0703329	55606483	TCTATCTAAAATACTTCCRATA			II	
-0.104719	cg0703596	cg0703596	32670378	AACATTT	50677405	AACATTT	I	A
-0.101245	cg0703796	cg0703796	34643500	AAAACTAATCTTTAACAATTTC			II	
-0.101434	cg0704148	cg0704148	57693376	ATTTCTATAAACTATTTCTAAT			II	
0.1059828	cg0704821(	cg0704821(	11754467	CTACAAAAAACCTCTATTTAA			II	
-0.138936	cg0705116	cg0705116	37752468	CAAAAAA	24601384	CAAAAAA	I	A
0.1037191	cg0705311	cg0705311	69632377	CCCAACA	18728350	CCCAACA	I	T
0.1505633	cg0705468	cg0705468	27678345	CAATAAA	12778447	CGATAAA	I	T
-0.166425	cg0706178	cg0706178	17644464	AACAACT	65610316	AACAACT	I	C
0.1038321	cg0706355	cg0706355	13760348	ACATTCA	30774479	ACGTTCG	I	C
-0.147835	cg0706449	cg0706449	58683483	TACCCTA	13674339	TACCCTA	I	T

-0.1137	cg07068181	cg07068181	28608460	TTAAAAAAATTTCCTCRTCTTA			II	
-0.109109	cg07075347	cg07075347	59760319	ACAAACC	26780402	ACGAACC	I	A
-0.122229	cg07075387	cg07075387	49604440	ACTATCA	42704427	ACTATCG	I	A
0.1868958	cg07078958	cg07078958	64671370	ACRCCACTCAAAAAATATAAA			II	
-0.110652	cg07082452	cg07082452	47687428	TAACATC	35795412	TAACGTC	I	C
0.12168	cg07083818	cg07083818	16708468	CRAACCAATCAAAATTCTCACT			II	
0.1321563	cg07092880	cg07092880	17741334	CTTTTAAAAAAAACTTTTAATA			II	
-0.1182	cg07093060	cg07093060	24677315	ACTTCACTAAAACATATAAACC			II	
0.1055903	cg07101208	cg07101208	16671320	RAAACTCATAACTCRTAACAA			II	
0.1036602	cg07101841	cg07101841	23606375	AAAAAAA	67630445	AAAAAAA	I	C
0.1804444	cg07103846	cg07103846	31649399	ACAATTC	60701397	ACAATTC	I	A
-0.112274	cg07105947	cg07105947	28654322	AATCCAA	29639424	AATCCGA	I	C
-0.105265	cg07106927	cg07106927	30800449	TACTACCTAAATTCTAACACAA			II	
-0.105656	cg07113199	cg07113199	56696398	CAAACAA	18634480	CAAACAA	I	A
-0.142433	cg07114310	cg07114310	16646394	AAACRTTTCTTCCTTTTCTTCT			II	
0.1121279	cg07114810	cg07114810	63676506	AAAATAACCRTAAATAAAACT			II	
-0.102635	cg07116631	cg07116631	69808469	AAACAAT	36794428	GAACGAT	I	C
0.1221692	cg07116712	cg07116712	20799343	CAACCCT	72652348	CGACCCT	I	T
0.1430759	cg07119157	cg07119157	37708397	AAACRTAAACRTAAATATAAT			II	
0.2438986	cg07124183	cg07124183	18742431	TCTCCATAAATAACAACCAATA			II	
0.130363	cg07128503	cg07128503	47802391	AAACCAAAAAACTTCCCTCCTA			II	
0.1252008	cg07134368	cg07134368	31604480	ACRAAAACATATATACACCCA			II	
0.1051308	cg07136254	cg07136254	18664440	CCTAAACAACTCACAACTCAA			II	
0.1715668	cg07136920	cg07136920	11658305	CAAAACC	10709499	CAAAACC	I	A
-0.135865	cg07137336	cg07137336	25632370	TAAAATAAACCCTTCCCCAAAC			II	
0.1157826	cg07139527	cg07139527	17733357	TATAATAACATCCCTAAACTTC			II	
0.137822	cg07143158	cg07143158	32685420	AAAATCA	35752322	AAAATCA	I	A
-0.10857	cg07161993	cg07161993	15623461	AATATACCAAAATTAAATTAAC			II	
0.1103269	cg07167933	cg07167933	69741325	TAAAAAAAACCTACTAATCCTC			II	
-0.111239	cg07173547	cg07173547	13618386	AAAAACTAAAAATAACCRAAT			II	
0.1365528	cg07179033	cg07179033	38633370	CTTTTAAACTATTATATAATCA			II	
0.1197351	cg07181702	cg07181702	61745374	ATAACTTATCAAACTAATATTA			II	
-0.227208	cg07182616	cg07182616	60691506	TCCTTTATCTTCTTTTCAAATTC			II	
0.1686386	cg07187202	cg07187202	60725377	TCAAACAATAATATATCCAAA			II	
-0.107223	cg07188591	cg07188591	73779374	TCAAAAC	32785438	TCAAAAC	I	C
-0.105009	cg07200874	cg07200874	62797430	ACCCAAA	40779370	ACCCGAA	I	C
0.177291	cg07200939	cg07200939	66618309	ATTCTTCAAACRACTTAAAAAC			II	
0.1514328	cg07201475	cg07201475	51673397	CAATTTACTAACAACATACCTA			II	
0.1213442	cg07204662	cg07204662	35802327	ATCTCCA	63636368	GTCTCCG	I	A
0.1004141	cg07205796	cg07205796	40763390	ACATAAC	29665340	ACATAAC	I	A
0.1268863	cg07213181	cg07213181	31748390	ACACAACTATCCAAAACTAAA			II	
0.1222849	cg07213780	cg07213780	13608319	AAAAAACTCCCAAACCTACTTC			II	
-0.204333	cg07215504	cg07215504	63601400	TATTCTTC	69745364	TATTCTTC	I	C

-0.11934	cg0721569	cg0721569	25803509	TTAACAC	69798377	TTAACGC	I	A
0.1103117	cg0721652	cg0721652	44631403	ACAATTATTCTAAAAACTTAA			II	
-0.452999	cg0721661	cg0721661	16609425	TATAAATTTACTTCTAAAATCR			II	
0.1014203	cg0722065	cg0722065	24778345	TCAAACTAAAATACAAATTTC			II	
0.109435	cg0722125	cg0722125	20797361	CTCCTTAACAACAAAATATTA			II	
-0.170922	cg0722320	cg0722320	65740494	AAATTCA	49653476	AAATTCA	I	C
0.1308138	cg0722718	cg0722718	68679473	AACCCTC	13794443	AACCCTC	I	C
0.1025972	cg0722824	cg0722824	56749353	AATTACA	60751355	AATTACG	I	A
0.1101831	cg0722976	cg0722976	26611405	TAACTCATCCTAAATAAAAAC			II	
0.1110163	cg0723035	cg0723035	72645482	AAAAAAC	46695468	AAAAAAC	I	A
0.1782406	cg0723200	cg0723200	46717453	ACTCAAAAAACCTTCAAACTA			II	
0.1459285	cg0724084	cg0724084	35619467	CAACAACTATTACTAATTCAA			II	
0.1038873	cg0724108	cg0724108	48806509	CAAAAAA	37709383	CGAAAAA	I	A
0.2426672	cg0724948	cg0724948	24611488	TAAACCCTACAAAAATTTCCA			II	
0.1884856	cg0725442	cg0725442	19650479	RTATACTTCCTCAATAAAAATA			II	
0.1191223	cg0725782	cg0725782	28751331	ACCACAA	39657373	ACCGCAA	I	C
-0.510695	cg0725840	cg0725840	41739423	ACCAACT	37800497	ACCGACT	I	T
-0.293593	cg0725898	cg0725898	61749335	AATTAAAATCAAACAATATAA			II	
-0.110518	cg0726059	cg0726059	60648498	CACCTACCTAAAAATCAATCC			II	
0.1027006	cg0726580	cg0726580	13716447	ATATCTAAAATAAAATATTTC			II	
0.1202848	cg0726760	cg0726760	14746373	AAAAACTAAAATCRTACAAAC			II	
0.1351286	cg0726824	cg0726824	30758428	CACATATTTTATATTAATTCAC			II	
-0.209355	cg0726872	cg0726872	12744392	RAAAACRAACTACRAATCTCC			II	
0.1417065	cg0727322	cg0727322	35708382	ACTCCCAAAACTTATATTTCA			II	
0.1735193	cg0728426	cg0728426	13671359	CCATATACTTTAACTTTAACTA			II	
0.1909759	cg0728523	cg0728523	64612381	TACAACTATATTCAACTAAAA			II	
0.1238365	cg0728567	cg0728567	27652378	AACCTATCTAAAAACATACCT			II	
-0.105189	cg0729411	cg0729411	58629344	ACTAAAAACAAAAACAAATTA			II	
0.1285006	cg0729478	cg0729478	68716445	CATAAAT	27622334	CATAAAT	I	T
-0.111318	cg0729524	cg0729524	57635507	AAACCCTAAAAACTTAAAAAA			II	
	cg0730150	cg0730150	71714478	CTAAAAT	67810399	CTAAAAT	I	A
0.100335	cg0731050	cg0731050	58778310	AAAAAAAAACCTAACTCAAAA			II	
-0.118184	cg0731102	cg0731102	59645339	AAAAAACRTAAACTTTATCAA			II	
0.1286579	cg0731673	cg0731673	53683400	CTAATCTATAACTAACCACCT			II	
-0.108257	cg0732214	cg0732214	48755323	AATCTAAAAACCCRACCTTAA			II	
-0.107645	cg0733712	cg0733712	41648304	AACAACTATAAAAAACACCAA			II	
	cg0735116	cg0735116	15625403	TTCTAAA	13644383	TTCTAAA	I	C
0.1012793	cg0735568	cg0735568	39768474	CRAAAAAATCACCCTCRTTTA			II	
-0.121989	cg0735592	cg0735592	25621344	TATCACT	61624473	TATCACT	I	A
0.153902	cg0735771	cg0735771	33649493	TACTACTACCAAAAACCACTA			II	
-0.140084	cg0736512	cg0736512	45618394	AAATCTAAACAAAACRTAAAA			II	
-0.171302	cg0736574	cg0736574	61780399	AAAACCACCRACTAATACTAC			II	
0.1359021	cg0737141	cg0737141	51725339	CTCCAAAACAATAATAAAAAT			II	

0.1065148	cg07371747	cg07371747	40673411	RACRAATTTATAAACACCCAA			II	
-0.10377	cg07376381	cg07376381	10710408	TCACTAC	19680443	TCACTAC	I	T
0.1552933	cg07380200	cg07380200	11717415	AAAACTACAAACATCCATTCA			II	
-0.142374	cg07381502	cg07381502	33659300	AACATAA	30603504	GACGTAA	I	A
0.2811573	cg07382347	cg07382347	18809480	AAACCAT	52801344	AAACCGT	I	A
0.1564745	cg07384522	cg07384522	35693419	TCTAACATAAAAAACCCRAAT			II	
0.1257988	cg07402939	cg07402939	29764440	TCTCTCCTTCTAATAATAAACC			II	
-0.105415	cg07404485	cg07404485	14653354	CTACTTATAAATATTCTATTAA			II	
0.1458015	cg07407736	cg07407736	64794458	ACRAAAATTCCAACTACTAAA			II	
0.1309362	cg07408232	cg07408232	10764503	ATTCTAAACATCTATCTATCCC			II	
0.1652496	cg07413957	cg07413957	24651408	CAAATTAAAACTTAACTATCA			II	
0.1810151	cg07414422	cg07414422	17763405	ACAACCAAATCATATCAAATA			II	
	cg07419704	cg07419704	13644507	AAATCAA	12806455	AAATCGA	I	A
0.1066759	cg07419795	cg07419795	53683423	AAAAATCTACAAAACAAACTC			II	
0.1172225	cg07421329	cg07421329	14643465	AAAAAATACAAAATTTATCTC			II	
0.1768061	cg07437923	cg07437923	41664365	ATAAATAATAATCCTTATCAA			II	
0.1115763	cg07438246	cg07438246	58738403	AACCCCATAAAACTTTAATCA			II	
0.1089521	cg07441278	cg07441278	32783348	TCCAAACACCACACTAAAACC			II	
0.1254566	cg07451803	cg07451803	29693347	TCTCCATTTAAAAACAACTCA			II	
-0.339884	cg07453440	cg07453440	22631335	TAAATTTTAAACCAAACTTCCT			II	
0.1118733	cg07453857	cg07453857	63781398	AACCACTATAACAAAATAAAT			II	
-0.239945	cg07455790	cg07455790	47702338	AACAAAA	44613324	AACGAAA	I	A
0.2402348	cg07456585	cg07456585	68672490	ATACAAAAAATATCTAAAAAA			II	
-0.169957	cg07460842	cg07460842	25602402	AAAAACTAAACCTCTATAAAA			II	
0.1054407	cg07463445	cg07463445	18688340	CAATAATAAAAATAATATCAC			II	
-0.154871	cg07466166	cg07466166	66752401	TCTCCAC	36759444	TCTCCGC	I	C
0.1026952	cg07470968	cg07470968	52763427	TCACTATAAAACTTTTACACAT			II	
-0.126246	cg07474670	cg07474670	68614381	CRATAATAAACAACTAAATAA			II	
-0.100529	cg07475394	cg07475394	18794413	TACCACA	28623314	TACCGCA	I	T
0.1685212	cg07476236	cg07476236	16794477	ACTCACTTAACAAATTCCCTAT			II	
-0.108293	cg07479618	cg07479618	71612425	AACTTCT	25622453	AACTTCT	I	A
0.1054952	cg07488141	cg07488141	69607345	CCTACCTCTAAATTCTACTCCT			II	
-0.161613	cg07490070	cg07490070	35716390	CCATAAA	47692406	CCATAAA	I	A
-0.154288	cg07492045	cg07492045	65743337	ACAAATA	57795458	GCAAATA	I	A
0.1075839	cg07493044	cg07493044	39621398	ATATCTC	32729495	ATATCTC	I	A
0.174662	cg07493197	cg07493197	52717438	AATCTCATACTTTCTATATCTC			II	
0.2054449	cg07495405	cg07495405	53603450	TATCCAACAAACAAAAATCAA			II	
0.1617897	cg07498088	cg07498088	32632486	ACACCTAACRAAAAAACRATT			II	
0.1327495	cg07499182	cg07499182	19642415	ACTCTAAACCATTTTTAAACTA			II	
-0.162041	cg07501029	cg07501029	48646448	TTTAATCTATCTTCCTAAATAA			II	
-0.114589	cg07506153	cg07506153	30804471	CCTCRATAACAAATTTTAAAC			II	
-0.191901	cg07509935	cg07509935	35669497	TAAACAA	40808423	TAAACAA	I	T
0.1044493	cg07517739	cg07517739	41655378	AAATAAAATACCTCRTTCAAA			II	

0.1145344	cg0751942	cg0751942	40786373	ATCAAAT	54737359	GTCGAAT	I	T
-0.123273	cg0751990	cg0751990	68617489	AAAATA	40707471	AAAATA	I	A
0.1299052	cg0752297	cg0752297	19647471	CTTATACACACTACRTACAAA			II	
-0.304825	cg0752371	cg0752371	15798465	TTTATTAAAATTTTCCTTAACC			II	
0.1070175	cg0752547	cg0752547	59768415	ACCCCCA	45696375	ACCCCCA	I	A
0.1111786	cg0752912	cg0752912	51782346	CAAAAA	55734325	CAAAAA	I	A
-0.127281	cg0753208	cg0753208	66676374	TCCATTC	52766421	TCCATTC	I	C
-0.136134	cg0753484	cg0753484	40738358	AACTAAA	28717345	AACTAAA	I	A
-0.16782	cg0753756	cg0753756	34743307	RACTTAAAAATTCTATAAAAC			II	
0.118324	cg0753782	cg0753782	60767362	ATACTAAAAAACAAACTTACT			II	
0.1328436	cg0756361	cg0756361	57705400	AAAACA	69681379	AAAACG	I	A
-0.153052	cg0756497	cg0756497	69750510	TATAATTCATAATTTTTCCCTA			II	
0.1104211	cg0757468	cg0757468	65680381	AAAAAATTCACCCCCRCAACC			II	
0.1029293	cg0758409	cg0758409	20662320	AAAAATATCTACCTTTATCTTA			II	
0.2767876	cg0758462	cg0758462	33708372	RTCTTCTAAATACRAAAACCC			II	
0.1459396	cg0758485	cg0758485	18629366	CTACAAA	26751504	CTACAAA	I	T
0.1159888	cg0758542	cg0758542	50730344	AACAAATCAATTTCAAAAAAT			II	
0.1061618	cg0758698	cg0758698	38602306	ATACCATTACTAACTTCCAAA			II	
-0.113556	cg0759272	cg0759272	36761355	CAAAAATATCAACAAACACTT			II	
-0.224485	cg0759277	cg0759277	12673368	AATAAAACCTAATAAACCCTC			II	
0.1201659	cg0759797	cg0759797	32649400	ATTCATTTCTATTTTCTACACA			II	
-0.111592	cg0759895	cg0759895	44802330	AAAAAA	54623508	AAAAAA	I	A
0.2426614	cg0759913	cg0759913	30633361	AACCTAAAAACTATACAAAAC			II	
0.2370452	cg0760062	cg0760062	71672475	CAACACC	12755445	CAACGCC	I	C
-0.123734	cg0760752	cg0760752	39677319	AAAATACTATATATTCTAAAA			II	
-0.126354	cg0761112	cg0761112	29737489	AAACCRAAAAAAAATTCCTAA			II	
-0.14511	cg0761580	cg0761580	60627488	TATACRCATCAAACCTAACAT			II	
0.1107133	cg0761968	cg0761968	27762364	TTTTAAA	39611482	TTTTAAA	I	A
0.233622	cg0762085	cg0762085	70743481	TTTCACAATCAAACCTAATAA			II	
0.1638928	cg0762169	cg0762169	53769468	CTAACTTCTCCTAAACACTTAC			II	
0.1193129	cg0762577	cg0762577	59768425	ACAAAAT	70628390	ACAAAAT	I	A
0.3304715	cg0762962	cg0762962	50723326	ACAAAA	15760401	ACAAAA	I	T
-0.107029	cg0763366	cg0763366	37800461	TATTCCTAACRTCTAAAATTTA			II	
-0.341844	cg0763919	cg0763919	19621471	CTACATAAAACCTCAAATCCT			II	
-0.11444	cg0764157	cg0764157	58611359	ATCATAAAATATCATATAAAT			II	
0.1804428	cg0764276	cg0764276	12687381	TTTACTTATTCCCACRACCTAA			II	
	cg0764961	cg0764961	45763323	CCAAACT	73790462	CCAAACT	I	C
0.1276408	cg0765218	cg0765218	49739311	CTCATCAAAATAACCTTTATTA			II	
-0.193929	cg0765526	cg0765526	68715408	AACTTAAACCAACTAAAACAA			II	
-0.10967	cg0766083	cg0766083	70620322	TAAAAACCTACTCAAACTCRC			II	
0.1171902	cg0766212	cg0766212	33664390	ACCAAA	12712374	ACCGAA	I	C
-0.142082	cg0766457	cg0766457	35709459	TTTCCAACRATACCCCAAAAA			II	
0.1115856	cg0766522	cg0766522	40601507	TAAAATCAAAAAAACAATTAA			II	

-0.114206	cg0767051(	cg0767051(	47794342	AAAAAAAAACCAATACCTATC			II	
-0.106083	cg0767281⁄	cg0767281⁄	11618327	CAAAAAATAAATCCCCAAAAC			II	
-0.113583	cg0767302(	cg0767302(	10645390	ATTTTTACCCCTTAAACTATAC			II	
-0.112818	cg0767413⁹	cg0767413⁹	66667457	ACAAAA⁄	22611489	ACGAAA⁄	I	T
-0.14431	cg0767636	cg0767636	48778458	CCAAACT	46705324	CCGAACT	I	T
0.1194801	cg0767715	cg0767715	18621333	TAATAAATAAATCATCACCTA⁄			II	
-0.146471	cg0768694	cg0768694	62687300	CACAAA⁄	70644435	CGCGAA⁄	I	T
0.1037025	cg0768973	cg0768973	47623328	TAACTCTAACTATAACATCAA⁄			II	
0.1138563	cg0769627	cg0769627	11720369	TAAAATTCTTTTCTATTACTAA			II	
	cg0769722	cg0769722	26785447	AATAAAC	13644463	AATAAAC	I	C
0.1132448	cg0769819(	cg0769819(	22657484	ATTTAACACCAACRAAATTCC			II	
0.123244	cg0770039	cg0770039	59645308	CTTCCCATAACTCTATAAAAA⁄			II	
0.1042991	cg0770130	cg0770130	54789502	TATACCAATAACTAATCTCCA⁄			II	
-0.108722	cg0770175	cg0770175	57744315	CTCTACT(	13608390	CTCTACT(	I	A
0.1432821	cg0770223	cg0770223	20646417	TTCTAATTACTCTCTACACTTC			II	
0.11729	cg0771108	cg0771108	22672493	CTAAATT	66748471	CTAAATT	I	A
0.2080329	cg0771216	cg0771216	36762442	CAAAAA⁄	63613473	CAAAAA⁄	I	C
-0.113395	cg0772462	cg0772462	62627470	AAAAATACAAAATCCRAAAAA			II	
0.1278539	cg0774030(	cg0774030(	60667476	TTCTCTA(	32658314	TTCTCTA(	I	A
-0.139923	cg0774570	cg0774570	28677340	CATAAAA	68655410	CATAAAA	I	T
-0.138871	cg0774725	cg0774725	64713395	ATTAAAA	39679370	ATTAAAA	I	A
0.1818912	cg0774796	cg0774796	30725447	AAAAACA	24621454	AAAAACA	I	C
0.1364633	cg0774959	cg0774959	52632437	ACCAAACCCACRAATCTAAAC			II	
-0.12195	cg0775240⁹	cg0775240⁹	42685376	AACAAAAAAACCTCAATCRAT			II	
0.1391922	cg0775336(	cg0775336(	65787348	CAAAATACTAAACATTAAAAC			II	
0.1010506	cg0776405⁹	cg0776405⁹	27785330	AACTACACATAAATATATTTA⁄			II	
0.1371812	cg0776598	cg0776598	58716480	CTACAAATCTTTTAACTACCA⁄			II	
-0.131264	cg0777442⁄	cg0777442⁄	26672398	RACCRAAATTACAACATTTCT			II	
-0.175558	cg0777488⁄	cg0777488⁄	43685418	AAACTAAAAACCCAAAAACTA			II	
0.2185503	cg0777704	cg0777704	71750361	TCCRTATCTAATATAACCACTT			II	
-0.112865	cg0778034	cg0778034	20804304	CRAATTAAATTAACTTAACRA(			II	
0.1405153	cg0778170	cg0778170	42706506	TAAATAC	66726492	TAAATAC	I	A
0.1165398	cg0778754	cg0778754	21627306	ACACRATCTAACAAAACATAA			II	
-0.417255	cg0779287	cg0779287	37774469	AATTCTCTAATCCATTTATAAA			II	
0.2340067	cg0779516	cg0779516	41604509	AAACAAC	41702418	AAACGAC	I	C
0.1111146	cg0780447(	cg0780447(	29663367	ATACTTCTATCTTAATCAACTC			II	
0.1122965	cg0780506⁄	cg0780506⁄	48751392	AAAATCAAAAAACTTAACTTA			II	
0.1748158	cg0780554	cg0780554	11719474	ATATCAT	33743421	ATATCAT	I	T
0.182264	cg0780711	cg0780711	28755369	CTTTACTAAAAACACACACAC			II	
0.2846488	cg0780737	cg0780737	56788301	TAAAAACAAATCATTCTAATA			II	
-0.106583	cg0782078(	cg0782078(	37641313	TAAAATA	69718340	TAAAATA	I	A
-0.128551	cg0782137⁄	cg0782137⁄	36780352	ACRATAATAACAAAAAAACCT			II	
0.1938998	cg0783131	cg0783131	61647402	AAATTTAAATATATTCCAAAA			II	

-0.158066	cg07832006	cg07832006	45781488	AACCTAA	70708348	AACCTAA	I	A
-0.122917	cg07839926	cg07839926	62620439	AACTCCA	52777432	AACTCCG	I	C
	cg07845136	cg07845136	13644465	TCCTACCAAAAAACTACRATA			II	
0.1429958	cg07846874	cg07846874	12763311	TAAAAATATATTTCCTCAACT			II	
-0.247996	cg07848485	cg07848485	28767464	CCTCTAAAATCTATCTCATCCA			II	
0.1918575	cg07850323	cg07850323	53702395	TAAATATTATCAACATAACCT			II	
-0.174416	cg07851262	cg07851262	56711481	ATAACACAACCTATAAACAAA			II	
0.1186125	cg07854113	cg07854113	10734336	AAATAAAATAAATCAAAAAA			II	
0.1832101	cg07855575	cg07855575	14780479	TCTATAAAAATAACATTAAAA			II	
0.1472152	cg07862461	cg07862461	34704447	CACAAAAAAAACCCTCCAAAA			II	
0.1004099	cg07863022	cg07863022	57771331	CTATCTT	68707371	CTATCTT	I	A
-0.292845	cg07866725	cg07866725	28615375	TAACTAAAAAATCAATACTAA			II	
0.1492693	cg07866909	cg07866909	41604333	ATATTTTCTAATCCTTCTTCAT			II	
-0.10029	cg07870541	cg07870541	55751327	CCAACTACTTAAAACRATAAA			II	
-0.216296	cg07870920	cg07870920	17666406	CAAACTATTAAACCTATTAAT			II	
-0.104612	cg07874011	cg07874011	16673473	ACTACTAAAATCATCRCCCAT			II	
0.1187772	cg07875068	cg07875068	46632507	AACTTCTACAAACACCTCTAA			II	
0.1186846	cg07876320	cg07876320	17621427	AAAACAAACTACAAACTCAAA			II	
-0.11348	cg07876831	cg07876831	50661376	CCTTCTATATCTAAAATATACR			II	
-0.297579	cg07878625	cg07878625	18757475	AACTATAAAAACCCAAATCAA			II	
-0.168422	cg07882838	cg07882838	55609344	AATTTACTACTAAACCTCCTA			II	
0.153387	cg07884867	cg07884867	20720372	AAAAAAATTTTAACCTCATAA			II	
-0.101768	cg07889003	cg07889003	49677481	CTCATCT	55756510	CTCATCT	I	T
0.105166	cg07896009	cg07896009	35729356	TCAATACTCTCCTATAATAAA			II	
0.1533995	cg07901514	cg07901514	28689449	AAATCTAATTCACTCACTATTA			II	
0.1086414	cg07904290	cg07904290	58651475	AAACCTC	39795354	AAACCTC	I	T
-0.1312	cg07906262	cg07906262	13766454	AATAACA	53717395	GATAACA	I	A
0.1012136	cg07909178	cg07909178	30800340	AACRTTCRATAAAATCRATTA			II	
0.1180405	cg07915976	cg07915976	42781406	CAAACTCATATTTCCTTAATAA			II	
-0.129832	cg07917609	cg07917609	69634403	CCCTAAACCTAAAACCTACRT			II	
0.1151134	cg07925064	cg07925064	41794502	ACAAAA	24744335	ACGAAA	I	A
-0.130853	cg07929768	cg07929768	27741510	TACCCAATAACCTAACACTTA			II	
-0.180628	cg07930587	cg07930587	63750350	TTACTCTATATTAAATCTACAC			II	
-0.107847	cg07932899	cg07932899	25694489	TCAAAATCACCCAAAACCCCC			II	
-0.122734	cg07933078	cg07933078	54680309	AAAAACA	28641391	AAAAACC	I	T
-0.100569	cg07936950	cg07936950	73760356	CCAAACC	74623336	CCAAACC	I	A
0.1425642	cg07943832	cg07943832	28624394	ACATTTTAAATCAATATTAATC			II	
-0.114411	cg07950979	cg07950979	33721323	CAATAAAATAAACTTTAACAT			II	
-0.113651	cg07952421	cg07952421	11676351	AAATTAATTTAATCCCTAAAA			II	
0.4522102	cg07955105	cg07955105	72695488	ACCAAAAACAACRAAACACA			II	
0.1457539	cg07960624	cg07960624	62713349	AAACAATACTTATATATCTAA			II	
-0.17885	cg07962934	cg07962934	60709444	AAACAAA	30743500	AAACGAA	I	A
0.1172881	cg07964219	cg07964219	48640371	TTCCCTACTTATTCTATCCTAA			II	

0.1512706	cg07964753	cg07964753	67661509	TTCCCCA	23759375	TTCCCCA	I	C
0.1271624	cg07970799	cg07970799	46613418	AATAAAC	31718497	AATAAAC	I	C
0.1012443	cg07971596	cg07971596	30638432	AATTCATTATATCTAAACTACR			II	
-0.198178	cg07973162	cg07973162	72765306	CAACTCATTCCTACCTAACACC			II	
0.1270259	cg07973309	cg07973309	71741400	ATAAACCCTTAACACACAACT			II	
0.1158289	cg07976787	cg07976787	44746439	TCTACCRTACATCTTTACTTAT			II	
0.115746	cg07979348	cg07979348	53691440	TCACATAACAACCACAAATCT			II	
0.1323496	cg07981266	cg07981266	67766331	CCCTAACTTAATTCTAAATCAC			II	
0.1386829	cg07984380	cg07984380	69795483	ACAATTTACACCAAAATTCCT			II	
0.1001793	cg07996838	cg07996838	28746438	ATAAAACCCCTTCACAAAAAA			II	
0.113793	cg08000731	cg08000731	52628380	AAAAAAAACAAAACCAAACA			II	
0.1227784	cg08003402	cg08003402	55749387	CTACRCTTAATACRAAACCAA			II	
-0.220164	cg08008233	cg08008233	50707487	CCCRATTCTCAAAACATAATA			II	
0.1814135	cg08010331	cg08010331	64762362	AACACTTACTTATACCAAACA			II	
0.1200455	cg08017634	cg08017634	39801427	TCCTAATAATACAACTACTAA			II	
0.1028074	cg08018143	cg08018143	71767433	TCCTTCCRATCCCACTTCCAAA			II	
0.3285781	cg08024264	cg08024264	66713451	TAAACTTTTCAAAAAATATCC			II	
0.1318196	cg08029299	cg08029299	48617371	AATCAAATATACCCCCCRACC			II	
0.1113562	cg08029969	cg08029969	61672364	TAAAAAA	73645392	TAAAAAA	I	A
0.1443585	cg08030922	cg08030922	36730347	AACTTTATTTAACACAACCA			II	
0.1103452	cg08035351	cg08035351	43606408	TTAATTAAATAACTATATAAT			II	
0.1179264	cg08038033	cg08038033	10739488	ACACACTCTTCTATTAAAAAA			II	
0.139995	cg08039587	cg08039587	10696356	CTTATAAAAAACCAACCCRAA			II	
0.125322	cg08041448	cg08041448	36801357	AAACCAC	14739409	AAACCGC	I	C
-0.334711	cg08048268	cg08048268	49647508	CATCCAA	41755442	CGTCCAA	I	A
-0.254431	cg08053137	cg08053137	35684432	ACCCAACAACCCAAAATCTAC			II	
0.1838312	cg08062206	cg08062206	39733387	AAAAAAA	35620477	AAAAAAA	I	C
0.124105	cg08064292	cg08064292	58725450	AAAAACCRAACAACACCTAAT			II	
0.1144995	cg08065229	cg08065229	56800391	CTCTTTATAACCATAAAACTTA			II	
-0.174301	cg08076437	cg08076437	38678341	CCCAATT	17774500	CCCAATT	I	C
0.1011966	cg08081780	cg08081780	53621420	CTTTCCRATTTAAAATATTATT			II	
-0.111927	cg08084154	cg08084154	46694365	AAACTAA	21755423	GAACTAA	I	A
0.1620789	cg08091666	cg08091666	65671470	AAAAAATATAAAATTTCCCCT			II	
0.1337979	cg08097817	cg08097817	37758335	TATCTAAAAATCATACTATTAC			II	
-0.115861	cg08101303	cg08101303	20654307	AACAAAA	28717434	AACGAAA	I	A
-0.232518	cg08102564	cg08102564	29676374	AAACAAAACAACCTAACTAAA			II	
0.1162637	cg08103472	cg08103472	27698360	CACCTCCCACCCTTAAAAAAA			II	
0.1210296	cg08106377	cg08106377	57799424	TCAAAAAATAAAAACAATACT			II	
-0.101535	cg08109808	cg08109808	54645374	ACTACTCTACCATTACATTCCA			II	
-0.148686	cg08118287	cg08118287	33687506	AATATAT	63705377	AATATAT	I	C
0.2213495	cg08119527	cg08119527	29757498	TCACATTCTAATACTCTAAAA			II	
0.2472957	cg08121408	cg08121408	50683352	CCCCTTATCTTTCACACTAACC			II	
-0.126469	cg08121453	cg08121453	29648478	AAAAAAA	69782310	AAAAAAA	I	A

0.1035403	cg08121686	cg08121686	67779397	CCCAAAAAAACCCRTTAATAA			II	
0.1852252	cg08123413	cg08123413	47778399	AAAAATCCAATATCTTCAAAA			II	
0.1144629	cg08125824	cg08125824	74679390	CTCAAATACTCACTAAAACAT			II	
0.2217754	cg08128264	cg08128264	12676359	ATAAACATCRTATAAAAAAAC			II	
0.1285927	cg08133669	cg08133669	65716403	AAAACATTATAACCACTCTTC			II	
0.1902505	cg08133699	cg08133699	66746321	CCTACATTAAACATTAACCAT			II	
0.1121895	cg08140114	cg08140114	17754498	TACTAAAATATATTAACCTCT			II	
0.1541265	cg08143045	cg08143045	39734330	AATTATATTAATCCRTTCTCAC			II	
-0.205791	cg08146708	cg08146708	24809380	ACATACA	58767470	ACGTACA	I	C
-0.331645	cg08146865	cg08146865	26716431	CTCTTTACTAAAAACTATTCAT			II	
-0.111951	cg08151623	cg08151623	67731429	CTCACAC	47650332	CTCGCGC	I	C
0.1268312	cg08157310	cg08157310	34648300	ACTACTTCATTTTTAAATTTTA			II	
0.2850244	cg08158105	cg08158105	26747343	ACCAACTCAACTATCCAATTC			II	
0.1383789	cg08160949	cg08160949	31700426	TATCACTACCAATACTCAAAT			II	
-0.176596	cg08166631	cg08166631	15660481	CTTAAAAACAAAAAATCTCAC			II	
-0.102788	cg08170293	cg08170293	38600397	CACCCAA	32703303	CACCCAA	I	A
0.1280339	cg08172105	cg08172105	29651467	TAAACCTAAATTCCATATCTTT			II	
-0.180323	cg08172479	cg08172479	43682494	ATCAATAATAAATAAAACTTA			II	
-0.128841	cg08174789	cg08174789	29778377	TAATAAA	66723452	TAATAAA	I	T
-0.202327	cg08176410	cg08176410	41780301	ATAACTCCCTTTCCTTTCCACA			II	
0.1067869	cg08177015	cg08177015	10682320	CTTAAAACCRAAAATTTAAAA			II	
0.1044523	cg08191781	cg08191781	53774317	TCCAAACTAAACATTTAAACA			II	
-0.112685	cg08194323	cg08194323	26713357	AAAATCCAAAAAAACCCTACA			II	
-0.111649	cg08195983	cg08195983	24722422	ACTCTAC	33772334	ACTCTAC	I	C
-0.29123	cg08200543	cg08200543	49726317	AAAATCCCATAAAAAAACTTA			II	
0.1149466	cg08200621	cg08200621	13714477	AAACTCC	49744305	AAACTCC	I	T
0.2485292	cg08203460	cg08203460	15742320	TCACAAATAACTATAAATTAA			II	
0.1308624	cg08206092	cg08206092	64636505	TTCATCCATAACTTAAAAATA			II	
-0.114741	cg08212862	cg08212862	13607487	AACRATCCTACATCTAAATCT			II	
0.2959591	cg08217354	cg08217354	21657325	TATTCACTCAACAACCCTTTAA			II	
0.1328193	cg08219170	cg08219170	23631406	AATATCTATCTCCTCCTCTAAA			II	
0.1046992	cg08219218	cg08219218	10722412	TAACACC	37623347	TAACGCC	I	T
-0.17244	cg08222513	cg08222513	65721447	TAAACAA	29784479	TAAACAA	I	C
-0.154286	cg08222618	cg08222618	38686484	CAAAACC	44694343	CAAAACC	I	C
-0.283437	cg08231696	cg08231696	38645482	AACACTA	71666438	AACACTA	I	A
-0.165362	cg08233148	cg08233148	35780324	CTTTATAAAACTCAAAATCAA			II	
0.1353603	cg08237565	cg08237565	55635419	RCCTTCCAAAACTCATAATAA			II	
-0.193351	cg08238319	cg08238319	35661439	TTACTAACTCAACCAAAAAAA			II	
-0.490015	cg08238375	cg08238375	47615483	CTTCACCRAATTTTATCTTACT			II	
-0.113397	cg08239694	cg08239694	56785368	AAAAATC	50644420	AAAAATC	I	A
0.1671234	cg08242232	cg08242232	61655384	CTAATAT	16797406	CTAATAT	I	T
0.10553	cg08242859	cg08242859	16673423	AACCTAAATCAAAAACTCAAA			II	
-0.125782	cg08243728	cg08243728	43786494	TACTATA	33657422	TACTATA	I	A

-0.412715	cg08250118	cg08250118	44675342	TCCAATTTCATACCCCCTCTCC			II	
0.4214152	cg08253809	cg08253809	62661502	TAAAATAATCTTTCTACCTCAA			II	
0.1548395	cg08258654	cg08258654	11782430	TCATTCCACTCCAATTAAATAA			II	
0.1149902	cg08263140	cg08263140	54700302	TCACTCTACTCCAAACACATTA			II	
0.1343079	cg08265392	cg08265392	51662354	TCCAATAAATATCACTTCCRTA			II	
0.1002246	cg08268692	cg08268692	68794349	ATATCTAAATCACTTATTAATC			II	
-0.223056	cg08269974	cg08269974	27605303	ATCAACC	16682363	ATCGACC	I	C
0.2672935	cg08272429	cg08272429	24608456	AACCAAATAAAATAATAAAAA			II	
0.1134489	cg08272591	cg08272591	70605446	AAATAATTCAAATAAATTAAC			II	
-0.141815	cg08274589	cg08274589	28666467	TAACCAA	37803421	TAACCGA	I	C
0.1158624	cg08282819	cg08282819	42753354	AATACACRAATTTCCTACCATT			II	
0.1659979	cg08285768	cg08285768	34793433	ACTCCAAAATAACTAACTCTA			II	
0.3769266	cg08289272	cg08289272	23652432	TTCCTAACAAACTTTAACAAAT			II	
0.1752833	cg08292959	cg08292959	70754447	ACAAAAA	36781418	ACAAAAA	I	C
0.1376459	cg08293002	cg08293002	20705346	RCRAAATAAACTAAACTTAAA			II	
-0.148726	cg08293242	cg08293242	47695457	TAAACCC	43706315	TAAACCC	I	A
0.2431689	cg08293392	cg08293392	58736354	CACRAAAACCCAACTCCACCT			II	
0.1042964	cg08296868	cg08296868	37627429	TCACAAA	27764490	TCACGAA	I	A
0.2414083	cg08299859	cg08299859	52694424	TTCTACRTATTCCTTTCTATTC			II	
-0.228682	cg08300475	cg08300475	10660302	AAAAACACACACAAACACAAA			II	
0.178906	cg08305942	cg08305942	20600451	AATAATACRAACACAACTAAT			II	
-0.10316	cg08308214	cg08308214	44803398	TACTTCATCTCTCTCTCTCTTTA			II	
0.1261563	cg08309687	cg08309687	50726380	ACTCTACAAACCACACTCAAA			II	
0.1352236	cg08312765	cg08312765	63701438	TTTTATCACAACAAAAATCTTA			II	
0.1002296	cg08316699	cg08316699	12625334	AAATATAACTACTACTAAAAC			II	
-0.100957	cg08317694	cg08317694	40661481	ACTCATA	58673376	ACTCGTA	I	A
0.1086729	cg08319103	cg08319103	33739454	TACTATT	52676471	TACTATT	I	A
-0.131433	cg08333400	cg08333400	22642441	CTATCTACTTAAAATACATACA			II	
-0.110216	cg08335008	cg08335008	54613424	CAAAATC	69684370	CAAAATC	I	A
-0.100484	cg08348121	cg08348121	70650432	AATAAAC	41667361	AATAAAC	I	A
0.1443407	cg08355045	cg08355045	52778306	AACATATAAACACAAAACTTA			II	
-0.156958	cg08355157	cg08355157	52777483	ACAAATCAAAAAATCCTTAAA			II	
-0.15133	cg08355456	cg08355456	49704335	ATAAAAC	42675320	ATAAAAC	I	A
-0.132641	cg08357436	cg08357436	14790391	AAAAACA	62719414	GAAAACC	I	A
0.1125311	cg08360511	cg08360511	36755457	AAAAACT	30758505	AAAAACT	I	A
0.1751365	cg08367801	cg08367801	35601365	TTCTTTAACAAAATTAATCRCT			II	
0.1016095	cg08368875	cg08368875	46750400	TATAAATTCATACTTAAAATA			II	
0.1008298	cg08373528	cg08373528	57676503	CAACCTC	38676483	CAACCTC	I	A
0.1732172	cg08373622	cg08373622	71729412	AAATACCTAAACTTAAAAAAA			II	
0.1275376	cg08373989	cg08373989	65751422	CCCCATCTATACACAAAATTA			II	
0.1548124	cg08378932	cg08378932	65704464	AACAAAAAAACAACAAATATT			II	
0.1058357	cg08381046	cg08381046	64661310	ATACACAAAAAAAACCTAAAA			II	
-0.168043	cg08381537	cg08381537	45777500	AAAAAAA	32712359	AAAAAAA	I	C

-0.148792	cg08390209	cg08390209	16629508	AACCTAAATTATAAATTCACC			II	
0.1626788	cg08392746	cg08392746	41786390	AATAATTTTCTTAAACAATAA			II	
0.1124759	cg08395748	cg08395748	63799460	AACAAAAATTACAAACTAAAT			II	
0.1419878	cg08395784	cg08395784	42682355	AACRCCRTAAAATAAATACCA			II	
-0.131724	cg08395793	cg08395793	70795332	AACCAAA	34745451	AACCAAA	I	A
0.2531378	cg08397993	cg08397993	71613387	AAAACRCCACAAACCATAAAA			II	
0.1315473	cg08401725	cg08401725	14735409	CAACCAACTCTCATCCTCAAA			II	
0.1277377	cg08401938	cg08401938	24802413	AACACTA	60775315	AACACTA	I	A
-0.107645	cg08404009	cg08404009	43785333	AATTAATCTCCAAAACTAACA			II	
0.1277192	cg08404513	cg08404513	10750439	AAATATTTATACATAATTATC			II	
0.1021813	cg08412215	cg08412215	41660507	ACAAATAATAACAAATTAAAC			II	
0.1062266	cg08419450	cg08419450	65690351	TAATCATAAATAAAAACAAAA			II	
0.1595522	cg08421351	cg08421351	30674480	ATAAACCAACTAAACTAACAA			II	
-0.22496	cg08422420	cg08422420	47792331	ACACAAA	14673478	GCACGAA	I	C
0.1065222	cg08426157	cg08426157	32668320	ATAAATT	55698420	ATAAATT	I	C
0.1085569	cg08429705	cg08429705	51699499	AATACTTACATATACACCTCT			II	
0.2088843	cg08430277	cg08430277	70781380	AAAATATACATTTATTTCCAC			II	
-0.124947	cg08431931	cg08431931	62738320	AAAAAAC	38635408	AAAAAAC	I	A
-0.138168	cg08433110	cg08433110	15624481	TACTATATAAACATCCRCTAA			II	
-0.193242	cg08439880	cg08439880	23712406	CCTAAAA	17785323	CCTAAAA	I	T
0.1073277	cg08442052	cg08442052	52701303	CCTATTCTATTTAAAATATCCT			II	
0.116593	cg08443563	cg08443563	54652399	TCTCCACRTATAAATCAAAAA			II	
0.1036612	cg08447044	cg08447044	55731332	AACTTACAACTCCCACTCRAT			II	
-0.105271	cg08449138	cg08449138	33641400	TATCTCTTAACTACCRTACCTA			II	
-0.137298	cg08449558	cg08449558	39708435	CAAATACTACTTCATTTTATAT			II	
0.1430532	cg08454015	cg08454015	10677480	CATACCCTTTACTTACAAAAC			II	
0.2197807	cg08460078	cg08460078	54647413	TTTTACRTCTCTATATCTTTTC			II	
-0.177994	cg08461772	cg08461772	65755313	ATACTACCCAAACRAACTTAT			II	
-0.100111	cg08465120	cg08465120	54709363	TTCTTATAATCRAATAACRTCC			II	
-0.198684	cg08470157	cg08470157	41736488	AAAACAA	59769377	AAAACGA	I	A
0.2373981	cg08476485	cg08476485	66810458	CTATCCAAAAATTTCTTAAAC			II	
0.1039985	cg08476984	cg08476984	12689487	AATAACTCTAACTAATAACCC			II	
0.1920692	cg08481002	cg08481002	13800314	AATAATCTTTAAACAAAATTA			II	
0.1785951	cg08484992	cg08484992	56696315	AAACAATAAATATCCCCCCAA			II	
0.1042475	cg08491487	cg08491487	35789426	CTACATA	50652323	CTACGTA	I	A
0.1493719	cg08494843	cg08494843	49659388	TATCTCAATAAACTACTTTCCA			II	
-0.130838	cg08495239	cg08495239	12625354	TAAAACT	24649304	TAAAACT	I	A
0.1245939	cg08498719	cg08498719	12646452	AATTAAAATTAATATTTTAAC			II	
0.1080869	cg08499861	cg08499861	71658448	CCCCCCATTTAATTTTCCTTTA			II	
0.1086856	cg08500138	cg08500138	22635426	ACAAACAACTACCCTAAAAAA			II	
-0.214189	cg08504662	cg08504662	47748432	CAAATACTCRACCAAATTAAA			II	
-0.143032	cg08506606	cg08506606	25649385	CCATAAAAAAACACTCAAATA			II	
0.1084335	cg08506672	cg08506672	22731330	ACTCAAAATCCTAAAAAACAA			II	

0.1172377	cg08510234	cg08510234	26625348	AACRCTTCTACAATAACCAACA			II	
-0.268577	cg08514194	cg08514194	72705460	ATACCAAACTATAATTATCTCA			II	
0.1019353	cg08514594	cg08514594	26704441	AAATTCTCRAACCAACAATAAC			II	
-0.111815	cg08516507	cg08516507	74769420	AAAAACT	31620311	GAAAACT	I	C
0.1478333	cg08519385	cg08519385	40635303	AAATTTTAAACAAAAAAATAA			II	
0.185554	cg08522473	cg08522473	53649408	TCTAATCACCTACAACCTTCTA			II	
-0.330454	cg08528516	cg08528516	50744305	ATTTCATTATTTCTTCATCCTAT			II	
0.1088546	cg08534016	cg08534016	31770413	AAATTCTATTAAAACCTTAATC			II	
0.1064101	cg08534830	cg08534830	72715472	CRATTAACAAACCCTACAACTA			II	
0.1143776	cg08544331	cg08544331	36602436	AAACRTATAAAAAATCTTCCAT			II	
-0.120667	cg08545129	cg08545129	49601481	AACAAAA	35603438	AACGAAA	I	A
0.1190709	cg08548044	cg08548044	70708459	AACATCC	57646326	AACATCC	I	C
0.182145	cg08549335	cg08549335	25671469	TCATTAACTCTTCACTTATTAC			II	
0.1326653	cg08554554	cg08554554	30790360	ATCAAAATAAAAATAACRCAA			II	
-0.131455	cg08555866	cg08555866	41732344	AAAACCA	67769494	AAAACCC	I	C
0.1114679	cg08556511	cg08556511	73691397	CTAATCTCRTATATTTATAATA			II	
-0.122766	cg08568145	cg08568145	46628382	RAACACAAACCCACRTAAAAA			II	
-0.120144	cg08570077	cg08570077	41775416	ATAACATCTATTACAACCATAT			II	
0.1066889	cg08570243	cg08570243	44686396	AAAAACCRACCATAACAAACR			II	
0.1185171	cg08573687	cg08573687	50731446	CATAATA	73728429	CGTAATA	I	T
-0.115114	cg08575621	cg08575621	17797489	TAATAAA	42789326	TAATAAA	I	A
-0.133398	cg08579577	cg08579577	36793409	AACTCAA	64755341	AACTCAA	I	A
0.1700161	cg08580224	cg08580224	21671300	AATATTT	73718316	AATATTT	I	T
-0.112162	cg08580942	cg08580942	46631351	TAAAAAAATTTCCAAACTACC			II	
-0.162092	cg08590939	cg08590939	22698447	AATTCTAAACCTCTAAAAATTC			II	
-0.11285	cg08593141	cg08593141	34654499	AAACTTCTTATAACCCAACCTT			II	
0.1326931	cg08594246	cg08594246	23637373	CTAAAACCAATAAAATTACRA			II	
-0.159769	cg08597142	cg08597142	12766306	CTACCCCTACTCTATCAAAAAC			II	
-0.100378	cg08599792	cg08599792	56784382	ATATACT	10679329	ATATACT	I	A
0.1124714	cg08600378	cg08600378	27601382	RATTATATCTACTAAACACAAA			II	
-0.132711	cg08601628	cg08601628	40606444	TCCTAAA	64681421	TCCTAAA	I	T
-0.11535	cg08602346	cg08602346	59802479	TTACACTAAAATTAACTTAATA			II	
-0.371724	cg08603678	cg08603678	74712324	TCCTAACAACATCTACATTTAA			II	
-0.412688	cg08605899	cg08605899	73641389	AAAAACTAATTATCTTACCATT			II	
-0.194252	cg08610426	cg08610426	44703481	TTTAAAA	34722474	TTTAAAA	I	A
0.1013988	cg08611810	cg08611810	14733362	AAACCCC	65701446	AAACCCC	I	A
-0.119779	cg08613577	cg08613577	38600454	CRATATTTCCTCAACCCCCTTT			II	
0.1248278	cg08616111	cg08616111	28786359	CCAACTAACCAAACTAAAACR			II	
-0.19056	cg08617160	cg08617160	16688382	AATAAAC	27638409	AATAAAC	I	C
0.122699	cg08619295	cg08619295	58688430	ATAAATTATCTATTAACATATT			II	
-0.113036	cg08621926	cg08621926	17740470	CTTAATCRCTAAACTTTAAACR			II	
0.1109802	cg08621957	cg08621957	31758403	ACRAAACTACCAAACACTAAT			II	
0.1227906	cg08622198	cg08622198	36738381	TAAATCAACCTCATAAAAAAA			II	

0.1077507	cg08624915	cg08624915	55739509	CACATTTTACAAATAAACCACC			II	
-0.142836	cg08626646	cg08626646	26754487	ACAACAA	20803365	ACGACGA	I	A
-0.182155	cg08629394	cg08629394	27702369	ATCATCTTAAAAATTTCCTTAA			II	
-0.155109	cg08632164	cg08632164	30708378	CAAATAC	47671432	CGAATAC	I	A
-0.177958	cg08633479	cg08633479	67714503	TCCCCAA	57641490	TCCCCGA	I	C
-0.192523	cg08634229	cg08634229	56607509	CACAAAC	74768387	CACAAAC	I	C
0.1010218	cg08635406	cg08635406	52656319	CRAAACCCAACTCCAAATAAA			II	
0.1278734	cg08638632	cg08638632	56799350	CTATACCTTCTAATACTCTAAC			II	
-0.106715	cg08640901	cg08640901	44605456	TACAAAACCCTAATTCAACTCC			II	
-0.107385	cg08643994	cg08643994	15644504	CACAAAC	38770333	CGCGAAC	I	A
0.1114974	cg08655953	cg08655953	34747398	TTAAAAA	53704486	TTAAAAA	I	T
-0.105303	cg08656770	cg08656770	52684461	TATAAAC	49781466	TATAAAC	I	A
-0.100925	cg08657228	cg08657228	67723380	AATTATATAAAATAATCCTATC			II	
0.136812	cg08660971	cg08660971	45613376	TCCTAAATCTATTCTTTCTTTC			II	
0.1411823	cg08661678	cg08661678	73644489	TCAATATCTAAATCAAAATCTC			II	
-0.197684	cg08669168	cg08669168	33740361	CAACCCTACAACTTACTAATTC			II	
-0.186671	cg08673225	cg08673225	10730369	TATACTACRCAAACRTATATAT			II	
-0.136723	cg08675117	cg08675117	65772402	TTAATATCTCAAATTCAAAACT			II	
-0.112968	cg08679971	cg08679971	33602320	CCCTTAA	31609499	CCCTTAA	I	T
0.1122788	cg08681473	cg08681473	36789373	CCCAAAAAATACTCCTAATACT			II	
-0.106177	cg08682981	cg08682981	35702373	AACTAAACCAAAACRAAATAC			II	
0.1507926	cg08692676	cg08692676	73808406	TAACTTACCCAAATTCTAATAA			II	
0.1294	cg08693140	cg08693140	63669365	ATCATAAAACCTTTTACAACCA			II	
-0.158391	cg08693172	cg08693172	40800304	TAAAACAAAACTTTCCRAACCA			II	
0.1318876	cg08697163	cg08697163	11621437	CTAAACCRTACCTAACCRAATC			II	
0.1134639	cg08697732	cg08697732	33712307	ACCATAACACCCCCATAATAA			II	
	cg08698010	cg08698010	58624364	AACCTACTTATTAAAACAACA			II	
0.2354706	cg08698943	cg08698943	23600491	CAACCCTATAATATAATTTATC			II	
0.1534717	cg08698997	cg08698997	28655508	TACTACC	44682395	TACTACC	I	C
0.1460714	cg08703522	cg08703522	69614440	AATATAT	66759488	AATATAT	I	T
0.1647442	cg08704154	cg08704154	41716415	TATACATAATATAATATTTACT			II	
-0.106525	cg08704562	cg08704562	26630392	TAAACCC	14673368	TAAACCC	I	T
0.137065	cg08708231	cg08708231	26660462	TCACATTCCAACACATACACAT			II	
-0.243041	cg08714433	cg08714433	62790489	CTCTATC	69754401	CTCTATC	I	C
0.1090403	cg08716255	cg08716255	20732476	TAACTAATACAATTAAATTTAA			II	
0.2031852	cg08720028	cg08720028	69803482	AATACTA	44618480	AATACTA	I	C
	cg08723131	cg08723131	54643455	AAAATCAAAACCAACAACAAA			II	
0.105781	cg08723977	cg08723977	72721356	CAATCTCCCAAATCCTAACCTC			II	
0.1085217	cg08725366	cg08725366	64717485	CAACTATAATAACTATCTTTAC			II	
-0.118984	cg08726801	cg08726801	26735476	AAAAATC	62677324	AAAAATC	I	T
-0.103642	cg08743751	cg08743751	19709509	ACCTTCCTATAATCCCTATTAC			II	
-0.138545	cg08745965	cg08745965	36726498	AAAATACTCCTCAACAACCTA			II	
-0.121936	cg08750439	cg08750439	31749440	ACCCTACTTACTATTACAACTA			II	

0.2404078	cg0875184(	cg0875184(	37652351	TCRATATCATAAAATAAACAA(			II	
0.2082422	cg0875187(	cg0875187(	66702311	CCATACAACATATACTTAACA/			II	
0.1982883	cg0875472:	cg0875472:	53774390	AACCAAA	57711316	AACCGA/	I	T
0.104081	cg0875659	cg0875659	11755499	CCCCCTCCTCCTATTCTACRTA			II	
-0.152531	cg0876242	cg0876242	72782327	ACATCTA	44675452	ACATCTA	I	A
-0.109778	cg0876260	cg0876260	64718325	TCAAACT	57715309	TCAAACT	I	C
0.1020696	cg0876676	cg0876676	41769319	AAAAATAAATCACACAAACAC			II	
-0.145468	cg0876768(	cg0876768(	26708436	AATAAAA	54725351	AATAAA/	I	T
-0.155159	cg0876782(	cg0876782(	64622436	TAAACAC	17761482	TAAACGC	I	C
	cg0877513	cg0877513	13644397	AAACCTA	10761385	AAACCTA	I	A
-0.273043	cg0877859	cg0877859	20629338	TAATACA	18760384	TAATACG	I	A
-0.10786	cg0878762	cg0878762	68644470	AAACCAT	20784419	AAACCAT	I	A
0.1010168	cg0878798	cg0878798	69718393	ACRATAAATACTCAATATATA			II	
0.108743	cg0878910	cg0878910	60601499	AATCTCA	47658449	AATCTCA	I	A
-0.123371	cg0880003	cg0880003	55608402	CTAAATTTAAAAACCRAATAC			II	
0.1064481	cg0880524	cg0880524	36619474	AAAAATAAAATAACACTAACA			II	
-0.213468	cg0880668	cg0880668	72794300	AACAACA	58644482	AACAAC/	I	A
-0.206844	cg0880804	cg0880804	57720364	AAATCAC	19705326	AAATCAC	I	A
0.1222917	cg0881210	cg0881210	49736318	ATAAAAC	70704466	ATAAAAC	I	T
0.1435689	cg0881402(	cg0881402(	10723353	AATTTAAAAAAAATCCAAAAT			II	
-0.144427	cg0881748	cg0881748	32807411	ACTAAAA	31629403	ACTAAAA	I	A
0.1289669	cg0881882	cg0881882	12738459	ATTCATA	62639458	ATTCGTA	I	A
-0.140105	cg0881902	cg0881902	64789365	ACAAAAA	34786465	ACGAAA/	I	T
-0.156422	cg0882078	cg0882078	37658307	ACATATACCTATTCTAAAAAA(			II	
-0.264219	cg0882335	cg0882335	50670510	AAAAAA/	63789405	AAAAAA/	I	C
0.1596132	cg0883107	cg0883107	43701438	ACTCCCA	50623425	ACTCCCA	I	A
-0.116033	cg0883260	cg0883260	22730507	ATACTCA	24800396	ATACTCA	I	A
0.1138977	cg0883748	cg0883748	46643471	CCCTTCCCTAAACTCAAAACT/			II	
0.2351668	cg0883980	cg0883980	50807329	TTCAAAT	42772412	TTCAAAT	I	A
-0.148587	cg0884044	cg0884044	53646483	AAACCTA	46678500	AAACCTA	I	T
0.1364833	cg0884244	cg0884244	73622480	CACCAAACAAATTTTACTATA/			II	
-0.122305	cg0884962	cg0884962	21611369	ATAAAAAAATTATATAATAAC			II	
0.1158443	cg0885483	cg0885483	32621455	AAAAATAAACACACCACACAT			II	
0.1867018	cg0885631	cg0885631	28731361	CTTCCCAACAACTATCAATCA(			II	
0.1234091	cg0885771	cg0885771	66670482	AACTCAATTTAACTCCCTAAA/			II	
0.1123181	cg0885829	cg0885829	54657471	AATTAACTTATCCAAAATCAC/			II	
-0.10516	cg0885854(	cg0885854(	11782329	ACAACCA	40764344	ACAACCC	I	C
0.1007907	cg0885921	cg0885921	24711352	AATCTCCCTTTCTACAAAACTA			II	
0.215509	cg0886143	cg0886143	43725335	AATAAAA	30676486	AATAAA/	I	C
-0.142614	cg0886434	cg0886434	47644483	AAAAAATACCTAAAACRCTTC			II	
0.1118246	cg0886666	cg0886666	29655384	CCCACAA	52639411	CCCGCGA	I	T
0.1341706	cg0886669	cg0886669	30707379	TAAAACTATAAACATAAACCA			II	
0.1263978	cg0886699	cg0886699	25793447	AATCCTA	61788475	AATCCTA	I	T

0.172286	cg0887003(	cg0887003(	13778435	TATACTTTATATATATATACCA			II	
0.1295656	cg0887009:	cg0887009:	44761460	TACTACTAATAAAATTTTTTCA			II	
-0.105398	cg0887064(	cg0887064(	66725454	ACCAACA	51730363	ACCAACC	I	C
0.1317891	cg0887123(	cg0887123(	15681417	AAAATAACCTACTCAAATACA			II	
-0.105561	cg0887394(	cg0887394(	33689432	ATAACCT	70689423	GTAACCT	I	C
-0.239677	cg0887984(	cg0887984(	19624427	ATTTACTATTTATTTTATATCT/			II	
-0.106657	cg0888820:	cg0888820:	16725414	CTAAACT	55726318	CTAAACT	I	C
0.1231115	cg0888891{	cg0888891{	24794482	AACTCCAAACACCTTACCCAA(			II	
0.1129554	cg0888911<	cg0888911<	12723366	AAACACAATTATTACAAAATA			II	
-0.106995	cg0889289(	cg0889289(	44664353	TACTCACCTACAAAAATATCC]			II	
0.1220433	cg0889303(	cg0889303(	34672391	ATAAAAATAATATTATATCTC(			II	
0.1348257	cg0889381'	cg0889381'	32715460	CAAAAACTCAATTTCCAAAAT'			II	
0.1184617	cg0889886(	cg0889886(	31694463	TTCAATAAATCCTCATATACTA			II	
0.1114935	cg0890630(	cg0890630(	45651328	ATTAACTAACTCCTTACTCAA/			II	
0.1414193	cg0891185:	cg0891185:	23691310	ATAAACTAAAAAAATTAAACT			II	
0.1114263	cg0891352:	cg0891352:	23809314	RACATTCTCACAAACTCTTATA			II	
0.2540009	cg0891683(	cg0891683(	34624375	AAAAACC	13683355	AAAAACC	I	A
-0.309842	cg0892045{	cg0892045{	34635319	TAATCTT.	43616387	TAATCTT.	I	A
0.1557393	cg0892359<	cg0892359<	37730375	AAATAACTAAAACAACAAAA/			II	
0.1257729	cg0892366(	cg0892366(	53698352	ACTTCCA	16617450	GCTTCCA	I	A
0.2145935	cg0892402:	cg0892402:	58652340	CCTTAACCTTCATACCAAAAA(			II	
0.1729476	cg0893090<	cg0893090<	32738498	AACACAA	66800498	AACACGA	I	A
0.1088693	cg0893328<	cg0893328<	74759488	ATACTATCCTAACATTACATAT			II	
-0.255871	cg0893879:	cg0893879:	40803324	TACCCAACCTAAAACAAACCT.			II	
0.1145408	cg0894252'	cg0894252'	72750374	TTTAATACCTACACCTTTAACA			II	
0.1657138	cg0894417(	cg0894417(	61667441	ACTACCCTTCCRCAAAAAACA(			II	
0.1166509	cg0894580:	cg0894580:	30799426	TAAAATTCTAAACCAAAATTA.			II	
0.1176092	cg0895918:	cg0895918:	49644505	ACTTTATCTAATTCTCCATTAA			II	
0.1076787	cg0896054(	cg0896054(	30662428	CTCCTTAAACCCAAATAAAAT'			II	
-0.387564	cg0896301:	cg0896301:	43682431	ATAAACCACACCAAAATAAAC			II	
0.2525921	cg0896473(	cg0896473(	40751468	TAAAAAAATTCATATCTTCAA/			II	
-0.328837	cg0896758<	cg0896758<	38708324	CTATAAACTCRCCACCCATTA(			II	
0.1460199	cg0897321'	cg0897321'	53746316	TTATTATTACTTTCCACTACAA			II	
-0.251488	cg0897583<	cg0897583<	58620476	AAAACACRAAAACTTACAACC			II	
0.5562427	cg0897731]	cg0897731]	31717420	CCAAACAAATTTCTACAATAT/			II	
0.1429918	cg0897793]	cg0897793]	50694496	CTTTAAAAACACTAAAAACCA			II	
-0.163419	cg0898557(	cg0898557(	29724312	ACCAACACRATTATATTATAC]			II	
0.1096339	cg0898597(	cg0898597(	11618323	TTTAAAACCRTTAAAAAAAAC'			II	
0.1249108	cg0899222(	cg0899222(	64715381	CTCACATCATTTCATAACTATA			II	
0.1100431	cg0899408:	cg0899408:	62731396	CACTATATCCTAAAATAAAAA.			II	
-0.11162	cg0899489]	cg0899489]	60713466	AACAAAA	19662452	GACAAAA	I	A
0.2349647	cg0899659'	cg0899659'	14613307	CRCCCACTAAAATAACTCATA/			II	
0.1394327	cg0899680:	cg0899680:	29671410	ACTTTCATATCTATAATCACAA			II	

-0.101829	cg0900681(	cg0900681(	68781379	CCTACTAAATAACAAACACTC'			II	
-0.102668	cg0901241	cg0901241	47722454	TTACTATTTCTTCAATTTCATA'			II	
-0.18862	cg0901480	cg0901480	46749397	ATATCATTTCCTACCTTATTAA			II	
-0.10277	cg0901523	cg0901523	65740305	AAAATTCCAAAAAACTCTCTC			II	
0.127443	cg0901792	cg0901792	40738312	CAAAAA	63803425	CAAAAA	I	C
-0.139267	cg0902517	cg0902517	52707318	TAACTACATAAACTCCACCTA(			II	
0.1056518	cg0902990	cg0902990	43605483	ATATCACTAATTCTTTCCACAA			II	
0.1006087	cg0903956	cg0903956	31788448	TCCTACCAACACRATTCAATA			II	
0.1855282	cg0904099	cg0904099	16652505	AATATTCTATAATTCTTTTAAA			II	
-0.102937	cg0904773	cg0904773	18789360	ACTATCC	20681432	ACTATCC	I	A
-0.161599	cg0905083	cg0905083	39669307	TCAATACRACTATAACATTAA			II	
0.2063588	cg0905134	cg0905134	70723444	ACCTCTATAATTCATTTTACAA			II	
0.370756	cg0905218	cg0905218	30675395	AATAATACTAAATCATTTAAC			II	
-0.102405	cg0905324	cg0905324	48664509	TATTTATTTTTAATTAACAAAA			II	
0.1043721	cg0905353(	cg0905353(	13687487	CCCAAAAAATACTCCAAAAAA			II	
0.1066536	cg0906562	cg0906562	56721316	ACAACAA	48793480	ACGACAA	I	C
0.144288	cg0906944(	cg0906944(	23607486	ACAAAAA	61723462	ACAAAAA	I	C
0.1077915	cg0907019	cg0907019	69715359	AACAAAAAATCCTCACAATAA			II	
-0.231272	cg0907334	cg0907334	62617399	AAAAAACATCCCCRCAATAAA			II	
-0.116846	cg0907555	cg0907555	44691391	TCAAACA	55726507	TCGAACA	I	C
-0.117508	cg0907584	cg0907584	15775384	ATCCAAATCCRAATAAACAAA			II	
0.1151825	cg0907988	cg0907988	29693349	AATTTTTCTTTTTTCCTACAAA			II	
0.3147087	cg0908078	cg0908078	25733472	ATTACCAAAATTCTAAATTCTA			II	
0.2229514	cg0908251	cg0908251	65781502	CCCTAAATTAACCTACCTATTT			II	
0.171411	cg0908424	cg0908424	53720344	CAAACTAACCACRTTTCAACR(			II	
-0.111257	cg0908439	cg0908439	26694311	ATAAATT	30630454	ATAAATT	I	C
0.1285828	cg0908815	cg0908815	18651434	ATCCAAAACCCTAATTAAAAA'			II	
-0.152467	cg0909118	cg0909118	45778436	CTTCTTA(	58766345	CTTCTTA(	I	A
-0.123889	cg0910027	cg0910027	65753302	ATAACAACAAATTATAACCCA			II	
-0.17846	cg0910033	cg0910033	34641304	AACCCAA	16627472	AACCCAA	I	C
0.1878902	cg0910106	cg0910106	50699371	AAACACTACACAAATTCCTTA'			II	
0.1558045	cg0910203(	cg0910203(	50621483	ATAAAACTATCATAACCTAAT(			II	
-0.125603	cg0910402	cg0910402	44752306	AAACCCCRTCTCCACCAAAAA			II	
0.106927	cg0910743	cg0910743	71729464	CAAAATA	43641419	CAAAATA	I	C
0.1522961	cg0911500	cg0911500	43694400	TTTATCTTATCTCCACTTTCAA			II	
-0.142534	cg0911744	cg0911744	10664468	AATACAA	25779417	AATACGA	I	A
0.1017227	cg0911968	cg0911968	14679432	TTCCCTCTCCTCCTTTCCAAAT			II	
0.1915237	cg0912627	cg0912627	46810317	TAAAAATATAATTAAAACAAA			II	
0.1302243	cg0912906	cg0912906	63697332	AAAAAAA	24746334	AAAAAAA	I	A
-0.238686	cg0913565(	cg0913565(	31614324	CCTACAC	10734365	CCTACAC	I	T
0.1718723	cg0913820	cg0913820	34645343	ATATAAACATTAAAAACACTA			II	
0.1056964	cg0914027	cg0914027	72623414	ATAATAAAATACAAAACCAAA			II	
0.119539	cg0914706	cg0914706	14689356	AAACCCTTCTCCTCCTAATTTT			II	

0.1897153	cg09148853	cg09148853	49790464	TCTCCAAAACCTAAAAACTAA			II	
-0.291457	cg09158015	cg09158015	33693506	TTTATAATTACTCTTTTCTATTA			II	
-0.10274	cg09163005	cg09163005	61695508	ATAATACRATATATTATATAA			II	
0.1425447	cg09166410	cg09166410	46645488	TTCCTATAACATCTAATATCAA			II	
-0.106381	cg09166536	cg09166536	23692396	CRCCAAATCCCCCAAAACCCC			II	
0.2342328	cg09168728	cg09168728	39751484	TTAAAAAATCCCTTCACCATTA			II	
0.1007187	cg09174817	cg09174817	34733453	ACCRAAAAACTTTATTTAAAA			II	
0.1912537	cg09182085	cg09182085	37645409	CRTAAATCACACTAAATAAAT			II	
-0.113946	cg09187505	cg09187505	18689460	AAAAACC	24679402	AAAAACC	I	A
0.1449839	cg09208740	cg09208740	38683434	TAATAAT	42741493	TAATAAT	I	T
-0.227203	cg09214175	cg09214175	21745343	ATACCCRTAAAAACTACTACR			II	
0.1083154	cg09222367	cg09222367	29764455	ACCATAAACTTAAAAAAATCT			II	
0.23688	cg09222892	cg09222892	35723454	TTATTTTATTCAAACCTCTAAA			II	
0.1769235	cg09225373	cg09225373	29799387	TATTTCACTCAACCTACCAATT			II	
-0.148582	cg09227665	cg09227665	14777384	AATCAAAACATAATTAAAAAC			II	
-0.102688	cg09231171	cg09231171	55751311	CRCCCTTCAATAATTAATAACT			II	
0.1041372	cg09234454	cg09234454	34761355	CACAAAT	47705509	CGCAAAT	I	C
-0.12519	cg09235562	cg09235562	57682351	TAACAAC	15675506	TAACAAC	I	A
0.1029178	cg09239705	cg09239705	50684442	TATTTCCCRAATCCTACAAAA			II	
-0.115011	cg09245003	cg09245003	53729338	CRAACCTATTCTTTCTAAAAA			II	
0.1418293	cg09247979	cg09247979	28668413	TCAAATTTTTAAAATTATAAA			II	
0.1112967	cg09258877	cg09258877	14635367	TCTTAACCCTCRTTTTAACAAT			II	
0.2149233	cg09261791	cg09261791	39749313	ACAACACAAAACTCTCTTAAC			II	
0.1422382	cg09266144	cg09266144	54763332	ACAACCATAAAAACACTAATA			II	
0.1323276	cg09268718	cg09268718	66806458	ACAAACA	13759348	ACGAACA	I	A
	cg09270866	cg09270866	67639301	TACTTACRACTATAAAACAAC			II	
0.1407489	cg09278695	cg09278695	52623414	AAACATTCATTCAAAAAACAT			II	
0.2925184	cg09279736	cg09279736	20706454	ACTTAAA	15790347	GCTTAAA	I	C
-0.169038	cg09284209	cg09284209	49729377	CCTCCRTAACCAACRACTTCTA			II	
-0.14169	cg09284949	cg09284949	32618445	AACCTAT	50615413	GACCTAT	I	A
0.1013712	cg09286880	cg09286880	73669337	AACCTACTCCTAAATATAATA			II	
0.1035592	cg09292660	cg09292660	47722311	CRCTAAACTAAAATATTCTACT			II	
-0.105116	cg09294156	cg09294156	14668374	TCACAAA	35739312	TCGCGAA	I	T
0.1030203	cg09294246	cg09294246	21626310	ATAACTACAAAAAAATCACTA			II	
0.1512091	cg09296453	cg09296453	49654360	CCACAAT	35808326	CCGCGAT	I	T
0.1003786	cg09301498	cg09301498	49688431	CCAAAAAACCTTCCAATACCT			II	
-0.127356	cg09307193	cg09307193	26614375	AAACTCATCTTTATAATACTAC			II	
-0.102492	cg09309269	cg09309269	14719371	AATAATATTTCACTTCATCACR			II	
0.2464393	cg09312897	cg09312897	52654394	CAAATACCAAACATACAATAA			II	
0.1554443	cg09317676	cg09317676	46637510	ATACATACATAAAATACAAAC			II	
-0.106427	cg09322259	cg09322259	56681355	CTAAACC	27673401	CTAAACC	I	T
	cg09323400	cg09323400	54606301	AATAAAAACCCCCAAATAACT			II	
-0.173946	cg09323727	cg09323727	42679433	TTTAACA	10738438	TTTAACA	I	A

-0.111323	cg0932411(	cg0932411(	55750413	AAAAAATTAAAAACTAAAAAC			II	
0.1024269	cg0934961:	cg0934961:	47637471	AACTAAAAACCCTAAAACTTC			II	
-0.124629	cg0935089:	cg0935089:	67715351	AACAAAA	54602303	AACAAAA	I	A
0.1433424	cg0935233\	cg0935233\	36766335	CCCAACTTAAAAACRACACAT			II	
-0.200535	cg0935251\	cg0935251\	49644447	CTAAACCCTAACCTAAAATCC			II	
0.1768826	cg0935455:	cg0935455:	24636339	ATTCACC	62621337	ATTCGCC	I	T
0.1218201	cg0935776l	cg0935776l	61605474	AAACCTAAAATACRACCTAAT			II	
-0.125447	cg0935911	cg0935911	46760494	CTAATAA	24619494	CTAATAA	I	A
0.1361258	cg0936004l	cg0936004l	50648414	AAATTTAAATAACTCACTTATT			II	
-0.192582	cg0936149:	cg0936149:	64613328	CAAAAAC	49665304	CGAAAAC	I	A
-0.1443	cg0936196(	cg0936196(	16705402	ACCAAAT	23736493	ACCGAAT	I	C
0.1731533	cg0936651!	cg0936651!	38653371	ATAACCAAACTCATAACTATA			II	
0.1047068	cg0937351l	cg0937351l	45691334	ATAAAACTACAATCTCAATAT			II	
-0.113614	cg0937506:	cg0937506:	51652388	ACACTTTTCTACAACTACAAA			II	
0.1918885	cg0937828	cg0937828	47684491	TCCTTACAACTCTAACAACCA			II	
0.1050507	cg0938006!	cg0938006!	55661463	TACTAAAATAACTAACTACRA			II	
-0.156585	cg0938748(	cg0938748(	42803315	ACTACAA	72613508	ACTACGA	I	A
-0.108945	cg0939291l	cg0939291l	27725506	CTAATAATATCCAACCCATAT			II	
0.1258323	cg0939519:	cg0939519:	68777485	AAATAAATTATAAAAATAATT			II	
0.1113785	cg0939943!	cg0939943!	37793317	CAAAACAACTACCAATATACT			II	
	cg0940621(	cg0940621(	73790463	ACACCTACTATTTAATTAAATT			II	
0.1121449	cg0940819:	cg0940819:	20795464	CCTTTAAAAACTTTCRTATTAC			II	
0.1848133	cg0941051:	cg0941051:	48782467	TACACCATCTTATAACTCATTT			II	
0.2123933	cg0941191(	cg0941191(	53774381	RAAACTCACATATCAAAATAC			II	
0.1400401	cg0941325:	cg0941325:	20802377	TATTTTTATTTTTACACCATAC			II	
-0.112517	cg0941394(	cg0941394(	35711450	ATTACATAAAAAATATCTTCT			II	
0.1136482	cg0941494\	cg0941494\	63742393	AAACTAA	46678324	AAACTAA	I	A
0.2355631	cg0941618\	cg0941618\	13620337	TAAAACATAAAAAACCAACAC			II	
-0.18109	cg0941703\	cg0941703\	69626417	CRCTTAATCAAAAAAATCCAA			II	
0.1056146	cg0941925l	cg0941925l	61797307	ACAACACAAACTACAATTTTA			II	
-0.122827	cg0941967(	cg0941967(	15624446	CCTTCRTCAAACTCCTAAAAA			II	
-0.112466	cg0942516	cg0942516	70661382	ATACCTAAAAAACCTCACAAA			II	
0.1489477	cg0942701(	cg0942701(	14785328	ACRTACCTAACTAAATACTAA			II	
-0.131201	cg0943064:	cg0943064:	69676349	CCAACAACCAAATAAATAAAA			II	
0.1258297	cg0943460:	cg0943460:	38809442	ATAAATCTATAAAACAAACCC			II	
0.1105408	cg0943517(	cg0943517(	59669343	ATAAATCATAATACCAAAAAT			II	
0.1079409	cg0943747!	cg0943747!	22650352	AAACAAC	45648320	AAACAAC	I	A
-0.166979	cg0943806!	cg0943806!	12618375	TTTCTATCCCCTATTAAAACTC			II	
0.3637553	cg0944245:	cg0944245:	27751347	CTCATCT	69745434	CTCGTCT	I	A
0.1110397	cg0944279:	cg0944279:	22708472	AACTCCCACAATATTTCCACTA			II	
0.1278058	cg0945121:	cg0945121:	17618421	ATAAATATTCAAAAAACCCAA			II	
0.145706	cg0945588l	cg0945588l	50789347	TAACTTTAATACACATAAAAA			II	
0.2738467	cg0946228l	cg0946228l	35725439	ACAACTC	46723333	ACAACTC	I	T

0.1681129	cg09467607	cg09467607	22690379	TACCAACCAAAATAACAACCA			II	
0.1252302	cg09478002	cg09478002	17627420	AACTATATTTAACCAACTATAT			II	
0.1279322	cg09487575	cg09487575	43673449	AAAAACCCAAATATTAAACCT			II	
-0.105943	cg09491897	cg09491897	52670370	AACCTAA	62692490	AACCTAA	I	T
0.1055395	cg09496634	cg09496634	23706474	AAAAATCTACAAATAAAATTC			II	
0.1855773	cg09501687	cg09501687	31735424	CACTCATTCTAACTTTAATATT			II	
-0.143244	cg09508496	cg09508496	10692335	TAAAATC	13645385	TAAAATC	I	C
0.1050662	cg09510698	cg09510698	21692351	TCTATACTCACCACTCCRATAT			II	
0.1060594	cg09516200	cg09516200	19782308	TAAAATAAAACAAACACTACA			II	
0.117517	cg09524613	cg09524613	48745352	AAACATTATTTCTAAAATCTA			II	
0.1353014	cg09532840	cg09532840	50750421	TATATTTTTCTCTTTAAAAATC			II	
-0.132484	cg09533869	cg09533869	19733415	TAACTCTATTTATCTACTACTC			II	
0.2370419	cg09534232	cg09534232	66770392	CTAATCTTACTCTTCRAATTAA			II	
0.1267743	cg09535226	cg09535226	32765492	AATCCATAAAACATCTTCTCTA			II	
-0.188434	cg09560078	cg09560078	22606468	TCCCCTA	13648395	TCCCCTA	I	A
0.1544196	cg09560533	cg09560533	57647300	AAATAAAAACTAATAAAACCC			II	
-0.107148	cg09576209	cg09576209	74797377	CTCAAATTCTACCCTCAACTTT			II	
-0.120502	cg09581911	cg09581911	74718307	CTACTACTTACTAACTACAAAC			II	
0.112415	cg09583302	cg09583302	46605357	ATCATACTTACCTCTAAACCTA			II	
0.1052741	cg09584711	cg09584711	41796336	AACATTACCCCTATTAAAAATT			II	
0.1269924	cg09592244	cg09592244	53629317	TAAATAAAAAATACAAACRAC			II	
0.1238832	cg09597022	cg09597022	10787338	TCTTCCC	42678415	TCTTCCC	I	A
-0.10694	cg09597400	cg09597400	23622386	TACATAA	31801457	TACGTAA	I	A
0.2341724	cg09603707	cg09603707	68689333	TAATACTACTAAAAAAAACTTC			II	
0.1684289	cg09603919	cg09603919	51701470	AAAATTAACCAACTCRTACCA			II	
0.1502718	cg09611620	cg09611620	29737300	CATAACTAATCTTTACTATTAA			II	
-0.300237	cg09616536	cg09616536	50800424	CCCCTAATCAATTATCAATTTT			II	
0.2638652	cg09619488	cg09619488	13684495	TAACAAA	48642443	TAACAAA	I	A
-0.17091	cg09619624	cg09619624	68746346	AAACAAC	37790478	GAACAAC	I	A
-0.142451	cg09623286	cg09623286	56608399	CATACCT	65619429	CGTACCT	I	C
0.1273669	cg09630417	cg09630417	40758440	AATAATAATACAATAATAAAA			II	
0.1096784	cg09630706	cg09630706	20687478	AATAAAC	18721434	AATAAAC	I	A
0.1461623	cg09632136	cg09632136	35642329	AATTAACACAAAACCAATAAT			II	
-0.29128	cg09636756	cg09636756	53767385	ATAATAAAACCCATATCAAAA			II	
-0.100863	cg09639931	cg09639931	31687307	AAAAATT	58640502	AAAAATT	I	C
-0.11209	cg09640425	cg09640425	21705366	AACAAAA	40779491	AACAAAA	I	A
0.3095427	cg09644806	cg09644806	51682447	TATATTAAAATTCACCATATAT			II	
0.1480746	cg09648454	cg09648454	12751316	AAAAAAATAACTAAACCATTT			II	
0.1381975	cg09650180	cg09650180	74722344	CTTCAAAAAAACTAAACCTCA			II	
-0.185089	cg09650895	cg09650895	54737362	AAAAATTTAACAACTTTAAAA			II	
0.1217184	cg09653371	cg09653371	18653342	ACAAAACACACTATTTCAACC			II	
-0.151147	cg09664314	cg09664314	31701446	TCCCTCTAAAATCAACTTTATC			II	
0.1088087	cg09665351	cg09665351	48780377	ACTCRAATCCTACTCTTAAAA			II	

-0.108115	cg09666011	cg09666011	60688471	AAACTAC	74774454	AAACTAC	I	C
0.3811402	cg09672255	cg09672255	62646475	AAAAATAAACATAACATTTTC			II	
0.1468589	cg09680007	cg09680007	59639463	TAAATCACTAAACTAACATAA			II	
0.1075003	cg09714852	cg09714852	52742438	TTCACATTAAAACRACAAAAC			II	
0.1136999	cg09715009	cg09715009	49694411	TAAAACCRATTTTATCCAAAT			II	
0.1000873	cg09715545	cg09715545	66620425	AAAACCTATCCTAAAATTAAC			II	
0.1047387	cg09717927	cg09717927	48701360	AAAAAAACTAAAAACCTAAAC			II	
-0.178035	cg09719342	cg09719342	56680359	CTTTACAAAACATTAACAAAA			II	
0.1047698	cg09723635	cg09723635	18749365	TTCCAAACTAACACTAATAAC			II	
-0.199146	cg09727206	cg09727206	37740333	CCAACTA	52756471	CCAACTA	I	C
-0.139449	cg09727210	cg09727210	65647467	AACAAAA	35657353	GACGAAA	I	C
0.1002712	cg09729274	cg09729274	57748486	AAAAAATTATCTATATAACAT			II	
0.1047001	cg09729624	cg09729624	12741436	AAACTCACRACAAATACAAAA			II	
-0.103183	cg09731946	cg09731946	64723470	TTTAAATAAAATCTATTACTTC			II	
0.1242436	cg09740450	cg09740450	64711383	TTCATACCTAAACAACCTCTCR			II	
0.1042643	cg09755784	cg09755784	69641461	TACTACATTCCCTATATATACT			II	
-0.124601	cg09760728	cg09760728	66609391	TCAATACACCCCTAACAAATTA			II	
0.1177624	cg09763439	cg09763439	64613357	TTAAAAAAAATAATCTCTCTTA			II	
0.2520639	cg09768983	cg09768983	34706422	AACAAAACTAAAAACATTTAA			II	
0.1024966	cg09773458	cg09773458	29730503	TATACAAACCATAACTTCCTCR			II	
-0.105217	cg09775533	cg09775533	33713314	AAAAAACCTAACTTAAACTAA			II	
-0.130241	cg09777775	cg09777775	26638501	AAAACAA	52687504	AAAACAA	I	A
-0.128836	cg09777776	cg09777776	22604405	TTCAACCTAAAATATAATCACA			II	
0.1095865	cg09779576	cg09779576	40607380	CATAAACTCATAAAATAAAAT			II	
0.1062591	cg09782560	cg09782560	32622429	ATAAACCCCAATAAATACTCTA			II	
0.1934865	cg09785377	cg09785377	41742374	ATAATATAACTATTCCACRACA			II	
-0.120258	cg09786098	cg09786098	67720469	AACATAA	32798444	AACGTAA	I	C
0.1857376	cg09790226	cg09790226	62741461	CTAAATCATCCACAACCCTCA			II	
0.1299784	cg09795809	cg09795809	70611337	TTAATAC	46645328	TTAATAC	I	C
-0.117625	cg09797202	cg09797202	70668390	TTCACTAACAACTTAAAAAATA			II	
0.1227471	cg09802066	cg09802066	33648424	AAATAAAAAAAATCCCTAAAC			II	
0.1048242	cg09804649	cg09804649	21670387	CACTATATAAAACTCTATTTAT			II	
0.1450757	cg09807817	cg09807817	21771379	AACTACTCCTTCAAAAAAACT			II	
-0.155251	cg09810089	cg09810089	39664424	TTAAACA	26752459	TTAAACG	I	C
0.1175562	cg09811510	cg09811510	18684453	TATAATCTCATTATTAACCATC			II	
0.1156345	cg09814029	cg09814029	45712411	ACCRTAACAAAAAAACCTTTA			II	
-0.137597	cg09829645	cg09829645	34701417	CTAAACACACTTACACRTAAA			II	
0.1203421	cg09829904	cg09829904	70657476	AAAAAAATATATTAACCTCTTA			II	
0.1063926	cg09830083	cg09830083	20631334	CTTAAAAAAAATCTAAAACTAA			II	
0.116988	cg09831875	cg09831875	38750445	AAAAATA	57622344	AAAAATA	I	A
0.1166693	cg09832276	cg09832276	21697348	TATATAAATATTTTCTCATACT			II	
0.1224561	cg09834172	cg09834172	61602344	AACATAACAACACCAAATAAC			II	
-0.12616	cg09834465	cg09834465	31765305	RCRTTTAAATAACTAAAAATTA			II	

0.1112198	cg0983764{	cg0983764{	20708437	ACTAAATCAAAAAACAACAAC			II	
0.1036366	cg0984047:	cg0984047:	58662317	TTAAAATCCCCAAACCAAATC'			II	
0.2698671	cg0984736{	cg0984736{	14607396	RAATAACATCCAAAACTTACR.			II	
0.1201304	cg0985291{	cg0985291{	44761470	CCACAACATAAAACCCCACCC'			II	
-0.187706	cg0985462(	cg0985462(	39768378	AACCCCCAATAAATTCTACTA/			II	
0.1270582	cg0985609:	cg0985609:	69773355	TTACTTC'	50633420	TTACTTC'	I	T
0.2440719	cg0986341:	cg0986341:	70614430	ATTAACCTAATTTTTCATCTAA			II	
0.2423653	cg0986614:	cg0986614:	29794391	RTAACATCTCCRAAAAACTCA'			II	
0.1068005	cg0986732:	cg0986732:	73678369	CTCAAAACAATAACTTCAAAT.			II	
-0.117191	cg0986745:	cg0986745:	59609402	TTTAAAACCAAAAACTAACAA			II	
0.1368991	cg0987433:	cg0987433:	20609458	CACCATAACRTATAACTACTC(			II	
-0.100572	cg0988328(	cg0988328(	41728417	CAATAAC	13794301	CAATAAC	I	A
0.1320235	cg0988441(	cg0988441(	61781459	ACCCAATCTCTACCCTCATCTT			II	
-0.113605	cg0988445	cg0988445	51639506	AACTAAAAAACRAACAATAAA			II	
0.1845168	cg0988550:	cg0988550:	37733313	TCACCAA	33735343	TCACCGA	I	C
0.1029465	cg0988790(	cg0988790(	57657397	ACTTCCRAAAATAAATTATAT]			II	
0.2020824	cg0989532:	cg0989532:	38715470	AATCAAA	39628431	AATCAAA	I	A
-0.121791	cg0989737²	cg0989737²	56749413	AAACAA/	70790434	AAACGA/	I	C
0.1005902	cg0989898{	cg0989898{	20754477	TACAAATATATAAAACTTATC]			II	
0.1280246	cg0990345:	cg0990345:	12791479	AAAACATCCTCTCTATCTTTAA			II	
0.1434277	cg0990692{	cg0990692{	40799410	ACTAACCAAAACAATAAACAC			II	
-0.195874	cg0990804:	cg0990804:	51631444	TCCAAAATATATCTAACTATA/			II	
0.1052369	cg0991665	cg0991665	63625400	CTCAAAACCATTAACCCCCACI			II	
-0.43302	cg0991684(	cg0991684(	25639320	CAAAAACTATCTATCCATCAA/			II	
0.2020301	cg0992395²	cg0992395²	14773345	AACCAACCTAACCTATATAAT/			II	
0.1114243	cg0992725	cg0992725	36675362	ACTAACT	65676467	ACTAACT	I	C
0.1679007	cg0992731	cg0992731	58759457	ATTCTAATACTAAATAAATAT]			II	
-0.130258	cg0993345{	cg0993345{	50635407	TTAATTA'	30770374	TTAATTA'	I	A
0.107947	cg0995786²	cg0995786²	64723399	CTATTAT.	46800423	CTATTAT.	I	A
-0.229176	cg0996308(	cg0996308(	64809446	CCAAACACTTACTAAAAATAT'			II	
0.112569	cg0997260'	cg0997260'	36629427	TTTATCACAACAATAAAAAAC'			II	
0.1162596	cg0998143:	cg0998143:	25773436	TAAACAAAAAATTTATTCACT/			II	
-0.168452	cg0998192(	cg0998192(	29649484	AACCAAC	74797315	AACCGAC	I	T
-0.10054	cg0998294:	cg0998294:	49748412	ACCTATA	18605351	GCCTATA	I	A
-0.133833	cg0998526(	cg0998526(	32797451	AAAAACRATAAAATCTACAAA			II	
0.1080013	cg0998903'	cg0998903'	24657327	ATAAATACCTTAAAAAAACTA			II	
0.1183519	cg0999019²	cg0999019²	65605381	CTAAAAAACTACAAACTACAT.			II	
-0.394509	cg0999331(	cg0999331(	14642304	TAAACTCTAAAATCRTATTCTA			II	
0.1045909	cg0999754(	cg0999754(	45748362	AACATAT	57671390	AACGTAT	I	C
0.1413697	cg0999815	cg0999815	66795482	TAACRCTAACATCCTAATAAA.			II	
-0.12497	cg0999847²	cg0999847²	39694500	AACTTAC	12708434	AACTTAC	I	A
0.1272727	cg1001762(	cg1001762(	46734487	ATCAAAAATCCAACATTACCA.			II	
-0.166318	cg1002187{	cg1002187{	53774376	ACCCAAC	63605419	ACCCGAC	I	C

0.1104871	cg10028823	cg10028823	67770391	TACAAAC	12704501	TACAAAC	I	A
-0.1468	cg10044179	cg10044179	20628397	ACAACAA	39648458	ACGACGA	I	A
-0.101482	cg10051493	cg10051493	50641429	ATACACTCACTATAAAATTACC			II	
0.1331112	cg10051588	cg10051588	36644327	ATAAATCATATTTAACTCCTCA			II	
-0.104157	cg10056482	cg10056482	42642363	AAACCCA	44755425	AAACCCA	I	A
-0.200788	cg10058204	cg10058204	46658359	ATCAAAATCTTCATTTTTAACT			II	
0.1232483	cg10063575	cg10063575	36606308	TCCTTCATAAATCACCTTATCT			II	
0.2161173	cg10064922	cg10064922	53768307	CACTAAACTTACTAATTTCATA			II	
-0.144492	cg10067510	cg10067510	64604404	AACATAC	21764398	AACGTAC	I	T
0.102703	cg10067523	cg10067523	74698434	CTAATCTTTACACTTTAACTCC			II	
0.1163941	cg10068464	cg10068464	41718349	AAAAAAAAACTTCTATTTTATA			II	
-0.21313	cg10070328	cg10070328	25698303	AAAATAAAAACTTAAAAACAT			II	
0.1034131	cg10072319	cg10072319	74723489	ATTTAAATCATTTCTCTCAATC			II	
0.1294481	cg10076068	cg10076068	10770329	CCRAAACCCTTAATATAAAAC			II	
0.1725504	cg10076560	cg10076560	19623402	CCAATCA	67797490	CCAATCG	I	C
0.1122279	cg10081994	cg10081994	39717404	TACTAATTACTAAAACATTCA			II	
-0.135771	cg10082647	cg10082647	42713510	AAATAAC	12690452	AAATAAC	I	A
0.1459139	cg10085138	cg10085138	51671322	TCTTCTTTAACRTACTTAAAAA			II	
-0.121469	cg10087780	cg10087780	18679499	RTTCTCCATTTACATATATATT			II	
-0.105198	cg10088890	cg10088890	60701395	CAATTATATTAAATCAAACAC			II	
0.1037779	cg10090591	cg10090591	26699355	TCCAATATAATACCTAAAATA			II	
0.1041588	cg10091686	cg10091686	65664331	AATCTCAATAACCRACCCCRA			II	
0.1556825	cg10091792	cg10091792	46644403	RAATAAATACTAATCACCATC			II	
0.1030583	cg10097313	cg10097313	35801339	ACAACTAAACAAACAATAAAT			II	
0.1319336	cg10098373	cg10098373	26730410	ATAACTCCCATAAATAAAATA			II	
-0.266739	cg10099275	cg10099275	36626469	CCTTTTTATTTCTATCTATATC			II	
-0.383151	cg10100585	cg10100585	10701414	AAAAAAAACCCTCCTATAAAA			II	
0.2498184	cg10104252	cg10104252	49750464	AATAAAC	62603505	AATAAAC	I	C
0.1025287	cg10104336	cg10104336	48667441	CTAATACATTAACACTAATAA			II	
0.1079321	cg10104420	cg10104420	65770434	RCRTTCATCCTATAAACACTTC			II	
0.1029766	cg10104674	cg10104674	63686350	AACACAAATTAAAATTTCAAC			II	
0.131926	cg10109635	cg10109635	66763415	CTCCTAAACCAAAACCCCTAT			II	
0.1285222	cg10109731	cg10109731	26610493	AAACAAT	57771424	AAACGAT	I	A
0.1366947	cg10110335	cg10110335	56642304	AACATAACAATAACTAAATAA			II	
0.1140518	cg10111629	cg10111629	17632460	TCCCRTATTTCATTTCTACACC			II	
-0.15875	cg10115321	cg10115321	59657306	ACTTATTCTCATATTTTCTAAA			II	
-0.164876	cg10117077	cg10117077	38767335	TAATTATATTAAAAACCAAAA			II	
0.1201427	cg10117599	cg10117599	58659388	TAACTAAATAAAAAACTCTAC			II	
0.10173	cg10127375	cg10127375	33693488	TCTACCTTCAATTAAATCTATA			II	
-0.144892	cg10130374	cg10130374	62620348	AACAAAA	35738443	AACAAAA	I	C
-0.14103	cg10140536	cg10140536	63698418	ATCTTAA	26738315	ATCTTAA	I	C
-0.134988	cg10141715	cg10141715	68626466	AAATACCCAAAACAATAACRA			II	
0.1296206	cg10143883	cg10143883	20708490	ATATAAAACCACAAACCTATA			II	

0.2345867	cg10144473	cg10144473	65705415	AATAACCCTTAAAAAAACCRA			II	
0.1508658	cg10144863	cg10144863	23692465	CAAAAAAACAAAACTCAATAT			II	
0.3637597	cg10146330	cg10146330	74684498	TCCATAC	33624416	TCCATAC	I	T
-0.123377	cg10150902	cg10150902	71758432	AAAACTTCATCTCTACAAAAA			II	
0.1452649	cg10152997	cg10152997	46736327	AAAACATAACTTAAATCATTC			II	
0.3932671	cg10166986	cg10166986	67703338	TACTTAATCCACATCCTAAAC			II	
0.1227044	cg10167233	cg10167233	62695354	TACTATATACCAACAAACCTAC			II	
0.1894067	cg10167378	cg10167378	13650306	AAACTAA	66699494	AAACTAA	I	A
-0.237407	cg10167978	cg10167978	65742433	TAAAATAACAAACTTTCCCACT			II	
0.2035786	cg10177987	cg10177987	71804313	TATACCTAAAAAATCCCCATA			II	
0.1097399	cg10180092	cg10180092	18728379	TCCATTAAAAATACATCATATC			II	
-0.117583	cg10184328	cg10184328	37717325	AATTAAACRCCCRCCTACTAA			II	
-0.110748	cg10193721	cg10193721	28779428	AAAAACA	15772359	AAAAACC	I	T
-0.147244	cg10197336	cg10197336	24672333	AAAATTAACACCAAAAAACTA			II	
-0.13875	cg10200291	cg10200291	43600311	ACCACAT	32739338	ACCGCGT	I	A
-0.129603	cg10200373	cg10200373	33809357	CCAAAAACCTCRTATCTCTTAC			II	
-0.14494	cg10202544	cg10202544	64646353	AAAAATTAACCTAACATACTA			II	
0.2167687	cg10202835	cg10202835	41624389	AAACATA	21791419	AAACGTA	I	C
-0.186633	cg10205431	cg10205431	14745467	ACAATCT	57800340	GCGATCT	I	C
0.1004167	cg10208777	cg10208777	40616479	CTATTTAATAATTACTTCTTCT			II	
-0.113237	cg10213302	cg10213302	58784432	CCTACAA	72762391	CCTACAA	I	A
0.1383818	cg10213658	cg10213658	68644458	TTATCTCCATTTCATATAATCT			II	
-0.137836	cg10213875	cg10213875	36762446	AATCACC	71698444	AATCACC	I	A
0.3996179	cg10217052	cg10217052	62734347	RAAAACCAAAATCAATCATAA			II	
0.1030939	cg10220544	cg10220544	68744315	TCRACCACTATAAAAAATTTC			II	
0.1101362	cg10221014	cg10221014	48768314	AAAAATATAAACCAAATATAC			II	
0.2113787	cg10224537	cg10224537	48788306	TTATAACAACTACCCACATTTA			II	
0.1817957	cg10225951	cg10225951	63694309	RAACCAAACRAAAAACTAACC			II	
0.1151281	cg10234477	cg10234477	34641380	CCTTTTTTCTCCCTCTCCCTCAC			II	
-0.131968	cg10235453	cg10235453	72747457	AAAAATAAAACTAACCCAAAA			II	
0.1383975	cg10240356	cg10240356	61792426	AACCAAAACCTTTTCTATCRA			II	
0.1113501	cg10241183	cg10241183	58737492	AATAATACAAATCTAATTTCA			II	
-0.111123	cg10243348	cg10243348	44789485	CTCCTAA	58730396	CTCCTAA	I	A
0.1240368	cg10243855	cg10243855	18793443	CCAATATTTACRTTCTAAACAC			II	
0.1837347	cg10249074	cg10249074	54753406	CRTAATACTAATCTATAAAAC			II	
0.156692	cg10252249	cg10252249	51767492	AACCCTT	47795418	AACCCTT	I	A
0.1161131	cg10262052	cg10262052	73673357	TTACTCACACRAACTATTTCTC			II	
-0.576526	cg10263003	cg10263003	16672373	CTCACAAACCTAAAATAAACA			II	
-0.138352	cg10265387	cg10265387	20622426	AATCCCA	14759462	AATCCCG	I	A
0.1495948	cg10266681	cg10266681	48690416	CAAAATTTCTAACATTCCTATA			II	
0.1008951	cg10288525	cg10288525	36721481	ATAACTAAACCATACRCRAAC			II	
0.143867	cg10289744	cg10289744	39617333	AATACAAACAACAACCTAACA			II	
-0.143621	cg10291990	cg10291990	69803510	ACCTCAA	47734337	GCCTCGA	I	C

-0.227292	cg10292584	cg10292584	16687334	TTAAAAA	39646344	TTAAAAA	I	T
0.1195452	cg10293824	cg10293824	58769488	AACTTAAAACTCRCTCAACCTA			II	
-0.147577	cg10295955	cg10295955	55774489	TCCTCAACACTCRTATATCRCC			II	
-0.113893	cg10297211	cg10297211	31682503	AATCCCA	57660444	AATCCCG	I	A
0.2102591	cg10306192	cg10306192	19754448	TCATTTATTAAAACAATACCTA			II	
0.1173892	cg10308629	cg10308629	37725360	TTTCATTCTCTATAATTAACAT			II	
0.1234359	cg10310917	cg10310917	32759433	CACTAAAACAATTACAACCAA			II	
0.1653567	cg10311048	cg10311048	12710436	CCRCCAAACCCACACCCAAAT			II	
0.1333914	cg10312513	cg10312513	48627497	CTATCAC	62620306	CTATCAC	I	T
-0.193192	cg10317314	cg10317314	11754397	ACCTCTA	46702490	ACCTCTA	I	A
0.104106	cg10318368	cg10318368	54669481	TCCAATCACAATTTAAATTTAA			II	
-0.100402	cg10319942	cg10319942	49746468	CAAAAAACCTCAACATACCCA			II	
0.1242	cg10328089	cg10328089	74799317	AAATCCCACAATATTAACTATA			II	
-0.278647	cg10328548	cg10328548	42739345	CCACCAT	42793430	CCACCGT	I	A
-0.162983	cg10329418	cg10329418	17781495	AACTCATCTTCTCCAAAATTTA			II	
0.1089876	cg10333144	cg10333144	72648369	CACTCTTAACTCTAAACACAAT			II	
-0.106651	cg10334741	cg10334741	62616428	ACACAAA	41794445	ACGCGAA	I	A
0.1037076	cg10336025	cg10336025	59765370	TAAAAATAATATAAACRAACA			II	
0.1136383	cg10336193	cg10336193	66689473	ATAACCACACTTCTTCATAATA			II	
0.1392902	cg10338539	cg10338539	32639331	RAATTTAACTTAATCRCCAATT			II	
-0.208797	cg10340904	cg10340904	67667448	TCTATTA	68789383	TCTATTA	I	A
0.1060922	cg10342016	cg10342016	73764451	CAACTCAACCCAATAATAAAC			II	
-0.10866	cg10347293	cg10347293	31743462	TACATCC	36775445	TACGTCC	I	A
-0.118032	cg10350492	cg10350492	28641376	AACAATTCTAACTAAACCTCTA			II	
0.1003797	cg10351284	cg10351284	59704379	CCCATAATATAAACRTATCCRT			II	
0.1309833	cg10352237	cg10352237	31715407	AATAAAAACCRTTTTTATCACR			II	
0.1156596	cg10370305	cg10370305	23744363	AAAACTCACATATATTTACTTT			II	
-0.15888	cg10372770	cg10372770	17666483	TCTCRACTTCTCTAAAAATACT			II	
-0.163347	cg10375597	cg10375597	16737369	TACACCTAAAATAAAAACTAT			II	
0.1483068	cg10384133	cg10384133	38628314	TCCAAACCTATTTCTCTCTCCA			II	
0.3346064	cg10385522	cg10385522	25755461	AACTTATTACAAACATCTCTAC			II	
0.1133963	cg10392572	cg10392572	12774369	TTTTCCCTATAAAACAACATAA			II	
0.1134418	cg10407894	cg10407894	65651440	AATAAAAACTTATAATATCTTA			II	
0.1456971	cg10409680	cg10409680	57807397	ATCCACCTTAAAATAAATCTCA			II	
0.1429234	cg10413089	cg10413089	26784326	AATCTATAACTTCCCAAAATAC			II	
0.1045401	cg10413861	cg10413861	18762412	TAAATTTACCCCTATATAATAA			II	
0.1763147	cg10415021	cg10415021	27769303	CTATACTATCTATATTTAACCT			II	
0.1510989	cg10423607	cg10423607	11618340	TCTATAAATTTAACATTCTTAA			II	
0.130736	cg10423842	cg10423842	61754407	TATCTATTATCCTCATTATTAT			II	
-0.129117	cg10424681	cg10424681	43636484	ATAAAAA	56690452	ATAAAAA	I	A
0.1127488	cg10436792	cg10436792	59662421	CCAAAATAAAAAAACCCTCAA			II	
-0.131583	cg10440578	cg10440578	70725460	AAAAAAA	29631307	AAAAAAA	I	A
-0.108682	cg10449329	cg10449329	28685469	AATAACTATTCCATTTTACTTA			II	

0.1087631	cg1046642l	cg1046642l	37620417	CATAAACTATTATTAAATCTAT			II	
0.1066413	cg1047569(	cg1047569(	69766330	TTTAATAATACACATATTACTT			II	
-0.118876	cg1048107:	cg1048107:	27771430	TAATAACTTAAAATTCTTACTT			II	
0.221998	cg1048251:	cg1048251:	72767486	AAAATAAAAACCCCAAAAAA(			II	
0.2195411	cg1048366(	cg1048366(	52770325	AAAAAAAAATATCCTCATAAT			II	
-0.10442	cg1048467:	cg1048467:	70756443	TACTTAATAATCCTTATCACAT			II	
-0.184535	cg1048606¢	cg1048606¢	26671487	AATACAA	74620448	AATACGA	I	T
0.1931662	cg1048805(	cg1048805(	73621324	AACTCAAAACRAAATCTACTT/			II	
0.124121	cg1049343(	cg1049343(	16743503	TAAACCACTCAACTAAACTCT/			II	
0.1202402	cg1049789⁄	cg1049789⁄	27600436	ACCTAACTACTTATTCTTTTAA			II	
-0.172928	cg1050002(	cg1050002(	42755347	AAATCCC	56698327	AAATCCC	I	A
0.1347331	cg1050070:	cg1050070:	52679452	ACCTATATCTCATAAAAAAAT(			II	
0.1066144	cg1050109:	cg1050109:	58622410	AAACAA/	29711507	AAACAA/	I	T
0.1305093	cg1050121(	cg1050121(	43774458	CAAAACT	66775341	CGAAACT	I	A
-0.164041	cg1050775¢	cg1050775¢	43648365	ACCACTACAAATAAAACCTAA			II	
-0.123221	cg1050796:	cg1050796:	33752434	CTAAAAAACTCACATCRTCCT/			II	
-0.103063	cg1051142¢	cg1051142¢	51782318	CCTTCACCTTATCRCTCAAACA			II	
0.1230623	cg1051332:	cg1051332:	69603337	TAAATCTACCCAAAAAACTCA			II	
0.149954	cg1051399:	cg1051399:	44786356	ATTAAAAATTATACTATCTAA/			II	
0.1001308	cg1051815:	cg1051815:	24682375	CTACCTATACTAAACAATATAT			II	
-0.18134	cg1052842⁄	cg1052842⁄	27765350	TACAAAA	41611371	TACAAAA	I	C
-0.131298	cg1053989{	cg1053989{	35729325	CAAATCT	38628452	CAAATCT	I	A
0.1028004	cg1054184:	cg1054184:	13613324	CTACACTCTCCCATCACAAAA/			II	
-0.158732	cg1054401{	cg1054401{	16767305	AAAAAA/	42611322	AAAAAA/	I	C
0.1401655	cg1054621(	cg1054621(	74613345	ATTATACCACAAATCCCAACT(			II	
0.1088899	cg1054907l	cg1054907l	59810379	CAAAATCCCAACTAAACACCC			II	
-0.111675	cg1055310:	cg1055310:	33767380	AAAATAAAACAAATACTACTT			II	
0.1004191	cg1055320⁄	cg1055320⁄	61671309	TACAAAA	19659376	TACGAAA	I	A
-0.325008	cg1055321¢	cg1055321¢	15624313	TTCAATACTCTAAATAAACTC(			II	
-0.153148	cg1055768:	cg1055768:	47625505	AATACCTCTAAAAAAACCTCT/			II	
0.1046342	cg1055790:	cg1055790:	15629450	ACAATATTATCTTTAAATACC(			II	
0.1305445	cg1055915l	cg1055915l	48602325	CACAAACCAAACTACTCTTAA			II	
0.1422875	cg1056056l	cg1056056l	65732339	TTACTTATTTACCACTAAAAA/			II	
0.1023259	cg1056400l	cg1056400l	41624467	ACTTCTAAAATACAATAAATT(			II	
0.3795986	cg1056806(	cg1056806(	39730413	CCAATTA	41722336	CCAATTA	I	A
0.1007373	cg1057449¢	cg1057449¢	39690462	CTAACTAAAAACCTAATACAA			II	
0.1584056	cg1057624:	cg1057624:	37667504	CCTACATCCAAACTAAAAATT			II	
-0.112606	cg1057877:	cg1057877:	72761345	CCTAAAA	11607503	CCTAAAA	I	C
0.1091034	cg1058667:	cg1058667:	73667307	ATCTAACTATAAAATAACCAA			II	
0.1141389	cg1058718:	cg1058718:	16804357	ATCACCAAAATTACTATAATC(			II	
-0.113401	cg1059341(	cg1059341(	19741306	AAAAACTAAATTTCTAAAACA			II	
-0.124253	cg1059938⁄	cg1059938⁄	17680427	CTCCTCCTAACCTATATATACT			II	
0.1025883	cg1060117l	cg1060117l	16611329	ACCTAACTCAAAATAAACCCT/			II	

-0.179511	cg1060612[illegible]	cg1060612[illegible]	54774472	AACCACA	52617432	AACCACC	I	T
0.1310906	cg1060986[illegible]	cg1060986[illegible]	20806344	CTCCATCCATCTATAAACTATC			II	
-0.199783	cg1061480[illegible]	cg1061480[illegible]	44746361	ATACTAC	45807398	ATACTAC	I	C
-0.113702	cg1061621[illegible]	cg1061621[illegible]	28810412	AAACCAACCCCATACAATAAT			II	
0.1087049	cg1062088[illegible]	cg1062088[illegible]	53719509	CAAAACC	39727434	CGAAACC	I	A
-0.100727	cg1062255[illegible]	cg1062255[illegible]	28629497	CACCCAC	70809301	CGCCCAC	I	C
0.2095342	cg1062616[illegible]	cg1062616[illegible]	26755468	TTAACTTCAACATAAAACAAT			II	
0.1141356	cg1063128[illegible]	cg1063128[illegible]	49739470	TCTCCTT	12756462	TCTCCTT	I	C
0.1033565	cg1063214[illegible]	cg1063214[illegible]	73771431	AAAAAAC	16606388	AAAAAAC	I	A
-0.124267	cg1063265[illegible]	cg1063265[illegible]	45693300	TTAATCCCTAAAAACATACCA			II	
-0.444445	cg1063277[illegible]	cg1063277[illegible]	49764464	TAATCAAAACCTCTAAAACAA			II	
-0.112566	cg1063289[illegible]	cg1063289[illegible]	49752455	AAAACAA	45646503	AAAACAA	I	A
-0.186097	cg1063589[illegible]	cg1063589[illegible]	47783382	AACACAT	19651386	AACGCGT	I	T
0.1030821	cg1064696[illegible]	cg1064696[illegible]	21727497	AAACACACAATACAAAACTTC			II	
0.1041579	cg1065165[illegible]	cg1065165[illegible]	67683365	CTCCACTCAAAATTAACCACC			II	
-0.118296	cg1065555[illegible]	cg1065555[illegible]	74623332	AAAATAA	35659384	AAAATAA	I	A
-0.104748	cg1065820[illegible]	cg1065820[illegible]	19749453	TCCCTCCCTATTATACTCTCCR			II	
-0.190665	cg1066231[illegible]	cg1066231[illegible]	27686326	ATTCTTC	47643473	ATTCTTC	I	A
0.1305032	cg1066430[illegible]	cg1066430[illegible]	60733432	CTCCCACTAAAACCTAAAATT			II	
0.1501645	cg1066634[illegible]	cg1066634[illegible]	71792334	AACTTTCCCATACATACTTAAC			II	
-0.105909	cg1067039[illegible]	cg1067039[illegible]	42685325	AAAAAAACCRATAACCTAAAC			II	
-0.199962	cg1067256[illegible]	cg1067256[illegible]	28763342	ACCTCCT	42751473	ACCTCCT	I	A
0.1258816	cg1067480[illegible]	cg1067480[illegible]	20619509	AATATAA	60731404	AATATAA	I	C
-0.114487	cg1068022[illegible]	cg1068022[illegible]	18770414	TTCAAAA	41604395	TTCAAAA	I	C
0.1849265	cg1068139[illegible]	cg1068139[illegible]	40676346	AACCAAAACCCCAAAAAATCT			II	
-0.161518	cg1068708[illegible]	cg1068708[illegible]	68731469	CACAAAA	64679387	CGCAAAA	I	C
0.1131422	cg1068713[illegible]	cg1068713[illegible]	55799382	AATACCATAAAATTAAACAAC			II	
0.1110565	cg1068940[illegible]	cg1068940[illegible]	62688381	AAATCCA	20720486	AAATCCA	I	A
0.156495	cg1069015[illegible]	cg1069015[illegible]	37773346	AAACAAT	49711327	AAACGAT	I	A
0.1396754	cg1069214[illegible]	cg1069214[illegible]	24663433	CAACCACRTAAAAAATTAAAT			II	
0.1441974	cg1069472[illegible]	cg1069472[illegible]	10773446	AAAAATCAAAAATAAACACAA			II	
-0.100497	cg1069554[illegible]	cg1069554[illegible]	33611395	ATAAATCCCTATATAATATTA			II	
0.1007263	cg1069635[illegible]	cg1069635[illegible]	56781474	CCAATACTTACAAAAACAATA			II	
0.1072986	cg1069917[illegible]	cg1069917[illegible]	26650456	ACTCAAAAAAATATATATTTA			II	
0.1665246	cg1070103[illegible]	cg1070103[illegible]	65615318	TAACCACTAAAATAATCAACA			II	
0.1398813	cg1071247[illegible]	cg1071247[illegible]	43792404	AAAATTCTTCCCAACTTTTAAC			II	
0.107953	cg1071358[illegible]	cg1071358[illegible]	33739367	AAATCTATAACTTCTCAAATTT			II	
-0.117291	cg1071527[illegible]	cg1071527[illegible]	51719344	TAAAAACTTCCCCCCAAAAAA			II	
0.1806626	cg1071979[illegible]	cg1071979[illegible]	70612465	TTACCATATCTACTAATCCTCA			II	
0.1261123	cg1072244[illegible]	cg1072244[illegible]	53683501	TAAATTTAATATCCTATCAAA			II	
-0.123147	cg1072463[illegible]	cg1072463[illegible]	51626331	TTCATACCCAACATCRTTATAC			II	
0.1242212	cg1072534[illegible]	cg1072534[illegible]	26603501	AACACATCTAAAATAACTCAC			II	
0.1265697	cg1072584[illegible]	cg1072584[illegible]	54602367	ATCATTAAACTTACTCATATAA			II	

-0.118891	cg1072766(	cg1072766(	21678464	AAAATATATCTACACATAACC/			II	
-0.130084	cg1072949(	cg1072949(	56800480	ACCCAAA	63799480	ACCCGAA	I	A
-0.11872	cg1073547:	cg1073547:	54683477	AAACTTCTAATTAAAACCTCT/			II	
0.1336279	cg1073560'	cg1073560'	16760467	ATATTCTTTTAACTCTATCRTC(			II	
0.1602075	cg1073630:	cg1073630:	64642498	ACCCAAACTAAACRAAATAAA			II	
-0.125166	cg1073864:	cg1073864:	44601351	AATACATTAAATTCATTCATTC			II	
0.1921972	cg1074141:	cg1074141:	63646479	TTACTTCACATATTATAAAATA			II	
0.1435328	cg1074407:	cg1074407:	37605414	AAAAAAAACCAAAATCTAAA/			II	
0.2355773	cg1074748:	cg1074748:	53606504	TCACAAA	65610464	TCGCGAA	I	A
-0.243986	cg1075026·	cg1075026·	57779501	TATTTATCAATATAAAAAATTC			II	
0.2800673	cg1075030(	cg1075030(	47659388	AATATACTACTTAAAATAAAA(			II	
0.1272792	cg1075361(	cg1075361(	19627345	CCTAAAAAAAACCRTTAAAAA			II	
0.1664058	cg1075439:	cg1075439:	26699375	ATACTCC	46663315	GTACTCC	I	T
-0.106297	cg1075981'	cg1075981'	14739477	ATAAAAACCAAAAAAACATCC			II	
-0.179104	cg1076163:	cg1076163:	48664460	TATAAAAAAACCCTACCCAAA			II	
-0.107853	cg1076323·	cg1076323·	64765384	TAAACCA	33699316	TAAACCG	I	A
0.2895731	cg1077253:	cg1077253:	66671425	ACCTCCTATCATTTCTTCATAA			II	
0.1345747	cg1077734'	cg1077734'	74689428	TCAACAAAAAATCTTATAATC'			II	
0.1208898	cg1078077:	cg1078077:	24632369	AAAAAAAAATCATCTACCTAA			II	
0.1345381	cg1078220(	cg1078220(	18809487	TATATCTAAAAACCTACCRTCC			II	
0.1926167	cg1078515:	cg1078515:	28625444	ATTCTAACCTCCTTTCCAATAC			II	
-0.219997	cg1079230:	cg1079230:	51612373	AACAAA/	31716425	AACAAA/	I	A
0.1655704	cg1079291:	cg1079291:	14625395	TAACTCTAACTCCTTCCTCATA			II	
0.1017136	cg1079511:	cg1079511:	22779306	CCTCCCA	39773476	CCTCCCA	I	C
0.1029451	cg1080034(	cg1080034(	60620379	CTATTTTCTATTAAAATAACCA			II	
-0.103392	cg1080357'	cg1080357'	57726430	AATTCRAATAAAACTAAAATC(			II	
	cg1080497·	cg1080497·	54606328	AATCAACAAATCAAAATACAA			II	
-0.14915	cg1080919:	cg1080919:	30762401	AACACA/	38713319	AACGCG/	I	T
0.1186809	cg1080928:	cg1080928:	17670482	CAAAAACTTCCCCTCTTACATA			II	
0.1076824	cg1081142(	cg1081142(	47696321	AAACCCCTTAAACCAATCTAA/			II	
-0.215825	cg1081263·	cg1081263·	67759420	AACAACA	70703360	AACGACA	I	C
0.1065843	cg1081288:	cg1081288:	45734474	CTCTTTAAAACTATCTTCTACA			II	
-0.290958	cg1081565'	cg1081565'	53795414	AAAACA/	51795457	AAAACG/	I	A
0.1248022	cg1081567]	cg1081567]	17802397	ATCATTTCCTAACTTATAAATA			II	
-0.11405	cg1082004:	cg1082004:	59617490	AACAACA	60758463	AACAACA	I	A
0.212857	cg1082073(	cg1082073(	54723450	RTAACAACCACAAATACRTAT/			II	
-0.400033	cg1082196·	cg1082196·	48742463	ATATTAAAAAACATTCRCTAA(			II	
0.1082559	cg1082351]	cg1082351]	39601303	ATATTATTTCCTAAAATATTCC			II	
0.1072266	cg1082765(	cg1082765(	41715408	AAACAACACRTACAAAAATCC			II	
-0.123111	cg1083124(	cg1083124(	19744411	ACTAAAT	48735439	ACTAAAT	I	A
-0.178413	cg1083209:	cg1083209:	50693368	TAAATCT	52631312	TAAATCT	I	C
-0.15411	cg1083778:	cg1083778:	26715316	AATAAAT	36715368	AATAAAT	I	C
-0.112762	cg1083972:	cg1083972:	28804403	TTAATAAAAACRCTAATCCCC(			II	

0.1707647	cg10844275	cg10844275	53653457	TATCTAAATTACCACAATCATA			II	
-0.122898	cg10844382	cg10844382	29765465	ATATAACCCCAAACTATCCTAC			II	
	cg10846615	cg10846615	67669371	ACTTCAT	10643503	ACTTCGT	I	A
0.1729279	cg10855427	cg10855427	37707303	CCTTTACCCACRTATAATCTCA			II	
0.2930345	cg10858640	cg10858640	61677417	TCRCAAAACTAAATAACTCAA			II	
0.1177042	cg10862468	cg10862468	52672494	CRAATACTATTACTATAATATT			II	
-0.127642	cg10862535	cg10862535	23757363	TTCTCTAACTAAACCAAAACRA			II	
0.1974615	cg10862960	cg10862960	59766311	AATAAAAACAACTAACCCTAA			II	
0.1803011	cg10863737	cg10863737	74759421	TTATTCCTACCTCCCACRCTCC			II	
0.1189755	cg10874097	cg10874097	47607347	TTTATCATCATATTTTTACATA			II	
0.1232178	cg10878230	cg10878230	55679444	TTTACATCCTCTAAAACCTAAT			II	
	cg10883621	cg10883621	13644317	TTAATCCAATTCTTTAATCCTC			II	
0.1351589	cg10888811	cg10888811	56793416	TACTAAATAATAACAAACACA			II	
-0.184886	cg10890644	cg10890644	66636357	TTAAATAAACTCTCAATAATTC			II	
-0.135468	cg10891570	cg10891570	20679318	AAATTTTCCAAAACTTATATAC			II	
-0.141632	cg10893278	cg10893278	41806353	ATTATCCAAAACAAAATTAAA			II	
-0.133698	cg10895875	cg10895875	18781332	CTAAAAATATTAATACTCCTA			II	
0.1202654	cg10900313	cg10900313	20670427	RTAAACTTTTATACCTATCCTT			II	
0.1845283	cg10902850	cg10902850	42604354	AAATTAATCAAAAAATTCATA			II	
0.1277139	cg10909185	cg10909185	69618411	AATAATA	69736444	AATAATA	I	A
-0.112607	cg10911287	cg10911287	63683505	CAAAACCATATAATCRTATCCT			II	
-0.124539	cg10912974	cg10912974	12672439	CAAAATA	53693413	CAAAATA	I	C
-0.121181	cg10920758	cg10920758	66645436	AAATAACTACCCCCCRAAACT			II	
0.1093745	cg10926851	cg10926851	59711416	AAATTAAAAAATCCCTAAAAA			II	
0.2114435	cg10930308	cg10930308	73636451	CAAACAA	49750505	CAAACGA	I	A
0.1230889	cg10937408	cg10937408	40607452	ATAAAAC	37633345	ATAAAAC	I	A
0.1478886	cg10940203	cg10940203	51614481	AAAAATT	58624406	AAAAATT	I	A
0.1170653	cg10946263	cg10946263	21687351	TATACATTAACATCTCAATTA			II	
0.1078365	cg10953158	cg10953158	22773335	CTCACCTCATAATCTACCTTTA			II	
0.1281908	cg10954330	cg10954330	25645386	ACCACCTTAAAAAATTTCCTA			II	
0.3838305	cg10957001	cg10957001	62702473	TTATTCATTTATTAAAACATTT			II	
-0.134426	cg10967023	cg10967023	13720352	ATAAAAA	28601475	ATAAAAA	I	A
0.1177076	cg10969465	cg10969465	74632373	AACACAA	25717329	AACGCGA	I	C
-0.106323	cg10970349	cg10970349	48671357	ATCRAAAAAATCACCAACAAA			II	
-0.159472	cg10973622	cg10973622	17606321	ATAAAAAATCAATACAAAAAC			II	
-0.206174	cg10974412	cg10974412	44729430	CTAACTCATCAATTCTAACAA			II	
-0.129408	cg10975354	cg10975354	39806406	TAATAAAAAAAAATACTCCCT			II	
-0.104242	cg10978585	cg10978585	71789303	TACACCC	59608377	TACGCCC	I	C
0.1179894	cg10979846	cg10979846	62606363	ATAAAATCAACCTTCACTACA			II	
-0.100567	cg10982045	cg10982045	10766315	TTAAAACTTCTACAAAAACCA			II	
0.1295922	cg10983484	cg10983484	56735443	ACAAACCCATAACAAAATCAT			II	
0.1069726	cg10987840	cg10987840	27796427	TATATATTTTCCCCATCAAAAC			II	
0.1184282	cg10989214	cg10989214	70728324	AAAACCA	53740485	GAAACCC	I	A

-0.274561	cg10989317	cg10989317	24719408	CTAAAATACAACAACTATACA			II	
-0.119398	cg10989981	cg10989981	66708439	CCTACTAATCTACTCRATAACA			II	
-0.105678	cg10990188	cg10990188	18656427	AAAATATATCCRCACCTAATA			II	
-0.226099	cg10990737	cg10990737	42615324	TACCCCTCCCCTTCTTAAAAAA			II	
0.1002041	cg10991855	cg10991855	69672418	CAAAAAAACTAACTAAAAACT			II	
-0.117332	cg10994944	cg10994944	64607349	CATCCTT	66644423	CGTCCTT	I	A
-0.143351	cg10995081	cg10995081	25735316	CACAAAC	13767374	CGCAAAC	I	A
0.1767006	cg10995422	cg10995422	23622307	TAAATCCACACTATCACRATTA			II	
0.1132427	cg10995873	cg10995873	36602457	TATAAACTACAAAAATAAAAT			II	
0.106256	cg10997190	cg10997190	20780397	CAAATACTAAACATAAAATAA			II	
-0.110123	cg11002686	cg11002686	70796444	TACCTAACCTCRTAAAAATATA			II	
-0.109737	cg11002923	cg11002923	28620325	TTCCCTACTCATAATCAAAAA			II	
-0.141276	cg11005628	cg11005628	70706315	AAAAAAC	47670353	AAAAAAC	I	A
-0.103699	cg11005845	cg11005845	30791422	AAACCTC	39669490	AAACCTC	I	A
0.1204076	cg11008123	cg11008123	46645482	ACCAAAA	54708500	ACCGAAA	I	T
0.1793214	cg11010744	cg11010744	21757438	CAACTCTCTCCAATATAACCAC			II	
-0.161261	cg11011512	cg11011512	10722313	AAAATAA	43809366	AAAATAA	I	C
0.217876	cg11012153	cg11012153	72808343	TAAAAAC	33661308	TAAAAAC	I	A
0.1189691	cg11015768	cg11015768	73789466	ACACCTCTCTCTATATATCTTA			II	
0.1009326	cg11016151	cg11016151	72708381	TAAAATTCTAAACTTACATTA			II	
-0.124159	cg11019791	cg11019791	68715414	ACACTAAAAAACTAACTAACA			II	
-0.156622	cg11024449	cg11024449	51660345	CAACTCT	58651460	CAACTCT	I	A
0.1176878	cg11026604	cg11026604	17765356	TTTCTCCTATTTACATACAATT			II	
0.1152308	cg11031567	cg11031567	21750367	ACTCTAACCCAAACCAAAACA			II	
-0.15524	cg11035303	cg11035303	11654432	AATATTACRATACCATTTTATC			II	
-0.250693	cg11036359	cg11036359	58702337	AATACAAAATAACCACTATAT			II	
0.114228	cg11038231	cg11038231	68803380	CCCCTACCTTAAAAATCATAAT			II	
0.1355621	cg11043993	cg11043993	40798374	CCCAACAAATACAACCACACC			II	
-0.208488	cg11044162	cg11044162	68801378	ACCACCCCTAATCAATTATCA			II	
0.1452152	cg11046772	cg11046772	29796509	ACCCTAAATACTTCTATTCAA			II	
0.1367173	cg11047442	cg11047442	36635354	CACAACA	44677485	CACGACC	I	T
0.1604477	cg11048797	cg11048797	33699510	TACCAACTCTTTATAACRAATT			II	
	cg11053062	cg11053062	11802432	ATATAATCTCACTCCACCCAAC			II	
-0.160723	cg11053632	cg11053632	38637330	TAAACTCTAAAAAATCACCTC			II	
-0.133304	cg11059159	cg11059159	12606302	AAATAAAAAACRCAATTAACA			II	
-0.110462	cg11060532	cg11060532	27709499	AAAAAAATTCCATAAAACTAA			II	
0.1192884	cg11063088	cg11063088	17792508	TTTTAATTAACRCAAAACATTC			II	
0.1401693	cg11066244	cg11066244	42649325	TTTTTACATACATTATCTCATT			II	
0.1441264	cg11066601	cg11066601	57787312	AAAATTC	34636361	AAAATTC	I	A
0.1070406	cg11068881	cg11068881	34643333	ACTTTACAACTTTAAATAATAT			II	
0.1252909	cg11073579	cg11073579	60741458	AACAAAATCTAAAAACCTAAT			II	
-0.113799	cg11074353	cg11074353	10705406	TAAATATAAACACCCACRCTA			II	
-0.124682	cg11074783	cg11074783	49661459	AAACAAC	42679464	AAACGAC	I	A

0.1214039	cg11081272	cg11081272	66631343	RAATCCCCTACCACRTACTTAA			II	
-0.136666	cg11082635	cg11082635	27700403	TAACACRTACTAAATATAAAAT			II	
0.1028112	cg11092836	cg11092836	13748326	AAAAACACAAATCCTACAACA			II	
0.1000921	cg11104088	cg11104088	31681490	AACCAAA	62791345	AACCAAA	I	A
-0.150354	cg11108474	cg11108474	10605372	TCTAAAA	42766351	TCTAAAA	I	C
0.1337218	cg11111460	cg11111460	25733438	ACCCAAACTTTAAATAAACTA			II	
0.1049508	cg11116274	cg11116274	71647392	CCTTTTCCRAAATTAAAAACRA			II	
0.1684201	cg11119767	cg11119767	11633490	ATACTAAAAAATCTCCTATTAC			II	
0.2251344	cg11123613	cg11123613	44666354	CATTTAAAATACTAACTACCCA			II	
-0.251892	cg11124135	cg11124135	70793333	AAATAAA	67682493	AAATAAA	I	T
0.1056508	cg11128021	cg11128021	56652322	AAAATAATAATACCAACCTCA			II	
0.1177914	cg11128212	cg11128212	30791331	TTTAAAATATAAATATAATCAT			II	
0.1020517	cg11128956	cg11128956	52764301	ATTAAAACCRTAAATAACCCCA			II	
0.101386	cg11132534	cg11132534	34739436	AAAAACCTCACTATCAACCCTA			II	
-0.133628	cg11133658	cg11133658	35656319	CATCAAAAAAAACCRTAAATA			II	
0.1241814	cg11139248	cg11139248	65602468	AAAACACAATAATTACCCAAC			II	
0.290427	cg11144103	cg11144103	33619359	AAAATCTAAAAACTCTAACCC			II	
0.1052511	cg11147866	cg11147866	21731367	AAACTTTACATAACCAAATATC			II	
0.117977	cg11155432	cg11155432	23722457	AACTCTCTATCTAATTAATTTC			II	
0.1084652	cg11156873	cg11156873	24770432	TTTCAAA	34671340	TTTCAAA	I	T
-0.163977	cg11166453	cg11166453	71682363	AAAATAA	20729361	AAAATAA	I	C
-0.119743	cg11168104	cg11168104	66770303	ACAACCC	39654448	ACGACCC	I	C
0.1467473	cg11169848	cg11169848	48640443	AAACTTCTTAAATAACTCAACI			II	
0.1123199	cg11173609	cg11173609	30675437	ACTTTAAAACAAAACTATAAA			II	
0.1458334	cg11173822	cg11173822	12663331	ATTAAAACCATATTTTCCCTTA			II	
-0.130355	cg11181693	cg11181693	17786435	AAAATAC	54779449	AAAATAC	I	C
0.1281864	cg11188666	cg11188666	68683459	AACTTTAAAAACTTTAAAAATC			II	
0.1032458	cg11198851	cg11198851	26635475	RAAACCAAAAACCAACACACC			II	
-0.110693	cg11199639	cg11199639	35723384	AACAATA	44733367	AACGATA	I	C
0.103714	cg11200743	cg11200743	15605331	TAATATCTACTAAAATCCAACC			II	
0.1065162	cg11201654	cg11201654	31712468	CATTATCTAACACCAAACTCCT			II	
-0.189715	cg11204974	cg11204974	74804386	CCTAAAATCCCAACCTTACCTA			II	
-0.10954	cg11208322	cg11208322	35677461	ATTCAAAAACACTAATAACTTT			II	
0.1330224	cg11214507	cg11214507	61632480	AATTTCCTATTTCTACATTTCC			II	
0.1190989	cg11214962	cg11214962	17728452	ACCAAAT	10767414	ACCAAAT	I	A
0.1237011	cg11216176	cg11216176	50717467	AAAAACAACRAAAACACTCRA			II	
0.2605044	cg11219411	cg11219411	51645476	ATCCTCTCATCTCCTATTAACA			II	
0.117305	cg11223933	cg11223933	45608323	AAAAAATTTAAATTATAAACTC			II	
-0.183621	cg11225091	cg11225091	21723504	AAATACRCRTCAACAATTTATT			II	
0.1440884	cg11229273	cg11229273	37646416	TACTAACCCCTACAAACRTCTA			II	
0.1438487	cg11231735	cg11231735	29646482	AAAAACCTTATCCCCTAAACTA			II	
0.1239249	cg11235594	cg11235594	33659459	ACCATTCCAATCTTAACAATAT			II	
-0.117698	cg11236452	cg11236452	23667335	ACCCACCAATATTTACTCAACC			II	

-0.122212	cg11238952	cg11238952	10745507	AAATACC	55675345	AAATACC	I	A
0.1049671	cg11244648	cg11244648	35662458	AAACAAC	53636494	AAACAAC	I	A
-0.104289	cg11245569	cg11245569	73703494	CAAACTA	63631386	CGAACTA	I	T
0.1655175	cg11256764	cg11256764	74600393	CATTAAA	27676391	CATTAAA	I	A
0.1316157	cg11257300	cg11257300	34664436	TACTAATTCTCCTTTCTCTCTTA			II	
0.1310783	cg11261412	cg11261412	56712505	CAAACTAACCATATACATTTTC			II	
-0.114896	cg11273655	cg11273655	61728324	AAACRAATACCCTTCTCTTTCC			II	
0.1260024	cg11276189	cg11276189	13703378	AAAACAAACTAAATCAACAAT			II	
0.1656764	cg11282828	cg11282828	71675337	ACACAAA	46766498	ACGCAAA	I	C
-0.104702	cg11290168	cg11290168	19810503	ATTACTAAAACAAAATCTTTCA			II	
-0.124807	cg11294620	cg11294620	15746406	ACACAAA	27760466	ACACAAA	I	A
0.2151856	cg11294750	cg11294750	45729316	AAACACATTACACATCTTATA			II	
-0.10359	cg11296826	cg11296826	43725393	AAAAACTTTCCCCCCAAAAAA			II	
0.1129351	cg11305940	cg11305940	47709386	ATCTATTTATTCTTCTTATATTT			II	
0.2489807	cg11307715	cg11307715	46738432	CCTTACTAACTTCTTCRAAATA			II	
0.1129959	cg11313468	cg11313468	45809489	TACATACCTATAATCAAAAAC			II	
-0.120746	cg11314617	cg11314617	20789330	ATCACTAAATATTATAACTAAT			II	
-0.122415	cg11315989	cg11315989	67658342	TAAAACRCRATAAACACTATCA			II	
-0.120716	cg11322406	cg11322406	43709503	TAAAACC	64619450	TAAAACC	I	A
0.1300247	cg11328149	cg11328149	20625410	AAAAATCTACTCTATCAACTAC			II	
-0.131542	cg11330362	cg11330362	67757423	AAAAATAACRAAAAATACTAA			II	
0.1186622	cg11331837	cg11331837	11745317	TAACAATACACTTAAAAAAAA			II	
-0.132709	cg11333230	cg11333230	43751304	CTTAACAAAAATTTACTAACRC			II	
0.1003339	cg11334097	cg11334097	66706319	ATCCCTAAATATCCTAAAAAC			II	
-0.127137	cg11342198	cg11342198	69764364	TAAAATT	65674451	TAAAATT	I	T
-0.128526	cg11343211	cg11343211	47637401	AAAAAAT	23764398	AAAAAAT	I	A
0.1341342	cg11344614	cg11344614	14770336	AAAAACAACCCCTTATCATAA			II	
-0.163436	cg11347582	cg11347582	66606414	AACCCCT	66679395	GACCCCT	I	A
0.1175687	cg11348583	cg11348583	70762458	ATAATTT	29807455	ATAATTT	I	A
-0.114401	cg11356767	cg11356767	10795372	CAACCAA	11779447	CGACCGA	I	C
-0.142622	cg11361344	cg11361344	23608346	CATTTAA	46604473	CGTTTAA	I	C
-0.107299	cg11367354	cg11367354	65625460	TCTCCTTA	38750478	TCTCCTTA	I	A
-0.11172	cg11376198	cg11376198	36695359	TACTCAA	39793413	TACTCGA	I	C
0.1279335	cg11381655	cg11381655	40714400	ATATTCATCTATCTTCAATTCT			II	
-0.13206	cg11382082	cg11382082	33670461	ATTAAAAAAACTACTTATTCAT			II	
-0.164863	cg11382666	cg11382666	54618301	ACAATTA	61739303	ACGATTA	I	A
0.1213752	cg11383134	cg11383134	12727427	CTAAAAA	48709346	CTAAAAA	I	A
-0.121231	cg11383474	cg11383474	46689417	TAACTATAATATATACRTCTAT			II	
0.1108473	cg11387985	cg11387985	10603474	TCCTCCACTAACCCATAAATCT			II	
0.1543142	cg11391732	cg11391732	49616463	TATCRAATAAAACTTCTTAAAA			II	
-0.160235	cg11395062	cg11395062	59607487	ATTAAAACACAAACACTTAATA			II	
-0.496492	cg11399582	cg11399582	35765460	CTCCAAC	69623425	CTCCGAC	I	T
-0.21682	cg11400162	cg11400162	65791488	TCAATCAAACCACATATATAAC			II	

0.1030546	cg11401293	cg11401293	74707387	AACACAC	18753308	AACGCGC	I	C
0.1014298	cg11401820	cg11401820	57610366	AAAATAAAAAAACTCATTCCA			II	
0.106505	cg11403739	cg11403739	71720352	TAAAAAACACRTTCCCATCTTA			II	
0.1181899	cg11406977	cg11406977	23807351	ATAAAACCTATAATAAACACT			II	
0.1055606	cg11410649	cg11410649	41707436	ACTACTAACCAAATCTTTAAA			II	
0.3107132	cg11418607	cg11418607	28663477	AACCTACCTAATTCCAAAACA			II	
-0.123502	cg11420142	cg11420142	40618306	ACRATCTTCATAAATTATTTAC			II	
0.1136298	cg11422312	cg11422312	15807379	TCTATATAAAATCCACACCACA			II	
0.1267234	cg11424376	cg11424376	15684506	AATACAACTAAAATCTATTCA			II	
0.1731393	cg11424776	cg11424776	12730499	CCCAAAACAAATATATCTTAA			II	
-0.137776	cg11434705	cg11434705	48736392	AAAACCT	60678355	AAAACCT	I	C
-0.132828	cg11435506	cg11435506	47788310	TCCAATT	12673349	TCCAATT	I	A
0.5106072	cg11437465	cg11437465	63779342	CATTAAC	40684324	CATTAAC	I	A
0.1786116	cg11438287	cg11438287	53729382	ATTTCCTATACRTCTAAAATAA			II	
0.1028571	cg11445109	cg11445109	30735412	AAAACAA	38697402	AAAACAA	I	A
-0.157791	cg11449070	cg11449070	31795338	AAAAAAC	10773428	GAAAAAC	I	A
0.1125709	cg11453712	cg11453712	39675490	ATAAACAAAACAAAATTCRAA			II	
0.1094906	cg11456896	cg11456896	12760376	AACTCCTCTTTCATAACTTTAA			II	
-0.157035	cg11459648	cg11459648	61650494	AATATTA	61741509	AATATTA	I	C
0.2005206	cg11463428	cg11463428	54630353	ACCTCATCATCATCCCAATAA			II	
-0.172384	cg11467638	cg11467638	49685357	AAACAATTACTACTTTTATCTA			II	
0.1183338	cg11468148	cg11468148	52715444	AACTACA	31789306	AACTACG	I	A
0.1358615	cg11471401	cg11471401	36806374	AAAATACTAACTCAACRTCTA			II	
0.2022153	cg11495604	cg11495604	45775369	TCTACAT	34713473	TCTACGT	I	T
-0.156765	cg11510586	cg11510586	29790445	AATCCCA	29707302	AATCCCG	I	A
-0.152298	cg11517468	cg11517468	17608352	CCCTCCC	57733485	CCCTCCC	I	C
0.1963443	cg11519708	cg11519708	66764502	ACCTCACACTATAACTCCRAA			II	
-0.115129	cg11528328	cg11528328	41789306	CCCTTAA	59802488	CCCTTAA	I	A
0.1427399	cg11529236	cg11529236	58607316	RATAATAATTATCRAAATTTAT			II	
0.1735656	cg11530408	cg11530408	29795504	TAAAAAAATCTAAAAATTATT			II	
0.1020963	cg11533712	cg11533712	60750330	ACTAAAACCCAATATAAACAT			II	
0.1121202	cg11534293	cg11534293	70761405	CCTTCRCCTCCRTAATACCCTA			II	
-0.123602	cg11539674	cg11539674	24742430	AAAAAAATATCCRATAACTAA			II	
-0.143503	cg11540707	cg11540707	71744395	AAAACCCRTCTCTACAAAAAA			II	
0.1126128	cg11547201	cg11547201	64696506	CCACAACCCTCAAAAACATAA			II	
0.1074324	cg11552072	cg11552072	42614480	ACAACCA	65626337	ACGACCC	I	T
-0.129796	cg11555067	cg11555067	18610442	TACAATAACRAACTCTATACTC			II	
-0.13056	cg11558654	cg11558654	62620354	AATAACRAATAATTAACCTAA			II	
0.1154602	cg11562312	cg11562312	42736499	CACTCCTAACTTTAAAACTTAT			II	
0.1035383	cg11564239	cg11564239	46721362	TAAAAAACCTCTACACACCCA			II	
-0.102561	cg11565911	cg11565911	24744444	TAACTTCCAATATAATATTCRT			II	
-0.102412	cg11568314	cg11568314	11631401	CAAAAAAACAAATAATTCCAC			II	
0.1338205	cg11571124	cg11571124	50647374	AAAAAAAAAATACTAATTCTA			II	

-0.100536	cg11574745	cg11574745	66802480	CTATTCTAACRCAAACTAAAA			II	
0.1109704	cg11580790	cg11580790	25751411	AATAATAACTAAATCAATAAC			II	
0.1174277	cg11583351	cg11583351	19794335	AATCCAACTATTAAAATTCTTT			II	
-0.102691	cg11583762	cg11583762	34739476	RTATCAATACTAAAACRCTTCT			II	
-0.142227	cg11586189	cg11586189	55749302	TTATATTCTAAAAATAAAAAC			II	
0.1867119	cg11587069	cg11587069	36628408	CTTCCCA	11720427	CTTCCCG	I	A
0.1004175	cg11600734	cg11600734	47754482	AAAAAAAACCAAATTTAAAAA			II	
0.111643	cg11604182	cg11604182	55609355	TAAACRCTAACRCTCTAACCCT			II	
-0.12593	cg11608150	cg11608150	10777317	TTAAAAC	61659390	TTAAAAC	I	A
0.133836	cg11611580	cg11611580	64602334	TTACCCAATATTTTCATTATAC			II	
0.2071889	cg11613741	cg11613741	59635467	TTAAAACAACTAACCATCCAC			II	
0.1541541	cg11615029	cg11615029	59612379	TACAACC	60706338	TACAACC	I	C
0.1015643	cg11618093	cg11618093	39746495	ACCTACAAAAATAAAACTAAA			II	
0.2088363	cg11620135	cg11620135	63721382	CTAAAAAATCRATCCACTCTA			II	
0.1055044	cg11626496	cg11626496	39632421	ACCTCTCTAATCTCAACCRAA			II	
0.1169755	cg11627968	cg11627968	65699398	AAAACCCTTAATAATTCCAAT			II	
0.1309506	cg11630939	cg11630939	34709464	CTAAACA	15666478	CTAAACG	I	T
-0.138507	cg11635197	cg11635197	58689347	CACACAT	58611358	CACACGT	I	T
0.1663271	cg11637968	cg11637968	65748382	AACTTCTAAAACCTTTTTTCCT			II	
0.1007431	cg11639615	cg11639615	56766398	ACRCTTATAATCAATACACTAT			II	
0.1040449	cg11645453	cg11645453	70678460	ATATAAATAAAACAACAAACA			II	
0.1218222	cg11645539	cg11645539	36627324	AATTCCCAAAAACTAACCAAC			II	
-0.113138	cg11651932	cg11651932	11709328	AAATATTTAATCTCCCTACCCR			II	
0.1066901	cg11655490	cg11655490	72635435	ATAAATCACTAAAAAAAATCC			II	
0.1025639	cg11657323	cg11657323	45643453	AAATAACCTATAACCTTTAAC			II	
-0.147019	cg11660815	cg11660815	22797392	AAATCCTTCRTACTAAAATCTT			II	
0.1013534	cg11662061	cg11662061	39723430	AATAATAAACCRCAATAAAAA			II	
-0.105513	cg11663393	cg11663393	67761493	TATAACA	15688477	TATAACG	I	A
0.1154724	cg11663691	cg11663691	17615336	TACTTTCTTTCCCTTTTAAAAT			II	
-0.182083	cg11666534	cg11666534	18631405	CAACAAAACAATTAACAAATA			II	
-0.209806	cg11667387	cg11667387	56612305	TAACTTA	14770408	TAACTTA	I	T
0.116732	cg11668419	cg11668419	74728327	TTAAACTCAATTAATAATATCT			II	
-0.239948	cg11685843	cg11685843	13703448	ATTCCAA	22787371	ATTCCGA	I	A
0.1261314	cg11689753	cg11689753	29610312	ATACTAAAAATACAAACCTAA			II	
0.1203964	cg11692403	cg11692403	58733431	TTCTAAACCATAACTATAATAT			II	
0.101835	cg11695041	cg11695041	74647411	CAACTTT	43604473	CGACTTT	I	A
0.1102794	cg11712506	cg11712506	54694405	TTTAAATCATTCATAAACAAC			II	
0.1195122	cg11719509	cg11719509	52666370	CCTAACA	10666383	CCTAACG	I	T
-0.105708	cg11721100	cg11721100	36771440	CCAAAAA	15759409	CCAAAAA	I	A
-0.201523	cg11723923	cg11723923	34635449	TAACTACACTATATAATCCAC			II	
-0.11319	cg11724147	cg11724147	50745468	AACAAAAAAATAACTAAATAT			II	
0.1037499	cg11725415	cg11725415	13727428	CTAACCAACTCTTACTAACCAT			II	
0.1134717	cg11728747	cg11728747	26802426	TAACAAAACCCAACTACTTCA			II	

0.2185671	cg11733135	cg11733135	52698501	AACCCCTACTAAATTTAACAA			II	
-0.155012	cg11737757	cg11737757	73704494	AAAACATACCTAATCCRTAAC			II	
0.103571	cg11740348	cg11740348	44639397	CACCTAA	37643418	CGCCTAA	I	A
0.1037145	cg11750736	cg11750736	59636326	ATAAAAACCCATAAAAATAAA			II	
0.177115	cg11752699	cg11752699	65627450	CTAAAAATATACCTCCCRTTT			II	
0.1103278	cg11758283	cg11758283	23682302	CTAAACCTAAACCACTAAAAT			II	
-0.132851	cg11764747	cg11764747	39628357	AAATAAAAAAAACCCRAATAA			II	
0.112469	cg11765312	cg11765312	68621315	TATCTAAATACCTCAAATCCA			II	
0.1595337	cg11769349	cg11769349	36795500	CCCACAA	38669411	CCCACGA	I	A
0.1684297	cg11778714	cg11778714	50746371	TTTTCRTATTTATTAAAAACTA			II	
-0.10245	cg11781306	cg11781306	26694315	TATTTAT	41715429	TATTTAT	I	A
-0.100945	cg11783520	cg11783520	71794496	ATAAAACCACRAACCCCCRCR			II	
0.239755	cg11784298	cg11784298	38799338	TTTCTTTTCTCAAATATTACCT			II	
-0.115465	cg11786587	cg11786587	20739373	ATACTCACTCAACATAACATC			II	
0.3916615	cg11791078	cg11791078	19636496	TAAAACTATAACTCAATTCTT			II	
-0.1208	cg11791288	cg11791288	10745380	ACATCCTACTATATACAATTA			II	
0.1305271	cg11798406	cg11798406	64656334	TATATATACAACACTCAACTT			II	
-0.105637	cg11801727	cg11801727	13714508	AAACAAC	58789321	AAACGAC	I	A
-0.102485	cg11802299	cg11802299	35756476	CAATAAAAATCAAAAAAACAC			II	
0.1280844	cg11807153	cg11807153	64719320	AACTCACTTTATAACRTAAATC			II	
0.1005921	cg11808100	cg11808100	30622386	ATATTAAAACCAAAAAACACR			II	
-0.137888	cg11818589	cg11818589	62638468	TACTTAATAACACTTTAAAAC			II	
0.1300474	cg11820650	cg11820650	25615372	TCATTACTCACCATCCTTAATC			II	
0.1525269	cg11824564	cg11824564	27806415	AAATCAATTTCTAAAAATTAC			II	
-0.118594	cg11825731	cg11825731	18661402	TAAAAAC	62632378	TAAAAAC	I	C
-0.205064	cg11826726	cg11826726	52788439	ATAAACA	16650359	ATAAACA	I	A
0.1068047	cg11834635	cg11834635	39775431	TAATCTAATCCRTCRCAAAATA			II	
-0.218676	cg11835544	cg11835544	52804376	CTCACTTCCTCTTCAAAACTAC			II	
0.1421595	cg11839566	cg11839566	17742472	AACRAAAAAAACCCAAAAAAA			II	
-0.102033	cg11839681	cg11839681	69605379	TAACCTA	59741403	TAACCTA	I	T
-0.110603	cg11841771	cg11841771	72702348	TCAATCA	19639374	TCGATCA	I	C
-0.215654	cg11841863	cg11841863	29652450	CAATACTTAACTCAATCAAATA			II	
-0.115937	cg11849573	cg11849573	48660405	AAAACTT	27635396	AAAACTT	I	A
-0.309061	cg11857805	cg11857805	30613358	ACTAACCAATACAAATCTCCA			II	
0.1270913	cg11859922	cg11859922	34662471	TAATCATCTCAAAACTAAAAC			II	
-0.186988	cg11861387	cg11861387	19803414	ACTCCCC	63613441	ACTCCCC	I	C
-0.108818	cg11861905	cg11861905	67809443	AAAACCT	30721352	AAAACCT	I	C
0.1049152	cg11871064	cg11871064	30688439	CCAAATCCTCTTTCAACTCAA			II	
	cg11871549	cg11871549	13644500	CAAATTCCCAAACAAATTAAC			II	
0.1566539	cg11874976	cg11874976	48786440	CATTCCT	45700445	CGTTCCT	I	T
0.1569063	cg11877812	cg11877812	26647449	TCCAAAATCTCTAATATAACC			II	
0.1039407	cg11879096	cg11879096	30655510	TTATTTCTTATTAATTAAAAAC			II	
-0.114562	cg11881038	cg11881038	24635354	CRAATTAAATCCATTTTCAAAC			II	

0.3369749	cg11882358	cg11882358	66678315	CCTTACTTTTCTATTCTATTAAC			II	
	cg11883141	cg11883141	22757330	ATAAAAAACTATCTAACATAC			II	
0.1702293	cg11884832	cg11884832	35693488	AAACTAATTTTAACATTATAAA			II	
0.1359209	cg11885357	cg11885357	13736432	AAACAAA	71638330	AAACGAA	I	A
-0.140173	cg11891395	cg11891395	51682484	AAAAAAACAATTTACTTATAA			II	
0.1263906	cg11897628	cg11897628	51606301	AAATATAAAAACCATAACTCA			II	
-0.116422	cg11897887	cg11897887	41757509	AAACCTATATCCTAATTAACAA			II	
0.154685	cg11898347	cg11898347	24804430	ATACCCTAACAATATATTCATT			II	
-0.155755	cg11900328	cg11900328	63649415	AAACCATCATCATTCCTACTTC			II	
-0.220177	cg11900447	cg11900447	23645312	TTACCAA	30728493	TTACCGA	I	A
0.1967297	cg11903206	cg11903206	37679389	AATTAAAAAATTCTCCCTACCC			II	
0.1412692	cg11907323	cg11907323	56729493	ACCATCCTTTCACAAAACACAA			II	
-0.162606	cg11909574	cg11909574	70734487	CAAAAAC	69691433	CAAAAAC	I	A
-0.109916	cg11917288	cg11917288	28715494	CTATACTCAACTTCTAATAAAA			II	
0.1051977	cg11918822	cg11918822	44775332	AAAACACTAACTAAAAAACAA			II	
0.2340713	cg11923920	cg11923920	16652322	AACRACCTATATAACTATCAC			II	
-0.132662	cg11934170	cg11934170	51707449	TAACAAA	21754348	TAACAAA	I	T
-0.119687	cg11941060	cg11941060	59797491	CTCCAAA	41669341	CTCCGAA	I	C
-0.17288	cg11941920	cg11941920	45725411	ACCTAAA	73626375	ACCTAAA	I	A
-0.287758	cg11942206	cg11942206	70765391	TCCTTCTATTTCTCCTTTTATCT			II	
0.1040324	cg11942594	cg11942594	32810500	TAAATAAAAACCAATTTTAAA			II	
-0.202416	cg11946719	cg11946719	14668488	CAAACAC	73718429	CGAACAC	I	A
0.1527917	cg11948905	cg11948905	14775413	AAATTTATACCRACCTAACTTC			II	
0.113852	cg11949175	cg11949175	55763401	AAAAATCCAAAACCCAAAATT			II	
0.149735	cg11953164	cg11953164	26752472	AAAAAAATTCCAACCRATTCA			II	
-0.386451	cg11956442	cg11956442	51600385	AAAAATCAATAAAAAATTAAA			II	
-0.164522	cg11957400	cg11957400	13796331	TATTATACACRTTAATAAACAA			II	
0.1992412	cg11969526	cg11969526	45736441	AACTCTTTAAAAATTCRAAACC			II	
0.1831126	cg11973981	cg11973981	49644409	ATTTAAAACAATAAAAACTAT			II	
-0.102402	cg11975397	cg11975397	61717500	CAACCAAAACCTCTTTAAAATA			II	
0.1082197	cg11976736	cg11976736	56765422	AAACAAAACATAAATATACAA			II	
-0.106051	cg11979875	cg11979875	28791379	AACTACTATAACCCTAAACAA			II	
0.1048631	cg11983196	cg11983196	11806442	AAATAATCCTTTACTTCTACRA			II	
0.1397672	cg11984971	cg11984971	37634448	AAAAAAATAATACTAACTTCC			II	
-0.195624	cg11986643	cg11986643	25709418	TACACTTACCRAAAAAATCTCT			II	
0.1195126	cg11986743	cg11986743	44644324	TCTCTTCRAAAAAAACTCTCTT			II	
0.1532804	cg11987751	cg11987751	11733380	ACTTCCT	65689343	ACTTCCT	I	C
0.4160441	cg11993350	cg11993350	62621397	AACTCCC	50713487	AACTCCC	I	A
0.1010199	cg12002455	cg12002455	55794335	TTTCCTTAAAAACTATAACAAC			II	
0.1052703	cg12005412	cg12005412	47709311	CTCTCCTTTTTTTATTCTCCACA			II	
0.1067102	cg12010476	cg12010476	35705472	CAACAAAACCAATATAAACTA			II	
0.3684176	cg12012426	cg12012426	66781467	AAAACAAACRAAAATCAACTT			II	
0.1578395	cg12014181	cg12014181	17657505	AAACATT	15640378	GAACATT	I	A

-0.154857	cg12024822	cg12024822	38736307	ACTAAAA	72718469	ACTAAAA	I	C
0.1128795	cg12025137	cg12025137	23680449	ATCCCAAATAACAACAACTCTA			II	
-0.245253	cg12036633	cg12036633	34610328	ACCTTAAAATACCAAACTCTAA			II	
0.11089	cg12044531	cg12044531	73684393	CTATACCTTAAAAACCTATAAC			II	
0.1001026	cg12051819	cg12051819	59730440	TTAACTAAACATTAAAATTACA			II	
-0.150173	cg12058262	cg12058262	30635302	TTAATTA	56774418	TTAATTA	I	A
-0.1264	cg12070022	cg12070022	48721390	ATTCCAA	27615377	ATTCCAA	I	C
0.1381571	cg12072024	cg12072024	71732385	TAAACCRATTCTAACCAATATA			II	
-0.210446	cg12074485	cg12074485	39644373	TATTCCAACCCCTAAATCTACA			II	
-0.137009	cg12076350	cg12076350	71604490	TACAAATACTACTATCTAACTA			II	
-0.222203	cg12078775	cg12078775	51701343	TCTAAAA	39654350	TCTAAAA	I	A
0.1222708	cg12082015	cg12082015	69749311	ATAACATAATATTAAATACTTC			II	
0.1003426	cg12089079	cg12089079	39701347	AATAATTATAAAACTTTTCTTC			II	
	cg12094863	cg12094863	13752450	CACTTCCRCTTACCTCTCATTA			II	
0.1240412	cg12096487	cg12096487	69672368	CATAAAAACTACRCRTCTAAA			II	
0.1224758	cg12097550	cg12097550	74670410	TACAACTTAAACAAAAACACT			II	
0.1183644	cg12097671	cg12097671	42757396	CTATTCTTCAATATCTCTTTTA			II	
-0.115538	cg12102973	cg12102973	59743375	AAAAAAACAAACCRATAAACT			II	
-0.133679	cg12105899	cg12105899	13790458	CAAAAAA	24602442	CGAAAAA	I	T
-0.110468	cg12109260	cg12109260	74638358	TACTAAA	51763428	TACTAAA	I	T
0.1257378	cg12111149	cg12111149	36794431	TAAAACTCTTTTACCACCCCCA			II	
0.1053723	cg12119988	cg12119988	38752400	TACTAAAACCCTACTCCCTCAA			II	
-0.13603	cg12121446	cg12121446	66659507	ACACATA	20782356	ACACATA	I	A
-0.106175	cg12121660	cg12121660	45689375	ATAAAAA	52608417	ATAAAAA	I	T
-0.156798	cg12123879	cg12123879	64640419	AACAATTATCAAAATTACTAAC			II	
0.1476087	cg12127811	cg12127811	51740387	ATATATAATATTAAATCTCTAT			II	
0.1741853	cg12128445	cg12128445	15615502	AAAAACTACTTTATTTAAAAAC			II	
-0.125168	cg12130015	cg12130015	11796352	TAAACAC	72630412	TAAACAC	I	T
0.1267521	cg12146763	cg12146763	20676448	CATATCTTATTCATCTTTATATC			II	
-0.147375	cg12159023	cg12159023	19779377	ATAAACA	57795385	ATAAACA	I	A
-0.108462	cg12160424	cg12160424	74764369	TAAAAAT	61763322	TAAAAAT	I	C
-0.120893	cg12161971	cg12161971	14739431	CAAACAC	73762406	CGAACGC	I	A
0.3507917	cg12167002	cg12167002	63634497	TACTACA	57679423	TACTACG	I	A
0.1282933	cg12168509	cg12168509	25749369	TACTCAACRTCACRTAACTATA			II	
-0.1579	cg12173150	cg12173150	14667323	AAAAATA	12605439	AAAAATA	I	C
-0.12789	cg12177182	cg12177182	32796360	AAAAACA	28663432	AAAAACA	I	T
0.1661988	cg12179658	cg12179658	10600406	ATAAACACCAAAACAAACCTA			II	
0.1194378	cg12198140	cg12198140	17740335	AATTTAAAAACACTAACRTCAT			II	
-0.249759	cg12208638	cg12208638	27767440	TTCAAAACCTAAAAAACRATA			II	
-0.217445	cg12209881	cg12209881	44636452	CCCTAATAAACACTAACCCCA			II	
0.225235	cg12213037	cg12213037	23751502	CTAAACRACAAAACAAAAACT			II	
0.1764701	cg12217954	cg12217954	29749437	ACCAATAACTCTATATAATCTC			II	
0.1281553	cg12219469	cg12219469	24798379	ACCTAAAATCACCTCRTAACTC			II	

-0.111967	cg12219707	cg12219707	51713307	AAAACCTTAACCCAATAATAT			II	
-0.131241	cg12226028	cg12226028	21727322	AATATTA	44763391	AATATTA	I	T
0.1498241	cg12226948	cg12226948	15672418	ACTACAATTAAATCRTAACAC			II	
0.1436125	cg12232463	cg12232463	48788346	AAAATCTAACTTCCAACCTTT			II	
0.1286	cg12232731	cg12232731	71697384	ATAACAA	42681335	ATAACAA	I	T
0.175789	cg12241333	cg12241333	32689477	TCACAAATCACCACAAAAATA			II	
-0.149048	cg12243267	cg12243267	65639315	CAAATAA	48785495	CGAATAA	I	C
0.1157418	cg12250645	cg12250645	40669385	ATTAACAAAAATCCCACCCCA			II	
0.1839934	cg12253071	cg12253071	71659403	CTATAAAAAACTAAATATATA			II	
-0.116288	cg12255897	cg12255897	74711503	AATCAAAAAACCCTACRTTCA			II	
-0.13098	cg12258179	cg12258179	15655389	ACTACTA	20620474	ACTACTA	I	C
0.1164765	cg12259892	cg12259892	53781503	TTAATTATTCTAAATATTCCTA			II	
-0.151354	cg12269972	cg12269972	43644410	CAATAAAAAATCAAAATAATA			II	
0.1600513	cg12271317	cg12271317	10739502	TAATTACTAACTTTAATTACRC			II	
0.3481065	cg12279734	cg12279734	32767338	AAATCAAATTCACACATCATA			II	
0.110742	cg12296550	cg12296550	28660318	CAATAATTAAAACACTCTCAC			II	
-0.112968	cg12297590	cg12297590	50692476	AAAACAA	66741323	AAAACGA	I	A
0.1563049	cg12303318	cg12303318	36649384	CRAACACAAAAATCAAACCTT			II	
0.1164808	cg12303813	cg12303813	51607504	CCRAACCAAAACATTATAAAC			II	
0.1199041	cg12307373	cg12307373	49606365	ACAATTTAAACTCCACAACAT			II	
-0.466653	cg12310025	cg12310025	72708489	CATCTAT	39781349	CATCTAT	I	T
-0.132341	cg12312457	cg12312457	30631400	CTTTCAC	39730423	CTTTCGC	I	A
-0.22024	cg12321669	cg12321669	29698322	RAACAATACTAAAACRCTAAT			II	
0.2223081	cg12341429	cg12341429	16642311	ACTCATCCCCTTACACTTTAAA			II	
0.206247	cg12342501	cg12342501	18752444	CCAATAAAACAACAATCRCTA			II	
0.1123271	cg12360046	cg12360046	70788465	AAAATAA	42634485	GAAATAA	I	C
0.1110103	cg12361987	cg12361987	14683391	TATCATAACCTACTACRACTAC			II	
0.180174	cg12370174	cg12370174	58620371	TCATCAATATATACTCTAAAA			II	
0.1416509	cg12377816	cg12377816	55643407	TATCTCTAATTCCCTATTAATT			II	
-0.112338	cg12383699	cg12383699	64618426	ACCATCC	60658391	GCCGTCC	I	A
0.4894441	cg12386614	cg12386614	42678409	AAACTAATAACCTTATTAAAT			II	
-0.191249	cg12388523	cg12388523	15780459	CATTTTACAACCTATATTAAAA			II	
0.4238711	cg12391372	cg12391372	17745430	TACTATCTAAAAACCCAAATAC			II	
0.1008284	cg12391576	cg12391576	47783490	CTATTAATCTATACRTCTATAC			II	
-0.100524	cg12392475	cg12392475	67717362	TCTACCT	45613312	TCTACCT	I	A
0.1094521	cg12395823	cg12395823	42695344	ACCATCA	11610410	ACCGTCG	I	T
-0.124754	cg12400864	cg12400864	67609391	CCCAAAAAACCRAAACCAAAA			II	
0.1063498	cg12401953	cg12401953	23721353	TAACTTAAATAAAATTTACAA			II	
0.1112951	cg12403162	cg12403162	37764433	CTAATTACTAAATAATAATCAC			II	
0.156376	cg12406683	cg12406683	39642444	ACTTTCAATAAAAACAACAAA			II	
0.1145756	cg12410980	cg12410980	63628360	ATTAATA	74804390	ATTAATA	I	C
0.1731881	cg12412775	cg12412775	64764403	RTCATACTAATTATACCCTACA			II	
-0.151489	cg12417704	cg12417704	50725348	AAAACAC	50627440	AAAACGC	I	A

0.1006397	cg1241804	cg1241804	15670433	CTTACCCTTAATAATAATTTTC			II	
-0.100615	cg1241938	cg1241938	38641411	AAACCCT	13767478	AAACCCT	I	A
-0.138199	cg1242010	cg1242010	64652348	ATAATAA	45732372	ATAATAA	I	T
0.147016	cg1242108	cg1242108	74634484	TACCCTATAAATAAATAATAC			II	
0.1453294	cg1242253	cg1242253	12612468	ACACACT	29704333	ACACGCT	I	A
0.1167544	cg1242586	cg1242586	52664318	AAAACAAAAAATCAACAAAA			II	
0.1147842	cg1242716	cg1242716	57684372	TTTTAAT	27774501	TTTTAAT	I	C
-0.142097	cg1243077	cg1243077	11781506	ACCTACACRAAACACTACTTT			II	
0.1996354	cg1243280	cg1243280	10741417	TTCRCATATACAATATAATTA			II	
0.111805	cg1243488	cg1243488	20673311	CTCACRAAATTTTCCRAAACC			II	
-0.242838	cg1243489	cg1243489	20616326	AAAAATTCRAAATCTATAAAA			II	
-0.255628	cg1244224	cg1244224	17757417	TCAACAT	65807459	TCAACGT	I	T
	cg1244484	cg1244484	39719478	AACCTAC	59656491	AACCTAC	I	A
0.1620967	cg1245001	cg1245001	26697303	ACAAATAAATACTAACTACTT			II	
0.138295	cg1245362	cg1245362	64643493	AACCCCCAACACCAACATCTT			II	
-0.320258	cg1245416	cg1245416	55636429	TTCCTCCCAATTCCCATAACA			II	
-0.110504	cg1246013	cg1246013	47668470	CACACAA	56640492	CGCGCAA	I	A
-0.151251	cg1246877	cg1246877	65744355	AATTACC	33762399	AATTACC	I	A
-0.194017	cg1246947	cg1246947	44602352	CCTACAA	24782322	CCTACGA	I	T
0.1194328	cg1247818	cg1247818	24720423	AAAATTCTAATCCAACCRCRA			II	
-0.236664	cg1247844	cg1247844	51764498	AATCACT	72769417	AATCGCT	I	T
0.1198309	cg1248300	cg1248300	53609398	AAAAAAACAATCTTCAAAAAT			II	
-0.126494	cg1248518	cg1248518	37798323	AATAACA	33742393	AATAACC	I	A
0.105372	cg1249376	cg1249376	15634478	ATAATAAAATAAACCCTAAAT			II	
0.1533902	cg1249420	cg1249420	61705307	TCTAAAAATCCTTTACAAACR			II	
0.1982616	cg1250030	cg1250030	19792426	CATAACTATATCTCCTCTCTTT			II	
-0.229889	cg1250128	cg1250128	16765481	AATCTAAAACCTACCCAAAAA			II	
-0.101242	cg1250176	cg1250176	15663356	AAAATAC	10749417	AAAATAC	I	A
-0.124509	cg1250508	cg1250508	33715503	AATTTTCTTTTCTCTCCTTTAA			II	
0.1375784	cg1251099	cg1251099	50637410	CRCTATAACTATTAAAATCCC			II	
0.3817115	cg1251565	cg1251565	21610491	AAAAAAAAAACTATATCTAAA			II	
-0.120947	cg1252397	cg1252397	10612336	CCTCTCT	69641414	CCTCTCT	I	C
	cg1252629	cg1252629	62624444	CCRAATCCAAATTAAACCTCA			II	
0.1177448	cg1253209	cg1253209	51755472	ACATACTTCTATACTAACATA			II	
0.1061064	cg1253700	cg1253700	60713304	CCTCACAAAAACACACAATAT			II	
-0.100443	cg1253941	cg1253941	58609470	ATATCTAACAAAAACTCTCAA			II	
-0.140075	cg1254635	cg1254635	61744370	TTTCACA	67790412	TTTCGCA	I	C
0.1300593	cg1254817	cg1254817	37622434	CATACCTATAAATAAATCAAT			II	
-0.161342	cg1255656	cg1255656	63705396	TAACTCATCCTAACTCATTTCC			II	
-0.207945	cg1256282	cg1256282	25616402	AATCACA	66711311	AATCACA	I	A
0.1026454	cg1256352	cg1256352	59700446	ATAACAACAAACATACCATAA			II	
0.1032152	cg1256578	cg1256578	36716357	CAATCAAATAAATACCCCACA			II	
-0.267931	cg1256988	cg1256988	11794464	AAATTAATTTTTAAAAACTCA			II	

0.1116675	cg12572665	cg12572665	21613381	TTCCCTAAAAATCTATAATAAA			II	
-0.135617	cg12580893	cg12580893	61808327	AACCTTAAAAAAAACTAAACA			II	
0.101812	cg12581298	cg12581298	29693368	CCTACACRACAAAATAATAAC			II	
-0.124846	cg12584960	cg12584960	55799317	AATCTATCCCCTAATCCTCATA			II	
-0.101634	cg12586386	cg12586386	21646472	CAAAACA	10807359	CAAAACC	I	C
0.1449285	cg12591668	cg12591668	67703325	ACATCTAATATAAAAAAAATC			II	
0.1137184	cg12592365	cg12592365	57675375	AAAATTCTTCCATACTACCTAA			II	
0.1128238	cg12593223	cg12593223	33788324	AACCCTTCTACTCACRTAAATT			II	
-0.117449	cg12597614	cg12597614	30728473	CRTAAACCCATATATACRAAAC			II	
0.1128085	cg12600530	cg12600530	13627485	ACAAACCACCTTTCACAAAAC			II	
0.1399729	cg12606694	cg12606694	62677356	AAATAACCACAAACAAAAATAA			II	
0.1040529	cg12612406	cg12612406	42723447	AAAATACCACTAATAAAAACT			II	
0.1039301	cg12618270	cg12618270	44631474	CCTACTTCTTACTAAAAAATAA			II	
0.1190513	cg12618587	cg12618587	12792490	CACTAATCCTAACATTCAAATT			II	
0.3671333	cg12619747	cg12619747	24654347	CAACATCRCTTTAAAATACTCA			II	
-0.311804	cg12622653	cg12622653	12675381	CTAACCTATACTACATTACCTT			II	
-0.105113	cg12623145	cg12623145	27656419	AATACAA	73632474	AATACGA	I	C
0.308625	cg12633154	cg12633154	50649400	AATAAAA	56766334	AATAAAA	I	A
0.140761	cg12635120	cg12635120	15798509	ACTTAACTTTAACTTCTATAAC			II	
0.1013287	cg12636499	cg12636499	66659489	AAACTTCCCTAAACCACTTCTA			II	
0.1050226	cg12637448	cg12637448	60623484	CRAATTTATCTATTACTACTAA			II	
-0.138396	cg12642717	cg12642717	36748372	CTTAAAA	17689406	CTTAAAA	I	T
0.1351146	cg12655768	cg12655768	18755483	TTTACAATTCTACTTCTCAACA			II	
0.1149531	cg12656291	cg12656291	44615392	CAAATTAAAAAACCTTTACATT			II	
0.1653149	cg12657416	cg12657416	36650391	AAACTTCRATATAATTTCTAAA			II	
0.1078849	cg12658929	cg12658929	68601326	CTACAACCCAAACTCTAAACC			II	
0.1053527	cg12668523	cg12668523	10768401	AACAAAC	37791311	AACAAAC	I	A
0.2735482	cg12671632	cg12671632	66621406	TACCTAAACATTTTAATCATCC			II	
-0.130482	cg12675583	cg12675583	45738312	AAAATAAAAAAAACTAATAAA			II	
0.1033454	cg12681402	cg12681402	16744343	TCTTTCAAACACCTCCRCTAAA			II	
-0.12064	cg12684052	cg12684052	63666330	AAACACA	69645424	AAACACA	I	C
0.1295115	cg12684958	cg12684958	25719432	AATAAATCATAATCCTATTCAA			II	
0.1259932	cg12692069	cg12692069	19807430	ATAAAAAATTACTAACACAAA			II	
-0.172606	cg12697337	cg12697337	32605374	AAAAAAA	18611433	AAAAAAA	I	A
0.1163046	cg12698626	cg12698626	45734417	CCCTATCCCCAATAAACTACCA			II	
-0.112803	cg12715285	cg12715285	56624398	ATTTACA	17604473	ATTTACA	I	A
0.1059195	cg12718582	cg12718582	59674465	TTACTCTAAACAAAACTCRACT			II	
0.1233469	cg12719844	cg12719844	69677404	AAATCCCTACACTAATTATAAC			II	
0.1199766	cg12719904	cg12719904	20778318	ATACTTTATAACATAATTAAAA			II	
-0.376363	cg12727363	cg12727363	60634435	AAACACAAAAAATCTAAAACR			II	
0.1106574	cg12727940	cg12727940	67795441	CAAAAAACATAAACAACCTAC			II	
0.1407546	cg12736877	cg12736877	10770349	CCAAAAA	50650477	CCAAAAA	I	T
-0.220693	cg12743416	cg12743416	66736391	TTCACATAAAAACTATCAAAA			II	

0.1043063	cg1274889(	cg1274889(	17670473	TAAAAAA	47654449	TAAAAAA	I	A
-0.204811	cg12770425	cg12770425	38801326	AAACCCTACAATATAATATCA			II	
-0.146589	cg12775223	cg12775223	27621489	AAAAAAATTCTTATTTATTCTC			II	
-0.117786	cg12798157	cg12798157	24768440	AATACAA	62638473	AATACAA	I	T
0.1138106	cg12807764	cg12807764	26801402	CAACTCCATTTTCTACATTTTC			II	
-0.112655	cg12813768	cg12813768	16676310	CTCCCAAAATCTAAAATTTCAC			II	
0.2173475	cg12814117	cg12814117	53619503	CTCACAC	69695446	CTCGCAC	I	A
0.3512494	cg12819537	cg12819537	19709454	TACTCTCCTTATATAAAATCTT			II	
0.1926998	cg12829045	cg12829045	29706324	CAACACCATCTCCCAATTTTAA			II	
-0.295403	cg12833683	cg12833683	69732368	ACATTAACATAATCATAATTA/			II	
0.1051517	cg12844324	cg12844324	17775456	TCAAACTCCCAAAACTACTTA/			II	
0.1059651	cg12846656	cg12846656	31662374	AAAATATTATAATTTCTAACA/			II	
-0.152261	cg12850379	cg12850379	41671327	AACCTAA	30766302	AACCTAA	I	A
-0.121881	cg12850396	cg12850396	62624375	TTCCACTCTTTACTATATTATA			II	
0.1276812	cg12854428	cg12854428	17691497	ATCCAACTCTTAAAATATCATC			II	
0.1152255	cg12854611	cg12854611	51755382	CAAATTTAAATAAAAAAATAC			II	
0.1764738	cg12856521	cg12856521	38625322	AAAAATCAACRACATATAAAA			II	
0.4127325	cg12858166	cg12858166	24803314	ACAACAACACACACAATTATT.			II	
-0.145643	cg12858577	cg12858577	48618427	CCTCACA	35705303	CCTCGCA	I	A
0.206622	cg12865025	cg12865025	19674471	TTTTCTCCTTATCATTTACCTAC			II	
0.1522775	cg12867728	cg12867728	65606362	ATAAACAACTTTCTAACTTCCT			II	
-0.127047	cg12868067	cg12868067	39648318	ACAAAAC	39735482	GCGAAAC	I	A
-0.135581	cg12868173	cg12868173	42635367	CAACATC	30607507	CAACGTC	I	T
-0.100085	cg12869097	cg12869097	41744326	AAAAACACTAAACTTTCACCC			II	
-0.220744	cg12870217	cg12870217	72752369	TCCTAAAACAAATCAATTTCTA			II	
-0.129424	cg12880095	cg12880095	59650300	ATAATTT	64739460	ATAATTT	I	C
-0.144208	cg12890750	cg12890750	49745331	ATAATAA	28793481	ATAATAA	I	T
0.1062618	cg12894371	cg12894371	26658403	ACTAACACAACTACTATTTCCC			II	
-0.129829	cg12906381	cg12906381	62636340	ACAAATT	14765369	ACGAATT	I	A
0.1583416	cg12916580	cg12916580	58790404	CACACAC	62624315	CACACAC	I	A
-0.139506	cg12927641	cg12927641	68672507	AAAAAAA	24708479	AAAAAAA	I	A
-0.104309	cg12934884	cg12934884	11642389	TTTCCCA.	68758402	TTTCCCG.	I	A
0.1428649	cg12936992	cg12936992	52695442	CCTTCACTCAACTAATTTTATC			II	
0.1064489	cg12937413	cg12937413	65750346	TATTTACTTATAACATACATAT			II	
-0.113085	cg12939425	cg12939425	61804397	ACACCAA	16640327	GCACCGA	I	C
0.1274002	cg12947138	cg12947138	68707481	TAAAACCTTTACAAACCAAAA			II	
-0.102816	cg12955305	cg12955305	69802385	ACACTTAAACAATATCTTCCTA			II	
-0.150003	cg12955789	cg12955789	26792344	CATCACC	56703446	CATCGCC	I	C
0.1296864	cg12964420	cg12964420	62633339	AAACTTCATTCTATATAACTTC			II	
0.1310313	cg12972705	cg12972705	51653422	ATTCATAACCCACAATTATCAC			II	
0.1146562	cg12980549	cg12980549	66767338	CTATCTAACTTTTAATAAAACT			II	
0.1235387	cg12981577	cg12981577	28711442	CTAATATTCCCAAATTACAAA/			II	
0.1126527	cg12995004	cg12995004	21612374	ATTTTTTCCTAATATCTTTTTCT			II	

0.108071	cg1299908	cg1299908	57650306 TATCCTAATCTCRACAAAAAA	II		
0.1003644	cg1299926	cg1299926	39645503 AAAATTAATTCTAACTAATAA	II		
0.1095189	cg1300250	cg1300250	34656427 ACTTATTAATCAACRATAATA	II		
0.1004291	cg1300450	cg1300450	43734355 CAACAA 12744437 CAACGA	I		C
0.1805474	cg1300833	cg1300833	67763423 CRAAAACTCCCTTCCRAATTC	II		
0.115737	cg1300981	cg1300981	15796314 TAACCAAAAAAACCRTAAAAC	II		
-0.135029	cg1301079	cg1301079	65732385 ACTATTATAAAACACACCCCC	II		
0.1094301	cg1301100	cg1301100	65677367 ATTCCTATCACCACACTACTA	II		
-0.138366	cg1301702	cg1301702	52689403 ACCCAATCAATACCATTTATT	II		
0.2263854	cg1301792	cg1301792	74607403 ATTCAATAAAACTATATTAAA	II		
0.1414913	cg1301955	cg1301955	20649506 CAAATCT 58748301 CAAATCT	I		A
0.1035121	cg1302253	cg1302253	29702360 ATCTATTTAACTCTAAAACAC	II		
-0.107956	cg1302290	cg1302290	74729492 ATAAACT 42717468 ATAAACT	I		A
-0.197739	cg1302451	cg1302451	48753301 AAAAAA 14609420 AAAAAA	I		A
0.1072765	cg1303102	cg1303102	61688421 AAAAAAAACATCACTAAAACA	II		
0.1292071	cg1303385	cg1303385	18605367 TAAAACAATACTTTCCTAACA	II		
0.1369593	cg1303724	cg1303724	43731416 AAACATTCCCRCTAAATCACC	II		
-0.12052	cg1304535	cg1304535	23605470 CRTAACTTATATATCCTTCCRT	II		
0.2002466	cg1304660	cg1304660	18809380 CATCTAATACRCAAAAAACTC	II		
-0.160801	cg1304715	cg1304715	42694384 CRAAAACRCCCTAATCCCTCT	II		
-0.212314	cg1304786	cg1304786	40640376 CCCTATC 68808498 CCCTATC	I		A
0.1136692	cg1305263	cg1305263	49751453 TTAACATTTTTTTAATCTATC	II		
0.1154668	cg1305435	cg1305435	18653470 TCCCTCTAATCTCTTTCCAATA	II		
0.1266958	cg1305845	cg1305845	46747447 AAAAATAAATAACATAATCCC	II		
-0.135259	cg1305881	cg1305881	20716341 CTACAAC 15736324 CTACAAC	I		A
0.114206	cg1306146	cg1306146	31625467 AACAAAACTTATTTTTAAAAA	II		
0.2083466	cg1306646	cg1306646	74730398 TACTACATATTCACTAATTTTC	II		
0.1013381	cg1306763	cg1306763	73615343 CAATAAAATACATACCAAACC	II		
0.2883348	cg1306797	cg1306797	72755340 AATTACTATCRATATCCCAAC	II		
0.2047825	cg1307150	cg1307150	74728372 TAATTAAACCTAAATAAACAA	II		
-0.1008	cg1307678	cg1307678	63802308 AAAATCTAAAAACTTATTTTC	II		
-0.121253	cg1307838	cg1307838	67681305 TCTAATA 10652505 TCTAATA	I		A
-0.162134	cg1307879	cg1307879	67702356 AAATAAAAATTCTACCTAATA	II		
-0.166581	cg1308056	cg1308056	34620490 AAACCTT 68688337 AAACCTT	I		A
-0.116074	cg1308149	cg1308149	62689375 TTCTAAAATACCTTCTAAACA	II		
-0.125108	cg1309942	cg1309942	10639300 AACTAATAAAACACTACACTA	II		
-0.129858	cg1310019	cg1310019	73796403 AACCTTTTAAAACATATACRC	II		
-0.133889	cg1311240	cg1311240	69686420 AACAAAT 49608353 AACGAAT	I		A
-0.15969	cg1312792	cg1312792	14629366 CTAAAACCTCTATTCCCRATA	II		
0.2233386	cg1313444	cg1313444	35631470 CCAAACTCCRACAAAATAAAA	II		
-0.270276	cg1313780	cg1313780	60688366 CTCTTCA 70753505 CTCTTCA	I		T
0.1217446	cg1313808	cg1313808	10675383 CCTCATT 71751402 CCTCGTT	I		T
-0.19393	cg1314213	cg1314213	40624323 AAAAAATAAAAATCAAATCA	II		

0.1017179	cg13153708	cg13153708	41729481	ACATTTTAATTATTTCCAAAAA			II	
0.3872594	cg13160852	cg13160852	56732304	ATAACCAAAAAACACAACCCA			II	
-0.101636	cg13168042	cg13168042	44665366	AAATAAA	48645411	AAATAAA	I	C
0.1110019	cg13168698	cg13168698	54790415	AAACCTACCTTCTAAAAACTTA			II	
-0.166839	cg13169132	cg13169132	28732384	AACCACT	17730383	AACCGCT	I	C
0.1234684	cg13169221	cg13169221	50629351	TACRAATCATTCCAAATAACTA			II	
0.1288944	cg13171679	cg13171679	34693303	CTTAATATAATACAAACCACTT			II	
0.1008987	cg13173392	cg13173392	72711319	TTTCCAACTTAAAACTATTACA			II	
0.1070763	cg13178597	cg13178597	38711495	TTTAACCACTCTAATCAAAAC			II	
0.2124274	cg13183651	cg13183651	24757360	CACCTTATACTCCCTCTTAAAC			II	
0.1342141	cg13184448	cg13184448	30610477	CTTACTCCTTTACTAACCCTAT			II	
0.2172931	cg13185413	cg13185413	68665427	TAAAAAA	33656307	TAAAAAA	I	A
0.1120493	cg13185984	cg13185984	11653303	AAAAAAAATCCRTAACCCAAA			II	
-0.124589	cg13205528	cg13205528	18746348	ACATATCCAACACAAACTCRA			II	
-0.119404	cg13207326	cg13207326	65656400	AAAAAAA	58709472	AAAAAAA	I	C
0.1026338	cg13211008	cg13211008	13615312	AAAACTATTACRTTAATACAA			II	
0.1444882	cg13213536	cg13213536	20774335	AAAAACCCAAAACAAAATAAC			II	
-0.156615	cg13219409	cg13219409	51703337	CAAAACCTAAAACTCTACAAA			II	
0.1305625	cg13219552	cg13219552	30733342	ATAATTATATAAACAACTAAA			II	
0.1247442	cg13221924	cg13221924	60630370	TACATAATACAACCTAACTTTA			II	
0.1025322	cg13225565	cg13225565	53699406	TTCATCA	53630336	TTCGTCG	I	T
-0.111644	cg13230208	cg13230208	39736499	ATATATAAAATATAAACATCA			II	
0.188866	cg13232075	cg13232075	48807345	AATTATACCTTTAATTTTATAT			II	
0.1325462	cg13235098	cg13235098	55804302	AAAATATTCCTTCATAATACCA			II	
0.1346889	cg13238479	cg13238479	24692327	AAAAATAACACTCCTTAAACA			II	
0.1014943	cg13257498	cg13257498	39804309	AAACCCTAAAAATACTTTCAA			II	
0.106111	cg13257690	cg13257690	17725308	ACTACAC	48652391	ACTACAC	I	A
0.129644	cg13263323	cg13263323	60798335	AAAACCAAATCTATTAACAAA			II	
0.2434832	cg13264811	cg13264811	66687414	ACAACRAATACCACTCAACAA			II	
-0.15106	cg13270055	cg13270055	61675434	TTCTCCTTCTAAATACAATTAT			II	
-0.14484	cg13275129	cg13275129	67743403	TTTCCTTTCTCTTTCTCRTCTAT			II	
	cg13280524	cg13280524	13644367	RATAAACAAACRACTAAAATA			II	
-0.107088	cg13283952	cg13283952	28728410	CCACTAC	21693303	CCGCTAC	I	A
0.1085211	cg13293488	cg13293488	10679379	CCCATTC	45674507	CCCGTTC	I	C
0.100339	cg13295089	cg13295089	59763340	ACTTTCACCTTAAACCTAACA			II	
0.2271728	cg13305444	cg13305444	22750457	AACTAACATCTATACATAACA			II	
-0.106884	cg13306087	cg13306087	31664352	ACTTTATAACAATCTACTATAC			II	
0.1506625	cg13306815	cg13306815	19670468	CATTCATTTAATTCTAACTCAC			II	
0.1357067	cg13312387	cg13312387	71734364	ATTATAAAAATTCCRTCAAATA			II	
0.1201521	cg13315147	cg13315147	44686337	AAACACTAAATAAACCAACAA			II	
0.1146694	cg13316054	cg13316054	34636377	ACCAACAAATATTCAAAAAAT			II	
-0.152842	cg13316813	cg13316813	32773489	AACRTTTAAACCTCAAACCTTA			II	
-0.271197	cg13319761	cg13319761	44669307	ATATAACTACCAACTAACATA			II	

0.1049597	cg13321105	cg13321105	71768423	CCATATTCATTAATAAAATTCR			II	
-0.10231	cg13323878	cg13323878	43608313	AAACTAA	10720370	AAACTAA	I	C
0.186998	cg13327911	cg13327911	12684348	AAAATACATAATTAACACAAA			II	
0.1190469	cg13328209	cg13328209	57655330	CCCCAAC	42684337	CCCCGAC	I	A
	cg13330868	cg13330868	17795352	CAAAACT	20737304	CGAAACT	I	C
0.1081883	cg13332142	cg13332142	37674436	CACCACTCTCCATCCCCTCTAC			II	
0.1678149	cg13338949	cg13338949	36617380	TCCTCATAAAAAAAAACTTTC			II	
-0.107395	cg13340899	cg13340899	15707495	ATCCATATTATAACRTATATCC			II	
0.1083172	cg13347397	cg13347397	46672501	TAAATACTTAAAAAAAAATAAT			II	
0.1067142	cg13351860	cg13351860	43675447	AATTAATCAAAAAACTAAATC			II	
-0.207272	cg13353717	cg13353717	46781383	TAATACTAACRATACTAAACT			II	
0.1194713	cg13356253	cg13356253	42751506	AACCAAA	57649361	AACCGAA	I	A
-0.127603	cg13356896	cg13356896	63763425	AAATAAC	15707487	GAATAAC	I	A
0.2523034	cg13359998	cg13359998	29763477	TATACAACTATTACACAAAAC			II	
0.1056162	cg13360002	cg13360002	73724421	AACAAATTCATAACCTCCCRC			II	
0.1130767	cg13365400	cg13365400	61745505	TATCCTTAAAATAAATTTTCAT			II	
0.1193485	cg13366774	cg13366774	45710478	CTCCRAAAAACACTTACTTAC			II	
0.1118133	cg13366863	cg13366863	62607382	CAACAATCATATCAAAAAATA			II	
-0.121511	cg13367883	cg13367883	35644510	ATCCACRAATTATCRAAACCT			II	
0.1059225	cg13375463	cg13375463	62719409	TATTATCAAAACCTCTAAACA			II	
-0.332594	cg13376960	cg13376960	31640450	CTATATT	30715301	CTATATT	I	C
-0.110114	cg13377264	cg13377264	17690478	TAAAAACTCATAAAATCTCTA			II	
-0.221865	cg13380890	cg13380890	56660355	AATTCCTTAAAAAAATCATTA			II	
0.1245215	cg13381110	cg13381110	20648364	TCATAATAACRTATCRTAATA			II	
-0.100362	cg13391079	cg13391079	54702388	ATACAAC	63745430	GTACAAC	I	C
0.1202835	cg13400249	cg13400249	41720443	TTTTTCTTTTTACTCTTAATTTT			II	
0.3396693	cg13401893	cg13401893	47792379	CCTAATA	39659478	CCTAATA	I	A
0.1132346	cg13408027	cg13408027	50619367	ACACACAAAAACAACCTAAAA			II	
0.1233443	cg13411286	cg13411286	25692487	AAAAATCCTCATTAAACTCCA			II	
0.1171274	cg13413719	cg13413719	32644368	AATATTTAACCTCCCCATTCAT			II	
0.1162929	cg13415078	cg13415078	12740426	RAAAAACTATACTCACTCCCA			II	
0.1036989	cg13425960	cg13425960	30702347	CCTATAACCATTAAATAACAA			II	
0.1142529	cg13430678	cg13430678	64741438	ACTAAAAAATATACCTCTACT			II	
-0.497157	cg13431688	cg13431688	48649402	TAAAAAAATCAATATAATTAA			II	
-0.206108	cg13435137	cg13435137	39755502	CTTCCCACTTCTCTTAAAACRT			II	
0.1011602	cg13439946	cg13439946	16700406	TACTAAA	15807510	TACTAAA	I	T
0.1077918	cg13441107	cg13441107	25778496	TTAACTCCAAATAAACCCTAA			II	
-0.103671	cg13447295	cg13447295	62628313	AACAAAC	49742397	AACGAAC	I	C
-0.117145	cg13448197	cg13448197	43789481	ATTAAAA	12730440	ATTAAAA	I	T
0.267734	cg13452812	cg13452812	44787453	ATCCTCACAACTAAAAAAATC			II	
0.1121997	cg13454226	cg13454226	19696331	AAAAAAATAAAAATTTATCTA			II	
0.1213982	cg13457522	cg13457522	24638403	CTTTAAAAACATTAAACATAA			II	
-0.101898	cg13458005	cg13458005	27735482	CCAAAAT	36709474	CCAAAAT	I	T

-0.290445	cg13462557	cg13462557	37731366	AATATCAACAAAAAAAAAATTC			II	
0.1105103	cg13466917	cg13466917	38778399	CCATAAACTAATTCTAACCCRT			II	
0.1212844	cg13468214	cg13468214	41673352	CRTTCCTTTACAAAATCAACTC			II	
0.1574985	cg13471188	cg13471188	18756308	AAATTCTATCTCATACTATAAA			II	
0.108512	cg13471254	cg13471254	29732479	CATTTTTCAATAATTCAAAATA			II	
0.1031902	cg13471735	cg13471735	39803450	AACCTATATACTCCAATAATCA			II	
0.2156147	cg13474262	cg13474262	52638466	TTTAATACTATTCTAAACTCTA			II	
0.1109433	cg13484608	cg13484608	20602304	ATAAATC	57658429	ATAAATC	I	A
0.1560562	cg13490235	cg13490235	64762462	CCTCTACTAACAACACCAACT			II	
-0.107904	cg13496755	cg13496755	61637457	ATATAACTAACACATAAAAAA			II	
-0.240614	cg13498757	cg13498757	39792356	AAAACAC	49742459	GAAACGC	I	A
-0.103428	cg13501907	cg13501907	35694441	RCRAAATATATATAACCCCACC			II	
-0.120007	cg13506281	cg13506281	66615479	AACCAAATATACTAAACTTAA			II	
	cg13507893	cg13507893	13644391	ACATAAATAACRCTTACAATA			II	
-0.154879	cg13518366	cg13518366	12695312	CAAATATATCAAAAAAACCAC			II	
-0.149098	cg13520532	cg13520532	38730337	AAAACAA	33620350	AAAACAA	I	A
0.1722327	cg13520770	cg13520770	57808455	AATTTAATCTCRAAATACCTAA			II	
0.1194794	cg13521941	cg13521941	14787487	AATTACCTATCCCTTCAACTAT			II	
-0.17626	cg13523718	cg13523718	57794434	ACTTAAATCAAACTAAAATAA			II	
0.1434022	cg13523731	cg13523731	66664363	CATCCATTTTAAAATTCCTTAA			II	
0.1180043	cg13530320	cg13530320	66646497	TAAATAAACCAAATACTCTAA			II	
0.1390566	cg13531667	cg13531667	29803329	AAAAAAATAATACAATATTTA			II	
0.248572	cg13538571	cg13538571	57745326	CTTTCATTCCAAAAAACTACCC			II	
0.1314838	cg13541353	cg13541353	27690439	CCTCCCA	21763337	CCTCCCA	I	A
-0.101707	cg13541970	cg13541970	25616349	CTCCTCCRAACTCCRACAAATA			II	
0.1210057	cg13548554	cg13548554	46607455	CRTATTATAATTATCTATATAC			II	
0.146414	cg13551074	cg13551074	73747308	TACTCCTCCTATACTAAATAAC			II	
0.100659	cg13564459	cg13564459	21762471	ATCTCAAAAATTAATACTCCT			II	
-0.237732	cg13564529	cg13564529	11616419	TCCCTAATAAAATAAAACCRA			II	
-0.176583	cg13566468	cg13566468	63681301	ACCACCTTCTCTACAAAAAAT			II	
0.1454232	cg13570892	cg13570892	72672431	AATAACTACTTAACCTTAACT			II	
-0.221191	cg13573375	cg13573375	46721383	CAACAAAACACAACAATAAAA			II	
-0.110948	cg13578160	cg13578160	67712382	TAACATT	70778385	TAACGTT	I	A
0.10026	cg13580286	cg13580286	34644314	TACAATCTTAAATTAAATCTAA			II	
0.1488151	cg13580758	cg13580758	74636371	CAAAATTATTATTACTTCACTA			II	
-0.11584	cg13581155	cg13581155	64768399	ATATTACCACCCRCCAAAACT			II	
0.3276251	cg13582457	cg13582457	41775406	AATATAAAAACCRAAAAATTC			II	
0.1033688	cg13583664	cg13583664	73671306	AACATAA	66714354	AACATAA	I	A
-0.331514	cg13585749	cg13585749	49600485	AAAATCA	60740487	AAAATCC	I	T
0.1022698	cg13586457	cg13586457	41752475	AATTAAAAACACRATATTAAC			II	
-0.101372	cg13593391	cg13593391	64652509	TAAACCT	60798489	TAAACCT	I	A
-0.106487	cg13596370	cg13596370	59628448	CTCAATT	62637351	CTCAATT	I	A
0.1207629	cg13601993	cg13601993	39620393	ACAAAAT	36651379	ACGAAAT	I	A

-0.205535	cg13603318	cg13603318	73652478	CAATACTTTTCATATTAAACCT			II	
-0.128517	cg13603879	cg13603879	13715484	AAAAAA	18624335	AAAAAA	I	A
-0.112709	cg13606025	cg13606025	73649470	ACAATATTAACCAAACCTAAT			II	
0.1077104	cg13610138	cg13610138	21652347	AAAAAATACRTTCTCTTTACTT			II	
0.14392	cg13610455	cg13610455	51627488	AAACCCAAAAAAAACTCTATA			II	
-0.190355	cg13615998	cg13615998	23611369	ACATACA	70657374	ACATACG	I	C
0.113174	cg13617603	cg13617603	28731396	AATTAATTATAAATATCCAAC			II	
0.1100656	cg13620034	cg13620034	64629354	AAAATACTAATAAAATCTTAA			II	
-0.105479	cg13622893	cg13622893	18704435	ATCCRCTTCCRATTCCCAAACT			II	
0.1515456	cg13631318	cg13631318	47616465	ACCAAAAACAAAACAAACCTT			II	
0.1748874	cg13641645	cg13641645	49614330	CTCTCATAACATTTATACTCTA			II	
0.1000357	cg13649864	cg13649864	13798483	ACTCTAAAACCTACACCTCTC			II	
-0.177471	cg13651207	cg13651207	20695422	AAACCAACTCCTACCCAATAT			II	
0.1111556	cg13652008	cg13652008	73691307	TTATTAAATCACTATTTACAAC			II	
0.1137869	cg13655986	cg13655986	25619417	CCTTAATCCAATTTACRACATT			II	
0.328109	cg13661648	cg13661648	70649379	TAAAAACTTCCAAAATCCCCC			II	
0.1069779	cg13670288	cg13670288	63774398	TAAACTA	63803416	TAAACTA	I	C
-0.122547	cg13671412	cg13671412	15647332	AACATAAAATACTATAAATAA			II	
-0.216762	cg13672200	cg13672200	45645324	CACAAAACCRTAAATCATAAC			II	
-0.242391	cg13672736	cg13672736	20690433	CATACCA	10609506	CATACCA	I	C
0.1104285	cg13683939	cg13683939	11708424	TACRAATTTAATAACTCCAAT			II	
0.1263687	cg13684379	cg13684379	46808320	ATAAAAACACTACAACTTAAA			II	
-0.21526	cg13685349	cg13685349	31701488	AAAAACTAACTAACTACAACR			II	
0.1289489	cg13685506	cg13685506	45677454	TCATTAACACAAAACCTATTT			II	
0.106768	cg13686739	cg13686739	15614366	TATATTATATACAAAAATACR			II	
-0.121779	cg13688186	cg13688186	47755386	AATAATTCTATCAAAAACATT			II	
0.1111861	cg13690543	cg13690543	20743482	AAACTAAAAATAACTCRTTCA			II	
0.1001187	cg13694680	cg13694680	73687362	ATATCCTTCCACCTATACTTTA			II	
0.1036807	cg13698168	cg13698168	64769494	AACATAACTCACTCAAACAAC			II	
-0.101209	cg13699152	cg13699152	55619346	TCAAACA	65658365	TCGAACA	I	A
0.1129639	cg13700458	cg13700458	27626458	ATATAAACRCCTAACCTAACC			II	
-0.131524	cg13706058	cg13706058	10640380	AACACCA	67719307	AACGCCA	I	T
0.1577004	cg13707567	cg13707567	33610388	TCACTACTTTTATAAATTCCCT			II	
0.1503538	cg13711314	cg13711314	52794450	TTAAAAC	19687301	TTAAAAC	I	A
-0.174839	cg13713218	cg13713218	57666459	ACTTAAC	24714498	ACTTAAC	I	A
0.1179686	cg13716760	cg13716760	12705336	AAAAAA	65645460	AAAAAA	I	A
0.1687208	cg13724111	cg13724111	64661376	AAAAAATAAAACAAATAACTT			II	
0.1206754	cg13724550	cg13724550	73734496	ATAATCTAACTAAATCACACT			II	
-0.193314	cg13725087	cg13725087	46769405	AAACCACTTCTCRCAAAATAC			II	
0.1009365	cg13727946	cg13727946	62699392	CCTCCCATTAATAATATCTAAC			II	
0.1163365	cg13728003	cg13728003	70610397	AACTTCRCAATTATTTCCACTT			II	
-0.155853	cg13729816	cg13729816	60602308	CTAAATA	17792313	CTAAATA	I	A
-0.193853	cg13735469	cg13735469	40754500	AAAAAAC	46743332	AAAAAAC	I	T

0.1183093	cg13738729	cg13738729	20662462	TAAACTCAAAAAAACCTTAAC			II	
-0.418405	cg13740636	cg13740636	37775382	TCTAACATCAATCATTTATCAA			II	
-0.291734	cg13748354	cg13748354	14625475	CRTACACTAACTCTCTAAAAA			II	
-0.141205	cg13749113	cg13749113	27651417	TATCTAA	15717451	TATCTAA	I	C
0.4493738	cg13749548	cg13749548	70725351	AAAAAA	59636392	AAAAAA	I	T
-0.272249	cg13752726	cg13752726	14643338	RATTCCCACTTATACRATAATC			II	
0.1054784	cg13753613	cg13753613	41669312	AATTACTTAAAAACATATTAT			II	
0.1081318	cg13754355	cg13754355	66771492	CCTAAATAAAAACTCTAAAAC			II	
	cg13755512	cg13755512	13644358	AAATAA	50618434	AAATAA	I	C
	cg13769916	cg13769916	50803368	TTAAACTCCAAAATCTAAATC			II	
0.109796	cg13771797	cg13771797	67666468	CAAACAA	63745415	CGAACGA	I	C
0.1138428	cg13772218	cg13772218	48807457	TCCACAT	25694326	TCCGCAT	I	C
0.1837902	cg13773305	cg13773305	32769322	AAAAACRTAACAAAATCCTAA			II	
-0.101623	cg13777717	cg13777717	57708408	CAAAACC	30628336	CAAAACC	I	A
-0.104269	cg13778567	cg13778567	69709303	ATTTCCTCACCACAAAAATTC			II	
0.1336305	cg13783191	cg13783191	21682334	CTAACAAAACTAAAACCTCTA			II	
0.1338842	cg13794404	cg13794404	41639341	CAATAAAATTCCCCAAAAAAA			II	
0.1328291	cg13797468	cg13797468	73751365	ATCTATAAATCTATTTTTATTA			II	
0.1209005	cg13798745	cg13798745	18768427	ATTCCTAATTTCTAATATCTCT			II	
0.1471436	cg13806489	cg13806489	44707417	ATTTTAAACTCTCCAAACRATT			II	
-0.223893	cg13808036	cg13808036	47649309	TATCCTTACAACTATCAATTCR			II	
0.1025663	cg13817037	cg13817037	67727454	AATAAATTTCTTTCATCCTAAC			II	
-0.250866	cg13821051	cg13821051	40694387	AAAAACATTTTACAAAATCCA			II	
0.1078082	cg13825288	cg13825288	31738487	CCAACCTATAAATTTCCATTAC			II	
-0.113705	cg13825930	cg13825930	15798480	ATCTCAA	33600463	ATCTCGA	I	A
0.1655418	cg13829104	cg13829104	60688420	RTTATAAATTTCACAATTCTCR			II	
-0.204828	cg13829625	cg13829625	59605469	CTCRAATACAAATAACTCAAA			II	
0.118173	cg13829849	cg13829849	55793300	ATATATACCAAAAAAAAATAC			II	
0.1818737	cg13830619	cg13830619	56803382	TTTCCCC	37715327	TTTCCCC	I	C
-0.122032	cg13842639	cg13842639	66679384	AAAATTC	27632433	AAAATTC	I	A
-0.130004	cg13847443	cg13847443	17788362	ACCAACAAACATATAAATAAA			II	
0.1473023	cg13857217	cg13857217	19772354	CAACCAAAACACAAAAAACA			II	
-0.214016	cg13857646	cg13857646	47803479	CTCAAACRACTATAACRACAA			II	
0.1077371	cg13859771	cg13859771	57630509	ACAAACATTAATAAATCTAAA			II	
0.1094849	cg13860281	cg13860281	15706467	TTACAAATATAAATACACACA			II	
0.1680824	cg13866263	cg13866263	69686405	ATCTCTT	30663316	ATCTCTT	I	A
0.1001879	cg13870539	cg13870539	25770477	AAAACCTCTATAAAACATAAC			II	
0.1375992	cg13874425	cg13874425	52603344	CCTCTTT	64705354	CCTCTTT	I	A
-0.343148	cg13874759	cg13874759	73714364	CATAATA	10734352	CGTAATA	I	C
0.1642688	cg13877915	cg13877915	73751347	ATCCTTCTAAATAATATAATTT			II	
-0.101817	cg13878646	cg13878646	29736459	CTTTAATTAAAACACCCCTAAC			II	
-0.168612	cg13885788	cg13885788	52680473	TTCCAACAAAACTCTACAATA			II	
0.1166318	cg13894852	cg13894852	52642474	AAAATAA	62668499	AAAATAA	I	A

0.1102747	cg1389565(	cg1389565(	22740391	TATTATCTATTAATATTATAAC			II	
0.2145148	cg1389734{	cg1389734{	53632377	CTACTTATCTAAAATACTACA/			II	
0.1948507	cg1389738{	cg1389738{	15654377	ATTACRAAACTAAAATACATC'			II	
-0.131357	cg1390077:	cg1390077:	15714346	TCAACAA	48699432	TCAACGA	I	T
-0.172407	cg1390264:	cg1390264:	65734370	CTTCTAA'	23712404	CTTCTAA'	I	C
-0.110636	cg1390529{	cg1390529{	20689494	TACATACATTTTAAAACCAAA(			II	
0.1401321	cg1391078:	cg1391078:	34779364	ACTACACTCTATACTTTCCAA/			II	
-0.228221	cg1391399(	cg1391399(	33706467	CAAATTCACCTACAAAAAAAC			II	
-0.151189	cg1391572(	cg1391572(	52735355	AAAACAAAAACAACTAATATA			II	
-0.106257	cg1391663:	cg1391663:	18655427	TAACAATAAACAATTACCCTT			II	
0.1294536	cg1391875<	cg1391875<	21719430	CACAACT	20681309	CACAACT	I	T
-0.162467	cg1392337l	cg1392337l	59739471	AACCAA/	61650427	AACCGA/	I	A
-0.133475	cg1392428'	cg1392428'	58759478	TAAAATAAAACTCCCCCAAAC'			II	
-0.138283	cg1392463:	cg1392463:	48740453	ACTAATT	71705427	ACTAATT	I	T
-0.123679	cg1392471:	cg1392471:	44757399	TTCCCAT.	34704415	TTCCCAT.	I	C
0.1137192	cg1392580(	cg1392580(	54709451	AAAAATAAATCACACCTCCTC(			II	
0.1016464	cg1392750<	cg1392750<	24795399	ACTCTATTCTTATCCCCCATAT			II	
-0.103176	cg1392847:	cg1392847:	73643338	CCTAAAAAATCCRACAACTCT'			II	
0.1508679	cg1393125(	cg1393125(	43640424	ACTCAAACACTTATTATCCTAT			II	
0.2015792	cg1393499(	cg1393499(	53634405	TATTAACRCTAATCCTCATATT			II	
-0.118328	cg1393762'	cg1393762'	67746374	AATATAT	52673303	AATATAT	I	A
0.1249536	cg1394197{	cg1394197{	27797357	ACTAATT	68657390	ACTAATT	I	T
-0.11841	cg1394333:	cg1394333:	68725365	ATACATTAAAATAATCCCCTA/			II	
0.1328613	cg1394450'	cg1394450'	44749313	CCAACCAATCRTAAACTACRA.			II	
0.1123897	cg1394557(	cg1394557(	18780428	TCTCTATTTACCAAACATCAAT			II	
0.1058095	cg1395429:	cg1395429:	14722349	TCTATATTTTAACTACCRACTA			II	
0.1034998	cg1396561:	cg1396561:	73643485	TAATATAATAAAATCAAAACA			II	
0.118005	cg1396932'	cg1396932'	70696502	TATCTTCTCATTATATACCCCT(			II	
-0.109082	cg1396966:	cg1396966:	59724471	AACACCA	67729467	AACGCCC	I	C
0.1138344	cg1397150<	cg1397150<	63642379	AAAAATTTACAACCTACATTC/			II	
0.1162762	cg1397242:	cg1397242:	15728324	TACTAATATTCATCCTCACCTA			II	
-0.121973	cg1397266l	cg1397266l	29688442	TAAAAAATATACACCTAATAC'			II	
-0.154734	cg1397456:	cg1397456:	71763399	TCATCCC'	56810505	TCATCCC'	I	T
0.1416755	cg1397958l	cg1397958l	62695310	ACTTAAATCAAACTATCAAAA.			II	
0.2243201	cg1398203'	cg1398203'	67788333	AAAATACTTTAAAAACTAAAC'			II	
0.1124112	cg1398428(	cg1398428(	74759420	CAATAATAACCCTAAAAACRA			II	
-0.165676	cg1398929:	cg1398929:	58727359	AAACCACTTTTAATACTAAAA/			II	
-0.140749	cg1399318:	cg1399318:	44692337	ATATTTT.	66773386	ATATTTT.	I	C
0.2983239	cg1399473(	cg1399473(	32778500	AAAAACTCCAACRAAATTTAT.			II	
0.1263556	cg1399542'	cg1399542'	31735405	ATAAAA/	17760352	ATAAAA/	I	T
-0.121637	cg1399692:	cg1399692:	58622429	AAAACTAAACAACATCATCRA			II	
-0.150478	cg1400102:	cg1400102:	47714395	AAAACAT	23603428	GAAACAT	I	A
0.1036409	cg1400990(	cg1400990(	60778356	CRTAACTAATTAAACCCACTA(			II	

-0.134146	cg1401359	cg1401359	15685493	TCCCAAA	14745503	TCCCGAA	I	C
0.1148121	cg1401550	cg1401550	17614455	ATAAACCTTTATACTATACA			II	
0.1142044	cg1401836	cg1401836	55785372	AAACRACCCATAAAACRAAAC			II	
0.1045677	cg1402117	cg1402117	63800497	AAAATAATTTTATTCTCCCRCC			II	
-0.126425	cg1402489	cg1402489	14639392	CCCATCA	39722331	CCCGTCG	I	C
0.1320175	cg1402610	cg1402610	56747341	ACAAACTACTTCAAAAAAAAA			II	
0.1486019	cg1403226	cg1403226	16779407	TTATCTCAAACCTACAAAAAC			II	
-0.11527	cg1403524	cg1403524	61732499	TTATAATCCCCAAAACAACCC			II	
-0.109459	cg1404181	cg1404181	66763350	AAAAAACATAAACCTAATATA			II	
-0.146695	cg1404416	cg1404416	13767316	ATAAAAAAATACATATACCTA			II	
-0.541571	cg1404466	cg1404466	49635493	TACTCAATTCCCTTATAAATTA			II	
0.1300275	cg1404756	cg1404756	13705510	AAATATAAAAATTTACTAAT			II	
-0.124477	cg1405223	cg1405223	27659421	AAAAAA	50627420	AAAAAA	I	C
0.1064289	cg1405427	cg1405427	48704449	AATCATTACCCTACCTTTAAA			II	
0.1095803	cg1405500	cg1405500	23702406	ATTAATAAACTACRTAAATCA			II	
0.2402989	cg1405684	cg1405684	70781354	AAACCAAACTAACTATAACCA			II	
0.1068234	cg1405983	cg1405983	34704426	CTTTCTAAAAAACCATTCCRA			II	
-0.254386	cg1406011	cg1406011	10718499	AAAACCC	11806350	AAAACCC	I	A
-0.124837	cg1406552	cg1406552	69708390	AACCACRCAAAATATCTAACA			II	
-0.10052	cg1406559	cg1406559	62675467	AAAATCCTTAAACCACAAAAA			II	
0.1125072	cg1406752	cg1406752	50729438	ATAATTACCACRACAAAAAAT			II	
-0.343376	cg1406818	cg1406818	49793437	TATTTATTTTATTTAATTATTA			II	
0.1132433	cg1406878	cg1406878	35601496	ACTTAACATTCCAAACTCTTAC			II	
-0.115075	cg1407545	cg1407545	74698433	ATATTCA	10612313	ATATTCA	I	T
-0.106191	cg1407935	cg1407935	32652312	TAAAAAA	66804337	TAAAAAA	I	T
-0.2064	cg1407946	cg1407946	47692328	ATACCCAAATACRAAACCAAA			II	
0.1168701	cg1408166	cg1408166	28661448	AAAAACCTAAATACTCCRAAA			II	
-0.132781	cg1408560	cg1408560	28693342	CAAAACA	52792431	CAAAACA	I	T
0.1004266	cg1408741	cg1408741	64747435	ACCCCCACAATACCTCATTCT			II	
0.119489	cg1408904	cg1408904	16772482	AACTAAATTTATCTAACRAAA			II	
0.1776106	cg1408910	cg1408910	36734471	AACATTCAACTTCTAAACAAA			II	
-0.12179	cg1408926	cg1408926	67756328	TAATAAT	26777382	TAATAAT	I	A
-0.113468	cg1409299	cg1409299	42766372	ACTAATTCCTTAAATCCTACA			II	
0.1299348	cg1409618	cg1409618	47710489	CAAACAAAAAACAACAAAAA			II	
-0.183471	cg1410055	cg1410055	42714442	AAATAAACCCTAATATCTATC			II	
-0.358875	cg1410470	cg1410470	24740407	TAACAAATTTCAAATCTACAA			II	
0.1084327	cg1410553	cg1410553	23766507	ATAAAAATCCAAAAAACCCAA			II	
-0.151557	cg1410578	cg1410578	54737451	RAAAACAATTAACRACCTACA			II	
-0.101658	cg1410668	cg1410668	47642418	ACAACAA	64766423	GCAACGA	I	A
-0.207326	cg1410856	cg1410856	29795429	AACACTT	13648308	AACGCTT	I	T
0.2324384	cg1411016	cg1411016	56687339	TAACCAACATAATAAAACCTT			II	
-0.127288	cg1411633	cg1411633	41652368	CAAAATA	54719324	CAAAATA	I	A
-0.10871	cg1411676	cg1411676	30664401	AAAATAT	39692389	AAAATAT	I	A

0.1018599	cg1412174(	cg1412174(	40715491	TAATAATTATAATATCCATCA/			II	
0.1449318	cg1412240:	cg1412240:	36711497	CCCAAAAAATAAATAACTCAA			II	
0.1293283	cg1412303⁄	cg1412303⁄	72770339	RCCTCCTCTCCAAAACCCACA/			II	
0.1077601	cg1413262(	cg1413262(	19770390	ACCTCCTAACAACCCTATTAA/			II	
0.1133833	cg1413310(	cg1413310(	27738492	AAAATTACTATTTAACATTTTA			II	
0.1144512	cg1414898	cg1414898	52708325	TAACCTACATTAAAAATTAAC			II	
-0.233957	cg1415106:	cg1415106:	69684374	TCCTTTAAAAACTTTCRTATTA			II	
0.1336976	cg1415246:	cg1415246:	26803312	ATATAACCATAAAACACAAAA			II	
0.1337196	cg1415297⁄	cg1415297⁄	61620504	AAACCAAACTCTCATAAAATT.			II	
-0.131732	cg1415752	cg1415752	32645447	AAAATCTCCTAATTAATTAAT/			II	
0.1078842	cg1415855(	cg1415855(	49786440	AAAAACTCTACACCCCATCAT			II	
0.1193378	cg1416043:	cg1416043:	22798367	CTACTACTCCTAATAAAAAAT/			II	
0.1303345	cg1416670	cg1416670	59676319	AAATATA	27655364	AAATATA	I	T
0.1526033	cg1416703:	cg1416703:	72735486	ACRAACTCTTAAACCRTACAA			II	
0.1618643	cg1417020	cg1417020	66762308	CCCTTTTCCCTCCTAATATCTT			II	
0.1026493	cg1417422	cg1417422	12618450	CCTCTAAAAACTCACCTCTCA(			II	
	cg1417708⁄	cg1417708⁄	13644487	TTAAACTAAATAATCCTTACT/			II	
-0.150027	cg1417928(	cg1417928(	57618362	AACTATAATCTCACAATTTAC(			II	
0.1092698	cg1418041:	cg1418041:	54715503	TAAAATCACACAACATCTATA.			II	
0.124089	cg1418495⁄	cg1418495⁄	18710350	AACTTCCTATTCCACCAATAA/			II	
-0.108778	cg1418781:	cg1418781:	38654363	ATAACTAATATTTTCTTTCCAA			II	
-0.108826	cg1418877⁄	cg1418877⁄	21618351	ATTCCCAAAATAATAAATTTT/			II	
0.1601783	cg1418911(	cg1418911(	57754348	TCTAAATCATCAAATCCTACA/			II	
0.1019802	cg1418953⁄	cg1418953⁄	16783506	ATTAACTCTATCTCTCAATCCA			II	
0.1267138	cg1418978:	cg1418978:	48730327	TATAAATCCCCRATTTCCTAA/			II	
-0.102527	cg1419229(	cg1419229(	66716342	ACTAACC	54642479	ACTAACC	I	A
0.1152477	cg1419517(	cg1419517(	26730510	CRAAAATTTTAAACTACTTCC			II	
0.1139746	cg1419711:	cg1419711:	18656448	TATAATTTCCTTCTCAAACTTT			II	
0.143919	cg1419822	cg1419822	55768438	AACCCACTACTTTCCTTCTAAA			II	
0.1677924	cg1420142⁄	cg1420142⁄	33795429	CTAATAA	24648384	CTAATAA	I	C
-0.125573	cg1420408	cg1420408	63639347	AATAAAATCTCCATCCTTATC(			II	
0.1470542	cg1420951(	cg1420951(	40741482	TAAAAACCTAAATAAAACTTT(			II	
0.1383093	cg1421203(	cg1421203(	33638311	TAAACCTAACAAATTTTAAAC.			II	
-0.111679	cg1421339⁄	cg1421339⁄	67685329	CAACTCA	33758460	CGACTCA	I	T
-0.104313	cg1421473(	cg1421473(	54746417	CAATAAA	31784456	CGATAAA	I	T
0.1278031	cg1421606(	cg1421606(	38746430	AAAAACT	28745335	AAAAACT	I	A
-0.103536	cg1422367	cg1422367	38743305	ACCATATTAAAATCRAACAAA			II	
0.1401159	cg1422732:	cg1422732:	42669375	ACTATATCRACRAATTTCTTCC			II	
-0.376759	cg1422810:	cg1422810:	31747469	TTATTTTCTCAAACACCAATTA			II	
0.1065378	cg1423491:	cg1423491:	14768483	AAACATCACAAACCACTTAAA			II	
0.3176209	cg1423812(	cg1423812(	69799509	AAACAAATAATAAAACTCCAA			II	
-0.11048	cg1423965:	cg1423965:	15745447	CTAAAACTAAAACTAAAACTA			II	
0.1135114	cg1424079(	cg1424079(	66778462	ACATTAAAAAAATAACRTAAC			II	

0.1282591	cg14241836	cg14241836	55738497	TAAATTTCATAACCCACRAAT			II	
0.1063199	cg14245898	cg14245898	56606470	TTAAAACACAACTACTCACAA			II	
0.1222877	cg14252237	cg14252237	20612393	CCTCCTACRAAAACAAAAACC			II	
-0.186759	cg14253596	cg14253596	36807492	CCTTCCCTAAAAAATATCAAT			II	
-0.104831	cg14260773	cg14260773	44645465	AATTAACATCATAAACCAAAT			II	
-0.185071	cg14266237	cg14266237	69689428	AAAAAAT	36775365	AAAAAAT	I	T
-0.133144	cg14270002	cg14270002	69655308	AAAACAA	36605363	AAAACAA	I	T
0.1712371	cg14271023	cg14271023	64732448	RAATTACCCAACTATAATAAA			II	
-0.146912	cg14276083	cg14276083	12607466	ACATTCC	35645443	ACGTTCC	I	C
0.1312933	cg14277298	cg14277298	28755433	TATTACAACTACCTCCCTCTTC			II	
0.1103968	cg14281039	cg14281039	65637437	TTCCTATACCCRAAAATAACT			II	
0.1095645	cg14283647	cg14283647	50728485	CCTAAAATACAACRATAACCA			II	
-0.105903	cg14283756	cg14283756	10749446	CCCCAAA	42607466	CCCCAAA	I	A
0.1076598	cg14288049	cg14288049	27600478	ATCAAAATAATATTACAAAAC			II	
0.1501856	cg14290070	cg14290070	13604340	TAAATTCTTTTTCTTATTAAA			II	
0.378945	cg14293674	cg14293674	29710310	TCATTATCACCCAAAATCCAT			II	
0.200318	cg14294953	cg14294953	42717353	AATACAAATATCAACTCCATA			II	
-0.213943	cg14294971	cg14294971	27708424	ACACAAAACACTATCAAATAT			II	
0.4619071	cg14302130	cg14302130	11677360	ATCTCCCCRCTCTAAAATACT			II	
-0.131176	cg14307477	cg14307477	14724497	TACAAAC	57715430	TACGAAC	I	C
0.1320259	cg14308311	cg14308311	58623424	TTTATCATCTTAATTATTCTAA			II	
0.179794	cg14308648	cg14308648	38611396	AAACATCCAATAACTATAAAA			II	
-0.145177	cg14310877	cg14310877	55658463	TTTCCRATTTTCCTACCCTTTT			II	
0.1673117	cg14316629	cg14316629	66809447	TAATCAACRTTACCAATAAAC			II	
0.1602327	cg14316660	cg14316660	45791404	CCCTTTA	71766344	CCCTTTA	I	A
-0.105078	cg14317384	cg14317384	74681441	ACCTACA	19676415	ACCTACG	I	A
-0.115644	cg14323204	cg14323204	16761452	CCCTAAATATTACTAAATCAA			II	
0.2460178	cg14323910	cg14323910	22757305	ACTACTACCTATAAACATTTC			II	
-0.130374	cg14324816	cg14324816	42657509	ATATCACAATCACAAATTAAA			II	
0.1213388	cg14324838	cg14324838	24619461	AACTCTATATTCAAAAACAAA			II	
-0.135906	cg14325153	cg14325153	62672390	TCAATAA	63721444	TCGATAA	I	C
-0.110638	cg14325184	cg14325184	27635449	CACTAAC	25748318	CGCTAAC	I	C
0.1030766	cg14325779	cg14325779	50772323	CCATAATATATTTATCATCTCC			II	
-0.203404	cg14329783	cg14329783	52733448	CAAAACAAATACCTCTAAATA			II	
0.1295226	cg14329889	cg14329889	38785507	CAATCAA	32800433	CGATCAA	I	C
0.1076887	cg14330868	cg14330868	63688403	TCCTCRAAACTATATCTAAAA			II	
-0.155056	cg14331206	cg14331206	35601349	AAAAACACAAAACRTTCTAAC			II	
-0.145189	cg14331226	cg14331226	36734408	ATTACTCCCTAAACCACCTAA			II	
0.1480138	cg14339466	cg14339466	37733330	ACTTTACAAATCTACTTAAAA			II	
0.1457422	cg14340463	cg14340463	66713386	TTAAATACCCCATTAAATAAA			II	
-0.148355	cg14340928	cg14340928	43655507	ATCAACATAACTACTCAAAAA			II	
-0.119367	cg14352508	cg14352508	70794396	AAACRCCTACCACCATACCCR			II	
0.1067192	cg14356919	cg14356919	39662411	CTAAATATAAATAACRCRCTA			II	

-0.160299	cg14361252	cg14361252	20779300	AAAACAC	43644329	AAAACGC	I	C
	cg14373189	cg14373189	54713385	CCTTAACTTACCCAACTTAATT			II	
-0.132342	cg14378564	cg14378564	49632458	CAATATACCAAACTCCATTCTA			II	
0.102601	cg14384416	cg14384416	36707502	AACTAACRAATTTCAAAACCRA			II	
-0.111842	cg14388237	cg14388237	21625490	ATAAAAAAAAATCTCCTATACT			II	
-0.126996	cg14395298	cg14395298	46769505	CCTTCCCA	22755310	CCTTCCCG	I	T
0.104918	cg14402224	cg14402224	60720388	AACTATAACTAACAAATAACA			II	
0.2525862	cg14408831	cg14408831	71685333	AAAAATATCTCRTAATCCAACT			II	
0.1159485	cg14414911	cg14414911	28722498	TAACACAATACCACAAACTTA			II	
-0.14736	cg14417784	cg14417784	18609379	CAACAAA	17793438	CGACGAA	I	A
0.1107551	cg14420715	cg14420715	40655403	AACTTACCTTAATATACCCAA			II	
0.1575126	cg14422240	cg14422240	28641377	ATCATTTAAATTTTAACATAAT			II	
0.2292498	cg14422500	cg14422500	64736341	TTATAATTAAAAAATCCTTTCT			II	
0.1285216	cg14425564	cg14425564	65709411	AATTCAA	11727418	AATTCAA	I	A
0.232985	cg14429748	cg14429748	38615468	ATAAAAATACACACACACACA			II	
0.100367	cg14431577	cg14431577	17720381	AATAACTAATACTACAAATATA			II	
0.1200539	cg14432251	cg14432251	73751357	AATTAATTTTAAATAAACACA			II	
0.1082242	cg14434755	cg14434755	67710342	TACRTCTTATCCACTTCTACAA			II	
-0.100843	cg14435550	cg14435550	10673464	CCTTTAA	18684454	CCTTTAA	I	A
0.1061591	cg14437446	cg14437446	27759462	TTATTCACCCTCAAACAACAAT			II	
0.1413494	cg14440272	cg14440272	14740455	AACTCCCATAATTAAAAATATA			II	
0.1298694	cg14454094	cg14454094	25606396	CTCCTTATATTATTCTTAAACT			II	
0.1026263	cg14457452	cg14457452	28653324	AACTAAATAAACACCACAATA			II	
0.1161119	cg14462402	cg14462402	22679490	AATTCAATACATAACTAAACC			II	
-0.157722	cg14463736	cg14463736	31733355	ACACTTAACCCAACTTAATTTT			II	
0.1058856	cg14467015	cg14467015	53763328	CATTAAAACCTCACAAAATTTA			II	
0.112606	cg14467415	cg14467415	17628308	AAACCCCAAATTTATCACACA			II	
0.2225897	cg14476852	cg14476852	36706322	TAAATTCAAACTTCCTAAATAC			II	
0.1018388	cg14485643	cg14485643	74770465	CCCTAATCACATAACCAATCRA			II	
-0.176993	cg14493094	cg14493094	40603491	ATTTAAAACTCTATAAAAACCT			II	
0.1938568	cg14497649	cg14497649	38770382	TAAAAATATAAATAAAAAACT			II	
-0.138267	cg14500486	cg14500486	70643436	ACACAAA	26786310	ACGCAAA	I	T
-0.16583	cg14506194	cg14506194	53678387	RTCATACTTATACCTCCCAAAT			II	
-0.130422	cg14515364	cg14515364	57740498	TCAAATT	21692339	TCGAATT	I	A
0.1001119	cg14519356	cg14519356	71655353	TATATATTATTTATTTCCATAA			II	
	cg14521995	cg14521995	11608485	AAAACAC	70606468	AAAACAC	I	C
-0.102857	cg14522441	cg14522441	38809464	CACCACA	64726319	CACCACG	I	C
0.1501022	cg14525390	cg14525390	54717343	ATAATAACTCACATATACTAA			II	
-0.147621	cg14531093	cg14531093	48658334	ACCACAT	23714502	ACCGCGT	I	C
-0.143778	cg14534987	cg14534987	67687408	AAAAACA	15645431	GAAAACC	I	A
-0.105987	cg14535332	cg14535332	26651379	CCCAAAA	56756319	CCCGAAA	I	A
0.1382557	cg14548901	cg14548901	45722301	CCATTTTTTAAAAACCAAACAC			II	
-0.110894	cg14550066	cg14550066	67643448	AACATCRCTCAAATTCTACCRA			II	

0.1147055	cg1455344	cg1455344	68711481	AAACTCCACAAATATCCTCAA			II	
0.122054	cg1455913	cg1455913	59614346	ACTAAAATAACAATAACAAAA			II	
-0.102637	cg1456278	cg1456278	49608347	AAATTAATAAATCTCCTACTTC			II	
0.1556566	cg1456942	cg1456942	25733453	AACCTAATTTTTAATATAAATT			II	
-0.10731	cg1457381	cg1457381	56676465	RACCTTCTAAAATATCTCCTAC			II	
0.1067245	cg1457770	cg1457770	65723423	CAAATTTAATAACAATCTTATA			II	
0.255098	cg1458399	cg1458399	21607472	CCAAAACCCCCTTATAAAACA			II	
0.2266372	cg1459263	cg1459263	33702337	ATACACAACATACTCCCACATA			II	
0.1927962	cg1459786	cg1459786	40691394	AACAACAAAACCACTAACCRT			II	
0.1463231	cg1459833	cg1459833	31791482	CAAAACC	59746308	CGAAACC	I	C
0.162829	cg1459847	cg1459847	54608430	RAATAACAAATAACCAACATT			II	
-0.111214	cg1460447	cg1460447	29608370	TTAAAAAAAACTCTTAATAAT			II	
0.1887338	cg1460452	cg1460452	48802381	AATATCC	62611436	AATATCC	I	A
0.1116987	cg1460535	cg1460535	29713486	TAAAATCTTATACCCTTTCTAT			II	
-0.112425	cg1460877	cg1460877	49753305	ATCACAA	39714411	ATCACAA	I	T
-0.227416	cg1460910	cg1460910	52683440	AAAAAAACTAAAATTCACTTT			II	
0.132916	cg1461145	cg1461145	52757336	ATATATCTACCAAAAATTAAC			II	
-0.184745	cg1461526	cg1461526	69601336	TCTCTCA	40624490	TCTCTCA	I	A
-0.152369	cg1462190	cg1462190	48627421	AAAACCTTAAACCCCCCATAA			II	
-0.116734	cg1462563	cg1462563	10802457	ACRAAAAAACTTAACCACCTA			II	
0.1902223	cg1462901	cg1462901	15711375	AAAATCCTAAAAAAACAATAT			II	
0.1006683	cg1463063	cg1463063	21656316	AAAATAATAATAAATACTTTA			II	
0.124501	cg1463626	cg1463626	25713488	AACAAAAAATATACACAACCR			II	
0.1016078	cg1464233	cg1464233	21795454	CTTCCTATAATCCCCACRATAA			II	
0.1017089	cg1464326	cg1464326	24747360	CCAATTTCCCTACTTCATCAAC			II	
-0.154688	cg1464524	cg1464524	64690436	TATAAAA	36600482	TATAAAA	I	A
	cg1464664	cg1464664	67606429	CATCCTATCTTAAACCACTAA			II	
0.1157508	cg1465143	cg1465143	32620478	CRAACTAAAACATAAATTCAA			II	
0.1223924	cg1465359	cg1465359	60626498	TTCTCAACAATCACTTACTTAA			II	
0.1267193	cg1465417	cg1465417	51679367	ATCACRAATACATTTTACCTAA			II	
0.1065799	cg1465912	cg1465912	74709337	ATTCTCRACAAACCAAATCCTA			II	
-0.104909	cg1465934	cg1465934	38621481	AACAACA	24713432	AACAACA	I	C
0.1160713	cg1465966	cg1465966	52679362	AAAAATA	47783399	AAAAATA	I	C
	cg1465969	cg1465969	13644387	CATCTAA	66685405	CATCTAA	I	A
-0.165812	cg1466143	cg1466143	51625352	CTACCCAAAACAATCTACAAA			II	
0.1546705	cg1466690	cg1466690	61604365	ACATACCATATAAACAAAAAA			II	
-0.158225	cg1466984	cg1466984	55635406	ATTCTAATTTACAAACAACAA			II	
0.194344	cg1466986	cg1466986	52722450	CAATATT	17619465	CGATATT	I	C
0.1544678	cg1467009	cg1467009	72621416	CTTCATATTTTCACCAATATAA			II	
-0.23156	cg1467925	cg1467925	28650444	AAACTCAATCCCAACAACATA			II	
0.1097367	cg1468594	cg1468594	17609450	TAAAACCTAACTACTTCTAACT			II	
0.4773594	cg1468604	cg1468604	23646341	AAATATCTCTAAAACTCAATTA			II	
-0.207794	cg1468729	cg1468729	15679356	TAACTCT	67606421	TAACTCT	I	C

0.119525	cg14688662	cg14688662	27700493	AAATACAACCTACTATAAAAC			II	
0.1095656	cg14691596	cg14691596	70786483	CRAAATTAAACCRCCTAACCC			II	
0.1090549	cg14696601	cg14696601	62721321	CTTAAAATATAAAAACACCTC			II	
0.1001473	cg14698238	cg14698238	33789329	CCACTTTTAAAAATTATAAATA			II	
-0.102338	cg14699257	cg14699257	45651331	ACATAAA	28619402	ACATAAA	I	A
0.1453303	cg14701867	cg14701867	39801313	AAATAAATTCTCTCAAACAATA			II	
0.1335059	cg14704124	cg14704124	11634302	TCTCAAAATAAATACCRATAA			II	
0.1544186	cg14705010	cg14705010	51719320	TAACTTC	39673433	TAACTTC	I	A
-0.120796	cg14708411	cg14708411	48720320	CACTAAA	40616345	CACTAAA	I	C
-0.141925	cg14710850	cg14710850	27638448	TTAAATATAAAAACAAATAAC			II	
-0.158935	cg14711243	cg14711243	18781419	CTCTCTCTCTATTACAATTTCC			II	
0.2697168	cg14715697	cg14715697	46756442	TCACACC	66644400	TCACGCC	I	T
0.4092659	cg14717122	cg14717122	16806408	ACCAAATCTACTTCATCCCTA			II	
0.159142	cg14731462	cg14731462	20687329	AATTCRATCTTACTCAAATAA			II	
-0.107932	cg14736087	cg14736087	48721339	AAATCTTAATAAAATAAACCA			II	
-0.147768	cg14738806	cg14738806	29643446	AAAAATTAAAAATAACTAAAA			II	
0.1348774	cg14740976	cg14740976	28727498	AATAATAAAACRTAAAAACAT			II	
0.1479671	cg14742445	cg14742445	39780473	TACAACACCCCCAAATCCCAA			II	
-0.111583	cg14742615	cg14742615	14627388	CRTTATACCTATTTTTTATCTT			II	
0.2271338	cg14753094	cg14753094	44624466	TCAATTACCTACTATTCTAAAA			II	
0.1674483	cg14758525	cg14758525	73712349	AAAATTCACCAAAACACAATC			II	
-0.121703	cg14759366	cg14759366	41780443	CAAAAACCATATACAAATACR			II	
0.1263691	cg14759494	cg14759494	14686387	TCAAATTCTCAAACTCCRAAC			II	
-0.100787	cg14780132	cg14780132	15659311	TTTCAAACTCCTCAAAACCAA			II	
0.1146658	cg14780466	cg14780466	61629303	AAACAAT	19625435	AAACAAT	I	C
-0.144745	cg14782559	cg14782559	50621445	AAAATCCRTCCTACTAAATTA			II	
-0.143259	cg14793137	cg14793137	46638458	CATTCAT	74760501	CGTTCAT	I	C
-0.103704	cg14795253	cg14795253	25701421	AATTCTA	58790505	AATTCTA	I	T
	cg14795409	cg14795409	13644342	CCCTAACTTCACTTTTACAATT			II	
0.2996336	cg14797147	cg14797147	24788474	TTCCAAATAAAAAACACTATA			II	
0.2767694	cg14799277	cg14799277	74607488	TCTTAACTACCAAAAAAACAA			II	
0.2788808	cg14799809	cg14799809	62630444	ATTTATTCCTTAATAATAAACC			II	
0.1646431	cg14804903	cg14804903	47779475	TCCATAACTATATAAATAAAA			II	
0.1080229	cg14811011	cg14811011	62676339	AAATCTACAACTCTTCAAACA			II	
0.1077294	cg14811014	cg14811014	69627345	ATTTAATTTTATTACTAAAATT			II	
0.1362821	cg14815891	cg14815891	59655461	CAAAAAC	24684340	CAAAAAC	I	A
0.1220356	cg14818277	cg14818277	46681424	ATTCAAAAAACCRAATAAATT			II	
-0.114781	cg14818413	cg14818413	70770367	TTTTATCTCCTCTCCAAATTTT			II	
0.1102012	cg14818621	cg14818621	49708459	ATCATATACTCCCTCTATATAA			II	
	cg14819368	cg14819368	13644372	AACRATAAAAATAACAAATAT			II	
-0.135952	cg14825858	cg14825858	17683414	ATACAATTTCTCTTAACAACA			II	
0.3082348	cg14829303	cg14829303	40753304	TCCATCATTTATCTTTAACRAC			II	
-0.137505	cg14831390	cg14831390	28714477	AAAAACC	25660312	AAAAACC	I	A

0.1491282	cg14832352	cg14832352	73681385	CAAAACAATCCRTACATCAAA			II	
0.1056674	cg14832904	cg14832904	22792504	RAAAACRATAACRATAACRAT			II	
-0.133957	cg14834300	cg14834300	36615392	AAAAAACTAAAAACRCTAAAA			II	
-0.100389	cg14839919	cg14839919	46686446	TTTATAATCCTAAACAATACTA			II	
0.1023025	cg14840848	cg14840848	42796501	CCTCATA	17759429	CCTCGTA	I	A
0.1121665	cg14851460	cg14851460	45778357	CATTCRTTTAAAATAAATAAA			II	
0.1073338	cg14851544	cg14851544	53669497	ACTTTACTAAATTCTAACTTCT			II	
0.1136774	cg14855378	cg14855378	58649403	ATTAAAAACCACAACCTAACT			II	
0.1303185	cg14856563	cg14856563	65740428	ACACATTAACTAAAAATTAAA			II	
0.1019621	cg14858786	cg14858786	33709449	AAACTAC	35701323	AAACTAC	I	T
-0.108309	cg14859874	cg14859874	14694304	TAAACAAAAATAAACCATCTC			II	
0.1559051	cg14873022	cg14873022	39715368	AATTATCCCRAACTAAATATA			II	
-0.102806	cg14877637	cg14877637	70646322	AAAATAATTCTCTCCAATAAA			II	
0.2007279	cg14877834	cg14877834	28804443	TCTACTCATCAAATAACACTA			II	
-0.115611	cg14882966	cg14882966	42626317	CCCACAA	31600403	CCCGCGA	I	T
0.1182527	cg14883605	cg14883605	57691462	CCCAAAAAAACTCTTAAAAAA			II	
-0.107584	cg14891195	cg14891195	74756405	CTTAAAC	18734405	CTTAAAC	I	A
-0.100396	cg14893857	cg14893857	25788482	TAACCRAATATATACCTAATC			II	
0.1207582	cg14894216	cg14894216	47779456	ACTCCCTCATAAATAAATATA			II	
0.1085078	cg14895374	cg14895374	64717338	TTATTAATACCTAAACTAACCA			II	
0.1752689	cg14900909	cg14900909	47622405	TTCACCT	73707479	TTCGCCT	I	C
-0.101226	cg14902204	cg14902204	74772470	CCRAAAATCCCAAACTATAAA			II	
0.116994	cg14909614	cg14909614	36667507	CAAACRAAAATACCAAAATAT			II	
-0.1334	cg14915878	cg14915878	47698493	CAAAACC	71778502	CGAAACC	I	A
-0.100621	cg14926196	cg14926196	35619359	AATAAAC	74680402	AATAAAC	I	A
-0.328781	cg14926485	cg14926485	28731492	ATATAAAAACATCTACACAAA			II	
-0.141627	cg14927745	cg14927745	56710417	AAAAAAA	64784332	AAAAAAA	I	A
0.1562108	cg14931776	cg14931776	40692459	RCAAATACCAAAAACAATAAA			II	
0.1036714	cg14931884	cg14931884	57642487	AAAAAAC	63670403	AAAAAAC	I	T
0.1061105	cg14936938	cg14936938	67655453	AACCATACAAACAAAATCATA			II	
-0.131528	cg14938419	cg14938419	51773359	AAAAAAC	40665371	AAAAAAC	I	C
0.1516106	cg14939821	cg14939821	36751420	AAAAAATTAATAAACTTCCTC			II	
0.1054267	cg14943002	cg14943002	49717489	CCACRACAAAACAATTATTCA			II	
0.1078486	cg14944696	cg14944696	34806455	TAACACTACCCRCAAAAAAAC			II	
-0.139046	cg14949065	cg14949065	38663360	ACAACTC	11652426	ACAACTC	I	A
-0.101129	cg14951292	cg14951292	68654498	CTAACACCTCRATTACCAATCT			II	
0.1221348	cg14953379	cg14953379	73726329	CTACAAAACCACTTCTTATAAT			II	
0.1052287	cg14960373	cg14960373	62676503	TAATACCCTATAACTTAACCTA			II	
-0.100652	cg14970273	cg14970273	18770301	ATCAACTTCTCCCTACCTCTCT			II	
0.1020624	cg14975347	cg14975347	11777315	CTTTTCTATCCCAATAAAATAA			II	
0.1344827	cg14977018	cg14977018	62660356	ACATACTTTTATTTTAATATAA			II	
	cg14981468	cg14981468	30768379	ATTCAAAAAACAACCAACAAA			II	
-0.111854	cg14983135	cg14983135	52602503	CAACCAC	12660507	CGACCGC	I	T

-0.118064	cg1498383(	cg1498383(	60699371	ATTAAACTACRCCAAAAACTC(			II	
-0.114897	cg1498434l	cg1498434l	43800433	AATATAA	55752431	AATATAA	I	C
0.1924786	cg1498689(	cg1498689(	48640421	AATTTACTAAATCTCAACTAC1			II	
-0.102646	cg1498778'	cg1498778'	16646398	CACTAAT	66768392	CGCTAAT	I	A
0.1005831	cg1498997'	cg1498997'	17783337	CTTAAAAATCTCTTATCTAAA(			II	
0.1126953	cg1499297'	cg1499297'	21762414	TACTACAACTATTTATTATCCT			II	
0.1088206	cg1499713'	cg1499713'	63806388	ATCTACTAAAAACTATAACCT/			II	
-0.104683	cg1499759'	cg1499759'	25771491	CCCAACTACAAAAAAATCTAT.			II	
0.1083333	cg1499903(	cg1499903(	12701332	AATAAAAAAAAAATCCCRAAT			II	
0.1052819	cg1500081'	cg1500081'	67606447	ATTTCACACCAAAAATTCAAA.			II	
0.1139743	cg1500478'	cg1500478'	28786454	TAACCATTCCACTTACTTTAAT			II	
-0.170445	cg1500795(	cg1500795(	49757401	TTCTAAT.	55726326	TTCTAAT.	I	C
0.1043226	cg1500923'	cg1500923'	64739341	AATCAACCRATAAAAACTACT.			II	
0.1605999	cg1501436l	cg1501436l	14649308	CAATACACCCTACRAATCAAA.			II	
-0.128753	cg1502056(	cg1502056(	70810338	TTCCTTC(	64604420	TTCCTTC(	I	A
-0.21604	cg1502260(	cg1502260(	55620414	AAAACA/	19793382	AAAACG/	I	A
-0.14502	cg1502520(	cg1502520(	48763405	ACCATAT	22691478	ACCATAT	I	A
0.1097822	cg1502772l	cg1502772l	22689478	TTACCTAATTACTACATTCTTT'			II	
-0.176843	cg1503301'	cg1503301'	39655305	CTCRTACATAAATTAATTCCR(			II	
0.1365741	cg1503355'	cg1503355'	74678456	AAAAAAAACCTCAAAACTAA/			II	
-0.109929	cg1504414(	cg1504414(	38798388	CACTATAAAACRAACAACATT.			II	
0.1083908	cg1504457'	cg1504457'	72727466	ACATAACAACATTCTTAAATT/			II	
-0.113051	cg1504476'	cg1504476'	64648420	AAAAACAAATCTCCAATCTAA'			II	
-0.102049	cg1504493'	cg1504493'	49637505	ACTATCAATAACATTTATAAT1			II	
0.1124847	cg1505487'	cg1505487'	33754404	TAACCAAAATACTTACRTAAC(			II	
0.1182336	cg1505578'	cg1505578'	61647408	AATTACTACCCAAACTCTATC/			II	
-0.105718	cg1505955'	cg1505955'	12650335	ACTAAAACACRACCAAATAAC			II	
0.1618482	cg1506008l	cg1506008l	66673325	AACACTA	30638301	AACACTA	I	A
-0.103167	cg1506059(	cg1506059(	73696342	AAAAAA/	72780487	AAAAAA/	I	C
-0.101026	cg1506855'	cg1506855'	56712415	AAAAAA(	46733418	AAAAAA(	I	A
-0.269746	cg1507470(	cg1507470(	39607394	TCACAAA	23616423	TCACGAA	I	C
0.1158327	cg1507993'	cg1507993'	32752434	TACATAT	14738451	TACATAT	I	C
0.1753198	cg1508043'	cg1508043'	40634450	RTATCTTTCTTTAACCTTAACA.			II	
0.2342657	cg1508480'	cg1508480'	42709321	ATTATATACCTATCCTAAAAA/			II	
-0.105188	cg1508790'	cg1508790'	41711429	CTCCAAC	19799359	CTCCGAC	I	A
-0.101645	cg1509221(	cg1509221(	41636348	CTAAAACRAAAATCTCCAAAA			II	
-0.115169	cg1509234'	cg1509234'	24699391	ATAAACA	17737346	ATAAACC	I	A
0.1013749	cg1509864'	cg1509864'	37710373	AAAAAACCCCRAAAAACAATA			II	
0.1138806	cg1510462'	cg1510462'	12797491	AATTAAA	31705328	AATTAAA	I	A
-0.171617	cg1510501l	cg1510501l	18792450	TAACTACATATACAAAACCTT/			II	
-0.114975	cg1511609'	cg1511609'	69657441	TACAAAATACAATTAAACTAC'			II	
-0.109867	cg1511648l	cg1511648l	63633329	TAAAAAC	19609360	TAAAAAC	I	T
-0.168077	cg1511775'	cg1511775'	27693398	ACCCTCCTAAAAACCAAATCC'			II	

-0.161184	cg15122993	cg15122993	56723420	ACAACAC	53652348	ACAACGC	I		A
0.1300034	cg15132295	cg15132295	40628329	ACATTAAAAATCRCCACACAC			II		
0.1223535	cg15135166	cg15135166	56688395	TATAACTTAAAACAAAATCCC			II		
0.1026542	cg15135286	cg15135286	26780484	AATCTCCTAACACTCTCRACTA			II		
0.1272505	cg15135762	cg15135762	14697311	CCCCACC	44624470	CCCCACC	I		T
0.2520041	cg15138289	cg15138289	48800482	ATATTTACAAAATATCCTAAAC			II		
-0.211049	cg15142694	cg15142694	16736447	AAAACAA	23617466	AAAACGA	I		C
0.1058511	cg15145174	cg15145174	30645385	AACATAA	73619499	AACGTAA	I		A
0.134068	cg15149645	cg15149645	68604460	ACRTCCTACATCTCAACRAAA			II		
-0.199939	cg15166039	cg15166039	14662427	TCAATCA	18800399	TCAATCA	I		C
0.1356314	cg15171791	cg15171791	54705472	TAAACCRTAATATAAAACCAA			II		
-0.124495	cg15174294	cg15174294	65800308	AATAACTATAATAAAACTTAA			II		
-0.157283	cg15177613	cg15177613	37632356	AAACCCC	48713472	AAACCCC	I		A
0.1346321	cg15187665	cg15187665	36727374	ATTACTT	25731376	ATTACTT	I		C
-0.126576	cg15190383	cg15190383	54644436	CTATTTTAACRACTATCTTTTA			II		
-0.123816	cg15193133	cg15193133	25755329	AACAAAA	31659427	AACGAAA	I		A
0.103781	cg15193215	cg15193215	24717412	ACAATTTAAAACTCACCRAATC			II		
-0.23577	cg15198148	cg15198148	12726408	AAAAAAAATTAAACCCCCCTC			II		
0.1121825	cg15199987	cg15199987	18726423	CTCACTACCCCAAACCAACAA			II		
0.126485	cg15200009	cg15200009	30605453	CTCTTACTACAAACATATCCTT			II		
-0.115004	cg15205428	cg15205428	67685347	CAAAAAA	41726414	CGAAAAA	I		C
0.1144578	cg15207055	cg15207055	73778471	AAAATTCTCACATTAAAAAAC			II		
-0.128133	cg15215348	cg15215348	72600300	AAATAAC	65779478	AAATAAC	I		A
	cg15219228	cg15219228	11753320	AAAAAAA	16794305	GAAAAAA	I		A
-0.281737	cg15226348	cg15226348	58751381	AATACTTCTCCTACTTTCCCAC			II		
-0.152001	cg15236541	cg15236541	56680387	CAAAAAT	69765318	CGAAAAT	I		A
-0.11193	cg15243856	cg15243856	56716505	ACCAAAA	30775414	ACCGAAA	I		C
-0.105727	cg15246131	cg15246131	37628397	ATTCACAACRAAATATAAATA			II		
-0.555453	cg15247483	cg15247483	68781455	TACTACTATTAAACTAAAACCC			II		
0.1124178	cg15249341	cg15249341	35748431	TATAAACTAAACAAAAATAAC			II		
0.1369164	cg15250633	cg15250633	26733393	ACTAACC	16683386	ACTAACC	I		A
-0.183022	cg15251140	cg15251140	39710464	TATTTCT	50745432	TATTTCT	I		A
-0.129453	cg15251385	cg15251385	43642427	CCCTACT	65714457	CCCTACT	I		A
0.1190722	cg15254640	cg15254640	56711445	TACCCCA	70679503	TACCCCA	I		A
0.1354103	cg15256387	cg15256387	65765430	ACACATTTATCTCAAAATTTTA			II		
-0.117102	cg15278109	cg15278109	50758462	ACTACTT	48714335	ACTACTT	I		C
-0.169125	cg15279541	cg15279541	46670416	CACTAATATTCACCTTAACCTA			II		
0.1724887	cg15282973	cg15282973	10728416	ACCTCCCAACATCCTTCTATAA			II		
0.12835	cg15286032	cg15286032	48760415	CACTATTACTTTAAAAACTATC			II		
0.1165501	cg15287806	cg15287806	47793319	TTATTATCTCAACATCATTCAA			II		
-0.271664	cg15290312	cg15290312	34647475	TCACACA	24790449	TCACACG	I		A
0.1121292	cg15292097	cg15292097	57607317	CCCAATCTAACAAATCTCTTCT			II		
-0.181237	cg15292356	cg15292356	21607304	RAATATAAATCCRACCAAATA			II		

-0.10003	cg15293181	cg15293181	46700503	CAAAAATACCTTTATATCTAA			II	
0.1995012	cg15295441	cg15295441	37744473	ACCCTTACATTCTTCTTATCCA			II	
-0.127778	cg15298323	cg15298323	27651418	CTACCTTCTAAAATCAACTCTA			II	
0.1508348	cg15309004	cg15309004	44725368	ACCAATAAAATAAACTCCTCT			II	
0.145217	cg15320905	cg15320905	38807401	AAACCTA	45758409	AAACCTA	I	A
-0.100238	cg15326535	cg15326535	39665482	TAATCTAACCCTAATCTCTCAC			II	
	cg15328131	cg15328131	12768428	AAATCATACRTCCTATAATTA			II	
0.2066817	cg15329552	cg15329552	20721361	TACAAACCRACCAACACCTAA			II	
-0.113917	cg15334096	cg15334096	60662308	CAACTACTACTAAACCRCTAT			II	
0.1052945	cg15340526	cg15340526	13611435	CRAACCTCAAAAACAAAACTC			II	
0.1725842	cg15345074	cg15345074	48773449	AAAAACA	29760424	AAAAACC	I	A
-0.112503	cg15347156	cg15347156	27772368	ACCAAAT	48705311	ACCAAAT	I	A
0.1337576	cg15359321	cg15359321	23642399	ACCAACCRAATCTATTAAAAC			II	
0.1044022	cg15360181	cg15360181	48806320	CTTTCATACTAAACAAATCTA			II	
0.1184091	cg15360451	cg15360451	33713458	TAAACAC	69692479	TAAACAC	I	A
-0.166419	cg15361697	cg15361697	68641388	AAAAAAC	54794394	GAAAAAC	I	A
-0.167119	cg15364618	cg15364618	65807359	TTAACTACRTAATAAAAAACC			II	
0.2562185	cg15365500	cg15365500	35642301	TAATCAATAAACCTCCTTCATT			II	
0.1055004	cg15381326	cg15381326	44630311	TTAACAAAATCAAACAACCAC			II	
-0.100796	cg15383633	cg15383633	21613480	RCTTCTACTATAAACTAACCR			II	
-0.108992	cg15389068	cg15389068	30747383	AAACATT	74746378	AAACGTT	I	A
0.1174581	cg15389191	cg15389191	17749315	TCTTTTCTTCATCAAATAATTA			II	
0.1946931	cg15392147	cg15392147	28645389	CAACATAAAAAAACTCCRTCT			II	
0.1036905	cg15393490	cg15393490	20622482	CCTCACTACCTATCCCCATAA			II	
0.1028286	cg15394255	cg15394255	58728307	TTCTATAAAAAAACTAAATAA			II	
0.1466612	cg15395971	cg15395971	51694313	TATTTCAACCCTTCCACTATAC			II	
0.1042201	cg15401477	cg15401477	10713410	ACTCAAATCAACCTCCCCAAT			II	
0.1583895	cg15403517	cg15403517	31703427	CCRTAAAATTTTCTTCCCRTAT			II	
-0.187933	cg15404665	cg15404665	41703431	AAAAAAA	29770308	AAAAAAA	I	C
-0.165022	cg15405432	cg15405432	18616329	TTAATTTAACCTTAATTTATAT			II	
-0.339174	cg15408512	cg15408512	34757439	AAACACA	54742333	AAACACA	I	T
-0.204736	cg15410411	cg15410411	39631417	CCCAAAA	32801472	CCCAAAA	I	A
-0.15367	cg15414745	cg15414745	72610507	ATAAACT	14768412	ATAAACT	I	A
0.1770211	cg15418499	cg15418499	31742425	CCAACACCATCAAACAAAAAA			II	
0.1775162	cg15425276	cg15425276	35773437	TCCTATAACTACAAAATATAC			II	
0.1357188	cg15427793	cg15427793	60680371	ATTTAAATAAAATAAACTCAT			II	
-0.102109	cg15431659	cg15431659	48761366	CATATTC	24686486	CATATTC	I	T
0.1206066	cg15435765	cg15435765	20696420	AAAACCC	31691373	GAAACCC	I	A
-0.460625	cg15436955	cg15436955	56792351	CCACCAAAACTCTCRCTACTA			II	
0.1141366	cg15443166	cg15443166	31604507	TATAAACCAAAATAATAAATT			II	
0.1720874	cg15444149	cg15444149	10800311	TCTATAT	46618328	TCTATAT	I	A
0.1120739	cg15450098	cg15450098	23785345	ATCCTTATCCTTTTAAACCTAA			II	
-0.105702	cg15465642	cg15465642	26715498	AAAAATA	51783462	AAAAATA	I	A

0.1508422	cg15466952	cg15466952	21606362	ACAACCTTAATACTCTATTTCT			II	
-0.104573	cg15467834	cg15467834	24807351	ACATCCT	55780486	GCATCCT	I	C
0.1560486	cg15477040	cg15477040	44725479	TTTTTCTAACTTACATACAAAA			II	
0.1028623	cg15491439	cg15491439	57685373	CCTAAAACTCAAAAAATAATC			II	
0.1396282	cg15498409	cg15498409	15694411	TACAATCTCCTTATCTCCCTCT			II	
-0.212547	cg15501526	cg15501526	58784502	ATAAAAC	16797443	ATAAAAC	I	C
-0.102773	cg15505615	cg15505615	35654344	AACTAAC	42775501	AACTAAC	I	C
-0.118833	cg15509177	cg15509177	29713300	RTAACACAAATTTAACCCCRA			II	
0.1200512	cg15512715	cg15512715	67790443	AAAAACATACATTTTCRTAAC			II	
-0.510535	cg15518264	cg15518264	73628441	ATCTAATTTCTCCTTACTAACC			II	
0.1106712	cg15531512	cg15531512	15600489	TAAAACATTTTCCAAATTTCTA			II	
0.1132762	cg15532640	cg15532640	57808427	ACTAAAA	30685341	ACTAAAA	I	A
0.1069363	cg15537850	cg15537850	38629356	ACCTTCATAACCACAAAAAAT			II	
0.1106339	cg15541170	cg15541170	26800467	TCACACAAAAACCTCTTTTCCT			II	
0.1002963	cg15543027	cg15543027	33733322	AATAACAATAAATCCTAAATA			II	
0.1450462	cg15544633	cg15544633	21638446	CACAAAA	11751488	CACGAAA	I	C
-0.115571	cg15548198	cg15548198	68666500	TACCTAAATTTTATTTACATAA			II	
0.1218254	cg15554966	cg15554966	12639475	TTCAAACAAAAAATACTCCCT			II	
0.1270555	cg15561493	cg15561493	45804321	TCTTTATACAAATTCCTCAAAA			II	
0.2835194	cg15567368	cg15567368	45616309	AACTAACATATATTCTACCCTT			II	
-0.242927	cg15568074	cg15568074	21673461	ACRCTAAATACTAAAACAACC			II	
0.1046416	cg15568617	cg15568617	50717475	TTATTATCCRATTCCTATTAAT			II	
-0.117495	cg15570148	cg15570148	51775306	TAAAATTCTAACCTAAACCRA			II	
-0.10613	cg15574437	cg15574437	46744474	CCCACTA	42759335	CCCGCTA	I	C
-0.110273	cg15575880	cg15575880	53732368	AAAAACT	52664438	AAAAACT	I	A
0.1915755	cg15581944	cg15581944	56612410	AAACCAAACCTAAAACAAAAC			II	
-0.155477	cg15584790	cg15584790	15687443	AAAAAAA	40654329	AAAAAAA	I	A
0.1774803	cg15591803	cg15591803	14631428	CCAAAAATTATAAAACTTCTTA			II	
-0.142363	cg15595755	cg15595755	16787451	AACAAAA	67647405	GACGAAA	I	T
0.1097499	cg15599668	cg15599668	26719502	CTAAAAAAAATTTAAATATCA			II	
0.1396115	cg15600437	cg15600437	36605384	AAACTAATTTCTTATTTAATTA			II	
0.1582679	cg15602677	cg15602677	47615484	AAAACATTTCCCTCATCCCAA			II	
0.1083469	cg15609373	cg15609373	18690463	TAATCATAATTCCAAATTTAA			II	
-0.14966	cg15611600	cg15611600	23664305	AAAAACTAAACCTCCATAAAA			II	
0.1128644	cg15619377	cg15619377	46684424	TCTTCCTATATACTAAATTCAA			II	
	cg15623480	cg15623480	13644384	AAAAAAC	35762463	AAAAAAC	I	A
0.1412095	cg15624335	cg15624335	44808445	ATCCCTACAAAAAACATAATT			II	
-0.101239	cg15626112	cg15626112	42735374	ACATAAATCRTAATCTCTTCA			II	
0.1437072	cg15633912	cg15633912	46646406	AAAACTAACRCRAAAACTTTC			II	
0.2058812	cg15634083	cg15634083	38800332	TAAAAAC	62793370	TAAAAAC	I	T
-0.260307	cg15642854	cg15642854	39634377	ACCACAA	61726325	ACCGCGA	I	T
-0.104794	cg15648389	cg15648389	31797330	TACRACRATTTTAAAACCAAA			II	
-0.320331	cg15652532	cg15652532	15619492	AACCTAAATTCTATAACTACT			II	

0.1020777	cg15655097	cg15655097	24757507	TATCTATATCTCTACAAAACAT			II	
-0.167019	cg15663948	cg15663948	21660393	TTCCACAATATCCTAAAAACCA			II	
-0.116202	cg15671450	cg15671450	35775311	CTCCAAATAAACCCTAATCTAC			II	
-0.334017	cg15677681	cg15677681	50760476	CTACRATCTCTTCTTTCTATTTA			II	
0.3940135	cg15680603	cg15680603	51706372	TAAATAC	48692417	TAAATAC	I	C
-0.137056	cg15682806	cg15682806	60729467	ACCCAAC	58645414	ACCCAAC	I	C
-0.11635	cg15682807	cg15682807	29656395	CACAACC	71664408	CGCAACC	I	C
0.1147933	cg15683497	cg15683497	66639442	AACCCAA	56773445	AACCCAA	I	A
0.1208519	cg15683950	cg15683950	36756465	ATTTTCAAAAATAATCCCAAAT			II	
0.1050481	cg15684661	cg15684661	61802507	ATCCATTCCTCAATATCCAACC			II	
-0.154136	cg15690347	cg15690347	26736445	CACTATC	73634351	CGCTATC	I	A
0.1425704	cg15692593	cg15692593	11651429	ATAAAAAAAAACATTAACAAA			II	
0.2951259	cg15693937	cg15693937	46625496	AACTTAAATCTACCAACACTCA			II	
-0.108087	cg15699085	cg15699085	35717465	ATCTACTAAACTCACTTCCTCT			II	
-0.12952	cg15699552	cg15699552	21738485	TACAAAT	70790419	TACGAAT	I	T
-0.115505	cg15704280	cg15704280	65743393	CTAACTAATAAAAATATTATA			II	
0.1627521	cg15704872	cg15704872	66726310	AAAATTTTAAAATCCAAAATA			II	
-0.110777	cg15706156	cg15706156	23704322	ACTACTA	37737444	ACTACTA	I	C
-0.235194	cg15706568	cg15706568	47785393	AATACAACCAACTTCTCTTCA			II	
0.1220206	cg15714682	cg15714682	57675362	CTTCTATAAAATAAAAACACTA			II	
-0.11482	cg15716623	cg15716623	39768442	ACRAAATACRATAATATAAAC			II	
0.2000272	cg15717617	cg15717617	60736362	AACRCTAAAATAAATCATACC			II	
0.1068166	cg15724876	cg15724876	62645487	TACTCTAACACATTAATCTATC			II	
0.1023991	cg15726123	cg15726123	31721364	CTAAAAACAAACCCCAACCTA			II	
0.1177712	cg15728692	cg15728692	12782313	ACCTAATAAAAACRAAAAATA			II	
0.1054847	cg15728909	cg15728909	34605452	ATAAACACTAAACCTTTTATAC			II	
-0.125536	cg15730931	cg15730931	17635330	TATCCRTCTTAAAATAACTACR			II	
-0.122809	cg15732107	cg15732107	60715449	TATAATCTCTCCACRATAATTT			II	
-0.15665	cg15737177	cg15737177	31610426	AACAACA	20809435	AACAACC	I	A
0.1629413	cg15740507	cg15740507	60703439	CATCAACACCAAAACTTTATCT			II	
-0.137936	cg15752756	cg15752756	74780342	CCAATACTAATTAATTCTTTTC			II	
0.1283833	cg15758008	cg15758008	42601307	ATAAAAAAACRAATTTCATATA			II	
-0.137563	cg15765638	cg15765638	43723466	CCTTTATAAAACAACCTAACTA			II	
-0.174416	cg15768613	cg15768613	16680480	CTAAAAAAAACTACATTTTTA			II	
0.1945089	cg15773974	cg15773974	44645334	TACTACA	16602440	TACTACA	I	A
0.1430093	cg15779891	cg15779891	50778398	ATTAATCTTCACAATAATCCTA			II	
-0.135655	cg15781156	cg15781156	66623406	AATCTAAACCCAAACCTAAAC			II	
0.2624826	cg15783848	cg15783848	30621385	TTTTATTCTATAAAACATACTA			II	
-0.115428	cg15786827	cg15786827	26714352	TATAAAT	50668485	TATAAAT	I	A
-0.106748	cg15788369	cg15788369	40613314	AATCCCA	25712368	AATCCCG	I	A
0.1476371	cg15790184	cg15790184	32663490	CCAACCACAACCTACRTAAAC			II	
0.2363854	cg15793258	cg15793258	63613330	AACTCCCTAAACCCTAACTACA			II	
-0.115649	cg15798862	cg15798862	42764356	CCCTTATCCCTATAATATTCAT			II	

-0.118306	cg1580134(	cg1580134(	14789485	CTACTTTTCCCAACTTCAACTA			II		
-0.122054	cg1581143:	cg1581143:	10741416	TTCTCTTAAACTTTTATAACCT(			II		
-0.128764	cg1581166{	cg1581166{	34771443	CAAACTT	39796501	CAAACTT	I		A
-0.107312	cg1581202(	cg1581202(	19700315	AAAACAC	69704377	AAAACGC	I		C
-0.120597	cg1581933:	cg1581933:	15621481	CTCCCCTTAATTAAATATACCT			II		
0.1079686	cg1582443:	cg1582443:	59628421	TTTAAAAATAATTCACRTTACT			II		
-0.118804	cg1582470:	cg1582470:	19774392	CCAACCA	25755347	CCAACCA	I		C
0.1585676	cg1582681(	cg1582681(	22705486	ACTAAAATTTCAACTAACATA			II		
-0.238825	cg1582908{	cg1582908{	60661444	CTCACAA	58706346	CTCGCAA	I		C
-0.103183	cg1582996(	cg1582996(	24632422	CCAACCATCTAAATTCRAATC]			II		
-0.115621	cg1583794:	cg1583794:	38745406	CCAACCAACTATAACATTAAC			II		
-0.117636	cg1584055	cg1584055	17633478	CAAATCCCTCATAAATAACTT			II		
0.1086645	cg1584441(	cg1584441(	24610482	ACCTTTATTTCTAAACAAAAA			II		
0.1012683	cg1584536:	cg1584536:	68737498	AATCTTCCCAATACAAAACTT			II		
0.1342882	cg1584727:	cg1584727:	43689412	CCTAAAAATTTCCCAACTTATA			II		
0.1022914	cg1585073(	cg1585073(	33748413	ACAATTACATAAACRACTTTC(			II		
0.1034916	cg1585244(	cg1585244(	33661343	CRAAACAAACTCCTAAAAAAA			II		
0.3046114	cg1585377]	cg1585377]	13754467	ATTCTAAATAAATCCAACAAT			II		
0.1034012	cg1585747(	cg1585747(	56623333	CTACAAAAATCTTAAAACAAA			II		
0.1696611	cg1586608(	cg1586608(	51799376	TAAATCAAAACATCCACAAAT			II		
-0.172301	cg1587462(	cg1587462(	58630411	AAATCAA	27706371	AAATCGA	I		A
0.1035675	cg1587470	cg1587470	60799356	AACTCTCAACACCATTTATATT			II		
0.2375034	cg1587752(	cg1587752(	37656439	CCAACAC	23680318	CCAACAC	I		A
0.1064741	cg1588179:	cg1588179:	17795419	ATACCAAATATTTATTTATATA			II		
0.1328417	cg1588309	cg1588309	27730328	AAAACAA	22789353	AAAACAA	I		A
	cg1589164:	cg1589164:	12768347	AATAAAATTTCTTTTAAAATAT			II		
0.1083472	cg1589512]	cg1589512]	26673478	AAAAACAAATACCAACAAACC			II		
0.1885768	cg1589533(	cg1589533(	39716426	TTTATAAATATTTCAAAAAAA(			II		
0.1578829	cg1589578:	cg1589578:	25801508	CTATAAATTCCTAACACATAA]			II		
-0.6258	cg1589743:	cg1589743:	60706393	TCCTCCTAAAAAATATATCAC			II		
0.2229933	cg1589980(	cg1589980(	37754400	AATAAATAAAATAACCTCAAA			II		
-0.104935	cg1590897:	cg1590897:	22676328	TCAAATAAATATTTCATCCAA			II		
0.2253095	cg1590944:	cg1590944:	64786316	AACCACATATCTTCCTACTAA			II		
0.1014017	cg1591116:	cg1591116:	66707467	AAATCTCTAAATATATTCATTT			II		
-0.113114	cg1591132{	cg1591132{	50775463	ATAAATTTATACAAAATCACC			II		
0.1267329	cg1591981(	cg1591981(	57785429	TAATTTATATATTACRTAATTT			II		
0.1004041	cg1592188:	cg1592188:	48629388	CTTTCTCAAAACTCCAACCAA]			II		
0.152045	cg1592456{	cg1592456{	34681444	AATAAATAACATCTCTTTCCCT			II		
0.127975	cg1593805(	cg1593805(	54774355	TTCCTCAAAACACTTCAATTA			II		
0.1001279	cg1594333:	cg1594333:	64668323	AAATTCCTCATTTTTTCCATAT			II		
-0.161189	cg1594659(	cg1594659(	11734316	TCCAAAAAAATATAAATTCCR			II		
	cg1594841(	cg1594841(	13644396	ACACAAAACTATAAACTAAAC			II		
-0.106126	cg1594856:	cg1594856:	31711352	CAAACTA	26660377	CGAACTA	I		C

0.1253988	cg15949553	cg15949553	61651509	ATATTCTCCTAACCAAAAATAA			II	
-0.116703	cg15951460	cg15951460	62699310	AAAAACRCAACTCRACCTTAA			II	
0.1223676	cg15957807	cg15957807	26701411	AATAATTCAATACATTTTTAAT			II	
-0.211847	cg15958422	cg15958422	66711467	CCTCTCTTATCAATACAAATAA			II	
0.2868934	cg15959464	cg15959464	59777444	TATTTATTTCAACCTCCRTATT			II	
0.1301691	cg15963988	cg15963988	15749386	TCAACCATCCACTCCTTAAACA			II	
0.2153563	cg15964080	cg15964080	66682491	CTTTATAACCTCAACCRTAAAA			II	
0.2422457	cg15964593	cg15964593	14786324	TATCACTACTTCAAATCTAAAC			II	
0.1104653	cg15965383	cg15965383	64680473	CTTTCTCTAATTAATAATACTA			II	
0.1062114	cg15978494	cg15978494	23618398	ACCACATATTCACATACACATA			II	
-0.293611	cg15979214	cg15979214	53601493	ACCTACRTAAACCAACAATATA			II	
0.1032614	cg15983986	cg15983986	33741381	AAAAATATAATCTACTACAAC			II	
0.1168683	cg15986469	cg15986469	10602301	CAACAACCATATTATCRTAAAT			II	
0.107919	cg15988287	cg15988287	22760510	AACACCTATAAATATTCCTAAC			II	
0.1519324	cg15988569	cg15988569	68758493	AAACAAAATAAATAAAACACT			II	
-0.235085	cg15991478	cg15991478	59631436	ATATTTC	14688462	ATATTTC	I	T
0.1680778	cg15998127	cg15998127	55707472	CAAAACT	36666312	CAAAACT	I	A
0.1096498	cg15999609	cg15999609	19649402	CTAATTCCCTTACTAAAACAA			II	
0.141508	cg16002799	cg16002799	33698330	AAATAAACRAAAAATAAATTT			II	
0.114593	cg16006965	cg16006965	32687493	TCTATAACTTAACTATTTTAAC			II	
-0.102144	cg16009558	cg16009558	74615493	CAAAAATTTTACCAAACCCTA			II	
0.1001637	cg16016034	cg16016034	34673336	ATTAAACCACAATACCAACAT			II	
0.1043682	cg16031973	cg16031973	63740383	ATAAATAACTTATTTTAACTTC			II	
0.3027641	cg16034991	cg16034991	67689422	TACATATATCCCACAAAACTA			II	
0.103938	cg16036046	cg16036046	20755439	CCTATATTAAACCTACAACTAT			II	
0.1146358	cg16039142	cg16039142	51755452	AAAACATAAAAAACATAACTA			II	
0.123456	cg16041798	cg16041798	54771465	ACTAAATATACTATATTTCCTA			II	
0.1035271	cg16042099	cg16042099	73674391	CTTAACATAACTAATAACTAA			II	
0.1158319	cg16048915	cg16048915	74727377	ATAAATTTAAACCTAAAACATA			II	
0.1082883	cg16050326	cg16050326	73642398	AAACCTAACACTCCCRAAACC			II	
-0.117392	cg16053005	cg16053005	10756465	ATACAAT	53707335	ATACAAT	I	A
0.1483279	cg16058274	cg16058274	73783407	AACTTACAATAAACATTTAAC			II	
0.1733482	cg16059161	cg16059161	53712386	ACTAAATTATAATCCCCAATA			II	
-0.129132	cg16060189	cg16060189	15760496	CTCTATTTTAAATAACCAACTA			II	
0.3946221	cg16060930	cg16060930	38683455	CTATAATTACAACRATAAACTA			II	
-0.175066	cg16074271	cg16074271	45695325	CRCACCTTCTACCTAACAAAAC			II	
0.2741827	cg16074990	cg16074990	40742491	CRCCCTACAAAATAAAATCAC			II	
0.1032562	cg16077493	cg16077493	13809348	AACCTATTATAAATTTTACAAA			II	
0.1292949	cg16077929	cg16077929	39679313	CAAAATCATCTTCTAATTCCAA			II	
0.1111376	cg16079414	cg16079414	65692337	AAACAAAAATAACAAAATATT			II	
-0.163378	cg16081854	cg16081854	14675462	AAAAATATCCRTAACRCCAAA			II	
0.1007558	cg16082695	cg16082695	35618508	ACCAACT	61792419	ACCGACT	I	A
0.2368017	cg16083558	cg16083558	58644371	AACTCRAACTCCTAACAAAAA			II	

0.1661486	cg1608419(	cg1608419(	44650375	ACAAAACTCTAAACAACAATA			II	
-0.133429	cg1608794(	cg1608794(	45777327	CCTAACT	18702508	CCTAACT	I	C
-0.122346	cg1609195	cg1609195	65657362	AAATACT	49721438	AAATACT	I	T
0.1175961	cg1610273	cg1610273	43808361	TTTTATCACCCAAAAAACATT			II	
0.16038	cg1610556	cg1610556	38611458	AAAAAATCAATATCCACACRA			II	
0.1453311	cg1610938	cg1610938	32601352	AACTAAAATAACTAAATCAAC			II	
0.105403	cg1611222	cg1611222	27622453	CCAAATA	32647322	CCAAATA	I	C
0.5510837	cg1611288(	cg1611288(	26722406	ACCCTTCCCCAAAAATTTAAA			II	
0.1297789	cg1611837	cg1611837	21779474	TAAATCACAAAACTTATACCT			II	
-0.332601	cg1612768	cg1612768	10784337	CATAAAA	70648368	CATAAAA	I	A
0.1246198	cg1613606	cg1613606	69748499	CAAAAATATTTAATTCACCC(			II	
-0.39168	cg1614290	cg1614290	26759327	AATAATA	40629405	AATAATA	I	T
-0.134529	cg1614365	cg1614365	55661306	ACACATA	47767338	ACACGTA	I	C
0.2830378	cg1614518	cg1614518	55618305	CACTACAACTATCTCACAATT			II	
-0.112353	cg1614992	cg1614992	41734416	CAAAAAA	29799492	CAAAAAA	I	A
-0.388345	cg1615611	cg1615611	70607365	TTCAAAC	73797430	TTCGAAC	I	T
0.4435889	cg1616293(	cg1616293(	33786330	CTTTATC	68681333	CTTTATC	I	C
-0.194469	cg1616756	cg1616756	23663418	CRCCATAAATAAAAACTCTCT			II	
0.1367513	cg1617209	cg1617209	42695463	TAAATAC	57651379	TAAATAC	I	A
0.1036057	cg1617660(	cg1617660(	15652470	CTTTAAAATCAACACCAACTC			II	
-0.110298	cg1617773	cg1617773	18663429	CCTAAACTCAATAAAAAATTA			II	
0.1284724	cg1617783(	cg1617783(	26746466	AATAAACTTCRCCTACTTCRTA			II	
-0.125943	cg1618079	cg1618079	30757342	ACACAAT	47612460	ACGCAAT	I	A
0.1113022	cg1618752	cg1618752	34809454	AAAAATATATTTCTCCCCAAC(			II	
0.1044898	cg1618964	cg1618964	63781485	CCRAATCTATCAACAAAACAT			II	
-0.136312	cg1618995	cg1618995	45679329	AAAAACC	45683363	AAAAACC	I	A
-0.125409	cg1619129	cg1619129	59785357	AAAAACRCTAAAATATAACTA			II	
-0.122832	cg1619257	cg1619257	56802419	AAAAACCCRCACAAAATAATA			II	
0.1361555	cg1619974	cg1619974	27783418	AAAACTAAAAAACCCATAACR			II	
0.1360726	cg1620225	cg1620225	61760497	TACAAAA	15748418	TACAAAA	I	A
-0.10188	cg1620481	cg1620481	48789442	ACTACTC	68603443	ACTACTC	I	T
-0.228562	cg1620805	cg1620805	34695480	TAATTCT	32786455	TAATTCT	I	A
0.1106783	cg1621035	cg1621035	44803341	AATCRACTCATTATTAACTCA			II	
0.1665783	cg1621097	cg1621097	27661497	TAATTACTTTTTATAACCTAAT			II	
0.1021302	cg1621415	cg1621415	52613413	TAACCAA	17673389	TAACCAA	I	A
0.2193121	cg1623379	cg1623379	19723383	TTTTAACTTTCCCACAAAACA(			II	
-0.10473	cg1623433	cg1623433	36711428	CTTCRCTATAAACCACRACCA			II	
-0.178574	cg1623570	cg1623570	60718451	ACAATTTAATTTCCTTAAAAA(			II	
0.2259991	cg1625566	cg1625566	63743407	ACTTCAA	63636440	ACTTCGA	I	C
0.1488408	cg1625753	cg1625753	12611483	TAACATACACACACATCCTTA			II	
0.1008243	cg1626185	cg1626185	71706473	CTACATACCTAAACAACACAT			II	
-0.152716	cg1626318(	cg1626318(	62685459	ATAAAATACCRCTTTTCRCTCT			II	
0.1136632	cg1627305	cg1627305	23623343	AAAATCTCTACCTCTCTCAAA			II	

-0.112134	cg16284437	cg16284437	53698374	TAACCTT	51644413	TAACCTT	I	A
0.1046756	cg16300553	cg16300553	27665417	CTTCAAAAAATTCATATACACA			II	
0.1018516	cg16306078	cg16306078	24761450	AACTTCATAAAACCAAATTTTT			II	
0.1140309	cg16310003	cg16310003	57621323	CAATCTCCAAATACCCCCTCAA			II	
-0.225758	cg16310958	cg16310958	59700357	AATACTC	46809418	AATACTC	I	A
-0.15993	cg16316472	cg16316472	64721346	ATTTACA	10688333	ATTTACA	I	A
-0.119394	cg16316624	cg16316624	44670340	TCACATA	45663379	TCGCGTA	I	C
-0.181977	cg16320888	cg16320888	26742496	AAAAAAA	49705394	AAAAAAA	I	A
-0.113155	cg16330146	cg16330146	30726435	ATACCAA	17606402	GTACCGA	I	T
0.1236013	cg16336556	cg16336556	52782469	CAATTCTAACAAAAATTTTCAA			II	
0.1056967	cg16337566	cg16337566	11661353	AAAAAAATTAAATTCCTACCA			II	
0.1731476	cg16342620	cg16342620	46759427	CATAACCCAATCAACTAAAAT			II	
-0.238359	cg16345566	cg16345566	52757468	AATCCCTCTAAATACAAAAAT			II	
0.1038376	cg16354345	cg16354345	37758333	CCCATATTTTATATAAATAATA			II	
0.1565853	cg16355591	cg16355591	27706301	ACAACCRAAATACACAACCTA			II	
-0.106423	cg16359799	cg16359799	63684417	TAAAAACCTATAAAAATATTT			II	
0.1066954	cg16371229	cg16371229	49810458	CAATTACACAATACAACACTA			II	
0.1026957	cg16372632	cg16372632	15747371	TTCCAAA	25733311	TTCCAAA	I	C
0.1390767	cg16375820	cg16375820	56793477	AACTTAAAAACAACCCATAAA			II	
0.1761037	cg16387046	cg16387046	49634405	ACCAATCAAAAACTATCAACC			II	
-0.17203	cg16394018	cg16394018	35675418	AATCCAC	38755351	AATCCGC	I	T
-0.119085	cg16397032	cg16397032	31606396	ATATTAA	68796418	ATATTAA	I	A
0.1086442	cg16397176	cg16397176	25706345	CTTAACCTACTTTATTTATCTT			II	
-0.178143	cg16397968	cg16397968	15795447	TTAAATCTAAACTAAACTCRT			II	
0.1970132	cg16398051	cg16398051	67633365	CCAACTC	34624302	CCGACTC	I	A
0.1009515	cg16398581	cg16398581	54711381	CAATATTAATATATTTCTACCA			II	
-0.174499	cg16400631	cg16400631	15771335	AAAATAC	39755401	AAAATAC	I	A
0.1082422	cg16405432	cg16405432	66643403	ACCATTACCCAAAACACTAAT			II	
0.1978374	cg16406892	cg16406892	43608374	AAAAAATAACRACTTTAAACA			II	
0.1932539	cg16412745	cg16412745	68659506	ACTAAACRTAAACTTCAACRC			II	
-0.103706	cg16414030	cg16414030	58663492	CRAACTCCCCTCACCTTCTAAA			II	
0.1141091	cg16414691	cg16414691	39688492	AAAATTTCTCTATTCTACCTCT			II	
0.1190104	cg16415646	cg16415646	18638488	TATATCTCAATAAATATATCCA			II	
	cg16416045	cg16416045	43706325	AAAAAAA	55629312	GAAAAAA	I	C
0.1245606	cg16418183	cg16418183	63772319	CACACCAAACAACCATCTAAA			II	
-0.16068	cg16419584	cg16419584	46647457	AAATTCC	74795414	AAATTCC	I	T
0.1351475	cg16421411	cg16421411	54701456	ACTATAA	61679335	ACTATAA	I	A
-0.182692	cg16423096	cg16423096	42782411	TAATATAATCTATAACCACAA			II	
-0.179357	cg16426293	cg16426293	63806360	AAAAAAA	18607354	GAAAAAA	I	A
-0.222785	cg16428357	cg16428357	12763437	AAAAATA	12702443	AAAAATA	I	A
0.1377229	cg16428517	cg16428517	19643510	ATCACTTAACCAACRTCACAC			II	
0.1061256	cg16432908	cg16432908	56670338	CCCTAAACAAAACAACCATAA			II	
-0.204909	cg16435686	cg16435686	63603337	AAATAAATCAATTACATATAA			II	

0.1109237	cg16436762	cg16436762	27747312	ACAAACATAAATCACRACATC			II	
-0.277218	cg16440058	cg16440058	61623436	ACAAACA	42729406	ACAAACC	I	C
0.1120127	cg16449636	cg16449636	11731360	ACAAAACTATTATAACTTTAAT			II	
-0.140504	cg16450309	cg16450309	36715420	CTACCAT	71656455	CTACCAT	I	A
0.1330306	cg16451306	cg16451306	74623409	TCCCAATATAAAACATTCCCCA			II	
-0.194651	cg16453056	cg16453056	59689453	AAAATACAACRAACAAATCAC			II	
0.1040922	cg16455339	cg16455339	57651414	AAATTATACCCAATTTTAAATC			II	
-0.112244	cg16458494	cg16458494	66632451	AAAAAAA	10603352	AAAAAAA	I	A
-0.102115	cg16460383	cg16460383	61602507	TCCAACCCTAAAATATCCTTAT			II	
	cg16467318	cg16467318	13644470	AAAAAACTTCCTCCAAATCCA			II	
-0.123208	cg16476432	cg16476432	53721323	ACAATATCTCAAAATCTCCCAC			II	
0.1911663	cg16477611	cg16477611	39660439	AAATTTATTACTTCTACTAAAC			II	
0.1269258	cg16479633	cg16479633	44641326	AATCACTAATAAATCACAAAA			II	
0.1014134	cg16481280	cg16481280	54714317	TTACAAAATCATTACTTAATCT			II	
-0.138242	cg16484499	cg16484499	32709458	CATCAACAAAAATAAAAAACT			II	
0.2906354	cg16490124	cg16490124	62797474	ACAAACAAAACTATCAACTAA			II	
0.1544791	cg16493238	cg16493238	66732473	ATATATACTACCAATAAATAA			II	
0.3242497	cg16494625	cg16494625	73728435	TATAATCTTTATTCCAACATCC			II	
0.1058856	cg16501561	cg16501561	73623317	AAAACAAAAAACTAATTAAAA			II	
-0.139668	cg16507569	cg16507569	31743395	CACTCTAATAAAATCCACAAA			II	
-0.177523	cg16514085	cg16514085	28678378	CTCCRTCCCATTAAAAAAATA			II	
0.103291	cg16514513	cg16514513	17773335	ACCCTAC	58738411	ACCCTAC	I	C
0.2594761	cg16515381	cg16515381	35604436	AAAATATAACRCAACTCACAA			II	
0.1038997	cg16516295	cg16516295	64797394	AAACCAAAATAAACTATAACC			II	
-0.148794	cg16527095	cg16527095	61749339	CAAACAAAATCRTTCCTTCCT			II	
0.1462642	cg16527629	cg16527629	21787337	CCCTTTAAAACTATAAAAAAA			II	
0.1184478	cg16531622	cg16531622	10689391	AAAATAAATAAACCTAAATTT			II	
0.1309639	cg16533146	cg16533146	14607344	AAAAAAAACATCCACAAAAAC			II	
-0.253425	cg16535653	cg16535653	58647457	TAAAATTAAAATTCTCCCCCA			II	
0.1832102	cg16540590	cg16540590	61661377	AACATACTACCCCAAAACATT			II	
0.1150123	cg16549644	cg16549644	69706449	AAATAAA	55803491	AAATAAA	I	A
0.1111948	cg16550264	cg16550264	70779475	AAAACTA	43672350	GAAACTA	I	C
-0.228418	cg16558994	cg16558994	54799486	TAATAACCACCAAAAAAAAAC			II	
0.1142541	cg16564828	cg16564828	11720494	ATCACRAAAAATATTAAAACC			II	
0.1443899	cg16566191	cg16566191	67803420	TTATAAAACAAACACAACCCT			II	
-0.117368	cg16568131	cg16568131	38617479	ACAAATT	16743341	ACAAATT	I	C
-0.106282	cg16570885	cg16570885	40644306	ATATTAACACTACTACATATCC			II	
0.1471606	cg16575544	cg16575544	38718324	AATCCTCCTAAAAACACCCAA			II	
-0.1014	cg16576544	cg16576544	30685373	ACAAAATCCRATAATTCTAAA			II	
0.136839	cg16576930	cg16576930	15746497	TCCTTAAACTCACCTTTACTTA			II	
0.1366734	cg16582419	cg16582419	31791419	AAATACA	56778505	AAATACC	I	A
-0.322963	cg16583552	cg16583552	40659477	ACATTCT	53641413	GCATTCT	I	C
0.1067075	cg16596317	cg16596317	10704506	AAAATAACTCAAACCCAAACA			II	

-0.154173	cg16600991	cg16600991	19676476	AACCAAA	72679384	AACCAAA	I	T
-0.137678	cg16602316	cg16602316	11806443	TACTAAA	46648400	TACTAAA	I	C
0.1102229	cg16603578	cg16603578	44617361	AACAATA	22607427	AACGATA	I	A
-0.140982	cg16606324	cg16606324	10633509	AACCACC	48719469	GACCGCC	I	A
0.1384419	cg16612072	cg16612072	21619432	AATCCAATAAACACRAAAACC			II	
-0.127349	cg16615357	cg16615357	26703479	AAAAATTTTATAAAAACCTTA			II	
0.1267298	cg16617257	cg16617257	41734407	ACCTACCACTCACTAAAAAAT			II	
0.1184735	cg16622899	cg16622899	22712323	CCAAATA	39604492	CCGAATA	I	C
-0.1039	cg16622906	cg16622906	32638312	AATTATA	13689444	AATTATA	I	A
0.1193766	cg16623941	cg16623941	43748403	ATTTAAACRAATACAAAATAA			II	
0.1027936	cg16627236	cg16627236	15764432	ATACTAAAATTATATATCCTTA			II	
0.1077748	cg16627911	cg16627911	55773343	TCTACTA	51629453	TCTACTA	I	A
0.1263313	cg16637458	cg16637458	33670508	CCTTAACAACTAAAAAAACCT			II	
-0.112787	cg16641638	cg16641638	42786453	AATAATT	54806449	GATAATT	I	A
0.2200589	cg16649728	cg16649728	36757390	CCAACTT	51722444	CCAACTT	I	T
0.1654699	cg16651314	cg16651314	37671494	CCTATTTTCATATTTCTAATCC			II	
-0.191399	cg16652920	cg16652920	40788502	ACTAATCACTAAACAACTACR			II	
-0.477228	cg16655291	cg16655291	74683493	ACACCCAAACTAAAAACAACR			II	
-0.101668	cg16655343	cg16655343	66739312	AATAAAAAAATATTCTTCCTC			II	
0.1005464	cg16656875	cg16656875	10784438	CAATTTTCATTACCCCTCCTCT			II	
0.1221485	cg16658460	cg16658460	20777316	TAATTACAAAAAATACTACCT			II	
0.1620326	cg16659773	cg16659773	41677386	ACAATAACACAAAAATAAATC			II	
0.1257239	cg16660494	cg16660494	57758504	ATAAATAAAATTCAATTTATC			II	
-0.160948	cg16662938	cg16662938	40787327	ACCTCCTAATTACTCACRCCAA			II	
-0.120094	cg16665310	cg16665310	57698390	TCCCATA	25800460	TCCCATA	I	C
0.1625218	cg16673522	cg16673522	49780451	CTCCTCATTCCCACAACCCAA			II	
0.1034482	cg16678159	cg16678159	42723496	ACCCTACTATCTACTCTTCACA			II	
0.119266	cg16681649	cg16681649	54754423	CTCCCTCTATAAAAATTAATCA			II	
0.1576221	cg16683060	cg16683060	42782316	AAACTCTTTCTATTTTAATCAT			II	
0.1203075	cg16686135	cg16686135	12638432	AAAAAAAAATCTCAAACAACC			II	
-0.122779	cg16692735	cg16692735	16718443	AAAAAAC	33758489	AAAAAAC	I	A
	cg16700025	cg16700025	61810327	CATTAAATAATCATCTCCRACT			II	
0.3327687	cg16702660	cg16702660	16631322	TCACAAAATCAAAACAAAATC			II	
-0.194558	cg16703135	cg16703135	46661465	ATAATAC	72773385	GTAATAC	I	A
0.1280525	cg16704476	cg16704476	12688449	TTTAAAACTTCTACTAATATAA			II	
0.263953	cg16704703	cg16704703	15797495	CRAAACAAAACATCTCCCAAA			II	
-0.182223	cg16705827	cg16705827	54802377	RTAAAAATACRTATCTATTACT			II	
0.1260255	cg16711011	cg16711011	48686329	AACAACA	61723395	AACAACC	I	A
0.1030479	cg16713321	cg16713321	50792364	ATCACCTTAAAACTCTTATATC			II	
0.1126983	cg16713625	cg16713625	63809322	TAAATAC	61685313	TAAATAC	I	C
-0.10248	cg16716316	cg16716316	61673380	AACAACC	13651442	AACAACC	I	A
0.1221179	cg16719517	cg16719517	46679337	TACATAA	32778503	TACATAA	I	T
0.1070346	cg16722884	cg16722884	32800508	AATACATAAAACAATATTTTA			II	

-0.10235	cg16722947	cg16722947	30626503	TATTCTAACATTAATCCCATTT			II	
0.1622316	cg16723716	cg16723716	34633487	ACCACAC	57732437	ACCACAC	I	A
0.1472095	cg16724332	cg16724332	72634429	TCTACAAAAACTTCRTAAAAC			II	
0.1055744	cg16724720	cg16724720	14750373	ACTTTCAATAATTCACTTTCAA			II	
0.1095255	cg16727923	cg16727923	26754342	CCATTATACCAATAAAAAAAA			II	
0.102289	cg16728846	cg16728846	66656509	CAAAACAAACATTCTACAAAA			II	
0.1711386	cg16735072	cg16735072	38734362	TATATACCAATAATCTCAATA			II	
0.2738643	cg16742075	cg16742075	65711321	TATTCTAATATTAATCCCAATT			II	
-0.141084	cg16744531	cg16744531	57607381	ACTAAAA	73703501	ACTAAAA	I	A
0.182982	cg16744961	cg16744961	60757411	TTTCTCCTATAAACCACTTCCT			II	
0.1139198	cg16747885	cg16747885	39769375	AAAAACT	52600460	AAAAACT	I	T
0.1053901	cg16747928	cg16747928	74666366	AAATAATCTACTAACTTAAAAC			II	
-0.107061	cg16748088	cg16748088	67644496	TATTTTATCTATAATTTATTTA			II	
0.1012799	cg16748433	cg16748433	62647492	ACRATAAAAAACCAAACCTAA			II	
0.1260486	cg16751493	cg16751493	61726311	CAAAAAAACACTTAAACCCCR			II	
0.1222122	cg16754788	cg16754788	43663360	TAATTACTCCTAAAACCTTCAA			II	
0.1139425	cg16755833	cg16755833	46701389	AATTTCA	11653323	AATTTCG	I	A
0.1346406	cg16765068	cg16765068	22662319	CTAAATCACCTAAACTAAAAC			II	
-0.193927	cg16767880	cg16767880	49709439	AACACAA	30660451	AACACGA	I	A
0.1017495	cg16768697	cg16768697	63725310	ATCTAAA	11665446	ATCTAAA	I	A
-0.14307	cg16775598	cg16775598	26788369	ATATTTACCTCCTCATTCTTTC			II	
0.1553755	cg16778382	cg16778382	29686413	AAACAAA	57612478	AAACAAA	I	T
0.1168207	cg16780454	cg16780454	17718342	AAAAAATTAACCRAAACRCTC			II	
0.1274886	cg16781907	cg16781907	15780398	TAATATATCCTTTCTTCTAATT			II	
-0.137094	cg16784985	cg16784985	65766375	AAAAAAA	63699429	AAAAAAA	I	A
0.1104448	cg16785291	cg16785291	11645344	TTACACAACTCAAAATCTAAA			II	
0.1097156	cg16787352	cg16787352	19624446	CTTTAACTCAATTACTACCAAT			II	
0.1199535	cg16788099	cg16788099	10720331	AAACACT	70768333	AAACGCT	I	A
-0.260946	cg16788857	cg16788857	53651382	TAAAAACCAATAAATATAAAA			II	
-0.11417	cg16789104	cg16789104	39704468	AAAAAAT	45642388	AAAAAAT	I	C
0.2898142	cg16791832	cg16791832	37644405	AAATCCATACAAAACCAAAAC			II	
-0.129898	cg16802801	cg16802801	11725370	TCTCCTCCTCCRAAATAAATTT			II	
-0.110704	cg16803846	cg16803846	29616304	TAAAACA	61796314	TAAAACC	I	T
-0.113184	cg16810310	cg16810310	57768347	AACCRAAATCAAAACCACATC			II	
-0.346302	cg16814680	cg16814680	11738460	AATAACTTTATAAAATTCCCCT			II	
-0.117883	cg16822572	cg16822572	73715405	TTTCAAA	21760444	TTTCAAA	I	A
0.1458271	cg16829924	cg16829924	48698301	TAAACTA	64800418	TAAACTA	I	A
0.1289538	cg16835531	cg16835531	10670397	ACCTATTAACTTCTAAAATAAT			II	
0.1797481	cg16836675	cg16836675	50727449	AAATTATTTCCTAATTACTAAA			II	
0.1812911	cg16841014	cg16841014	22679452	ATAAAATATATCCAAAATATA			II	
0.3076231	cg16848221	cg16848221	61804390	TATAAACCACTATACCCRACCT			II	
0.1258844	cg16852369	cg16852369	37748353	CCACTAATAATTTCTACACTAA			II	
-0.114529	cg16854917	cg16854917	10729435	ACTAAAA	51693408	ACTAAAA	I	T

-0.106856	cg1685535	cg1685535	43625305	TCTATCA	72735337	TCTATCA	I	A
0.1069602	cg1686039	cg1686039	17760330	TTCCCAAATACTTCTAAAATA			II	
0.1163412	cg1686352	cg1686352	37648364	AAAAAAACCTCTCCTATTTTA			II	
-0.119981	cg1686465	cg1686465	54668354	CTAATCCAAAAAATAAATCCC			II	
-0.116068	cg1686758	cg1686758	44708468	TTATTTC	23715328	TTATTTC	I	A
0.2813806	cg1687143	cg1687143	21642333	ACCATTCAAACTACTTTTATTC			II	
0.102445	cg1687418	cg1687418	35752411	CTCTCAATTACAAAAAAATTC			II	
-0.128398	cg1687708	cg1687708	61683422	CAAATAT	10674439	CAAATAT	I	A
0.1287643	cg1687768	cg1687768	46660353	AAAAAACTACCATTACCAAAA			II	
0.1587756	cg1689056	cg1689056	27803308	CCTAAAACTACCCACCCCATA			II	
-0.168199	cg1689819	cg1689819	33771462	TCCTTAAACAAAACCTAAAAT			II	
0.14983	cg1690426	cg1690426	15693510	TACCTACCTACCTAAACCCCA			II	
0.1644936	cg1690893	cg1690893	70749340	TCCACCTCTAAAAACTATCCT			II	
-0.150879	cg1691437	cg1691437	20636316	TCACTAA	44607314	TCGCTAA	I	C
0.323177	cg1691790	cg1691790	44682343	AAAAAAATAACCACCATTACA			II	
-0.165847	cg1692172	cg1692172	35751384	AAAATTCAATACCTCTCCCTA			II	
-0.109864	cg1692949	cg1692949	60667430	TAAAACAATAATACTAAATCC			II	
0.1338406	cg1692973	cg1692973	15669433	TATACAAACRCTTAAACACAC			II	
0.1525443	cg1693177	cg1693177	70737340	TAACCTC	51735392	TAACCTC	I	C
0.1079666	cg1693182	cg1693182	45695381	TATAACTTTTACACCATTATAA			II	
0.106005	cg1693282	cg1693282	42802482	ACAACTTAACTCTAATAAAAA			II	
0.1003477	cg1693642	cg1693642	71731399	AAAAAAC	46620479	AAAAAAC	I	C
-0.113352	cg1696757	cg1696757	35635502	CCCTAAAAAAAATAATTAATT			II	
-0.109323	cg1697751	cg1697751	33808376	TAAAAAAACACCRAAAAACCC			II	
0.1545755	cg1698821	cg1698821	48614330	ATAAATCRACCTCTACTTCTAA			II	
-0.134302	cg1698920	cg1698920	37653313	ACCCACCAAAAAAAACACRTA			II	
0.1242365	cg1699999	cg1699999	30712357	TTTTTCAATTCTTTATATTCAT			II	
-0.140238	cg1700762	cg1700762	30775418	AACTTCCRAAAATTTAATAAA			II	
0.10131	cg1700981	cg1700981	36691300	ACRAAAACTAAAATTCCCAAC			II	
0.1802241	cg1701369	cg1701369	14613376	AACAAAA	11784376	AACAAAA	I	T
	cg1701584	cg1701584	73809492	AACCRAACCCCTCAAAACCAA			II	
-0.216095	cg1701759	cg1701759	25761437	AAAACTA	55660322	GAAACTA	I	A
0.4738392	cg1702590	cg1702590	31682354	CTAATACCTAACACTCRATTTA			II	
0.1167865	cg1703284	cg1703284	47601482	TTTAAATATACRTTTACTATCT			II	
-0.118766	cg1703362	cg1703362	20651472	TTTACTAAAATACCCAAACCC			II	
0.1006681	cg1703389	cg1703389	55690443	AATTCAATTCAACCTTACRCA			II	
0.2194172	cg1704452	cg1704452	12616427	CTAAAATCTCTAAAACATATT			II	
0.1059249	cg1704703	cg1704703	32799388	TTACRACTTATATATCCCAATT			II	
-0.122852	cg1704861	cg1704861	29783469	TAACACT	46791320	TAACGCT	I	A
-0.135167	cg1705080	cg1705080	11688438	AATCCTAACCCTATATAATAT			II	
0.1108918	cg1705112	cg1705112	15719372	TTCRTATTAACCTAAAAACAA			II	
-0.111368	cg1705558	cg1705558	19643331	ATCCTAA	56656336	GTCCTAA	I	A
0.1379762	cg1705613	cg1705613	14802443	ATCTCATTTCATTCATCTATAA			II	

0.1273254	cg17058383	cg17058383	70617305	TAATTCTTAAAAAATCAACTCT			II	
-0.198775	cg17059388	cg17059388	18662368	AAATCTACACAATACATAATA			II	
0.1114122	cg17066275	cg17066275	12657472	TATCTTAATATTAATAAAACRT			II	
-0.108617	cg17070988	cg17070988	27606354	AATTCAC	71630392	AATTCAC	I	C
0.189421	cg17074339	cg17074339	11762494	CTACTCCCAAAACCTAAAAT			II	
0.1391307	cg17080138	cg17080138	25603418	TCCTTTAACTAAAACAACCTC			II	
-0.156146	cg17082366	cg17082366	43632386	CAAACAC	55654463	CGAACAC	I	A
0.1032604	cg17084358	cg17084358	58779333	CACTAAATTCATTCTTACATTT			II	
-0.10232	cg17093485	cg17093485	22676510	ACCTCTT	32753378	ACCTCTT	I	A
0.1501019	cg17096289	cg17096289	33628390	TCAAAAA	46699443	TCAAAAA	I	T
0.1182752	cg17097293	cg17097293	30690393	ATAAAAATAAAAACCRACRAC			II	
-0.154438	cg17098979	cg17098979	46633412	TTAAACAAACTTACTACAAAA			II	
0.1102264	cg17100047	cg17100047	14740471	TAAACTTAACAAAACRCTAAA			II	
0.131082	cg17112812	cg17112812	23659497	ATCACTC	64687370	ATCACTC	I	T
-0.176694	cg17125742	cg17125742	58737438	TTACCAA	50649421	TTACCAA	I	A
0.2201142	cg17126924	cg17126924	44698367	CTCTTTCTAAATATTCCRATTA			II	
0.1058873	cg17133045	cg17133045	54744446	AACACAATAATTATACCATAT			II	
-0.123131	cg17137077	cg17137077	52762384	AAAACTC	70634493	AAAACTC	I	A
0.1012809	cg17143179	cg17143179	61729372	AACTCAAAACACTAAAAATCA			II	
-0.102686	cg17149911	cg17149911	20707449	AAAATTATAAATCTCTCCTTTT			II	
0.1220544	cg17151123	cg17151123	21770331	TCTCAAACCTCACATAATTAAT			II	
-0.123265	cg17152375	cg17152375	49763499	CTACATC	74665311	CTACGTC	I	A
-0.204167	cg17155524	cg17155524	21615390	AAACRAAATAAACCRAAACTA			II	
0.10598	cg17162031	cg17162031	50706343	ATAAAAAATTAAACCATTCRA			II	
0.3019905	cg17162911	cg17162911	45807323	CCCTTTACTTCCTATTACTTTT			II	
0.1073971	cg17164093	cg17164093	55707310	AAAATTATAAACACAACCTCC			II	
0.1130946	cg17164345	cg17164345	60691426	TCATCAATAAAACATAATAAC			II	
0.2250962	cg17165241	cg17165241	69692400	CCACATC	23810364	CCGCGTC	I	T
0.1527894	cg17166262	cg17166262	67633425	TTATTTACTTCRAACAACTCAA			II	
0.1375283	cg17167832	cg17167832	40617361	AATACCCATTTTCCAATAATTA			II	
-0.108043	cg17168875	cg17168875	13743444	ATAAACCATAAATACCATCCA			II	
0.1061571	cg17178175	cg17178175	69748353	CCATCTCAACCAAAATAAAAA			II	
-0.120377	cg17178900	cg17178900	24757439	ATACATA	18682492	ATACGTA	I	A
0.1672069	cg17180633	cg17180633	60646378	RAAAATAATAACTCAAAAAAA			II	
0.1091811	cg17181941	cg17181941	40626314	TATCACAAATAACCACAAACC			II	
0.1197638	cg17186103	cg17186103	43674488	AACATTTTCACTCAAAAAAAT			II	
-0.193682	cg17187785	cg17187785	26692361	CCTCCRTCTCCACAATATAACC			II	
-0.116786	cg17193417	cg17193417	51661351	TAACAAACCCCACCCTCCTCC			II	
0.1734882	cg17196051	cg17196051	58774337	ACCRATACATAAATAATTATT			II	
-0.19195	cg17196155	cg17196155	24754456	AAAAAAATACTATTAATTCCC			II	
0.1073738	cg17198051	cg17198051	69694432	AACCAAATATATATAACRAAA			II	
-0.11396	cg17205386	cg17205386	53759508	TCCACCCCCAACCAAAACTAA			II	
0.1006501	cg17207064	cg17207064	36718408	AATCATACAACTAAAAACTAC			II	

-0.123267	cg17207724	cg17207724	38676425	AAAAATA	64765509	AAAAATA	I	A
0.2298062	cg17209769	cg17209769	57677340	AAACTAACAAAACAAAATATC			II	
-0.112488	cg17214408	cg17214408	74666430	TAAAAAA	66699412	TAAAAAA	I	A
0.1075129	cg17216092	cg17216092	34794502	ACATAAAAAATTCCTATAACT			II	
0.1029227	cg17220055	cg17220055	70646350	AAACTAACAAAAAAAATTCAT			II	
-0.227171	cg17220237	cg17220237	73774471	AATCATCACRTTTTAAAACTCC			II	
-0.106441	cg17221315	cg17221315	42636396	CRCTTTATAAAACCTCTAACCT			II	
-0.115848	cg17225604	cg17225604	66787301	ATCTCTATATAACTAATACRAA			II	
0.102698	cg17240198	cg17240198	28741440	AACTCAACCCATTAAAAACCA			II	
-0.127634	cg17242351	cg17242351	34734432	AAATCCC	65731463	AAATCCC	I	A
0.1277869	cg17246929	cg17246929	28738380	AAAATCATACCAAAATACTCR			II	
0.197942	cg17248267	cg17248267	55762375	CRCCCAACCAAATAAAACTTT			II	
-0.118076	cg17250082	cg17250082	47751472	AACAACAACATCTACAAATAC			II	
0.1374457	cg17251423	cg17251423	57779473	CCAAACC	33693464	CCAAACC	I	C
0.1073603	cg17253407	cg17253407	26789304	CTACAACAAAAACCCTTAACC			II	
0.163192	cg17253981	cg17253981	39748337	TTAAAACATCRAAACTAAAAA			II	
0.1212957	cg17257516	cg17257516	45621509	CTCTAACACAAACCAATAACA			II	
0.1417145	cg17272838	cg17272838	49706393	AAAACAACCTCCCAAAATTCC			II	
0.1363982	cg17273096	cg17273096	39645372	AACCTAAAAAAACAAATTTAT			II	
0.1329828	cg17273683	cg17273683	24688344	TACAAAACCCATTATTAAATA			II	
-0.116315	cg17276853	cg17276853	64668397	CAACAAC	18768374	CAACGAC	I	A
-0.164343	cg17277199	cg17277199	44648340	TTTCCCA	74611303	TTTCCCG	I	C
-0.190402	cg17292337	cg17292337	34808478	CCCACCTCTAACTCTTAAAACT			II	
0.2197661	cg17294865	cg17294865	18639475	CAAACAACCCTAATACCAACC			II	
0.1190613	cg17295834	cg17295834	62803373	TTCCTTATTTCATTTATAAATA			II	
-0.194622	cg17302580	cg17302580	33720308	TAAAAACCTACTCTATACTCA			II	
0.1150652	cg17312546	cg17312546	69604352	TCCTAATACCCTTAAAAAATA			II	
0.111391	cg17316073	cg17316073	55632466	ATCCTCTAAATTCAAAAAACA			II	
0.133077	cg17330048	cg17330048	48702307	AACTAATTAAAAATAACCAAC			II	
-0.138088	cg17330251	cg17330251	61784461	AAATAAAACTTTTAACTAAAA			II	
-0.183363	cg17331757	cg17331757	28761395	TACCTTTCTAAACCAAACCAA			II	
0.1135171	cg17333767	cg17333767	61796381	AATCRCCATTTAAAATAAACR			II	
0.1069854	cg17333973	cg17333973	60796449	ATCATTCACACTTCAATATAA			II	
0.1240088	cg17334266	cg17334266	64752491	AAACTAC	65758332	AAACTAC	I	A
0.122942	cg17335001	cg17335001	70662462	AAAAAAAACAAAAACAAACT			II	
0.1070034	cg17341345	cg17341345	52784434	ATCCTCTCCCTCTATTTTCATC			II	
0.2021699	cg17343184	cg17343184	62626346	CACAAAAATAACACAAAAACT			II	
0.1278659	cg17344932	cg17344932	37756330	ATATCAA	38659340	ATATCAA	I	A
-0.120085	cg17345036	cg17345036	50705397	AAAAAAA	55735450	AAAAAAA	I	C
0.1544649	cg17345450	cg17345450	35667417	AAAAATCCTCCCAAATAACAA			II	
-0.108032	cg17347178	cg17347178	65677341	CAAAAAAACTAACACAAAATA			II	
-0.255796	cg17348244	cg17348244	34619474	CTAACCTAACTACAACACCTA			II	
-0.207964	cg17351927	cg17351927	69628380	AAAAACCTCAAAATACAAATA			II	

-0.183388	cg17355877	cg17355877	32770399	TATACAACCRCTATAAATACA			II	
-0.122855	cg17360552	cg17360552	40713497	ATAACRCTACCAAAACTAACT			II	
0.1642425	cg17367816	cg17367816	44703447	AAACAAAATTTAACATAACA			II	
0.2170005	cg17369694	cg17369694	59740473	AAAACTAAAAAAACCACAAA			II	
0.2577129	cg17370616	cg17370616	38638395	ACATCTCTAACAATTTCTACA			II	
0.1294042	cg17382302	cg17382302	34648306	AATTCCACATAAAAAACCTAA			II	
0.1176763	cg17384124	cg17384124	14738462	ATCTACCTATTTAAACCAATA			II	
0.1129804	cg17384769	cg17384769	12738359	AATTTTCTTTATAAACTCRTCT			II	
-0.102915	cg17385708	cg17385708	72697330	AATATAACCRAAACCTATAAA			II	
0.2688403	cg17386240	cg17386240	56635354	TAAATTTAATAAATATTTCTTC			II	
0.1374567	cg17386473	cg17386473	58756404	ATAAAAAATAAATTCAAAAAA			II	
-0.461671	cg17388779	cg17388779	42751448	TAATAAAATTCACRTTAAATA			II	
0.1790711	cg17389813	cg17389813	70801395	TTCTCTCTAATTCATATCCTAA			II	
0.2848505	cg17393140	cg17393140	44749493	AACCTAAAACAAAAACCCTTC			II	
0.1082622	cg17395555	cg17395555	13802506	CTCCAAAAACATACTAAACTC			II	
-0.117889	cg17399283	cg17399283	60611360	TAAAAAC	63726326	TAAAAAC	I	A
0.7159346	cg17404449	cg17404449	64711377	CAACTATATTATAAATTCAAA			II	
-0.101443	cg17413084	cg17413084	33798377	AAAATTAATATATAATAAATC			II	
0.1192034	cg17415326	cg17415326	57742313	TACCRTATAAACTAAAATAAC			II	
-0.101641	cg17415381	cg17415381	38694432	ATAATCATAAACCTAATCTTC			II	
0.4214469	cg17421046	cg17421046	62610408	ATTAATAATTATCCAATTAAA			II	
-0.110457	cg17422543	cg17422543	50708387	TTTACTA	40618459	TTTACTA	I	A
0.1825851	cg17422915	cg17422915	54728465	TCATACTCCCACTATACTCTTA			II	
0.1261456	cg17425069	cg17425069	40649492	AAAAACRTCAAATAACATACA			II	
0.1117373	cg17426923	cg17426923	46713318	TTCCAAACACAAAATTCTAAA			II	
0.1278358	cg17442194	cg17442194	63790401	CAACTTTTAAAATCTTCACTAA			II	
-0.129264	cg17443359	cg17443359	60715486	CTAACTA	13669344	CTAACTA	I	A
-0.112093	cg17445765	cg17445765	43701337	ACCTTAA	61732401	ACCTTAA	I	A
-0.101947	cg17449954	cg17449954	31684386	AAACAA	22617405	AAACAA	I	A
-0.125351	cg17459023	cg17459023	60658314	CCACAAA	48644424	CCACGAA	I	A
-0.182564	cg17460368	cg17460368	56761388	TATACCTCATTTACACTAATCA			II	
0.1075377	cg17468459	cg17468459	66656324	AAACATTACTCTATCTACCAA			II	
0.1690773	cg17473712	cg17473712	49701335	CTTAAATCACTTAAACCAACT			II	
-0.103002	cg17474785	cg17474785	19620473	AATAAACTACTATAATAAAAA			II	
-0.164304	cg17474790	cg17474790	58636369	ACTTCCRAATAACTAACAAAA			II	
0.200087	cg17477578	cg17477578	42763346	CTAAACCTAAACTTCATAAAC			II	
0.1641881	cg17482114	cg17482114	46717450	TAAAATT	40620377	TAAAATT	I	A
0.1062292	cg17482424	cg17482424	16662422	CCTTATCACCAAAATACAAAA			II	
0.1062172	cg17490196	cg17490196	35727500	CRATCAACCAATTTCCCCRAA			II	
0.1341359	cg17491304	cg17491304	54757498	TAACTTATAAAATAAATTCTA			II	
0.1174001	cg17504791	cg17504791	21760487	CAAAATTTATAAACTCAATAA			II	
-0.124868	cg17515480	cg17515480	22677494	CRAAAAACCTAACCTCTAAAA			II	
0.1050131	cg17516247	cg17516247	55616388	TTTAATTAACAATTCACATTTC			II	

0.1099072	cg17516997	cg17516997	20670464	ATCTACATTAAATAAAAATTTA			II	
0.1048957	cg17519156	cg17519156	13806418	CACCATAATTTTATTACCTTAT			II	
-0.174568	cg17529386	cg17529386	53793338	AATAAAA	59806471	GATAAAA	I	C
-0.240002	cg17536720	cg17536720	30749407	CTCACCCCCATACCCATAACCA			II	
0.1868199	cg17537073	cg17537073	60713348	AATACAT	12623339	AATACGT	I	A
-0.140435	cg17543884	cg17543884	29697321	AAAAACC	47758315	AAAAACC	I	C
-0.124531	cg17544364	cg17544364	58678487	AAAATTATTTACCCCAAAACA			II	
0.1073387	cg17544584	cg17544584	61713469	CTTCCTACTAAAAAAATATTAA			II	
-0.135593	cg17546649	cg17546649	56642318	CAAAACATAAAAACAAATTCA			II	
-0.10435	cg17553149	cg17553149	50636422	AAAAAAC	40688384	AAAAAAC	I	A
0.1029873	cg17564498	cg17564498	73792452	TTTATAAATAACAAAATCCCA			II	
-0.124893	cg17566592	cg17566592	13668422	AAAATAACTAAACTACRAACA			II	
0.115022	cg17569842	cg17569842	47650435	TCACRAAATACAAATAACACT			II	
0.1367352	cg17581485	cg17581485	31794477	TATTTAAACCTCTTCTAACTTA			II	
0.1716151	cg17583957	cg17583957	61737317	CATTTACATTATTTCCTAAAAT			II	
0.101143	cg17586885	cg17586885	24687467	CACACAA	20660370	CACGCAA	I	A
0.2491621	cg17588704	cg17588704	11801433	CACAAAACCTAAACTAACAAA			II	
0.1166216	cg17588812	cg17588812	33720486	ACTACCA	49762315	ACTACCA	I	C
0.1463037	cg17590135	cg17590135	46682420	ATAACAA	64784322	ATAACAA	I	C
0.1290119	cg17593342	cg17593342	63762391	TAATAAAAAATTCAAATTAAA			II	
-0.115893	cg17597639	cg17597639	32787408	AACRAACCCTATTCCRAAAAA			II	
0.1272133	cg17601785	cg17601785	24640334	CACCTCACAAAAAAACCCTTA			II	
-0.100629	cg17611045	cg17611045	51783342	TTCAACA	12662447	TTCAACG	I	A
-0.197176	cg17611936	cg17611936	70725376	ACCTAAA	53803433	ACCTAAA	I	C
0.1326859	cg17613924	cg17613924	44667300	ACTTACT	21715415	ACTTACT	I	T
-0.13059	cg17619311	cg17619311	62762491	TTACTCT	33638303	TTACTCT	I	C
-0.269891	cg17626671	cg17626671	19642505	TACAATCTATCCTTACCCCTCT			II	
0.1304278	cg17631184	cg17631184	49774438	CTACTAAATAAAAACTCRCAA			II	
0.1180954	cg17633431	cg17633431	60767302	TTAACCAACTAAAAAAACAAT			II	
-0.10286	cg17639056	cg17639056	60754322	CAAAAAA	27808404	CGAAAAA	I	C
0.1097761	cg17643624	cg17643624	10698454	AAATAACAAAAAATTCTACTA			II	
-0.118511	cg17646499	cg17646499	21603415	AAAATACTTTATTTAAAAACA			II	
-0.148848	cg17655970	cg17655970	38694502	AAAAAAA	26658331	AAAAAAA	I	T
-0.179955	cg17658874	cg17658874	38622405	ATACAAATACATTTCTACCCAT			II	
-0.187589	cg17662493	cg17662493	73732393	CACATCCCTTCCCCTATATCCA			II	
0.1207178	cg17687367	cg17687367	46774480	ATTAATTCTTAATAAATAACTT			II	
0.1204482	cg17694130	cg17694130	11600347	CACTATT	35689408	CACTATT	I	T
0.1171219	cg17695537	cg17695537	62691303	ATACTTTCTATTAACTACTACC			II	
0.123436	cg17706972	cg17706972	40715497	CCCTAAAATACCAAAACATTTA			II	
0.1380851	cg17707274	cg17707274	29773376	TTAATATTTTCTACTAATAACT			II	
0.3474425	cg17707870	cg17707870	43670432	CATTCCACACATTTCTAATTCC			II	
-0.191892	cg17711541	cg17711541	57621337	TAACAATTTATTAAACTCCTCR			II	
-0.144021	cg17713488	cg17713488	29754432	ACCTACA	57727428	ACCTACA	I	A

-0.205696	cg17714861	cg17714861	39643303	CTCTATC.	48785319	CTCTATC.	I	A
-0.124512	cg1771610(	cg1771610(	15612475	TATTTTA(	40706329	TATTTTA(	I	C
0.1715669	cg1772320(	cg1772320(	20761434	ATCTTATTCATCCCCTCTTCTA(			II	
0.2935002	cg1773197:	cg1773197:	50780340	ATATAAC	11685308	ATATAAC	I	A
-0.151277	cg1773559:	cg1773559:	61740315	ATCTTATTTCTTTATATATTTCT			II	
0.2140073	cg1773777(	cg1773777(	29669336	TATACCC	50711333	TATACCC	I	C
0.1029518	cg17738521	cg17738521	64607510	TTAAAACTCCTAACAATATTC(			II	
-0.179426	cg1773861:	cg1773861:	33678338	AAAAAATTCTTACTAAAAAAA			II	
-0.10513	cg1774144٤	cg1774144٤	15746504	RAAACTTTTTATTATACTTCCT			II	
0.1907637	cg1774487٤	cg1774487٤	51762459	CTTATCAAAAACTAACAAAAC			II	
-0.299603	cg17749961	cg17749961	47763379	AACTCTAAAAATTAAATAAAC			II	
-0.113389	cg1775447:	cg1775447:	12657385	TACCTAC	72717470	TACCTAC	I	A
0.1365459	cg1775548:	cg1775548:	73631423	TACCAAATTATCAAACACCTA(			II	
-0.313765	cg1775925	cg1775925	33732332	CATTAAA	49729500	CGTTAAA	I	T
0.115703	cg1775959:	cg1775959:	69703437	ATTTCTTTCTAATTAACCACRT			II	
0.1103988	cg1776126:	cg1776126:	70764415	ATCTTACAACTATTTAAATATA			II	
0.116604	cg1776356(	cg1776356(	44796313	CTCAAACATATACTAAATCATT			II	
0.1053252	cg1776483٤	cg1776483٤	29623345	CTTTCAAAATATCTTAATTTTC			II	
0.1095819	cg1776499	cg1776499	38698498	AACAAAACTAAAACTTCAACA			II	
-0.127015	cg1777657(	cg1777657(	30768498	CTAATAA	42792354	CTAATAA	I	A
-0.10074	cg1778044	cg1778044	41752359	ACCTACRACCTTAACAATAAA(			II	
0.1083285	cg1778369	cg1778369	24653317	ACTTTACATCTAACTATTAATA			II	
-0.125004	cg1778525(	cg1778525(	31652419	AAAACAC	60723394	AAAACGC	I	T
-0.106316	cg1779481:	cg1779481:	58733440	AACAAAA	39617452	AACGAAA	I	C
0.1237014	cg1779794(	cg1779794(	54673319	ACCRAAATTACTCCTCCACCC(			II	
-0.346846	cg1780326٤	cg1780326٤	12663410	TAACACTAAACATCATCACRT			II	
-0.17923	cg1780411	cg1780411	71690317	CACTCAA	21626456	CGCTCAA	I	A
0.100799	cg1780818:	cg1780818:	22641331	ATAAAACTATAAAATTTATTC(			II	
-0.204879	cg17812431	cg17812431	20662375	TTAACATTAAAAAATCACACC			II	
0.1294457	cg1781251:	cg1781251:	38646365	CATACRCATCTCTACCTTCTTC			II	
0.1763644	cg1781617:	cg1781617:	21802414	CCCAAAATCRATAACTACTAT(			II	
-0.157809	cg17817521	cg17817521	74747366	AACTTAAATAAAACRCRTAAC			II	
-0.19515	cg1782006(	cg1782006(	39603403	TACCCCCACAAAAACATCRAT.			II	
-0.135353	cg1782294	cg1782294	64808470	TACTAAC	10634487	TACTAAC	I	C
0.1125023	cg1782689:	cg1782689:	42747303	ACAATTACATTTACCACTAATT			II	
-0.125296	cg1783117(	cg1783117(	69692402	ATACTACCTCATTCTCTATTCC			II	
-0.145887	cg1783303	cg1783303	49649482	TTTCCTTAACCCAATAACACCT			II	
0.284	cg1783716	cg1783716	69810325	TATAAAT	71724461	TATAAAT	I	T
-0.172409	cg1783816:	cg1783816:	10653390	CAAACATCCCTAAAATCAAAC			II	
0.1739466	cg1783836٤	cg1783836٤	66669365	AAATTAAAAAAATAATCAAAA			II	
-0.151161	cg1783975٤	cg1783975٤	70682329	ATTATTACCTCAACATCTTCCT			II	
0.1208454	cg1784004	cg1784004	58761423	TTTACAAATATACTCAACAAC.			II	
0.2335583	cg1784025(	cg1784025(	18672453	CTCTAACTTAAAATTAACCCTA			II	

0.1037852	cg17840997	cg17840997	52603312	ACRACTAAAATAAATTTTTCCA			II	
-0.193425	cg17843418	cg17843418	42727506	TATCCAA	33790411	TATCCGA	I	C
-0.101041	cg17845680	cg17845680	70783337	CTTAATATCACTACTTAATAAA			II	
-0.107316	cg17848407	cg17848407	51725388	RAACTCCCAAATATAATTACAC			II	
-0.256878	cg17851276	cg17851276	36699438	AATCCACAAAACCAAAAAAAC			II	
0.1027952	cg17852326	cg17852326	64767390	CTACAATTAAATATATCAATAT			II	
0.1536143	cg17855222	cg17855222	58603355	TAACAATATTTTCCTAATTCTT			II	
0.1179899	cg17862558	cg17862558	38697493	AACATATCTCTTCAAAAATATT			II	
0.1033866	cg17862903	cg17862903	23774440	ATAATACCAAACRAAATCAAT			II	
0.1307446	cg17867776	cg17867776	40605438	RCTTCCCRATAAAAACAAACTA			II	
-0.192245	cg17871621	cg17871621	35794392	CTTAACCCCRAAACRCAACTAC			II	
0.127314	cg17874109	cg17874109	52697443	TAAAACAAAAATTAAAATTCA			II	
0.2022412	cg17876294	cg17876294	40769337	TAATAACCAAAACRCTCTTCTC			II	
0.1382723	cg17878501	cg17878501	26600357	TCAATACCATTACAACAAACA			II	
-0.109898	cg17880703	cg17880703	48690350	RCAACTAAAAAATACAAATAC			II	
0.1055588	cg17883068	cg17883068	67673459	ATAAATCAAAAACCAAACTAA			II	
0.1061789	cg17884766	cg17884766	31729411	AAAACTAAAAACTATAATCAT			II	
0.1032433	cg17885794	cg17885794	61754331	CCAATCACTAAAAAACAATCC			II	
0.1768849	cg17894362	cg17894362	65804408	CACAACAACCCTAACAAAACT			II	
0.1746683	cg17894577	cg17894577	64615429	CTTTCTTTTTACAACCTACTAC			II	
	cg17901463	cg17901463	13609457	TATCCCAATACCCCAATATCAT			II	
0.1439167	cg17903506	cg17903506	46677482	AAACATAACTATTACATCCTCT			II	
-0.15835	cg17906851	cg17906851	16669375	CCTAAAA	68732485	CCTAAAA	I	A
-0.132268	cg17911021	cg17911021	73700473	TTTCTAAATCTAAAAAAAAAC			II	
-0.197236	cg17913405	cg17913405	68658442	AAAAACA	51785358	AAAAACC	I	C
-0.115184	cg17915676	cg17915676	23747310	CCTTCCA	16603441	CCTTCCA	I	C
0.1471486	cg17922998	cg17922998	31729313	ACRAACCATCCAAACCAAAAT			II	
-0.113782	cg17929042	cg17929042	61732494	AAAATAC	61785438	AAAATAC	I	A
-0.384419	cg17929630	cg17929630	42778478	CATTTTCA	24628430	CATTTTCC	I	T
-0.107324	cg17932096	cg17932096	54680411	TAAAAAA	71634319	TAAAAAA	I	C
0.1426922	cg17934277	cg17934277	43775397	ATTTAATAATATCCCTATCCTT			II	
0.2281895	cg17943323	cg17943323	10725345	AATATCT	74710311	AATATCT	I	A
-0.121964	cg17951978	cg17951978	54721318	CCATCAAAAATTACRCTTTTCT			II	
0.1056198	cg17958516	cg17958516	26612425	ACAAAACCAACTACTTCCAAC			II	
-0.118963	cg17962638	cg17962638	57691346	TAACAAC	48753432	TAACAAC	I	C
-0.20511	cg17967673	cg17967673	42652479	CACCACA	47683506	CACCACG	I	A
0.1058573	cg17972013	cg17972013	55801377	CCAACCAATAATAAAATATAA			II	
0.1450306	cg17976473	cg17976473	18628445	TATAAAACCCAAAAAACTATA			II	
0.1107633	cg17991919	cg17991919	48806380	ATCACATAACATATAATAACRC			II	
-0.228886	cg18001722	cg18001722	11803395	TTTTACRCACRAAAACTTTATT			II	
0.2323685	cg18013856	cg18013856	14741457	AAACCATAAAACTAAAATCRC			II	
-0.134231	cg18015035	cg18015035	55684336	AAAAACT	53622345	AAAAACT	I	A
0.2583807	cg18015625	cg18015625	51612387	AAACAAAAACATTTAATTCAT			II	

0.3126044	cg18021992	cg18021992	22634366	AACCTCATTATTCTTCAATAAT			II	
0.1125967	cg18025438	cg18025438	39724429	AAAAACC	53600384	AAAAACC	I	A
0.127504	cg18027903	cg18027903	64659303	TCTATACTTATTCTTAATCCTT/			II	
-0.26741	cg18029321	cg18029321	14773369	CACCTAA	15627427	CGCCTAA	I	T
-0.369439	cg18049365	cg18049365	20749317	CTTTCAAAAACTCTAAAATCA/			II	
-0.230151	cg18058072	cg18058072	19698435	CTCATTCATTTCATACTTTTTCT			II	
0.1021158	cg18065195	cg18065195	56632432	ACCRCTACTAACAACTAAACR/			II	
-0.15862	cg18065318	cg18065318	57722402	AATAATA	28716370	AATAATA	I	A
0.1752836	cg18066181	cg18066181	15764473	CTAATTAAATCAAAAACCCAA			II	
0.1314448	cg18066206	cg18066206	40766387	AAAACTAAAAAAATTCTCRTT(			II	
0.1234475	cg18073702	cg18073702	10662424	CAACCAATAATATTAAATAAC			II	
0.1103124	cg18075691	cg18075691	44667386	TAAAAAC	40687509	TAAAAAC	I	A
-0.1171	cg18077580	cg18077580	65638428	ACAATCT	55721395	ACGATCT	I	A
0.1019554	cg18085660	cg18085660	18797349	ATATTATAAACTACATACRCT			II	
-0.135857	cg18087477	cg18087477	46682331	RAAAACCATTAATTCTTTCTAT			II	
0.1058723	cg18087694	cg18087694	61657497	CTAATCCCTCACATCTTTTAAT			II	
0.1325563	cg18088906	cg18088906	67808397	TAATTTAAATATCACTTCTAAT			II	
0.1146267	cg18089380	cg18089380	35673462	ACTAAAAAATATACTATCACC/			II	
0.1660911	cg18089426	cg18089426	46749487	AACCTAC	21664493	AACCTAC	I	C
0.1980699	cg18093448	cg18093448	50789402	AACTAATACAATTTATTATACT			II	
0.1596284	cg18096251	cg18096251	15772317	AACACCA	31766326	AACGCCC	I	C
0.1004345	cg18098433	cg18098433	46705300	TAATAATATAACAACCRTCTT			II	
-0.22747	cg18098534	cg18098534	45759375	AACCAAATTAATAATACTAAA			II	
-0.185104	cg18101138	cg18101138	56696302	AAAACAA	33732501	AAAACAA	I	C
0.1557791	cg18101140	cg18101140	73696454	ACRAAACAACRAACCAATTTC			II	
0.1009825	cg18102738	cg18102738	16785412	TACTAAAACTACATCTCCAAA			II	
0.1584307	cg18104070	cg18104070	74644413	CACCCATAATATACACATTTTT			II	
0.1092128	cg18109485	cg18109485	23629378	ATTTCTCRAATAACTAACAAA/			II	
0.1971397	cg18111114	cg18111114	51629438	TCAACTATAAAACATAAAATA			II	
0.1002852	cg18113731	cg18113731	67743477	AAATAATCCTAAACAAACCTC			II	
0.1313249	cg18116815	cg18116815	20656325	CACTATTTAACCTATTTCAATC			II	
0.142206	cg18120259	cg18120259	60660316	AAAACATAATACAACCTACTA			II	
-0.140565	cg18127410	cg18127410	18628506	AAAAAA/	30793406	AAAAAA/	I	A
-0.148696	cg18128914	cg18128914	41774408	ACAAAAA	31634314	ACAAAAA	I	A
0.1679237	cg18129296	cg18129296	52643353	AACATTTCAAAACTTAAACTCT			II	
-0.11002	cg18129748	cg18129748	64665323	ATAAAAA	27793432	ATAAAAA	I	A
0.1102698	cg18129996	cg18129996	27733307	ATTTCAAAAATCTAACCATCC/			II	
0.105994	cg18132363	cg18132363	59669394	TTCAAAAAACRACAAAAAACT			II	
0.3001747	cg18137779	cg18137779	16712471	TCACAATAACCACTTTAATAA/			II	
-0.103404	cg18138788	cg18138788	67667410	CRACAACTAAAAAAAATTACT			II	
0.1010239	cg18142730	cg18142730	17650424	ATCACTACTTTATTTAATTACA			II	
-0.134865	cg18146506	cg18146506	31784507	CCAAAAA	35749469	CCGAAAA	I	A
0.1066548	cg18167946	cg18167946	49786505	AATAATAACCTCATCTCCCTAT			II	

-0.169742	cg1816892⁄	cg1816892⁄	65652445	AACATCTTATCCATAAAAATA(			II	
0.137552	cg1817054	cg1817054	51703355	CTAATACCAAATATATAATAT/			II	
0.2081508	cg1817251(	cg1817251(	47666425	ACAATCAATAACTCAACATTT			II	
0.2254567	cg1817883(	cg1817883(	62753314	TAATAAACACTCCCCCAAAAC			II	
-0.105449	cg1818214	cg1818214	65806464	TTTCCAA'	14759469	TTTCCGA'	I	A
0.1393242	cg1818421	cg1818421	39675302	ATTTTCA.	60713458	ATTTTCG.	I	T
-0.130776	cg1818911	cg1818911	24641487	AAACCACCACTTATAAAATTT			II	
0.1770639	cg1819174	cg1819174	39654422	CTACTCTATCACTATTAAACTA			II	
-0.118207	cg1819759⁄	cg1819759⁄	30777480	TTACCTT'	22606501	TTACCTT'	I	A
-0.103151	cg1820685	cg1820685	71800437	TCATCAAAACTCCAAATTCCC			II	
0.102721	cg1820935	cg1820935	39698449	CAACCTT	30614324	CAACCTT	I	C
0.1202112	cg1821762	cg1821762	63639332	AAATTTC	29753412	AAATTTC	I	C
0.1002821	cg1822134	cg1822134	44712443	CACAAATAATCCRCTCACTAA/			II	
0.1273358	cg1822919(	cg1822919(	32720509	ACRTAATAACCTCTATAATAT(			II	
-0.385677	cg1823223	cg1823223	37622316	ACCCAAA	19617432	ACCCAAA	I	C
0.1326065	cg1823574(	cg1823574(	74796359	TAAACCTACTCATAAAAATAT/			II	
-0.12774	cg1823658⁄	cg1823658⁄	56748399	TAAAAATTTAACCCRAACAAA			II	
	cg1823739	cg1823739	54606493	AAAACCAAAACAAAAAAACC			II	
0.1007781	cg1823773	cg1823773	59617323	CCATTCCTAAAACTCCTAATTA			II	
-0.261718	cg1823951	cg1823951	31669324	CCCCAACCATAATCATAACAC.			II	
-0.108923	cg1824039(	cg1824039(	34758345	ACTAAAA	20710362	ACTAAAA	I	C
0.1074727	cg1824155	cg1824155	36766333	AAAACAACAAAAACTAAAAA(			II	
0.1149931	cg1824384(	cg1824384(	59632301	AAATCACTTCRAAATCATTTC			II	
0.1031601	cg1825694	cg1825694	70761332	CTAATCC	18774336	CTAATCC	I	A
0.108896	cg1826316(	cg1826316(	32613407	CTAAATCAACATACAACCACA'			II	
0.1132903	cg1826409	cg1826409	51614455	AATAACTCAAAATAATTCTAT/			II	
0.1776353	cg1828533	cg1828533	62704331	TACCAACTACRCAAAACCAAA			II	
0.1095606	cg1828563(	cg1828563(	42663455	AATAAAAACTCTATAAAAATA			II	
-0.147138	cg1828853	cg1828853	12763351	ATAATAC	24637492	ATAATAC	I	A
0.1206571	cg1830246(	cg1830246(	63637364	AAACCCCTCTATAACTAAAAA			II	
0.1052384	cg1831063	cg1831063	22750426	TATTCATTCTAACTCAAATTAT			II	
0.1210127	cg1831309⁄	cg1831309⁄	20750478	RAAACATCATAATCACTATCA/			II	
0.117412	cg1831341(	cg1831341(	56641438	AAAAATCAAATCTTTTTTCCC			II	
0.1184685	cg1831936(	cg1831936(	59609370	TTAACTCAAATTTCTCACTACR			II	
0.129956	cg1832192	cg1832192	38679509	ATAAACAATATAAAATTCCTA(			II	
-0.110356	cg1832202	cg1832202	73790449	TAACAAAAATCTTCCCAAATA.			II	
-0.140279	cg1832258	cg1832258	38604422	CACATTT	32672417	CACATTT	I	C
-0.157782	cg1832712	cg1832712	43622441	ATCTTAA	72679507	ATCTTAA	I	A
0.1641619	cg1833281⁄	cg1833281⁄	24660360	CTTACAACTAATCTAACTCAA/			II	
0.1491073	cg1833283	cg1833283	15750345	ATAAAATAAACAATTCTAAAA			II	
-0.171808	cg1833309	cg1833309	60758320	TCTACAATTCTTTACTTCAAAA			II	
0.2231824	cg1834663⁄	cg1834663⁄	25688486	AAAATCCRACTCCAAATTAAA			II	
0.2930605	cg1835921	cg1835921	63713492	AACTTCCTAAAACACTAAAAC'			II	

0.1574388	cg18361425	cg18361425	38652398	AAACAATTAATCACCTAATAA			II	
-0.120388	cg18363918	cg18363918	44720423	TATATAT	72657405	TATATAT	I	A
0.1641316	cg18369034	cg18369034	41755416	TCATCCCACTAAAATTCCACA			II	
-0.102709	cg18373318	cg18373318	57639333	TTTTACA	70647447	TTTTACA	I	A
-0.108046	cg18383984	cg18383984	59712332	AAACAA	62658412	AAACGA	I	T
	cg18384097	cg18384097	54661343	TTAAAAACATCAAATCTATAA			II	
-0.184111	cg18391209	cg18391209	15607400	CCTAAATACTTAATACCAAAA			II	
0.1584497	cg18395354	cg18395354	50618432	ACACACTAAACCTAATATAAA			II	
-0.208239	cg18396987	cg18396987	42719428	RATAATACRAACAATACAAAA			II	
0.1351091	cg18397450	cg18397450	71715420	AAAACAA	39692388	AAAACAA	I	A
0.1265922	cg18400281	cg18400281	44685449	AAAACATTTATACRAAATCAA			II	
-0.386823	cg18403673	cg18403673	35631307	AAACTAAAAACAACTCAACTA			II	
0.1169848	cg18408273	cg18408273	67686379	AAAATCAACTTTATCAAACTC			II	
-0.163195	cg18413218	cg18413218	62675325	AAATCTTTAAAACAATACCTT			II	
-0.203665	cg18417245	cg18417245	30753390	ATTCCTTTCTTATAAACTTCTT			II	
-0.11558	cg18417622	cg18417622	22740372	CRTAACTTAATACTACACRCT			II	
0.119258	cg18418242	cg18418242	24632416	AACTAAATTCAATCTTACTAA			II	
0.198792	cg18424635	cg18424635	21718331	TTAATAATAACCTTATACCAA			II	
-0.138098	cg18431856	cg18431856	55604411	AATCCACRTCCTAAATAACAT			II	
0.1163236	cg18434356	cg18434356	72748422	ATAAATAATTTCCTAATTCRCT			II	
0.102613	cg18436272	cg18436272	17740354	AAAATAACTCTAACCTCATAA			II	
-0.12538	cg18437193	cg18437193	12788411	AACAAAT	58659490	AACGAAT	I	A
0.1348948	cg18437551	cg18437551	10800325	ATCRCCTAAATCAAATATAAA			II	
-0.231269	cg18443741	cg18443741	22728380	TCTTTAAATACATATTCTACTC			II	
-0.189389	cg18444757	cg18444757	20699419	CRTATTTCCAACACAAAATTTA			II	
0.1230713	cg18446441	cg18446441	42799423	TCTATTCTCCTCCTACTCAAAT			II	
0.1377949	cg18456803	cg18456803	47788300	AATTTACATATTTAACCTAACA			II	
0.100613	cg18458353	cg18458353	22756322	CACAAAAAACAAAACAAACRT			II	
0.1486713	cg18476530	cg18476530	74600356	TATTATC	44758432	TATTATC	I	C
0.1172266	cg18476566	cg18476566	30646465	AACAAATTTACAATAAAAACC			II	
0.111286	cg18480654	cg18480654	70607379	TAAAAAACAAACTATAAAAAA			II	
0.1148528	cg18486231	cg18486231	52645457	AAATACTTTCCTAACTATATAA			II	
0.1230933	cg18492943	cg18492943	17605353	TCAAAATTCTAAAACCTAATTA			II	
0.1581235	cg18497238	cg18497238	43603334	AACCTTTCCCTACTAAAATCTC			II	
0.1167981	cg18498115	cg18498115	74803328	CCAACCTAAATACTAATATAC			II	
-0.108598	cg18500448	cg18500448	18746310	AACATAA	21703329	AACGTAA	I	T
0.1322493	cg18502238	cg18502238	14718442	AAAAAAACACRTTACTTTCAT			II	
0.1390706	cg18503829	cg18503829	35600395	CACATAAAAAACACAAACCCA			II	
0.1276995	cg18504989	cg18504989	35754305	ACRAAAAACCAAAACAACTTC			II	
-0.112645	cg18509524	cg18509524	36778350	CCATAAAATACRCCCTTCTAA			II	
0.1041792	cg18512930	cg18512930	43642477	RAAAAACTCCCRAAAAAATTC			II	
-0.121647	cg18514705	cg18514705	18775345	CTCCAAAAACAATACTATATA			II	
0.1095858	cg18526142	cg18526142	31777387	TCTATTTCATTAACCTATAAAA			II	

0.123915	cg1853074[illegible]	cg1853074[illegible]	27674480	CAATAACTACTAAAAACTAAC[illegible]			II	
0.1993686	cg1853866[illegible]	cg1853866[illegible]	57613469	CATCCTCCTACCCTCTTTATTA[illegible]			II	
0.1110601	cg1854067[illegible]	cg1854067[illegible]	47703489	TATAAAA	34638494	TATAAAA	I	T
0.1517766	cg1854488[illegible]	cg1854488[illegible]	56619423	ACTACATTACTAAAACCACCA[illegible]			II	
0.1367189	cg1855021[illegible]	cg1855021[illegible]	47738353	AATAATATTACCTCTTCCTACC[illegible]			II	
	cg1855896[illegible]	cg1855896[illegible]	24730370	TATAACA	62631352	TATAACA	I	A
-0.182236	cg1855896[illegible]	cg1855896[illegible]	32634441	TTATATTTCATTTATCTACATA[illegible]			II	
-0.315104	cg1855990[illegible]	cg1855990[illegible]	33808339	ACCTCCTAACRAAACCCTAAA[illegible]			II	
-0.176286	cg1856167[illegible]	cg1856167[illegible]	23642315	AAAAAA[illegible]	25610431	GAAAAA[illegible]	I	A
0.1179315	cg1856655[illegible]	cg1856655[illegible]	69619467	CTTCTACCAAATAATACCCCA[illegible]			II	
0.1182747	cg1857414[illegible]	cg1857414[illegible]	41738380	AAAATCTCATAAAACTCAAAT[illegible]			II	
-0.419786	cg1857604[illegible]	cg1857604[illegible]	68603321	RTAAACTCTACTTTTCTAATTC[illegible]			II	
0.1205476	cg1857897[illegible]	cg1857897[illegible]	44742476	TAATTTACAAATATAAAAACC[illegible]			II	
0.1211913	cg1858688[illegible]	cg1858688[illegible]	59686310	AAAAACA	25634375	AAAAACA	I	A
-0.102758	cg1858747[illegible]	cg1858747[illegible]	66713393	TTCCATT[illegible]	16746438	TTCCGTT[illegible]	I	A
0.1119716	cg1859405[illegible]	cg1859405[illegible]	42643354	TCTATACTTCTACAAAAATTTA			II	
0.1012937	cg1859470[illegible]	cg1859470[illegible]	16771328	ATCTCCCACACTAAACATATT[illegible]			II	
0.1022209	cg1859513[illegible]	cg1859513[illegible]	50743404	CAAATTTATACAAAATAATTC[illegible]			II	
0.1120371	cg1859534[illegible]	cg1859534[illegible]	49600459	TTCAATTTTCCCATAAATCATA			II	
0.1605324	cg1859662[illegible]	cg1859662[illegible]	67608471	AAAAACC	56619403	AAAAACC	I	A
0.1184967	cg1859693[illegible]	cg1859693[illegible]	45791316	AAACCCAAAATACAAAATAAT			II	
0.1371964	cg1859920[illegible]	cg1859920[illegible]	22725391	AAACCAAATTCTCACACCATC[illegible]			II	
0.111929	cg1860912[illegible]	cg1860912[illegible]	20639370	ATATTCTCTACAACTTAACCTC			II	
-0.109142	cg1861216[illegible]	cg1861216[illegible]	19683443	TACAAAAATTCTTACATCTAA[illegible]			II	
-0.124897	cg1861473[illegible]	cg1861473[illegible]	72666418	TCAAACTAAAACTACTAAAAA			II	
0.1432321	cg1861881[illegible]	cg1861881[illegible]	72789442	TTCTTCTCAAAAATCCCCAAA[illegible]			II	
0.287259	cg1862167[illegible]	cg1862167[illegible]	54794373	CCCTAACCCTAAAAACTACAC[illegible]			II	
-0.101226	cg1862185[illegible]	cg1862185[illegible]	49649455	TCCTAAACAAAACATTCCAAT[illegible]			II	
-0.123252	cg1862530[illegible]	cg1862530[illegible]	38786353	TAAAACATCCRAAAAACTTAA[illegible]			II	
0.1420608	cg1862885[illegible]	cg1862885[illegible]	15752508	TACTATAAAAAAATAACAAAA			II	
-0.100158	cg1863064[illegible]	cg1863064[illegible]	30663428	AAAACAC	71604489	GAAACGC	I	T
-0.103641	cg1863421[illegible]	cg1863421[illegible]	65675489	ATTATCTTTTAAAATTTAAAAA			II	
-0.146431	cg1864470[illegible]	cg1864470[illegible]	24744359	ATATTAC	42742445	ATATTAC	I	A
0.1011642	cg1865021[illegible]	cg1865021[illegible]	26727330	CCTTCAAAATAAACAACTTAA[illegible]			II	
0.1714313	cg1865036[illegible]	cg1865036[illegible]	44770510	TACAACATATATCTCCTTTAAA			II	
-0.139179	cg1866765[illegible]	cg1866765[illegible]	18600413	AAATTTC	47657432	AAATTTC	I	C
-0.12202	cg1866851[illegible]	cg1866851[illegible]	73803381	TACCCAAAACTCAATTAACAT[illegible]			II	
0.1236123	cg1867203[illegible]	cg1867203[illegible]	61724489	TTCCAATTAATCACAAATTAA[illegible]			II	
-0.10013	cg1867696[illegible]	cg1867696[illegible]	62800469	RAACACTCCATCAAAACCCCC[illegible]			II	
-0.149826	cg1867864[illegible]	cg1867864[illegible]	30651474	RACCCRAATAAAAATATACRA[illegible]			II	
-0.19206	cg1867950[illegible]	cg1867950[illegible]	24634453	ATCCCAC	45682460	ATCCCGC	I	A
0.1130465	cg1868018[illegible]	cg1868018[illegible]	55777484	TCCCTTAATCTCRTTAATTACT[illegible]			II	
	cg1868475[illegible]	cg1868475[illegible]	48604510	AACAACATAACCAAATAACTT[illegible]			II	

0.2581907	cg18698799	cg18698799	71781488	ACTAAATAAACTATTCCCAAA			II	
-0.323535	cg18699744	cg18699744	17610311	ATCAAAT	50803478	ATCAAAT	I	C
0.1004679	cg18707191	cg18707191	56623390	AAATCAATTACCAATAATTAC			II	
-0.110508	cg18709881	cg18709881	13716416	CACTACT	35614448	CACTACT	I	A
0.1010795	cg18709904	cg18709904	36760466	TACACATCCCRCTACACTAAAT			II	
-0.165272	cg18710053	cg18710053	57759456	AATTAAA	30709321	AATTAAA	I	A
0.1138744	cg18710162	cg18710162	52787416	AACAAAATTACAACACAACAA			II	
0.1759358	cg18711535	cg18711535	17630435	CTTCTAAATCAAACTTCATTAC			II	
0.1867899	cg18715193	cg18715193	55607332	AAAACTTCCAAAATCCCCCAA			II	
-0.114828	cg18719563	cg18719563	62610332	AAACTTT	42774428	AAACTTT	I	C
0.1067972	cg18720803	cg18720803	36780455	ACCCTAAATAATCAACAATAA			II	
-0.100907	cg18723276	cg18723276	24728328	AAATTAT	69768312	GAATTAT	I	C
0.1155358	cg18730865	cg18730865	44794322	AAACTACCCTATACTTTAACTC			II	
0.10712	cg18736835	cg18736835	62763467	TAAAAAAAACAATAAATATCA			II	
0.1566658	cg18748525	cg18748525	64734310	TATAAATCAATCTCACRTCCCC			II	
-0.163294	cg18755531	cg18755531	65673494	TACAATA	26705407	TACGATA	I	C
0.1009873	cg18756771	cg18756771	58784415	AATAAAAAACAATAAAACTAT			II	
0.145358	cg18756931	cg18756931	18673412	TACTCTCTATACCTAATCTACA			II	
-0.105717	cg18757172	cg18757172	21604380	ACAAACT	50633484	ACAAACT	I	A
-0.219723	cg18757817	cg18757817	46676349	TCCAATAAACATTATCTACCCT			II	
0.1803087	cg18761958	cg18761958	59759492	ACAACCTATAACTACTCAACT			II	
0.1501355	cg18761976	cg18761976	13705477	AATCAAATAACTCACACTAAA			II	
-0.111995	cg18762806	cg18762806	18783464	CTCAACC	61769414	CTCGACC	I	A
0.1246113	cg18764164	cg18764164	54684365	ACCAACCCTAAAATATAACTC			II	
0.1054272	cg18764771	cg18764771	23773404	TTACTTTCRCCAAAAACTCTCC			II	
-0.140943	cg18765565	cg18765565	30701412	ATAAAAC	69737303	ATAAAAC	I	A
-0.183061	cg18770944	cg18770944	34736429	ACTAACTAAAAAACRCCTAAA			II	
-0.138546	cg18771553	cg18771553	30745384	AATACCAACCTATCCCCCCAA			II	
-0.106655	cg18771570	cg18771570	16795398	ATTTCRAAAACTTATAAACACT			II	
-0.196286	cg18771937	cg18771937	45603442	CCCAACAACATAAAACTACCT			II	
0.1217269	cg18773937	cg18773937	66607479	CATTTTACAAATATATCACCAT			II	
0.1305732	cg18774170	cg18774170	15750484	TATTTAAAAATAAATCCCRTTT			II	
0.2266313	cg18776460	cg18776460	59716352	TAACATAAACTCTAACTTACCT			II	
0.1314995	cg18782604	cg18782604	33640410	ACTCTCT	69771399	ACTCTCT	I	A
0.1120118	cg18786224	cg18786224	15670369	AATCCRAACTCAAAATCCCAC			II	
0.1183402	cg18790340	cg18790340	21802416	CCCCAAATTTAATCTAAAAAA			II	
-0.528201	cg18792536	cg18792536	59607453	ATACCAC	37656490	ATACCAC	I	C
-0.112581	cg18797653	cg18797653	65706357	AATTCATAAACTACTTCRAAA			II	
-0.190787	cg18801505	cg18801505	29715318	CAACTAACAACAAACTACTCT			II	
0.2917731	cg18805164	cg18805164	51795502	CAAAAAA	49795427	CGAAAAA	I	A
0.1278639	cg18806716	cg18806716	34733372	CATTTCCCACRTATCAAACACC			II	
-0.1151	cg18808261	cg18808261	31775510	TTACAAAACRAAATCCTTTTCA			II	
-0.410905	cg18816122	cg18816122	40800307	ACTTTTA	60804314	ACTTTTA	I	C

0.116274	cg18816397	cg18816397	63781395	AAAAATCCTTACTCAAAATTAA			II	
-0.101911	cg18822951	cg18822951	39802455	TACTAATAAACRCACACCATCA			II	
0.5531551	cg18828268	cg18828268	45742508	CCACATAAACACAAAAAAACA			II	
-0.124472	cg18828365	cg18828365	68786375	CACTACTTAATAAAAAAACCA			II	
0.167147	cg18832247	cg18832247	32721320	CCTCATTTAACCRAAAACTACT			II	
-0.178863	cg18832619	cg18832619	64706315	AATAAATACACAAAAAATAAT			II	
0.1179463	cg18842353	cg18842353	62643339	CTTCCATCTACCTCTAAACAAA			II	
-0.174288	cg18843258	cg18843258	23606377	CCAAAATCCRCCCCTACTCCTC			II	
0.2723467	cg18845598	cg18845598	64729449	ATCTAAAATATACTCTTTAAAT			II	
-0.2716	cg18845950	cg18845950	23655331	TCCAAAA	39745490	TCCGAAA	I	C
0.3927707	cg18847598	cg18847598	37742467	TAAATCAAAAACCCTCAACTA			II	
0.1978634	cg18861767	cg18861767	66694457	ATCCACTAAAAAAATAACCTA			II	
0.1067184	cg18862210	cg18862210	30730442	TTCCCACATACAAAATAAAATC			II	
0.1214975	cg18864124	cg18864124	54703465	AAAATAATACAACCAATTTACC			II	
0.1307789	cg18866210	cg18866210	71749347	AATACCRTCACTTATTTTATCT			II	
-0.142956	cg18867912	cg18867912	68708477	ATTCCCT	65731382	ATTCCCT	I	A
0.1040664	cg18872016	cg18872016	14605399	AAACRAAACAAACCTCTAACC			II	
-0.248603	cg18873509	cg18873509	40700434	AAATTAAAAATACCTAAAATTC			II	
0.136863	cg18873965	cg18873965	54692329	ATAAAAC	10612501	ATAAAAC	I	C
0.110063	cg18879828	cg18879828	18622333	TAACTCACAACAACCAAAAAT			II	
0.1458839	cg18886347	cg18886347	15615488	ACAAAAAAAAACCAAACCCCA			II	
0.1030164	cg18889780	cg18889780	41772482	CTAATAAATAACAAATACTAC			II	
-0.114631	cg18898632	cg18898632	73652446	TATCCCTTTTAATTATTATTTAC			II	
0.1047029	cg18904552	cg18904552	57804445	AACAAACAAAAAACTATCTAA			II	
-0.149414	cg18912209	cg18912209	12717429	CAAAAAC	33798456	CAAAAAC	I	A
0.1335448	cg18912855	cg18912855	73627398	TACACAA	52719449	TACGCGA	I	C
-0.152076	cg18913843	cg18913843	27768316	TTAATAAACATCCTACTATTCA			II	
0.1295932	cg18914751	cg18914751	22696464	CAATTATCCCTCTTATAACTAA			II	
-0.107751	cg18921980	cg18921980	26643444	AACTAAA	18749330	AACTAAA	I	A
0.1460382	cg18930910	cg18930910	67639417	AAAAATCAATACAACRCAAAA			II	
0.1242495	cg18932008	cg18932008	13771476	ATAAATTTTAACACAATACCTA			II	
0.1036861	cg18937395	cg18937395	36698324	CRCTATCCCRAAAAAAATTCRC			II	
-0.159431	cg18938907	cg18938907	28607449	ATCATAAACATAATACTAATTA			II	
-0.323611	cg18940047	cg18940047	29738408	CAAAAAA	18643318	CGAAAAA	I	C
-0.127844	cg18944640	cg18944640	17610306	AAAAAAA	24766383	AAAAAAA	I	A
-0.340795	cg18949721	cg18949721	10737407	TTTTTACCACATATTAAAATAC			II	
-0.167408	cg18964082	cg18964082	60705361	CCTCTAAAAATCCCTACATTAA			II	
0.1169479	cg18966152	cg18966152	60689504	ATAAAAATCTAAAATTTCAAAT			II	
0.1027602	cg18973863	cg18973863	53621510	ACCTAAATAAATATTCCAAAAC			II	
0.1653118	cg18974921	cg18974921	25750367	AAATTCTAAAAAATACACTATA			II	
0.110471	cg18982923	cg18982923	73759472	ACCATTACATTATTTAATTACR			II	
0.1118393	cg18984983	cg18984983	49726348	AATAAAAACTTCCATATAAAA			II	
-0.126469	cg18989057	cg18989057	47798444	AAAACCA	58669418	AAAACCC	I	T

-0.12126	cg18997918	cg18997918	73799498	AAAAAACTCTTTAACACTAAT			II	
0.1092718	cg19001290	cg19001290	33738369	CAAAAAATCTAAATTCAAACC			II	
-0.133535	cg19005485	cg19005485	19753347	CRCTAATTATATTATACAATAT			II	
0.1246907	cg19017553	cg19017553	12791403	TCATAAATAAACTCTCAAAATA			II	
0.1106395	cg19024575	cg19024575	66683313	ATAACTAAAACTAACAACCTT			II	
0.1685332	cg19026811	cg19026811	72688464	ACCAAAAAACTACCTCCAATA			II	
0.1042808	cg19028462	cg19028462	23709435	AATAAAAACAATTATATCAAA			II	
-0.100216	cg19039925	cg19039925	18772410	TTTAACACTCTATCCRTTCTCC			II	
0.1038544	cg19043679	cg19043679	28634462	AAACCTATAAATATCTAAAAC			II	
0.1249273	cg19043800	cg19043800	37690425	TAAATAAAACTAACTAACATTA			II	
0.1547851	cg19045970	cg19045970	38642499	CAAATTCTCACACCATCCAAA			II	
0.1037239	cg19046933	cg19046933	62747452	ATTAAAA	56669392	ATTAAAA	I	A
-0.101625	cg19047660	cg19047660	39632473	AACCAAT	19788466	AACCGAT	I	A
0.1142505	cg19050555	cg19050555	53626330	ATTTCTAACCTTTATTATTATA			II	
-0.245096	cg19054158	cg19054158	50692499	ACAAATC	38620328	ACGAATC	I	A
0.1178374	cg19061696	cg19061696	16654445	AAACATAAATATAAACCATTC			II	
0.1094997	cg19064302	cg19064302	16806431	TCAAACTACTATATTAATCATA			II	
0.1210673	cg19070088	cg19070088	51641443	TATAAAATATCTTCCCTACCCT			II	
0.1039726	cg19077494	cg19077494	59686444	TCAATTTCCCATATTCAAAAAT			II	
0.1857898	cg19081571	cg19081571	39616332	TCCTCAA	64729355	TCCTCAA	I	A
0.1251172	cg19084031	cg19084031	22697405	ACACAAAATTTCCTCTAAAAT			II	
-0.100636	cg19084628	cg19084628	15678303	AAACCAA	21616417	AAACCGA	I	A
-0.343041	cg19089141	cg19089141	13650302	TTCTCACTTAAACRTATACTTT			II	
0.1099368	cg19101566	cg19101566	41708499	ACACAAAACTCATAACAAACT			II	
0.1220481	cg19103175	cg19103175	69780387	TCATATT	55632318	TCATATT	I	C
-0.166153	cg19106932	cg19106932	70727507	AATATAA	13769303	GATATAA	I	A
0.1088406	cg19111378	cg19111378	14791415	TAAAAACAACACTAACCTAAA			II	
0.1660802	cg19112359	cg19112359	43715460	ATCTCAATTCAAATTTCAAAAC			II	
-0.115347	cg19112977	cg19112977	39655422	AATAAAC	58802319	AATAAAC	I	A
-0.113663	cg19113686	cg19113686	62787444	AAATATT	52670483	AAATATT	I	C
-0.107037	cg19116197	cg19116197	58774450	ACAAAAATATCTAAAATCAAA			II	
	cg19121987	cg19121987	17612323	AAAAACT	29675408	AAAAACT	I	A
0.126976	cg19137348	cg19137348	53769423	ATACTTTA	68711421	ATACTTTA	I	A
-0.177336	cg19140335	cg19140335	53809370	CAAACAC	22730398	CGAACAC	I	C
-0.105484	cg19140429	cg19140429	20787496	AAAATCA	63779377	GAAATCC	I	A
0.1001754	cg19145082	cg19145082	18765434	CATAAAAACAATATTATTAATC			II	
0.1651466	cg19145320	cg19145320	42631416	CTCTAACAAAAAAATAACCCT			II	
0.1145484	cg19152818	cg19152818	72749376	CTCTAAACACTAAAACAAAAA			II	
0.1092454	cg19153228	cg19153228	19720454	CTATAAATAAACATTTACCCRA			II	
-0.12081	cg19155932	cg19155932	43749462	ACAAAAA	39638415	ACGAAAA	I	C
-0.207357	cg19156046	cg19156046	24621302	AAAACACRCCATTTATATTCTC			II	
-0.127881	cg19162300	cg19162300	19617336	ACAACAC	51760401	GCAACGC	I	C
0.1637457	cg19163396	cg19163396	14795356	TTTTAAAACTAATTCTAATTAT			II	

0.1176264	cg1916393[illegible]	cg1916393[illegible]	32709467	ATTCTCC[illegible]	37750441	ATTCTCC[illegible]	I	A
0.1086566	cg1917850[illegible]	cg1917850[illegible]	70712376	ACTAAAATCAACRTAAACCTA[illegible]			II	
0.125966	cg1917991[illegible]	cg1917991[illegible]	74661377	ACTAAAATACACACAACTTCC[illegible]			II	
-0.138453	cg1918152[illegible]	cg1918152[illegible]	25651436	ACAAAA[illegible]	10665457	ACGAAA[illegible]	I	C
0.1081491	cg1918291[illegible]	cg1918291[illegible]	41702481	TTTTAACTTTTTAATTAACTT[illegible]			II	
-0.123399	cg1918316[illegible]	cg1918316[illegible]	34778379	TAATCAC	27645367	TAATCAC	I	A
-0.219592	cg1918339[illegible]	cg1918339[illegible]	69684305	TTCAACAAATTCTCCCCAAAA[illegible]			II	
0.1158299	cg1918820[illegible]	cg1918820[illegible]	64642350	CTTATAAAACCAATAAAACCC[illegible]			II	
-0.12934	cg1919127[illegible]	cg1919127[illegible]	48655375	CCACTCT[illegible]	24616329	CCGCTCT[illegible]	I	C
0.159168	cg1919262[illegible]	cg1919262[illegible]	32620442	ACACTAAACTAAAAAATTTTT[illegible]			II	
0.1089409	cg1919464[illegible]	cg1919464[illegible]	63739487	AAAAAAACAAACRACCTAAAT[illegible]			II	
-0.139801	cg1919640[illegible]	cg1919640[illegible]	28656499	AACAAAC	64758359	AACGAAC	I	A
0.105971	cg1919774[illegible]	cg1919774[illegible]	60653364	AAATAA[illegible]	12766507	AAATAA[illegible]	I	A
-0.179268	cg1919788[illegible]	cg1919788[illegible]	49737495	ATACCTC	42794429	ATACCTC	I	A
-0.125684	cg1920058[illegible]	cg1920058[illegible]	18783416	TCCTTAA[illegible]	21641317	TCCTTAA[illegible]	I	A
0.1156462	cg1920095[illegible]	cg1920095[illegible]	57715418	TTTCCTATTCATATCRAATCTA[illegible]			II	
0.1051227	cg1920114[illegible]	cg1920114[illegible]	12668447	RAATCACCCAAAAAAACCTAA			II	
0.1062704	cg1920357[illegible]	cg1920357[illegible]	41729330	CAAAACA	73749471	CGAAACA	I	C
0.1481211	cg1920492[illegible]	cg1920492[illegible]	52618403	AACCCTACAAACCTAAAAAAT			II	
0.1097174	cg1921808[illegible]	cg1921808[illegible]	46650473	CTAAAAA	58732404	CTAAAAA	I	A
0.1259654	cg1922239[illegible]	cg1922239[illegible]	53804325	AAATAATACAACCRTATAAAC[illegible]			II	
0.1111079	cg1922595[illegible]	cg1922595[illegible]	62655484	TACTTATAACAACCCCAAACC[illegible]			II	
0.1081972	cg1922921[illegible]	cg1922921[illegible]	41615359	ACAAAAACTATAAAACTACCA			II	
-0.102041	cg1923238[illegible]	cg1923238[illegible]	57630337	AACTCCA	48795476	AACTCCG	I	A
-0.107568	cg1923632[illegible]	cg1923632[illegible]	16618399	CCTAAATTTAAAAACCACTAA[illegible]			II	
-0.203554	cg1923667[illegible]	cg1923667[illegible]	39622372	ACAAAAAATCCAATAACTAAC			II	
-0.103769	cg1923904[illegible]	cg1923904[illegible]	36808446	AAATATACTACTATAACAAAC[illegible]			II	
0.3287034	cg1924261[illegible]	cg1924261[illegible]	72628304	CTAAAAAAATACCTACCCCCA[illegible]			II	
0.2062804	cg1924403[illegible]	cg1924403[illegible]	27615359	TTCCCAAAATAATCCTAAACA[illegible]			II	
	cg1924594[illegible]	cg1924594[illegible]	59606503	AAATCAATATCCTAAACTCCT[illegible]			II	
0.252597	cg1924981[illegible]	cg1924981[illegible]	17795473	AAACATT	24793332	AAACATT	I	T
-0.15488	cg1925582[illegible]	cg1925582[illegible]	69638391	TACCAAA	46603391	TACCAAA	I	C
-0.108112	cg1925980[illegible]	cg1925980[illegible]	70606369	AAATAAATTTTTATTTCTTTTT[illegible]			II	
-0.114913	cg1926360[illegible]	cg1926360[illegible]	43620445	CACRATAAAAATCCRCTCCAA[illegible]			II	
-0.119098	cg1927634[illegible]	cg1927634[illegible]	52623476	ACACAAC	66609339	ACGCAAC	I	A
-0.121016	cg1927673[illegible]	cg1927673[illegible]	74733353	ACTCACA	20648379	GCTCGCA	I	C
0.2211357	cg1928552[illegible]	cg1928552[illegible]	71768474	CACCCTCACCATAATCTAATA[illegible]			II	
0.2499465	cg1929169[illegible]	cg1929169[illegible]	13748308	TAAACTAAATTATATCCTCAA[illegible]			II	
-0.16347	cg1930019[illegible]	cg1930019[illegible]	10720447	AATAAAAATACTACCCAAAAT			II	
0.4303614	cg1930040[illegible]	cg1930040[illegible]	38724489	ACTATCAACAATTCTATATTTT			II	
0.1455556	cg1930054[illegible]	cg1930054[illegible]	18720498	TCCCTAACTAATTTCCTAACTT			II	
	cg1930189[illegible]	cg1930189[illegible]	29810355	CTATTATTCTTAAAACTATATA			II	
0.1292037	cg1930352[illegible]	cg1930352[illegible]	69647415	ACTCCAACTATAAATTCTAAA[illegible]			II	

0.1130758	cg1930649(	cg1930649(	16807337	AACCTCC	43714304	AACCTCC	I	A
0.1358398	cg1930898(	cg1930898(	11636377	CAAACCCCTAAATCAAAACTT.			II	
0.1619087	cg1931137:	cg1931137:	60640379	TAAAATTTTCAAAACTAATAC'			II	
0.1322046	cg1932328(	cg1932328(	63752427	CTTATATATCAACCAACCTTAT			II	
0.1170384	cg1932579	cg1932579	16661434	CAAAAACTTCRAAAAACAACT			II	
0.1340421	cg1933198(	cg1933198(	72621403	AAAAACCAAAACTAATAATAC			II	
0.254488	cg1934029(	cg1934029(	51696419	CCACAAAAATCAAACRCTAAA			II	
0.1047919	cg1934487(	cg1934487(	68703407	TTTCCAATTCCAACTAATAAAT			II	
0.1182027	cg1935305:	cg1935305:	23654413	CACAAACTTTAAAATTAAACA.			II	
0.1167045	cg1936021:	cg1936021:	48673335	ATAACTTTTCTTAAATCCCATA			II	
0.1099373	cg1936247(	cg1936247(	31614361	TCTACCTACAAACTATAACCC/			II	
-0.281949	cg1936277-	cg1936277-	36649386	AAAAAAC	13798457	AAAAAAC	I	A
-0.161336	cg1936717(	cg1936717(	35634328	AATCTCA	28751497	AATCTCA	I	A
0.1347609	cg1936785(	cg1936785(	54795417	CCTCTTTAAAACCTCAAACAT/			II	
0.1794634	cg1937250'	cg1937250'	60705397	AAACATTTTTTAAACTACTTA			II	
-0.12695	cg1937334'	cg1937334'	37624483	ACCTCTATTTATATTTTCRTTA.			II	
0.17375	cg1937541(	cg1937541(	29773383	ACTCTATAAACACTATCRCRA(			II	
0.1006929	cg1937760'	cg1937760'	74654306	AATCACCTAAATTATTTATAA/			II	
0.1129022	cg1938015(	cg1938015(	62608399	AAACAAAAATATTTACAATTT(			II	
-0.126339	cg1938336(	cg1938336(	62758370	AAAAACA	61672505	AAAAACA	I	A
-0.109234	cg1938937:	cg1938937:	60619466	CAATTAC	59765345	CAATTAC	I	A
-0.110812	cg1939255	cg1939255	35758373	AATAACTAAAAACATAAACRC			II	
-0.104561	cg1939819:	cg1939819:	65712353	AACACTA	42639385	AACACTA	I	A
0.1038683	cg1939845'	cg1939845'	71803437	AAAATCTCAAAAAAAAATTTA			II	
0.1443691	cg1940331:	cg1940331:	38619467	AAACAAAAACTATAAAAACCA			II	
0.1018537	cg1940588:	cg1940588:	28672500	TTCCTCCAATCCAAAATCTTTT			II	
-0.121481	cg1940857:	cg1940857:	22647322	ACAATAA	26687438	GCAATAA	I	A
0.1200179	cg1941173-	cg1941173-	60751362	AAAAATTTCACAATAAAAAAC			II	
-0.509402	cg1941574(	cg1941574(	68625351	ATATCTCAACAATAAAATAAA			II	
0.1678464	cg1941831(	cg1941831(	60709383	ACACRTTTATTACCATAACRTA			II	
0.1047959	cg1941852:	cg1941852:	40631412	AATTCTTTCATATAACATTCTC			II	
0.117084	cg1941929	cg1941929	39699342	TACCAAA	71736313	TACCGAA	I	A
-0.117625	cg1942657:	cg1942657:	74655465	TTCAATA	14802305	TTCGATA	I	T
0.1424689	cg1942860:	cg1942860:	46662383	TCCTTTACTAAATCCAAATCRT			II	
-0.133796	cg1944264'	cg1944264'	52704394	TAAACTA	74665486	TAAACTA	I	A
-0.152594	cg1944796:	cg1944796:	46763425	AATAATC	59680396	AATAATC	I	A
0.170133	cg1944796(	cg1944796(	24796508	AAATTTATTTTCTTAAAAAAA(			II	
0.1061295	cg1944994(	cg1944994(	66788487	CCAAACCCTCTTTTCCTATAAC			II	
0.1280113	cg1945081(	cg1945081(	30628447	AATTTCCCTAAAATACTATCA(			II	
0.1490498	cg1946319:	cg1946319:	69779335	AAACTAAAACTAACCTCAAAT			II	
0.1337909	cg1946944'	cg1946944'	19685405	ACACCCCTACCRAACRAATAT			II	
0.1040463	cg1947081:	cg1947081:	50687414	CRAAAATAAAATTAAACCTCR			II	
-0.125859	cg1947146(	cg1947146(	44792377	ATATTAAACTCCAAAAAAACC			II	

-0.120111	cg1947590	cg1947590	27728477	CCAAATT	32691488	CCGAATT	I	C
-0.10837	cg1947678	cg1947678	35650493	CTACTCC	46680357	CTACTCC	I	C
-0.108032	cg1948168	cg1948168	58680416	AAAAAAAAATATACAAACRA			II	
-0.311008	cg1948409	cg1948409	25692469	CTAACTATTCTCRTAAATCTTA			II	
-0.103584	cg1948430	cg1948430	26682315	AAAAAA	58741495	AAAAAA	I	C
-0.126198	cg1948786	cg1948786	61791398	CACTAAA	21674385	CGCTAAA	I	A
-0.224344	cg1949122	cg1949122	43711300	CAAATTCATATATCTAAATTTA			II	
0.1263287	cg1949561	cg1949561	31707422	AAAATAAAAAACCCCCAAAA			II	
-0.127717	cg1950324	cg1950324	24659420	ACCTTCT	66736311	GCCTTCT	I	A
0.1205926	cg1950625	cg1950625	15645445	CACTCCCTTTTACCTTACACAT			II	
-0.143509	cg1950939	cg1950939	37622407	ACTCACC	20617345	ACTCACC	I	C
-0.115666	cg1951472	cg1951472	72789432	AAACAATAAAATCAACRTATC			II	
0.1366673	cg1951692	cg1951692	32758486	CTCCCTAAATACTCTACTAAC			II	
0.2203825	cg1952023	cg1952023	65796424	TAAAAATAAAATCCCTAAACC			II	
0.1136655	cg1952161	cg1952161	37671427	AAAAAA	54784313	AAAAAA	I	A
-0.114874	cg1952549	cg1952549	22736306	ACTATAT	55730490	ACTATAT	I	T
-0.1009	cg1952970	cg1952970	53619307	AATTAAA	36739481	AATTAAA	I	A
0.100453	cg1953678	cg1953678	37640352	AATAATAAAAACAATCCCATA			II	
-0.221052	cg1953771	cg1953771	52694336	ACCCAAA	43710358	ACCCGAA	I	A
0.1129759	cg1953998	cg1953998	12758378	TAAAAAAATACATCAACTCCT			II	
-0.126609	cg1954593	cg1954593	59809335	TAATAAAATTTCTCAATATCC			II	
0.1035851	cg1955396	cg1955396	32663387	ATAAAAATATACTCTAAAATC			II	
-0.131566	cg1955677	cg1955677	10671472	CCATAAA	27751496	CCGTAAA	I	C
0.1159197	cg1956021	cg1956021	53731484	ACCTATTCRATAAATCAAATA			II	
-0.109607	cg1957077	cg1957077	32771462	ACCACAA	15679450	ACCGCGA	I	C
-0.178907	cg1957921	cg1957921	66676426	ACAACAA	62762448	ACGACGA	I	A
0.1076593	cg1958423	cg1958423	19601371	TAATACCACATTTCTCAAATA			II	
-0.173425	cg1958567	cg1958567	44725418	AAAAAACCCTAACTTTATTTC			II	
0.1931161	cg1959687	cg1959687	71733384	ACCTCTACACTACTAATAAAT			II	
0.1188229	cg1960541	cg1960541	42717455	CTTCATTATCCAAATTAAAAA			II	
0.143629	cg1960943	cg1960943	37692420	TAAAACCAATTTACTTATTCCC			II	
-0.100154	cg1961075	cg1961075	47697491	ACCCCCT	27663422	GCCCCCT	I	C
-0.106024	cg1962362	cg1962362	13677339	TAAAACC	54604468	TAAAACC	I	A
0.1945831	cg1962898	cg1962898	70743474	CRTATACTACAAATAACTATA			II	
-0.113658	cg1963260	cg1963260	59792327	CRTCAAAACCRCATTCCCAAA			II	
0.1369313	cg1963540	cg1963540	68605351	AAAAAAAATAACTCCACTAAA			II	
0.1424907	cg1963662	cg1963662	43728500	AAAATCCTAACRCTACTTAAA			II	
0.1187162	cg1964082	cg1964082	63719500	TACRATCTAAAAACCCTAAAC			II	
0.1084995	cg1964132	cg1964132	28633339	AAAAAA	34644367	AAAAAA	I	C
0.1327665	cg1965070	cg1965070	51600390	TCAAAAATCTACACTCTATAA			II	
-0.219161	cg1965111	cg1965111	65652432	AAAATTAAACACAACAACTAC			II	
0.1255717	cg1965175	cg1965175	50649322	TATACCCCAACCAATCRTAAA			II	
-0.208803	cg1965324	cg1965324	43737438	CAAAACCTAAACTCTTATTAA			II	

-0.34337	cg1965642:	cg1965642:	41794410	ATCTAAATAACCCTAACATAT/		II	
-0.187785	cg1967839:	cg1967839:	36727471	AAAATAA	70710392	AAAATAA I	A
0.1279182	cg1968341	cg1968341	53760422	ATCACTTTCATAACAAATCCA/		II	
0.1722554	cg1968349	cg1968349	50780489	AAACAACTATTAAACAATTCR.		II	
0.1464705	cg1968933(	cg1968933(	46677368	ACTAACTAACATCCCATTCTTA		II	
0.1324708	cg1968987(	cg1968987(	31612432	ACRAAAATAAAAACTACAAAT		II	
0.1016231	cg1969126(	cg1969126(	36743375	TAAACTACATACCAAAATAAA		II	
0.1052633	cg1969679	cg1969679	34711400	AATTTCTCTAAACTCCRTAATA		II	
0.109591	cg1970521(	cg1970521(	32679304	ATAACTCTATCCACAAAACCC		II	
0.1341747	cg1970765:	cg1970765:	57743487	ACTAAAATTTATATATAATAC(		II	
0.175631	cg1972208:	cg1972208:	15638325	CCTATAACTACTAAAATTAAA		II	
0.1461662	cg1972456	cg1972456	47774317	AATAAATCAAAAAACCAAAAT		II	
0.3819413	cg1972663(	cg1972663(	38736359	ATTACCTATAACTTCTCTCAAT		II	
0.1553613	cg1972671	cg1972671	53789502	ATACATAAATCTAACCTATTTT		II	
-0.100863	cg1972822:	cg1972822:	63645353	ATAAAAACTTCCRCCTCACTTT		II	
-0.124238	cg1972993(	cg1972993(	20728450	ATACTTAAATACACCCTATAA/		II	
-0.101771	cg1973026	cg1973026	72622502	ATCACCAACCAAATCTTTCCA/		II	
0.1580333	cg1973373(	cg1973373(	63663437	TCTTCTACTAAATACCTTACTC		II	
-0.146807	cg1973542	cg1973542	44720402	CTCCAACAAAAAATAAATCCC		II	
0.1153455	cg1974593(	cg1974593(	60730367	TAAATATTTAATTTACTTAAAT		II	
0.10978	cg1974989	cg1974989	24694465	AAAAATA	44652349	AAAAATA I	C
0.1079698	cg1975260:	cg1975260:	35665438	TCCATAATTTAAATACTCATAT		II	
-0.106479	cg1975483:	cg1975483:	17683336	CTCATCC.	43763498	CTCGTCC I	C
-0.224854	cg1975581:	cg1975581:	10773369	CCTACCT.	26610429	CCTACCT. I	T
0.1133095	cg1975883	cg1975883	64717398	ACCACATCTAATTAATACAAA.		II	
0.2009186	cg1975967	cg1975967	15716438	AACACAA	65697462	AACGCAA I	A
0.1194863	cg1975984	cg1975984	43696369	CCAACTTTTCTAATTCTCAACA		II	
0.1746565	cg1976698	cg1976698	20616320	CCTCCCTAAAAACCAATTTTTT		II	
0.1385635	cg1977067	cg1977067	19680502	AAATTAAATCCAAATAACCTC(		II	
0.3030837	cg1977468:	cg1977468:	50611302	CTTCATAAATTTCTCAACCACC		II	
-0.126982	cg1977576:	cg1977576:	22670481	AAATATA	73781407	AAATATA I	A
-0.26681	cg1977837:	cg1977837:	53777447	ACACATA	20734362	ACACATA I	T
0.1033849	cg1977864	cg1977864	50607419	CACTTAAATACTACTTCTTCCT		II	
-0.234619	cg1978701:	cg1978701:	50641413	CTATCAA	40741305	CTATCAA I	C
0.2633759	cg1979127	cg1979127	59625487	CAAAACATAAAATCCCCTCTT		II	
0.1274149	cg1979888	cg1979888	10622317	AAACAAC	42682438	AAACGAC I	A
0.116861	cg1981607:	cg1981607:	31784354	TTCAATAACCCACATATACAC		II	
0.114136	cg1982787:	cg1982787:	67742337	ATAAAAT	31648360	ATAAAAT I	A
-0.100019	cg1983037:	cg1983037:	21709404	AAAACAT	60749337	AAAACGT I	T
0.1341548	cg1983256:	cg1983256:	13755419	TCTACAAAATCTAAAACATCT		II	
0.1009627	cg1983619	cg1983619	26695382	TTTTAAATAATAAAATCCACA		II	
-0.167368	cg1984389	cg1984389	29795448	AACACCA	62706410	AACACCC I	T
0.1055218	cg1984428:	cg1984428:	49775320	ACTCCAACATAACCACCTTAT/		II	

-0.293505	cg19847577	cg19847577	70786455	AAAAAAAAAACCACTTATTCA			II	
0.1427361	cg19848732	cg19848732	22745427	TAAATATTCTTTACCTTCTAAC			II	
0.1215084	cg19848924	cg19848924	18686334	CTAACCCAAATACTAAACCCT			II	
0.1129137	cg19858214	cg19858214	59655427	CAAAAATTTCCTTTAACTAAA			II	
0.1026523	cg19864060	cg19864060	61679361	TTTTAATCAAAACCTATAAAA			II	
-0.151929	cg19873923	cg19873923	17781439	ATCAATAATTTTAACTTAACAT			II	
0.1448436	cg19876092	cg19876092	71761486	AAAAAAAACCAACAAAAAACT			II	
0.1212	cg19885037	cg19885037	35712386	AAAACAATAATTCAATAAAAA			II	
0.158824	cg19885625	cg19885625	28772411	ATAATACTCTCTAAATCTACTA			II	
0.118446	cg19889856	cg19889856	59725332	ACTTCAAATACCAATTCCTTCC			II	
0.124176	cg19894728	cg19894728	74609367	AACTCCATATCACCCCTATAA			II	
-0.15417	cg19897275	cg19897275	41718424	AAACAAA	42744398	AAACGAA	I	C
-0.155385	cg19898108	cg19898108	11674459	AAAATTTAATAAAAACTCTAA			II	
-0.135274	cg19899539	cg19899539	42755505	AACCACCATAACTAAAAACAA			II	
0.1837466	cg19903927	cg19903927	44719347	TACAACC	29742380	TACAACC	I	C
-0.12434	cg19904352	cg19904352	31611431	RAACACTAATAAATAAATATC			II	
0.1091401	cg19907257	cg19907257	21714440	ACAAAAC	45748377	ACAAAAC	I	C
-0.106937	cg19912739	cg19912739	17667498	ATTCTAAAAATTCCRAAATAC			II	
0.1629469	cg19921651	cg19921651	42773329	CCTTTAATATCAATAACCCAA			II	
-0.15143	cg19927508	cg19927508	15756427	AAAACAAAATATAATTACCRT			II	
0.1025258	cg19930004	cg19930004	17623415	ATATTACTATAAACTCAAAAC			II	
0.1086926	cg19937039	cg19937039	15742318	AAACTAAATCTTACTAAAACR			II	
0.1309586	cg19937061	cg19937061	31777487	ACTACAC	56706476	ACTACGC	I	C
0.1342466	cg19947463	cg19947463	53645471	ACATCACRACAATAACRACTT			II	
0.1154838	cg19954234	cg19954234	44758434	TCAAAACTTCCCACCCTCTTAA			II	
0.1736458	cg19958593	cg19958593	45670346	CATTCTCTATTATAATTAATAA			II	
0.1409395	cg19960616	cg19960616	50669464	ATAAAACAACTAAAAACATTT			II	
-0.239912	cg19961153	cg19961153	72760479	ACCAAAC	52661376	ACCGAAC	I	A
-0.204018	cg19967668	cg19967668	56696355	ACTACAA	29681401	ACTACAA	I	A
0.1967898	cg19972648	cg19972648	48651395	ATAAAAACTTAATAAAATAAT			II	
0.1005492	cg19974120	cg19974120	57652366	TATCRACAAATAAAAATAACT			II	
-0.120377	cg19975301	cg19975301	39678468	ACAACAT	29753506	ACAACAT	I	T
0.1965168	cg19976037	cg19976037	23677489	ACTTATTCRTCTAAAACACTA			II	
-0.137109	cg19976449	cg19976449	44698394	AAAATAAATCATTAATTCATA			II	
0.1829832	cg19979108	cg19979108	45742455	ATACCATACTTATATACTTAA			II	
0.1020062	cg19982031	cg19982031	70707453	CATTCCCAAAAACAAAAATCC			II	
0.1183572	cg19983948	cg19983948	63794307	ACTTTCATATCCCRTTAAAAAT			II	
0.1149268	cg19987900	cg19987900	72739359	CCCTATTACATTAATTCTTTAA			II	
-0.163297	cg19997062	cg19997062	59762379	AACCTAA	22730465	AACCTAA	I	A
-0.107278	cg20002177	cg20002177	25675354	TAACTTACATACAAAAAAAAA			II	
0.1261015	cg20002901	cg20002901	18674314	ACCCTAA	73703502	ACCCTAA	I	C
-0.134478	cg20006652	cg20006652	60601461	CAATAAA	55626498	CAATAAA	I	A
-0.176318	cg20007021	cg20007021	61733478	CAAAAAA	63671402	CGAAAAA	I	A

-0.260576	cg2002014:	cg2002014:	32673453	CCACCAA	68605367	CCGCCGA	I	C
-0.105944	cg2002072:	cg2002072:	51764502	ACACCTAAACRAAACTTTATA(			II	
-0.248572	cg2002203(	cg2002203(	44656440	CTATAAATAATTTCTATCCAA/			II	
-0.103264	cg2002636	cg2002636	55661387	AACCCACTAATTAAAAATTCA'			II	
0.1274414	cg2002794(	cg2002794(	63760366	AAAAAATCCTTCCTAAAATAA			II	
0.1775788	cg2003567{	cg2003567{	44729502	TCTAAACCAACAACAACCRCC'			II	
0.1257024	cg2004569(	cg2004569(	42794428	AAAATCTAAACAAAATAACAC			II	
0.1077635	cg2004804:	cg2004804:	35687429	AACATAACCRTAAAACCAAAA			II	
0.105581	cg2005048	cg2005048	63714318	TAAACATCTTCTAACACACTA/			II	
-0.105224	cg2005311(	cg2005311(	45641463	TAAAACC	31810473	TAAAACC	I	A
0.1126327	cg2006063:	cg2006063:	39792380	CTATACCTAACAAAAAATCTA			II	
-0.164771	cg2006415:	cg2006415:	49624409	TATAACATCTTTACAATCCTTC			II	
0.1030498	cg2006678:	cg2006678:	67725489	AAATATTTCTCATAAAAATAT/			II	
-0.144632	cg2007331:	cg2007331:	47630313	CATAAAC	17625416	CGTAAAC	I	C
0.1388848	cg2007612(	cg2007612(	49774361	CCAATAAAAATCAAAATAAAA			II	
0.1134461	cg2007651(	cg2007651(	41782446	TTCCTAAAAACCTAACCCCRC(			II	
0.1148425	cg2007864(	cg2007864(	66678384	CCTTCTAATACATACAACTATT			II	
0.1232282	cg2007880	cg2007880	66714495	AAAATCTAAATTTCCATTCCA/			II	
0.1217049	cg2008745	cg2008745	53631452	AAATAAACATACACACCTTTT			II	
-0.192838	cg2008751{	cg2008751{	10682449	TAATACA	49799315	TAATACG	I	C
-0.118501	cg2008824:	cg2008824:	69751485	CTTTAACCRTTAAAACRTTTAC			II	
0.1321267	cg2008979{	cg2008979{	37764504	ATTAAACRATACTATAAATCT(			II	
-0.169645	cg2009006(	cg2009006(	41707350	CCTAAAAAATATAAACTCTCT			II	
-0.110559	cg2009028:	cg2009028:	58795481	CTAATAA	67686425	CTAATAA	I	T
-0.147656	cg2009434:	cg2009434:	27791426	CCTACAAAAAAACCCCCTCAA'			II	
0.4940095	cg2010607	cg2010607	49631492	ATAATAACCAATTCAAAAAAC			II	
0.1128095	cg2011452	cg2011452	72763473	ATAAATAACTCTCRTTTCCTAT			II	
-0.101819	cg2011615{	cg2011615{	33765311	TAAAATT	59601326	TAAAATT	I	A
-0.102207	cg2011979{	cg2011979{	55721375	TTAACTATCACCATAATCAAA(			II	
0.1400142	cg2011989	cg2011989	50617369	TTTTAAACAATATCATTTCTAA			II	
-0.120975	cg2013286:	cg2013286:	38701360	TCCATAT	22791459	TCCATAT	I	A
-0.109139	cg2013774(	cg2013774(	63661307	TACAAAT	62647384	TACAAAT	I	T
0.1143575	cg2014139{	cg2014139{	63670429	ATCAACT	25809507	ATCGACT	I	A
-0.155691	cg2014276:	cg2014276:	73630387	TTTCTCTTACCRTACRACTACA			II	
0.1497231	cg2014426	cg2014426	63723458	AAATAATAAAATCCAACCCAA			II	
-0.136691	cg2016460	cg2016460	24804471	AAATTCA	68657454	AAATTCG	I	C
0.1037753	cg2018405(	cg2018405(	55689349	TAAATCACAAATTCATAATCA			II	
0.1138955	cg2018801	cg2018801	10758349	CCTTATTAACTAATTAAACTAT			II	
-0.26412	cg2018821:	cg2018821:	12636503	TCTTTCCTTCTTTTCCAAATCA			II	
-0.175316	cg2018849(	cg2018849(	45773423	CTATAATACCTAAAAATTCCC			II	
-0.118151	cg2018924	cg2018924	41702417	TAACCAC	58683406	TAACCGC	I	C
-0.100134	cg2019092:	cg2019092:	57708313	ATTCCTCCTAATAAATATTTAT			II	
0.2539717	cg2020518{	cg2020518{	29676313	TAATAACCRAAATAAACCRAA			II	

0.1100344	cg2020732[illegible]	cg2020732[illegible]	15787404	TAAAAAAACAACACACTTACT	II		
-0.100088	cg2020860[illegible]	cg2020860[illegible]	74785399	CCTAAAAAAAACRCTCTATTT[illegible]	II		
0.1229516	cg2021291[illegible]	cg2021291[illegible]	45745470	ACAAATCAAATAAAACATAAC	II		
0.1401559	cg2021487[illegible]	cg2021487[illegible]	40789333	CCTACAAATTAAAATTCTACC[illegible]	II		
	cg2021857[illegible]	cg2021857[illegible]	73790507	AATAAAAAACRACACCRAAA[illegible]	II		
-0.109066	cg2022320[illegible]	cg2022320[illegible]	28672338	CAAATTAATATAATAAATACA	II		
0.107057	cg2022368[illegible]	cg2022368[illegible]	42650326	CTACTAC 16610447 CTACTAC	I		A
0.128695	cg2023129[illegible]	cg2023129[illegible]	36657311	TCCCAATATCAAAACTAAAAA	II		
-0.278328	cg2024288[illegible]	cg2024288[illegible]	15736503	TTCACAA 71710493 TTCACGA	I		C
-0.154994	cg2024611[illegible]	cg2024611[illegible]	54789496	AAAAACC 71748377 AAAAACC	I		A
0.2602279	cg2024932[illegible]	cg2024932[illegible]	11758417	CACCCAACACAACTAAAAACT	II		
0.1190189	cg2025034[illegible]	cg2025034[illegible]	67750401	CCCRAATCAACTACTTAAAAA	II		
-0.437658	cg2025537[illegible]	cg2025537[illegible]	26731480	TTCTCAT 30771301 TTCTCAT	I		A
-0.108152	cg2026233[illegible]	cg2026233[illegible]	47808302	ACTAAAATACCAACTTCAAAA	II		
-0.131343	cg2026406[illegible]	cg2026406[illegible]	48676422	CATAATA 70637481 CGTAATA	I		T
-0.101805	cg2026496[illegible]	cg2026496[illegible]	49718390	ACTAAAAATCATTTACTTTAA[illegible]	II		
0.1823941	cg2026732[illegible]	cg2026732[illegible]	41696395	TTCCRTAAATATCCCTAAATA[illegible]	II		
-0.120818	cg2026904[illegible]	cg2026904[illegible]	33757366	AAAACCA 20798360 AAAACCC	I		A
0.1266826	cg2027264[illegible]	cg2027264[illegible]	41747302	AAAAAACTAAAATTTCTACTA	II		
0.1037337	cg2027326[illegible]	cg2027326[illegible]	67803443	ATCAAAATCTACAAACAAATA	II		
-0.139119	cg2027678[illegible]	cg2027678[illegible]	49660309	CRAACCCTCTAATAATTCCCTT	II		
-0.141348	cg2028160[illegible]	cg2028160[illegible]	56683470	CACTTCT 74788335 CGCTTCT	I		C
-0.157881	cg2028255[illegible]	cg2028255[illegible]	43628367	AACTAAA 30753492 AACTAAA	I		C
0.1208768	cg2028401[illegible]	cg2028401[illegible]	44796422	TACTTCTAATAAAAAACCAAC	II		
0.1239273	cg2028423[illegible]	cg2028423[illegible]	49746434	AATCACTATTTCAACTACACA[illegible]	II		
-0.102751	cg2028554[illegible]	cg2028554[illegible]	31603433	CTACTCCCAAATATAACCAAA	II		
0.1029623	cg2028793[illegible]	cg2028793[illegible]	60624477	TTTAACTCCCACTTCTAAATAA	II		
0.1286322	cg2028861[illegible]	cg2028861[illegible]	50689426	AAACAAAACCTAATAACRAAC	II		
0.1251827	cg2028985[illegible]	cg2028985[illegible]	48606406	AAATAATATAAACAAACACAA	II		
-0.199656	cg2029967[illegible]	cg2029967[illegible]	72697402	CTTTCTTTCCATTAAAACAACA	II		
-0.256559	cg2030051[illegible]	cg2030051[illegible]	53768450	ACRCATTAAAACAAAACRAAC	II		
0.1220938	cg2030078[illegible]	cg2030078[illegible]	58697445	CTAAAACCCRAAACCACACAC	II		
-0.129821	cg2031060[illegible]	cg2031060[illegible]	53748416	ACTAAAA 22764447 ACTAAAA	I		C
0.1956587	cg2031100[illegible]	cg2031100[illegible]	67768324	CCCAACTTCCAATTTCTAACTC	II		
-0.121676	cg2031167[illegible]	cg2031167[illegible]	48606478	AAATATA 48723444 AAATATA	I		T
0.1444915	cg2031201[illegible]	cg2031201[illegible]	17683345	TATTTTATTTCCTCATACCTTA[illegible]	II		
0.1099146	cg2031241[illegible]	cg2031241[illegible]	60802385	AAAACTAAATTCAAAATCAAA	II		
-0.133203	cg2032108[illegible]	cg2032108[illegible]	48745481	ACACAAA 53754400 ACGCAAA	I		A
0.1205382	cg2032557[illegible]	cg2032557[illegible]	39771403	AAAAACAAAAACATACACRA[illegible]	II		
0.1315153	cg2032784[illegible]	cg2032784[illegible]	24655434	ACCATAC 41653386 ACCATAC	I		C
0.1041821	cg2033115[illegible]	cg2033115[illegible]	58644321	AATAACAAAACCTCRAACCRT[illegible]	II		
0.1078694	cg2033250[illegible]	cg2033250[illegible]	45620310	TAATCCAAACATCCAAAATCC	II		
0.1165327	cg2033411[illegible]	cg2033411[illegible]	33801340	AAAATTACTCTAAAAAAATAA	II		

0.2652687	cg20336016	cg20336016	58689457	AAATAAAAATCTTACRAAAAA			II	
-0.110233	cg20337996	cg20337996	28626323	CCAAACCCAAACAAAATTCCT			II	
0.112949	cg20340720	cg20340720	16728426	TCCTAACTCTACTCTAATTTTC			II	
-0.100745	cg20340866	cg20340866	11678484	CACTTAA	65669459	CACTTAA	I	A
0.1220489	cg20344388	cg20344388	44636396	TTTAAACCTCTACAAAAAAC			II	
-0.120852	cg20347665	cg20347665	68779491	CAACTAA	14756391	CAACTAA	I	T
-0.126595	cg20364183	cg20364183	49676321	TACCATTCRTATTACAACCAAT			II	
-0.12558	cg20370184	cg20370184	70714459	CAAACAAACCTAAATCAAAAC			II	
0.1243628	cg20372591	cg20372591	61778393	TATCACCTCTACCTTTATATTT			II	
-0.124964	cg20372745	cg20372745	67706308	ATCCCAA	12660310	GTCCCAA	I	T
-0.120795	cg20377305	cg20377305	39711501	TATCTTT	16773420	TATCTTT	I	C
-0.29226	cg20381372	cg20381372	15777504	AAACAACTAATCTATATCTTC			II	
0.1719257	cg20384620	cg20384620	19794463	AACAAA	64625488	AACAAA	I	A
0.1142273	cg20387775	cg20387775	13726478	TACCTACTTTTTAAACTTTATC			II	
0.1076878	cg20388732	cg20388732	67755386	ATACTATAAATAAACAACTAA			II	
-0.175965	cg20390711	cg20390711	56715415	ACAAATA	53601482	ACAAATA	I	A
-0.14854	cg20399616	cg20399616	63653479	CCATAAA	28680319	CCATAAA	I	A
0.1127526	cg20401549	cg20401549	66684405	CAAACRCCAATTTCACTTTCTA			II	
-0.40049	cg20402783	cg20402783	66662387	TCTCCTA	33633366	TCTCCTA	I	A
-0.144107	cg20403938	cg20403938	34752304	AAACTAA	28762345	AAACTAA	I	C
0.206005	cg20409752	cg20409752	48623369	TCTATTAAATAAAACCACCCA			II	
0.1033579	cg20411555	cg20411555	25618398	ATTAAATAATAATCAAACCTA			II	
0.1081591	cg20418529	cg20418529	64762372	TAAACCT	39730349	TAAACCT	I	T
0.2038854	cg20426275	cg20426275	36706349	TATAAAAAACCCAAAACCAAA			II	
0.1177011	cg20433858	cg20433858	17795371	ACTCAAAAATAAAACTAACTC			II	
0.1197544	cg20434422	cg20434422	71609502	ATCTAAAATCTACAAAACTAT			II	
-0.106593	cg20441912	cg20441912	47727341	AAAACCA	67792481	AAAACCC	I	A
-0.10607	cg20450471	cg20450471	12656497	TATTATC	38620431	TATTATC	I	A
0.2137923	cg20453861	cg20453861	63637430	ACTTAAACACATAAAATAATT			II	
0.1983959	cg20457051	cg20457051	74687328	TAACCTTTTAAAATTTTCTTTA			II	
0.180906	cg20459037	cg20459037	27804461	CCATATACCTCCTAAAACTAA			II	
0.1000714	cg20464155	cg20464155	27634393	TAAATTTCTACCAAATTAAAA			II	
0.1094919	cg20464884	cg20464884	62676315	ACRAAATACTAACTTACTTTTT			II	
0.1035421	cg20469003	cg20469003	59735428	ATCATAAATCAACATATAAAC			II	
0.1801365	cg20469507	cg20469507	27692475	TAACTTCTAAATTAAATTTATC			II	
-0.140819	cg20477705	cg20477705	37713507	CACCCRCTTATACRTCTTATAA			II	
-0.295331	cg20479209	cg20479209	17633366	TTCAAATAAAAAAAATCAACA			II	
0.1886533	cg20488673	cg20488673	16785488	CTAAAAAACCCAATAACTAAT			II	
0.1047944	cg20494779	cg20494779	42605490	AAACCCTATATTCTACCAATA			II	
0.1035699	cg20503907	cg20503907	59775378	AAAAATCRACAATACCCCAAT			II	
-0.156506	cg20503915	cg20503915	41745467	AAAACTA	39679510	AAAACTA	I	A
0.1189358	cg20506807	cg20506807	21800380	TACAAAA	46796390	TACAAAA	I	A
0.1745426	cg20507276	cg20507276	25806371	AAAAACACCAATATATAAAAC			II	

0.1546832	cg2051003	cg2051003	11709493	TAAACCCTAAATTCTAACCRC			II	
0.1233997	cg2051341	cg2051341	68734497	ATAATTAAAAAAAACCTATCT			II	
0.127323	cg2052239	cg2052239	20643447	TCTATACTTCCCTCATTATACT			II	
0.1355735	cg2052878	cg2052878	28708301	TTCTCCTCTCRAAACTCAAAA			II	
-0.225969	cg2053084	cg2053084	47798492	TAAAACTATCAACCAATCCCC			II	
0.1683344	cg2053398	cg2053398	57741364	RAACCAACTCTACRTCTATCTT			II	
-0.190722	cg2053697	cg2053697	48795471	TCCTTTTTCTATTTACTAATAA			II	
0.19122	cg2054023	cg2054023	13606314	TCACTCACTAAAACTCTAATTT			II	
-0.369669	cg2054929	cg2054929	73796474	TCAAAAACAAAATATAATCCT			II	
0.2758084	cg2055001	cg2055001	20688329	AATCTAA	74727490	AATCTAA	I	A
0.116143	cg2055300	cg2055300	16702322	RCAAAAACCTATAAACRAACR			II	
-0.105632	cg2055437	cg2055437	70636447	CRAAATACATATTAATAATAC			II	
-0.172014	cg2055921	cg2055921	33655451	TTCTAAACCRAACAAATCRAA			II	
0.1369014	cg2056126	cg2056126	38732415	TAAAAACTCTTCTTAATTTATA			II	
-0.156943	cg2056193	cg2056193	32730395	TAAATTCCTCCCTATACCCCTC			II	
-0.166597	cg2056327	cg2056327	49686368	AACAAAT	33708300	AACAAAT	I	C
0.1134551	cg2056810	cg2056810	36666492	CATCAATTAACCCAATTCTAA			II	
0.2748303	cg2056842	cg2056842	12740464	TATCACTTATTCACTATCTTCT			II	
0.1140765	cg2056980	cg2056980	59676454	CTCATTAACTTCTTAAATAAC			II	
0.1507272	cg2057659	cg2057659	58762444	CCATACAAAAAACCTATAATC			II	
0.1032069	cg2058085	cg2058085	13714328	CCTATTTCCTACCTAAAAAAC			II	
-0.120312	cg2058187	cg2058187	71619415	TCTAAAA	65808494	TCTAAAA	I	C
0.1069377	cg2058484	cg2058484	54797458	CACCTACATACAACRATAATT			II	
-0.13306	cg2058499	cg2058499	74778381	AAAAAAC	48629465	AAAAAAC	I	A
-0.131308	cg2059201	cg2059201	11637351	AAAACAT	15737394	AAAACAT	I	C
0.120021	cg2059212	cg2059212	39750490	ATCTAAAAAAAATAAACTACC			II	
-0.3007	cg2059239	cg2059239	69708495	AAATCTAAAAATAAATATTTT			II	
0.158851	cg2059283	cg2059283	43730356	AACCCCTTTATAACRTCACAA			II	
0.1069165	cg2059677	cg2059677	36751506	AACTAACTAAATAAATATACT			II	
-0.110435	cg2061045	cg2061045	62753398	CCCTACC	39645307	CCCTACC	I	A
-0.127805	cg2061732	cg2061732	18710494	TTCCTCTCTCAAAACAACAAA			II	
-0.144514	cg2063381	cg2063381	73667326	CRCTTTATAAAAACTCTAACC			II	
-0.134706	cg2063980	cg2063980	35692332	TCCATTAATATTACCATCTTAT			II	
0.2325593	cg2064037	cg2064037	18642456	ATCCTTT	31658443	ATCCTTT	I	C
0.1369686	cg2064167	cg2064167	10738373	AATTTATCCTAAACCAAATAT			II	
0.1412884	cg2064344	cg2064344	42606486	TATTCRTTATTCCAATCAATCT			II	
0.1881453	cg2065164	cg2065164	47692461	CAAACTATTATTAAACCTATTT			II	
-0.11995	cg2065312	cg2065312	32640330	ATTTAAAATAATAATATATAT			II	
0.1233282	cg2065770	cg2065770	12673404	ATAACAAAAATATCAACCACC			II	
-0.135671	cg2066291	cg2066291	39793457	ACAAAA	27705428	ACGAAA	I	T
0.1489976	cg2066304	cg2066304	73745465	TAATAATTCTATATAACRCTA			II	
0.1027895	cg2066444	cg2066444	44618449	TCCAAAA	49704310	TCCAAAA	I	C
0.1161782	cg2067625	cg2067625	24744337	TATCTCACTAACCAATAAAAA			II	

0.1138012	cg2067883:	cg2067883:	50695330	AACAATCCAATCAACACAACA			II	
-0.179733	cg2067898	cg2067898	63806509	TAACACCAAAATATTTACACA			II	
0.1056624	cg2067940:	cg2067940:	43772339	RAAAAAACAACTAAAATCTAA			II	
0.1176112	cg2068344:	cg2068344:	62708473	ACCTAAA	54684413	ACCTAAA	I	A
0.1896375	cg2068620	cg2068620	59637311	ATAAAACCTCRACCATAACTA			II	
0.1019867	cg2068627	cg2068627	71638430	AAAACAC	63712320	GAAACAC	I	C
0.1387974	cg2069561	cg2069561	70661373	AAACCATATAACTAAATAAAA			II	
0.1012762	cg2069927	cg2069927	71654483	TTCTACTAACCATATAATACA			II	
0.1020523	cg2070220:	cg2070220:	28808505	ACACTAATAACTCTCCRATAT			II	
-0.122303	cg2070496:	cg2070496:	21621390	CACCTAAAACTATCAATAACT			II	
0.1550421	cg2070589:	cg2070589:	15653321	TTCTACCTATTCRACATCTTTA			II	
	cg2071381(	cg2071381(	69709355	CCAAATT	13644459	CCGAATT	I	T
-0.113845	cg2072059	cg2072059	12740331	AAAAACC	28690392	AAAAACC	I	C
0.130006	cg2072403:	cg2072403:	59755484	TTATACATTCAATAAATCATA			II	
-0.258243	cg2073193	cg2073193	49698416	ACCCAAA	10682396	ACCCGAA	I	C
-0.11067	cg2073506	cg2073506	28732348	ACAACTCTAAATCATCCRAAT			II	
0.1954733	cg2074104(	cg2074104(	40687318	TTACCCAAAATAAACTACAAA			II	
-0.141503	cg2074152	cg2074152	34749417	AACRAACCCTATTCCRAAAAA			II	
0.1076451	cg2074648:	cg2074648:	73669372	CCCCTAAAATTAACAATCTCC			II	
0.1127151	cg2075139	cg2075139	11652370	ATAACTAAAACTCRACTCTAT			II	
0.3142838	cg2075602(	cg2075602(	53647446	AATCCRAAAAACTAAAAATAT			II	
0.109229	cg2076478(	cg2076478(	54722475	TCAATCACTATCCCTCRTACTT			II	
0.1120414	cg2076734	cg2076734	44648351	AAAACTAAATTCTCAATCATA			II	
0.1687727	cg2076985(	cg2076985(	44637311	CAATCATAAATTCCCACAAAA			II	
-0.101842	cg2077314	cg2077314	52728317	AAAAAA	13657389	AAAAAA	I	C
0.1125737	cg2077860(	cg2077860(	25686351	TCAAAATATCCTAACTTACTC			II	
0.2096466	cg2077891:	cg2077891:	60629456	AAAAAAAAATATACAAAACCC			II	
0.1280639	cg2078397(	cg2078397(	43801338	TCAATATATATCTAATTTTAAA			II	
0.1078657	cg2078948:	cg2078948:	74659383	AAACATACCAAAAACTACTCR			II	
-0.123295	cg2079079	cg2079079	26770430	RAAATATAAAAACCRACRCCA			II	
-0.281732	cg2079129	cg2079129	71714440	ACCACCACCATATCCAACTAA			II	
-0.117094	cg2079537:	cg2079537:	57723448	AACTACC	63695473	AACTACC	I	C
-0.302446	cg2079541	cg2079541	63659363	RCACACCACCACACCCAATTA			II	
0.2106094	cg2079806(	cg2079806(	69666477	AAAAAATAAACCCATATACTA			II	
-0.108476	cg2081175:	cg2081175:	53690493	AAAAAACAACATCTACCRAAA			II	
0.1579022	cg2081198	cg2081198	31628448	CTCAAAC	12625453	CTCGAAC	I	T
-0.227368	cg2081417	cg2081417	25742488	ACCAAAA	51751377	ACCGAAA	I	T
0.117727	cg2082104:	cg2082104:	48808387	ACAACCTCAACAATTACTACT			II	
0.1154289	cg2082171:	cg2082171:	57656364	AAAATCCTCATATAACACCTC			II	
0.1546395	cg2082352	cg2082352	35605502	ATATCCCCTAAACACACAAAA			II	
0.1485388	cg2082674(	cg2082674(	47760413	CAACCATCCAAAAATAACCTA			II	
-0.233938	cg2082828	cg2082828	31612369	AACCATA	74658363	AACCGTA	I	T
0.1014387	cg2083570	cg2083570	39713425	ACTCTAAAACRTCTAACCTAA			II	

-0.138759	cg2083920(	cg2083920(	72782462	ATCAAAA	14649377	ATCGAAA	I	A
-0.13485	cg2084486l	cg2084486l	31733489	AAAATATAACAATTCAACAAA			II	
0.1325819	cg2085098l	cg2085098l	46766420	CACAAACAAATTATATAACAA			II	
-0.147304	cg2085984l	cg2085984l	69633331	ATATTCRCTACAACCATACCCA			II	
0.1072684	cg2086421	cg2086421	37637493	ACCAAAC	29685483	ACCGAAC	I	C
-0.12122	cg2087676(	cg2087676(	39770411	AATAAACTCAAAATAAACCRC			II	
-0.102029	cg2088488	cg2088488	37803322	ATTAAAA	60728315	ATTAAAA	I	A
-0.140573	cg2088736(	cg2088736(	25669327	TAAAAAA	47748497	TAAAAAA	I	A
0.1143295	cg2089393(	cg2089393(	30740505	AAAACATTACTATAATACTAA			II	
0.1464788	cg2089978l	cg2089978l	48636352	RTTTAAAACCTTAATTACCCA			II	
0.2443235	cg2090433(	cg2090433(	62603309	CRACTTAACCAAATCATAATA			II	
-0.15073	cg2090713(	cg2090713(	13706331	CCAAAAA	19607309	CCGAAAA	I	T
0.1077694	cg2090740	cg2090740	33773300	AAAAACTACAAAACTACTCTT			II	
0.1149626	cg2091036l	cg2091036l	23728464	TCCTAATATTTACATATACATA			II	
0.1471278	cg2091642	cg2091642	53682313	TAACCTAACCACAAAAACAAC			II	
0.1035536	cg2091707	cg2091707	17716311	CAAAACTATCATTAAACATTC(			II	
0.2551301	cg2091749l	cg2091749l	70618476	TAAAAATCTTATCACTAACRA(			II	
0.1058682	cg2092442	cg2092442	30640339	CRATCCAACCACRAAACTCCT			II	
0.1074037	cg2092739	cg2092739	13804374	AAATTACTACAAACCCAAACA			II	
0.1172974	cg2092765(	cg2092765(	13616379	ATTTTTACTTTTTACCAAACCC			II	
0.1677403	cg2094118	cg2094118	58669414	TCTACAACCTCCTAAACAAAC			II	
-0.129992	cg2094508	cg2094508	40674373	CCTAACC	29626407	CCTAACC	I	A
0.1030867	cg2094827l	cg2094827l	36623459	TACTTACACAAATAAAAATAA			II	
-0.120719	cg2094836	cg2094836	49686431	TCCAATT	19704373	TCCGATT	I	C
-0.112705	cg2095164	cg2095164	51768470	TTCTAAAAAAACCRATATCTA			II	
0.2759559	cg2096032	cg2096032	58758453	CCATAAAAAAACTTCTTTAAA			II	
0.1249079	cg2096915l	cg2096915l	30775370	ACTACTCTATCTAAAACRATCA			II	
0.1118208	cg2097819	cg2097819	49784457	CTACCTTACCTTCTACCTCCCT			II	
0.1200039	cg2097892	cg2097892	68705431	CAAACAT	13754460	CAAACGT	I	T
0.1167104	cg2097915	cg2097915	38808325	CTACTAC	71720494	CTACTAC	I	C
0.1520174	cg2097938	cg2097938	40604470	AATAAAATTCCCATTCTTATAA			II	
0.1900771	cg2098116	cg2098116	67601502	TCACTAATTAATACCTCTACAA			II	
-0.116283	cg2098145l	cg2098145l	11725350	TCTACTA	40708411	TCTACTA	I	C
-0.114286	cg2098683	cg2098683	72743474	AAAAATCRCTACAAACATAAA			II	
0.1062389	cg2098743l	cg2098743l	63774318	TTTAAACTATATCTTTAAAACA			II	
0.1886873	cg2099115	cg2099115	38770375	ATCCCAACCTTCATTTTCAAAC			II	
0.1075608	cg2099508	cg2099508	14801411	AAAACAT	35782396	AAAACGT	I	A
-0.122501	cg2099635l	cg2099635l	13809302	AACACAT	61637328	AACACGT	I	C
-0.141916	cg2100551(	cg2100551(	35610381	CCCAAAA	44640449	CCCGAAA	I	C
0.1328402	cg2100568	cg2100568	27653315	AATTTCCCCACTTTATCTCTTC			II	
0.1808829	cg2101205	cg2101205	14677460	CAAACTAATAATTTAAACCRC			II	
0.1060485	cg2101788	cg2101788	71619357	CACAACT	47602308	CGCGACT	I	A
0.3224225	cg2102831	cg2102831	71711491	TTTTTAAAACCACTTACAATCT			II	

-0.397032	cg2103256	cg2103256	19687445	TTTCTAT	50644406	TTTCTAT	I	C
0.1056796	cg2103440	cg2103440	26684317	AATACTATCCCACAAACACAC			II	
0.1057419	cg2104009	cg2104009	70668361	ACAATAATATCTACAATAACR			II	
-0.101658	cg2104010	cg2104010	59656331	AACCACA	67614499	AACCGCC	I	A
0.1039101	cg2104557	cg2104557	10792451	AATTACAAAAATATTTCACAA			II	
0.1357679	cg2104870	cg2104870	59735421	TTAAACATTTCTATCATTCTCA			II	
0.1710447	cg2104939	cg2104939	71773502	AATATCACATCTCRATAACTC			II	
0.1393884	cg2105276	cg2105276	51790432	CAAAACC	70742444	CAAAACC	I	A
-0.115255	cg2105491	cg2105491	63721486	CCCTACACACATATATCRATC			II	
0.2289566	cg2105555	cg2105555	12765328	TAATCAATAAAAAATATTTCA			II	
-0.122895	cg2106278	cg2106278	38635332	ACATCAA	34689472	ACGTCAA	I	C
-0.25493	cg2107008	cg2107008	64787328	ATATTTAAAATAATACCTTCTT			II	
-0.306228	cg2107807	cg2107807	72651462	CCTAAAA	37638335	CCTAAAA	I	C
0.1020657	cg2108227	cg2108227	38628338	CAACCAT	64679354	CAACCGT	I	C
0.1243418	cg2109317	cg2109317	12741508	TTCTCATTAAAAAAAATTCCTT			II	
-0.112193	cg2109380	cg2109380	51790502	AAATAAT	51609351	AAATAAT	I	T
0.1313206	cg2109778	cg2109778	58700335	CTAAAATACTAAACCTAATAA			II	
-0.108679	cg2110587	cg2110587	34635360	TAAAAAA	37612334	TAAAAAA	I	A
-0.160566	cg2110648	cg2110648	50801389	TAAACTA	45649507	TAAACTA	I	C
0.2370687	cg2111472	cg2111472	10785309	ACTCACA	39705421	GCTCGCG	I	C
-0.105151	cg2111626	cg2111626	59722425	AACAAAT	30706349	AACAAAT	I	A
0.101202	cg2112160	cg2112160	23692348	AATATCTCTCTTCCTACTTAAA			II	
-0.139855	cg2112662	cg2112662	56731381	TATAAAATACTTTTTCTCAAAC			II	
-0.116461	cg2113086	cg2113086	74646366	AACCAAA	24683387	AACCAAA	I	C
-0.368576	cg2113979	cg2113979	27621380	AAACTATCCCTTTCCTCCTTAA			II	
-0.154321	cg2114406	cg2114406	26735430	TTCTCATTCATAAACCCCACAC			II	
0.1001192	cg2114862	cg2114862	39620319	AATATTCCTTAAAATCACRTA			II	
0.1636967	cg2114935	cg2114935	27800342	TATTAACTCAAATCCTACAAC			II	
-0.106169	cg2115365	cg2115365	48737317	AACTACT	60749334	AACTACT	I	A
-0.101365	cg2115638	cg2115638	62695318	CCCATCC	38725337	CCCATCC	I	T
-0.158663	cg2115816	cg2115816	37710386	AAATTAA	13616441	AAATTAA	I	A
0.1226372	cg2115850	cg2115850	51683349	AACAACA	24639308	AACGACC	I	A
0.1151745	cg2116014	cg2116014	39644459	AACACTTATCTTATTATACTAC			II	
-0.188139	cg2116430	cg2116430	74616396	ACTAATA	19793383	ACTAATA	I	A
-0.203834	cg2116740	cg2116740	36707329	ACACCAA	28725425	ACACCGA	I	C
-0.12051	cg2116905	cg2116905	66643428	AAAAATTTATCAAATACTAAA			II	
0.1231564	cg2117568	cg2117568	33757409	AACCACRAACCAACTAAACRT			II	
0.1174456	cg2118609	cg2118609	22682417	ATTCCCAACAAAATACAATAC			II	
0.1020989	cg2118706	cg2118706	33657429	AATAAAAATAAAACTCCCAAA			II	
0.1044651	cg2118719	cg2118719	16608507	AAAAAACAAAACCAAACACR			II	
-0.105599	cg2118777	cg2118777	22600395	AAAAACA	11635390	AAAAACA	I	A
0.107557	cg2118840	cg2118840	48623506	TAAAAACAAAAACACAACAA			II	
-0.111199	cg2119512	cg2119512	24682453	TTCAATCACRACTTAATTTAA			II	

0.1158072	cg2119733(	cg2119733(	14633388	CTACACC	50615402	CTACGCC	I	T
0.1028107	cg2119992	cg2119992	59607454	ATCCTTAACCTTCTCAAAACA(			II	
0.2334414	cg2120227(	cg2120227(	54709323	ATTATCATCRACTTCATTTTT/			II	
-0.261877	cg2120324!	cg2120324!	16695486	AAACCAAACTAAATAACTTCC.			II	
-0.1245	cg2120795!	cg2120795!	28669333	ATAAACA	35692508	ATAAACC	I	T
0.1538031	cg2120827(	cg2120827(	43681422	CTTCTAA(	16804410	CTTCTAA(	I	A
0.1481028	cg2121064	cg2121064	15645328	TCRTTTTACCTTTAAAAACCTC			II	
0.1894664	cg2121742	cg2121742	67633484	TTCAAAAATACAATTTAAAAC			II	
-0.101107	cg2121754(	cg2121754(	29667425	CAATAAA	31734421	CGATAAA	I	C
-0.162021	cg2122184(	cg2122184(	11714457	CACCTAA	17682427	CACCTAA	I	C
-0.101983	cg2122957(	cg2122957(	72766402	ATTAACA	28702423	ATTAACG	I	A
-0.113122	cg2123248!	cg2123248!	13630343	CAACCTT	40804496	CGACCTT	I	T
0.1326327	cg2123403	cg2123403	44734473	CTATATACTTTCTAATAACCTA			II	
-0.404437	cg2123408	cg2123408	14786388	TACTATCTCAATTATTCACTAT			II	
-0.134377	cg2124244!	cg2124244!	17615461	RACTTTCAAATACCTCTATAA/			II	
0.2346141	cg2124306	cg2124306	10697439	TTATTAACCAAATAACTTCCA(			II	
-0.192065	cg2124413!	cg2124413!	42731360	CAAAAA/	68807439	CGAAAA/	I	T
0.1153308	cg2124772	cg2124772	44771401	TCAACCTACTTTCTAATCATAT			II	
-0.122465	cg2125197(	cg2125197(	28692399	AACTTTC(	22688415	GACTTTC(	I	C
0.1042213	cg2125729	cg2125729	11648326	CCTCCCCTTTATATACCCRACR			II	
0.1368731	cg2126554!	cg2126554!	22798463	AAATATCAAAAAAAATATATT			II	
0.1059971	cg2126591	cg2126591	54640409	CAATCCC	37717456	CGATCCC	I	C
-0.134398	cg2127227!	cg2127227!	54669345	ATATAAACTTCACRTATACTTT			II	
-0.166636	cg2128032(	cg2128032(	38729303	CATAACTTTTAAAAAATACCA/			II	
0.100632	cg2129134	cg2129134	39752480	CCATCTTCAAAACTCTAAATT/			II	
0.1816685	cg2129185(	cg2129185(	32715333	TAACAAACATAACAACTAAAA			II	
0.2211905	cg2129736(	cg2129736(	43669411	RTATTCACCCTAAATATTTCC			II	
-0.112148	cg2130099	cg2130099	10605381	TAAAAA/	14789475	TAAAAA/	I	A
0.2676408	cg2130134	cg2130134	16715416	ACAACTCTAATATAAAAACAA			II	
0.1165125	cg2130428!	cg2130428!	54764434	TAAACCTATAATTTACACAAA(			II	
0.122535	cg2132310(	cg2132310(	50718335	TAAACCACATCCTCCCAATAA/			II	
-0.10641	cg2133181	cg2133181	39744307	TCTACAC	14634387	TCTACGC	I	C
0.1116381	cg2133230	cg2133230	26794493	CCRCCAATAACRACACCTCAA.			II	
0.1182245	cg2134032(	cg2134032(	63719354	CCCAAAA	44756317	CCCAAAA	I	A
-0.10123	cg2134594!	cg2134594!	66710318	TTAAAAA	21782313	TTAAAAA	I	A
-0.156707	cg2138531	cg2138531	72739466	CCTTCTACCCTATATTAAAACC			II	
0.1585353	cg2138574(	cg2138574(	67616437	ACATAAA	74709458	ACATAAA	I	C
0.1393516	cg2139270(	cg2139270(	19729465	TAATCRACTAAACRAAATTTT/			II	
-0.100378	cg2139754(	cg2139754(	31744475	ATCTATATTACTAAAAATATA/			II	
0.1342022	cg2140085	cg2140085	43710318	CTTTTCACTTCTATATTATCCT/			II	
-0.183855	cg2140493!	cg2140493!	60704396	AAAAAA/	51742335	GAAAAA/	I	A
0.111352	cg2140881	cg2140881	34738319	CTTATACTTTTTAACTTCRATT			II	
0.1696	cg2141490!	cg2141490!	15671471	ATTCTCCAACAATTTATAAATT			II	

0.1292825	cg21415227	cg21415227	65715345	AAAACACCTAATAAAACAAAT			II	
0.2126825	cg21435684	cg21435684	35794359	ATAACAC	57688310	ATAACGC	I	A
-0.107006	cg21436572	cg21436572	37779393	CATAAAT	35648401	CGTAAAT	I	A
-0.128001	cg21437417	cg21437417	29648403	TAATCCRAAAAACTAACACRA			II	
0.1381845	cg21440776	cg21440776	25778431	AATATAATTCAAATTCTCTTTT			II	
0.1622816	cg21442271	cg21442271	17615445	TATACAATATCCTCATTAACRA			II	
-0.142427	cg21445553	cg21445553	33647476	CAATAAC	14602378	CGATAAC	I	C
0.2191097	cg21446772	cg21446772	69795464	CCCTTCTATCTCCCACAATACA			II	
0.1862804	cg21446981	cg21446981	69682374	CTTTAAAAAACCCTAACTATTT			II	
0.17848	cg21450228	cg21450228	46640502	CTAAAATTACTTTTACAAAAA			II	
0.1092511	cg21450627	cg21450627	74746464	ATTAAAATACAAAACCTATAA			II	
-0.153065	cg21450784	cg21450784	55615448	AACATCA	70740454	AACGTCA	I	A
0.1174245	cg21461981	cg21461981	21782468	AAAAATCTATAAAAACTCCTC			II	
-0.106449	cg21471580	cg21471580	11682463	CATATAA	40651448	CATATAA	I	C
0.1055397	cg21474104	cg21474104	18751494	TTTAAAATCCCRAAAACATTA			II	
0.1387815	cg21474247	cg21474247	27742412	ACTTTTAACATTATCATAAAA			II	
-0.106571	cg21482472	cg21482472	69797492	CCCAACA	61690493	CCCGACG	I	A
-0.110409	cg21483883	cg21483883	37804335	ATTTAAA	36737432	ATTTAAA	I	T
-0.12067	cg21484940	cg21484940	20628314	AACCACT	57756386	AACCGCT	I	A
0.1241345	cg21490751	cg21490751	19684498	CAAAACTAACATACTAAAAAA			II	
-0.107307	cg21493951	cg21493951	27713418	TAAAACA	48651339	TAAAACA	I	A
-0.177075	cg21498547	cg21498547	45773411	TAACCACAAACAAAATAACTT			II	
-0.121878	cg21513803	cg21513803	32806307	CAAAAACAAAAAACATAAATA			II	
-0.140441	cg21524061	cg21524061	26665478	TCCRAAAAACAACCATTCCCT			II	
0.1412847	cg21526778	cg21526778	28675375	ACTACTTCTATTTCTAAAAAC			II	
0.1091157	cg21527078	cg21527078	42730477	ACAATCC	47738478	ACAATCC	I	T
0.1153879	cg21527616	cg21527616	38647423	CTATATCCTATAACCRAAACR			II	
0.1281613	cg21528927	cg21528927	14672460	ATAATCAATAAACRAACTTCT			II	
0.1080016	cg21529477	cg21529477	71704423	CRTTACCAAAAACTAATTACR			II	
-0.144249	cg21530267	cg21530267	59617486	RAAAAATCTACAATCATAATC			II	
0.1257265	cg21531389	cg21531389	41625395	TTAAATCACTAATACCAAAAA			II	
-0.123958	cg21535947	cg21535947	39635363	AAACTTA	52656337	AAACTTA	I	C
0.1234834	cg21537187	cg21537187	24764306	CTCCCTCTCTCCRTCATCCAAT			II	
0.1329616	cg21543434	cg21543434	25663371	AAAAACCCTAAAAAACCTAAA			II	
-0.120381	cg21548955	cg21548955	60786424	AACTTAA	38790471	GACTTAA	I	A
0.1031018	cg21552300	cg21552300	17661452	AACTATCRCTCCCACAAACAA			II	
-0.106889	cg21557146	cg21557146	62615452	AATTCAAATTCCTAAAACCAA			II	
-0.123711	cg21561701	cg21561701	32757473	CAAAAAA	44642452	CGAAAAA	I	T
0.4939809	cg21566433	cg21566433	50669491	AACRTTAAACAAATCTTTATAC			II	
0.1113094	cg21574271	cg21574271	50724467	AAATTCCTTCAAATTCCTAAAA			II	
0.1354442	cg21574822	cg21574822	12645459	TAATTTACRTATTACRTAATTT			II	
0.1396113	cg21578457	cg21578457	62623454	AAAAAATAACTCTTTACTATTT			II	
0.2520058	cg21578644	cg21578644	11631406	AAAAACAATAAAAAATTCATA			II	

0.1010095	cg21582112	cg21582112	52731499	CACCAACATAAACAAAAATTA			II	
-0.134495	cg21586152	cg21586152	42654505	CTCATAA	16670326	CTCGTAA	I	C
-0.119195	cg21587006	cg21587006	66612314	AAAAAATCAAAAAACCTAATA			II	
0.1446179	cg21589417	cg21589417	22623481	AACTATAAAACCTAAATTTCAT			II	
-0.216897	cg21593159	cg21593159	25634351	TAAAAAATACATATATATTCTT			II	
-0.119574	cg21593409	cg21593409	12716411	CRTAAATAAACTCRATATAAA			II	
-0.102799	cg21593676	cg21593676	50643403	TAATTAACCTAAAAACTCRCTA			II	
0.2365878	cg21594702	cg21594702	46660491	AATTACTCAATCACATTAAAAT			II	
-0.111595	cg21598489	cg21598489	17665351	AATTTTAACTCATCCAATAAAA			II	
0.113623	cg21605781	cg21605781	36656336	ACCAATCTTATTAACTTCTAAA			II	
0.1215977	cg21609584	cg21609584	52647497	AAAAATAATATATTATATTAA			II	
0.1202484	cg21610839	cg21610839	55730303	AACAATTTTAACAATTTTAACC			II	
0.2732792	cg21610927	cg21610927	19687435	CAAATCACACTTTAAACTCCA			II	
0.1344649	cg21613620	cg21613620	10728413	AAACTATTCTTTATAATTTTCA			II	
0.1470962	cg21614201	cg21614201	42692387	AAAACATAAAACCTTTTTATCC			II	
0.1081953	cg21617357	cg21617357	49799346	TTCRTCTTTCAAAATAATCCTT			II	
0.1270061	cg21623445	cg21623445	31708474	ACCAAATCATATTAAACCTTCA			II	
-0.283477	cg21630843	cg21630843	13610445	TTTATCCATTTTCTTTAATTACC			II	
	cg21634944	cg21634944	38617440	TTAACAA	58629367	TTAACGA	I	A
-0.161901	cg21635870	cg21635870	52726337	AAACAAA	47718377	AAACGAA	I	A
0.2664737	cg21642050	cg21642050	46765335	AAAAAAAAACRTAAATTTTAA			II	
0.1013409	cg21643262	cg21643262	28801369	CCCRAAACTATACCTTATATAC			II	
-0.102409	cg21645268	cg21645268	58727386	TCAAACTAAAAATTCCACAAAT			II	
0.1543244	cg21645759	cg21645759	48612396	AAACAAATAACTAAACTAACT			II	
0.1023585	cg21646392	cg21646392	72635404	TAACTTCCAAAAACAAACAAA			II	
0.1709303	cg21647182	cg21647182	72714356	ACTAACCTTATTCCRATATCCR			II	
-0.111509	cg21648376	cg21648376	28640480	TAACCTC	14658488	TAACCTC	I	T
0.1203206	cg21655171	cg21655171	71731387	AAAAATTTAAAAAAATCCTTCA			II	
0.1130258	cg21670717	cg21670717	22640468	ATTTTCACCATAAATCATTTAC			II	
0.106064	cg21672992	cg21672992	74799351	AAAAATAAAACAACCCTCATT			II	
0.1651715	cg21679468	cg21679468	39808413	TTCAACAAATCTAATCAACTCT			II	
-0.10745	cg21690450	cg21690450	59707422	CTACCRATTTCTTTCTATTCTTA			II	
0.1363128	cg21690921	cg21690921	14715322	AAAACATTCRAATCACTCCCTA			II	
-0.130694	cg21692620	cg21692620	65690437	AAAAAAA	49801463	AAAAAAA	I	T
0.1460942	cg21696374	cg21696374	46777321	TCTCTAATATATATTTTATCCT			II	
-0.183444	cg21697769	cg21697769	72804500	ACTCCCATTCACACCTTAAAAC			II	
0.1229213	cg21712377	cg21712377	29754329	AAAACTACACTACRTAATAAC			II	
-0.165693	cg21717724	cg21717724	71663496	RCRCACAAAATATACAATTACT			II	
-0.162539	cg21722795	cg21722795	60720343	CAAATCA	28699467	CAAATCA	I	A
-0.110352	cg21733531	cg21733531	33698309	ATAATCC	73719447	ATAATCC	I	C
0.2224078	cg21735068	cg21735068	37686370	AACCCAATTAATCAACATTCTT			II	
-0.112021	cg21741284	cg21741284	42783350	TATATAC	70787499	TATATAC	I	A
0.2545014	cg21741515	cg21741515	32773475	AACAAAAAACCCCTACTTCTA			II	

0.1121395	cg21741998	cg21741998	38683447	AATAACATATACCTACAATCCT			II	
0.1571288	cg21749951	cg21749951	72665489	AAACAACRAAAACCTAAACAA			II	
0.1222272	cg21750589	cg21750589	62758508	TTCCTCAATATAATATTATCTA			II	
-0.117666	cg21762589	cg21762589	63757329	TTAAAAACATTATTAATCCAA			II	
0.1041331	cg21767657	cg21767657	60616459	ATATATAAAATTTAATACCTCA			II	
0.1672097	cg21770393	cg21770393	66640321	AAATCAAACRTCAAAACCTAC			II	
-0.139688	cg21777188	cg21777188	40634335	CATTAATATCTACAATCACTAT			II	
0.1423106	cg21778987	cg21778987	15666369	AAATCACCCATTTCCTTTCTAC			II	
0.1314158	cg21780600	cg21780600	15696410	ACACCAAAAACCCCATTCAAT			II	
0.1206828	cg21783442	cg21783442	12680303	ACTAAAACTATTTAAACCATCT			II	
-0.13848	cg21783672	cg21783672	60644498	TATTTACTAACCCTAACTAAAC			II	
0.1175252	cg21792983	cg21792983	35623406	CAATTTCTACATCRCCTCAACT			II	
0.1091262	cg21794767	cg21794767	56615495	TAACTAAATAAAATAAACAAA			II	
0.140084	cg21805731	cg21805731	50674364	TCTTTCTAATCCCTACTCTCTC			II	
0.1158406	cg21805880	cg21805880	36696500	TAAATCTACACRTAACTAAATT			II	
-0.212596	cg21809927	cg21809927	47711327	TCTACCA	23679433	TCTACCG	I	C
0.1435485	cg21810411	cg21810411	29679377	ACCCTATAAATATTTCTAAAA			II	
-0.225851	cg21811911	cg21811911	48689493	CTCRAAACTACRAAACTCRTTA			II	
-0.112993	cg21812850	cg21812850	39717419	AAAAATAAATTTAACTCTAAA			II	
0.1406311	cg21814550	cg21814550	16735421	AATATTACAATCAATACTAAA			II	
0.1096016	cg21823426	cg21823426	60751458	TTAACCCAATATTCTAATCTCC			II	
-0.286239	cg21829038	cg21829038	46608371	CCCCACC	13645391	CCCCGCC	I	C
-0.301794	cg21830932	cg21830932	31754304	AAAAATACCCTATATAAATCA			II	
0.1203685	cg21831183	cg21831183	65707402	CRCCTTCATTAAATTACCAATC			II	
0.1860765	cg21833776	cg21833776	73772414	AACTTTATCAATATTTTTCCAT			II	
0.1368726	cg21839504	cg21839504	44680459	AAAAAAC	47676368	AAAAAAC	I	T
0.1466432	cg21841632	cg21841632	55683413	CTAAAACCAAAAACTTACCAA			II	
0.1328647	cg21842920	cg21842920	66777402	TATCACRAAAAATATACTATA			II	
0.1044475	cg21847118	cg21847118	37664311	AAAATAATACTAATCCCAAAA			II	
0.1181465	cg21854332	cg21854332	11607314	TCATTATTAATTACCTAATCCT			II	
-0.100147	cg21855211	cg21855211	12780345	AAAATAAACCCCRTATACTAA			II	
0.1101924	cg21859562	cg21859562	19654372	CTACTTAAATTTCTTTTCTAAA			II	
0.1748837	cg21860675	cg21860675	50647478	CTAAATTCRCCTTACTAACTAC			II	
0.1532317	cg21862099	cg21862099	56698492	CRTCACACRACCAACACATAA			II	
0.1090682	cg21864016	cg21864016	40755448	AAATATTAAATTTATTCAACA			II	
0.1187366	cg21870038	cg21870038	37664451	ATCATCTCAAAAACCAAAAAC			II	
0.1074671	cg21881798	cg21881798	61795328	CRAAACAAAAAACRCCTAAAA			II	
0.2285498	cg21885112	cg21885112	73753465	TCCCAAA	56694324	TCCCAAA	I	C
0.1767368	cg21902325	cg21902325	60770320	ACACAAAAAACAAACCCAAAT			II	
-0.122788	cg21902394	cg21902394	70630496	AAAATAAAACCTAAATTTCAA			II	
-0.109372	cg21903646	cg21903646	42616369	ACAAAAT	58643484	ACGAAAT	I	C
-0.119354	cg21911276	cg21911276	15730437	CCTCTCCTAAACACCRAAAAA			II	
0.220919	cg21913335	cg21913335	26716330	CTATAAC	71672317	CTATAAC	I	A

-0.126322	cg2191591(	cg2191591(	53602438	AAACTCA	24619471	AAACTCA	I	A
-0.114691	cg2192056(	cg2192056(	47651462	CCRAACTATATTCCTACAACTA			II	
-0.106232	cg2192680	cg2192680	41692372	AACATAAACAAAAACTTATAA			II	
-0.239354	cg2192799	cg2192799	25632330	CCCAATCACCAAACCCACTAA			II	
-0.187237	cg2193171	cg2193171	24684390	ATAAAAT	25616423	ATAAAAT	I	C
0.1098549	cg2193198(	cg2193198(	73685456	AAATTACTCCTAATCAACACA			II	
-0.179032	cg2193236	cg2193236	26719328	TTAAACC	25696394	TTAAACC	I	A
-0.102406	cg2193739	cg2193739	69623491	TTTCAAATAATAATAAAACTC(			II	
0.1097676	cg2193854	cg2193854	69691430	ATTCATAAAAATAAACACCTA			II	
0.146566	cg2194563	cg2194563	30642361	AATTCAA	56781505	AATTCGA	I	A
0.1385775	cg2194663	cg2194663	56710498	CCTAACAAAATTAAACAAACT			II	
-0.132747	cg2195172	cg2195172	42767403	CTACAAA	25663443	CTACAAA	I	C
-0.155773	cg2195465(	cg2195465(	24766355	CTCTAACCTAAAAACTCTAAA(			II	
-0.107812	cg2195839	cg2195839	46665387	TAAACATAAACTCCTATAAAT			II	
-0.120381	cg2196979	cg2196979	63779384	AAACACC	44754485	AAACGCC	I	T
-0.151352	cg2197813	cg2197813	11782505	AAACATTTAACACCRTACTAA			II	
0.1501139	cg2199481	cg2199481	16603301	AAATTTAAATCACACTCTTACT			II	
0.1617525	cg2199565	cg2199565	63727351	AAATATTATTTACTAATAATC			II	
-0.119883	cg2199606	cg2199606	29712302	ACTACCACRCCCTAACCACATA			II	
0.1948797	cg2199772	cg2199772	55681501	CTTAACA	70735507	CTTAACA	I	C
0.1921954	cg2200721(	cg2200721(	13793461	ACACAAACATCAATACTTTCA			II	
0.1282242	cg2201411	cg2201411	31741504	ACTCAAC	18749447	ACTCAAC	I	A
-0.171597	cg2201808	cg2201808	18651414	AAAATAATAAATTTTTACTAA			II	
0.1561897	cg2202608	cg2202608	60740480	CAATTTCCAAATAATTATTTTT			II	
0.1779488	cg2202901	cg2202901	40750308	TCCTACA	10720446	TCCTACA	I	A
0.1399077	cg2202987	cg2202987	29656450	CCTAAAAACAACTTATTTCAC(			II	
0.1130586	cg2203202(	cg2203202(	62667465	AAAAAACTCATAAATAAAATC			II	
0.3389283	cg2203724	cg2203724	44755506	TATTCATTCTCTAACTTTAACC			II	
-0.163749	cg2204020	cg2204020	27756392	TCCAAAACCATAAAATTATAA			II	
0.1289569	cg2205163(	cg2205163(	32731457	CCTAATAACCCTTTCCTATAAC			II	
0.1856953	cg2205491	cg2205491	18731335	AAAAAAAACAATTTTTAAAAC			II	
0.1015039	cg2206404	cg2206404	72703306	CAAATACCTACTACTTAACTCA			II	
-0.177348	cg2206840(	cg2206840(	66663338	CTTATTA	27656445	CTTATTA	I	T
-0.117384	cg2206944	cg2206944	74738411	CAAATCT	27704357	CAAATCT	I	C
0.1599643	cg2207642	cg2207642	61764309	AAAAACCCCTAAAAACTTATA			II	
0.2672325	cg2207661	cg2207661	12701365	ACTAAAAAATTCCACRAAATC			II	
0.1171459	cg2207731	cg2207731	39626373	ACCTCCTCCCTCATCAAATATT			II	
0.1276506	cg2207845	cg2207845	53692421	AAAAAAATTATCCTCATAAAT			II	
-0.100945	cg2208280(	cg2208280(	11799427	ATCACAC	20794463	ATCGCGC	I	C
	cg2208505	cg2208505	24730483	AAACTTA	57749407	AAACTTA	I	C
	cg2208735	cg2208735	12768443	ATTAAAAAAATATCACCTCCA(			II	
-0.378596	cg2209492	cg2209492	37642502	AAACCCAACCAACAAAAAACA			II	
0.1103911	cg2209526	cg2209526	30706332	CATTAAAATATCTTAATTCTCA			II	

0.1183908	cg22105162	cg22105162	56718419	CTAACTAAACCAATTACATACA			II	
-0.222569	cg22106220	cg22106220	37606332	CAATAAACCAATAAAAATAAT			II	
0.1211602	cg22110158	cg22110158	56661428	CAATAAACAAAACACTAACTC			II	
0.1020842	cg22111167	cg22111167	47652314	ATCTAATTCCAAAATATTTTCR			II	
-0.105747	cg22111694	cg22111694	60693421	TACAAAACAAACTTCRTATTA			II	
-0.185098	cg22117893	cg22117893	59706422	TTAAAATAACCACACCCTATT(			II	
0.1003012	cg22118297	cg22118297	15809455	CATTCCTCTACCCTCCAAATAT			II	
-0.163678	cg22121557	cg22121557	41734419	TAACAAC	40791476	TAACGAC	I	C
0.1536731	cg22127309	cg22127309	27724371	ACTCAAACCAAAAAAATCACA			II	
0.1160674	cg22140866	cg22140866	53785452	AACTAACTATCACTCCTACATT			II	
0.1504182	cg22142142	cg22142142	37691483	ATACRAAAAATCRAAAACCAC			II	
-0.123414	cg22143285	cg22143285	74665360	ACTTAATTAACACTACAAATTT			II	
-0.102394	cg22144900	cg22144900	73794378	ACACCCA	38745448	GCACCCG	I	A
-0.106306	cg22147163	cg22147163	47795484	RAAATAAACTTAAAACRCACA			II	
0.1193317	cg22151773	cg22151773	33752365	ATTACTTTTAATATCTATAAAC			II	
-0.148011	cg22152229	cg22152229	11755402	CAATACC	12615500	CAATACC	I	C
0.1419997	cg22159647	cg22159647	10709400	CCATCAAATAAAAATTACCAA			II	
0.1086847	cg22161115	cg22161115	38611463	AACTTCCAAAACTAAAATAAC			II	
0.1244215	cg22164009	cg22164009	49680432	CAAAATCAAACCTAAAACACT			II	
-0.147993	cg22172057	cg22172057	43740344	AAAAATAAAAACCACTTATAC			II	
-0.104427	cg22185043	cg22185043	38609393	AAAAAAA	50609306	AAAAAAA	I	A
-0.203054	cg22189786	cg22189786	50767389	CCTTTAA	72751456	CCTTTAA	I	A
-0.191217	cg22190023	cg22190023	37667321	AATCCTTCCCCACAAACATTA(			II	
-0.169921	cg22198853	cg22198853	61682431	TTATCTC(	12810408	TTATCTC(	I	C
-0.127408	cg22199969	cg22199969	73764363	AATAAAA	45746334	AATAAAA	I	C
0.1001351	cg22202381	cg22202381	73628497	TACATAATAATAACTTAAAAA			II	
	cg22203829	cg22203829	67639510	CAAAATTAAATTATCCATCTCT			II	
0.110246	cg22209918	cg22209918	30609421	CTACATAAAAACAATACTATA(			II	
-0.145116	cg22210497	cg22210497	24779331	AAAAAAATTAAACACCTTTCA			II	
0.1143598	cg22214800	cg22214800	62789388	AAAAATCTCCTACTAAAAACA			II	
0.1078504	cg22216196	cg22216196	69605422	ATACTCA	58784418	ATACTCA	I	A
0.1270478	cg22222799	cg22222799	47789410	AAAACTTCTACAAACAAACAA			II	
0.1048878	cg22223119	cg22223119	39794491	CACRTAACATCAATCTTATATC			II	
0.1126444	cg22231758	cg22231758	13740491	AAACAAA	69793339	AAACAAA	I	A
0.1463968	cg22234930	cg22234930	32803418	TAAAAACAAATCAAAATATAT			II	
0.1093373	cg22236066	cg22236066	25762347	TAACCTCATCCTCTCTAAATTT			II	
-0.173289	cg22237018	cg22237018	27797434	ATACCTACCTTTCTAATAAAAA			II	
-0.173454	cg22237495	cg22237495	59714316	TTCTTACTCTTCACAATACTAC			II	
0.1600306	cg22237644	cg22237644	15786353	CAAATACTTACTATATACTTAC			II	
-0.114769	cg22242857	cg22242857	61748404	AATCAATATTTTTTATTTTATA(			II	
-0.120672	cg22243109	cg22243109	39666377	TCAAAAC	19677413	TCAAAAC	I	A
0.1012938	cg22247748	cg22247748	20605478	CTCTTCTACAACAAAATCRACA			II	
0.1241471	cg22251684	cg22251684	59672310	AAATCTACTTTACTTAACTAAA			II	

0.1506361	cg22260897	cg22260897	17786473	AAAAACTACACTCAAACTCAA			II	
0.1417457	cg22264980	cg22264980	15656323	TAACTCCAAAAAATACAAAAC			II	
0.1000473	cg22274117	cg22274117	33784443	CTACACAACATCAACTTATTCA			II	
0.1030949	cg22278087	cg22278087	56639356	ACTACAAAACTACTTCCCCTAA			II	
0.127737	cg22282089	cg22282089	48656396	AAATCTCACRTCTCTACTAAAT			II	
0.1236263	cg22290140	cg22290140	28751506	AACTCTA	70754366	AACTCTA	I	A
-0.104149	cg22296585	cg22296585	57770413	CRCTAAATTTCAAACTAAATAC			II	
-0.275863	cg22304519	cg22304519	53635488	CCCTTTAATTAAAATCAAAATC			II	
0.1454996	cg22306009	cg22306009	71772431	ACAAAAAATATTTTAAAAATT			II	
0.1171365	cg22316451	cg22316451	36810353	CTAAACTTTAAAATACRCTACT			II	
-0.201358	cg22316614	cg22316614	34764306	CCAACAA	67739463	CCAACGA	I	A
0.1026057	cg22321327	cg22321327	49710463	CAAATATACTTTCCAAAACTAT			II	
-0.117895	cg22325823	cg22325823	43632388	TCTTAAATATCCCCAAAATCCC			II	
0.1465127	cg22335802	cg22335802	39743399	CAAATCA	34757343	CAAATCA	I	A
0.3074989	cg22336867	cg22336867	55637506	CAAAATCTTAAACCCAAAAAA			II	
0.1686523	cg22337626	cg22337626	36714448	AACACCAATCTTTACATAAAA			II	
-0.165153	cg22340508	cg22340508	12808405	AACTTTC	24758434	GACTTTC	I	T
-0.237885	cg22341310	cg22341310	70701466	TCCRAAACCACTAAAAACTATA			II	
-0.115766	cg22343728	cg22343728	26622337	ACAATAA	59671385	GCGATAA	I	C
-0.127376	cg22352772	cg22352772	47718389	CRACRACRACTACACTAAAAC			II	
-0.111448	cg22361106	cg22361106	63806459	AAATATACAACACCAATTCTA			II	
0.1217917	cg22364890	cg22364890	57682467	TTATTAATTTCTCTACCCACTA			II	
-0.149448	cg22367705	cg22367705	65647306	CTAAAAC	59700369	CTAAAAC	I	T
0.1166649	cg22367981	cg22367981	25645372	CTTAAACTTCTTTTTAACTCTA			II	
0.2358912	cg22373097	cg22373097	35685405	TTTTCCCTTCAATTAAAAATCT			II	
0.1052707	cg22390040	cg22390040	59608335	ATAACAAAATACRCACATCCA			II	
0.6942551	cg22402121	cg22402121	23779301	TAAATAT	34774468	TAAATAT	I	A
0.1140352	cg22408108	cg22408108	60731414	ACTACCCAAATAATTAAAAAC			II	
-0.112226	cg22411185	cg22411185	47682498	AAATTATTCRTTCCTCCTATTA			II	
0.1224598	cg22420514	cg22420514	17693448	AAAAACAAAATTATAAAAATC			II	
0.135219	cg22422251	cg22422251	73660487	ACAACRATACTCTATAATTAA			II	
0.1227504	cg22428691	cg22428691	51644345	AACTACTCCTATCAACTATTCA			II	
0.1174997	cg22437405	cg22437405	36771482	AAAACACTATAAACTCAAAAC			II	
0.1461534	cg22442617	cg22442617	62754307	CCTATCCAATAAATCAAAACRC			II	
0.5093445	cg22443212	cg22443212	65611468	AAAACCTTCAAAACAAACATA			II	
0.1501974	cg22450406	cg22450406	27629332	TCCCTACAACCTCATAAATAA			II	
-0.124257	cg22452230	cg22452230	11725483	AAACTCC	42645456	AAACTCC	I	C
-0.112096	cg22455667	cg22455667	52783348	CAATCCA	41720477	CGATCCA	I	T
0.1152522	cg22473333	cg22473333	27676319	AAACCTATAATCTAAAAACTTA			II	
-0.106885	cg22481162	cg22481162	66810335	CCATAAT	19775311	CCATAAT	I	A
0.2748025	cg22502308	cg22502308	47810318	ATAAATAAATATATCTCACAC			II	
0.2541334	cg22508145	cg22508145	59700445	TAATCAAAAACTATAACCCACC			II	
0.1202094	cg22508930	cg22508930	73600419	ATACAAAATAACCTTTTTAAAA			II	

-0.116953	cg22509113	cg22509113	58605415	ACTAATT	63801383	ACTAATT	I	A
0.1104484	cg22530232	cg22530232	48703391	CAAACAAATAAAATCRAACTA			II	
-0.110653	cg22534288	cg22534288	44735506	CCAAACA	51738369	CCAAACC	I	C
-0.13292	cg22541254	cg22541254	14721442	AATAAAATAAAACCTAACAAA			II	
0.1673455	cg22542451	cg22542451	17773359	ATAAATCTTATCTACCTTAAAA			II	
0.1026473	cg22543374	cg22543374	59706414	TCTATCCACCTAAATCCCATAA			II	
-0.227996	cg22543924	cg22543924	63771360	AATTATTCTCAAACTAAATTTA			II	
0.1733639	cg22545121	cg22545121	62657346	CAAACCC	49710451	CAAACCC	I	A
0.1157399	cg22550815	cg22550815	72640301	CAAACTCTATAACTTACAAAA			II	
0.1201236	cg22561794	cg22561794	43675403	TTTATCCTACCTTAATACTATA			II	
0.1458736	cg22563815	cg22563815	35731370	ACAAACACTTAACACCTCTAAT			II	
-0.153154	cg22569496	cg22569496	61810439	CCTAAAA	10741321	CCTAAAA	I	A
0.1204345	cg22576265	cg22576265	46701454	TTAAAAAACTATAAATTCCAAT			II	
-0.122151	cg22577606	cg22577606	53709439	ATACCCCTAATAACRATAACTA			II	
0.1047185	cg22579075	cg22579075	55763419	TCTCCTATTCTAAAAAATCTAA			II	
-0.128235	cg22585954	cg22585954	29749339	ATTATTATCCTAATATTCTAAT			II	
0.1149976	cg22587600	cg22587600	30659497	AAAAACCCAAATCATACTAAC			II	
	cg22588144	cg22588144	12768471	AATCCAA	16693306	AATCCGA	I	C
-0.107249	cg22589728	cg22589728	73648503	TCAAATAAACAAAAACCAACT			II	
0.1103241	cg22589822	cg22589822	63618321	TCTACCCCCACTAAATAATAA			II	
0.1016623	cg22597210	cg22597210	43696442	RAATCAAAACTTCAAAAACCA			II	
0.1065768	cg22601415	cg22601415	54726304	CAATAATTCCCATAACTCTCCA			II	
0.1036603	cg22603268	cg22603268	27673429	TAAACAAATCTTACTCCTATAA			II	
0.1206228	cg22611120	cg22611120	23694300	AAACAATAAAATATCTATTATA			II	
0.1032549	cg22612448	cg22612448	44674486	CTCAAAC	13747358	CTCAAAC	I	T
-0.106823	cg22620689	cg22620689	48767360	TCCAAAACTCAAATAACTTAAT			II	
-0.115532	cg22622899	cg22622899	38644415	CCAAATCRCTACATACCTATAA			II	
0.1558413	cg22624907	cg22624907	30641306	CATCATTCAAAACAATAACTAA			II	
0.1132473	cg22627029	cg22627029	35617474	CAACTCC	36799401	CAACTCC	I	T
0.1016591	cg22631616	cg22631616	49803433	AAAAAAAACTAAATTACCACA			II	
-0.155254	cg22632523	cg22632523	35766468	ATACAAC	37606499	ATACAAC	I	A
0.1037528	cg22634633	cg22634633	18779345	AAATAAACAACTAAATCTATA			II	
0.1170984	cg22635491	cg22635491	63726451	CACCATA	34610498	CGCCATA	I	A
0.1289597	cg22637941	cg22637941	67726462	CTTAAAATTTTCAAACTCAAAA			II	
0.1002604	cg22640819	cg22640819	20771450	ACTTTTAACCAATTATTTCCAC			II	
0.1112375	cg22647689	cg22647689	16783379	CCCAAAAAAAACAACTCCTAC			II	
0.1257066	cg22653140	cg22653140	62627395	CCTTCCA	48804333	CCTTCCG	I	A
-0.178109	cg22653957	cg22653957	39672463	AAAACAA	52765451	AAAACGA	I	A
-0.165277	cg22657059	cg22657059	31653389	CCTATATATACCCTAAAACTAT			II	
-0.114334	cg22659262	cg22659262	64795501	ACAACCA	35739490	ACGACCA	I	A
-0.104045	cg22660933	cg22660933	47756478	CCTTCCTA	13763395	CCTTCCTA	I	A
0.2935995	cg22664298	cg22664298	29680347	ACRTCCAAAACACAAAAAATA			II	
-0.10875	cg22672606	cg22672606	11633459	ACATAAA	57720491	ACGTAAA	I	C

-0.228449	cg22673542	cg22673542	55723396	AAAAACCTTAAAATAACCTAC			II	
-0.107931	cg22676075	cg22676075	36774424	AATAAAA	49760474	AATAAAA	I	C
0.1119271	cg22678674	cg22678674	32674499	AACAATA	46613423	AACAATA	I	A
0.1014403	cg22678739	cg22678739	10612410	TTAATAACRACTTACTAAAAC			II	
-0.108005	cg22679014	cg22679014	27807312	RAAATAATTTCTTCATTCATCT			II	
-0.100055	cg22681709	cg22681709	46668356	TACTAATAACTCACTACACATA			II	
-0.217676	cg22681945	cg22681945	49730486	CAAAATAAAAAAACTAAATCA			II	
0.1038514	cg22682200	cg22682200	58691497	TTTCCRCACACAACRATAACTT			II	
0.105518	cg22683010	cg22683010	57666362	CTTCATACRCRACTTCATATTT			II	
-0.123427	cg22683036	cg22683036	19808382	TTTTCCATATTCAAATTATTTC			II	
0.1104213	cg22684370	cg22684370	13609479	CTTCCAA	44713442	CTTCCGA	I	A
-0.163609	cg22696546	cg22696546	17676435	TAACRACTACRTAATACCTAA			II	
0.1653413	cg22699314	cg22699314	66748409	AAATTTACRCAAAACACAAAA			II	
0.1270798	cg22699620	cg22699620	49665394	ACATATTTCTTTTCCCRTTCAA			II	
-0.161404	cg22700015	cg22700015	22767393	TAATCAA	52774327	TAATCGA	I	A
-0.153472	cg22700790	cg22700790	67694374	CCAAACT	53774307	CCAAACT	I	A
0.1082546	cg22700822	cg22700822	22648488	TTTAATCTAAATTCTATACACC			II	
-0.114425	cg22705954	cg22705954	53786440	AAAAAAAACRAACRCCTAAAT			II	
0.1143681	cg22708961	cg22708961	54703456	AACACTA	70629384	AACGCTA	I	A
0.115006	cg22711676	cg22711676	55697508	TATATAAATATCTAAATACAC			II	
0.1008368	cg22716262	cg22716262	70793361	AATTTTATTTCAACTTAACTTC			II	
0.394307	cg22717235	cg22717235	23800358	CCAAAAATTCCTAATCCCCAA			II	
-0.182639	cg22717478	cg22717478	45700416	TAAACAA	37802319	TAAACGA	I	C
0.1023447	cg22727432	cg22727432	16809362	ATAACAAAACTAAAAAATTCC			II	
0.1100367	cg22730029	cg22730029	12650394	TAAACCCAATTATTCTACTAAA			II	
0.1941114	cg22730047	cg22730047	73676317	AACCAAA	15721361	AACCAAA	I	A
0.1077178	cg22732126	cg22732126	27620327	CAACCCTAACAAATCTTTAAAC			II	
0.2367625	cg22741595	cg22741595	26754377	TCTATTCAAAAACCAAAATCRA			II	
-0.118795	cg22745102	cg22745102	66648508	TTTTCTTCCAAAAAAATCCTAA			II	
0.1049562	cg22745624	cg22745624	70606419	ACRTATTTCAAAATCRAAACCA			II	
0.1173385	cg22746865	cg22746865	60650501	AAACCTAAAACTCTAAAAACC			II	
-0.118266	cg22747650	cg22747650	63759321	ATATTAAACAAAAACTTAAAA			II	
0.1044173	cg22759261	cg22759261	31682388	ATTTCCATTAAAATATACRTTT			II	
0.1277982	cg22763593	cg22763593	41798424	AATAATAACTATAAAAAACTC			II	
0.2717314	cg22764591	cg22764591	19801466	AAACCCA	58622419	GAACCCC	I	C
0.311546	cg22771759	cg22771759	63691435	AATACCCAAAAACCACAAAAA			II	
0.1454968	cg22775600	cg22775600	69745429	CCTATAAACTAACTCAAAATTC			II	
0.2774979	cg22777560	cg22777560	14786382	TTACACCAATAAATACATCATT			II	
-0.292067	cg22781764	cg22781764	27713505	TCTAAACACACTTCTTAACACT			II	
0.1451589	cg22786472	cg22786472	13717427	CCAATATAACTCTTCCTTAACT			II	
0.1263264	cg22786748	cg22786748	49745403	AACCAAACACAATCCCTATTCT			II	
-0.148722	cg22789318	cg22789318	70685378	AAAAACC	22676408	GAAAACC	I	T
-0.103867	cg22789982	cg22789982	31797416	AAAAAAT	59652457	AAAAAAT	I	C

0.1115251	cg2279087(	cg2279087(	46786313	ATCATATACCATATATCAAAC]			II	
-0.120545	cg2279198	cg2279198	67764353	AATAAAA	57737497	AATAAAA	I	A
-0.10237	cg2279306	cg2279306	57707436	ACCAAAC	15678323	ACCAAAC	I	C
-0.119557	cg2279534	cg2279534	41687317	ATAAACA	15801383	ATAAACC	I	A
-0.190057	cg2280189(	cg2280189(	31802445	ACTAAACAAATAAAACACCAT			II	
0.1113901	cg2280201	cg2280201	74740492	ACATCCAACATTTAACATCAC]			II	
-0.108927	cg2280229(	cg2280229(	17684406	AAAAAAAATAACTATTTCCTA			II	
0.1124072	cg2280452	cg2280452	39610321	TATTACTAAAACRAATAACTA			II	
-0.178068	cg2280480	cg2280480	24717506	ATAAAATATAATCTAATCTAC			II	
0.121536	cg2281261	cg2281261	12751429	CCCATAAAATAATAAAACTCA			II	
0.1050116	cg2281402	cg2281402	27604327	ATCAAAT	36741365	ATCGAAT	I	A
-0.115589	cg2281646	cg2281646	30630456	CTAAAAA	13682306	CTAAAAA	I	A
-0.15238	cg2282657	cg2282657	48779380	AATACTA	19609302	AATACTA	I	C
-0.135924	cg2282918	cg2282918	30777395	ATCRTAAAAACTCCACCTAAA			II	
0.1278018	cg2283347	cg2283347	69655472	AAAACTTAACCCCAACCATTT			II	
0.1723925	cg2283414	cg2283414	39767488	CAAAAACACTACAACTAAACT			II	
0.121069	cg2283454	cg2283454	73755435	TAAATCAAAAACTCTAAATTA			II	
-0.12405	cg2283563(	cg2283563(	30777385	ACCAAATTAACAACAATACRT			II	
-0.137929	cg2285194	cg2285194	27650394	CTCTACCTAACRATAAACTCCF			II	
-0.100633	cg2285632	cg2285632	60663333	CAACATA	18745388	CAACGTA	I	A
-0.107248	cg2286235	cg2286235	13670335	TTCCACTCCCAACTCCTAAACT			II	
-0.192079	cg2286370(	cg2286370(	14810313	CCAACAC	36777310	CCAACAC	I	A
-0.113395	cg2289106(	cg2289106(	67760498	CAACACC	37743313	CGACACC	I	A
0.1107113	cg2290047(	cg2290047(	47648350	CTCTAAATAACCCTAAACCTC			II	
0.142488	cg2290440(	cg2290440(	63689409	CTCACTTTTATCTTACAACATT			II	
-0.27738	cg2290551	cg2290551	10777300	CCAACAT	62767353	CCGACGT	I	A
0.1383104	cg2290729	cg2290729	25656423	CCACCCCTACAAAATAATACA			II	
-0.124517	cg2290908	cg2290908	31672308	ATTTTCATCATTAAAACTATAC			II	
0.1606972	cg2291734(	cg2291734(	33807428	RCAATCTCCCTTCTACTAATAA			II	
0.105298	cg2291804	cg2291804	56689381	TTTCCCRAAAACACCCAATAA			II	
0.2218972	cg2292686	cg2292686	21730447	CTCTCTCAACATTTTCCCCTAC			II	
0.1252899	cg2293115	cg2293115	44794321	CTAAAACTTTAAAACATAATA			II	
0.1066338	cg2293145	cg2293145	33604408	TATTAAACTTCATTACAATAA]			II	
0.2850172	cg2293380(	cg2293380(	53623508	ATCATTCTCAAATTTCCCTAAA			II	
0.1146722	cg2293542	cg2293542	58611458	CAAAATCCAAAAACCTAACTA			II	
0.1204156	cg2293625	cg2293625	29744499	CCCCTCTAATATAACTCTTTTA			II	
0.1102591	cg2293735	cg2293735	10764462	TATAACCTCAAATATCRTATTT			II	
0.1082474	cg2293970	cg2293970	11632479	CAAATCCTAAACTAACTACCC(			II	
0.1271241	cg2294480	cg2294480	24604355	TTTTTTTTTTTCTACCTTCAAA			II	
-0.11463	cg2294976	cg2294976	42718400	CRATACRTACAATAAAAATCC(			II	
0.1200871	cg2295083	cg2295083	38624309	CATAACCCTCTCTCACTATCCT			II	
0.118256	cg2295368	cg2295368	59715476	AACCTAAATTTCTCCTAAAAC(			II	
0.1664849	cg2295663	cg2295663	25629423	ATTACAT	41654421	ATTACAT	I	T

0.1068175	cg22969108	cg22969108	51682366	ACAAATCATATTAAATCAATT			II	
-0.222471	cg22970003	cg22970003	18714369	TAACTTATTCCCAATACTACTC			II	
-0.107723	cg22971216	cg22971216	27643360	AAAACTACTTCCCCTAACTTCC			II	
0.2320217	cg22972806	cg22972806	46726489	CACACAA	43680309	CACACAA	I	A
-0.115335	cg22979783	cg22979783	67806326	ATATAAC	24759318	ATATAAC	I	T
0.15683	cg22984587	cg22984587	37602338	AAATAAC	29733506	AAATAAC	I	C
-0.179294	cg22986662	cg22986662	32810386	AAAAAAC	62806350	AAAAAAC	I	A
-0.100419	cg22991320	cg22991320	23775482	AAAAATA	19733510	GAAAATA	I	T
-0.112528	cg22992497	cg22992497	46762356	TCTAAAATTTATCAACAAAAC			II	
-0.104109	cg22992730	cg22992730	48680468	ACAAAAT	23639303	ACAAAAT	I	A
-0.157546	cg22993195	cg22993195	44704364	AATAAAA	22743479	AATAAAA	I	A
0.5956249	cg22996768	cg22996768	43608419	TTCCATTATACCCTAAAACCA			II	
0.1023701	cg22999620	cg22999620	31727365	AACAAAA	47793449	AACAAAA	I	A
-0.359266	cg23005102	cg23005102	68655481	AAAAAAC	54730315	AAAAAAC	I	C
-0.102891	cg23008562	cg23008562	41675351	TATAAAC	38711325	TATAAAC	I	C
0.2277082	cg23009094	cg23009094	59611434	AAAATAC	51731434	AAAATAC	I	C
-0.10574	cg23010629	cg23010629	35619380	CCCCTAAATAATCTTAAAAAC			II	
0.1437973	cg23012310	cg23012310	50664382	AAATATT	30761362	AAATATT	I	C
0.1880468	cg23012654	cg23012654	12657463	ACAACAACTATCCTAAATAAT			II	
-0.236082	cg23019589	cg23019589	31615444	CAATTTAAATTAAACTCAACT			II	
0.1874286	cg23022053	cg23022053	67711384	ACCAATTTCTCTCACAAAACA			II	
0.1871668	cg23028740	cg23028740	49756401	ACTCATTCTTATTTTTCAAAAC			II	
0.1130295	cg23030200	cg23030200	37721486	AAACAAAAAATACCTCAAAAA			II	
-0.115864	cg23034922	cg23034922	43682329	TTCCTCRCCAAATTAATAAAC			II	
-0.111165	cg23037265	cg23037265	36602441	TATAATAAAATTAATAACCCT			II	
0.1059931	cg23045277	cg23045277	61622368	ATCCTTAAAAACATATTCTTTC			II	
0.2055765	cg23051926	cg23051926	39676303	ATATACTACCAATTCCTTAAAA			II	
0.2737093	cg23053506	cg23053506	68659434	TAAAACC	44808484	TAAAACC	I	A
0.3082822	cg23058319	cg23058319	36693466	CCTAACC	67652467	CCTAACC	I	A
-0.102766	cg23061114	cg23061114	46647510	AAACCAA	28752344	AAACCGA	I	A
0.2189348	cg23061795	cg23061795	73733463	TCCCCCTAAATCTCTAACTAAA			II	
0.2087563	cg23066280	cg23066280	72707452	CCAACCA	57736329	CCGACCA	I	T
0.1242616	cg23069046	cg23069046	53733425	CTTTCTTTACTTATAACAAAAA			II	
-0.164267	cg23074932	cg23074932	28749411	CRCCAAAAACTTAAATTTAAA			II	
0.1060148	cg23075611	cg23075611	56645475	AATCCTTACAATTATCTTAACT			II	
0.1493584	cg23079063	cg23079063	39756485	CACTCTAAACATATCCTTATTA			II	
0.1257737	cg23084506	cg23084506	33767397	CCTTAAATCCTACCCACTTCCA			II	
-0.131061	cg23085143	cg23085143	27684492	RCAAAAAAAAATCAAAAATCA			II	
0.1091407	cg23090207	cg23090207	47796459	CTCTACCCTAAACAACRTCCA			II	
0.1525276	cg23098068	cg23098068	12711505	CAAAACTAAAATTCAAAACTA			II	
-0.116795	cg23098789	cg23098789	23786356	RACTCAAAATACCCATCRCRA			II	
-0.119492	cg23106045	cg23106045	27662435	TACTACCTAAAACCCTTCRCTA			II	
-0.105323	cg23109867	cg23109867	68739412	TCTATAC	57770329	TCTATAC	I	A

-0.112681	cg2311461(	cg2311461(	67614345	CTAAACC	58772358	CTAAACC	I	A
-0.126239	cg2311738(	cg2311738(	39656417	TTTAAATATAAAAAATACTTA/			II	
-0.142172	cg2312325(	cg2312325(	26689339	AATACAT	64768303	AATACAT	I	T
-0.273421	cg2312851(	cg2312851(	34773406	ATAAATTTAATTCTTATCCTAA			II	
0.1274688	cg2313083:	cg2313083:	41611411	AAATACCATCCTTTTACTAACT			II	
-0.125638	cg2313674:	cg2313674:	17660464	CAACATT	66795481	CGACGTT	I	A
-0.106017	cg2314185l	cg2314185l	72804303	ACTTAAACCCAACCACCTAAT/			II	
0.1005059	cg2315650(	cg2315650(	69727490	TATCAAATAAACTAATCAAAA			II	
0.11251	cg2315818(	cg2315818(	32629444	ATAACCA	12713425	ATAACCG	I	T
0.5681564	cg2315997(	cg2315997(	72704311	AAAATAAAAATTATTATAACC(			II	
0.105302	cg2316259(	cg2316259(	59687470	AATTATATTCTCTAAAACCTTC			II	
-0.106733	cg2316742:	cg2316742:	59770497	CTCTAATTTCCCAATCTTTTAT(			II	
-0.136118	cg2316852(	cg2316852(	67794317	CCAAAAAATACTTAAAAACCT			II	
0.10437	cg2317110:	cg2317110:	19799376	TACTTCTCCCAAAAATAATAT/			II	
-0.103254	cg2317340:	cg2317340:	61624403	AAAATTT	63721342	AAAATTT	I	A
-0.125366	cg2317368(	cg2317368(	18794419	AAAACCT	62765313	AAAACCT	I	C
0.1132191	cg2317557:	cg2317557:	51749314	AAAACCAACCCAACAAAAATT			II	
0.1098408	cg2317661:	cg2317661:	59602301	TTCTACTCTTTAAAATCCACRC			II	
-0.130121	cg2318284(	cg2318284(	34670367	CATTCTA	65739445	CGTTCTA	I	A
-0.14455	cg2318731(	cg2318731(	59663416	AACCTTCCACCCATTAAAATT			II	
-0.163334	cg2318868(	cg2318868(	63604477	AACCTCC	25783483	AACCTCC	I	A
0.293646	cg2319576:	cg2319576:	44748359	RTATTTTTCTCTACTATCTTTAT			II	
0.1072862	cg2320272:	cg2320272:	12694323	CATCTATAAATAATATAAAAC			II	
0.1201758	cg2320935:	cg2320935:	59758416	AAAACAAAACTAACTAACAAC			II	
0.1175476	cg2320994l	cg2320994l	37721439	AAAAAATACTATAAATTAATC			II	
0.1332673	cg2321052l	cg2321052l	49695350	TAACACATTTTCTTTATCTATT/			II	
0.1207998	cg2321244:	cg2321244:	74799377	TTAATACTAAACCAATCCTCC(			II	
0.1268975	cg2321443:	cg2321443:	57770404	CTCAACAAATTTTAACAAAAC(			II	
-0.107386	cg2321476(	cg2321476(	62803315	CAAATTA	22614428	CGAATTA	I	C
-0.143324	cg2321835(	cg2321835(	60646428	ACACTAT	18724379	ACACTAT	I	C
0.1647452	cg2321836:	cg2321836:	70727307	CCACAAAACCAAAAACTACTT			II	
-0.172759	cg2321855(	cg2321855(	45740474	AAACAAC	35708392	AAACGAC	I	C
0.1491842	cg2321895(	cg2321895(	58717327	AATAAATAACAAAAACAAAA/			II	
0.1191912	cg2321925:	cg2321925:	26748333	ATAAAACCAAAATAAAACAAT			II	
-0.122508	cg2322435(	cg2322435(	55717393	AATACAA	30647326	AATACGA	I	C
-0.141787	cg2322691(	cg2322691(	23658400	ATCCCAA	43769447	ATCCCAA	I	A
-0.146432	cg2322852(	cg2322852(	12778419	TCATCAC	49678399	TCGTCAC	I	C
0.1508376	cg2323083(	cg2323083(	54682481	AAAAAACAAACTTAATTTCCT(			II	
-0.189645	cg2323163l	cg2323163l	39659445	TCCTAAACATCTAACTTAATAT			II	
-0.216168	cg2323341(	cg2323341(	15758300	CAAATAACTAAATACTAAAAA			II	
-0.100481	cg2323879:	cg2323879:	13741416	TCTACCT	64642364	TCTACCT	I	A
	cg2324214:	cg2324214:	36799442	CCCACAC	62803492	CCCGCAC	I	A
-0.114976	cg2324491(	cg2324491(	70620478	AAATACA	16603329	AAATACC	I	A

-0.138059	cg2324794	cg2324794	46605301	AAATTAA	54684461	AAATTAA	I	A
0.1122293	cg2324842	cg2324842	33740452	AAATAATAACACACCTTAAAT			II	
-0.101283	cg2325057	cg2325057	40613384	AAAACAT	62690339	AAAACGT	I	C
0.1170224	cg2325648	cg2325648	71759373	ACAACCC	63640366	ACAACCC	I	C
-0.127442	cg2325954	cg2325954	29766366	CCTCTCCTAAATAACRAATTA			II	
0.1118174	cg2326117	cg2326117	18708502	CAACTCATTAACCCTTAACTC			II	
0.1131959	cg2326863	cg2326863	63735467	AAAAAAAACAACTAAATAAA			II	
	cg2328152	cg2328152	59606390	ACCAAA	43696426	ACCGAA	I	C
0.1509398	cg2328883	cg2328883	68682381	TAATACAATAAATAACCCCCC			II	
-0.116822	cg2328902	cg2328902	58802339	CAAACTACRACCTAAAACCCC			II	
-0.108791	cg2328985	cg2328985	23752376	CCTAACAACACCTCTACTAAT			II	
0.1621413	cg2329031	cg2329031	48724355	CCTTAAAAAAACCAAATCCTA			II	
0.2231183	cg2329679	cg2329679	72682370	TAAATATTTTAAAAAATTAAA			II	
0.130327	cg2329886	cg2329886	11700305	CTTCCAA	34632345	CTTCCAA	I	T
-0.288898	cg2329991	cg2329991	19700344	TTCTAAC	20734385	TTCTAAC	I	A
0.1427254	cg2330274	cg2330274	44725301	ACATATATATATTTATCTATAT			II	
0.1080047	cg2330299	cg2330299	10626312	TAAAATCTCACTAAAAAAATA			II	
-0.138368	cg2330512	cg2330512	31657469	CCCCCAA	23720345	CCCCCGA	I	C
0.2197034	cg2330589	cg2330589	64730380	CTAAAAA	73795306	CTAAAAA	I	T
0.3399447	cg2333143	cg2333143	24708349	TCAAATCTTCTAAATACAATA			II	
0.1885607	cg2333349	cg2333349	58633483	ACTCCAAACTAACCTACTCCT			II	
-0.105266	cg2333648	cg2333648	21771471	AACCAATAAAAAACTCAACAT			II	
-0.102678	cg2333765	cg2333765	59667438	AACRACTCTCACCTCATCAAC			II	
0.1563255	cg2333838	cg2333838	49774469	ACCACAAAATAAAAAACACA			II	
0.1708007	cg2334550	cg2334550	42759326	TAAAATATCAAAACATTTACA			II	
-0.120629	cg2334897	cg2334897	31695334	AACAAA	42783446	AACGAA	I	T
0.1113695	cg2335170	cg2335170	21606469	ACAAACTATTAAAAAAAACTA			II	
0.1814604	cg2335224	cg2335224	44763338	TAAATAA	73707305	TAAATAA	I	C
-0.136273	cg2336404	cg2336404	42654436	AATCAAC	56704416	AATCGAC	I	A
0.1565812	cg2337147	cg2337147	46780375	TAAAAAACATTTTCCATTTATC			II	
-0.200608	cg2337158	cg2337158	61718377	ACACTAACCACTAAAAAATAC			II	
-0.124922	cg2337655	cg2337655	41759380	TCATTTTTTCRCCATCRTTAT			II	
-0.134971	cg2339011	cg2339011	14778329	AAACCAC	13618360	AAACCGC	I	C
0.6040583	cg2339238	cg2339238	29658437	TATTAACTCTACCRTTCCAAA			II	
0.1174801	cg2340044	cg2340044	53742492	AAACTCCCTCTCTAAAAATCA			II	
0.102062	cg2340179	cg2340179	41668374	CAAAAA	31702423	CGAAAA	I	T
0.1469278	cg2340230	cg2340230	44670458	AATTATTCTTAAAACATTCTTT			II	
0.2350529	cg2340473	cg2340473	33614370	CTCTAATTCCCTCTTATATTAT			II	
-0.114487	cg2341486	cg2341486	60804361	AACCAAA	49684479	AACCAAA	I	A
0.1564485	cg2341807	cg2341807	63758467	CTCCTAACCAAAAACTCTCAA			II	
0.1279255	cg2341896	cg2341896	22654362	TAAAACCAAATCATCTAAATC			II	
0.2625141	cg2342139	cg2342139	14675470	ATTCTACAAACCACAAACTAT			II	
0.3088818	cg2342189	cg2342189	49705387	AATTCTTCCAAAAACATCAAA			II	

0.1380913	cg23429749	cg23429749	52632490	CRCAAAAAACATAACAACTAT			II	
0.4687389	cg23438516	cg23438516	50695345	AAATACAAACRCAATTTATCRT			II	
0.1042161	cg23440389	cg23440389	18738325	TTACRTATTCATAACTCCATTT			II	
0.1606121	cg23441248	cg23441248	59728457	AAAATATAATCAACACACAAT			II	
-0.116748	cg23442198	cg23442198	23739407	ACCTAAA	62718433	ACCTAAA	I	T
0.1074091	cg23462305	cg23462305	74612343	TCTCCCTAAACRTTATATATTA			II	
-0.218009	cg23463680	cg23463680	22625473	ACAATATTAAATTTACCTAAAA			II	
-0.156965	cg23464743	cg23464743	51804485	CRAACTAATTTTACATACACCT			II	
-0.154369	cg23476877	cg23476877	66602389	AATCAAA	66725344	GATCAAA	I	C
0.1123167	cg23481024	cg23481024	16732386	TATTTTTCAACATAAACRACCA			II	
0.1150312	cg23481862	cg23481862	74770344	AACACTAACTAAAACCAATAA			II	
-0.18342	cg23490161	cg23490161	59606495	CCCCAAT	34632365	CCCCGAT	I	C
-0.256442	cg23496178	cg23496178	69629438	AAAACTTTCRTCCTTACCCAAC			II	
-0.114969	cg23497306	cg23497306	38764414	AAATACA	53764352	GAATACA	I	C
0.1786569	cg23497569	cg23497569	45679428	CACAACTACCAATCCAACTATA			II	
0.1067709	cg23501005	cg23501005	20681329	TCAAAATACTAAAATACATAT			II	
0.1037101	cg23505044	cg23505044	20795336	CATAAAA	16688394	CGTAAAA	I	T
0.1197544	cg23508201	cg23508201	50687494	CAATCATCCTAAAACAACTCA			II	
0.2213913	cg23513018	cg23513018	42775378	AAAATACACAAAATACCTTTTA			II	
0.1617854	cg23515496	cg23515496	63698447	ACAACCCCAACTAACRAAAAA			II	
-0.205702	cg23517115	cg23517115	25611479	AACTCTCAACACCTAACCRTAA			II	
-0.20685	cg23517941	cg23517941	62643304	CCACAAA	23765336	CCACAAA	I	C
0.1209797	cg23521140	cg23521140	52617473	AACCAAA	47673385	AACCAAA	I	A
-0.317347	cg23523359	cg23523359	40683383	ACCTTCCCTAAAATTATAAAA			II	
0.1047092	cg23543481	cg23543481	13689325	CCTATAAAAACRTAAATATAA			II	
-0.137428	cg23549902	cg23549902	36622495	AACTATT	65717441	AACTATT	I	T
0.106637	cg23550704	cg23550704	34740420	ATACTTTCTTCAACACACAACT			II	
-0.139113	cg23551722	cg23551722	73611464	CTAACTCCCAACTTAAAATTCT			II	
0.3798285	cg23564471	cg23564471	57798316	AAAAACTAACTATCCTCCCATA			II	
-0.118603	cg23567120	cg23567120	36634317	AAAATAATCAAAAAAACCTCC			II	
0.192421	cg23579537	cg23579537	26799415	ATCAAAACAATACCAATCTAC			II	
0.1205034	cg23583501	cg23583501	31753327	CAACCTCTAAATCACCTTTCTA			II	
	cg23586272	cg23586272	54606442	TTCTATTAAAATAAAAAACTTA			II	
-0.112175	cg23588049	cg23588049	51692442	AAAACTTACTTACRTCCTCCRT			II	
0.2996596	cg23590389	cg23590389	65610329	ATACATC	52657479	ATACATC	I	A
-0.167393	cg23591463	cg23591463	18644450	AATACAAAAAACACTTTTTCA			II	
0.1143503	cg23591609	cg23591609	65720370	AAAAACATAATAAATTAAACC			II	
-0.116662	cg23595304	cg23595304	11609369	CATAAATACTTCTCTTTAAACC			II	
-0.157434	cg23596123	cg23596123	24660376	CCTTACAATTTTTTCAAACTTC			II	
0.1018863	cg23603995	cg23603995	14792375	TCCTTACCTACTAACTCCACCT			II	
-0.157871	cg23610373	cg23610373	27760360	AAACAAA	14661413	AAACAAA	I	C
0.1211841	cg23611697	cg23611697	50719487	CAACAAAATCTTCTAATAATA			II	
-0.103578	cg23616046	cg23616046	53728375	TCATTAACTATACRAACTATCA			II	

-0.106449	cg2362236(	cg2362236(	15693488	ATAACAC	67650322	ATAACGC	I	A
0.1155933	cg2363017(	cg2363017(	14642480	CTATTCCCCCTATCAAATAAA/			II	
-0.10012	cg2363132]	cg2363132]	50666408	ACTCTATATACCRCTATCTTCT			II	
-0.11833	cg2363434(	cg2363434(	45738341	CAAAAA/	24724448	CGAAAA/	I	C
0.2063085	cg2363657]	cg2363657]	41790318	AAATAAATTTTCAATATTTTCC			II	
-0.121689	cg2363804(	cg2363804(	54804489	AATCAATTAACATATTAATTA(			II	
-0.106587	cg2363864(	cg2363864(	73647450	AATATAC	51656335	AATATAC	I	A
0.1055954	cg2364227(	cg2364227(	48642510	TTCTCCAACCACACTAACCCC]			II	
0.1955	cg2364908(	cg2364908(	27711488	AAAAACC	29791450	AAAAACC	I	T
-0.102764	cg2365608(	cg2365608(	49691371	TCAAAAA	66651389	TCAAAAA	I	T
0.1162818	cg2365905(	cg2365905(	48605322	ATTCCAAAACTACTAATCCCT/			II	
	cg2366438(	cg2366438(	50730307	AATAAATTTAAAAAACACTAT(			II	
	cg2366477(	cg2366477(	29803410	CTATTCAAAAAAATACAACCR'			II	
-0.116488	cg2367063(	cg2367063(	39727415	CCACCTC	71635485	CCGCCTC	I	A
0.104049	cg2367339'	cg2367339'	56774429	AAATAAAATCAAAAAACTTAA			II	
0.1045024	cg2367934(	cg2367934(	31602493	AATAAATCATACAATATCCAC.			II	
0.1067154	cg2367937(	cg2367937(	57767364	CCAACCAAACCCAAAAAAACA			II	
-0.118741	cg2368174(	cg2368174(	65620344	ACCAAAC	25792325	ACCAAAC	I	A
-0.123984	cg2368186(	cg2368186(	25694317	AAACAAAAAAATTTACAAAAC			II	
-0.114733	cg2368301(	cg2368301(	66739327	ACTCCAA	21636467	ACTCCGA	I	T
0.1818705	cg2368380(	cg2368380(	72631320	TCTATAAACATTATCACCTACT			II	
0.2450945	cg2368743(	cg2368743(	65801402	AACCAAAATTACCAAAATCAA			II	
-0.119259	cg2369141(	cg2369141(	68601485	AACCTATTTCCATTTCAATAAT			II	
0.1914025	cg2369449(	cg2369449(	15604455	CTCCTAATAAACCCRCTACCCF			II	
-0.269477	cg2369827]	cg2369827]	70810465	ACTCCTACCATCTCTTACRACT			II	
0.1148244	cg2370515(	cg2370515(	51728396	AAAACCTCACAACCAACCAAA			II	
0.118448	cg2371201(	cg2371201(	41796445	AACTACA	17703398	AACTACG	I	C
-0.14912	cg2371390(	cg2371390(	53693410	ACCTAAAAAAATACRATCAAA			II	
-0.104132	cg2372707(	cg2372707(	29693481	ACACAAATCCAAAAAAATCTT			II	
0.108564	cg2373060(	cg2373060(	11773436	TCACCAA	10627324	TCGCCAA	I	A
-0.107721	cg2373262(	cg2373262(	27652365	CTTCCCAACATCCAAACCCAC.			II	
-0.144422	cg2373339(	cg2373339(	35610394	AAACAAATAACAACCCCTTAA			II	
-0.109582	cg2373719(	cg2373719(	63690442	ATATTAA	30723353	ATATTAA	I	C
0.1380024	cg2374223(	cg2374223(	56640382	CTCATCACTAATTAAAACRCC(			II	
0.1378801	cg2375882(	cg2375882(	64672430	TCTACAA	66743443	TCTACAA	I	C
0.1073673	cg2377222(	cg2377222(	52666371	AACACTAACTCAAACCTATCT/			II	
0.167632	cg2379321(	cg2379321(	10789310	TCCCCCA	53791392	TCCCCCA	I	A
0.1423491	cg2379333(	cg2379333(	67739465	TCATCAACATTTTATCTCTCAA			II	
-0.290078	cg2381248(	cg2381248(	14714457	ACATTTTCCCAAAAATCCAAA(			II	
-0.149912	cg2381339(	cg2381339(	59678432	ACAATTAAACCAATTATTTAA]			II	
0.1278294	cg2381902'	cg2381902'	70699431	ACTTAAAAAACCTTTAAAAT.			II	
0.2120784	cg2382795(	cg2382795(	64759481	CAACCAC	60656358	CGACCAC	I	A
-0.134105	cg2382868(	cg2382868(	49800321	AACRATAAAACCACAACCACT			II	

	cg2383589	cg2383589	64778312	TTCTAAC	28624444	TTCTAAC	I	A
0.2591873	cg2383592	cg2383592	36600420	TAATAACRTCAAAAACCTCAA			II	
0.1027181	cg2383800	cg2383800	29808339	TAAACCCRACATTAACATTTC			II	
-0.201323	cg2385386	cg2385386	46628393	AAAACAC	19687406	AAAACGC	I	C
-0.117015	cg2385539	cg2385539	19709465	AAAAAAAAATCRAAACATCTA			II	
0.1697864	cg2385661	cg2385661	37808387	CAATTATTACTATCAAAAACA			II	
0.1563925	cg2386367	cg2386367	43604369	TCCATAAAACTAACTTTACTCA			II	
0.1105232	cg2386420	cg2386420	57781427	ACTTTAAATTATATAACCTCCR			II	
0.1400386	cg2386923	cg2386923	28782332	TAAAAACATAACTTTACACAC			II	
0.1145879	cg2387399	cg2387399	17694510	TACAACCAAAAATAAAAAACT			II	
-0.202617	cg2387614	cg2387614	63806393	AACTAAA	64756379	AACTAAA	I	T
0.123224	cg2387826	cg2387826	60638333	AATCCTCATTCTATACCTTTAC			II	
0.1008308	cg2388192	cg2388192	30680503	ATAAAAA	42733395	ATAAAAA	I	A
0.2019672	cg2388783	cg2388783	48673495	CCCAATTTAAAACCAATCATA			II	
0.312647	cg2389276	cg2389276	48640474	TAAACAAAAACAAAACCAAAA			II	
0.1114869	cg2389543	cg2389543	67722339	TTAAACTAAAATAAAAACCAA			II	
0.1362621	cg2389952	cg2389952	21750485	TTAATAACAACRCCTCATCTTC			II	
0.3504651	cg2390578	cg2390578	48748494	AATCAAAAAACTCTTCCAAAA			II	
-0.112449	cg2390590	cg2390590	35718450	ATAAAAA	59743342	GTAAAAA	I	A
0.1105111	cg2391045	cg2391045	12614492	CAAAAACTAAACAATATTTAA			II	
-0.106022	cg2391552	cg2391552	32806360	ATCTTTACTATATTTATTTTCT			II	
-0.10389	cg2391831	cg2391831	36794403	AACAACCTTACAATAACTTCC			II	
-0.101648	cg2392437	cg2392437	63705465	ATTATAT	47704307	ATTATAT	I	A
-0.144466	cg2392643	cg2392643	69633370	TCCAAAA	54786464	TCCGAAA	I	C
-0.100537	cg2392816	cg2392816	56691490	TAACACT	34772365	TAACGCT	I	T
-0.118118	cg2392876	cg2392876	30742476	CCACAAA	40630333	CCACGAA	I	A
	cg2392903	cg2392903	13644375	TAAAATCTACCCAACRACAAA			II	
-0.115205	cg2393031	cg2393031	52704340	TAAAACRAAACRATTAAAAAC			II	
0.1430107	cg2393729	cg2393729	72720393	ATAACRTTTCTACATAAAAAA			II	
-0.19066	cg2393900	cg2393900	38746305	AAAATCA	51725359	AAAATCA	I	C
-0.114914	cg2394271	cg2394271	16778433	CTAAACC	34674323	CTAAACC	I	A
0.360688	cg2394765	cg2394765	71638364	CCCRAAATTCCCCAACCTATA			II	
-0.135437	cg2394839	cg2394839	44670393	ATAACCTAACRTTCAAATTAA			II	
0.1059487	cg2394881	cg2394881	37602467	ACCTTAA	74744491	ACCTTAA	I	A
-0.106509	cg2394899	cg2394899	39790306	AAAAACC	12633348	GAAAACC	I	C
0.187797	cg2395870	cg2395870	47736360	CRTCRTAAACRTACACCAAAC			II	
0.1175073	cg2395977	cg2395977	61661388	TCTTCCCTCATATTTATTTCAA			II	
0.1299093	cg2396636	cg2396636	22690431	RCRCCTAAAACAAAACCAAAC			II	
0.1024268	cg2397118	cg2397118	54750474	TTACTATTTCCCAAACCTTTTA			II	
-0.115086	cg2397447	cg2397447	63659338	AAAACCAAAATAAAAATCTAA			II	
0.1659187	cg2397698	cg2397698	29800510	AACRTCTCTATTAAAAAATAC			II	
0.1014723	cg2398170	cg2398170	22734320	AAAAAATCTACTTCCCAACAA			II	
-0.117035	cg2398537	cg2398537	59733300	TAACAAT	33766476	TAACGAT	I	T

0.2066036	cg23988024	cg23988024	32618471	CACCAAA	42732388	CACCGAA	I	A
-0.130626	cg24000218	cg24000218	65621380	TCCCCAA	39740461	TCCCCAA	I	C
0.2568897	cg24007926	cg24007926	29756506	AAACAAA	46755377	AAACAAA	I	C
0.1079503	cg24009067	cg24009067	72650436	AAAACRACAAAACATCCRAAA			II	
-0.107308	cg24013589	cg24013589	26810484	CTCTAAATAAAAACATCCCCTC			II	
0.2407884	cg24014558	cg24014558	44688390	TCTATACTAACTTCRCCAAAAA			II	
0.2240797	cg24016855	cg24016855	26718464	TAAAAAACTAATCCCACACCTA			II	
0.1251823	cg24018148	cg24018148	73806350	AACTCCTAAACTAAAATCTTAC			II	
0.144685	cg24032445	cg24032445	17682431	CCTAAACTAACCCACAAACAA			II	
-0.104399	cg24033103	cg24033103	32630505	AAAAACC	22792481	AAAAACC	I	C
-0.121738	cg24033122	cg24033122	13790358	AAACTCTAAAAAAACCRAAAA			II	
	cg24033503	cg24033503	12682383	ACAATCA	13644361	ACGATCG	I	C
0.2342547	cg24036791	cg24036791	12614397	CAAAAAA	47768375	CAAAAAA	I	A
-0.101315	cg24046087	cg24046087	53665466	ATACAAA	59795326	ATACAAA	I	A
0.1246463	cg24046616	cg24046616	72694474	TAAACCTAACCATAAAAAAAT			II	
-0.152976	cg24051234	cg24051234	52757446	CTCTACTAACTTTAAACCAAAT			II	
-0.114375	cg24051749	cg24051749	70679390	AACCAAAATTAATCAAAACCT			II	
0.207155	cg24057558	cg24057558	71674454	CRACCTCTAAAAATAAACCAC			II	
-0.121165	cg24060917	cg24060917	24667399	CTATATCCACATCAAATCTCCC			II	
0.3124983	cg24062389	cg24062389	70637368	AAAAAATACCAACTTTAAAAT			II	
-0.153278	cg24065641	cg24065641	25672361	CCATCCTATTTATCTCCTTTTCA			II	
0.1003518	cg24076588	cg24076588	31766373	CTACAACTACAACTCTAAATTC			II	
0.1220903	cg24076747	cg24076747	22733497	CCTAATATATATTACAAATATC			II	
-0.330413	cg24077401	cg24077401	42744339	RAACTCCTAACCTCAAATAATC			II	
-0.16159	cg24080129	cg24080129	67640506	AATCAAAAATCTTTATTTACAA			II	
-0.155763	cg24082680	cg24082680	51756305	TCAAATTATTTTCAAACATTAA			II	
-0.15482	cg24084927	cg24084927	47735449	TCTTTTTAAATAAAATCTCACT			II	
-0.150233	cg24097094	cg24097094	63650427	ATACTAATCAAACAATCACAA			II	
-0.103163	cg24100293	cg24100293	62648337	AATTACTATTACTTCTTTATCA			II	
-0.168524	cg24100357	cg24100357	38660364	ACTCCCA	59766507	ACTCCCG	I	A
0.114746	cg24100841	cg24100841	54650349	TACATAACCRAACTATCTATAA			II	
0.1413137	cg24103044	cg24103044	31723420	ACCCCACCCCAAATAATACAA			II	
-0.242505	cg24114014	cg24114014	54677411	AATAACC	59640472	AATAACC	I	A
0.1170067	cg24115019	cg24115019	64600307	AAACAACAAACCCTCTTCCAA			II	
0.1072057	cg24116380	cg24116380	18614375	AAAATATCCTTCTCCCATCTAT			II	
-0.101098	cg24117504	cg24117504	56702336	ACTTTTTTCCTACTATACACAA			II	
-0.114441	cg24120210	cg24120210	27697486	ACAAAAA	71668435	ACAAAAA	I	A
-0.121263	cg24126849	cg24126849	70700395	AACTTAA	37676493	AACTTAA	I	C
-0.145249	cg24127414	cg24127414	42665471	AACTACRTAAAATTTTACATTA			II	
-0.115252	cg24130284	cg24130284	41607326	RTTCTAAACTACCTAAATATAA			II	
0.119629	cg24134182	cg24134182	47714360	AAAATAATACAATTACTTTAA			II	
0.1881254	cg24139874	cg24139874	59650333	ACTCTTCTCTCTTTAAAAAACA			II	
-0.164774	cg24142775	cg24142775	29730323	TTTCTAA	54661507	TTTCTAA	I	T

0.2370199	cg24154853	cg24154853	54680357	AAAACAT	72777479	AAAACGT	I	C
0.1151772	cg24156181	cg24156181	35765340	AACCTCAATCCTAATCRCATTT			II	
0.1416696	cg24158878	cg24158878	73610478	ACCAAATATCAAAATAAATAA			II	
-0.1313	cg24160421	cg24160421	41739424	ATCACTACAAAATATTTACCCT			II	
-0.205138	cg24166916	cg24166916	13752399	AACCTTAAAAAACTAAAAATT			II	
0.1280712	cg24169486	cg24169486	47650434	AACTCTTATAAACCACTAATAT			II	
0.1161285	cg24170085	cg24170085	55767342	CTCTAAA	20663507	CTCTAAA	I	C
0.132541	cg24181174	cg24181174	60718359	TAACCAAAAACATCAAACAAA			II	
0.1201942	cg24181662	cg24181662	72677445	ATAAACAAAACCCTAAAACAT			II	
0.1426881	cg24183504	cg24183504	47684365	CRTTTTAAACACCTTATATTTC			II	
-0.102058	cg24184022	cg24184022	22600456	TCTCCAC	71690340	TCTCCGC	I	A
-0.128428	cg24185656	cg24185656	56687360	CTTTACC	55750345	CTTTACC	I	A
-0.130815	cg24192559	cg24192559	54676328	CACTAAT	72666434	CGCTAAT	I	C
-0.115823	cg24192660	cg24192660	40765501	ACAAAAT	62705329	ACGAAAT	I	A
0.132902	cg24199384	cg24199384	29724343	TTATAATAATTTATTACAACAA			II	
0.1190358	cg24205048	cg24205048	22657328	CATTTAAAAAATAATCACCTCC			II	
0.1449503	cg24208588	cg24208588	52723466	AAAAAACTAATTATCTTTTAAA			II	
0.1163542	cg24210810	cg24210810	37769364	TTATACACCAAACATCTATAAC			II	
0.1020708	cg24233679	cg24233679	60729308	AAAAAACCTCRAATATCAAAA			II	
0.1223294	cg24238409	cg24238409	13672463	CTACACATTTCAAATTATACAA			II	
-0.120229	cg24264239	cg24264239	51699370	ATTATTAACTAAAATCCAAAA			II	
-0.169577	cg24265722	cg24265722	30724305	TAAAAACCTAAAAACCTAAAA			II	
-0.101063	cg24280832	cg24280832	57670475	CTAAAAA	16687490	CTAAAAA	I	A
0.1071697	cg24284460	cg24284460	16800439	TTTCCRAAAAAATATCTTAAAA			II	
-0.247938	cg24284539	cg24284539	18760487	TCTCAAA	54751387	TCTCGAA	I	A
-0.113005	cg24287218	cg24287218	17713396	CAAAACTATAATCTTAAAAACC			II	
-0.120173	cg24289452	cg24289452	30605485	AAATACA	63790501	AAATACA	I	A
0.1072662	cg24296187	cg24296187	21741468	CTATATTTTTCAAACTCAAATA			II	
0.2614737	cg24299087	cg24299087	38674411	CAAAAATTAAATAAACAACAC			II	
0.1809598	cg24299306	cg24299306	44711379	AAATAAAATACCCATTAAATTA			II	
0.10316	cg24304617	cg24304617	73697392	CCTTTTTAACAATAACCTAAAA			II	
0.519186	cg24309769	cg24309769	31759311	ACCRATACAAACCCTAACCAA			II	
0.1224775	cg24311643	cg24311643	56688344	TACCACATAATCCRACAAACTC			II	
0.1001408	cg24312520	cg24312520	27670303	TTTTCTAACTTTATTTTAATTTC			II	
0.1494268	cg24320034	cg24320034	59641444	CCTCCTA	47739385	CCTCCTA	I	C
0.1662725	cg24321971	cg24321971	62765508	TCAACRCCTATAAAATAAACA			II	
-0.130968	cg24324837	cg24324837	15742383	AACTACC	13698310	AACTACC	I	T
-0.113582	cg24325991	cg24325991	31734427	AAACTAAAACTTTCCCAACTCA			II	
0.1973034	cg24330456	cg24330456	40790492	ATACAAA	71730422	ATACGAA	I	C
0.1206515	cg24343430	cg24343430	28716473	AATTAAAACCAAAAATATATA			II	
-0.167129	cg24355182	cg24355182	71711502	AACAAAA	12653509	AACGAAA	I	C
0.187304	cg24359926	cg24359926	20672497	AAAATAAACTAACTACAAAAA			II	
0.1184008	cg24360871	cg24360871	71656418	TAATAATATTCTAATTCACRCT			II	

0.1382847	cg24363393	cg24363393	31666380	TAAATATCACAACRCCRAAAA			II	
-0.108406	cg24363621	cg24363621	48790407	AAAAAAATTACRTTAAACCCT			II	
-0.106169	cg24367840	cg24367840	48762444	CATAAAA	37767480	CATAAAA	I	C
0.1375162	cg24367957	cg24367957	47744433	ACTTAAAACACTTAAAAATAA			II	
-0.110148	cg24384244	cg24384244	27644355	AAATACT	36635317	GAATACT	I	A
0.1069801	cg24392939	cg24392939	57755443	CTCTTTACACCAATATCCTATT			II	
-0.143861	cg24393317	cg24393317	64658453	AAAACTC	28662378	AAAACTC	I	A
-0.122935	cg24399349	cg24399349	68718419	TATCCCT	55710367	TATCCCT	I	C
0.1096056	cg24406240	cg24406240	67648488	CAATAAAAATTTCACAATCTA			II	
0.1284152	cg24408200	cg24408200	44687303	CTAAAAATAACAATAACCATC			II	
-0.40997	cg24417155	cg24417155	72680508	AAAATACTTTCTATTATCCCTA			II	
0.1061104	cg24419602	cg24419602	59679345	AAATAATTTTTCCTTCTCTAAA			II	
-0.129184	cg24421906	cg24421906	43631335	CTAAATCCAAACCCRCAACTA			II	
0.1055688	cg24425972	cg24425972	73741498	ATAAATTATCTTCACTAATTCA			II	
0.1227915	cg24439686	cg24439686	53698457	AAACTACACRAAAACRCRCCT			II	
-0.191116	cg24440658	cg24440658	20731321	CAAAAAA	52792477	CGAAAAA	I	A
-0.191916	cg24450112	cg24450112	52684506	CRATCTTACCAAATATATCTAA			II	
0.3149379	cg24451872	cg24451872	39659345	TAACAAAAACAACTCCTTTTA			II	
-0.225361	cg24452451	cg24452451	14612502	ATACTCA	58778375	ATACTCA	I	A
0.1281903	cg24457118	cg24457118	23806421	AAAAATATAACCTACCAATAA			II	
-0.120346	cg24460126	cg24460126	22710467	AAATTTA	12605372	AAATTTA	I	C
-0.105014	cg24461304	cg24461304	37723323	TCTATACCTCAATTTCCTCTCT			II	
0.115118	cg24464500	cg24464500	48775334	ACCTACAAATTTTAAAACTAA			II	
0.1697233	cg24465329	cg24465329	60633364	AAAAAATCAAAATAAAAAATC			II	
0.2809118	cg24470466	cg24470466	22782397	AATACACTTTCTTTCCCACTCT			II	
-0.162027	cg24471210	cg24471210	26675310	AACCAACRTAAACAACAAAAT			II	
-0.174173	cg24476033	cg24476033	58697331	AAAAAAA	28755447	AAAAAAA	I	T
0.1618155	cg24478129	cg24478129	64749312	AAAAAAAAATCACAACCTAAA			II	
0.1265233	cg24479382	cg24479382	54699405	AATATATATAAAAAAACACTA			II	
-0.394731	cg24485927	cg24485927	50692366	AAATAAAACAAATACRAACTA			II	
0.1021696	cg24494491	cg24494491	27809338	CCACACCTAATCACCAATAAC			II	
-0.218957	cg24506221	cg24506221	61722487	ACCTCCA	69724377	ACCTCCA	I	C
0.1020429	cg24507760	cg24507760	68607444	AACAAAATAACCAAAATATTC			II	
-0.330506	cg24508426	cg24508426	66660332	TAAAAAC	36656493	TAAAAAC	I	A
0.1805693	cg24522768	cg24522768	34629506	CAACCTCTAAAAACACRTCTA			II	
-0.116776	cg24527098	cg24527098	19792331	CTTAAAACCTAATACCCAACC			II	
-0.114029	cg24533097	cg24533097	71673405	TTCACAATTTAATAAAAACTA			II	
0.1136181	cg24536349	cg24536349	21699463	TACTCACRCTAAAAAAACCCT			II	
-0.145725	cg24536782	cg24536782	11672327	ACAAAAA	35767456	ACGAAAA	I	A
0.1203994	cg24539599	cg24539599	41691439	TTAATCCACACTACAAACCTA			II	
0.1091792	cg24541550	cg24541550	40673371	CCRACCAACTAACCTCAAAAC			II	
-0.217508	cg24550212	cg24550212	45679347	AACAACA	47717394	AACAACC	I	A
0.1380206	cg24556754	cg24556754	33642301	AATCAACTACCTACTAAATAA			II	

0.1463248	cg2456769	cg2456769	42736321	TCACAAAAAAAACTACTTAAC			II	
-0.121939	cg2457111	cg2457111	59631496	RTAAACTAAAACCACTCCCAA			II	
0.2178702	cg2457759	cg2457759	35619362	ACCCTTACCAAACTAAATCAC			II	
-0.107547	cg2458541	cg2458541	12692438	AACAATC	59677393	AACGATC	I	C
0.1233577	cg2458620	cg2458620	21656447	TTTCAAAATCAACTCTACCAC			II	
0.1444506	cg2458718	cg2458718	39810504	TATAATAACTCATACTTTAAA			II	
-0.106306	cg2459252	cg2459252	70651325	AATCCAA	40737350	AATCCAA	I	A
0.1000795	cg2459257	cg2459257	21710401	TATTTAATAAAACCTAAAAAC			II	
-0.129252	cg2459391	cg2459391	61624328	AACAATCRAACTAATTTTACA			II	
0.1017056	cg2459463	cg2459463	52750506	TAAATCAAAAACAAAAACRTC			II	
0.1648728	cg2459782	cg2459782	35631360	ACACCTA	74648484	ACACCTA	I	C
0.100055	cg2459970	cg2459970	30746490	AACAAAAAAATCTAATTTCAA			II	
-0.124567	cg2460785	cg2460785	52763510	TAATTTC	52626476	TAATTTC	I	C
0.1298109	cg2460910	cg2460910	50675301	AATAAATTTACTACACCAAAC			II	
0.1686951	cg2461395	cg2461395	61737472	CCATTATTCCAATACTAAAAT			II	
0.1040105	cg2462598	cg2462598	22730469	RATACCRAAACAAAATCRCAA			II	
0.1024079	cg2462923	cg2462923	16694492	AATCTCAAATATATCTTTATA			II	
0.2779493	cg2463633	cg2463633	22607313	AAAACA	74769447	AAAACA	I	A
0.1282465	cg2463696	cg2463696	10804440	ATATAAA	12717471	ATATAAA	I	A
0.1731954	cg2463704	cg2463704	28656357	AAAAACC	39746472	AAAAACC	I	T
0.1056609	cg2464838	cg2464838	31751309	TTACCTTCCCAAACCCRTAAA			II	
-0.105223	cg2464871	cg2464871	71797347	AAAAAACACAAACCTATCCCR			II	
0.1764359	cg2465091	cg2465091	65768461	AACTATATAATCCTATAAACT			II	
-0.213338	cg2465261	cg2465261	52635432	ACTACAC	74687433	ACTACGC	I	C
0.1089135	cg2465570	cg2465570	20762315	AACATAAATTCTATAATCAAT			II	
0.1143229	cg2466116	cg2466116	59699382	AACTAAATCCTACACAAATTT			II	
-0.129911	cg2466123	cg2466123	30641453	TAAATAT	35761407	TAAATAT	I	A
-0.101284	cg2467045	cg2467045	28689388	ACTAAAA	55775453	ACTAAAA	I	C
0.1026502	cg2467277	cg2467277	55760423	CACAAAA	48672464	CACAAAA	I	A
-0.107984	cg2467450	cg2467450	11728364	ATAAAAC	29619326	ATAAAAC	I	C
-0.158532	cg2468063	cg2468063	11688465	TTAACTT	71701313	TTAACTT	I	T
-0.135427	cg2468064	cg2468064	12746311	TATCTTTACAAAAACAAAACC			II	
0.1944229	cg2468074	cg2468074	27802424	AAAAAACAATATAAAAAATAT			II	
0.1339304	cg2468655	cg2468655	68782416	AAATCTCTTTTACTTTAAAAA			II	
0.1045592	cg2468655	cg2468655	36667388	TTACTCTATCATATAAATAAA			II	
0.1110779	cg2468780	cg2468780	48707310	ATTACAACATACCTCACAACT			II	
-0.257072	cg2468902	cg2468902	23719446	CCAAATAACCTACAATTAACR			II	
-0.144439	cg2469009	cg2469009	30685388	CAAAAAC	12747325	CGAAAAC	I	A
-0.212084	cg2469116	cg2469116	23766456	TTATCAA	54758300	TTATCAA	I	A
-0.164017	cg2469133	cg2469133	45715405	TTACCCTTTATCCTATCCCTTA			II	
-0.128493	cg2469355	cg2469355	47721303	CTACTCACAATTCAATTATCA			II	
0.5347426	cg2469483	cg2469483	20694418	CAAAACCCTACAATTATAAAA			II	
-0.136826	cg2469743	cg2469743	54700308	AATATAAAATATCAAAAAAAA			II	

0.1354762	cg2470422(	cg2470422(	54634418	TTTTTTCTACCCTACTACAAAT			II	
0.1026954	cg2470748(	cg2470748(	30779434	ATTATCC'	29621508	ATTATCC'	I	A
-0.132394	cg2470757:	cg2470757:	37737351	ACTAAAA	22726360	ACTAAAA	I	C
-0.135621	cg2470900l	cg2470900l	60731329	AACCTCA	16657340	AACCTCG	I	C
0.104286	cg2472105(	cg2472105(	28716403	ACTAAATTCACTATATAAAAA(			II	
0.2778124	cg2472257.	cg2472257.	50656303	CAACAACAAATTCTACCCATA.			II	
0.1537847	cg2472552:	cg2472552:	32785398	AAAATAAACRTATAACRCTAT.			II	
0.1942942	cg2472983!	cg2472983!	59719371	ATACTTCTACRACTCAAAACA]			II	
0.1317488	cg2473673/	cg2473673/	60699433	CTACAAACTAAAAAAACTATC.			II	
-0.120288	cg2474051:	cg2474051:	19624417	ACAAAAA	74651303	ACAAAAA	I	T
-0.173014	cg2474936:	cg2474936:	39680485	ACAAATT	18799420	ACGAATT	I	A
0.1300964	cg2475309/	cg2475309/	74793327	ATATCCCACCCCCAAATATTA/			II	
-0.139054	cg2475376(	cg2475376(	10639318	ATACAACAATACAATAAAATA			II	
0.1637828	cg2475513.	cg2475513.	39620346	TAAAATACTCATTTCCAAATA/			II	
0.1055082	cg2475545!	cg2475545!	24624480	CAACAAA	55695461	CGACAAA	I	C
0.3936825	cg2476058l	cg2476058l	69733370	ACATTAAAATATAATACAATA.			II	
0.1148415	cg2476578:	cg2476578:	52708465	CACRACTAAATCATACTCCRC]			II	
0.2873369	cg2476938l	cg2476938l	24754359	AATAATACCAAAAATCTAATT(			II	
0.127893	cg2478197:	cg2478197:	32606446	ACCTCAATTAAACATATAAAA'			II	
0.1435989	cg2478617/	cg2478617/	41677319	CCTCRTCCATCTACTTAACCAA			II	
0.2729481	cg2478759:	cg2478759:	42792334	RCCAATACCTAAAAAACATAA			II	
-0.138043	cg2479121{	cg2479121{	29765492	CATCTAA	56755477	CATCTAA	I	C
-0.134802	cg2479664/	cg2479664/	22726355	AAAAAA(	17683424	GAAAAA(	I	C
-0.217953	cg2480022(	cg2480022(	64678343	TACTACAACTATAACRACTCA/			II	
0.1334046	cg2480926!	cg2480926!	50603507	AATAAACACCCAAATCAATTA.			II	
0.110381	cg2481073:	cg2481073:	41786312	CTCATAA	59756367	CTCATAA	I	A
0.1080827	cg2481926(	cg2481926(	15679456	TATAAAATAAAAACAACTAAT			II	
-0.105174	cg2482067:	cg2482067:	50789471	AATAAAA	18647489	AATAAAA	I	T
0.1124521	cg2482437l	cg2482437l	12641307	TCAAACRACAACCRTCACAAA'			II	
0.102296	cg2482447:	cg2482447:	46719372	TCTAAACAAAAACACTACTTA/			II	
-0.153702	cg2482533/	cg2482533/	24658347	AAAACAA	46787365	AAAACGA	I	T
0.1329395	cg2483439/	cg2483439/	10618304	ACCATATATATTCACTAAAAA'			II	
-0.379824	cg2483682(	cg2483682(	22686440	TACAACA	17725391	TACAACA	I	A
-0.158539	cg2483806:	cg2483806:	38702310	CAAAAA(	31642452	CGAAAA(	I	C
-0.111748	cg2483831(	cg2483831(	27744377	CCCATTTTCCTTCTCTCTTTAT/			II	
0.1059957	cg2484593:	cg2484593:	71800302	CCACATCCTAAACATAAACAA'			II	
0.2096146	cg2484600!	cg2484600!	16757458	ATAACCCCTAACCTTCCAAAT(			II	
-0.207189	cg2484784(	cg2484784(	57762353	TAACCAT	62768401	TAACCAT	I	A
-0.13473	cg2485638:	cg2485638:	28806483	CRAAACTTACRTCAACRACTA]			II	
-0.173005	cg2485667:	cg2485667:	25747460	ATATTCACCRACAAATTACTA/			II	
0.1124531	cg2485922(	cg2485922(	69663383	TCTTTCTATAACCAACTAAAA(			II	
0.1529288	cg2485964{	cg2485964{	12639454	TCAATCTCAAAATCCTAAATA/			II	
0.1416759	cg2486139!	cg2486139!	16740353	ATAAAAATCTCTACTCAATCT/			II	

0.1026492	cg24861747	cg24861747	28663414	ACCAAAAACRACACTATATTA			II	
0.1247197	cg24865482	cg24865482	48699411	ACCTCATATAATTATCRTAAAA			II	
0.1143962	cg24866764	cg24866764	29671350	CCAAAATAATACATCTACAAA			II	
-0.204822	cg24867279	cg24867279	36727436	CAAAAAA	28609422	CAAAAAA	I	A
0.1266774	cg24870391	cg24870391	44690397	TTAACTTCCTAAAATCTAAAAT			II	
-0.43924	cg24872173	cg24872173	59689343	TCAAATCTTCAATATTCTACTT			II	
-0.180336	cg24873924	cg24873924	29785495	AACACAAATAAAACCTAAACT			II	
-0.118216	cg24877509	cg24877509	52639464	ATACACTTAAAACCAAAACTC			II	
0.1078698	cg24877964	cg24877964	66606448	CCRCTACTAATCACTAATAAA			II	
0.1247513	cg24882709	cg24882709	60703325	AAAAATATATATCCTAATACTA			II	
-0.168555	cg24883219	cg24883219	44800449	TAAAATTAACCTCTATAATAAT			II	
0.1047212	cg24886788	cg24886788	14800312	ACCATAACAATCACAAACCAA			II	
-0.10628	cg24888257	cg24888257	43667408	AAAATAA	38600394	AAAATAA	I	A
0.1889035	cg24893378	cg24893378	44810332	TTCTAAAAATACAAACTCCCT			II	
0.1518573	cg24896460	cg24896460	70722452	ATAAATATATATTTATATTCAC			II	
0.1331706	cg24897392	cg24897392	59659411	CTCTAAATCTCTCTACCAAACA			II	
0.1006311	cg24900563	cg24900563	53796500	AATCAAACTTAAACAAACATC			II	
-0.120211	cg24906015	cg24906015	47620370	ACAAACATAATTTCTATTTTCT			II	
-0.108453	cg24908198	cg24908198	58770325	TTTTCAC	17807473	TTTTCAC	I	A
0.1040526	cg24911283	cg24911283	55669478	TCATAAATAATATACTATAAA			II	
0.1428739	cg24911837	cg24911837	36781471	AATTCAATCAATAAAAACCAA			II	
0.1032126	cg24912023	cg24912023	25801336	AATCCTATAATTACTCCRATTT			II	
-0.125767	cg24912560	cg24912560	47689501	RATTTAATAACCTAAACCACC			II	
0.1014787	cg24913168	cg24913168	64614493	AAACAAA	59706366	AAACGAA	I	A
0.2746815	cg24917131	cg24917131	33787301	TTAAAAACAATTATCAAACTA			II	
-0.183655	cg24923430	cg24923430	17678303	ACATATA	63754349	ACGTATA	I	C
-0.147485	cg24923931	cg24923931	38724324	ACTCAAC	55774308	ACTCGAC	I	A
0.1053319	cg24925741	cg24925741	20633404	CRACCAAAATCATTTCACCCA			II	
0.1082575	cg24926185	cg24926185	62631457	AAAACCTATTTCTTTAAACTAA			II	
0.1131032	cg24927769	cg24927769	57729426	AACACACTAAAATTAAAATTA			II	
0.1080373	cg24933157	cg24933157	32625369	TAATATTTAAACCTAACCATA			II	
0.1014532	cg24940951	cg24940951	38653505	CAAAAATTCCATCTACAAAAC			II	
0.4240594	cg24941469	cg24941469	15772351	CTACACRTAATACTTACRAAC			II	
0.1032996	cg24941603	cg24941603	69621483	ACAAATC	10765423	ACAAATC	I	C
0.2837002	cg24946117	cg24946117	46801340	TCAATCCCAATCCCAAAAAAT			II	
0.2042333	cg24947637	cg24947637	72609377	AATAAAA	19674510	AATAAAA	I	A
-0.107381	cg24948743	cg24948743	22636366	CAACCCC	15613336	CAACCCC	I	A
-0.217292	cg24955829	cg24955829	13663506	AACTTAAACTTTCTAATACTAT			II	
0.2255872	cg24955955	cg24955955	20670468	ATCTACCTAATCTACTAAAAA			II	
0.1770026	cg24957518	cg24957518	67780307	AAAAAACCTCCCATCCCTATA			II	
-0.218721	cg24958687	cg24958687	10629423	ACAATACATTTTCCTAAAAAA			II	
-0.178882	cg24960960	cg24960960	17612325	ACCAAAA	37779367	ACCGAAA	I	C
0.1008669	cg24961286	cg24961286	72637374	TACAAATATCTTCCTTACCAAT			II	

0.1532111	cg2496636	cg2496636	49617431	CCTAAATCRTATCTAAACCAA			II	
0.1029734	cg2496886	cg2496886	19687446	TAATACTAACTATTCCTATTCA			II	
0.3433301	cg2496949	cg2496949	15736439	AAACTTTCACAACTTTATTAA			II	
-0.172073	cg2497472	cg2497472	28661329	ACAAAC	20728454	ACGAAA	I	A
0.1037127	cg2497521	cg2497521	18722400	ATTTTATAACAATATAAATAA			II	
0.1587471	cg2497916	cg2497916	44756319	RCRACAACAAAAATCACCATT			II	
-0.151917	cg2500624	cg2500624	42722446	TACAAAA	55680418	TACAAAA	I	A
-0.161482	cg2500772	cg2500772	69692414	ACCAATA	68723455	GCCAATA	I	C
0.1820286	cg2501375	cg2501375	25682361	TCCAACCTTATATAAAATTAA			II	
0.1136842	cg2501422	cg2501422	49645350	TAAAAAACRACCATTATATCT			II	
0.1029758	cg2501503	cg2501503	67632390	TCCTATTTACTCAAACCAAAA			II	
0.1295277	cg2501722	cg2501722	29608371	TTACCAT	46697347	TTACCGT	I	A
-0.230319	cg2501762	cg2501762	27739443	AAACCTA	28777354	GAACCTA	I	C
	cg2501797	cg2501797	47616304	CTATTAA	59810484	CTATTAA	I	A
-0.189537	cg2502699	cg2502699	72730386	TAAAAA	38693418	TAAAAA	I	A
-0.100786	cg2502944	cg2502944	37766331	AAAATTATTATCCACCATATA			II	
0.1041565	cg2503494	cg2503494	48777493	AAAATCCTACAAAATTCACAA			II	
-0.174083	cg2503563	cg2503563	61738421	CCACAAC	30677325	CCGCGAC	I	A
0.1700986	cg2504657	cg2504657	23646384	AAAATATACACTCCACTCACA			II	
0.1091462	cg2505940	cg2505940	42669397	CCACCCRAACAATCCTCAAAA			II	
0.1265304	cg2505969	cg2505969	61613414	TCATAATAAAAAAACCACAAC			II	
0.1189612	cg2506513	cg2506513	12621490	AATCRCCATCTAAATAATTCT			II	
-0.167298	cg2506666	cg2506666	11755371	AACTTCC	38749389	AACTTCC	I	T
-0.152017	cg2508536	cg2508536	57758371	AACAAAATCTACRCTAAATTC			II	
-0.284095	cg2509909	cg2509909	19746429	AATACTTTCCAAAAAAATTAA			II	
0.1252843	cg2510341	cg2510341	40688342	TAAAATTCCAAACTAAATAAT			II	
0.2072613	cg2510423	cg2510423	26625304	CAAAACAATCACTAAAACTAA			II	
0.1011076	cg2511418	cg2511418	24629421	CAACATTCCTACTTTCTACCCA			II	
0.1168226	cg2512356	cg2512356	37785319	CCTTTATAATCAACCACAACA			II	
0.4028873	cg2512630	cg2512630	24616484	TCTATATTACCCAAACTAATCT			II	
-0.100692	cg2512912	cg2512912	27730408	AACAAA	41638407	AACAAA	I	C
-0.164192	cg2513464	cg2513464	21646334	CTAAAATAATCTAACTAAATA			II	
0.1037503	cg2513875	cg2513875	39780366	CTACACA	11757314	CTACACG	I	A
0.1908996	cg2514021	cg2514021	60681365	TAAAACCACCTAACTTTAATA			II	
-0.117294	cg2515375	cg2515375	57695325	CRAACCAAAAACRAATATCTA			II	
-0.12378	cg2516307	cg2516307	21787372	TCCTATTCCCCTCCTATTCCAA			II	
	cg2516554	cg2516554	18794378	AACAAA	49604510	AACAAA	I	A
0.1135628	cg2517111	cg2517111	19601447	TTAAAACCAACCTATCTCTAC			II	
-0.137459	cg2517411	cg2517411	42628414	TAAATAACCACATACCTAAAC			II	
0.1047553	cg2517433	cg2517433	24627320	ATCTTTTATAACTAACTACCTT			II	
0.1210031	cg2517460	cg2517460	67697330	AACTAAA	21705410	AACTAAA	I	A
0.1080126	cg2518117	cg2518117	45797474	AACATCACAACATTCTTACTT			II	
0.1117523	cg2518614	cg2518614	71800390	TTATATTTATATATATATACAA			II	

0.1184387	cg25194822	cg25194822	29809481	TTAAAAATACTTCTTTCTTAAA			II	
-0.106855	cg25196716	cg25196716	64647415	TAATCCTAAACCCTCCRAACT			II	
-0.174074	cg25202367	cg25202367	61615420	AACCAAAACAAACCCCTTCTA			II	
-0.1028	cg25203245	cg25203245	67684364	AATTTTCCTTAAACAACCACA			II	
-0.161097	cg25205438	cg25205438	18792373	AAAAAAAACTACTACCTTCCR			II	
0.1664214	cg25217276	cg25217276	63639328	ATTAATTCTTCCTTCCTCTTTT			II	
0.134693	cg25219495	cg25219495	71607444	CRCTAAACCTAATCCACAAAA			II	
-0.204593	cg25220063	cg25220063	30798484	AAAAAAC	26616353	AAAAAAC	I	C
-0.163391	cg25222155	cg25222155	46784473	TTCACTA	11652474	TTCGCTA	I	A
-0.108481	cg25226891	cg25226891	74719354	CCAATAACCRCCTAAAAATTA			II	
0.1041216	cg25227803	cg25227803	65744387	CTCTAAAAATTCTAACCATAA			II	
0.1006835	cg25242423	cg25242423	57751408	AAAAACTCCTAAATTAAAAAC			II	
-0.133002	cg25243082	cg25243082	55626484	TAACAAA	43698461	TAACAAA	I	A
-0.24251	cg25246894	cg25246894	12721379	CTCTAAAAAAAAACACAAACC			II	
-0.187922	cg25247689	cg25247689	23714413	TCAAAACTTACCAAATTACTT			II	
0.1266949	cg25249286	cg25249286	36623314	TCTATAA	71712407	TCTATAA	I	A
0.1473061	cg25249362	cg25249362	17794346	AATAACRAAATCCAATCTATA			II	
0.1003583	cg25252017	cg25252017	36690375	TCAACAATATACTTTAAATCC			II	
0.1050739	cg25252197	cg25252197	42767506	TAAACAAAACCCAAACATATA			II	
-0.146066	cg25257671	cg25257671	57675383	AAATTCC	36743314	AAATTCC	I	C
-0.104498	cg25270899	cg25270899	54630401	ACAATAACATCTCTATCCTCA			II	
-0.110142	cg25277809	cg25277809	17760506	TAACTACTATATTCCCACTACC			II	
0.1016147	cg25281849	cg25281849	53728444	ACTACTAAAATATACATTACT			II	
0.4904483	cg25283336	cg25283336	20702363	TTTCAAAAAACAATATACACA			II	
-0.107253	cg25290227	cg25290227	65797502	CAAATACTTCTCAATTTTCATA			II	
-0.130671	cg25291250	cg25291250	32617420	TTCATTATATCACACACAAAA			II	
0.108933	cg25296938	cg25296938	39603442	ATCTTACCAACCACRCAAAAT			II	
-0.326013	cg25298319	cg25298319	46643482	ATAACCA	51700386	ATAACCA	I	C
0.1342644	cg25307332	cg25307332	20611310	CTTTAAATCTCAATTACCTCAT			II	
0.1088001	cg25312054	cg25312054	48710475	TAAAACAAAAAATCAATACTA			II	
0.1070181	cg25312970	cg25312970	14611497	ACCCCACAAACCRTCTATACA			II	
-0.123671	cg25319279	cg25319279	11772429	AACCRATTTAATTAATCTCRA			II	
-0.109729	cg25325592	cg25325592	19657413	AAAAACA	15603304	AAAAACA	I	A
0.1130057	cg25326289	cg25326289	69638390	AACTCTA	20604404	AACTCTA	I	A
-0.107483	cg25326998	cg25326998	52608434	AATACAC	46762308	AATACAC	I	A
0.1918439	cg25336892	cg25336892	12802321	TCAAACACAAACRAACAAAAA			II	
0.2491737	cg25339052	cg25339052	74719359	TAACCTACAAACTATATTTAA			II	
-0.118507	cg25340688	cg25340688	53782363	AAACTAA	38662399	AAACTAA	I	A
0.101261	cg25345738	cg25345738	41694346	TTATATCTATATCATACTTCTC			II	
0.1466835	cg25356468	cg25356468	40666356	TTAATACAAAATCRATTAAAT			II	
0.114548	cg25357666	cg25357666	67661507	AACCTCAAACAACAAACAATA			II	
-0.112338	cg25365934	cg25365934	33620336	ATAAATA	59714368	ATAAATA	I	A
0.1043493	cg25369262	cg25369262	60723452	CTAATCACCAAAATAATATCA			II	

0.1104254	cg25371267	cg25371267	46633425	ATATCTAAATAATCAATCAATA			II	
0.1700462	cg25372296	cg25372296	66787371	AATACTA	66810354	AATACTA	I	A
0.1575176	cg25375162	cg25375162	65732308	AAACTTTAAACCAACCTATTC			II	
0.122825	cg25376032	cg25376032	45623463	ATAAAAA	19763424	ATAAAAA	I	A
0.1964592	cg25381253	cg25381253	28714405	ATCTCTTCTTCATATCAAATCC			II	
0.1050662	cg25382821	cg25382821	26632370	ACTTTTCATAATCTCRAATACA			II	
-0.244342	cg25388952	cg25388952	24657323	CAAACTACAATATATATAAAA			II	
-0.100315	cg25395546	cg25395546	21733385	TCTTCCTAAACRCCRAACRCTA			II	
-0.118354	cg25399309	cg25399309	59792335	ACCCAATAACTAAAAAAAACC			II	
0.1081036	cg25400378	cg25400378	25778421	CCTAACT	42739471	CCTAACT	I	A
0.1851405	cg25408960	cg25408960	67762367	TTACTAAAAAATATAATAACRA			II	
0.1930005	cg25410789	cg25410789	19742358	ACAACTCTAACAAATATCRAA			II	
-0.114626	cg25417675	cg25417675	46678372	TCAAAAA	48657349	TCAAAAA	I	C
0.224561	cg25420101	cg25420101	65616357	AACTAAATAACTACCAATTCCA			II	
-0.130938	cg25423077	cg25423077	53686471	AAAAAAA	70602466	GAAAAAA	I	A
0.1413963	cg25424279	cg25424279	44730478	AACTTTTCATAACTCACAAAAC			II	
-0.102833	cg25425994	cg25425994	55685434	CAATACTTTTCCRACACCTAAA			II	
-0.100655	cg25432807	cg25432807	58726341	TACCTAA	73736305	TACCTAA	I	T
0.1379996	cg25434864	cg25434864	63772451	AACTCCA	59708488	AACTCCA	I	C
	cg25436245	cg25436245	60784379	TCCACAA	51657370	TCCACGA	I	T
0.108284	cg25437886	cg25437886	14705510	TAATAATAATACAATCTTTCTA			II	
0.1026028	cg25438876	cg25438876	46644374	TCCTAAAATTTTACCTAAAAAT			II	
0.1592988	cg25445671	cg25445671	38619346	TACAAATCCCAATTCRAACTAC			II	
0.1012682	cg25446460	cg25446460	25720424	CATAATAACTTATACATTATAA			II	
0.1220195	cg25450121	cg25450121	23733451	CCRACTCAAAATAACAAACTC			II	
-0.152691	cg25452165	cg25452165	58610460	AAAAAAA	10801428	AAAAAAA	I	T
0.1028102	cg25455408	cg25455408	56705368	CTTCACCTAACTAAAAACAAA			II	
0.111756	cg25462858	cg25462858	19638351	AAACTATATCTTCATCTTCCTC			II	
0.1772217	cg25467833	cg25467833	56651484	ACAAATAATAAAAATAACAAC			II	
-0.170766	cg25468618	cg25468618	69778302	AAAAACA	23627486	AAAAACC	I	C
-0.100805	cg25468928	cg25468928	31686334	ATATTTTAACAAAACATACTAA			II	
0.1092607	cg25469418	cg25469418	47711344	CTAACTC	68633335	CTAACTC	I	A
-0.169213	cg25470758	cg25470758	73769489	ATAAAAT	72647366	ATAAAAT	I	C
0.1079484	cg25481160	cg25481160	13663473	AAATCCCTATCAAATAAAAATT			II	
0.1186213	cg25483907	cg25483907	22723355	CAAAAAAAAAACCAATATTAA			II	
0.1010242	cg25488284	cg25488284	57793476	TAAAAAACATATATATCCATA			II	
0.2350157	cg25498731	cg25498731	73789377	CTAAATTTTAACTCTCCTCTAT			II	
-0.147055	cg25516803	cg25516803	41793409	TTAAAATCCCAAAATTCAAAA			II	
-0.166839	cg25531220	cg25531220	19725311	ATATAAATAACCCCATCTCCA			II	
-0.128245	cg25532201	cg25532201	26658455	AATCATC	52798333	AATCGTC	I	T
0.1548585	cg25535982	cg25535982	14607324	CTTTTATCTTTTCATTTCTTACT			II	
0.2274309	cg25536467	cg25536467	34807466	TTCTAATAACAAATAATCACCT			II	
0.1444024	cg25538571	cg25538571	64636442	CAACACAACTAACCTTCCAATA			II	

0.1357291	cg25542319	cg25542319	49701389	AATAATATAATCAAAAATAAA			II	
0.1151707	cg25542878	cg25542878	56757475	AACTTCT	22721337	AACTTCT	I	A
0.1133862	cg25544384	cg25544384	16604387	CATCRTAACAATAACAATATA			II	
0.1038274	cg25546488	cg25546488	53758452	TTTCCTTCCTATAACAAAATAT			II	
0.1117507	cg25547135	cg25547135	23714488	TACCTTTTCAAAACCATTCTTC			II	
	cg25551699	cg25551699	13644385	ATTCCCAATACTTAATACRTAA			II	
0.1176236	cg25556008	cg25556008	48631359	CRAACTTAAACTCRAATATAA			II	
-0.1022	cg25558406	cg25558406	49637374	CATAACA	11728357	CGTAACG	I	A
0.1195691	cg25568241	cg25568241	55683481	TTTCCACATTATATACACTACC			II	
-0.16542	cg25569462	cg25569462	66638340	TCAATAATTAACRCRATACCT			II	
-0.111753	cg25569480	cg25569480	54634366	ATTTTAA	66626421	GTTTTAA	I	T
-0.118544	cg25576711	cg25576711	53674496	AAAAAATTAACTACRTAATAA			II	
-0.354333	cg25576961	cg25576961	67768472	ATAAATTCCTAAAATAAAATT			II	
-0.128672	cg25584814	cg25584814	56767305	ACCCATA	31807363	ACCCGTA	I	A
-0.269567	cg25584930	cg25584930	19769332	CRAAAACCTCCAAACAACCAT			II	
0.1077788	cg25588844	cg25588844	27759392	AATATACTTCAAAACAAATTT			II	
-0.178199	cg25593194	cg25593194	33751503	CATACCCAAATTCATTCCCTAA			II	
-0.119999	cg25594826	cg25594826	27779474	ATATATTAAACTCAAATAATCA			II	
0.1547919	cg25596287	cg25596287	35611367	TCTTCAAAATTTCAAATATTCC			II	
0.1545402	cg25597117	cg25597117	58743426	ACCAAACTAAAAACCTTCAAC			II	
0.1079204	cg25597623	cg25597623	32647412	CCCTAAAAATATACCCAATAA			II	
-0.118147	cg25597745	cg25597745	43678384	TAAAAACAAAAACTCTCCCRA			II	
-0.20437	cg25598710	cg25598710	14714508	CTAACTACTATCAAAATTACA			II	
-0.199193	cg25601709	cg25601709	25670452	ACTCTCAACCTCATTTTCCATA			II	
-0.109136	cg25605313	cg25605313	43703500	CAATACTAAAAACCCTCRAAA			II	
-0.121947	cg25606780	cg25606780	13640314	AACCCAA	35797396	AACCCAA	I	C
0.1165841	cg25607216	cg25607216	47759413	AATCCTCATTCAAAAAACTAA			II	
-0.141207	cg25610515	cg25610515	14744466	AAATAAA	13797329	AAATAAA	I	T
0.148101	cg25611057	cg25611057	52649309	TTCTATT	45790361	TTCTATT	I	T
0.1075931	cg25613061	cg25613061	23731348	TTAAAAT	38682366	TTAAAAT	I	C
0.1890648	cg25614253	cg25614253	12786346	TAAAAAC	70791391	TAAAAAC	I	A
0.1379604	cg25618559	cg25618559	67639508	RAAAATATAAAACACCCACCT			II	
-0.136244	cg25618731	cg25618731	19602485	AAACAACCCTTAAACCTAATT			II	
0.1817566	cg25626472	cg25626472	56619478	AAAAAACTACCATTCCACRTA			II	
-0.167218	cg25627675	cg25627675	13775313	TCCCTAAACAAAATATATATA			II	
0.1249876	cg25633069	cg25633069	72743407	CAAAATTACTCCTCTTCACCCC			II	
	cg25635000	cg25635000	13644333	CCCTAACAACCATAAATTTTAT			II	
0.1308822	cg25636466	cg25636466	54810503	CCACAATATTATATCACCACAT			II	
0.1047102	cg25641515	cg25641515	10757490	AAAATATCTCCTCATAATCCTT			II	
0.1318529	cg25642955	cg25642955	33807370	ACCAAAACTCTAATTCCCTAA			II	
-0.102712	cg25644095	cg25644095	61724465	TCCCCRAATAAACCTAAAAAA			II	
0.1839136	cg25644478	cg25644478	35724359	CATCTCACTATTTCTCCACTAC			II	
0.3878244	cg25649515	cg25649515	25669495	ATAAAAATAAAATTACTAAAA			II	

0.1100954	cg25652751	cg25652751	25660390	TACATTCTCCCAAAATCCCAA/			II	
-0.196105	cg25655482	cg25655482	41648465	CTCCTCTATACCTTAATAACAA			II	
0.1027218	cg25659902	cg25659902	32630357	TTCTAAAAAATCTAACTTAACT			II	
0.1097613	cg25664050	cg25664050	50613420	AATAAAATTTTTATAACATCT			II	
0.192666	cg25666210	cg25666210	13665497	AAAAAAACAAAACTCTCTTAC			II	
0.1051006	cg25671242	cg25671242	26733308	AAAAAATCTCCAAACTCCAAA			II	
-0.120174	cg25674027	cg25674027	35754413	AAACCCCTTTCCTAACATCACT			II	
0.1231503	cg25674883	cg25674883	24782425	AAAAATTCAACCTATAAAACA			II	
0.1665294	cg25677394	cg25677394	43683426	TACCTAACAAATTACTACCTA/			II	
0.1080056	cg25687874	cg25687874	33636301	TATCCTTAACAAAAAAAAACAA			II	
-0.108835	cg25690958	cg25690958	18631435	ACTAAAA	50701307	ACTAAAA	I	A
0.1444655	cg25692139	cg25692139	26791369	AAATCCAAAAATAACCTAACA			II	
0.2283396	cg25692928	cg25692928	29610437	ATTCTCTAACACTCTAATATCA			II	
0.103038	cg25694790	cg25694790	66718303	TTAATCAAATACAATAAAAAC			II	
-0.108476	cg25695029	cg25695029	20753405	ATATACA	34643314	ATATACG	I	A
-0.252943	cg25697727	cg25697727	66760371	TAAAATA	35687481	TAAAATA	I	A
0.1350674	cg25701646	cg25701646	20738426	AAAAAACCCAAACTAAAACA/			II	
0.1202706	cg25705492	cg25705492	42733480	TCCATTA	11778350	TCCATTA	I	A
0.1082855	cg25707745	cg25707745	43624350	ACCCACATACAACTTTACCAA			II	
0.1240943	cg25709335	cg25709335	29620365	AAAACTCTCAATAAAAAATCC			II	
0.1217234	cg25710298	cg25710298	31786453	CTACAAACCAAATTCATACAT/			II	
0.1110531	cg25718748	cg25718748	34656411	ATTATAAAAAACCTAAAAAAT			II	
0.1807215	cg25735490	cg25735490	58601483	TAACACT	28662440	TAACGCT	I	T
-0.207898	cg25736251	cg25736251	63709335	TAACCAC	50624488	TAACCGC	I	A
-0.132158	cg25736982	cg25736982	42624311	ACAACTCTAATAAATCCTTTTA			II	
0.1134816	cg25738176	cg25738176	31778309	RATTTAACCCTCRATCRAACA/			II	
-0.105478	cg25746134	cg25746134	45616428	CTCACCC	71657317	CTCACCC	I	A
0.1480687	cg25747670	cg25747670	54620484	CAACCACRTAAATAACCRCTA			II	
0.1110699	cg25749254	cg25749254	65713432	CAAATAAAAACTTTTAATATA/			II	
-0.105481	cg25753631	cg25753631	22639435	TAAACCA	43710382	TAAACCA	I	A
0.1166782	cg25753720	cg25753720	32777378	TCAATAAACTCATACTATAAT/			II	
0.1139895	cg25755851	cg25755851	57716321	AATAACTTCAAACCTTAATTTC			II	
-0.180408	cg25757140	cg25757140	55763352	AATTACTCTTCTAATTTAATTC			II	
0.1049095	cg25763212	cg25763212	40639344	CCCTATCCAAATAAAATCTTA/			II	
0.124741	cg25765192	cg25765192	10671490	ACATTCATTCATAAAATCCAC/			II	
0.1702272	cg25771052	cg25771052	13616491	AAATACTCTTACCTCAACCTCT			II	
0.1121719	cg25771113	cg25771113	48691468	ACTTTAAATTTTTATTTTCTAC(			II	
-0.216629	cg25771854	cg25771854	36780433	ATTCATAAATAAATTAACCTCT			II	
0.1090947	cg25780024	cg25780024	73614460	TACTATTTAAATCACRTAAATA			II	
0.1569482	cg25781755	cg25781755	67767325	TTTACTTTAAATATTAAATCRT			II	
	cg25783987	cg25783987	13644438	CCTCACTTACCTTAAAAAATA(			II	
0.1073111	cg25784280	cg25784280	20699410	CATAATCCTTCCTCCCACCATA			II	
0.1444913	cg25788793	cg25788793	68742426	ACAACTCRACAATACCCRACT			II	

0.1144449	cg25790133	cg25790133	24628433	TATTCTATTCTCTTTTTAAAAC			II	
-0.144634	cg25804357	cg25804357	56611493	TAATAAATAATAATTTAAACA			II	
-0.125582	cg25817503	cg25817503	31787453	ACCTACAAATAACAAATATTC			II	
0.1038076	cg25825627	cg25825627	69600381	AAAATAACCATAATCTCCRAA			II	
0.15821	cg25827670	cg25827670	53622440	TAAAAAAACTCCAATAAAAAT			II	
0.1064329	cg25828963	cg25828963	72722475	CTTAATATTTAATCCTAACAA			II	
-0.159721	cg25832410	cg25832410	54781444	AAAAAA	42720419	AAAAAA	I	A
-0.120549	cg25832925	cg25832925	43714394	CCAAAAT	36726440	CCGAAAT	I	A
-0.109874	cg25848037	cg25848037	69774465	CCTACAA	29642457	CCTACAA	I	A
0.2035818	cg25856090	cg25856090	22744405	AACAATAACTCACTACTTAAC			II	
0.1257849	cg25873514	cg25873514	27674380	TTCAAAAAAATAATTCTAAAC			II	
-0.427604	cg25879395	cg25879395	28614346	CAAAAAACRAAACTCAATTCC			II	
0.240307	cg25880954	cg25880954	50802381	CRCCCCTCAAAAACCTAACAA			II	
-0.144562	cg25886479	cg25886479	65601307	ATCAAAA	44698438	ATCGAAA	I	A
-0.120569	cg25888867	cg25888867	16784456	AAAAATATCTACAATACCCAA			II	
0.1227987	cg25892587	cg25892587	57739357	TATTCRAACRATATTACAATA			II	
-0.11038	cg25896603	cg25896603	41801497	TAAAACT	48769415	TAAAACT	I	A
-0.142231	cg25897043	cg25897043	11768491	AATCTACCATTTCCTTCAAAA			II	
-0.195589	cg25910261	cg25910261	15796467	ATCTATAAAAAAAACAACACT			II	
0.1226353	cg25912009	cg25912009	47703395	AAAAAAACRCAAATTTTAACR			II	
0.1931987	cg25914522	cg25914522	27699304	ATACRATAAAAATCCRATATC			II	
-0.154428	cg25919922	cg25919922	37687374	CAAACAC	18734419	CGAACAC	I	A
-0.391462	cg25929399	cg25929399	14621300	AAAACACAAACTTTCCTATTA			II	
0.151659	cg25932869	cg25932869	72731368	TTCCAAC	14691388	TTCCAAC	I	A
-0.162782	cg25937216	cg25937216	64668503	TACAACT	31790314	TACAACT	I	C
0.1243055	cg25937451	cg25937451	41673508	TCAAATAAACTACCACCAATA			II	
-0.107867	cg25951210	cg25951210	33737452	AAAACAC	37624370	AAAACGC	I	C
-0.185296	cg25951717	cg25951717	14715421	CAAAATATCTATAAAAATTCT			II	
0.1060335	cg25954269	cg25954269	73614359	AATTTTAAAAACAAACTTCCT			II	
-0.157405	cg25954539	cg25954539	70697407	AAAATACTAATCAAAAATCCC			II	
0.1024307	cg25956089	cg25956089	32617505	ATCTTACTAACATACTCAAAC			II	
0.1018619	cg25956106	cg25956106	33647352	AACTTTCCATCCTAACTTATAA			II	
0.1552505	cg25959131	cg25959131	39609353	TTATCTTAACTAATCCTTCAAT			II	
0.1570581	cg25964213	cg25964213	74775451	ATAATACTACAACAACAATAT			II	
-0.141615	cg25966908	cg25966908	40606387	ACAAAAACTCCCRTATAAATC			II	
0.1485974	cg25977769	cg25977769	66729380	RAAAAAACTATAATCTCTACT			II	
-0.117816	cg25980637	cg25980637	46736398	AAAAAAC	19772372	AAAAAAC	I	A
-0.141256	cg25983380	cg25983380	67706311	CAAAAAC	71663305	CGAAAAC	I	A
-0.12471	cg25985355	cg25985355	18673434	ATACTCT	24707378	ATACTCT	I	C
0.251114	cg25985455	cg25985455	57639456	AACAACAAAATATAAACRTAA			II	
-0.141614	cg25987452	cg25987452	27789451	TTTAATA	10603416	TTTAATA	I	C
0.106554	cg25996061	cg25996061	26777302	AACCCAACCTCAAACCTTTCT			II	
0.1046607	cg25996077	cg25996077	14794347	CTTACCACTAAAAATCTAATT			II	

0.1029992	cg25997306	cg25997306	67738491	ACTCCTATATATATTTCTCTAT			II	
-0.175188	cg26000542	cg26000542	13707418	CCCAACCRCCAAACCTAATAA			II	
-0.138714	cg26000619	cg26000619	49723391	CTCAAAT	32777403	CTCGAAT	I	A
-0.126158	cg26004771	cg26004771	43686443	TAAAAAA	49758509	TAAAAAA	I	A
-0.124092	cg26013028	cg26013028	17626301	ATTCTCT	41699495	ATTCTCT	I	A
-0.101864	cg26019680	cg26019680	27660307	CTCAACA	58793389	CTCGACG	I	A
0.1005767	cg26027052	cg26027052	15694442	AACTTCACTCTCTAAAATCATA			II	
	cg26027669	cg26027669	67758452	ACCTTTA	36725493	ACCTTTA	I	A
0.1396387	cg26034313	cg26034313	51738305	TTCTCTAAATCACATCTTAAAA			II	
0.5282301	cg26035071	cg26035071	30783484	CAACTCCRCAACCAAAATCTA			II	
-0.117613	cg26035633	cg26035633	61612472	AATAACTACCTCAAAAAAACT			II	
0.1478252	cg26037936	cg26037936	22744448	CTAATCCCAACAAATTCTAAA			II	
0.2255244	cg26038514	cg26038514	11809492	AAAAAAAACAAACCCTCAATA			II	
-0.169821	cg26053291	cg26053291	18687306	AAAAACA	60664323	AAAAACA	I	A
0.1481614	cg26067096	cg26067096	71710417	CTCRCACTATATTAAAACTAAC			II	
0.1239161	cg26069044	cg26069044	12603426	TACAAATAATATTAATACAAC			II	
0.1444794	cg26069539	cg26069539	31662423	AACAAAATCAATATTAACTAC			II	
0.102263	cg26069973	cg26069973	46804344	ATTTTAAATCCCAATTTAAACA			II	
-0.17533	cg26076233	cg26076233	44657335	ACATAATAAAATCTCAATATA			II	
-0.119273	cg26077133	cg26077133	10724432	CCAAAAAAAAATCTACTCTCA			II	
0.1433165	cg26086902	cg26086902	55766497	ATTTCTC	21660459	ATTTCTC	I	A
-0.136479	cg26088294	cg26088294	60663334	TAAAAAC	73801426	TAAAAAC	I	T
0.1446812	cg26089705	cg26089705	62735412	AAATAAAACCAACAACATCTA			II	
-0.107421	cg26100986	cg26100986	30670307	RAAACACAACAAATCTTCAAT			II	
0.1169806	cg26103179	cg26103179	47663399	CRAAACATCAAATACCCTTAT			II	
0.1822625	cg26103306	cg26103306	59764449	TTACCTAAAAACTTAACAACC			II	
-0.126718	cg26114378	cg26114378	31604358	CTTAACT	39695312	CTTAACT	I	A
-0.152489	cg26115216	cg26115216	38731321	TAAAAAC	55617402	TAAAAAC	I	A
0.1186487	cg26116326	cg26116326	25741478	TCTCTTACTCTACCTTAAATCA			II	
0.1222908	cg26120902	cg26120902	64650457	AAATCATTATAACACAAATAT			II	
0.1882641	cg26124596	cg26124596	57687319	RAATAACTCAACACCCACTAT			II	
0.1076929	cg26126295	cg26126295	25720316	RCAAAAAACAACCTAAAATTA			II	
-0.103786	cg26127836	cg26127836	32679334	AAAAACATTCACRTCTAATCA			II	
-0.146363	cg26135573	cg26135573	52734438	TAAAACC	32772485	TAAAACC	I	T
-0.189673	cg26137417	cg26137417	49642328	CACCTTTCTCCCTCCCTAACAA			II	
0.1412832	cg26140475	cg26140475	42804422	TAAAAAAATTACAAACRATCT			II	
0.1019545	cg26144567	cg26144567	23648361	TATAAAC	37618434	TATAAAC	I	T
0.1207222	cg26158270	cg26158270	45735423	CTACTCCCAAAATACCCCTATC			II	
0.1170293	cg26161057	cg26161057	16681300	ATAAATTAAATAATTTTAAAA			II	
0.1702114	cg26166854	cg26166854	22682412	AAAACAATCAAAATTCACACT			II	
0.1239429	cg26169783	cg26169783	21710418	AAAACTAAAACTCTACAAAAA			II	
-0.103135	cg26170569	cg26170569	68740498	AACAACA	52687301	AACGACC	I	C
0.2528326	cg26173773	cg26173773	55714323	CAAACCA	64648378	CGAACCC	I	T

0.1508851	cg2617465(	cg2617465(	53648435	AAATACTCTAAAAACCTTCTT/			II	
0.1464899	cg2617544{	cg2617544{	67607446	ACTCCTCACTTAAACATACTA/			II	
0.1390514	cg2617704l	cg2617704l	21601334	ATTCATTTCCAACCTTCTACTT			II	
0.1429689	cg2617709(	cg2617709(	34651357	CCAAAATCACTCAATTAATAA			II	
-0.124243	cg2617940(	cg2617940(	24615388	CTTTAACTCCCCAATCAAACTT			II	
0.1528952	cg2618173l	cg2618173l	39672354	ATTCCCCAAACATAACTATTA(			II	
-0.103941	cg2618476:	cg2618476:	55672420	TTTAACTACCTTTTCAAAACTA			II	
0.1477403	cg2618862l	cg2618862l	61802303	CAACCACCAATCAAAACTCAT			II	
-0.102974	cg2619793(	cg2619793(	23737425	ATATATATATCCATATATATCC			II	
0.1018702	cg2620159(	cg2620159(	30699308	TCTCCRTAAACTACRAACCTTT			II	
0.1005002	cg2620967(	cg2620967(	24801488	CCCTAAAACRAACAAAACAAC			II	
-0.23613	cg2621172<	cg2621172<	16665412	CTCTAAACATATCTACAATTTA			II	
0.1038982	cg2622011(	cg2622011(	59617342	AAACCACRTCACCCCTCAAAA			II	
-0.4122	cg2622722:	cg2622722:	11801364	ATCACAA	70688369	ATCACAA	I	A
-0.122468	cg2622812:	cg2622812:	38777402	AATCATA	13800396	AATCATA	I	A
0.165228	cg2625158:	cg2625158:	33790464	CTTACATAAACAACCAATTCT/			II	
0.1003007	cg2626476(	cg2626476(	12612401	ACAACATAACTACAAACATAA			II	
0.1076044	cg2627630l	cg2627630l	58641425	CRTTTACATATCACCCATACCT			II	
-0.107051	cg2627632'	cg2627632'	69759301	AACTACAAATCTTCCTAAACC/			II	
-0.107978	cg2627898'	cg2627898'	57773508	RTACTAATTTACTCACAAAAA			II	
0.1210888	cg2628279:	cg2628279:	50743508	ACTAACA	35771465	ACTAACA	I	A
0.1388886	cg2628683(	cg2628683(	56743399	CATCCCT	52663310	CATCCCT	I	A
0.2770569	cg2628708(	cg2628708(	70697461	ACCTAACCCAACTAAACCCAC			II	
0.1154261	cg2628715:	cg2628715:	28777449	TTTAAAAATTCTTTAAACTACC			II	
-0.222125	cg2628752l	cg2628752l	48732370	AATACTATCTTTCTACTATTTA			II	
0.1100446	cg2629495:	cg2629495:	71770374	TACTTTCACTATACTTTATAAT			II	
-0.138707	cg2629577<	cg2629577<	27637485	TAAACTCTTAATATCATAATTT			II	
0.3007739	cg2629637l	cg2629637l	32610338	TATCAAATTTAATCCCACCCTC			II	
-0.129741	cg2631025(	cg2631025(	19693331	CTTTACACCTAATTAACTCCTT			II	
0.1195459	cg2631042(	cg2631042(	66734380	ATTAAAC	50763482	ATTAAAC	I	A
-0.148466	cg2631055l	cg2631055l	26661506	TAAAAAA	36664364	TAAAAAA	I	A
-0.102063	cg2631694(	cg2631694(	45600479	ACTAACATAAATATCCCACCA			II	
0.2025616	cg2632273(	cg2632273(	60620301	ACTAAAAACTATAATATCTAA			II	
-0.203538	cg2632379'	cg2632379'	57763307	TCATTAA	58702312	TCGTTAA	I	A
-0.135056	cg2632863:	cg2632863:	30791406	TAAAAAT	17743418	TAAAAAT	I	A
0.1377713	cg2633080(	cg2633080(	63768469	TCAAATAAACTATCTACCAAA			II	
0.1737745	cg2633451{	cg2633451{	40773309	ATTCACTAAATTTAACTAACA			II	
-0.117615	cg2634000:	cg2634000:	23653500	AAAATAA	13707336	AAAATAA	I	C
0.1143692	cg2634520:	cg2634520:	24719508	AATAAATTTCCTAAAAACTAA			II	
-0.100685	cg2635265:	cg2635265:	25639317	TTATATTCTTACCCCAAAACCC			II	
0.1329414	cg2635359{	cg2635359{	56697335	ACTCAAAAAACTCRTTCCTCC/			II	
-0.114448	cg2635575:	cg2635575:	26645385	CRCTTACTCTACRTAATAATAC			II	
0.1349538	cg2636143(	cg2636143(	26635492	AATAAATACTTAAATAAAAAA			II	

0.1811543	cg26361533	cg26361533	38608410	AAAAATAAAAATCATATACCT			II	
-0.125558	cg26368752	cg26368752	71640387	AACAAAC	16671344	AACAAAC	I	T
-0.102067	cg26373733	cg26373733	30695325	TCCTAAA	45787391	TCCTAAA	I	A
-0.112302	cg26379258	cg26379258	38675371	CACAAAA	61735303	CACGAAA	I	A
-0.341548	cg26387458	cg26387458	44610504	TTTTTAAAACAAAATCTCTCTC			II	
0.1009895	cg26388509	cg26388509	37715492	CAAACTATACAATAAAAAATC			II	
0.1742955	cg26391794	cg26391794	61735467	AAACAAATTCCCATCCAACAA			II	
-0.122617	cg26393629	cg26393629	63741303	CCATACAACCACCAATAATTTA			II	
0.182361	cg26394788	cg26394788	51750435	CCAACAC	65769395	CCGACAC	I	C
0.1214261	cg26395694	cg26395694	65681467	AAAAATCAACCCAAAAACAAA			II	
0.1206271	cg26395761	cg26395761	52649436	TACRTACAAACTTCAATAAACC			II	
-0.173031	cg26398228	cg26398228	32682322	AAAACTTATTCTATACTACCTA			II	
-0.101771	cg26406176	cg26406176	59727345	RACTCTAAAACTAAACACACR			II	
0.1754072	cg26409826	cg26409826	12685507	TCCAAAAAAATACTAATCCAA			II	
0.1117378	cg26413192	cg26413192	33663333	CTTATTAATCCCAACTCAAACT			II	
-0.106364	cg26419287	cg26419287	45633360	AAAATAATTCTTCCACAACCCA			II	
0.2589192	cg26422465	cg26422465	50734334	ACTACRAACACCRTCCATCAAT			II	
0.106645	cg26422762	cg26422762	39603443	CCAATACCTACAAAAAAACCT			II	
0.1090406	cg26429017	cg26429017	38702376	CACAAAAACTCACAAAACAAA			II	
0.1475817	cg26429022	cg26429022	34655409	AATCCRACCTATAATTTATTAT			II	
0.1396711	cg26440059	cg26440059	59636307	ACATTCAAAAAAACTCCTACA			II	
0.1158334	cg26440142	cg26440142	57664313	RCCAACCTCCTTATCCTTATCC			II	
0.1451485	cg26441877	cg26441877	71732460	CTTCATTCCAAAACCCCAAATT			II	
0.1003885	cg26444097	cg26444097	58806385	CRATAAAATACCAAAAAATTA			II	
-0.102613	cg26445985	cg26445985	44800343	ATACACAAACTCATATATACAC			II	
0.1050352	cg26450149	cg26450149	69747316	ATTTTATAAACCAACTAACAAC			II	
0.1707134	cg26450259	cg26450259	23687451	AACCAAA	51794485	AACCAAA	I	C
0.1820357	cg26451345	cg26451345	74750436	CAAAACTTATACAAATCTATA			II	
-0.154327	cg26451735	cg26451735	47622446	ACACACA	16697437	ACACACC	I	A
-0.149711	cg26456183	cg26456183	59694308	ACATTTCTTTTCCCTATAATAA			II	
-0.133356	cg26457160	cg26457160	43639448	AAACAAATTCCCRTACCCCAA			II	
0.13715	cg26458748	cg26458748	18645396	AACTCTTTTCTAACTAACTAAA			II	
0.1057395	cg26461267	cg26461267	58808317	AATTTACRTTTCATTTTCTTTT			II	
0.1065996	cg26461710	cg26461710	47652340	AATAAACCCCCATTTCTACAA			II	
0.1724367	cg26462055	cg26462055	48657505	ACTAATACCAAAAATATTACA			II	
0.1587134	cg26465155	cg26465155	22688368	ACCAACCATAATCTTACTAAA			II	
0.1290464	cg26465602	cg26465602	15791438	CAAATAA	11686361	CAAATAA	I	T
0.1949764	cg26469387	cg26469387	55800379	CAAAAAAAACTTATAAACTTC			II	
0.1186789	cg26469895	cg26469895	18752376	AAAAAACTCCAAAAACCCTTC			II	
-0.192772	cg26472636	cg26472636	51751360	CAAAAAT	39664404	CGAAAAT	I	A
-0.133967	cg26476502	cg26476502	48690435	TCTAAAACTACAACCTCATAA			II	
-0.317008	cg26477117	cg26477117	24774328	ACAAAAAACCTCTAATACAAA			II	
0.1056041	cg26494916	cg26494916	43673381	ACAATACCAACACACAAAAAC			II	

0.1390049	cg26497631	cg26497631	49677468	TCTTACAAAATTACAAAATATT			II	
0.1011625	cg26498966	cg26498966	34658301	ACCAACT	55807442	ACCAACT	I	A
	cg26500816	cg26500816	69624364	AATATAAAAACTTAAACTAAC			II	
0.1303562	cg26504263	cg26504263	69779320	ATAAAATCCAATAAACCAACC			II	
0.1794722	cg26504614	cg26504614	54705308	ACAAAAAAACCTCRCAACCTC			II	
-0.121195	cg26507422	cg26507422	19698378	AACCCTAAAATCTCATACTAA			II	
-0.12939	cg26507637	cg26507637	53661308	AACCACC	62741421	AACCACC	I	C
-0.146613	cg26512469	cg26512469	38741327	TAAAAAC	41740366	TAAAAAC	I	C
-0.189739	cg26513743	cg26513743	30608337	TAATTAAAATATCTCTCCTAAC			II	
-0.224408	cg26515689	cg26515689	30779505	CACCATAAAATATATCAACTA			II	
-0.110484	cg26520012	cg26520012	21677360	ACCACAA	23712417	ACCGCGA	I	C
0.1048059	cg26525463	cg26525463	25746318	AATTCTATTTAAAAACTAACC			II	
0.1332426	cg26531231	cg26531231	34794479	AACTCATCAAACRACTATAAC			II	
-0.115156	cg26531700	cg26531700	49604443	CAATCTCAAACACTTCCRAAA			II	
-0.185955	cg26539321	cg26539321	64633410	CCAACCC	44747338	CCAACCC	I	A
0.1293284	cg26547816	cg26547816	55630445	CTAAAAATCCAACTATTCCRAT			II	
-0.16751	cg26548410	cg26548410	13797473	TTAAATCTCRAAAACATCTAA			II	
-0.108901	cg26550861	cg26550861	65611427	AACACCA	60674478	AACACCA	I	C
0.144706	cg26555052	cg26555052	55781355	TACTCCA	56735502	TACTCCA	I	T
-0.102988	cg26556336	cg26556336	39802498	AACRTATTAACATCCCCTTTTA			II	
0.115959	cg26557270	cg26557270	57621386	CTCCCACACCCAAAATAAACC			II	
0.1148004	cg26557756	cg26557756	55671425	TATAAAATAACCCATAAAAAC			II	
-0.350882	cg26566189	cg26566189	51692407	ATTCTTAAAACAAAACCTAAA			II	
0.1272978	cg26567385	cg26567385	35810338	TTCCAATACTACTACATCATAA			II	
0.1222554	cg26567736	cg26567736	46682415	ATTCAACCCCAAATTTAATTCC			II	
-0.11811	cg26570714	cg26570714	31716491	AAAAAAACCAACTTAAATTCT			II	
-0.156605	cg26570901	cg26570901	71802374	ATAAAAA	49785337	ATAAAAA	I	C
-0.110723	cg26576353	cg26576353	11695399	TCAAAATAAACAACCTCATAC			II	
-0.132087	cg26583344	cg26583344	33799355	CCCACAA	36642399	CCCGCAA	I	A
-0.170121	cg26590082	cg26590082	24751415	AACTTACCCAAAAAACACTAT			II	
0.2091301	cg26590106	cg26590106	61648332	TTACTCCAAAATCTCAAACCA			II	
0.1265721	cg26599871	cg26599871	46766455	ACTACCRCRCAAATTCAAAAA			II	
-0.111223	cg26601187	cg26601187	34774405	ACAACAT	48758480	ACAACGT	I	T
0.1072002	cg26603275	cg26603275	56671362	TCCCTACTAAAATATCCTTTAA			II	
-0.100423	cg26606589	cg26606589	29720320	CCCACCT	58680349	CCCGCCT	I	C
0.1118857	cg26607031	cg26607031	53770489	TATATACTACCTTACTCCAAAT			II	
-0.183559	cg26607620	cg26607620	65650307	AATACAA	74790425	AATACGA	I	C
-0.127374	cg26618441	cg26618441	44629409	TCTTTACCRCAAATTCTTTCRT			II	
-0.256172	cg26620530	cg26620530	72664469	ACTAAAA	62672337	ACTAAAA	I	C
0.1487561	cg26640927	cg26640927	55773392	AATAAAAACTCCCTCTATCAA			II	
0.205133	cg26642774	cg26642774	71617460	TCTAAATTATTTAAATCCAAAT			II	
-0.118137	cg26643967	cg26643967	22660445	AACCCTA	65702504	AACCCTA	I	A
-0.130554	cg26644049	cg26644049	50714320	ACAAACA	56650334	ACAAACA	I	C

0.1156634	cg26644674	cg26644674	35767342	AAAATACATAATTAACTCCTA			II	
0.1888946	cg26645432	cg26645432	61731305	TTACCTTTTCCAAAAACRACA			II	
0.1097748	cg26648185	cg26648185	69716389	TTAACATCCTCTAAAAACAAA			II	
0.2338095	cg26651280	cg26651280	21716506	CACCTACCACCCTCCCTACAAT			II	
0.1510522	cg26651303	cg26651303	69748313	TATCCTA	57691326	TATCCTA	I	T
0.1442543	cg26658509	cg26658509	17793361	AATACCTACAACCTTCRAAAT			II	
-0.103001	cg26659805	cg26659805	67781376	CAAACAC	70743379	CGAACGC	I	C
	cg26665035	cg26665035	32769455	ATACAAC	73635397	ATACGAC	I	A
-0.112183	cg26667091	cg26667091	58684433	AAACRTTTTTCCTCCCTTAAAT			II	
0.1888096	cg26673648	cg26673648	20643483	CCCTCTAAAAATACCCTCTAA			II	
0.1270596	cg26675336	cg26675336	25790323	AAAAATAACATTTATATAATA			II	
0.1032393	cg26676440	cg26676440	29735503	AAACTAACCCCRCTCCAATTT			II	
-0.146686	cg26679004	cg26679004	24802369	CTTAAAA	37789498	CTTAAAA	I	A
0.1294638	cg26680608	cg26680608	29604349	AAAAATCAAAATCTATAATAT			II	
-0.162797	cg26684363	cg26684363	50792331	TTTAAAA	19714383	TTTAAAA	I	T
-0.120265	cg26687173	cg26687173	60616472	RAAAAATAACRACAACTTACA			II	
0.4980344	cg26690407	cg26690407	41734414	AAACTAAAAAATCTAAATAAA			II	
-0.106097	cg26693329	cg26693329	56719484	ACRAACCCTATTTCRAAAAAA			II	
0.1110657	cg26700919	cg26700919	54775355	CRATAAAAATAATATCTCCRA			II	
0.1442463	cg26705599	cg26705599	44686317	TAATCRCTATCAAATATACRC			II	
0.1708494	cg26705764	cg26705764	56712347	CTATTTA	55669349	CTATTTA	I	C
-0.184148	cg26715559	cg26715559	19747404	CAAAACC	16738355	CGAAACC	I	C
-0.163577	cg26716902	cg26716902	47627353	ATTAACA	66605321	ATTAACA	I	A
-0.15311	cg26717554	cg26717554	42807306	TAAACAA	54764402	TAAACAA	I	A
-0.132859	cg26725029	cg26725029	19733366	CTACTTC	10692372	CTACTTC	I	A
0.1275628	cg26730543	cg26730543	60736495	TTACCCAAATCTACTCTTCATA			II	
-0.123233	cg26734249	cg26734249	51800501	CTACAAAACCATTTTTAATTA			II	
0.1339008	cg26738024	cg26738024	21643304	AATCACTACACCCRACCTAAA			II	
-0.102594	cg26739149	cg26739149	57760454	AACACCT	20729387	AACGCCT	I	T
-0.21807	cg26739327	cg26739327	15674421	ACCTTCTCTCTCTCTAAATCTA			II	
-0.206128	cg26744682	cg26744682	25706443	ACAAAAA	32762320	ACGAAAA	I	A
-0.129807	cg26751645	cg26751645	43756408	AAAAATT	56742483	AAAAATT	I	C
-0.126482	cg26753908	cg26753908	45736477	CACAAAA	19683378	CACAAAA	I	T
-0.170196	cg26757229	cg26757229	52677329	CATAAAACCTTATAAAATATC			II	
0.1033576	cg26763945	cg26763945	21756503	AACCTTCATTTTTAAACTTACT			II	
0.1033697	cg26767974	cg26767974	69641403	AAAAAAAACAAAACCATCTAT			II	
0.1231449	cg26772386	cg26772386	47753436	CACATTATCTAAATCACATCC			II	
-0.146737	cg26775123	cg26775123	44723436	CRATCTAAAAAATAAAACTTA			II	
0.2381559	cg26776784	cg26776784	29600381	AACCCCTAAATCACTAAAATT			II	
-0.102223	cg26784201	cg26784201	38639415	TCAAAAA	29731349	TCGAAAA	I	A
0.2697506	cg26786615	cg26786615	41696393	AATTAAAATAAAAAACCAAAC			II	
-0.107442	cg26787381	cg26787381	18622475	TCTCACT	38648505	TCTCACT	I	T
0.1047878	cg26788216	cg26788216	63738432	ATCAAAAAACAATCAATAATA			II	

-0.109541	cg26791257	cg26791257	25630315	ACAATTC	64734485	ACGATTC	I	A
0.1049179	cg26794700	cg26794700	25603505	ACCATAACATATAACCRATATA			II	
0.1511274	cg26796043	cg26796043	51642431	TATATTC	66716324	TATATTC	I	A
0.1786346	cg26796083	cg26796083	70615357	TCTTACAATACCATCCTTACTA			II	
0.1032344	cg26800883	cg26800883	38756410	TTAACATACCTAAATAACCATA			II	
0.1410035	cg26801241	cg26801241	35631475	TTAATCAAACCAAATCAAATA			II	
-0.140603	cg26802063	cg26802063	64604428	ACAAAAT	71645485	ACAAAAT	I	A
-0.101276	cg26803268	cg26803268	21803377	TAACAAT	47640414	TAACGAT	I	A
0.1201543	cg26809868	cg26809868	40618348	TAACAAACTAACTTCTATCCA			II	
0.1527593	cg26811705	cg26811705	14659426	AAAAAAC	30701330	AAAAAAC	I	A
0.1425784	cg26813483	cg26813483	58803504	TAACATCRATCCCTAAAAACR			II	
0.1963193	cg26815032	cg26815032	10657320	CCATCCTTCATTAAAAAATATA			II	
0.1106654	cg26817755	cg26817755	38804485	ACAACCACAAAACATTTCTA			II	
0.1032529	cg26818786	cg26818786	13611506	TCAAACAACTTTTTCTTTCTC			II	
-0.122788	cg26820259	cg26820259	40647335	CATTCTAATTCACATCCCCCAA			II	
-0.103577	cg26822175	cg26822175	43780356	CATTCAAAATAACACAATAAC			II	
0.1384075	cg26826183	cg26826183	38808475	TTTTATACATTTACTTATTTAT			II	
0.2136557	cg26828599	cg26828599	55632485	CAAATACTTTTTCCCTAAATAT			II	
-0.115445	cg26841048	cg26841048	10786344	TCCCTATAATTTCRCTAAAATC			II	
0.1279871	cg26843807	cg26843807	74679492	CATCAAACAATAAAAAATTCA			II	
0.1452947	cg26854588	cg26854588	20690445	AAATCAAAATTCAAAAAAACC			II	
0.131018	cg26855015	cg26855015	34739493	AAAAACTATTAATTTACAAAC			II	
-0.104862	cg26856631	cg26856631	31766367	TACAACAAAACATAATCAACC			II	
0.1919323	cg26864826	cg26864826	14772371	TCCTTTTATAAATACAATATAC			II	
0.1232764	cg26871180	cg26871180	67801384	AAAATCATAAAATAATCAAAC			II	
-0.136899	cg26873880	cg26873880	65743318	ACTTCTCTCATACTTTAAAAAC			II	
-0.127081	cg26874229	cg26874229	66674405	CAAAAAA	42694358	CAAAAAA	I	A
0.124374	cg26879644	cg26879644	72640510	AAAATTCTATCAATCCTAAAA			II	
-0.180401	cg26881268	cg26881268	42661402	ACACAAACTTCATAACTACAC			II	
-0.196227	cg26882525	cg26882525	14744359	AAATCACACACTCAAATACCT			II	
0.1193291	cg26915799	cg26915799	21626380	TCCACCTACTAAACAATTTTTC			II	
-0.103181	cg26915952	cg26915952	30713425	TAAACAA	36747372	TAAACAA	I	A
0.1313547	cg26925980	cg26925980	23688339	AAAATTCAATAACTATATCAC			II	
-0.118217	cg26927544	cg26927544	17756352	TTACCAA	60699427	TTACCGA	I	A
0.1455062	cg26928789	cg26928789	60716382	AAAAAAAAAACACTTCTAAAA			II	
-0.14427	cg26929925	cg26929925	48754322	CAAAATT	25803318	CGAAATT	I	A
-0.131245	cg26930498	cg26930498	36600316	CACRTAAAATATAACCCACCC			II	
-0.177116	cg26936989	cg26936989	69669477	ACTCCCAAAACATCACTATCT			II	
0.104432	cg26939946	cg26939946	22713399	TCAAAATCCCTAACAAAAACT			II	
	cg26940725	cg26940725	67799462	ATCCATTAACATAAACRAATTA			II	
-0.656263	cg26941787	cg26941787	70637510	CAAAAAAATCTTCTAACTTTCT			II	
-0.108884	cg26941870	cg26941870	63650347	TACAAAA	65750312	TACGAAA	I	T
0.1273714	cg26942829	cg26942829	24618335	CCCAAAAAAAAATATTCTATA			II	

0.1135057	cg26945978	cg26945978	21758419	CAAAACATTAAAATTATAAAT			II	
0.1300477	cg26949055	cg26949055	59707352	TCCATTCAATAAATCACTACTT			II	
0.7126956	cg26951705	cg26951705	42739502	ACTTAAA	49667370	ACTTAAA	I	C
0.1069575	cg26955290	cg26955290	38625407	AAAACTAAAAAAACRTCATCA			II	
-0.136627	cg26955881	cg26955881	63760457	CAAAAAAACAAATCCTAAAAA			II	
-0.112625	cg26968378	cg26968378	17663376	TTCAAAACRAAAACCAATTTA			II	
0.1261153	cg26970042	cg26970042	43685411	ACTAAACCCCTCTAAAATTAT			II	
-0.115731	cg26972554	cg26972554	16777377	TAATAAAAAACCTCAAATACR			II	
0.1063399	cg26975184	cg26975184	57631400	TCAATAA	36712464	TCGATAA	I	A
0.2281658	cg26978379	cg26978379	22712463	AAACCAACACAACCAAACCTA			II	
0.1357068	cg26980152	cg26980152	59747386	AAATATTACTAACTTAAAATT			II	
0.1170523	cg26980937	cg26980937	24794368	TCTTCAAAATTCCACATTACC			II	
-0.108996	cg26982601	cg26982601	50742459	AAAAAA	23730422	AAAAAA	I	C
0.1447473	cg26987928	cg26987928	16658418	TCCTTAATCCCAAATAACTTA			II	
-0.120466	cg26991571	cg26991571	42649388	ACAAATA	18632350	ACGAATA	I	C
0.3363984	cg26995506	cg26995506	39753355	AATAACTTTCTCCTAATCACR			II	
0.1329498	cg27000233	cg27000233	73632481	CAACATCAAACAAAAAAATTC			II	
-0.132846	cg27000496	cg27000496	55698357	ATAAACT	66762343	ATAAACT	I	A
0.2458925	cg27000503	cg27000503	74713304	CTACTAAATTCTCTTTTACTAC			II	
0.1522497	cg27005118	cg27005118	39757339	AAATAAAAACATCCCCACRAA			II	
0.1258447	cg27010159	cg27010159	74726465	AAACAAATACTACAAAAATAA			II	
0.1091702	cg27014438	cg27014438	43626331	TTTTCCTTTAAAATAAATTTCC			II	
0.1079788	cg27018309	cg27018309	30775481	TCACTTTAATATACTACTCTTC			II	
0.4661825	cg27018984	cg27018984	55730337	ATAAATACCTAAAACCTCTTC			II	
-0.234236	cg27020349	cg27020349	37797354	CTACTCC	21763385	CTACTCC	I	T
-0.139674	cg27023360	cg27023360	29785438	AACTCCT	37634479	AACTCCT	I	T
-0.172794	cg27023871	cg27023871	37744504	RAAAAACAAAATTCCCACRAA			II	
0.1157741	cg27024408	cg27024408	40638350	AACAAAAAATTACTTAATATA			II	
0.1117509	cg27026192	cg27026192	40729374	AAAAATACTATAACTAAAAAA			II	
-0.128863	cg27032146	cg27032146	60613493	TCAAAAA	22725397	TCAAAAA	I	A
0.1009908	cg27035997	cg27035997	58804463	AAAAAAACAACAAATACAAA			II	
0.1176778	cg27049594	cg27049594	56651508	TCAAAACATCTACRAATAAAA			II	
-0.295952	cg27049643	cg27049643	65639453	TTTCTAC	36691449	TTTCTAC	I	T
0.1201226	cg27050407	cg27050407	72697501	CCCAACCTCATCCTTCCTAAAT			II	
-0.154761	cg27053299	cg27053299	61762319	AAACTTC	21655443	AAACTTC	I	A
-0.106483	cg27058480	cg27058480	71679446	TAATATACTAAACRAAATATA			II	
0.1139106	cg27061791	cg27061791	49754427	ATTCTAAAAAATAACRTATCR			II	
-0.156861	cg27069422	cg27069422	52798451	AAATACA	14672356	GAATACA	I	C
-0.136684	cg27070397	cg27070397	16613327	CTTTCCCRTAATAAAACTACC			II	
-0.104222	cg27071152	cg27071152	49759419	TACTACA	74642481	TACTACA	I	A
0.1437456	cg27073349	cg27073349	28774445	ATATTTAAAAAACAACTAACA			II	
-0.187438	cg27074174	cg27074174	42657472	TAAAATT	53774367	TAAAATT	I	A
0.103161	cg27074505	cg27074505	63669414	TCTTTTCCAATTCCACAATACT			II	

0.1104968	cg27074995	cg27074995	25671495	AAAACCRCACCCCAACCCCTA			II	
-0.192191	cg27079096	cg27079096	18698302	AACATTCTTTACRATATCACCA			II	
0.1944752	cg27079194	cg27079194	22628361	AAAACACTCATACCATACRAA			II	
-0.147942	cg27080348	cg27080348	23660470	TTCACTA	67760433	TTCACTA	I	A
	cg27081243	cg27081243	33704307	CCCATTT	13644439	CCCGTTT	I	C
0.1335551	cg27083087	cg27083087	26697460	AAAAATA	69671508	GAAAATA	I	A
0.1321464	cg27084028	cg27084028	33774509	CTAAAAACAAAATAACTTACR			II	
0.1044698	cg27084232	cg27084232	55610395	ATTTAAATAATATTTCRAAAA			II	
0.1687777	cg27086157	cg27086157	40698319	ACRTACCCCRTAAAAATCAAA			II	
0.1171716	cg27086308	cg27086308	55662450	AAAAAATAAAACCACAAACCT			II	
0.1878473	cg27092248	cg27092248	58623436	AACTAAACTATTAATCAATTTC			II	
-0.210022	cg27097438	cg27097438	26787346	ATACAAA	56786397	ATACGAA	I	T
0.1181056	cg27100140	cg27100140	41639327	ATCRAATATCATTCAAACAAA			II	
0.2300423	cg27107292	cg27107292	33650305	ACCTTATCACCTCTACCATCAT			II	
0.1304287	cg27107963	cg27107963	61804348	AAAAATAAAAACCTCATAAAA			II	
-0.453823	cg27112983	cg27112983	22625499	TAATCAA	38637352	TAATCAA	I	A
0.2084748	cg27115863	cg27115863	22626378	CAAACCA	14698339	CGAACCA	I	T
-0.110143	cg27119318	cg27119318	47786401	ACTCCCT	49631300	ACTCCCT	I	C
0.1104102	cg27120543	cg27120543	62612508	CCAAAACCCRTTAAATCCCTA			II	
0.1207931	cg27122706	cg27122706	44638414	AATCAACTAAAAACAAAAATA			II	
-0.145577	cg27134251	cg27134251	54780387	CATTTATTACAAAACRTAAACA			II	
0.2110692	cg27135799	cg27135799	38624406	AAAAACATCATAATCTTCCAA			II	
-0.133779	cg27137258	cg27137258	40786356	ACTAAAC	16786492	GCTAAAC	I	A
-0.174944	cg27138088	cg27138088	25769382	AACAAAA	23785427	AACGAAA	I	A
-0.114523	cg27140058	cg27140058	20615401	AACCACA	35637328	AACCACC	I	T
0.1533047	cg27141751	cg27141751	69713489	AACACTATTAAAACTTCTATCC			II	
-0.486374	cg27141807	cg27141807	55690505	AAAACCAAAATCAAAATTCCC			II	
0.1673579	cg27145347	cg27145347	33750455	CTCAAAAATACCRTATCTATCC			II	
0.1451889	cg27145458	cg27145458	56637356	AATATAATCTACTATCCATTTC			II	
0.1112444	cg27147556	cg27147556	67807427	ACAAACA	29706375	ACAAACA	I	C
-0.22887	cg27149073	cg27149073	11611421	AAACTAAAAAAACCCTACRCC			II	
0.1456082	cg27149973	cg27149973	40726435	CCTCATACCTAAATATACRTCA			II	
0.1073868	cg27151677	cg27151677	44778424	TACRACCACTAATATTCAAAA			II	
-0.120831	cg27151770	cg27151770	30703368	CCCTAAC	64807415	CCCTAAC	I	C
-0.101843	cg27152219	cg27152219	48629373	ACAACAA	11693393	ACGACGA	I	A
0.1516035	cg27156510	cg27156510	73639456	TCATAAAAAATATACTTAAAA			II	
-0.10677	cg27158572	cg27158572	33730338	AACACCA	39658484	AACACCC	I	A
0.1180523	cg27159987	cg27159987	14676344	TCATATTTTCCAATAAAATAAA			II	
0.1057225	cg27166177	cg27166177	72774459	ACTCAACCATAACRATAACCA			II	
-0.129049	cg27166673	cg27166673	68632365	AAAAAAA	44726414	AAAAAAA	I	A
0.1241864	cg27173148	cg27173148	18733325	CAACTAA	35674409	CGACTAA	I	A
0.1261226	cg27175112	cg27175112	18779347	AAATAACCTAACRTCTCTCCA			II	
0.1107465	cg27177158	cg27177158	20601316	TCCAAACATAAAAAAAACTAT			II	

-0.116053	cg2717829:	cg2717829:	25683343	AATAATAAAAATCCCTACAAC'			II	
-0.144095	cg2717937:	cg2717937:	42797416	CCATAAC	64680471	CCGTAAC	I	C
0.1088335	cg2717981(	cg2717981(	53803352	TAATACTAAACRCCCCTAAAA(			II	
0.1164251	cg2718137:	cg2718137:	69721302	ATAAATC	55727389	ATAAATC	I	T
0.1391373	cg2718303(	cg2718303(	71680393	AAATACATTCCTAATTCATACF			II	
0.1280446	cg2718651:	cg2718651:	58668500	AATACTAAAAAACCAAAAAAC			II	
0.1199025	cg2719002:	cg2719002:	53748501	AAAAATTAACTCTACAATATT			II	
0.1424553	cg2719969:	cg2719969:	41754334	AAAAATACAAAAACCAAAAA/			II	
0.112103	cg2720025:	cg2720025:	58746331	TAAAAACAACAACATAACTAT			II	
0.1122703	cg2720089:	cg2720089:	66639482	CRTCACRAATACTAATATAAT/			II	
-0.235307	cg2720291:	cg2720291:	29707314	RCACTAATCATCRTACTAACC/			II	
0.102164	cg2721496(	cg2721496(	27609506	TTATAAT.	15718384	TTATAAT.	I	C
0.139533	cg2721918:	cg2721918:	57769383	AACCACAAAACTCTTTCCCCA/			II	
0.1400692	cg2722614:	cg2722614:	16780412	CACATCA	38724384	CACGTCA	I	A
-0.119659	cg2722774:	cg2722774:	31712431	ATAATAA	37619383	GTAATAA	I	C
0.1251567	cg2722827:	cg2722827:	25764475	AACCTCTACAAACACRAACAT.			II	
0.1201915	cg2723076:	cg2723076:	49780490	ACCACTAAAAAACACCACTAC			II	
0.1165243	cg2723236(	cg2723236(	27709322	CAAACAAATTTAAAACCATTC'			II	
0.1544836	cg2723315l	cg2723315l	13736483	TCTAAACCATAAATACACATT/			II	
-0.230283	cg2724497:	cg2724497:	74728376	AACAATT	33694484	AACAATT	I	A
0.1153097	cg2724769:	cg2724769:	44759351	ACAAAA/	31768322	ACGAAA/	I	A
-0.101796	cg2724858:	cg2724858:	16718309	ATAAAACTAAACTTACCCTAA.			II	
-0.236689	cg2725115:	cg2725115:	37707300	AAACAA/	12721408	AAACGA/	I	A
-0.113387	cg2725192(	cg2725192(	43694332	ATTACAT	52770307	GTTACAT	I	T
-0.127494	cg2725348:	cg2725348:	66610377	AAAACCTACAAACAATACCCC			II	
0.3104981	cg2725798:	cg2725798:	20738498	ATACAATAATACTATCTTAAC]			II	
-0.116059	cg2725856l	cg2725856l	65616364	CRCTATCCAAATCTACAAATC(			II	
-0.175262	cg2726173:	cg2726173:	54743425	AAAACAAATAATTAAATACCA			II	
0.1122667	cg2726399(	cg2726399(	19633313	ACTTAAAATAACACTTAACAA'			II	
-0.170758	cg2726432:	cg2726432:	39635355	AATACAA	46659312	GATACAA	I	C
0.103133	cg2726883:	cg2726883:	74723367	TTAATTATAAAACAAAAACTC'			II	
-0.209012	cg2727224(	cg2727224(	35664454	ATATACAATTATTTAACAACA/			II	
0.2376561	cg2727322:	cg2727322:	45740413	TCACAACTTAATTCAATCTAAT			II	
0.1060884	cg2728239:	cg2728239:	14747339	CATACACAATTCCCTACACAC/			II	
0.1063636	cg2728412(	cg2728412(	64691332	ACCCAAATCRCTTTCRAACAA(			II	
-0.15487	cg2728419:	cg2728419:	47622464	ACAATACTAACCRACAACACT.			II	
-0.109848	cg2729130:	cg2729130:	33710361	ACCTCCATCTTATAACTAAAC(			II	
0.1078797	cg2729146:	cg2729146:	51768317	TAATTCCCACCCTAATTCCCAA			II	
0.1248415	cg2729209:	cg2729209:	72810314	ATATTCTTAAAACTTTCCTAAA			II	
0.1338452	cg2729222(	cg2729222(	41607394	TATAATCCCAAAAATCCCTAA.			II	
0.1003638	cg2729565:	cg2729565:	29685464	AATTCTCTCTAATCAACCCTAC			II	
0.1245906	cg2729755:	cg2729755:	25602349	AAACCAACTATAAAACCATTC.			II	
-0.183867	cg2729834:	cg2729834:	26760337	AAAACTA	39706440	AAAACTA	I	C

0.1164799	cg27307781	cg27307781	26809452	AAAAACT	58752493	AAAAACT	I	C
0.3257825	cg2730873{	cg2730873{	33793335	TTTAAAACCAAACTTAAAATA.			II	
-0.130635	cg2732662(	cg2732662(	66679486	ACCACTA	63773438	ACCGCTA	I	A
-0.159344	cg2732867:	cg2732867:	11704424	AAAAAATAACAAAAAATACCC			II	
-0.120306	cg2733000(	cg2733000(	14736422	CTTACCCCCAAAACAACAACR.			II	
0.1063729	cg2733129:	cg2733129:	54688488	CRCCTCTCTACRAAACATCCTT			II	
0.1058112	cg2733658′	cg2733658′	12720447	TAACACA	71636304	TAACGCG	I	A
	cg2733883(	cg2733883(	13644452	CCRCTCAACCCAAACCTATAA(			II	
-0.24691	cg2734291(	cg2734291(	33666436	CCCACCT	15745448	CCCACCT	I	A
0.2263276	cg2734752:	cg2734752:	61768307	TTCCTTA.	42802448	TTCCTTA.	I	A
-0.132761	cg2734851:	cg2734851:	66743491	ACAACA/	58797317	ACGACGA	I	T
0.1055437	cg2735057(	cg2735057(	16797429	CTACTATACTTAATAAAAAAA(			II	
0.1165702	cg2735571:	cg2735571:	60743476	AATTCCRAATCTAAACCACTA/			II	
-0.15809	cg2736252:	cg2736252:	44659426	AAAAAA/	29653431	AAAAAA/	I	A
0.1975835	cg2736298(	cg2736298(	63766336	AATCACAACCACAACTATTTT/			II	
0.1092125	cg27363141	cg27363141	27793347	ACAAAAC	21680509	ACGAAAC	I	C
0.1765362	cg27365991	cg27365991	20770382	ACAAACACAACAATAAAAACT			II	
-0.191054	cg2736676(	cg2736676(	21651349	ACACTAA	14699327	ACGCTAA	I	A
-0.119132	cg2736909(	cg2736909(	46808467	CACAACA	44740455	CACGACC	I	A
0.10013	cg2737145(	cg2737145(	11810404	ACATTTCCCCCACAAAAACAA(			II	
-0.108972	cg2737245:	cg2737245:	18690453	CAAAACC	19678397	CGAAACC	I	T
-0.218545	cg2737853′	cg2737853′	31615459	AATATTAAACACCAACATTTT			II	
-0.113443	cg2737940′	cg2737940′	44699377	TTATTAA	48766488	TTATTAA	I	C
-0.111252	cg2738341{	cg2738341{	29768342	CTTCRTTAAATATCAAACCACT			II	
0.1097521	cg2739181(	cg2739181(	54750357	CATAACA	56763451	CATAACG	I	A
0.1074677	cg2739290⁄	cg2739290⁄	71676300	TAATCCTAAAACACTATAACR			II	
-0.102402	cg2740540(	cg2740540(	59651484	ATCCTCT.	70740307	ATCCTCT.	I	A
0.1274582	cg2740607(	cg2740607(	59665410	TATCCTTCAATATTCAAATACC			II	
0.2226923	cg27408171	cg27408171	39791422	ACAAAA/	12749411	ACGAAA/	I	A
0.1171927	cg2741285′	cg2741285′	28717352	AATAATATTATAAAATATCAA.			II	
0.4139948	cg2741486{	cg2741486{	44664477	ACTCAAA	21610445	ACTCAAA	I	C
0.1331104	cg2741633′	cg2741633′	71665371	ACTTATAATTACTAACTTCTT			II	
0.1081323	cg2742234:	cg2742234:	53700466	AAATACAAACACAACCAACCA			II	
-0.129537	cg2742774:	cg2742774:	16749426	ACAATAA	30747495	ACGATAA	I	A
	cg2743104(	cg2743104(	26641373	AACCTAC	32768334	GACCTAC	I	T
0.2458772	cg2743347(	cg2743347(	42614354	ATAAAACCAAAAACAATATAA			II	
-0.116237	cg2743436{	cg2743436{	42682384	CTAAAACAACAAATAAAACCT			II	
0.1045666	cg2744086(	cg2744086(	56672320	AACTACTTAACATTATTTTACC			II	
-0.112798	cg2744135⁄	cg2744135⁄	14756460	AAACTTAAAAACCAAAACCAA			II	
0.1038696	cg2744333:	cg2744333:	28752456	AACACRTCTTAAAACTACACC			II	
0.4482122	cg2745074⁄	cg2745074⁄	50802392	TTTCTATCTCTTACTCATAATA.			II	
-0.140723	cg2745136:	cg2745136:	24611496	AACRAACTATCAAATCRATTA(			II	
-0.102541	cg2745225:	cg2745225:	58690410	ACAAACTTATCCCTTTATTTAC			II	

-0.106571	cg2745292	cg2745292	55621476	AAACCAT	40702446	AAACCGT	I	T
0.1142153	cg2745410	cg2745410	73724445	AACCTCAATAACTCAAACTAA			II	
0.1382517	cg2746125	cg2746125	62610464	CTAAAATCACRTAATACAACC			II	
-0.1578	cg2746787	cg2746787	14632431	TACTACTCCTCACTAAATACC			II	
0.280302	cg2746888	cg2746888	29620403	AAAATAC	12793431	GAAATAC	I	A
-0.12149	cg2746973	cg2746973	38713346	AAACCTA	31774471	AAACCTA	I	A
0.2742402	cg2748142	cg2748142	44744456	CRCTTCRAAAACAAAATTTCT			II	
0.1109793	cg2748466	cg2748466	21666391	AAAAAA	11780432	AAAAAA	I	A
-0.120887	cg2748510	cg2748510	17605506	ACATAAA	20768326	ACATAAA	I	C
0.1030461	cg2748874	cg2748874	48773302	TACATCTCTAATCCCTTCCTTA			II	
0.1109278	cg2749416	cg2749416	20717367	AAATAA	20632490	AAATAA	I	A
0.148219	cg2749489	cg2749489	61748336	AATAAAAAACTTACCCTACAT			II	
0.2578897	cg2750229	cg2750229	62806352	ATCCCAA	62687463	ATCCCAA	I	C
-0.100242	cg2750395	cg2750395	45729369	CAAAAAACACRAAATCCAAAA			II	
0.1188539	cg2750818	cg2750818	42743387	AATCCTCTAACACCAATAACA			II	
0.1004396	cg2750912	cg2750912	34691381	CTCTATCCAATTTATTCTATT			II	
0.2236403	cg2750996	cg2750996	30789337	ACTCTAATAACCRACAATACC			II	
-0.180011	cg2751403	cg2751403	50745409	TAAAAAC	46671445	TAAAAAC	I	C
0.1185526	cg2751433	cg2751433	39653495	CAAAACTTCCAACAAAATTAA			II	
-0.307141	cg2751692	cg2751692	19682423	TCAAAAACCCTAATTCAAACT			II	
-0.111673	cg2751756	cg2751756	45782402	AAAAATA	45698475	AAAAATA	I	C
0.1187139	cg2752054	cg2752054	67703307	TTTATTTAACTTTCCTCTTAAT			II	
0.32875	cg2752116	cg2752116	34808321	AAAAAA	50637434	AAAAAA	I	C
	cg2752642	cg2752642	10643372	ACAAAA	64765396	ACGAAA	I	A
0.105091	cg2752677	cg2752677	30783419	ATTTTTAAACTTCTCATCTATA			II	
0.1361911	cg2753385	cg2753385	34615334	AAAAACTCATTAATAAAACAA			II	
-0.120861	cg2753719	cg2753719	17804389	CRAACCAAATATATTCTCAAT			II	
-0.153181	cg2754189	cg2754189	73804305	TTATTATAAAAATACTCRCTA			II	
0.1015756	cg2754234	cg2754234	72691401	CCACCCT	52700469	CCACCCT	I	T
-0.223978	cg2754304	cg2754304	11634337	CCCCRAAAAAAACTTACCAAA			II	
0.1131514	cg2754467	cg2754467	66683447	AAAACRAAAATACAACTATCA			II	
-0.171346	cg2754611	cg2754611	35768310	AAACACAACTATTATTTATAA			II	
0.2814462	cg2754918	cg2754918	60697329	AAAAAAAAATTACAAAACCA			II	
	cg2755066	cg2755066	32793334	TCACAAA	54606331	TCGCAAA	I	C
-0.103826	cg2755392	cg2755392	12790451	CACCCTC	64639473	CGCCCTC	I	A
-0.182746	cg2755778	cg2755778	19616307	CATAAAATAAACTCTACRAAC			II	
0.1471479	cg2756758	cg2756758	42654353	TATTAAAAATTAATAAATATT			II	
-0.161503	cg2757212	cg2757212	19647409	AACCCAA	51674379	AACCCGA	I	C
0.1363796	cg2757562	cg2757562	15736467	ACTCCACACCCCTAACTCCTTA			II	
-0.10963	cg2757714	cg2757714	16659309	CAAACTA	44767473	CAAACTA	I	A
0.1016914	cg2758232	cg2758232	14704347	CAATCCRACAATAAATAACTA			II	
-0.140945	cg2758330	cg2758330	27634389	AACCTAC	32665472	GACCTAC	I	A
0.1131933	cg2758514	cg2758514	24620463	AAACCAACACACCTAACTTAT			II	

0.1156557	cg2758835(	cg2758835(	48702341	ACTACAACAAACAAAAAATCT			II	
0.1291422	cg2759810	cg2759810	72641436	AAATATATAACTTCCCRTATA/			II	
0.1123894	cg2760157	cg2760157	26690489	AACTAAAAAAAACTTCAAAAA			II	
-0.162521	cg2761232	cg2761232	51801389	AAAAACTACCRATAAACAATT			II	
-0.134924	cg2761448	cg2761448	62601359	TCTAATA	47732508	TCTAATA	I	A
0.148048	cg2762172	cg2762172	62733489	TTCAACATCACACCAAACTAA			II	
0.1096166	cg2762199	cg2762199	41605444	AATAAACAAATATTAACTTCT/			II	
-0.307871	cg2762513	cg2762513	15722311	AAAATATCATTTCTAAATAAC/			II	
0.2281281	cg2762618	cg2762618	16807466	ACCAAAT	66634378	GCCAAAT	I	C
-0.100636	cg2763919	cg2763919	49621366	AAAATCTATTCCRAAATTTCTA			II	
0.1057788	cg2764025	cg2764025	73669303	AAAACCATCCCCCTTTATACTT			II	
-0.174941	cg2764247(	cg2764247(	58749395	AAATAAA	48615364	AAATAAA	I	C
0.1097667	cg2764821	cg2764821	25667382	AAAAAATAACCCAAATATATA			II	
0.1062943	cg2764827(	cg2764827(	67670425	CTATACACATTAAAAACCTCC			II	
-0.143215	cg2765521	cg2765521	27784335	TACTCCA	25657320	TACTCCG	I	A
-0.102379	cg2765904	cg2765904	50766426	AATTAAT	51699366	GATTAAT	I	C
0.1852823	cg2765979	cg2765979	58690368	TATTACCTCTAATTTAAAACCR			II	
-0.123543	ch.22.4827	ch.22.4827	62659432	AACTAACTACAAATACTCACC			II	
-0.129173	rs10033147	rs10033147	53636471	CCCACTC	37774439	CCCACTC	I	T
0.148117	rs1019916	rs1019916	43769491	TCTCACAACTCAAATCCCATC/			II	
-0.121469	rs10457834	rs10457834	15748350	TTACTTCACTATTTAATTCACT			II	
-0.299441	rs10774834	rs10774834	22788456	TATAACT	30718329	TATAACT	I	T
-0.166876	rs10796216	rs10796216	14622465	TAACTAA	41635319	TAACTAA	I	A
0.2946505	rs10882854	rs10882854	47771383	CTTAAAC	39668349	CTTAAAC	I	C
-0.143493	rs10936224	rs10936224	34619331	TCTTTCA	30630453	TCTTTCA	I	T
-0.153106	rs11034952	rs11034952	58692423	AATCATC	44652497	AATCATC	I	C
-0.174347	rs11249206	rs11249206	60753422	TAAATCTCCCCTCTCTTATTCC			II	
-0.158488	rs13369115	rs13369115	44627385	ACTAACA	74780462	ACTAACA	I	A
-0.18639	rs133860	rs133860	52724359	TCTCACTCATCCCTCTCCCTTT			II	
0.140877	rs1414097	rs1414097	23759362	CCCACTA	39621311	CCCACTA	I	C
-0.222741	rs1416770	rs1416770	28667385	AACCTACTATATTAAATCTTTC			II	
0.1094236	rs1467387	rs1467387	45739435	AACCCAATCCAAACCAATATT			II	
-0.316369	rs1484127	rs1484127	33780375	CACTAACCAACCTACATAAAT			II	
-0.157557	rs1510480	rs1510480	69633374	TCACCTTCCATTAATTACTTCT			II	
0.3207767	rs1941955	rs1941955	33709340	TAAACCCTACTAATTCATAAA/			II	
-0.477242	rs2032088	rs2032088	63663495	TTACTTCTATAACACCACACTT			II	
-0.329962	rs2125573	rs2125573	25698376	TTCTCCAACCATTCAACCTAA/			II	
-0.307094	rs213028	rs213028	10622451	ACCTCCT	24684377	ACCTCCT	I	T
0.2962424	rs2208123	rs2208123	12714403	AATATAC	19761497	AATATAC	I	T
-0.286562	rs2235751	rs2235751	17621345	ATCCCAAAACAAACCTCCCCA			II	
0.3074717	rs2385226	rs2385226	46691371	TTACCAT	17623494	TTACCAT	I	C
-0.342941	rs2468330	rs2468330	23632464	ATCACACTTTTCATCACTCCAT			II	
0.1626822	rs2521373	rs2521373	12625304	CTACTACCTAATTAATAACAC(			II	

-0.287038	rs264581	rs264581	52695345	AACCATTTACAAAAATAAAAA			II	
0.2879038	rs2804694	rs2804694	13748334	CTTATTCCCATCATATTTCACT			II	
-0.104655	rs2857639	rs2857639	23793421	TAAAAAAAAATTCAACCTTCT			II	
0.3117841	rs3936238	rs3936238	42802473	AATACCC	29627504	AATACCC	I	T
0.1435546	rs4331560	rs4331560	10654345	CATAACATTTCTCATCCAATA			II	
0.1676485	rs4742386	rs4742386	34623456	TCATAAATCACCAAATCTCCT			II	
0.4692939	rs5931272	rs5931272	39615340	ATCTAACAATTTATTCTCATCC			II	
-0.147488	rs6471533	rs6471533	11697340	TAACTAC	55602361	TAACTAC	I	C
-0.102864	rs654498	rs654498	36707408	CTAAAAA	15665335	CTAAAAA	I	T
0.1497186	rs6626309	rs6626309	15806372	CTTTCTC	69767339	CTTTCTC	I	T
-0.160248	rs798149	rs798149	48789496	ACACAACCTCCAATACTTCCA			II	
0.1563471	rs845016	rs845016	37661486	TAAACTAATATACCAATTTAC			II	
-0.368202	rs877309	rs877309	54760445	CCCCAACCCAAAACCTCTATA			II	
0.1587971	rs9292570	rs9292570	48712372	CTAAAAT	14806497	CTAAAAT	I	T
0.1279564	rs9363764	rs9363764	36689314	TAACTCCCTACCACCTAAAAC			II	
0.1298546	rs939290	rs939290	70759471	ATTTTATTTTCACTTTCCACAA			II	
-0.452934	rs951295	rs951295	11711409	AACAAAC	39778441	AACAAAC	I	C
0.1704655	rs966367	rs966367	16795360	ATAACTCCTTTAACTTTAAACA			II	

COLOR_C	FORWARD	GENOME_	CHR	MAPINFO	SOURCES	CHROMOS	COORDIN	STRAND
	CACGCTC	37	2	237027592	TGAGAAA	2	236692331	R
	TCTCTAA	37	X	103810908	CGCTAAC	X	103697564	R
	TATACAC	37	14	22989583	CGTCCTG	14	22059423	F
	TCTCTGT	37	2	1165351	CGGGGTC	2	1155351	F
Red	CCCTCCC	37	16	629015	CCCCCTG	16	569016	R
	TTTTAAA	37	1	167127353	ATGCAAT	1	165393977	R
Red	CCCTGAG	37	6	31650808	CGGGGGC	6	31758787	F
	TCTGGAT	37	1	23521272	CCTGGGA	1	23393859	F
	ACATTGT	37	4	123506194	TTTGAAG	4	123725644	F
	GAATTAG	37	16	11099199	TGGCTAA	16	11006700	F
	GCCACCT	37	8	59170968	CGTCTGT	8	59333522	R
	TTCTGGC	37	12	54090754	CGCAAAA	12	52377021	F
	ACAAATC	37	16	3511787	CGTGTTT	16	3451788	F
	AGCCCTG	37	8	144399335	CGCACAC	8	144470710	R
	TTATGCT	37	7	87027887	CTGTATC	7	86865823	F
	GTGCTGA	37	6	169539646	CGCCGCA	6	169281571	R
	CTTATCG	37	19	56688439	GGAGGAC	19	61380251	F
	GTCCATT	37	1	223575793	TCTTTCTC	1	221642416	R
	AATGTAT	37	16	24712522	CTGAAGC	16	24620023	F
	TGGGGTT	37	2	46040236	CATGCAG	2	45893740	F
	TTTGTTG	37	2	242835364	CGGGTCT	2	242484037	R
	TTGAATC	37	1	55504649	GAAAGCT	1	55277237	R
	TTCATTT	37	16	86377053	TTACAAG	16	84934554	R
	TTGGGTG	37	2	237079099	ACCCTGT	2	236743838	R
	GTTTTCA	37	1	161391807	CGCCTTG	1	159658431	F
Grn	CTCGATC	37	4	122853693	CGGCGCA	4	123073143	R
	AAACCTC	37	16	5121151	CGGACTA	16	5061152	F
	GGCTTCT	37	1	53040491	GAGAATC	1	52813079	R
	GCTGGGC	37	17	78472159	TAGGAGT	17	76086754	R
	ACTATGT	37	1	233089275	CGGTAAC	1	231155898	R
Grn	CACCAGA	37	12	132944010	TGCACAA	12	131454083	F
	ACTGCAT	37	1	160121487	ACCTTCC	1	158388111	R
Red	CCAAGGA	37	13	112996967	CTGGGGT	13	112044968	F
	CTCAAAA	37	18	59218797	GATCCAA	18	57369777	F
Grn	TGGGGAA	37	15	22993172	TCCTCTTC	15	20544613	R
	CCCGTTC	37	16	420108	CGCCCAC	16	360109	R
	TAATGTG	37	1	161280266	CGGGGAA	1	159546890	R
	ACAGAAC	37	10	73857843	CGCTGGC	10	73527849	R
	CTGGTAA	37	5	146784171	CGTCCCT	5	146764364	F
	CCTTCCG	37	12	75784855	CGGGACA	12	74071122	R
Red	GTGTCCG	37	16	1069760	GTGACCG	16	1009761	F

	TCAAAAC	37	6	32466447	CGGCAGA	6	32574425	F
	AGGTGAA	37	Y	21664296	AGACTAA	Y	20123684	R
	TGTAGGG	37	6	166251659	CGAGATC	6	166171649	R
	GGCCCCA	37	6	32033006	CGATCTT	6	32140984	R
	TATGTTC	37	1	95703607	GTCACTC	1	95476195	F
	GCAATTT	37	1	34645109	GGCCAAA	1	34417696	F
Grn	GCATGTG	37	5	135416412	GATGCGC	5	135444311	F
	CAGAAGC	37	15	71747687	CAAATCA	15	69534741	F
	ATTCTAG	37	13	43252734	CGGGATC	13	42150734	R
	TGCAACA	37	6	56507796	CGAATGC	6	56615755	R
	GGCACAC	37	15	62899159	GTGCTCC	15	60686451	R
Red	AGATGCA	37	1	9409810	CTTCCGT	1	9332397	R
Red	TTAAATC	37	8	1245703	TTTCATG	8	1233110	R
	GGGCTGA	37	6	133663589	CGGAAGC	6	133705282	F
	TCAGCTC	37	2	24344064	CGGGCAC	2	24197568	R
Grn	CGATCGT	37	20	29551622	GGCTCTT	20	28165283	F
	TCCATGG	37	16	81254719	CGGAGGC	16	79812220	R
Red	GGCCCGC	37	3	10149974	ACCTGCT	3	10124974	R
	CAGTCTG	37	3	117719971	GCAAAGT	3	119202661	F
	TGTGTGA	37	4	13922944	CGTGGCC	4	13532042	R
	CCAGGCT	37	1	3446295	CCATGCC	1	3436155	F
	ACCACAC	37	3	124531371	CGCTCCC	3	126014061	F
Red	CACAGTT	37	1	54822031	CGCCAGC	1	54594619	F
	AATTCTT	37	6	139861189	TAAAGGC	6	139902882	R
	GATATTC	37	22	41492007	CGTGTTG	22	39821953	F
	GAGGCAC	37	17	19291120	CGCCAGC	17	19231713	R
Red	TCTTCGA	37	13	113496682	CGCTCCA	13	112544683	R
	TTTTTTTT	37	6	72199637	TTCACAT	6	72256358	R
	GCCAACA	37	4	6665199	TTAAAAC	4	6716100	F
	ACAAGTC	37	5	158466226	CGGACCC	5	158398804	R
Grn	GCGGCCT	37	6	32552246	CGGGCGC	6	32660224	F
Red	CCATTGG	37	Y	21239348	CGCACGT	Y	19698736	R
	CGCGAGT	37	X	129300008	GCACAGT	X	129127689	R
	CTGCTGG	37	12	31255279	TCCAGGT	12	31146546	R
Red	ACCCCTT	37	6	30652268	GGCAGCT	6	30760247	R
	CTGGCAG	37	12	122233375	CGGCAGC	12	120717758	F
	AAATATA	37	3	64307530	TCTGAAA	3	64282570	F
Grn	CTCTGCA	37	12	1609346	GCCCGGC	12	1479607	F
	AGTCTTT	37	10	120516119	CTACTCT	10	120506109	F
	AATTCTT	37	17	928957	CGGGGGC	17	875707	R
	CTAGGGT	37	1	36021674	TGTGTTC	1	35794261	F
	TCACAAT	37	15	22904606	AGGCTGC	15	20456047	R
	AGGGGAC	37	8	1339852	AAGTGAC	8	1327259	F

Red	AATAAAT	37	10	26931912	CGGCAGC	10	26971918	R
Grn	TCCCCAA	37	X	12993156	CGGGCCC	X	12903077	F
	TACTGGC	37	3	129116393	TGAACAT	3	130599083	F
	GACGGGA	37	17	77922972	GCTGGAC	17	75537567	F
	TCATGTC	37	4	1986542	CGCCCCC	4	1956340	R
	TCAGAAT	37	1	150779776	CGCCTCG	1	149046400	R
	GTGGCCT	37	18	47016218	CGAAGTC	18	45270216	F
Red	CGTCTCC	37	12	126346041	CCGTTTC	12	124911994	R
	GCTAAAT	37	2	128569347	CGTCCAC	2	128285817	F
	CCCATCT	37	1	217306763	ACCCAGA	1	215373386	R
	GCAGAGA	37	17	724374	CGGGCCA	17	671124	F
	GACAGCC	37	8	4183880	CGACGAT	8	4171288	F
	AAGCCGC	37	6	156983315	AATTCCT	6	157025007	F
	CAAAGCC	37	5	140563151	CGTCCAC	5	140543335	R
	TTCCTGT	37	Y	14649908	CGGACAA	Y	13159918	R
	ACTTGTA	37	21	46220161	CCACCTA	21	45044589	F
	GGCTAGA	37	16	28300253	CGGGAGC	16	28207754	F
	GAGGAAA	37	16	88238863	CGTTGGC	16	86796364	F
	GCTCCTT	37	17	39222690	CGTGTGG	17	36476216	R
	TAGAAAA	37	11	103584918	CGGTCTC	11	103090128	F
	GCTCTGG	37	2	231855218	CGGCTGC	2	231563462	R
	TGACGGC	37	20	61047376	CGCTCAG	20	60480771	F
	CTGAATG	37	8	131453955	ATTCATC	8	131523137	R
Red	CTGTGAC	37	8	141555295	TAGAAGC	8	141624477	F
Red	TGTCGTC	37	11	67383545	CGCCGTC	11	67140121	R
	CTTCCGG	37	22	38071168	GCCCGGC	22	36401114	R
	TCGTGCC	37	10	62144786	CGGATGC	10	61814792	F
	CTTTCTT	37	8	2021420	GCTGGGC	8	2008827	R
	GGGTGGC	37	9	90620271	CGGAATC	9	89810091	R
	AGCAACC	37	19	50861774	AGGTTCG	19	55553586	R
	GCATGCA	37	15	22442000	CAGTTGA	15	19943364	R
Red	AGTGGTG	37	1	161582581	CGTTTCG	1	159849205	R
Red	TGCTGAA	37	1	235099120	CGCGCTG	1	233165743	F
	CTCATTC	37	2	61977683	TTCAATA	2	61831187	R
	CTGGCAG	37	4	190230730	CGGGCCC	4	190467724	F
	TGAGGCC	37	1	247803166	CTATAAT	1	245869789	F
	CCAAGGC	37	17	15340112	CCTCTCA	17	15280837	R
	CCCTCTG	37	10	135341933	CGGGGCT	10	135191923	R
Red	CAGTGAC	37	16	89152443	CGGGAGA	16	87679944	R
Grn	ACCGCCG	37	11	58736081	TGTTTGC	11	58492657	F
	ACAGCAT	37	6	57178623	AGTTGCC	6	57286582	F
	AGATTTG	37	1	169671116	CGATGAC	1	167937740	R
	GAACAAA	37	8	29033680	TTTGTAA	8	29089599	F

	GCCTGGT	37	1	236915027	CGGGGA	1	234981650	F
	CAAAATC	37	6	37682596	CGGCTGC	6	37790574	F
Red	GTTTCTC	37	4	155254194	CGACTGT	4	155473644	R
Grn	GAACTTT	37	1	8377787	AGCCTGT	1	8300374	F
Red	AAGCCTT	37	6	38202492	CGTCTTG	6	38310470	F
Grn	GTCCGGC	37	12	115109730	CGGCCAC	12	113594113	R
	GGGATCC	37	18	77236278	GCAGCTG	18	75337266	F
	CCCACCC	37	9	135454197	CGGCTTT	9	134444018	R
	CTATCTG	37	1	156974025	CGAGAG	1	155240649	R
	GGAGGA	37	11	57195187	CGCCCCC	11	56951763	F
	GGAGAA	37	19	51017311	CCTGTGC	19	55709123	R
	CTCCCCC	37	6	32186049	GCTGTCT	6	32294027	R
	TCTCTAC	37	16	16963007	CTCATAT	16	16870508	F
	GCCCCTT	37	19	58609269	CGGGGTG	19	63301081	F
	GGAAAG	37	2	10501297	CGCCTGG	2	10418748	R
	GCCTTAA	37	3	111721343	CGGTGTA	3	113204033	R
	TGACAG	37	15	24722944	AGGGCG	15	22274037	R
Grn	GCGTCCC	37	16	30572815	CGGCAGT	16	30480316	F
	CCGCCTC	37	3	13974800	GAAAGG	3	13949801	F
	AAGTGG	37	6	29908976	CGTAGCA	6	30016955	F
	CCTAGAG	37	1	145589534	TAATATT	1	144300891	F
	GAGGAG	37	4	178364342	CGTGCGA	4	178601336	F
	TAGTCCA	37	7	8476098	GTACAAA	7	8442623	F
Red	TTGGAGA	37	5	16509323	CGCCTAG	5	16562323	F
	AGAGAG	37	6	33040769	TACAGAA	6	33148747	R
	CGAAACC	37	7	140135611	AAGCTGC	7	139782080	F
	GCTGATC	37	8	2178551	CGAGTCC	8	2165958	F
Grn	CTAGGTC	37	X	100807848	CGCGCTT	X	100694504	F
	CTTGCAC	37	8	23712355	CGGCTAT	8	23768300	R
	GGGCTGC	37	1	2847213	CGAGGCC	1	2837073	R
	TGTAAAA	37	7	138162679	CGGAAA	7	137813219	F
Red	ACTTAGC	37	2	119887680	CGCAAG	2	119604150	R
	GTAAGAT	37	2	220197716	TCAAGGA	2	219905960	F
	GCCTTTT	37	6	32493873	TGATCCA	6	32601851	R
	AGAGAA	37	8	599525	CGGGGCC	8	589525	R
	CCACCTG	37	10	49348564	TCAGTGC	10	49018570	F
	AGGTGA	37	2	208199465	TTCCTAT	2	207907710	F
Red	CCCTGCG	37	2	176936581	CGCCCAC	2	176644827	F
	TTCACTG	37	1	64434320	CGGGGG	1	64206908	R
	CCCAGAA	37	1	59221344	AGGGGC	1	58993932	F
Red	GCGGCTG	37	19	1120191	TCCACGA	19	1071191	R
	GCGTCGG	37	17	76676366	CGGTCTT	17	74187961	F
	TCTATGA	37	12	19604192	CGAGCTG	12	19495459	R

	CACTTCT	37		2	1625268	CGGACCT		2	1604275	R
	ACTTGCA	37		19	40904851	GGGATA		19	45596691	F
	TGCTGGA	37	Y		21036986	CGGTAAC	Y		19496374	R
	GCTTTTT	37		4	152096720	CGGTTCT		4	152316170	F
Grn	GTTCCTC	37		19	41531916	GGTATCC		19	46223756	F
Red	ACTCAAA	37		2	48844908	GGGAAA		2	48698412	F
	CAAATTA	37		7	65610409	TGTGCAG		7	65247844	R
	ATTTTTC	37		3	150826472	CTGTGGC		3	152309162	R
	ATATGAG	37		2	26701355	CGCCCTT		2	26554859	R
	TGCATTT	37		13	36049807	CGCTGGT		13	34947807	F
	AGTCACA	37		6	44119147	GGCATCT		6	44227125	R
	GGCTCGG	37		16	67686943	CGGCTCC		16	66244444	R
	GTGGGGT	37		7	106505961	TCGGAAT		7	106293197	R
	GCTCCTG	37		16	67918840	TCACCGA		16	66476341	F
	TTTACTT	37		6	32223341	CAGAACA		6	32331319	R
	AAGGCGC	37		6	164137946	CGTGGCC		6	164057936	R
	GATGAAA	37		10	3894968	CGTGAGT		10	3884968	F
Red	CCCTTCT	37		16	88094415	CGCGTGC		16	86651916	R
	AAAGGAT	37		1	209607166	TAACTAG		1	207673789	F
	CCTTATG	37		4	56274131	CGGCGTG		4	55968888	R
Grn	ACGCACC	37		16	89167792	CGGTCAC		16	87695293	R
	CCTGGGA	37		6	43737136	CAATGTG		6	43845114	F
	GGCAGGA	37		9	139616839	GGGATGC		9	138736660	F
	CACATAA	37		1	118239410	CGGCTCA		1	118040933	F
	CTCCTCG	37		5	170845058	GTTTGTC		5	170777663	R
	GGGGCAC	37		16	87616248	GTGCTGG		16	86173749	F
	ATCACTG	37		3	195578280	AGATGTT		3	197062677	F
	ATAATTT	37		12	49763288	GGTCACA		12	48049555	R
Grn	TGTCCAG	37		19	54899778	GGGGCAC		19	59591590	F
	AATTCAA	37		5	9765708	CGCCCAC		5	9818708	F
Red	GGGCCCA	37		1	209405064	CGCGCCC		1	207471687	F
	CTGGGAC	37		1	230250356	CGAGGTC		1	228316979	R
	CTCCATG	37		11	6590092	CGCCCCC		11	6546668	R
	TCTCTAT	37		8	103138381	CGTCACC		8	103207557	F
	CCTGGTA	37		2	65355269	AGAATGC		2	65208773	R
	TGTTGCA	37		7	104881282	AATATGC		7	104668518	R
	CCCCCGT	37		17	17109740	CGCCAGC		17	17050465	F
	TTGTGAG	37		2	879464	CGGGGTC		2	869464	R
	AATCAAC	37		5	169548502	CGGCACT		5	169481080	F
	AAAGTTC	37	Y		6312218	CGGACTG	Y		6372218	R
Red	AGCCTGC	37		10	129949058	TCCATCT		10	129839048	F
	TTCAAAA	37		6	156717406	CGGTCTG		6	156759098	R
	AGATCCA	37		22	41983798	AGGCCTG		22	40313744	R

Red	GGAGCTC	37	13	114741241	CGGGCCT	13	113759343	R
	TACAAAC	37	8	72917147	TAAATCC	8	73079701	R
	ATATTTG	37	4	83955429	CGCAGGC	4	84174453	F
	GGATTCG	37	17	79961522	CGGGGAA	17	77554811	R
Red	TTCGAAA	37	5	74908170	TCCAGCC	5	74943926	F
	GCTGGAT	37	2	153468347	CAGGGCT	2	153176593	R
	CGTGATT	37	16	10772249	CGGCCTC	16	10679750	F
	TAACAGC	37	7	134250149	GAAGTCT	7	133900689	F
	CGCCTCC	37	19	50837025	CGCTCTG	19	55528837	F
	GCGTGGA	37	6	31124550	TCAGAGA	6	31232529	F
	TCCCCAG	37	6	26305371	GAAAACT	6	26413350	F
	GAGCACT	37	5	178549790	CGCCAGC	5	178482396	F
	TATTTTC	37	12	94640673	AATTGCC	12	93164804	R
Grn	TGTGAGG	37	13	112166963	GATAATC	13	110964964	R
	TTTCCTT	37	18	5193969	CTGGTAG	18	5183969	R
	CCCTTCA	37	20	44168629	CGACTTA	20	43602043	R
	CCCAACC	37	17	48267062	CGGCCAC	17	45622061	F
	TCCTCAC	37	15	89786496	GAAGGCA	15	87587500	F
	AGCCACC	37	4	182862370	GATCATG	4	183099364	R
	AATGCGC	37	16	57149847	AGGGGTC	16	55707348	F
Grn	GCGGTGA	37	1	59042065	CGCACGC	1	58814653	R
	AAGGTGC	37	13	100651721	GAAGAAA	13	99449722	F
	TTCCCAG	37	10	134066136	AGGACAC	10	133916126	R
	CGAACTT	37	4	189330030	CTTCCTG	4	189567024	R
	ACCATGC	37	Y	17568445	ACCCAGC	Y	16077839	F
	AGTGGCC	37	17	78958651	GCCAATG	17	76573246	F
	CCCAGGC	37	18	76073021	AGGGTTG	18	74174009	R
	GCTGTAG	37	6	142007733	CTCAGTG	6	142049426	F
	CAATTGA	37	10	63240299	ACTATGG	10	62910305	R
Red	GATGTGT	37	1	113285940	GGAGGTA	1	113087463	R
	CCCGGCG	37	10	4868386	CACCAGC	10	4858386	R
	TCCAGAA	37	9	132458129	CGGGAAA	9	131497950	F
	GGCTGCT	37	7	1787944	CGGCTGC	7	1754470	R
Red	GCTGACC	37	2	239889410	CGCCAAC	2	239554347	F
	GAAGAAT	37	4	1941849	CGCAGAC	4	1911647	R
Grn	GCCACTT	37	7	31006	GGAAAAA	7	126078	F
	CTGCTAA	37	8	2337813	GACCATT	8	2325220	R
	CAATTTG	37	15	101084442	TAGAAAC	15	98901965	R
	TAGTCAG	37	7	80352237	CGCAGCA	7	80190173	R
Red	TCCCGAC	37	X	106567457	CGCCCCC	X	106454113	F
Red	TTCCCGC	37	4	190906046	CGCCCTA	4	191143040	R
	CAACACT	37	22	43811071	ACGAGGC	22	42141015	F
	AGGGACT	37	2	186727863	CGCAGAT	2	186436108	R

	GCAGAAA	37	17	31340147	CGTGCTT	17	28364260	R
Red	TTAGGTC	37	16	10856534	CGGCTGC	16	10764035	F
	ACTGAGT	37	13	80755568	CGCGTGT	13	79653569	R
	TGCTGTG	37	12	45703832	AATAAAT	12	43990099	F
	GCGCACC	37	9	137298813	CGGGTGC	9	136438634	R
	CACCACA	37	15	72530044	CGGAAGC	15	70317098	F
	TGGTATC	37	7	158032120	CGGAGCC	7	157724881	R
Red	CACATGT	37	16	88990161	CTGCTGT	16	87517662	R
	CTCACGT	37	16	31343428	ACTCAGA	16	31250929	R
	AGGTAGT	37	1	8083892	AACGCAC	1	8006479	F
	AGGGCTC	37	12	10954529	TGGGCTT	12	10845796	R
Red	CTGGACT	37	16	1052756	GACGGCC	16	992757	R
	GTAAATG	37	X	103498585	CGTATCA	X	103385241	F
Red	GGGGAGC	37	1	20915592	CAGGACC	1	20788179	F
	TTGGCAG	37	1	248100591	GTGTGTA	1	246167214	F
	TATTACA	37	6	30072616	GCAGCTC	6	30180595	F
Grn	GGGGCGC	37	6	159360761	GCAGCAC	6	159280749	F
Red	TGAAAAC	37	2	234847769	CGCGCAT	2	234512508	F
	ACTGTAT	37	1	248636313	AAGTAGT	1	246702936	R
	GGCCTTT	37	6	166260532	CGGCAGC	6	166180522	F
	TCTTGCT	37	16	23916466	ACTTACT	16	23823967	R
Red	GCGGCTC	37	14	106094144	CGGCCCT	14	105165189	F
	AGCAGAC	37	14	37445440	CGGTAGC	14	36515191	F
	GGCGACT	37	11	13986348	CGGGTAT	11	13942924	F
	ATTCACG	37	1	248790981	GACAAGA	1	246857604	F
Red	AGCCGAT	37	1	6086018	CGGCACA	1	6008605	F
Red	AGGTCCT	37	1	49364657	GTCCAAG	1	49137244	F
	CCACTAA	37	X	35937697	TCACAGG	X	35847618	F
	GCTACCA	37	15	39876248	CGCACTA	15	37663540	F
	TTAAAAA	37	16	86326446	CGACCGT	16	84883947	R
	TGCTCCT	37	1	146552329	AACGGGA	1	145018953	R
	GCGCAGC	37	8	10584169	GGCACAC	8	10621579	F
	GCTTGTT	37	1	215179216	CGGCATT	1	213245839	F
	CTGGCCG	37	1	27676652	GGACGAC	1	27549239	F
Red	CCACGAC	37	18	76725495	GAACGGA	18	74826483	F
	TCTGCTT	37	2	234847683	CGAGCTG	2	234512422	F
	GGAGGAC	37	7	158246263	CGGCAGC	7	157939024	R
	GCCTGAT	37	1	207849914	CCCAATC	1	205916537	F
	AAAAGTC	37	11	67383662	CGATGGC	11	67140238	F
	GATGATC	37	19	57179660	TGTGACG	19	61871472	F
Red	CCCTCTC	37	12	116044025	GTGCCTT	12	114528408	R
Red	TGGGTGG	37	16	33483591	AAAGCGC	16	33391092	R
Red	GCTTGGC	37	17	56565644	CGGGCCC	17	53920643	F

	ACAGTCA	37		19	38039666	CGGCCTC		19	42731506	F
Red	CCTGGCT	37		19	5909063	TGTCCGC		19	5860063	F
	ACCCTAC	37		14	52466952	GGGGTTA		14	51536702	F
	TAACACT	37		2	166930521	TTCCTTT		2	166638767	F
	CAGCCCT	37		14	105293199	CGCCCTC		14	104364244	R
	CTTACCT	37		1	152196674	CTAAGAC		1	150463298	F
	GGTTTTC	37		3	177451285	AACATCA		3	178933979	F
Red	TGCTTGG	37		6	32847704	CGCGTCT		6	32955682	R
Red	ACTCTTA	37		17	73316505	CGGGCAC		17	70828100	R
	CATAAAA	37		2	230675880	CGCCTAG		2	230384124	R
Grn	CCTCAAA	37		7	27206907	TGACACA		7	27173432	R
	CTTCTCG	37		2	857746	AACCTGA		2	847746	F
	GCCCTTT	37	X		70323289	CGCCTGG	X		70240014	F
	TGCCCAA	37		1	36852956	CCCTTCT		1	36625543	R
	ACTGACA	37		18	11947875	CGGCCAA		18	11937875	F
	CGGGATC	37		4	187761401	CGACAGT		4	187998395	R
Red	TCCAGGC	37		1	207991122	CGTCCAT		1	206057745	F
	TGCCCAA	37		2	48962273	CGCAGAC		2	48815777	R
Red	CATTACA	37		14	99613313	CGCTTGG		14	98683066	R
	AAACCAA	37		10	16933505	CGGGCCA		10	16973511	F
	CCCATCA	37		22	20464947	CTCATTC		22	18844947	F
Red	CGCCCAG	37		6	30039142	CGGCCCA		6	30147121	F
Red	AGGAGTC	37		21	46237117	CGCGCCC		21	45061545	R
	ATCTTCA	37		21	28215206	AGAAGA		21	27137077	F
	TGGCGCC	37		12	8025861	TAGGAGC		12	7917128	R
	CTGGGCT	37		7	150093664	GTGCTGC		7	149724597	F
	TGAAACC	37		4	121991345	CGCTCAG		4	122210795	R
	CACACTC	37		1	19810215	AACTCCT		1	19682802	F
Red	ATACAGA	37	X		101854294	CGGAATT	X		101740950	F
Grn	GTCCTAC	37		6	31797416	ACGAGAT		6	31905395	F
	CTGCAGA	37		16	90124400	GGCTTCT		16	88651901	R
	GAAGGCT	37		16	88990188	ATGGAGC		16	87517689	R
	AAGCTGC	37		1	161409970	AGTGGTG		1	159676594	F
	TTCATAG	37		6	32605043	CGGGGCA		6	32713021	R
Red	GATAAGC	37		5	140475652	CCAGCAC		5	140455836	F
Grn	CTGTAAA	37	X		39680221	AAATACC	X		39565165	F
	ACACATT	37		15	45493530	CGGACAA		15	43280822	F
	AGATGAT	37		1	217314284	CAGGGAA		1	215380907	F
	GCTATTT	37		6	16690986	GCTCCAG		6	16798965	F
	GACCGGC	37		3	183952212	CTGCACT		3	185434906	F
Red	CAGCTTC	37		14	106374554	AAGAAGA		14	105445599	F
	GACTTCT	37		8	98956478	GACCCCT		8	99025654	F
	CTGCATT	37		7	129499233	CGGAACA		7	129286469	F

	AGACTGA	37	14	103862143	CGGAGTC	14	102931896	R
	GGAAGCA	37	6	32627885	CCTGTGT	6	32735863	F
	TACTTAT	37	5	142444988	TACATTG	5	142425181	R
Grn	CCGCTGT	37	1	47799638	CGGGCTG	1	47572225	F
	TAATACT	37	12	66139263	CGGGAGA	12	64425530	F
Red	TTCAGGG	37	19	24270468	GGGAGGC	19	24062308	R
	CGCACTC	37	4	3669900	CTTGGAC	4	3639698	R
	TGGCTAA	37	X	55935071	TTTACAC	X	55951796	R
	CATCTCA	37	7	151384763	ACCTGAG	7	151015696	F
	TTTTTTTT	37	13	95309323	TTTTCTTT	13	94107324	R
	CACGGTC	37	3	13246006	AGGCAGC	3	13221006	R
Red	GCGCTCT	37	16	2892766	AGGTTGA	16	2832767	R
Grn	ACGGACA	37	1	201488725	GGATTAC	1	199755348	F
Grn	GTATTCG	37	1	40598394	CGCCTCT	1	40370981	R
	TCTGGAT	37	18	43305784	CCACTCT	18	41559782	R
Red	CCAGCAC	37	1	201258280	GAATACC	1	199524903	F
	TCCAAGT	37	1	245314273	CGCCTCG	1	243380896	F
	GGGGCCC	37	10	729711	TGGTGAC	10	719711	R
	TCTTCTC	37	1	221056674	CGAGGAC	1	219123297	F
	GGGGGCC	37	16	1393766	CGCTCAG	16	1333767	R
Grn	TCTGTTT	37	5	4101132	CTGTTGG	5	4154132	F
Grn	AGCTACA	37	3	195709661	CACATTA	3	197194058	R
	TATGAGT	37	5	140226474	GTATTTC	5	140206658	F
	AAGGATC	37	3	195849513	AAGAAGC	3	197333910	R
	GGGTTCG	37	16	27487047	GCCTTTC	16	27394548	F
Red	TTGTTGG	37	5	140242159	CGCCAGC	5	140222343	R
	AACTGTT	37	17	80169878	AACCATT	17	77763167	R
	TTCACTT	37	3	115376349	CGGACTA	3	116859039	F
Grn	GGGCGGT	37	6	164255524	GGTGGCC	6	164175514	F
Grn	GCAGGCC	37	1	17216009	CAGGGCC	1	17088596	R
	ACTCTGC	37	1	54163070	CGGTGGC	1	53935658	R
	TCTACGG	37	22	42394853	CAGGAGC	22	40724799	R
Red	GGGCGAC	37	1	45279684	CGAGTTT	1	45052271	R
Red	ACGAGCT	37	6	170686343	CGCAGGC	6	170528268	F
	TGCCCTT	37	Y	9478342	CGCCTGG	Y	10088342	R
Red	AAAAAAT	37	16	23765258	CGCTTTA	16	23672759	F
	AGTACTG	37	19	15568935	CGGCCCC	19	15429935	R
	AAATCGT	37	1	162760584	TTCCGAA	1	161027208	R
Red	CGGCCTA	37	16	55363058	ATGCAGC	16	53920559	F
	CCGCATG	37	16	85620244	TGATACT	16	84177745	F
	TGCATCG	37	16	17516862	CGGGGAC	16	17424363	R
	GTGCGGT	37	15	40268610	CGGCACA	15	38055902	R
	AATCCTA	37	4	169535985	CGGTAAA	4	169772560	F

Red	GCCGCGC	37	6	33378345	AGGGGAC	6	33486323	R
	CCACGCT	37	5	140564161	CGGCCCC	5	140544345	F
	CGGGCCC	37	2	44313980	ATTTCTG	2	44167484	R
	TGTCTAC	37	2	240723304	GCTGGAA	2	240388241	R
	ATGAAAC	37	7	96899839	TGAGGTC	7	96737775	R
	TATTGCC	37	X	24169878	TACTAAG	X	24079799	F
	TTTCAAT	37	8	15094114	CGGGACA	8	15138485	F
	TCTTACA	37	14	52816915	CGCCTCT	14	51886665	R
	GCAGTGT	37	22	32599511	CGCACCG	22	30929511	F
	CCACCAG	37	9	129377854	ACTTCCG	9	128417675	R
	AGCAGGA	37	10	115465924	GATCTAT	10	115455914	R
	GGCCCGA	37	7	2883003	CCTGCCT	7	2849529	F
	ATTTCTC	37	15	49277592	CGTGGGA	15	47064884	F
	AGTTCCC	37	11	106239635	CGGTGTT	11	105744845	R
Red	TTGCTGG	37	16	818807	CAGGGGC	16	758808	F
	ACTAAGT	37	14	101539195	AAATGAC	14	100608948	R
	AACTTTA	37	12	32437813	CGCGAAC	12	32329080	R
	GGCATAC	37	17	41521676	CGAACAA	17	38877202	F
	TCCGCAG	37	10	118387327	CGCCCAG	10	118377317	R
	CTTTCGG	37	4	183601697	CGGTGGA	4	183838691	R
	CTTTGTG	37	6	158053980	TCTGTTT	6	157973968	R
Red	GCTGGGC	37	20	62221766	CGGCCTC	20	61692210	R
	AGGCTGA	37	22	37593672	GAAAAGC	22	35923618	R
Red	GGGACAA	37	7	56855195	CGCCTCC	7	56822689	F
Red	GTCCTAT	37	14	101175302	CGTCACG	14	100245055	F
	GTAATAT	37	11	127007896	CGCACGC	11	126513106	R
	GCCCGGC	37	4	1186469	CAAAGAC	4	1176469	R
Grn	TCTCAGT	37	11	85645743	CGGCCTT	11	85323391	R
	AGCTCCC	37	6	31095800	GCAGGGC	6	31203779	R
Grn	TCTCGCT	37	X	129254804	CAGGTGC	X	129082485	F
	GGGATGC	37	14	105695807	CGGGCCC	14	104766852	R
Red	ACCAGAC	37	5	140553091	CGCCCTC	5	140533275	F
	ACAGTGC	37	2	19549980	GGGGCCA	2	19413461	R
	ATTCTCA	37	15	65032199	CGGTCAC	15	62819252	F
	CGACCCC	37	2	241611991	GCCCGAC	2	241260664	R
	GTAATGA	37	2	52759434	CGGGTGT	2	52612938	R
Grn	GACGGGA	37	16	2867446	CGCCGCC	16	2807447	R
	TATGTCA	37	20	11869467	CAGAGTT	20	11817467	F
	TCATGCT	37	11	101313190	AGAATAC	11	100818400	F
	TGAATGA	37	5	140563213	CGCCTGA	5	140543397	F
Red	TCCCTCT	37	12	119592035	CGCGCCC	12	118076418	F
	ACACCTG	37	12	122116709	TGCTTCC	12	120601092	F
	CGGGCGC	37	11	1262311	CGGTCAC	11	1218887	R

	CAGGGAT	37	7	2582200	CGTCTGG	7	2548726	F
Grn	CGAAGAC	37	7	150020401	CGCCCAC	7	149651334	F
	TCCGGAG	37	5	140568075	CGGTTTC	5	140548259	R
	CCGGCTC	37	5	1856932	CAGGCAC	5	1909932	F
	CACTCAG	37	12	66025635	AAACATC	12	64311902	R
	TCCCTTT	37	7	27206461	GCCCTGG	7	27172986	F
Grn	ACGCACC	37	7	56242554	CGGCCCG	7	56210048	R
Grn	GGGGGCC	37	6	30039025	CCTGGAA	6	30147004	R
Red	CTCCTGT	37	8	145938564	CGGTCTG	8	145909373	R
	CTCAACA	37	2	27887409	CGCTTTG	2	27740913	F
	AAAGCCA	37	17	65072689	CGCAGCC	17	62503151	F
	GGAATGA	37	2	241230376	CGGGCAC	2	240879049	F
	GAGAGGC	37	3	184241840	TGAGGGC	3	185724534	R
	CCGTCAG	37	2	121372064	CGCTCTG	2	121088534	F
	GCAGTAC	37	8	2075820	CAAAAAT	8	2063227	F
	CGTGCAG	37	16	21199840	CGTCGTT	16	21107341	R
	TTACTTA	37	9	126772537	GTGAAAA	9	125812358	R
	ACTGCTC	37	12	1042034	CGTACTA	12	912295	R
	CGGCTGC	37	1	2885134	CGGACCA	1	2874994	R
	GCTGGGC	37	7	25899194	CAGATTC	7	25865719	R
Grn	TTGTACC	37	X	123094675	TAACTTA	X	122922356	F
Red	TCTGTAC	37	17	75405842	CGCCATT	17	72917437	R
	CAGTAAC	37	6	152126441	TGATCCA	6	152168134	R
	GATGGGC	37	19	51866949	CGCCTCA	19	56558761	F
	AAACGTC	37	16	85123554	ATCTGAT	16	83681055	R
	AATACAA	37	20	4983091	CGGTTTC	20	4931091	R
	CAGGAGC	37	21	38580129	CCATACC	21	37501999	F
	CCTAGAA	37	6	32489203	CGCAGTC	6	32597181	R
Grn	AGGTTTG	37	7	4911022	CAGCCAC	7	4877548	F
	GGCCCGA	37	7	157260189	CGGGTTA	7	156952950	R
	CAGGAGA	37	12	26522758	CGGGCTG	12	26414025	R
Red	GTGACTC	37	21	40176597	CGGTGCA	21	39098467	R
	GTTCATT	37	5	140248564	GTTGGTC	5	140228748	R
	CTGTTAC	37	3	177182762	CGTTGGG	3	178665456	F
	TTGTGGA	37	2	33827715	AGTGGCT	2	33681219	R
Red	GCAGGGC	37	2	239925497	CGGAATC	2	239590434	F
	GAGGATC	37	22	40573054	GGGACTT	22	38903000	R
Red	GTGCGAC	37	19	49700021	CGGCGAC	19	54391833	F
	CACAGTC	37	2	190119363	CAGGGTC	2	189827608	F
	CCGGGCT	37	5	178565001	CGGGTGC	5	178497607	F
	TCTTCAG	37	3	187664092	CGCTTGC	3	189146786	R
	ATCCAGG	37	4	9479622	CTCAAGC	4	9088720	R
	GGGGCCA	37	6	34524766	CAGCAAC	6	34632744	R

	CAACCTC	37	2	136496804	CGTAAAC	2	136213274	R
Grn	TCACGGT	37	8	144810339	TGGCTGG	8	144882327	R
	GACAGTC	37	15	61091220	CGGCATG	15	58878512	R
	TCTGAAT	37	13	77090294	CCCTGAT	13	75988295	F
	TCTGCAG	37	17	57915740	CGCTTTT	17	55270522	R
	CGGCAGT	37	2	238322334	TGCCTAA	2	237987073	F
	TCCTAAG	37	2	192198841	TTCATTC	2	191907086	R
	TTCCTGT	37	3	14289162	CGAGTTA	3	14264166	R
Red	AAAGATC	37	16	87856264	CGCGCCG	16	86413765	R
Grn	TTGCGCA	37	17	17109239	CGGGTCG	17	17049964	R
	GAGGCCT	37	6	30652688	CGGGAGA	6	30760667	F
Red	GTGTCTC	37	1	3081226	CGCCTGC	1	3071086	R
Red	CGGCAGC	37	2	177017449	TTGATCT	2	176725695	F
Red	CGCCCTG	37	5	140237151	CTACTCT	5	140217335	R
Red	TTCAGCC	37	12	123259789	CGCCAAT	12	121825742	R
Grn	AAGCTTT	37	2	176936397	TTATTCG	2	176644643	R
	AAAAGCC	37	13	41593385	CAAACAT	13	40491385	R
	CTCTTAT	37	1	206729040	TGCCAGA	1	204795663	R
Red	TCAGGCC	37	7	158703106	CGCGCTC	7	158395867	R
Red	CAGGCGC	37	17	3771578	CGTGGGC	17	3718327	F
Red	CTGGTCT	37	3	133502909	CGGGTGC	3	134985599	F
	TGCGTGT	37	16	73099565	TCTCCCC	16	71657066	R
	GCGAGGC	37	3	49236800	CCCTGGG	3	49211804	R
	GCTGAAA	37	8	79885164	CGCCTGT	8	80047719	F
	AGCCGTT	37	20	52786323	AAAACAA	20	52219730	R
	AGTCTCA	37	Y	14648161	CGCTGTG	Y	13158171	F
	CCAGATC	37	4	24745718	CGCTTCT	4	24354816	F
	GGAGTGA	37	5	175299260	GAAAGGC	5	175231866	F
	AAATGAA	37	2	174827779	CGTGCAT	2	174536025	R
Grn	GTATACA	37	X	37430689	CTCCCCT	X	37315608	F
	TTCTCCT	37	4	187630905	CTTCTGC	4	187867899	F
	GGCTGTC	37	19	21264896	CGTGCCC	19	21056736	R
	GCTTCCC	37	19	54275893	CGGCACA	19	58967705	R
	CAGCGCC	37	8	72917363	GTCCGGA	8	73079917	F
	TGGGAGC	37	16	2334163	CGCACCA	16	2274164	F
	AGAAAAC	37	13	41186645	AGACTTG	13	40084645	R
Grn	CGCGGGT	37	6	29895074	CAGCACC	6	30003053	R
	CCCTTCA	37	6	29898751	CGGCAGT	6	30006730	F
Red	CCCCGCG	37	8	1431308	CGGTGCA	8	1418715	R
	GCGGGGC	37	6	29895005	CGCCCTT	6	30002984	R
Red	TCAGGCC	37	16	86411222	CGAGCCT	16	84968723	R
	CGCCGCG	37	Y	9234365	CGGTGCC	Y	9294365	R
	AGAGGTA	37	7	28447591	CGAGGGC	7	28414116	F

	AAAGTGA	37		14	59947673	AAGATCA		14	59017426	R
	AGCCTGA	37		16	4295972	TGACTGT		16	4235973	F
	CTGTACT	37		7	135433715	TCCATGT		7	135084255	R
	GTGAAAA	37		1	12893344	CGATTGG		1	12815931	F
	CATAAAC	37		1	85809159	CGCATGG		1	85581747	F
	CTTTGGG	37		5	176958396	TGCTGTT		5	176891002	R
	AAAATCC	37		4	154622943	AATGCTG		4	154842393	F
	GTGCTTG	37		19	48246266	CGACGCA		19	52938078	F
	GGCTGGC	37		5	3116792	AGTGAAC		5	3169792	F
	CCTCACT	37		19	52514445	ATCACCA		19	57206257	R
	TCCAGGG	37		5	140725147	CACCAGT		5	140705331	F
	AAACAGC	37		3	13420826	CGAGGAA		3	13395826	F
	CCGTGTT	37		3	54353711	CGCTCTC		3	54328751	R
	TATGTGA	37		5	123589203	CGTTGGC		5	123617102	R
	TTATGAG	37		11	93467095	CGTAGTA		11	93106743	R
	GCTAGGC	37		8	33342441	GGAGCTC		8	33461983	F
	AATTATT	37	X		25019025	CGGGCCA	X		24928946	F
	GCCACCG	37		22	32057065	AGCCTGT		22	30387065	R
	CTCCCAG	37		16	33734837	CGCCCAC		16	33642338	F
	TATGTTT	37		6	169857450	AAAGATA		6	169599375	R
	ATGGTGA	37		2	8829827	AGCTAGC		2	8747278	F
	GGTAGCT	37		7	150018095	CGAGGAC		7	149649028	R
	TGAGTTT	37		2	67102333	GAGGGTT		2	66955837	F
Grn	CCGGCCG	37		8	144809598	TCGGCCT		8	144881586	R
Grn	ACTGGAC	37		4	7763618	GCTCCTC		4	7814518	R
Red	TTGCCGA	37		12	115134918	CAAAGGA		12	113619301	F
	GTTTGGT	37		15	75953954	CGCATAG		15	73741009	F
	GAGGCCT	37		12	5195150	AGTCACT		12	5065411	F
	GTTCCGT	37		2	1657462	CGGACAT		2	1636469	R
	TGCGAGT	37		13	110747452	CGACTAT		13	109545453	R
	GCCTGGG	37		17	13447853	CGGCGCA		17	13388578	R
Red	GCTGCCG	37		13	111281479	TCGTCAG		13	110079480	R
	CTTGCTG	37		3	25833304	CGACCAT		3	25808308	F
	GAGTTGG	37		16	1880436	CGGTTCA		16	1820437	F
	AACATCT	37		15	39875777	CAGCACC		15	37663069	F
Grn	CTGAAAT	37		4	1202930	CGCATGT		4	1192930	F
	GCCTGTT	37		16	84743502	CGGGAGT		16	83301003	F
	TCACCCT	37		2	43521066	CGGGTCA		2	43374570	R
	TTCCAGA	37		7	98960493	CGGAATC		7	98798429	F
Grn	ATGTCTA	37		6	74063982	AGGCTTT		6	74120703	F
	GGCCCGC	37		6	30227583	CGCCTCC		6	30335562	F
Grn	GCACAGC	37		11	498864	CATCAGA		11	488864	F
Grn	GCCGCAC	37		6	35733710	CGGCTCA		6	35841688	R

Grn	CAGGTCC	37	17	8250623	ACTGCCC	17	8191348	R
	GTGAGTG	37	9	130705120	CGGCTCC	9	129744941	R
	TGTCATT	37	4	155254497	CGGCAGC	4	155473947	R
	TAGAAGA	37	7	108101162	CGTGCAT	7	107888398	F
	TCGAAAA	37	17	80246403	CGGACTT	17	77839692	R
	GCTCCAG	37	2	3315680	TGGGGCA	2	3294687	R
	ATTACCT	37	15	93629544	CGCAGGC	15	91430548	R
	TCTCTGA	37	6	1686746	TGGCCAT	6	1631745	R
Grn	ACCTGTG	37	16	15018856	CGCCTGC	16	14926357	R
	CAATAGT	37	4	146018691	ATACCGT	4	146238141	R
	CCCAGTT	37	3	75657183	CGTGGTG	3	75739873	R
Grn	CTTGGAG	37	6	101847656	CGGGGCT	6	101954377	F
	CAGTTCA	37	X	42638571	CATTGAC	X	42523515	R
Red	TCACCTA	37	16	25075152	CAGTTCT	16	24982653	R
	CGTGAGA	37	11	783889	GAAGGAT	11	773889	F
	GCGCGGC	37	22	24384245	GAGTCCT	22	22714245	F
	GCCATTG	37	17	54230996	AATGAAA	17	51585995	F
Red	GCTGGCA	37	20	43343365	CGGGAGC	20	42776779	F
	TGTGATA	37	16	51465744	CGGTATA	16	50023245	R
Red	GAAGAGC	37	8	43131353	GGCGGAC	8	43250510	R
	TTGCGGC	37	2	91634652	CAGCAGC	2	90998379	F
	TCTCTGA	37	8	1365659	CGGGGCT	8	1353066	F
	GGGGTGC	37	15	92459894	TTTCCCA	15	90260898	F
	TCAGTGG	37	2	242919814	CGCACAT	2	242568487	R
Grn	TGCGCCC	37	15	25123688	TTATACG	15	22674781	F
	AGGCCGA	37	6	28446840	CGCAGCT	6	28554819	R
	GAGTCAC	37	7	129933014	CGGGGAC	7	129720250	R
Red	CGGCACT	37	3	195578240	CGCGGCA	3	197062637	R
	TCAATAT	37	16	21740820	ACCAAAC	16	21648321	R
	ACCATAT	37	14	58762197	GCTGATA	14	57831950	R
	CTGTGAT	37	6	33041229	CTGAGTC	6	33149207	F
	CAGGTTA	37	6	32526702	CGGGAGC	6	32634680	F
	AAGTCAC	37	6	1706830	CGCAGGC	6	1651829	F
Grn	AGACAAT	37	4	4866091	TCCACAA	4	4916992	F
	ACAGAAC	37	15	48741995	AAATGGA	15	46529287	R
Grn	CGAGTCG	37	6	35754906	CGCGGAC	6	35862884	R
	AGCTTAT	37	7	1018969	CGCATTT	7	985495	R
	CCTGCTC	37	11	71276654	CCTGATC	11	70954302	R
	AAATACA	37	5	122836394	AACTATG	5	122864293	F
	GTGAAGA	37	11	121626930	TCTGTGC	11	121132140	F
	CACCTGT	37	6	73973719	TAAATTT	6	74030440	F
	CGTTAAC	37	9	100070007	CCCGAAC	9	99109828	R
Red	GAAAACA	37	14	28733613	GAAGAAC	14	27803364	R

Red	ATCACTA	37		14	106806179	ACCAGGC		14	105877224	R
	CTTGGAT	37		12	133036016	CGCCCTG		12	131546089	R
	GGGTTAA	37		17	79792936	CGTGCAG		17	77386225	F
	GTCATGC	37		16	87465786	ATTCTCC		16	86023287	F
	ATTAACC	37		5	145304100	CGGAACC		5	145284293	F
	GGGAGAC	37		6	166108110	CGCTGCT		6	166028100	F
	CTGCCCT	37		16	12156525	ATTTCCT		16	12064026	F
	ATATGAT	37		20	62439108	CGCTTGT		20	61909552	R
Grn	ACTAGTA	37		5	140480872	TAAAGCC		5	140461056	R
Grn	CGAGATC	37	X		8434367	TCGGTAG	X		8394367	F
	CAGAGCC	37		6	28863428	GTTACTA		6	28971407	F
	GGTGTCA	37		8	37005619	GCGAAGC		8	37124777	R
	CCAGGAT	37	X		76648652	CGGGGGA	X		76565308	R
	GCTGAAC	37		13	19692744	CGGGCAC		13	18590744	R
	GCCCAGC	37		8	15093686	CGGAGGC		8	15138057	R
	TCCCATT	37		7	94954059	CGGGGAC		7	94791995	F
	CCTGATC	37		5	149493008	TCACCTG		5	149473201	R
	GGCCCGC	37		2	54087008	CGGACCC		2	53940512	F
	TATTAAC	37		16	82660328	CGGGGGA		16	81217829	R
	TGATCCA	37		1	6660403	CAGCCAC		1	6582990	F
	GCTGCTC	37		7	105223849	AGGCCGC		7	105011085	F
Red	GCCGCGA	37		16	86465884	CGGGGGT		16	85023385	R
	CAGGTTA	37		8	980822	GTTTGTC		8	968229	R
	TAAACCA	37		14	94491763	CGCCTGA		14	93561516	F
	TGTCCTC	37		22	38201848	TCTTGAT		22	36531794	F
	CTGACAG	37		1	8085193	CGGTGAC		1	8007780	R
	TCTCCTT	37		6	100650602	GTAGCAA		6	100757323	R
	TGTGTTT	37		16	8995926	AACAATC		16	8903427	R
Red	TCCTAAA	37		16	11655159	AAGAGGC		16	11562660	R
	GGGGAAC	37		1	2121521	TCTCAAC		1	2111381	R
	TTCTGCT	37		16	88308391	GTTATTA		16	86865892	R
	CCCTCTC	37		1	25040257	CGTCCAT		1	24912844	F
Red	GGAGCTC	37	X		125687041	CGCGTGT	X		125514722	R
	TACTTAG	37		5	155548458	TTGATTA		5	155481036	R
	CCCCAAG	37		5	148541626	GTTTGCT		5	148521819	R
	CCTGATT	37		1	112107941	CGAGGTT		1	111909464	R
	CCTGACC	37		12	58934471	CGGAAGT		12	57220738	R
	GGACCAT	37		8	142239716	CACTGGA		8	142308898	F
Red	CTTGGAT	37		5	140480770	GTACTAG		5	140460954	F
	CGTAGGC	37		3	138725204	CGCCTCG		3	140207894	R
Red	ACGTCAG	37		1	2121724	CGGCGTC		1	2111584	R
	GGGTTGT	37		1	899182	CGGTCTG		1	889045	F
	TTTCCAG	37		12	54623795	ATTTATT		12	52910062	F

	TGTGTGT	37	1	40203715	TTTCTTT	1	39976302	R
	CCAATGC	37	2	152214027	AGTTTTT	2	151922273	F
	CTGTCAT	37	7	102783873	GCTGAGA	7	102571109	F
	GGGAATC	37	20	36932385	TGCAGCC	20	36365799	F
	AATTCCC	37	6	32829208	CTTCATT	6	32937186	F
	CATGTGT	37	6	148644988	CGCCCTG	6	148686681	R
	ATCAGCA	37	15	86270435	ACTTACA	15	84071439	F
	TCTCTCC	37	20	68396	GCCATGA	20	16396	R
	GCACTGG	37	1	24522592	CGCTTTC	1	24395179	R
	TTCATTT	37	6	30079177	CGCAAGT	6	30187156	R
	TGCCTTA	37	7	82435009	CGGAATA	7	82272945	F
	TACAAAC	37	11	84028562	TTGGATG	11	83706210	R
	CACTGCT	37	1	51986254	CGGGCAA	1	51758842	R
	TGGGTTG	37	2	190151266	CGGGAGC	2	189859511	F
	GCTCCGC	37	X	9432530	CCCCCAC	X	9392530	R
	AGGAATA	37	12	15907517	ATAGCTA	12	15798784	R
	GAACTCA	37	6	100896803	AATGTCC	6	101003524	F
Red	GGCACAT	37	17	973713	TTGCAAA	17	920463	F
	GTGGCAC	37	6	49605296	TATATAT	6	49713255	F
	CTTCCCA	37	6	33053608	ACAGTCT	6	33161586	R
	GACTGTG	37	19	46584209	CACTTCC	19	51276049	R
Grn	TTAAGCG	37	X	71351858	GAGAGGC	X	71268583	F
	AGCTTCA	37	19	49689673	CGGCTAG	19	54381485	R
	TGCTTGA	37	2	224967286	CTATTGC	2	224675530	F
	CTCAGCG	37	11	108408962	GACCTCC	11	107914172	R
Grn	TCAGCAA	37	7	2768561	AGTGCCT	7	2735087	R
	TGTGTGG	37	6	101847541	CGTCATC	6	101954262	R
Grn	AGCGCTG	37	19	12831888	CGGCCAC	19	12692888	R
	TGGCATA	37	Y	22917894	CGGAACT	Y	21327282	R
	GACACCC	37	X	95940519	CGCGCAA	X	95827175	R
	GCATTTA	37	9	103788959	CGCCATC	9	102828780	R
	GGACCGT	37	17	65487462	ATGTAAC	17	62917924	R
	AATTCCA	37	12	68704235	GGTGTGA	12	66990502	F
	TTATATT	37	5	86707074	CGGTGTT	5	86742830	R
	TGGTCAG	37	15	52331471	CGCCTGG	15	50118763	F
Grn	GGAACCC	37	3	138739874	CGTGTCC	3	140222564	F
	AGGCACC	37	3	12792283	CGACTGA	3	12767283	F
	CTCAGGC	37	1	17674864	ACCCATA	1	17547451	F
	ACACACA	37	X	70151117	CGCAATC	X	70067842	R
Red	GCCGCGA	37	17	20687681	CGCGCCA	17	20628273	F
	AAGTGCA	37	14	59198210	CGCAGAC	14	58267963	R
	TCCTATT	37	15	59904009	CGGGAAC	15	57691301	F
	GCCATTG	37	10	93174498	CTTTATT	10	93164478	F

	ATTCATC	37	1	151665715	CGAGCCA	1	149932339	R
	CATCTTC	37	1	149212475	CGAGCCA	1	147479099	R
	TCCCAAA	37	11	114165695	AAAAAAT	11	113670905	F
	CACTCCA	37	16	85163406	CGGACAA	16	83720907	F
	TCTTTCT	37	4	129212177	CCTTTTC	4	129431627	R
	AAAATGA	37	16	474430	CGCGAAC	16	414431	F
	AAGTTTG	37	9	94182501	CGTGTTC	9	93222322	R
	AGACCAT	37	10	45072520	GAAACAC	10	44392526	R
Red	GTAAAGC	37	8	143660412	CGGTTTG	8	143657414	R
	AGTATGA	37	2	192161142	CGTCAGA	2	191869387	F
Red	CCGGGGA	37	21	47010818	CGCCCCA	21	45835246	R
Red	AACCTCA	37	15	78505051	CAGCGGT	15	76292106	F
	TGTAGAG	37	11	13938802	CGTGCAT	11	13895378	R
	TAAGTGG	37	1	27284253	CGCAATT	1	27156840	F
	ACAATTA	37	3	170451961	GAGACCC	3	171934655	R
	AATGCTC	37	5	79040203	CGTGCTA	5	79075959	R
	TTCCTCA	37	10	76642824	ATTATTT	10	76312830	F
	CCTCCAG	37	4	159091837	CGCTGTC	4	159311287	F
	TCTGGAG	37	10	81320623	CGTGCAG	MULTI	MULTI	R
	CAACACA	37	11	12284949	AACGAGA	11	12241525	R
Red	CACGGCC	37	1	29189789	CCGGGTC	1	29062376	F
	GCAGTTT	37	1	12100631	CGAGGTT	1	12023218	F
	AGACTAA	37	6	29648736	CGCCCCT	6	29756715	R
Red	CAGTGGC	37	17	79459563	CGCTCAA	17	77074158	R
Red	GGGAACC	37	X	19002980	CGGGGGC	X	18912901	R
Red	TGGCGGC	37	5	5887361	TCGGTGG	5	5940361	F
	CTGAGCG	37	2	172972681	CGCCCTG	2	172680927	F
	GTTTTCC	37	6	31651094	CGGTGTC	6	31759073	F
	ATCCGCT	37	16	4001959	GTCCATG	16	3941960	F
	AAATAAA	37	6	30588193	CGAAGTC	6	30696172	R
Grn	CTGCAGG	37	8	111214984	GCGGCTG	8	111284160	F
Red	GTTCCAT	37	10	43846539	CGCGAAT	10	43166545	F
	TAAGTCT	37	17	37385773	CGCCTCG	17	34639299	R
	AGATGAT	37	6	30022807	CGGCTAA	6	30130786	R
	AAATGAT	37	8	117587078	TTCTCTG	8	117656259	R
	GCCACCA	37	21	15434350	GGGGGGC	21	14356221	F
	AGGGAGC	37	10	43577025	CCATGGG	10	42897031	F
	CCTCCCT	37	12	56114155	CGCCTGG	12	54400422	F
	TTGTCAG	37	16	20780262	CGGTTAT	16	20687763	F
Red	GGGGCGC	37	16	1276278	CGTGGCT	16	1216279	R
	ACTCCTC	37	7	148321082	CGTCACA	7	147952015	F
Red	AGCGCCC	37	21	15352848	CGCCGCT	21	14274719	F
	GCATATA	37	10	135052900	TTCTTAC	10	134902890	R

	TCACATC	37	10	27232252	CGGTAAC	10	27272258	R
	CCGCGGT	37	X	152989807	CGTGCAG	X	152643001	F
Grn	CCCGGTC	37	X	70474483	GCAGGCC	X	70391208	F
	ACACGCT	37	10	15355978	CAAGGCA	10	15395984	R
	CCCTGGG	37	3	136069371	AGAGTAC	3	137552061	F
Red	ACTCCCG	37	10	130831421	TAAAGAC	10	130721411	R
	AGAGACA	37	10	48828109	AGGGGGA	10	48448115	F
Red	AGGCACA	37	3	48694451	GGGCCCT	3	48669455	F
Grn	CAGGTCC	37	14	99641151	CACCTTG	14	98710904	R
	AGCTTAG	37	10	75576192	CGGATTG	10	75246198	R
	GAGATAC	37	15	51390542	ACACGCT	15	49177834	R
	CCATAGA	37	1	170898554	CGGTGAA	1	169165178	R
	AATGAAT	37	3	39234924	AGCAGGA	3	39209928	R
	GATTGCC	37	4	154386540	CGCTGGT	4	154605990	F
Red	CCGCAAC	37	10	52434778	CTGGGAC	10	52104784	R
	CAAAGTC	37	11	93913200	CGTTGTA	11	93552848	R
Grn	CCGCCAG	37	11	50257496	TGCCGGT	11	50214072	R
Red	AAGCCTT	37	13	112157919	CGCCGCA	13	110955920	R
	AACCCAC	37	4	87849250	CAACATC	4	88068274	R
	TAGAGAC	37	6	37616410	GCCTAGG	6	37724388	R
	TTTAAAA	37	10	15212066	ATTTGCC	10	15252072	F
	CATTCCT	37	11	134608598	CGCACAC	11	134113808	R
Red	CCGCCGC	37	X	17879424	CGCAGAC	X	17789345	R
	AGTCCCA	37	19	24270073	GGGGTCC	19	24061913	F
	GCATGGC	37	5	1246274	CCACACG	5	1299274	F
	GGAGGCT	37	5	16895511	CTGAGGT	5	16948511	R
	AAGAAAA	37	16	90148388	GGAAATT	16	88675889	F
	GGCAAAC	37	1	4036953	CGCACCC	1	3936813	F
	TACTGAG	37	17	48256402	CGATCGG	17	45611401	F
	TATGCCG	37	5	1054262	CGGCCCA	5	1107262	F
Red	TCCCGGC	37	1	1144658	GTGCCTG	1	1134521	F
	ACCCGTC	37	8	1140574	TCACCAA	8	1127981	F
	ATAAGTA	37	2	152804107	AGAAACA	2	152512353	R
	TTTGCCT	37	16	55866890	CTCTCTC	16	54424391	F
	CAACATA	37	5	32223673	ATTCCAT	5	32259430	R
Red	CTGCTTC	37	5	126409227	CTGAGAT	5	126437126	F
	GGCTTCG	37	6	31650790	CGGTCGC	6	31758769	R
	GCCCTGG	37	5	33937182	CGCCAGC	5	33972939	R
	GGGCCAA	37	7	1955432	AGTGTTG	7	1921958	F
	GGCTGCA	37	5	140710469	CGGCATC	5	140690653	R
Red	TGGGTTT	37	16	33956717	GTTTGTG	16	33864218	F
	GGTTAAT	37	11	61732658	TACCATC	11	61489234	F
Grn	TGCTTTG	37	17	1390182	CGGCCTC	17	1336932	F

	GAGCTGC	37	3	169649593	TGGTGGC	3	171132287	R
	GTGAAAT	37	13	94885861	CGCTGGA	13	93683862	R
	AGGATTT	37	5	140220686	CGGAAGT	5	140200870	F
	CTTGGCT	37	14	95731916	CTCACAG	14	94801669	F
	CGATGCC	37	2	66801399	CGAGCTG	2	66654903	R
	GCTTTTC	37	2	207931932	AAGCATT	2	207640177	R
	CTATGAT	37	Y	16945913	TCATGAT	Y	15455307	F
	AATGTCA	37	7	39646802	CGACCAC	7	39613327	R
	TACTGGA	37	19	56349119	TGGCCCC	19	61040931	R
	TAATATT	37	10	60567288	CTCATAT	10	60237294	F
	TTTCAGC	37	3	177397780	TGCCCTT	3	178880474	F
	GTGGCAT	37	Y	23561638	CGGCCTA	Y	21971026	F
	TGCCTCC	37	7	150412715	TCCATTC	7	150043648	F
	TGGTGAA	37	7	158056848	AAGCCTC	7	157749609	R
	GATGAAC	37	5	169123192	CGGCCCC	5	169055770	R
	ATGCTTC	37	10	131209556	CGCCATC	10	131099546	F
	TCCTAAT	37	16	55866842	CGCATCT	16	54424343	F
	CCTCGGG	37	6	53219672	TTCCTTA	6	53327631	F
	TAGGAAA	37	6	36887307	AGCCATA	6	36995285	R
Red	CAGTCAT	37	15	22383188	CGTGCAC	15	19884552	F
	GGGGAAC	37	5	33321127	CGAGAGC	5	33356884	R
	GCTGGGC	37	6	31650786	CGCTTGA	6	31758765	R
	TTTTCCTC	37	7	46851524	GTAAGCT	7	46818049	F
	TCAGATT	37	12	76577488	AGACAGC	12	75101624	F
	CAAGTTA	37	17	41521655	AGACTGC	17	38877181	R
	AGTCGGC	37	5	41869679	CGGGTTA	5	41905436	R
Grn	AAGGGTC	37	16	75563489	GGCACCA	16	74120990	F
	TGGATCA	37	12	93140078	GAGCCTT	12	91664209	F
	GCCATCC	37	6	150266173	CGTGACC	6	150307866	R
	AGTGTTA	37	17	13502304	TGATTAA	17	13443029	F
	TCCCTGC	37	15	89354338	ACAGGGC	15	87155342	R
	CTCAGAC	37	10	134833335	GCGGGCA	10	134683325	F
	AGCATTG	37	Y	9384707	CCGGGCC	Y	9994707	F
	GTGGCCA	37	5	140530028	CGGGTTG	5	140510212	F
Red	CCGCACC	37	16	857454	CGCCTGT	16	797455	F
	CAGGGAC	37	21	36419648	TGCAGCT	21	35341518	R
Red	GCGTCCT	37	12	75784884	GATTGGC	12	74071151	R
	CCGGGCC	37	19	10747142	GGGCCTG	19	10608142	R
	AGGTTTC	37	5	125808164	CGCTGCC	5	125836063	R
	GACCTGC	37	16	90114563	GCCTGTC	16	88642064	F
	AAATGTC	37	16	63072440	CGCCCTA	16	61629941	R
	CGGACAC	37	6	32941126	GCGCCCT	6	33049104	R
Grn	CAGTGGC	37	7	46202794	TGCCACC	7	46169319	R

	GATCTCA	37	6	32367970	GTAAGAT	6	32475948	R
	CACTTGG	37	15	44223008	AGAACTC	15	42010300	R
	CGGCGGC	37	19	39056126	CGGGCAT	19	43747966	R
Grn	GCAGCCC	37	19	39056216	GCCGGGC	19	43748056	R
	GGGGCCA	37	1	221055401	GTCCTTG	1	219122024	F
Red	GGGCGCA	37	1	226111372	CCCACGG	1	224177995	F
	ACCCTGC	37	15	60966344	ACAGAAA	15	58753636	R
	TCCACAA	37	21	28340304	CGCTCTG	21	27262175	R
	CATTGTA	37	15	74275622	CACATTT	15	72062675	R
	TCTTTGC	37	8	1203186	TGGCTGG	8	1190593	R
	TTTTTGG	37	6	149806235	CGGGGGC	6	149847928	F
	TGGATGC	37	8	6876781	CGGCATT	8	6864191	F
Grn	CCGCTCC	37	2	48844916	CGCACCT	2	48698420	F
	CTGAGGC	37	17	15083645	CGCAAAC	17	15024370	R
	TTTCTAT	37	8	22250005	CGTCACC	8	22305950	R
	CAGCCAC	37	12	106484618	CGCTCTT	12	105008748	F
	GAGTAAA	37	5	2135054	CGCAGCC	5	2188054	R
	TGGAGTC	37	20	6514844	CGTAACT	20	6462844	R
	GGAGGAC	37	8	1365502	ATAAAAC	8	1352909	F
	GAAAGAA	37	18	56117145	CGAATCC	18	54268125	F
	TCTAACC	37	12	48356074	CTTTGCA	12	46642341	F
	ATAAAAT	37	2	135594780	AGAGCAC	2	135311250	F
	ACATTTC	37	5	59072686	CGTGAAA	5	59108443	R
	CTGGAGT	37	2	39319447	GGGAGGA	2	39172951	R
	TAAACAC	37	7	157348266	GCAGTCA	7	157041027	F
	CCGCGCG	37	20	58515583	AGGCGCC	20	57948978	F
	CTCAGTG	37	12	122297408	CGGCAGC	12	120781791	F
	CATCATC	37	12	10251536	GCATGTC	12	10142803	R
	TAAGAGT	37	3	97887864	CGCCAAT	3	99370554	R
	AGGCTGC	37	12	10095902	TCCTGCG	12	9987169	R
	GTTTTTG	37	17	57918600	GTTTTTT	17	55273382	R
	TTCCATC	37	10	78319050	CGCAGGA	10	77989056	F
	CCACCTC	37	12	52206773	CGCGGCT	12	50493040	F
	GCGACAC	37	1	247802739	CGCCTCC	1	245869362	R
	GGGACAA	37	20	61731334	CTGCAGA	20	61201779	F
	ATTTCCA	37	Y	22680010	AGTTTAG	Y	21089398	F
	AAATCAC	37	10	128086694	AGCCATT	10	128076684	R
	ACAGCAC	37	2	241834907	GGACCTG	2	241483580	F
Red	CGCCCAC	37	21	18984770	CGCCGCC	21	17906641	R
	CAGCATG	37	10	130279912	GCATTAA	10	130169902	F
	TCCCCAG	37	2	37996000	TGTGTCT	2	37849504	F
	AAACAAA	37	2	229291442	CGGTAAA	2	228999686	F
	GGCGCAC	37	6	170338826	CCTGGGA	6	170180751	F

	GCATGAA	37	16	69310261	CGCTGCT	16	67867762	F
	ACCCTGA	37	5	95478736	CGTGGAC	5	95504492	R
Grn	CAAAATA	37	16	57333902	TCCTAGG	16	55891403	R
	TGGCTAC	37	16	67978429	CCTTCGT	16	66535930	R
	GGAGCTT	37	4	3043199	CGCACTG	4	3012997	R
	CGTCACA	37	1	247569605	AGAGCAC	1	245636228	R
Grn	GCAGGAC	37	5	179588673	CCGGCAC	5	179521279	F
	TGAGATG	37	1	11761416	TGCCACT	1	11684003	R
Red	GGCTGTG	37	11	5959923	CAGCTGA	11	5916499	F
Grn	ACGCGGC	37	2	231855524	GTTGCAG	2	231563768	F
	CCCCCAC	37	12	132637860	CGCCTCC	12	131203813	R
	GATTCGC	37	15	100890963	GATGCAA	15	98708486	F
Red	AGTCCTC	37	X	105421602	CGTGCTG	X	105308258	F
	ATAACTT	37	2	11674057	CGCAGGC	2	11591508	F
	CTTCAAC	37	11	41735702	TCCCTAC	11	41692278	R
	CTGGGAT	37	2	47761452	CGGCTTT	2	47614956	R
	GGGAAGC	37	10	1975000	CGTAGCT	10	1965000	F
	GAAGGCC	37	7	86337918	CGTGGCA	7	86175854	R
Red	ACTTGGT	37	11	977280	CGGCCAT	11	967280	R
Grn	GTGGCTT	37	7	154998107	CGGGGAA	7	154629040	R
	GAACCTG	37	X	39547825	GCCTGGG	X	39432769	R
	ACACTGA	37	16	87339708	CGGCAGC	16	85897209	F
Red	TATATAT	37	7	72723152	ATTTTTT	7	72361088	R
	GAGACTC	37	1	93913524	GCAGTGC	1	93686112	F
	AATGACC	37	12	72149634	CGGGTCA	12	70435901	F
Grn	GGCACCC	37	10	124578366	TCCTCGC	10	124568356	F
	TGTCTTA	37	4	7844918	CGGTTCG	4	7895818	R
	GTGTTAC	37	17	25583370	AGGAGCC	17	22607497	R
	AGAGATC	37	1	227692291	CGGCACA	1	225758914	F
Red	GGTCGGC	37	X	150067276	CGCCCCC	X	149817934	R
	TGTGGGG	37	16	87367481	CTCCAGG	16	85924982	F
	TATCATG	37	3	183382726	GCAACTC	3	184865420	F
	CAAGTTA	37	13	41593416	ACACCAC	13	40491416	F
	ACCACCA	37	15	92661549	CAGCCAA	15	90462553	R
	CCTCCAT	37	5	170024515	CGACCAT	5	169957093	F
	AGAGAAT	37	6	21033836	CGGGAAA	6	21141815	F
	GGCTGTG	37	12	95671146	CGGGCAA	12	94195277	R
	CGCAGAT	37	16	55794731	GCGGGCC	16	54352232	F
Red	GGACCCT	37	6	32938824	CGCCTCT	6	33046802	R
	GGAGATC	37	5	177548615	TCTGAAG	5	177481221	R
	CTGCGGT	37	16	87491587	CGGGTCG	16	86049088	R
	TAAGATT	37	12	83125178	CGTTAGT	12	81649309	F
Red	GGGTCCT	37	17	74002944	CGAATTC	17	71514539	R

	TGACAGA	37	2	88716767	TTTCTTC	2	88497882	F
	TGCTGTC	37	19	292245	TCACAGA	19	243245	R
	AAAGGA	37	4	13514574	CATTTGT	4	13123672	R
	CTGTAGC	37	3	129799733	ATTCACC	3	131282423	R
Grn	TCTCTGG	37	10	86016643	CGGCTTC	10	86006623	F
	GCTTTCT	37	10	671406	CGGCCTT	10	661406	R
	TTGGTAA	37	7	136989898	CGCTAAC	7	136640438	R
	GTATCCC	37	X	8696915	CGCCCAA	X	8656915	R
	TGACATT	37	15	52526871	CGCAGCA	15	50314163	F
	TCCTCCG	37	3	125473844	CGCCCAG	3	126956534	R
Red	GGAAGCC	37	1	182921851	GTCGCTG	1	181188474	F
	TTTTTATT	37	8	11664728	TGAGATG	8	11702137	R
	TATTAAA	37	15	40268421	GATTATG	15	38055713	R
	CGGCTTC	37	4	190960010	CGGGGTC	4	191197004	F
	GTAATTT	37	2	242986091	CGCCCCA	2	242634764	R
	TCCTGAA	37	16	3613509	CGACCTC	16	3553510	R
	CCAGTTG	37	11	2924899	TGGCTTC	11	2881475	F
Red	AGAATAC	37	5	140762957	CGTGCCC	5	140743141	R
Grn	AGCCCTG	37	22	19420170	CGCCCTG	22	17800170	R
	GGGTTGT	37	10	79284013	TTCGACA	10	78954019	F
	CTGAGAA	37	11	103480630	CGTGGGC	11	102985840	F
	GTGTTGG	37	16	80972238	CGGTCGT	16	79529739	F
Red	TGGCTCA	37	6	16181250	CGGCCGC	6	16289229	R
	GTCCCGT	37	6	32489963	CGTGTCC	6	32597941	R
	CAGACAC	37	11	20119229	CGGAGCT	11	20075805	F
	CCTTTCT	37	2	135214041	AGATCTC	2	134930511	R
	GGGGGTC	37	1	221057236	CGGGTGC	1	219123859	F
	TCCCTAT	37	2	178970574	CGCAAGT	2	178678820	R
	CTTTATT	37	2	38099030	CGCTGCA	2	37952534	F
	TTTGGGA	37	1	152161885	AAAGTCC	1	150428509	F
	TGGCATA	37	10	99449502	CGGGATC	10	99439492	R
	TGACAGA	37	15	78535419	CGCTGTT	15	76322474	F
Red	GTGGATG	37	5	2253242	CGCGAGC	5	2306242	F
	AGCGTGA	37	6	169470822	TCTCTAA	6	169212747	F
Red	CTAGCAC	37	3	48698519	CGCTGGC	3	48673523	F
	GGGAATC	37	19	35800589	CGCCGGC	19	40492429	R
	TTGGGTG	37	11	68625231	CGTCCTT	11	68381807	F
Red	GATATAT	37	8	28174814	GGTGTGG	8	28230733	R
	TTAAATC	37	18	32289550	CGATACA	18	30543548	R
	TGCTCCC	37	6	32606884	CGCCAAC	6	32714862	R
	GAGACGC	37	3	23243300	AACTTCC	3	23218304	F
	ATCCTAG	37	8	61626185	CAAACCT	8	61788739	F
	GTGGCCT	37	Y	9214477	ATTCACG	Y	9274477	R

	TGGAAAC	37		21	46912386	CGAGCGA		21	45736814	R
Red	GCAGGCC	37		2	233251773	CGTTCTG		2	232960017	R
	ACAGAAC	37		11	119887950	CTTTGGA		11	119393160	R
	ATTCTTA	37		2	178853377	TGAACAA		2	178561623	R
	CATGCAT	37		16	87339553	CGGGACT		16	85897054	F
	TTGGTGC	37		12	56365894	CGGTTGA		12	54652161	F
	GGAGAGC	37		17	48267239	TGTGCTC		17	45622238	F
	TTTCCGT	37		6	22113516	CGCAAAA		6	22221495	R
Red	GGGGTCC	37		10	134720057	CGCTCCA		10	134570047	R
Red	CTGCCCT	37		1	4771682	GCTGGGC		1	4671542	R
	CAATGAA	37	X		119133091	CGGTTGG	X		119017119	R
	AAGGCTT	37		8	72917432	AGTCACC		8	73079986	F
	AACGGGC	37		3	171523157	CGGCCAT		3	173005851	F
	CCGCCTC	37		1	246304208	AGATAGA		1	244370831	F
	TTAAGTC	37		16	14051717	CGGAGTC		16	13959218	R
	GCTGTTT	37		11	111312361	CGCTTTT		11	110817571	F
Red	TCCTTTC	37	X		19140359	CGCCTCC	X		19050280	F
	TCCATCT	37		20	10492319	CGCAGAT		20	10440319	F
	AGAAATA	37		17	20840716	GAGCCAC		17	20781308	F
	TGAGCGA	37		6	88757302	CGCCTAC		6	88814021	F
Red	CGGGTGC	37		19	1525453	CGCAGCC		19	1476453	F
	TCCTTCC	37		11	110225729	TCCTTCC		11	109730939	R
	GTGACCA	37		4	186061165	CGGACTT		4	186298159	R
	ATCCTAT	37		8	100885941	TTGAAAA		8	100955117	F
	TCTGATG	37		6	31382931	CGCCTGG		6	31490910	R
	CCCCAGG	37		19	55735946	GGTCTGC		19	60427758	F
	GCAGCCC	37		1	16345207	GGACAAA		1	16217794	R
Red	AGTTTCA	37		20	57427650	ATCCTGG		20	56861045	R
	CTGCGGT	37		7	51539131	GCGGGAC		7	51506625	R
	GAGGTAC	37		19	5346237	GGTGGGT		19	5297237	R
	ATCAAGC	37		8	129082086	CGAAAGA		8	129151268	R
	CGTCATT	37		16	11410036	CGCTGGT		16	11317537	R
	GCACAGC	37		1	115826274	CGCCCAC		1	115627797	R
	TCAGCTC	37		3	31118948	CGCACTA		3	31093952	R
Red	GGACGTC	37		19	19323996	TATCCTT		19	19184996	F
	ATTATTA	37		6	32605694	CGGATGT		6	32713672	F
Red	AGCTGCC	37		6	47210376	CGCCCCG		6	47318335	F
	CCTTGGG	37		6	32605283	CGGTGGT		6	32713261	R
	GTAGAAC	37		13	111281129	GTCATCC		13	110079130	R
Red	CTCATGG	37	X		43741601	CAGGCCC	X		43626545	F
	CCCATGG	37		6	74064298	GGAATGC		6	74121019	R
	CAGCAAA	37		16	66545712	CGCATGA		16	65103213	F
	AACTGGA	37		11	45734486	AGGCTGA		11	45691062	F

	GTCTCGC	37	4	2366485	GGGGTGA	4	2336283	R
Grn	CCCCAAG	37	8	145657956	GGCCCAC	8	145628764	R
Red	CGTTATA	37	17	79614795	CGCCCTC	17	77225200	R
	AAGGACC	37	4	1520151	CGGCCTC	4	1489445	R
	CTCCACG	37	1	161349006	AGCAGCT	1	159615630	R
	TGCCTGT	37	16	67687392	CGGAGTC	16	66244893	R
	CACACAC	37	12	117799749	CACAATG	12	116284132	R
	TACAATT	37	6	36515676	CGCACAT	6	36623654	R
	AATACCC	37	2	197944718	TCAATAA	2	197652963	R
	GGAAGAT	37	16	65105516	AAATAGC	16	63663017	F
	CAAGGGA	37	12	4829617	CGGCTTT	12	4699878	R
	TAAGTAA	37	16	55176084	CGTGAGC	16	53733585	R
	AACATTC	37	16	11374865	CAGGAGT	16	11282366	R
	TCTCAGG	37	6	29855338	AGGCCCC	6	29963317	R
	AAAGACA	37	11	79608291	TTAGCCG	11	79285939	R
	TGTTGGA	37	16	27437892	GGAGTCT	16	27345393	R
	CCTAGGG	37	20	36932397	CGCATGC	20	36365811	F
	ACTTCAT	37	16	53856169	AAGTCAC	16	52413670	R
Red	TGTGCGA	37	14	81879375	AGGCCGT	14	80949128	R
Grn	TTTCTCT	37	2	98357802	GCTTTCC	2	97724234	F
Red	GCTTCAG	37	1	1229351	CGCCCCG	1	1219214	R
Red	CGCCGCC	37	19	10679729	CGCAACT	19	10540729	F
Red	GAGGCCC	37	18	14748285	TTCAAGA	18	14738285	R
	TAAAAAA	37	7	55516872	GCATGGC	7	55484366	R
Red	GTGCCTT	37	14	98629206	CGGGCGT	14	97698959	F
	TAACGGC	37	6	29944670	AATTCCT	6	30052649	F
Red	GCCCTGG	37	2	202483740	GGCGTAA	2	202191985	R
	ATCCTGT	37	19	14586979	TGGGTGC	19	14447979	F
	TCCCCTG	37	15	71532066	CTACCAC	15	69319120	R
Red	GCCACAT	37	11	1947791	GCAGGGC	11	1904367	F
	TACAGTT	37	2	87040878	CGTTGAT	2	86894389	R
	GCATCTG	37	17	1975245	CGCCGTG	17	1921995	R
Red	GCCCGGC	37	8	21905599	TGGTAGA	8	21961545	F
	GCCACCA	37	11	30741136	CGGCGAC	11	30697712	F
	GCACAAT	37	2	224897425	CGTCAAA	2	224605669	F
	GCCATTT	37	20	57394000	GAATCCA	20	56827395	R
Red	GGCAAGC	37	16	88497077	ATTCTGA	16	87024578	R
	ACCTCAT	37	1	28560936	CGCCCAG	1	28433523	F
	CGGCTGA	37	5	76010658	CGCCTTT	5	76046414	R
	TGTGTGC	37	17	1553585	CGCAGCC	17	1500335	F
Grn	AGCAGCC	37	12	124929991	AGCAGGT	12	123495944	R
	AATGATG	37	1	15733844	GTCAGCC	1	15606431	R
	CATCACA	37	21	45658538	GTGAAGC	21	44482966	R

	GAAAGGT	37	6	21277954	CGGTGAA	6	21385933	F
	TGCCTCC	37	6	11702389	CGCATGC	6	11810375	F
Red	CCACAGA	37	Y	9193029	CGCATGC	Y	9253029	F
Grn	TCACCGT	37	17	1029917	CGGCGTC	17	976667	F
	CTCACCT	37	17	48268233	CGGACGC	17	45623232	F
	CCTTCAG	37	1	48173399	CGGAGAA	1	47945986	R
Red	CCCGCTC	37	20	61905353	CGGCTGT	20	61375798	F
Red	ATTGCTT	37	10	6183455	ATCCGGG	10	6223461	F
	TTGCACT	37	2	108159066	CGGCAAT	2	107525498	F
	TTCATTT	37	7	116738693	CGTGGGA	7	116525929	F
	CAGAAGT	37	8	38033655	CAGGCTA	8	38152812	R
	ATGGCGC	37	7	1976457	GTGCTGC	7	1942983	F
Red	CCTGTAA	37	22	37493737	CGGTGGC	22	35823683	R
Grn	GTAAAAA	37	12	54408664	CGAATGC	12	52694931	F
	CAGGGAA	37	4	186033378	CGCTGTA	4	186270372	F
Red	CACGTGG	37	10	133981681	GCCAGAA	10	133831671	R
	ATAATGT	37	5	140740607	CGACAAA	5	140720791	F
	GGATTCT	37	16	75232330	GACTGCT	16	73789831	R
	TCCCCTT	37	6	28557411	GATAAGA	6	28665390	F
	GCCTGTC	37	6	37598264	AGCCCAT	6	37706242	R
Red	CGAATTT	37	1	147801721	CGGAACC	1	146268345	F
	TATTGCT	37	2	80329553	CGAGGGA	2	80183064	R
Red	CACGGCT	37	6	33245490	CGCGCAC	6	33353468	R
	TAAATAT	37	1	94445292	CGGATAC	1	94217880	R
	CCGTGAG	37	19	491572	TCTGCCT	19	442572	F
	GTTGTAC	37	6	28185726	TGAAACA	6	28293705	F
	GACTCAT	37	16	15583606	AGTTTTC	16	15491107	R
	AGTCACA	37	12	125223679	AAGATTC	12	123789632	R
Red	GCGTTCC	37	13	114964125	CGGGGAC	13	113982227	R
Red	AGCGGGC	37	X	12993214	CGAAGTC	X	12903135	F
	CAGGGGC	37	7	2425741	TCCTGCA	7	2392267	F
	ATTTAGA	37	Y	15817871	AGGTCTT	Y	14327265	F
	GACACAC	37	6	150211725	CGGGGGC	6	150253418	F
	TCTCCTC	37	13	114797865	CAGTACA	13	113815967	F
	GGGCTCC	37	17	67457828	CGGGATC	17	64969423	R
Red	CATTCTG	37	1	234908226	GTCGGTG	1	232974849	F
	TCACCGC	37	13	111474862	CGGACAC	13	110272863	F
	AGACCAC	37	5	1007359	CGCTACC	5	1060359	R
	AGAACTG	37	1	92415048	CGGTTTT	1	92187636	F
	TACTTAA	37	6	166610956	CGGCTGC	6	166530946	F
	AGTGACC	37	1	227947250	CGCCTGT	1	226013873	F
	CGGCATC	37	8	1472227	AGCTGTG	8	1459634	R
	CAGGTCT	37	16	81353400	CGCTCAT	16	79910901	R

Grn	GCCCAGA	37		20	33762673	ACACCAC		20	33226334	R
	TTAATCT	37		16	24759640	CGGCAAC		16	24667141	R
Grn	CCGCGCA	37		13	102106348	CGGCGTC		13	100904349	F
	CCACCCT	37		19	1456886	CGCCAAC		19	1407886	R
	AAGTTGA	37		8	49502967	AAAGCAA		8	49665520	F
	AAGAAAC	37		8	41707736	ACAGTTA		8	41826893	F
	CTCCACT	37		17	41522069	CGGAAGA		17	38877595	F
	CGCACAC	37		7	150020125	CAATACA		7	149651058	F
	TTAAGTT	37		11	55431152	CCCAGGA		11	55187728	F
	GGGAGCC	37		8	43131535	GTGGAGC		8	43250692	R
Grn	CCACGCC	37	X		153237590	GAGATGC	X		152890784	F
	GGATGAC	37		10	69609995	CGAATTT		10	69280001	R
Red	CCCGCCC	37		6	32633015	CCCTTTC		6	32740993	F
Grn	TCCCTCC	37	X		70474012	CGCCCAG	X		70390737	F
	TGAAATT	37		1	247803637	CGGCTCT		1	245870260	R
	GCAGCAC	37		17	75315081	GAAAAGT		17	72826676	R
	ACTCAGT	37		1	177896505	CTTTGTT		1	176163128	R
Red	GCCTTCA	37		2	129494558	CGCCCAC		2	129211028	R
Grn	GGTTGGA	37		1	146550796	GCTCCAC		1	145017420	F
	CCACAGC	37		6	26615071	AGAAATT		6	26723050	R
	AGTGAAC	37		11	10331045	GGAGGGT		11	10287621	F
	GTCACTG	37		10	114635839	CGGCCCC		10	114625829	F
Red	CTCTGCC	37		16	1328562	GCTCTCA		16	1268563	F
Red	AAAAGTC	37		15	53045142	TTGGTAT		15	50832434	R
	AGGGCGC	37		19	44860820	AATTCTG		19	49552660	F
	AGAATCC	37		9	2013970	CGTAATG		9	2003970	R
	CTTGTGG	37		7	18330092	TACCCGT		7	18296617	F
	AAAAAGA	37		2	11196659	AGACGAT		2	11114110	R
	AGAACTC	37		1	110654497	CGTGCAT		1	110456020	R
Grn	GCAGGAT	37		6	25882463	CGGGGCC		6	25990442	F
Grn	CGCCGGC	37		20	57465448	GAGTAGC		20	56898843	R
	TTGCTCA	37		8	11839764	CGGGGAC		8	11877173	R
	CAGACCC	37		22	50007725	CGGGGCC		22	48393729	R
	CCATCTG	37		11	2365581	CGGACTT		11	2322157	F
	TCCCCCA	37		10	131412643	GATCGTC		10	131302633	R
	CAGAGAT	37		13	66919912	CGCAGGC		13	65817913	R
	CCCACGC	37		16	87588893	CGGACTC		16	86146394	R
	TTGGGGA	37		4	104021583	TCTTTTTC		4	104241032	F
	TGTAGAG	37		8	6913030	TCTTCTTT		8	6900440	F
Red	TGGCTCT	37		16	90038083	CGATGGC		16	88565584	F
	CCCAGAT	37		7	29519323	GGATGCA		7	29485848	R
Red	AGCTGGA	37		11	1957880	CACTTAC		11	1914456	F
Grn	CCAGTAA	37		16	46603015	CTGTCGG		16	45160516	F

Grn	CGGAGCA	37	6	30038975	CGCCTGG	6	30146954	R
Red	CCAGAGC	37	11	688091	CCGGGGA	11	678091	F
	CACAAAA	37	18	76549840	CGACACC	18	74650828	F
	GCATATG	37	3	196347230	CGGAAGA	3	197831627	F
Red	GAGGTCC	37	12	34547930	CGGGAGA	12	34439197	F
	CCCCCAT	37	6	29819602	TCCAGAC	6	29927581	R
	TGTTGTT	37	16	6844322	CGGGCTA	16	6784323	F
Red	GTTGAAG	37	16	88496788	CCGGAAC	16	87024289	R
Red	TAGGCGC	37	1	4008051	AGGGTGT	1	3907911	R
	GCGTGCC	37	10	43846574	CGGTCTC	10	43166580	F
	GGAAACT	37	15	74281983	TACTTCTC	15	72069036	F
	TATGGCT	37	3	108125523	CGCAATG	3	109608213	F
	AGTCACA	37	19	7525578	CGGGCGA	19	7431578	R
Red	GGCGGTG	37	12	52473731	CGCCGCC	12	50759998	R
Grn	GACACCC	37	5	140536920	GAAAACC	5	140517104	R
Red	GTCCCGC	37	19	50249776	GGTCCCC	19	54941588	F
	TCATCCA	37	5	140501661	CGAGGCC	5	140481845	R
	ATTGGGA	37	10	49810606	CGGGCCA	10	49480612	F
	AGTAGTT	37	13	94725495	CGATGGT	13	93523496	R
Red	CCCACAG	37	6	30039175	CGCAGCC	6	30147154	F
	CAAGGAT	37	5	140481256	AGGCCTA	5	140461440	R
	CCCAAGA	37	6	28446794	CGGGGAC	6	28554773	F
	AAGCGCC	37	11	9781412	TACATGC	11	9737988	F
	ATTTTAA	37	6	80838251	CTGCCAA	6	80894970	R
	TAGTGGG	37	13	36049380	GAAAAGC	13	34947380	F
Red	GGGGACC	37	7	55516724	AATACCA	7	55484218	R
Red	TGCAGCC	37	X	31284813	GGGGCAA	X	31194734	F
	TAGTTGG	37	1	65611925	CGGAGTT	1	65384513	F
	TATGCAT	37	4	54548670	CGACAAC	4	54243427	R
Red	CCACGCT	37	11	60534991	CGGGGCT	11	60291567	F
	ACCTACT	37	1	3153293	CGGGCTC	1	3143153	R
Grn	TTTTTGTC	37	22	16868045	CGGCTTT	22	15248045	F
	GCCTCCA	37	6	31731881	ATGAATC	6	31839860	F
	AGTGGCC	37	2	173118470	CGCACCG	2	172826716	R
	CATCAGA	37	6	47065652	CGGGAGC	6	47173611	F
	TTCATCG	37	5	178684658	CGCTGGA	5	178617264	F
	AGATGTG	37	1	207842833	GGAGGAT	1	205909456	R
	CTCAGGT	37	20	4128752	CGCCCGG	20	4076752	F
	GGAGTGC	37	22	46481099	CGGCCAC	22	44859763	F
Grn	ACGGGCC	37	15	101095730	CCTCCTTC	15	98913253	R
	CAGTTCC	37	1	19261678	CGGAGGC	1	19134265	R
	GGTAGGT	37	1	38701867	CGCAGGA	1	38474454	R
	AAAGTGC	37	22	50630972	CGGAGGA	22	48973099	R

	AGGCAGA	37		4	185677549	CTCATCA		4	185914543	F
	ACATCCT	37		2	36656950	TTCCCAA		2	36510454	F
	TATCACT	37		15	33692812	GAAATTT		15	31480104	R
	GTCCAGG	37		7	101556171	TGCTCTT		7	101342891	R
	CAGCTAA	37		10	7652712	TCACTTA		10	7692718	R
	GATGAGC	37		3	10436869	CGGAAGT		3	10411869	F
	AGGGTGC	37		16	819064	TGTGTCT		16	759065	F
	CTTGTCT	37		11	128323520	CGGATCA		11	127828730	F
Grn	TCCAACT	37		6	32805548	CGGCAAC		6	32913526	F
	GGCGGCC	37	Y		14074417	CGCTCCT	Y		12584417	F
	ACCACGT	37		1	161368947	TAGTTTA		1	159635571	F
Red	CTGGCGA	37	Y		9363365	CAACGCA	Y		9973365	R
	GTGATTT	37		17	54509207	GAGAGCA		17	51864206	F
Grn	AGGAGGA	37		15	62516405	GGCGGGA		15	60303697	R
	AACGGGC	37	X		153626979	CGGGGGA	X		153280173	R
	ATAGGCT	37		3	187704482	CGGGGTA		3	189187176	F
	GGACCCC	37		9	140247365	GTGGAAC		9	139367186	F
	CTTGAAA	37		20	21003839	CGGGCTT		20	20951839	R
	TAGCCAT	37		3	100889156	CATGGTG		3	102371846	F
	GTTCTAG	37		6	84153455	CGCCACC		6	84210174	R
	GGGCAGC	37		16	75143200	CGGGCTC		16	73700701	F
	ATACCAG	37		10	34391256	CGGGGAT		10	34431262	R
Red	CGGCCGT	37		10	77159055	AGGAGTC		10	76829061	R
	AAATTCA	37		14	67729501	TTTAGTG		14	66799254	R
	TGCAATA	37		2	213587996	CACCACC		2	213296241	F
	AGATACT	37		3	54119792	CGCTGCA		3	54094832	F
	CAACCTG	37		3	15311154	CGGAGGT		3	15286158	R
	ACATCCG	37		6	44243750	ACCAGCA		6	44351728	R
Red	ACAATAC	37		12	124939480	ATATGCA		12	123505433	R
	CCTCCCA	37		7	76094015	CTAAACC		7	75931951	F
	AGTCGGT	37		12	118313817	ATACATT		12	116798200	R
Grn	GCCTTTG	37		6	25882590	CAGACGC		6	25990569	R
	CCCTGCA	37		1	235147744	TTCACAG		1	233214367	R
	TTCTCTG	37		4	25099040	CCTTGGG		4	24708138	R
Red	CAGCGGA	37		19	42592983	TTACATT		19	47284823	R
	ATTATCA	37		5	140537884	AAGTTTA		5	140518068	R
	CGTTCAG	37		7	27163820	TGAGTCA		7	27130345	R
Red	CCTCTGC	37		21	44258195	CATGGGT		21	43131264	F
	CCTCAAC	37		12	96251912	CTGAAAT		12	94776043	F
Red	GAGCGCC	37		2	62857522	CGCCCCC		2	62711026	F
Grn	GTATAAT	37		2	239030728	CGGTCGG		2	238695467	F
	GGAGTGA	37		6	30040291	CGAGAGC		6	30148270	F
	TGGTTTG	37		5	80716263	AGTCTCC		5	80752019	F

	ATGCAGA	37	17	40700314	CGCCTCT	17	37953840	F
	CGCTGGA	37	19	50735340	CGGGGCT	19	55427152	R
	AAAACAA	37	1	16679780	CGGACTT	1	16552367	F
	AAGTCAC	37	7	75666768	CGTGCTG	7	75504704	R
	GACTTTG	37	17	10002773	CGGATCA	17	9943498	R
	ACGGATA	37	16	89337953	CGGCATT	16	87865454	R
	GCGAGGA	37	2	95999906	CGGGTCA	2	95363633	R
	GGCTGAC	37	19	39055712	GGAGCTC	19	43747552	R
	ATAACAA	37	18	33485300	CGCCAAC	18	31739298	F
	TGAACCC	37	Y	15016101	CCTCTGC	Y	13525495	F
	CAGAACT	37	20	4795621	TCACCTG	20	4743621	F
	TCCCTGT	37	19	58868858	CGGAACC	19	63560670	F
Red	GCTGATC	37	5	140595072	CGTCCGC	5	140575256	F
Grn	TCCGGGG	37	8	49427283	CGGCCTG	8	49589836	R
Red	TGGAGAA	37	3	195870092	CGGGGAC	3	197354489	F
Grn	AAGTGAC	37	4	141391200	TCAGAGC	4	141610650	F
	CACACAC	37	16	89555682	CGCCCTG	16	88083183	F
	CTGCCGC	37	1	202129894	AAAGCAC	1	200396517	F
	CCTGGCT	37	5	131762326	GGATGGA	5	131790225	F
Red	GGTTTCC	37	7	2769253	CGCAAAC	7	2735779	R
Red	GAGCGGC	37	12	54389264	TGTGTCG	12	52675531	F
	AAAGATA	37	4	96012317	CGCAGAC	4	96231340	F
	TTCTATC	37	18	76473146	AATGGAC	18	74574134	R
	CACATTT	37	10	28720006	CGGTGGC	10	28760012	F
	ATGGTCC	37	18	13916330	TTTTGGG	18	13906330	R
	AGCCCGC	37	11	9549286	CAAGGGT	11	9505862	F
	TGGCTTT	37	10	7859623	CGGATTT	10	7899629	R
Grn	GGACTGC	37	X	71351803	GATCTGT	X	71268528	R
Grn	CGGTGCC	37	18	77623598	GAGGGGC	18	75724586	F
	CCACTTC	37	2	85832389	GTACACA	2	85685900	R
Red	TGGGACA	37	6	28584103	GAGGCCA	6	28692082	F
Grn	GAGATTA	37	Y	2803070	CGGCCCC	Y	2863070	R
	GCTTTAC	37	5	1868279	CGGCACA	5	1921279	R
	ACTCTGC	37	16	88849875	GTCCCGT	16	87377376	F
	TCACGAT	37	10	43882723	CGGACAT	10	43202729	F
	ATCCTCC	37	11	95888212	ATTTACC	11	95527860	F
	CCCAGGT	37	6	31275741	AAGGACA	6	31383720	F
Red	TGCAGGC	37	10	54538505	CGCCACC	10	54208511	R
	TGAACGC	37	12	54376094	AAGGGAC	12	52662361	F
	CGTTTAA	37	18	77508326	AGCAAAC	18	75609314	R
	GGCTTTC	37	7	2071723	CGGGCCT	7	2038249	F
Red	TGGCTGC	37	6	30458102	CGGCAAC	6	30566081	F
	GCGCCGC	37	19	1071622	CGGGGCC	19	1022622	F

	GAAGGAT	37	16	67550511	CTATTTC	16	66108012	F
	GTGTTGC	37	6	159360705	GGCTGAC	6	159280693	F
Red	CCTGCCT	37	17	48263173	TTCTGGG	17	45618172	R
	GTTCAGA	37	16	55867072	GGAGGGC	16	54424573	F
	GCAGGAC	37	19	6476951	CGGCCAC	19	6427951	F
Red	GCGTCTT	37	1	248100585	AGAGCAC	1	246167208	F
Red	GAGATAA	37	16	857863	GCCTGGG	16	797864	F
Red	TCCAGGC	37	Y	22737896	TGACTGT	Y	21147284	R
Red	AGGGCAC	37	19	40421743	CGGCCCC	19	45113583	R
	CCCACCA	37	8	124688770	CGGAAAT	8	124757951	R
	CACACCC	37	1	51986130	CGCTTAC	1	51758718	R
Red	CGCCTGC	37	Y	2658057	GCCAGGC	Y	2718057	R
	CTCGGAG	37	7	37123550	AGCTTGC	7	37090075	F
Red	GCCTGGG	37	2	198650880	CGCCCCC	2	198359125	R
Red	CAGAGCC	37	20	61813415	CGGGCAC	20	61283860	F
	GCAAACC	37	11	86230664	CGCTGTT	11	85908312	F
	AGCTGTC	37	10	32229887	CGGGGGT	10	32269893	R
Red	AGTTCTG	37	12	38938054	TGGGAAA	12	37224321	F
	GCTTTTTC	37	2	69538690	CGGGAAT	2	69392194	F
	CTCCAAC	37	13	24337521	CGGGGCT	13	23235521	R
	ACAAACC	37	2	220233953	AACATTT	2	219942197	R
Grn	GAGCGAC	37	20	61447110	CGCCGTC	20	60917555	F
	CCAGGCT	37	10	17259531	AAGGCAT	10	17299537	F
	TCTGTGG	37	13	112860456	ATAATGG	13	111908457	R
	CTGTGCA	37	17	74679597	CTGGGGC	17	72191192	F
	ACCAGCC	37	4	722598	AGGAGCC	4	712598	R
Red	GCACCGC	37	6	149778058	CGCGGGT	6	149819751	F
	TGGAGGC	37	5	149493099	TGTCCCA	5	149473292	R
Grn	CTGCTTC	37	16	12192430	GTCTGTA	16	12099931	R
	CCTGGGC	37	11	4615063	CGAGCTG	11	4571639	F
	TGGGTGA	37	11	7695433	CCAGGCC	11	7652009	F
	CATCTAC	37	3	19987815	GAACTGA	3	19962819	R
	TGCACAC	37	17	77707066	TCATGTC	17	75321661	F
	CTCTGTC	37	2	27403477	GGGAAAT	2	27256981	F
	ATAGAGA	37	7	8477597	CGGGGAC	7	8444122	F
	CTTGTGA	37	11	57195350	GCTTTTG	11	56951926	F
	TCAAAAC	37	3	193506005	AAATTCA	3	194988699	R
	AGAAATT	37	6	24903167	CGGTCAG	6	25011146	R
Red	ACGGGAA	37	15	35086967	TGCCAAG	15	32874259	F
	GGGATTC	37	X	100183878	CGGGCCA	X	100070534	R
	AACAACC	37	1	112154295	CGGTTAA	1	111955818	F
	GGCAAAA	37	10	118459383	ACAGTTC	10	118449373	R
Grn	AAGGAGC	37	6	28584155	CGCGGTT	6	28692134	F

	GGGGAGC	37	10	64344052	ACTAATG	10	64014058	F
Red	TTTGGAG	37	4	183062301	CGGTCCC	4	183299295	F
	AGCCTGA	37	22	30783737	CGGCAGC	22	29113737	F
Red	AAAATAA	37	5	177387597	GCTCCTC	5	177320203	F
	GGAGGA	37	7	64034943	CGTGCTC	7	63672378	R
	TTCCAGC	37	10	4386802	CGCAAGT	10	4376802	F
	TTAATAA	37	6	29893605	CGGAAA	6	30001584	R
Red	GAGGGCC	37	12	132638107	CGGCCAA	12	131204060	R
	GTTGATA	37	5	98108198	TCCCCAA	5	98136098	F
	GAGGAAT	37	3	118491179	CGCTTTC	3	119973869	R
	GGGGCCT	37	11	132910642	TGACTTC	11	132415852	F
	TTGCACA	37	13	106756690	GTTTCTG	13	105554691	F
	ATAGAAT	37	1	208135621	CGTGCTG	1	206202244	F
	CGTTAGG	37	16	21311480	CGGATTT	16	21218981	R
	AAGAAAT	37	7	65512561	AGCTCCT	7	65149996	R
	GTTGGGG	37	22	50492970	CGCTCAC	22	48835097	R
	TTGGCCG	37	7	157996423	CCCATCC	7	157689184	R
	AGTAGCT	37	7	64451426	CTCATTG	7	64088861	F
	TGGAAGC	37	12	54385275	CCAGCTT	12	52671542	R
	TGTCACA	37	6	126074639	CGGTGAC	6	126116332	R
	TGCTCTT	37	8	11995002	AATTTCC	8	12032411	R
	GATCCTC	37	17	46591291	CTGTTCC	17	43946290	R
	TGGAGCT	37	10	45065415	TAGCCCA	10	44385421	F
	GTCCTCG	37	11	118842390	CGCGGTC	11	118347600	F
	TCTGGTG	37	10	70977210	CGGTTGC	10	70647216	R
Red	GCCTCCG	37	16	87339657	CGGGTCA	16	85897158	R
Red	CAGCATC	37	X	106871544	CAGGTAT	X	106758200	F
	CTCCAGA	37	6	32408596	TATTACT	6	32516574	F
	GCCGGGC	37	9	138377834	CGTCTTG	9	137517655	R
	GCGAATT	37	16	14380714	AAGGCTC	16	14288215	R
	CTTAACT	37	1	22143533	CGCTGAG	1	22016120	F
	CATCTTT	37	1	207840521	CGCCATC	1	205907144	R
	ATACTCT	37	7	17691191	CGTGCCA	7	17657716	R
	AGGCAGC	37	11	660455	CCTGAAG	11	650455	R
Red	CTCCCTA	37	1	147801103	CGGAGGC	1	146267727	R
	GTCTTTC	37	6	91145465	CGGCTTT	6	91202186	F
Red	GTGGCTG	37	6	30652396	GCAGGGC	6	30760375	R
	TCATATA	37	1	152333423	CGCAAAT	1	150600047	F
	CCAGAAC	37	8	145014989	CGCCAAC	8	145086977	F
Red	ACAGACC	37	1	6190318	CGGCGGA	1	6112905	R
Red	GGCCCGA	37	1	223566761	CGCTGCA	1	221633384	R
	ACACAGC	37	7	2768911	GGGGCCC	7	2735437	F
Red	GTACCGT	37	8	82530967	GGGAGA	8	82693522	F

	AAAAAA	37	1	241800323	CGCACAT	1	239866946	R
Red	CACTGGC	37	16	2749496	GGGCCCT	16	2689497	R
	GCAGAGC	37	6	3527443	CGCTTAC	6	3472442	R
	TAAAATC	37	1	212761056	CGGTTAA	1	210827679	R
	AAAGAA	37	1	222440048	AGGCTAC	1	220506671	F
	CCCCTCC	37	5	148942547	CGGCAGA	5	148922740	R
Grn	CTTAAAA	37	1	11979188	CGGGGCT	1	11901775	R
	GCGCCGC	37	8	144668756	AGGCACC	8	144739899	F
	GTTCAAA	37	11	8754370	ATGGCAT	11	8710946	F
Red	GAACTAC	37	1	182669315	CGGCGGT	1	180935938	F
	ATTAATC	37	2	186988953	CGGTTCT	2	186697198	R
	GCCACGT	37	4	190236439	CGAGTGA	4	190473433	R
	AACTGGC	37	1	184909381	CGGCACC	1	183176004	F
Red	CCCCACA	37	17	80970799	TGGACTG	17	78564088	F
Red	ACGTGAC	37	17	5019989	CGAGTCG	17	4960713	F
	ATGAGCA	37	7	104619961	CGGGCAA	7	104407197	R
	GGCCCAC	37	16	55794910	TCCGCAC	16	54352411	R
Grn	CACCAAC	37	3	197385493	CTGTGCT	3	198869890	F
	GTATTAA	37	13	47613485	CAAGCTC	13	46511486	R
	TTCTATG	37	12	102626348	TTCTGTA	12	101150478	R
	CCAGTAC	37	11	61007964	CGGGGAC	11	60764540	F
	GTGAGAA	37	6	32549361	GGAAGTC	6	32657339	R
Grn	CCTGCGC	37	1	248100427	CGGCCAC	1	246167050	F
	GGCATAC	37	10	80008319	TCCCTAT	10	79678325	R
Red	GTGGCGC	37	X	117250962	CGCTACA	X	117134990	R
	TGCGACG	37	16	27298091	CGGCCTT	16	27205592	R
	TTAGTGG	37	6	33039804	TTAAACC	6	33147782	F
Grn	AGACCCC	37	8	11538245	GTGAGCA	8	11575654	F
Red	CGCCCAG	37	19	2251061	CGTGGCC	19	2202061	F
	CGGAAGC	37	6	32606445	CTTCCTT	6	32714423	F
	CCAAAAC	37	5	140535469	ACTTTTT	5	140515653	R
	GTAAGTT	37	20	61821263	GAGAGGC	20	61291708	F
Grn	GCAGCCA	37	11	76751369	CGGACCC	11	76429017	F
	CGGGCGT	37	17	40805777	GAGCCAC	17	38059303	R
	AGTTTAT	37	11	109786133	CGTTCTG	11	109291343	F
Grn	TGGTTGG	37	5	166354050	CGCCGTC	5	166286628	R
Grn	AGCTGGC	37	16	88975960	GGACGCC	16	87503461	R
Grn	AGGTGGC	37	4	798711	GCAGCCA	4	788711	R
	AAACAAA	37	6	13915058	AAGTATT	6	14023037	F
	AGGGTCC	37	6	76147474	TGGAATG	6	76204194	F
	CACATGG	37	6	29980240	CGTGTAC	6	30088219	R
Red	CGGACTT	37	7	95026106	CGCCCTT	7	94864042	R
	CTTTCCA	37	1	48458049	TCTTTAA	1	48230636	F

	GACCAAC	37	1	56721795	CGTGGCA	1	56494383	F
	TCAGCCA	37	5	38765698	ATATGAT	5	38801455	F
	TCCCATA	37	1	27977270	CGGGGGC	1	27849857	R
	TTTTTTTA	37	1	92099858	CTTGAGC	1	91872446	F
	CTTTACA	37	15	82469848	CGCACTT	15	80256903	R
	CTCACTC	37	2	54483498	CGTGCTG	2	54337002	F
Red	TGGCGGC	37	7	1000210	AAGAGCT	7	966736	F
	CCTGTAT	37	13	19692715	CGTGTCT	13	18590715	F
Grn	ACGGCAC	37	7	4911051	CGGCCCG	7	4877577	F
	TTGAGTT	37	2	102510535	AGCTTGA	2	101876967	R
	CTTAGGC	37	4	62707027	CGTGAGC	4	62389622	R
	AAGAATT	37	4	186760378	CGGGGTA	4	186997372	F
	GGCCCAA	37	2	33951647	ACTCCCC	2	33805151	R
	GCTGTCA	37	6	131148736	CGGGGAC	6	131190429	R
	TGTGTGT	37	1	153854324	TGGAGGC	1	152120948	R
	GAAGCAC	37	5	112608004	CGGCCAT	5	112635903	R
Red	GGTGCGC	37	1	2847220	GGGGTGC	1	2837080	R
	AGGCATG	37	10	95656819	CGCAGCC	10	95646809	R
	GAACACT	37	16	88201606	CGCCCGC	16	86759107	R
	GTGGGAC	37	3	46618527	CGGCATG	3	46593531	R
	TCAGGGA	37	6	30899162	CGGCAAT	6	31007141	R
	GGATGTT	37	10	65442289	CGTCCAT	10	65112295	F
	CCAGAAC	37	11	131936494	CGCTCCT	11	131441704	R
Grn	CAAGGAC	37	1	1133248	CGCCCCG	1	1123111	F
	TTACTGG	37	7	94953770	CGCTCAC	7	94791706	R
Red	CCTCCCT	37	2	68017838	CAGACTA	2	67871342	R
Red	CTCAAGC	37	2	241719496	AATGTGG	2	241368169	F
	ATACTTG	37	2	152872424	GCCCACC	2	152580670	R
	GTGCCAG	37	Y	2655740	CGTAACA	Y	2715740	R
	ACCCTCT	37	17	146926	CGCCCCA	17	146926	R
Red	GGGAGGC	37	14	71131956	CGCTCCC	14	70201709	R
	TACCTGC	37	8	6876155	CGCCCTG	8	6863565	F
	CAGCACC	37	7	45002736	GAGTCCG	7	44969261	F
	TTTCTTAC	37	2	174057774	CATCAAA	2	173766020	R
	GAATGGC	37	16	89048704	CGGGCAC	16	87576205	R
Red	CTGGGCC	37	10	3138534	CGGCCTC	10	3128534	R
Red	AGCACGC	37	10	6183528	CGCCCTG	10	6223534	F
	AGCAACC	37	13	75980679	TCCAAGG	13	74878680	R
	ATGGGTA	37	Y	9170562	ACTACCC	Y	9230562	R
	GAAGGCA	37	14	101270817	TTCATGT	14	100340570	F
	CTCAGCG	37	5	125931362	CTTTAAT	5	125959261	F
	AATCACC	37	21	46225890	CGTGGGT	21	45050318	R
Red	AGGGAAC	37	17	70636940	AGACCTG	17	68148535	R

Red	CCAGGGC	37	5	37836246	CGACTGC	5	37872003	R
Grn	AACTTCC	37	2	119496171	ATGAATA	2	119212641	F
	GAGCCAA	37	3	149310862	TAGGGAA	3	150793552	F
	ATGAATG	37	1	217306700	CGCCCCA	1	215373323	F
Grn	TCACGTC	37	5	759544	CGCCGCG	5	812544	F
	GGCTCAG	37	21	45246164	CGGGCAC	21	44070592	R
	CAGCTGT	37	4	54729555	CGCAGGA	4	54424312	R
	AAGCTGC	37	1	184808004	CGGCAGA	1	183074627	R
	TTTAAAA	37	21	46629219	CGGCTTC	21	45453647	R
	CACCCGT	37	22	38435259	CCTGACA	22	36765205	F
	GGACCCC	37	22	22902047	CGCGGCC	22	21232047	R
	TGCATCA	37	5	55148534	CGGTTGC	5	55184291	F
	TTTTCAG	37	16	1722957	ATGTGGG	16	1662958	F
	GTAAACA	37	1	246785611	CGGTTCT	1	244852234	F
	GTACCCT	37	19	3458194	GGCCATA	19	3409194	F
Grn	GTCCGGG	37	10	119292300	CGCCTCC	10	119282290	F
	GGCTCAA	37	16	636227	CGGGCCC	16	576228	F
	AGTTGAC	37	1	94240208	CGACCTG	1	94012796	F
	AGCAGAC	37	8	98610472	CGGCCCC	8	98679648	R
	GCGCGGC	37	16	8963642	CCAGTGG	16	8871143	F
	AGGAAGA	37	6	149806273	CTGCCCT	6	149847966	F
	TTACTTT	37	17	57918500	AAACAAA	17	55273282	F
	TAAATCA	37	4	185749877	AGTCACA	4	185986871	F
	GGTGTGC	37	10	124578263	CCACTGG	10	124568253	R
	CAATTTC	37	1	230645569	CGGCCCA	1	228712192	R
	AAGAGAC	37	7	44163975	ACTTAGG	7	44130500	F
Red	CTACCTG	37	14	74194516	CGAGTGC	14	73264269	R
Grn	TTCTAAC	37	20	4665645	CGCCGCC	20	4613645	R
	ATTATGA	37	8	49892745	TTGAGGT	8	50055298	F
	TTTGTTT	37	1	205912819	CGGGGTC	1	204179442	R
	TAAATGT	37	4	16609513	TGGCACG	4	16218611	R
	GACTGAA	37	11	110627909	CGCATAC	11	110133119	F
	CTTAGAA	37	19	52023213	ACAGCAA	19	56715025	F
Grn	CCGGCCC	37	X	19140658	CGCCGCC	X	19050579	F
	TGGTCTG	37	17	16593582	CGCCAAC	17	16534307	R
	GGGTCGT	37	15	96869027	CGGCTCA	15	94670031	R
	ACCTTGA	37	2	11850727	CGTACCA	2	11768178	R
	CAAAACA	37	2	102589889	TTGGGCA	2	101956321	R
	GAATGTG	37	13	43988852	TCTTTTG	13	42886852	R
	TGCTCAG	37	22	46480891	CTGAGGC	22	44859555	R
	GAGAGAA	37	4	167021679	CGGTGAT	4	167241129	R
	GGGTTTG	37	6	44039484	CGGAGAC	6	44147462	F
	CTGTTCA	37	1	7259290	GCTTCCC	1	7181877	F

	GGAAAAT	37	16	78218445	CGGTGAT	16	76775946	R
	ACTAGCC	37	1	31136147	CGGCACC	1	30908734	R
	GACATTT	37	6	82459454	ATGCCAA	6	82516173	F
	GACACCA	37	16	1107501	AGAGGGC	16	1047502	R
	GTCATTA	37	1	94559714	CGTCAAG	1	94332302	R
Red	AGGCCGT	37	6	116886211	CGCGCCT	6	116992904	R
	AGTGAAA	37	2	85390041	AGGTTCA	2	85243552	F
Red	CCCGGGC	37	19	45849853	CTGCTGG	19	50541693	R
	TTGTAAC	37	6	44039689	CGGGCTT	6	44147667	R
Red	GGGGGAC	37	6	170554795	CGCGAGC	6	170396720	F
	TGGGGGA	37	10	72593165	AACAAAA	10	72263171	F
Red	TAAGCCT	37	11	28399977	AAAAGCC	11	28356553	R
Red	CCCTGCT	37	17	18023971	CGGGGTC	17	17964696	R
	GTTAGCG	37	16	2164019	CGGGCTA	16	2104020	R
	ACCTGAG	37	16	87234895	CTGCTCC	16	85792396	F
	ATGCTCT	37	3	185412244	CGAGCTA	3	186894938	F
Red	TGGAGAA	37	1	221055665	CGCCGCC	1	219122288	R
	CTGTCTC	37	2	237654635	TTTCAGA	2	237319374	F
Red	GCTTTCC	37	10	79596240	CGCCCCG	10	79266246	F
	TCAATTT	37	7	20010777	CGCTAAG	7	19977302	R
Red	CCACGGT	37	16	88885124	CGGCGCC	16	87412625	R
	GAATCGC	37	3	31494586	CGCCCAC	3	31469590	R
	AACTGGC	37	12	3948619	GGCCATT	12	3818880	F
	TTTAAAG	37	11	14495049	CGGCCTA	11	14451625	F
	AGTAAGC	37	11	12181467	GGACGCT	11	12138043	R
Red	ACAGAGT	37	17	78830472	CGCACAC	17	76445067	R
	GCCACAA	37	12	52762005	CGGTCAA	12	51048272	F
	TAAGAGA	37	4	54187654	CGTGGCA	4	53882411	F
Red	CTCCGGG	37	10	131770171	AGCGTCG	10	131660161	F
	GGGGCTA	37	4	76932862	CTGAGAT	4	77151886	R
Red	ATGTGGC	37	7	817454	ACAAAGT	7	783980	R
	CAAAGCC	37	5	135416205	AGTGACA	5	135444104	R
	GCAGCTG	37	4	81387148	CGGTAGC	4	81606172	F
	GTTCCAG	37	1	161334734	TATTTCT	1	159601358	R
	ATTTAGT	37	17	80860250	TAACCCT	17	78453539	R
	CAGGCTG	37	5	158086454	TTCAATA	5	158019032	R
	TAAGAAC	37	11	111312453	GTTATTG	11	110817663	R
	TAGTTAA	37	5	159203122	CGGGGAT	5	159135700	F
	TATTTTT	37	1	179322762	CGACAAC	1	177589385	R
	GCGCGCT	37	19	50931622	GCCCTTT	19	55623434	F
	ACTTACC	37	2	209010890	CGGGAAC	2	208719135	F
	GGACACT	37	1	8298617	CGCCTTT	1	8221204	R
	AGCCCCT	37	11	29358540	CGCTTGG	11	29315116	F

	GGAACTC	37		11	77299962	CCTCTTC		11	76977610	F
	GCTTCAA	37		6	86862059	ACACAAT		6	86918778	F
Grn	GGGCGCA	37		3	126261298	GCCGCTG		3	127743988	F
	GAGGCTC	37		17	27703877	CGCCTGC		17	24728003	R
Red	TGAGGAC	37		19	29218101	CGGGGTC		19	33909941	R
Red	GCTCCTT	37		11	13986162	CCTTGGC		11	13942738	R
	TGTGACT	37		1	33169558	CGCGAGC		1	32942145	R
	CCAGTGG	37		7	72982668	GCAACTT		7	72620604	F
	AAAAGGC	37	Y		9387798	CGCTGGG	Y		9997798	R
	ATTTAAC	37		13	53382856	CGTGTGT		13	52280857	F
	GAGTAAA	37		14	38071768	CGCACAC		14	37141519	R
	AAACTGT	37		13	100517027	GAGTCAC		13	99315028	R
	CTGCTGC	37		10	134460392	CGGGTGC		10	134310382	R
Grn	ACACAGC	37		19	24270007	TGGACAA		19	24061847	R
	AAAGGGA	37		19	54216680	CGGTAAC		19	58908492	R
	AAATGGA	37		1	235787279	CGGCTCA		1	233853902	R
	GAGGTGC	37		16	2749387	CGCGGCA		16	2689388	R
Red	GGGGTCT	37		19	36485360	CCGTCAG		19	41177200	F
	CGTAGCG	37		6	127797022	CGGCAAC		6	127838715	R
	GGGGCCC	37		3	49236808	CGCGGCC		3	49211812	R
Red	GAACGGC	37		19	1071208	GCGCGTG		19	1022208	F
	TTTGGTC	37		6	130774523	CGGTAGC		6	130816216	R
	GGAAGAA	37		17	74911808	GATTTTA		17	72423403	F
	CCCTTTC	37		1	230513809	CGTGTGA		1	228580432	R
	GATGATC	37		11	2418102	GTCCGGG		11	2374678	F
	GCAGCAC	37		19	14843922	TCTGATA		19	14704922	R
	GCTATGC	37		8	116663921	CCACACA		8	116733095	R
	CTGGAAA	37		1	196743672	TTTTTGA		1	195010295	R
	ACTTTCC	37		1	109741037	CTTGGGC		1	109542560	F
	TTTTAAG	37		10	18425598	TTAGTAT		10	18465604	F
	CAACTCT	37		6	29973221	CGCAGGC		6	30081200	F
	CTGTAGT	37		17	32581220	CGGGAAT		17	29605333	R
	AGCCGTG	37		9	96623111	CAGGCTG		9	95662932	R
	GAAATAC	37		4	183167912	TATGAAT		4	183404906	R
	GCTGTAT	37		11	34393106	CGTGTGA		11	34349682	F
Red	CCCTAGC	37		6	28584172	GACCGGC		6	28692151	F
	ACAATTT	37		19	52452317	GTTTGCG		19	57144129	R
	GGCTTCT	37		19	13113482	CGCAACT		19	12974482	R
Red	GCACCGC	37		5	14406732	CTTATCG		5	14459732	F
	CGGCCTC	37		11	67383862	TGGGGTG		11	67140438	R
	AAAGCAA	37		8	11212812	CGGGTCC		8	11250222	R
Red	GAACAGT	37		17	75315486	CGCTCCC		17	72827081	F
	AAATCAC	37		17	2308689	AGACATC		17	2255439	F

	TGGATGG	37		13	113407548	CGCCCCT		13	112455549	F
Red	CCAGGGC	37		2	198365150	CGCCTCA		2	198073395	R
	CAGTTGC	37		16	28079611	CGCCTGG		16	27987112	R
	ATGTCCA	37		19	55173030	CGCCCAG		19	59864842	R
	GCGCCGC	37		7	158712832	CGGAGGC		7	158405593	F
	GAAACAA	37		10	5047487	CGGCAGC		10	5037487	R
	TTTCTACC	37		10	13696795	CGGGGAC		10	13736801	R
	TGTGCTG	37		8	142427117	GACTATG		8	142496299	F
	AGGCTTG	37		17	76895722	CGCTGCA		17	74407317	F
Red	CCCAGAC	37		10	116019713	GACAGGC		10	116009703	F
Grn	GGCTGCT	37	Y		7142046	GCTGGAT	Y		7202046	F
	GAAAGAT	37		2	109636680	CTTTTAAC		2	109003112	F
Grn	TTTTTGAA	37		5	180645302	AAGGAAA		5	180577908	R
	TCTCTTGC	37		6	32633354	CGGGACT		6	32741332	R
Red	GTGCGGC	37		2	97136556	CGCCCTC		2	96500283	R
Red	GCTGTGC	37		16	30485684	GGAGAGC		16	30393185	F
Grn	CACTCAC	37		22	42394638	CGGGGGC		22	40724584	F
Red	AACGGGA	37		4	132647861	TTTTGGCT		4	132867311	R
	ATTGTTTT	37		1	168215442	CGGTGCT		1	166482066	F
Grn	CTGGCGG	37		2	238647913	GCCGACT		2	238312652	F
	TAAAGGA	37		15	44507997	CGGACAT		15	42295289	F
	CAAAGGC	37		19	54269519	CGGGCAC		19	58961331	R
	CTGGGTC	37		6	15509388	CGGGGAC		6	15617367	R
	TCTCAAT	37		3	197209034	CGCTCAG		3	198693431	R
Red	CAGGCGC	37		12	133003907	CGTCACC		12	131513980	R
	GGCTGGC	37		4	58061991	GCATGGT		4	57756748	F
Grn	TTTTTAGT	37		19	53018151	AGCTCAT		19	57709963	F
	TGTTTCAT	37		16	67471172	CGCTGGA		16	66028673	F
	AGAGGAA	37		2	109622481	CGGGCTG		2	108988913	R
	ATCCTTCC	37		6	34105785	GCATCCA		6	34213763	R
	TGTCTGG	37		10	15110983	CGCCAAC		10	15150989	R
Red	GCGGTCG	37	X		153236825	TGTTTCGC	X		152890019	F
	CTTGACA	37		16	70808233	CGTGAGA		16	69365734	R
	GGGTGAC	37		19	11276691	ATTTAAT		19	11137691	F
	AGACAAT	37		6	32628953	CGCTGTC		6	32736931	R
	TGACCCG	37		6	22878214	CTTATCT		6	22986193	R
Red	AGTGTGG	37		1	234908381	CGGCCAC		1	232975004	F
	AGAGCCC	37		16	930179	CACAGGC		16	870180	R
Red	GCTCCCTC	37		4	186960991	CGGCACC		4	187197985	R
	CGCCTAG	37		17	77923314	CGGGGCT		17	75537909	R
	AGTGAGC	37		1	246668601	GTGAGTG		1	244735224	F
Grn	CCGACGC	37		4	1994761	CGAGCTG		4	1964559	R
Red	ATATGCT	37		11	76998857	CGATGGT		11	76676505	R

	ATTCCTG	37	2	189064557	GGAAAGC	2	188772802	R
	TTACAGG	37	3	10182561	CGGTAAT	3	10157561	R
	AAGGCCC	37	7	42896398	CGGAACA	7	42862923	R
	TTTGTGC	37	11	119341250	CGGCTGC	11	118846460	F
Grn	CTTGGGG	37	11	58598444	CGGGATC	11	58355020	F
	AGAAGAA	37	14	24098569	AGCAAAT	14	23168409	R
	ATTCTCT	37	14	41469306	CCCTCTG	14	40539056	R
	TTCCCAG	37	9	141105974	CGGCAGC	9	140225795	F
	TGGAACT	37	13	113109379	ACCCCCA	13	112157380	F
	AAGAAAA	37	6	32829144	GGGTAAC	6	32937122	F
	ATATATA	37	12	94761621	CGGGCTT	12	93285752	R
	AGATTAA	37	5	140562819	GGCTGCT	5	140543003	F
Red	CGCTCCC	37	Y	2804320	TGTGCCC	Y	2864320	R
Red	TCACTCT	37	X	134654334	GCTAGGA	X	134482000	F
	AGAGGGC	37	5	17360014	TTCTGGG	5	17413014	R
	AACAGAA	37	5	87389026	CGGGGAA	5	87424782	R
	GTTGGTG	37	10	33016632	GCAATTG	10	33056638	R
	TAAAGTC	37	10	108921320	GCCCTCT	10	108911310	R
	GGGGCCC	37	17	34539744	CGGGAAC	17	31563857	F
	TTTAACT	37	10	81895943	CACCCTG	10	81885923	F
	CTCAGGT	37	14	22362071	GTCTGGG	14	21431911	F
	AGGGTCC	37	14	52783454	CGTAGCT	14	51853204	R
	GCTTAGG	37	15	45493327	TGGAATG	15	43280619	R
	CACATCC	37	15	97311928	AGCCGTT	15	95112932	F
	ATCATTC	37	17	17580647	CGAGGGC	17	17521372	R
Red	GCCATGT	37	8	43132451	CGGCTGC	8	43251608	F
Red	CCTCAGG	37	1	9099298	CGGAGCC	1	9021885	F
	TGGAGCA	37	4	72670093	AATCTGT	4	72888957	R
Red	CTTCTTA	37	12	66262214	AAGCCCC	12	64548481	F
	CAAGCTG	37	15	22921184	CGGAAAA	15	20472625	R
	TCCTATG	37	7	18906433	ACCACAC	7	18872958	F
	CATTTCT	37	3	72570318	CGGGGTC	3	72653008	R
	AGGAACA	37	7	50347487	CGACAGT	7	50318033	F
Red	GGGTGCC	37	14	104394831	CGCCTGG	14	103464584	F
	CAGGGTC	37	6	30459317	AACCAGC	6	30567296	F
	ACCATTC	37	3	150187338	TCCCTTA	3	151670028	F
Red	CCGAGCT	37	3	123166882	CGGCCGC	3	124649572	F
	GTGGCTT	37	15	63116514	CGGTTCC	15	60903567	F
	TGGCTCA	37	8	2122339	CGGCGCC	8	2109746	F
	GAAATCT	37	1	8083186	CCAGATG	1	8005773	F
	TTGAAGG	37	3	187086530	CGACCCT	3	188569224	R
	CCAACGC	37	3	139252106	CGCACAC	3	140734796	R
	TAGATAG	37	2	160764918	CGGCCAC	2	160473164	F

Red	AGTCGCT	37	2	132587079	CGGCCTC	2	132303549	R
Red	CAGCTGG	37	14	102415401	CGGCCTG	14	101485154	F
	ACAGTCA	37	7	38880852	CGTGAGC	7	38847377	R
	GCCCGCG	37	17	15652854	GTGGAGC	17	15593579	R
	ATGTACA	37	16	3570555	CGCAAGC	16	3510556	R
	AGGGGGT	37	3	13922158	GGTTGGG	3	13897159	R
	AAGTGAC	37	6	32362638	CGGGCTG	6	32470616	R
	GCCAGGC	37	22	46451319	TATGTCT	22	44829983	R
	ACAGGCA	37	20	61160996	GGCAGCC	20	60571441	R
	ATACTCT	37	1	8940460	CGGGATC	1	8863047	R
	CTATGTA	37	1	28573052	CGAAGCA	1	28445639	R
Red	CCCTCAG	37	19	57630446	TGCAGTC	19	62322258	F
	TTTTGTTC	37	11	103453960	CGGTCTT	11	102959170	F
	CCTGAAC	37	5	126409372	CGGAAAA	5	126437271	F
	ACACGTT	37	1	39513326	CGCTCCC	1	39285913	R
Red	CGTGAAC	37	16	841550	CGACTTC	16	781551	R
Red	ACGGGGC	37	19	1852165	GGAAGCT	19	1803165	R
	TATCACT	37	7	94045725	TTCCAAT	7	93883661	F
	ATTTCCA	37	7	73446865	TGTCATT	7	73084801	R
Red	CAGGTCC	37	11	118842387	CGGCGCC	11	118347597	F
	TCAACTT	37	2	240965916	CGGCCGC	2	240614589	F
	GCAGTGC	37	17	33390854	CGCAGTC	17	30414967	F
	TATGACC	37	5	90458832	TTTTTCTT	5	90494588	F
	ACTCATA	37	1	161676469	CTGACTA	1	159943093	F
	ATGCCCC	37	16	85028831	CGGGAGT	16	83586332	R
	GTAGTGA	37	8	69497246	GTCCTAA	8	69659800	F
	AAGACAC	37	14	91883268	CGGGTCA	14	90953021	F
	ATTTCTCT	37	8	25166343	CAAGAAT	8	25222260	R
	GGTAGGT	37	10	28970534	GTGTCTC	10	29010540	F
	ATTGTTTC	37	1	240855271	CGGAGGC	1	238921894	F
Red	GAAACAC	37	14	99744403	CGTGTTT	14	98814156	R
	AACCTGT	37	Y	23566572	GAAACAC	Y	21975960	R
Red	TGGGTCT	37	16	1124349	GGGGCAC	16	1064350	F
	CTTAGGT	37	14	30649039	CGGACTT	14	29718790	F
	CAGCCAA	37	16	17499310	CGAGAAC	16	17406811	F
	CCTTTATT	37	3	109404367	CTATAAG	3	110887057	R
	GCCAATG	37	7	126885001	CTCGGCT	7	126672237	R
	GGAAAAA	37	3	48310897	TGTGTGC	3	48285901	R
	CAGTTTA	37	4	185329364	CGGTCCT	4	185566358	R
Grn	TTAGTAG	37	16	55364843	GCGAGGT	16	53922344	F
	ATACTTC	37	7	158854447	TTACTCA	7	158547208	R
	CAGCCCA	37	6	29527870	CGAGAAT	6	29635849	R
Red	CTCGGCT	37	X	152677200	CCGAGCC	X	152330394	R

	GTGAGTA	37	2	204731519	CGGACTG	2	204439764	F
	AGCCATA	37	10	85985331	CTGTGTG	10	85975311	F
	CCCCCCA	37	2	121846490	CGGCTGT	2	121562960	F
	GCAGGTC	37	2	2295969	CGCCCGC	2	2274976	R
	TTTCAAT	37	15	96869132	GGGTGTT	15	94670136	R
	GCCCTCA	37	7	27146430	TCTCAGG	7	27112955	F
	CTGCCAT	37	6	29427519	CGGCACC	6	29535498	R
	CAGAGGC	37	14	105555223	GGTGCCC	14	104626268	F
	CTGCCAC	37	3	49697458	GCATACA	3	49672462	R
	CCCTCCC	37	7	14031822	CGTAGTA	7	13998347	R
	ACACGCT	37	12	31499320	GTTAAGG	12	31390587	R
	GAGAGAC	37	12	378771	ATTGTCC	12	249032	F
	TCCAAAC	37	16	86327786	CGGGTAT	16	84885287	F
	CTATCTC	37	6	31323261	CGGGATC	6	31431240	R
	AGGACAC	37	11	126944154	TGCCAGC	11	126449364	F
	GAAGCGT	37	5	168719	CGAGAAA	5	221719	F
	GCGCCTG	37	14	93153553	CACCTGC	14	92223306	F
	ATTCTGA	37	1	215923004	GCTCCTC	1	213989627	F
	GGCTCAG	37	17	75378036	CGGGGTA	17	72889631	F
	GCAGTTG	37	5	107896573	CGAGATC	5	107924472	R
Red	GCGCCCC	37	10	135498991	CGGAGCC	10	135348981	F
	GAGCAAT	37	18	9704392	CGAAACT	18	9694392	F
	CTTCTTA	37	14	104436161	CGCCCCA	14	103505914	R
	ACCCCCA	37	11	44597119	GTACCCA	11	44553695	F
	CAGTGCA	37	10	32701586	CGCTTTA	10	32741592	F
	TCTAACC	37	16	83982669	CCTTTTG	16	82540170	R
Red	AACCCGC	37	12	131171031	TATCAGC	12	129736984	F
	GATATGG	37	7	138803256	CGGGGGC	7	138453796	F
	TGATTAA	37	16	9828141	ATGAAAC	16	9735642	R
	ATCTAAA	37	10	135343193	AATGAGC	10	135193183	R
	GAGTATA	37	11	12098045	CGACAGA	11	12054621	F
	GTCATGT	37	5	156722426	CGGGTCC	5	156655004	F
Red	CTCATTG	37	11	11642011	GGGTCTG	11	11598587	F
	ATAACTA	37	12	64383297	CGGCATA	12	62669564	R
	GGCTTGA	37	16	84520021	CGCCTGA	16	83077522	F
	CATGGTG	37	14	100233454	CGGAAAC	14	99303207	F
	CAGGTCT	37	7	158533877	CTGCTGT	7	158226638	F
	GAGGTCA	37	6	43818920	TTTCCTC	6	43926898	R
Grn	ACGAAGA	37	17	37308828	CGCCCCA	17	34562354	R
	GGCCGGC	37	11	65810869	CAGGATC	11	65567445	R
Grn	CGGCCCG	37	11	5960015	CAGCTGC	11	5916591	R
	GGTTACT	37	9	44401998	CGGGGTT	9	44341994	R
Red	GGCCAGA	37	8	145728507	AGGCCTG	8	145699315	R

	TTTGTGA	37	15	94346066	CGGCAGC	15	92147070	F
Red	GTGGTTT	37	5	178450646	AGAGAAC	5	178383252	F
	TTAGATG	37	10	7839613	GCTTAAT	10	7879619	R
	CTACCAT	37	14	21467273	CGGATAC	14	20537113	R
Grn	GGGCCAC	37	17	79495519	TTCGTCG	17	77110114	F
Red	AACCATA	37	19	55644229	CGGTAGC	19	60336041	R
Red	AAAAAGA	37	5	72705952	CGCCCAG	5	72741708	F
Red	GGCAGCA	37	10	26681244	CGCGAAT	10	26721250	F
	CTGTTCA	37	10	49742271	CGTTGGG	10	49412277	F
Grn	CCTATCT	37	20	32856898	AGGAGCA	20	32320559	F
	GTGGGGC	37	7	150067712	TAGGGAA	7	149698645	F
	GGAGCCC	37	16	90143788	CGCTGAT	16	88671289	R
	AACTTCT	37	15	70250464	TCCCAGG	15	68037518	F
	AATGTAA	37	14	82546712	TGCTGGA	14	81616465	F
	GCAGCCC	37	17	16570473	CGCAGCC	17	16511198	R
	CTCTATG	37	11	130482015	GCCCTGG	11	129987225	R
Red	TTGTGGT	37	17	79961614	CGCAGCC	17	77554903	F
	TATCCTA	37	13	26776254	CGGTTCT	13	25674254	R
	TCATCCA	37	3	141560204	TATTCTT	3	143042894	R
	CTCCTGC	37	6	167789539	GGTGGCC	6	167709529	R
Grn	GACTGCC	37	6	156983263	GGCCACC	6	157024955	R
	TGTGTGT	37	4	1874366	GAGCCAC	4	1844164	F
	CAGACAC	37	3	69058876	AATACTC	3	69141566	F
	GGCACGT	37	8	103751006	CGCTGAG	8	103820182	R
Red	GGCCCTA	37	6	28558006	TCCTTTCA	6	28665985	F
	TGTGTAG	37	17	48276347	TTTTTTTT	17	45631346	F
	CTTATCA	37	2	102117488	CGCAATG	2	101483920	F
	CCCTGTC	37	12	130968528	CTAGATG	12	129534481	R
	AGGCTGC	37	14	50527872	CGGGGGC	14	49597622	R
Red	GAGACGC	37	19	21106043	TGGGAAC	19	20897883	R
	CCCTTTG	37	8	98361938	AGCTACA	8	98431114	R
	TGTGCGT	37	19	58867669	CGCCCAC	19	63559481	R
	TCAGAAT	37	10	112794609	TTGATAA	10	112784599	R
Grn	GCCACCG	37	14	104552397	CCCACGC	14	103622150	R
Red	CGCGCAC	37	16	66436664	GAGCGTC	16	64994165	R
	CCAACCT	37	17	80535367	CAGGGCT	17	78128656	F
	AATGGTA	37	2	170142388	ACCTATG	2	169850634	F
Red	ACCTCCG	37	1	2121349	TCTCTGC	1	2111209	R
	CCATTGT	37	22	23489041	CGGTCCC	22	21819041	R
	AAGGGGC	37	4	183601649	GACTGGC	4	183838643	R
Red	CCAGAAT	37	14	75590896	TCTCATT	14	74660649	R
Red	ACGCAAC	37	19	3478662	CGGGCTT	19	3429662	R
Red	ACCGCAC	37	19	2250901	TCGGACC	19	2201901	F

	TCATGCT	37		13	111522755	CGCTGGT		13	110320756	F
	AGAACCC	37		17	43170343	CGGACTC		17	40525869	R
	AGGAGTC	37		17	113233	CGTGCTG		17	113233	R
	AGCGAGC	37		11	67141686	TGGGAGC		11	66898262	R
Grn	GCGCGCC	37		12	48357394	CGGCACC		12	46643661	R
	AAGATGA	37		4	4137166	CCCCCCA		4	4188067	R
	AAATCAA	37		2	225875689	CGAAGGC		2	225583933	F
	GGGGAGA	37		17	5019761	CGCCCCT		17	4960485	R
Red	GGAAGGC	37		19	18889003	CGGATTG		19	18750003	R
	TTCTAAG	37		4	164092065	CGGTTGA		4	164311515	R
	GTCACAG	37		6	33039481	CGTAGGT		6	33147459	F
Red	GCCCCGC	37	X		37706736	GGAAGGC	X		37591680	R
Red	TGCAGTC	37		7	76145672	GGAGCGC		7	75983608	F
	ATTGTTA	37		14	29250912	CGCTGGC		14	28320663	R
	ACTCTGG	37		12	10456885	CGGTGCA		12	10348152	R
	CCAGTGA	37	Y		9528418	CGACACC	Y		10138418	F
	GGCTGTG	37		6	32553920	TTTTGCA		6	32661898	R
Grn	CCCATAA	37		2	242693323	CGCAGCA		2	242341996	R
Red	CCTCTCA	37		10	124893766	CGCCTAA		10	124883756	R
Grn	GTCCCAG	37		1	2266933	CGGCGAC		1	2256793	F
	TCTTCTC	37	X		48748398	AACAACC	X		48633342	R
	GCTGCTG	37		16	5092012	CGGTGAC		16	5032013	R
	TTTAAGG	37		3	57032141	AAAGTTA		3	57007181	R
	AAATGAC	37		3	63428587	TTTATTA		3	63403627	R
	TTTCACT	37		11	93586725	AACCATT		11	93226373	F
	TGAAAAA	37	Y		6136711	CGGGGAA	Y		6196711	F
	ATGCATT	37		10	86056416	CGGAAAC		10	86046396	R
	GCCTGGA	37		12	54384744	CGGCCAC		12	52671011	R
	AACTCAC	37		6	25701717	GTGTGCT		6	25809696	R
	CTTTAGG	37		3	16225307	AGAGAAT		3	16200311	F
	CAGCATC	37		18	49865270	AGTTTCC		18	48119268	R
	CCTTTCA	37		6	6001380	CGCCCCA		6	5946379	R
	TAATCAG	37	X		100663101	CGCCCTA	X		100549757	F
	TCACCTT	37		6	32712979	CGGTCCC		6	32820957	F
	GTGAAAA	37		16	86743939	CGGGGGC		16	85301440	R
	TCAGAGT	37		10	77863526	CTGCCTC		10	77533532	R
	ATGCCCA	37		17	32581035	CGAGAGA		17	29605148	F
	CTCGGAA	37		10	729916	CGGCGTC		10	719916	R
Red	AACGTTC	37		18	77292063	CGGCCAA		18	75393051	F
Red	GACAGCA	37		8	47529280	GCGGCGC		8	47648445	R
	CAGGCAA	37		7	64314295	TCTCCAG		7	63951730	R
Red	CGAGTGC	37		16	86795490	GTCAGGC		16	85352991	R
Red	CCGGCCC	37		16	88706426	CTGAACG		16	87233927	F

	TGGAGAA	37	6	122115942	GTCACAG	6	122157641	R
Red	GAAACCT	37	17	80255419	GAGAAGA	17	77848708	R
	AGACAAA	37	2	101369330	CGTGGTT	2	100735762	R
Red	TGCCAGC	37	8	72917350	CGCGCTT	8	73079904	F
Red	CGATTCG	37	11	66326798	CGCTCTC	11	66083374	R
Red	TGGATCC	37	16	66600478	ACGCCAA	16	65157979	R
	GGTAGGC	37	16	869560	GCTTATC	16	809561	R
	GGAAGAT	37	3	23170838	CGCTGAC	3	23145842	R
	TACTGAT	37	3	149192609	CGGGGCC	3	150675299	R
Grn	TGTCTGG	37	12	132845481	GGAAAGC	12	131355554	R
Grn	TTGTTTC	37	17	38708465	CGCTCCG	17	35961991	F
	GATTAGG	37	7	5937190	TAGAGGC	7	5903716	R
	GGCATCC	37	8	36641571	CGGAGTC	8	36760729	R
	AGAACGA	37	6	168918703	CGGTTCA	6	168661552	R
	TGTCGCC	37	2	80101372	CCTTGGC	2	79954880	F
	GGGACTC	37	17	74868670	CGGGCTC	17	72380265	R
	AGGGAAA	37	16	66413050	CGGGTAA	16	64970551	F
	CACCTTG	37	5	149493258	CCCTTCA	5	149473451	F
	TGTTAGC	37	3	129205070	ATCATAT	3	130687760	R
	GCTGTCA	37	4	1309416	CGCCCCA	4	1299416	F
	CGCTGTT	37	17	57120	TGGAAAT	17	57120	R
	GGTTCAT	37	3	131637172	AGTTCAG	3	133119862	R
	TGAGCTT	37	8	118186764	GGCAACC	8	118255945	R
	GGTTCAG	37	17	1553415	CAAGTGC	17	1500165	R
Red	GGGCCAC	37	6	30039027	TGGAAGC	6	30147006	R
Grn	GCCCCAC	37	5	33938213	CGGGGGC	5	33973970	F
	TGAGCAC	37	15	101262162	CGACTCT	15	99079685	F
Red	GACGAAT	37	6	106434354	ACACAGC	6	106541047	F
	TGAGGCC	37	16	88989069	CGCCTGT	16	87516570	F
	TGTATGC	37	5	140711852	GATCCTT	5	140692036	R
	GTATTTC	37	3	159886981	CGCTGAG	3	161369675	F
	CTGGTGT	37	16	25005762	CGGGTTC	16	24913263	F
Red	CGGGCGC	37	5	2915123	GAATCCG	5	2968123	R
Grn	GCAGCCA	37	17	17109570	GTCCCGG	17	17050295	F
Red	AGGTAGC	37	16	88472944	CGGGTCC	16	87000445	R
	AGGCCCT	37	10	72647652	TCTTGCC	10	72317658	R
	ATATATT	37	12	54073597	CTTTTCCT	12	52359864	R
Grn	TCTGCCC	37	7	43288757	CGGAGCC	7	43255282	F
	TCGCGGC	37	6	42695153	CGGCTGG	6	42803131	F
Red	AGTTCTT	37	Y	21729069	CGGCCTC	Y	20188457	R
	CAATTTC	37	10	33710900	ATTCCCA	10	33750906	F
	GTTTTAT	37	19	56696870	CGAGGAC	19	61388682	F
Red	CCCCAAC	37	1	150948030	CGAATAC	1	149214654	R

	GAGGGA(	37	10	1517218	CCTGTGA	10	1507218	R
	CAGCAGA	37	5	31197518	CGGACAC	5	31233275	R
	CCCTTTT(	37	8	22960397	CGGGGGA	8	23016342	R
Red	GGTCACC	37	21	46921982	TCCTTCT(	21	45746410	F
Red	CGCGGTG	37	13	111286999	CGGCTCC	13	110085000	F
	GCAACTC	37	3	195273304	CGGGGAC	3	196754593	R
	CACCAGC	37	11	67568593	CGGTAGA	11	67325169	R
Red	CCCCCTC	37	5	141660565	CGGTAGC	5	141640749	F
	TTGTATT(	37	3	150093970	TGGATTG	3	151576660	R
Grn	CCAGGTC	37	7	72848703	CGCGCCC	7	72486639	F
	ATTAGAT	37	3	149685395	AAGTCAC	3	151168085	R
Red	TCCCCGC	37	6	13860661	CGCTGTT	6	13968640	F
	ATGAACT	37	6	41885417	TCACAGG	6	41993395	R
Red	CCCTCCT	37	8	97158015	TCAGCTC	8	97227191	R
Red	TGGCCCC	37	1	33235324	TCCACCT	1	33007911	R
	TGGCCCT	37	1	201665422	CGCCCGA	1	199932045	R
	TGCTCAC	37	8	19029020	ACTAGAC	8	19073300	R
	TCCTCCA	37	1	19581542	CGAGCTA	1	19454129	R
Red	CCCGGCC	37	16	81520589	CGGCGCT	16	80078090	R
	AATAGGC	37	2	192402991	TACTTGA	2	192111236	R
	GTTTAAG	37	8	119504312	CGCAATG	8	119573493	F
	GCTCAGG	37	16	49518536	GGACTGC	16	48076037	F
	AATCCCT	37	3	56896467	CCCTGTT	3	56871507	F
	TGTTCGC	37	7	137671793	CGGCTCC	7	137322333	F
Grn	AGGGAGC	37	6	32634362	CGGATCC	6	32742340	R
	GGAGGTC	37	16	2630397	GCCTGGG	16	2570398	R
	TGTCTTA	37	2	214992482	CATTAAG	2	214700727	F
	TAAGTTT	37	11	13282917	GGATATT	11	13239493	F
	CTTTACC	37	6	29911004	TCATTTT	6	30018983	R
Red	GGGAGGC	37	6	169825927	CGCTGCC	6	169567852	R
Red	AGTGCCA	37	11	66885240	GATGTGT	11	66641816	R
	CCCGGGC	37	6	33036504	GACAACC	6	33144482	F
	TTCATTA	37	2	103089678	CGGCTTC	2	102456110	R
Red	CGATCTC	37	7	44614305	CGCACCA	7	44580830	F
Red	AGGGGTT	37	1	19384827	CGGGGTC	1	19257414	R
Red	CCCACCA	37	4	165898825	CGGGGCA	4	166118275	R
Grn	GCAGACC	37	10	65801432	CGCCGTG	10	65471438	F
	TCCATAC	37	10	31026101	CGTGCAC	10	31066107	F
	CAGCCAC	37	13	112836631	ACAGGAC	13	111884632	F
Red	CAGCTCG	37	7	64035417	CAGTCCC	7	63672852	R
Red	TAGCAGT	37	17	21226409	ACTGCCA	17	21167002	F
	TGTTTAT	37	4	129292585	CTTTATT	4	129512035	F
	TCCCACA	37	7	150822006	CGTTGCA	7	150452939	F

	AGACATC	37	6	155739835	CGCCTGC	6	155781527	R
Grn	TTTCAGC	37	22	50721120	AGCCTTC	22	49063247	F
	CCGTTTC	37	8	9009352	GCTCTGT	8	9046762	F
	TTCTGAT	37	21	31709690	AGGCTGA	21	30631561	R
	GCGCATC	37	6	30707585	AAACCGT	6	30815564	F
Grn	CGTGGGA	37	13	24882296	CGGAATC	13	23780296	R
	TCAACTG	37	8	134401000	GTGGCCA	8	134470182	F
	TATGGTA	37	10	60147807	CATAACT	10	59817813	F
Red	TGCTGTT	37	10	11088146	CGGCACC	10	11128152	F
Grn	GAGAAGT	37	7	105596483	TAGTTCC	7	105383719	F
Red	GCAGCGT	37	6	42695625	CGCCCCG	6	42803603	F
Grn	TCGGGGC	37	6	29911095	CGCCCGT	6	30019074	R
Red	GCGTAGA	37	16	28634766	GTTGCTC	16	28542267	R
	ACTTTGT	37	12	76706337	CGGTCAC	12	75230468	F
Red	CGGAATC	37	5	140579470	GGCGGGC	5	140559654	R
Red	ACGACTA	37	6	31650760	TTTGCGA	6	31758739	R
	GAACAGC	37	12	312595	CGCCGGC	12	182856	R
Red	AGGGAG	37	Y	6114255	ACCGCCC	Y	6174255	R
	CACGTCA	37	20	62402415	GCCAGCT	20	61872859	F
	GGGGGGC	37	5	1246291	CGTCAGG	5	1299291	F
Grn	AGAGCG	37	2	232348602	CGCCCCG	2	232056846	R
	CAAGCTG	37	16	58704495	CGCCAGA	16	57261996	R
	GGGTGAC	37	7	76030355	TGGTGTG	7	75868291	F
	AATTCAA	37	8	119107030	AACCCTT	8	119176211	R
	GAGGAGC	37	8	1905131	CATCTCT	8	1892538	R
	GTCCCTT	37	20	31668448	AGCATTA	20	31132109	R
	GGTCACA	37	4	94759825	CGGGAA	4	94978848	R
	TCCTGAA	37	18	74964030	CGGTGCC	18	73093018	R
	GCCACCA	37	19	37466940	CGGAGAT	19	42158780	R
Red	CCCACAC	37	7	158075388	TGTCTGG	7	157768149	F
	CGAAACA	37	14	104663241	GCACTTG	14	103732994	R
	GTGCTTA	37	11	36055687	TATCTCT	11	36012263	R
	AGGAGAC	37	13	26622819	CTTTGAG	13	25520819	R
	AATTTTC	37	20	18566614	CGGAGCC	20	18514614	R
	CATTCTC	37	4	155253864	CGGGGAT	4	155473314	F
Red	GGGCGGC	37	X	50213977	CGCTAGG	X	50230717	R
	TCAGTAG	37	7	1075592	CGGCATG	7	1042118	R
	GGATGGC	37	7	151565731	CGTGATC	7	151196664	R
	TTCCAGC	37	11	107583406	TACTGCT	11	107088616	F
	ATTTCCC	37	20	62957648	CGGACAT	20	62428092	F
	CTTATCG	37	6	170405014	CGGCCTG	6	170246939	R
Red	TCGAGCA	37	4	4228644	GCTGGTT	4	4279545	F
	TGCACTG	37	6	32489750	CCTGACG	6	32597728	F

Grn	CCGCTCC	37	11	118842484	CGCCTGT	11	118347694	F
	TTACCGC	37	10	44501010	CGCTTTG	10	43821016	R
Red	GGCAAAA	37	6	101846805	GCTAGCA	6	101953526	R
Red	GAGAGGA	37	17	1954986	CGGCCCC	17	1901736	R
	GACAAGC	37	12	132869069	CGAGGGC	12	131379142	F
	GAGTGAA	37	1	77711859	CGAGTGA	1	77484447	F
	GCCTGGC	37	11	14004854	TGCCTGC	11	13961430	R
	ACTGTGA	37	11	26743425	CGTGTAC	11	26700001	F
	ATGTGAA	37	16	1021780	ACAGTTT	16	961781	F
Grn	AGAATCA	37	21	45360804	CGGGGTT	21	44185232	R
	TCTCCAA	37	8	37005558	GTTATTG	8	37124716	F
	TGGGCGC	37	1	7023864	CGCCCCT	1	6946451	F
	TGTGCCT	37	2	3753297	CGGTCTG	2	3731172	R
Red	CGCTCTC	37	3	47205656	CGCAGGC	3	47180660	F
	TCTGTGG	37	16	86029348	CGCCCAG	16	84586849	R
	AAAAAGT	37	5	40841590	GAAATCC	5	40877347	R
Grn	GGCGGCC	37	X	103411244	CGCCCCG	X	103297900	R
	CCACTTC	37	7	139761087	TGTGCCC	7	139407556	F
	GAAGTGA	37	8	3269952	CGTTCAG	8	3257360	F
	GCTTGAC	37	17	32612684	CGGGGGT	17	29636797	R
	AACTTAA	37	11	122051193	CGTGGAA	11	121556403	F
	CCAATAA	37	6	31976720	CGGCCTG	6	32084698	F
Red	TGCGTGA	37	8	80783997	CGGGGAT	8	80946552	R
	GGGGGCC	37	3	184280712	TTGTTTA	3	185763406	R
Grn	TCCCGGA	37	5	140530467	TGCTAGA	5	140510651	R
	CAACCCC	37	X	20134557	CGGGGAC	X	20044478	F
	AAACAAC	37	13	28912746	CGGTCTG	13	27810746	F
	TACGTTC	37	7	134017084	TGGCAGC	7	133667624	R
	GCGCGCA	37	20	3653555	CGGGGAC	20	3601555	F
	TCCGGAA	37	6	29648468	CGCCTGG	6	29756447	F
	GGGAGAC	37	2	131722957	CGGGCAC	2	131439427	R
	GCTTCGC	37	X	153096786	CGGACAC	X	152749980	F
	ACTCAGG	37	7	55100608	CGCTGGA	7	55068102	F
	GGTTGGA	37	16	83967808	CGGCCCC	16	82525309	F
	CTGGAAC	37	11	77299960	CGCGCTC	11	76977608	F
	GACCAGC	37	Y	21666309	TTGTCCA	Y	20125697	R
	TATCTGA	37	20	4795381	CGGGGAA	20	4743381	F
Red	TCTTCTC	37	16	2903246	AGGAGCC	16	2843247	R
	GGGCTTT	37	1	6561043	TTTAAAT	1	6483630	F
Red	ACGCAGT	37	1	1886501	GGACAGC	1	1876361	R
	GGCCTCA	37	7	75780412	TTGGTCA	7	75618348	R
	TGGGTTG	37	21	34444104	TGGGTCC	21	33365974	R
	AACCCAT	37	7	17822217	CTGTCTA	7	17788742	R

	CTCATAG	37	6	40324498	TTTTCCT	6	40432476	R
	TTTCCAG	37	7	2078981	AGCAGTC	7	2045507	F
	AATTTTA	37	8	37820326	CTAGGGC	8	37939483	R
	GAAGAAC	37	11	3174092	AAAGTAA	11	3130668	R
	TCAAGCC	37	1	25069938	CGGGCAT	1	24942525	F
Red	TCCTTGT	37	X	117633362	CGCGCCG	X	117517390	F
Red	CGGGGCT	37	4	174422191	AAAGTGC	4	174658766	R
	TTGTTTA	37	6	9139720	AAAACTC	6	9084719	F
Red	CTGAGAA	37	X	118986703	CGCCCTC	X	118870731	F
	GTTGGAG	37	6	31082631	CGAGACT	6	31190610	F
Red	GACGCTG	37	12	132682698	CGGCGCC	12	131248651	F
Red	ACCGCGT	37	2	242950311	CGCACTG	2	242598984	R
	GGCCCCT	37	17	72860652	CGGCCCA	17	70372247	R
Red	ACGGGCC	37	22	18985691	GACAGAC	22	17365691	R
Red	GTTCATA	37	2	100902129	TCCTGTC	2	100268561	R
	ACACATC	37	17	77300674	CGAAGAA	17	74812269	R
	GGGCATC	37	20	61456784	CGGACAC	20	60927229	R
Red	AACGGGC	37	4	942005	CGAGCCT	4	932005	R
Red	TCAGCGC	37	10	43846376	AGATGGA	10	43166382	F
Red	CCAGGCC	37	16	90148775	CGGCCGC	16	88676276	R
	GGTAGAC	37	2	201003360	CGCTTGA	2	200711605	R
	CTGAAGC	37	4	15429515	CCAAAGA	4	15038613	F
	TATTTGA	37	14	95078636	CGGACTG	14	94148389	R
Grn	CAGCCCC	37	2	241896910	CGCGTGC	2	241545583	R
	GAAAAAA	37	12	54383639	AAAAATT	12	52669906	R
Red	CTCGGGA	37	12	11700489	TGGGACT	12	11591756	F
	AGCGGCT	37	4	7436239	CGGAGCA	4	7487140	F
	ACTTGGA	37	18	24131379	AGCAGTT	18	22385377	R
Grn	AGTGGCC	37	16	3230726	TAGCGGC	16	3170727	F
	GCTCATA	37	X	39436753	GCAACAC	X	39321697	R
	TTCACCA	37	2	113942204	AAAGTCC	2	113658675	F
	GCTGGTC	37	9	68809732	AGGGTGC	9	68199552	R
	AGAAACC	37	Y	21726406	CGGCTAC	Y	20185794	F
	TCAGCCA	37	19	21265364	GGGTCCA	19	21057204	F
	AAAATAC	37	11	30344200	CGAGTAA	11	30300776	R
	TTCTGTT	37	17	74260776	ATCTGGG	17	71772371	R
	GTAGAAT	37	6	101846791	CTGGCAA	6	101953512	R
	CCCGGGA	37	1	41350292	TTGGTGG	1	41122879	F
Red	CCACACG	37	6	30039206	CTTCTGG	6	30147185	F
	GTCCCAG	37	11	64594467	TCCTCTG	11	64351043	F
Red	CCCGTGG	37	11	34852392	CGGGCAC	11	34808968	R
Red	TCAGTGT	37	16	86653215	CGTCGGA	16	85210716	R
	AGCCCCT	37	2	233282380	CGCTGTC	2	232990624	R

	GACATCC	37	13	113535686	CGGGTTG	13	112583687	R
Red	CGCCTTT	37	18	11147385	CGTGAGT	18	11137385	R
	ACTCACC	37	6	30782234	CGGTGAC	6	30890213	R
	GAGGTTC	37	16	7138889	AACCTCT	16	7078890	R
Red	TTGGGCC	37	7	157225062	CGGAGAC	7	156917823	F
Red	GCCGTCT	37	X	139593371	CGCGGCC	X	139421037	F
	GAGCAA	37	16	31888794	CGAGATC	16	31796295	F
	CCCTTCT	37	21	34773372	GTATAAA	21	33695242	R
	GTCCGTC	37	11	2722119	CGGGGAC	11	2678695	F
	GAAAAC	37	6	32713188	TGGAATT	6	32821166	R
Red	AGAAGGC	37	16	67313019	CGCGTGG	16	65870520	F
	TCAATCC	37	7	158804287	CGCCTCT	7	158497048	R
	ACCAGTG	37	3	119279147	CGGCTCA	3	120761837	R
Red	CGTCCCT	37	X	102000758	ATAGCAA	X	101887414	R
	AGTAGAC	37	20	56179171	GTTTCAC	20	55612577	R
	GGACAG	37	4	57528377	CGCTTAC	4	57223134	F
	TCGGATC	37	10	101088672	CGGCTGG	10	101078662	F
	ACGCTTA	37	6	5404958	AAACCCA	6	5349957	F
Red	TGTTTAG	37	22	45704914	CCTTGTG	22	44083578	F
Red	TGTGTGT	37	6	29796985	CGGAGGT	6	29904964	F
Red	GCTAAGA	37	Y	22917937	AACAGCC	Y	21327325	F
Red	ATCCAGG	37	17	39537455	CGGAGCA	17	36790981	R
	AGTCCTC	37	2	101966951	TCACCCT	2	101333383	R
Grn	CGCCGGC	37	X	153284899	AGCCGTG	X	152938093	R
	GTTGACT	37	13	50985639	CGCACAC	13	49883640	R
Red	GTAGCTG	37	16	1087129	CGGCTGG	16	1027130	R
Red	AGGCTGA	37	16	888938	CGCGCGC	16	828939	R
Red	CCACACG	37	13	113630878	CTGGGCT	13	112678879	R
Red	GGCCGGC	37	16	1493759	GCTGGAC	16	1433760	F
	GTGCTGC	37	1	212643521	CGCCCCA	1	210710144	F
	GACCAGC	37	1	1227138	GGCCTCC	1	1217001	F
	CTCTGTA	37	3	59735420	CGAAGAA	3	59710460	F
	AACAACT	37	2	242988706	AACAGTC	2	242637379	R
	AGGCCCC	37	20	62225388	CGACCTG	20	61695832	F
	AGCGATC	37	1	63249213	TCATTTC	1	63021801	F
	AGGCTCA	37	19	2058455	CGGAGCA	19	2009455	F
	GTCATCT	37	3	30349908	GTAATAA	3	30324912	R
Red	GTAGTCT	37	16	2051739	TGGCCGG	16	1991740	F
	GGGTGG	37	11	60608291	CGGCCAT	11	60364867	R
Red	GTGGTGA	37	3	113956240	CGGGAGC	3	115438930	R
Red	GGGGGCT	37	2	46049176	ACTGAGC	2	45902680	R
	GGGCATA	37	12	8403616	ATATATA	12	8294883	R
	ACACAG	37	X	99891793	TACCCTC	X	99778449	F

Red	CTGCCCG	37	16	1128698	CGGCCAA	16	1068699	F
	CACAGCC	37	6	36409495	CGAGGGA	6	36517473	F
	TGACTCA	37	11	128080305	CGGCTTA	11	127585515	R
	GTACATC	37	2	242490217	CGCGCCC	2	242138890	R
	AAAGGAA	37	2	202330990	CGTTCAC	2	202039235	F
	AACTATC	37	4	83384006	TTCCTTTT	4	83603030	R
	CAGTCCA	37	4	38330015	CGCCTAT	4	38006410	R
	CAGGCCC	37	1	38180356	TCTGTAA	1	37952943	F
	ACCAGAT	37	5	136478693	GGCAATC	5	136506592	F
	AAGCTGC	37	5	19766434	TCTTACTC	5	19802191	R
	GTGTGTC	37	3	11178683	GGTTAGG	3	11153683	F
	GACAGGC	37	18	5456880	CGAGAAC	18	5446880	F
	GCGGCAC	37	16	81740096	GGCCAGT	16	80297597	R
Grn	CAGGTGA	37	11	7110148	CGCGCCC	11	7066724	F
	TGGTGGT	37	20	62559021	GGATAAA	20	62029465	F
	TCCTTCCT	37	16	57541087	TGGGGAC	16	56098588	R
	GGGTGTG	37	19	7937329	AGGGGGT	19	7843329	R
	GGCTGAC	37	7	1000336	CGGGTGT	7	966862	F
Grn	ATGCCCG	37	13	112997365	CCAAGGT	13	112045366	R
	CCAGGAC	37	10	52434647	TACCTTCT	10	52104653	R
	GTGAAAC	37	11	130283454	ATTCACT	11	129788664	F
Red	TTTCTCTC	37	2	240174613	CGCACCA	2	239839550	F
	CAAAAAC	37	19	53107200	CGTCCAA	19	57799012	R
	GCTCAGG	37	6	33052354	AGGTTTC	6	33160332	F
	GGACCCT	37	7	4855406	CGCCCCA	7	4821932	F
	GGCTGGC	37	2	97573176	GGATGGC	2	96936903	F
	ACCCAGC	37	18	77278806	CGGGTGC	18	75379794	R
Red	GCTCAGA	37	5	140053350	CGGGAGC	5	140033534	R
	GAGTTTTC	37	6	29860039	CTGCTCCT	6	29968018	F
	GCAGACT	37	16	31439106	CTTCACA	16	31346607	R
Grn	AGACGCT	37	X	152864830	CCAGGGC	X	152518024	F
	GGGTGGT	37	1	234635574	CGGGCCA	1	232702197	F
Red	CACGTGA	37	17	35969640	CGCCCCA	17	33043753	R
Red	CTCCTGG	37	16	88990232	CGGCATT	16	87517733	R
	TGCCTTTA	37	1	217113226	CGTTGGA	1	215179849	R
	TTCTCCAT	37	20	259925	TGGATGG	20	207925	R
	CAGCTTC	37	1	53661448	CTGCTTAC	1	53434036	R
	ACCCGGC	37	16	857576	CGGCCGT	16	797577	F
	AAAAACA	37	1	15631449	TGAGCGC	1	15504036	R
Grn	CACTCCTC	37	2	11009092	CGGGACA	2	10926543	F
Red	TACACGT	37	11	93277161	CGGACCA	11	92916809	F
	GAATAAC	37	12	125225316	TTCACCC	12	123791269	F
Red	CTGAGAC	37	1	89990868	CGGCGCC	1	89763456	R

	CCACCTT	37	11	71150247	CGCCGTC	11	70827895	R
	GAAGGA	37	12	53490352	CGCCCAT	12	51776619	R
	AGCTCAC	37	8	1789169	CGCTCCA	8	1776576	R
Red	GGGGGC	37	18	47825363	GCGACAC	18	46079361	R
	TTAACAA	37	20	44352696	CGGTGGT	20	43786110	R
	TGCCCCT	37	4	7301176	GTGCTAA	4	7352077	R
	TGATTAG	37	13	44811779	TTAGTCA	13	43709779	F
	AGTCACC	37	17	79068856	CGGGGCT	17	76683451	F
Red	CGTGGGC	37	19	46387962	CGGCCCC	19	51079802	R
	GGATGCC	37	5	2643172	CGTTCCC	5	2696172	R
	CTTTTCTC	37	3	170040557	CGGGGTC	3	171523251	F
Red	CAGAACT	37	19	57109049	CGAGGGT	19	61800861	R
	GGAGCAT	37	Y	9368165	CGGCAGT	Y	9978165	F
Red	CTAGGCA	37	11	18477299	CGTGCGC	11	18433875	F
Grn	GCTGCCT	37	11	133109552	CGCGGTG	11	132614762	F
	AGACTCG	37	2	794763	CGCCTCG	2	784763	R
	TCAGTGA	37	7	51539323	CCTGGGC	7	51506817	R
	ACTGTTA	37	11	10716164	CGGGAAC	11	10672740	F
	TCTCGGT	37	6	901264	AACGTGT	6	846264	F
	TCACGCC	37	8	144158344	CGGGGTT	8	144229719	F
	CTCAGCC	37	5	31379132	GAGTCAC	5	31414889	R
Red	ATTTCTA	37	11	32354742	ACTGTAC	11	32311318	F
Grn	GCCTTTG	37	12	54376127	CGGGCCT	12	52662394	R
	GTGGTGG	37	3	23818150	AGGGCTC	3	23793154	R
	TTGAAAA	37	2	80179759	CACAAAA	2	80033270	F
Red	ACATGTT	37	12	6898647	GAGACAC	12	6768908	F
	GAGGGAC	37	Y	21240004	AACTTCA	Y	19699392	F
Red	ACCGGAC	37	X	16964684	GACGGGC	X	16874605	R
Red	CCTGCCT	37	12	132972807	GATGTTT	12	131482880	F
Grn	TCACAGG	37	17	9806770	AGTTCTA	17	9747495	F
Red	ATGCAAC	37	2	36782386	CGAAAGC	2	36635890	R
Red	CCACATG	37	1	8442507	CGCATGC	1	8365094	R
Grn	AATAAAT	37	19	56688423	CGGCCAC	19	61380235	R
Grn	CCACCTC	37	12	8025394	GCGAAGC	12	7916661	F
	GGCTTCT	37	22	45135965	TTTGCTG	22	43514629	R
Grn	GTGCAGC	37	X	53349902	CGGGTAC	X	53366627	F
Grn	ATGCGCT	37	1	206897270	TTCTCTT	1	204963893	F
	CCTCTGG	37	19	15312129	CGTCCTG	19	15173129	F
Red	GTCAAAC	37	19	613111	AGGGGCA	19	564111	R
Red	CGAGAGC	37	16	87873837	GCGGGTC	16	86431338	F
	GGAGCAA	37	6	46293571	ACATAAC	6	46401530	F
	GACCAAC	37	17	30488883	ATTCAGG	17	27512996	R
	CCAGCAC	37	18	77292443	ATGTGAA	18	75393431	F

	TGGCCTT	37	5	174174566	TGAGAAC	5	174107172	R
	CATCTAA	37	19	45457306	CTATGTT	19	50149146	F
	AGTAAGT	37	4	99935464	TAGGGCT	4	100154487	R
	ATAGCTT	37	3	124232190	GAACCCC	3	125714880	F
	GGAGGCC	37	4	139531098	CCATGAG	4	139750548	F
	TTTTCTGC	37	1	151967449	AATTTAG	1	150234073	R
	GGAAATC	37	6	158690902	GAAGACC	6	158610890	F
Red	GTTCTGC	37	11	70309970	CGCCAGA	11	69987618	F
	TCTAAGT	37	3	39444897	CGGCCTA	3	39419901	R
Grn	GGGCGCC	37	19	911965	CGCTCCA	19	862965	R
	CCGTCGT	37	2	10302454	TCGATTC	2	10219905	R
	GCTCCCA	37	16	56643342	CGGTCAG	16	55200843	R
	TGTGCTG	37	7	154720452	AGGGCCC	7	154351385	R
	TCATTGG	37	17	27338116	CCAGGCT	17	24362242	F
	ATCCCCG	37	2	133110927	TGTAGAG	2	132827397	R
	TGGCGCT	37	5	1594808	CGGGGAC	5	1647808	F
	ACATGGT	37	22	17672168	CGGAGAC	22	16052168	R
	CTTTTCCC	37	15	95847523	GGCTGTG	15	93648527	F
Red	TTGAGTG	37	6	26598149	TGCATAG	6	26706128	R
	GGTAGTG	37	1	2373387	AGAGGCT	1	2363247	R
	TGTACCT	37	4	185021514	CGGGCCC	4	185258508	R
	TCCATAT	37	6	168363958	CGGCTAC	6	168106807	R
Red	CCGCAGC	37	14	101696494	CGCCCCC	14	100766247	F
	CGGAGCT	37	16	88385387	CCCACCA	16	86942888	R
	GAGTGCC	37	19	50861923	CGGCCAC	19	55553735	F
	CACAAGC	37	5	1462729	GCGGCTG	5	1515729	F
	ACCCCAG	37	4	7812988	GGGAGGC	4	7863888	F
Grn	TTACGTG	37	2	1827764	GTGTTGG	2	1806771	R
	AACAGAC	37	1	43389028	GTCTGTG	1	43161615	F
	TAAACAC	37	4	141923701	ATTTCTG	4	142143151	R
Red	CCGGGCC	37	20	61885236	CGTCCTG	20	61355681	R
	GGAAAAT	37	4	54554571	TACAGCC	4	54249328	F
Grn	AGCAGCC	37	7	158121270	CGGGAGC	7	157814031	F
	TGCAAAA	37	3	72941472	GTTTCTC	3	73024162	R
	ACAGGAC	37	6	29972393	CGAGTGT	6	30080372	R
Red	CGGCGGC	37	3	44622596	GGGAAGC	3	44597600	F
	CAGAAAT	37	6	31087240	AAGGGCT	6	31195219	R
	AGCTAAT	37	16	77535527	GTGCATA	16	76093028	R
Grn	CGCTGGC	37	14	24780682	CGGGAAC	14	23850522	F
	TTCTCCC	37	2	66664871	CGACGAT	2	66518375	F
	AAGGAAA	37	3	45241685	TCAGGGA	3	45216689	R
	ACCATCT	37	16	17898759	CGCTAGG	16	17806260	R
	TTTTTAAC	37	11	49017551	CTGCTGC	11	48974127	R

	CGGCCCT	37	17	10195469	CGGATCC	17	10136194	R
	AACAAAA	37	1	153174654	CGCTTTT	1	151441278	R
	TTTCTAT	37	3	184934396	CGATGGC	3	186417090	R
	GGGCCCC	37	1	228359130	CATGTCC	1	226425753	R
Grn	CCGCCTC	37	7	32945575	CGCCCGC	7	32912100	F
	TTCCCCT	37	6	32829025	GGCCAGA	6	32937003	F
	TCAAAGC	37	11	1455362	CGAGAAC	11	1411938	F
	GTTTCAG	37	2	113912567	AGCACAA	2	113629038	R
	AAAAAAA	37	4	16628713	CGGCTGC	4	16237811	F
	CTGGGCA	37	9	136151958	CTTTCAA	9	135141779	F
	TGTTTGG	37	17	39143567	CAACCTA	17	36397093	R
	TGCGCAT	37	14	93698216	GGATCTA	14	92767969	R
	CACTGCT	37	15	78133639	CGAAGTT	15	75920694	F
Red	GGCGCGA	37	14	24780734	CGTCTTC	14	23850574	F
Red	GCTGCTG	37	14	102422152	GGCACGT	14	101491905	F
	GGGCTAA	37	5	10490335	CGAGGGC	5	10543335	R
Red	CGCGACC	37	16	2229298	CGCCCGT	16	2169299	R
	AACAGGA	37	6	31597708	GTGCCAA	6	31705687	F
	TCATGAC	37	1	1905080	CGGCCCC	1	1894940	R
	GAGCCCC	37	10	105389403	CGCCTCT	10	105379393	R
	CTGATGG	37	16	67978450	CGGCCCG	16	66535951	R
	AGCAGTC	37	16	9326393	CGGGCTT	16	9233894	F
	AACCCGC	37	16	88349881	CGCAGCT	16	86907382	F
	GACTACT	37	10	116337203	CGACTCT	10	116327193	R
	AGGCTTA	37	10	14099154	AATTAAG	10	14139160	F
	CTTAATT	37	22	50174028	CGCAGAA	22	48560032	R
Red	CAGGAAT	37	16	88821905	CCAGGCA	16	87349406	F
	ATGTATC	37	3	9850429	CCCAGGC	3	9825429	R
	TATGATA	37	17	452707	AAAGGAC	17	399457	R
	TTTCAGT	37	X	35937780	TGGTAAC	X	35847701	R
	GCCGCGC	37	14	104394621	AAGCTTC	14	103464374	R
Red	GACCAAC	37	14	102515026	GCGCACC	14	101584779	R
Grn	GCAAACA	37	11	18477303	CGCCTTC	11	18433879	F
	TGGAGGA	37	8	15094107	CGGTGAC	8	15138478	F
	ACTCTAT	37	5	140620284	GCAGATA	5	140600468	R
	GAAGGTC	37	8	141248582	TGCCATC	8	141317764	F
Grn	GAGAAGC	37	5	180671201	AGGTGAA	5	180603807	R
	CCGCCTC	37	2	86092937	CGGGCAC	2	85946448	R
Red	CGTCCGG	37	14	102422310	CGGCTTT	14	101492063	F
	CAGGCAA	37	8	135609134	CGAGGGA	8	135678316	F
	CCAGTTG	37	21	47469104	CGGCACC	21	46293532	F
	TGCAGAA	37	14	32350324	ATGATGA	14	31420075	R
	TGTTTAT	37	9	4116989	CTGGAAC	9	4106989	F

	TATGTTA	37	7	13889871	CGCCTTC	7	13856396	F
	CTGCTTT	37	8	17979647	CGCCCAG	8	18023927	R
Grn	AGGTTTT	37	5	140214372	CCGAGAC	5	140194556	F
	GAAGCAA	37	14	89796856	CAGAATC	14	88866609	R
	TGAGCAC	37	1	108115416	CGGGCAA	1	107916939	F
Grn	CCACGCT	37	5	140627173	CGGCCCC	5	140607357	F
Red	CCGGCAC	37	5	140222653	CGCGGCA	5	140202837	R
	ACGGGTC	37	13	114309225	CCTGCCT	13	113357226	F
Red	CTCCCAG	37	14	76446087	GGCTGTG	14	75515840	F
	GTATGAG	37	1	224753137	AGTTCTG	1	222819760	R
	TGCGTGT	37	7	64894780	TGTCAAG	7	64532215	F
	AAGCCTG	37	14	101493361	CGGATTG	14	100563114	R
	TCTGTAA	37	11	60534911	TCGCCTC	11	60291487	R
	CCCCCTG	37	10	37106999	TATTAGA	10	37147005	R
Red	GCCTTCC	37	7	150020025	GGAGACC	7	149650958	R
	GCTGGAC	37	1	44569177	CGCACCT	1	44341764	F
	AAAGGAC	37	16	69660388	GTTACCT	16	68217889	R
	TCTCAAG	37	16	78027119	TCTTCTC	16	76584620	F
	GGGCGAA	37	11	2022021	CGGCCAC	11	1978597	F
	CTGAGAT	37	2	75785554	CGGCACT	2	75639062	F
	AACCAGC	37	5	1510512	CGGCACC	5	1563512	F
	ACGTATT	37	10	3178915	CTGCTCA	10	3168915	F
	GACTCAG	37	8	89270978	TACATTT	8	89340094	R
	GCAGGGA	37	2	47497026	GCCTCTT	2	47350530	R
Red	GCACACA	37	1	6085995	CAGTGTG	1	6008582	F
	GCGCCAC	37	1	246860007	CGCAGGC	1	244926630	R
	TCCCAAA	37	5	178322737	CGGCCCC	5	178255343	R
	ACCCGCA	37	7	158965466	TCAGATG	7	158658227	F
Grn	AAATTCG	37	14	99725468	CGGGGTC	14	98795221	F
	CCGGCAC	37	8	144302512	CGGGCAT	8	144373887	F
	TGTCAAA	37	2	1658233	AGTTCCT	2	1637240	F
Red	GCCTGAA	37	13	24269867	AGGGGCC	13	23167867	F
	CCAACCG	37	15	78287690	CGGTTCT	15	76074745	R
Red	AATCTGC	37	5	140174841	CGCCCGC	5	140155025	R
	AACGAAT	37	5	159827857	CGGGCTG	5	159760435	F
	ATAATAA	37	16	24266347	CGGCATA	16	24173848	R
	CGGGGTT	37	1	2139235	TGCAGGA	1	2129095	R
Red	CAGCTGG	37	X	30671305	CAGAGGC	X	30581226	F
Red	ACTCTTC	37	10	129794994	CGCAGGC	10	129684984	F
Red	GACGTCC	37	2	1788153	CGCCCCG	2	1767160	F
Grn	GTGACTT	37	6	25882402	CGCCACC	6	25990381	R
Grn	AGGATTT	37	7	64043012	ACGTTCG	7	63680447	F
Red	GTGGGAT	37	18	29304111	CGCAGCC	18	27558109	R

	GCAGGCC	37	8	144966879	TGGAGAC	8	145038867	F
Red	TCCTCCC	37	7	44104860	ACGGACC	7	44071385	F
Red	GTGGCCC	37	4	14451768	ACTATCG	4	14060866	R
	CAACATG	37	3	15311183	CGCCACT	3	15286187	F
Grn	TCGGGCG	37	8	22547688	TGGCGTC	8	22603633	F
	AGGGGGT	37	19	18385672	GGGCCAC	19	18246672	R
	TTAGGGG	37	11	85601106	CGGTGGC	11	85278754	R
	AATTGGA	37	3	174092757	CTTCACT	3	175575451	R
	ACAAACC	37	1	88298973	CGGCCCC	1	88071561	F
Grn	GTTCCCG	37	17	75315527	AGAGAGC	17	72827122	F
Red	CCACGAA	37	17	80970159	GCAGTTC	17	78563448	F
Grn	AGCCGTA	37	1	35586588	CGCGGGA	1	35359175	F
	ATTCATA	37	1	160769081	ACTACCT	1	159035705	R
Red	CAGTCCG	37	12	52258163	CGCCCGT	12	50544430	F
	AAATCTT	37	7	51538921	AACGTTT	7	51506415	R
	TCGCACG	37	12	7070679	CGCCCTC	12	6940940	R
Grn	GCGTTCC	37	16	3345463	CGGCTGT	16	3285464	R
Red	TTACTCA	37	15	96887959	CGACCCT	15	94688963	F
	GGGGCTC	37	1	1142932	AACGTGA	1	1132795	R
	GGCAACA	37	5	141743488	CTCCATA	5	141723672	F
	CCTGGAC	37	16	85747424	AGCCAAC	16	84304925	R
	TAGCTGG	37	1	231820076	CGGGGGC	1	229886699	R
	CAAACCC	37	1	145412774	CTAAGCA	1	144124131	R
Red	CCAGGGA	37	1	53970693	CAGGGCC	1	53743281	F
	GTGGCGC	37	18	501137	AAGGTGC	18	491137	R
	TGAAACA	37	17	80969525	GTGATGA	17	78562814	R
Red	GCCGGCG	37	1	12609308	CGCCCGC	1	12531895	F
	ATTCCCT	37	X	48957691	CGTTGGA	X	48844635	F
	CACTCAG	37	2	85890281	CGCCTCT	2	85743792	R
	CCGTCCC	37	11	18727949	AAGGCTC	11	18684525	F
	GAGAGGA	37	6	30039548	TTTTGAG	6	30147527	F
	CACCTTG	37	17	57918682	CGACTGG	17	55273464	R
	CCCACTG	37	14	33409812	CCTTTGT	14	32479563	F
	TCCAGAC	37	6	14917345	CAGACAA	6	15025324	F
Grn	CCGCTCC	37	1	148854315	TCAGAGC	1	147120939	R
Grn	GCACAGC	37	4	137316569	ACCCGGC	4	137536019	R
	AATCTGG	37	12	94699141	CGACACA	12	93223272	R
	CTGGCAT	37	2	151768348	AGTTTAC	2	151476594	F
Red	GTCCCCT	37	11	64081430	CGAGTAC	11	63838006	F
Red	AGCCGCA	37	16	1187722	CGCACTT	16	1127723	F
	ACGAGCT	37	2	238794837	CACAGCT	2	238459576	R
	AGAGCCC	37	7	158342600	CGTGACC	7	158035361	F
Grn	CAGCTCC	37	16	88497007	CGGCGCC	16	87024508	F

Red	AGGTACC	37	5	140215806	TTGACGC	5	140195990	R
	GTGGCCG	37	10	90712739	CAGTTGT	10	90702719	F
	ACCTTTG	37	15	27035245	GTGGGTT	15	24586338	F
	CTCTGTG	37	1	2336575	CGTCAAG	1	2326435	R
	AGACTGT	37	10	30276523	TCCTTGA	10	30316529	R
Grn	TCTTAGG	37	13	100583872	GAATTCA	13	99381873	R
Grn	CGCTCTG	37	7	63652720	AACCCTC	7	63290155	F
Red	GTTCCGA	37	8	61627281	AGTTACG	8	61789835	F
	TCCAGGG	37	11	130596374	GACTCAT	11	130101584	R
Red	CTAGGAC	37	16	86466427	AGGGAAC	16	85023928	R
	ATCCTCT	37	10	28971331	CGGAGTC	10	29011337	F
	CCTTCTC	37	10	12438782	CGGGGAC	10	12478788	R
Red	TGAGTTA	37	7	47493388	CGGGACT	7	47459913	R
	TGAAGTG	37	12	133304604	GGACCCT	12	131814677	R
	TGAATGT	37	5	36657993	CGGTCAG	5	36693750	R
Grn	ACGCAAC	37	6	170732353	GCCGCAC	6	170574278	R
Red	AGATGAC	37	2	3322283	CGGGATC	2	3301290	F
	ATTGAGA	37	18	42255459	GTTGAGA	18	40509457	F
	GGGCTCC	37	6	161100122	ACCTACC	6	161020112	R
	AATTTGT	37	22	38032768	CGGGGGT	22	36362714	R
	TCCTGGA	37	12	2750053	CGTCCAC	12	2620314	F
	TCAGATA	37	8	127990758	CGGAATC	8	128059940	F
	TAGGAGC	37	11	60701918	CGGCCCC	11	60458494	R
	TGGCAGC	37	16	55794031	CGCTTCC	16	54351532	F
	AATCTGG	37	5	149493034	CATGTGC	5	149473227	F
	GTGGTGA	37	6	139447669	ACAGCTA	6	139489362	R
	GAGCAAA	37	3	30667873	GCCTATC	3	30642877	R
	GTATTCC	37	3	63047382	CGTCCCA	3	63022422	F
Red	CCTCATC	37	6	32976018	CGGACCC	6	33083996	R
	TTCCAGG	37	X	19904780	GACCCTG	X	19814701	R
Red	TTTGCGG	37	12	46411898	CTGGAGT	12	44698165	R
	AAAAACA	37	2	65528222	CGGCGGC	2	65381726	F
	TTGCCAG	37	12	75785089	GAGGACC	12	74071356	R
	ACATTGC	37	17	73316721	CGTTTCC	17	70828316	R
	CAGGACC	37	16	28948179	CGGCCCT	16	28855680	F
	GCGAGCA	37	2	796476	GCAGCTG	2	786476	R
Grn	GATGGGA	37	1	109826064	CGCCCCG	1	109627587	F
	GTGGCAC	37	11	129246718	CGAGGGT	11	128751928	R
Red	CCCCGGG	37	19	1852127	TGTCACT	19	1803127	R
	CCTGTCT	37	15	31764211	GCTGCTG	15	29551503	R
	CTCTTGC	37	7	12151221	GATCTGG	7	12117746	R
	CTGTTGC	37	6	170478434	CGGGAAT	6	170320359	R
	AGACATC	37	6	128977386	CGGAGAC	6	129019079	R

	CCTTGAG	37		16	11012292	GCGAGTT		16	10919793	F
Red	GGCGGGC	37		1	32741518	CGATCCT		1	32514105	R
	TCCCCAG	37		15	48874903	AGACTGC		15	46662195	R
Red	TCCGTAG	37	X		40440191	CGCTTCC	X		40325135	R
Red	TCGAAGC	37		6	30039408	GGGCCGT		6	30147387	R
	TCCCGTG	37		6	31088208	CGGCCTC		6	31196187	F
	TCCCTCG	37		13	112927331	CTCTCCT		13	111975332	R
	GCATCTA	37		7	94953653	CGAGCCC		7	94791589	R
	CTCCCGC	37		4	141391254	CGGGGAT		4	141610704	R
	CTTACTT	37		6	30113978	TTCTGGA		6	30221957	R
	AGGCTCT	37		16	69478081	AAATTAG		16	68035582	R
	TGGATGG	37		10	14504379	CGGAGTA		10	14544385	R
Red	GCAGGGC	37		4	1398139	GGGTCGC		4	1388139	F
	GGATATT	37		4	20195367	AAAGTCT		4	19804465	R
	GAAGCCA	37		8	16908905	AGAAATA		8	16953276	F
	GCTCTGA	37		16	4506231	TGGGTGG		16	4446232	R
	CTGTGAG	37		3	14446645	GCCCCAT		3	14421649	R
	GGGTCCT	37		3	48256968	CGAGTTC		3	48231972	F
	TCATCCT	37		5	53927032	CGGCTGT		5	53962789	R
	CCCACCT	37		18	11948154	GGGTTTT		18	11938154	F
	TGAGAGA	37		3	170806983	CGAGCTG		3	172289677	F
Red	GAATATA	37		6	31650735	AACGAAA		6	31758714	R
	TGTGTCA	37		14	74704714	CGGCCTT		14	73774467	F
	GAGCCCT	37		6	66804631	GAGACTC		6	66861352	R
	CTATCTG	37		8	119152014	CGGGGTC		8	119221195	R
Grn	CGCGATC	37	X		77395088	TCTCCGC	X		77281744	F
	AATCATG	37		11	29207078	CACTGTG		11	29163654	R
	CAGCTGA	37		12	124831017	GGTGATG		12	123396970	R
Red	GTGACCC	37		17	79466438	CGTCCAG		17	77081033	R
	GTGGAAC	37		13	21680693	CGCAGCA		13	20578693	F
Red	GTTTGAC	37		17	693388	AACTTCT		17	640138	R
	ACAGAGT	37		7	47560215	CTGCCTC		7	47526740	F
Red	GTCCCTT	37		2	97505464	CGCGTTC		2	96869191	F
Red	CAGCAGC	37		5	180257795	GCAAATC		5	180190401	F
Red	AAGCAAC	37		17	34817706	CGGGATT		17	31891819	R
	AGATAAT	37		17	53573969	CGTCATC		17	50928968	R
	TCACTTC	37		18	34325827	GTCCAGC		18	32579825	F
	CGCAGGC	37		10	134672797	CGGCCCT		10	134522787	R
	AAGATTC	37		13	33825496	CTCTGAG		13	32723496	R
	TCAATTC	37		1	245843929	CGGCAGA		1	243910552	R
	TGGACGC	37		10	131665884	CGGAACA		10	131555874	R
Red	GGCCTTC	37		14	24780167	CGTGTGC		14	23850007	F
	CACAAAC	37		10	132364391	AATGAGA		10	132254381	F

Red	CCCTGCA	37	16	922132	CGGCAAC	16	862133	R
Red	GGGGCAC	37	X	18443723	CGAAACC	X	18353644	R
	AACATGT	37	7	134833299	CGATGCA	7	134483839	F
	GGTCCTT	37	19	54254388	TTGTTGG	19	58946200	F
Red	TATGATC	37	7	1332973	GCCCCCA	7	1299499	R
Red	CCTTTCC	37	21	47054539	CGCCGCA	21	45878967	R
Grn	CATTATT	37	2	71018354	TCCATTC	2	70871862	R
Red	GCCCTGT	37	20	43935283	CGTGCGC	20	43368697	R
	TAGAAAC	37	7	51539514	CGCTGCT	7	51507008	R
	CGAAATC	37	11	122727440	CGGCAGC	11	122232650	R
Red	CAACTCG	37	16	70794856	CGTGCAC	16	69352357	R
	CTATTAT	37	6	32451079	CGTTACA	6	32559057	F
	TGATACT	37	11	134585270	CGGAAAA	11	134090480	F
	CAGTTTA	37	6	152126180	AAGGTAT	6	152167873	R
	GCAAGTC	37	1	2265881	CGGAGAC	1	2255741	F
Red	CTGGCTC	37	1	221055545	CTGCAGG	1	219122168	F
	TAATGCG	37	1	11917550	CGGTGAA	1	11840137	R
	TGCATGA	37	3	123838429	TGCCATT	3	125321119	R
	TCCCGTT	37	16	55365146	AAAAGTC	16	53922647	R
	AACAGCC	37	1	19210017	CGGGCCC	1	19082604	F
	GAGAAGC	37	16	28943019	CGGTCTC	16	28850520	F
Red	CCGAGGC	37	6	142409756	AAAGGAC	6	142451449	F
	GTCGGCT	37	5	415885	CGGGGTC	5	468885	R
Grn	ACACAAC	37	7	158533890	CAGCGCC	7	158226651	F
	GAGCGCA	37	Y	7142752	GAGTGCT	Y	7202752	F
	TTCTTGCA	37	5	14342307	GGCCGGC	5	14395307	R
	TACCGCT	37	2	139660277	CGCAAGC	2	139376747	R
Red	TAGTTGA	37	6	152126080	TTTTAAA	6	152167773	F
	TCTGCTC	37	2	171680715	TTCACAA	2	171388961	F
	GAGTGTT	37	5	134490074	TGACTTC	5	134517973	F
Red	TGGTCGG	37	16	89036057	GCAAGAT	16	87563558	F
Red	GCTCAGT	37	10	133961825	CGTGCTT	10	133811815	R
	CCTGGTT	37	19	21860793	GTTCCTG	19	21652633	R
	AGGCATC	37	10	5004250	TACATAA	10	4994250	R
	TGTACTT	37	22	42537241	TCATGGG	22	40867185	R
	GCCCTCT	37	10	135040139	TTGCTTG	10	134890129	F
Grn	TCCCGTG	37	12	110906223	CCAAGCT	12	109390606	F
	GAAAGGA	37	4	4587217	CGTGTAC	4	4638118	F
	GCTCTGT	37	12	7948517	ACTTAAA	12	7839784	F
	TGCCCAG	37	17	32909375	AAGAGCC	17	29933488	R
Grn	GCGGGCC	37	16	15489794	GCCGAGA	16	15397295	F
	AGTCCAC	37	5	1420305	TTCCAAC	5	1473305	R
	GCAGAGT	37	11	125552186	CGGGGGT	11	125057396	R

	AGCCGAC	37	17	5019840	CGCCCGC	17	4960564	R
	GCCATGT	37	9	140356830	AGAGGAT	9	139476651	F
	TTGGAGT	37	7	42896801	TTTTTGCC	7	42863326	R
Red	CTCCCGG	37	X	105066526	CGGGGCA	X	104953182	F
Red	CTATGAT	37	16	80966860	CCGGGCT	16	79524361	R
	TTTCCTAT	37	12	66050928	CGGTGAT	12	64337195	R
Red	GGAATTC	37	X	17755642	CGCGAGA	X	17665563	R
	ATGGCGT	37	1	31393915	GACTCTG	1	31166502	R
	TTAAAGG	37	4	146097432	CGGTACA	4	146316882	R
Grn	AGCCGCC	37	4	719908	CGCACCC	4	709908	R
	CAAAGTC	37	7	150485638	CGGCATC	7	150116571	R
	TCACCTT	37	5	33788976	TTCCCATA	5	33824733	R
	GGCCCGC	37	5	13986034	GTGCCAA	5	14039034	R
Red	GGGCCTT	37	4	147164858	CTCTGCTC	4	147384308	F
	AATGAAT	37	2	71983542	TCTAGTTA	2	71837050	F
Red	GATTTCCT	37	20	61877875	CTGAATT	20	61348320	F
Grn	CCTGAGG	37	17	80899280	CAGAAGC	17	78492569	F
	TTCCACTC	37	1	115397409	GAAATAC	1	115198932	R
Red	GAGCCAC	37	18	77246369	TTCTCTGC	18	75347357	F
Red	GGCGCTC	37	16	88496476	CGTCGAT	16	87023977	R
Red	TGCAGGC	37	5	1868357	CGCGCTT	5	1921357	F
Grn	GGCTGAC	37	Y	22917087	AGGAACA	Y	21326475	R
	GTCATCTA	37	5	42840698	CCAAGCC	5	42876455	R
	ACAGTCT	37	5	179588838	CGCTGGG	5	179521444	F
	GGCCAAC	37	5	52598132	AGAGTAC	5	52633889	F
	TTGATGA	37	7	94538894	GCTACAC	7	94376830	F
	CAAACAA	37	Y	6263841	CGCAAAC	Y	6323841	F
	ATTTCTCA	37	X	101915091	ACCGGAA	X	101801747	R
	CTGGCTT	37	8	23712364	AACTGGC	8	23768309	R
	TGCTGGC	37	8	144702879	CCGTGTC	8	144774022	R
	GGTAGAC	37	11	118842413	GGATTGA	11	118347623	F
Red	GGCCTAA	37	3	105542204	CGCCCTT	3	107024894	R
	CATTCCA	37	6	901310	CGTGTTA	6	846310	R
	TAGGGGC	37	6	29942706	CGGGAGC	6	30050685	F
Grn	GGAGGTC	37	5	180028818	GGACGAC	5	179961424	R
	CATTAGC	37	13	99201496	CGATGTT	13	97999497	R
	TGGAACA	37	6	30114011	AGATCAC	6	30221990	F
Red	AGGACAA	37	1	9779309	CGCCTCC	1	9701896	R
	TGAAACC	37	17	8699116	TTTACTA	17	8639841	R
	GATCAAC	37	5	172216309	CGGGGCC	5	172148915	R
Red	GGCAGGC	37	5	71146876	CGCGACC	5	71182632	R
	GGTTCTCA	37	5	140536320	CGAGGTC	5	140516504	F
	GGTTACT	37	7	30855293	AATTTGA	7	30821818	R

Red	TAGCATT	37		1	115397374	CGGGAA		1	115198897	F
Grn	TGCCGAG	37		10	121771388	AGCTCCG		10	121761378	R
	CCCGCCT	37		4	187810446	CCTGCCA		4	188047440	F
	GACTTTT	37		7	11568529	CGCTGGT		7	11535054	R
	AGGTAA	37		19	16302087	CGCTCTG		19	16163087	F
	TGAGATT	37		3	3073276	CGGGGG		3	3048276	R
	GTCGTTC	37		19	4552673	CGGGGG		19	4503673	F
	ACCTGTG	37		5	172347685	AGTGAA		5	172280291	F
	GTAAGTA	37		5	25393894	CTGTGGG		5	25429651	R
	CCAGCCA	37		20	1914231	ACAGAA		20	1862231	F
Red	TGAAGA	37		17	75315108	CTGTCTT		17	72826703	R
	TCAGAA	37		12	312592	CGGGCC		12	182853	R
	AATTCCA	37		2	19849829	CGGGAT		2	19713310	F
	CACCTGT	37		17	20687481	CAGCTA		17	20628073	R
	AAAGGG	37		4	121569769	CGAGTA		4	121789219	R
	GCCGAG	37		7	3134592	CTGCTGG		7	3101118	R
	ATTAGGA	37		12	116814302	CGAGGT		12	115298685	R
	ATAGGGT	37		12	28346883	GGACAA		12	28238150	F
	TGGGCA	37		13	114161463	CGGGAG		13	113209464	R
	ATCTCCC	37		7	148977647	ACTGTGG		7	148608580	R
	GGCACTG	37		15	67417607	CGGGTG		15	65204661	R
	AGTATTA	37		12	52108375	AAAGAA		12	50394642	R
Red	CATCTTT	37		6	32546614	CGGGAG		6	32654592	F
	GCCGTAT	37		1	2107669	CGTGGC		1	2097529	R
	TATCAGG	37		2	221529390	AGTCTGA		2	221237634	R
Red	ATGGGA	37		16	5482346	CGATGA		16	5422347	R
Red	TCTGGGG	37		1	41303570	CGGGGA		1	41076157	R
	AGATTGT	37		17	74073876	CGGCTGG		17	71585471	R
	ATGAATA	37		12	54414427	AGACTCA		12	52700694	R
	CGCGGA	37		5	33938225	CGACCTG		5	33973982	F
Red	CGGTGA	37		16	90114301	GCGGGG		16	88641802	R
	GCTGTTC	37		2	232055508	ACCCAGT		2	231763752	F
	ATCATAG	37		6	32823566	CGGAAG		6	32931544	R
	CCCCTA	37		2	1480789	CAAGGT		2	1459796	F
Red	GCCCCTC	37		16	88534585	CGCACC		16	87062086	F
Red	GAGGGA	37		2	172953134	CGGCCCA		2	172661380	F
	CAGACAT	37		6	155568918	CATTTTG		6	155610610	R
	GCTAAG	37	Y		15451782	AGTGGA	Y		13961176	F
	ATGCCAT	37		4	69435601	AATTGGT		4	69118196	F
	GCAGGG	37		10	27639156	CGCCACA		10	27679162	R
	TGAGCG	37		8	119208486	CGCAAG		8	119277667	R
Red	CGTGCTG	37		8	144809621	CGCGCCT		8	144881609	R
	TGGGTCA	37		21	46847898	TCCCTGC		21	45672326	R

Grn	CAGGGCC	37	8	142112622	TTCCCCA'	8	142181804	R
Grn	TCATTGC'	37	6	6614719	AGTGGGC	6	6559718	F
	TGGTTAT	37	13	77540409	CGGGTAT	13	76438410	F
	GGATACC	37	4	69435250	CGCTGGC	4	69117845	R
	CCTCTGG	37	11	69357823	TAGACCC	11	69067004	F
	GGCATGT	37	16	25598190	CTGCCGT	16	25505691	F
	AATGCAC	37	20	35198387	CACATAA	20	34631801	R
	CAGTGGC	37	6	30720311	CCTAACT	6	30828290	F
	ATGAGAC	37	6	32547019	CAATTTA	6	32654997	F
	GGCAGTC	37	7	111382084	TGAGACC	7	111169320	F
	CGCGGGC	37	11	316247	AAAAGGC	11	306247	R
	GGATGAC	37	3	15311021	CGGAGAC	3	15286025	F
	TTCTCGT	37	18	44557819	CCGATTC	18	42811817	F
	AACATTT	37	6	33038032	GCACTTA	6	33146010	R
	CCGGCGC	37	8	144659831	CCTGGTG	8	144730974	F
	GTGGCAC	37	4	162110754	CCTTCCG	4	162330204	F
	TTTTGGA	37	17	75084281	CGTGAAC	17	72595876	R
	TATTTTTC	37	1	201531514	CGGAAAT	1	199798137	R
Red	GGGCAAC	37	16	28270611	TGAGAAC	16	28178112	R
	GTTTTCT	37	4	90675036	CGGAGTT	4	90894059	R
	TTGTTAT	37	7	130005993	CGGGTGC	7	129793229	R
	GCGGGGC	37	3	71354056	CACACTC	3	71436746	R
	GCTCCGC	37	6	29911334	CGGGGCC	6	30019313	F
Grn	GACGCGC	37	6	29648901	GGACCGC	6	29756880	R
Red	ATTTCTTC	37	3	133502702	CGTCCAG	3	134985392	F
	ACCCCTT	37	12	54385436	CGCCCCC	12	52671703	R
Grn	TGGAGAC	37	14	102959357	GAAAGGC	14	102029110	R
	TGCAGCG	37	15	98196234	CGCCACC	15	95997238	R
	CCTTTATC	37	19	16606621	TCTTTATC	19	16467621	R
Grn	ATTCAAT	37	16	54315634	CCCAGTT	16	52873135	F
	ACATCTG	37	13	97909235	TTTCCGA'	13	96707236	F
Red	TGCTGCT	37	16	2848577	GGGCTGC	16	2788578	R
	CGCCTGC	37	17	79961769	GGGAGTC	17	77555058	F
	TATCCAC	37	21	32255313	CGACACA	21	31177184	F
Red	AGGTCTC	37	7	96652222	CGGGGCC	7	96490158	F
	TGCTGAG	37	17	19620263	GACAGGC	17	19560855	R
	GTGTTGG	37	15	40731325	ACCTCCC	15	38518617	F
	CAGATTA	37	1	245951603	CAGAAAC	1	244018226	F
	CTCCGCA	37	17	78029642	CTACTCT	17	75644237	F
Grn	CATAATG	37	1	248753499	AATATAT	1	246820122	R
	CTGTGTG	37	7	131340667	CGGGAGC	7	130991207	R
	CACACTT	37	1	53595480	CCCTTGT	1	53368068	F
Red	CTGTGAG	37	1	248855364	AGAGGAC	1	246921987	F

	ATTTCCA	37	1	2981840	CGGGTAC	1	2971700	R
	TAGCAAA	37	7	102583144	CGGGTAC	7	102370380	R
	CTGACAT	37	14	31607371	TCAAATG	14	30677122	R
	GAACTGT	37	16	893139	CGGGCTC	16	833140	R
	CACTCTT	37	1	222790218	CGGGGGA	1	220856841	R
	AAGGGTT	37	16	55521135	CTGCATT	16	54078636	R
	GTGGTGT	37	6	139482238	GCTGAAC	6	139523931	F
	GTAAGCT	37	1	171807195	CGCGGTC	1	170073818	F
Grn	CTTCTTA	37	1	167393634	GCGTGCA	1	165660258	R
	TGTGATC	37	3	48335857	TCTTTGC	3	48310861	F
Grn	TCCTTGC	37	16	3242737	CGGCCCG	16	3182738	R
	ATCTTCT	37	11	101300838	CTACTTC	11	100806048	R
	AAGCCCC	37	12	125509502	CGCAGGC	12	124075455	R
	CTCACAG	37	Y	8150875	CGGTGGC	Y	8210875	F
	AGCGCCC	37	1	40599011	TTGGGGA	1	40371598	F
Red	ATCGTAC	37	16	12667646	CGGGAAA	16	12575147	R
	TGGGACC	37	16	58028859	CGGTGCT	16	56586360	R
	CTCATTG	37	16	16951653	TCAGTGA	16	16859154	R
Red	CCCGTCA	37	8	2078112	CGCTATA	8	2065519	F
	AAGGCCA	37	13	98605770	TAGCTCC	13	97403771	R
	CAAGCCC	37	19	18902527	CGGGTCT	19	18763527	F
	AATTGGA	37	6	26457353	CCAGACT	6	26565332	R
	AGACGTC	37	19	50862004	GGATCCA	19	55553816	R
Grn	AGTCAAC	37	3	61235829	GCTCTAC	3	61210869	R
	TAACCGC	37	1	2121449	GAATCCC	1	2111309	F
Red	TGTCTGT	37	17	56247036	CGTGGGT	17	53602035	F
	TCACTGG	37	7	11999601	CGTGAGT	7	11966126	F
	ATGACTG	37	16	19268548	TCATCCA	16	19176049	F
	CAACGTC	37	5	140530606	CGGCTCT	5	140510790	F
	ATGTTTG	37	4	15444984	CGCCACA	4	15054082	R
	TCGTGCC	37	22	24384573	GTGTCTG	22	22714573	F
Red	AACAAAC	37	4	151503501	CGACCCC	4	151722951	F
Grn	GCCGGTG	37	6	30079280	TGAGCAC	6	30187259	R
Grn	TAGAGAA	37	4	941054	CGCGGCC	4	931054	F
Red	TTGGGTG	37	1	16349079	AGCACTG	1	16221666	R
	GGTGACT	37	17	81047721	TTTGTAA	17	78641010	R
	GGGAGCT	37	8	982243	CCTTCCA	8	969650	R
	AGCAGGC	37	5	404713	CGGTTTG	5	457713	F
	TTGTTAA	37	5	112483149	TTCACCG	5	112511048	R
Red	TTTGTTTT	37	19	47636543	CGGCTCA	19	52328383	F
Red	GACTCGG	37	2	864706	CGTGAGA	2	854706	R
	GACCTCC	37	7	128032651	CGGGCCC	7	127819887	R
Red	GGCCCGC	37	19	45905890	TGCTGTA	19	50597730	R

	TCCCAAA	37		1	182858007	CGGCAGT		1	181124630	R
	AAATTCT	37		14	23568017	CGGGCCT		14	22637857	R
	TACAGGC	37	Y		9237650	CATTCCA	Y		9297650	R
	GGCCCCC	37		1	32864238	CACTCTG		1	32636825	R
	TTCCATG	37		7	151290088	CCAGTAA		7	150921021	F
	ATGCAAA	37		11	76998832	CGGGTGA		11	76676480	F
Grn	CAACTTC	37		19	853054	GTCGGCC		19	804054	R
	GATGTGG	37		5	150689593	GCCAGAT		5	150669786	R
	TAGTTAC	37		8	28576644	CGGTTTT		8	28632563	F
Grn	CACCAGA	37		15	45492927	TGGCCGA		15	43280219	R
	GGCAGGA	37		16	27437900	GTGCACG		16	27345401	F
	GTGCAGC	37		15	86038622	CTCCAGA		15	83839626	R
	GATCTGG	37		2	241585355	CGACAGA		2	241234028	F
Grn	CCTGCAC	37		17	74878420	ACAAAAC		17	72390015	F
	GAGGCCC	37		11	65246590	CGGCCAC		11	65003166	F
Red	GACGCTG	37		13	112997541	TAGGCCC		13	112045542	R
	AGGGAGC	37		16	56710807	ACGGAAC		16	55268308	F
Red	TCTATGT	37		15	101266461	TCACGAG		15	99083984	F
	AGGGACT	37		10	13259274	TCTGCGT		10	13299280	R
	AAACCTT	37		4	3392274	AAAGCAC		4	3362072	R
	AGCTTGA	37		16	79692354	ATAGTAC		16	78249855	R
	ACTGCCT	37		6	4133029	GCTTCAT		6	4078028	R
	AAGCCAC	37		21	35320596	CGGCGCC		21	34242466	F
	GTGTTAA	37		12	68814447	TTTATCA		12	67100714	F
	GCTCAAG	37		6	150357289	CGGGTTG		6	150398982	F
Red	GGAGGAC	37		15	78632144	CGCAACC		15	76419199	R
Red	TGGCCCC	37		17	77086388	TACTGTT		17	74597983	R
	GCCCCGA	37	X		10126881	TGTCTAC	X		10086881	F
Red	GAGCAGA	37		16	89225495	CAGGGTC		16	87752996	F
Red	AAGCTTC	37		3	170077111	CGCACCC		3	171559805	R
	TGTTCAA	37		6	80787529	GCATGTA		6	80844248	R
	CTTAACC	37		7	158809128	CAAATCA		7	158501889	R
Red	CGGATGC	37		11	67383691	ATGGGGC		11	67140267	R
Red	ACAGCTT	37		17	56565641	CGCCGGC		17	53920640	F
Red	AGCAGCC	37		11	61322922	CGCCCGC		11	61079498	R
	CAGTATC	37		5	98111529	TCTTTAA		5	98139429	F
	TGCTTTA	37		10	134334241	CGGAAAA		10	134184231	R
Red	ACCCAAA	37		6	42672105	CAGCCTC		6	42780083	F
	AACGGCC	37		3	87036080	AATGCCT		3	87118770	R
	ACCTCAG	37		6	31780239	CCCATCT		6	31888218	F
	TTGTGAA	37		3	149191167	CGTGCTT		3	150673857	F
	ACTGCCA	37		5	112294315	TACACAG		5	112322214	F
Grn	ATGCTCT	37		19	310296	AGGGAGC		19	261296	F

	TCTGCCG	37	9	22005563	GCCTAAG	9	21995563	R
	ACTCTCA	37	7	105134734	ATAGTTT	7	104921970	F
	CCTGGTA	37	16	67605701	GCAAAA	16	66163202	F
	GGAGCCT	37	4	708242	ACGCCGT	4	698242	F
Red	ATTTCAG	37	16	858205	AACCAAC	16	798206	R
	AATCCAG	37	6	29981250	GGGCGCC	6	30089229	F
	TTTTCCA	37	11	34296462	AGCCAGC	11	34253038	R
Red	AGGCAAC	37	7	158155932	CGTGGCC	7	157848693	R
	GGCCGCC	37	13	109793152	CGCCCCT	13	108591153	F
	CTTTCTC	37	7	72868821	CGGCCTC	7	72506757	F
	TTTCTGC	37	12	102137391	CAAATAA	12	100661522	F
	TTGATTT	37	10	90013989	AGTCATA	10	90003969	F
	CATGATT	37	15	93978246	TGAGCCA	15	91779250	R
Grn	TGCCGCG	37	5	1594733	GCACGGC	5	1647733	R
Grn	GTTTTGA	37	7	18794174	ATGAATT	7	18760699	F
	GGCTGCT	37	19	2583601	CGCTCAG	19	2534601	F
	TTGGGAT	37	16	15858790	AGATATA	16	15766291	R
Red	GCCAATT	37	22	42394659	CGCCCCG	22	40724605	F
	TGACTTC	37	6	1684468	CGCCCCA	6	1629467	R
Red	GTGAAGA	37	3	133502540	CCTGGAG	3	134985230	F
	AACATCA	37	5	13745921	CTATTCT	5	13798921	R
	ACATGAT	37	2	112456811	CTCCACG	2	112173282	F
	GAGGCAC	37	6	165902024	ACTTGCA	6	165822014	R
	TCTGGGA	37	19	3135146	GTCTCTT	19	3086146	R
	CGTGTTA	37	3	8813493	CGGGAGC	3	8788493	R
	GAAGCTC	37	1	240078452	CGCAGTC	1	238145075	F
	ACAATGC	37	6	29829406	CGGCAAA	6	29937385	F
	TACCTCG	37	7	95026248	TGCTGCC	7	94864184	R
	TCGAAGC	37	1	156627572	TCTTGTG	1	154894196	R
Red	ATGCAGC	37	X	30671302	AGGGCGA	X	30581223	F
	AGTGGTA	37	3	122684038	TGTCCAG	3	124166728	R
	AGCATAT	37	10	5588169	CGGGTTC	10	5578169	F
	GATAGGC	37	11	78131601	GTGGTCT	11	77809249	F
	GGCAGGC	37	16	88977278	GACAGTC	16	87504779	R
Red	GGGGCTC	37	6	30039130	CTGCGTA	6	30147109	F
	AGATCTG	37	5	168591808	GTCTCAA	5	168524386	R
Red	TCGCGGG	37	10	135273341	CGCCACG	10	135123331	F
	ATCAGGA	37	8	7079106	GTTGAAG	8	7066516	F
	TCGCGGC	37	6	1555572	CGCACCC	6	1500571	F
	TTTCTGA	37	4	77743172	CAGGCAC	4	77962196	F
	GCGTGGA	37	19	58868256	AGGTGCT	19	63560068	R
	GCAACCC	37	10	82295502	CGCAACA	10	82285482	R
	CTTGAAA	37	5	3959743	CGGTGAC	5	4012743	F

	TGCAGGC	37		17	738869	ACGCTTC		17	685619	R
	GGTCTAG	37		21	46075092	CGGCCAC		21	44899520	R
	GCGTGTA	37		7	157874020	GGTTCTC		7	157566781	F
Grn	CGCGTGC	37		6	32064206	CGGCCCC		6	32172184	F
	CTTGGCA	37		19	52218453	CGGCTGC		19	56910265	F
	GCTCCCT	37		3	111730545	CGGCTGG		3	113213235	R
	AGTATGT	37	Y		15015995	TTTCATT	Y		13525389	R
	CTTGGGA	37		5	35771584	CGAGTAC		5	35807341	F
	CGGGGTC	37		18	75886680	CGTGTGC		18	73987668	R
	CCTCTTG	37		1	25246922	AGCGTGT		1	25119509	F
Red	CCCATAG	37		7	117854304	GACGAGC		7	117641540	F
Grn	GGTTTCG	37		11	17249958	CGGCCCT		11	17206534	R
	ATTTTCT	37		7	30387954	CATTGGC		7	30354479	R
	GAACCAA	37		20	62330618	TCAGGGT		20	61801062	F
Grn	CGGGAGC	37		16	3345604	CGGCCCC		16	3285605	R
	CTCCATC	37		11	102048882	TGGTCTC		11	101554092	F
	GCACTGC	37		16	967350	CGCCCAC		16	907351	R
	AACCAAC	37		7	1245797	CGGGGCC		7	1212323	R
	AACACTC	37		2	110969853	AGAGCCC		2	110327142	R
Red	CTTGGCC	37		11	2193041	CGGAGCC		11	2149617	R
Red	GCGAAAC	37		6	31371073	CGCGACC		6	31479052	R
Red	GCGCCGC	37	X		8751402	CGCTTAC	X		8711402	F
Red	AGAAAAA	37		5	180593235	CGCGGAA		5	180525841	F
	ATATTTG	37		11	119806225	CGCTGGC		11	119311435	F
	ACCCAGC	37		5	41869963	CGCCCCC		5	41905720	R
	AGGAGTT	37	Y		15866447	GACTTCT	Y		14375841	F
	TCGCTAG	37		16	2007559	TGAAGCC		16	1947560	F
	TCTATCC	37		8	12549925	TACCCCT		8	12594296	R
Red	ACTCCGT	37		8	103540839	CGCGTGC		8	103610015	R
	TCGGCTC	37		13	28562900	GTTATAT		13	27460900	R
Red	CCGACGT	37		8	10555466	CGCCTTC		8	10592876	R
	ACTGGGA	37		13	113242997	TGCACTG		13	112290998	F
	TGGATTC	37		8	109235928	CGTAGGC		8	109305104	R
	TCAAGGC	37		7	105969911	CGTGGTT		7	105757147	R
Red	AGGAAGC	37		19	49249123	CGGCGCC		19	53940935	F
Red	CGCAGTC	37		2	54087178	CGCCTGC		2	53940682	F
	CTGTCTC	37		17	20687698	GGTATTT		17	20628290	F
	GCGGCAC	37		19	13112808	CAGCTGG		19	12973808	F
Grn	TTTCGCA	37		19	345312	CGCGTTC		19	296312	R
	GTGTTAG	37		10	6364079	TAAATTG		10	6404085	R
	GCAGCTA	37		2	128569178	TTGGTCG		2	128285648	F
	GCCTCAG	37		2	219181531	CGGAACT		2	218889775	R
	GTGGCCT	37		1	239979505	GAATCAC		1	238046128	F

	TGAGACT	37	16	31538718	ACATTTT	16	31446219	F
Red	GCGGCAC	37	X	53123489	CGCCCCG	X	53140214	R
	CCTTTAA	37	12	96188933	CGCCCAT	12	94713064	R
Red	TTGGCCC	37	7	65971372	CGCCGCA	7	65608807	F
Grn	CGCGCGC	37	19	57630524	TCCCCGG	19	62322336	F
Grn	CACACAC	37	6	169326603	CACAAGC	6	169068528	R
	TGGGGCA	37	Y	9214483	CGGGGCC	Y	9274483	R
	AATAATA	37	2	192446259	TATGCCT	2	192154504	F
	CACACGT	37	3	48694454	GCAGGGC	3	48669458	F
Red	GCAGCAC	37	4	141500606	CGCGGGC	4	141720056	F
Red	CCGTGTG	37	1	2425888	CGAGCAT	1	2415748	R
Red	CGGCAAC	37	6	116886276	TGTGAGC	6	116992969	R
	CGTGGGC	37	20	170641	GTTGTGT	20	118641	R
	TGTCTAT	37	3	153778467	CGCATAC	3	155261157	F
	GAGACAT	37	4	185381476	CGGGAAC	4	185618470	F
	AGCCTTA	37	4	53757661	CGAGGTC	4	53452418	F
	AGCCACC	37	Y	2708972	GTACTGC	Y	2768972	F
	TGGAGAC	37	19	5993794	CGGGACA	19	5944794	R
Red	GCAGCGA	37	19	38281047	CGCAAGC	19	42972887	R
	GGGCCAC	37	17	48266593	CGCTGTC	17	45621592	F
	CTTCCTT	37	19	46526489	GCTGGGC	19	51218329	R
	CACAGGT	37	6	134299594	CGTGCAA	6	134341287	R
	TCCTCAC	37	7	6653510	CGCAAGC	7	6620035	F
	GGGACAT	37	19	46526333	GAAGCAA	19	51218173	F
	CCGCAGC	37	13	112630296	CGGCCAC	13	111678297	R
	TGCACGT	37	17	81014091	CCATAGC	17	78607380	R
	TGTGACT	37	3	58319343	CGGGGCT	3	58294383	R
	TTCCAGC	37	10	3509758	CGCACTT	10	3499758	F
Grn	TCCCATC	37	16	88989212	TGCTGCC	16	87516713	F
Red	TCCTTCA	37	17	44898785	CGCAGGA	17	42253784	F
	AGTCTTT	37	17	13297839	GTACATG	17	13238564	R
Red	GCAGCGC	37	19	38886789	CGGCCTG	19	43578629	F
	GCTTAGA	37	11	132992485	CACATTC	11	132497695	R
Grn	AGAGACC	37	4	132896736	CTCTGTC	4	133116186	F
	AGGAAAA	37	11	95433344	AACTAAT	11	95072992	R
Grn	GTGAAGC	37	2	716366	CGGCAGT	2	706366	R
	CTACGCC	37	20	55043694	GAGTCAC	20	54477101	F
	GGGCTAC	37	5	85577255	CGCAGCT	5	85613011	R
	TGCCTGC	37	7	18329916	CGACTTT	7	18296441	R
Red	GATTCCT	37	12	1974122	CGGTCCT	12	1844383	R
	CAGGGCC	37	5	66462471	CCTTCCT	5	66498227	F
	GGAACCC	37	5	135416529	GGGTACT	5	135444428	F
	GCGAGGA	37	11	128321880	CGCCCAA	11	127827090	F

	TTACCTA'	37	11	28650063	CGCCCCC	11	28606639	F
	AATTACC	37	5	34452378	CGGTGGT	5	34488135	R
Red	CGCTGCC	37	6	3293098	CGTGCTC	6	3238097	R
	AAAGGTC	37	11	1892392	CGGAGGA	11	1848968	F
Red	AGCCAGA	37	6	31275881	GCATCTG	6	31383860	R
Grn	CGGCTGT	37	12	2031417	TCAAGCT	12	1901678	F
	ACCCGAC	37	6	37521698	CGTATCT	6	37629676	R
Red	TTTTCCTC	37	5	51426	CGGCTCT	5	104426	F
Grn	CGTGAAT	37	7	51539583	TGGACGC	7	51507077	R
Red	CAGTGGC	37	4	41880319	CGCCAGC	4	41575076	R
Red	CAGCGCC	37	5	1594579	CGCCTCC	5	1647579	F
Red	TCGGCCG	37	6	42694995	AGACCAT	6	42802973	F
	AACGATA	37	2	27713044	CGATGAA	2	27566548	R
Red	TGCGAGC	37	7	156336675	AATCTCA	7	156029436	F
	TTGACCT.	37	X	151999825	CGCAAGC	X	151750481	R
	GAACTGT	37	17	55110904	CGAGCAC	17	52465903	R
Red	CCCTGAC	37	19	55888156	CGGCCCC	19	60579968	R
Red	CTGGGCT	37	16	88053104	CGCCCTG	16	86610605	R
Red	CAGGGGC	37	6	2515318	CGGACGC	6	2460317	R
	TTGGTAC.	37	2	86289505	ATTTGAA	2	86143016	R
Red	CGGGGCC	37	17	20744547	CGCACCC	17	20685139	F
Red	GCGGCAC	37	5	13810279	GTTCGTG	5	13863279	F
Red	TGCAAAC	37	22	18531447	CGTGCCG	22	16911447	F
	CGGAAGA	37	Y	22738029	CGGTTGA	Y	21147417	F
Grn	GAGGGTA	37	4	108641437	AGGAGGC	4	108860886	R
Red	TGCCCCT.	37	3	11178745	ACTCCCA	3	11153745	R
Red	CTCTCTT	37	7	96652481	CGTAGGA	7	96490417	F
	CAGATGA	37	8	40235245	CGCCAAA	8	40354402	F
Red	ACTGGCT	37	6	156983304	CGTCCCA	6	157024996	F
Red	GGCGGAC	37	19	19748461	GAGCCTG	19	19609461	R
	TCTTTCTT	37	8	33211598	ACCAAAC	8	33331140	F
	ACTGCAC	37	3	139060370	CGGAAAA	3	140543060	F
	AGCCACT	37	21	30517941	AAAGTAA	21	29439812	R
	TGCTTTA	37	10	45474339	TTCCCAG	10	44794345	R
	ATATTTC'	37	3	99824269	ACTCAGT	3	101306959	R
	TAAGGAA	37	1	149979253	GTTAACT	1	148245877	R
Grn	GGGGCCA	37	5	346238	CGCAGGC	5	399238	R
	TGCAGCT	37	17	66573573	ATCTCCC'	17	64085168	R
Grn	ACTTCTT	37	13	112062652	CGGCGGA	13	110860653	R
	CGCCACG	37	1	33772997	AGAAGTA	1	33545584	F
Red	AAGCATC	37	6	169689892	CGGCTTT	6	169431817	R
	AAAGTTC	37	12	109596187	CGATGAC	12	108080570	F
Red	TCTGGCC	37	17	77111749	CGGCATC	17	74623344	R

	CTCCTAT	37	13	21680560	CGTGCCG	13	20578560	F
	AGCAGTC	37	5	2388038	CGGCTGC	5	2441038	F
Grn	CGGGACC	37	1	41350183	CGCTGCT	1	41122770	F
	TGTCTTG	37	2	95788279	GGGTAGC	2	95152006	R
Grn	CGCCGCA	37	19	51607432	CGGGCCA	19	56299244	F
	TATTAAT	37	1	161572679	CGCATTC	1	159839303	R
Grn	GGGGCTC	37	3	10149979	CTGAACT	3	10124979	R
	GAAGTGA	37	5	168894603	GCTCCAG	5	168827181	R
	GGTTCCA	37	3	8631797	CGGCAGC	3	8606797	F
	TTTTCCA	37	10	85955302	CGGAAAC	10	85945282	F
	GGGACCT	37	15	89958944	CGTGGGC	15	87759948	F
	ATAATTA	37	6	114515500	CGAACTG	6	114622193	F
	AGAGAA	37	17	13941263	TCAGTGA	17	13881988	R
	TCTGCTA	37	17	8263420	CGCCAAA	17	8204145	F
	CTCATTT	37	16	7917211	TAGGCTG	16	7857212	R
	ATTAGTA	37	8	126649807	GCATTCT	8	126718989	F
Red	CCTGGTC	37	5	415575	CGGCCCC	5	468575	R
Red	GCAGTGA	37	11	2584917	TAATCTT	11	2541493	F
	ATGTTAA	37	1	7462176	CGTCAAA	1	7384763	R
Red	GCAGCTG	37	16	420230	CGCCAGC	16	360231	R
	CTAATGC	37	13	31455173	CGGAGAC	13	30353173	F
Red	GACACCG	37	17	80297325	CGGCCAC	17	77890614	F
	GTGTGTG	37	1	1111095	CGCAGGC	1	1100958	R
	CAGGCAC	37	7	27211117	CGCCAAC	7	27177642	F
	TGCACTG	37	5	106801945	CGGCTCA	5	106829844	F
	GGGTCCA	37	1	248100614	CTGCCCT	1	246167237	F
	ATACAAG	37	11	132946455	CGCCGCT	11	132451665	R
	GTCTCCA	37	5	87661076	CGGGTAC	5	87696832	R
	CCCAACA	37	17	7960424	TCCTTGA	17	7901149	R
	AGAAGG	37	2	77235218	TAAACCA	2	77088726	R
	TGCTGTG	37	6	32634009	GGAGAA	6	32741987	R
	GTGCTGA	37	11	3535099	CGTCTTC	11	3491675	F
	AAGACA	37	6	40323935	TATTGTT	6	40431913	R
	GTCAACT	37	10	133036065	GAACACC	10	132926055	F
	AATCTCT	37	3	168308798	CGGCCTT	3	169791492	R
	AGTATGA	37	2	183321813	CGTGTAC	2	183030058	R
	AACAGAC	37	5	140589751	CCAGCAC	5	140569935	F
	CATGATG	37	4	183062576	TTGAGGC	4	183299570	F
	AAAATAA	37	7	126866923	TCACATC	7	126654159	R
	GCAAAAC	37	19	2525459	ACTATGT	19	2476459	F
Red	TCCCGCC	37	X	118987153	GACAGAC	X	118871181	F
	AAACAGA	37	6	130747460	GCCCACT	6	130789153	R
	GCCTGGC	37	3	11010256	CGCCCTG	3	10985256	F

	AAACGAC	37	X	70752659	CGGATAC	X	70669384	R
	TATATGG	37	1	95167783	TACTGTT	1	94940371	R
	CACTTAG	37	3	8704494	TGTCATT	3	8679494	R
	TCAGTCC	37	20	44657115	CGCTGGG	20	44090522	F
Grn	AAACAGA	37	7	158110068	CAGGAAA	7	157802829	R
	GCCCCCA	37	16	34404054	CGCCAAC	16	34261555	F
	GCTCAGT	37	20	52199594	CGAGGCA	20	51633001	R
	CAAATGT	37	7	84177905	CCTACCA	7	84015841	F
	TGTCCTG	37	5	103849273	CGGGCAC	5	103877172	F
Red	AGCAGGC	37	16	85202191	ACTGTCC	16	83759692	R
	GGAGGCC	37	8	1365175	CGGGGAC	8	1352582	R
	AACTTTG	37	8	95113521	CGTGTGA	8	95182697	F
	TGATGAC	37	6	32610735	CGCCATC	6	32718713	F
	TATATTA	37	3	44802549	CGGAGGA	3	44777553	R
	CAAGGAT	37	12	115117551	CCAGGGC	12	113601934	F
Grn	ACAGGCC	37	16	1709722	ACGGCAA	16	1649723	F
Grn	TCCTCGG	37	16	4462122	CGGGGTC	16	4402123	R
	AACAAGC	37	2	12147206	GCAGAAA	2	12064657	R
	CCTCTGG	37	16	3142911	CGGCAGC	16	3082912	F
Grn	TGCCACG	37	1	228246780	TCGGGCA	1	226313403	R
	TTTCCAT	37	5	1245902	TCCAAGT	5	1298902	R
	AATGGAC	37	16	10837135	CGGGACA	16	10744636	F
	AGTCCTG	37	17	13927712	TTACCAG	17	13868437	F
	TTGAACC	37	10	61596106	CGTCTGT	10	61266112	R
	GCATTTT	37	12	123757860	CGGTGAC	12	122323813	R
Grn	CACACAC	37	5	346247	ATGAATT	5	399247	F
	GCTGTCT	37	15	96890598	CGCCCCT	15	94691602	R
Red	AGAAAAC	37	16	936993	CTTCTTA	16	876994	R
	AGAGGAA	37	8	48675911	CGCATTG	8	48838464	R
Grn	TCAGTCC	37	8	48676054	GACCCAC	8	48838607	R
	GTTTGGT	37	5	43487508	CGGGAAT	5	43523265	F
	TCTGTAA	37	3	164659583	TGAAACT	3	166142277	F
	CCAACAC	37	12	69769552	AACCCCG	12	68055819	R
Grn	TAGGTGC	37	20	2361461	CAGGATC	20	2309461	R
	CAAAATA	37	1	79594960	CGTGCTG	1	79367548	F
Red	TACCTTG	37	16	31439393	CGGGCCA	16	31346894	R
	CTTCTGC	37	X	119133700	TCCCTCT	X	119017728	F
	GAGCTTT	37	4	40675171	CGTGGGT	4	40369928	F
Red	GATGGTT	37	8	103750904	CGGGCGC	8	103820080	R
Red	GGTGGAC	37	5	140625848	CGCCAAT	5	140606032	F
	GAGGCCT	37	11	95962058	CGCGTGA	11	95601706	R
	GAGTGTT	37	1	221070193	TGGTAAA	1	219136816	F
	CCTGGCC	37	16	10922178	CGGGGAC	16	10829679	R

	TGGCTAA	37	X	138525808	CTCCAAA	X	138353474	F
	CCGGGAA	37	1	17004834	CGCCTCT	1	16877421	F
	AACCTAC	37	12	14926986	TAGTACG	12	14818253	R
	GCTGTCT	37	13	111989685	TCCTATA	13	110787686	F
	AAGTATC	37	20	61455862	GCCAGGT	20	60926307	R
	GTAGTCT	37	1	211926526	TAGGAGC	1	209993149	R
	TGCCTAA	37	8	1914991	CCGGAAC	8	1902398	R
	GCACACA	37	20	55665224	CGCAGGA	20	55098631	F
Red	AGGCCAC	37	6	106434429	CGGTCCG	6	106541122	R
Red	GCGCTTC	37	6	29856353	CGAGGAC	6	29964332	F
	ACACAAC	37	14	100016312	TGCCCGT	14	99086065	R
	AGATCCT	37	15	90963532	CGGTAAC	15	88764536	F
	GAAGGGC	37	1	25734099	TGTTTGT	1	25606686	F
	AGAGATC	37	6	52217264	GTTTCAC	6	52325223	F
	CCTCTCT	37	12	19167591	CGTGGCA	12	19058858	F
	AGCACGC	37	16	49518414	GCCCTTC	16	48075915	R
Grn	TGTGTGT	37	1	94560697	CGCAAGT	1	94333285	R
Red	CCTCGTT	37	19	384251	CGGGCGC	19	335251	F
	CCCATCA	37	11	5279386	CGCACAC	11	5235962	F
	GGTGGGA	37	17	695428	GGACCTG	17	642178	F
	CACTTAT	37	6	128530306	CAAGTTT	6	128571999	R
	TACTTTG	37	2	230664097	CTTGGCC	2	230372341	R
	AGTTTGC	37	14	52347968	CAGCACA	14	51417718	R
	GGAGTCT	37	14	73075591	CGCATGC	14	72145344	R
Red	AGCACCG	37	17	1546403	CGCCCCG	17	1493153	F
	TCTCTTC	37	1	147806668	CGGGTTT	1	146273292	R
	GATTCAT	37	7	98438683	GGCATTC	7	98276619	F
Grn	GCTGATC	37	6	30039403	GCTTGGG	6	30147382	R
	CAAGCCT	37	12	124585152	CGGCCAT	12	123151105	F
Red	CGGAGCC	37	19	51190179	CGCGCCC	19	55881991	R
	CCTAGAC	37	X	148556545	CGTGATG	X	148364449	F
	GAACCAA	37	3	184528162	GCTAAGC	3	186010856	F
Red	CAGCGCT	37	6	28698008	CGAGAGC	6	28805987	R
	AGAGCAC	37	7	101208183	CGCCCCC	7	100994903	R
Red	TGGCTGC	37	6	29692035	CGGCAAC	6	29800014	F
	CCAACAT	37	7	57324701	CGCCATT	7	57328643	R
	CCTGGGG	37	14	69430369	AACTCAT	14	68500122	F
	AATGGAC	37	17	30770961	CGCCCAT	17	27795074	R
	AGTCAAT	37	6	32632000	AGGTACC	6	32739978	R
	CTGGATT	37	12	46009735	TGCATAC	12	44296002	R
Red	GAGCTGA	37	6	106434131	CGCAACC	6	106540824	R
	TGGGCCC	37	12	113535362	GTGGAAC	12	112019745	R
Red	CCGTCGA	37	2	176936436	CGCCGCC	2	176644682	R

	CGGGGCA	37	X	14048281	AAAAGTT	X	13958202	F
	TTCCACG	37	10	126686762	GCTGGGA	10	126676752	F
Red	CGCTCGC	37	14	73958573	GACAGAC	14	73028326	F
	TTCATGA	37	11	70266139	CCAACTT	11	69943787	R
	CTGCGCA	37	19	21265421	CGCCGCT	19	21057261	R
Red	CCGCGTG	37	16	85242446	CGCCAAC	16	83799947	R
	CTGGTCT	37	14	61933059	CGCCATA	14	61002812	R
Red	AGAGCAC	37	7	96650096	CTAATAG	7	96488032	R
	CATATTT	37	1	94587905	CGGTCCC	1	94360493	F
Red	GAACAGC	37	2	231715028	CGTTGGG	2	231423272	R
Grn	GGCAGTC	37	1	242220475	ACCGGAT	1	240287098	R
	AAATTAC	37	1	209877970	CGGGTAC	1	207944593	F
	GGGTTGA	37	10	127560294	TAAAGCT	10	127550284	F
	CCCCACA	37	6	30772293	CGCCACT	6	30880272	F
	GAGTAGA	37	6	28483411	CCTTGCA	6	28591390	R
	TGCTCTC	37	17	53847116	GCTAGAC	17	51202115	R
Red	AAGCCTG	37	7	156309358	ACTGCGA	7	156002119	F
	TCTAATA	37	6	101306732	CGTATTT	6	101413453	R
	GACTGTT	37	17	29645782	AGTAGAT	17	26669908	F
	ACAACTC	37	2	216593517	AGAACAA	2	216301762	R
	ACCTGAT	37	2	7342228	CGGGGAC	2	7259679	R
	TCCTGGC	37	Y	20501241	CGGGTCA	Y	18960629	F
	AGAACAT	37	2	224564400	ACACCAT	2	224272644	F
	CAAGAAT	37	6	32607224	CGAAGGC	6	32715202	R
	ATCCTTT	37	16	11603344	CGAGTGA	16	11510845	F
	TCATCCT	37	16	11267859	TTGCATG	16	11175360	R
Red	ATTGTCT	37	1	16490791	AGACTAC	1	16363378	F
	ATTTTTCT	37	3	49538552	AGAGCAT	3	49513556	R
	CTAGCAC	37	21	47716443	CGCCCTG	21	46540871	R
	GAGTCAC	37	12	113215623	CAGCACA	12	111700006	R
	AGCGGTC	37	9	123605666	CTTCGTC	9	122645487	F
	GCATAGT	37	15	85552114	TGCCTGG	15	83353118	F
	AGGTGCA	37	16	89167018	CGTGGCC	16	87694519	R
	CGGGGCC	37	8	128427306	CGGGGGC	8	128496488	R
	CCGGGCA	37	5	499552	CGGCACC	5	552552	F
	TAAATAG	37	4	184256627	CGGGAAA	4	184493621	F
Red	CACACCA	37	16	78539954	CGGATTG	16	77097455	F
	CCCTGGG	37	4	187581093	CGCTGGG	4	187818087	F
Red	GCTTGTT	37	17	72565887	CTCGTCT	17	70077482	R
	GCAAAGT	37	15	98514710	ACTCCCA	15	96315714	F
	CAATGAT	37	6	82459960	TGAATGT	6	82516679	F
	GAGAGGT	37	3	47388736	CGGATCT	3	47363740	R
Red	GGTCTTT	37	19	3191030	GCAACTC	19	3142030	R

	CTTTCCT	37	2	36825704	CGGTACC	2	36679208	R
	CGGGTCG	37	2	71944165	GCTATAT	2	71797673	R
	CTAGCAC	37	16	47882395	CGCATAT	16	46439896	R
Red	CCGGGAC	37	17	78935269	AGCCTGG	17	76549864	R
	TGGAATT	37	16	87712353	CGTTGGA	16	86269854	R
	CTGTGAA	37	5	150169781	CGGGGCT	5	150149974	R
Grn	GGCGGCC	37	19	39056084	TGGGGTC	19	43747924	F
	TTGACGT	37	6	33092130	CGGGATC	6	33200108	R
	TGCCCGC	37	17	78735324	GAAATGA	17	76349919	F
	TGAATCT	37	6	149097976	CGGATCC	6	149139669	R
	ATCCTTG	37	2	181337276	ATGTTTT	2	181045521	R
	TCTTTGT	37	8	97747124	GGCTCTA	8	97816300	R
	GAGATGA	37	8	55060709	CGGCTAC	8	55223262	F
	AATGCTC	37	5	143397012	ATCCATG	5	143377205	F
Red	CCACCAT	37	10	100175082	CGGGAGT	10	100165072	F
	CAGCCAA	37	2	241776193	CGGGCTA	2	241424866	R
	GGGAGG	37	12	2339614	TCAGGTT	12	2209875	F
	AAGCCTT	37	12	4699232	CGAGAA	12	4569493	F
	TCATAAT	37	11	18414888	TCATACT	11	18371464	F
	GCCTGGG	37	16	72096944	CGGGAA	16	70654445	F
	GGTGCCC	37	17	37024020	AGGTGGA	17	34277546	F
Red	TATTTGG	37	6	33288332	TCTTCCC	6	33396310	F
Red	GGTCTCC	37	X	149530886	CGCTGGC	X	149281544	F
	ATGTTCC	37	13	22458760	GATGCTA	13	21356760	R
	ATGTTCT	37	3	64725835	AGGTTAG	3	64700875	F
	TTTTGGG	37	3	71545533	ATGGCTG	3	71628223	F
	AGGAGA	37	3	64670013	CGGGCGA	3	64645053	F
Red	ACTTTAT	37	7	155817478	CGGGAAT	7	155510239	R
Red	TCTTCTG	37	22	18985632	GGGCAA	22	17365632	R
Grn	TTTGCTT	37	4	154605468	CGTACCT	4	154824918	F
	CACGCAC	37	7	123459295	CGTGTTC	7	123246531	F
Red	AAGTGAC	37	7	229695	CGGCACC	7	324778	R
	CAGTGTA	37	11	114165661	ATTGACA	11	113670871	R
	AGTTGTG	37	18	77134251	TGGTAAA	18	75235239	R
Grn	ACGCGAC	37	17	38024394	AAAAGTT	17	35277920	R
Red	AGCGGGT	37	7	158790006	CGCTTCC	7	158482767	F
	TGGCGTT	37	16	85242503	CGGCAA	16	83800004	R
	GAGGACT	37	16	48157560	GGAAAT	16	46715061	F
	ACCCAAA	37	20	62225654	CGCCCTG	20	61696098	R
	CGAGGA	37	12	131030259	CGCCGTG	12	129596212	F
	TTTGATG	37	5	89352028	CAGGACA	5	89387784	F
	AACCACC	37	1	72190606	CGAGCCT	1	71963194	R
	CCATCAA	37	X	9755283	CTCGGGT	X	9715283	F

Grn	TCGGATC	37	X	56590113	CGGCCTA	X	56606838	F
	CAGAGTA	37	5	178455197	CGCAGCC	5	178387803	R
	CCACTTC	37	1	153521909	GAGTCAC	1	151788533	F
	CAGAGTT	37	7	157628140	CGGGTTC	7	157320901	R
	GAAGCAC	37	5	175241829	CGGCTGT	5	175174435	R
	AGAGAGC	37	16	85196350	CGAGTCC	16	83753851	R
	GCCTGTG	37	13	112630399	GGGAGAC	13	111678400	R
	ATTTTCA	37	5	146716604	TTTACAA	5	146696797	F
	TACCATT	37	6	17866039	CGGACAC	6	17974018	F
Grn	GGAGACC	37	16	12618726	CGGCCCA	16	12526227	R
Grn	GAGGCTC	37	7	2962240	GGCGAGC	7	2928766	R
	ACAAGAA	37	8	102550720	CGCCAGA	8	102619896	R
	CATAAAA	37	11	193112	GACTCAC	11	183112	F
	GGCTGCT	37	21	45336903	CGGGCTC	21	44161331	R
	AGGAGGT	37	12	125223650	TCATGCC	12	123789603	F
	CAGCTGC	37	4	1047097	GCTGCAT	4	1037097	F
	GCCGTTT	37	X	133118344	CGGACAA	X	132946010	R
	AGTCAGT	37	6	3473380	TAAAAAC	6	3418379	R
	TCATCAG	37	4	183935060	ACAGAGC	4	184172054	R
	ATCCCCT	37	11	107583377	CGTGGAT	11	107088587	F
	ATGGGAC	37	20	32857151	CGAGCAC	20	32320812	R
Red	GACCAGC	37	21	45336673	CGCACAC	21	44161101	F
	GTCACTC	37	19	24269890	TCAGCCT	19	24061730	R
	CCCATGA	37	15	25442386	ATGGACT	15	22993479	F
	GCCTCAG	37	15	92058845	CGGAGGT	15	89859849	R
	ACCATAA	37	15	60644157	CGGTGAC	15	58431449	F
Grn	CCCCTCA	37	16	3016378	CGGCCCC	16	2956379	F
	GTGAGTC	37	15	33571230	CGGCTTC	15	31358522	R
Grn	TTAACAC	37	4	183062729	TTAATAC	4	183299723	F
	TGGTGGG	37	10	102111107	CGGGCAA	10	102101097	R
	TACTACC	37	4	183063534	CGGGCTG	4	183300528	F
	TACTACG	37	6	168815615	CGCCAGC	6	168558464	R
	GCTTGGA	37	14	50490901	GCTGCTC	14	49560651	F
Grn	TCCTGCA	37	1	211652688	TTAGACG	1	209719311	F
	TCCTGTT	37	3	159590447	GTAGTCT	3	161073141	F
	GGAAGGC	37	1	39212920	CCGTGGC	1	38985507	R
	TTCTGTG	37	2	71723690	TGGGCAC	2	71577198	R
	ATATTGA	37	Y	2823782	AAAAAAT	Y	2883782	F
	TCTTGAC	37	5	131561058	TTGGGAG	5	131588957	F
Red	CTGCGGG	37	12	124907162	CGGCTGC	12	123473115	R
	GCATATT	37	14	63121958	CGCATAT	14	62191711	R
	TGGAATT	37	2	108745580	CGGCTCT	2	108112012	R
	GTAGGGC	37	1	35586503	CGCCTGG	1	35359090	R

	TGGCAGC	37	3	48466751	CGCCTGC	3	48441755	R
	TGGAATT	37	7	22764397	CGCAAGC	7	22730922	F
	GAGGAGC	37	1	230250326	GGTGGCA	1	228316949	F
	AAAATGA	37	20	24929822	CGCCCCT	20	24877822	F
	AGCATCT	37	10	131930596	CGATTGG	10	131820586	R
Red	GGCATTA	37	Y	15016590	TTACTTC	Y	13525984	F
	CCAGATA	37	6	4610575	TTAGCCT	6	4555574	R
	GACTGTA	37	3	124861521	CGGCATG	3	126344211	F
	TCCAAAC	37	5	135329478	CGCTGGT	5	135357377	R
	AGGCTGT	37	4	6665095	TTGGGAC	4	6715996	R
	TAGTATA	37	7	157913147	ACCATGA	7	157605908	R
Red	TGGGTGT	37	12	95943114	CGCCCTT	12	94467245	R
	GAGACAC	37	15	89595811	CCCAATC	15	87396815	F
	GGGGCGC	37	4	2366831	GCTGAAC	4	2336629	R
Grn	GGGCTAC	37	20	57463991	TCACCGA	20	56897386	R
	AGTCTTC	37	13	103532304	CTTCCGG	13	102330305	R
Red	GTAAAGC	37	1	24968182	AGTCAAC	1	24840769	R
Grn	TATCAGG	37	6	28584076	CGCGGCC	6	28692055	F
	AATATTC	37	5	10226648	ACAGATA	5	10279648	R
	TGGCACA	37	17	75315244	CGGGTTT	17	72826839	F
	TTTTCAA	37	5	77808947	CGGGTGA	5	77844703	F
	GGGAGAC	37	15	102025217	CCAGGAT	15	99842740	R
	TGATCCG	37	12	111620313	TCAGAGC	12	110104696	R
	GCCCAAC	37	16	87248612	CGCCTTG	16	85806113	F
	ATTTATA	37	10	65460017	GCCAGCC	10	65130023	F
Grn	ACCCACA	37	2	1886105	GCTGACT	2	1865112	F
	GATTATT	37	4	90481402	TTCTAGT	4	90700425	F
Red	TACAATA	37	13	112063695	CGCGGCA	13	110861696	R
Red	CGCACGC	37	8	145728526	CGGCCCC	8	145699334	R
	TGCACTC	37	16	4017270	CAAGCAC	16	3957271	F
	TTACACA	37	X	7892382	CGGTTCT	X	7852382	R
	AACAATC	37	2	118614378	GGGCAGC	2	118330848	F
Red	CTATCGC	37	10	82296072	CGGTCCT	10	82286052	F
Red	TTGTGGC	37	2	112898400	CGGCGAC	2	112614871	F
	TGACAGC	37	15	24346942	AGGGCGC	15	21898035	R
	GCATTTG	37	1	44300942	CGGTCGC	1	44073529	R
	CTTTGGT	37	14	76289228	TAGGGAA	14	75358981	F
	TGGCCTC	37	10	131529435	GAGCTCT	10	131419425	R
Grn	TGACCCC	37	11	8931473	AGCGTGT	11	8888049	R
	CTGTGTG	37	14	32926900	GGCGCTG	14	31996651	R
Red	CGCCGCC	37	X	30907439	AGCTTGC	X	30817360	F
	ACTCCTT	37	2	65085243	CGGGAAA	2	64938747	R
Grn	GCTGAGC	37	1	207669922	ACCCGGC	1	205736545	R

Red	GAGGCCT	37		7	1952561	CGGCCGC		7	1919087	F
Red	CCCAACC	37		21	15352983	CGCCGCT		21	14274854	F
	GAGCCAC	37		16	1939295	CGGTGGC		16	1879296	F
	TATTCAA	37		4	16255438	CGGTGAC		4	15864536	R
Red	CCCCACG	37		7	36194394	CGGAGA		7	36160919	F
	ATCAACA	37		10	91453851	TCAAGGT		10	91443831	R
	CCCTTCC	37		12	53822143	CCTTCAT		12	52108410	F
	AAGAGAC	37		10	729846	ACTGGAC		10	719846	F
Red	GGTTGCG	37		2	48844971	CGCAGTG		2	48698475	R
	TTCCTTC	37	Y		8681688	CGGTGAT	Y		8741688	R
	TTGTAAA	37		2	114036953	GGAGGG		2	113753423	R
	TGGGTAT	37		7	27475596	AAGTGA		7	27442121	F
	ACGCAGC	37		4	152148822	TTTAAAT		4	152368272	R
	CCTCTCC	37		8	142139187	CGGGGCC		8	142208369	R
Grn	ATGTTCC	37	Y		21729144	CCAATCG	Y		20188532	F
	AACTCTC	37		2	100908637	CGCCCTG		2	100275069	F
Red	GGCCTGG	37		12	107348855	CGCGGAT		12	105872985	R
	TCTCCTC	37		7	25991722	CGGGCAC		7	25958247	F
	CAGCACC	37		10	121837310	CGGGAAT		10	121827300	R
	CAAATAA	37		8	16637442	AATTATA		8	16681813	F
	CTGGATC	37		11	118869922	CGAAGA		11	118375132	F
	CAGCCGA	37		8	12458558	CGGAGTT		8	12502929	F
	CCCAACT	37		1	2844356	AGTGGAT		1	2834216	R
	CAACCAC	37		14	25591076	CGGGAGC		14	24660916	F
	CCTTCCT	37		11	68550390	TGACTCC		11	68306966	R
	TGCTTTT	37		12	18845488	CGAGAGT		12	18736755	F
	ATCTGAA	37		10	107733752	CGGGGAC		10	107723742	R
Grn	GACAAAA	37		12	3563899	AATAGGC		12	3434160	F
	CATACTC	37		12	19925091	TAATACA		12	19816358	R
	CAGCACC	37		6	32526414	CGCAGGC		6	32634392	R
	AGTGGGA	37		4	79052724	GCACAAA		4	79271748	F
	GTGAGCC	37		17	20203992	TCCTGGG		17	20144584	R
Red	CTGGGAC	37		16	88472541	GGGCGGT		16	87000042	F
	CTCCTGG	37		3	12197630	GCATGGC		3	12172630	R
	GGTCGGT	37		1	113067124	CCCGTAT		1	112868647	F
	TGACGTA	37		6	31635971	CGCATTT		6	31743950	R
	GGGAGAC	37		1	111740345	AGTTGTA		1	111541868	R
	CTGCCAC	37		7	624424	GGCTGGC		7	590950	F
	TCACTGT	37		14	69134577	CTGCCTT		14	68204330	F
Grn	ATGAAAT	37		13	111935412	GGCAGAC		13	110733413	F
Grn	CGCCCAA	37		19	57630596	CGGCGCC		19	62322408	F
	GACCTCG	37		12	101603453	GGTGCCC		12	100127584	R
	AACTCCA	37		19	45187222	CGGGCCT		19	49879062	F

	GCCACAC	37	1	43473978	ATGGCCC	1	43246565	F
	TCCTGCT	37	8	53235234	AGAGAGA	8	53397787	R
Red	ATCTGAA	37	22	50482250	CGGTTTT	22	48824377	R
	TCGGGGC	37	14	92303710	AGACTTC	14	91373463	F
	TGTGCCT	37	20	4721766	CGAAGGA	20	4669766	R
	AAAATTC	37	5	177941617	ACTTGGT	5	177874223	R
	GGGAGTC	37	6	72004311	ACTGTGT	6	72061032	R
Red	CCCTGCC	37	1	228756711	CGGCCCG	1	226823334	R
	GTCAGGA	37	15	57825722	AGGATGA	15	55613014	F
	TCAGTTC	37	15	42302705	CGGTGAC	15	40089997	F
	AGCTGAC	37	3	171002363	CGGCAGC	3	172485057	R
	TGCGCCT	37	7	138349158	GTTAGGC	7	137999698	F
Red	CACCGGA	37	14	24780691	CGGCCTT	14	23850531	F
	CAGCAGC	37	8	2034467	AAATTGG	8	2021874	R
Red	CACAGAC	37	5	126409007	GCCGCGT	5	126436906	F
	CCATGGT	37	16	88052956	CGAAGAT	16	86610457	F
	CCGTGGC	37	14	92303685	CGCCAAC	14	91373438	F
Grn	CAATGGC	37	5	25190672	CGGCCAA	5	25226429	F
Grn	AGAGCTT	37	5	140215762	GCGGTCT	5	140195946	R
	CTCTGGG	37	8	23775498	TATTTGA	8	23831443	R
Red	GGGGTCT	37	Y	2802975	CGCCTCT	Y	2862975	R
	TTGTGAA	37	7	26183581	TATCTCC	7	26150106	F
Red	TTATATG	37	7	48129814	AGTCACC	7	48096339	F
	TCATAAA	37	6	32607174	CGGGGCT	6	32715152	F
	ATCTAGC	37	1	60171660	CGGCCAC	1	59944248	F
	GGTTGTT	37	12	47618577	GGGATGT	12	45904844	R
	TATTAAA	37	2	168673805	CGTATAA	2	168382051	F
	CACGGAA	37	16	89647782	AACCAGC	16	88175283	F
	CTCACAC	37	12	6585594	CTTTTTC	12	6455855	R
	GAGAAGA	37	16	88497293	CGGGGTT	16	87024794	R
	GTGGACC	37	1	244920686	CGGTGCA	1	242987309	R
	AAGGACT	37	1	88026790	ATAATGC	1	87799378	R
Red	TCCGTGG	37	13	102105129	CGGTGAA	13	100903130	F
	CCCGAGC	37	17	1096705	CAGTATT	17	1043455	R
	TGGGACA	37	4	83542146	GTGATGC	4	83761170	R
Red	GGATCGC	37	Y	20508275	AGCCCTT	Y	18967663	F
	TGTCTTT	37	8	39770525	TACTCAC	8	39889682	R
	GGTTGGA	37	2	235766844	TCACAGA	2	235431583	F
Red	CGCCGCC	37	X	20009123	GGTCCCG	X	19919044	F
	GGAATTC	37	2	125183768	CGCATCC	2	124900238	F
	ATCATTA	37	13	33455187	TGGCTGA	13	32353187	R
	AGTGAAT	37	3	271185	CGCATTC	3	246185	R
Grn	GGCGCGT	37	X	30327615	CGCCCCC	X	30237536	R

Red	TTGCTGG	37	10	1772472	CGGATCT	10	1762472	R
	CTTTCTG	37	14	105620358	ACTTGGG	14	104691403	R
	CCTGGAC	37	4	187884368	CCTCAGC	4	188121362	F
Red	CCCACCT	37	X	70474613	GGTCCCG	X	70391338	R
	AGTCATC	37	11	102576374	CGGAAA	11	102081584	R
	ACAGGC	37	7	134354803	CGCCAG	7	134005343	R
	CCTCATC	37	6	29815787	ACTGGA	6	29923766	R
	CCACTTC	37	12	27996771	CGGCAA	12	27888038	F
Red	AGTGCA	37	1	32535981	CGAGGA	1	32308568	R
Red	CCAGCC	37	12	31272114	CGCGGA	12	31163381	R
	TCCCTGA	37	2	232507774	CCAGTCA	2	232216018	R
	GCACAG	37	3	127441767	CGTCTTA	3	128924457	R
	GGGGAG	37	2	120063914	CGCCTCG	2	119780384	R
Red	GTGGCCT	37	20	60745245	CCACCGT	20	60178640	F
	CGAAGA	37	7	95026181	ACTCATC	7	94864117	R
	CAAACA	37	8	54628500	CGCCTCA	8	54791053	R
Red	CAACGG	37	11	128321749	ACGCGA	11	127826959	R
	GGCTGGA	37	3	140987789	AGGGGT	3	142470479	F
	TAGGCC	37	1	206848389	TAACCA	1	204915012	R
	CTGAAAA	37	Y	2655977	AATTTGA	Y	2715977	R
Red	TCTTGGC	37	16	28756008	CGAGCA	16	28663509	F
	AAAAGC	37	8	57802622	AGCTCAG	8	57965176	F
Red	CGCTTCC	37	X	40440170	TACGTCC	X	40325114	R
	GGGCCG	37	5	33937673	GCAGTTC	5	33973430	R
	GCACCGT	37	8	125617225	CCATAGT	8	125686406	F
	AGTGGC	37	22	46481248	CGCCCAG	22	44859912	F
	CTTTGCT	37	11	27303972	CGGAAA	11	27260548	F
	CTGGACT	37	19	12377156	CGCGAC	19	12238156	F
	GGTGTCC	37	19	45033565	GCACCTG	19	49725405	F
	GAGAAG	37	9	45733081	CCAAAC	9	45623077	F
	ATGCCTC	37	6	32558175	CGGAGA	6	32666153	F
	GTATGAG	37	1	56046489	TTTCCCT	1	55819077	R
	TCCAAGA	37	7	148517345	GTGAGA	7	148148278	R
	GGGTTCC	37	6	31239320	CGGCCCG	6	31347299	R
	TTGCCAT	37	15	22921426	ATCTGTG	15	20472867	R
	TAGCTAC	37	6	164614646	GAGTTTA	6	164534636	R
	AATGGTG	37	11	110890228	CGGCTGG	11	110395438	F
	CAGAGG	37	8	68418480	CTGTGGA	8	68581034	F
	ACTGTGA	37	6	56200769	GTCTGTT	6	56308728	F
Red	GCCGGC	37	6	4079350	GTGGGA	6	4024349	R
	GGATTCC	37	16	28889110	CGCTCTA	16	28796611	R
Red	ATGGGA	37	1	115397857	CGCCCTA	1	115199380	R
	TTATCTT	37	12	131320160	ATAGCTA	12	129886113	R

	AGAAAA	37	21	27010696	ATAAGCT	21	25932567	R
	AACAGCT	37	X	154293850	CGAGAG	X	153947044	F
	GGATTAC	37	7	92459517	AATGACT	7	92297453	F
	GTTCCAT	37	6	167524238	CGGCAGT	6	167444228	R
	CAGCTCT	37	13	112241077	GAGGGA	13	111039078	R
	TCTTTTT	37	7	124307584	CGATGA	7	124094820	F
Red	AAACTCG	37	11	133658113	CGAAAC	11	133163323	R
	AAAGAC	37	7	132617821	ACTCAGA	7	132268361	F
	TGGCTGA	37	1	161168957	AAGCCA	1	159435581	R
	TGAGCCA	37	11	61728829	CCTGGCT	11	61485405	R
Red	GCAGGA	37	X	149861761	CGCCCAG	X	149612419	R
	TCCCTCT	37	1	152299041	CCTGTAT	1	150565665	R
Red	GGTGCG	37	14	103593520	CGCCCAG	14	102663273	R
Red	ACGTGG	37	1	207997020	CGGGACT	1	206063643	F
	TGGGCTG	37	14	104782902	CGCTTCC	14	103853947	R
	CTCCGGC	37	10	102107251	TGGGAA	10	102097241	F
	CCGCTTG	37	7	65971211	CTTCACC	7	65608646	R
	CTGTCAG	37	5	133839271	CGCAGA	5	133867170	R
	TTTAGAA	37	6	3293720	TTGAAGA	6	3238719	F
	GCCTGGT	37	1	1190541	TGCCTGT	1	1180404	R
Grn	GGGCAG	37	11	1858572	CGGCACC	11	1815148	F
Red	GGGCCC	37	1	6526309	CAGGTCT	1	6448896	F
	TTAGACA	37	14	104315223	TACACTC	14	103384976	F
Grn	GGATGA	37	14	101535442	GAAGGG	14	100605195	F
	TCCCTCC	37	14	21175838	CGTCTTT	14	20245678	F
	AGTCATC	37	2	235160451	CGCTTGA	2	234825190	R
	AGCACTT	37	5	140619567	GAATAA	5	140599751	R
Red	GTCTACA	37	2	20871195	CGGCCCT	2	20734676	R
	GCCATGC	37	12	115355126	CGCCCAC	12	113839509	R
	TTCTTGG	37	1	7023625	ATGCCTC	1	6946212	R
	ATTAAGC	37	1	3286322	CGGGAA	1	3276182	F
	GGATGA	37	2	121669723	ACAGGC	2	121386193	R
	TTTTAAT	37	2	122187299	CGTGGG	2	121903769	R
	GAAGTGA	37	13	45288461	CTTCTGG	13	44186461	R
Red	TCCCTGG	37	6	30039442	CCAGTTG	6	30147421	R
	GGGTTCG	37	16	67918754	TGGCTGA	16	66476255	F
	GCCACCC	37	1	233249267	CTGCATC	1	231315890	R
Grn	TGCCTGG	37	12	7781093	CGCCCAC	12	7672360	F
	TGAGTGG	37	3	14514657	TCTGGCT	3	14489661	R
	GATGCAC	37	13	36048892	TCACCAG	13	34946892	R
	CTGGTCA	37	X	106750120	GGGGCTC	X	106636776	R
	TTAATGA	37	5	140709986	TCCTCCT	5	140690170	F
	CACGGA	37	20	61290162	CCTGACT	20	60760607	F

Red	AACCGAC	37		19	56215426	CGGAGAC		19	60907238	F
	AGTGGGT	37		8	81963379	TCCATCC.		8	82125934	R
Grn	CCGCGGC	37		7	51539190	CGCCAAC		7	51506684	R
	TTGTCTC(	37		11	128321662	GACCAGC		11	127826872	R
Red	CAGTCAC	37		7	158280480	CGAAGCC		7	157973241	F
Grn	CCCTCGT'	37		4	2366555	CGCCCAC		4	2336353	R
	ACACTCT	37		7	48319696	TGGCTTC.		7	48290242	R
Grn	TTTCCTG(	37		15	39006617	TCTCCTT'		15	36793909	R
Red	TCGCTGC	37		13	50252564	AGGGGG(		13	49150565	R
	TGGGGCT	37		4	69435593	CGCTGCT		4	69118188	F
	ACCATAG	37		15	81122346	GGTCAGA		15	78909401	F
Red	CTGCCTC'	37		6	32552453	CGCAGCC		6	32660431	F
Red	GGGGGCC	37		12	2339439	GGCGCGT		12	2209700	R
	TTATGGG	37		10	111728983	AGCACAC		10	111718973	F
	GAGAGTC	37		2	47053771	TCCACTC.		2	46907275	R
Red	CTCGCGC	37	X		45710949	CGCCCGC	X		45595893	F
	CTTTTTC/	37		15	94943926	CCCTCCC'		15	92744930	R
Red	AAGCGGT	37		12	54384666	GTTCTTC'		12	52670933	R
	TCCACAC	37		13	43360920	TCCCACT'		13	42258920	F
	ATACCCA	37		3	119348585	GCTTTCC(		3	120831275	R
	GAAGAG/	37	X		101906537	CGCACCC	X		101793193	R
Red	ACTTTGG	37		6	160023927	CGCCCGC		6	159943917	F
Grn	CACTGCG	37		6	31937437	CGGCTAC		6	32045416	F
Grn	TTTAAAA	37		19	48077006	CGGCAGC		19	52768818	R
	ATGGAA/	37		3	134512480	GCCAGAC		3	135995170	R
Grn	CCGCCCT	37		19	50962411	CGGGGCC		19	55654223	R
	GCTGGAC	37		2	20871002	CGAGCCC		2	20734483	F
Red	GGACACC	37		17	35300645	GGATCCA		17	32374758	R
Red	TGTCTAA	37		6	31698089	CGCCCAT		6	31806068	F
	CCAGGG/	37		6	30496072	AACCACC		6	30604051	R
	ATTTCTC/	37		8	143666890	GGAGTCA		8	143663892	F
	TACATCC	37		8	18432000	TGGGTCC		8	18476280	R
	GTAAGTA	37		13	41227982	CAGTACT		13	40125982	R
	GTGAAC/	37		6	24936965	CGTGTGC		6	25044944	F
	GGCGGC/	37		17	76676243	CGCGCCT		17	74187838	R
	CATAGCC	37		1	95163986	CGGGGCT		1	94936574	F
	GGAAAAT	37		4	119989952	CGCCACT		4	120209400	R
	GGCGGTC	37		1	39981410	GGGGGCT		1	39753997	R
	TAATAAG	37		7	12735409	CGGGTTT		7	12701934	F
	TTCAGGA	37		13	112630569	AAATTTA		13	111678570	R
	TGGGCAT	37		2	3675155	CGGTCTG		2	3653030	F
	CTCTGTT'	37		7	135433639	CGCCCCA		7	135084179	R
	CGTTTCC.	37		22	22055412	CGCACAT		22	20385412	F

	GGCCAGC	37	7	150824021	GGATGTG	7	150454954	R
Red	CCCACCA	37	3	10149963	GCCCGGC	3	10124963	R
	ATGGAAA	37	13	112984976	GACTTCT	13	112032977	R
	GGCATGT	37	11	60680515	CGGAGCT	11	60437091	R
	AAGAGGA	37	7	158050299	CCCAGGC	7	157743060	F
	CTTGTGA	37	12	110888857	CGGTATG	12	109373240	F
	TAGAGAT	37	14	73927547	CGCCAAC	14	72997300	F
	CCGAATC	37	14	76819370	GAGAAAC	14	75889123	F
Red	ATCTTCG	37	19	55038290	TCGCGAG	19	59730102	F
	TTCAGAA	37	2	18063316	CGGAGTA	2	17926797	R
	GAGGACC	37	7	47990680	GTGTGCT	7	47957205	R
	TTTTTTC	37	17	45335202	CGTCCAA	17	42690201	R
Red	GGGAAGC	37	7	158109694	CGTCCTT	7	157802455	F
	TCATCTT	37	1	92101219	CGCCCCT	1	91873807	R
	CCCTGCT	37	1	2023794	CGGACTC	1	2013654	R
Red	GTGGGGC	37	15	96903559	CGGAAAA	15	94704563	R
	CACATAT	37	14	81405846	CGGCTTG	14	80475599	R
	CGCTTGC	37	7	87556005	CAACAGC	7	87393941	R
	TGTGTAT	37	6	26351915	AAGGAGC	6	26459894	F
	AAACACA	37	14	76445988	ATGTCTG	14	75515741	F
	GAATCAC	37	13	48011758	CGTAAAC	13	46909759	F
Red	TTGGCCC	37	12	54376019	GGCAGGC	12	52662286	R
	AGTTTCC	37	10	116444356	GACTCTG	10	116434346	R
Grn	GCCTCAA	37	7	102096976	CCTCCCA	7	101883981	R
	ATGCTCT	37	13	114829496	TGTTTTC	13	113847598	R
	AGGTGCA	37	2	177016837	CGGGCTG	2	176725083	R
	GTGGCTA	37	5	140603270	GTCAGCA	5	140583454	F
Red	GCTCCCT	37	4	190906077	CGCACAC	4	191143071	F
	CTGGACG	37	2	9437253	AAGGGCT	2	9354704	R
	GAGAAAC	37	1	234667461	CGCGGTG	1	232734084	R
Grn	CACGCCG	37	5	126409211	CGCCGGC	5	126437110	F
	ATATTCC	37	1	153014310	TCTTTGA	1	151280934	R
Red	CTGCAGT	37	19	289902	GAAACGC	19	240902	F
	AGCCAAT	37	4	168824531	TCATTTC	4	169061106	R
Red	TGTCAAT	37	2	198174542	CGCCGCA	2	197882787	R
	CTCTCCC	37	17	80970892	TAGCAAC	17	78564181	F
	CTTTCTT	37	15	40269214	TGTTGGG	15	38056506	F
	ATATTTA	37	7	141630634	TGTTGTT	7	141277103	F
	CGGCCCT	37	17	139511	GGCAACA	17	139511	F
Red	GGACGAC	37	17	18553663	CGCCCGG	17	18494388	F
Grn	GCCGGGT	37	12	131303194	CGCCGCC	12	129869147	R
Grn	GAGGTGC	37	1	32741454	AATGGAT	1	32514041	F
	GCGGGGC	37	X	9432900	TGGTAGG	X	9392900	R

	AGTGTCT	37	6	47665926	CGGGACA	6	47773885	R
	GTAGCCT	37	20	828445	TGTGACC	20	776445	F
Red	GCCTCCT	37	2	119599189	CGCCCCC	2	119315659	F
	TTCTCTT	37	2	235732349	CTTTGCC	2	235397088	R
	GAAAGCT	37	7	4242962	CGCAGAC	7	4209488	F
	CCTGGCA	37	10	135342218	CGGCGAT	10	135192208	F
	ACTCAGC	37	19	46526483	TCTCTGG	19	51218323	R
	AATGAGC	37	2	101398178	CGGCCCC	2	100764610	F
	GTGAGAA	37	6	41383225	CGGGCAA	6	41491203	F
	CTGTGCT	37	6	31937470	CGGCCAC	6	32045449	F
	AAAGAGC	37	5	96314976	CGGGAAC	5	96340732	R
	TGAACCA	37	2	26624604	CGGAGCT	2	26478108	R
	GAAGATA	37	2	242620306	ACTAAAT	2	242268979	R
	CTTCTTT	37	10	5446942	TGGATAA	10	5436942	F
	CACTCAG	37	8	101174067	CGCTTAC	8	101243243	F
	CTCGCCC	37	12	54377225	TTATCCA	12	52663492	F
	GCCAGGC	37	7	56242407	CGGGCCA	7	56209901	R
	CACAAAC	37	4	152147390	CGCCTGA	4	152366840	F
	AAAGTAA	37	3	106447444	CGTCTGT	3	107930134	R
Red	CGTGCGG	37	11	75139736	CGACCAC	11	74817384	F
	TTGCAGT	37	12	125271306	CGTGAGC	12	123837259	R
Grn	AGTGAGC	37	X	148713660	CGCCGCC	X	148521467	F
	GGGGGAC	37	10	134974555	GATGACT	10	134824545	R
	TCTCCTT	37	5	146253830	CGGGATC	5	146234023	R
Red	GGCGGAC	37	6	30039476	CAGGCGC	6	30147455	R
Red	CATGGCG	37	17	77478569	GTGGGGC	17	74990164	R
Red	GGCCACC	37	5	177434438	CGCCCTT	5	177367044	R
	AGCTGCA	37	5	36243704	CGCAAAA	5	36279461	R
	ACCTACC	37	10	50890136	TCACCTC	10	50560142	R
	CAAGACA	37	10	122262397	CGGGAGA	10	122252387	R
	TCTATTA	37	12	99118507	TATTCAT	12	97642638	R
Red	CGAATAA	37	12	115134886	CGGCTTA	12	113619269	F
Grn	TGCTTGC	37	6	21761486	AGCGCGA	6	21869465	R
	TACCCTG	37	1	1555396	TCGGGGA	1	1545259	F
	TGGGTTG	37	2	86423274	CGCCCTG	2	86276785	R
	ACTCATC	37	7	47539511	TGGCTCA	7	47506036	R
	CCACCCT	37	9	79792074	GATGAGC	9	78981894	R
Grn	TCCTGGG	37	6	30079102	CGCCCGG	6	30187081	F
	GACTCTG	37	22	21479855	CGTGCCA	22	19809855	F
	AGTAACC	37	1	6521490	CGGCGCC	1	6444077	R
	TGTGATA	37	15	52018762	CAGACCC	15	49806054	R
	CTGTATC	37	2	9319526	CGCCCTT	2	9236977	F
Red	GGGTACC	37	3	75704824	GAGGCCC	3	75787514	R

	CATTATT	37		10	99278449	CGACAAC		10	99268439	R
	TTCCTAA	37		6	32629920	CTGCTGG		6	32737898	F
	TGCCTTT	37		10	129144210	CGGCACA		10	129034200	F
	CCTACGC	37	Y		16637142	CGGGAA	Y		15146536	R
	TAAACAA	37		6	109450463	AAAAAA		6	109557156	R
Red	GTGGCGC	37		1	32741152	CGCTTCC		1	32513739	F
Red	CTGATAG	37	X		48858587	CGCGAGC	X		48743531	F
	TATGCTT	37		6	32522872	CGCCCTC		6	32630850	F
	AATGGTT	37		3	114102615	CGTCTAG		3	115585305	F
	ACTGCTA	37		6	75589206	CGCTTCA		6	75645926	R
	CATCCGG	37		7	55412682	CGCCCTA		7	55380176	F
	CCCAGAT	37		3	49237327	CGGAGAC		3	49212331	F
Red	GCTTCTC	37		5	140590533	CGCTCAC		5	140570717	F
Red	AGGTGCC	37	X		140271314	AGGCCTC	X		140098980	R
Red	AGGCCTT	37		11	33097335	CGGACTC		11	33053911	R
	TGTCTGG	37		7	171613	AGCTCTC		7	266696	R
Grn	CAGGCGC	37		12	131303247	CGCTGGG		12	129869200	R
Red	GAATCCT	37		20	62313737	CGGATCC		20	61784181	F
	ATGTATG	37		2	86106058	CACCTCT		2	85959569	F
	CTAAGGA	37		2	12609420	CGCCTGA		2	12526871	F
	AGCTCGT	37		22	48896579	CGGCAGC		22	47275243	R
Red	CCGTTGA	37	X		70150787	CAGCTCT	X		70067512	F
	ATTTCAG	37		15	27020039	TTCTCCT		15	24571132	R
	TAATAAT	37		17	79378207	CTCTGGC		17	76992802	F
	CAGGCAA	37		3	43465503	CGCTGGG		3	43440507	R
	GCACTGG	37		6	29759078	ATACAAC		6	29867057	F
	TTTCAAG	37		11	101576844	CCCTGCC		11	101082054	F
	CTTTTTG	37		10	50536966	CCAACAA		10	50206972	R
	GAGAGGC	37		3	64670017	CCACCCC		3	64645057	F
	CTAGCAT	37		12	2353479	CGGTGTT		12	2223740	F
Red	TCCCTGG	37		8	142852219	CGGCTTG		8	142850126	F
	TTTCATA	37		17	80450903	CGCCACT		17	78044192	R
	AGGTTAC	37		6	13924544	CGGGAGC		6	14032523	F
	CTCCGGC	37		16	88053094	CGAGATC		16	86610595	R
	GAGGAGT	37		2	128453445	CGCACTC		2	128169915	F
	CCCAGAA	37		17	46652399	AGAGGAT		17	44007398	F
	TATGTCC	37		10	79622515	CGGACAC		10	79292521	F
	CTAATCT	37		14	20691777	CGCAGCT		14	19761617	F
Red	AAAAAA	37		1	185373486	AAGATTC		1	183640109	R
	AAATGAC	37		8	40925730	CGGTATT		8	41044887	F
	ACGCGTG	37		2	241581770	CGGCCCC		2	241230443	F
	CATCTGG	37		6	153066907	CGGACAC		6	153108600	R
Red	GGGTCAC	37		6	79339334	GAGCGGC		6	79396053	F

	TTTGGAA	37	11	78130236	AATCCCC	11	77807884	R
	ATAGCAC	37	6	166856074	GGCACGT	6	166776064	R
	GTGGGCC	37	11	44037829	GGGACAC	11	43994405	R
Red	CTAAATC	37	10	7859676	GGCCAAA	10	7899682	F
Grn	AACGGAC	37	13	19919156	CGCGTGA	13	18817156	F
	CCTCACT	37	4	183063637	CGCCTCC	4	183300631	R
	CCTCTGT	37	10	134334283	CTTTTCC	10	134184273	R
	GCAGCTC	37	2	174024669	CGGCTGC	2	173732915	F
	TGGCCAT	37	10	30643845	ATTTGGG	10	30683851	F
Red	ATATATA	37	8	38032157	CGTGTAA	8	38151314	R
	GCAAAAT	37	5	80595275	AAATAAT	5	80631031	R
	AGAGTTG	37	12	46068618	CGCTGAA	12	44354885	F
	GGAGAAA	37	7	158280771	CGGGGAC	7	157973532	F
	TTCTTGA	37	2	218114431	GAGGCCT	2	217822676	F
	GAGGAAC	37	8	27779092	ATCAGAA	8	27835011	R
	GTAATTA	37	7	131422820	GGACACA	7	131073360	F
	CAGACCA	37	17	40561514	GGATCTG	17	37815040	R
	GGGGGGT	37	15	68835517	GGCTTTG	15	66622571	R
	GAAGGCA	37	5	27038802	ACTCTCT	5	27074559	R
Red	ACAGAGT	37	5	1491579	CGCCCCC	5	1544579	R
Grn	TCAGCGC	37	1	247681781	CGCCGCA	1	245748404	F
Grn	CCACGGC	37	5	1857477	GCGACCC	5	1910477	R
	TTCAACA	37	11	67142030	GGCTTCT	11	66898606	R
	CGGGAAA	37	2	153419601	CGAGGGA	2	153127847	F
	AACATTA	37	5	101925501	TTAGAGC	5	101953400	F
Grn	TGGGACC	37	6	169825345	CGCCTCC	6	169567270	R
	AGCTTTG	37	4	83718165	CGGATGC	4	83937189	R
	AGTGAGC	37	6	1056970	CGGCCCC	6	1001970	F
Grn	CGTCTGT	37	8	39172111	CGCGAGC	8	39291268	F
	TTCAAAA	37	6	28120928	AATATCT	6	28228907	R
	CAGGACA	37	6	29690766	ATTGTCT	6	29798745	R
	AGCTTCC	37	14	71022665	CGTCCTT	14	70092418	R
	ACGTAAA	37	7	77788546	CGGGCAA	7	77626482	F
	AAATAGT	37	1	210419938	ATTTCCT	1	208486561	R
Red	CTTCTTG	37	1	161280287	CGCACAC	1	159546911	R
	ACCCAAA	37	1	230493133	CGGCTGC	1	228559756	F
	CCCCACC	37	3	46661640	CGCCAGT	3	46636644	F
	GAGTTGG	37	17	43319137	GGAGGTT	17	40674920	R
	GTGACGC	37	Y	22737663	AGTGCGC	Y	21147051	R
	TTCAACA	37	3	57015101	GCTGGCC	3	56990141	R
	TGCTCAG	37	2	238065451	GGGACCT	2	237730190	F
	ACCCACC	37	2	110907413	CGCCTGA	2	110264702	R
	TGCAAAT	37	5	140724362	CCCACCA	5	140704546	R

Red	GGGAAG	37	X	34674799	GGATGCC	X	34584720	R
Red	GACTAAT	37	12	149495	CGGCTGC	12	19756	R
Red	AGCTGCA	37	11	8679885	CGACATT	11	8636461	F
Red	AAACTCT	37	5	180085446	CGGCGC	5	180018052	R
	TTTTGAG	37	1	113229103	CGGGGA	1	113030626	R
	TACAATT	37	17	80969749	CGAGCTG	17	78563038	R
	ATCTCGC	37	5	180497114	AGCGAG	5	180429720	R
	GGCTGCC	37	7	158815080	CGGAGC	7	158507841	F
Grn	CCGGCTC	37	5	178266137	CGCCCAC	5	178198743	F
	AATGGCT	37	X	77151316	TTGCTAG	X	77037972	R
Red	AGGTGG	37	4	157119935	ACACAA	4	157339385	R
	TTTTTGC	37	13	94957721	CGGGCTG	13	93755722	R
	TCCTCTT	37	1	40236125	GGGGCTT	1	40008712	F
	CTTATGT	37	13	46202047	TCTGTTT	13	45100048	F
	GGCAGC	37	8	142161887	CTTGCTG	8	142231069	F
	ATTGAGG	37	19	41782183	CGGCCCC	19	46474023	R
	CCGGGTC	37	8	72757456	CGCATGG	8	72920010	R
	GCTGGTC	37	9	69147680	AGGGCG	9	68437500	R
Red	TCTCGGA	37	X	114468360	TGGAACC	X	114374616	F
	GCTCACC	37	6	31689459	AAAATCT	6	31797438	F
	GGAGCCC	37	X	129114238	GAGGTGC	X	128941919	F
	AAATAAC	37	17	35161825	CGGGAC	17	32235938	R
	TTTGCCA	37	X	47077877	CGCCAGC	X	46962821	F
	AGAGGA	37	6	32362407	CGAGGTC	6	32470385	R
Red	AGATATG	37	7	52155829	CGGATGT	7	52123323	R
Red	TACTTCT	37	17	15686309	CGGCTCC	17	15627034	R
	CTCCCAA	37	21	27010160	GGAACA	21	25932031	R
Red	CCCTCCC	37	6	168502341	GACCCCT	6	168245190	F
Red	TGACTAA	37	1	196746875	GTAATTT	1	195013498	R
Grn	CCAGCAT	37	7	56605830	CGGCCGC	7	56573324	F
Grn	TACCAAG	37	2	220361467	CGTTTAG	2	220069711	R
Red	ACAACAC	37	7	96651983	CGGGAG	7	96489919	R
Grn	GGCCGCG	37	1	19600730	TGCTCGG	1	19473317	F
	TACATCC	37	19	51818377	TATTCAT	19	56510189	R
	TACCATT	37	5	43038611	CGCTGGA	5	43074368	F
Red	TTAGCTG	37	6	20032841	GCGATTG	6	20140820	F
Red	CCATTGG	37	6	29648590	CTGGGG	6	29756569	F
	CCTTGGC	37	8	43136445	CGGCTGG	8	43255602	F
	GATCCCT	37	13	64009677	CGCAGTG	13	62907678	R
	CCTCCCT	37	5	53751919	CGGAACC	5	53787676	F
	TCCAAAC	37	17	14139857	TTGAGGC	17	14080582	F
Red	CTTCTCG	37	15	74345103	CGCCTGA	15	72132156	F
	ATAATGA	37	6	170455498	CGGCTCG	6	170297423	R

Grn	GTGTGGA	37	21	47404268	CGCGTGA	21	46228696	R
	CCACTGG	37	15	82425284	CGTGTTG	15	80212339	R
	ACCTTCC	37	15	22923741	CGGGGA	15	20475182	R
	TCCCTGA	37	11	103721961	TGGAGCC	11	103227171	F
	GCCCTGG	37	1	24122853	CGGGCC	1	23995440	R
	ACTTGAG	37	15	67323243	CGCACCC	15	65110297	R
	CTTCTCT	37	8	92570895	CGATGCC	8	92640071	R
	GACCGTT	37	5	1110019	CTGTGTG	5	1163019	F
	CTTTTTC	37	1	230220668	CGTCCAG	1	228287291	F
	CCGGCGA	37	5	2643177	CCAGGG	5	2696177	R
Grn	TACAGGA	37	19	1851995	GGAGCC	19	1802995	R
Red	ATGGCG	37	7	95026073	CGCCTCA	7	94864009	R
Red	AGCTGTA	37	6	33036958	CATTGAC	6	33144936	F
	CTTTATG	37	13	110970196	CGCTATG	13	109768197	F
Red	GTGTTCG	37	10	135343248	GAGACA	10	135193238	F
Red	CCGTACG	37	X	53449647	CGGCAA	X	53466372	F
	GCCGCG	37	9	139741803	CGGGCC	9	138861624	F
	CCAGATG	37	13	27174884	CGTCTTT	13	26072884	R
Grn	TCCCAGG	37	5	138714337	CGGGGC	5	138742236	R
	TCAACAC	37	15	32607260	CCTCATC	15	30394552	F
	GGAGCTA	37	4	154707153	GACAATT	4	154926603	F
Red	CACCCAC	37	10	670815	AACTACG	10	660815	R
	CCTGTCT	37	12	52888480	AGGTGCT	12	51174747	R
Red	ACAGCA	37	20	62053198	CGGCCCG	20	61523642	F
Red	TGGGACC	37	9	72027409	GATCCCG	9	71217229	R
Grn	CCGCTCT	37	1	7023784	CGCGCCC	1	6946371	R
	GGTGTGT	37	7	1981254	CCTCACA	7	1947780	F
Red	CAGCGC	37	21	36041683	CCCTTAA	21	34963553	R
	CATGAA	37	5	142098838	GTAATAA	5	142079022	F
	AGTAGTT	37	5	180028658	GGGAGG	5	179961264	F
	AGTGTCT	37	22	46934981	CGTGTTC	22	45313645	R
	TGGGTGG	37	1	153234342	CTTCGCC	1	151500966	F
	TACTGGG	37	7	127291444	CGCTCTC	7	127078680	R
	GGCAAA	37	10	94250865	GGACCC	10	94240845	R
	GATCTGT	37	5	80501337	CGGGCA	5	80537093	F
Red	TTCTCAT	37	2	133014805	CGCTTCC	2	132731275	F
	ACAAGC	37	2	99081350	GCAATAA	2	98447782	F
	CTCCTAC	37	5	140580769	GTAGCGA	5	140560953	F
	GCACCCA	37	8	144406532	ACTCCTG	8	144477907	R
	CCATGTG	37	2	241644188	CGTCAGT	2	241292861	F
	ACAAATC	37	12	72233249	CGCAGG	12	70519516	R
	TCAACCC	37	22	30947032	CGGTCAG	22	29277032	R
	ACCTTGT	37	12	122851438	CGAGTAC	12	121417391	R

	TGAGCGA	37	6	5087031	TATTCTGA	6	5032030	F
	AGGAAGA	37	10	99356163	ATGGTGG	10	99346153	R
	AAGGGGC	37	5	176099987	GTCCAGC	5	176032593	R
	AACTGAC	37	8	39172020	TATCAAT	8	39291177	R
	TCAATGA	37	1	201857591	TGTGTTC	1	200124214	R
Red	TGTGGAG	37	4	146656091	CGGCTCT	4	146875541	R
	ATCAAGT	37	3	4794020	GAGGGAC	3	4769020	R
	TGGCACG	37	5	177433673	CGCTACC	5	177366279	F
Red	CTTCCGTC	37	5	135415948	TTGGGAC	5	135443847	R
	TTGAATG	37	6	114661277	TGCCCAG	6	114767970	F
	CTTGTAA	37	6	127780352	CGACCAC	6	127822045	F
Grn	GGGCACT	37	17	48263189	CGCCGAC	17	45618188	R
	GACTGAC	37	X	152000347	CGGGGAT	X	151751003	R
	GCCGCAC	37	17	79011140	TGAAGGC	17	76625735	R
	CAGGGGC	37	13	25320388	CCTCTCTA	13	24218388	F
	CAGGCCT	37	7	139351108	CGCCACG	7	139001648	F
Red	GTGAAGT	37	17	7960449	CTGAGCG	17	7901174	R
Red	CCCTGGC	37	11	66193993	CGCTACG	11	65950569	F
	ACTGACT	37	4	159732036	CGCTTCA	4	159951486	R
	CTTTTCAA	37	3	171543413	CGGCTCA	3	173026107	R
	TACGGAC	37	3	52864694	CGACAGC	3	52839734	F
	TCCTGGG	37	6	110732115	GTTCCCA	6	110838808	R
	AAGAGCC	37	8	1327546	GGTATTT	8	1314953	F
	CACTTCC	37	12	12038352	TGAGTCA	12	11929619	R
	CATGTGG	37	11	64116250	GGTGACC	11	63872826	F
	GCTGTGC	37	20	61924423	GGTCCTTC	20	61394868	R
	TGATATTC	37	13	97928231	ATGGTAG	13	96726232	F
Red	AAGCCGA	37	X	100663213	CGCTTAC	X	100549869	F
	GTTTCCCA	37	4	119759900	GCTTTCTT	4	119979348	F
	TGGCCCC	37	22	23922551	AGCAGGC	22	22252551	F
Red	TCCTCGCC	37	10	104835919	CGGCGTC	10	104825909	R
	CAAGAGC	37	1	68025026	CGGAGGA	1	67797614	R
Red	GGGTGGC	37	4	176349171	GTTCCGG	4	176586165	R
	AGGAGGC	37	10	6821048	TGCTGGG	10	6861054	F
	ATCCAGC	37	10	72191087	TCTGGGC	10	71861093	R
Red	GACAGCC	37	12	133035885	CGGCTTTC	12	131545958	F
	TCTGAAA	37	5	113699683	CGCCAGC	5	113727582	F
Red	TACCTTTT	37	X	129520156	CGGGGGA	X	129347837	R
Red	AAGACAC	37	X	20134346	CCAGGGC	X	20044267	F
	AAAGCCC	37	13	112820997	GGCTGCA	13	111868998	R
	AAACTCC	37	7	27703436	ACAGAAC	7	27669961	F
	CCTGCGA	37	4	736485	TGGCCAG	4	726485	R
	AGTCATA	37	7	29037910	CGGACCA	7	29004435	R

	TTGGCTT	37	1	81878937	CGGTAGC	1	81651525	R
	CTTCCTG	37	1	2885168	GAACATA	1	2875028	R
Red	AACGGAC	37	10	134420208	CGCGCCC	10	134270198	R
	AAATCCC	37	17	10634140	CGGCTCA	17	10574865	F
	CCGTTGA	37	6	32526669	TGAAAAT	6	32634647	F
	CAAGTTC	37	6	7998697	TAGGCCT	6	7943696	R
	AGGCCAC	37	X	68050216	AATGGGA	X	67966941	F
	ATATAGG	37	2	84714510	CGAGGCT	2	84568021	R
Red	TGAAACT	37	7	1622775	CCCACGG	7	1589301	F
	CTCATTC	37	10	79605162	TTTCGTG	10	79275168	F
Red	AGAATGC	37	5	140201656	CGCCCTC	5	140181840	R
	TGACTTG	37	6	116248269	TGAAGCC	6	116354962	R
	CTGATTT	37	6	32610971	CGGGTGC	6	32718949	R
	CGCGTCT	37	5	178208610	TGCTCAC	5	178141216	R
	TACCATT	37	5	36273196	CGGAAAA	5	36308953	R
	TAGAGGT	37	1	31988382	CGAGAGA	1	31760969	R
	ATAAACA	37	21	40145361	GTATGTA	21	39067231	R
Red	ACCCAAC	37	1	228659093	CGCCCAG	1	226725716	F
	AATAACC	37	17	12922839	CGCCCTC	17	12863564	R
	ACTGTTG	37	5	140098496	ACTCACT	5	140078680	R
	GAGGGGC	37	6	29910755	CGGTCAG	6	30018734	R
	TCAGGAT	37	10	134800741	CGCTGGT	10	134650731	F
	CAGTGCT	37	5	81553088	CATTACT	5	81588844	R
	AAATCAC	37	17	80970825	CGGACGT	17	78564114	F
Grn	TCGCTGA	37	X	48901162	TGAGGGC	X	48788106	F
Red	CTGGAGC	37	8	43131431	GTGGACA	8	43250588	R
	TTATACA	37	4	2402019	GGTCTGG	4	2371817	R
	GAGTGTC	37	7	158279846	CGTGGTC	7	157972607	R
	TGACAGC	37	10	129090134	CGGTAGC	10	128980124	F
Red	CGTAGGT	37	3	49940919	CGTGAAC	3	49915923	R
Grn	CACCCAT	37	12	115134956	TCGATCA	12	113619339	F
	CTATCTC	37	3	25646267	CGGTAGC	3	25621271	R
Red	AAGCCAC	37	7	150119075	GAAGCTT	7	149750008	R
	GAATCTG	37	14	100441223	CTGGCCA	14	99510976	R
	CTTCTCA	37	1	240776316	CGTCTTT	1	238842939	R
Grn	GCTCAAG	37	10	47083392	CGCTCCA	10	46503398	R
Grn	CCCACCC	37	5	176045983	CGGGCCA	5	175978589	F
	CCCTCCA	37	17	54230878	CGCCTCC	17	51585877	R
	AGAAAGA	37	X	117957616	AAATTCC	X	117841644	R
Red	ACTCTTC	37	14	101908830	CGTTCCT	14	100978583	R
	TCCAGAT	37	10	19918310	CGGAGGA	10	19958316	R
	TACAAAA	37	7	110496797	TATTTCT	7	110284033	F
	TCCACAT	37	6	154408701	CGCATGG	6	154450394	R

	CTTCAAC	37	3	101658926	CTTACTT	3	103141616	R
	GTGGGCG	37	8	134081000	TGAAGAC	8	134150182	R
	CTTGGCT	37	6	167631788	AGCTGGT	6	167551778	R
Red	CACAGAT	37	3	138152902	CGTCATT	3	139635592	R
	CCTACTT	37	7	96652153	CGCTAAC	7	96490089	F
	CAGCTCT	37	5	137785499	CGGGCAC	5	137813398	F
	CAGAGTA	37	20	23550632	CGCAATG	20	23498632	R
	CCTACCC	37	Y	2655866	TACCCTG	Y	2715866	R
	TCTGTCT	37	8	2263331	CGAGTCA	8	2250738	R
Red	GGGCATC	37	12	54384763	CGCTGCC	12	52671030	R
	AAAGGAC	37	3	70911187	CGGGGTT	3	70993877	R
	GGAAATA	37	8	122685859	CGGTTTT	8	122755040	F
Red	AGAGCTA	37	11	58598338	CGCCCTG	11	58354914	F
	GCCAGCA	37	13	68745297	TGTGCTC	13	67643298	R
	CCAGCTT	37	19	50479865	GGACACT	19	55171677	R
	GCGCTCC	37	4	176987009	GCGACCT	4	177224003	R
Red	GGGCTCT	37	4	155254326	CGGACTC	4	155473776	R
Grn	AGGGCTA	37	3	133502564	CTCCGGG	3	134985254	F
Red	GCTGTGA	37	6	79339195	ACCTGGG	6	79395914	F
	CTCCTTG	37	19	43383986	CCTTCTG	19	48075826	R
	GCAATAA	37	3	167659189	GGGTGGA	3	169141883	F
Red	TGACGGA	37	2	233216220	CGCAGCT	2	232924464	R
	TCTCAGG	37	5	53920730	GATTTGT	5	53956487	F
	TAAACAA	37	2	135970350	CGTTGCA	2	135686820	F
	ACTAGAC	37	1	23521307	AAGAAGT	1	23393894	R
	CTGTGAG	37	4	39531927	CGGGGAC	4	39208322	R
	CCGGCTA	37	12	2017093	GTTGTAC	12	1887354	R
	AGCAACC	37	2	209145835	GCTCTTT	2	208854080	R
	TTATTTT	37	7	16457583	CGCATGC	7	16424108	R
	TAACTGC	37	1	159439981	CGCAGGC	1	157706605	R
	TGCAGAA	37	19	40031368	CGGATGT	19	44723208	R
	CTCAGAA	37	4	122721721	ACTACTA	4	122941171	F
	TCTAACA	37	1	98518557	GATGGTC	1	98291145	F
	ACAGGAC	37	4	54765826	CGCAGAT	4	54460583	F
	AGCAGCT	37	6	32634316	GCACTTA	6	32742294	R
	TATAAAA	37	18	29205358	CGGGAAC	18	27459356	R
Grn	GTGAGAC	37	1	1663860	CGGCCAC	1	1653720	F
Red	GGCCTGG	37	6	32557404	AGCTCCC	6	32665382	F
	CATTTCT	37	10	121609179	CGTGATG	10	121599169	F
	AATTCCT	37	2	204674074	TCTCCTT	2	204382319	R
	GCAGCCC	37	13	112628711	CGGCGCC	13	111676712	R
	GGTTTGG	37	4	1366463	CGGGAGC	4	1356463	R
Red	GCGTTCG	37	10	729479	GGGCATT	10	719479	F

Grn	GGCTGAC	37	6	90597394	GCTGAAA	6	90654115	F
	AGTGTGT	37	14	55263199	CGGAGCT	14	54332949	F
	AGGAGGA	37	15	63758958	CGGGGGC	15	61546011	R
	AAGATCT	37	14	51187290	CGGCATA	14	50257040	R
	TGTTTGC	37	1	114717638	CGAGAAC	1	114519161	R
Red	TAGTCGT	37	X	152736032	CGCGCCC	X	152389226	F
Grn	ATTCTCT	37	7	130130189	CGGCCCT	7	129917425	F
	TACTTCA	37	2	172377981	GGACCGC	2	172086227	F
	GACCCCG	37	10	133036449	GTTCCAG	10	132926439	R
	TGCAGGT	37	6	31275791	CGAGGAA	6	31383770	F
Red	GCTGTGA	37	6	30419543	CGGTCCT	6	30527522	F
	GTCAAAC	37	2	147789639	CGAGCAA	2	147506109	F
	AACCGAC	37	13	47470350	CGCTCGG	13	46368351	F
	AGCGGCT	37	6	164092588	CGTCATG	6	164012578	F
	GAGCCCC	37	17	7381683	CGCTCCT	17	7322407	F
	AATTGTT	37	12	54068942	ACAACTT	12	52355209	R
	GTTGCTG	37	8	75880384	CGGAGCA	8	76042939	R
	ATGGTCT	37	19	13320368	CGGGACC	19	13181368	R
Red	ACGCCGC	37	X	125715154	CGGACCC	X	125542835	F
Red	AAAATAA	37	8	144850435	CGGTGCG	8	144922423	F
	CAATAAA	37	19	617195	AAAGCTC	19	568195	F
	GTGCTCA	37	15	65214588	GCTGGGC	15	63001641	F
Red	CCTGGGA	37	13	25115869	GCACATG	13	24013869	R
Red	CCAGCTT	37	17	46621709	CGCTGTT	17	43976708	F
	TGGGCTG	37	19	55417361	ACAATTG	19	60109173	R
	GCAGTTC	37	10	92079600	CGGAGTA	10	92069580	F
	AAGACAC	37	10	6014983	AAGACTC	10	6054989	F
Red	CCCCCGC	37	X	152783792	CGCCCAC	X	152436986	F
	TCATTTT	37	17	14483597	CGTGTCC	17	14424322	F
Red	CTGGCCA	37	19	613818	GTGAGCA	19	564818	F
Grn	ACTAAAC	37	7	4133290	TGGAGAT	7	4099816	R
Red	TCCTCCG	37	11	77907332	CGGGTAT	11	77584980	R
Red	CACAACG	37	2	242056226	TGCTACG	2	241704899	R
	AGGTCAC	37	6	33036934	GCTCAAC	6	33144912	F
Grn	CGGCGGC	37	6	170338591	GGGGGTC	6	170180516	F
Red	ATGCTTC	37	6	168918116	AGGAACA	6	168660965	R
	TCGGGTG	37	3	11178652	CGCTTCT	3	11153652	R
	AGTATAT	37	3	16843387	CGCTGTG	3	16818391	F
	GAAAGGC	37	11	66317822	TCAGGAC	11	66074398	R
	TATGTGA	37	17	53339697	CGGCAGT	17	50694696	F
	TGTACCC	37	1	1666808	TGGGCGA	1	1656668	R
	CAGGTTG	37	11	33474484	CGCAGGA	11	33431060	R
	AGATACA	37	10	5936426	CCTGAAG	10	5976432	F

	AAGGGA(	37	11	77033664	GGACCTT	11	76711312	F
Red	CAGCCGC	37	15	76484473	CGACGCT	15	74271528	R
	GTTAATG	37	2	153504453	CTGCAGT	2	153212699	F
	ACTTAAG	37	2	100926650	AGGTCTG	2	100293082	F
Red	GGAGTGC	37	22	46451518	ATGACAC	22	44830182	F
	GTCCGCT	37	15	31355430	CGTCTTG	15	29142722	R
Grn	CAGGGCC	37	19	57630536	CGCCCAA	19	62322348	F
	CTCACTG	37	15	27597471	TTGGCAG	15	25271066	F
	CAAGATC	37	4	19740944	CGCCACT	4	19350042	F
	CACCAGC	37	10	4868398	CGGCTGA	10	4858398	R
Grn	GGGAAG(	37	19	49232187	ACTGCTG	19	53923999	F
	CATTTAC	37	18	56963742	TGGTTGT	18	55114722	F
	TCTTCCG	37	2	75136334	CGAGAAC	2	74989842	F
	CCCACAT	37	4	108972693	AATTGCT	4	109192142	F
	ATACAGC	37	4	174538350	CGGCATT	4	174774925	F
	TACTAGG	37	6	32728862	CGGCTCT	6	32836840	R
Red	CTGATGC	37	6	158080750	AAGACGA	6	158000738	F
	ATTTCTT	37	6	130031784	GGACACA	6	130073477	F
	GTTTCTG	37	1	205283250	CGGAACA	1	203549873	F
	CCTCTCC	37	11	103305609	CGAGACT	11	102810819	R
Red	TCTTGTC	37	6	25882481	CGGCCGC	6	25990460	F
Red	ACTCCAG	37	X	63615339	CTTTCGC	X	63532064	F
	GAAGCAC	37	6	31240814	GGCAGTC	6	31348793	F
	GAAGCAC	37	2	161887154	CGCTGCA	2	161595400	F
	GGGCATC	37	2	8530521	CGGAGGA	2	8447972	R
Grn	TGGCAGC	37	19	15568511	GAAGTGC	19	15429511	R
	GTGCCTT	37	7	50109467	GTCATAG	7	50080013	R
	TCAGGAC	37	11	95824265	CGTGAAC	11	95463913	R
	TTTGTTC	37	12	58758818	CGTGGGT	12	57045085	R
Red	TCGCTTC	37	6	134436620	CGAGCTG	6	134478313	R
	AGAAGGA	37	1	33608053	CGTTAGG	1	33380640	R
	CATGAGC	37	1	117233299	CGTGCAG	1	117034822	F
	GTGTACA	37	15	90755932	CGTCTGC	15	88556936	F
	CATAAAC	37	6	33037662	CGGACCA	6	33145640	R
Red	ACTCGCT	37	5	140537692	TCTACCT	5	140517876	R
Red	AGGCACC	37	5	1196154	CGGCCAT	5	1249154	F
	ACTTCTT	37	3	180546915	CCAAGGA	3	182029609	F
	AGGGCCC	37	5	136636409	CGGAACC	5	136664308	R
	GTCACCA	37	10	116394557	TAGTTGC	10	116384547	R
	CAGAGAA	37	1	146548032	CTTTCAG	1	145014656	R
Grn	GCCCAGC	37	15	101912471	CGCTCCC	15	99729994	R
	TCAGTTC	37	1	25698385	CGTGGTG	1	25570972	F
Red	TGCATTC	37	1	3659656	CGGCGGC	1	3649516	R

	ACCCATA	37	5	141900671	CGGCCCC	5	141880855	R
Red	GGGAGAC	37	X	48755596	CGCAACT	X	48640540	R
Red	AATTAGT	37	5	140090300	CGCCCCT	5	140070484	R
	GATGTTC	37	10	1083304	GCCCTAT	10	1073304	F
Red	ACAGAAC	37	6	2515337	GCACGCT	6	2460336	R
	CTTCACT	37	14	105830606	CGGGCTG	14	104901651	R
Grn	CTATTGT	37	1	168198488	TTTTGAT	1	166465112	R
	CAGGAAA	37	21	19191096	CCTGCAC	21	18112967	R
	TCCAGAG	37	17	39094308	CGGGCAC	17	36347834	R
	CAAACGC	37	7	158342394	CGCTCCA	7	158035155	F
	CTCGCCA	37	17	19627822	CGCCTGC	17	19568414	R
Red	GCAGTAT	37	1	17004764	TCAGCGT	1	16877351	R
Red	GGCGGGA	37	8	56685709	AGCCTGC	8	56848263	R
	CGCTCCC	37	5	98112437	CAGGTAC	5	98140337	F
	GGTGCTC	37	2	148394181	CGGCAAT	2	148110651	R
	ATTCAGG	37	2	30669597	CGAGACC	2	30523101	F
Red	CGCCGCC	37	1	158147629	CGCGCAC	1	156414253	F
Red	CGGGGCC	37	3	49236860	CGCCCCG	3	49211864	R
Red	CACACAC	37	X	153151235	CGCCCCC	X	152804429	F
	TTTGCTT	37	17	10602001	CGGAACA	17	10542726	F
Red	TTTTTTT	37	19	45724963	CGGTGTG	19	50416803	F
	AAAAAAA	37	1	23474871	AGAAGAC	1	23347458	R
Red	CGTGCTG	37	15	34782820	CGCCGCC	15	32570112	F
	TCTTAGC	37	12	43253257	TGATAAA	12	41539524	F
	ATAATCG	37	17	57743594	CTGAAGC	17	55098376	R
	TGGAGTG	37	10	133810178	CGGGCCT	10	133660168	F
	GTGGTGC	37	10	134411480	CGGGCTG	10	134261470	F
Red	AAATGCA	37	2	240898385	CGTGAGA	2	240547058	F
	TAAGACA	37	10	75913585	CGCTAAA	10	75583591	R
	CAGACAA	37	2	28022223	CGCTGAA	2	27875727	F
	ATAAAAC	37	5	16614241	CGGAGAC	5	16667241	R
Grn	CAGGCTC	37	X	153084761	CCTCTCT	X	152737955	F
	CTGGGGC	37	17	17875168	CGAGTCC	17	17815893	R
	GTTTAAC	37	6	166260964	CATGCTT	6	166180954	F
	ACAGCTG	37	17	2886453	CTCACAG	17	2833203	R
	GAACTAC	37	2	177016167	CGTGTAA	2	176724413	R
Grn	GAACTTT	37	7	155333337	TTTCGCA	7	155026098	R
	AAAAGAC	37	12	55640113	CGAGGGA	12	53926380	F
	ATCCTCC	37	11	116664039	GGCTCAT	11	116169249	R
Red	GCAGAAA	37	5	1219873	GGTCACA	5	1272873	R
	ATGGAAT	37	7	94034808	CGGTATT	7	93872744	R
	CTCCATG	37	2	241644240	AGTCAGC	2	241292913	R
	GGTGTCT	37	18	11151764	GGTTAGT	18	11141764	R

	TAAGGGA	37	2	72788795	CGTCTTT	2	72642303	R
	CCGTTTC	37	13	112985499	GCCTTGG	13	112033500	F
	GGAGGGA	37	7	1080836	CGGAGCC	7	1047362	R
	AGCCTCC	37	13	114965839	CGCCTGC	13	113983941	F
Grn	CCGCCCG	37	11	77299805	CGCCCGG	11	76977453	F
	TTTTTCA	37	1	192520335	CATCTGA	1	190786958	F
	AAGTGGT	37	17	78765948	AAGTTCT	17	76380543	F
	CGGTGAC	37	17	80255510	GCCCTTC	17	77848799	F
	TCTGTGC	37	13	113652368	CGGGGCC	13	112700369	R
	AGCCTTG	37	5	86687001	CGAGATA	5	86722757	R
	GTCTGGC	37	6	131520996	AATAACC	6	131562689	F
	CAGCAAA	37	6	30103459	GGGTGCC	6	30211438	F
	ATAGCCT	37	8	8667411	CTACTTC	8	8704821	R
	TGTGTTT	37	X	125954246	CGGAAGA	X	125781927	F
	TGGATAG	37	8	55180538	CGCCTGC	8	55343091	F
	AGCGCCC	37	1	4844027	CGGGCCA	1	4743887	R
Grn	CCACGGC	37	2	97778927	CGCTGGC	2	97142654	F
Red	GCGGGCC	37	6	30039435	CGCCGCC	6	30147414	R
	AGAACAA	37	12	121345228	CTTGGCT	12	119829611	F
	GCTCCTG	37	10	105408599	AGCTTCC	10	105398589	F
	GGAGCCC	37	4	1496981	CGGGACC	4	1466309	R
Red	ACCATTT	37	18	12255318	CTTGAAG	18	12245318	R
	ACCTTGC	37	5	75918112	CGCTCTT	5	75953868	R
	CTTGTAA	37	17	35302352	CGGCTTT	17	32376465	F
	GCGGTGC	37	9	139607421	GACTTCG	9	138727242	F
	CGTCGCT	37	13	111281384	CGGCAAC	13	110079385	R
Red	TCTGGGC	37	21	46851112	GGCAGGC	21	45675540	R
	CACTTCA	37	6	422935	GCCTGAA	6	367935	R
	GTCCCCA	37	12	2017510	AAGTGGA	12	1887771	F
	ATGAACA	37	12	113229989	CGCTAAG	12	111714372	R
Grn	CTTGGAT	37	17	19883602	AGACACA	17	19824194	F
	TTGATGT	37	4	74921425	ATAAATC	4	75140289	F
	GTGCTTC	37	4	157897045	TAAAAAA	4	158116495	R
Red	GAGTTGA	37	2	90016302	AGAAGGC	2	89653603	R
	TGGAAGA	37	17	32581466	CCTGTCC	17	29605579	F
Red	CTGTGAT	37	1	3504125	GTTTGCA	1	3493985	R
	CAAAGTC	37	8	1312875	CGCAAGC	8	1300282	R
	TCACTTG	37	8	131685278	CGGGAAC	8	131754460	R
	GACTATG	37	12	132853388	CGGTAGA	12	131363461	R
	GGTCAGA	37	3	127468611	AACACAC	3	128951301	R
	AGAAATT	37	3	145969718	CGGGAGA	3	147452408	F
Red	AGATGCC	37	6	30039010	CGGCCGC	6	30146989	R
	GTATCTA	37	7	138229989	CGCCAGC	7	137880529	R

Red	CAAGGCC	37	1	27676205	CGACCGC	1	27548792	F
	TACAAAA	37	6	33585071	CGCATTT	6	33693049	R
	GGTGCTC	37	22	18531077	CGGGTGT	22	16911077	F
Red	GGACTGC	37	1	19600719	CGCCGCC	1	19473306	F
	GTTTTGA	37	5	146864669	CGATTGC	5	146844862	F
	TATTTAC	37	1	115398123	TCCCAGG	1	115199646	F
Red	CAACCTA	37	2	108157524	CTCGCAC	2	107523956	R
	CATGGAC	37	14	22370643	CGGATGA	14	21440483	R
	TTCAAGG	37	10	77228852	AGCACCA	10	76898858	F
	TGAAAGC	37	8	100649114	CATTAAC	8	100718290	F
	AAACCCC	37	8	61626646	CAGACTC	8	61789200	R
	GCTACAG	37	5	72683866	CGAGGTA	5	72719622	R
Red	GCTGCGA	37	8	54935605	GACCTGA	8	55098158	R
	TTTGTTG	37	15	89640878	CGCATTC	15	87441882	F
	CCTAGAT	37	8	89281994	TCCAGCT	8	89351110	R
	AAAGGA	37	6	29981404	CGTGGCT	6	30089383	R
	ATCCCGT	37	11	46389249	GGAGTCA	11	46345825	R
	TTTTGTC	37	6	33033176	CGGGCTT	6	33141154	R
Red	GCCTTGC	37	17	75385278	CCTCGCA	17	72896873	R
	ATATTAA	37	6	33042551	CGAGGA	6	33150529	F
	ACATGGA	37	6	32549283	CGGTCCC	6	32657261	F
Red	CCCTAAT	37	12	128752246	GCGGGGC	12	127318199	F
Red	GGGCGTC	37	10	44185097	CGGCACC	10	43505103	R
	AATAGGA	37	5	80493	GGGGCAC	5	133493	R
	GCCTGAT	37	14	26862142	CGGCTAA	14	25931982	F
Grn	AGCAGCA	37	17	629613	CGGAGTC	17	576363	F
Red	CATTAGT	37	5	140097950	CGCCCCT	5	140078134	R
	GTAGGAA	37	6	109812795	CGCTCTC	6	109919488	F
Red	GTAGCGA	37	22	32599516	GCGGGTT	22	30929516	F
Red	AGAGAAC	37	6	4403020	CGGATGC	6	4348019	F
Red	TCGGCCA	37	6	109611667	AGAAGAC	6	109718360	R
Red	CGCCTGG	37	6	28558070	TTTCCCG	6	28666049	F
	GCTCATT	37	1	92002689	CGGTCTC	1	91775277	R
	GAAATGC	37	7	158820418	ATTTGCT	7	158513179	R
Grn	GGAGGCA	37	5	130344188	CGGGCCC	5	130372087	R
	AGGCAAC	37	5	71475303	CGGGCCA	5	71511059	R
	TAAAGTT	37	7	19759345	CACTTGA	7	19725870	R
Grn	TGGGGCC	37	5	179588551	CATCGCC	5	179521157	R
	TTGCTTG	37	13	50975679	AGCTTCA	13	49873680	F
	TGCCAGG	37	8	1915076	TTCATGG	8	1902483	F
	GTCTCCA	37	7	158084782	CGGGCTG	7	157777543	R
	TCACTGT	37	3	131756545	TGGTGTT	3	133239235	R
	AAGTTTC	37	5	17395952	CGTGGTA	5	17448952	R

	CACCTCA	37	7	69058543	ATCCTGG	7	68696479	R
	AGTTAAC	37	12	94376970	CGTGGTG	12	92901101	F
	TTAGATA	37	11	76998821	CTTATTA	11	76676469	F
Grn	ATAACTG	37	11	93063662	CGGCTCC	11	92703310	R
	GGACCTG	37	14	51411481	GGAGGCT	14	50481231	R
	GGTTCTT	37	6	32609094	GGCCAGA	6	32717072	F
	CCTAGCC	37	3	42725643	CTATTAT	3	42700647	F
	CCCACTC	37	12	126465510	TTCCTAT	12	125031463	R
	GATGCAA	37	5	140086190	CCCAGTC	5	140066374	F
	TCCTTCC	37	7	116909621	CGTAGCA	7	116696857	F
Red	CACTCAC	37	6	31324916	CAGGTCT	6	31432895	R
	GTCACAG	37	5	171831028	CGGCAGC	5	171763633	R
Red	TGCGCTT	37	5	147699892	ATGGGCT	5	147680085	R
Red	GCAGCGC	37	3	160170242	GAGGAAC	3	161652936	F
	GATGAGA	37	7	107574783	GAGGAAC	7	107362019	R
	TGGGAAA	37	12	109248326	AGGGCAC	12	107772455	F
	GTGACCA	37	11	67141961	CGGACCA	11	66898537	F
	GTAAAAA	37	6	28092048	GTGACTT	6	28200027	F
	TGCCTTA	37	11	57195382	ATCTAAT	11	56951958	F
	CCAGGAA	37	6	32633114	CGGGCTG	6	32741092	F
Red	GGCCTCC	37	3	10149882	CCCTATC	3	10124882	R
	CCAAGAC	37	1	206677719	TAACATT	1	204744342	F
	TGCAGAC	37	11	10339086	CCCTCTA	11	10295662	F
	GTGCTGG	37	1	85584420	GAAGTGA	1	85357008	R
Red	GGCGGGA	37	1	234350834	CTGCAGC	1	232417457	R
	GCAAGAC	37	3	43927675	CGTCTGC	3	43902679	R
	ACTTGAG	37	11	19078052	GCTGCAT	11	19034628	R
	AATCAAC	37	1	5526684	AGTGGGC	1	5449271	R
	AACCCAC	37	15	22947765	CGGTAGA	15	20499206	R
	CAGCGGT	37	1	24373210	CGGTCAG	1	24245797	R
	AGACAGA	37	6	32520916	CGACTCA	6	32628894	F
Red	GGTGATC	37	12	115137528	TCTGGTA	12	113621911	F
	GACTCTC	37	1	92203667	CGGGAAC	1	91976255	R
Red	TAGGGCC	37	6	101846797	AAACCTT	6	101953518	R
	CTAGCCC	37	1	16345368	TCTAGAA	1	16217955	R
	TTTATGG	37	14	70827109	CGCTTTT	14	69896862	F
	ACAGTGC	37	6	114053961	GCCTTTT	6	114160654	F
Red	GTCCCGT	37	1	182921688	GACGAAT	1	181188311	R
	GGGGAAT	37	12	124810173	TGAGGCC	12	123376126	F
	ATGAGGT	37	1	36689477	CAGGCTC	1	36462064	F
Red	GCAAATC	37	7	2768988	CGTGGTC	7	2735514	F
Red	GCCGCAC	37	2	233251770	CCTCGTT	2	232960014	R
	TGAGCGC	37	6	42695332	CGGCCCA	6	42803310	F

	AAGCCAA	37	14	65078521	CGGCATA	14	64148274	F
	ACCCAAT	37	11	122047028	CGTTGCT	11	121552238	F
Grn	TCACTGA	37	13	23755059	GGATGAA	13	22653059	F
	CCTATTT	37	15	77481069	GACCTGC	15	75268124	F
Grn	GCAGTGC	37	1	1362340	GGCCGCT	1	1352203	F
	CATTGCC	37	6	30043924	CGGCCCC	6	30151903	R
	GGGAATC	37	8	41258634	CGCTTGC	8	41377791	R
	CATTTGG	37	11	69245569	TTCCAGC	11	68954750	R
	AGGAGAC	37	6	153405859	TTGGCCA	6	153447552	F
	GCATTTT	37	17	39151857	CGCTATT	17	36405383	F
	CCTCTAT	37	2	238647857	CGCCAGC	2	238312596	R
Red	CGGCCCA	37	6	30039202	CGGCCGC	6	30147181	F
	GTGACCT	37	13	30061917	CGGCACA	13	28959917	F
	GCATATG	37	1	2705849	CATGTCC	1	2695709	R
Grn	TGGGCCG	37	4	4860190	CGGGGTC	4	4911091	R
	AGCCTTT	37	7	158541253	GAGCTAT	7	158234014	F
	GAGGGTA	37	17	48136824	GAAGCCC	17	45491823	R
	CAGCCTG	37	2	240020028	AGGGCCT	2	239684965	R
	ACTCATT	37	6	152640475	CGTTAGT	6	152682168	R
	AGACTGT	37	17	6495080	CGTTTCC	17	6435804	R
Red	CCTGACT	37	14	93106414	CGCAGTC	14	92176167	R
	CTGTCAC	37	1	27524410	TGTGTAA	1	27396997	F
	CCTCTGT	37	1	204556835	ATTGTGC	1	202823458	R
	TGTGATT	37	4	123090679	CGGTAGC	4	123310129	F
	CTTGCCG	37	13	36049673	GGAGTGC	13	34947673	R
	CTCTTTC	37	8	130713856	CGCCCCC	8	130783038	F
Red	AATGACA	37	14	101908752	GCTGCAC	14	100978505	F
	GTTTTAC	37	15	86062960	GAACCAC	15	83863964	F
	GCGTGGT	37	2	239999466	CGCAGCC	2	239664403	R
	GAGAGTC	37	22	36960499	TCTCCTT	22	35290445	R
	GTAGCTT	37	11	96076288	TTCCTTT	11	95715936	R
	CCCGCAG	37	4	39772940	GTGAGCA	4	39449335	R
Red	GTCCTAG	37	2	121412005	CCGCTGC	2	121128475	F
Grn	TGGTTGT	37	2	879510	CCCGTTC	2	869510	R
	GGTATCA	37	7	155492281	CTTTCAC	7	155185042	F
	TACCTGT	37	1	225838196	ACTGACA	1	223904819	R
	CAGGGTC	37	5	71713491	CGGGAAT	5	71749247	F
	TTTCAGC	37	6	140364611	CGGTGAT	6	140406304	F
	GGCGCCC	37	2	232395300	TTGTGAG	2	232103544	F
	ACGACTG	37	10	135341528	CGGCGTT	10	135191518	R
	ATGATGT	37	10	63388439	CCAGCAA	10	63058445	F
	CCCATTG	37	4	146018723	CGGCAGA	4	146238173	F
	ACAATAA	37	11	48509778	CGGGAAA	11	48466354	F

	GAATGGC	37	1	217313824	CGACTCC	1	215380447	F
Grn	AGAAAG	37	8	1899406	CGGTCAG	8	1886813	F
	ATTGAAC	37	6	55965977	CGACTGA	6	56073936	F
Red	CTCCGAC	37	12	125223502	CCCCGAC	12	123789455	R
Grn	AAATGGT	37	7	79084196	CGGAGCT	7	78922132	R
	ACCACAC	37	3	27575278	ACCACTC	3	27550282	F
	CCCGGGC	37	6	4661470	CGGTCCA	6	4606469	F
	TTTTTGC	37	10	7670827	CGCTACA	10	7710833	R
	AAACGTC	37	17	146670	GGATGCT	17	146670	F
	TATAGCT	37	6	30856386	CGGCCCA	6	30964365	F
	AGCCCAA	37	6	32634276	CGGGAG	6	32742254	F
Red	AGACGCC	37	2	54087189	GGCCGGC	2	53940693	F
Red	CTGCGTC	37	2	198650987	CGCGCCC	2	198359232	R
	CATTTCT	37	1	230241764	CGGTCTT	1	228308387	F
	CCTGGTG	37	4	47035074	ACAAATT	4	46729831	F
	GTGCTGG	37	Y	27210334	CGTCACC	Y	25619722	F
	CTGCAAG	37	12	10336861	CGGTAAT	12	10228128	F
	AACACTT	37	5	177505451	CGGCCCC	5	177438057	F
	TCCCAGA	37	X	53350895	CGGGAGC	X	53367620	F
	TAGCAGC	37	1	27899025	CGTCCGT	1	27771612	F
Grn	GCTCAGT	37	19	40375973	CGGGACC	19	45067813	F
	CCCCCCT	37	6	30419135	CGGGTCC	6	30527114	F
	ATATATT	37	12	76414534	CGTTGGC	12	74700801	R
	CAGGAGC	37	18	60646614	CATAGTG	18	58797594	F
Grn	AAGCCAC	37	13	25212556	GTGCAGC	13	24110556	F
	TAGCTCA	37	1	8480963	CGGAGTC	1	8403550	F
Red	GGCGCGC	37	6	30039432	CCTGGTG	6	30147411	R
	ATTTTTT	37	9	116133216	CACACAA	9	115173037	R
	TTAACAT	37	2	191365138	AAAGTCC	2	191073383	F
	TTTCATC	37	6	3592887	CGAAACA	6	3537886	R
	CCATGGC	37	13	111287117	GAAGGCT	13	110085118	R
	CAGCAAT	37	19	50550359	CTGTAGC	19	55242171	R
	CTTGGGG	37	3	11596003	CGGTCCC	3	11571003	F
	GTGTGGA	37	9	101869277	CGGTGTG	9	100909098	R
	CATAAAT	37	17	3814718	TTCCCAC	17	3761467	R
Red	GGGAGCA	37	1	991708	CGCCAGA	1	981571	R
	TACTAAG	37	9	37780478	CGGTACA	9	37770478	R
Grn	GCCACAC	37	6	887704	GGCGGAC	6	832704	R
Red	TCAGAAC	37	10	43846281	GTTGAGA	10	43166287	F
	CGTGGCA	37	9	2234921	TCCTCAC	9	2224921	R
	GGAAAAC	37	10	3823758	CGGGCCT	10	3813758	R
	TTTGGTG	37	9	99615182	CGAGGGA	9	98655003	F
Red	CTAGGCT	37	10	27702309	CGGCAGC	10	27742315	R

	GCAGCTC	37	1	62499140	CGGGAAT	1	62271728	R
	GGAGTCG	37	17	57116416	CATGGAC	17	54471198	R
	AGCCCCG	37	4	1046988	GTTCCTT	4	1036988	R
	GCTCCTA	37	2	20638399	GGTTCTG	2	20501880	R
	AATGGTT	37	9	131081198	ATTTTCA	9	130121019	R
	CACGGAT	37	9	16442831	GCCTATG	9	16432831	R
	AGCCAGT	37	1	5137221	TTGATGC	1	5037081	R
Red	AGGCTTC	37	17	21191452	CGGCTGG	17	21132045	R
	TATACAT	37	1	217004524	CGTGTCT	1	215071147	F
	TTGGTGC	37	9	92681430	TGTGGCT	9	91721250	R
Red	AGCTCGC	37	1	152161927	CGCTCCG	1	150428551	R
	CAAATAC	37	2	176997504	CGCTCAA	2	176705750	F
	TCTGCGG	37	13	29914200	GCCAAGT	13	28812200	R
	TATGCCA	37	22	32650997	CATAAAT	22	30980997	F
	TGGCTAG	37	19	55417390	CGCCTCT	19	60109202	R
Red	GGCGTGT	37	10	135273122	GGGGCAC	10	135123112	F
	CAAGGAA	37	11	3117181	ATTTGAT	11	3073757	F
	GCAGGCT	37	9	140330627	CGCCCCC	9	139450448	R
	ACCAAGA	37	7	158098282	CGCCTTG	7	157791043	R
	ATTAACT	37	17	76607622	ATCCATT	17	74119217	F
	CTGTCTG	37	10	135269787	AGGTGGA	10	135119777	R
	TGAGGTA	37	5	112825132	CGGGCAC	5	112853031	F
	CAATAAA	37	13	102108074	CGCAGTC	13	100906075	F
Red	CCTACAT	37	9	32426330	CCTCCCA	9	32416330	F
	AGCTTGT	37	6	112688607	CGGTAGC	6	112795300	R
	ATTCAGC	37	1	209997303	GTATTGT	1	208063926	R
	TTTTCCAC	37	2	243017952	CGGCACC	2	242666625	F
	GCTGAAC	37	14	62004353	TCTCAGA	14	61074106	R
	GGTTAGG	37	9	136822302	CGGGCCT	9	135812123	F
	TGAGTTC	37	1	42954129	CCACCTT	1	42726716	F
	ACAGTTC	37	15	45449437	CGCATAT	15	43236729	R
	ACATGGT	37	19	4033718	AGCAAGA	19	3984718	R
Red	GCCCCTC	37	7	72813978	CGGGGAA	7	72451914	F
	CAGCCCC	37	8	22925391	CGCAGCT	8	22981336	R
	CACAGAC	37	4	57824450	AGAGTTG	4	57519207	F
	TACAGGC	37	5	135415781	TATTGCC	5	135443680	F
	CAGGGTC	37	5	172324773	ATGTGAG	5	172257379	R
Red	GCCCCTC	37	9	132016282	CGCAGCC	9	131056103	F
Red	GGTGACT	37	2	875437	CGCCCCT	2	865437	F
	GTCTCCT	37	15	42186830	CGGGCCA	15	39974122	R
Red	AACAGAA	37	11	132582488	TGAACCT	11	132087698	F
Red	TGCTTGG	37	9	137662926	CGCAGCC	9	136802747	F
Red	ACCAATC	37	9	127534760	CGCATCC	9	126574581	F

	TCTCTTTC	37		17	63926873	AGTGCTT		17	61357335	R
Red	GGAGGAC	37		19	33572088	CGGGCCC		19	38263928	R
	CTCTTAA	37		17	68070495	CGGTGGC		17	65582090	R
	ATCAGTA	37		12	59281920	AAAAATA		12	57568187	R
	AGGATGA	37		20	37054900	AGCCCAA		20	36488314	R
Grn	TATAAGG	37		12	54380956	GCATGCG		12	52667223	F
	TAACAAA	37		6	32843055	ATTAATT		6	32951033	F
	AAAAAGA	37		8	72755052	GAATGCT		8	72917606	F
	TGAGACC	37	X		69509727	TCCGCTT	X		69426452	F
	TCTTTCC	37		11	13987766	CGGCAAT		11	13944342	F
	GAGAGGA	37		9	6932042	TCTCATA		9	6922042	F
	GCCTCCT	37		9	101264699	CTCTGAA		9	100304520	R
	GAAGACA	37		9	119475712	CGGGCCT		9	118515533	R
	GCATTTG	37		2	33359356	TATTAAG		2	33212860	F
	CATCTCA	37		10	129197384	CTTAATC		10	129087374	F
	TCCACCA	37		6	33035284	AAAAGCT		6	33143262	R
Grn	GGAGCAC	37		11	2176877	TGAGCTG		11	2133453	R
	GCCCGCC	37		19	5335254	CGGACGC		19	5286254	F
	TCCACCC	37		12	54387530	ACAAGGC		12	52673797	R
Grn	CCATTGC	37		9	135114066	CGCCGTG		9	134103887	F
	CTAAGGT	37		9	136152547	CGCCCAG		9	135142368	F
	TTATTTC	37		10	1511173	CGGGGCC		10	1501173	R
	AAACATT	37		14	81879247	CGGGCGA		14	80949000	F
	TGCAGCT	37		5	11851713	CATTAAC		5	11904713	R
	ACATACA	37		17	48211226	CGGCCAC		17	45566225	R
	GACTTTT	37		1	147144052	GTGATTC		1	145610676	R
	CCAATAA	37		6	31587680	GGCTAGC		6	31695659	F
	TCTGTGG	37		11	27016671	TATCCTT		11	26973247	F
	CAGTGCA	37		13	111522651	GCATGAC		13	110320652	F
Red	GGCTCTG	37	X		30671314	CGCGCCC	X		30581235	F
	TGGGATT	37		2	86251199	CGCTGTG		2	86104710	R
Red	TTCACAG	37		6	85824168	CGCCGCT		6	85880887	R
	GATTCCT	37		14	84720664	CACTACT		14	83790417	F
Red	GCCATTG	37		12	121147668	TTGGGAC		12	119632051	R
Red	GGAGACC	37		9	100000033	CGAGCTC		9	99039854	R
Red	TCAGTTC	37		9	15371248	CGGAGGT		9	15361248	F
	TACAGAA	37		9	18825658	AGAAATC		9	18815658	F
	ACCCTTT	37		5	142001033	TGGTCTG		5	141981217	R
	GTGCCAA	37		9	135879019	CGCCCAG		9	134868840	R
	CTGGCTT	37		19	8953473	CGAGAAT		19	8814473	F
	GACAAAT	37		6	1387836	CGCTCAG		6	1332835	F
Red	GGCCCGC	37		19	52954722	CTGGGTG		19	57646534	F
Red	TCGAGGA	37		8	144810657	CGAGCGC		8	144882645	R

	CCAACTT	37	9	36989127	GAGCTCA	9	36979127	F
	CAGATTT	37	11	93754223	CGGCTCA	11	93393871	F
	CCCTGCT	37	7	2289888	CGTCAGG	7	2256414	F
Grn	ACTCAGG	37	15	24506388	CGCGCTT	15	22057481	R
Red	GAGACCT	37	14	75722495	CGCAGAC	14	74792248	R
	GGGGTCC	37	9	138591683	GTTCCCA	9	137731504	R
	TATCAAG	37	9	38477418	CGGGAAA	9	38467418	F
	CCTTTCA	37	9	139635449	CGCCCAC	9	138755270	F
Grn	TCGTGCG	37	2	24583121	GGGTGAA	2	24436625	F
	CAGCAGT	37	9	138586311	TGAGCTC	9	137726132	F
Grn	GCGGGTC	37	17	4901063	CGAGCGC	17	4841787	F
Grn	TAAAGTC	37	19	15982569	CGGCACC	19	15843569	R
	CTCAAGC	37	14	105576362	CGCCCAG	14	104647407	F
Red	GTCCACG	37	5	76250527	CGGGTCA	5	76286283	R
	ACACCAC	37	6	32609783	CGGGAGA	6	32717761	R
	TTTTCAC	37	2	43618836	CGATGCT	2	43472340	F
	TGTTGTT	37	9	126816959	CGGCTGT	9	125856780	R
	GAATTCA	37	3	98848062	TCTGTGA	3	100330752	R
	GGGGACT	37	7	75829463	TTCCTGG	7	75667399	R
	TAGCGAT	37	10	28030033	CGAGAAC	10	28070039	R
	AATCCTG	37	Y	24453658	CGGCGCC	Y	22863046	F
	TATTATT	37	9	36100589	CGGAGGC	9	36090589	R
	CAATTCA	37	2	101124858	CGCTGAT	2	100491290	R
	GCCTCTC	37	9	34953950	CGACAAC	9	34943950	F
Red	CAGTAGA	37	X	8751330	CGGACCC	X	8711330	R
	ACATCCA	37	12	115118806	CGGGGTC	12	113603189	F
	TCCCAGC	37	19	16989699	TCGGGTG	19	16850699	F
	GATAACT	37	9	72735830	TGTATGC	9	71925650	F
Grn	AGTCTGT	37	12	9555480	TTTCCCC	12	9446747	R
Red	TGACTGT	37	9	38488083	CGGGCAC	9	38478083	F
	CTTTTTAC	37	15	24675297	CCAACAA	15	22226390	F
	TTAAAAG	37	6	30045936	CGGCTGT	6	30153915	F
	GCAGTAC	37	9	140356757	TCAGGCG	9	139476578	F
	TTAGACT	37	10	101421142	CGATATT	10	101411132	F
	AAAAATA	37	7	55638724	CGCATGA	7	55606218	R
Red	GTCTTCA	37	6	169092843	ATCTCTT	6	168834768	R
	AATTGAT	37	10	95848649	CGCAAGC	10	95838639	F
Red	TGATTTT	37	9	115835616	CGCCGGC	9	114875437	R
Grn	CACCCTC	37	17	77704250	CGTGGTG	17	75318845	F
	GCTATTT	37	19	58951672	TCCTTCT	19	63643484	F
	GTGATAC	37	12	95840895	TTTAATT	12	94365026	F
	AGCCTTA	37	6	52454298	TCCAACA	6	52562257	R
Red	GTCGGGC	37	17	47502298	GGAGTAC	17	44857297	R

	CAGGACA	37		9	84228185	GTTGTCT		9	83418005	F
	ATACCTG	37		1	1549699	CGGACTT		1	1539562	F
	TTTCCAA	37		9	77282233	TTGCGAA		9	76472053	R
Red	AGAATGT	37		12	130824015	CGCCGTG		12	129389968	R
Grn	AAGGAAC	37		11	5959945	CTTCTGA		11	5916521	R
	ACCCAAA	37		5	141485167	CGGTGGA		5	141465351	R
	GACCCAT	37		6	32549849	CGGCATG		6	32657827	F
	CGTCTTG	37		5	172970	AAATTCA		5	225970	F
	GTGCACC	37	X		152908309	CGCACCA	X		152561503	F
	TTTCTGC	37		11	35644854	GACAGTA		11	35601430	R
Red	CTGCGCA	37		6	30039380	CGCCCGC		6	30147359	R
Red	CGGGGGC	37		9	34810228	GACCGGC		9	34800228	R
	AGGGAGC	37		1	154194049	CGCACAC		1	152460673	R
Red	GCACAGC	37		1	1168432	CGACCCG		1	1158295	F
Grn	GAAACAC	37		11	10750890	TTCCCAT		11	10707466	F
	CCAATCC	37		9	84646696	AGAGTGA		9	83836516	R
	GATGTTA	37		5	39800321	CGGGGAA		5	39836078	F
	AAACATT	37		1	6063654	CGTTTCC		1	5986241	R
	CGTGGGC	37		1	179777372	CTCAGGC		1	178043995	R
	CCTTGGT	37		9	27575280	CGTCTAA		9	27565280	F
Red	CCCCGCC	37	X		153236838	CGCCCAG	X		152890032	F
Red	CACCCAT	37		15	65143918	CGCTGAA		15	62930971	R
	AGAGAGT	37		4	87959251	TGCATTG		4	88178275	R
	CGTTTGT	37		12	10096112	CAGCCAA		12	9987379	F
	CGTACAC	37		9	94533180	CGGCCGA		9	93573001	F
	GTTGTAG	37		5	32445523	CGTTCTA		5	32481280	F
	TTCCGGC	37		19	10928696	GATATGA		19	10789696	R
	TCTTTGT	37		7	127526253	ATCTTCT		7	127313489	F
Grn	ATGGGAA	37		9	137662888	AGCGCCC		9	136802709	F
	GAAAGCA	37		5	74344416	AGGATTT		5	74380172	F
	AGAGCTT	37		14	59183596	GCTGGTG		14	58253349	F
	TTTATTA	37		9	99776416	GAAAGAT		9	98816237	R
Red	GGAGACC	37	X		153210107	CGGGACC	X		152863301	R
	CAAGTTG	37		7	127222520	CTTGGGT		7	127009756	R
	GATGAAT	37		6	119862526	AAATGCT		6	119904225	F
	ACCACTC	37		17	10220829	CGGGAAA		17	10161554	F
	AACTGAA	37		17	57187728	CGGTAGT		17	54542510	R
Grn	GCCCTGG	37		9	134699541	GTGTTTT		9	133689362	R
	CTCTTTGA	37		7	154719422	CGACAGC		7	154350355	F
Red	ATACTGA	37		9	117134861	CGCTCCA		9	116174682	F
	AGGTCGC	37	X		53350200	GAGCTGA	X		53366925	F
Red	GCATAGA	37		19	46526321	CGGGCGC		19	51218161	R
	CTGTTCCA	37		9	93983210	CGCGAGT		9	93023031	R

Grn	CCTCGGC	37	2	131046122	CGCCGTT	2	130762592	R
	CGAACCC	37	10	104535020	CGGCCTG	10	104525010	R
	GCGCAAC	37	6	29911265	CGCACGT	6	30019244	R
	ATTTCCC	37	9	826657	GAGTGGT	9	816657	F
Grn	GGGGCAC	37	9	139943146	CCCGTCG	9	139062967	R
	AATGCCA	37	10	46010523	CAAACTG	10	45330529	R
	GAGGCGA	37	1	211909305	TGTCTCA	1	209975928	F
	TGTATAA	37	4	127811266	CGGAAGT	4	128030716	F
	AATCACG	37	4	169645874	CGATCAG	4	169882449	F
	CCAGCTG	37	8	134271505	TGGAGGC	8	134340687	R
	AACCAAC	37	6	32312351	GCTCAAT	6	32420329	R
	ATCCTGA	37	6	31439497	GATGTGG	6	31547476	R
Grn	GAGGTGC	37	19	853145	GGGAGGC	19	804145	R
	TGACCCT	37	3	185676640	CGCAGGC	3	187159334	R
	GCTGCCG	37	2	241860995	TTAGTAA	2	241509668	R
	ATTCAGC	37	9	115771599	AGCCAAA	9	114811420	R
	CTGGGGT	37	9	132598917	CGTAAGC	9	131638738	R
Red	CCTTAGG	37	19	18054643	GGGACCC	19	17915643	F
	GAGACCT	37	9	139406352	CGGCTGT	9	138526173	R
	TCAACAG	37	5	140579303	AAATCCT	5	140559487	R
	GCAAGGT	37	13	109439124	TGGTTAC	13	108237125	R
	TATAACC	37	9	110231817	GTTTATT	9	109271638	F
	TGTGACT	37	9	133838188	CGGAGAC	9	132828009	F
Red	CAGCCCG	37	6	32552515	CGCTTTA	6	32660493	F
Red	TGGAGGA	37	7	231526	CGCCAGC	7	326609	R
	GGCTGCC	37	6	127796989	TGCCCAG	6	127838682	R
	GCTCCGG	37	21	44782497	AAGGCCT	21	43606925	F
Red	ATTAGTC	37	9	130454671	CGGAACC	9	129494492	F
	TTTTTAGC	37	9	35848330	CGAAAGA	9	35838330	R
	GATACAA	37	14	91820590	CGCAGAA	14	90890343	F
	CACCACA	37	11	1682006	ACATTCA	11	1638582	F
Red	GACCCGC	37	13	23412409	CGCTACC	13	22310409	R
	TCCGGAG	37	19	6425737	CGACCCC	19	6376737	F
	CTAACCT	37	11	67141628	AGACAAA	11	66898204	F
	CCGCCAC	37	9	32979252	CGGAGCA	9	32969252	R
	AGCTAAC	37	13	21889774	GGCAAAT	13	20787774	R
	GGAGTGA	37	6	47666329	CGGCTTC	6	47774288	F
	CGGGGAC	37	X	9433597	CGCTCCA	X	9393597	F
Red	GCACCAT	37	19	50733985	GCAGCGA	19	55425797	F
Red	TGACAAC	37	12	8717487	CGCTCAA	12	8608754	F
	TGAGACC	37	19	8619925	GGCCAAC	19	8525925	R
Red	AAGGCCT	37	X	77154916	CGCGAGA	X	77041572	F
Red	GCGCCTG	37	19	14607017	CGCAGCC	19	14468017	F

	GCTAACT	37	2	134391847	GGTGATT	2	134108317	R
	AGCAAAA	37	5	121499498	CGCTGTG	5	121527397	R
	TTGGGAG	37	18	6283067	CGTCACT	18	6273067	R
	TTTTGCTC	37	9	100502742	CCTCCTA	9	99542563	F
	TTTGACC	37	Y	14652924	AAGTTAC	Y	13162934	R
	AAAATGT	37	14	52708188	AGCCTGC	14	51777938	R
	TCTTGTTC	37	Y	19678535	CGGGTCA	Y	18187929	R
	TCTTTTCA	37	9	28596895	TATAGCC	9	28586895	F
	AACCCAC	37	6	83893273	CGGACTG	6	83949992	R
	TCTCAAA	37	9	132937709	CGGGAGC	9	131977530	R
	ACAAAGC	37	2	206585563	CGGCCTG	2	206293808	F
	GGAATCT	37	9	138749560	CGGCCTC	9	137889381	R
Red	CTGGGCG	37	14	104574715	CGAGGGC	14	103644468	R
	TGAGGAT	37	11	70424559	CGCACAC	11	70102207	F
	AGCCCCC	37	7	155191709	CGAGAAC	7	154884470	F
	CTTCCCAT	37	21	43835592	CTCTAAA	21	42708661	R
	TTATACG	37	7	75269642	CGGCAGC	7	75107578	F
	AGTGGCC	37	9	82286351	ACTGTGA	9	81476171	R
	TGCCGTA	37	4	7374582	CGGGAGT	4	7425483	R
	AATTTGG	37	12	20003184	CGCTATG	12	19894451	F
	GATGCGT	37	2	97651611	CGGGCAC	2	97015338	F
	CTCAACC	37	7	42896343	TTCCCAG	7	42862868	F
	AGTTCCT	37	12	30878975	CTGAGTC	12	30770242	R
	TTTTAGC	37	15	77436852	TTGACTC	15	75223907	F
	CCAGGGA	37	11	65342283	CGGTCTT	11	65098859	F
Red	GGTAGAC	37	19	46417639	GCTGGCC	19	51109479	R
	TGCTGAG	37	17	80849463	CGGGAGT	17	78442752	F
	CAGAAAA	37	4	174723167	ATAGTTT	4	174959742	F
	TCTCAAA	37	14	67983484	CGTGTTC	14	67053237	F
Grn	TGACCTT	37	4	183063397	CTAGTGA	4	183300391	F
	CTCGCCT	37	6	170190660	GTGGGGT	6	169932585	R
	TGAAGCT	37	11	114166636	CGGGATC	11	113671846	F
	CCTCTGG	37	11	66738758	GAGCCTG	11	66495334	R
Red	GCTCTTTC	37	8	38508585	CGGTGTT	8	38627742	F
Red	AGCTCTC	37	14	24439157	CGCAACC	14	23508997	F
Red	CCGCAGC	37	7	27146445	GGAAGCT	7	27112970	F
	TGGGCTG	37	16	857981	CGCCAGT	16	797982	R
	CCCGGAC	37	2	113190197	CTGTATC	2	112906668	F
	ACAGATT	37	7	137013465	CGCAGGC	7	136664005	F
	CTCTTCTC	37	11	103677503	CGGGGCT	11	103182713	R
	CACCTGT	37	1	22328026	AACAGGT	1	22200613	F
	TGGGGCT	37	12	46005115	TGGGGCT	12	44291382	R
	CTTTAGA	37	11	6378691	CGGGGCT	11	6335267	F

	GACCCAC	37	7	48124921	GGGTTTC	7	48091446	R
	AGGAATC	37	19	15248535	CGCAGCT	19	15109535	R
	TTTCACT(	37	2	137077233	CTCCTGC(	2	136793703	F
	AGTGGCA	37	13	110132672	CGGGAAC	13	108930673	F
	CCTTTTG/	37	2	33226136	CGCTGAC	2	33079640	F
Red	CGCGAGT	37	19	12995624	CGGACGC	19	12856624	R
Red	CCTTCCC(	37	13	111935521	CGCAGAC	13	110733522	F
	GAGTCTG	37	2	238410067	GGTTACC	2	238074806	R
Grn	TTCCTGC(	37	6	74064434	ACGTTCC	6	74121155	R
	TTTTACA(	37	9	32455910	GTTACAG	9	32445910	F
	TATACTG	37	19	55669189	TCCTGTG(	19	60361001	F
	CCAAGCC	37	2	227662014	CGGTCAC	2	227370258	R
Red	CTGTCTT'	37	6	31164848	CCCCAGG	6	31272827	F
	TGATTGT(	37	9	128512035	TCAGAAT	9	127551856	F
	AATCTGC	37	14	68665820	GAATTCT	14	67735573	R
	GCATGGA	37	10	18995362	CGGCACC	10	19035368	R
	CCTCCCA	37	9	91361323	ATACAAA	9	90551143	F
	GCTCCAC	37	9	136134067	CGGCCCA	9	135123888	R
	AATGACT	37	6	33032830	TCTCCCC(	6	33140808	R
Grn	CTCATGA	37	19	51330469	CGGGTAA	19	56022281	R
	TGTCTGG	37	9	114812406	TTGTCAT(	9	113852227	R
	TCTGGAT	37	10	3568949	CGCCATC	10	3558949	R
	ACCAGGC	37	6	32574199	TTCCGAT'	6	32682177	F
	CAGAAAC	37	6	168436353	AATCAGC	6	168179202	R
Red	GATTTTC'	37	9	115851947	CGCCGGC	9	114891768	R
Red	GGGTCAC	37	8	216788	ACCTGCG	8	206788	R
	TGACCCA	37	19	22800693	CCTGGGT	19	22592533	R
	TATTCTT(	37	6	32628305	CTACTGC	6	32736283	F
	GTCTAGA	37	7	63498313	CGGGGAC	7	63135748	F
	CATATTC.	37	20	34700675	CGGTGGC	20	34164089	R
Grn	GGCTGTG	37	3	138763899	CGGCCCG	3	140246589	R
Grn	GTGTCCA	37	22	21025079	CGCTGGC	22	19355079	R
	CTTCAGC	37	9	111230512	CATGGTG	9	110270333	R
	ATCGTGG	37	7	75779857	CGGCCCG	7	75617793	F
Grn	TACTTGG	37	14	104696094	CGATCAA	14	103765847	F
	GGACTTG	37	17	12631363	CGAAGGC	17	12572088	R
	TCTCCAC.	37	17	77922738	GGAGCAC	17	75537333	F
	CTCATGC	37	X	70318366	TTGCTCC(	X	70235091	F
	AAAGAG/	37	13	27927490	CTTTGCA(	13	26825490	R
	CCAGGCT	37	19	58665643	TGAGTGC	19	63357455	F
	CCCAATT	37	5	126408756	TCAACAT	5	126436655	R
	CGAGGCC	37	9	839557	GGCGCCT	9	829557	F
	GGCTTGT	37	21	46890997	TGGGTGT	21	45715425	R

Grn	CGTCCCC	37	13	111465457	CGCCCTC	13	110263458	F
	ACATCAC	37	6	31509244	CTTGACT'	6	31617223	F
	GGCTTAG	37	1	28417750	AGTGTGC	1	28290337	F
	CGCAGGC	37	2	193393	ACTAACG	2	183393	F
	TGTTTCC'	37	5	150051778	CGGCGGC	5	150031971	R
Red	TACCCAC	37	13	23412250	CGGCTAA	13	22310250	R
	TCCATCC.	37	7	50570049	ACTGTGA	7	50537543	R
	GGCTCTA	37	18	77836355	GGGATGT	18	75937343	R
	CGGGGCT	37	6	43964296	CGTTTCT(	6	44072274	R
Red	GACTGCC	37	2	131009681	CGGCCCT	2	130726151	F
	AACTAAA	37	2	225798831	CGATCAC	2	225507075	R
	GGGCTGA	37	6	37425031	TCATTTG.	6	37533009	F
	CAACTTG	37	2	198666752	CGAGCAC	2	198374997	R
Red	ACGCCCG	37	9	126794772	AGTTCAG	9	125834593	R
	ATTCCAC.	37	9	120160335	TAAAGAT	9	119200156	F
	CATCGCT	37	12	70002985	GTAACTG	12	68289252	F
	CCTTGCG	37	7	157260817	GTTAGTT'	7	156953578	F
	TGGAAAC	37	12	115107540	CGGGCTT	12	113591923	R
Red	CCTGGGA	37	9	93799278	CGGGGCC	9	92839099	R
	TGCATCA	37	9	2817906	CGCTGGA	9	2807906	R
	GGAATTT	37	6	141301409	GCTCCCA	6	141343102	R
	ATCACAG	37	4	99352003	CGGAATT	4	99571026	R
	AAGAAA/	37	9	123257038	ACTAGGT	9	122296859	R
	GAAGAG/	37	11	120678447	CGGAGCT	11	120183657	F
	CCCATCG	37	Y	15817372	CGGAGTC	Y	14326766	F
	CTGGATC	37	Y	23757241	CGGGAAT	Y	22166629	F
	GTCTTCA'	37	9	89149587	CGGGAGT	9	88339407	F
	AGAGATT	37	8	8506370	GGATTCA	8	8543780	R
	CCCAATG	37	9	134484581	CGTCCTC.	9	133474402	R
	TCGTCTG	37	4	132896579	CGGCTGC	4	133116029	F
	GCCTGAG	37	4	528497	CGGGGGC	4	518497	F
Red	CTCGCAG	37	6	159655392	CGCGCGC	6	159575382	F
	TGGGTTG	37	2	218295405	TCATGCT'	2	218003650	F
Red	GCCCCGC	37	2	636606	CGCAGCC	2	626606	R
	CATGGTG	37	7	83097669	CGGTGTG	7	82935605	R
Grn	CACAGAC	37	7	157263638	AAGGCAC	7	156956399	R
Grn	GAGACGC	37	5	140582125	CACCACG	5	140562309	R
	CCACAAC	37	14	91017836	CGCTGAA	14	90087589	F
Grn	GCCGTCC	37	14	102973717	GCCGCGT	14	102043470	F
Red	CGTCGCC	37	2	27341893	CGCCGCC	2	27195397	F
Red	AGACAGC	37	21	35831954	CCCGGGC	21	34753824	R
	TGTTTAT/	37	2	11430093	CATTTTT	2	11347544	R
	CTGGTGA	37	19	55417500	ACATCGC	19	60109312	F

	TAGCAGA	37	2	154262055	GGCTCCA	2	153970301	R
	ATGCTGA	37	2	88367362	CGCGGCA	2	88148477	R
	CCATAGC	37	9	40698808	AGTTGAT	9	40688808	F
	TAAATGG	37	9	126523663	CGCAAGC	9	125563484	F
	GTCCTCA	37	12	107348689	ACCTTCT	12	105872819	R
	TATCGGA	37	9	13280025	CGCAGGT	9	13270025	R
	TTTAGCT	37	3	10019040	CAGAGCC	3	9994040	R
	TATTTGG	37	15	31749573	TGCACAG	15	29536865	R
	GTGGGTG	37	5	1300309	ACAGCAC	5	1353309	F
Grn	AGCGGTC	37	9	96623480	CGGAGCC	9	95663301	F
	TCATGCC	37	15	84363061	GATAACA	15	82154065	R
	CCTGCCC	37	X	133507642	TAAAGGC	X	133335308	R
Red	GGAAACC	37	5	178577570	AATGTCC	5	178510176	R
	GATTTTC	37	9	93881805	GGAGTCT	9	92921626	R
Red	GGCCTCT	37	19	17571873	CGCATTA	19	17432873	F
	GACATCA	37	10	111989324	CGTGCAG	10	111979314	R
	AGGAGGC	37	1	203526848	TGTGTCT	1	201793471	R
Red	GCGGGAC	37	1	35586649	TCTCTCA	1	35359236	R
	GTATCGG	37	11	65363274	GGGCCTT	11	65119850	R
	ACATCAC	37	9	100000026	CGAAAGC	9	99039847	R
	CAAATTT	37	X	47229908	AAATCCT	X	47114852	F
	ACTGTGT	37	7	128003225	GAATGAT	7	127790461	R
	GGATGCA	37	9	136133628	CGGGAGC	9	135123449	R
	AAAGTTT	37	11	35547903	CGCAGGA	11	35504479	R
	AAAGCTG	37	9	27408242	CAATTTC	9	27398242	F
Red	CAGGAAA	37	6	32552205	TGTGGGG	6	32660183	R
	GGCATTT	37	10	126158871	CGGGCCT	10	126148861	R
	TCTCCCT	37	7	157209551	GAACTGA	7	156902312	F
	CGCACTG	37	17	76676303	TCTCAGC	17	74187898	R
	TTTCCTG	37	9	130151001	TCACGAG	9	129190822	R
	GGCGTGA	37	2	120063972	CGAACCA	2	119780442	F
Grn	CCCTACA	37	2	233246431	AGCAGCA	2	232954675	F
Grn	GTGGCCG	37	1	54151053	AGGAATC	1	53923641	F
Red	TCTGCTG	37	11	64216417	CGTGGCT	11	63972993	F
	AAATGTC	37	19	35736529	TACCCAA	19	40428369	R
	TCATAGC	37	6	31937758	CATACCA	6	32045737	R
	ATGTGGA	37	5	112405789	CGGTTAT	5	112433688	F
Grn	TACAGCG	37	1	155247706	CGGTATT	1	153514330	R
	CCTGTAG	37	19	10232899	TTCATAT	19	10093899	F
	TTCCCCA	37	12	54385526	CGCTCAG	12	52671793	F
	GGTAGTG	37	2	223784609	AAAACCT	2	223492853	F
	TGCGTCG	37	6	8776747	AATATCT	6	8721746	F
Grn	CCCTCCC	37	12	31272119	TGGCTCT	12	31163386	R

	GGAGGCT	37		7	1688174	GGTGCAC		7	1654700	F
	CGCCAGC	37		2	133014784	CGGCATG		2	132731254	F
	AGCAATC	37		11	86644657	CGGCTAT		11	86322305	F
	CCATTCT	37		19	18091245	CGCTTGG		19	17952245	R
Red	GTGGGTT	37	X		47092993	CGCCCCC	X		46977937	R
	TTGGGTT	37		10	64193068	AATGAAT		10	63863074	R
	CAGTCTT	37		12	132514956	CGCCAGT		12	131080909	R
Red	GGCCTGC	37		11	71724583	CGCACGC		11	71402231	F
Grn	GCTCCAA	37		5	1060679	CACTGGG		5	1113679	F
	TTAACTT	37		1	227689829	CGGGAGC		1	225756452	R
	TGCGTGA	37		3	128597701	TCTCTCT		3	130080391	R
Red	GTCTGTG	37		17	77127487	CGTTGGT		17	74639082	F
	AGAGAAC	37		10	96796860	CGGCAGC		10	96786850	F
	CACTCTG	37		10	129789429	CGGGATC		10	129679419	F
	GGGACTC	37		6	24724537	CGTCCTC		6	24832516	R
	TTGGGAG	37		12	115102713	GAGGTTG		12	113587096	R
	GTGAAGT	37		10	34476091	ATGGTAA		10	34516097	F
	CCCCTTG	37		22	30195299	CGGCCAC		22	28525299	F
	GAGGTGC	37	Y		17569363	GTTATAC	Y		16078757	F
	CCATCAC	37		11	43852883	CAATTGC		11	43809459	R
	TTCTAGA	37		18	12041672	CGCAGTC		18	12031672	F
	GGACGTC	37		20	43945254	CGGGGCC		20	43378668	R
	TGGGAAC	37		6	166117676	CGCAGCC		6	166037666	R
	GATCCAG	37		4	155254602	TTCAGAC		4	155474052	F
Grn	CCGCGTC	37		2	20870812	GGGCAGT		2	20734293	R
	CTGGTGT	37		6	33131893	CGGCTTT		6	33239871	F
Grn	GGGCACC	37		2	1480944	CGCGCTC		2	1459951	F
Red	TGTCATT	37		3	146262023	CGGCAAC		3	147744713	R
	CTCAAGT	37		3	128267611	CGCAAAA		3	129750301	R
	GCAAGTT	37		5	124309096	CGAGACA		5	124336995	R
	AAGCCCA	37		5	72602175	CTTGGCT		5	72637931	F
	TCCACTA	37		6	32608095	TTTGTTC		6	32716073	R
	ACTTGCC	37		2	86418754	CGCGCCT		2	86272265	R
	CCCCGTT	37		17	73055792	GGTCTGC		17	70567387	R
	GTAAGAC	37		10	103333981	TTTAATT		10	103323971	F
Red	CCCCCCT	37		20	29611903	CAGAAAC		20	28225564	R
	GTATGAA	37		7	41956427	CGCTTCA		7	41922952	F
	CTCTGCG	37		3	56717657	CGGTTTT		3	56692697	F
	CTGTCCC	37		17	75598365	TCATGTG		17	73109960	R
	AGGGTCT	37		5	78531681	CGGGTTC		5	78567437	F
	GCAGTTT	37		1	24921758	CGGTATC		1	24794345	F
	TGTGTAA	37		12	11287271	CGGGTTA		12	11178538	R
Red	GCTCGCC	37	X		30671327	CGCCCCC	X		30581248	F

	GATCATT	37	17	48275004	AGGGCAC	17	45630003	R
	CCGGGTG	37	2	54087028	GAGGCGC	2	53940532	F
	TCTTCCA	37	1	152506886	CGCCGTT	1	150773510	F
	TAAACAA	37	7	129868685	TTATGAT	7	129655921	R
Red	GTTTCGT	37	13	112927276	CCTCGTG	13	111975277	R
	AGTACAT	37	1	20235923	CGCAGAC	1	20108510	F
	CGTGTCT	37	14	102438734	CTTTGCT	14	101508487	F
	GGCTGCA	37	5	149683334	TTAAAAA	5	149663527	R
	TGTCACG	37	1	19111089	CGGCCAT	1	18983676	R
Red	ACCCTGG	37	12	6436676	CGCCGAA	12	6306937	F
	AGAGTTG	37	1	154238265	CGTGGTT	1	152504889	F
	TCCTCAG	37	4	146841701	ATTGTCC	4	147061151	F
	TTTCGTG	37	8	104032876	CGCCAAC	8	104102052	R
	GTCACCT	37	10	34151366	CGCTGGC	10	34191372	R
Red	TGCGGAC	37	2	3699353	CCCGCGA	2	3677228	F
	CTTTCCC	37	14	57021828	CGAGGGA	14	56091581	R
Red	CACATCA	37	18	30353000	CTTGGGC	18	28606998	R
	CCCAGCG	37	14	102554969	GACCGGC	14	101624722	R
	AAGACTC	37	11	65794352	CGGGAAA	11	65550928	R
	GTCACTA	37	8	28930481	CGGAGGA	8	28986400	F
Grn	TCCTGGT	37	4	7735312	CGCGGTC	4	7786212	R
	ACCACCC	37	2	131046313	CGGAGGT	2	130762783	F
	GAGAGGA	37	6	35454069	CGGGCTC	6	35562047	R
Red	GGGGCCC	37	20	43935471	CGGCTCT	20	43368885	R
Red	GGGCTGA	37	6	37616482	CGCCTTC	6	37724460	R
	GGGGAGC	37	16	88793811	CGCAGAC	16	87321312	R
Red	GGGTGTC	37	2	241562485	AGAAGGC	2	241211158	R
	AGGGAGC	37	6	31238803	CGGGAGA	6	31346782	F
Red	AGACCAC	37	10	369977	GGAGGGC	10	359977	F
	AACTTTT	37	8	122118171	GCCATGC	8	122187352	R
Grn	AAGGGCT	37	19	3595702	CGCTCCG	19	3546702	R
	CTGTCAT	37	10	128947230	GAGGATT	10	128837220	F
	TCACTAG	37	11	47256035	CGGGCAT	11	47212611	R
	TTTGCAT	37	3	115155686	CGGCTGT	3	116638376	R
Red	GGCTGCT	37	2	27373128	CGCCGCC	2	27226632	F
	AAAACTC	37	16	4525986	TGACACC	16	4465987	F
	AGTTCTT	37	7	141285961	CGGGGAC	7	140932430	R
	CTGCCCA	37	21	47530705	GATGCCC	21	46355133	F
	ACGCCGA	37	19	37794573	TCAGCTT	19	42486413	F
	CATCGCC	37	8	810491	TTTTCTG	8	800491	R
	ATCTATG	37	3	194314457	CATGCTT	3	195795746	F
	ACAGCCC	37	2	70942718	CGGCAGC	2	70796226	F
Red	CAACCTG	37	7	48129822	CGACCGC	7	48096347	F

	AATGGCT	37	19	29218262	TTGGGCT	19	33910102	F
Grn	GAGGCAC	37	2	97136729	AGTATAG	2	96500456	R
	ATTTGCC	37	3	158427635	ATTTGCT	3	159910329	R
Red	TCTTGCA	37	12	115105220	CGCTAAT	12	113589603	F
	TCATTAG	37	1	71555947	TTAAAAG	1	71328535	R
	ACCAAAC	37	1	3721781	CGCTCCG	1	3711641	R
	TTTTTCTT	37	5	82771274	TCTGCTG	5	82807030	R
	GGTTCTC	37	6	49681178	CGGGTCT	6	49789137	F
	CGCAAAA	37	9	115822072	CGGGGGC	9	114861893	R
	GGGCACC	37	8	17454394	TTTCACA	8	17498671	R
	CCTGATG	37	5	72802430	CGCTGGC	5	72838186	F
Grn	TGGGTGG	37	19	50931432	CGCCGCT	19	55623244	F
	GTGCCCT	37	1	49085506	CGCTGAT	1	48858093	R
	CATGAAT	37	6	32609212	AGTGCAC	6	32717190	F
Red	CCCCACC	37	13	91827286	TTCCTTC	13	90625287	F
Red	AAGCGCC	37	17	46908359	GGGGCGC	17	44263358	R
Red	TCCATTT	37	1	152161237	ACCATGT	1	150427861	R
	CTGCATT	37	X	7137413	TGCCTAG	X	7147413	F
	AGCTGGT	37	1	2082458	TCGTGCA	1	2072318	F
	TCTCATT	37	1	223889141	AAAGGGC	1	221955764	F
	GCGAGGC	37	17	35501295	CGTAACC	17	32575408	F
	ATACGTT	37	10	126434204	CATAGCA	10	126424194	F
	CAAGGAT	37	5	140501784	GAAGCAA	5	140481968	F
	GGCGTGC	37	22	16288741	CGGATGC	22	14668741	F
	CGGAATC	37	6	152464791	CGGGGCC	6	152506484	R
	CAAAAAC	37	11	129310863	GTTGCTG	11	128816073	R
	CACCTTC	37	Y	22737505	CTGAGGC	Y	21146893	F
Red	AAGGGTA	37	1	149680232	CGGGTCT	1	147946856	F
Grn	GGAAATC	37	3	13974467	GAGAGAA	3	13949468	R
Red	CTAATTT	37	7	130130203	GAGAGAC	7	129917439	F
Grn	TTCAATG	37	13	112547653	TCACGAG	13	111595654	F
Grn	ACCGAGT	37	11	1892307	TGCATGT	11	1848883	R
	GATGCTT	37	2	160758480	TATCTTT	2	160466726	R
	ACAAACT	37	3	14160960	CGACTTT	3	14135961	R
Red	AGGCCAC	37	10	3169259	CTCCGGC	10	3159259	F
	GGGGCAC	37	1	247511364	CGCCCCA	1	245577987	R
Red	TCGGATA	37	4	4860061	GTGAGCC	4	4910962	F
	AACTGGC	37	19	30365441	CGGCCAC	19	35057281	F
Red	TCTGCTT	37	4	167448796	CGGAACA	4	167668246	F
	CTGGTGC	37	4	940614	GGCTACA	4	930614	R
	CAAACCC	37	5	140166589	GCAAGGT	5	140146773	R
Red	GCCTCCC	37	19	51330265	CGCCTGA	19	56022077	R
	AGCCGCC	37	3	10150083	CGCCCCC	3	10125083	F

Red	CGACTCC	37	5	140256817	GCAGCGC	5	140237001	R
	GGATCGC	37	17	76503526	CATTAGG	17	74015121	R
	GAACCAC	37	1	16060610	CGGGCAC	1	15933197	R
	TGTTGCC	37	2	33359281	ATCTCCT	2	33212785	F
Red	TTCTGCC	37	2	66805086	CGCCTTT	2	66658590	F
	CATCTGT	37	6	33051749	CGGGATT	6	33159727	F
Grn	CAGCTCC	37	3	48694534	CGCCGAC	3	48669538	F
Red	AGGAACT	37	5	6755386	CGCGAAC	5	6808386	R
	AAAACAC	37	16	28550619	CGGCCAC	16	28458120	F
Grn	CCCGCAG	37	6	160023626	TCAGTCA	6	159943616	F
	ATCCCAG	37	15	40272006	CGGGACC	15	38059298	F
	AAGCGGT	37	19	21265264	GTGGCTG	19	21057104	R
Red	GAGTCCC	37	19	50249737	CGATGCC	19	54941549	F
Grn	AAACCCA	37	5	123679203	CGCTGCG	5	123707102	R
	TGAAATA	37	19	18888799	CGCCAGC	19	18749799	R
Red	CTTCCTC	37	17	19883474	AGCGGGC	17	19824066	F
	ATGCCTC	37	6	1841153	CGCCCTC	6	1786152	R
	CTGCTCC	37	1	46858748	CGGTGGC	1	46631335	R
	CACTTTT	37	6	85821320	TCACTGC	6	85878039	F
	TGCCAGG	37	1	55909399	CGGCAGC	1	55681987	R
Grn	CTTTAAC	37	1	6526051	CGGAAAC	1	6448638	R
	CTGAGGT	37	4	152096590	CGGCTGC	4	152316040	F
Red	TGAAAGA	37	19	18344520	CGCGGAC	19	18205520	R
Red	CTCCCCG	37	6	35479628	CGCGAGC	6	35587606	F
	TGCTGAC	37	5	7540955	CGGACCA	5	7593955	R
Red	TCTGCCG	37	X	73642618	CGCCCGC	X	73559343	R
Grn	AGCAAAC	37	20	43935462	CGCGGGT	20	43368876	R
	TGTGGAC	37	10	134109919	CGGGTAC	10	133959909	R
	ACATCCC	37	3	111853297	CGGGTGC	3	113335987	R
	ACCAGGC	37	12	68405565	GTAGGCT	12	66691832	F
Red	CTAGATA	37	17	80627638	CGCTCAA	17	78220927	R
Red	CCCAGTC	37	8	8770972	TATTTCT	8	8808382	R
Red	AGCCTAG	37	12	8025582	CGGGAGC	12	7916849	F
Red	CCCCGAC	37	22	24384393	TGCCCCA	22	22714393	R
	AACTCTT	37	7	14031941	CGCTACA	7	13998466	F
Grn	ATGTCAG	37	10	29214117	GCTGCTT	10	29254123	F
	GCAACGA	37	6	171036378	ACTGGTG	6	170878303	F
	CTGGGCG	37	12	125346253	CGGCCCC	12	123912206	F
	GATTTCT	37	7	158069943	CGCACAC	7	157762704	R
	CCTACAT	37	10	237794	CGGTGGC	10	227794	R
Red	CGGCAAC	37	17	76897139	TCACACG	17	74408734	F
	CCTCAAT	37	17	15176491	CCAATCT	17	15117216	R
	CCGTTTG	37	17	80557543	GATGTGA	17	78150832	F

	GGAATCC	37	2	1166311	AAAGATC	2	1156311	R
	GCTGAAA	37	3	151309377	CGATCTA	3	152792067	F
	TTGTTAC	37	6	160182438	CGCTCCC	6	160102428	F
	CGCCCAG	37	6	39040812	CCAGTGG	6	39148790	R
Red	TCTCATC	37	12	114162813	CGCCGAT	12	112647196	F
	CACACTA	37	8	1310764	GGTCTAG	8	1298171	R
	TGCTTCT	37	2	180427325	AATCATA	2	180135570	R
	GTTGTCT	37	15	79973958	GCAAGCC	15	77761013	R
	GCAGATC	37	7	157730196	AGCTACT	7	157422957	R
	TTAGCCG	37	3	50648891	GGACCTC	3	50623895	F
Red	AGGTGGC	37	Y	21239461	GAGGACC	Y	19698849	R
Red	GTCCTCC	37	10	88702795	CGCGGCC	10	88692775	F
	AATTCCT	37	3	24203479	CGCCCAG	3	24178483	F
	CATGAAA	37	3	143567492	TTTCATA	3	145050182	R
Red	AGGGACC	37	12	131057032	TGGACAC	12	129622985	R
Red	GGAACTC	37	5	140535810	CGAGCTG	5	140515994	R
	GCTGCGG	37	14	24780551	CGCCATC	14	23850391	F
	TTGTGCC	37	6	149356915	GGTCAAT	6	149398608	F
	ATACTGA	37	7	107205253	TAACAGC	7	106992489	R
	CGAGGTC	37	X	21674175	CGCCCAG	X	21584096	F
Red	GGAGCAC	37	5	140475049	CGGCACC	5	140455233	F
	GTATGAC	37	11	74654369	CTTTTCT	11	74332017	R
	ACCAGCC	37	11	59209777	CGCGACT	11	58966353	R
	GACCCCA	37	1	207996459	CGCAGTC	1	206063082	R
	CAAGCCT	37	12	87232821	CGCTTAA	12	85756952	R
	AACTGCG	37	1	95010070	CGGTAAA	1	94782658	R
	CGCATGA	37	19	43769725	CGATCCT	19	48461565	F
	CGGCTTT	37	2	31806275	CGTGGGC	2	31659779	F
Grn	ACTCTCC	37	19	4724679	GGAAAA	19	4675679	R
	GCACTGC	37	5	126409310	TAGTTTG	5	126437209	F
Red	TGTCCCC	37	10	135105487	CGGGAGA	10	134955477	F
Red	TATTGAG	37	12	54392884	CCCAAAA	12	52679151	F
Red	ACCAGGC	37	6	74064047	CGCCCCC	6	74120768	F
	ACTCAGA	37	11	112028288	CAGCACC	11	111533498	F
	TTTTGCC	37	14	69341905	CCTGTAA	14	68411658	R
	CAAAACC	37	7	64450926	TTTAAGT	7	64088361	F
Red	AAGGCCC	37	3	194901357	CGGCGGT	3	196382646	F
Red	TCTCTCG	37	17	48270097	GGGACCC	17	45625096	R
	GCACACA	37	14	103687580	CACCAGC	14	102757333	F
	TATGACA	37	7	12247070	GTGGACC	7	12213595	F
Red	GACAGAT	37	7	8029589	TCTATGT	7	7996114	F
	GTGTCTC	37	1	221057561	TCCTTGT	1	219124184	F
Red	TAAAAGC	37	X	153218916	CGGTGCC	X	152872110	R

	AAGATTT	37	1	65889855	CAACCTT	1	65662443	R
Grn	GCAGGGC	37	21	44574022	CGGGCCA	21	43447091	R
	CACGAGT	37	17	4124222	TTTTCTGC	17	4070971	F
	GGGATTC	37	10	133657199	CTAGGGC	10	133507189	R
	ACAAGGC	37	22	18832673	CGGATGA	22	17212673	F
Grn	GGTTTCCT	37	10	2543763	CGCGGTT	10	2533763	F
Grn	CCTCTGTC	37	20	61447369	GGCTGAC	20	60917814	R
	TGTCCCTC	37	13	19919208	CGGCTCT	13	18817208	R
	CTTTGCAC	37	14	101539455	GAAGCAT	14	100609208	F
	CTGGGCT	37	2	101086893	TCTGGTT	2	100453325	F
	TACACCG	37	8	123706630	GAAGCAT	8	123775811	R
Red	TCCAGGG	37	12	129554566	CGGCCTC	12	128120519	R
	ACGCAAA	37	8	144661051	CCTTCAT	8	144732194	F
	CGTGCTG	37	2	238224257	CACACAC	2	237888996	F
	GTGACAC	37	1	11922765	CGACTGC	1	11845352	R
Grn	AGCTCGA	37	2	99771531	CACGAGC	2	99137963	R
	ACGATTA	37	5	140588139	CGCAATA	5	140568323	F
	ATTTTTGT	37	19	54183186	TCAAACA	19	58874998	F
	TAATATT	37	7	12411370	CTTTGTGC	7	12377895	R
	CTAGACC	37	7	563891	CGTGGCC	7	530417	F
	GCCGCTG	37	6	32551949	CGGCCCG	6	32659927	F
	CTTGTCTC	37	7	30430591	CGCAAGA	7	30397116	F
	CAGGCCC	37	3	61235985	AAGGTTC	3	61211025	F
Grn	CGTGCGC	37	7	2019874	CCCGCTG	7	1986400	R
Red	GCCCCTA	37	6	33041218	AGGAGCT	6	33149196	F
	CTTCCACT	37	11	35551852	CGGCTGT	11	35508428	F
Red	ACAATTA	37	6	29692475	CGCGGTC	6	29800454	F
	GTCTGTG	37	1	111733668	CGGCTTC	1	111535191	F
Red	TGGACGA	37	5	1867978	CGCAGAA	5	1920978	F
	TCCTTAGC	37	5	150005953	CGAAGGC	5	149986146	F
	ACCTGGT	37	1	17309539	CGCAGAC	1	17182126	R
	TGTCTAA	37	2	1166067	CGGGCTG	2	1156067	R
	TATCTTTC	37	5	41087721	AGTCATA	5	41123478	F
	GAGCCCC	37	13	45563400	GAGACTC	13	44461400	R
	TGGAGAT	37	10	80270722	CGAGTAA	10	79940728	F
Red	CGGGGTA	37	7	27224343	CGACCAC	7	27190868	R
	TTAGCAA	37	4	48137323	TCCCTGC	4	47832080	F
	CCGCCAA	37	8	131025902	CATGAAT	8	131095084	R
	ACAGCCA	37	10	3179703	CGCTTGC	10	3169703	R
Red	ACCTGCG	37	6	31238908	CGTGCCC	6	31346887	F
Red	CGAGTCC	37	10	82295864	GCCGCGC	10	82285844	F
	AGAAAGA	37	12	54448769	CGCTGTG	12	52735036	F
	CCTGATC	37	2	30669759	CGCGAAC	2	30523263	R

	CATCACA	37		2	43323957	GTCTGTG		2	43177461	R
	AGGTCCT	37		13	113030673	TCCACAG		13	112078674	R
	GCCCGGA	37		6	29895116	TCCAGGT		6	30003095	F
	TGTTGAG	37		11	44881786	CGCCTCC		11	44838362	F
Grn	TGGCAGA	37		6	33037413	TGAATAC		6	33145391	F
Grn	AGCATGT	37	Y		9747821	CGGCTGC	Y		10357821	R
Grn	TTCTGAC	37	X		78622682	CGCAGCC	X		78509338	F
Red	CAGATGA	37		1	1226929	AGCCCAC		1	1216792	R
	GGGGCTT	37		11	17498952	CGCTGTA		11	17455528	R
	GCTCTCT	37		5	1795014	CGAGGCT		5	1848014	F
Red	GAACCGC	37		19	50931515	CGGCGAC		19	55623327	F
	ATGGAGA	37		6	101993140	TGAGAGA		6	102099833	F
	CAGTGGA	37		5	76429218	GCTTGGA		5	76464974	R
	TCCGCGG	37		5	140457609	CGGCCCT		5	140437793	F
Red	GCCCCTG	37		1	106623593	CGGGCTC		1	106425116	F
	GTCCCCG	37		7	45808275	CGCCTCA		7	45774800	R
	GGACAGA	37		2	103239842	GAGTTTT		2	102606274	R
Grn	TATCCGG	37	X		151806528	ACTACTG	X		151557184	F
	CCTGGCT	37		22	49447845	CGGGGTA		22	47833849	R
	ATACCAC	37		17	39217766	TTCTGTG		17	36471292	R
	GCAATGA	37		4	7769884	CGGGGCC		4	7820784	F
	GGGCCTG	37		8	38782090	GCGCTGG		8	38901247	R
	CAGGACA	37		3	30714671	CGCAGTG		3	30689675	F
	CCAACAC	37		3	46718979	TAGGGGC		3	46693983	F
	CGGGCCC	37		22	51135736	CCTAGTG		22	49482602	F
	TGAGTAG	37		18	64271469	CGAGCGT		18	62422449	R
	GAAGCCC	37	X		149530190	CGCCCAA	X		149280848	R
	CAACTCA	37		12	131171530	CGCAGCC		12	129737483	F
Red	ACCCAGC	37		12	8717467	GGCAACC		12	8608734	F
	TCATACA	37		3	99988576	ATCAGCA		3	101471266	F
	AGCTCGG	37		6	32634481	CAGTGCT		6	32742459	F
	TTGTCTC	37		5	4866318	CGTGGAA		5	4919318	R
	AGGACGC	37		7	152600816	CGGGAAT		7	152231749	R
	TTAAACC	37		7	37484562	CGTCGTC		7	37451087	F
Red	TACCCTG	37		18	77292134	TGCTGCA		18	75393122	F
	TACACCA	37		3	141022255	TTAATCT		3	142504945	R
	GCCAGAA	37	Y		7569967	CGTTGAG	Y		7629967	F
	TGGACGC	37		7	2100357	CGGCCTT		7	2066883	F
Red	GGAGCCA	37		12	133014399	TGTGGGT		12	131524472	R
Red	AGTGATT	37		19	15833777	GATCCCG		19	15694777	R
	CCAGCTG	37		11	494944	CAACCAC		11	484944	F
	AGGTTGC	37		19	4638503	CGCCCTG		19	4589503	R
	GACCCCA	37		7	76129360	CCTTGTC		7	75967296	R

	GTTGGGC	37	19	38345819	TGCTTTT	19	43037659	F
	ATTACTC	37	4	54392970	TCTCTTG	4	54087727	R
Red	CTCGCCT	37	14	101366368	CAAACTT	14	100436121	F
Grn	CGGTCCC	37	13	25506235	GGGACGC	13	24404235	R
	CAGAAAT	37	16	85832091	CGGGAAC	16	84389592	F
	AGTTGTT	37	11	18617206	TTAAAGA	11	18573782	R
Grn	GTCGGGC	37	15	45403733	CCAACCA	15	43191025	F
	TCCCACC	37	4	124572578	CTAGAAT	4	124792028	R
Grn	GCCGCCG	37	1	247802935	CTCGCAG	1	245869558	R
	GGACTCT	37	1	147801025	CGTCCCA	1	146267649	R
	CATCGTT	37	2	231734413	CAGCCAA	2	231442657	F
	GGGCCAT	37	2	242887344	CGCATGA	2	242536017	R
	TGGGAGA	37	21	46850665	CGGGCCA	21	45675093	R
	CCAGCCA	37	5	16785399	ATCTTCC	5	16838399	R
	TACCTCT	37	6	31913250	CGGGTCT	6	32021229	F
	CTTCAAG	37	15	23692512	CAGTTGC	15	21243605	F
	CGCTCTC	37	15	40583422	GGGGCAC	15	38370714	R
	GAATTGT	37	2	52656979	CGGAATC	2	52510483	F
	TAGTATC	37	7	104945248	TACAGGA	7	104732484	R
	AGGAAAT	37	11	61450294	GGGTCAC	11	61206870	R
Red	AACCTGA	37	X	43741557	GGATCGA	X	43626501	F
	TGACCTA	37	7	123713651	CGGGAGT	7	123500887	R
Red	AGCCCTG	37	6	30039376	CGCGCCC	6	30147355	R
	TACTAAC	37	12	106361273	CGCTCTG	12	104885403	F
Red	AGGTTAA	37	12	122265088	GGGGCAC	12	120749471	R
	AACAAAA	37	7	126984590	CGCAGAT	7	126771826	R
	GACAAAA	37	5	141625843	GAAACAA	5	141606027	F
	GGATATA	37	10	20210338	CGAGGAA	10	20250344	R
	GAGTTAA	37	1	214650225	TATAAAT	1	212716848	R
	ATTGTTA	37	4	78852682	CCTCCTG	4	79071706	F
	TTAATTG	37	2	69586350	GTAAGTA	2	69439854	F
	TGTTGAA	37	7	126698829	CAGATAA	7	126486065	F
	AGATCAT	37	5	17381798	GCCACAT	5	17434798	F
	CCAATCA	37	11	58208367	AATCTCT	11	57964943	R
	TAGTTAT	37	5	140556888	TGGATTT	5	140537072	R
	ACAGCAC	37	4	2431872	CGGACAC	4	2401670	F
	AGTAGCA	37	10	27909711	CGGTACA	10	27949717	F
	GCGAGAC	37	14	103687675	GTGGATG	14	102757428	R
	AAAGAAC	37	17	68984117	TCCTCAA	17	66495712	F
	GTTGGAG	37	8	113655436	CGGCTGC	8	113724612	F
	GAAGGGT	37	6	30421089	CCAGGAC	6	30529068	R
	TAGCCTG	37	17	80040611	CACAGGA	17	77633900	R
Grn	CAAGGAT	37	1	19600454	CGGACTG	1	19473041	R

	AAGCAGA	37	5	141879669	TATTCTC	5	141859853	F
	GGGAATC	37	6	30079192	AGGGCGC	6	30187171	R
	TTCTTTTA	37	4	157682848	CGTGCCT	4	157902298	R
	AAAAGAA	37	2	150845309	CTCTCTTC	2	150553555	F
	TTTACTCC	37	21	46763239	GTTTGTTT	21	45587667	F
	TACTTTCC	37	4	15895695	CAGCCAT	4	15504793	F
	CGGCGAC	37	21	46812511	TTTGTAG	21	45636939	F
	ACAGAAA	37	4	71382958	CGCAAGC	4	71417547	R
	CTGAGCA	37	1	221335745	CGGCTCT	1	219402368	F
	TGGGAAC	37	8	119097956	CCACATG	8	119167137	R
	ACATGTA	37	6	30122432	CCTACGT	6	30230411	R
	CCCTGTTC	37	11	70336800	GAGATGT	11	70014448	R
	TTCGAAA	37	4	1192053	AGCAGCC	4	1182053	R
	TAGGGCT	37	13	27516202	ACACCTG	13	26414202	F
	CCTTGAA	37	10	118380345	GGCAGGA	10	118370335	F
Red	CTCTTCTC	37	6	29526376	CGGCAGT	6	29634355	R
Red	TCCCAGG	37	2	239997987	CGAGGAA	2	239662924	R
	AGTGATG	37	1	243050875	CGAAAAC	1	241117498	R
	ACCCAGC	37	19	55669872	CGGGGCT	19	60361684	F
	CTGAAAT	37	3	111852325	CTGTGGC	3	113335015	F
	GGCCGGC	37	6	101846707	CGTCAGA	6	101953428	F
	TAAAAGC	37	1	205579533	CGACCAT	1	203846156	R
	GTATTTTA	37	5	163786073	CGGCAAC	5	163718651	R
	CAGTGAT	37	19	52146598	GCATGTG	19	56838410	R
	AAACAGA	37	18	40696164	CTATGTTA	18	38950162	F
	AGGGAGC	37	7	38361199	CGCCCAC	7	38327724	F
	AAATTCT	37	14	64905375	CGCTGGG	14	63975128	R
	CCTGAGT	37	5	10513925	CGGGTGC	5	10566925	R
	GTCAAAT	37	12	130084035	CGGATTA	12	128649988	R
	CCCACGG	37	1	26074002	GACCTGG	1	25946589	F
Red	TGGAAGC	37	4	13300500	ATACAGT	4	12909598	F
	AATGAAA	37	14	94179847	GCTTGCA	14	93249600	R
	CTGGAAA	37	18	76498163	CGGATGC	18	74599151	R
	GCACCAC	37	10	94350577	TCTATTTT	10	94340557	F
	GAGGAAA	37	1	117487269	TGTGGTTC	1	117288792	R
	CACGTAC	37	22	18531028	GCACCTT	22	16911028	F
	CTGTGGA	37	2	240567630	GCCCTGC	2	240232567	F
	TACGAAT	37	6	135172385	CGGTAGA	6	135214078	F
	TTTCTTTA	37	14	50862509	CGGGTCA	14	49932259	R
	CTTAAAT	37	2	168244895	CGCTTTG	2	167953141	R
	CCTGGGG	37	5	308268	CGGCCTG	5	361268	F
Red	TGGAGTC	37	11	133900846	GCCGGCT	11	133406056	R
	ATAAAAC	37	6	35109722	ACTCGAA	6	35217700	F

	GCCACAC	37	11	61716821	CAGAGCT	11	61473397	F
Grn	TGGGGAC	37	10	129947807	CCTGACT	10	129837797	F
Red	TGTGAAG	37	5	178488100	AAATACT	5	178420706	R
	TTGGGGG	37	1	9224376	CGGCCCC	1	9146963	R
	AGAGACC	37	12	27627468	GAGAATC	12	27518735	F
	GAATTCC	37	15	34339800	CGGGAGC	15	32127092	F
Grn	CTGGCTG	37	11	126310323	CGGGCGC	11	125815533	R
	CCCGCCT	37	1	201123745	CCCTTCC	1	199390368	F
	GGCACAA	37	1	11207707	CGGATCT	1	11130294	F
Red	TCACGGC	37	15	40268777	CATGAGA	15	38056069	R
	CCTCCTT	37	10	51589507	CGTCCAC	10	51259513	R
Red	CAACCAC	37	22	32750777	CGCCACG	22	31080777	F
Grn	GGTGGGT	37	5	140176361	GCACGTG	5	140156545	R
	TCCCACC	37	17	53684393	CGGATGA	17	51039392	R
Red	GGACTTG	37	21	45198592	CAAGGGA	21	44023020	R
Red	GCTCATT	37	3	194573365	CGGACCC	3	196054654	R
Grn	GCACAGA	37	1	9341141	CGGGTCT	1	9263728	R
	TGGGCAC	37	5	179759	GCCATGA	5	232759	R
Red	AACGAGC	37	8	103750821	TGGGTGC	8	103819997	R
	TCACCAG	37	6	116381609	CGGGACT	6	116488302	R
	TCTGTCC	37	18	10032765	CGGAGAC	18	10022765	R
	CATCCAA	37	16	12059019	ATGAACT	16	11966520	F
Red	CGAGCCC	37	7	23245900	ACGCAGT	7	23212425	R
	GGGGGCT	37	17	79609161	GAGATAT	17	77219566	F
	GGGGAGC	37	6	1555389	CGAATCT	6	1500388	R
Red	GCGAGTC	37	18	76674571	CGCGGCT	18	74775559	F
	GAGGAAC	37	8	140926709	AAGGCGC	8	140995891	R
	GGGAGAC	37	10	48828106	CGCCGCT	10	48448112	F
	CTATCAA	37	6	147235033	CGCAGAC	6	147276726	F
Red	GTGTGGT	37	14	104625420	TGCAGAC	14	103695173	R
Red	AGGAGGA	37	7	12151356	CGCCCTG	7	12117881	R
Red	GGAGATC	37	10	135379215	CGCGCCT	10	135229205	R
	ACCCACT	37	13	107028903	CGGTGAC	13	105826904	R
	GGAGGAA	37	5	112882744	CGTGAAA	5	112910643	R
Red	TCGGCTC	37	6	42099392	TGGCCAA	6	42207370	R
	TCTTTTTT	37	2	20001490	TTTAACT	2	19864971	F
	CGGGGGC	37	5	140188119	TTCGCTG	5	140168303	R
	AACAGCC	37	5	75698461	CGCAAGC	5	75734217	R
Grn	CGAGTTC	37	10	131350999	ACTTCGG	10	131240989	R
	TGGTTTT	37	11	131934835	AACATAC	11	131440045	F
	GTACCGA	37	18	57634307	TGCATGC	18	55785287	F
	CGTCTCC	37	5	140710509	TAGGATG	5	140690693	R
	TTAAAAA	37	3	129132966	GGGTCTC	3	130615656	F

Red	TAAATCT	37	18	76751616	CGGCCAC	18	74852604	F
	CAGCGAT	37	8	8131077	CGCCCAG	8	8168487	R
	TATTCAA	37	6	126000798	CGCCAGC	6	126042497	F
	TGTGACC	37	12	122297080	CGGCTCC	12	120781463	F
Red	CACGCTT	37	20	25281332	AATGCTC	20	25229332	F
Red	CGGGGAA	37	4	77997182	CGGCTCC	4	78216206	F
Grn	CCACTTTA	37	11	61159649	TCGCGTG	11	60916225	R
Red	CCACCGA	37	6	22356972	AGGGAGA	6	22464951	F
Red	CGGGCCC	37	14	102422355	GTGCCGA	14	101492108	F
	AAAGCAT	37	2	33295138	CGCTGAG	2	33148642	R
	TGCTAAG	37	8	10657514	CGGCTCT	8	10694924	R
	TCAGGAT	37	3	159493194	ATGACCC	3	160975888	F
	GCCTTCCT	37	6	32633102	ATCCCTCT	6	32741080	F
	TTAGTAC	37	15	49012706	CCATATT	15	46799998	F
	CGCTCTG	37	16	10346074	CAGCCGC	16	10253575	F
	AATGCTC	37	3	138739900	AGAGGCC	3	140222590	F
	GCACTTTG	37	1	233248845	AATTACA	1	231315468	R
Grn	GAACTGT	37	16	55794595	TTCCAGG	16	54352096	R
	ATGTATTA	37	5	55289001	CGGGGAA	5	55324758	F
	CACAACC	37	12	55248207	CCAATCA	12	53534474	F
Red	CGCTGGG	37	11	14927549	CGTGCGG	11	14884125	R
Red	ATTGATC	37	12	114204091	ATATTAA	12	112688474	F
	CACATTTG	37	5	110899314	CGGTCTA	5	110927213	R
	AAGGTTT	37	10	3164740	CGCCACC	10	3154740	R
Red	CACATCA	37	15	100821466	CCGGCTC	15	98638989	R
	GGGGTCA	37	1	59986591	CGGTTCTG	1	59759179	R
Red	AAACAAA	37	1	91300215	CGCGTGG	1	91072803	R
	GTTGAAG	37	14	95973710	CGGCTGG	14	95043463	R
	TTTCAGTT	37	12	115118777	GAAAGTC	12	113603160	R
	GCACACC	37	14	105344761	CTGGGCG	14	104415806	R
	GAGCAGA	37	3	133502952	GGACTCC	3	134985642	F
	ATGACCT	37	3	98534089	AGGTTTC	3	100016779	F
	AATCAAA	37	7	41743001	ATGTCTCA	7	41709526	R
Grn	GTGGTGG	37	2	172967431	GAAAGAC	2	172675677	F
	ATATCTTG	37	16	51360402	CGGGATT	16	49917903	F
Red	GGCTCTG	37	10	129947858	CGACCCC	10	129837848	F
Red	AGGGCAC	37	2	10302391	CGCCCGT	2	10219842	F
	TTCCATTA	37	14	22279816	CGTGATC	14	21349656	R
Red	CGCGCTT	37	17	40192112	CGCGCAC	17	37445638	F
Red	AAGGGGC	37	20	34190258	AGGGGTA	20	33653672	R
	CTGCAGT	37	16	3317428	CGTTGCC	16	3257429	F
	GACCCCA	37	10	13544863	CGGGGTC	10	13584869	R
	AATATAT	37	10	47964834	CGAGAAC	10	47484840	R

	GTGCTGG	37	11	94307971	CAGGCAT	11	93947619	R
Grn	TTCCCAC	37	20	32856846	CGCCCTG	20	32320507	F
	TTGGTAA	37	5	73234433	CGAGCAC	5	73270189	R
Red	ACCCAAT	37	3	170626822	CGCGAGT	3	172109516	R
	CCTGGCC	37	5	71475356	CCCAGTG	5	71511112	R
	GAGGGAT	37	7	76145632	GAGTGCA	7	75983568	F
	CCACAGC	37	6	157264715	AATTGTA	6	157306407	R
Red	GCGCTCC	37	15	95870262	AGGAGGC	15	93671266	R
	GAAATTA	37	17	818890	CCAGCCC	17	765640	F
	GTGAGGA	37	12	106460704	CGGGGCC	12	104984834	F
	CAGATCC	37	7	44229319	CGGGATC	7	44195844	R
	GGTCTTT	37	16	50896074	CGCATAA	16	49453575	R
	AAATCAC	37	8	20080666	GTCACTA	8	20124946	R
	CGTTCGC	37	6	32120955	TACAAGC	6	32228933	F
	TTTTACC	37	6	111659754	CGGTGAA	6	111766447	F
	AATTTGC	37	1	235292369	CGCGTTA	1	233358992	F
	GCCGGGA	37	11	7928236	CGGGGAC	11	7884812	F
	TTGAGGT	37	6	29868207	GTAGTCT	6	29976186	R
	AAATCCC	37	5	55460876	AGGCAAA	5	55496633	R
	GAGATGA	37	6	33158020	ACTCTGA	6	33265998	F
	AACCGCA	37	6	32552152	TCCGTCC	6	32660130	R
Grn	CGGAGAC	37	17	48263159	GCCCTGC	17	45618158	R
	CAATGTA	37	17	79787551	CGAGGGT	17	77380840	R
	AGGGTTA	37	3	129147846	CGGGGCA	3	130630536	R
	CTCGCAG	37	12	54383678	CGTCTAT	12	52669945	R
	TCATGCA	37	6	34524698	CCTTTGG	6	34632676	F
	TAAAATG	37	3	24782112	AAGTGAA	3	24757116	F
	CAACAGT	37	12	27901072	GGAGAAC	12	27792339	F
	CACTGTC	37	6	29922522	AAGATTG	6	30030501	F
	GGATCCT	37	5	43802496	ACATGCT	5	43838253	F
Red	AATCAGT	37	17	79652024	CGCCTTT	17	77262429	R
Grn	GGGAGGA	37	12	115813214	GGGGCTC	12	114297597	R
	TCGCTAC	37	1	21023132	GGTGACC	1	20895719	R
	AAGGGCT	37	13	113660298	TCACGGA	13	112708299	F
	TTGTACT	37	5	2642995	TGTGAAG	5	2695995	R
Grn	AGCAGCT	37	6	28983079	GCAGGTT	6	29091058	F
	TGTTGGG	37	3	185476452	TGTTAGC	3	186959146	R
	CTCTACC	37	9	115851255	ATCCTCC	9	114891076	F
	CGCGGAC	37	7	129410227	CAGGATC	7	129197463	F
	TTTGACG	37	8	61197710	CCTTGAA	8	61360264	F
Red	AGAACAC	37	6	30038998	CGACCTG	6	30146977	R
Grn	CCGCCCT	37	5	140553150	GCATTCT	5	140533334	R
	ACCAACC	37	1	85755348	CGCCTCA	1	85527936	F

Red	CCCAGGC	37	12	54389477	CGCTCCG	12	52675744	R
Grn	TTATGGT	37	14	93698053	CGCCGAT	14	92767806	R
Red	AGGGGTC	37	7	51081446	AGCGGTC	7	51048940	R
Red	AGGTTCT	37	3	10149918	GGCCGCC	3	10124918	F
	AGGTCTT	37	3	53380856	CGGAAA	3	53355896	R
	GGGCCGT	37	5	135416594	CGGGGTT	5	135444493	F
	CGTATCA	37	12	15039560	CCTGCCA	12	14930827	F
Grn	TGGGTGG	37	7	1577003	CGGTGAC	7	1543529	R
Red	CGTCGCC	37	5	140529775	CGACCCT	5	140509959	R
	GACAAGC	37	11	71010448	TTTAAGC	11	70688096	R
	CGCGTGT	37	10	134420036	TGCTGGG	10	134270026	R
Red	GCCGACC	37	7	157867015	TCTGCTG	7	157559776	R
	GCCTCTC	37	13	113585948	CGCTCGT	13	112633949	R
Red	AGGATTC	37	X	19141251	GATGGTT	X	19051172	R
Red	CCCAGGC	37	11	65685316	CCAGCTT	11	65441892	R
	AAGGCTC	37	11	121699840	CGTGGAC	11	121205050	F
	GTCTGTT	37	1	7432614	CTGATCA	1	7355201	R
	TTGCGTT	37	12	122012243	CACCCAG	12	120496626	R
	CACCTCC	37	6	29701563	CGCTGTA	6	29809542	R
	TTCCAAC	37	6	108145930	AGTTTTC	6	108252623	R
	ACATTAA	37	5	166711687	CGTCTGA	5	166644265	F
	GCGTTAT	37	6	4352453	CGGGGGC	6	4297452	F
	CTAAATG	37	5	57073212	CGGGGGC	5	57108969	R
	TGAGAGC	37	10	114116449	CGGGGAA	10	114106439	R
Grn	GCCTTTT	37	13	100218136	CGGCACC	13	99016137	R
	AAAAAGC	37	18	33485594	TCCTCAT	18	31739592	F
	TCCTCCA	37	11	63529520	CCCTACT	11	63286096	F
	TGTCATC	37	11	86643955	TCCCTCT	11	86321603	R
	AGTTTTA	37	13	99974673	GGCTCTT	13	98772674	F
	CAGAGGA	37	4	128791896	AAGAAGA	4	129011346	F
Red	GGCTCTT	37	10	135259860	CGCGCCC	10	135109850	F
	TGTAGTG	37	16	81518253	CGGGTCT	16	80075754	R
	ATCTATA	37	14	22361282	CACAAGC	14	21431122	F
Red	CAGGGAC	37	7	55517132	GTAGTGC	7	55484626	F
	ATTGTTT	37	12	107350911	TTGAAAC	12	105875041	R
	CCCCCGT	37	10	3509710	GGGGCAC	10	3499710	R
	CCTCTGC	37	11	1048635	CGGAAAC	11	1038635	R
Red	GCACTTT	37	2	568198	AACAGCC	2	558198	F
	AGCTGGA	37	11	73577813	TCACCTT	11	73255461	R
Grn	CGCAGGT	37	12	124968359	CGGCTCT	12	123534312	F
Red	CGTTGTG	37	15	45493438	AGCAACC	15	43280730	R
Red	CTCGAGC	37	21	37442385	CGCAGGT	21	36364255	F
	AAAGGGA	37	3	109735618	CGGAACT	3	111218308	F

	GCTGCAG	37	6	30228431	CGGGCTG	6	30336410	F
Red	TCCTAAA	37	12	125149524	CGATGGC	12	123715477	F
	CATACCT	37	16	75149501	CGTGGGA	16	73707002	F
	GTCAGAT	37	1	222544693	CGGTCAC	1	220611316	F
	CGTTGTA	37	13	84453665	CATTGTG	13	83351666	R
	TGGAAAA	37	11	68776397	AGAGCAC	11	68532973	F
	TGTGTGT	37	8	11994639	GTGTGCC	8	12032048	F
	CGCTGCA	37	6	29911366	CGGGCTG	6	30019345	F
Red	CAATCAT	37	19	17905626	GCTAGGC	19	17766626	F
	TATCTCA	37	12	9885998	CGCCAGA	12	9777265	R
Red	GGCACCC	37	16	31551613	GGGGACT	16	31459114	R
	TGCAGTT	37	6	4370460	CGACCTT	6	4315459	F
	AGAGAGA	37	16	3334273	CGGGGGC	16	3274274	R
	GCCGGGC	37	8	1792758	CGGCTCC	8	1780165	R
	CTTCTCT	37	2	242101289	AGAAGAA	2	241749962	R
	AGCCAAC	37	8	97803770	CGGTGAC	8	97872946	R
Red	GCAGTCG	37	17	80866063	CGCAGCC	17	78459352	R
	CCTCAGC	37	16	2749792	CGGGCCA	16	2689793	R
Red	TACATCA	37	17	113262	CGCGTCA	17	113262	R
Red	GGGCTGC	37	7	1787831	GTCTGAG	7	1754357	R
	TGAATTC	37	3	18877557	CGCTGAT	3	18852561	R
Red	CCTGTAA	37	22	37493863	CGGTGGC	22	35823809	R
	CCCTTTC	37	3	151178760	CGAGCCC	3	152661450	R
	GCACCAG	37	17	48273001	CGGGGCC	17	45628000	F
Red	GGGGAGC	37	21	44105474	CGCGGGC	21	42978543	F
	ATGTTTG	37	7	133974228	TACACAG	7	133624768	R
	ACTGCCT	37	14	102975405	TTTAACT	14	102045158	F
Red	TAGAGCT	37	11	67141792	AGGCGCT	11	66898368	R
	AGGATGA	37	3	192577951	CGGCCAT	3	194060645	R
Grn	AGCCCCA	37	17	46507777	GAAGGAT	17	43862776	R
	GTAGAAT	37	16	73102243	AATCCAT	16	71659744	F
	TTCACGC	37	X	40036466	CTCCTCC	X	39921410	R
Red	GAACTGC	37	15	27111572	TAGGACC	15	24662665	R
	GATAATC	37	11	66104993	GCCGAAC	11	65861569	F
	CTTCATT	37	8	91681699	ATAGCTT	8	91750875	F
Red	CCACCCT	37	X	48776486	TTTCAAG	X	48661430	R
Red	ATGCTAA	37	11	36383991	CGCCCAC	11	36340567	R
	GCCCCTG	37	17	79524882	CCTGTTG	17	77135326	F
	AGCTCCC	37	17	52981853	GATTGTT	17	50336852	F
	GAGCAAC	37	17	78725375	TGAGATG	17	76339970	F
	GGTTCAC	37	19	6362787	GTGAGCC	19	6313787	F
	CTGCCTG	37	8	1843398	CGGAGTC	8	1830805	R
Red	GAGTTAG	37	19	52206691	CGGAGAT	19	56898503	R

Red	GTGCCTG	37	6	761284	CGCCCAG	6	706284	F
	GACTTTC	37	6	112005207	CGGGGAT	6	112111900	R
	ATTTGAG	37	2	116601613	CGTGTCT	2	116318083	F
	AAATAGA	37	3	42306150	TAATCCA	3	42281154	F
Red	CGGAGAC	37	19	49200180	CGGCCCC	19	53891992	R
	CAAGCAC	37	10	65682643	CCATTCA	10	65352649	R
	GCTGATA	37	11	75210723	CGGGAAA	11	74888371	F
Red	ACTGCAC	37	2	161504834	CGCGGCC	2	161213080	F
	CCATCTC	37	13	36050158	CGGAAAC	13	34948158	F
	CCTCCAA	37	7	82136223	CGGGCAT	7	81974159	F
	TAGAAAC	37	12	54377550	CCTTGGA	12	52663817	F
	AAAAAAA	37	1	224698362	ACCTGCC	1	222764985	F
	ACCTGGG	37	2	11728079	CCACCTC	2	11645530	R
Grn	GTGGACC	37	20	42788699	CGCAGCC	20	42222113	R
	ATTCTAA	37	18	722818	AAAAAAT	18	712818	R
	ACAGCTC	37	7	51539678	AAGTTCA	7	51507172	R
	AAAAGCT	37	19	58952249	CGCCAAA	19	63644061	F
	CCCCTGG	37	3	11178593	CGGGTGA	3	11153593	F
Grn	AGTGACC	37	20	2731371	TAGCCTC	20	2679371	R
	CCATCAC	37	3	66475150	CGATGGT	3	66557840	F
	GCAGGCC	37	3	193988639	CGGGGTT	3	195471333	F
Grn	GATATTG	37	10	729226	AGAAGGC	10	719226	R
	CTGGCAT	37	1	218575437	CGAGAGT	1	216642060	R
	ACTTCAC	37	3	75834271	AAAGAGA	3	75916961	R
	CCTGTGT	37	4	7373065	TGGATCG	4	7423966	R
	GCAAGAT	37	15	25490008	CCCACCA	15	23041101	R
	GAGTTCT	37	11	1001560	TTTTCAG	11	991560	R
	TAAAATC	37	5	140588424	CGCAGTT	5	140568608	R
	GCACGCC	37	14	77041277	CGCTGCA	14	76111030	R
Red	GACTTTA	37	5	14380323	AGCAGAA	5	14433323	R
	GGCCGGC	37	12	103352694	GCCGAGC	12	101876824	F
Red	CTTGATG	37	6	28557765	CGCTCAC	6	28665744	F
	CATTGCT	37	1	19291857	CGGCAGT	1	19164444	F
	GCGAGCA	37	2	238647755	CGCACGC	2	238312494	R
	AAATATA	37	11	128568943	TTACTGA	11	128074153	R
	TGGCATT	37	3	159852976	ATTCAAT	3	161335670	R
	GGGCGTC	37	11	58944103	CGGCAGT	11	58700679	F
	ATATATT	37	8	69348660	CGATACT	8	69511214	R
Red	CCCGCAG	37	5	140731399	CGAGCTC	5	140711583	F
	AGAACCC	37	12	75785295	CGGACAC	12	74071562	F
	ACACACA	37	7	158152141	CGGGGGC	7	157844902	F
Red	CAAAGGC	37	5	25190850	GTCCTGG	5	25226607	R
	AGCCTGC	37	5	149493243	TCTCATT	5	149473436	F

	CGTCTGC	37	13	113624866	CGGAAA	13	112672867	R
	TAGAGAC	37	4	177262070	CGTGATA	4	177499064	F
	GCCTGAA	37	10	93044533	GTCTTAA	10	93034513	R
Grn	AAAGGCC	37	12	115103351	CGCCTGC	12	113587734	R
	CTCAGCC	37	11	11642133	CGGGTGA	11	11598709	R
	GTGGGTC	37	3	115524190	CGGAAGA	3	117006880	R
Red	GACTGGT	37	4	110524473	CGGCTCC	4	110743922	F
	GAAGGTT	37	6	135072384	CGGCCCA	6	135114077	R
Red	AAGAGCA	37	1	149294708	CGCCTCA	1	147561332	R
Red	GCCATTC	37	6	31238788	TCAAGGC	6	31346767	R
	GGCCGGC	37	19	3655825	TAGGAGT	19	3606825	F
	CAGCCCG	37	2	241562085	CGCCACC	2	241210758	R
	TTTGTGA	37	10	80312528	CGGGCCC	10	79982534	F
Red	GGGTCCA	37	7	112032599	ATCACTC	7	111819835	F
Red	GAGCCGC	37	X	30907432	TTGCCAA	X	30817353	F
	AAACACT	37	17	25773641	CGGCTTC	17	22797768	F
	TTGAAAA	37	1	243877447	ACACAGT	1	241944070	R
Red	AGCAGCT	37	2	162197875	AGAACTC	2	161906121	F
	TTCTACC	37	6	106546824	ACTCAAA	6	106653517	R
	AAAAACT	37	2	69812277	CGAGGAT	2	69665781	F
	CAAGGCA	37	15	83949772	CGGCAAC	15	81740776	F
Red	TTGAGAA	37	X	48660379	CGCCTCC	X	48545323	R
	GCTCACA	37	4	2305734	GGCGGGA	4	2275532	R
	GCCATCA	37	2	1886127	TAAAGAA	2	1865134	F
	GCCACAT	37	8	13209750	CCTTTAC	8	13254121	F
	CAGTTGA	37	8	41393714	AGGTTGT	8	41512871	R
	TTGGCTT	37	11	77271314	CGTGGCT	11	76948962	F
Red	GGTCCCC	37	11	400385	CGGGCTG	11	390385	F
	TGGTTAC	37	6	27521041	TATTTAC	6	27629020	R
	TGTGATT	37	1	169599754	GTGCCCA	1	167866378	R
	GTTGAAG	37	11	119023062	TAAACCA	11	118528272	R
	GTATGAC	37	2	178109973	CATCTCA	2	177818219	R
Red	GGCCGGC	37	1	205818956	GTGCGTG	1	204085579	R
	TCTTATC	37	1	31282519	AAGATAA	1	31055106	F
	ACCTCAT	37	2	97004840	CGCCCAG	2	96368567	F
	TGTGCTT	37	2	121442251	ACATTTT	2	121158721	F
	TCCCGTG	37	11	132107476	CTCCGTC	11	131612686	F
	CGCCCAC	37	X	73834683	CGCAAAT	X	73751408	R
	TCTTTTTA	37	8	37005572	CCGATGC	8	37124730	F
	ATCCATT	37	7	152345227	AAGAAAT	7	151976160	R
	ACAGAAC	37	10	130279674	CGCAGGT	10	130169664	F
	GGGGAGC	37	22	49840886	CCACCCC	22	48226890	R
	TATGGTT	37	2	17982866	CGGGACC	2	17846347	R

Red	CAAGTTG	37	X	129244725	AGAGATC	X	129072406	R
	GACACTC	37	4	189359992	CGACAGC	4	189596986	R
Red	GAGGCTC	37	X	2886295	TGGAAGA	X	2896295	R
	ACAAATT	37	7	141536997	CGAGAAT	7	141183466	R
	CCACAGC	37	1	42248998	GGCTGGC	1	42021585	R
	CCAAGAA	37	7	34911385	CGGGACA	7	34877910	R
	TTCCCCT(	37	6	27791827	CGCCAAC	6	27899806	F
	CGAAGCC	37	17	22193895	TCTCTGT(	17	22118022	F
	CACAGTG	37	13	52701588	ACTCAGC	13	51599589	F
Red	GCTGGAC	37	15	35086890	GGGTCCC	15	32874182	R
	ACATTTT	37	2	47604176	CGCGTTA	2	47457680	F
	ATGAGCC	37	2	36815511	GCCCAGC	2	36669015	R
	CCACCCA	37	6	31275875	CGGCTCA	6	31383854	R
Grn	AGATCAT	37	5	139088815	CGCGCCC	5	139068999	R
	GAATCAC	37	11	133917375	CGGCCCT	11	133422585	R
	CCTTTTT(	37	4	114366508	CGGATCA	4	114585957	F
	TGGGGTC	37	6	4358810	CGGCCAC	6	4303809	R
	TTCGTAC.	37	4	152597659	AAACAGC	4	152817109	F
	GGCTACT	37	6	55761410	ACCTGAG	6	55869369	F
	TGAGTAA	37	1	24921731	CGGGTAT	1	24794318	F
Red	ACGAGGC	37	17	32964596	CAGCGGC	17	29988709	F
Grn	GGCGCCT	37	2	24397845	TTTCCCG(	2	24251349	R
	AGCCAGC	37	12	31272112	CCACCTC	12	31163379	R
	CTGCCAG	37	6	33050124	CGGGCCT	6	33158102	R
	AATCACG	37	13	29132547	TCCTTAT	13	28030547	F
	CATTGCA	37	8	857079	CGTTTGC(	8	847079	R
	CCATAGA	37	7	24561256	CGAGCCT	7	24527781	F
	GCCAGCA	37	8	54557599	TCCTCTA(	8	54720152	F
	CTGGCGC	37	5	80257322	CGGGGCT	5	80293078	F
	CCCCTCC(	37	7	94953956	CGGTCAG	7	94791892	F
	TCAAGTT	37	2	80459300	CGATGTT	2	80312811	R
	ATGCTCT(	37	22	17680561	ATCGCCA	22	16060561	R
	CAAAGCA	37	11	2847019	TCATTCA(	11	2803595	R
Red	CACACAC	37	7	158050952	GGGCTGC	7	157743713	F
	ATTTTCT	37	10	128846231	AGGGAAA	10	128736221	F
	ATAACTA	37	5	86709409	CGAATAC	5	86745165	R
	TTTGCAG	37	2	189834269	CGCCTGA	2	189542514	F
Red	ATGAGGA	37	17	38183730	ATGTCAA	17	35437256	R
Grn	CCGCACG	37	X	100306958	GGGAGG(	X	100193614	F
	GTCCTTA.	37	6	166259938	GAAATCC	6	166179928	R
	GACTGCG	37	5	140052701	AAGGGGC	5	140032885	R
	ACAGTCC	37	7	786861	CGCATCC	7	753387	F
	TGATCAG	37	6	32186244	CGGGACT	6	32294222	R

	TGCGTTG	37	1	1998716	GTGCAGC	1	1988576	R
	AGGCTCT	37	6	32725332	CGGTAAC	6	32833310	F
	TTTACAG	37	10	42673902	CGCTATC	10	41993908	F
	GGCCAAC	37	6	32485396	GAGCTGA	6	32593374	R
	CGAGGGC	37	14	56233068	CATCTCT	14	55302821	R
	CCAGTAC	37	15	34726650	GTTCCAC	15	32513942	R
	ATGACTG	37	2	197940256	CGAACGT	2	197648501	F
	TGAGAGT	37	6	149336332	ATTTTCT	6	149378025	F
	GACGACA	37	20	60745058	CGGCCCC	20	60178453	R
	CTCTTAC	37	5	135384080	CGGCAAA	5	135411979	R
	ACCATGA	37	2	114715532	TAAAGGA	2	114432002	R
	TGCTCAC	37	3	109537321	CGCATGT	3	111020011	F
	ACTTCAG	37	11	122060265	CGCAGCC	11	121565475	R
	CCAAGGT	37	7	2764129	GCCTGGA	7	2730655	R
	CCCCCCA	37	5	108820864	CGCCATC	5	108848763	R
Red	AGCGATC	37	5	110406107	TAGGAAC	5	110434006	R
	ATAAACC	37	7	76221076	AGCTGTG	7	76059012	F
	GAAGAAC	37	5	31796726	CGGAAGC	5	31832483	F
	GTTGTCA	37	1	79350918	CGGCACT	1	79123506	R
	GGTGACA	37	17	61514690	CGGCCCA	17	58868422	F
	ACATGCC	37	6	32608879	CGCACAC	6	32716857	F
Red	ACCTTTG	37	2	114259669	TTTGCTA	2	113976139	F
	AAGCCTA	37	11	126507786	CATGCTC	11	126012996	F
	TCCCAGC	37	7	149964288	CGGCTTC	7	149595221	R
	TCTGAAG	37	10	35464474	TCCAAGC	10	35504480	F
	GGATGCT	37	11	26743403	AACTTTT	11	26699979	F
Red	TGCTTAC	37	1	91300231	CTGGCTG	1	91072819	R
Red	CAGGAAC	37	7	64403162	CGGCTCC	7	64040597	F
Red	GAAGCCA	37	1	40105667	AGGCAGC	1	39878254	F
Red	GCAGCCC	37	5	140188392	CCACGAC	5	140168576	R
	CCAACTT	37	3	114566265	ATGCCTC	3	116048955	R
	TTTAAAG	37	1	92208474	AACATTA	1	91981062	R
	GACCCAT	37	1	64287452	TTAAGTC	1	64060040	F
	TCAGAAC	37	3	160936577	GTAGGCT	3	162419271	R
	TGATTAG	37	8	2082244	CTTCCGA	8	2069651	F
	TTCTTTT	37	8	129552914	TAAGCCT	8	129622096	F
Red	TTACCGC	37	3	123103936	CGGCACC	3	124586626	F
	GGTAGCA	37	10	28970998	CTTATCA	10	29011004	R
	CCCGTGG	37	2	232507842	CGTGTTC	2	232216086	R
	GGTCAGA	37	11	130476866	CGGGGAC	11	129982076	R
	GTTTTATC	37	5	67466813	CGTAAAA	5	67502569	R
	CAGGAGC	37	19	48049039	CGCCCCC	19	52740851	R
	GTTTCTT	37	1	174933574	CGTCATT	1	173200197	R

	AGCATTG	37	15	53100108	CGGCTCC	15	50887400	R
	TTATTTA	37	2	207873436	ACCATGG	2	207581681	F
Grn	CGACTCG	37	6	170452270	GATGGA	6	170294195	R
	CCCCACC	37	2	778668	CGACGTT	2	768668	R
Red	ACACCTG	37	13	24902500	GGTGCGT	13	23800500	F
Grn	GACACCC	37	5	140502918	GAAAACC	5	140483102	R
	GATAACT	37	10	7896363	CGGCTCG	10	7936369	R
	GCCGGGC	37	7	43288771	CGGCCCG	7	43255296	F
	TTTTTAA	37	3	44802566	AAAGCAT	3	44777570	R
Red	CACGAAA	37	6	27483069	AGGGGAC	6	27591048	F
	AGGCAAC	37	7	133247477	TTGTAAG	7	132898017	F
	TGGGAAA	37	6	144417471	GAATGAC	6	144459164	R
	GGCAGAC	37	4	3371566	CGGGATC	4	3341364	R
	CCTTTTC	37	6	148914562	GTTTGGG	6	148956255	R
	TGTTCCT	37	17	67536299	ATTTGCA	17	65047894	R
Red	CCAAGTG	37	6	166259959	CACGCAA	6	166179949	R
	GCCACCA	37	2	218707806	CGATTCA	2	218416051	R
Grn	GGGAACC	37	2	241689985	GCTGCCA	2	241338658	F
Grn	GGCCAGA	37	2	48647546	CGCCTGG	2	48501050	F
	TTCTCAT	37	6	14037614	CGGGTTG	6	14145593	R
	GACCGAT	37	5	1594843	CGGGGCT	5	1647843	R
	CGATAAC	37	5	71475111	CGGGGAA	5	71510867	F
Red	ATTATGC	37	3	192289245	TTCAGCG	3	193771939	R
Grn	GCGCCCC	37	7	151411526	GCCTAGG	7	151042459	R
Red	TCAGGAA	37	17	77245327	CGGTGGC	17	74756922	R
Grn	GGTCCCT	37	3	42947565	TTGCTCT	3	42922569	F
	AGGTGGC	37	7	143130320	CGCTACC	7	142840442	R
	CAGAAAC	37	11	70266172	TGCTAAA	11	69943820	R
	CTCACAC	37	3	122712138	CGGCTGT	3	124194828	F
Grn	CCTACTC	37	X	46618639	CGCAGGA	X	46503583	F
	AAAGCGC	37	13	111281037	CGGGAAC	13	110079038	F
	CAAGCCT	37	3	124601778	CGGAAGC	3	126084468	F
Red	TCTGGTG	37	13	112985463	CGGACAC	13	112033464	R
	AAATATG	37	3	29456667	CGAGGGC	3	29431671	R
	TTTCTCC	37	22	45806309	CGCCCAG	22	44184973	R
	GCACGGC	37	13	80510936	CGCTGTA	13	79408937	R
Red	CAGTGAC	37	4	14858290	CGGAGAC	4	14467388	F
	TTAAAAT	37	2	121274655	TACTTTC	2	120991125	R
	TGGAAAC	37	6	152126337	CCTAGAA	6	152168030	R
	CTATGGT	37	11	102401523	CGAGGCT	11	101906733	R
	ACCATAC	37	13	40108007	CGGGTTC	13	39006007	R
	GAGATCC	37	6	26124704	AGCAGTT	6	26232683	F
Red	ACTACCC	37	19	10077935	ACCTACA	19	9938935	R

Red	CCCATGA	37	7	151423659	CTCTGTC	7	151054592	R
Grn	GACTGCA	37	X	70315718	TGTTTTG	X	70232443	F
	CTCACAG	37	10	118931160	TCTTATT	10	118921150	R
Red	ATTTGGA	37	10	31108190	ATGTGAC	10	31148196	F
	AGAAAA	37	5	140552505	CGGTTCG	5	140532689	R
Grn	CCCCCAA	37	17	79122455	CGGCACA	17	76737050	F
	CTAACGC	37	6	143224179	CGGTTTA	6	143265872	R
	CACACCT	37	13	92426491	CGGGCAA	13	91224492	R
	TCAGAGC	37	Y	22917782	GGACTTT	Y	21327170	F
	TGCTCTT	37	1	17308158	TTGTCAG	1	17180745	R
	TTCCATT	37	2	30669863	CGGAGGT	2	30523367	R
Red	GGGCTCG	37	4	798611	CGGCCTC	4	788611	R
	GACTCCT	37	5	71541917	GCCAAAT	5	71577673	F
Red	CAGCTCT	37	19	18888081	CGTTGAG	19	18749081	F
	GACATAT	37	8	26481696	TTTCTTT	8	26537613	R
	ATTTCCA	37	7	18653918	TCTTGCA	7	18620443	R
	GTCTGAA	37	6	33083457	CGGCAGC	6	33191435	R
	TCTTTAA	37	4	124534980	CGTGCAA	4	124754430	F
	CATGATG	37	4	3853774	GCAGGGC	4	3823572	R
Red	CTGGAAA	37	X	46306513	CGCCACG	X	46191457	F
	GCGTGCC	37	19	22700869	CCTGCGG	19	22492709	R
	GGGCAAT	37	4	83766494	CTTTGCA	4	83985518	F
Red	CCGCGGC	37	5	140554081	CGACAAC	5	140534265	F
Grn	TTTCTTC	37	X	20285083	CGCCCGC	X	20195004	F
	TGATTTC	37	3	171759519	CGGGCAC	3	173242213	R
	GCCCTTG	37	7	158106772	GGCACTG	7	157799533	F
Red	CGCTCTG	37	19	55417496	CGCCCGG	19	60109308	F
	CAACTGT	37	7	63491010	CGCCACA	7	63128445	F
	ACCCCAA	37	16	20364284	TGACATT	16	20271785	R
	AGCTTGC	37	Y	15863901	ATACGCA	Y	14373295	R
	TTCCACT	37	7	158377440	CGGCTCA	7	158070201	F
	CCCGGTG	37	8	3086479	GCTTGAG	8	3073886	R
	CCATGTG	37	1	103343469	CGCTGCA	1	103116057	R
Grn	GCGGAGC	37	1	207842917	TGCTGGC	1	205909540	R
	AGTTTTC	37	13	100394412	CGGCTTC	13	99192413	R
	CATTCCA	37	6	32577714	CGGTCAC	6	32685692	F
	CGTCACG	37	12	123259751	TTCCTTA	12	121825704	R
Red	AAACGAA	37	Y	9340946	CGAAGAC	Y	9950946	F
	AAGCTAC	37	2	13863069	AGACATC	2	13780520	R
	GGCATTG	37	1	12120304	CGCTGGC	1	12042891	R
	GCTCAAA	37	21	46077562	TTATTGC	21	44901990	R
	TTGTGTC	37	4	18580125	CGAAACT	4	18189223	F
	AGAGGAA	37	6	32822857	TCTAACT	6	32930835	R

	CCATGGG	37	10	132944904	CGCCCGT	10	132834894	F
Grn	GTTGTTC	37	17	77922775	TGTCCGG	17	75537370	R
	TTGCCCT	37	5	78281827	TTAGTGT	5	78317583	F
	GTCAGCT	37	5	126408806	GGCTCCC	5	126436705	R
	CACTTGC	37	4	9476719	GTCCACA	4	9085817	F
	CCCCAAG	37	1	221055964	CGGAGA	1	219122587	F
	TTCAGAA	37	12	106215054	AGCAGTA	12	104739184	R
	CTAAACC	37	4	106137048	CGGCCTG	4	106356497	F
	TGGGGA	37	1	221058805	TGATGCC	1	219125428	R
	GCGACCT	37	7	2728288	CTTCCCG	7	2694814	R
	GTGGGCC	37	2	233216150	TTGGCCC	2	232924394	R
	GTTTAAC	37	16	85879145	CGCTGAA	16	84436646	F
	AATTTTC	37	15	90115219	CGGGAGC	15	87916223	R
	GTTACCA	37	3	152215049	CGGGCA	3	153697739	F
	GGGCGCC	37	19	2900938	CAGCTGA	19	2851938	F
	CTACTAA	37	15	57277634	TGAGTCA	15	55064926	R
	TGGCCAT	37	12	50641260	CGCCTGA	12	48927527	F
	GTAAAAC	37	1	176521193	CGTACAG	1	174787816	R
	TGTGTTA	37	1	55117812	ACAGCA	1	54890400	F
	CGACCAC	37	10	15600165	TTTCTTT	10	15640171	F
	CCGCCTG	37	1	110230473	GTCCCAG	1	110031996	F
	TTCATAC	37	10	88395453	CGGGGA	10	88385433	F
Red	TGGAGCA	37	17	78935070	CCTGGAG	17	76549665	R
	TCGTATA	37	15	45493416	CGGCATT	15	43280708	F
Grn	CGTCTTT	37	17	25290204	AAAGGCC	17	22314331	F
Grn	AGAGACC	37	2	98928898	CCTTCCA	2	98295330	R
	GCCTTCT	37	8	61863697	CGGGCCA	8	62026251	R
Red	CGGATGT	37	7	70200532	CGGAAGA	7	69838468	R
Red	GTAGGGC	37	6	30038712	CATTTTC	6	30146691	F
Grn	GAGAGAC	37	4	58060773	CGGGCCA	4	57755530	R
	ATGAAGA	37	2	216982485	TTTGGTG	2	216690730	R
Red	AGGAATC	37	1	203876248	AATGTCT	1	202142871	F
	GAGCTAA	37	1	119960007	CATCAAA	1	119761530	R
	TGGCTCT	37	10	80952606	CAGGGCC	10	80622612	F
Grn	TTAGCCC	37	6	90597340	TGGCAGC	6	90654061	R
Red	GATTCAA	37	19	38040009	CGCCCAT	19	42731849	R
	CCAGCGC	37	5	9547468	CAGCCAC	5	9600468	R
	TGTGGAA	37	10	124319733	CGGAGAC	10	124309723	R
	TATCAAC	37	5	131892242	CGCACAT	5	131920141	F
	TGGAACA	37	10	121146289	CGAGAAC	10	121136279	R
	TTCTTAC	37	5	8621127	CGACATG	5	8674127	R
Red	GAGCGGA	37	X	19002577	AGGGACT	X	18912498	F
	TCAGCCA	37	2	168674479	GGCAAAC	2	168382725	F

	TCTTTCA	37		20	3202228	CGCCAGC		20	3150228	R
Red	GACCCCC	37		1	228756789	CGCCTTT		1	226823412	R
	ACAAGCT	37		7	15601624	CTGTACT		7	15568149	F
Red	AGGGCGA	37		5	140230326	CGACCAA		5	140210510	R
	TCCCTAT	37		18	30349476	CGCAAAC		18	28603474	R
	ATATGTT	37	Y		2807288	CGGCAAA	Y		2867288	R
	GGAAGGA	37		2	202817042	CGGTCCC		2	202525287	R
Red	AGAGGCT	37		4	140201433	GATGGTG		4	140420883	F
	TCTTGGC	37		6	29981274	TGGTTAA		6	30089253	R
	CCAGTCT	37		22	46459890	GAGCTAA		22	44838554	R
	AATTCCA	37		3	10202595	CGGCTGC		3	10177595	R
Red	CGTCACA	37		19	58566643	TGAAGGC		19	63258455	F
Red	CTCCCTG	37		7	96655798	GCGGTCT		7	96493734	R
	ATAAACT	37		4	1202898	CGGCTCT		4	1192898	F
	GGTTCTG	37		1	115397597	GGAGCCA		1	115199120	R
	CTGGGCT	37		19	56269246	CGTTCAA		19	60961058	F
	TGGAGAC	37		11	61281763	CGGGGCT		11	61038339	F
	TTAGCCC	37		3	55232663	CGGCTTC		3	55207703	R
Grn	AGCGGGC	37		14	101175970	CGCCCCG		14	100245723	F
	AGGCCTA	37		4	184060895	CGGGTGA		4	184297889	R
Grn	GAGGGGA	37		5	2205553	GACGCCC		5	2258553	R
	GTCATGT	37		11	115062924	CGAACAT		11	114568134	F
	ACCTCCA	37		14	86088124	ACCAAAT		14	85157877	R
Grn	TCCAGCT	37		5	4942469	AGGGCAC		5	4995469	F
	CATTAAT	37		11	67142001	CGAGGCA		11	66898577	R
	GGTAAGA	37		17	4487109	GCTGGAC		17	4433858	F
	GAGAAAC	37		2	205333978	ACCCATG		2	205042223	R
	GCCAGTC	37		11	93754390	TTTCTCG		11	93394038	R
	AAAGGAC	37		6	32498493	CAACTGT		6	32606471	F
	CTGGAGC	37	Y		16636984	CGGGGGC	Y		15146378	F
	AGTACAC	37		6	46288667	ACTATTT		6	46396626	F
	ATAAGCC	37		6	43894639	AGGCATC		6	44002617	F
Red	GGCGGCC	37		11	9113184	CGGCCCC		11	9069760	F
Red	GGACAGA	37		15	74244249	GCAGGGA		15	72031302	F
	AAGGTAC	37		3	74003742	ACATTTC		3	74086432	F
Red	GGGCGGA	37		3	49941408	CGCCCAT		3	49916412	F
	CAGAGTT	37		7	65334477	CGCCACA		7	64971912	R
	GCGTGTT	37		6	166260572	TCAGAAC		6	166180562	R
	CTTCTGC	37		1	56163457	CACAGTA		1	55936045	R
	CATAACA	37	X		43515245	GGCAGCT	X		43400189	F
	GCAGAGC	37		15	48034358	CGATGTC		15	45821650	F
Red	TGCGAGC	37		20	2633340	CGCGTCG		20	2581340	R
	AGAGAAA	37		12	125205034	CGGATGA		12	123770987	F

	TCCAGGC	37	Y	7177888	GCATCTT	Y	7237888	F
	TTTGTCC	37	8	131080137	TGATACC	8	131149319	F
	AGAATTG	37	2	161214856	CAATCAG	2	160923102	R
	GAACTCC	37	10	3179821	GGTGGAC	10	3169821	R
Red	GCCTTCG	37	1	92952533	TTTCCGG	1	92725121	R
Red	GGATGTG	37	10	24755438	CGGGGAA	10	24795444	F
	ACTAATT	37	10	48905601	CGGTTGT	10	48525607	R
	GGCTGCG	37	5	175626484	CGGCTCC	5	175559090	R
Red	CAAGTAT	37	10	94334836	CGAAGAC	10	94324816	F
	TGGCCTA	37	2	237620848	CATCAGA	2	237285587	F
Grn	TGAGCTC	37	17	80159595	CAACCTT	17	77752884	F
Grn	TGTCAGA	37	6	170732253	AGGTTTC	6	170574178	R
	CTGCCAA	37	13	30607830	CGTCCTT	13	29505830	R
	GCACGGC	37	10	134827324	CGGGCCC	10	134677314	R
Grn	TGCTTCC	37	12	11700321	CGGCAGC	12	11591588	F
	TAAGACT	37	7	113011963	CGTCACC	7	112799199	R
	ATTATCC	37	5	151476553	GGGAATT	5	151456746	F
	TGCTGCC	37	1	22645936	AGGCCAC	1	22518523	R
	TCTAGCC	37	10	63167726	CGCTATG	10	62837732	F
	CTTCACT	37	14	96563269	CGGTACT	14	95633022	F
Grn	GGCGGTC	37	X	108780378	CGGGGCC	X	108667034	F
	CTAGCCC	37	12	3563268	CGGAGGC	12	3433529	F
	GACAGGC	37	17	39204922	CGGCCCC	17	36458448	R
Red	CTGCGGA	37	17	41477468	CGGACAA	17	38832994	F
	CCACCCC	37	7	92533866	TGAGTCA	7	92371802	R
	ATCCAAC	37	10	6183575	ATAACTC	10	6223581	F
	TGCTCTT	37	1	11783294	GCCAACT	1	11705881	R
	ACCTGGC	37	11	76414904	GTGAGGC	11	76092552	R
Red	AGCCCGC	37	6	90597356	CGCACAC	6	90654077	F
	GGAGGAC	37	4	6902932	GGCCCCT	4	6953833	F
	TCCCCAA	37	1	223904225	CGGCAGA	1	221970848	F
	AATAAAA	37	19	57633694	CGCCTGG	19	62325506	R
	GCAATTC	37	3	13420961	CGGCTAT	3	13395961	F
	TAAATAT	37	8	122651271	CGCCAGA	8	122720452	F
	AATTTCA	37	5	60960963	CGCTAGT	5	60996720	R
	CAGAAAT	37	6	170055332	GGCAAGC	6	169797257	F
Grn	ACTGGGT	37	10	123909456	CGCGGAC	10	123899446	F
Red	GTGATTA	37	6	30079139	GTCTTAG	6	30187118	F
	GACTGAC	37	13	24247649	CGGCATT	13	23145649	F
	TGAGCTC	37	8	126698738	CGGGTTG	8	126767920	R
	TAAGGTA	37	19	54210518	CTGCAGT	19	58902330	F
	GAGAGGA	37	1	29190137	CGGGGCT	1	29062724	F
	CCTCCTG	37	19	8574557	GCTTCCT	19	8480557	R

	CCTGGGG	37	2	238795355	GGCAGTT	2	238460094	F
Red	CCGCCCC	37	19	51829984	TGTGTGT	19	56521796	R
	CAAAACC	37	14	52733216	CGATGCC	14	51802966	R
Red	AATCTCC	37	5	53813164	TTTTGCA	5	53848921	F
Red	GACACCC	37	X	152736387	CGCCAGC	X	152389581	R
	TCACCCC	37	1	202129566	TGGGGAC	1	200396189	R
	CCAGAAC	37	1	223747670	CGAGGTT	1	221814293	F
	GACAACA	37	7	3801491	CACACTG	7	3768017	F
	CTCGGTG	37	1	115398073	GTGGTAC	1	115199596	F
Red	GCAAAGT	37	14	105830631	AGAGCAC	14	104901676	R
	GACAATC	37	10	24633303	AGACATT	10	24673309	F
	CCCTTGT	37	11	63828569	GACTGAA	11	63585145	F
	TAATTAG	37	4	115484805	CGGGAGT	4	115704254	F
	TCCACCA	37	14	77371470	GATCTTT	14	76441223	R
	AAACCAT	37	5	140579296	CGCGTTC	5	140559480	F
	GAGACGC	37	10	88023185	CGGTTCC	10	88013165	F
	GACAAGC	37	7	42760642	CGGACAT	7	42727167	R
	TGAACAC	37	6	32490421	TAGTGAT	6	32598399	R
	TCGTTCC	37	4	37245254	GTCCACG	4	36921649	F
	CTGTGTA	37	4	15376215	TAAGTAA	4	14985313	F
	AGAGGTC	37	17	9677572	AAGTAGC	17	9618297	R
Red	CTGCAGT	37	8	1497307	CGCCACC	8	1484714	F
	CCCACGT	37	10	2564642	TCGCCTG	10	2554642	F
	CTGTTGT	37	12	21594328	CTTTAAA	12	21485595	R
	CAGGAGC	37	18	12035841	CGGGGCC	18	12025841	F
	GAATTCT	37	13	32480222	CTGTTCT	13	31378222	R
	GTGGTGT	37	13	41593519	CGCCCAT	13	40491519	F
	ACAGCCC	37	19	42211171	ACAGAGA	19	46903011	F
Grn	GCCCCCC	37	11	67141747	CGCCTCG	11	66898323	R
	GGCCACA	37	14	20482899	GCAAATT	14	19552739	R
	TAACCAC	37	5	75376460	AAAAAAC	5	75412216	F
	ACCTTCT	37	14	21156471	CGGATGC	14	20226311	R
	AGTCAAC	37	7	44203304	CGAGCAC	7	44169829	R
	GCTGCTA	37	14	75083495	CGGCAGA	14	74153248	F
	GCCACTG	37	4	85411336	CAGCCTG	4	85630360	F
Red	TTTCTCT	37	7	149322992	AGCGTGC	7	148953925	R
	CCGCGAT	37	10	651119	AAGGAAC	10	641119	F
	GTGAGGC	37	10	729956	CGGGCCC	10	719956	F
	CTGGGAA	37	19	46286198	CGGGAGC	19	50978038	R
	GAGTCTG	37	8	62052594	CGGACCC	8	62215148	F
	CCCGGGA	37	4	1496880	CGGCTCC	4	1466208	F
	AATCAGT	37	19	44330665	CGCAAAC	19	49022505	F
	TTCTGAT	37	5	41763261	CGGTCTC	5	41799018	F

	GCCGGCC	37	16	28634844	CGGAGGC	16	28542345	F
	GCCGAAT	37	14	103839038	ATCCTCC'	14	102908791	R
Red	GGTTTTA'	37	12	54405379	CGGAGAA	12	52691646	F
	AAACACA	37	2	122259959	CTGCATT.	2	121976429	F
	TGGCCTC	37	11	63435428	CGTCTCC.	11	63192004	F
Red	TTCTATC(	37	6	149151596	TGTGGCA	6	149193289	F
	ACTTGGT	37	18	35063285	TGTGTTT(	18	33317283	R
	GGTCGCG	37	9	140356475	CCTCCTG.	9	139476296	F
Red	ACTCGCA	37	1	148855262	CGCCGCC	1	147121886	R
	TGGCAGC	37	4	154155288	TTCTGCC.	4	154374738	F
	TCCCTCC(	37	19	2791705	CGCTGGG	19	2742705	R
	CCCATGC	37	14	76015669	TGAACTC	14	75085422	F
	TTTACCA'	37	14	70317582	GGTTTAC	14	69387335	R
Red	GCTGGGC	37	6	30652399	GGGGGCA	6	30760378	R
Red	TCATCAC	37	2	24397810	TTCCGTT'	2	24251314	R
	CTCCTGA	37	5	142623446	CTGTGCT'	5	142603639	R
	AGAAAAA	37	11	55111658	CGCCTAT	11	54868234	R
	CATTCTG.	37	13	39458059	AGATTTA	13	38356059	R
	TTCTGAA	37	11	26951784	CGAAGAC	11	26908360	F
Red	GACCCTT	37	4	132645884	GAGGACC	4	132865334	F
	TTCGAAG	37	3	129827549	GACCCAC	3	131310239	R
	CCGCGGC	37	6	29911087	GGCCAGC	6	30019066	R
	GTTATTA	37	6	29945409	CGGCTTA	6	30053388	F
	CTGCTTC(	37	2	2295901	GCAGGAA	2	2274908	F
	TCCTGGC	37	8	41583523	CGGGACA	8	41702680	F
	CTGTGTG	37	17	48275324	CGCCAGC	17	45630323	F
	TGATTCC.	37	10	82112873	CGGCTCT	10	82102853	R
	GGACGGC	37	3	10150065	CCTGGAC	3	10125065	F
	GTGTTCA	37	X	62780753	CGCCACG	X	62697478	F
	CCCCACT	37	19	44200812	CGGGAAC	19	48892652	F
Red	CCGGCCG	37	6	4079528	CGGGGCC	6	4024527	F
	GGGGTTG	37	1	26737262	CGGTGGC	1	26609849	R
Red	TCCCCTC.	37	5	5887068	GTGTTGC	5	5940068	F
	GCATTCA	37	10	130831384	CGGTGAC	10	130721374	R
	AGAGTGA	37	10	133909949	CGGCTCC	10	133759939	R
Grn	CTTCCGA	37	2	99438903	AGGTTTC	2	98805335	R
	TATTTCC'	37	5	140557227	GCCCAAA	5	140537411	R
	AACGAAC	37	4	37668528	TCCAATT.	4	37344923	R
	AAAGGGC	37	2	233217476	CGCCCAG	2	232925720	F
	GAGCTAA	37	5	135416331	CGTCACT	5	135444230	R
Red	CACCCTC	37	X	40439984	CGCCCTA	X	40324928	F
	AAAATAC	37	14	35629311	CCCTTAA	14	34699062	R
	CTCACTC.	37	19	39060314	CGGGCAC	19	43752154	R

	CCTTAGG	37	6	32301513	CGCAGAC	6	32409491	R
Grn	GCCCTGC	37	10	118387207	GTCAGGT	10	118377197	F
	GCCCAGT	37	3	139253838	CGGATTT	3	140736528	F
Red	CCTGGCC	37	18	72837627	CGGCCAC	18	70966615	F
	GGAGATC	37	14	50474530	GCACATC	14	49544280	R
Red	GGCTCCC	37	5	126409061	CGCTGTG	5	126436960	R
	TATCTTT	37	17	33390808	GCAAGAT	17	30414921	R
	AGAAGGC	37	15	64736348	CGGTAGT	15	62523401	F
	CACCATG	37	6	33035286	CGCGAAA	6	33143264	R
Grn	ACCCTCC	37	5	180048155	CGCCCAG	5	179980761	R
	GCCACCA	37	4	75608133	CGAAGGC	4	75827157	F
Grn	GGGCTTG	37	19	57630710	GAGTTGT	19	62322522	R
	ACAGCCA	37	5	34792020	CGTCATT	5	34827777	F
	CATTAAA	37	2	174719603	CGGGACC	2	174427849	R
	AGGATTC	37	6	36409367	CGCAAGC	6	36517345	F
Grn	GCTAAGC	37	5	110229987	TGCGATG	5	110257886	F
	AGCACCT	37	10	112261994	CGAGGTT	10	112251984	R
	CCTCTCC	37	1	20095795	ACTCTCT	1	19968382	R
Red	CCCCGCT	37	X	25025364	GCAGGCT	X	24935285	R
	GAACTGA	37	2	208572165	CGAGAAC	2	208280410	R
	ACTCCTC	37	22	18268631	CAGCCTG	22	16648631	F
	AGTGATT	37	15	91807630	GTCAGGT	15	89608634	R
Red	AGTGCGC	37	8	48676000	CTCGGCC	8	48838553	F
	GGAGTAC	37	11	5248426	CGGCTGT	11	5205002	R
	ACCTCCA	37	6	116381957	TGCTTTC	6	116488650	R
Red	GACCCGC	37	5	669397	CGGGCTC	5	722397	R
	CCGCAAC	37	X	55516224	CTGGCTG	X	55532949	F
	GGTGGTG	37	19	6710827	ATGCCAG	19	6661827	F
	CTTTGGT	37	2	154143768	CGCCGAC	2	153852014	F
	ATTTCTTC	37	12	54385517	CGCCCTG	12	52671784	F
	AAACTAT	37	2	113594611	ATTTGCA	2	113311082	R
	TGTTTAT	37	5	80260554	CGATTGC	5	80296310	F
	GCGCAAA	37	12	59311232	CGCCAAC	12	57597499	R
Red	CGATCTG	37	6	100895050	GCTCTCT	6	101001771	F
	CCTGGGG	37	11	69830436	CGGAGAA	11	69508084	F
	CCCATCT	37	5	167831317	CGGAGCA	5	167763895	F
Grn	AGGCGGT	37	7	76145562	ATGCCAC	7	75983498	R
	AGCAGCC	37	5	135416613	CGGGGGC	5	135444512	F
	CCAGGGC	37	1	227963164	CGGCACA	1	226029787	R
Red	TATATGA	37	19	36265700	CGAGAAA	19	40957540	R
	AGAGAGA	37	10	30721971	ATTTCCC	10	30761977	F
	GAACCGA	37	3	18464935	TGCAAAC	3	18439939	R
Grn	ACCTTAA	37	5	164064	ACTTTTA	5	217064	R

	CCTGTGT	37	6	32489555	CGCGCCA	6	32597533	R
	CCTCGCT	37	5	141783822	CGCCTCA	5	141764006	R
	CAAAACC	37	17	59937758	CACATGA	17	57292540	R
	CTTTCAA	37	5	78281819	CGCTTGT	5	78317575	F
	CGCAGAC	37	6	15300007	CTCATTT	6	15407986	R
	CTCATGG	37	19	55417436	CGCTCTG	19	60109248	F
	GAGCAGA	37	13	97794052	TTCCATC	13	96592053	F
	AACCCCA	37	12	133177075	CAGGGTC	12	131687148	F
	ACTTGAT	37	7	143746594	TCTAGGA	7	143377527	R
Grn	GCGGGCC	37	6	31276434	TCCGGGA	6	31384413	R
	TTCATCC	37	11	123048161	GAATCAC	11	122553371	R
	CTGCCCC	37	10	82189478	TCCACTG	10	82179458	F
	TAGTTTC	37	6	168815477	TCCCACA	6	168558326	F
	TCTTAGC	37	2	227288863	CGCACGT	2	226997107	R
	GGAATGC	37	15	74343476	CGGGTCT	15	72130529	R
Red	GGGCGCC	37	19	33623072	CGAGATC	19	38314912	F
	TGAGATC	37	17	72192970	CGCCCTC	17	69704565	F
	AATTTAA	37	17	21727476	CGCCCAC	17	21651603	R
Grn	TCGTGCA	37	12	133378609	ATGGGGC	12	131888682	R
	TGTCCCT	37	11	46942432	CGGTTCA	11	46899008	F
	GGAGCCA	37	11	57792403	CGGTGTG	11	57548979	F
	TGTATTA	37	4	41258374	TGGTAGA	4	40953131	F
	GCGGAGA	37	2	242989856	CGGGGAA	2	242638529	F
	ACTGTGT	37	10	93624759	ACAAACA	10	93614739	F
Red	GGGCCCA	37	4	170696239	CAAAGAC	4	170932814	F
Grn	CGGCGTC	37	14	105830859	TGCGCGG	14	104901904	R
	CGTTAAC	37	2	10517323	TAATGAA	2	10434774	R
	TTAAAAG	37	5	135342478	CGTCCAG	5	135370377	R
Red	GGGATGC	37	2	175594943	CGGGCTG	2	175303189	F
	GGGTGGC	37	6	30039151	CGGCCCC	6	30147130	F
	TGCTGAG	37	1	85426586	CGGAATA	1	85199174	F
	TCAGTTT	37	13	84454283	GCTGTCC	13	83352284	F
	GGGGTCC	37	1	110230456	CGGTTTA	1	110031979	F
Grn	CCGGGGA	37	7	153749736	CGGAGGA	7	153380669	R
Red	GCAGGAC	37	10	1517083	GAGGAGC	10	1507083	R
	CCAAGCA	37	14	99858181	CGGTGGC	14	98927934	F
	ATCTGAA	37	17	32895602	CTCTAGG	17	29919715	F
	TGGGAAA	37	1	86077581	CGCTGTC	1	85850169	F
	GAGCTAC	37	13	97794113	CCTGGGT	13	96592114	F
	CACAAAC	37	11	78131895	GGTTCTA	11	77809543	R
	TGAATAT	37	10	126086354	CCATTAC	10	126076344	R
	TTCCTGC	37	10	135342936	GTAGAGC	10	135192926	F
Red	GGGAAGC	37	X	48660440	GAGGCCC	X	48545384	R

	GTTGCAA	37	12	132263618	GAGAACT	12	130829571	R
	CGGCTGG	37	3	14602840	CGAGGCC	3	14577844	R
	TCAAAAT	37	3	197272328	GCTGGTT	3	198756725	F
	AAGGTTG	37	10	34817409	CGCAGCT	10	34857415	R
	AGTCAGC	37	17	76794560	CGGAACT	17	74306155	R
	GCTCCAG	37	17	42980188	CGGCAAC	17	40335714	F
	GTCCTGG	37	13	113677628	CGGCACC	13	112725629	R
	TTTCACT	37	11	6744048	TTGACAC	11	6700624	R
	ATGCATG	37	5	176784448	AGCCTGT	5	176717054	R
	TTGCTGA	37	13	37702050	CGGAGGT	13	36600050	F
	GGGGTCC	37	6	29911091	AGGTTCT	6	30019070	R
Red	GCCAAAC	37	6	30038922	GTTGGAG	6	30146901	R
Red	CGGGTCA	37	17	46619923	GGCCGGT	17	43974922	F
	GTCTTGG	37	12	49662405	TTTCTAG	12	47948672	R
Red	GGTCTTA	37	7	1099421	GCGGGTC	7	1065947	R
	TCCTTTC	37	2	45581335	AACATGC	2	45434839	F
	TTTTAAA	37	6	106536036	CGCCGTT	6	106642729	R
	CAACGTG	37	4	190123606	CGCCACC	4	190360600	F
	CAGATGT	37	2	158723291	CAGTTTC	2	158431537	R
Red	CCACGGC	37	1	1190137	CGCGGAC	1	1180000	F
	TCCCTCA	37	15	38361362	CACAAAA	15	36148654	R
Red	ACAAATC	37	10	6274873	GAACCGA	10	6314879	F
	GAGACCC	37	6	170054730	TCTCACT	6	169796655	F
	CATTGAA	37	6	46623767	CACAGAA	6	46731726	F
Grn	CCGCCGC	37	7	64043193	CGCGCGC	7	63680628	F
Red	CCCCCGC	37	17	61926700	GGTGTAG	17	59280432	R
	CATAAAC	37	8	39844956	CGCAGGA	8	39964113	R
	AAGTGGT	37	2	169346613	CGGGACA	2	169054859	R
Red	GCAACGC	37	X	133678716	CGCGTCC	X	133506382	F
Grn	AATTAAT	37	3	101406347	GAATGTT	3	102889037	F
	GGTTCCG	37	6	30421114	CAAGGAT	6	30529093	F
Red	CGTCCAC	37	11	65769518	GGGAACT	11	65526094	F
Red	CTCCCCT	37	6	1555311	ATGCTTT	6	1500310	F
Grn	CCTGTGT	37	17	77922865	CGCCACA	17	75537460	R
Red	AGATGGA	37	17	48858746	CGTCAGA	17	46213745	F
	CATATTA	37	3	142854187	ATAGAGA	3	144336877	F
	CAGTTGC	37	11	75130069	TCTGGCA	11	74807717	F
	AGGTCCC	37	2	242869012	TCTGGGC	2	242517685	F
	GCTTAAT	37	2	173902435	CGGCGCC	2	173610681	R
Grn	AGGTGGA	37	20	56725873	CGGCGAA	20	56159279	F
	CCAAATA	37	13	95652307	AAACACC	13	94450308	R
Grn	CCTGGCG	37	6	33860869	GCAGCGC	6	33968847	F
	CACCGTT	37	12	133382933	CGCTGTA	12	131893006	F

Red	CAACACA	37	7	944607	CGCAGCT	7	911133	R
	CATGCCA	37	6	30850581	CTGGGGT	6	30958560	R
	CAGCAGC	37	6	30955358	CGCCTCA	6	31063337	R
Grn	GGGGTTG	37	20	59542589	CGCCCTC	20	58975984	R
	TATCCCT	37	15	30264290	CGGTGGT	15	28051582	F
Red	GGGCGGT	37	7	150020108	CGGGAGC	7	149651041	F
	CCAATCC	37	2	233216865	TCAACAG	2	232925109	R
	CACACAA	37	2	10340837	TTGTGAA	2	10258288	F
Grn	TTCCCCA	37	12	114395548	CCGCTCT	12	112879931	R
	AACGCCC	37	17	38708638	CGGCTTG	17	35962164	F
	CAGGGTC	37	12	125223540	GAGAAGC	12	123789493	R
Red	GCGTATC	37	6	110721138	AACGGAC	6	110827831	R
Red	ACAAGAA	37	8	120429518	CGGACAC	8	120498699	F
Red	GAGGGGC	37	10	135246701	CGCCCCA	10	135096691	F
Red	CGGCTAA	37	21	36041605	TCCTTGA	21	34963475	F
	CTGAAGA	37	13	100470738	CGGACAC	13	99268739	F
	AGCAGAT	37	2	127951364	CGCCTTT	2	127667834	F
Grn	TAGTGGC	37	6	28322086	CGCCTGC	6	28430065	F
	TTGTCAC	37	19	20576478	CGTTGGA	19	20368318	F
Red	CTGCAGG	37	13	111111172	CTGGGGA	13	109909173	R
	CCTAGGC	37	12	7129898	AATGGTA	12	7000159	F
	AGACTTA	37	19	13276344	CGGAAGT	19	13137344	R
	TGGTATT	37	7	73476337	CGCTCCC	7	73114273	F
Red	AAAGGTC	37	10	134953542	GGCTCCG	10	134803532	F
	TCTGATG	37	6	32141882	CGCCAGT	6	32249860	R
	TTGGCGT	37	7	76624761	CGCCCGT	7	76462697	F
	GGAAATT	37	20	4795444	CGGGGGC	20	4743444	F
	CTAGGGA	37	1	161569954	CGATGGC	1	159836578	R
	CCTAGTC	37	Y	16636990	TCCCAGG	Y	15146384	F
	GACGCCC	37	10	110671988	AGTCAGT	10	110661978	F
Red	AGTGCAA	37	10	29747315	CGCACGC	10	29787321	R
Grn	GAGGAGC	37	17	7790048	TGCCAAG	17	7730773	F
	CGTGTTC	37	X	9434519	CGAGTCA	X	9394519	F
	TCTGGCT	37	7	140135515	CGCCCTA	7	139781984	R
Red	TGCTTCC	37	1	41303922	CGCCCCC	1	41076509	F
Grn	GGCTGTG	37	2	24625812	CGGAGTC	2	24479316	R
	TAGTTGG	37	2	161209326	ACCCTCA	2	160917572	R
	TTATAGA	37	13	27769437	CGCACAC	13	26667437	R
	TGTAGCA	37	12	6162821	CGCCCAG	12	6033082	F
	TCCACTG	37	6	16962712	CGGATGC	6	17070691	R
	AGCTCTT	37	6	54200888	CGGGAAT	6	54308847	R
	GAGCCGC	37	5	502074	TGTTGTT	5	555074	F
	AGCCAGC	37	12	67040901	CGGGAGT	12	65327168	R

Red	TGGACTA	37	19	37569299	CGCTCCC	19	42261139	F
	GACCATG	37	17	7820248	CGTGATG	17	7760973	F
	ATGAACC	37	2	46542361	CGGGTTG	2	46395865	R
	TTCCCCT(	37	11	19463903	TTATGTA'	11	19420479	F
	GAGGAG(	37	17	46560683	CGGTGGA	17	43915682	F
	GCAAAGA	37	2	88771031	AGGACCA	2	88552146	R
	AAAACA(	37	6	52288804	CACAGAA	6	52396763	R
	GATGACC	37	12	131369535	CGGCATG	12	129935488	R
	GTGCATA	37	12	113516445	CGGGGAA	12	112000828	F
	GCAACCC	37	10	123731471	CGGATAC	10	123721461	F
	ACTCCAG	37	1	42420460	CGGACTA	1	42193047	R
Red	TATTTAG	37	8	6664522	GGAGAA(	8	6651932	F
Red	CTCAGTG	37	1	248791716	CGGGCTC	1	246858339	R
	AAGGAA(	37	6	31529897	CTCTTTG.	6	31637876	F
	TGCCCAG	37	15	79722028	CGCGAAT	15	77509083	R
	AGAATG(	37	1	219634786	CGGCAA(	1	217701409	R
	GGCAGA(	37	18	77292410	CGCCAG(	18	75393398	F
	GACTTAG	37	10	72138124	CGCCCGG	10	71808130	R
	CTCCTCT	37	19	55309528	CGGGGAA	19	60001340	F
Red	GCCACCA	37	X	150067114	CGGGCG(	X	149817772	R
Red	CTACGGG	37	19	36485356	CAGTTAC	19	41177196	F
	TGCGCCT	37	10	4868333	ATGGCTG	10	4858333	F
Red	ATTCCCC	37	X	105855052	CGGACGA	X	105741708	F
	GTGCTCT	37	4	104123084	CGGGCCA	4	104342533	F
	CCCAGAT	37	8	129165545	CGGCTCG	8	129234727	R
	TTGTGGT.	37	5	34687565	TCCTCCA.	5	34723322	F
Red	CCGCCAT	37	4	967324	CGCCGCT	4	957324	F
	ACACACA	37	1	13877579	AAAATTT	1	13750166	R
	GAAGAA(	37	10	115388944	CGGGCCC	10	115378934	F
	GGGGCTC	37	19	17219073	CACGTTT.	19	17080073	F
	GCAAACA	37	4	79077191	ATTCTTT(	4	79296215	R
Red	TGTGTTC(	37	5	95295965	CGGAAG(	5	95321721	R
Red	GCGTGG(	37	20	61919450	CGGGAC(	20	61389895	R
	GCGGGCT	37	7	98889946	CGCCGTG	7	98727882	F
Red	CGCGAG(	37	3	126194923	TGGGCTG	3	127677613	R
Red	CGGGCTG	37	17	17628656	CGCAGGA	17	17569381	R
	TTTTCTCT	37	11	12785773	GGTTTAT'	11	12742349	R
	CTACAAT	37	15	96891476	CGCACAT	15	94692480	R
	TGCACTC	37	2	237551858	GTTTCCC'	2	237216597	R
	TCTCCAT(	37	15	68564546	AGCTGG(	15	66351600	R
	GCAGCCT	37	10	135341870	CACCCCT	10	135191860	F
	CCAAATG	37	14	99613263	CGCCTTG	14	98683016	R
	GGCTGCT	37	1	41828130	CGGGGG(	1	41600717	R

Grn	AGGACAC	37	6	39271655	CCGGATT	6	39379633	R
Grn	CAGCACG	37	18	77172117	CGCGTGA	18	75273105	R
	GAATCTC	37	9	22005288	AGAGAGC	9	21995288	F
	GAAGAGC	37	4	119990940	CGAGGCA	4	120210388	F
Grn	CAAATTT	37	11	123940566	CGGGAAC	11	123445776	F
Red	CGCCGCA	37	X	73513453	CGCTAAG	X	73430178	F
	TCCTCAT.	37	2	218925608	AAGTTCA	2	218633853	F
	GGGAGAC	37	15	76016056	CGGGTGA	15	73803111	R
Red	CCAGCCC	37	1	248300479	GCCTTCT(	1	246367102	F
	TGGACAA	37	2	158301839	ACTCCCT	2	158010085	F
Grn	TTTGCAG	37	2	177016490	GCTCACC	2	176724736	F
	AGTGCCG	37	X	83757504	CGTCACG	X	83644160	R
	AATCCAG	37	6	32493994	CGGCTGG	6	32601972	R
	GCTGCTG	37	4	141391053	GAGGGTC	4	141610503	R
Red	AGGGCTC	37	22	49717457	AGAGGAC	22	48103461	R
Red	GAAAGAC	37	X	134555711	CGTGCCC	X	134383377	R
Red	CCGGTCA	37	22	25160075	GGTTGAA	22	23490075	F
	CTAAGAA	37	3	112876728	GTAATGA	3	114359418	R
Red	GGCTACC	37	8	41583498	CGTCTGA	8	41702655	F
	GGACCAC	37	14	35032169	GAAAAAC	14	34101920	F
	GTTTATA.	37	15	81028959	CGTGAAT	15	78816014	R
	GTTGCCA	37	5	10401195	CGGTATG	5	10454195	R
Grn	ATCGGGA	37	12	8025640	CCGTAAG	12	7916907	R
	CGGGCAC	37	1	230513337	CCTGTTC(	1	228579960	F
Grn	CAGGGGA	37	4	57458863	GCCGCGC	4	57153620	R
Red	CCGCACA	37	6	10720630	CGGTGCC	6	10828616	R
	TCTGTTG(	37	14	22362410	CGGATCC	14	21432250	R
	CTTTGTG.	37	6	29913343	AAGAACC	6	30021322	R
	GAAGAGC	37	6	2972896	CGCCATA	6	2917895	R
	GCCAATG	37	15	79729041	TTCATTG	15	77516096	R
	ATGTTTT(	37	8	100770219	GAAACCA	8	100839395	R
Grn	GTTCTCA.	37	2	231714995	CGCCTCC	2	231423239	R
Red	TGCGACG	37	10	135278901	TGGGACC	10	135128891	R
	AGCAGCC	37	5	139040928	GTGTGCT	5	139021112	F
	TTCTCCT	37	4	187984958	GTCAGGA	4	188221952	R
	GGCAATC	37	6	118873071	CGATGAA	6	118979764	R
	CGCCAAC	37	6	29649084	CGAGCCT	6	29757063	F
	GGGTGTA	37	19	18503517	GCGGTCT	19	18364517	R
Grn	TTCTTTA	37	12	116864616	CGCCCCG	12	115348999	R
	TGGTGGC	37	5	140594406	CAGAGAT	5	140574590	F
	CCGGGTG	37	12	11700343	AAATTGA	12	11591610	F
	TACGAGC	37	12	10096118	GTGCCCC	12	9987385	F
	AAGGTGA	37	14	101505898	AGAACCT	14	100575651	R

	GTTTCTC	37	14	36563446	TCTAAGT	14	35633197	R
Red	CCCATCC	37	7	94953810	AGGGTGA	7	94791746	R
	CTGCTAC	37	5	67845133	TCACTTT	5	67880889	F
	AGCATAA	37	5	74908142	GGCAGCT	5	74943898	R
	TCAGCCA	37	6	5512308	CGCCCAG	6	5457307	F
	GTGGATA	37	19	55477759	CGGAGAT	19	60169571	F
	CCGAGCA	37	15	42186945	GGACTAC	15	39974237	R
	AGCCCAC	37	6	7106952	CGCTTCG	6	7051951	R
	ACAGGCT	37	2	179923444	CGCCCCT	2	179631689	R
	CCTGTGG	37	22	25571929	CTGAGGT	22	23901929	R
	GAAGTGA	37	5	83681563	CGGTTAG	5	83717319	F
	GGTCAGC	37	7	112063079	ATGGGTC	7	111850315	R
	ATGCTGT	37	3	32400704	CGGCCCA	3	32375708	F
	GCGAGAT	37	10	6183872	CGCACTT	10	6223878	R
	CGAGCCC	37	11	2813121	TGGGGGC	11	2769697	R
	CCACCTT	37	2	74357872	TGCTTGG	2	74211380	R
	CTTTCCT	37	14	71022823	CGGCTGC	14	70092576	R
	ATGTGGC	37	12	56115029	CTTCTGC	12	54401296	R
	ACCACAC	37	5	76010598	CGGCTGA	5	76046354	F
	CCTCTCC	37	2	8144208	AAGTGTT	2	8061659	F
Grn	GTTCCAC	37	11	1712765	AGGGGTC	11	1669341	F
	GACCTGT	37	5	153039227	CCATGGT	5	153019420	F
Grn	CCCCGGG	37	X	110039080	CGCGCAC	X	109925736	R
Red	GCTCAGA	37	17	650208	CGCGACC	17	596958	R
	GGGGGGC	37	10	50829959	CGCCCCC	10	50499965	R
Red	ACTCTAG	37	4	183063459	CGGGTCG	4	183300453	F
	CCGGGAA	37	17	56283364	CGTCTTT	17	53638363	R
	ATTTTGT	37	19	10231711	CTCCCTG	19	10092711	F
	TATAAAG	37	2	145753993	CGCAAGA	2	145470463	R
	TGCTGAT	37	6	32522400	CGCTCAG	6	32630378	F
Red	GTCAGTG	37	3	194786989	GGGTGTG	3	196268278	R
Red	GCACGCT	37	12	297831	CGCACGT	12	168092	F
	CAAACAC	37	12	24974720	CGGGATC	12	24865987	F
Grn	GTGGGGA	37	8	145737513	CGGGCGC	8	145708321	R
	TTCTAAT	37	17	77906735	AAAACAT	17	75521330	R
Red	ATCAGTC	37	6	7758958	AGGCGGC	6	7703957	R
	GCAGTAC	37	12	113408421	CGGAAAA	12	111892804	F
Red	AGCACTC	37	1	201618284	CGCTCGC	1	199884907	F
Red	ACTGGCA	37	18	22006089	CGATCGC	18	20260087	F
	AGGTCTA	37	17	79045765	CTGCAGA	17	76660360	R
	TTTGGAA	37	21	36421941	CGCTGGC	21	35343811	F
Red	CCCTTCC	37	8	48675647	AACACCC	8	48838200	F
	TCACTGT	37	5	54448226	CGGATTA	5	54483983	R

	AGCTGCT	37		15	29213748	GAAAGGA		15	27001040	F
	CCTGTAG	37		17	13249936	CGCGCCA		17	13190661	F
	TTGCGGG	37		10	34344580	TGGCCCA		10	34384586	F
	GTTTTTC	37		7	134298937	CGCCACA		7	133949477	F
	CACACTT	37		1	218382922	TTTAGTC		1	216449545	R
	GTTCTAA	37		14	92301922	TCAATGG		14	91371675	R
	AGCAAAA	37		4	144208277	AGAAAAC		4	144427727	R
	ATAGATG	37		3	4762242	AGGCAGT		3	4737242	F
	AGCCCCA	37		11	64815313	CGAGGCT		11	64571889	F
	CTCCCGG	37		7	134856654	CTTCAAG		7	134507194	F
	CCTGGGC	37		7	105423113	GCTCCAT		7	105210349	R
Grn	GGGCGGC	37	X		152740227	GGGCGGC	X		152393421	R
	GCAGAAT	37		12	54383692	CGGCTAG		12	52669959	F
	GCCGAGC	37	X		150067102	GCCACCA	X		149817760	R
Grn	CCACTGA	37		15	23811369	TGCAGCC		15	21362462	F
	TTCACAA	37		11	102139781	AGCACTA		11	101644991	F
Grn	TCCTGGC	37		1	161450473	GCAGGAC		1	159717097	F
	GAAGTCT	37	X		149531880	TTCTGGG	X		149282538	R
	GTTTCAG	37		10	44494208	CTTTGATC		10	43814214	R
	GTGGCTA	37		16	31439191	GAACAGA		16	31346692	R
	GTACCCG	37		9	115841081	TGTTGCTC		9	114880902	R
	GGAGGCT	37		14	94759767	CGAGAGC		14	93829520	F
Grn	CGCGCCC	37		13	25320133	GCTGCGC		13	24218133	F
	GTGGTTTC	37		7	1113237	CATCACG		7	1079763	F
	ACAGTGA	37		11	108408969	CAAAGCT		11	107914179	F
	GCTTCTG	37		14	87265617	ATTCTCTC		14	86335370	F
	AAAAGCT	37		2	19886939	CGTTCAA		2	19750420	F
Red	TGCCCCT	37		2	233271154	CGGGCCA		2	232979398	R
Red	CAGCGGC	37		5	140535759	GCTGCAA		5	140515943	F
	GCCTGGT	37		10	79110632	CGGTGAC		10	78780638	R
	CCAGTCA	37		21	42839625	CGGAGAA		21	41761495	F
Red	CTGAAAC	37		11	2779993	CGGGAAT		11	2736569	F
	TGTGTCA	37		17	80096661	CTTGTTCC		17	77689950	R
	CCAAAAT	37		5	140246626	CGCCAAC		5	140226810	R
	CAAAACA	37		5	171870875	TACCATG		5	171803480	R
	TTATCTTT	37		20	36530548	CGGCTTG		20	35963962	F
	TAATCAC	37		4	154491023	CGGGAGA		4	154710473	F
	AGTTCAC	37		14	59844444	CGGGCCC		14	58914197	F
Red	GACTCCT	37	X		144900177	CGCCGGC	X		144707869	F
	TTGAAAT	37		7	96649956	CGGAAAA		7	96487892	R
Grn	TCTTGGTC	37		2	238777656	GCCCTAA		2	238442395	F
Red	GGGAGTC	37		6	142238173	CAGTGGC		6	142279866	R
Red	CTACCGC	37		14	24780404	CGCCGCC		14	23850244	F

Grn	AGCCGGC	37	X	19905497	CCGCCGA	X	19815418	R
	TAAACAC	37	7	5179884	CGGCCTC	7	5146410	R
	AGGTCCA	37	6	32549496	TGTGAGT	6	32657474	F
	GATTGCT	37	7	130875551	CGCTCCA	7	130526091	R
	GAGGAAC	37	4	154174325	CGCTCCT	4	154393775	R
	TTGGCTT	37	12	3563432	CTGAGCC	12	3433693	F
	GAAGGGT	37	14	77926864	AGGTCTG	14	76996617	F
	CCAGCTC	37	10	73770523	CGGCCAC	10	73440529	R
	AGAGAAC	37	3	123339334	GAACATC	3	124822024	F
Red	TGAGAGC	37	6	37617864	CGGGTGC	6	37725842	F
	ACGCCAC	37	14	21467375	CGCCTTC	14	20537215	R
	CGGGGCT	37	X	12993759	CGGCATC	X	12903680	R
	CCGAGTG	37	7	102574504	CGCACCA	7	102361740	F
Grn	CGCCGCG	37	8	52322009	CGTGGGC	8	52484562	R
	AGACTCC	37	14	69784107	CGGAGCT	14	68853860	R
	TCTGGCC	37	22	46481816	TCCTGGG	22	44860480	R
	TGTTTGT	37	4	107434718	CTTCTAA	4	107654167	R
	CTTTGGT	37	22	45608713	AGATCTG	22	43987377	R
	CATCTGT	37	10	96442154	AGTAAAC	10	96432144	F
Grn	CCTGCGT	37	10	135379242	CGGGCTT	10	135229232	R
	GGTCTCA	37	8	1321375	TTTGGCC	8	1308782	R
	GTGATGG	37	12	3384898	TTGGGCG	12	3255159	R
	AAGCAAC	37	11	72327160	CGCTGAG	11	72004808	F
Red	CCAGGTG	37	17	36718549	CGCATCG	17	33972075	R
	GAGGAAA	37	18	34917603	CGCTGCC	18	33171601	R
	GAATCCC	37	6	170058205	CGCTGTG	6	169800130	R
	ATAAAAC	37	6	30920086	CGAAGCT	6	31028065	F
Red	GCGCACC	37	X	17674384	CGGCCCT	X	17584305	R
	AAGTAAA	37	7	94954144	TGGCTGT	7	94792080	F
	GGAAAGA	37	11	27017005	CGGGTGT	11	26973581	R
Red	AATTCTG	37	11	119970504	TCCATAT	11	119475714	F
Red	CACTGAA	37	10	27235598	TACAAAT	10	27275604	F
Red	ATCTCTC	37	1	2928616	CGCCGAC	1	2918476	R
	AGTTATT	37	19	55416928	CGACTTG	19	60108740	F
	GTTCTGG	37	20	61731979	CGGCCCC	20	61202424	F
Grn	CGGCCCC	37	7	63642083	GGGTTCG	7	63279518	R
	ATTTTAG	37	10	64323709	GAGTCAC	10	63993715	F
	ATGACTA	37	6	134026410	CGGTGGT	6	134068103	F
	GCAGAGC	37	12	313104	CTTTCCT	12	183365	F
	AAGTGAA	37	12	11080457	TGTGATA	12	10971724	F
Grn	AATATTG	37	7	96648205	TGACCGC	7	96486141	F
	TGCCAAA	37	6	39769426	CGCCTGT	6	39877404	R
	CGTGTTC	37	10	1251771	CGTCAGC	10	1241771	R

	AATGCAC	37	3	30512797	GAAGAA	3	30487801	R
	TTATGTG	37	11	61159687	CTGGAAA	11	60916263	R
	GAATTAA	37	4	147557774	CGGGAG	4	147777224	R
	CCCCAGT	37	17	77478637	CTGCAGG	17	74990232	F
	GGAGGGT	37	18	77678514	CGACCCC	18	75779502	R
	ACTAAGA	37	12	99130587	AAATTAG	12	97654718	R
Red	TGGGGAC	37	7	158059142	CTGCTGC	7	157751903	R
	ACTATCA	37	19	49202858	CCCAGTA	19	53894670	R
Grn	CCCAGCC	37	19	41317837	TTCACGA	19	46009677	R
Red	GGAGCCT	37	15	79104481	CGGCCGT	15	76891536	F
	GAAGCCC	37	6	30039374	ACCCAGC	6	30147353	R
	ACCCGAC	37	17	8124867	CCGGGTC	17	8065592	F
Red	TTGGACG	37	15	40268687	CGCAGGA	15	38055979	F
	GGTTGCA	37	14	70826997	CGTCTCA	14	69896750	F
Red	CCTGCTG	37	1	207842927	CGCCCCC	1	205909550	R
	TACGTGT	37	10	68688049	CTGGGAC	10	68358055	F
	GATTTAT	37	1	230992616	CGGTGTA	1	229059239	R
Red	TTCTCAC	37	7	149469722	GGGGCCC	7	149100655	F
	ACCCACT	37	8	37660108	CGGGACC	8	37779266	F
	TCCTTCT	37	2	133429169	CGGGGAC	2	133145639	F
	CGGCGTG	37	1	41303356	GGGCCCT	1	41075943	R
Grn	AGGCTGC	37	X	18443740	CGGCAGA	X	18353661	R
Grn	TCTGCGC	37	10	4868331	GGCTGGA	10	4858331	F
	CAGTCAT	37	18	33548566	CGGCTTG	18	31802564	R
	GTCCTCA	37	1	168091000	ATCACTG	1	166357624	R
	ACCGCTA	37	19	58728728	CGGGCCT	19	63420540	F
	AGTACCC	37	1	246416688	CGGGAAC	1	244483311	R
	ATGGCAC	37	4	22239907	CGGCAGC	4	21849005	F
	GACCGCA	37	1	247457841	CGGTTAC	1	245524464	R
	GGGGGTT	37	1	156624237	CGGGAGC	1	154890861	F
	GTTCTGA	37	1	2120985	CGCATTA	1	2110845	F
	AGATGCA	37	2	64839958	CGGCCTG	2	64693462	R
Grn	CCCGGAC	37	20	25128805	ACTAAAA	20	25076805	R
	GGACTCT	37	1	244763541	CCAGCTT	1	242830164	F
Red	AGAAATC	37	2	1746207	CGGGTGA	2	1725214	R
	AACCCAC	37	2	97331035	GTTTTGT	2	96694762	R
	CAGCAAC	37	10	10403467	CGCCACT	10	10443473	R
Red	GCAGAAC	37	8	62052207	GCGCAGC	8	62214761	R
	GTCTTAG	37	2	236234204	CGGCAAC	2	235898943	F
Grn	TGGCATA	37	10	3138418	CGCGTGC	10	3128418	F
	GAAGGAA	37	11	1892074	ATGGCAC	11	1848650	R
	TGATGGT	37	7	143081287	AGTCCAA	7	142791409	R
	CCTCCTC	37	1	226107899	CGCAGGC	1	224174522	F

	TGCGCAG	37	19	55038263	GGTGAAC	19	59730075	F
	TTTTTAA	37	12	124780809	CAAACCC	12	123346762	R
	CCCATTC	37	10	104512523	CCTGGCT	10	104502513	R
Red	TCAGTCT	37	12	54391631	CACTTGG	12	52677898	F
	TAGATGG	37	21	46829943	TTGGGCC	21	45654371	R
Red	CTTCCCT	37	X	71351980	CGACCTC	X	71268705	R
	CTGGGTG	37	3	115758614	GCCATTC	3	117241304	F
	AGAGGAC	37	6	31838544	CGCCCTG	6	31946523	R
	GTGCACA	37	11	132939610	GTCACCT	11	132444820	F
Red	CCGGCAC	37	X	30907117	CGCCGCA	X	30817038	F
Grn	GACCCCA	37	7	96650668	TGTCTTT	7	96488604	R
	ATTCTAT	37	19	36801408	AACAGCT	19	41493248	R
Red	CTTACAC	37	7	158122229	GGCAGGC	7	157814990	F
	AGGGATA	37	6	158136705	CGTGGCC	6	158056693	F
	TTGGCCC	37	17	40439433	CGGGTTG	17	37692959	R
Red	CGTTGGC	37	6	127796683	GCAGATC	6	127838376	F
Red	GAAGCGC	37	12	25055967	CCATGGG	12	24947234	R
	CCTCTCC	37	Y	7141681	AAACGCC	Y	7201681	R
Red	GGGCGGC	37	12	54381013	CGCCCGT	12	52667280	R
Grn	GGAACCC	37	12	54377457	CGGTCTT	12	52663724	F
	CCGTGGG	37	1	7122726	CTGTTGG	1	7045313	F
	AACCCTT	37	7	130600075	TTGAGTA	7	130250615	F
Red	TGAAGGC	37	6	166260012	CGCGGAC	6	166180002	R
	AGAGGTC	37	7	127720749	GTGAGGA	7	127507985	R
	TCCCATC	37	1	248101009	CGGCAGC	1	246167632	R
	GCCTCAG	37	1	84831593	TCTGAGG	1	84604181	R
Red	CTGAGTC	37	4	190905801	GGAACCC	4	191142795	F
Red	GCCGTTA	37	6	28584061	CGGGCGA	6	28692040	R
	AAATGGC	37	2	158687877	CTTGAAC	2	158396123	F
	CAGTTCC	37	6	47668044	CGCTGAG	6	47776003	R
	GATGGAA	37	17	9546550	CATATAC	17	9487275	R
	CTCTAAT	37	1	203372112	AGATTTC	1	201638735	R
	ATTCTGT	37	6	2773876	CGAAGAA	6	2718875	F
	CCTAACC	37	11	103403056	CGTTCCC	11	102908266	R
	AGACTTC	37	11	74323002	CGGTAGC	11	74000650	R
	TTTGAAA	37	X	11682132	ACCCGCT	X	11592053	F
	TGATGAT	37	8	142505578	CGGCTTC	8	142574760	R
	GATGTGT	37	14	100497940	CGTGGTC	14	99567693	F
	AGACAAC	37	18	13916113	AACCCTG	18	13906113	R
	CCTGGAG	37	6	31474086	AGAATCC	6	31582065	R
Red	CCGACTG	37	X	152912606	CGCCCCG	X	152565800	F
Red	GTCAGGA	37	6	170547656	TGCAAAA	6	170389581	R
	GTGGGCC	37	1	248100600	GAGACAC	1	246167223	F

	TGGCCTC	37	1	8960134	CGGAAAT	1	8882721	R
	GGCCCAC	37	8	131457460	CGCAGCT	8	131526642	F
	ATAAAAT	37	7	17316151	CGGACAA	7	17282676	F
	CCATCAA	37	7	158032352	TCTCCTCT	7	157725113	R
	TCCTCAA	37	3	9976241	AAAGCTC	3	9951241	F
	GCGAGGC	37	7	158533927	CGCTGCT	7	158226688	R
	GTTTACG	37	13	100989375	CGCGCCC	13	99787376	F
	AAAAAAT	37	8	91683243	CGCATTC	8	91752419	F
	AGGTTTA	37	7	150263322	CAGAAAC	7	149894255	R
Red	CCACACT	37	7	157370486	GGTCTGG	7	157063247	R
	CAGCCCA	37	15	33586239	CGGGACA	15	31373531	R
	CAAACAT	37	15	40421005	CGCGGGC	15	38208297	R
	AGCTGCT	37	6	36076933	CGGGCCC	6	36184911	R
	AAAATTT	37	17	73316280	AAAAACT	17	70827875	R
	CTGGGGT	37	6	30039900	GGATTCC	6	30147879	F
Grn	AGCAGTT	37	X	84258823	GACAAAT	X	84145479	F
	TAGTACT	37	1	215177441	ATCAGTT	1	213244064	R
	ACCTATA	37	1	170044647	GTCACTT	1	168311271	R
	GTTTCAG	37	12	101381320	TCATTGG	12	99905451	F
	TGTGGCT	37	4	188131622	CATGCAA	4	188368616	R
	GGATTCA	37	6	36891482	CGGGCCT	6	36999460	F
Grn	TGACGGC	37	14	21191754	TCTAGGG	14	20261594	F
	TAAAATG	37	19	1070986	CGGGTCC	19	1021986	R
Red	CGTGGCG	37	2	89065319	CGGCCCC	2	88846434	R
Grn	AGCTGGT	37	15	40364740	AAAACAT	15	38152032	F
	GCTATTC	37	2	65290738	TCTGAGA	2	65144242	R
	GGCTTGG	37	15	83332087	CGGGGCA	15	81129142	F
	CGAGGCC	37	20	33292126	CGGCACA	20	32755787	R
	AACCATG	37	12	68592548	ACTGACT	12	66878815	R
Red	CCCCTGC	37	2	9235898	CCCTGCC	2	9153349	R
	TGAGTCC	37	6	33040610	TCCTCTCT	6	33148588	F
	GTCTCAC	37	6	27799380	CGCCAAC	6	27907359	R
	CGCAACC	37	10	112404997	CGACCCA	10	112394987	R
Grn	ACCACTT	37	15	31355304	GTCCTTTC	15	29142596	F
	TATAAAA	37	7	48124396	ATTTATCC	7	48090921	F
	AGATTCC	37	12	49061475	CGGGTAA	12	47347742	F
	GCCAGCC	37	15	50170642	CGGCTTT	15	47957934	F
	CAGTACA	37	17	40936820	TTTAGAA	17	38190346	R
	AGTTGTT	37	2	28509570	TAACAGA	2	28363074	F
Red	TGGGTGT	37	X	153095300	CGCACTC	X	152748494	F
	TTAGGGC	37	17	6734940	CGGTGGC	17	6675664	F
Grn	CCCTAAG	37	17	80969547	TCCAGAA	17	78562836	R
	TGAACTC	37	19	17762314	ATCTCAC	19	17623314	F

	CGATTCA	37		1	23521332	GCAGTCC		1	23393919	R
	AATTGGC	37		1	19186260	CGGTGGC		1	19058847	R
	CTGCCCC	37		3	28944854	CGGGCCA		3	28919858	R
Red	ACATTTC	37		10	12853879	GCCTGAA		10	12893885	R
	CAGGGTC	37	X		150396221	TAAGGCC	X		150146879	R
Grn	TGGAGAC	37		1	20238347	CGGTGTG		1	20110934	R
	TTTAACC	37		17	35853648	AACCATA		17	32927761	F
	AGATGGA	37		10	95706927	CGCACTT		10	95696917	F
	CGTCCTC	37		7	5731340	CACTAGT		7	5697866	F
	TCAGGGA	37		2	242843504	ACCTGAA		2	242492177	F
	TTCCTCC	37		7	157869970	CGACCCT		7	157562731	F
Red	CCCACTG	37		5	171534017	CCGGGTT		5	171466622	F
Grn	GGAGGCT	37		2	3699334	GAGAGCC		2	3677209	F
	TTGAAAA	37		3	41460736	CGGTGTT		3	41435740	F
Grn	GCCTGGC	37		12	48336164	ACCCGGA		12	46622431	R
	ACCCCCC	37		6	42695674	CAACTCT		6	42803652	F
	AGCAATG	37		1	230566509	CGAAGAC		1	228633132	F
	CATTCCC	37		5	218153	CGGGGCT		5	271153	F
	CACACAT	37		3	55517496	CCCTGGG		3	55492536	F
	CTGTGCT	37		11	126660703	CGCCCCC		11	126165913	R
	GAGAACA	37		17	80394529	CGGACGT		17	77987818	R
	AGGAGGC	37		7	158187725	CGGCTCC		7	157880486	F
	AGCAGAC	37		17	73074561	GAGCTGA		17	70586156	F
	ATTTCCT	37		13	31422123	AATCATG		13	30320123	F
Grn	CGCCGCC	37	X		20009138	AAGGAAC	X		19919059	F
	TTATGAA	37		4	152090378	CGGTATT		4	152309828	R
	GGGAGGC	37		1	110670021	GGAAGAC		1	110471544	R
	TTAGCAT	37		1	234754601	CAATGTG		1	232821224	F
	CCTCCTC	37		5	5057500	GACATGC		5	5110500	R
	GCCGGCC	37		5	1857306	CGGCCCC		5	1910306	F
	GCTAGGC	37		8	30087400	CCACCAC		8	30206942	F
Grn	GACATAT	37		6	30419612	CGCCTGC		6	30527591	R
	GAGGCCA	37		17	76179594	CACACCA		17	73691189	F
	ATTAAGA	37		6	32414433	AAAAGTA		6	32522411	R
	CTGCTTT	37		17	46618614	AAGGGCA		17	43973613	R
Red	GGAAGTC	37		20	29611924	CGTCTGT		20	28225585	R
Red	ACGGGCC	37		4	940893	CGCATGC		4	930893	R
	TGATTGG	37		6	32709158	CGAGGTC		6	32817136	F
	CACTGAT	37		7	1055600	GGATCCT		7	1022126	F
	CCGCAGC	37		4	730453	TGTCCCC		4	720453	R
	AGTTTTT	37		17	37024042	AGCCATC		17	34277568	R
Red	TAGACAT	37		1	204452127	CGCTGTA		1	202718750	R
	TTGGTGG	37		11	71524500	CTCTGGG		11	71202148	F

Red	CGAGGAC	37	6	28584121	CGCCTTC	6	28692100	F
	CATAGGC	37	X	118827407	GGGTGTG	X	118711435	F
	GTATGAG	37	6	28322103	ACAAACA	6	28430082	R
	AGAACCC	37	8	1400221	TGTTCGC	8	1387628	F
Grn	GCTGCCG	37	11	73054121	GCCGGGC	11	72731769	R
	CTGCGTG	37	6	9399379	ATGAGCT	6	9507365	R
Red	TCCGGTC	37	7	27208285	CGCCCTC	7	27174810	F
Red	ACTGGTG	37	X	40440209	CGGGTCC	X	40325153	R
	AGCAGTC	37	2	101680441	CGCAGGA	2	101046873	F
	GGTCCTG	37	2	9705768	TTTAGGG	2	9623219	F
	CAGGTGT	37	14	100618491	CGGGCTG	14	99688244	R
Red	CCTAAAA	37	6	33041221	CGCTCCT	6	33149199	F
	CATTTGC	37	14	96284990	AAAACTC	14	95354743	R
	TTTATTT	37	4	141877033	CCTAATA	4	142096483	F
	CACATTT	37	18	44627440	CGGACAT	18	42881438	R
	GAGAAGC	37	7	129996313	CGGAAGA	7	129783549	R
	GTTCACC	37	3	195578259	CGGCCAC	3	197062656	R
	GTCTCGT	37	2	240121297	CGAATGC	2	239786234	F
	GGCAAGC	37	3	115120356	CGCGTTA	3	116603046	R
	TCATAAC	37	12	7863229	TTTTTGC	12	7754496	R
	CCAATCG	37	1	209878178	CTACAAC	1	207944801	F
Red	ACTCCGC	37	2	74875227	CCTGGCC	2	74728735	R
	TTGAACT	37	4	79258587	CGGCCAC	4	79477611	R
Grn	TTCAGCT	37	X	64754701	TCCGATT	X	64671426	F
	CCTTGCC	37	X	152774831	TCTAGGA	X	152428025	F
	ATCACAG	37	1	182002021	CATGAGA	1	180268644	F
	TCTTAGC	37	6	7787925	CGCAAAT	6	7732924	R
	GATGTAC	37	1	1227307	CGTGTGT	1	1217170	F
Red	CATCTAT	37	1	12184574	CGGCCTC	1	12107161	F
Grn	AGACAAC	37	20	52199748	CTGCTGC	20	51633155	F
	TTTAAAG	37	3	22422855	CGGAGCA	3	22397859	R
	ACATGAC	37	6	33049983	CGCAATT	6	33157961	F
Grn	CTCGCCC	37	19	52954615	TCTGCTA	19	57646427	R
	CGGCAGC	37	1	226150312	CGCTGCA	1	224216935	R
	ACTAGTT	37	8	124284393	CGAAAGA	8	124353574	R
	GGGAGAC	37	1	181290986	CGGTAAC	1	179557609	F
Red	GGGCCAA	37	8	283841	AAGACGT	8	273841	R
Grn	CACCCGC	37	1	27687232	CGCACCC	1	27559819	F
Grn	CTCACAC	37	7	63353570	CCCGGGA	7	62991005	R
	GGGCCTT	37	4	159191054	ATTTCCC	4	159410504	R
	GATTCAC	37	1	15538911	AGACTAC	1	15411498	F
Red	CGGGAGC	37	14	105400489	CGCCCGC	14	104471534	R
	TGAGAAC	37	8	38847958	TTTTAAA	8	38967115	R

Grn	CCGAGCA	37		2	795935	TTTCTGT		2	785935	F
	CAGCCTC	37		14	97832998	GTGCTAT		14	96902751	F
	AAGTCGA	37		17	182928	CGGCCAT		17	182928	F
Red	CGAGGCC	37	X		139014368	CGCCCCC	X		138842034	F
	TCATCAT	37		13	59169584	CGTGGGC		13	58067585	F
	TTCTTCT	37		2	189762162	TAAACAT		2	189470407	F
	CATAGTA	37		12	25611175	ATGTCAC		12	25502442	F
Red	GTCAGGA	37		11	20134289	CGGGCTC		11	20090865	F
	CCCTGTC	37		2	233216361	CCTGCAC		2	232924605	R
	TGGTGCT	37		12	113652889	GGTCAGT		12	112137272	F
Grn	CACGGAC	37		6	887772	CGCGGAC		6	832772	R
	AGGGAA	37		5	110105162	TGTTTGA		5	110133061	R
Grn	GGGCAGC	37		7	997617	CGCGTGG		7	964143	F
Grn	GCACTCG	37		4	1202966	CAACCGT		4	1192966	F
	TTTGTGA	37		6	2528653	TCTCATT		6	2473652	F
Red	CAGGCGT	37		3	56717625	CGGGCTT		3	56692665	F
	GGCTCTT	37		14	93502930	TGGGGTG		14	92572683	F
Red	GTTAACG	37		1	202778869	TAAGAGA		1	201045492	R
Grn	CAGCGCC	37		1	207843084	TGGGCTG		1	205909707	R
Grn	GCTGTGC	37		3	132115350	CGGGGCC		3	133598040	R
Red	GCCGCTG	37		13	102105049	AGCAGAT		13	100903050	F
	CTCACTT	37		19	7766960	GTGTCTC		19	7672960	F
	GGTAAAC	37		5	68787747	CGGGAA		5	68823503	R
Grn	TCCGCAA	37		5	118603938	CGGGCCT		5	118631837	R
	GTTAAGT	37		3	119243933	GACTATC		3	120726623	R
	CGCTGGG	37		7	64035529	CGGTAGT		7	63672964	F
	CTTTCCA	37		1	3096986	CGCGGCC		1	3086846	R
	ACCCACC	37		5	43316468	CGAGATC		5	43352225	R
Red	AGATTGA	37		19	1456157	AGCTGCT		19	1407157	R
Red	CGCGCTG	37	X		53461271	CGCTCCG	X		53477996	F
Red	TTCTGTG	37		20	59542578	GGGTTGG		20	58975973	R
Red	GGTCTCC	37		8	7537673	AGCGACC		8	7575083	R
	TCCTGTT	37		1	3208865	ACACTTG		1	3198725	R
Red	TCAGCTT	37		9	136098495	CGCTCAA		9	135088316	R
Grn	CGCCTGC	37		5	1594676	ACACCGC		5	1647676	R
	ACGGGGC	37		9	140268976	GAGATTT		9	139388797	F
	GTTCAGC	37		3	99378032	ACCACGA		3	100860722	F
	TGTAGCA	37		8	108208344	TTCCCAA		8	108277520	R
	AGGCTGT	37		8	144659627	CGGCGGT		8	144730770	F
	AGAGAA	37		5	114830673	AAAAGCA		5	114858572	R
Red	CCCCTGC	37		2	26205876	CGCCAGC		2	26059380	F
	GCTTCAA	37		2	19082803	AAGAGCA		2	18946284	R
	GAAAGA	37	X		21858304	CGCTCTA	X		21768225	F

Red	CATCTCG	37	3	193587490	CGCAATG	3	195070184	R
	TCAGAGT	37	4	38622735	CGTGTTT	4	38299130	R
	CCTTATT	37	1	226599374	CGGAGA	1	224665997	R
	CTCTGGC	37	9	91997686	AGCCAAC	9	91187506	R
Red	TGGGGA	37	X	139585911	CGGAACC	X	139413577	R
Red	TGGGTGA	37	14	103687728	CTTCTAA	14	102757481	R
	CTGAGCA	37	9	100881995	CGTTTTG	9	99921816	F
	GCGTGCG	37	8	72917695	CGCCAGT	8	73080249	R
Grn	TAAAACA	37	1	110752307	CGATGAA	1	110553830	R
Grn	GCTGGAC	37	19	3595778	CGCGGCC	19	3546778	F
Red	CCTCTCC	37	12	115124768	ATTGGCG	12	113609151	F
Red	GAGGGCC	37	6	30079203	CGGAAA	6	30187182	R
	GATTTCA	37	6	143607364	CGTGGAC	6	143649057	R
	GGGGCAT	37	9	124363848	CGGAACA	9	123403669	R
	CCTGTGT	37	9	120510294	ACTTTCA	9	119550115	R
	CAGGATC	37	9	139371233	CGGAAGA	9	138491054	R
Red	AGCTGGC	37	8	1914262	CGCCCAG	8	1901669	F
	TCCGGAT	37	12	114123213	CGGCCTA	12	112607596	R
Grn	GGTTCCG	37	9	140311919	GACTTTC	9	139431740	F
	TCTGATT	37	9	115824717	CTCCCCT	9	114864538	R
	CCAGGAC	37	X	152000116	GGTGTCA	X	151750772	F
Grn	ACACTCA	37	7	144100802	CGGTCCC	7	143731735	R
	GCCACAA	37	12	75785232	TGTAAAC	12	74071499	F
	TTGGCTT	37	1	152162025	ATGACTT	1	150428649	F
	TGTGAGT	37	17	48098926	CATCTTC	17	45453925	R
	AGTTTTC	37	6	77563066	AACAAAC	6	77619785	F
	TTGCTTC	37	6	1599123	CGGGCAC	6	1544122	F
Red	GCCGTGC	37	2	237123218	TAGGAGC	2	236787957	R
	TCCCTGT	37	13	20714863	CAGCTCT	13	19612863	R
	TATTGTA	37	1	244525971	GGGCCTA	1	242592594	F
	GCTCATG	37	7	157348237	AGACCAC	7	157040998	R
Grn	CCCCTGC	37	X	102319001	TCTGCGC	X	102205657	R
	CTGCTGA	37	17	45699260	CGCCAAT	17	43054259	F
Red	TCAGGGC	37	8	131000521	CCCAGGC	8	131069703	F
Red	GGCGGGC	37	10	135171718	CGGCCCG	10	135021708	R
	CCCTTTG	37	1	22303523	CTTCTGC	1	22176110	R
Grn	ATTCCCT	37	2	105118572	CGCGCGC	2	104485004	R
	GCTGAGT	37	13	36049335	GATCGGC	13	34947335	F
	CCAGCGC	37	1	247537159	TCTATGT	1	245603782	R
	CTCTTGA	37	17	73056681	TTTTCAC	17	70568276	R
Red	ACCCTAG	37	1	149186607	CGGGGTC	1	147453231	R
	ATATAGA	37	13	52736798	CGGTGAC	13	51634799	R
	TGTTCGC	37	6	11901382	TTCTCCA	6	12009368	R

	AGCGGGT	37	11	13985911	GGGCACC	11	13942487	R
Red	AGCCCGC	37	17	80255457	CGCTCAC	17	77848746	R
Red	TCCCCGC	37	8	70623422	CGTGAAT	8	70785976	F
	GTCTTTC	37	3	11688349	GATCCGG	3	11663349	R
	TTTTTCT	37	12	6658378	CGCAAGC	12	6528639	F
	CTGTATA	37	22	18738315	CGTCGCT	22	17118315	F
Grn	TCCAGTA	37	20	23969237	CGCGGGT	20	23917237	F
	TTATTTT	37	2	222807796	CGCTTTG	2	222516040	R
	GGTGACT	37	7	37534909	TTTGAGG	7	37501434	R
	ACTTAAA	37	3	23727711	TGGAATT	3	23702715	R
	CACTTAC	37	2	113930840	CGGGGA	2	113647311	F
Red	GGGGCTC	37	19	519609	CGCGGGA	19	470609	F
	TCCGCAG	37	1	202003813	GAGATCT	1	200270436	F
Grn	TTGGCCC	37	8	22132874	CGCGGCC	8	22188819	R
	CAAGGGA	37	3	181721674	CGGTTAG	3	183204368	F
	CTCTGAA	37	5	131268130	CTTTTGG	5	131296029	F
Red	CTCCTCC	37	13	81229358	CCCGGCG	13	80127359	R
Red	ACATACA	37	7	37487352	CGCCGCC	7	37453877	F
Red	CTGTCCG	37	7	48129992	AGCCGCT	7	48096517	R
	GTGCTGT	37	3	168268421	CGAGCTC	3	169751115	R
Red	GAGGCAA	37	6	32632338	TAAGGCA	6	32740316	R
	TACCATG	37	8	1651128	AGCCACA	8	1638535	R
	CACGTAC	37	11	45698622	AGGAGCA	11	45655198	R
	CTCACCC	37	4	38825551	CCGAAAC	4	38501946	R
	TGGCAGT	37	1	243584861	CTGCTTC	1	241651484	R
Red	TCACCTC	37	3	11610214	CGCATCC	3	11585214	F
	GTTCCGA	37	3	64547373	TGTGTCC	3	64522413	F
	GATCACT	37	12	308821	CGGGAAC	12	179082	F
	GAGCCTC	37	11	1892092	CGAGGCA	11	1848668	R
	GAGGCCT	37	5	140481267	CGGTGGC	5	140461451	R
	ATACTCT	37	22	27006498	TGAGTCA	22	25336498	R
Grn	GACAGTA	37	13	25875133	CGCCCAG	13	24773133	R
	CCTTTTC	37	7	135662562	TCCCTCT	7	135313102	R
	GTGCGGT	37	1	228475463	CGCAGTG	1	226542086	R
Red	GCTTCAG	37	5	140794993	GGCTTGA	5	140775177	R
	TGTGCTT	37	2	175629224	CGGCTCA	2	175337470	F
	TGGGACT	37	5	140503914	GTTCAAG	5	140484098	R
Red	CCGTGCG	37	13	114193150	CGGGAGC	13	113241151	R
	TCCTAAG	37	15	101936156	ACGTTAA	15	99753679	R
	GTAAGTG	37	4	96051461	AGTTCCT	4	96270484	F
	ACAGCGC	37	4	2431998	CGGACAC	4	2401796	F
	AGCCCTA	37	11	84028582	CGGCAAC	11	83706230	R
	TCTGCAT	37	17	100892	CGGACTG	17	100892	F

	TAATAAA	37	12	54421335	ACCAACA	12	52707602	R
Grn	GCGTCGG	37	19	853540	CGCGGAC	19	804540	R
	AAGACCT	37	10	839609	GAGAATC	10	829609	R
	GTGCTTG	37	2	170834853	CGCCAAC	2	170543099	R
	AATAAAC	37	2	112391213	GGGAGAT	2	112107684	F
	CCGTGCG	37	16	88706389	CGGGGCT	16	87233890	F
	AAGGAAC	37	4	88142277	CGCGCAT	4	88361301	R
	TGACTTC	37	5	82771513	ATTGCTC	5	82807269	F
	TTTGGTG	37	2	240874769	ATTTAG	2	240523442	R
	AAAGAAC	37	12	105630720	CGGAAGC	12	104154850	F
	TCAAAGC	37	2	101508685	CGGCCAT	2	100875117	R
	GTTGGCA	37	3	23861312	CGTCCAG	3	23836316	R
	TGACCAG	37	5	14230166	CGTGCAA	5	14283166	R
	CCTGCAC	37	13	97927242	GACTATT	13	96725243	F
	GCAGTGC	37	4	119888794	CGGGTAT	4	120108242	F
	GTACTGA	37	11	20389052	CGACGTC	11	20345628	R
	CTTTTAC	37	12	177995	CCAGATC	12	48256	F
	ATATGAT	37	4	108744891	CGCAACT	4	108964340	R
Red	GGGCGCC	37	X	68835885	CGCCCCA	X	68752610	R
Red	AATGGGT	37	6	101847058	AAGCGGA	6	101953779	R
	AGACGTC	37	7	151553615	GGGAAGC	7	151184548	R
	TGGTCCT	37	8	144646939	CCGAGGC	8	144718082	F
	AGTTTGT	37	8	11663584	CAGGCTG	8	11700993	F
	TCAAGCT	37	4	6673551	AACAGAT	4	6724452	R
	TGATTTA	37	3	23710303	GGCTTCC	3	23685307	F
	AAGCCAT	37	2	1657483	CTGGCCT	2	1636490	F
Red	TGGAATC	37	1	116311395	CGCACGC	1	116112918	F
	GCCACAC	37	7	1097952	CGCACAC	7	1064478	F
	AGATAAA	37	13	30733388	TTTTCAC	13	29631388	F
	AATGGCT	37	15	86584249	AAGATGC	15	84385253	F
	AAATCCT	37	6	127785332	CGCTACA	6	127827025	R
	ATGAAAT	37	13	102276634	CGCTAAA	13	101074635	R
	AGTGAGA	37	1	240562805	CGGCTCC	1	238629428	R
Red	AGGGTCT	37	17	40835849	AGGAGGA	17	38089375	F
	GGAAACC	37	6	169978203	CGGAGCC	6	169720128	R
	GTGCTTT	37	2	236436519	CGGATAC	2	236101258	R
	GCCTCAG	37	6	32829062	GAGCTGC	6	32937040	R
	ACATCTC	37	9	123604514	CGCACAA	9	122644335	F
Red	GCGGCGC	37	5	140712459	CGGGCAT	5	140692643	R
Grn	GGAGAGC	37	7	63560711	GTGATCC	7	63198146	R
	TATATAT	37	8	97975467	CGTCACT	8	98044643	F
Red	AAGTGAA	37	11	2908036	TGTGTGC	11	2864612	F
	CGCAGTG	37	3	196048411	GCAGGAC	3	197532808	R

	TAGCCAG	37	6	36788637	CGATCCT	6	36896615	R
	AGCCAAA	37	2	239860241	AACAGCC	2	239525178	F
	AATATCT	37	6	28321301	TCCTCAG	6	28429280	F
	AGTCTTT	37	10	133796476	TGAAAGC	10	133646466	R
	AGAGACC	37	8	47751623	CGTGCTC	8	47870788	F
	CAAAGTC	37	3	13057143	AGTCAGA	3	13032143	F
	TGGACCG	37	5	140536238	CGCCCAG	5	140516422	F
	AAATGCC	37	6	136915367	CGGACCA	6	136957060	F
	GAAAGTC	37	7	34013871	CACCAGC	7	33980396	F
	CTTCTGG	37	1	201923663	CGAGGGC	1	200190286	F
	TTCAGTA	37	7	99137009	ATTTGCT	7	98974945	F
	GAGGCAC	37	4	25818940	AGTTTCT	4	25428038	R
	CTCTGAG	37	7	120657213	CGCAGAC	7	120444449	F
	GAACATT	37	1	204560985	CTTTCTG	1	202827608	R
	TCCCACG	37	17	4464294	CGGCCAC	17	4411043	R
Grn	TGCCCCA	37	6	30079265	TCTGCCG	6	30187244	R
	CCCTGAC	37	6	32549139	CCCTGTA	6	32657117	F
	CCCTGCT	37	17	77923275	CGAGAAT	17	75537870	F
	GGATCTG	37	1	9097241	AGGATGA	1	9019828	R
	CAGTCTC	37	14	53170147	CGGCTCT	14	52239897	R
	TTTGATG	37	6	1001966	TGGCCCA	6	946966	F
Grn	CGAGGCC	37	15	101593831	CGCTCCC	15	99411354	R
	TTATGCT	37	7	150381356	CGGATGC	7	150012289	R
	TTGATAC	37	14	94873879	CGGGACC	14	93943632	F
	AAAAAAT	37	11	114466093	CGGTGCA	11	113971303	R
Red	GGGAGAT	37	7	156337168	CGGCCAA	7	156029929	F
	GAGCTTG	37	3	66853898	CGGGATC	3	66936588	F
	CAAAACC	37	7	968493	GTCACGA	7	935019	F
	CCTTGTC	37	17	48268237	CGCCATC	17	45623236	F
	GGGTGTT	37	1	144989624	CATTGTT	1	143700981	F
	ACGGCAC	37	2	242805838	CGCCACT	2	242454511	R
	TGCTCCA	37	1	170344621	TGCTTGA	1	168611245	R
	GAGGCTC	37	3	71586357	TGAGTTC	3	71669047	F
	GCTGAGC	37	20	60692839	GTCACAC	20	60126234	F
	GTGGCTA	37	14	96526884	AATATTG	14	95596637	R
	GTTCAGA	37	17	33390736	TCATCTC	17	30414849	R
	GAGAACA	37	11	8931708	GGGGCAC	11	8888284	F
Grn	CAGTCCC	37	7	150026567	CGGTCCC	7	149657500	F
	AGAGGTA	37	5	77888194	CGCTCTA	5	77923950	F
	CACGCTA	37	17	19883326	GGATGAA	17	19823918	R
Grn	TTTGTAT	37	6	28447115	GCGGAGT	6	28555094	R
	CCACCTC	37	18	55289797	CTCTCCT	18	53440795	F
Red	GGAAGAA	37	19	48495301	CGCGTCG	19	53187113	F

Red	CGCAGTG	37	5	140182627	AGACTCA	5	140162811	R
	CCATTCA	37	7	1138716	CGTGACA	7	1105242	R
	CGCTGGC	37	8	10555382	GCATGGA	8	10592792	R
	ACCGGCC	37	8	135494242	CGCTCTG	8	135563424	R
Grn	GAACCGC	37	5	1594715	GTGGGAT	5	1647715	R
	GAGCTGC	37	2	36922916	AGTTACT	2	36776420	R
Red	GTCAGAC	37	15	45493106	CGGGCCT	15	43280398	R
	TCAGCGA	37	1	149162518	TTCAGGT	1	147429142	R
	TTCTCAG	37	2	879563	TTCATGA	2	869563	F
Red	GTCTAAG	37	1	200271342	GATTCGG	1	198537965	F
	TCTGATT	37	7	90879360	CGTGGGC	7	90717296	F
Grn	ATGGATC	37	19	52206258	CTGCAGA	19	56898070	F
	GCAGCCC	37	15	26487777	TCTGACC	15	24038870	F
	TTAACTC	37	2	145425418	CGTGGCA	2	145141888	F
Red	AGGGCTT	37	4	1594995	AGGCGCC	4	1564954	R
	ACAGCAC	37	17	948956	AGCATTT	17	895706	R
	GTATGTA	37	8	96805352	CGTCTGA	8	96874528	R
	GGATTAT	37	17	77351122	GGTGTTA	17	74862717	R
	CGCAGGA	37	16	20360217	CGCACAA	16	20267718	F
Grn	CTTCTGG	37	17	6411087	CGCAGGC	17	6351811	F
	ACACCTC	37	4	155312410	CGGAGAA	4	155531860	R
Red	AAGCCAC	37	6	138192658	ACTCAAC	6	138234351	R
	TTCGCTC	37	19	50861789	CGCAGCC	19	55553601	R
	ACCAGAC	37	12	41830372	CGGACAC	12	40116639	R
Red	GCACAGC	37	2	236674257	TCCTGCA	2	236338996	F
	CTGGAGA	37	8	1790861	CGGGCCT	8	1778268	R
	TTGCTAT	37	15	100121570	CGGTCTC	15	97939093	F
	TGTACAA	37	6	33037690	CGACAAA	6	33145668	F
	TACAAGA	37	2	241611931	CGACCCC	2	241260604	F
	ACATAGC	37	11	19081590	CTGGTGG	11	19038166	R
	AGAGTTT	37	15	66478674	CGGGGCA	15	64265728	R
	GGCAGAC	37	3	160168922	AGATGCC	3	161651616	R
Red	CAAGTAC	37	2	90016264	CTTGTTA	2	89653565	F
Grn	CTGCAGG	37	X	47004150	CGCGACA	X	46889094	F
	GAATTCT	37	3	63399168	CGCTTTT	3	63374208	R
	ACTTGGA	37	1	3335858	CGCACCG	1	3325718	R
	CACCTCT	37	4	6691093	CCTCCTC	4	6741994	R
	GAGGGTC	37	7	134879060	AAGGGAT	7	134529600	F
Grn	CGCCGCC	37	7	100203350	CGGCAGT	7	100041286	R
Grn	TTGCTAC	37	6	27661905	AAGCTTA	6	27769884	R
	ATAGCCA	37	7	102102804	CGGAAAC	7	101889809	R
	CCAGTCC	37	17	3499093	CGACTGA	17	3445842	R
	AAATTAC	37	17	41545935	CGAGCCA	17	38901461	F

	GGGCCTT	37	5	135270833	TGACTAA	5	135298732	F
	CACACGC	37	22	25758637	AGTGAGC	22	24088637	F
	TGATACC	37	11	130036542	AGTGAGC	11	129541752	F
	CCATCAC	37	11	48026889	CGTGGGC	11	47983465	R
	AGTCCAC	37	2	91758369	CGGCCCT	2	91122096	F
	TGCCTGT	37	13	114212078	TAAAGTG	13	113260079	R
	TTACTTCT	37	3	64547310	ATTCCTCT	3	64522350	R
Grn	CGGCGCA	37	6	28584138	CGCCCTA	6	28692117	F
	AGTTCAG	37	6	111907043	CGCCCAG	6	112013736	F
	TGAGGTC	37	13	59686676	CGTCACC	13	58584677	R
	AGAAGTC	37	15	90891614	TACGGAA	15	88692618	R
	TCCTCAA	37	17	68070411	CTTGGTT	17	65582006	F
Red	GCAACGC	37	6	88757358	GCACCCG	6	88814077	R
	TGGTAGG	37	11	64594647	CGCCCAC	11	64351223	R
	ATAGAAC	37	18	33078241	TTACTTTT	18	31332239	R
Grn	GTTGGTG	37	5	140208954	CAGTGCC	5	140189138	R
	TGGGCCC	37	3	65334190	CATCAGA	3	65309230	F
	CACCTTT	37	4	69536319	GCTTCCA	MULTI	MULTI	R
	AGGATTT	37	6	14729030	AAGGTCA	6	14837009	F
	CTGCAGG	37	10	1405937	GGAATGC	10	1395937	F
Red	CGGTCAG	37	18	12947723	CGTCCCG	18	12937723	R
Red	CTCCGCC	37	22	42395067	CGCCACC	22	40725013	R
	GGTAAAA	37	5	140552347	ATCCTTC	5	140532531	R
Grn	GAGGAAC	37	6	1594411	CGCGCTC	6	1539410	F
Grn	CCGCGCA	37	2	233323840	GGTGGGC	2	233032084	F
	CTCTAGA	37	13	102103051	ACATAAT	13	100901052	F
	CTGGATC	37	12	65155719	AAGATTA	12	63441986	F
	GGTTGGT	37	6	33128903	CGCCGTA	6	33236881	F
	ATCGGGT	37	5	175561172	CGCCCAC	5	175493778	R
	TGTCTTCA	37	11	113546261	GAAATCT	11	113051471	F
Red	GGCGCTG	37	19	15564105	ATGCTCA	19	15425105	R
	CACTGGC	37	17	76421442	CGGACTC	17	73933037	R
	CCCTCCC	37	13	99095684	ACGTGGC	13	97893685	R
Red	GTACTCT	37	17	69199815	GGACAAC	17	66711410	F
	TGGACTT	37	15	72519739	CGGTCTC	15	70306793	F
	TTCAGCT	37	8	103742123	GACCTCA	8	103811299	R
	ACACAAA	37	13	93990004	CGTTCTCT	13	92788005	R
	GGATGCT	37	1	27964774	CGGCACT	1	27837361	R
	CGGGCGA	37	3	36986992	AAGTACT	3	36961996	R
	GGATTGG	37	18	76751952	ATCAATA	18	74852940	F
Red	CTCCAGA	37	17	75789365	TCAAAGC	17	73300960	R
	AGGAAGA	37	2	134326204	CGCATGT	2	134042674	R
	AGAGAAC	37	12	45751145	CGGCCAC	12	44037412	R

	ATGTCAA	37	21	46822283	CGGGTTG	21	45646711	F
	GCTTGAA	37	3	133020205	AACTCCA	3	134502895	R
	ACATTCC	37	6	16713613	CGCCATT	6	16821592	F
	GGAGGGC	37	10	21462145	CGGGTGT	10	21502151	R
	ATCTTCT	37	13	30686841	CGGTCCC	13	29584841	F
Red	CCAAGCC	37	17	76417995	CGCCGCC	17	73929590	R
	TGAGAGC	37	2	207741102	CGGGTCA	2	207449347	R
	TCCTAAC	37	2	227560785	CGGCTGG	2	227269029	R
	CGAAGGA	37	5	180670469	CGCAGCT	5	180603075	R
	AGTGACT	37	20	60645197	TGAGCTT	20	60078592	F
Red	AGTAGTC	37	17	70636625	CCAGCGC	17	68148220	F
	ACATGGA	37	11	47639458	AAATATA	11	47596034	R
	TTGCGGT	37	17	72667062	CGCAGGT	17	70178657	F
Red	TGTCTGC	37	1	159001170	CGACCGA	1	157267794	R
	GTTTTGG	37	19	39265241	CGGGGAC	19	43957081	F
	TGGGATG	37	1	46420220	CGGGTGC	1	46192807	R
Red	CACAGCA	37	19	22891978	CGCCTTA	19	22683818	F
	GCCCGGC	37	19	48048583	CGGTCCC	19	52740395	R
Grn	CTCCCAG	37	22	46287028	GCGATGC	22	44665692	F
	GGACCTC	37	7	12151481	CGCCCTG	7	12118006	F
	CCTGAAG	37	1	33909498	CGTGTTC	1	33682085	R
	TTCCCTG	37	7	2953836	CGCCGGC	7	2920362	F
Red	GCTGGGC	37	1	27481531	CGCGGCC	1	27354118	F
	GTGACTG	37	1	231964048	CGAAGCA	1	230030671	R
	TGTGGAT	37	21	32129060	CGAGTGA	21	31050931	R
	TATTTGG	37	8	17220621	TAACAAA	8	17264992	R
Red	CACTCCC	37	13	113735031	CGGCGAT	13	112783032	F
	GGGCTTT	37	7	3215287	CTGCCCA	7	3181813	R
	TATGAGC	37	13	101860408	CGAACCC	13	100658409	F
	TACCACA	37	7	157997479	CGTTGCT	7	157690240	F
	TAAAAAC	37	5	112370191	CAGCGGT	5	112398090	R
	CGCAGGC	37	1	223926325	CGTGCAC	1	221992948	F
	TCACATA	37	1	38000170	CGTGCCA	1	37772757	R
	GGTCTCT	37	6	37958953	CGTGTGC	6	38066931	F
	AAGTGTA	37	17	78253912	CGGCCAA	17	75868507	R
	CTAATTC	37	2	235422165	CCCTGCA	2	235086904	F
Grn	GTCACGT	37	15	69754698	CGGCCAC	15	67541752	F
Red	GTATGGC	37	6	29895037	CGGTCCA	6	30003016	F
	ATGAAGT	37	5	54287462	AGCCTGT	5	54323219	R
Red	GAGCGAA	37	1	149155849	CCATGAT	1	147422473	F
	AGGGCTC	37	5	6434966	CGGGGCT	5	6487966	R
	AAGGAAA	37	19	17015427	CGCTCCA	19	16876427	R
	TTCTACC	37	12	69016159	CGGAGGC	12	67302426	R

Red	GAAAAG	37	2	91777482	GCTGGTT	2	91141209	R
	GGCAAG	37	7	158110152	CGGTGTG	7	157802913	F
Grn	GCGGGGA	37	7	5184014	CCAGAC	7	5150540	F
	TGCAGCA	37	6	101846779	GTAGAAT	6	101953500	R
	GAAACCT	37	3	182883005	CGCCCGG	3	184365699	R
	TTGTGCC	37	15	48732134	CTGTCCA	15	46519426	R
	GCCTCAT	37	12	9065171	ATTATTC	12	8956438	R
Red	GGGCCTC	37	19	52094375	CAGGCC	19	56786187	F
	AAGCTCT	37	19	11276529	AGGCTCT	19	11137529	F
	GGCCATG	37	5	180335743	CGGGAA	5	180268349	F
	AATGAA	37	15	78856949	CGGGTTT	15	76644004	F
Red	ACCCGCT	37	19	13409671	CCTGGAG	19	13270671	F
	AGACATT	37	9	123007424	CGGTTGA	9	122047245	F
	GACGGG	37	7	91763103	CGGGTCG	7	91601039	F
	CCTCTGA	37	7	94250352	CGGTGTG	7	94088288	F
	TTTTAGG	37	5	112810343	TTATTAT	5	112838242	F
	CCAAATC	37	2	46883368	CGCTCCC	2	46736872	R
Grn	TCCGCTA	37	20	13200929	CGCGGTG	20	13148929	F
	CTGTTCC	37	3	71439885	CAAGTAA	3	71522575	R
	TGATCAT	37	15	91912699	CTGCCCC	15	89713703	R
	CGCCGG	37	19	10172841	CGCCCCA	19	10033841	F
	AAGGAAA	37	10	61900940	CGGCAAT	10	61570946	R
	TCTCACT	37	19	53092499	AAGCAAA	19	57784311	R
	CTGGAA	37	1	20307906	CGGCTTT	1	20180493	R
Red	TTAGCTG	37	1	217203173	CGCAGCT	1	215269796	F
	ATGGAAA	37	11	123295944	CCAAAA	11	122801154	R
	TAGAGG	37	X	45016993	CGCTGG	X	44901937	R
	TTATTTG	37	1	80447732	ATCATTC	1	80220320	R
Red	GGAATA	37	6	32520615	CGAGGC	6	32628593	F
	TCTGCTG	37	4	141575212	AAAAAA	4	141794662	R
Red	TGTGTGT	37	10	3711048	GTGCAG	10	3701048	F
	CAGCTTT	37	7	48044273	GGTAGG	7	48010798	F
Red	CCCCGCG	37	12	115109934	CGCCATG	12	113594317	R
	TCGCCTA	37	8	134203841	TTGGAGT	8	134273023	F
	AAACCC	37	19	22990650	CGGGTAT	19	22782490	F
	TGCCAA	37	11	19876031	CGCAACA	11	19832607	F
Red	CTTCCTC	37	10	81214225	CCTTCCG	10	80884231	R
Red	TCCTCCT	37	2	233274144	GAAACG	2	232982388	R
	CATTGTT	37	5	140557256	CTGTATG	5	140537440	F
Red	GCTCTCC	37	13	39526272	CGCTGAA	13	38424272	F
Red	TTTGGGG	37	17	46608355	CCTTCCT	17	43963354	F
	AAACTCC	37	5	128795827	CGTCCAG	5	128823726	R
Grn	GCGCTCC	37	X	12156718	GCGTGG	X	12066639	R

	CCCATTT	37	1	248758128	CGTGACA	1	246824751	F
Grn	AAGTCTT	37	6	135203613	AGTAGAA	6	135245306	F
Red	TGCTATG	37	20	16552305	CGCTCTG	20	16500305	F
	ACCCGGA	37	6	1555080	TGGTAAC	6	1500079	R
	TGGCCTC	37	8	28175021	AGATGGT	8	28230940	R
	ATGCAGT	37	2	178499509	GCTGATG	2	178207755	F
	AGCAGAC	37	1	37732046	AAAATAA	1	37504633	R
	CAAGACC	37	10	120001266	TTCCGCA	10	119991256	F
	GCCTGAG	37	2	2570283	TTCATAC	2	2549290	R
	CTCAAGC	37	1	63249209	CGCCCGA	1	63021797	F
Red	AATCTCG	37	6	6737080	CTTCCGA	6	6682079	F
	CCTGTCT	37	1	246798258	CGCACCA	1	244864881	R
	ATGTGGG	37	2	61847725	CGACACC	2	61701229	R
	TCTGATA	37	21	46348691	CGTGGCT	21	45173119	R
Red	TAGACCG	37	1	228743131	CGCGGGC	1	226809754	R
Red	CGCTCTC	37	1	149186391	CCAGGCT	1	147453015	F
	CAAGGCT	37	3	137414342	CGGATCT	3	138897032	F
	CAGGAAC	37	X	30327819	AGGAGAC	X	30237740	R
Red	AGTGGTG	37	1	2254245	CGGGCTC	1	2244105	R
	AAGGGAC	37	15	53075421	GTGTGAG	15	50862713	F
	CCACTTG	37	10	28419523	ATTTTAT	10	28459529	R
	CTCCCTC	37	1	150554875	CGCCGTA	1	148821499	R
Grn	ACGCAGC	37	12	29542598	TGGGCGC	12	29433865	R
	CCCATAA	37	13	49853124	CGTGTGA	13	48751125	R
	CCATGTT	37	13	41593582	GGACCCA	13	40491582	R
Red	AGAAGGC	37	1	161410551	CGCCCTC	1	159677175	R
	TCCATGC	37	4	4993033	CGGACGC	4	5043934	F
	GGGGAAC	37	11	77532707	CGCCTCT	11	77210355	R
	TCGACAG	37	19	50931616	CGGAGCC	19	55623428	F
	TACATAT	37	6	150276311	CGTGTTT	6	150318004	R
	TGGGAGC	37	4	8633794	CGTGCTC	4	8684694	F
	CCACCCA	37	7	150020218	TATTGAA	7	149651151	R
	GTGTGGT	37	15	32206583	CGGCAGA	15	29993875	R
	GTAGGCA	37	2	135125439	ATGATGG	2	134841909	F
Grn	AGATAAC	37	12	58329936	GAACCCC	12	56616203	F
	GGGGGAC	37	13	24902376	GTGCCCA	13	23800376	R
	ATGATGT	37	20	24446527	CGGGCAC	20	24394527	F
	GAAGCCA	37	5	177612982	TACACCA	5	177545588	F
	TCTTACTA	37	19	35720295	CTGGGCA	19	40412135	R
	CCACCTT	37	4	26198975	CAGTGTG	4	25808073	F
	CAAGCAA	37	6	31034042	ACCAGAC	6	31142021	R
Red	GAAACAA	37	17	981643	CGCCGCC	17	928393	F
Grn	CTGTGTT	37	14	65068635	GAAAAAT	14	64138388	F

	AGATTCG	37	12	128869988	CGAGACA	12	127435941	F
Red	AAACCCC	37	17	43037114	CGCCCGG	17	40392640	R
Grn	GAGCGCC	37	5	140503531	CGCGGAC	5	140483715	R
Red	TCATGTT	37	13	111935508	GTAGGCC	13	110733509	F
	AGTGACC	37	8	142452509	CTGGGCA	8	142521691	F
	TGCTAGC	37	1	31732891	CATCCAA	1	31505478	R
	GGAGACA	37	1	164682021	CGGAACA	1	162948645	R
	AGAAGGT	37	3	124780157	GTTGCTA	3	126262847	F
	TTAGAGA	37	7	150765472	CGGTGGT	7	150396405	R
	CACCTTC	37	6	32713827	CGAGCCT	6	32821805	F
Red	CATTGGG	37	6	29556225	GTCGAAT	6	29664204	F
Red	AAACACC	37	8	6633067	CTGGAAA	8	6620477	R
Grn	AGATCGC	37	X	47077986	AGTACTA	X	46962930	R
	TCCCTCC	37	X	56258808	CGGCCGC	X	56275533	F
	ACCATTC	37	11	2925623	GGGCTTG	11	2882199	F
	ATTGTTG	37	3	158189615	AGAAGCA	3	159672309	F
	TCCAGAC	37	11	88288530	GAATCAC	11	87928178	R
	GCTGTGC	37	15	25434030	CGGCCAA	15	22985123	R
	CAGGCGC	37	6	101847388	TCTGCCT	6	101954109	R
Red	GCCACTC	37	2	72371255	CAACGTG	2	72224763	F
	TTTGGCC	37	6	32774788	TCCACTC	6	32882766	R
Red	GCAGACT	37	5	28928346	CCAGCAC	5	28964103	F
Red	CGCGAGC	37	7	5862983	CGCCCCC	7	5829509	R
	GTTTTTCA	37	2	173647945	CGGCCGA	2	173356191	R
	CTCTCGC	37	6	33288296	CGGCTGG	6	33396274	R
Red	CCCACCT	37	5	126409198	CCGGCGT	5	126437097	F
	AGGCATT	37	11	84659234	CACCCCT	11	84336882	R
	GTCACCA	37	8	6875661	CGGCCCC	8	6863071	F
	AAACCCA	37	13	44453235	CAGTCTC	13	43351235	F
	CAGCGGC	37	22	51001387	TTCCCGA	22	49348253	F
	TTAACTA	37	3	108021293	CGGAATT	3	109503983	R
	GGTAAGC	37	5	180581761	TGAAGCT	5	180514367	F
	AATGTGT	37	4	124430271	CGGTTTA	4	124649721	R
	ATTTATG	37	6	32605704	TCATTCT	6	32713682	R
	CCTCCAC	37	6	136257948	AGGATCC	6	136299641	R
	CTTCCAT	37	10	90752569	CCCTCTA	10	90742549	R
	TTAAGCT	37	11	55606216	GTGGCCT	11	55362792	F
	AGTAAAC	37	22	31498218	AGATCCT	22	29828218	R
	TTTGTGA	37	20	19266250	TTTTTTTT	20	19214250	R
	TAGTGTT	37	5	140572766	CGCTGGA	5	140552950	F
	TTGACTC	37	10	128590997	ATGGCCC	10	128580987	F
	TGTGATG	37	12	31771672	ACCTAGG	12	31662939	F
Red	GCCCAAC	37	17	56566398	CGGTATC	17	53921397	F

	AGAGGGA	37	12	15983280	CAAGTCA	12	15874547	F
	AGCTCAG	37	7	157406032	GACTTAT	7	157098793	R
	CTGGGTA	37	2	47572939	CGTTCCA	2	47426443	R
Red	AGGAGGC	37	2	150037200	CGCTGCC	2	149745446	F
Red	CGGTGCT	37	2	72371261	CGCGTAT	2	72224769	F
Grn	CGTGGCG	37	7	64043119	GAGTGGC	7	63680554	R
Red	TGGGTCT	37	3	194790434	AAGGAAC	3	196271723	R
Red	AGAGGTC	37	20	56725871	CGCGGCC	20	56159277	F
	TGTTCCTC	37	19	22891847	CTGAGAT	19	22683687	R
Red	TGACCTC	37	19	4784940	CGCGCGC	19	4735940	F
Red	GGTTGTT	37	3	49756301	AGTAGAC	3	49731305	R
	TAATGTC	37	19	33719749	TCCATTA	19	38411589	R
Red	TGGAGGC	37	2	239012831	CGGGCTC	2	238677570	R
Grn	CTCGATC	37	14	56979854	GAGAAGC	14	56049607	F
Grn	CAGGCCC	37	11	2891065	TATGGGC	11	2847641	F
Grn	CAGCTTT	37	15	101862689	GAAATGC	15	99680212	R
	TAGCCTT	37	1	18700603	CGCCCCA	1	18573190	F
Grn	GACTCCA	37	14	102050627	AAATGTT	14	101120380	F
	GCCGCTA	37	14	97493395	CGGGAAC	14	96563148	R
	CTATGGG	37	5	10091925	CGAGCCC	5	10144925	R
	CCACACT	37	14	52733243	CCAATTT	14	51802993	R
	TATGGGA	37	1	244545617	CGGGAAA	1	242612240	F
	AGTCTCA	37	12	64575346	CGCATGA	12	62861613	R
	CTTCCCT	37	1	149155775	TCCTCGC	1	147422399	R
	TATGAGA	37	10	91136753	GTAATGA	10	91126733	R
	CCAAACC	37	4	190587808	CGGGGAA	4	190824802	F
	GAGAAAA	37	3	105466016	TATGCTG	3	106948706	F
Red	CCACGCA	37	7	158075705	TGAGGCC	7	157768466	F
Red	AGCCAGC	37	1	33631017	CCTGGCC	1	33403604	F
Red	GGGGGGC	37	2	95540265	GGGCCGC	2	94903992	R
	ATCCAAG	37	11	106269962	CGCACTC	11	105775172	R
Red	CATCTGG	37	7	158032496	CGCTTCC	7	157725257	F
	CTCCAGA	37	6	6543402	TTTCTTC	6	6488401	R
	GTAGGCA	37	5	140718731	CGGACTC	5	140698915	R
	AAGCAAT	37	15	25145254	GTCCTTA	15	22696347	F
	AGTACAT	37	10	28975991	CGCTGCA	10	29015997	R
	CCCTGTT	37	1	13111908	CGTGGTC	1	13034495	R
	ACCCCCG	37	7	138349443	CAAGAGA	7	137999983	F
	GCTCGGG	37	8	144635444	TCTGCCC	8	144706587	F
	ATTTTAC	37	2	172650722	CGCTTAC	2	172358968	R
	CTCACCC	37	10	134920382	GCTCAGA	10	134770372	R
	TCTAAAG	37	1	149162430	ACTACCT	1	147429054	F
Red	TGGCACC	37	1	41304105	TCTGTGC	1	41076692	F

Red	CCTCTGC	37	1	6526398	CGCGTGT	1	6448985	R
	CGGGCGC	37	X	27882053	TTAGATA	X	27791974	R
Red	CCCATCT	37	17	4440116	GGTGCAT	17	4386865	F
	GTGGAGC	37	2	175922785	CGCAGTG	2	175631031	R
	TAAAACC	37	10	132909289	GGTGCCA	10	132799279	F
Red	GACGCAC	37	2	26950956	CGGCGCC	2	26804460	F
	GCGGTGC	37	3	14615941	CTTGAGC	3	14590945	F
	ATAGACA	37	2	114033830	CGGCAAA	2	113750300	R
Red	AAACAAA	37	12	132845336	CGCCCAA	12	131355409	F
	TTATCAA	37	12	2690385	CGCAATC	12	2560646	F
	GAAATCC	37	7	157345746	GTTATGT	7	157038507	R
	TAGCTCC	37	5	166938213	TCTGATT	5	166870791	F
	GCAAGTG	37	13	114775133	CAAAGGC	13	113793235	F
	TGGACAC	37	5	174771462	ACTTCTC	5	174704068	R
Red	TGGAATG	37	1	227635558	AAAATTT	1	225702181	R
Grn	CCCCAGC	37	1	86081894	GGAACCT	1	85854482	R
	AGATTCT	37	10	3503185	AAGCCAC	10	3493185	R
	AACGTCC	37	5	177823864	TCTGCTC	5	177756470	F
Red	CCTGTGA	37	3	4344661	CGTTCTA	3	4319661	F
	GCCTGGC	37	7	1099788	CGGCCAC	7	1066314	R
Red	CGCAAAA	37	11	67383651	AGCCTCC	11	67140227	F
	TCTCATC	37	6	33037640	TGTTTTC	6	33145618	F
	CCCACTG	37	1	33793808	ATCTGTG	1	33566395	R
	GAGATGA	37	13	21728944	CGCCCCT	13	20626944	R
	CTAAGCA	37	1	232086152	AAAAATA	1	230152775	R
	CTTTCCA	37	4	95680414	CGGAGGA	4	95899437	F
	ATGACAC	37	2	176928222	TAATGCT	2	176636468	R
	GCCTAAG	37	5	157003983	TCAGCAA	5	156936561	R
Grn	CTAACCA	37	X	70752794	CGGGTTG	X	70669519	R
Grn	TCCAGAC	37	1	2885244	ACACTGT	1	2875104	R
	GGCTAAC	37	2	12674976	CACAAAA	2	12592427	R
Grn	ACCACCG	37	19	2250956	GGGCGGC	19	2201956	F
	GCTCTAT	37	14	59109856	CGTGGAC	14	58179609	R
	CAGCACA	37	2	9518751	CGCTGGG	2	9436202	F
Grn	TCCGCGC	37	2	98206733	CGCTGGC	2	97573165	R
Red	CCCGCGC	37	1	247537224	GTCCCAG	1	245603847	F
Grn	AATAGAC	37	7	47976869	TCGTCAC	7	47943394	F
	TTTTGGT	37	7	20198439	AGGAGCA	7	20164964	R
	GCAAGGT	37	4	47427537	CGATCGA	4	47122294	F
	CTCAGGG	37	2	5605864	CGGTATC	2	5523315	F
Red	GCGGAGA	37	X	25025519	TCTGCCT	X	24935440	F
Red	TCCCTCC	37	5	6633531	CCCGCAC	5	6686531	F
Red	CTCTGAG	37	6	106434169	AGATGCC	6	106540862	R

Red	TCAGCTC	37		5	140530410	GAGTTGG		5	140510594	F
	CTCTTAC	37		5	179741104	CGCTCAA		5	179673710	F
Grn	GCTTTTT	37		6	28661310	CGGGTAC		6	28769289	F
Grn	TGTCCAA	37		14	93252030	GCAACCC		14	92321783	F
	ATTCATT	37		3	188888584	CTCTCCT		3	190371278	R
	GTCTTGT	37		13	42848367	AGCTCAT		13	41746367	F
	ACTTAGT	37		1	32264315	AGGGAGC		1	32036902	R
Grn	AAGGACC	37		1	18809531	GCCGAGA		1	18682118	R
	GTCTGTT	37		8	23656074	CGGCTCA		8	23712019	R
	GCAGGGC	37		14	102554846	CGCTTTA		14	101624599	R
	TTTCTGT	37		4	38006787	CGCATCC		4	37683182	R
	TCATCTG	37		1	44287964	CTTGAAG		1	44060551	R
	CCAAACA	37		5	147777756	CGCAATA		5	147757949	F
Red	GATAATG	37		7	158159286	CGCAGAC		7	157852047	F
Red	CTCTTCC	37		7	157406096	TTCTGGC		7	157098857	R
	GGTAAGC	37		8	22561893	CATATGT		8	22617838	F
	GCTATTC	37		17	31888899	AAGATCT		17	28913012	F
Grn	GGGGAAC	37		7	154706386	CCCCCGG		7	154337319	R
Red	ACTCCTG	37		14	101539489	CTGGGGC		14	100609242	F
	TCAATGT	37		6	33039606	CGGGAGA		6	33147584	F
	TCACTGA	37		6	33050741	CTCCAGG		6	33158719	R
	TCTCTGC	37		6	32634344	CGGGTAC		6	32742322	R
	TCCCCTG	37		3	73620925	GCGGCTC		3	73703615	R
	CTGGCTC	37		2	109803622	CCACAGC		2	109170054	R
	AAAAGCA	37		6	148079619	GAAATAT		6	148121312	F
Red	CCCCCTG	37		10	1120539	CGGCAGC		10	1110539	R
	GATGTTG	37		2	150179794	CGAGGGC		2	149888040	R
Grn	CAGCGCC	37		14	44002338	TGGGTGG		14	43072088	R
Red	CCGGCGC	37	X		125714973	GGTCGAC	X		125542654	R
	TTCCTGT	37		2	105877824	AGAGAAC		2	105244256	F
	CCAAAAC	37		1	203275927	CACTGGC		1	201542550	R
	GGGTGCT	37	X		12993075	CGGGGTC	X		12902996	R
Grn	CGGGCTG	37		12	2339513	GAGCCGC		12	2209774	R
	TTCCCAG	37		2	80688129	GTTGACT		2	80541640	R
	GGCTTCG	37		10	135342560	CGCTTCA		10	135192550	F
Red	TGAGTCA	37		8	141248667	CGCGTCG		8	141317849	R
	TGCCATT	37		2	178178939	CGGCATT		2	177887185	R
	CCTGCCA	37		11	123892725	CGGTGAC		11	123397935	F
Red	CGGCCGA	37		6	74064064	CGCCCCC		6	74120785	F
	GGGCTGA	37		14	24665079	TCCTGGC		14	23734919	F
	ATAGCAC	37		2	9816010	CGGGAAA		2	9733461	F
	TGGAGCA	37		2	10942687	TTCTACA		2	10860138	R
	AGTATCT	37		14	78758042	CGCAAAA		14	77827795	R

	CTGTTCG	37	14	102050815	GCAGAA	14	101120568	R
	GGGATTA	37	14	106892175	AATGCAC	14	105963220	F
	ATTTTAA	37	14	80440817	TACGTGT	14	79510570	R
	AATTTAT	37	15	50140549	CGTCTCT	15	47927841	R
Red	ACAAAGC	37	4	187126114	CGTCCCA	4	187363108	F
	GAGTCTG	37	5	536783	CGCTCAG	5	589783	R
	CTCTCAC	37	14	104770790	CGGAACA	14	103841835	R
	GAAGAGC	37	6	32633163	GGGCTGA	6	32741141	R
Grn	CGCAACC	37	5	176543964	CGGCCCA	5	176476570	F
	GCTTTAA	37	2	130989361	CGCCAGC	2	130705831	R
	GGTCCTT	37	11	89655871	CGGCTGT	11	89295519	F
Grn	CCGCCAG	37	5	218452	CCCCGGT	5	271452	R
	ATCGCAG	37	7	1142861	AAGCTTT	7	1109387	F
Grn	GAGTTCC	37	5	140626788	GGGTACA	5	140606972	F
	TGGAGCT	37	7	19417585	ACAGCTA	7	19384110	F
	GCCCATG	37	3	195870009	CAGGGTC	3	197354406	R
Red	GGGAGGC	37	17	9129824	CGTGGGC	17	9070549	R
	GGGCTTC	37	17	38677884	AGTCATC	17	35931410	F
	CTGGTAG	37	10	65670534	GGGTGCA	10	65340540	R
	GCGGGCA	37	8	127570482	CGGGGGC	8	127639664	F
	TGGGACC	37	5	6581849	GCTCTCA	5	6634849	R
Grn	TGAGCTC	37	20	62052997	CCACAGA	20	61523441	R
Red	GGCAGAA	37	2	37617601	CGAGCGA	2	37471105	F
	GCTGGAG	37	10	133038915	CCTTCCC	10	132928905	F
	GGGAAGC	37	14	23623684	CGGTGCA	14	22693524	R
Red	CGGCTCT	37	7	5184155	CGCCGTT	7	5150681	F
	TGCCTAA	37	12	80427292	CGGCATA	12	78951423	F
	TTTGTTT	37	17	6546898	TGACTCC	17	6487622	F
	GTGAGTA	37	1	204951209	CGCTCAC	1	203217832	F
	TGATGAG	37	5	83429	CGAGGCT	5	136429	R
	GGAGAGC	37	13	51482661	CGCCTCT	13	50380662	R
	GCAGGCT	37	14	85292067	CGCTTGG	14	84361820	R
	AATTTTT	37	6	112409052	CGGGACC	6	112515745	F
	GGAGGCC	37	12	75784862	AGGCTTG	12	74071129	R
Red	CCTGATC	37	7	157102769	ATGCATC	7	156795530	F
	TGGATTG	37	12	114851495	GTGCAAA	12	113335878	R
	AAAATTG	37	12	70092840	CGGGAGT	12	68379107	R
	AGTTTGA	37	11	46879516	CGATCAG	11	46836092	R
	ACTGGAA	37	5	140530222	CTTGCAG	5	140510406	R
	TCCGTGC	37	6	157198648	CCTTGCC	6	157240340	R
Grn	AAAAACA	37	1	149298587	AAACAAA	1	147565211	R
	CTCTGTT	37	2	102666444	CGGCACA	2	102032876	R
	GATTTCT	37	X	133931641	CGCAATG	X	133759307	F

Red	AGGAGTC	37	17	40706682	GTAGCGC	17	37960208	F
	TCTAGGG	37	13	111089337	TGTTCCC	13	109887338	R
	CCTCCCT	37	15	101587658	CTCTGTG	15	99405181	R
Grn	CACAGTG	37	4	967822	CGGAAAC	4	957822	R
	CCCCTCC	37	6	32828996	CGTCAGG	6	32936974	R
	TACCAAA	37	8	43101403	CGCATGC	8	43220560	R
Red	AGATCCT	37	22	25160033	AGTGTGC	22	23490033	F
	ATATGAG	37	4	11631423	TCTCCAG	4	11240521	F
Red	TGTTTAG	37	2	200775458	CGGGGCC	2	200483703	R
Red	CACCCGG	37	6	88757371	CGCCGCC	6	88814090	R
	AGGAAAT	37	1	47901632	CGTCCTC	1	47674219	R
	GCACTGC	37	3	187467427	CGAACAC	3	188950121	R
	CCGCCAG	37	3	140660432	CGCCCCT	3	142143122	R
Red	CGACTTC	37	11	118842650	CCGCCTC	11	118347860	R
	GATAAGC	37	1	180166190	AATGGGC	1	178432813	F
	CAGGGGA	37	17	37560925	GTGAGTC	17	34814451	F
	TCACTGC	37	12	131715734	CGTCAGA	12	130281687	R
Red	TGCCACG	37	2	202004875	GCCAGGC	2	201713120	R
	GCGCAAC	37	6	29895175	CGCACGT	6	30003154	R
Red	CAGCAGC	37	11	3254065	CGGTCTG	11	3210641	R
	TAGAGAA	37	10	115086103	CTATAAG	10	115076093	F
	TACTGTT	37	6	159392353	GCCAGAA	6	159312341	F
	AGTTGTC	37	11	94036363	ACCTATT	11	93676011	R
	GCCCTGC	37	7	47445681	TCCTGAT	7	47412206	R
	CCTTTCCA	37	10	121346762	CTCCTGC	10	121336752	R
	ATCCGAG	37	13	32835962	GGACCTC	13	31733962	R
Grn	GGCTCAA	37	6	30039132	GGCTGCG	6	30147111	F
	CCAGCAC	37	10	134910285	CCTGGGG	10	134760275	F
	GGAGTTT	37	7	16966323	CACAGAT	7	16932848	R
Red	TGTTCAC	37	17	48270089	CGGCCTC	17	45625088	R
	TAACAGT	37	6	32409386	TTCCCAG	6	32517364	F
	GGGCGGC	37	1	839752	CGCCCTG	1	829615	R
Grn	TTCAAAG	37	6	36930021	ATGTTAG	6	37037999	R
	CCTCCAC	37	15	96890880	CGACCGC	15	94691884	R
Grn	CGGCAGT	37	17	41437982	TCTGCAG	17	38793508	R
	CTTTGCA	37	22	29225779	CGGTGAT	22	27555779	F
Red	GGGAAGA	37	6	30039006	TCCCCCA	6	30146985	R
	CTCATCA	37	4	172509909	CATCAAC	4	172746484	R
	AAATAAT	37	1	152333596	CATTTTC	1	150600220	F
	AAAAAAA	37	2	238252272	CGGAGAC	2	237917011	R
	CATCTTG	37	4	95799256	CTTAAAG	4	96018279	F
Red	ATGTCCA	37	17	39240789	CGCCCCA	17	36494315	F
	GCGTAGA	37	12	133177546	ACGGTGC	12	131687619	F

Red	CCGCTGC	37	22	30685664	TTCTGGC	22	29015664	F
	CAGCTGG	37	17	70710154	CGGCCTT	17	68221749	R
	AATTTGG	37	17	74868604	CGAGCAC	17	72380199	R
Grn	CTCCGAT	37	6	10720455	CGCCCAC	6	10828441	F
	GTAGTGG	37	15	80189782	CGAGTCG	15	77976837	F
	GGGGCCA	37	2	236409849	AGTTATT	2	236074588	R
	AGTTTTC	37	5	43411929	CCATAGA	5	43447686	R
	ATGAGAC	37	14	96722482	CGGCAAT	14	95792235	F
	CATATGT	37	6	167175937	CGGCACC	6	167095927	F
	GTGGTCA	37	6	170467487	GCAGCCA	6	170309412	R
Red	GGGGGCC	37	19	292271	AGCTGGC	19	243271	R
	GCATATA	37	12	124827315	CGGCAGC	12	123393268	R
Red	GGAGAAA	37	8	59058656	ATGAAAC	8	59221210	R
	GAGCCCC	37	1	12606996	CGAGCAC	1	12529583	F
	GGCATCT	37	10	27639136	GGGCAGC	10	27679142	R
	GACTCAG	37	1	227684434	CGGCACA	1	225751057	R
	ACATTTA	37	6	32546626	CGGTTTA	6	32654604	F
	AATCTTG	37	6	32549935	ATCAAAA	6	32657913	F
Red	AAATTAG	37	X	71401384	GTGAAAC	X	71318109	R
	ATAACCA	37	15	101206599	AGGAACT	15	99024122	R
	TCTTTCCA	37	1	161368787	CGTGACT	1	159635411	F
	ATTTCTG	37	5	140479025	ACAACCT	5	140459209	R
Red	AGTTGTC	37	2	88583805	CGCCTCC	2	88364920	F
Grn	GCTATGA	37	1	228890884	TCCGAGG	1	226957507	R
Red	GAGAGGC	37	19	46996713	CGCAATC	19	51688553	R
Red	ACTGGGC	37	9	140312446	CGGCCTC	9	139432267	R
	TCCTGAC	37	1	114605704	CGCAAAA	1	114407227	R
	CCATAGG	37	12	25056083	CGCAACC	12	24947350	F
	GACCCAA	37	1	7762151	CGAGCCC	1	7684738	F
Grn	TTGGTTT	37	4	940644	AAGGTCA	4	930644	R
Red	CGGGAGC	37	7	56437746	CGGCCTC	7	56405240	R
	AAGCCAT	37	2	160058036	CGAGCAA	2	159766282	R
	CGTTGCG	37	15	20150114	TGACCTG	15	18410128	F
Red	ATGTGGC	37	17	79485043	CGGCTTC	17	77099638	F
Grn	GGCGGGA	37	X	9983205	GGGAGCC	X	9943205	R
	GAAGGAC	37	8	103750999	GTCGTGG	8	103820175	R
	GTTATTA	37	3	186928338	CGTGCTT	3	188411032	R
	TCTGGTT	37	17	80006606	CGGTGAC	17	77599895	R
	TATCCAC	37	8	107058373	TACTGTT	8	107127549	F
	GGACACA	37	5	140625794	CGGTGGC	5	140605978	F
	GCAGGTT	37	13	22560659	ACGTCTC	13	21458659	F
	CTAGGGA	37	1	115732999	GGAAATC	1	115534522	R
Red	AGCGCCC	37	X	68385233	CGCTTTT	X	68301958	F

Red	TGCCCCC	37	19	594272	CACCGAC	19	545272	R
Grn	CTCGGCC	37	X	19140934	CGGGACC	X	19050855	F
Grn	ATAAGAC	37	2	206842760	AAGCAGC	2	206551005	R
	AAGAGCC	37	19	526333	CGGACCC	19	477333	F
	CTGTGAA	37	11	65196222	TCTGAGT	11	64952798	R
	CTAAGCC	37	8	142192568	CGGTCAA	8	142261750	R
	CGTCGCC	37	Y	9194584	GAGAAAC	Y	9254584	R
	TTCTGGG	37	6	39290715	CGCCCCC	6	39398693	F
	GATGGAC	37	6	31106235	CTGAACT	6	31214214	R
Grn	TCCTCCT	37	19	37797500	AGGAGCC	19	42489340	F
	AAGAGCT	37	16	30485383	AGCTCTA	16	30392884	R
Grn	GGCGGGT	37	14	101005128	GCGGTCG	14	100074881	R
Red	ATTCTCA	37	11	11606082	CAAGGGC	11	11562658	F
Red	CGCTCTC	37	X	153095663	CGCGCCC	X	152748857	F
	TGCGGCT	37	2	88427630	GAACCTG	2	88208745	F
	CCCATCT	37	10	111912029	CGCTGGC	10	111902019	R
	TTTAGAT	37	1	39340282	CGTGATT	1	39112869	F
	TCACCGC	37	17	212481	CGGACAC	17	212481	F
	TCCAGGC	37	6	30419401	TGTGTCC	6	30527380	R
	TAATTTA	37	13	103478447	AAGAATC	13	102276448	F
	TATACTA	37	17	69357976	CATCCTA	17	66869571	F
	GCAGCCG	37	1	18808559	TGCAGCT	1	18681146	F
	AATCCCC	37	4	54357010	CTGGTGT	4	54051767	R
	AGGCTGC	37	19	40372977	AACTCCT	19	45064817	R
	AGTGCGC	37	6	32797488	CGGAGGC	6	32905466	F
	AACTCCC	37	1	63249199	CAGATTA	1	63021787	F
	GTGGCTC	37	10	6316599	CTTTTTA	10	6356605	F
	TCCACAG	37	6	170467790	CGGGAAT	6	170309715	F
	GGCTCTT	37	6	21916568	ATTGCTA	6	22024547	F
Red	CCCGGTC	37	7	1311498	CGGGCCT	7	1278024	F
	TCCCTCC	37	6	29649024	GCATGGC	6	29757003	F
	CAAATGA	37	6	30039801	CGAGGGA	6	30147780	F
Red	CCAGTCC	37	6	28584167	GGTGGCC	6	28692146	F
	ACCTCTG	37	5	167899230	GGCAGCA	5	167831808	R
	ACGTCAA	37	5	114791124	GAATGTC	5	114819023	F
	CCATACT	37	1	183116950	CTTTTTC	1	181383573	R
Red	GGCTGTA	37	7	4876057	GCAGAAC	7	4842583	R
Grn	GCTTGGG	37	4	581937	GACTTGG	4	571937	R
	AAACACT	37	5	140579644	CGAGCCT	5	140559828	F
	CAGTGCT	37	12	3019650	CGGTTTT	12	2889911	F
	GAGCTTT	37	5	179982651	AGATGAT	5	179915257	F
	TTTCCTTT	37	1	200558537	CGGTTTT	1	198825160	R
Red	TCGCACC	37	X	107019430	CGCTTCG	X	106906086	F

Grn	ACAGGCT	37	7	158122151	GGAGCGT	7	157814912	F
	ACTGGTA	37	17	14989749	CGGCTCC	17	14930474	R
	CAGAGGA	37	6	33137570	CGGGGAC	6	33245548	F
	AGTCACA	37	8	48675771	TCACTGC	8	48838324	F
	ACCCTGT	37	5	78282669	CGGGGGA	5	78318425	R
	TAATGCT	37	13	106971568	GCTCTTG	13	105769569	F
Grn	ATGACTC	37	3	15311269	CGGCAGT	3	15286273	R
	GTGGGAC	37	10	123900861	AGCCAAA	10	123890851	F
	GGGGCAC	37	17	74074118	TGAGCAA	17	71585713	F
	CACCATG	37	Y	9384015	CGGTGCC	Y	9994015	R
Red	CTCCGGG	37	7	55431084	TCTCCGC	7	55398578	R
Red	ACCTGGC	37	7	2643340	CTTTACC	7	2609866	R
Grn	GCGTCCT	37	19	292131	CGCTGAT	19	243131	F
Red	GAGGCGC	37	19	17439877	GCGGAGT	19	17300877	F
	AGCCACC	37	13	24365000	CGAGAGC	13	23263000	R
	GAAGCCA	37	19	52133837	ATTTGAG	19	56825649	F
	GAGGTCT	37	7	22122872	GGGGACT	7	22089397	R
	CTGTTAC	37	19	46527410	TGTGCAC	19	51219250	R
	ACTGCAG	37	19	18761792	AGGGGCC	19	18622792	F
	GCACCAC	37	10	93998677	TACACAT	10	93988657	F
	AGTGGTA	37	10	43084809	CGGAAAA	10	42404815	R
	AAGAAGC	37	12	131258414	AAAGACC	12	129824367	F
Red	GGGGCTG	37	10	77871958	CTGGAGC	10	77541964	R
	TGATGTT	37	3	42139507	CGAAGGC	3	42114511	R
Red	GGAGGTC	37	10	12999599	CGCACCA	10	13039605	F
	TCACTCA	37	6	33041268	CGGAGAC	6	33149246	F
Red	CCCTCAG	37	6	170231220	GGATGCA	6	169973145	F
	GTAAGAC	37	1	212434331	CGCAGTA	1	210500954	R
	CCCGATT	37	6	166577958	AGAGGTT	6	166497948	R
	CACCAAC	37	12	10096152	GGTGAGC	12	9987419	F
	TGAGATC	37	1	169079632	CGAGTCA	1	167346256	R
	ACATCGA	37	12	8995965	CCGATGC	12	8887232	R
	CAAATAC	37	1	159748210	GCCACAT	1	158014834	R
	GTCAGCC	37	17	40489584	TTTCTGA	17	37743110	F
Grn	AGCTCTC	37	10	25238461	CCTCCTG	10	25278467	R
	GAGAGCC	37	11	27406919	CGGAGGT	11	27363495	R
Red	CTCCCCC	37	19	49891574	AACTGCC	19	54583386	R
	TAAATCT	37	1	184943911	CGTGATC	1	183210534	R
Grn	TCCGCTG	37	6	30038955	ATGCGGA	6	30146934	R
	CAAACCT	37	8	117457111	ATTGAAA	8	117526292	R
Grn	AGGCCGA	37	21	45842649	CGGCGCT	21	44667077	R
	TGAAAAC	37	3	47138673	CGGTGTT	3	47113677	R
	CTCGCTC	37	7	27163929	GGTAATG	7	27130454	F

	AATGTGT	37	10	131215469	CGGAGTC	10	131105459	R
	GTGAGCA	37	15	25430653	CGTGGCT	15	22981746	R
Grn	GCGGCCA	37	2	162197869	CATAAAA	2	161906115	F
	TACAAGT	37	1	161931993	CGGAGGT	1	160198617	F
Red	CCCCGGC	37	3	42947690	CGCCCCA	3	42922694	R
	GTAACCA	37	3	97591258	TCTTTGC	3	99073948	F
Red	TGTGGAC	37	12	2760969	CGCAGGC	12	2631230	F
Grn	CGGGACC	37	7	55517045	CGGCCCT	7	55484539	F
	CCAAGAA	37	11	88153520	AATGAAC	11	87793168	R
	TTGGGTC	37	17	80914695	CGGGGAC	17	78507984	R
	TAGGAAT	37	17	40808068	CGGAGTC	17	38061594	F
	TGTTAAA	37	19	5510310	CGGGGCC	19	5461310	R
	GGCCAAC	37	18	11947936	CGGTCTT	18	11937936	F
	CATCCCA	37	13	20764859	TGAATTG	13	19662859	R
	GAGACTA	37	17	76588724	GACTGCA	17	74100319	F
Red	ACTCTGC	37	10	82295723	CGGGGAC	10	82285703	F
	ATGATTT	37	21	44103949	CGGGGGA	21	42977018	R
	TTGGCAG	37	1	32177995	CGTCTTT	1	31950582	F
Red	TCTTTTCT	37	19	292102	CGAGCTT	19	243102	R
	CAACCGC	37	1	155167337	CGTAGTC	1	153433961	R
Grn	GCTAGTC	37	1	179713828	CGGGCCT	1	177980451	R
	TGATTTA	37	12	115104460	CTATGCC	12	113588843	R
	GGATTTC	37	1	215196452	CGAAAGC	1	213263075	R
	ACTAGAC	37	2	172452502	CGCCAGC	2	172160748	R
	TTATTTA	37	6	32608858	GTGCACT	6	32716836	F
	ATGGTGA	37	12	98850691	CGGCCAC	12	97374822	R
Red	AGAGAGC	37	19	6710658	CGCAGGA	19	6661658	F
	CCATGCT	37	1	246592123	CGCCGAT	1	244658746	F
	AGGCTTG	37	21	46675571	GTGTGTG	21	45499999	F
	CAGCCGC	37	2	795989	AATAAGC	2	785989	F
	CCAAAGT	37	4	183006582	CGTGTGT	4	183243576	R
Grn	GCCCTCT	37	1	110230401	GCCTCCA	1	110031924	R
	CGCCTTC	37	1	207992403	GCAGAGT	1	206059026	F
Red	GCGGAGC	37	10	124578209	CGCACAC	10	124568199	R
	TGAGGAC	37	11	115571126	AGCCTCT	11	115076336	R
	ATCTTCC	37	6	28697846	TTGGGAC	6	28805825	F
	ACAGAAC	37	5	140579046	CGGAGGA	5	140559230	F
	GAGTCAC	37	8	143781340	CGTGAGT	8	143778342	R
Red	CTGTGCC	37	8	216659	GCGGAGC	8	206659	R
	GATGGTC	37	5	112824920	TGATCCA	5	112852819	R
	GGCAGGC	37	11	10715360	CGACCAC	11	10671936	R
Red	CGGGTAC	37	20	45170494	GGCAGCC	20	44603901	R
	AGGTGCC	37	1	3721846	CGGCACA	1	3711706	R

	TTCCCTC	37	7	154719693	CACAGGC	7	154350626	F
	AGGAGA	37	17	80921117	TGAGCTA	17	78514406	R
	AAGGCAC	37	1	230240631	CCCTTGC	1	228307254	R
Grn	AGCACCC	37	5	179059426	GACGGTC	5	178992032	F
	GCAGATC	37	1	222162028	CGTCTTA	1	220228651	R
	TTTCCTA	37	2	109826107	CGACTGC	2	109192539	F
Red	CACCTTC	37	13	19918951	AGTCCAG	13	18816951	F
	TCTTATT	37	12	114887657	GTTTGGT	12	113372040	R
	CCCAGCG	37	6	32633157	CGCCCTG	6	32741135	R
	GTGAACA	37	2	113379847	AAGTCAC	2	113096318	R
Grn	CGCGCTC	37	X	117480246	CGGCGGT	X	117364274	R
	TTGCTTG	37	2	26578508	GCAAGGA	2	26432012	F
Grn	CGCACCG	37	2	91937265	TAATTTC	2	91300992	F
	ATGACTT	37	8	59698273	GTAAATT	8	59860827	F
	AGTGATC	37	6	42186179	CATTATT	6	42294157	F
	CTTCCGC	37	10	130279707	GTGCCGA	10	130169697	R
	ATAAATT	37	1	17500642	GTCTCAG	1	17373229	R
Red	GTGCCCC	37	17	4437925	GGGACAC	17	4384674	R
Red	GATGAAT	37	8	81787111	CGCTCAT	8	81949666	R
Red	TGCGCTC	37	6	30039013	CGACGGC	6	30146992	R
	AGAAAAC	37	7	2802942	CGGGCTG	7	2769468	R
	CCGCGAC	37	X	102863004	CGCCCAC	X	102749660	R
	TAAAAAT	37	17	80970434	CGGGCCC	17	78563723	F
Grn	GCCGGCC	37	6	44243304	GCTGCGC	6	44351282	R
	TCCAAGA	37	7	127078268	CGGTGTG	7	126865504	F
	CGGAGCA	37	1	210013268	CGTGGTG	1	208079891	F
Red	GCCCGCG	37	6	36930062	TGGGTGT	6	37038040	R
Grn	TTGGCCT	37	1	156211892	GCTGGGC	1	154478516	R
Red	GTTATGG	37	11	48374446	CGATACT	11	48331022	F
Grn	CAGGCTG	37	5	140189548	ATAAAGC	5	140169732	R
Red	GCAAAGT	37	12	116044032	CGCCAGC	12	114528415	R
	GACTCAT	37	13	38231017	CGGCTCT	13	37129017	F
	GATACAA	37	17	80735406	AGGGGCA	17	78328695	R
	TGGGTCT	37	3	134047274	AATCTCT	3	135529964	R
	CACCACC	37	2	23746926	CGGAAGA	2	23600431	R
	CCTCCTT	37	15	55513621	TTGCAAC	15	53300913	R
	GCCACGC	37	10	118387095	CGGGGCA	10	118377085	R
Red	GGGGGCC	37	11	67383802	CGGAGAC	11	67140378	R
Red	CAGGCGC	37	3	10749371	CGGCCCG	3	10724371	F
	CGGGCTC	37	8	125150452	CGCATCA	8	125219633	R
	AGGTCGT	37	6	28697819	CGGGGCT	6	28805798	F
	GGGAGTA	37	22	43168851	CGGCTCA	22	41498795	R
	ACCAAAC	37	6	28876508	CGTGGGA	6	28984487	R

	CCCAGCC	37	10	97419053	TTTTTCT	10	97409043	F
Red	CTCTGGG	37	14	101175645	ATTGTCC	14	100245398	F
Grn	CACTCAC	37	7	101961796	GCTGGAC	7	101748516	F
Grn	AGGCTTC	37	4	3235319	GGCCTCG	4	3205117	F
	TTTTGTG	37	18	11818701	CGGGACC	18	11808701	F
	ACAATTC	37	5	76034887	AGCAGCA	5	76070643	R
	TCCTCAA	37	19	49339397	CGGAGAC	19	54031209	F
	AAGTAGC	37	1	225955869	CGGAACA	1	224022492	F
	TTTTATT	37	5	102114455	CGAAGCT	5	102142354	F
Red	CGTGGCC	37	1	2229140	CGCAGAT	1	2219000	R
Red	CTCAGAA	37	6	167589370	GCGGGTT	6	167509360	R
	TTCTCCT	37	15	71890066	TGTCCCA	15	69677120	R
	CGTCAGA	37	6	101846767	TGCAGCA	6	101953488	R
	CACTAAA	37	2	133062155	AAAGTGC	2	132778625	R
Grn	GCTGTGA	37	14	101908865	CGACAAC	14	100978618	R
	ACTATTT	37	6	32557970	CATTAAG	6	32665948	F
	TCCTTCC	37	19	937444	CGGGCAA	19	888444	R
	CACCTTT	37	11	78052863	ATGATAC	11	77730511	R
	TATGAAG	37	15	33111288	CCTCAGT	15	30898580	F
	CTACTCT	37	18	74118243	CTCGTCC	18	72247231	R
	GGGCCAC	37	15	22982193	CCAATGC	15	20533634	R
Grn	CCCCGCC	37	4	163085276	CGGCCCC	4	163304726	R
Grn	AGCTGCA	37	6	37617956	GGGAAGC	6	37725934	F
	CCCGACT	37	4	7502706	GCTACAG	4	7553606	F
	AAAACTC	37	17	74260692	GTGAACA	17	71772287	F
Red	CACAGTG	37	8	108441547	CTCATAA	8	108510723	R
	ATACAAA	37	10	35893624	CGGATAT	10	35933630	F
Red	CCCCATC	37	13	112984728	CGGGGCC	13	112032729	F
	AAGTACT	37	7	158075968	CGGTTCA	7	157768729	R
	GCAATCC	37	6	112111043	CGTGTCC	6	112217736	R
Red	CACTACT	37	3	143690208	GGGGCGA	3	145172898	F
	GTAGAGC	37	12	12620789	CCATGTG	12	12512056	R
Red	AGCGCTC	37	12	124929963	TGCAGCA	12	123495916	R
Grn	GGAACCC	37	12	130822603	CGCCGCC	12	129388556	R
	AGAACGC	37	6	29895260	CGCCTGT	6	30003239	F
	GTGGATT	37	13	98126969	CGGAAAT	13	96924970	R
	GAGAGAC	37	1	18684587	CGCCATG	1	18557174	F
Red	CGAAAAC	37	3	12595473	CGGCGCA	3	12570473	F
	GTCGGAC	37	3	150480910	GGGGCTT	3	151963600	F
	TGCTGAT	37	12	2017134	TGTTCAC	12	1887395	R
	GACAAAC	37	5	155901557	CTTTCTG	5	155834135	F
	TCCTGCC	37	20	17680544	CAGTCTC	20	17628544	F
	GCCCTGG	37	2	135072000	CGGGCCT	2	134788470	R

	TGGATTC	37	5	1228214	CCAAAAA	5	1281214	R
	ACCACAA	37	1	180069210	CGCGCCC	1	178335833	F
	ATACTAA	37	1	220000086	CGATGGC	1	218066709	R
Red	GGCGGCC	37	6	28853021	CGCCCCC	6	28961000	R
	TTCTGTT(	37	17	32612675	TGACTTC	17	29636788	R
	AAATATC	37	11	18300953	CGGTGTA	11	18257529	R
	ACAGCTT	37	11	14431708	CGCCACA	11	14388284	F
	ATTGCAA	37	13	102686228	CGTCTCC.	13	101484229	R
	TTTTTGTT	37	10	72163076	CGCTGCT	10	71833082	F
	GTCCTGG	37	10	3088502	AGAGTGT	10	3078502	F
	CCAGAGC	37	18	14457820	GGAATTA	18	14447820	F
	TTTGGAC	37	15	41118060	CCATGGC	15	38905352	R
Red	CAATTTC	37	6	31785597	CGGTTTC	6	31893576	F
	GAACATC	37	5	59837426	CGGTTCT	5	59873183	R
	GGGTGTG	37	5	73188814	CGGTGGT	5	73224570	R
	AAACAGA	37	1	169072106	TCTGAAT	1	167338730	F
	GGCAGTA	37	11	10320161	GTCAGAC	11	10276737	R
	CTTGATT(	37	2	58482767	CGGTGCT	2	58336271	R
Red	AGGCAGT	37	11	128812992	TTTTCACA	11	128318202	R
	TCAGGAT	37	4	24780369	CGCAGCT	4	24389467	R
	AAATTGG	37	7	65227864	CGGTGAT	7	64865299	F
	GGTGCCA	37	2	110979750	CGGTGGC	2	110337039	F
	CTTCGCG	37	11	7110561	CGGCAAC	11	7067137	F
Red	ATCCGCG	37	13	44971694	CGCGTGG	13	43869694	R
	CCACCTT	37	2	792213	CGGCAGC	2	782213	F
Grn	AAGGAGA	37	8	8679450	CGGGTCG	8	8716860	F
Red	AGACTTT	37	19	40904691	CGAGAAC	19	45596531	R
	GGCCAGC	37	1	2245221	GACCAGC	1	2235081	F
	CCTCTGC	37	1	151557485	CGGCTGC	1	149824109	R
	GCAGGTA	37	7	147732939	CGGGTGA	7	147363872	F
	CTGCCGA	37	2	88427637	CGGCCAC	2	88208752	F
	GGGCTGT	37	20	61731415	AGAGATT	20	61201860	R
	CTAAGCC	37	13	79694063	TGCACGT	13	78592064	F
Grn	CCCTGTA	37	4	115416755	GCAGATC	4	115636204	F
	CCCTGCA	37	11	56615725	CGCAGCA	11	56372301	R
Red	CAGCTGA	37	17	75522344	CGGCGCC	17	73033939	F
Red	GGGTGGC	37	8	6481608	CAGCCCC	8	6469016	R
	TCTTCCT(	37	6	32297615	CGGGAAA	6	32405593	R
	CTAGGGC	37	5	415729	CGCCCCA	5	468729	R
	CGCGCGT	37	15	31355362	AAGAGCC	15	29142654	F
	GAAACAA	37	5	140515961	CGTGGAA	5	140496145	F
Grn	CCTGCGC	37	5	1594678	CGCGGCA	5	1647678	R
	CTGAGTA	37	15	67361773	CGGCAAA	15	65148827	R

	ATTATTC(	37	8	8932588	CTGAATC	8	8969998	R
	CAGCACA	37	10	85321586	CGGGGCT	10	85311566	R
	GAAGGTA	37	6	32606845	AACTTTC.	6	32714823	R
Red	GAACCCA	37	11	67764320	GCGGGGC	11	67520896	F
	CATCTTG(	37	17	38249266	CGAGACC	17	35502792	R
	GCAGGGA	37	7	1420943	CGACTCT	7	1387469	R
Red	AGGGCCC	37	20	259898	CGGGAGC	20	207898	R
Grn	ACCTGGG	37	1	247803033	GCCAGTG	1	245869656	F
	AAAGTCC	37	10	49654342	CGCCTGG	10	49324348	F
	TTGGTGG	37	8	61785026	GAGAGGC	8	61947580	R
	CTGTTGG	37	14	89604062	CGGCCCT	14	88673815	R
Red	CCCTCGC.	37	17	75238703	TTGCCGT(	17	72750298	R
Grn	AGTGGGA	37	1	247802802	GGGCCTG	1	245869425	R
Red	TTGAGAA	37	1	154947060	CGGAGGC	1	153213684	F
Red	GGAAGAA	37	5	140554819	CGCGTCT	5	140535003	F
	TCAGTGC	37	7	157495282	CGGGGCC	7	157188043	F
	AAGTAGT	37	13	47326127	CGACTCA	13	46224128	R
Red	TGTCTCA.	37	1	35586358	CGCGGCC	1	35358945	F
	AGTATTC	37	6	29794657	CGCACTG	6	29902636	R
	CCCCAGG	37	22	20283373	CGGCACC	22	18663373	F
	CTTCATTA	37	Y	7431190	CGGTGTG	Y	7491190	R
	GAGCAGC	37	10	93100919	CGAGTCA	10	93090899	R
Red	TTCTTTCC	37	1	150335507	CGGCACC	1	148602131	R
	ATTCCAA	37	4	155254016	GCAGAGT	4	155473466	R
	TAATGCC	37	6	156954565	ATGCTTT(	6	156996257	R
	GACTCAT	37	12	1391371	AGGATTC	12	1261632	F
	ATGTTCT'	37	2	18158708	CGGTGGC	2	18022189	F
	TGGATTT(	37	4	152147803	CGTCACA	4	152367253	F
	AATTTAT'	37	12	47610734	CTTTGTG(	12	45897001	F
	AAGCGTC	37	7	76109396	CTGTGTT(	7	75947332	F
Grn	GCGGCAC	37	3	42727489	CGCCTGT	3	42702493	F
	GGGTCAC	37	12	25454990	CGGTGCC	12	25346257	R
Red	TGTGGCC	37	5	1650942	CGCTAAT	5	1703942	R
	CCTGACC	37	6	32522683	CGGCTCT	6	32630661	R
	TTTGGCG	37	X	47433716	CGCCAGC	X	47318660	R
	CGGATTT	37	22	18531591	CGCTATG	22	16911591	R
Red	CTTCACT(	37	8	141294201	GGCAAGC	8	141363383	R
	AGCTCAG	37	7	134212463	TGAGACC	7	133863003	R
	CTGTGAG	37	11	65631880	GGATGAC	11	65388456	F
	CATAATA	37	8	25107121	TCTTTTGT	8	25163038	R
Red	TCTGCTG(	37	22	49847690	GACTGGC	22	48233694	F
	AACAGAA	37	10	25162298	ACATCAC	10	25202304	F
	CTAGGTG	37	11	133771378	CGGGCAA	11	133276588	R

	GTGGAAC	37	5	113699604	CGCCTGC	5	113727503	R
	CTGAGGC	37	1	17018684	CGGGAAC	1	16891271	R
	CTACCAG	37	6	110964408	CGTCAGG	6	111071101	R
	ATCTGTG	37	11	100760935	CGAGAGC	11	100266145	R
	GGTGAAC	37	5	140517411	GGGAAA	5	140497595	R
	AAGAAA	37	1	235796187	TTAGTTC	1	233862810	F
	AGACCAT	37	6	32640493	CGGAACC	6	32748471	F
Grn	TTCTCTC	37	1	247802774	GAGGAGC	1	245869397	F
Red	CGGGGGC	37	5	140222253	TTCGCTG	5	140202437	R
	AGACTCC	37	X	70474172	CGCCGTC	X	70390897	R
	AGATTGA	37	10	102239027	CGCCAAC	10	102229017	F
	CAGTGCC	37	19	12940950	GGGGCTC	19	12801950	F
Red	TTTTCAC	37	4	40267141	TGACAAC	4	39943536	F
	CCTAAAG	37	12	113842581	CGCCAGC	12	112326964	R
	CGCAGAT	37	12	2881762	CAGGACT	12	2752023	F
Red	TGGCCTC	37	2	242713790	CGGCCAC	2	242362463	R
	CCTGGTG	37	10	3163518	CGCACTG	10	3153518	F
	CTCAGGA	37	6	158234655	CAGCAGT	6	158154643	R
	TCCATGA	37	10	112580109	CGGACAC	10	112570099	F
Grn	CCAGTGC	37	22	18531243	AGATTCC	22	16911243	R
	CCAGCAC	37	13	101315319	CAGTGGC	13	100113320	F
	AGCGCTC	37	2	128453484	CGCCCCC	2	128169954	F
	TTTTGTAC	37	7	127409937	CGCTGAG	7	127197173	R
	GAAGAAC	37	6	33033484	CGGCAAT	6	33141462	F
	ACTATAT	37	10	113944114	CGCTAAG	10	113934104	F
	ACCTCAG	37	15	56531754	CGCGCCT	15	54319046	F
	TTGAATT	37	7	98976900	TCTTACC	7	98814836	F
Grn	GTGGCCG	37	2	233217079	CGCCGCT	2	232925323	R
	ACAGTTA	37	8	38578802	CGCGGAC	8	38697959	F
	CCTTCAA	37	13	110434467	GGGACAA	13	109232468	R
	CGGTGTT	37	19	468773	CCCCACA	19	419773	R
	GGGGCCC	37	11	5960081	ACCGGTT	11	5916657	R
Red	AAGGCTC	37	8	1439535	GGGAGCA	8	1426942	F
Red	ACGCAGC	37	7	158769805	AGCTCTG	7	158462566	R
Red	AGAAAAT	37	17	73286282	CGGAGAC	17	70797877	R
	ACCACTG	37	2	30887962	CAGGCAC	2	30741466	R
	TGGGCTT	37	2	196399403	GGCCTGC	2	196107648	R
Red	GAGAGGC	37	5	135416398	GAACTGA	5	135444297	F
	CCCAGAT	37	12	108078835	TGTATCT	12	106602965	F
	GAAACAC	37	10	64875536	CGGCAGT	10	64545542	R
	TCCAGGC	37	15	100517183	CGGGCCT	15	98334706	F
Red	GTTCGGG	37	10	77155376	GTGGGTG	10	76825382	F
	GCAAGTC	37	1	46649132	CGGCCCA	1	46421719	R

	CCCGTGT	37	2	9062790	CGGGTGT	2	8980241	R
Red	GTAGCCC	37	1	98510328	CGCGCTA	1	98282916	F
	CAGCTGA	37	3	30383960	AACTTTG	3	30358964	R
Red	CCCGAGA	37	11	1529608	ATGGAGC	11	1486184	R
	TCCTCCT	37	5	77178418	TCTCTTCT	5	77214174	F
	AACTCAA	37	3	23496403	CTTTTCAT	3	23471407	F
	GGGTGGC	37	17	80084596	CGCAAGC	17	77677885	F
	GGCTGGC	37	X	131157336	CGAGCCC	X	130985017	F
	GGGTGGC	37	5	66462293	CGGAGGC	5	66498049	F
Red	AAAGGTA	37	10	133558485	CGCCCAT	10	133408475	F
	CCTATGC	37	1	31197913	TGCTAAA	1	30970500	F
	TCTGGCC	37	11	20053759	CGAGGGA	11	20010335	R
Grn	GAGAGTT	37	6	31651059	TCAGAAC	6	31759038	F
	CTTCTTTT	37	5	76785615	ACTAAAT	5	76821371	R
Red	GGACACC	37	18	59221601	GGGAGAC	18	57372581	F
	AGAGGTC	37	11	65683543	GCTTTTCA	11	65440119	F
	TAAGGTG	37	13	114928103	CGCTTGT	13	113946205	R
Red	TCTTCTG	37	22	22987072	CGGCGCC	22	21317072	F
Grn	TCCTTGC	37	8	83353753	CGGCTCT	8	83516308	R
Red	GCATCAC	37	17	60744005	TCCACGA	17	58097737	R
	TTGGCAT	37	3	45677346	CGAAGGT	3	45652350	R
	CCCTTCT	37	5	85110900	CCTGGGA	5	85146656	R
	AGAGAAA	37	5	2387924	GCAGGTC	5	2440924	R
	TTGCTAT	37	4	176486240	CGGGCAT	4	176723234	F
	GGGATAT	37	17	46970175	CGGCTCA	17	44325174	F
Red	ACCGTTG	37	22	42524984	CGGGGAA	22	40854928	R
	CCACTCA	37	19	42274924	TTCACCT	19	46966764	R
	CCAAATG	37	10	105032988	CGTGTCT	10	105022978	F
	TCATTTTA	37	1	212335061	CAGATAA	1	210401684	R
Grn	CGGCCTG	37	8	144810034	GGAAGCC	8	144882022	R
	ACAGATC	37	3	17680687	TGTTTTGA	3	17655691	R
Red	CGGTGCT	37	1	15541349	CTAACTC	1	15413936	R
Grn	TGCAGGC	37	1	31280143	CGCGGCC	1	31052730	R
	CACAGCC	37	3	71111489	AATCCCT	3	71194179	R
	AGCCTAC	37	3	41723017	AAGGAGA	3	41698021	F
	GAGCTGC	37	2	242048127	AAAGGGC	2	241696800	F
	TTTCTAA	37	2	138045743	CGCCCTT	2	137762213	F
	AAGAGCA	37	3	42307519	CGCCCAG	3	42282523	F
	AAGATGC	37	6	101878898	TGTAAGT	6	101985619	R
Red	CCCTCCC	37	13	42002627	GATCGTC	13	40900627	R
	GCCATGC	37	11	74030400	CGCATGA	11	73708048	R
	CCCACAG	37	13	19960816	CGGCATC	13	18858816	R
	TGTGTGA	37	8	49502710	CGCCGCT	8	49665263	F

	GCAGGAA	37	5	112540429	CGGTTAT	5	112568328	F
Red	AATGGGC	37	4	6474782	CGCGCAA	4	6525683	F
	TTTCATGT	37	13	44000493	CGTGCTG	13	42898493	F
	GAAAGGT	37	8	90986930	TTCCTTCC	8	91056106	F
	CTAAGTC	37	8	112691450	CGTCGAT	8	112760626	R
	TTCTTCAC	37	13	41070900	CGGCCAT	13	39968900	F
	AGGCAAC	37	19	2525384	GGGCTTA	19	2476384	F
Red	GGGCCTG	37	X	133594204	CGTGGCG	X	133421870	R
	CAGAAAC	37	3	190104266	TTCCACA	3	191586960	F
	GTCTTCTC	37	4	189026860	CGAGTCA	4	189263854	F
Red	CTTGACC	37	2	48844892	CGCCCCC	2	48698396	F
	GGGCGCC	37	14	24780557	CGTGCTT	14	23850397	F
	ATACTGA	37	15	99709980	TGGATTC	15	97527503	F
Red	CTGCACT	37	19	345306	CGCAAGC	19	296306	R
	TATGAGG	37	15	83332065	CGCATTT	15	81129120	F
	GTCCTTG	37	2	10037561	CGCAGCA	2	9955012	F
	TCGGTTCT	37	1	17394374	CGATTGA	1	17266961	F
	GTATATG	37	X	37765018	TGTGTTG	X	37649962	R
	AAGTGTC	37	1	28843736	CGAGAAA	1	28716323	F
	TCTCCTTC	37	1	161349116	CGCTGAC	1	159615740	R
	GTGCAGC	37	3	50306456	CCTGAGG	3	50281460	R
	AGATCAT	37	6	32634245	CGGCTCT	6	32742223	F
	TTCTGCTT	37	2	114151353	CGGCGCC	2	113867823	R
	GAATTTG	37	12	129337339	CGTGCGG	12	127903292	F
	GGGGCAC	37	19	17434267	AATACTG	19	17295267	R
Grn	CTCTAAA	37	3	47397852	GGCCCAC	3	47372856	R
	GCATGGC	37	11	126976228	GTCCTCA	11	126481438	R
Red	CCTGGGA	37	1	23495577	CGCGGGC	1	23368164	R
Red	GCAAGCA	37	3	197121419	CGGGAGC	3	198605816	F
Grn	CAGCTCC	37	5	177823856	TTGAAGT	5	177756462	F
Red	CAACGCC	37	8	143561205	TGAGGGC	8	143558207	R
	GAGCTGC	37	2	241644166	GGAATGT	2	241292839	F
	TGAGAGC	37	16	2848409	AACAGCC	16	2788410	R
	TCCTTCCC	37	10	31939075	GGAGGCT	10	31979081	R
	CCTGGTG	37	11	8707082	CGGTGCC	11	8663658	F
	AAGGAGT	37	5	78599729	CGGAATA	5	78635485	F
	ATTTTCCC	37	22	26952678	CGCTGCC	22	25282678	F
	TCTTACCA	37	6	46652700	CACAATG	6	46760659	F
	GTTTTCCA	37	12	132965879	CGAGGCT	12	131475952	F
	AAGGCAC	37	5	33050111	CGGGGCC	5	33085868	F
	TGGAAGA	37	19	3030324	CCCCGGG	19	2981324	F
	AGTGATC	37	3	46521313	ATCTCAC	3	46496317	F
	GAATAAA	37	3	177309023	CGGTCAT	3	178791717	R

	ACCATAT	37	15	77748245	CGGAAAA	15	75535300	R
	TTGGCCC	37	2	240019300	TCCTCTG	2	239684237	R
	GACAAGT	37	6	30652202	TCTAAGA	6	30760181	R
	ACCAGGC	37	6	32522905	CGTGATA	6	32630883	F
	CAGGAGC	37	12	71148346	AAAGAAC	12	69434613	F
	ACCTAGG	37	4	89667785	CGGAGGC	4	89886808	R
	CACAGCC	37	12	103325781	AACCCCT	12	101849911	F
	TCTAAGA	37	12	107347673	CGAAGAA	12	105871803	F
	CTCCCAG	37	3	11178758	GCCTGAC	3	11153758	R
	TTTATAG	37	17	41522193	CGGCATG	17	38877719	F
Red	CACCTCC	37	17	4439481	CGGGGCT	17	4386230	R
	AAAGGTA	37	17	79258813	CGGGGCA	17	76873408	F
	TGGCCAC	37	3	139724110	CGGATGT	3	141206800	R
	TGTAAAA	37	20	17582752	CGAGGTC	20	17530752	F
Red	GCAACGC	37	X	136512132	GTATGCG	X	136339798	R
Red	GACGGTT	37	19	291986	TGAAATG	19	242986	F
	GTCTGCT	37	3	57969522	CGGTGCT	3	57944562	R
Red	GTGTGTG	37	Y	9307448	CGGGTGT	Y	9917448	F
	TGCTAAA	37	3	34884326	CGGTAAC	3	34859330	F
	ATCCCCG	37	13	111281144	AGGCTCT	13	110079145	R
	AATGGGT	37	2	286830	TACAAAC	2	276830	F
	TAGAAAA	37	6	170732120	CGCCTCA	6	170574045	R
Red	AGCCATC	37	17	48263204	CGAGATC	17	45618203	R
Red	TCGTCTC	37	3	61235869	TGGCCGC	3	61210909	F
	TCGCACC	37	6	160182554	CGCATAG	6	160102544	F
	GGGAGCC	37	17	3848506	CGACCCC	17	3795255	F
Red	CCCAGCT	37	1	202129865	CTCACCC	1	200396488	F
	GACTCAG	37	1	115722330	AGCCACC	1	115523853	R
	TGGTGTG	37	10	3823907	CGGTGCC	10	3813907	R
Red	CAACGCC	37	6	25732923	TAAGCCA	6	25840902	F
	ACTGTGC	37	2	200904751	CGACTAA	2	200612996	F
	AACAGCT	37	1	9335794	CGGGTAA	1	9258381	F
	GCACCAC	37	19	38039469	CGGGTCA	19	42731309	F
	GGTCTTG	37	1	207039833	CCTGTCC	1	205106456	F
	TCAAAAA	37	1	13184954	CATTCAT	1	13107541	R
	AGTCTGA	37	3	121307887	CGGCCAC	3	122790577	F
	ATTCCCA	37	3	30140788	CGAGCTC	3	30115792	F
	ACTCTGC	37	1	247420421	CGCCAAT	1	245487044	F
	TGCTTTC	37	5	14348251	CGACATA	5	14401251	R
	TGCGGGC	37	4	13537089	TTACTTA	4	13146187	F
	GGTCTCC	37	7	4180723	CTCACTT	7	4147249	F
	TTGTGCA	37	18	32289350	CGGGGGC	18	30543348	F
	ACCAAGC	37	4	10034580	CAGCTCG	4	9643678	F

	CATAACT	37	4	2627014	ATTCTGT	4	2596812	R
	TGTCAAG	37	10	29834263	CGGCGGC	10	29874269	F
	CACGCAC	37	4	7788342	CGGATGA	4	7839242	R
	GCTGGGA	37	12	127631198	CGCCTCC	12	126197151	F
	GTCATAT	37	17	15255513	CGACTCG	17	15196238	F
	CAGTCTG	37	13	49672483	CGGCAGT	13	48570484	R
Red	CCCGGCC	37	X	153096039	AAAAAGC	X	152749233	R
Red	CAACACA	37	X	25021346	CGGAAGC	X	24931267	F
Red	GCTAGCG	37	X	73756753	CGCCCTC	X	73673478	R
	TGCTGTG	37	7	143028708	GCAGTGA	7	142738830	F
	CCCAGAC	37	17	81014413	TCAGGAC	17	78607702	R
	AGAAACA	37	15	65272560	CGGCTCA	15	63059613	R
	ACCCAGC	37	1	47900630	GCCCCTC	1	47673217	R
Red	CACAGAA	37	11	50257625	CGCACAT	11	50214201	F
	GAAGGAA	37	X	130423014	GAGATAT	X	130250695	R
	GTTCAAA	37	10	3666169	ATTCGAA	10	3656169	F
Red	ACCCGCG	37	13	59398086	TGGGGCT	13	58296087	F
	TCCTGTTC	37	10	33342522	CGGAGGC	10	33382528	R
	GAGGCCC	37	7	157405965	CGCTGTC	7	157098726	F
	TCTTCCG	37	4	7844982	GGAAGGC	4	7895882	R
	ACCTGCT	37	Y	24452543	TGCGGTA	Y	22861931	F
Red	GTTGGCC	37	13	100150906	CGCCCCC	13	98948907	F
	TGTCCAC	37	17	39597601	CGGGGTT	17	36851127	R
Red	ATTCCAG	37	7	102107737	CGGACCC	7	101894742	R
Grn	CACGGCC	37	8	58172855	CGGTGGC	8	58335409	F
	GAAATAC	37	21	46001680	CAAATGA	21	44826108	F
Grn	CCCCGGG	37	2	177016619	GAGGCGC	2	176724865	F
	GATCGAC	37	2	232349118	CGGAAGC	2	232057362	R
	GCCTCCA	37	15	71092216	GTTTTGA	15	68879270	R
	AATCAGA	37	6	31323677	CGGTTCC	6	31431656	F
	TTTGAGT	37	3	168822459	CGTGTTT	3	170305153	R
	GTCATGT	37	10	47696803	ACTTTCC	10	47166809	R
	TCTTTAC	37	3	50855436	CGGCAGA	3	50830440	R
	AATCTCT	37	8	17207503	TGATACT	8	17251874	R
	ATTGGAG	37	7	55516751	CGCGGCC	7	55484245	R
	GATCAGC	37	7	140955751	CGCCTAG	7	140602220	R
Red	GCTTTTTC	37	1	16163967	AGAAAGC	1	16036554	R
Red	GGGCGCC	37	20	57465439	CGTCGCT	20	56898834	R
Grn	TGCTTGA	37	7	65971099	GTGCTCT	7	65608534	R
	CTCAGGT	37	20	60713905	CGCCCAC	20	60147300	F
Grn	ATTTATT	37	13	20531342	TTTAGTG	13	19429342	F
	AGCCAAC	37	2	42328001	GCCCAGC	2	42181505	F
	GGTTTTA	37	2	228331319	CGGTTTT	2	228039563	R

	AAGAAAT	37		2	133104748	CGCCTTT		2	132821218	F
	GAGGTCA	37	X		43741890	CGGTATT	X		43626834	F
Red	GGTGCGC	37		19	2251067	CTCGAGT		19	2202067	F
Red	GCCCCTT	37		1	178456093	TGGAGGC		1	176722716	R
Red	AGCTCTC	37		1	182921660	CGAGTTT		1	181188283	R
Red	AAGCCCC	37		3	127347876	CTCGGCG		3	128830566	R
	GAAACAA	37		6	36821364	CGTGGGC		6	36929342	R
Red	CCCGGCG	37		1	92952467	GCCTTTA		1	92725055	F
	ATCCTTG	37		11	117110208	TCTCTGA		11	116615418	R
	GGACCCC	37		1	209982407	CGTTAAC		1	208049030	R
	CGGGATA	37	X		153059444	GTGACTG	X		152712638	R
	TTGCTTC	37		11	120417671	TAATCCC		11	119922881	R
	AATGGTA	37		8	131265658	AAGAGAC		8	131334840	R
Red	AGGGCAC	37		2	89064631	CGCCGCG		2	88845746	R
	ATTACAT	37		7	123252579	TCGCACT		7	123039815	F
	CACACTA	37		11	124613956	CGGAGAC		11	124119166	R
	TCGGCCC	37		17	49021653	GCAGGAT		17	46376652	R
	GGTGGTT	37		7	80080213	CGACTGT		7	79918149	F
	AAATGTC	37		12	120930045	CGGTATA		12	119414428	R
	TACCTTT	37		8	10049871	CGCTCAC		8	10087281	R
Red	TTAGGAG	37		17	10204251	ATTTCTC		17	10144976	R
Red	TTTGGGG	37		4	132652001	TGAAGGC		4	132871451	R
	CGATGGC	37		20	43371550	GATAAAC		20	42804964	R
	TCGCGCC	37		7	100202882	GGACACA		7	100040818	F
	AGGAGTC	37		12	119591664	CGGGACT		12	118076047	R
	GAAGATC	37		4	154515617	CGGAATC		4	154735067	R
Red	CTCGGCC	37	X		37430775	CTTGGCT	X		37315694	F
Red	GAGGACC	37		3	138739916	TAGAGGC		3	140222606	R
	GCCGGAC	37		5	173191648	CTCTTGC		5	173124254	R
	CAGCTGT	37		11	46765391	CGCTGAC		11	46721967	F
	CGAGTCA	37		12	132970851	GGTGACT		12	131480924	F
	AACTGGA	37		4	119095240	CAGAGAA		4	119314688	F
	CCGTCAG	37		12	14926744	CGTTCAC		12	14818011	R
Red	TGAGGAC	37		5	179059668	CGCAGGC		5	178992274	F
	CACCACA	37		6	31747041	ACCTTTC		6	31855020	F
	AGGGCTA	37		8	126525558	GAGGAGA		8	126594740	R
Red	AAGACCT	37		1	29446085	CGGGGCC		1	29318672	R
	TCTTCCT	37		11	12309622	CGCACCA		11	12266198	F
	TCCTTCCA	37		1	27881600	CGCCGTG		1	27754187	F
	GTGGCTT	37		6	6614447	AGGCAGT		6	6559446	F
	ACAAAAT	37		13	34371837	CGGTGGC		13	33269837	F
Grn	GGGCGGC	37		2	130986931	CGCCCCA		2	130703401	R
Red	AGCAGCC	37		7	158075624	CGGTGGT		7	157768385	F

	AAGCCTC	37	11	3534637	GGTGCTC	11	3491213	R
	AAATTGT	37	17	39072167	CGAGAAC	17	36325693	F
	ACTCAGG	37	4	114389632	TTCATTTC	4	114609081	F
	AAATAAA	37	8	125274483	CGGAAAA	8	125343664	R
	GAGGGGT	37	2	153355619	CGGGAGC	2	153063865	F
	CAAGGCC	37	3	104078476	CGAAGTC	3	105561166	F
	CCATATG	37	4	103172826	CGCTTCTA	4	103391849	F
	TATATAT	37	10	76983413	AGCCACC	10	76653419	F
	CTCGTGTC	37	5	1222775	TATGTGTC	5	1275775	F
	GAATTGG	37	10	766041	CGAGTAT	10	756041	F
	CACACCT	37	19	56154382	CGCCTCC	19	60846194	F
	AGGTTTA	37	18	57636670	CGGAATT	18	55787650	R
	CACGGAC	37	5	1075733	CGCCTGA	5	1128733	F
Red	GGCAGAC	37	1	2121039	ATCACAA	1	2110899	R
Red	GAAGACA	37	14	73392919	CGGCCCC	14	72462672	F
	AAGCCAA	37	7	11456440	TTACATA	7	11422965	R
	CTACAGA	37	6	27521330	CAGCATG	6	27629309	R
	AACCTTT	37	5	74303397	GTTTACA	5	74339153	F
	TTGGGAG	37	1	247511469	GCTGCAG	1	245578092	F
	CACTTGC	37	4	5960351	TGCTGAT	4	6011252	R
Red	CGCTGCC	37	19	58554479	GCTAACA	19	63246291	R
Red	TATTGCA	37	5	79378955	CGAAGCC	5	79414711	R
	CTCCCATC	37	17	74086286	CCTGGCC	17	71597881	F
	GGCCAGA	37	17	77923675	CGGCAAA	17	75538270	F
	CCCAGCC	37	6	30019655	ATACTAT	6	30127634	R
	GCCACTG	37	5	152502171	GCTTTCAC	5	152482364	R
	AGTGGCT	37	3	151460366	CGGATTT	3	152943056	F
	TCCTAGTC	37	6	5442953	CGACTTC	6	5387952	R
	CTGTGGC	37	X	13588301	CGCAGCC	X	13498222	R
Red	TGCTCCTA	37	17	71024449	GTTAGAC	17	68536044	R
Red	ACGCTGG	37	14	24780540	TAGGGGC	14	23850380	F
	GATGCTC	37	6	101846967	CGCCAAC	6	101953688	R
	ACTGTGG	37	2	86248333	CTGAGGA	2	86101844	R
Red	GGGCGCC	37	X	53461386	CGGCCCG	X	53478111	R
Red	TGTGGAG	37	5	135416394	CGCCCGC	5	135444293	F
	CCTGCCC	37	6	112620506	CGGGGTT	6	112727199	F
	GTTTTATT	37	17	19807272	CGGTAAC	17	19747864	R
Grn	CTCAATC	37	X	18693413	CGCCCCC	X	18603334	F
	CAAGATA	37	14	23005866	CGGCTGT	14	22075706	R
	TGCTCCCC	37	12	133013600	TGTGTTCT	12	131523673	F
	ATAAGCT	37	1	14824776	CGACCTG	1	14697363	R
	CGTTAGA	37	1	149156606	GCTTGCTC	1	147423230	R
	GGACAAC	37	11	10921186	CGGTGCC	11	10877762	R

	ACAAGTA	37	12	2445561	AAAATAC	12	2315822	F
Red	CCCCACG	37	X	30671338	AGCAAAC	X	30581259	F
Red	GGGCGGC	37	6	157041268	TCCTGGG	6	157082960	F
Red	TGGAGAA	37	6	111888446	CGCAGGC	6	111995139	R
	TCTTTTTT	37	11	28642652	CGTGCCA	11	28599228	R
	GGCTCGG	37	7	155299687	AAGCTGT	7	154992448	F
	TTGTTTTT	37	13	49323899	CGTTGTG	13	48221900	R
	CTAGAGC	37	14	21191859	CATACAG	14	20261699	F
Grn	AGAGCAA	37	4	7844952	CCGGCAC	4	7895852	F
	AGGTTGG	37	3	4783306	CGGAAGC	3	4758306	R
	GGGACAC	37	7	107700124	CGCTTCT	7	107487360	F
	GTCCTTG	37	4	33062845	AAGCTTG	4	32739240	R
	CGGAGAC	37	13	19919158	GCTCTGA	13	18817158	F
	GTGGATG	37	3	62665094	CGGGACT	3	62640134	R
	CTCCTCA	37	10	80076484	CGAGCTC	10	79746490	R
	CAAACAC	37	14	52482697	AAATGGT	14	51552447	R
	AGCCCCG	37	1	3561008	CGGGTCT	1	3550868	F
	GCCGGGT	37	22	46481785	CGGGGTC	22	44860449	R
	GTGTTGC	37	2	233734820	CGGGGCT	2	233443064	F
	ATTTACT	37	7	5186528	ATCCGGC	7	5153054	R
	CAAATAA	37	5	177882205	CGGGTTG	5	177814811	R
	GTCTCCC	37	1	221057573	CGGCACT	1	219124196	F
	AAAAGGC	37	8	61626625	TTCATTC	8	61789179	R
	ACAGCGC	37	4	2432313	GGTGAAA	4	2402111	R
	TCAGCGC	37	7	140263173	TACACAG	7	139909642	R
	TTGGGGC	37	7	47375903	CGCCCTG	7	47342428	F
Grn	GATTGCA	37	10	99912042	GGCCAAC	10	99902032	F
	ACAAGTC	37	6	47666248	AAGGCTT	6	47774207	F
Red	CCCTCCT	37	15	74494900	ACACACC	15	72281953	F
	TGGGTGT	37	19	50862121	CGGGTTC	19	55553933	F
	CCAGACC	37	7	95026121	GACAGGT	7	94864057	F
	AACTAAC	37	2	37962248	CGCTAGG	2	37815752	R
	TCATGCC	37	4	155662795	CGGCCAC	4	155882245	F
	TTCAGAT	37	10	91470135	CGCCACC	10	91460115	F
	CCCAAGA	37	2	175307665	CGGCCTT	2	175015911	F
	TGTCCTG	37	11	35611044	CCAGCCA	11	35567620	F
Red	GGGCTGC	37	16	1098847	CGTGCAG	16	1038848	R
	GAGGGAA	37	15	31846670	CGGGCCT	15	29633962	F
	CTTGCTG	37	2	240143798	CGCTGGC	2	239808735	R
Red	TCTCTGG	37	5	1594282	CGGGGGT	5	1647282	F
	CGGCGGC	37	5	140480941	CGACAAT	5	140461125	F
	CTGACTC	37	16	10723694	CAAAGGC	16	10631195	R
	GATTTGC	37	5	148832884	CAATGCC	5	148813077	R

	ACTCTCC	37	Y	24331358	CTTGCAA	Y	22740746	R
Red	TTTCCCC	37	19	2037662	GCCAGCT	19	1988662	F
	AACCAGC	37	17	6339466	GTGTAGG	17	6280190	F
	AATTTAA	37	6	90192032	CGGCCAT	6	90248751	R
	CTAGGGA	37	6	31382102	CGCAGCT	6	31490081	R
	AGGACCA	37	16	88990409	CGCTGTG	16	87517910	R
Grn	ACAAGCC	37	16	46603021	CGCACCT	16	45160522	F
Grn	GCTTCTG	37	13	113688268	TGGGGAC	13	112736269	R
	CCACTAT	37	4	141419962	CGGGACC	4	141639412	F
	CTGGGAC	37	6	30000200	CGGCAAC	6	30108179	F
Grn	CCCCCCC	37	10	42672589	GCCGCGC	10	41992595	F
	GCAGCAC	37	6	109871787	CGGGATC	6	109978480	F
	AGGGATC	37	4	40336255	ACTCATC	4	40031012	R
	AAAGAAC	37	6	26746687	CGCCCGA	6	26854666	F
Red	GAGTGGC	37	22	48730391	CGGCCCT	22	47109055	F
	TTTCAAC	37	5	142527658	CGCCTTT	5	142507851	F
	CAGTCCC	37	19	475587	CGGGGAC	19	426587	F
Grn	GGCGCGC	37	6	74072376	GACACCA	6	74129097	F
Red	CAGGGAA	37	5	180028767	CGGGGAC	5	179961373	F
	GGTGTCA	37	15	87197932	CGGTGAC	15	84998936	R
	ACCCCTC	37	6	116382179	CGGCTGC	6	116488872	R
	GGAAGTT	37	7	42278096	ATAAAGT	7	42244621	F
	CTCTGGT	37	6	31096127	TTCTTGG	6	31204106	R
	ACCCAAC	37	11	5384517	CGGTGAC	11	5341093	F
	TTCTCCT	37	11	131758433	CGGGATC	11	131263643	R
	GACTCTA	37	1	248756151	AGAGGAC	1	246822774	R
Grn	GACAGGA	37	6	30419576	ATGGAAC	6	30527555	F
	ACTACTG	37	10	126135176	CAAAGTA	10	126125166	R
Red	AGTAGGC	37	X	119443941	CCCGCAG	X	119327969	F
	CTAAAGT	37	16	23313240	CGCCCCC	16	23220741	R
	TCTTCCT	37	6	32548321	CGAGAGC	6	32656299	R
	CCCAAGT	37	16	2749429	CTGCCGC	16	2689430	R
Red	CGGTCAG	37	X	110653460	ACAGCGT	X	110540116	R
	GATGCCT	37	5	1298965	CCCTGCT	5	1351965	F
Grn	CTAGCAC	37	7	64403082	CCCGCCT	7	64040517	R
	GTCCGGT	37	1	9602701	CGCCAGC	1	9525288	R
Grn	CCGCTGC	37	8	1497078	AGTGCGA	8	1484485	F
	CATGATG	37	5	140213825	CTTTACC	5	140194009	R
Grn	AGGTTGA	37	12	115102726	ACTAAGC	12	113587109	R
	ACCTTCA	37	4	120726319	ATAAGAC	4	120945767	F
	CCTGGAG	37	19	18170383	CTGGGTT	19	18031383	R
Red	CTTGGCG	37	19	36485282	AGCCCTG	19	41177122	F
Grn	TGTGAGA	37	19	57276917	ACAAACA	19	61968729	F

	TGATGCC	37	10	3138505	GGGTGCA	10	3128505	R
	CCCGCAG	37	6	33048502	CGCTGTG	6	33156480	R
	TCACTCT	37	21	32927013	CGCAGGC	21	31848884	R
	AGGCCTG	37	16	88909028	ACCTGCC	16	87436529	R
Red	CAGTTTC	37	7	158533204	CGTGGCT	7	158225965	R
	CCAACAC	37	11	3225550	GTGCCTG	11	3182126	F
Grn	GGCGATC	37	19	2251588	CGAGCGC	19	2202588	R
Red	CACATGC	37	11	2919763	GTGCGGC	11	2876339	F
	TGTTTGT	37	11	65352232	AGCGTTT	11	65108808	R
	TTTGGCA	37	5	177209285	CCTCTGA	5	177141891	R
	ATATTTG	37	15	86133334	CGGGGTA	15	83934338	R
	CGGCAGA	37	11	1248959	GGCTGAC	11	1205535	R
Red	TTGTAAA	37	10	88023135	CTTGGAG	10	88013115	R
	TCATTCC	37	6	136915556	AAGGTCA	6	136957249	R
Red	CCTTGGG	37	7	140135365	CGCGCTG	7	139781834	F
	TCCCATC	37	19	33623091	GAGGGTC	19	38314931	F
	AAAACTC	37	5	5887642	CGGGTGC	5	5940642	R
	ATTCCCT	37	3	195384694	CGGGGCT	3	196869875	F
	ATGAGTC	37	18	13375474	CGCACTG	18	13365474	R
	CTTGGCC	37	13	111745598	GGTCGCT	13	110543599	R
Grn	CCAGGCT	37	6	43248039	CTATTTG	6	43356017	R
Grn	CCTCTTC	37	6	30419491	CGAGGCC	6	30527470	R
Red	TGGGTCC	37	17	40835920	GTTGGCA	17	38089446	F
Red	GGTGCGC	37	19	853334	CGCCGTG	19	804334	F
Red	GCAAAAC	37	17	80921188	CGCCCTT	17	78514477	F
	ATAGTCA	37	6	30039600	TGCCCAG	6	30147579	F
	GGCGGGA	37	X	12994598	TGCAAAA	X	12904519	F
	TTTTTTTC	37	6	33228461	CGTTGTT	6	33336439	F
Red	TCAGAAC	37	17	78997350	AGCGCCT	17	76611945	F
	CTGCGGC	37	6	29906977	CGGCTGG	6	30014956	R
Red	TGTCTCT	37	19	50249584	ACGGGGC	19	54941396	F
Grn	CACCCTG	37	20	43945364	CGGCCTT	20	43378778	F
Red	CACTTGG	37	11	888956	CGGCTGG	11	878956	F
	GTGAGCC	37	11	102191336	CGTAAAC	11	101696546	R
	TGTTTTG	37	3	46263244	GCCTTCA	3	46238248	R
	GGGCTGT	37	2	240143979	GAGGGAC	2	239808916	R
	ATAGATG	37	21	44110482	ACATTAT	21	42983551	F
	GATGGGT	37	16	819440	CGGGCCA	16	759441	R
	TCTGCAC	37	16	701656	CGGCCCC	16	641657	R
Red	ATCCTCA	37	5	150050950	TCGAGAA	5	150031143	R
	AGGGGGC	37	16	86593603	ATTGAGA	16	85151104	R
Red	CCCCCTT	37	20	25128777	TCTCACT	20	25076777	R
	CTCTGGC	37	2	102844110	TCAGAAA	2	102210542	F

Red	CGATCTG	37	X	153200109	GCGATTC	X	152853303	F
	AAGAGCC	37	11	56587595	CCATGGC	11	56344171	F
Red	CCCGCCC	37	4	7472958	TGTGTTC	4	7523858	F
	CTTTTCA	37	7	13985664	CGCAGAT	7	13952189	R
	TTTATTG	37	7	4228948	CGCCAAT	7	4195474	F
	AGTCAGA	37	16	47495768	CGCCGGC	16	46053269	R
Red	AATATCT	37	5	78281964	ACAGAGT	5	78317720	F
Red	GGACAAA	37	10	18549536	TGACGGT	10	18589542	R
	GGAACCC	37	16	2886233	CGTTCCC	16	2826234	F
Red	AGAGCCC	37	11	118781408	CGCCTCC	11	118286618	R
	CAGAGTC	37	13	111980537	GGCATCG	13	110778538	F
	AACTGCT	37	6	55956315	CATCCTT	6	56064274	R
	CTAATTA	37	6	19812885	CGCTGAA	6	19920864	F
	TTCATTA	37	5	40841493	CAAGCAA	5	40877250	F
	GTCCCAC	37	6	51953096	CGCCAGA	6	52061055	R
	GGTAAGT	37	22	27018010	ATTCAGG	22	25348010	F
	TTCCGGT	37	19	10231701	CGGTTGT	19	10092701	R
	CATGCTC	37	11	68842998	AGATGCT	11	68599574	R
	TCGGTCT	37	17	46622454	CCCTGTA	17	43977453	F
	TTCATGT	37	2	215887370	CGGATGA	2	215595615	F
	GGTGGGC	37	17	38440015	CGTCACT	17	35693541	F
	AGTTGCC	37	12	91729835	CGGTGTA	12	90253966	F
	CTACGAA	37	7	32526065	ACAGCAA	7	32492590	R
	TGTTATC	37	11	33760479	CCTTTTG	11	33717055	R
	CGGTAGT	37	3	125105935	CGCTGCT	3	126588625	R
	TGCAGGC	37	6	160023689	CGCACCA	6	159943679	F
Red	GGCTCAC	37	2	105853672	CGGCTCC	2	105220104	F
	TCTGCAT	37	15	92238013	CGGGGTT	15	90039017	F
	GAAAAAA	37	11	94782548	CACAGAC	11	94422196	F
	GTTTGGA	37	5	150677867	CGTCATT	5	150658060	R
	AGCTCTT	37	1	201852272	CCACCTA	1	200118895	R
Red	TCTGTGG	37	1	178456270	CGCGCGC	1	176722893	F
	CTGTCAT	37	10	19002068	CGTGGCT	10	19042074	F
Red	TGCCCCA	37	4	132896405	CGAGAAA	4	133115855	R
	CTTCTGC	37	Y	22682393	CGTAATA	Y	21091781	R
Red	ACGAGGC	37	5	138714136	CGGGGTT	5	138742035	R
	ACACCTC	37	16	71458897	ACGTGGA	16	70016398	R
	TCGGGGA	37	14	92410260	CGCACCC	14	91480013	F
	CTTCTCC	37	17	19314395	CAGAATC	17	19254988	F
	ACCTGTT	37	13	27825886	CGCTGCT	13	26723886	R
	TATCATC	37	12	76023400	CGGGGAC	12	74309667	R
Red	CCGGCTG	37	16	88598739	CGGTGCC	16	87126240	F
	AAATAGC	37	6	13408158	CGGAGAT	6	13516137	F

	ATTAAAA	37	12	109214864	AAAGCAT	12	107738993	F
	GGAAACT	37	15	34144832	CGCCATT	15	31932124	F
Grn	GACACCA	37	19	56612697	GCTTGGG	19	61304509	R
	TAAAAGA	37	17	7015607	GGGCTGC	17	6956331	F
	TACCGGC	37	X	21393407	AGGGGA(	X	21303328	F
	AGTTTTA	37	6	28447087	TCAGGGC	6	28555066	R
	ATTTGGA	37	11	111815419	CGGCCAC	11	111320629	F
	TGGGAAC	37	5	126409454	CGGACTC	5	126437353	R
Red	CTCTCCT(	37	7	140267040	CGGCTGA	7	139913509	R
	CAGATCT	37	6	90714949	CGGAAGT	6	90771670	F
	CTCGTTT(	37	17	76676673	CGGAGAC	17	74188268	F
	ATTGTCA	37	14	56991775	CGCCATT	14	56061528	R
Grn	CTGCCCG	37	8	48677407	CGGAGCT	8	48839960	R
	TTTTTAGA	37	15	62767926	CCTTAAT	15	60555218	F
Grn	CTTCCGC	37	5	140568535	GCGGGTA	5	140548719	F
	ATAGTAC	37	2	145226929	CGAAGGC	2	144943399	R
	GGAGGTC	37	10	131560029	CGCCGAC	10	131450019	R
Red	TCGAGGC	37	8	142231004	GTGGGCT	8	142300186	F
	AGACCTC	37	19	1950427	CGCGCCT	19	1901427	F
	ATTAGAC	37	17	13972210	GGTGGAA	17	13912935	R
	ATCCCCA	37	12	119591747	CGCACCA	12	118076130	F
	CTAAAGT	37	6	31607674	CGGGATT	6	31715653	F
	AGCAGCC	37	16	8943122	CACTTTA	16	8850623	F
	AATGTGT	37	15	90796557	TGGGTAC	15	88597561	F
Red	GTCTTGA	37	6	144643175	CGGGCGT	6	144684868	F
Red	GTGAGTG	37	16	51393415	AGCTCCT	16	49950916	F
	TGGACCT	37	17	80848259	CGGGAAC	17	78441548	R
	TTTTTACT	37	15	61327619	CGGCAGC	15	59114911	F
	TGTCCTT(	37	16	57836960	CGCTCCA	16	56394461	F
Red	CCTCCCC	37	7	96652115	TCAAGGA	7	96490051	R
	CAATCCT	37	14	70321227	CGGGTTC	14	69390980	F
	GATCAAT	37	11	124439146	CGGGGCA	11	123944356	R
Red	TGCGCAA	37	Y	21906893	CGAAGGA	Y	20366281	F
	AGCTTTCT	37	6	24832728	CGCATGA	6	24940707	R
Red	GGTCCCT	37	13	100548780	GGGCTTC	13	99346781	F
	TCTTCAT(	37	X	19361727	AGTGTAC	X	19271648	R
	CTGCAGC	37	16	67009163	CGCATCT	16	65566664	F
Grn	GAGTTCC	37	5	140531474	GGGTACA	5	140511658	F
	TAAAGAA	37	7	30810504	TTTCCCGT	7	30777029	F
Red	AAAGCGC	37	7	39649443	TGCTGCA	7	39615968	F
	AAAATCC	37	4	3640481	CGTCTTT(	4	3610279	R
Red	ATTAAGA	37	6	101847318	CGGCTGC	6	101954039	R
	GGGATGC	37	2	37684473	CTTTTCCA	2	37537977	F

	CTTGTAG	37	19	17953473	GGGCCGC	19	17814473	F
	ATAAAAT	37	11	4389638	ACATTCT	11	4346214	R
	CCAGGTT	37	14	32350428	GAGCACT	14	31420179	R
Red	CGGGGGC	37	5	140182129	TTCACTG	5	140162313	R
Grn	GGAAATC	37	6	33386103	CGGCTCT	6	33494081	R
Red	GCGTCAT	37	3	11610239	CGCCTCC	3	11585239	F
	AAGATGA	37	7	6836436	TGGAAGC	7	6802961	R
	CCACGGC	37	19	468830	CGCAGTG	19	419830	F
	GGATCAC	37	13	114872606	CGTACCC	13	113890708	F
	TTAACCT	37	7	154720213	AAAAGTC	7	154351146	R
	CTTATTC	37	3	11178798	CGGCAGC	3	11153798	F
Red	GGCGGAC	37	5	126409153	CGCCGCC	5	126437052	F
	GTAAGGA	37	12	122129167	CGAGTGC	12	120613550	R
	TTCTGTC	37	6	32547167	CCTTATC	6	32655145	R
	AAGATGA	37	18	76629213	CGGCTGT	18	74730201	F
Red	GGGGCAC	37	16	89147812	TGGTCAG	16	87675313	R
Red	CAGAGGA	37	22	37921640	CGCCAGC	22	36251586	R
Grn	TTTGAAC	37	21	40759574	CGCAGCC	21	39681444	R
	AACTTGA	37	7	15555129	CGGGAAT	7	15521654	R
	GGCCCAT	37	12	31149418	CGCATAG	12	31040685	F
	CTCTCCT	37	8	1365049	ATTTGTT	8	1352456	R
	GGCAGCT	37	7	9767724	CGGCTGA	7	9734249	F
Red	TGAGGGT	37	16	2892782	CGCACAC	16	2832783	R
Red	GCCGCAC	37	11	842605	CGGCTCC	11	832605	F
Red	GGGCTCT	37	4	102712216	CGCCCTG	4	102931239	R
	AAAATGA	37	10	50231850	CGGAGTC	10	49901856	F
	CAGAGCT	37	3	140786844	CGAGTGC	3	142269534	R
	CAACCAT	37	11	67795659	TCAGAGC	11	67552235	F
	CTTTGGC	37	2	215362367	CGGAAAT	2	215070612	R
Grn	CTCGGGG	37	17	41477350	ACAAACA	17	38832876	F
	AGCCCGC	37	5	1594330	GGCTGGC	5	1647330	F
	GAGCCCC	37	17	74868725	CTCATGC	17	72380320	R
	GTCACGG	37	17	78472016	CGCCTGC	17	76086611	F
Grn	AGCATTA	37	16	49620536	CCCTGGC	16	48178037	F
Red	TCACTAC	37	13	20139028	GCGGCGA	13	19037028	F
	CCCAGCC	37	17	19277143	CATGGAC	17	19217736	F
Red	GCCCTGT	37	19	292210	GGCACCC	19	243210	R
	TAACTCT	37	7	158578750	CGTACTC	7	158271511	R
	TCGGCAA	37	22	49498376	CTCAACC	22	47884380	F
Red	AGCCAGC	37	X	11683995	CGCCGCT	X	11593916	R
Red	TACAACA	37	4	1203001	CGGCTGG	4	1193001	F
	AGACAAC	37	2	11515031	AATGGCC	2	11432482	R
	AGGTTCT	37	1	184121585	CGGGGCT	1	182388208	R

	CTGTCAT	37	12	54371246	ATGGTAG	12	52657513	R
Grn	ACATCAC	37	22	22987126	CGCGGGT	22	21317126	R
	AGCTGCT	37	1	1226776	GATGCTG	1	1216639	F
Red	GGCAAGA	37	15	74249189	CGGATGC	15	72036242	F
	GTCTATA	37	1	233636022	CGCCAAA	1	231702645	F
	AGTGATC	37	11	46732376	GTGCTGG	11	46688952	F
	GTTTACC.	37	7	94034528	CGGGTTT	7	93872464	R
	TAAGAAA	37	17	48067475	GAGGTGC	17	45422474	F
	CAGAGAC	37	17	79816678	CGACAGC	17	77409967	F
	TGGGAAC	37	4	7763703	GTCACGG	4	7814603	R
	TCAGCCT	37	16	89258862	CACTGGT	16	87786363	R
Grn	GCGCGGC	37	10	135343280	TTGTGAT.	10	135193270	F
	GGCCCCT	37	17	70680294	CGGATAA	17	68191889	R
Red	GAGCTCC	37	6	27447492	CACGTCA	6	27555471	R
Grn	GCGGGGC	37	20	43935291	GTGGTGG	20	43368705	R
	CAGTGTT	37	2	127772338	ACCTCTG	2	127488808	F
	GAAGAGC	37	6	29706048	CCACTAG	6	29814027	R
	CCCATCC	37	11	45628269	CGAGGCA	11	45584845	F
	AAGTCAC	37	4	82057467	CTGAACC	4	82276491	R
Red	AGTTCCG	37	21	47716529	AGCAGTT	21	46540957	F
Red	GGGAGGC	37	2	87018054	GCGGGGC	2	86871565	F
	TACCCAG	37	X	118892422	TGGAGCT	X	118776450	R
Red	AAAAGGT	37	4	8155589	AGACGGA	4	8206489	R
Red	GCGTCCG	37	X	15353768	CGTCCGA	X	15263689	F
	GAGGGCC	37	X	67913388	CGCCAGA	X	67830113	R
	CCCAGGC	37	19	43709360	CGTGCAC	19	48401200	R
	GCTCTTT	37	6	31275767	CGACAAC	6	31383746	R
	GCTGTGG	37	11	1891872	CGGCACC	11	1848448	R
	GGTGTAG	37	11	2171007	CTTAGAG	11	2127583	R
Grn	GCTTAGC	37	1	1992046	CGGCGTG	1	1981906	F
	GTAACTG	37	16	51475605	CGAGCAC	16	50033106	F
	GCCAGAC	37	3	38664451	TGTGCAG	3	38639455	F
	ACAAGTT	37	7	81481504	CACAGCT	7	81319440	R
	CACATCA	37	2	20793466	ATACACA	2	20656947	F
	CCGGGGT	37	19	52302551	CCCAGGT	19	56994363	F
	CGGCGGC	37	4	1044797	CAGTGCT	4	1034797	F
	GTGTCAG	37	19	22193479	CCTCCAT	19	21985319	R
	ACTATAT	37	1	47488934	CGGCAGC	1	47261521	F
	TGGGATC	37	1	39853529	TATTCTT	1	39626116	F
	TCTTCAG.	37	6	30980847	GTGATCC	6	31088826	R
	GCATGAC	37	2	207042563	CGGTTAT	2	206750808	F
	ATTGGGA	37	14	53878048	GGCCAGC	14	52947798	F
Grn	GGTGGTC	37	11	49914049	AGAACTC	11	49870625	F

Grn	GGGATGC	37	21	37442289	AGAGACT	21	36364159	F
	AAAGAGA	37	10	105357975	CGGGCTG	10	105347965	R
Red	GGGAAAA	37	X	64196642	CGCTCCC	X	64113367	F
	ACTCGCA	37	5	140625750	GGGGATC	5	140605934	R
	GATTTAC	37	19	6746538	TTGCCCC	19	6697538	R
	GACAGGC	37	9	130516695	GCCTCTC	9	129556516	F
Red	AGATGAA	37	15	96887867	TGGCGCG	15	94688871	R
	GACTCTC	37	5	180033511	CGCTCAG	5	179966117	F
Red	GGTTTTC	37	6	30079256	CGCTATT	6	30187235	R
Red	GAAATAC	37	19	52134296	TTCCTTG	19	56826108	R
Red	GGAGGAC	37	X	100914892	ACGACGC	X	100801548	R
	CAAACTT	37	17	56273748	TGCTGTG	17	53628747	F
	GGGGAAC	37	Y	6778543	CGCCTCC	Y	6838543	R
Red	GGCCCCC	37	17	5019638	CGGGGCT	17	4960362	F
	CTGTAGT	37	6	32492198	CGTGAGT	6	32600176	R
Grn	TTCTAGT	37	6	30038929	ACGAGAC	6	30146908	R
	AAAAAAA	37	5	123036376	CAAGCAC	5	123064275	F
Red	TTGGCCT	37	17	56565286	CGCCGCG	17	53920285	R
Red	GCCGAGC	37	5	2169769	CGCCCTC	5	2222769	F
	GGCTCCA	37	10	103406305	CGCCTTG	10	103396295	R
Red	CCGGTTT	37	15	101715819	CGAGCTC	15	99533342	F
	TATTCCG	37	5	1594863	GTGTTGG	5	1647863	R
Grn	TAAGCCA	37	22	22987059	TTATTAA	22	21317059	F
	AGTGACC	37	2	232055581	TTCGTTA	2	231763825	R
Red	AAACATT	37	1	1672644	CGTGTTT	1	1662504	R
	GCAGCCG	37	19	50400714	GATCCTA	19	55092526	F
Red	TTCCTCC	37	2	127839539	GTCCTCT	2	127556009	F
	AAAATCC	37	6	29575566	GTCCTTC	6	29683545	R
Red	GCCGCCA	37	6	2958808	CGCTGGG	6	2903807	R
	TTGGGAA	37	10	11387394	ATGATAT	10	11427400	F
Grn	ATGCGCT	37	6	29648564	GCTCAGA	6	29756543	R
	GAAGTGT	37	3	32748004	CTTGTGG	3	32723008	R
	TTAATAA	37	7	1715242	GATACAA	7	1681768	R
Red	CAGTCCG	37	13	23270850	GCGGTGA	13	22168850	R
Red	GAAACTG	37	7	148638192	CGGGATT	7	148269125	F
	TAGGCGA	37	1	1687481	TGGGGCC	1	1677341	F
	GGGGGAC	37	19	19650978	TGAGACA	19	19511978	R
	TTCCTGA	37	2	178197762	CGTTGGT	2	177906008	F
	GTGCTGA	37	7	128731386	AGCTTGA	7	128518622	F
	TGAATCA	37	Y	16634795	CGGCGAT	Y	15144189	F
	TGCAGGA	37	8	143645661	CGCAGCA	8	143642663	F
	TTGCAAC	37	6	101846650	CGGCCAC	6	101953371	R
	CCACTCT	37	1	85686316	CAGGCTT	1	85458904	R

Red	ATGGGGA	37	16	88361648	CGGCGAC	16	86919149	F
	GTTTCCT	37	10	32428473	CGCCTTG	10	32468479	R
	TTTGCTG	37	20	39135472	TGGGGTC	20	38568886	R
	GGCTGAC	37	8	22266134	GCTGCTC	8	22322079	R
Red	CCTGGGT	37	7	965995	GAGGTGC	7	932521	F
Red	CAAGACC	37	10	99338074	GGACCTG	10	99328064	R
	CAGGGGC	37	7	155623690	GCTTCGG	7	155316451	F
Red	GCAGAAC	37	18	77126192	CGGTCAC	18	75227180	R
Grn	CGGAGGC	37	2	26951130	ACATGGA	2	26804634	F
	TGACCTC	37	19	48374536	GCATCTC	19	53066348	F
Red	CGGTGCC	37	16	87022483	CGGCGGT	16	85579984	R
	GAAGATT	37	2	151061125	GTGAAGA	2	150769371	F
Grn	AGCCCCT	37	12	9555721	GTCCCAA	12	9446988	F
	AAAAGCA	37	6	160024002	CGCGGGC	6	159943992	R
	GCATTTT	37	7	94039079	GTCCTCT	7	93877015	F
	TCAAAGT	37	18	14427976	CGTCCAA	18	14417976	R
	TACCTAG	37	Y	21877683	CGTCCCC	Y	20337071	F
Grn	TGGGTCC	37	15	23035493	TGGGGGC	15	20586934	R
	ATGTGCG	37	3	131068941	CGGCTTC	3	132551631	R
	AAACAGA	37	16	90095949	CGTCACA	16	88623450	R
Grn	CCCGGCC	37	20	61447330	CGCCCGG	20	60917775	F
	TTTGTGT	37	3	38346525	CGGCAAC	3	38321529	R
Grn	TGATGAG	37	19	43711249	AGGGAGC	19	48403089	R
Red	GGGCTCG	37	12	57780104	GCGGGAC	12	56066371	R
	TGAAAAC	37	1	235099089	CGGGTTC	1	233165712	R
	AAGAGAT	37	X	48124969	AGAGCTC	X	48009913	R
	AAATTTC	37	1	2885085	GGACCAC	1	2874945	F
	GCTTGCG	37	1	1571801	TGTTGTA	1	1561664	R
Red	CCACTGT	37	15	75248086	CGCAACA	15	73035139	F
	GGTCTAG	37	1	247802703	CCCGAAC	1	245869326	R
	TCTGCAT	37	10	129685902	CGGCTTT	10	129575892	F
	ATAGCTC	37	15	24673917	GACACAA	15	22225010	F
	AAGAGGA	37	17	76858248	CGAGTCG	17	74369843	R
Grn	CCTCGCA	37	12	9722091	CGGCTGG	12	9613358	F
Red	GTTCCAG	37	2	97136508	CGGTCCC	2	96500235	F
	GGGTAAT	37	3	127256188	CGCCTGG	3	128738878	R
	TATTGCT	37	1	78602941	ATTAAGA	1	78375529	F
Grn	CGCGGGT	37	6	30419551	AACCCGA	6	30527530	F
	CAGGGGT	37	17	203392	CTCCACA	17	203392	F
Red	GCTGCTG	37	5	140712441	CGCCTCC	5	140692625	R
	CCGAGGA	37	1	16472728	AATCCGG	1	16345315	R
Red	TTGACGT	37	2	200320750	CGCCGCT	2	200028995	F
	ATTAGAG	37	2	15498900	AACCAAC	2	15416351	R

	ATTTCTT	37	6	161459571	CTGCAAC	6	161379561	R
	GGGTTCT	37	2	102759782	CGTAGCC	2	102126214	R
	ACGATAC	37	22	31498863	GCTGGAA	22	29828863	R
	TGACAAC	37	12	133177383	GGGGCTC	12	131687456	F
Red	TTTTCCA	37	5	140569144	TCTGGTG	5	140549328	R
	AGACCAC	37	3	189901160	CGCTGAA	3	191383854	F
	CCCCACT	37	11	60608634	GTGAGCA	11	60365210	F
	TTCAACA	37	13	113105794	CGGCTCT	13	112153795	R
Grn	CCGCTCT	37	17	73056755	GCCAGGT	17	70568350	R
	GTTGGCA	37	15	81666528	CGGGCAC	15	79453583	R
	CTAATAA	37	2	169920014	CGAGTAC	2	169628260	F
Grn	CGCTGCA	37	6	117802710	CGCGCTG	6	117909403	R
	CGGGGTC	37	22	46460338	AGAGATC	22	44839002	F
	TGTGCAG	37	19	10928172	TGTACAC	19	10789172	R
Red	AAAGGGA	37	12	95840848	CGGCCCT	12	94364979	R
Grn	GGGAGCT	37	12	115173713	GGTTGAT	12	113658096	R
	CCTAGAT	37	17	1053794	GTTGCCT	17	1000544	F
	AGTGTAG	36	22	49888838	ACTGACT	22	48274842	F

Red

Red
Red
Grn
Red
Grn

Red

Grn

Red
Red

Grn

Red

Grn
Red
Red

Red

Grn

PROBE_SN	PROBE_SN	RANDOM_	METHYL2	UCSC_REF	UCSC_REF	UCSC_REF	UCSC_CP	RELATION
				AGAP1;AC	NM_01491	Body;Body	chr2:23702	N_Shore
				IL1RAPL2	NM_01741	TSS200	chrX:10381	N_Shore
rs78011553	rs74038932							
	rs5024308			SNTG2	NM_01896	Body	chr2:11630	S_Shore
	rs59476302			PIGQ;PIGQ	NM_14892	Body;Body	chr16:6294	N_Shore
							chr6:31650	Island
rs8192525				HTR1D	NM_00086	TSS200		
rs35314525								
rs79733927	rs60223258			CLEC16A	NM_01522	Body		
							chr12:5408	S_Shore
rs75093195	rs9925461			NAT15;NA	NM_00108	5'UTR;5'U	chr16:3507	S_Shelf
				TOP1MT	NM_05296	Body	chr8:14439	Island
				CROT;CRO	NM_02115	Body;Body		
							chr6:16953	Island
				GALP;GAL	NM_00114	5'UTR;5'UTR		
	rs79738529			PRKCE	NM_00540	Body		
	rs75239392						chr2:24283	S_Shelf
				PCSK9	NM_17493	TSS1500	chr1:55505	N_Shore
				LOC73227	NR_02440	Body		
							chr2:23708	N_Shore
				TRPC3;TR	NM_00330	1stExon;Bo	chr4:12285	Island
rs3760027				ALG1	NM_01910	TSS1500	chr16:5121	N_Shore
				C1orf57	NM_03232	Body	chr1:23308	S_Shelf
rs28421229								
				ATP1A4;AT	NM_14469	1stExon;5'UTR		
rs2182924							chr13:1129	N_Shore
				CDH20	NM_03189	Body	chr18:5922	N_Shelf
	rs3751568			CYFIP1;CY	NM_01460	Body;Body		
	rs75038007			MRPL28	NM_00642	Body	chr16:4198	Island
			TRUE	MPZ	NM_00053	TSS1500	chr1:16128	N_Shelf
				ASCC1	NM_01594	Body		
rs12518292				DPYSL3	NM_00138	Body		
				GLIPR1L2	NM_15243	TSS200	chr12:7578	Island
							chr16:1069	Island

			BCORL2	NR_00292	Body	chrY:21664	N_Shore
		TRUE					
			TNXB	NM_01910	Body		
	rs1160317		RWDD3;R	NM_00112	Body;Body	chr1:95699	S_Shelf
	rs1467834		C1orf94;C1	NM_00113	Body;5'UTI	chr1:34642	S_Shelf
			MIR886	NR_03058	TSS200	chr5:13541	Island
	rs55999058		THSD4	NM_02481	Body		
			DST;DST;I	NM_00114	Body;TSS200;Body;TSS200;TSS20		
			SPSB1	NM_02510	5'UTR		
	rs72193420					chr8:12456	Island
rs9493599	rs11753937		EYA4;EYA	NM_17210	Body;Body;Body		
	rs33913065		PFN4	NM_19934	Body	chr2:24345	N_Shore
rs2424830						chr20:2955	Island
			PKD1L2;PI	NM_05289	TSS1500;TSS1500		
			C3orf24;C3	NM_00116	TSS200;TS	chr3:10149	Island
	rs2085323					chr3:11771	S_Shelf
rs11589175	rs35026651		MEGF6	NM_00140	Body	chr1:34474	N_Shore
			ITGB5	NM_00221	Body		
			SSBP3;SSE	NM_00100	Body;Body	chr1:54821	Island
			EP300	NM_00142	Body	chr22:4148	S_Shelf
			MFAP4	NM_00240	TSS1500		
			ATP11A;A	NM_01520	Body;Body	chr13:1134	N_Shore
	rs72616127					chr4:66656	N_Shore
			EBF1	NM_02400	Body		
rs67999531	rs9269968		HLA-DRB	NM_00212	Body	chr6:32551	Island
			TTTY14	NR_00154	TSS200	chrY:21238	Island
			AIFM1;AII	NM_14581	TSS1500;T	chrX:12929	Island
			DDX11;DD	NM_00439	Body;Body;Body		
			KIAA1949;	NM_00113	Body;1stEx	chr6:30654	N_Shelf
rs11541079	rs6746		LOC33879	NR_00280	Body	chr12:1222	N_Shore
			LOC10029	NR_02841	TSS1500		
	rs36101953		C10orf46	NM_15381	TSS1500	chr10:1205	S_Shore
			ABR;ABR;	NM_02196	Body;Body	chr17:9262	S_Shelf
			KIAA0319I	NM_02487	5'UTR	chr1:36022	N_Shore
	rs73426852		CYFIP1	NM_01460	5'UTR		
						chr8:13412	N_Shore

			LOC73178	NR_02679	TSS200		
			TMSB4X;T	NM_02110	TSS200;TS	chrX:12993	Island
rs17325066	rs77812985		RPL32P3;S	NR_003111	Body;TSS1500		
rs11651991	rs66505868		TBC1D16	NM_01902	Body	chr17:7792	Island
	rs2234570		WHSC2	NM_00566	Body	chr4:19874	N_Shore
			CTSK	NM_00039	5'UTR		
	rs6507921		RPL17;SN	NM_00098	Body;TSS1	chr18:4701	N_Shelf
						chr12:1263	Island
			WDR33;W	NM_00100	TSS1500;T	chr2:12856	S_Shore
			ESRRG	NM_00113	5'UTR	chr1:21730	N_Shore
			NXN	NM_02246	Body		
rs57102073	rs10046752		CSMD1	NM_03322	Body		
			PCDHB16	NM_02095	1stExon	chr5:14056	N_Shore
						chrY:14649	S_Shore
	rs4818734		UBE2G2;U	NM_18268	5'UTR;Bod	chr21:4622	N_Shore
						chr16:2830	N_Shelf
	rs12446637					chr16:8823	S_Shore
			KRTAP2-4	NM_03318	TSS1500		
						chr2:23185	Island
	rs35405169		GATA5	NM_08047	Body	chr20:6104	N_Shore
						chr8:13145	N_Shore
			EIF2C2;EI	NM_01215	Body;Body	chr8:14155	N_Shelf
						chr11:6738	Island
		TRUE	LGALS1	NM_00230	TSS1500	chr22:3807	N_Shore
rs72806131			ANK3	NM_02098	Body		
			MYOM2	NM_00397	Body		
						chr9:90621	N_Shore
			NAPSA	NM_00485	3'UTR	chr19:5086	Island
						chr1:16158	S_Shore
rs72360220	rs76383292					chr4:19022	Island
						chr1:24780	S_Shore
			CDRT4	NM_17362	3'UTR		
			CYP2E1	NM_00077	Body	chr10:1353	Island
rs61432632							
			SELL;SEL	NM_00065	Body;Body		
			KIF13B	NM_01525	Body		

			ACTN2	NM_00110	Body		
rs3846891	rs58350918						
			DCHS2;DC	NM_01763	Body;Body	chr4:15525	Island
rs1467013						chr1:83777	Island
			BTBD9;BT	NM_00109	Body;Body;Body		
			TBX3;TBX	NM_00599	Body;Body	chr12:1151	Island
rs7408083	rs7408082		NFATC1;N	NM_00616	Body;Body	chr18:7723	Island
						chr9:13545	N_Shore
			ARHGEF1	NM_01478	Body;Body		
rs17159355			SLC43A3;S	NM_19932	TSS1500;T	chr11:5719	S_Shore
			ASPDH;AS	NM_00111	TSS200;Bo	chr19:5102	N_Shelf
			NOTCH4	NM_00455	Body		
			ZSCAN18;	NM_00114	Body;5'UTI	chr19:5860	N_Shore
			HPCAL1;H	NM_00214	5'UTR;5'UTR		
			TAGLN3;T	NM_00100	Body;Body;Body		
						chr15:2472	Island
						chr16:3057	Island
						chr3:13974	Island
rs2571420	rs74218766		HLA-A	NM_00211	TSS1500	chr6:29910	N_Shore
			NUDT17	NM_00101	TSS200	chr1:14558	Island
		TRUE	AGA	NM_00002	TSS1500	chr4:17836	S_Shore
			NXPH1	NM_15274	Body	chr7:84731	S_Shore
rs33685			FAM134B;	NM_01900	TSS1500;Body		
			HLA-DPA1	NM_03355	Body		
rs61023017						chr7:14013	Island
rs77215738							
	rs41307264		ARMCX1	NM_01660	5'UTR	chrX:10080	Island
		TRUE	STC1	NM_00315	TSS200		
						chr1:28470	Island
	rs76859177		TRIM24;TI	NM_00385	Body;Body		
rs2385400			RESP18	NM_00100	Body	chr2:22019	S_Shore
			HLA-DRB	NM_00212	Body	chr6:32489	S_Shelf
						chr8:59990	N_Shore
						chr10:4934	Island
						chr2:17693	Island
			ROR1;ROF	NM_00501	Body;Body		
	rs12752760						
			SBNO2;SB	NM_00110	Body;Body	chr19:1118	Island
			CYTH1;CY	NM_01745	Body;Body		
			AEBP2;AE	NM_15320	Body;Body		

rs922217			PRX;PRX	NM_02095	Body;Body	chr19:4090	Island
			NCRNA00	NR_00154	Body		
rs17505733			SH3D19;SH	NM_00112	TSS200;5'UTR;5'UTR		
						chr19:4153	Island
			GTF2A1L;	NM_17219	TSS200;Bo	chr2:48844	Island
			CRCP;CRC	NM_00104	Body;Body;Body;Body;Body		
			MED12L	NM_05300	Body		
			OTOF;OTC	NM_00480	TSS1500;TSS1500;TSS1500;Body		
			MAB21L1;	NM_00558	1stExon;Bo	chr13:3604	Island
	rs6934989		TMEM63B	NM_01842	Body	chr6:44119	N_Shore
			RLTPR	NM_00101	Body	chr16:6768	Island
			PIK3CG;PI	NM_00264	5'UTR;1stE	chr7:10650	N_Shelf
			NRN1L	NM_19844	1stExon	chr16:6791	Island
		TRUE					
	rs28662301		BANP;BAN	NM_01786	Body;Body		
rs74740134							
			TMEM165	NM_01847	Body		
	rs8060043		ACSF3;AC	NM_17491	Body;Body	chr16:8916	Island
			VEGFA;VE	NM_00117	TSS1500;T	chr6:43737	N_Shore
rs11145847	rs3739939		FAM69B	NM_15242	Body	chr9:13961	N_Shore
						chr5:17084	N_Shore
rs17841362							
			SPATS2	NM_02307	5'UTR	chr12:4976	S_Shelf
			LOC28569	NR_02711	Body		
						chr1:20940	Island
	rs35980577		GALNT2	NM_00448	Body		
			DNHD1	NM_14466	Body	chr11:6591	N_Shore
	rs73700237		NCALD	NM_00104	TSS1500	chr8:10313	S_Shore
			RAB1A;RA	NM_00416	Body;Body	chr2:65356	N_Shore
			SRPK2;SR	NM_18269	Body;Body	chr7:10488	N_Shelf
			PLD6	NM_17883	TSS200	chr17:1710	N_Shore
rs78475669							
			LOC10010	NR_00358	Body;Body		
						chr10:1299	Island
						chr6:15671	N_Shore
			PMM1	NM_00267	Body	chr22:4198	N_Shore

	rs9796214					chr13:1147	N_Shelf
						chr8:72917	N_Shore
rs7682744			COPS4	NM_01612	TSS1500		
			ASPSCR1	NM_02408	Body	chr17:7996	N_Shore
						chr5:74907	S_Shore
	rs7571693		FMNL2	NM_05290	Body		
	rs7204640		TEKT5	NM_14467	Body		
			AKR1B15	NM_00108	TSS200		
						chr19:5083	Island
			CCHCR1;C	NM_00110	Body;Body	chr6:31125	N_Shore
rs994379							
	rs6863024		ADAMTS2	NM_01424	Body	chr5:17855	N_Shore
			PLXNC1	NM_00576	Body		
			LOC64259	NM_00114	Body	chr18:5196	N_Shelf
			WFDC6	NM_08082	TSS1500		
rs72653146	rs72653147		COL1A1	NM_00008	Body		
			FANCI;FA	NM_00111	TSS1500;T	chr15:8978	N_Shore
			CPNE2	NM_15272	Body		
			TACSTD2	NM_00235	1stExon	chr1:59042	Island
						chr13:1006	S_Shore
	rs79274715		STK32C	NM_17357	Body		
						chrY:17567	Island
						chr17:7895	S_Shelf
						chr18:7607	N_Shore
	rs7768524						
						chr1:11328	N_Shore
			AKR1E2	NM_00104	TSS200	chr10:4868	Island
	rs894418		PRRX2	NM_01630	Body	chr9:13245	N_Shore
						chr7:17840	S_Shore
rs11124201						chr2:23988	S_Shore
			WHSC1;W	NM_13333	Body;Body;3'UTR;Body;Body;Body		
						chr7:31003	Island
						chr8:23380	N_Shore
	rs55719509	TRUE	LASS3	NM_17884	5'UTR	chr15:1010	Island
			TUBB4Q	NM_02004	TSS200	chr4:19090	Island
	rs12483886		MPPED1	NM_00104	5'UTR	chr22:4380	S_Shelf

		ACCN1;AC	NM_00109	3'UTR;3'UTR		
	rs80312210	NUBP1	NM_00248	Body		
	rs9601330					
	rs76507469	ANO6;AN	NM_00114	Body;Body;Body;Body		
		RXRA	NM_00295	Body	chr9:13729	N_Shore
		PTPRN2;P	NM_00284	Body;Body;Body		
	rs76188937	CBFA2T3;	NM_17593	5'UTR;Bod	chr16:8899	N_Shelf
		ITGAM;IT	NM_00114	3'UTR;3'U	chr16:3134	S_Shore
		ERRFI1	NM_01894	5'UTR	chr1:80855	N_Shore
		TAS2R7	NM_02391	1stExon		
		ESX1	NM_15344	Body	chrX:10349	N_Shore
	rs3215400	CDA;CDA	NM_00178	1stExon;5'UTR		
		OR2L13;O	NM_17591	1stExon;5'U	chr1:24810	Island
	rs3094136	TRIM31	NM_00702	Body	chr6:30071	S_Shore
					chr6:15936	Island
		TRPM8	NM_02408	Body	chr2:23484	Island
		OR2T3	NM_00100	TSS1500		
		PRKCB;PR	NM_00273	Body;Body		
rs61986373	rs61986372					
	rs10131376	SLC25A21	NM_03063	Body;Body		
rs74361853		SPON1	NM_00610	Body	chr11:1398	Island
rs35176217		OR2T11	NM_00100	TSS1500		
rs35732565	rs35629555	KCNAB2;	NM_00363	TSS1500;T	chr1:60862	N_Shore
rs77207688		AGBL4	NM_03278	Body	chr1:49364	Island
		CXorf22	NM_15263	TSS200		
		THBS1	NM_00324	Body	chr15:3987	S_Shelf
	rs71390811					
	rs79295829				chr1:14655	Island
rs10100209		SOX7	NM_03143	Body	chr8:10583	Island
	rs78980985	KCNK2;K	NM_00101	1stExon;5'UTR		
		SYTL1	NM_03287	Body	chr1:27675	Island
					chr18:7672	Island
		TRPM8	NM_02408	Body	chr2:23484	Island
	rs13308736	PTPRN2;P	NM_00284	Body;Body	chr7:15824	N_Shore
rs74631495	rs12143527	CR1L	NM_17571	Body		
					chr11:6738	Island
	rs72629157	ZNF835	NM_00100	Body	chr19:5718	N_Shelf
	rs7954579					
					chr16:3348	S_Shore
		HSF5;HSF	NM_00108	5'UTR;1stE	chr17:5656	Island

	rs11665759				chr19:3803	Island
		VMAC	NM_00101	Body	chr19:5903	S_Shelf
		C14orf166	NM_01603	Body		
	rs59490038	SCN1A;SC	NM_00116	TSS1500;TSS1500;TSS1500		
					chr14:1052	Island
rs7533656		HRNR	NM_00100	TSS200		
		PPP1R2P1	NR_027771	Body	chr6:32847	Island
	rs55784104	GRB2;GRE	NM_00208	Body;Body		
		TRIP12	NM_00423	Body		
					chr7:27208	N_Shore
rs73911131						
		FOXO4;FO	NM_00593	3'UTR;3'UTR		
		STK40	NM_03201	TSS1500	chr1:36851	S_Shore
					chr18:1194	Island
rs79421768		LOC14869	NR_02681	TSS1500		
		LHCGR	NM_00023	Body		
rs2669153		CUBN	NM_00108	Body		
					chr22:2045	S_Shelf
rs34779657		RNF39;RN	NM_17076	Body;Body	chr6:30038	Island
		SUMO3	NM_00693	Body	chr21:4623	Island
		ADAMTS1	NM_00698	Body	chr21:2821	N_Shore
		SLC2A14	NM_15344	TSS1500	chr12:8025	Island
rs940453		ZNF775	NM_17368	Body	chr7:15009	Island
		C4orf31	NM_02457	5'UTR	chr4:12199	N_Shore
		CAPZB	NM_00493	Body	chr1:19811	N_Shore
		ARMCX5;	NM_00116	5'UTR;TSS	chrX:10185	Island
rs35682610	rs56280220	HSPA1B	NM_00534	1stExon	chr6:31795	S_Shore
		PRDM7;PR	NM_05299	3'UTR;3'UTR		
		CBFA2T3;	NM_17593	5'UTR;Bod	chr16:8899	N_Shelf
					chr1:16141	N_Shore
rs9282000	rs9272416	HLA-DQA	NM_00212	TSS200		
rs31852		PCDHB2	NM_01893	1stExon	chr5:14047	Island
					chrX:39680	Island
		SHF	NM_13835	TSS200	chr15:4549	S_Shelf
					chr1:21731	S_Shelf
	rs56142053	ATXN1;AT	NM_00112	5'UTR;5'UTR		
		VWA5B2	NM_13834	Body	chr3:18394	S_Shelf
		MATN2;M	NM_03058	Body;Body		
		UBE2H;UE	NM_00334	Body;Body		

		MARK3;M	NM_00112	Body;Body;Body;Body;Body		
rs1063349		HLA-DQB	NM_00212	3'UTR		
		ARHGAP2	NM_01507	Body;Body		
	rs7543016	CMPK1;CM	NM_00113	1stExon;1st	chr1:47799	Island
					chr12:6613	S_Shelf
		ZNF254	NM_20328	Body		
					chr4:36711	N_Shore
		PRKAG2;P	NM_01620	Body;Body		
					chr3:13245	Island
		TMPRSS8	NR_02686	TSS200	chr16:2892	Island
		RPS10P7	NR_02666	TSS1500		
					chr1:40598	N_Shore
		SLC14A1;S	NM_01586	5'UTR;5'UTR;TSS1500;5'UTR		
rs12025174		PKP1;PKP1	NM_00029	Body;Body		
	rs79977228				chr1:24531	N_Shelf
		DIP2C	NM_01497	Body		
	rs2491259	HLX	NM_02195	Body	chr1:22105	N_Shore
		BAIAP3	NM_00393	Body	chr16:1393	Island
rs16872792						
		SDHAP1	NR_00326	Body		
rs34884782		PCDHA6;P	NM_03184	Body;Body	chr5:14022	N_Shore
		GTF3C1	NM_00152	Body		
		PCDHA6;P	NM_03184	Body;Body	chr5:14024	Island
		CCDC57	NM_19808	5'UTR	chr17:8017	N_Shore
	rs10934303	GAP43;GA	NM_00204	Body;5'UTI	chr3:11537	N_Shore
					chr6:16425	Island
					chr1:17215	Island
		GLIS1	NM_14719	5'UTR		
		WBP2NL	NM_15261	1stExon	chr22:4239	Island
		BTBD19	NM_00113	3'UTR	chr1:45278	S_Shore
rs35743243	rs35479904	FAM120B	NM_03244	Body	chr6:17068	Island
					chrY:94781	Island
		CHP2	NM_02209	TSS1500	chr16:2376	N_Shore
		RASAL3	NM_02290	Body	chr19:1556	Island
		HSD17B7;I	NM_01637	5'UTR;1stE	chr1:16276	Island
		IRX6	NM_02433	Body	chr16:5536	Island
		XYLT1	NM_02216	Body		
		EIF2AK4	NM_00101	Body	chr15:4026	Island
		PALLD;PA	NM_01608	Body;Body		

		PHF1;PHF1	NR_027692	TSS1500;T	chr6:33377	Island
		PCDHB16	NM_02095	1stExon	chr5:14056	Island
					chr2:44314	N_Shore
	rs2412045					
		ZFX;ZFX	NM_00341	5'UTR;1stE	chrX:24167	S_Shore
	rs79939347	SGCZ	NM_13916	Body	chr8:15094	N_Shore
		RFPL2;RFI	NM_00109	TSS200;5'UTR;TSS1500		
		LMX1B	NM_00231	Body	chr9:12937	Island
		CASP7;CA	NM_03333	Body;Body;Body;Body		
	rs2533872	GNA12	NM_00735	Body	chr7:28834	N_Shore
	rs57272256	MSLN;MIF	NM_00582	3'UTR;TSS1500;3'UTR		
		BICD1;BIC	NM_00171	Body;Body		
		MIR2117	NR_031751	TSS1500		
		PNLIPRP2	NM_00539	Body	chr10:1183	Island
		ODZ3	NM_00108	Body		
		ZDHHC14;	NM_15374	Body;Body		
	rs2297428	GMEB2	NM_01238	Body	chr20:6222	Island
	rs229545				chr22:3759	N_Shore
rs80278387					chr7:56855	Island
rs12895946					chr14:1011	Island
		LOC10013	NR_033182	Body	chr4:11881	N_Shore
					chr11:8564	Island
		PSORS1C1	NM_01406	5'UTR		
					chrX:12925	Island
		BRF1;BRF	NM_00151	Body;Body		
		PCDHB7	NM_01894	1stExon	chr5:14055	N_Shore
					chr2:19551	N_Shore
		RBPMS2	NM_19427	3'UTR		
					chr2:24161	Island
		PRSS21;PR	NM_14495	Body;Body	chr16:2867	Island
	rs2223709				chr20:1187	N_Shore
		PCDHB16	NM_02095	1stExon	chr5:14056	N_Shore
		SRRM4	NM_19428	Body	chr12:1195	Island
	rs75580812					
		MUC5B	NM_00245	Body	chr11:1262	Island

rs3735106	rs3735105	C7orf27	NM_15274	Body	chr7:25832	N_Shore
rs2159234		LRRC61;A	NM_02394	1stExon;5'U	chr7:15001	Island
		PCDHB9	NM_01911	1stExon	chr5:14056	N_Shore
rs10475117					chr5:18567	Island
	rs6461991	HOXA9	NM_15273	TSS1500	chr7:27203	Island
					chr7:56242	Island
		RNF39;RN	NM_17076	Body;Body	chr6:30038	Island
					chr8:14593	Island
		SUPT7L;SI	NM_01486	TSS1500;1s	chr2:27886	Island
	rs73340969	HELZ	NM_01487	3'UTR		
rs11677287						
					chr3:18424	N_Shore
rs35670997		MYOM2	NM_00397	Body	chr8:20754	Island
		LHX2	NM_00478	TSS1500	chr9:12677	N_Shore
	rs72280493	RAD52	NM_13442	Body		
					chr1:28850	Island
					chr7:25898	S_Shore
		STAG2;ST	NM_00104	1stExon;TS	chrX:12309	S_Shore
		SEPT9;SEF	NM_00111	Body;Body;Body;Body;Body		
		ESR1;ESR1	NM_00112	5'UTR;TSS	chr6:15212	N_Shelf
rs12610907	rs1968241	ETFB	NM_00198	Body	chr19:5186	N_Shelf
		KIAA0513	NM_01473	3'UTR		
	rs2423084	SLC23A2;S	NM_20332	5'UTR;TSS1500		
rs2835668		DSCR9	NR_02671	TSS1500		
		HLA-DRB5	NM_00212	Body	chr6:32489	N_Shore
	rs300537	RADIL	NM_01805	Body		
					chr7:15726	N_Shore
		ITPR2	NM_00222	Body		
		ETS2	NM_00523	TSS1500	chr21:4017	N_Shore
		PCDHA7;P	NM_01891	Body;Body	chr5:14024	N_Shore
					chr2:33824	S_Shelf
		TNRC6B;T	NM_00102	Body;TSS1500;TSS1500		
		TRPM4	NM_01763	Body	chr19:4969	Island
rs34993694	rs2303639	ADAMTS2	NM_01424	Body	chr5:17856	N_Shore
	rs2233631	SPDEF	NM_01239	TSS1500		

							chr2:13649	N_Shelf
			TRUE	FAM83H	NM_19848	Body	chr8:14480	Island
				RORA	NM_13426	Body		
rs2065724								
				TMEM49	NM_03093	Body		
	rs77985030			COL6A3;C	NM_05716	5'UTR;5'UTR;5'UTR;5'UTR;5'UTR		
				MYO1B;M	NM_00113	Body;Body;Body		
				PLD6	NM_17883	1stExon	chr17:1710	Island
rs2213944				KIAA1949;	NM_00113	Body;1stEx	chr6:30654	N_Shore
				PRDM16;P	NM_02211	Body;Body	chr1:30809	Island
				HOXD4	NM_01462	Body	chr2:17701	Island
				PCDHA6;P	NM_03184	Body;Body	chr5:14023	Island
				CCDC62;C	NM_20143	Body;Body;Body		
							chr2:17693	Island
				ELF1;ELF1	NM_17237	5'UTR;1stExon		
				RASSF5;R	NM_18266	Body;TSS1	chr1:20673	N_Shore
				WDR60	NM_01805	Body		
rs12948785				CAMKK1;	NM_17220	Body;Body	chr17:3771	Island
		TRUE					chr3:13350	Island
							chr16:7309	N_Shore
				CCDC36;C	NM_00113	TSS200;5'U	chr3:49236	Island
				CYP24A1;	NM_00112	Body;Body	chr20:5278	N_Shelf
							chrY:14649	N_Shore
				CPLX2;CP	NM_00100	5'UTR;5'U	chr5:17529	Island
	rs2358390			SP3;SP3	NM_00311	Body;Body	chr2:17482	N_Shore
rs6610361	rs76720014			LANCL3;L	NM_19851	TSS200;TS	chrX:37430	N_Shore
	rs75367100			FAT1	NM_00524	Body		
				ZNF714	NM_18251	TSS200	chr19:2126	N_Shore
							chr19:5427	S_Shelf
							chr8:72917	Island
	rs45452892			ABCA3	NM_00108	Body	chr16:2334	Island
	rs60004847			FOXO1	NM_00201	Body		
							chr6:29894	Island
rs404607	rs2517742						chr6:29894	S_Shelf
							chr8:14307	Island
	rs2517764						chr6:29894	Island
							chr16:8641	Island
				TSPY4	NM_00116	Body	chrY:92342	Island
				CREB5	NM_18289	5'UTR	chr7:28448	N_Shore

	rs74924576	C14orf149	NM_14458	Body	chr14:5995	N_Shelf
	rs75603638					
		FAM180A	NM_20585	TSS200		
rs79857560	rs75890562					
rs3768224		DDAH1;DI	NM_00113	Body;Body		
		FAM193B;	NM_01905	Body;Body	chr5:17695	Island
rs9992037	rs13105517	TLR2	NM_00326	5'UTR		
		EHD2	NM_01460	3'UTR	chr19:4824	N_Shore
rs74410832						
					chr19:5251	S_Shelf
		PCDHGA2	NM_01891	Body;1stEx	chr5:14072	Island
		NUP210	NM_02492	Body		
	rs78775756	CACNA2D	NM_01839	Body		
		MIR1304;S	NR_03163	TSS200;TSS1500;TSS1500;TSS150		
	rs10097189	MAK16	NM_03250	TSS1500	chr8:33342	Island
					chrX:25020	N_Shore
					chr22:3205	N_Shore
rs62039242					chr16:3373	S_Shelf
		WDR27	NM_18255	3'UTR		
	rs78552162				chr2:88251	S_Shelf
		ACTR3C	NM_00116	5'UTR	chr7:15001	N_Shore
		FAM83H	NM_19848	Body	chr8:14480	Island
	rs78181720	AFAP1;AF	NM_00113	3'UTR;3'UTR;Body		
					chr12:1151	N_Shore
		PXDN	NM_01229	Body		
		HS3ST3A1	NM_00604	Body		
		CARKD	NM_01821	Body	chr13:1112	S_Shelf
rs75506928		OXSM;OX	NR_02693	Body;Body;Body		
		FAHD1;FA	NM_00114	Body;Body	chr16:1876	S_Shelf
		THBS1	NM_00324	Body	chr15:3987	S_Shelf
		LOC10013	NR_02456	TSS200;TS	chr4:12029	N_Shore
	rs34438310	USP10	NM_00515	Body		
		THADA;TH	NM_02206	Body;Body		
		ARPC1A	NM_00640	Body		
		DPPA5	NM_00102	TSS200	chr6:74063	Island
	rs9391807	HLA-L	NR_02782	Body	chr6:30227	Island
		RNH1;RNH	NM_20338	Body;Body	chr11:4948	S_Shelf
rs2817054					chr6:35733	Island

	rs1043060		FAM102A;	NM_20330	3'UTR;3'U	chr9:13070	N_Shore
			DCHS2;DC	NM_01763	Body;Body	chr4:15525	Island
						chr7:10809	S_Shelf
						chr17:8025	N_Shelf
			TSSC1	NM_00331	Body	chr2:33176	N_Shore
	rs11074139		RGMA;RG	NM_02021	Body;5'UT	chr15:9363	N_Shore
			GMDS	NM_00150	Body		
						chr16:1501	Island
rs35955010			ANAPC10;	NM_01488	5'UTR;TSS	chr4:14601	N_Shore
			GRIK2;GR	NM_00116	Body;Body	chr6:10184	S_Shore
			PPP1R2P9	NR_002191	TSS1500	chrX:42637	S_Shore
	rs58867145					chr16:2507	N_Shelf
rs6519497			GSTT1;GS	NM_00085	5'UTR;1stE	chr22:2438	Island
	rs58481121		ANKFN1	NM_15322	Body		
rs73907520			WISP2	NM_00388	TSS1500		
						chr8:43131	Island
rs2599251	rs55793262					chr2:91634	Island
rs11785793						chr8:13651	S_Shore
	rs12439297		SLCO3A1;	NM_00114	Body;Body	chr15:9245	S_Shore
rs12906774			SNRPN;SN	NM_02280	5'UTR;5'U	chr15:2512	Island
		TRUE	CPA4;CPA	NM_00116	1stExon;1stExon;5'UTR;5'UTR		
			OTOA;OT	NM_17066	Body;Body;Body		
	rs7153897		FLJ31306;	NR_02943	Body;Body	chr14:5876	N_Shelf
			HLA-DPA1	NM_03355	Body		
rs67418040			HLA-DRB	NR_00129	Body		
rs74589225			GMDS	NM_00150	Body		
						chr4:48664	N_Shore
			FBN1	NM_00013	Body		
			C6orf127	NM_00101	Body	chr6:35754	Island
	rs10259248					chr7:10146	S_Shelf
			KRTAP5-1	NM_00101	1stExon		
	rs1944694						
	rs7740081		KHDC1	NM_03056	TSS1500	chr6:73972	S_Shore
			KIAA1529	NM_02089	Body	chr9:10006	Island

			DYSFIP1	NM_00100	TSS200	chr17:7979	S_Shore
			ZCCHC14	NM_01514	Body		
rs77153132							
rs9365910							
rs76745887			SNX29	NM_00108	Body		
	rs4809352					chr20:6243	Island
			PCDHB3	NM_01893	1stExon	chr5:14048	N_Shore
			VCX3B	NM_00100	Body		
rs209140						chr6:28863	Island
			DKFZp686	NR_002801	TSS1500		
			SGCZ	NM_13916	Body	chr8:15094	N_Shore
			PON1	NM_00044	TSS200	chr7:94953	S_Shore
			CSF1R	NM_00521	TSS200		
			GPR75;LO	NM_00679	5'UTR;1stE	chr2:54086	Island
		TRUE	CDH13	NM_00125	TSS1500	chr16:8266	N_Shore
			KLHL21	NM_01485	Body	chr1:66617	N_Shore
rs34777947						chr7:10522	Island
	rs7460592					chr8:98368	N_Shelf
	rs34756202		OTUB2	NM_02311	TSS1500	chr14:9449	N_Shore
rs76469054			H1F0	NM_00531	1stExon	chr22:3820	N_Shore
			ERRFI1	NM_01894	5'UTR	chr1:80855	N_Shore
			USP7	NM_00347	Body		
			LITAF;LIT	NM_00113	5'UTR;5'UTR;Body;5'UTR		
			C1orf86;LC	NM_00114	Body;Body	chr1:21210	Island
						chr16:8831	N_Shelf
rs4649016							
			DCAF12L1	NM_17847	TSS200	chrX:12568	S_Shore
			ABLIM3	NM_01494	Body		
			ADORA3	NM_00108	TSS1500		
	rs35650130		SLC45A4	NM_00108	TSS1500	chr8:14223	S_Shelf
rs17844388			PCDHB3	NM_01893	1stExon	chr5:14048	N_Shore
			PRR23A	NM_00113	TSS200	chr3:13872	Island
			C1orf86;LC	NM_00114	Body;Body	chr1:21210	Island
			KLHL17	NM_19831	Body	chr1:89431	Island

			PPIE;PPIE;	NM_20345	TSS1500;T	chr1:40204	N_Shore
	rs76327981		TNFAIP6	NM_00711	TSS200		
	rs77675604		RPL19P12;	NR_02666	TSS1500;5'UTR;5'UTR		
rs5743496			BPI	NM_00172	TSS200		
	rs6940682						
			AKAP13;A	NM_14476	Body;Body;Body		
		TRUE	DEFB125	NM_15332	1stExon		
			TRIM31	NM_00702	Body		
rs12668093			PCLO	NM_03302	Body		
			DLG2;DLC	NM_00114	Body;Body		
			EPS15	NM_00198	TSS1500	chr1:51984	S_Shore
			TBL1X;TB	NM_00113	5'UTR;5'U	chrX:94328	N_Shore
			EPS8	NM_00444	5'UTR		
			SIM1	NM_00506	Body	chr6:10089	N_Shore
rs12947905	rs2256987		ABR;ABR;	NM_02196	Body;Body	chr17:9755	N_Shore
			RHAG	NM_00032	TSS1500		
			HLA-DPB1	NM_00212	Body		
						chr19:4658	S_Shore
			NHSL2;RG	NM_00101	Body;TSS2	chrX:71350	Island
			TRPM4	NM_01763	Body	chr19:4968	S_Shelf
			EXPH5;EX	NM_00114	Body;Body;TSS200		
	rs11354		GNA12	NM_00735	3'UTR		
			GRIK2;GR	NM_00116	Body;Body	chr6:10184	S_Shore
			TNPO2;TN	NM_00113	5'UTR;5'U	chr19:1283	Island
			RPS4Y2	NM_00103	TSS200		
			DIAPH2;D	NM_00672	Body;Body	chrX:95939	S_Shore
						chr9:10379	N_Shore
			PITPNC1;P	NM_18167	Body;Body		
rs34242076			MDM1	NM_01744	Body		
			CCNH	NM_00123	Body	chr5:86708	N_Shore
rs72732970	rs75575681		MAPK6	NM_00274	5'UTR		
rs35509575			PRR23B	NM_00101	TSS200	chr3:13873	Island
			TMEM40	NM_01830	5'UTR		
	rs12731347		PADI4	NM_01238	Body		
			SLC7A3;SI	NM_03280	TSS200;TS	chrX:70150	S_Shore
rs9891975						chr17:2068	Island
			GCNT3;GC	NM_00475	1stExon;5'UTR		
			LOC10018	NR_024467	Body;Body	chr10:9316	S_Shelf

			SNX27	NM_03091	Body		
rs6686649							
			NNMT	NM_00616	TSS1500		
	rs7187759						
						chr4:12920	S_Shelf
rs73494349			RAB11FIP	NM_01470	TSS1500	chr16:4749	N_Shore
			NFIL3	NM_00538	5'UTR	chr9:94183	N_Shore
rs61613048			MYO1B;M	NM_00113	Body;Body;Body		
						chr21:4700	Island
	rs41279230		ACSBG1	NM_01516	Body		
	rs7131580						
			C1orf172	NM_15236	5'UTR	chr1:27286	N_Shore
	rs74875985						
	rs78629934		CMYA5	NM_15361	Body		
			MYST4	NM_01233	Body		
			FAM198B;	NM_00103	Body;Body;Body		
	rs17884389		SFTPA2	NM_00109	TSS1500		
			MICAL2	NM_01463	3'UTR		
			OPRD1	NM_00091	Body	chr1:29189	Island
rs78207116							
	rs9278239						
						chr17:7945	S_Shelf
			PHKA2	NM_00029	TSS1500	chrX:19002	Island
						chr5:58870	Island
						chr2:17297	N_Shore
						chr6:31650	Island
			MRPS18B	NM_01404	Body	chr6:30584	S_Shelf
						chr10:4384	Island
						chr17:3738	S_Shelf
			NCRNA00	NR_026751	Body		
rs71330028						chr21:1543	N_Shore
	rs2506015		RET;RET	NM_02063	Body;Body	chr10:4357	S_Shelf
		TRUE	RDH5;RDH	NM_00290	1stExon;5'U	chr12:5610	S_Shelf
	rs67410356		ACSM3;AC	NM_00562	5'UTR;5'UTR		
rs71384648	rs12930897		TPSG1	NM_01246	TSS1500		
rs1024576							
rs76309452			C21orf81	NR_02727	TSS200	chr21:1535	S_Shore
	rs2270194		VENTX	NM_01446	Body	chr10:1350	S_Shore

			NCRNA002	NR_026795	TSS1500		
rs5987137		TRUE	BCAP31;B	NR_02445	Body;TSS1	chrX:15298	Island
			ZMYM3;Z	NM_00117	5'UTR;TSS	chrX:70473	S_Shore
			FAM171A1	NM_00101	Body		
rs56057807	rs77541628		STAG1	NM_00586	Body		
		TRUE	PTPN20B;	NM_00104	TSS200;TS	chr10:4882	Island
			CELSR3	NM_00140	Body	chr3:48693	Island
			BCL11B;B	NM_02289	Body;Body	chr14:9964	Island
			CAMK2G;	NM_17217	Body;Body;Body;Body;Body		
			TNFAIP8L	NM_20738	Body	chr15:5138	S_Shelf
			XIRP1	NM_19429	TSS1500		
			KIAA0922;	NM_01519	TSS1500;T	chr4:15438	N_Shore
						chr10:5243	Island
			PANX1	NM_01536	Body		
			LOC44160	NR_00303	Body	chr11:5025	Island
	rs1163860						
			MDGA1	NM_15348	Body	chr6:37616	Island
			NMT2	NM_00480	TSS1500	chr10:1520	S_Shore
	rs76244168					chr11:1346	S_Shelf
			RAI2;RAI2	NM_02178	5'UTR;1stE	chrX:17879	Island
		TRUE	ZNF254;Z	NM_20328	1stExon;5'UTR		
			SLC6A18	NM_18263	3'UTR	chr5:12459	Island
			MYO10	NM_01233	Body		
	rs62054659					chr16:9014	N_Shore
	rs78333027						
			SLC12A7	NM_00659	Body	chr5:10546	N_Shore
						chr1:11455	N_Shore
rs10108815	rs10108808					chr8:11372	S_Shore
			CACNB4;C	NM_00100	Body;Body;Body;Body		
rs12149359	rs56278207		CES1;CES	NM_00126	Body;Body	chr16:5586	Island
			FLJ44606;	NM_00116	TSS200;TS	chr5:12640	Island
						chr6:31650	Island
			RXFP3	NM_01656	1stExon	chr5:33936	Island
	rs2056481		MAD1L1;M	NM_00355	Body;Body;Body		
	rs72806843		PCDHGA1	NM_01891	1stExon;1st	chr5:14071	N_Shore
rs74515554	rs77770048						
			FTH1	NM_00203	Body	chr11:6173	N_Shelf
			MYO1C;M	NM_03337	5'UTR;Bod	chr17:1390	N_Shore

	rs35104720	SAMD7	NM_18261	Body		
		GPC6	NM_00570	Body		
rs77093485		PCDHA6;P	NM_03184	Body;Body	chr5:14022	N_Shore
		CLMN	NM_02473	Body		
					chr2:66802	N_Shore
		NLGN4Y;N	NR_02831	Body;Body	chrY:16941	S_Shelf
					chr7:39649	N_Shelf
		NLRP11;N	NM_14500	TSS1500;5'UTR		
		BICC1	NM_00108	Body		
		RBMY2EP	NR_00157	Body		
	rs10248822	GIMAP1	NM_13075	TSS1500		
		PTPRN2;P	NM_00284	Body;Body	chr7:15805	N_Shore
		DOCK2	NM_00494	Body		
rs73376840	rs877430					
		CES1;CES	NM_00126	Body;Body	chr16:5586	Island
rs77028978	rs9349666					
		C6orf89	NM_15273	Body		
	rs475947	OR4N4	NM_00100	1stExon		
					chr6:31650	Island
		MIR2117	NR_031751	TSS1500		
		OXCT1	NM_00043	Body	chr5:41869	Island
		CHST5	NM_02453	Body	chr16:7556	Island
rs77153281		PLEKHG7	NM_00100	Body		
		ULBP2	NM_02521	Body	chr6:15026	S_Shelf
		HS3ST3A1	NM_00604	Body	chr17:1350	N_Shore
		ACAN;AC	NM_01322	5'UTR;5'UTR		
					chr10:1348	N_Shore
					chrY:93854	N_Shore
rs3836745		PCDHB6	NM_01893	1stExon	chr5:14053	N_Shore
		PRR25	NM_00101	Body	chr16:8573	Island
		RUNX1	NM_00175	Body		
		GLIPR1L2	NM_15243	TSS200	chr12:7578	Island
		SLC44A2;S	NM_00114	Body;Body	chr19:1074	Island
		GRAMD3;(	NM_00114	Body;Body;Body;Body;Body		
		LOC10013	NR_02733	TSS1500;TSS1500		
		BRD2;BRD	NM_00111	Body;Body	chr6:32939	S_Shore
	rs1552364					

			BTNL2	NM_01960	Body		
			FRMD5	NM_03289	Body		
			RYR1;RYR	NM_00054	Body;Body	chr19:3905	Island
			RYR1;RYR	NM_00054	Body;Body	chr19:3905	Island
			HLX	NM_02195	Body	chr1:22105	N_Shore
			PYCR2	NM_01332	Body	chr1:22611	N_Shore
			RORA	NM_13426	Body		
			ADAMTS5	NM_00703	TSS1500	chr21:2833	S_Shore
			STOML1	NM_00480	3'UTR		
	rs76631426		ZC3H12D	NM_20736	TSS200		
	rs7018270		DEFA3;DE	NM_00521	TSS1500;TSS1500;TSS1500		
			GTF2A1L;	NM_17219	TSS200;Bo	chr2:48844	Island
			SLC39A14;	NM_00112	5'UTR;5'UTR;5'UTR;5'UTR		
			NUAK1	NM_01484	Body		
						chr5:21367	N_Shore
rs79683292						chr8:13651	Island
rs3834611	rs1135519		MIR122	NR_029667	TSS1500		
rs4760673	rs76081873		TMEM106	NM_00114	TSS1500;T	chr12:4835	N_Shore
			ACMSD	NM_13832	TSS1500		
			PDE4D;PD	NM_00116	Body;Body		
			SOS1	NM_00563	Body		
			PTPRN2;P	NM_00284	Body;Body	chr7:15734	S_Shore
	rs74759139	TRUE	C20orf177;	NM_02210	1stExon;5'U	chr20:5851	Island
		TRUE	HPD	NM_00215	TSS1500		
			CLEC1A;C	NM_01651	5'UTR;1stExon		
			OR5H15	NM_00100	1stExon		
			MIR21	NR_02949	TSS200		
	rs3741704					chr12:5220	N_Shore
						chr1:24780	Island
			HAR1B;HA	NR_00324	Body;TSS1	chr20:6173	N_Shore
			TTTY10	NR_001542	Body		
rs4468809	rs4359646		C2orf54	NM_00108	1stExon		
		TRUE	BTG3;BTG	NM_00113	5'UTR;5'U	chr21:1898	Island
	rs4750963						
rs72884175	rs72799821						
rs7559444	rs7559434						
						chr6:17033	Island

			SNTB2	NM_00675	Body		
			SLC12A4;S	NM_00114	3'UTR;3'UTR;3'UTR;3'UTR;3'UTR		
						chr4:30430	Island
			RASGEF1C	NM_17506	5'UTR	chr5:17958	Island
			C1orf187	NM_19854	5'UTR		
						chr2:23185	Island
						chr12:1326	N_Shore
			FLJ42289;F	NR_02814C	TSS1500;T	chr15:1008	S_Shore
			MUM1L1	NM_15242	5'UTR		
			GREB1	NM_01466	TSS200		
			KCNK12	NM_02205	Body		
rs12220734							
			GRM3	NM_00084	5'UTR		
			AP2A2	NM_01230	Body	chr11:9752	S_Shore
						chrX:39547	Island
rs2278269							
			NSUN5;NS	NM_01804	TSS1500;T	chr7:72722	S_Shore
			FNBP1L;FN	NM_00102	TSS200;TS	chr1:93913	N_Shore
rs75096987			RAB21	NM_01499	Body	chr12:7214	S_Shore
						chr10:1245	Island
			AFAP1;AF	NM_19859	Body;Body		
						chr1:22769	N_Shore
			CD99L2;CI	NM_13444	TSS200;TS	chrX:15006	Island
	rs75105531		FBXO31;FI	NR_02456	Body;Body	chr16:8736	N_Shore
			KLHL24	NM_01764	Body		
			ELF1;ELF1	NM_17237	5'UTR;1stExon		
			SLCO3A1;S	NM_00114	Body;Body		
			KCNIP1;KC	NM_00103	Body;Body;Body		
			CDKAL1	NM_01777	Body		
			VEZT	NM_01759	Body		
rs7200011			CES4	NR_00327	Body	chr16:5579	Island
			BRD2;BRD	NM_00111	5'UTR;1stE	chr6:32939	N_Shore
			N4BP3	NM_01511	Body		
			ZCCHC14	NM_01514	Body		
		TRUE	TMTC2	NM_15258	Body		
			EVPL	NM_00198	3'UTR	chr17:7400	N_Shore

| | | | | | | | |
[truncated: 325,348 more chars]
